# Supplementary figures and images for: A deep learning approach for the detection and counting of colon cancer cells (HT-29 cells) bunches and impurities (part 1 of 6)
Source: PeerJ Comput Sci. 2023 Dec 5;9:e1651. doi: 10.7717/peerj-cs.1651 (PMC10773923; doi:10.7717/peerj-cs.1651)

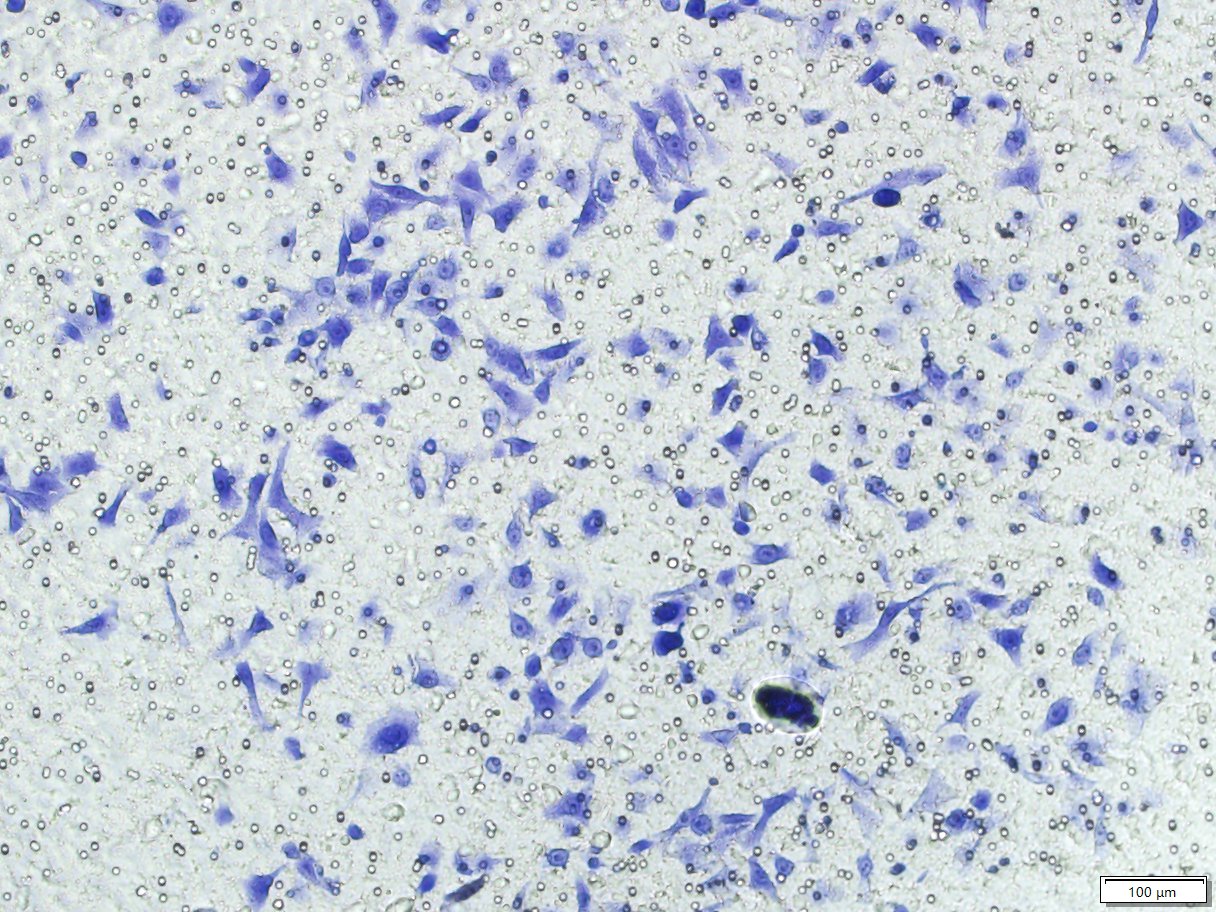

Supplement: Supplemental Information 2 [file peerj-cs-09-1651-s002.zip › Dataset 1/0+1.jpg]

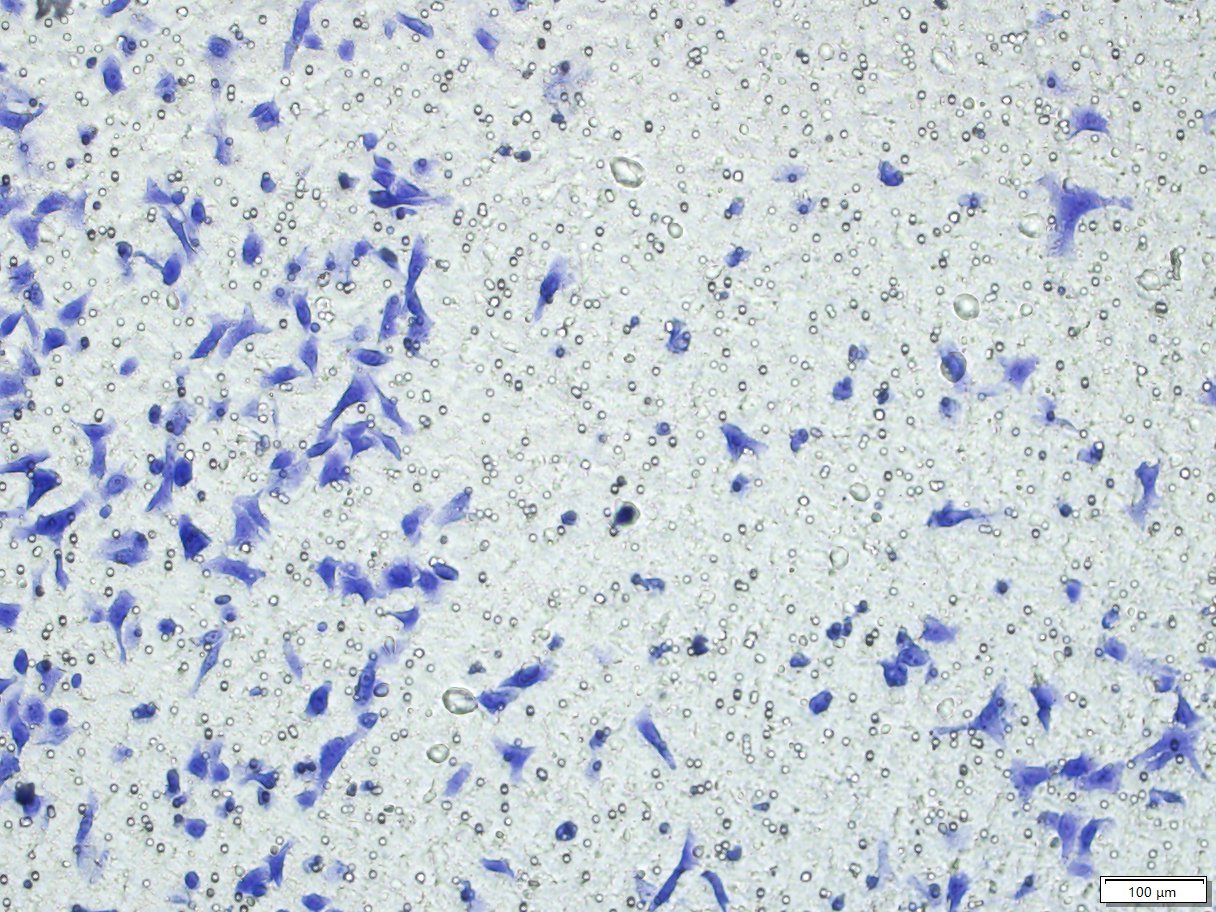

Supplement: Supplemental Information 2 [file peerj-cs-09-1651-s002.zip › Dataset 1/0+2.jpg]

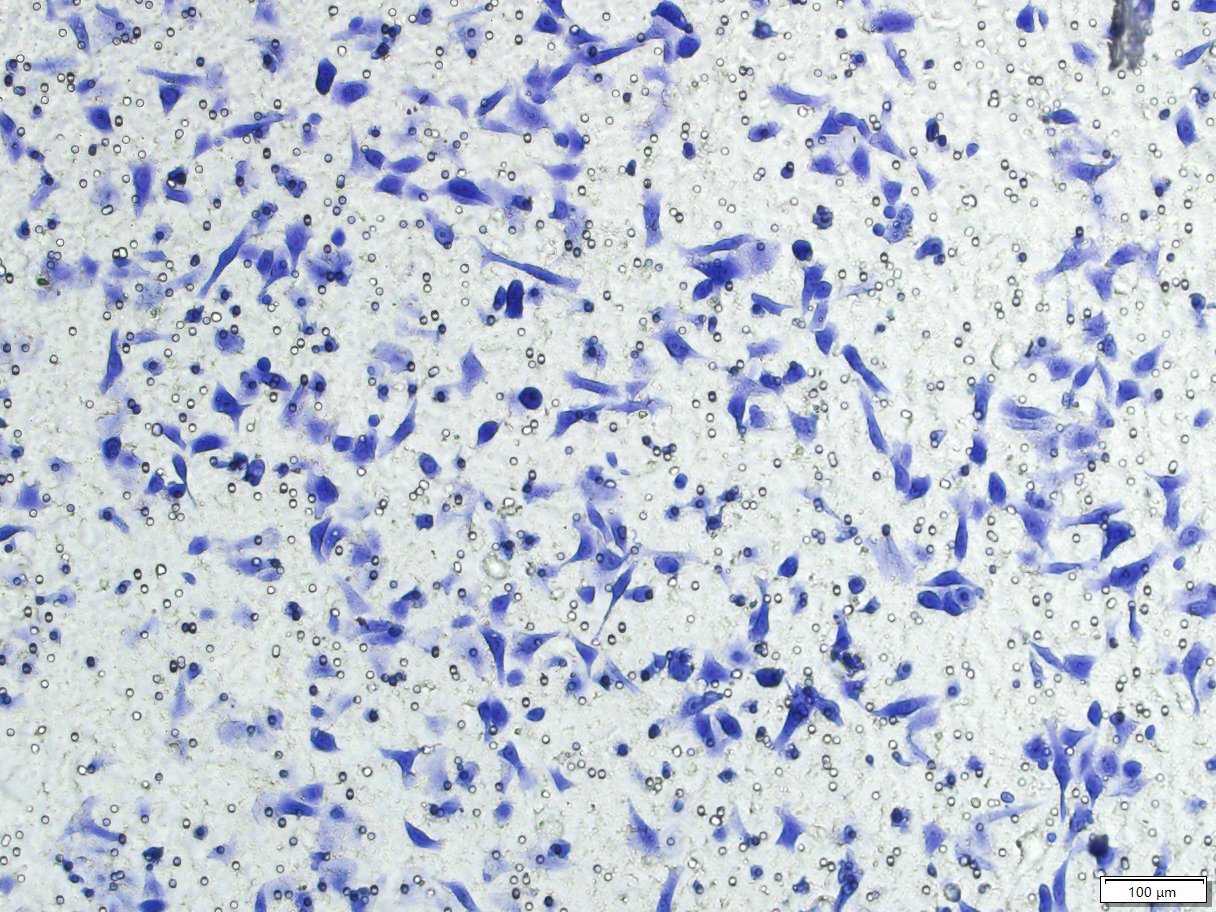

Supplement: Supplemental Information 2 [file peerj-cs-09-1651-s002.zip › Dataset 1/0+3.jpg]

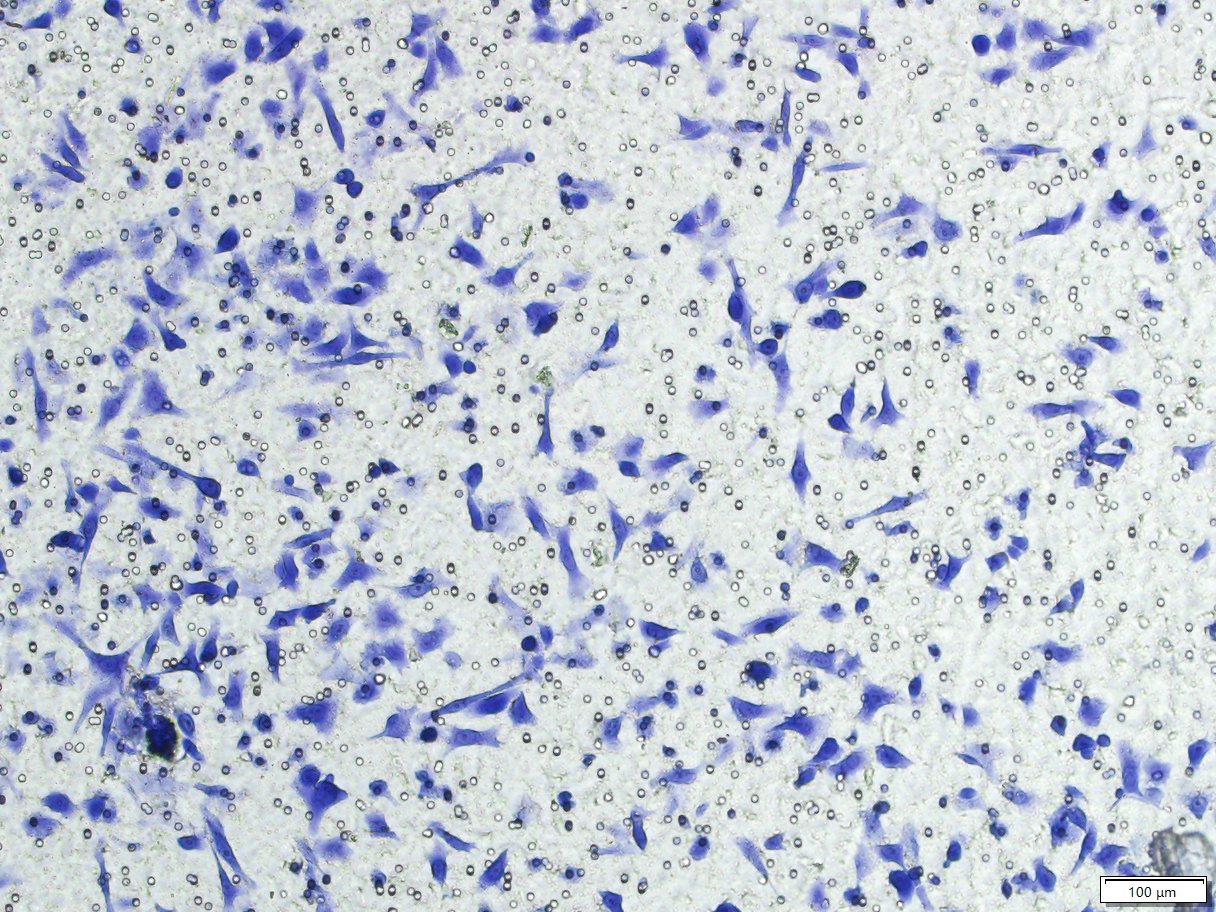

Supplement: Supplemental Information 2 [file peerj-cs-09-1651-s002.zip › Dataset 1/0+4.jpg]

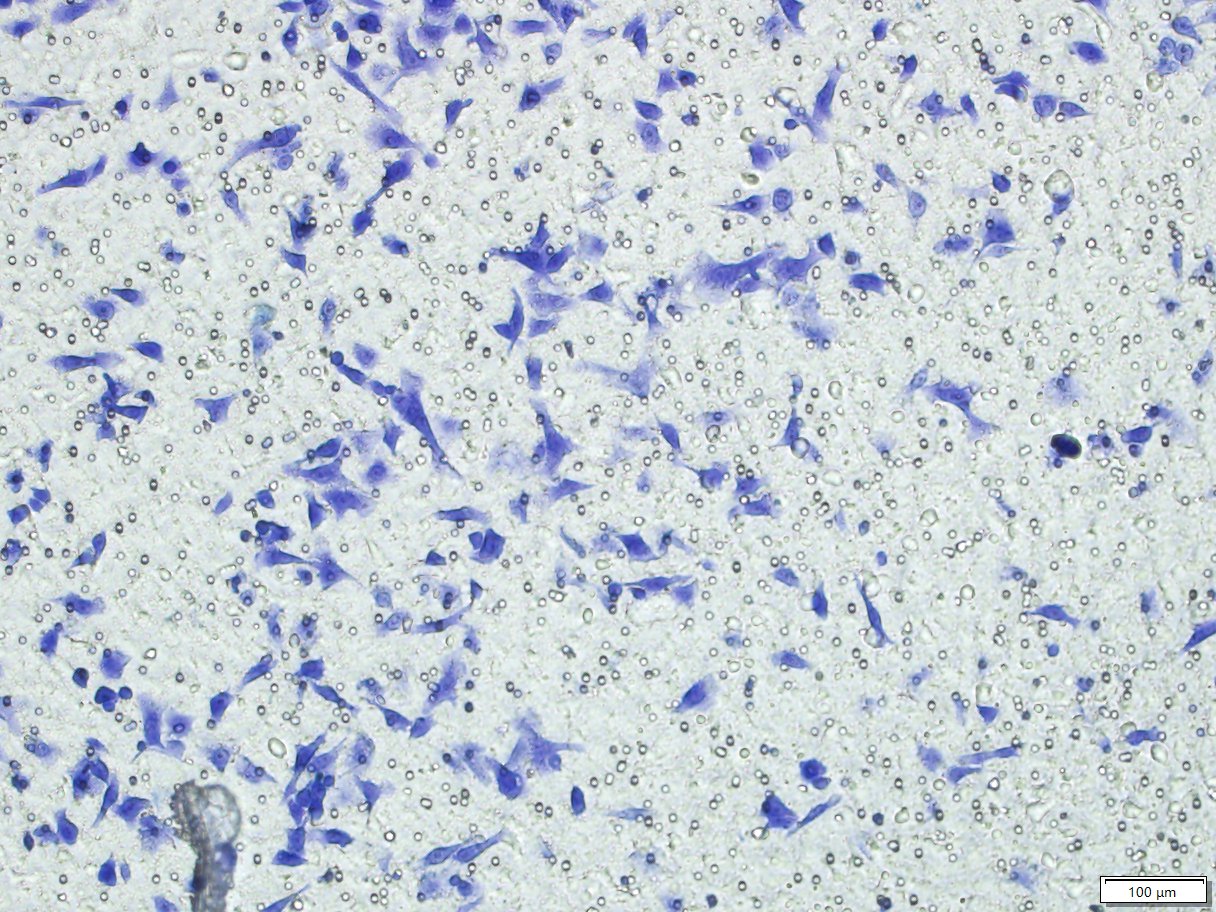

Supplement: Supplemental Information 2 [file peerj-cs-09-1651-s002.zip › Dataset 1/0+5.jpg]

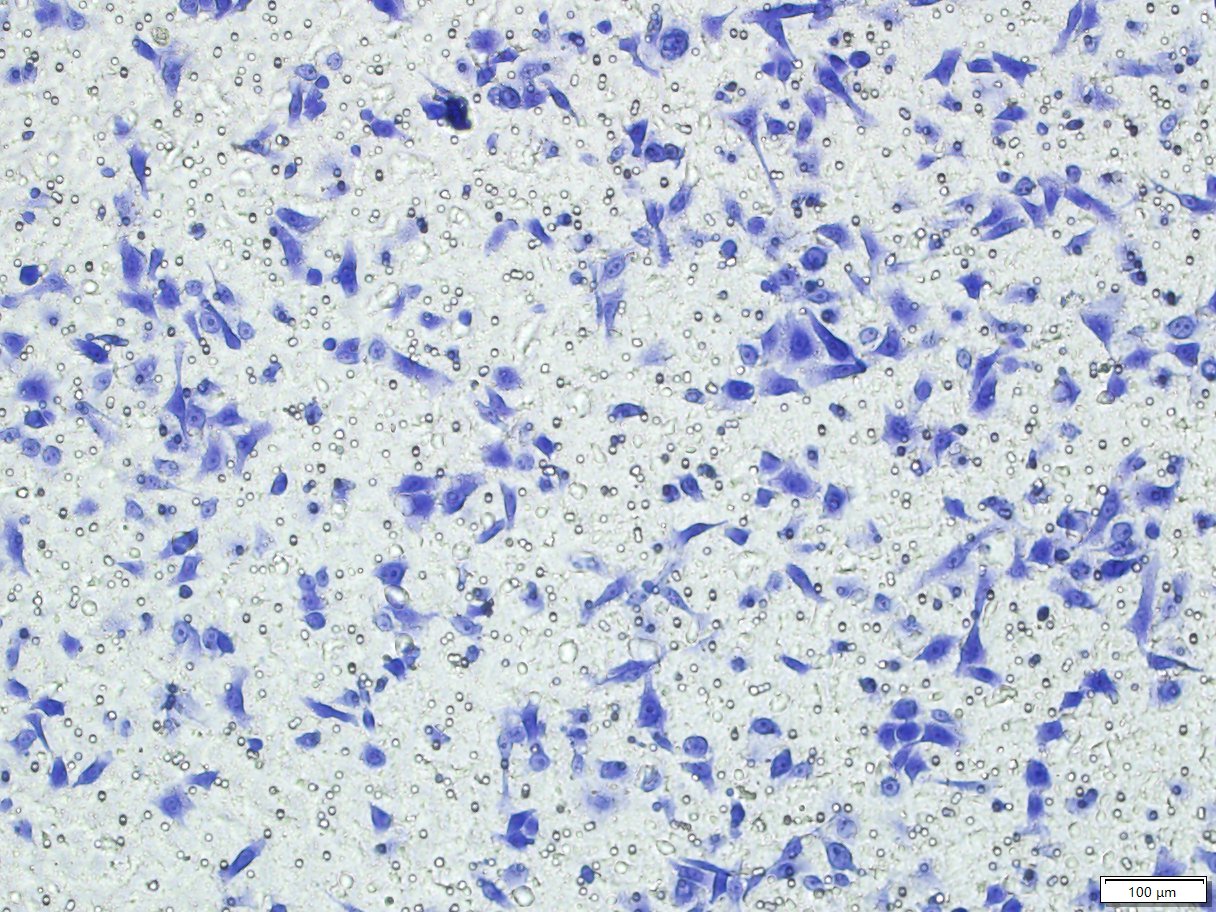

Supplement: Supplemental Information 2 [file peerj-cs-09-1651-s002.zip › Dataset 1/0+6.jpg]

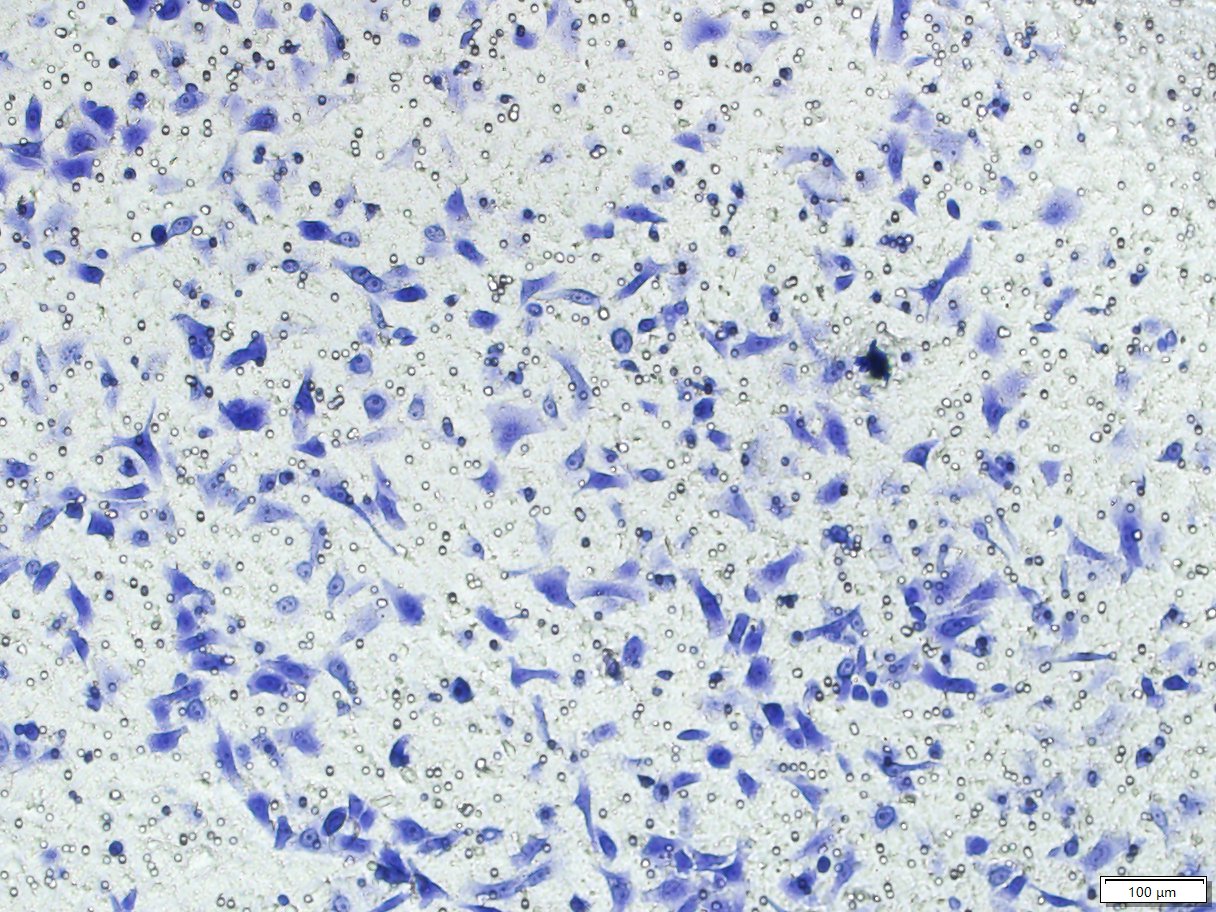

Supplement: Supplemental Information 2 [file peerj-cs-09-1651-s002.zip › Dataset 1/0+7.jpg]

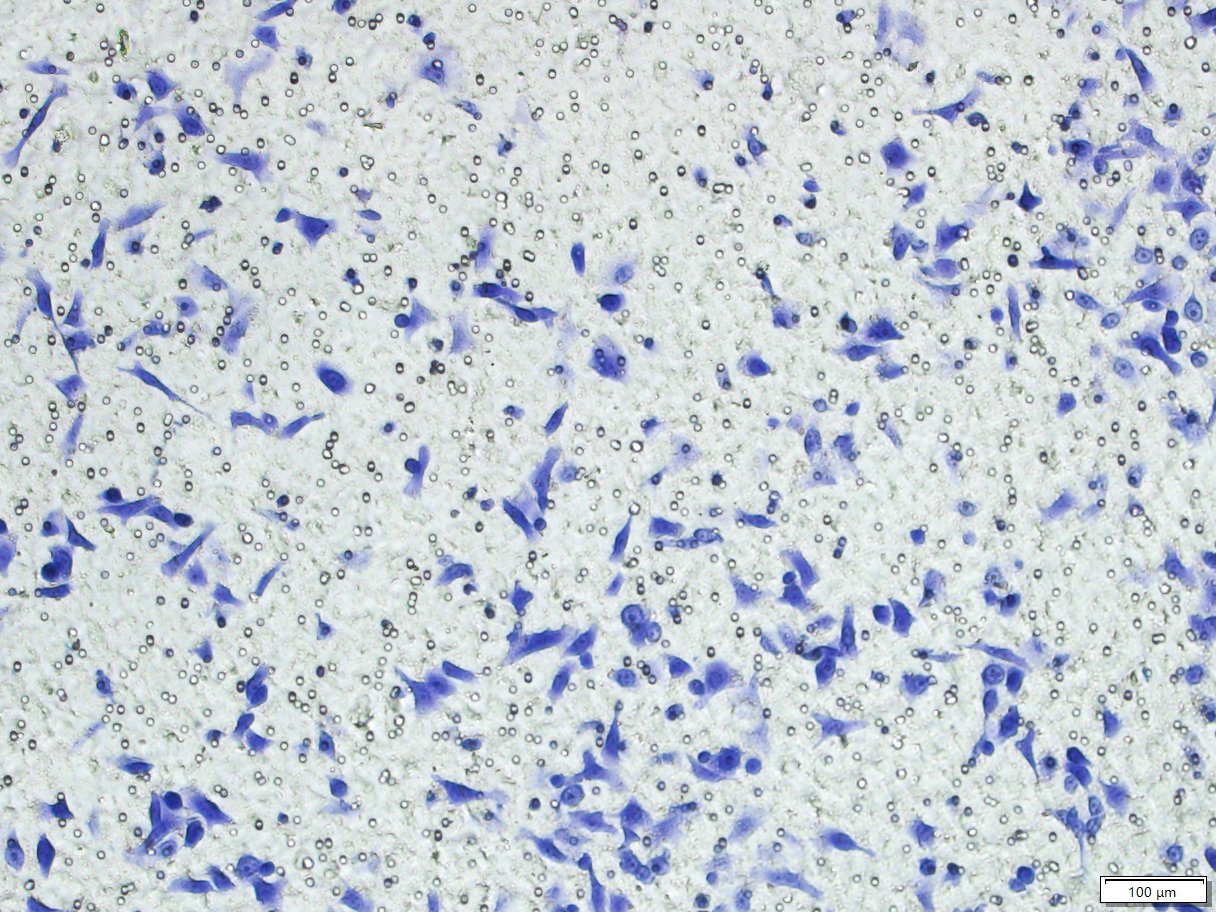

Supplement: Supplemental Information 2 [file peerj-cs-09-1651-s002.zip › Dataset 1/0+8.jpg]

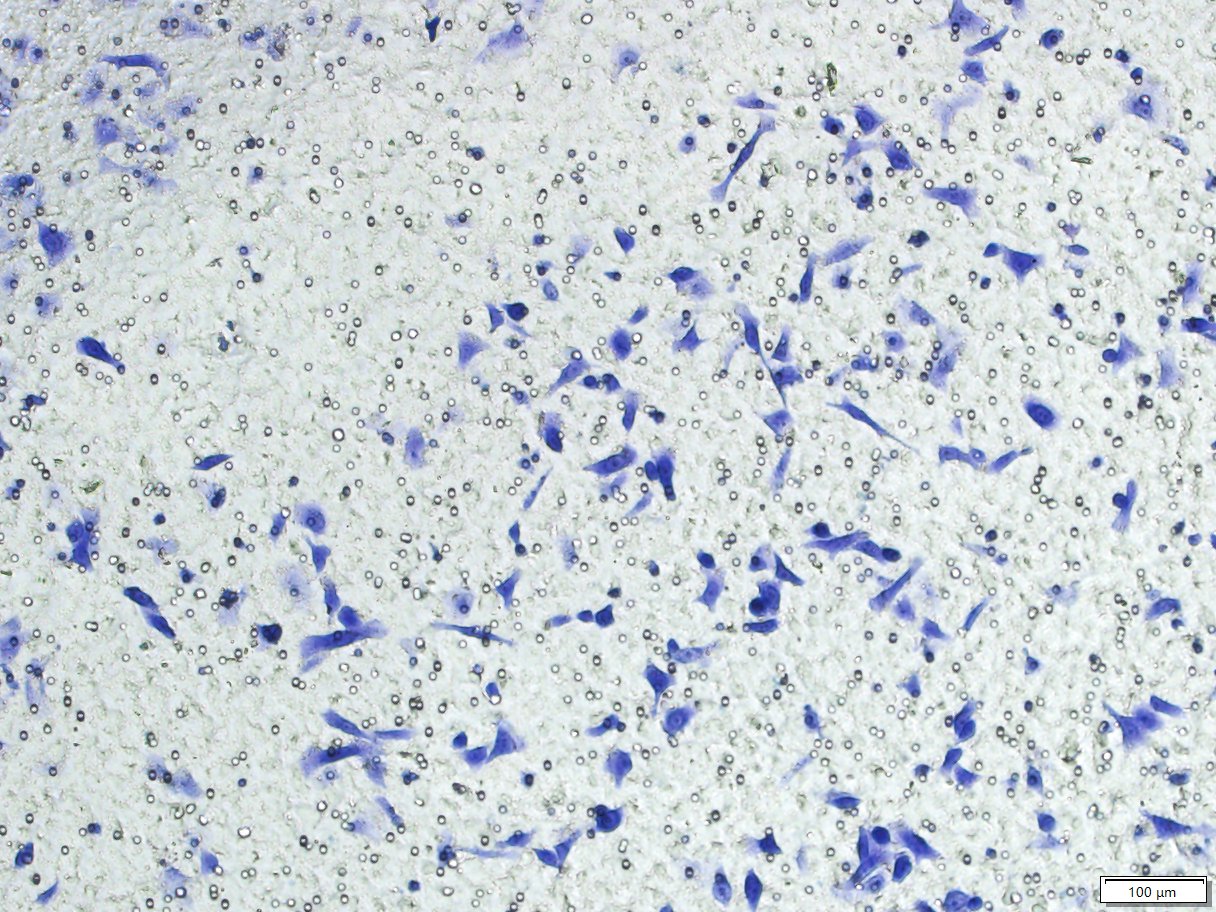

Supplement: Supplemental Information 2 [file peerj-cs-09-1651-s002.zip › Dataset 1/0+9.jpg]

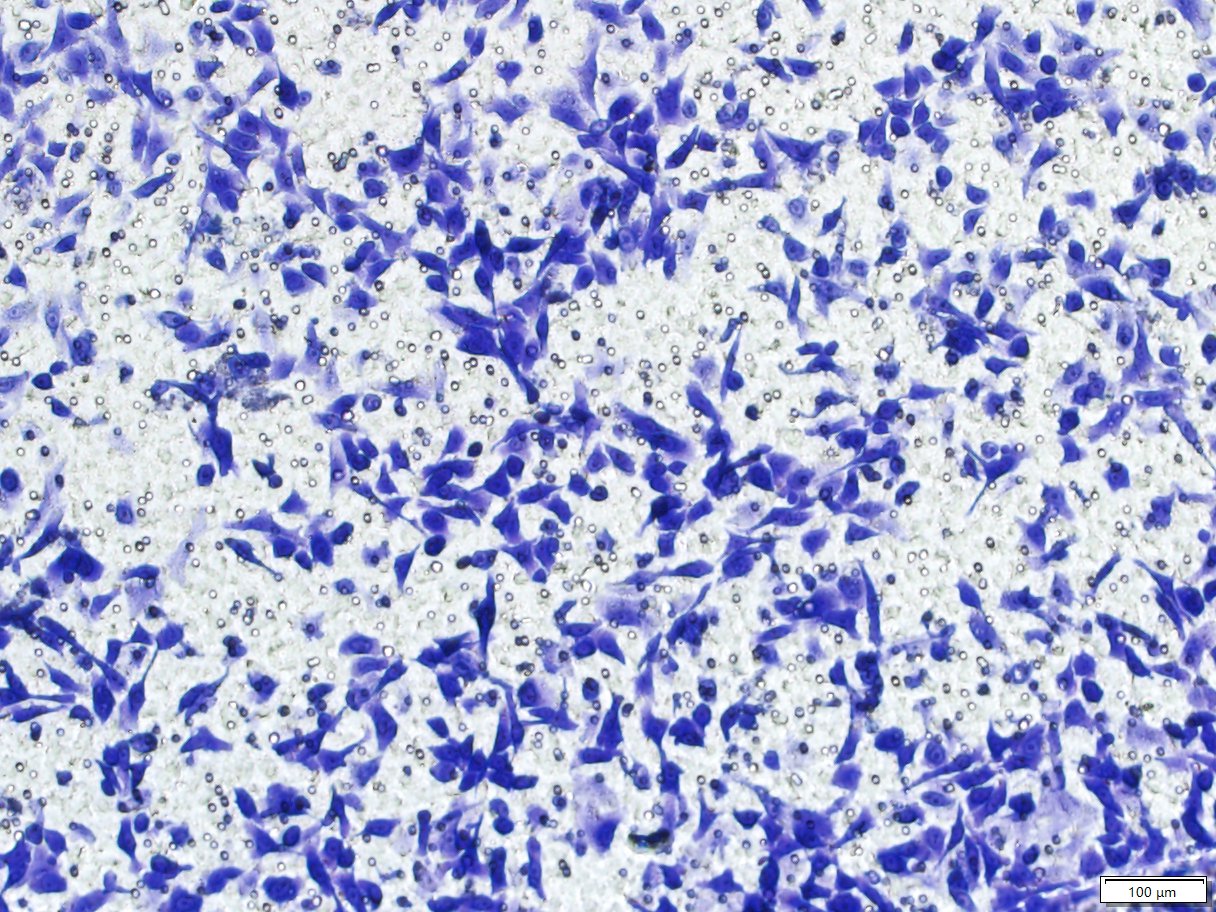

Supplement: Supplemental Information 2 [file peerj-cs-09-1651-s002.zip › Dataset 1/0-1.jpg]

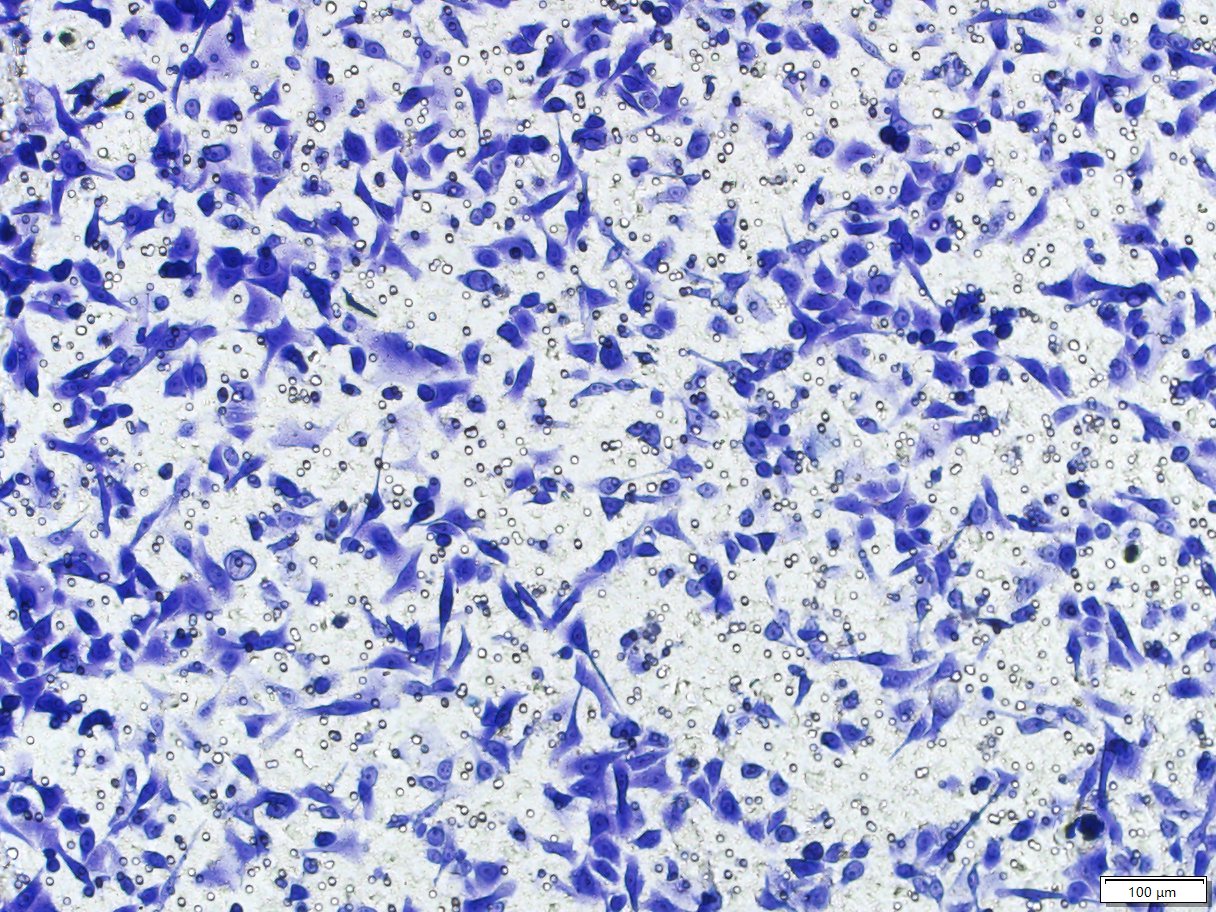

Supplement: Supplemental Information 2 [file peerj-cs-09-1651-s002.zip › Dataset 1/0-10.jpg]

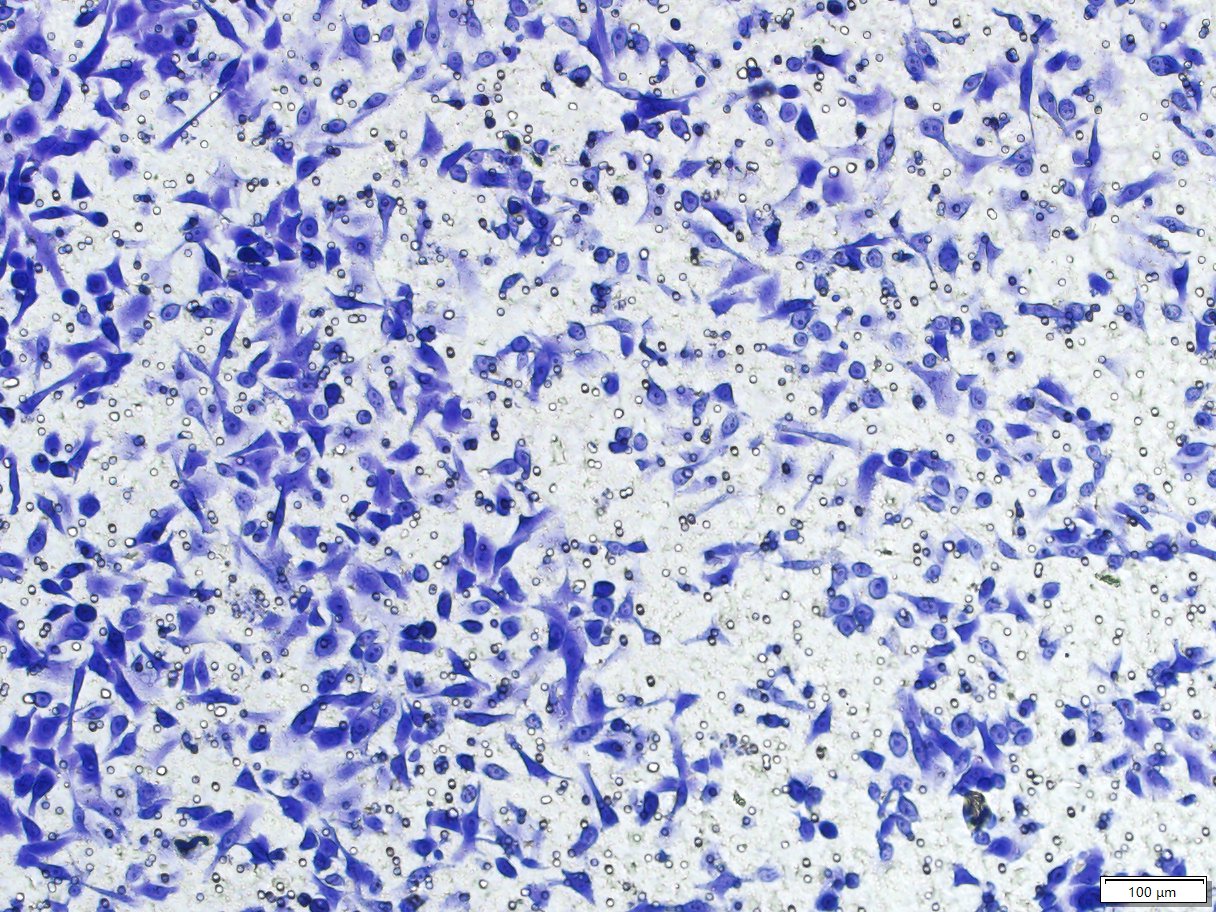

Supplement: Supplemental Information 2 [file peerj-cs-09-1651-s002.zip › Dataset 1/0-11.jpg]

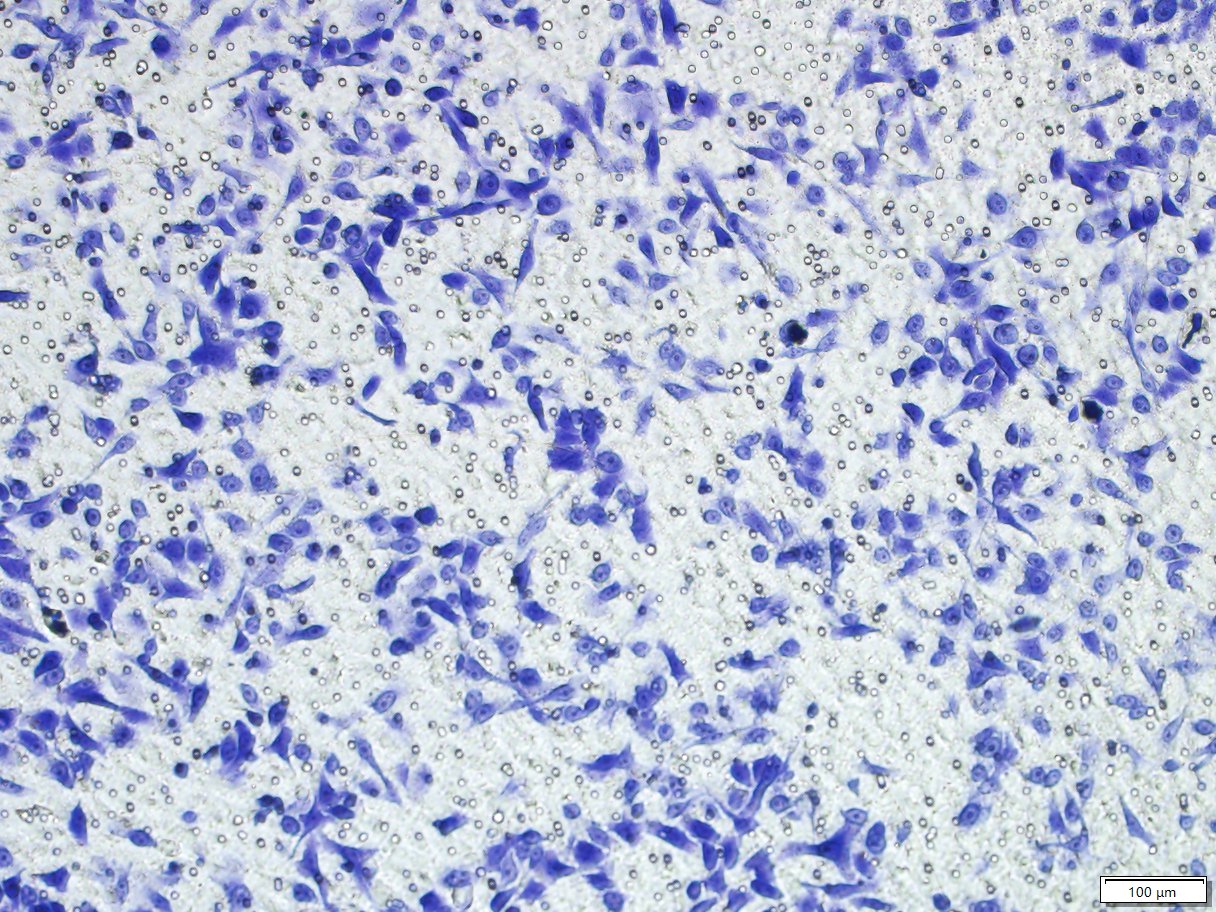

Supplement: Supplemental Information 2 [file peerj-cs-09-1651-s002.zip › Dataset 1/0-12.jpg]

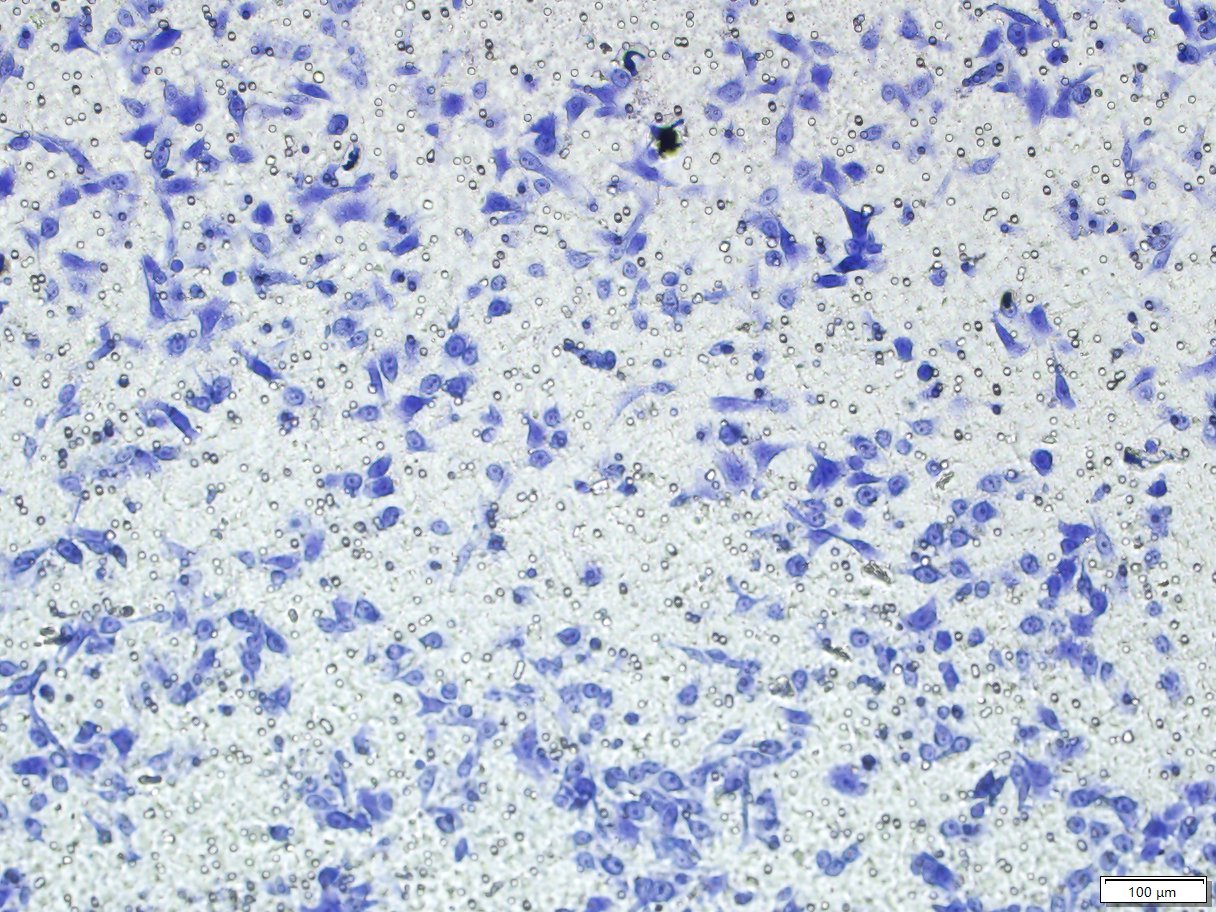

Supplement: Supplemental Information 2 [file peerj-cs-09-1651-s002.zip › Dataset 1/0-13.jpg]

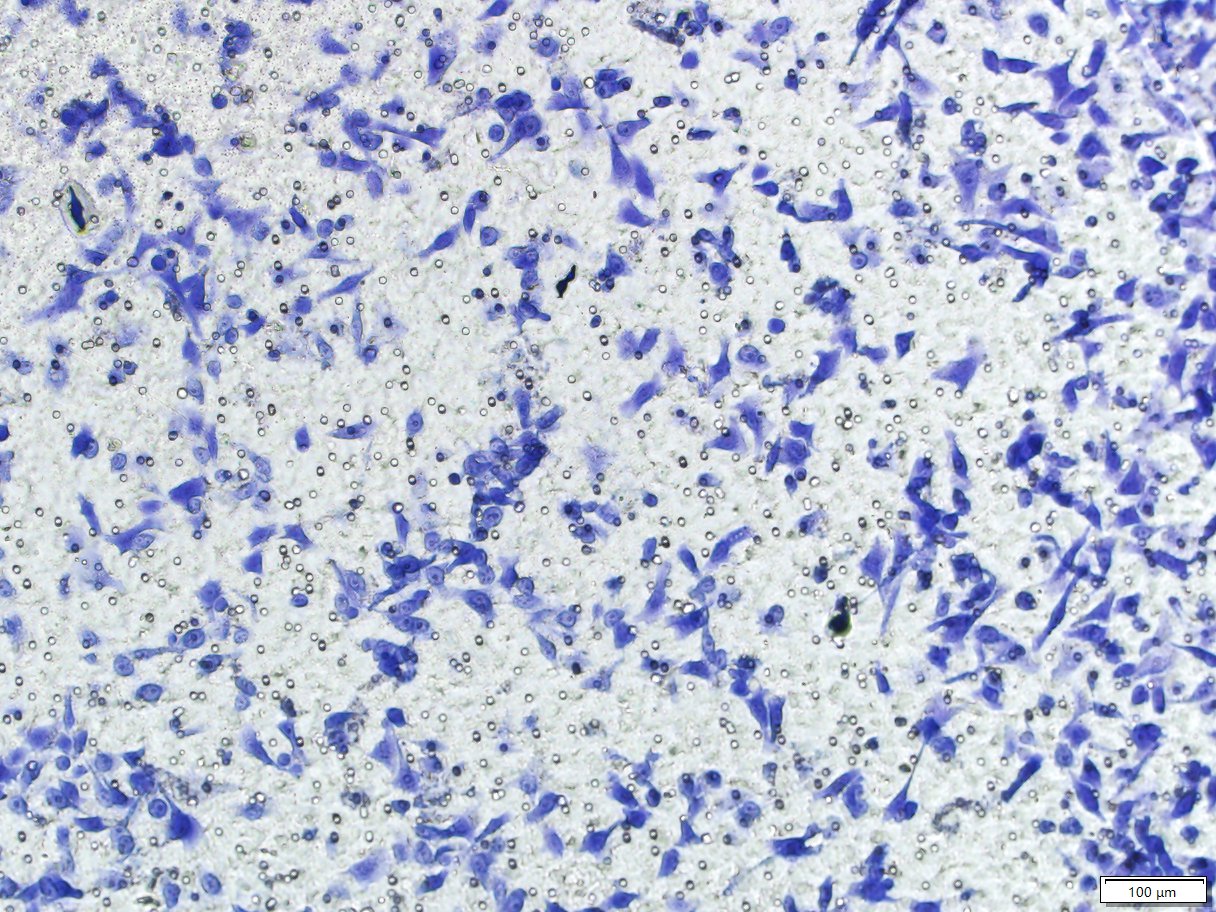

Supplement: Supplemental Information 2 [file peerj-cs-09-1651-s002.zip › Dataset 1/0-14.jpg]

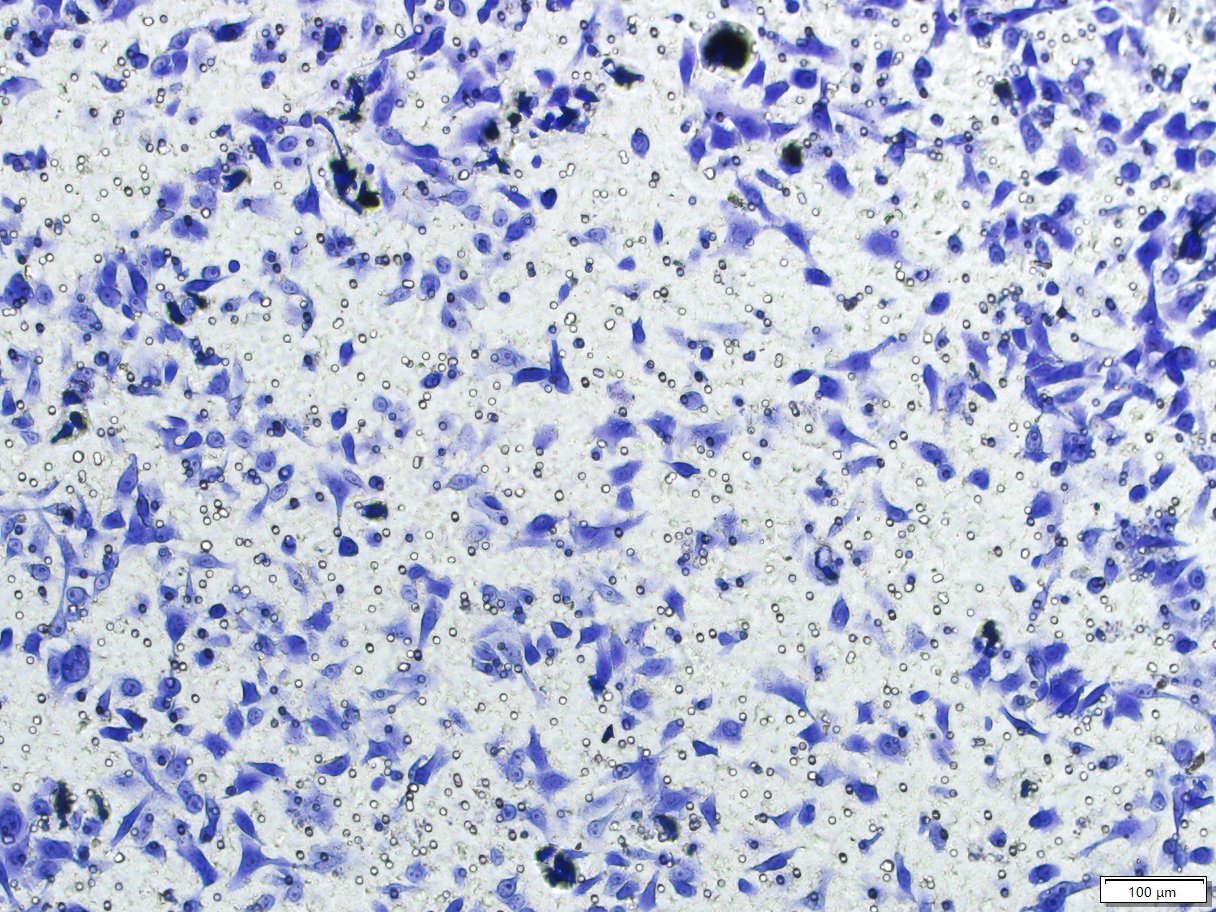

Supplement: Supplemental Information 2 [file peerj-cs-09-1651-s002.zip › Dataset 1/0-15.jpg]

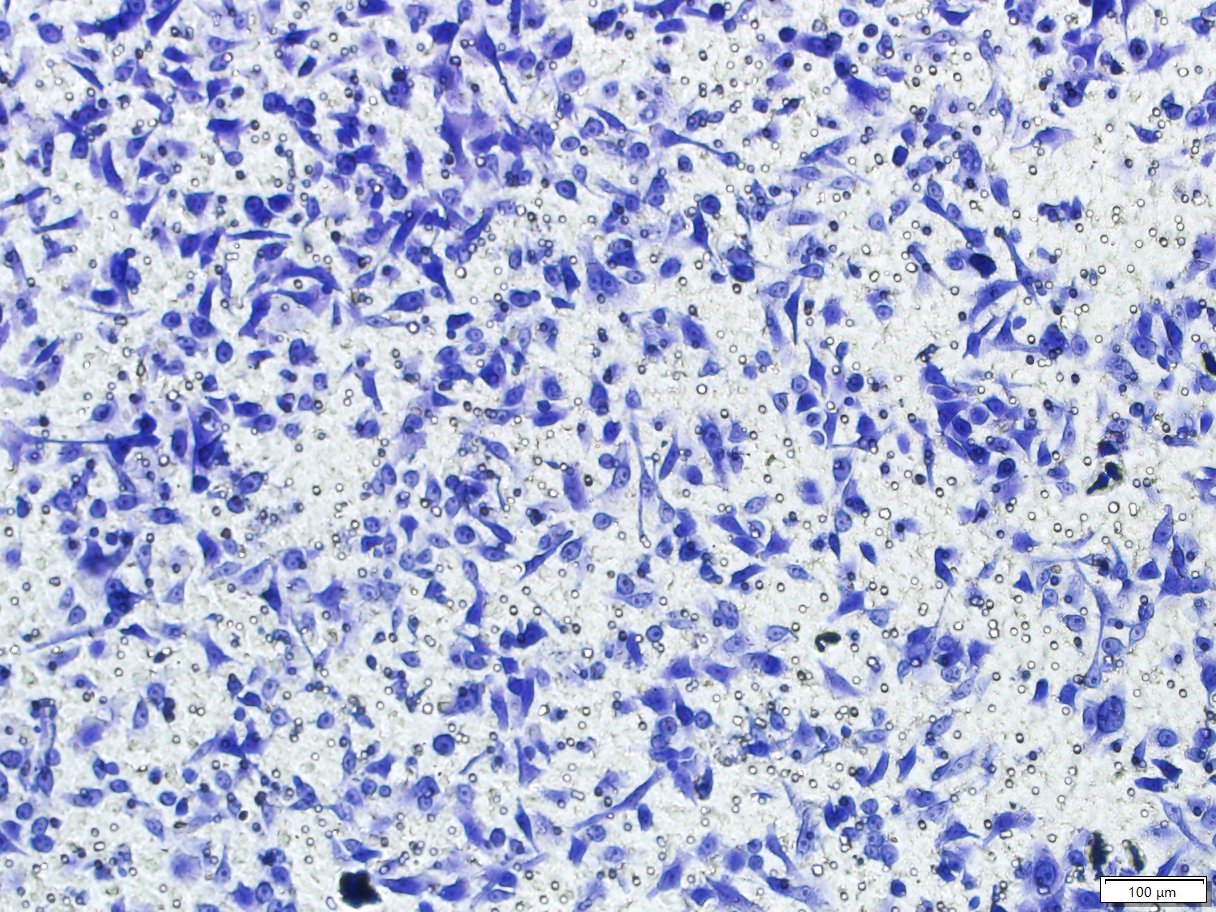

Supplement: Supplemental Information 2 [file peerj-cs-09-1651-s002.zip › Dataset 1/0-16.jpg]

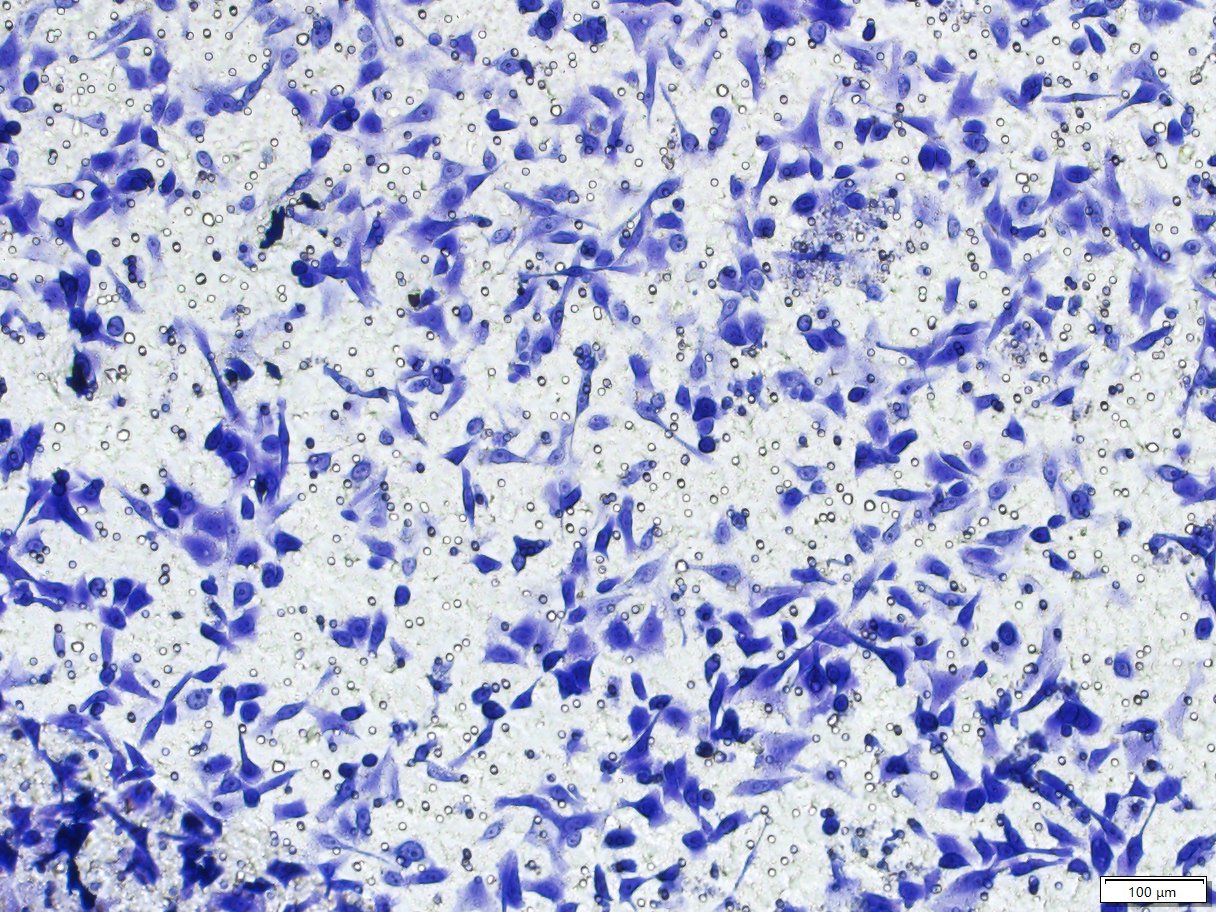

Supplement: Supplemental Information 2 [file peerj-cs-09-1651-s002.zip › Dataset 1/0-2.jpg]

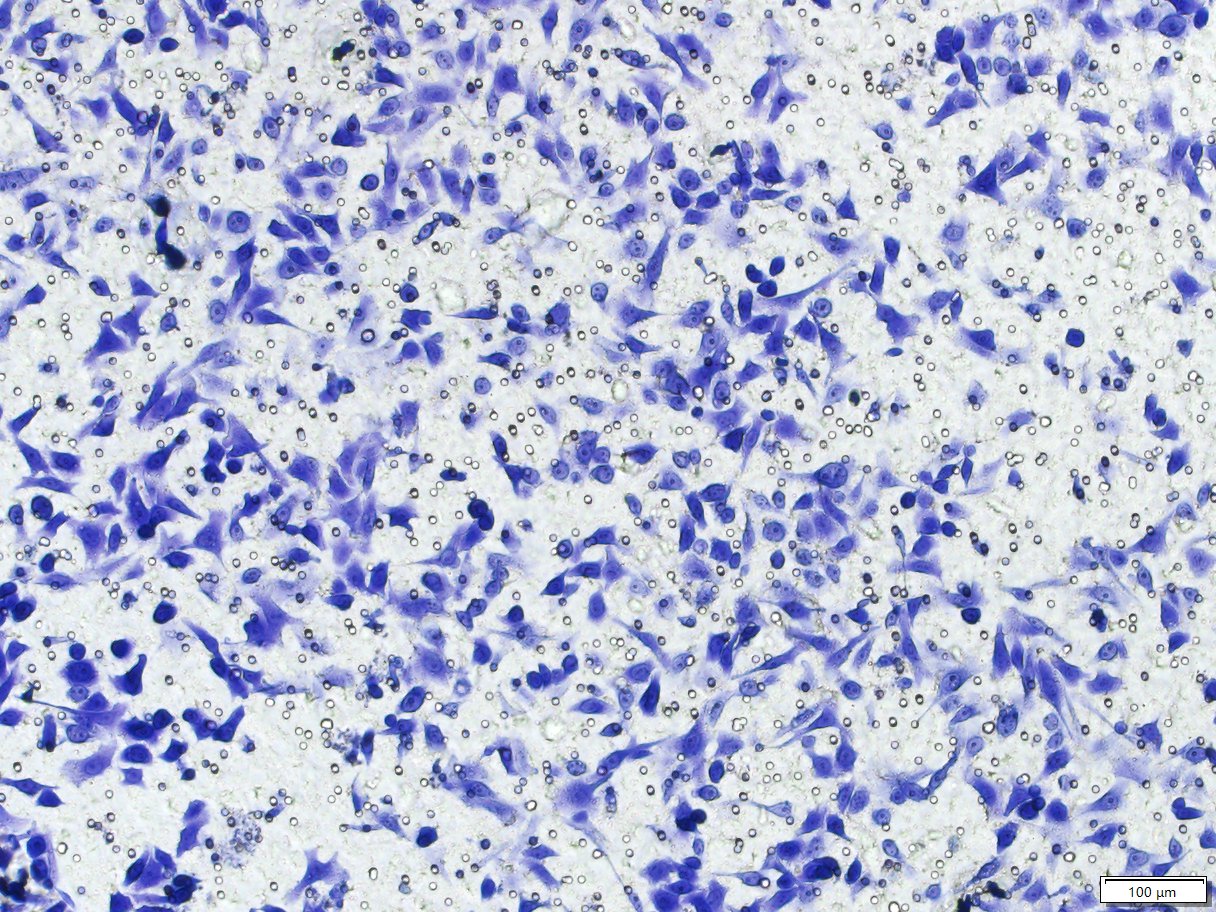

Supplement: Supplemental Information 2 [file peerj-cs-09-1651-s002.zip › Dataset 1/0-3.jpg]

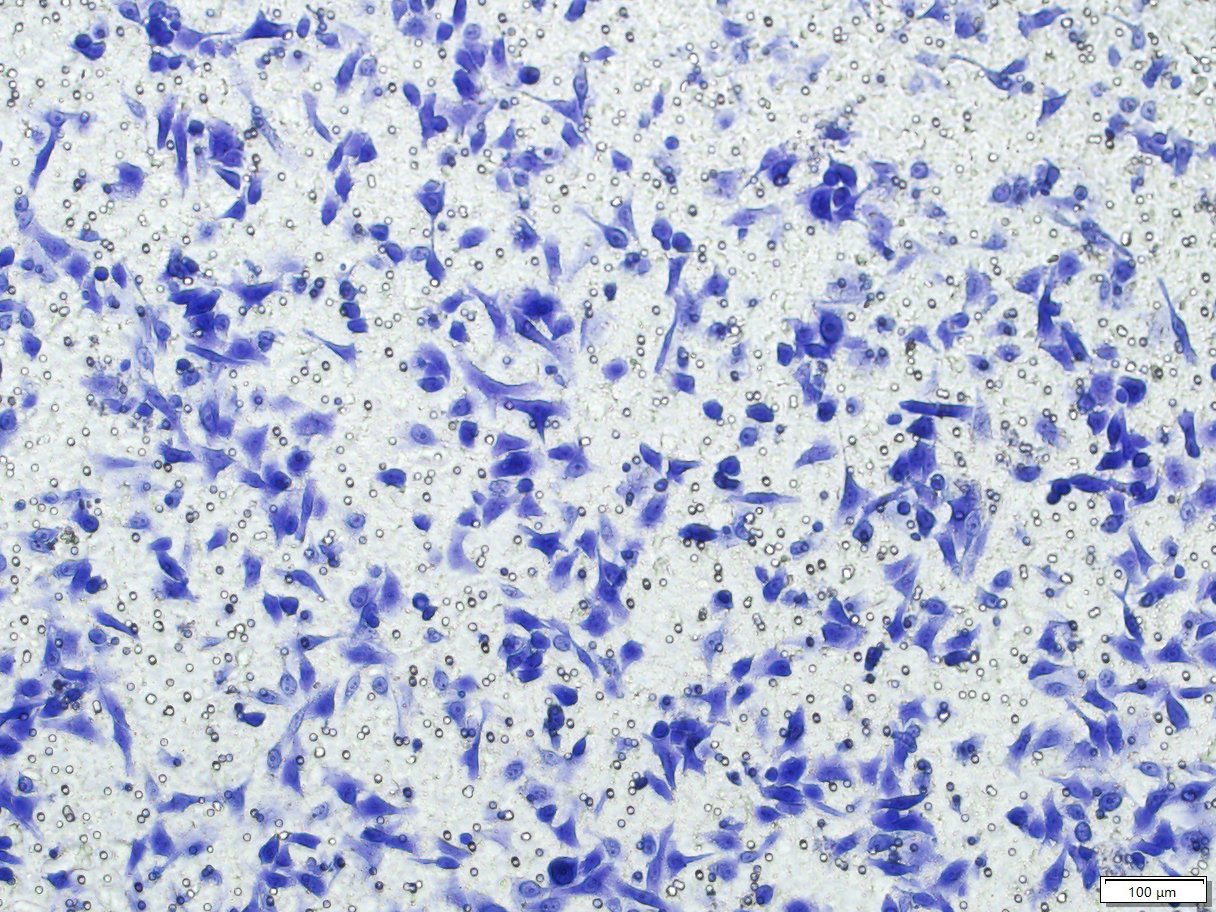

Supplement: Supplemental Information 2 [file peerj-cs-09-1651-s002.zip › Dataset 1/0-4.jpg]

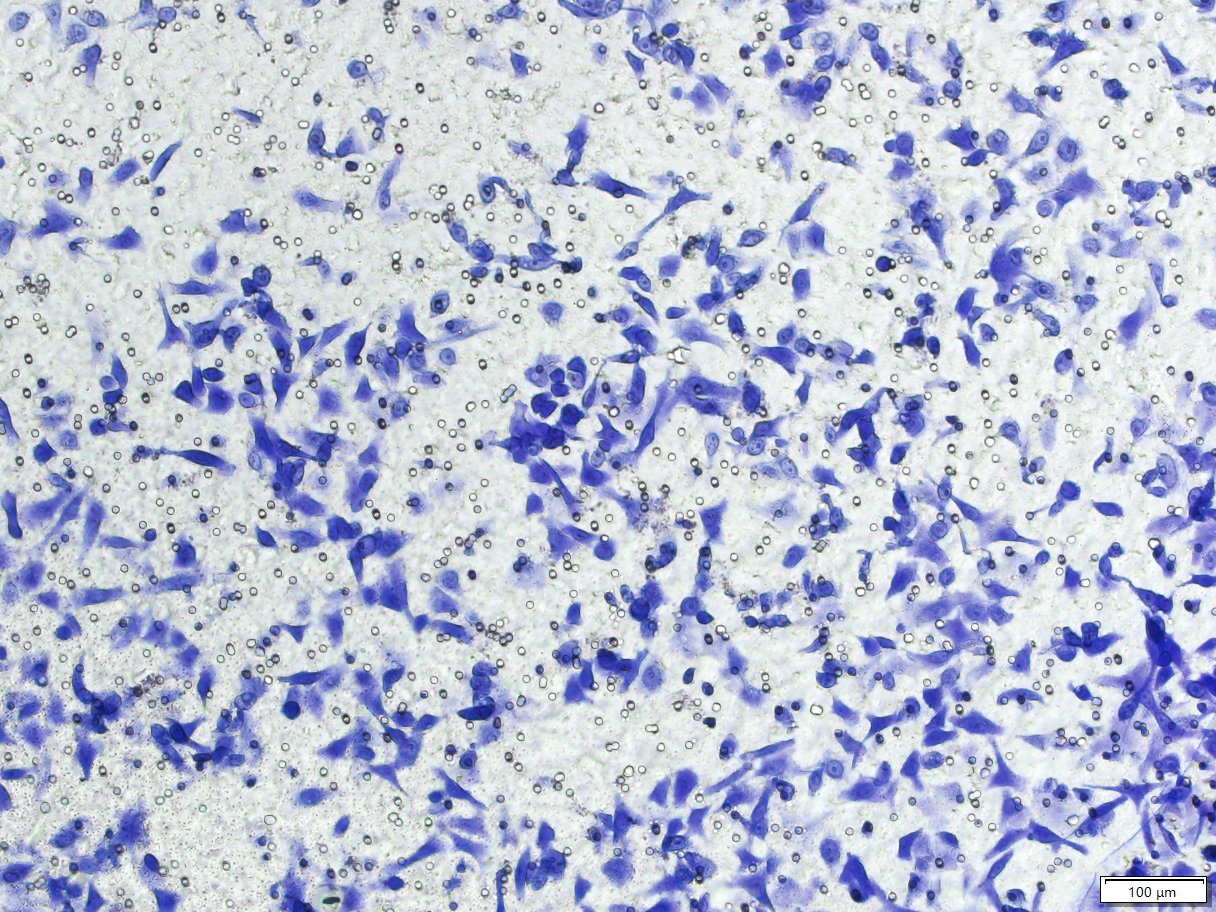

Supplement: Supplemental Information 2 [file peerj-cs-09-1651-s002.zip › Dataset 1/0-5.jpg]

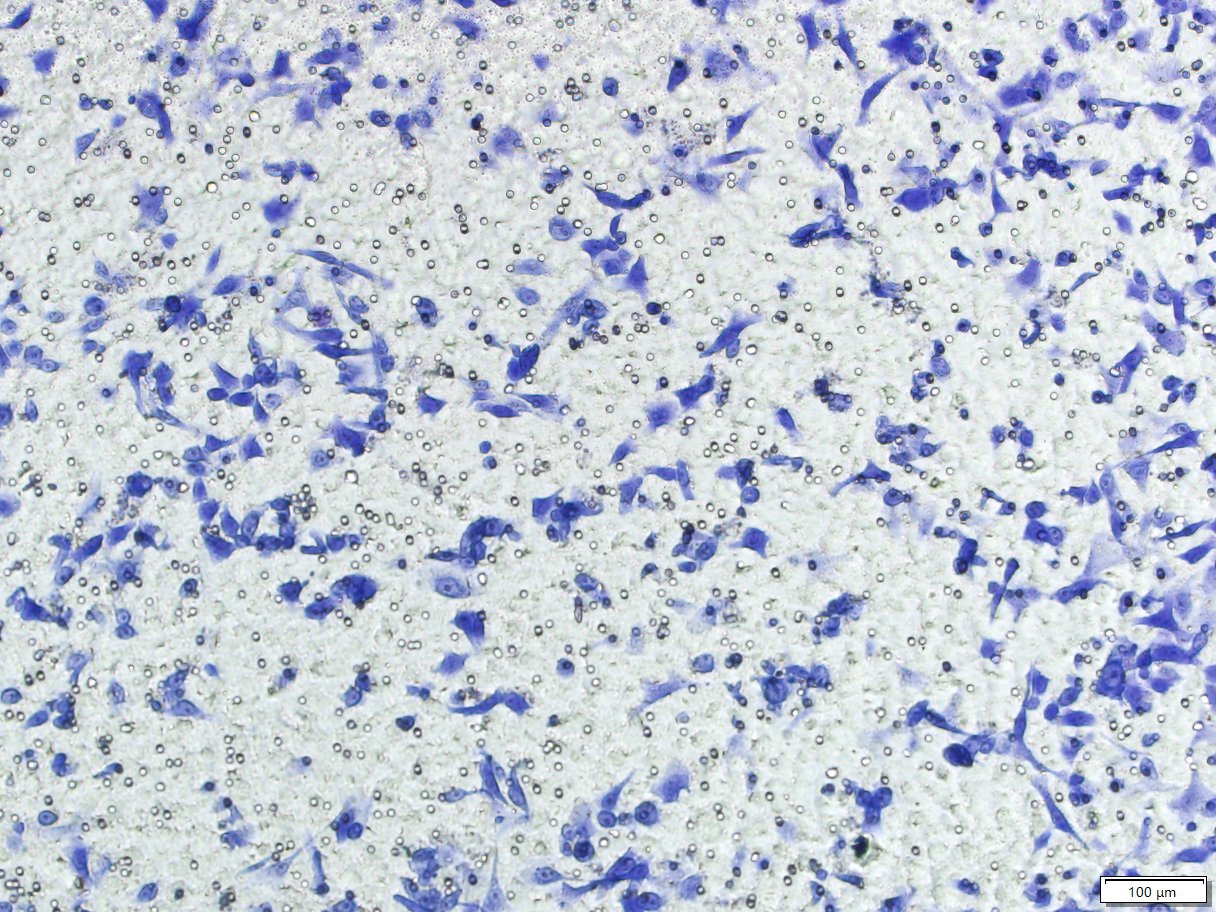

Supplement: Supplemental Information 2 [file peerj-cs-09-1651-s002.zip › Dataset 1/0-6.jpg]

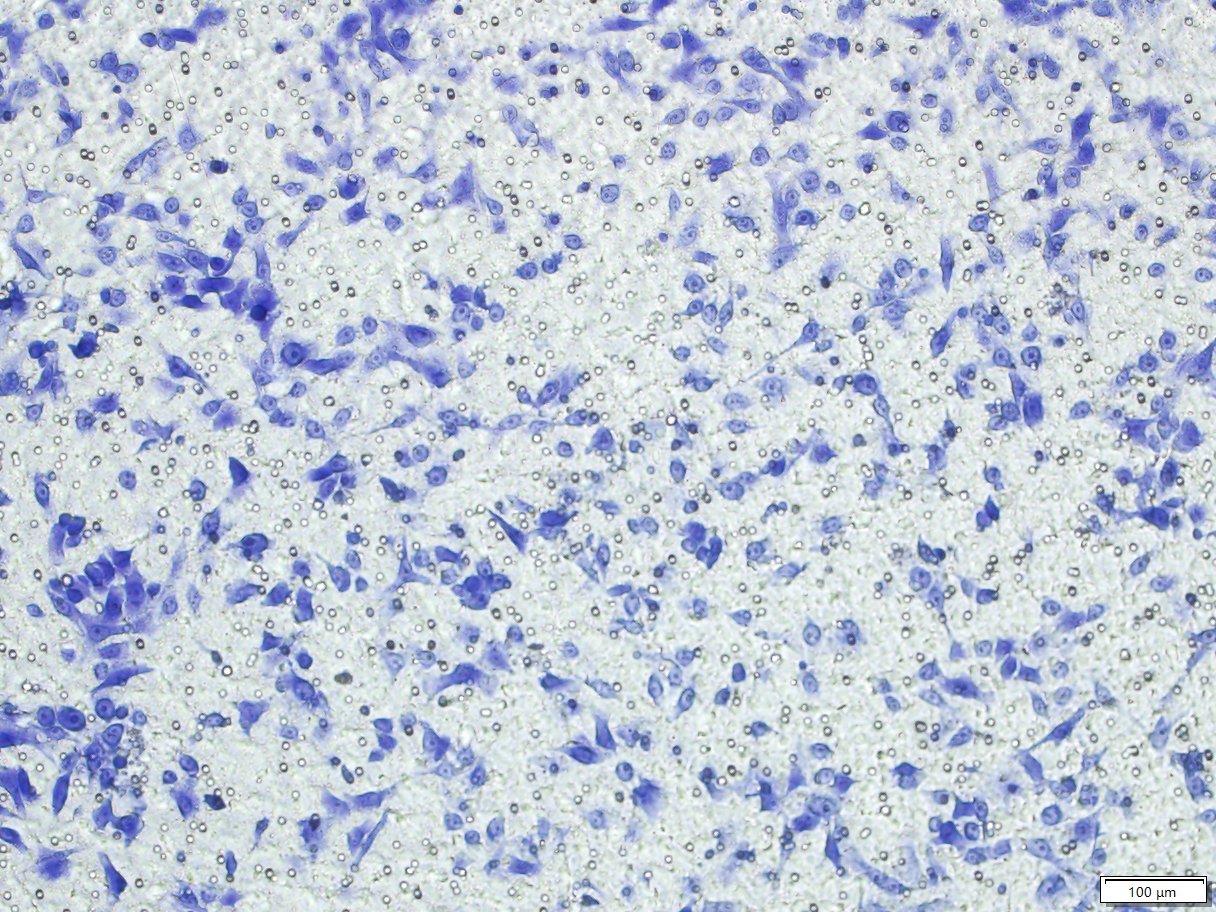

Supplement: Supplemental Information 2 [file peerj-cs-09-1651-s002.zip › Dataset 1/0-7.jpg]

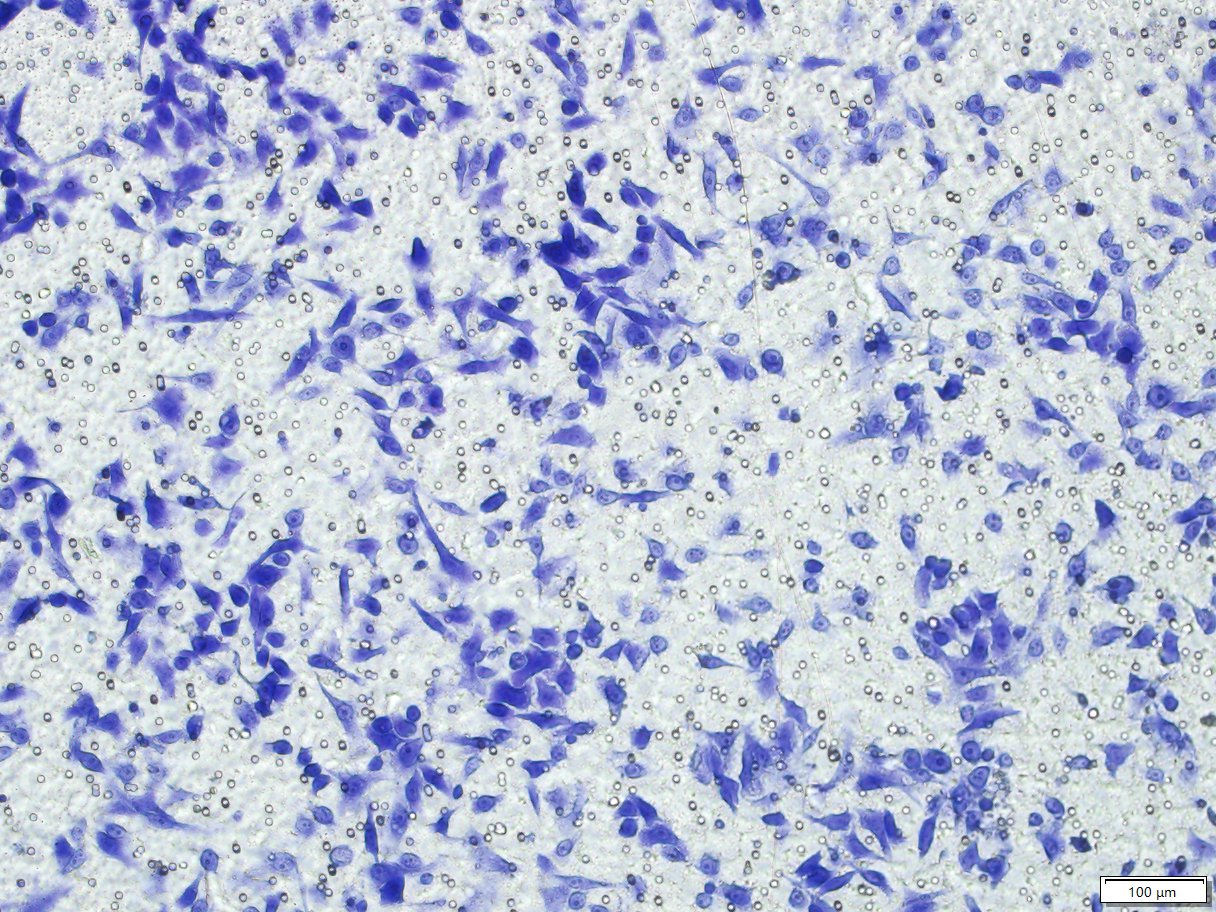

Supplement: Supplemental Information 2 [file peerj-cs-09-1651-s002.zip › Dataset 1/0-8.jpg]

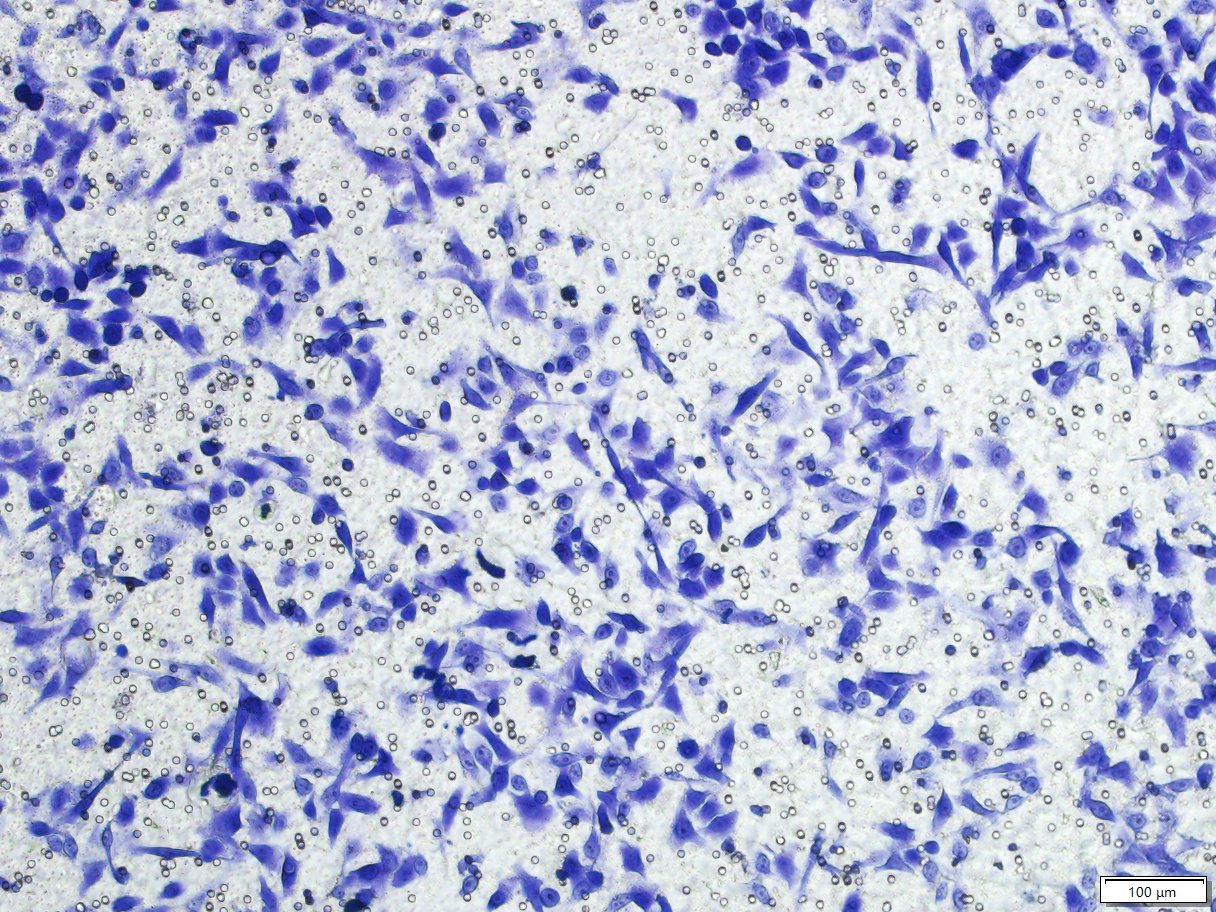

Supplement: Supplemental Information 2 [file peerj-cs-09-1651-s002.zip › Dataset 1/0-9.jpg]

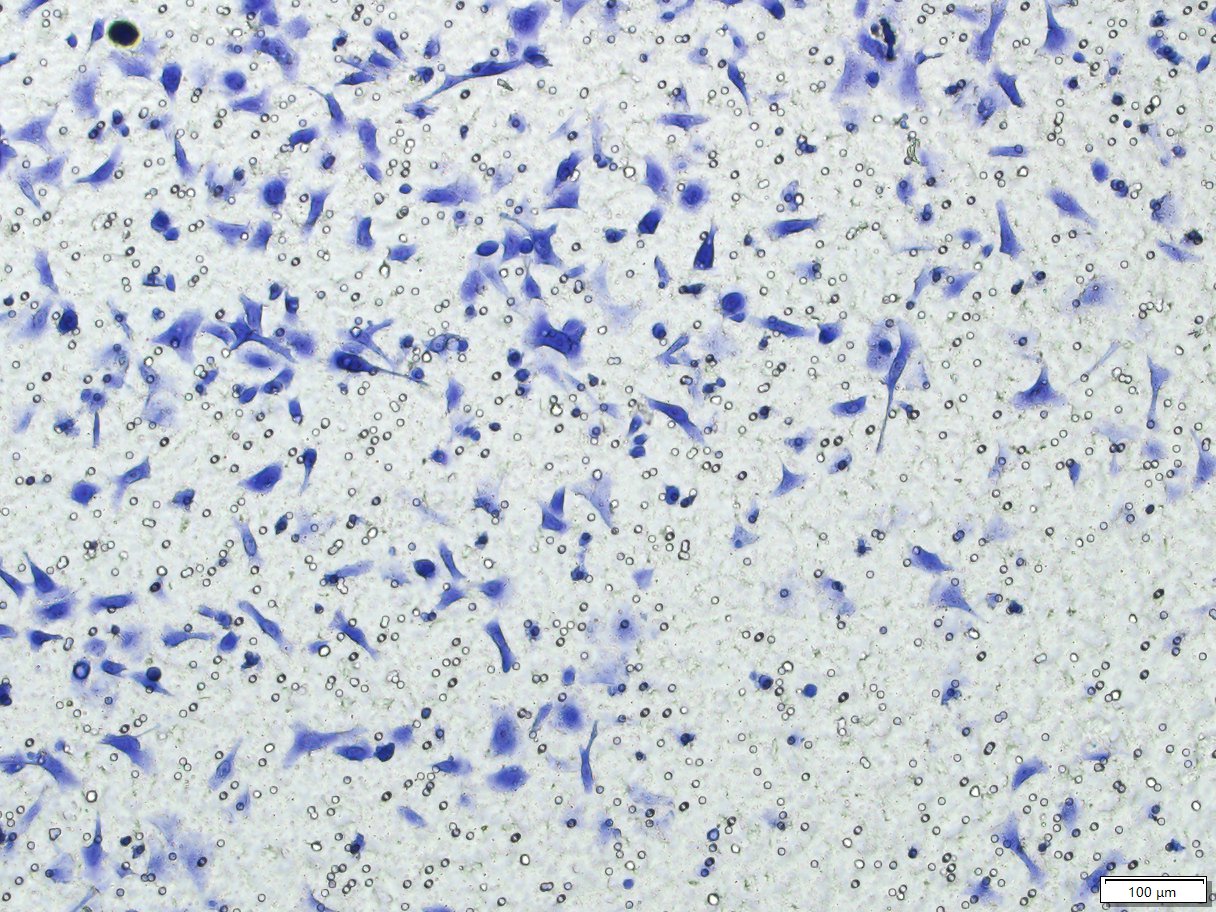

Supplement: Supplemental Information 2 [file peerj-cs-09-1651-s002.zip › Dataset 1/1+1.jpg]

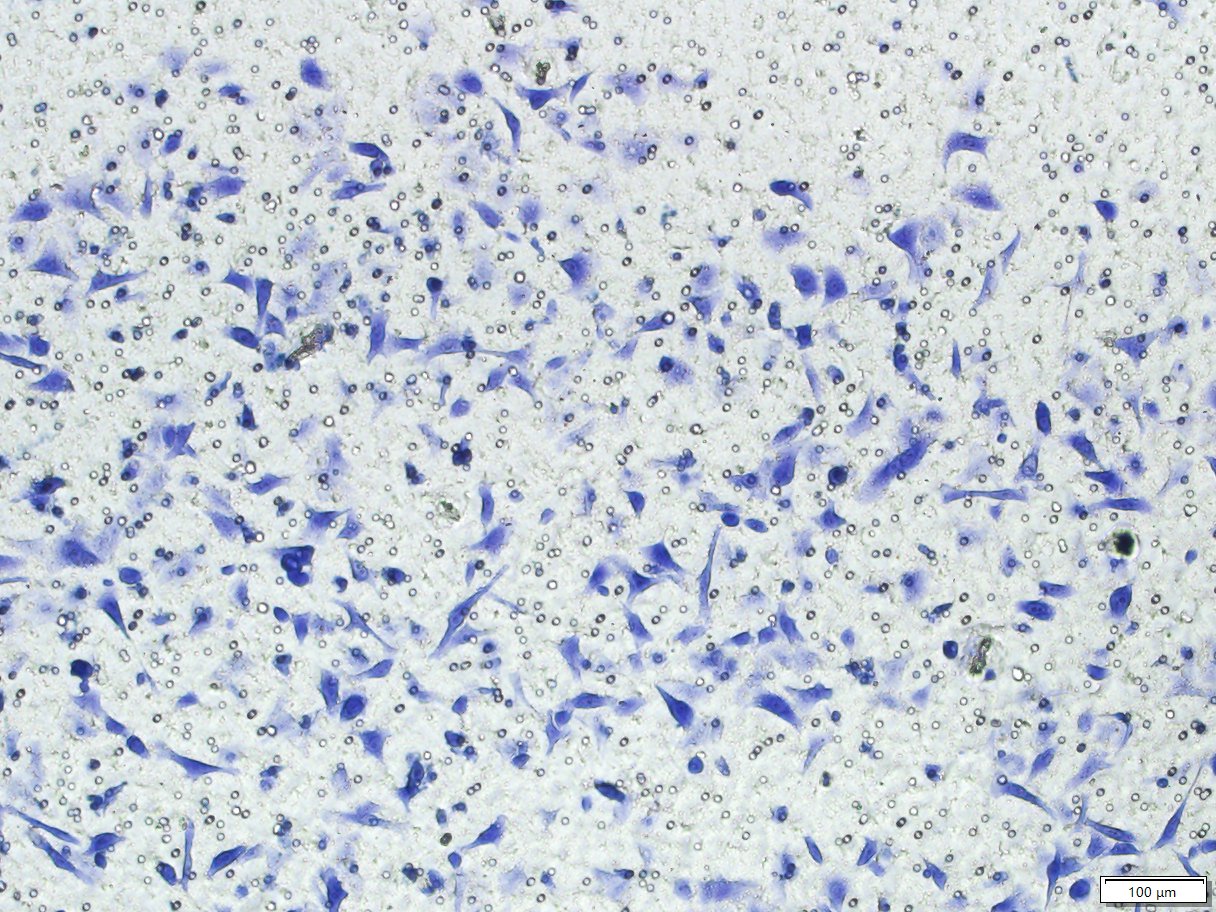

Supplement: Supplemental Information 2 [file peerj-cs-09-1651-s002.zip › Dataset 1/1+11.jpg]

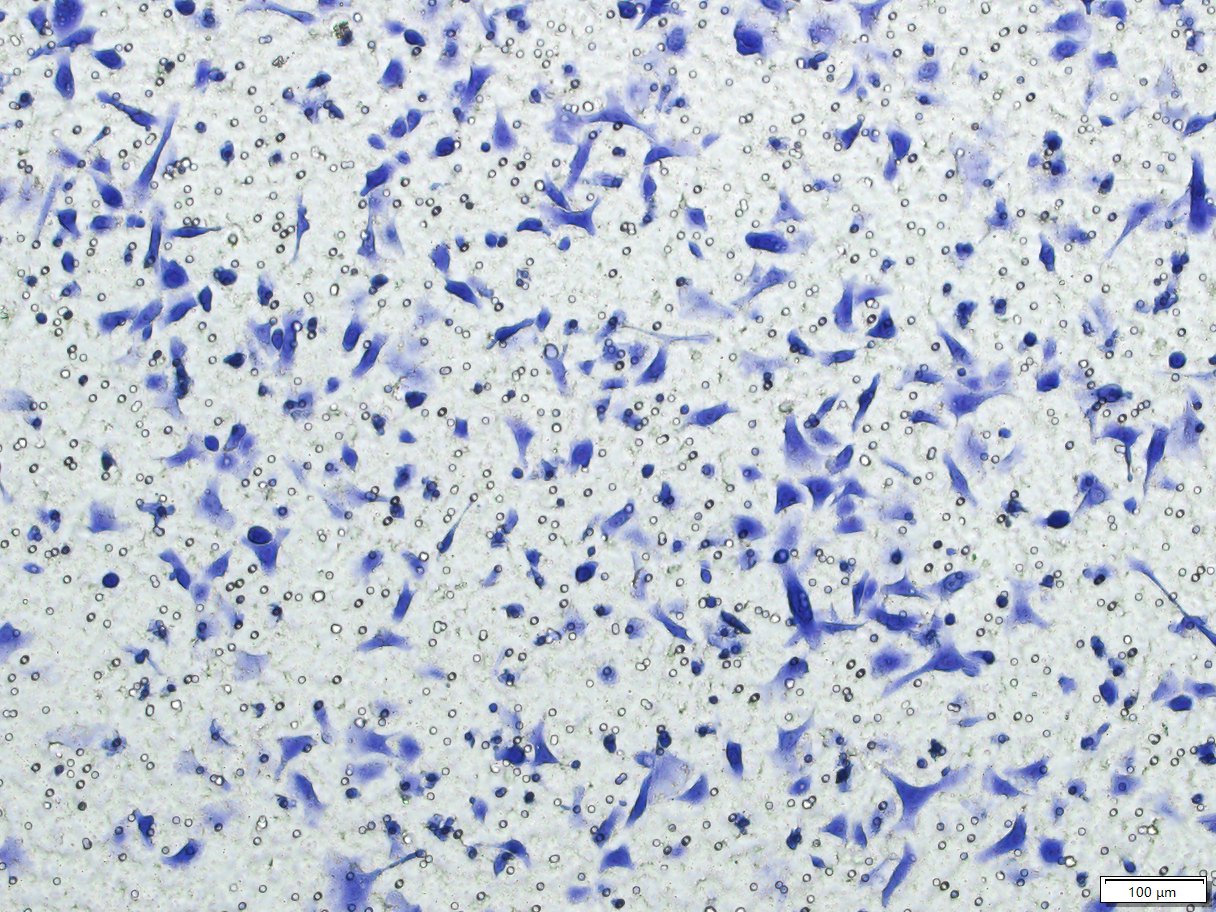

Supplement: Supplemental Information 2 [file peerj-cs-09-1651-s002.zip › Dataset 1/1+2.jpg]

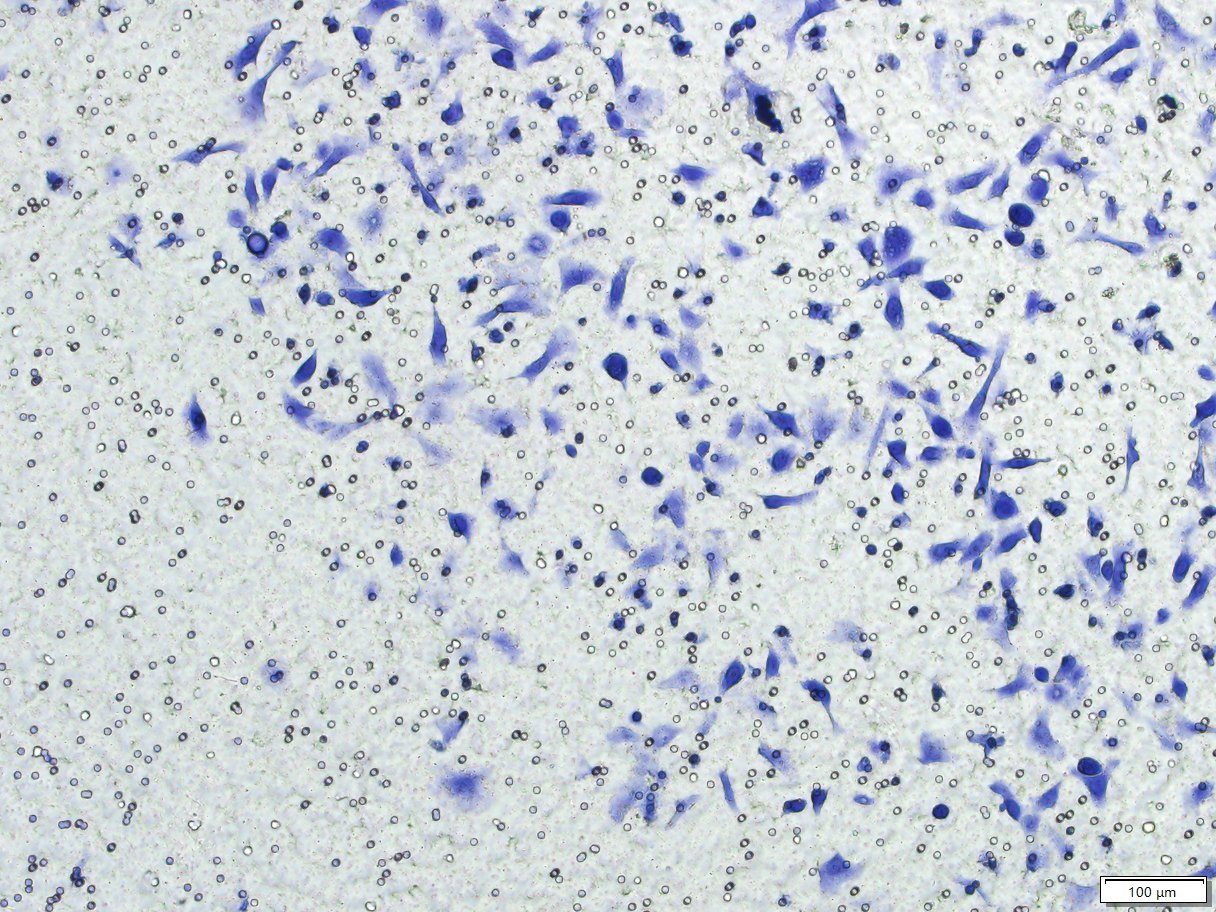

Supplement: Supplemental Information 2 [file peerj-cs-09-1651-s002.zip › Dataset 1/1+3.jpg]

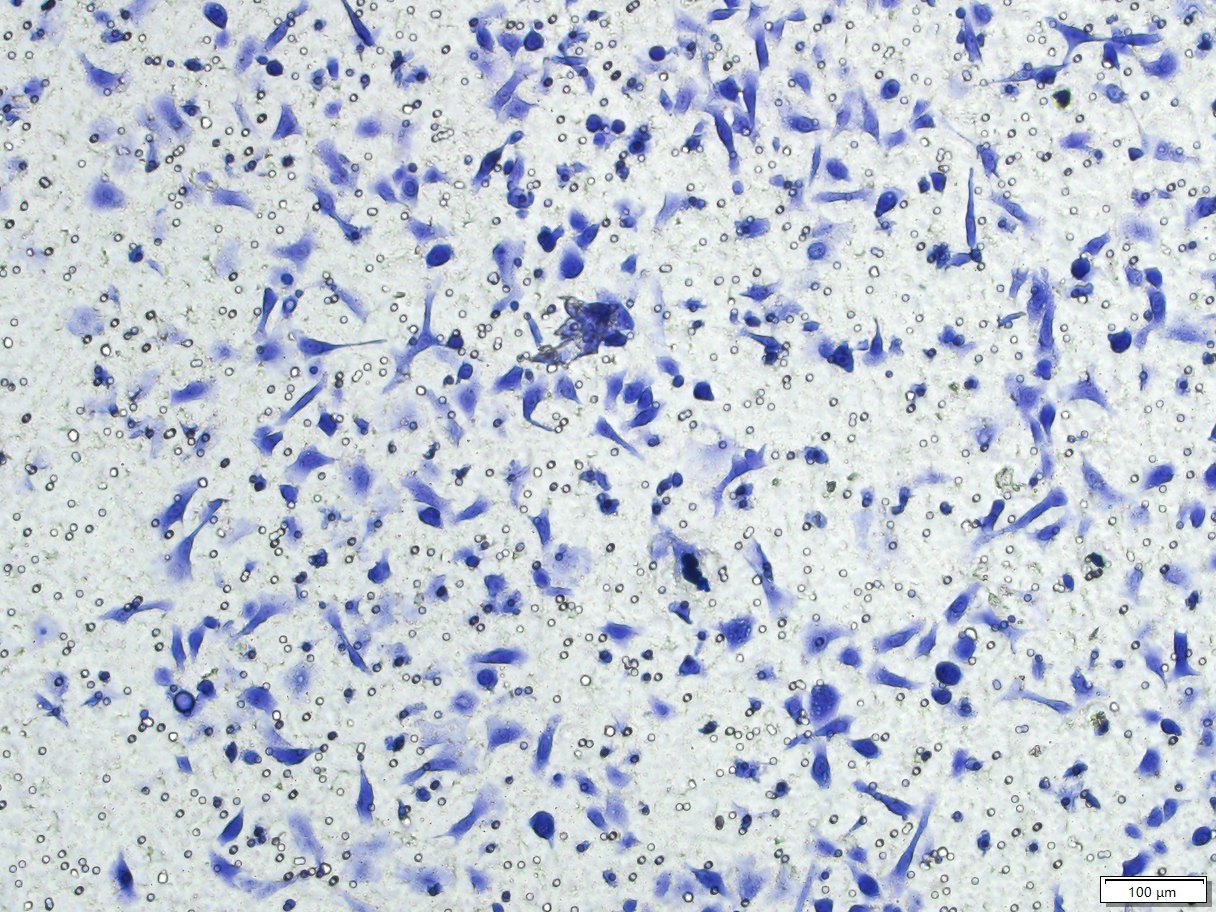

Supplement: Supplemental Information 2 [file peerj-cs-09-1651-s002.zip › Dataset 1/1+4.jpg]

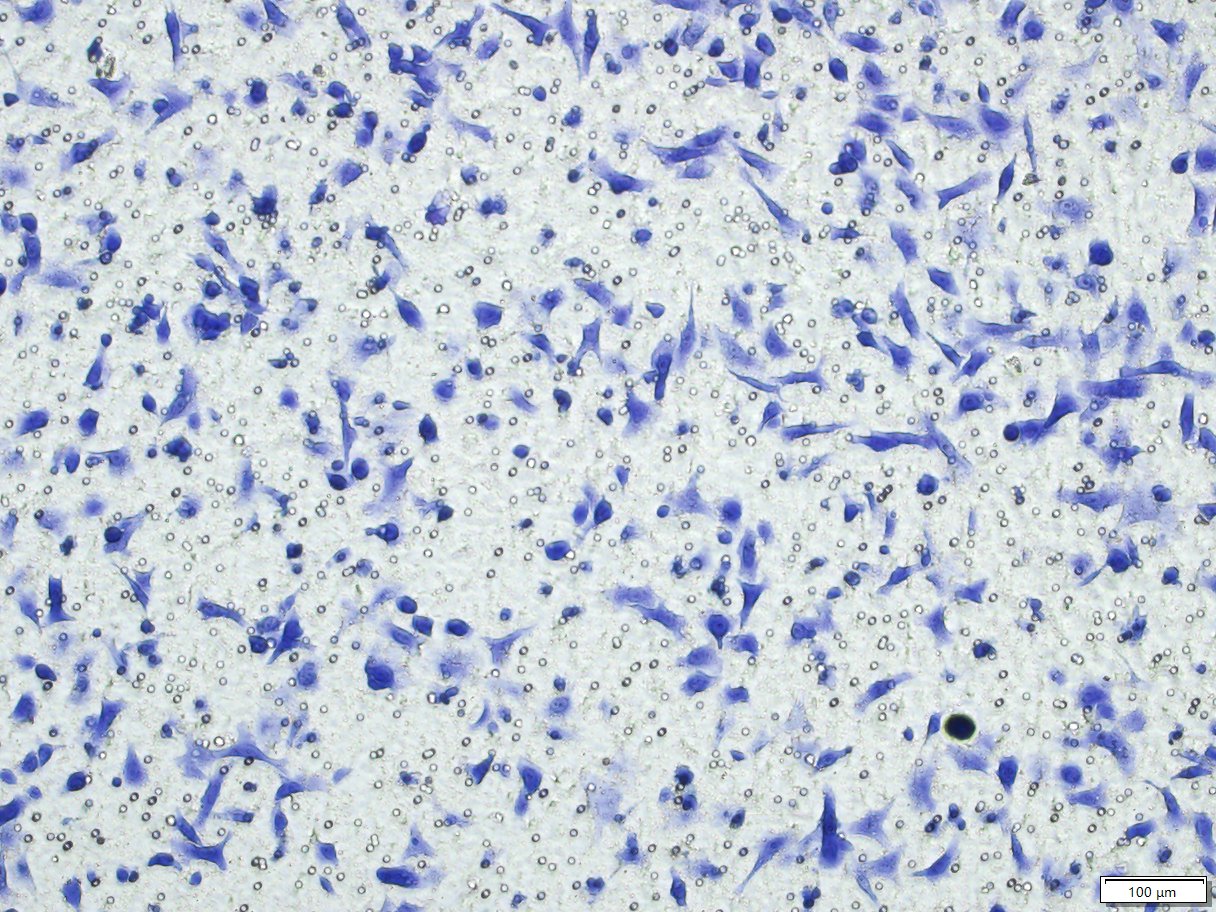

Supplement: Supplemental Information 2 [file peerj-cs-09-1651-s002.zip › Dataset 1/1+5.jpg]

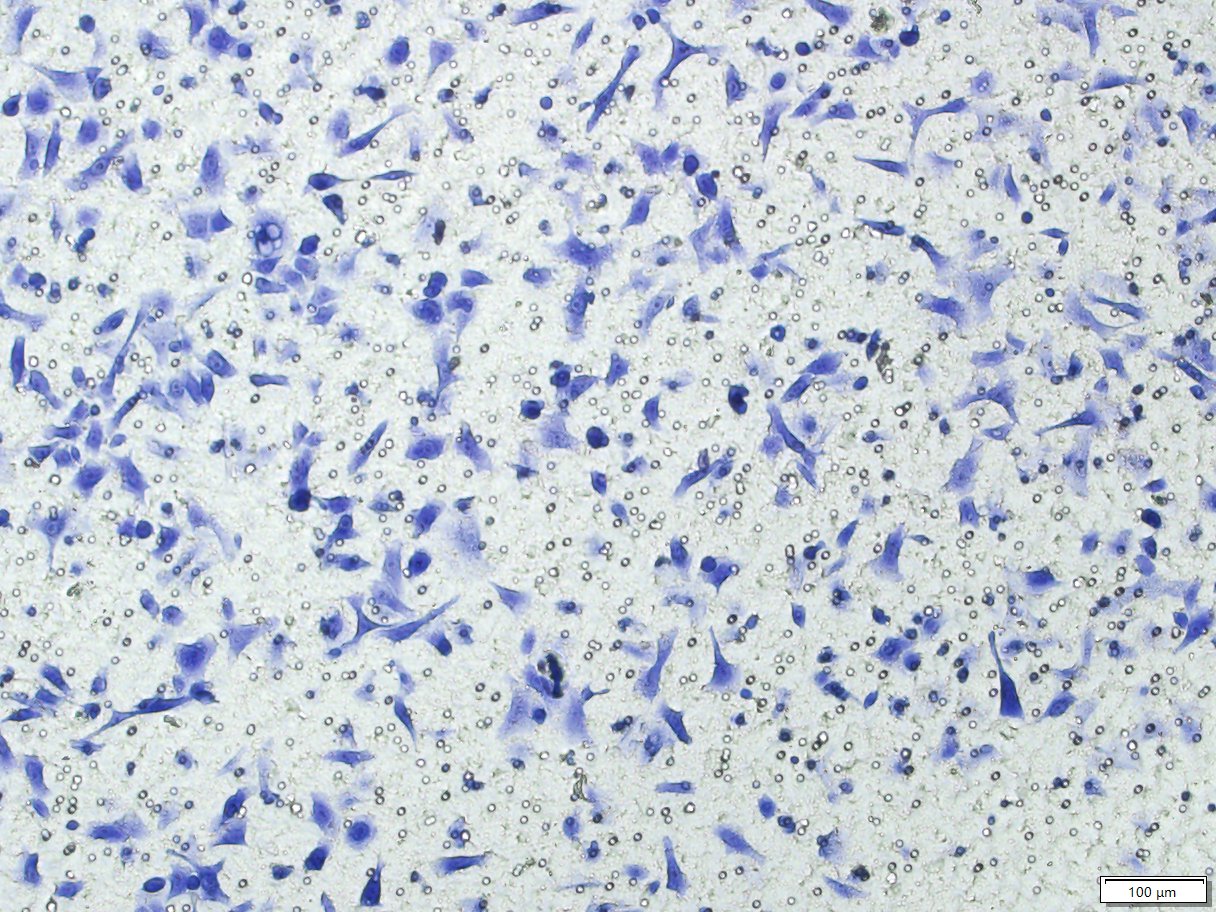

Supplement: Supplemental Information 2 [file peerj-cs-09-1651-s002.zip › Dataset 1/1+6.jpg]

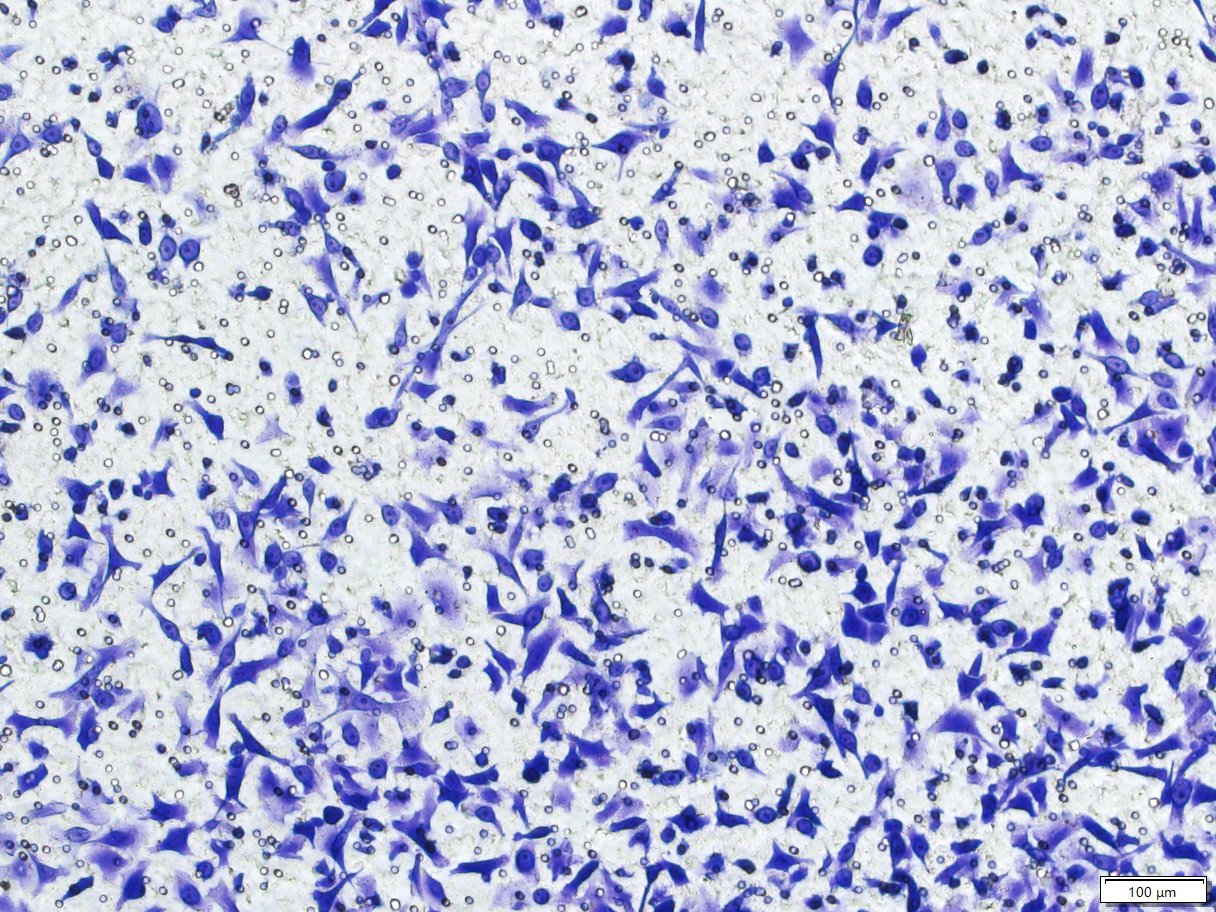

Supplement: Supplemental Information 2 [file peerj-cs-09-1651-s002.zip › Dataset 1/1-1.jpg]

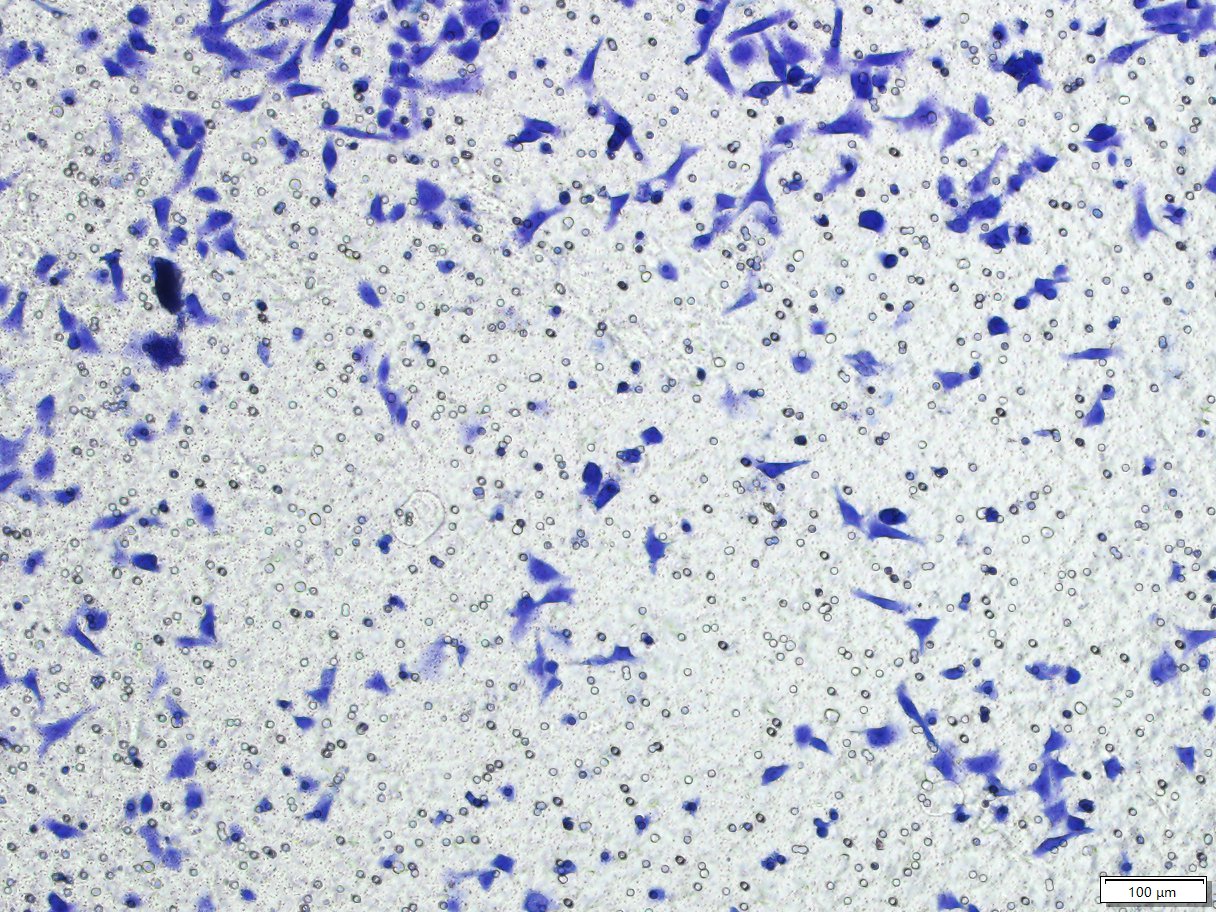

Supplement: Supplemental Information 2 [file peerj-cs-09-1651-s002.zip › Dataset 1/1-10.jpg]

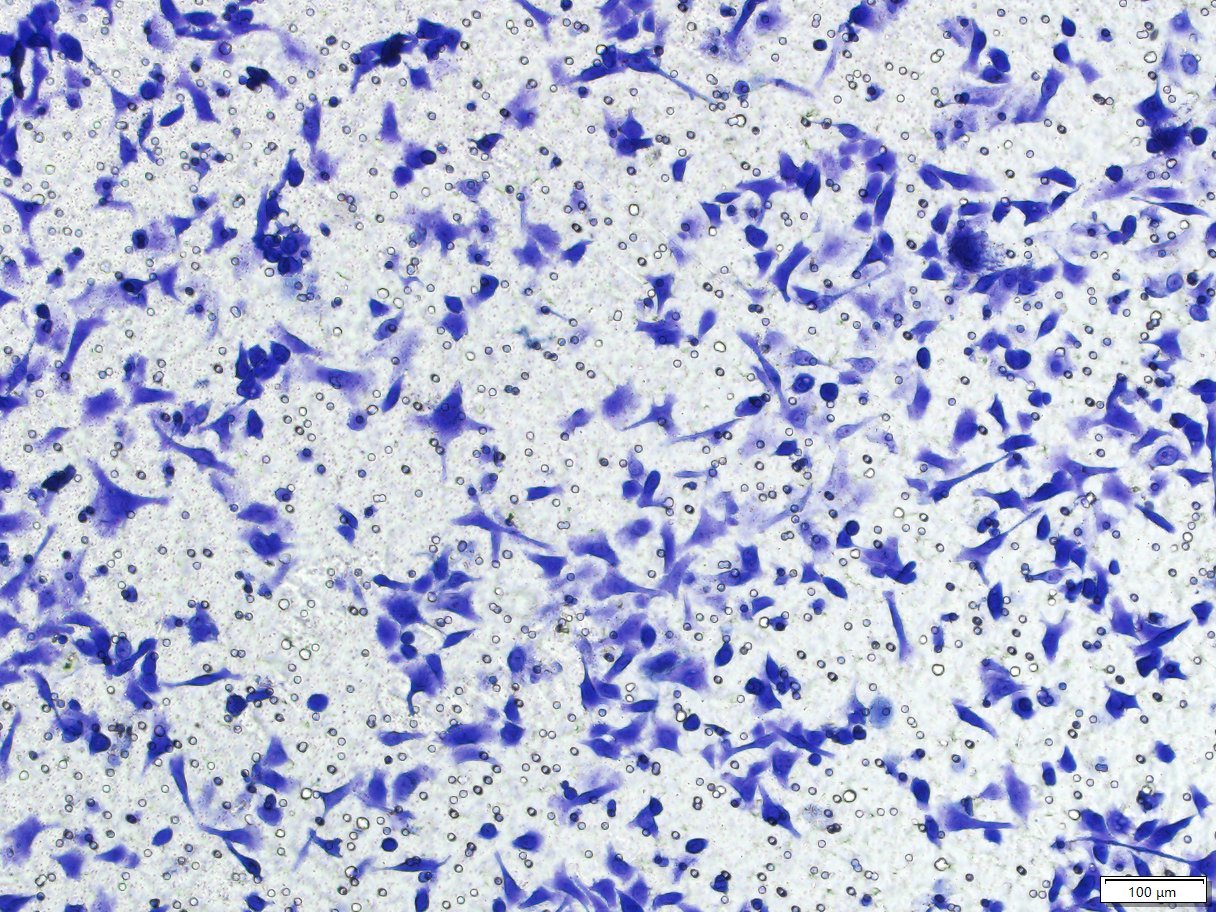

Supplement: Supplemental Information 2 [file peerj-cs-09-1651-s002.zip › Dataset 1/1-11.jpg]

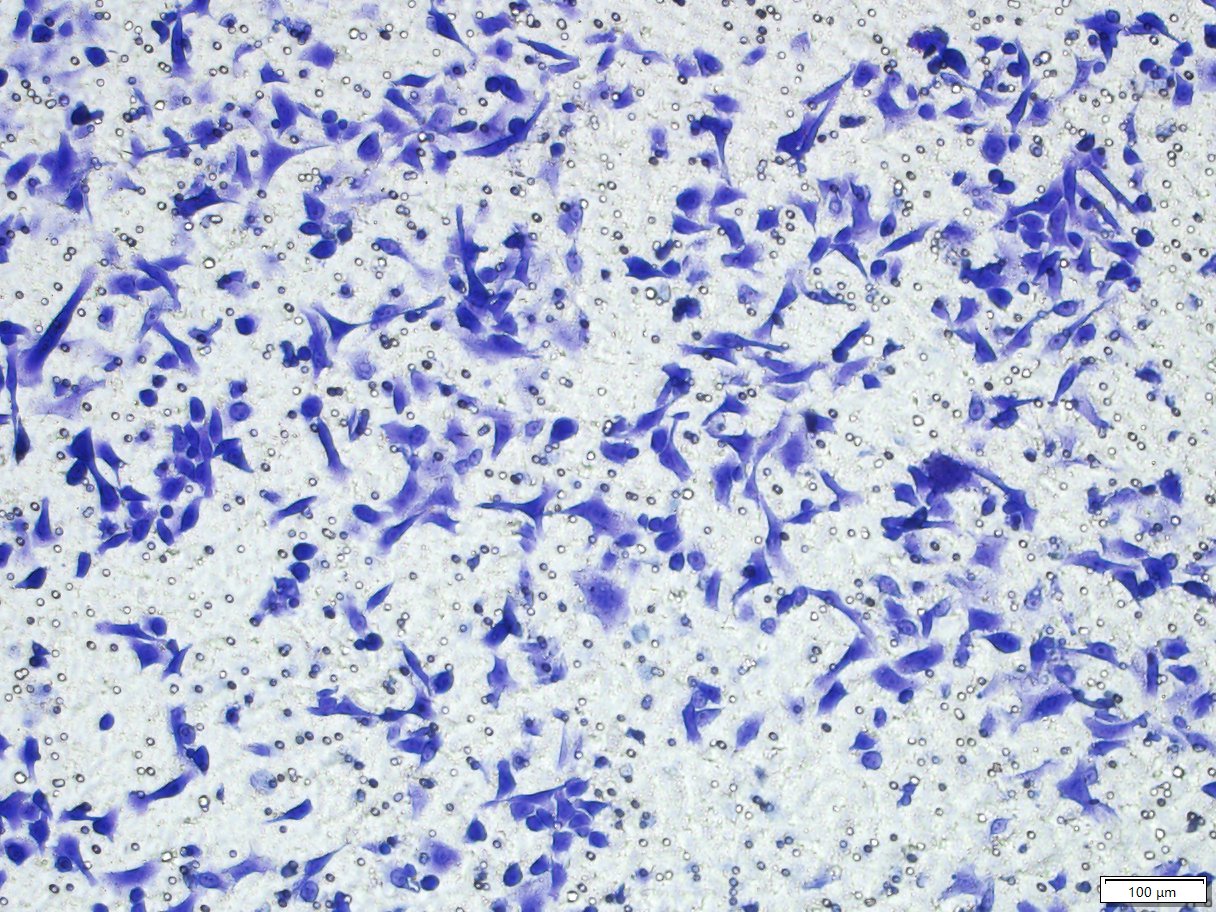

Supplement: Supplemental Information 2 [file peerj-cs-09-1651-s002.zip › Dataset 1/1-12.jpg]

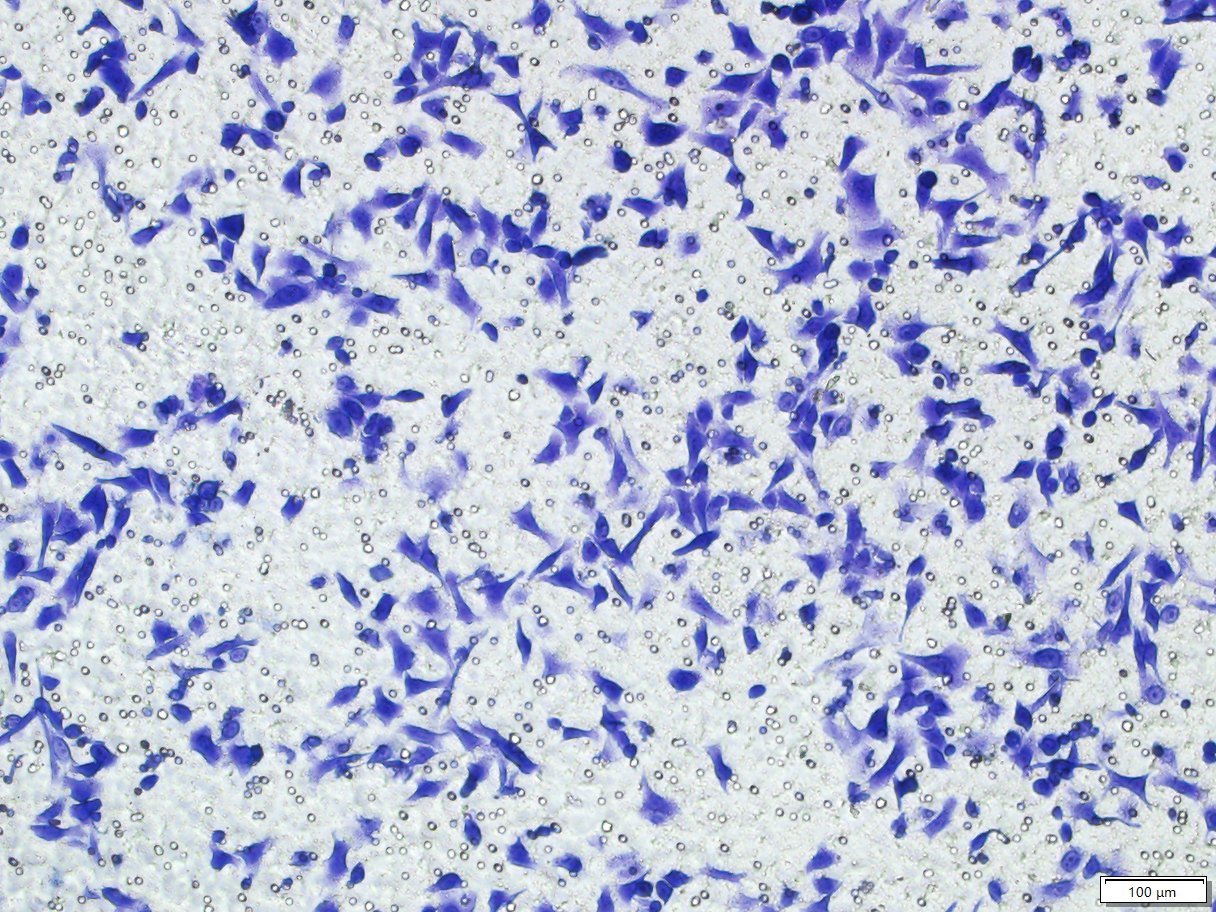

Supplement: Supplemental Information 2 [file peerj-cs-09-1651-s002.zip › Dataset 1/1-13.jpg]

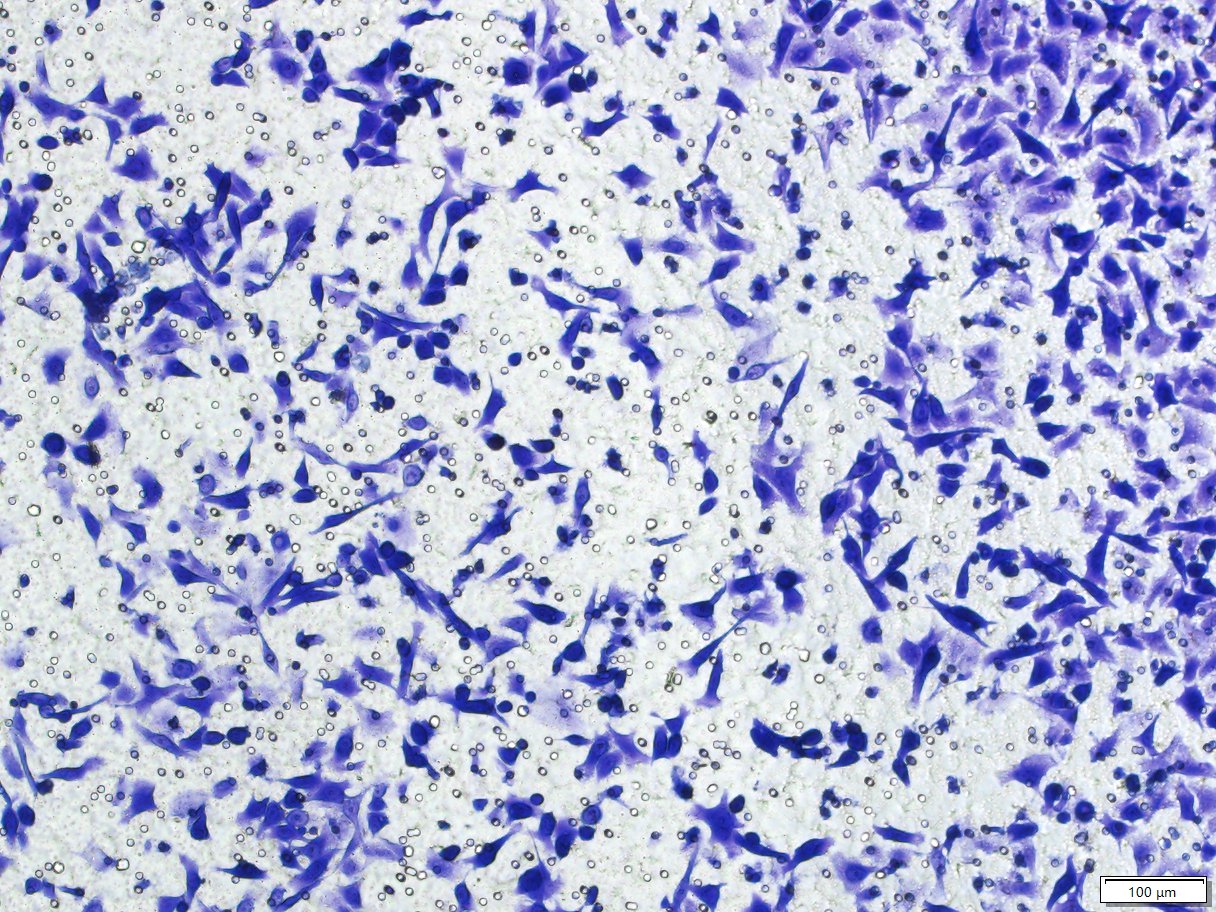

Supplement: Supplemental Information 2 [file peerj-cs-09-1651-s002.zip › Dataset 1/1-14.jpg]

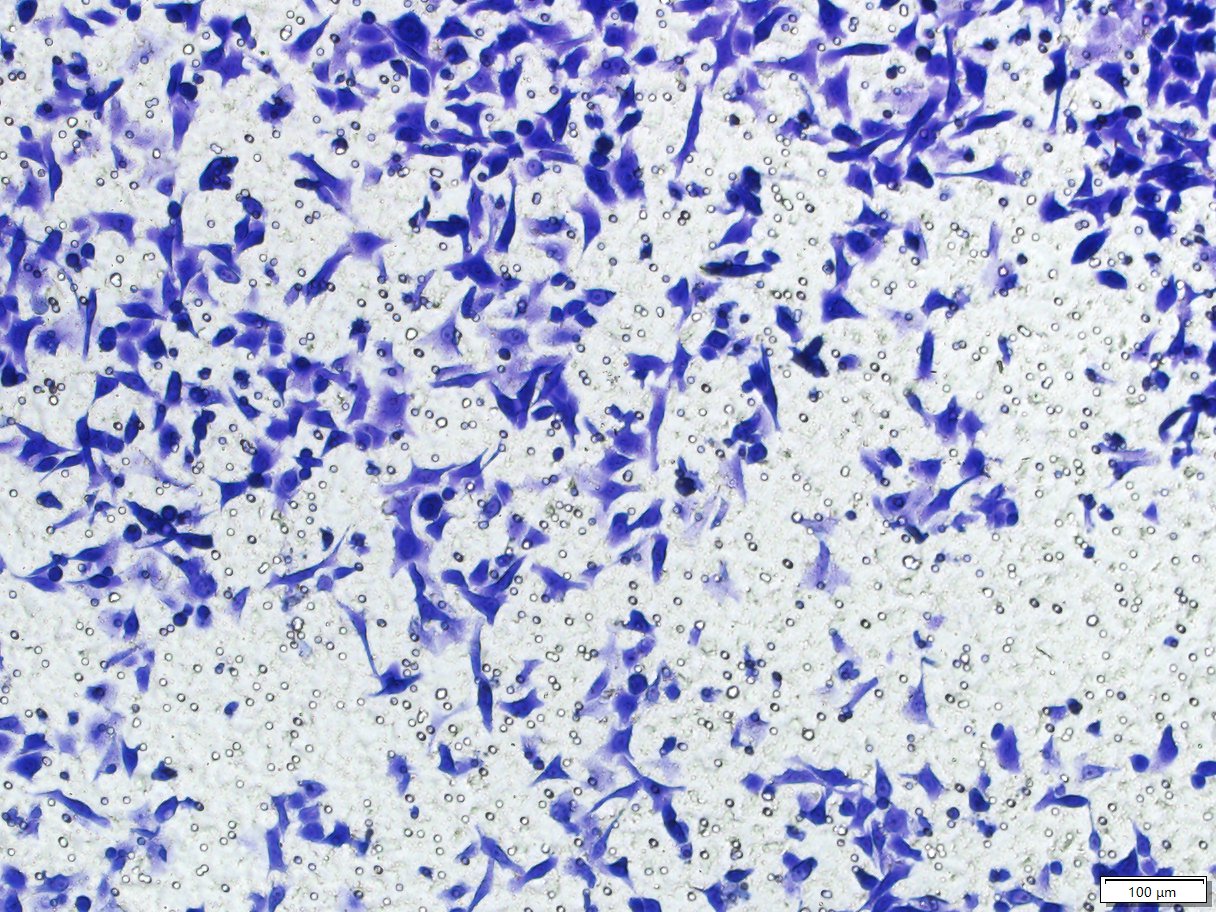

Supplement: Supplemental Information 2 [file peerj-cs-09-1651-s002.zip › Dataset 1/1-15.jpg]

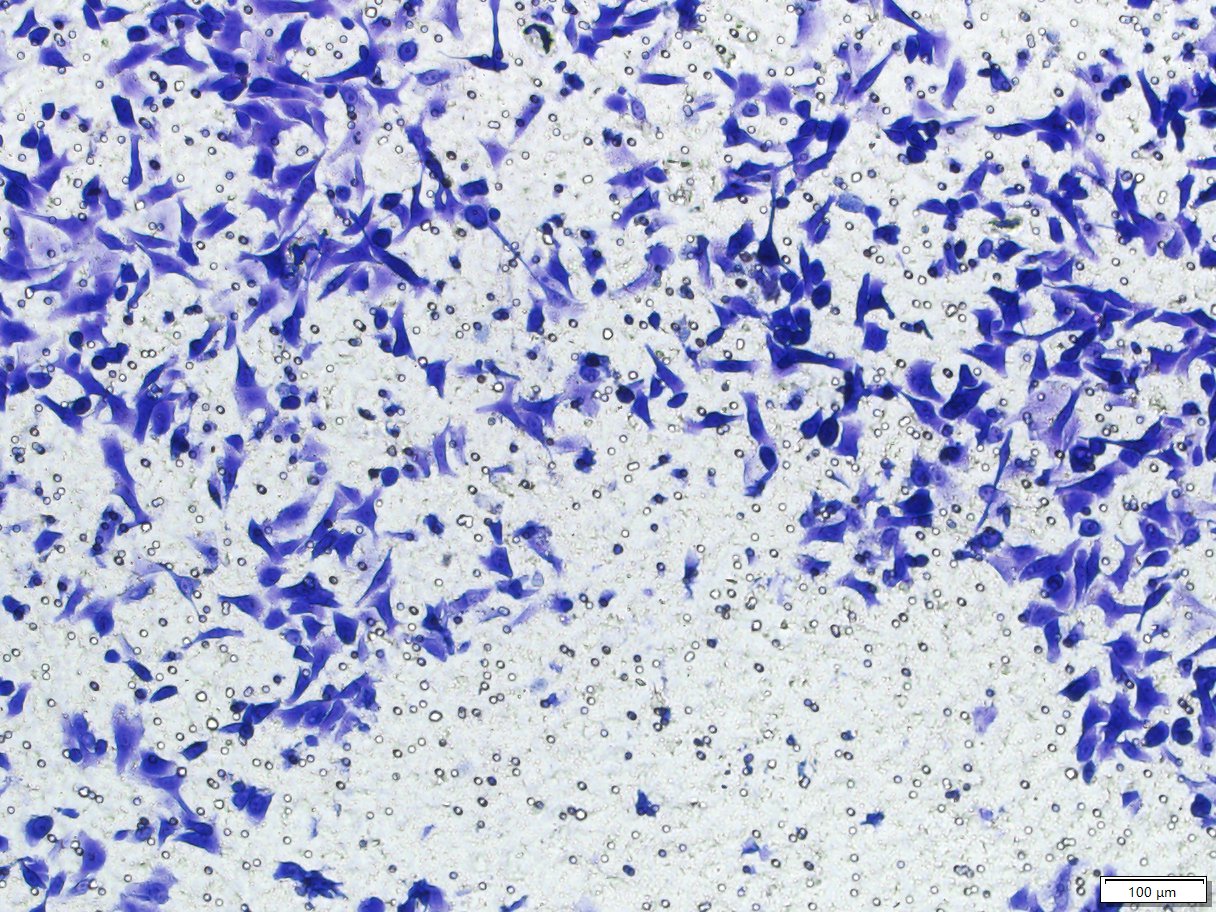

Supplement: Supplemental Information 2 [file peerj-cs-09-1651-s002.zip › Dataset 1/1-16.jpg]

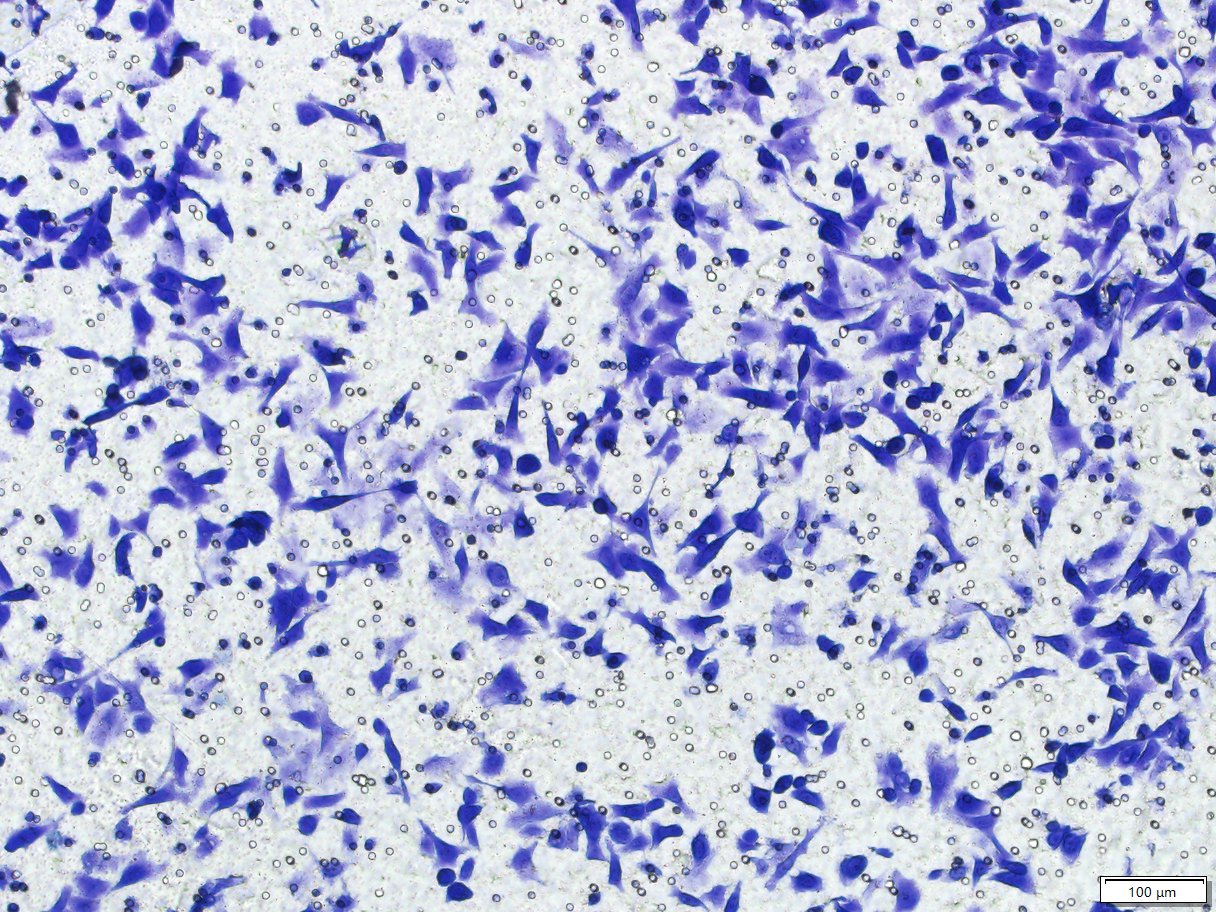

Supplement: Supplemental Information 2 [file peerj-cs-09-1651-s002.zip › Dataset 1/1-17.jpg]

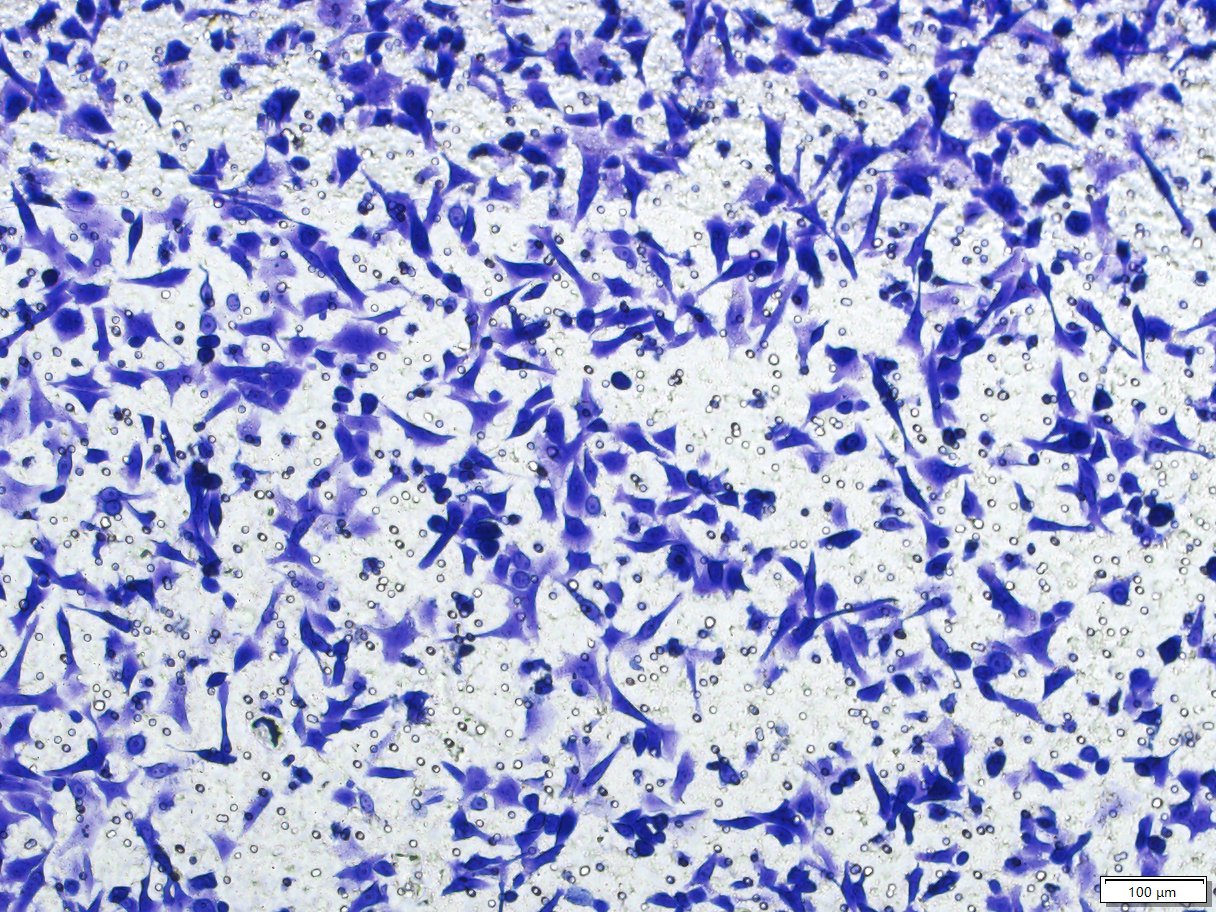

Supplement: Supplemental Information 2 [file peerj-cs-09-1651-s002.zip › Dataset 1/1-18.jpg]

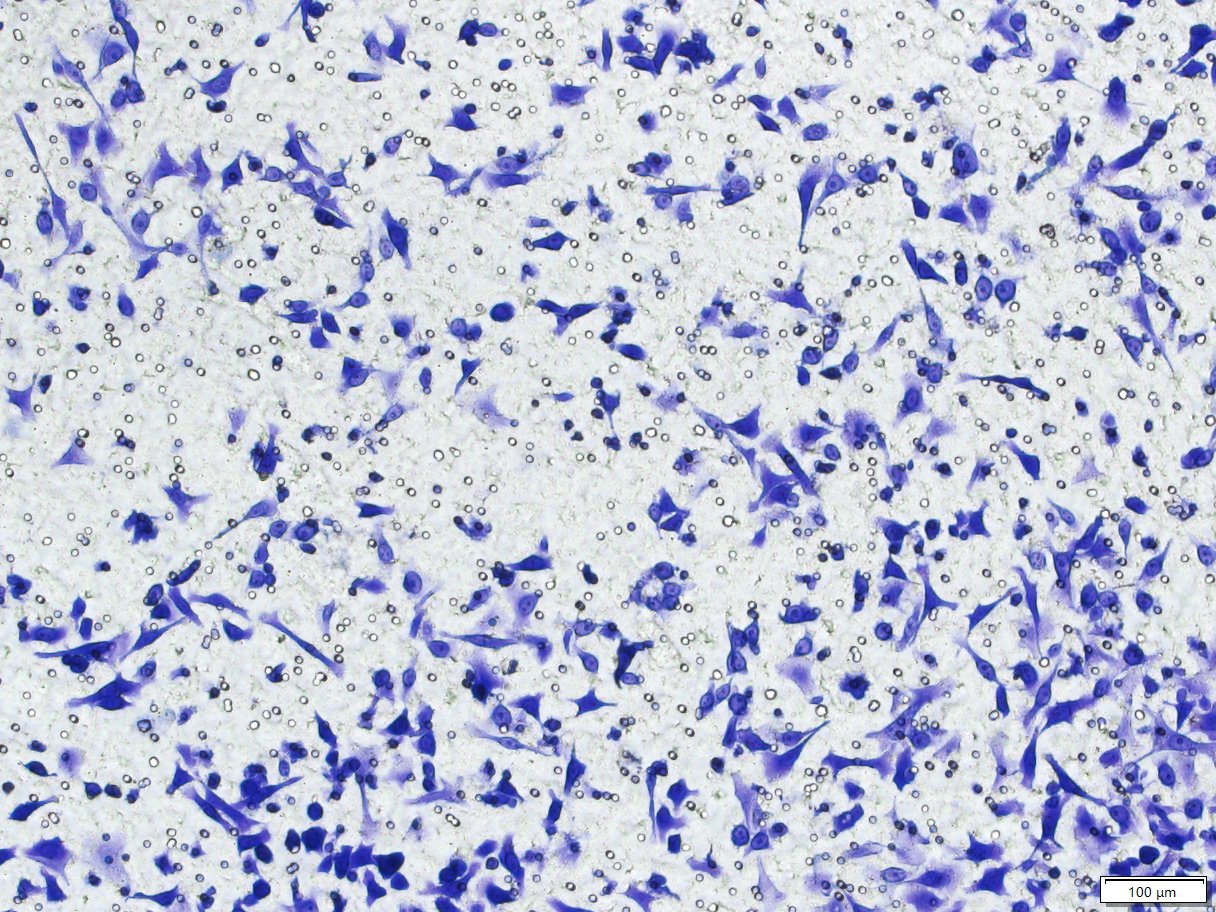

Supplement: Supplemental Information 2 [file peerj-cs-09-1651-s002.zip › Dataset 1/1-2.jpg]

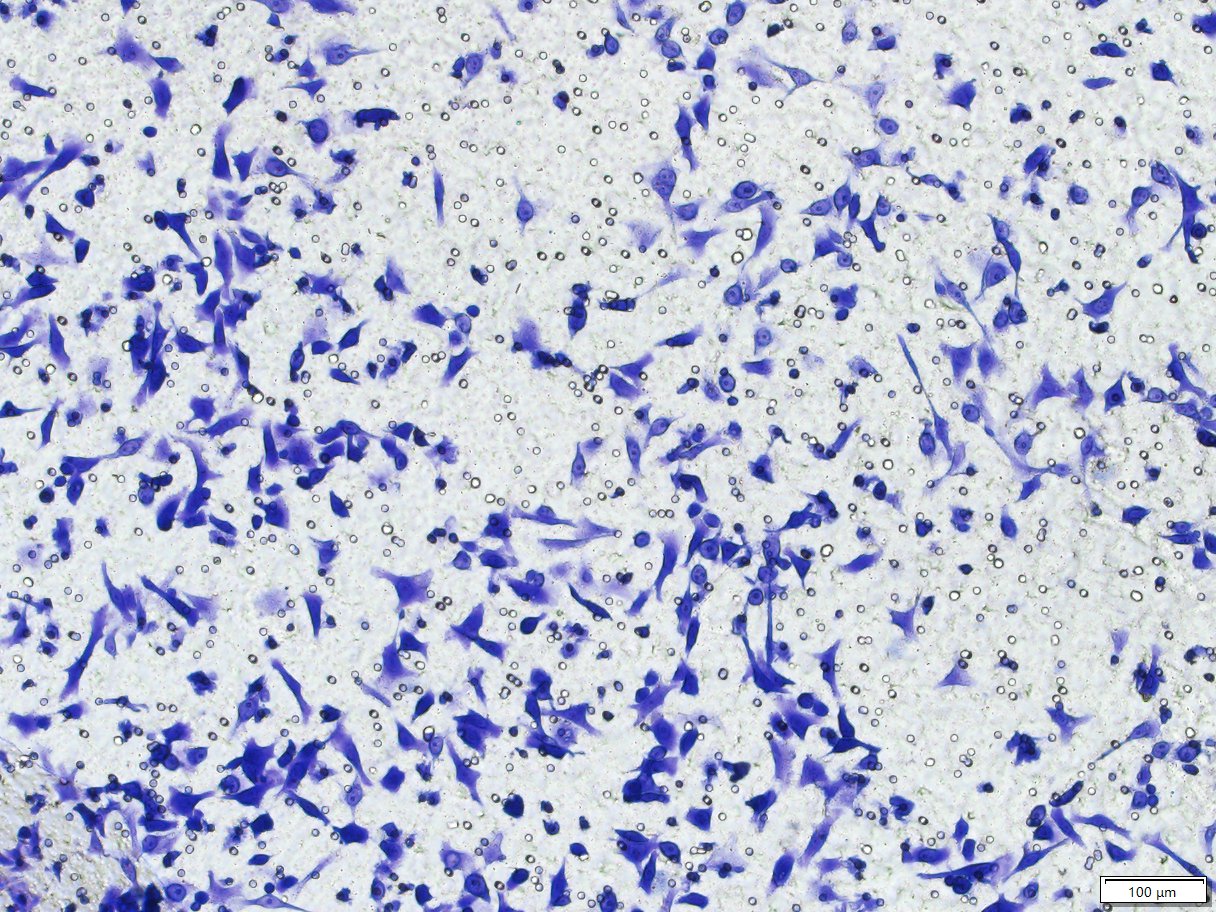

Supplement: Supplemental Information 2 [file peerj-cs-09-1651-s002.zip › Dataset 1/1-3.jpg]

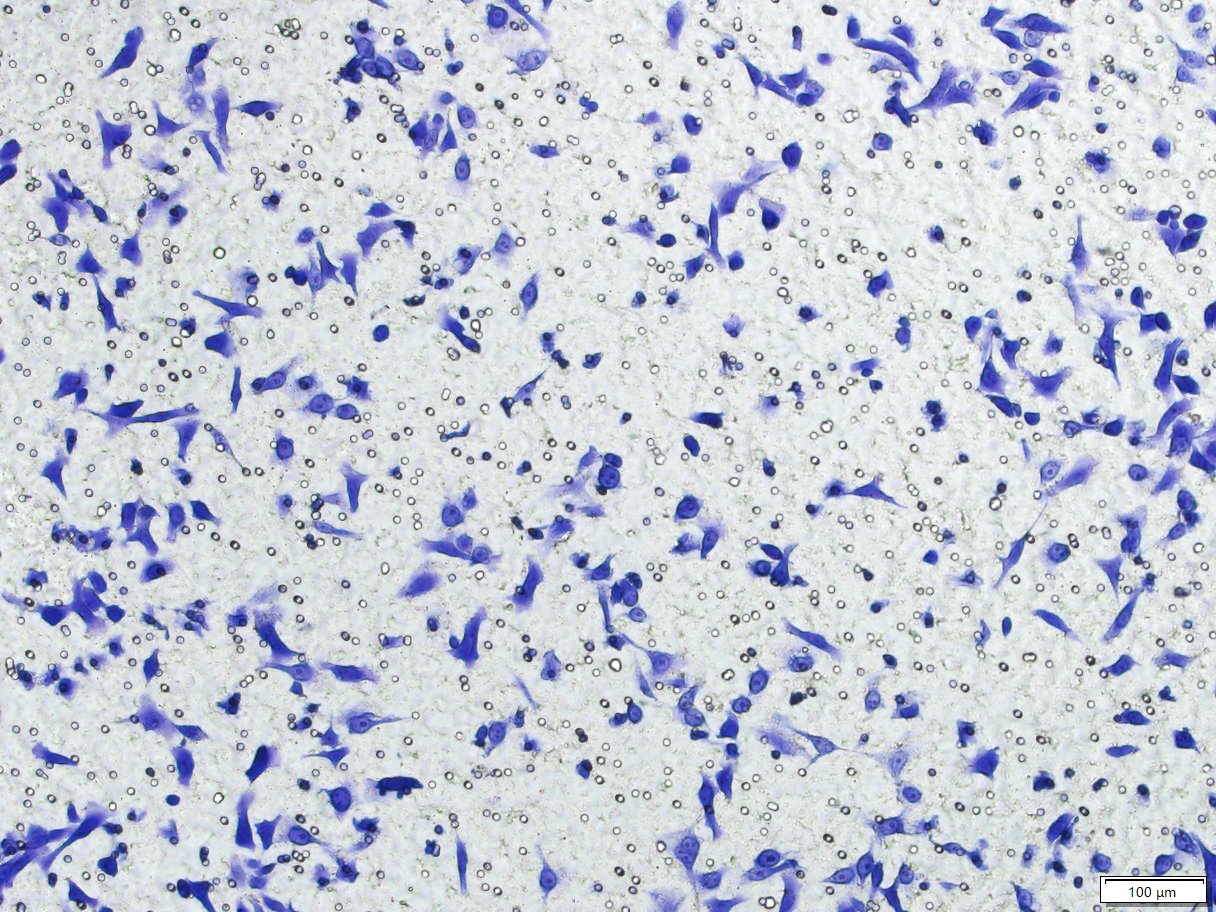

Supplement: Supplemental Information 2 [file peerj-cs-09-1651-s002.zip › Dataset 1/1-4.jpg]

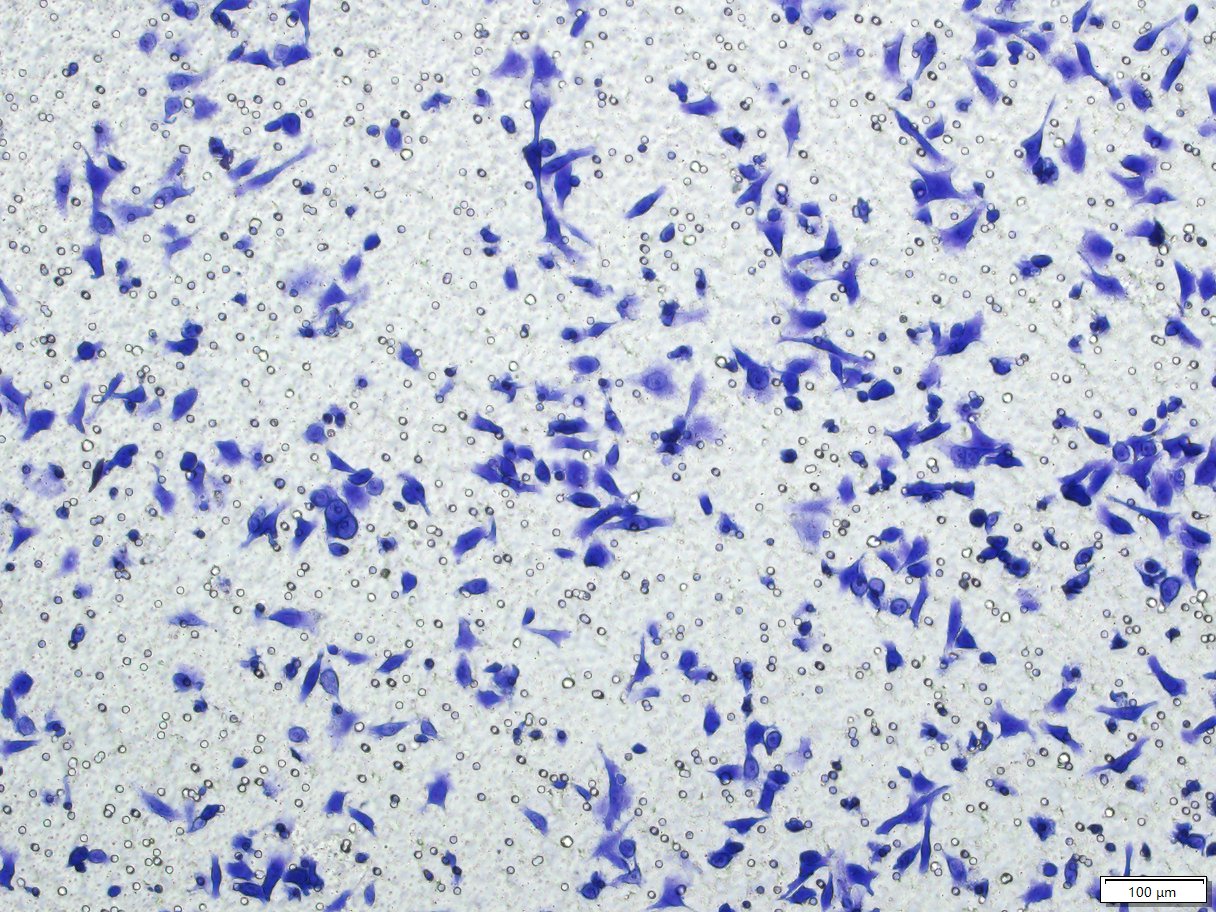

Supplement: Supplemental Information 2 [file peerj-cs-09-1651-s002.zip › Dataset 1/1-5.jpg]

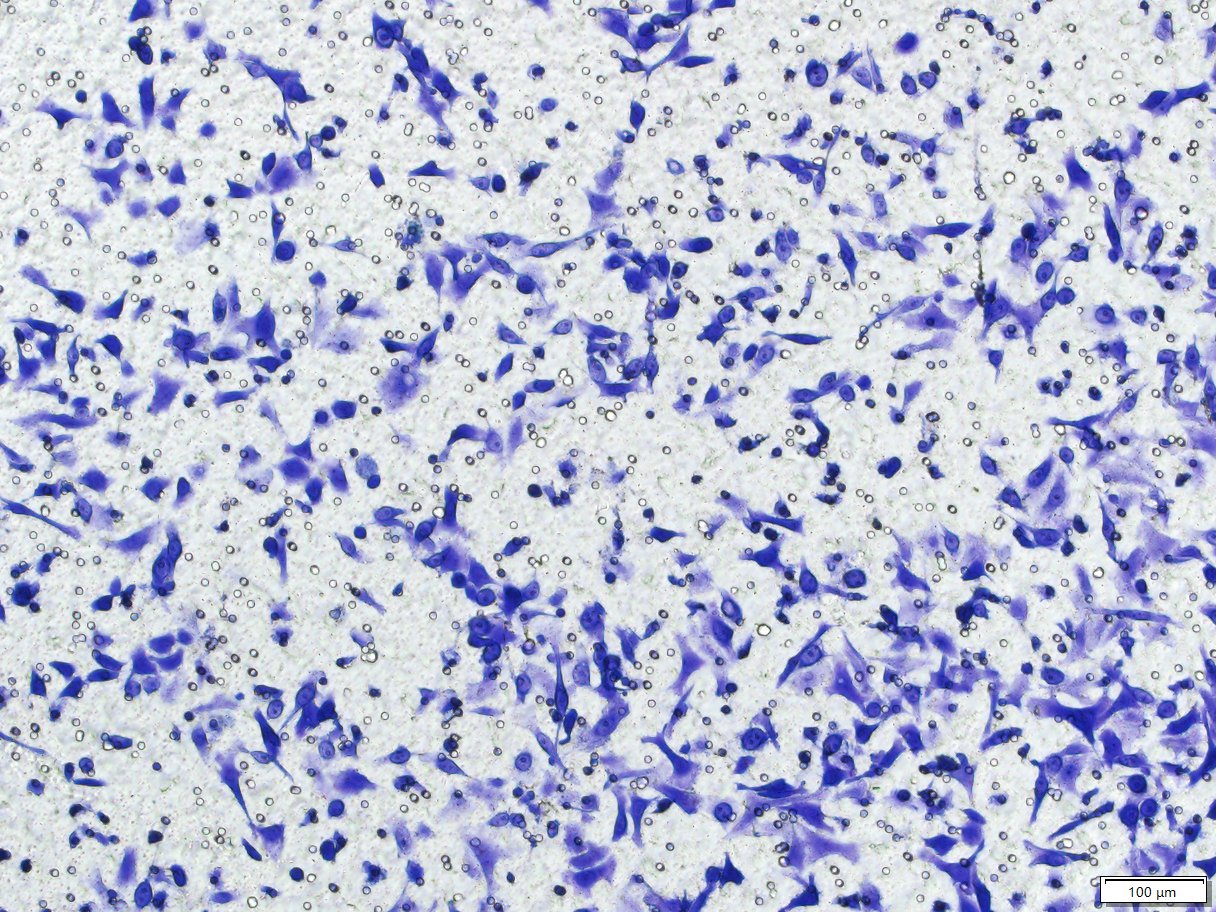

Supplement: Supplemental Information 2 [file peerj-cs-09-1651-s002.zip › Dataset 1/1-6.jpg]

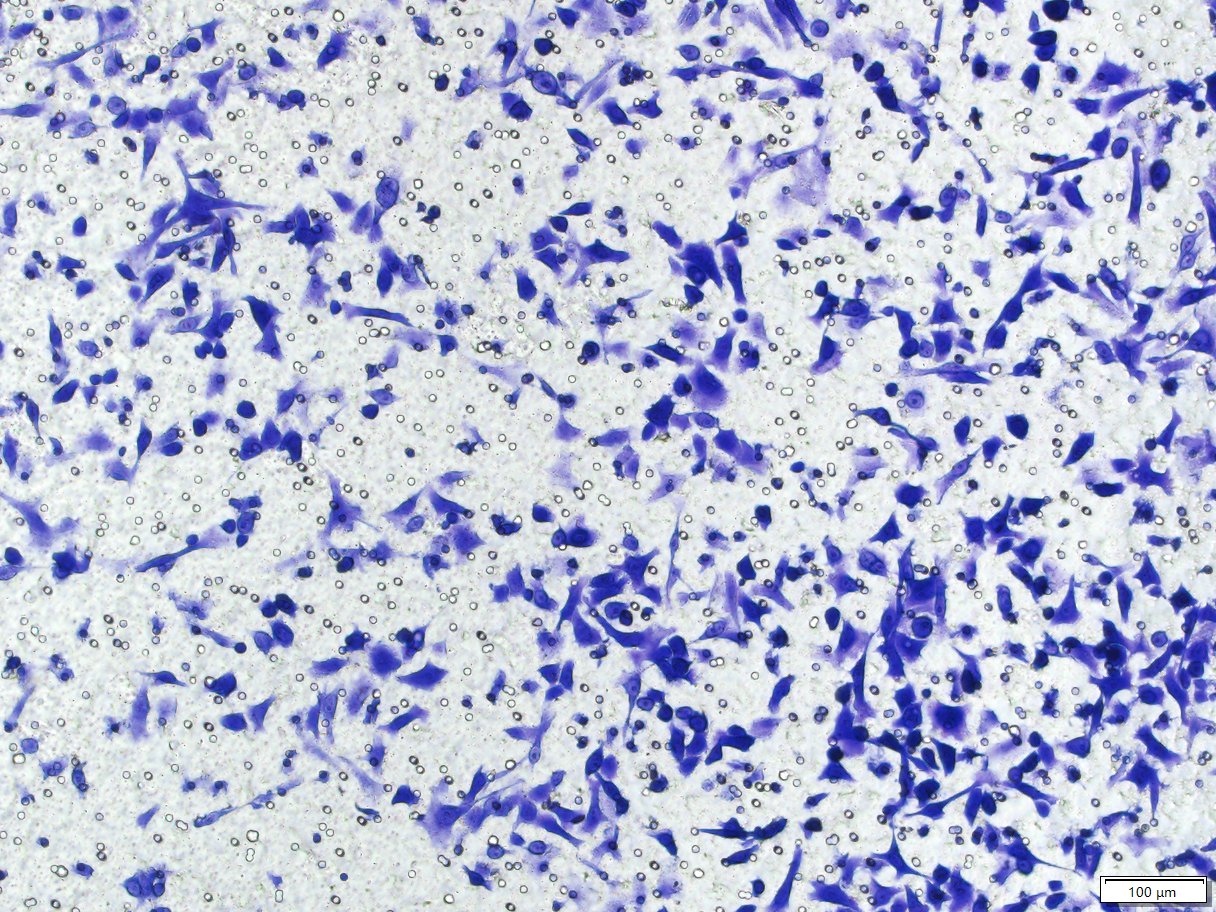

Supplement: Supplemental Information 2 [file peerj-cs-09-1651-s002.zip › Dataset 1/1-7.jpg]

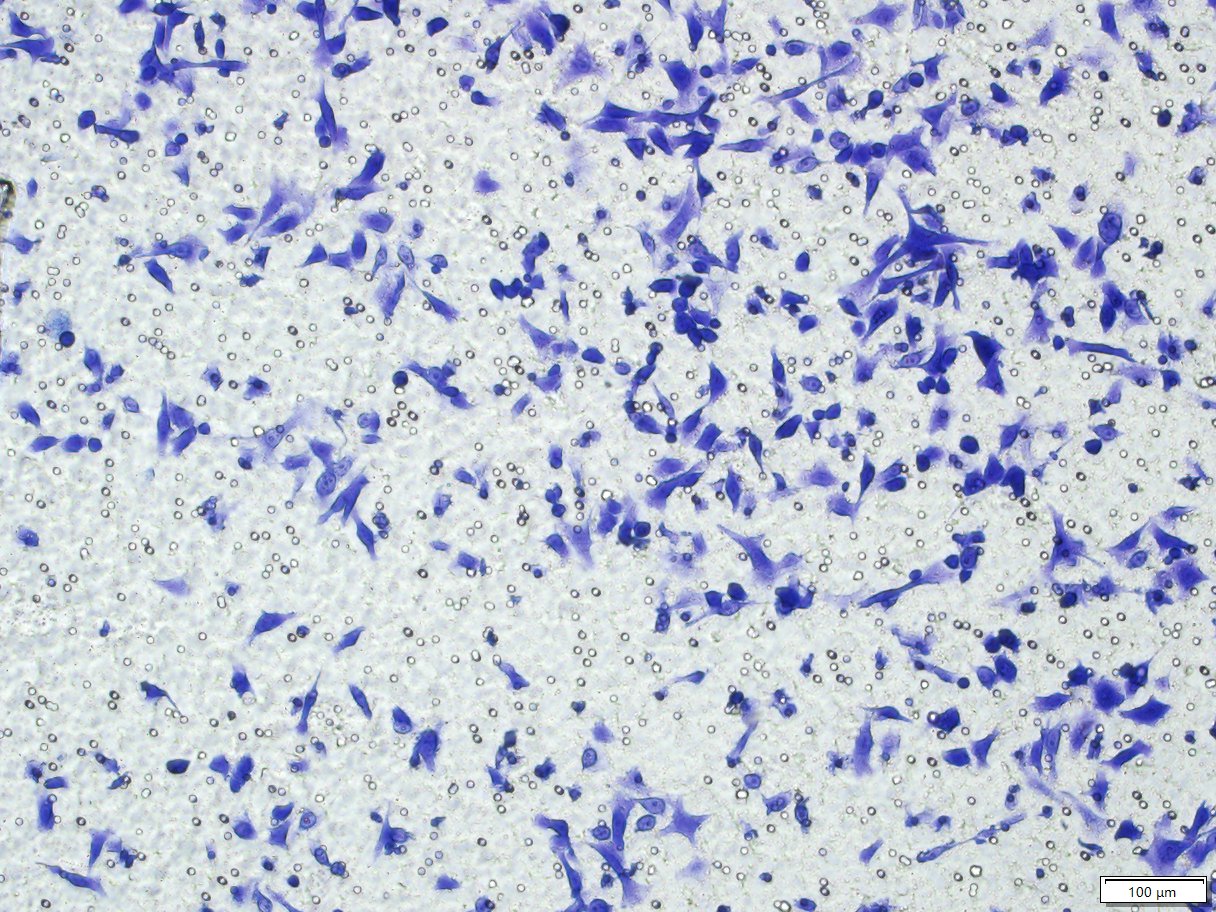

Supplement: Supplemental Information 2 [file peerj-cs-09-1651-s002.zip › Dataset 1/1-8.jpg]

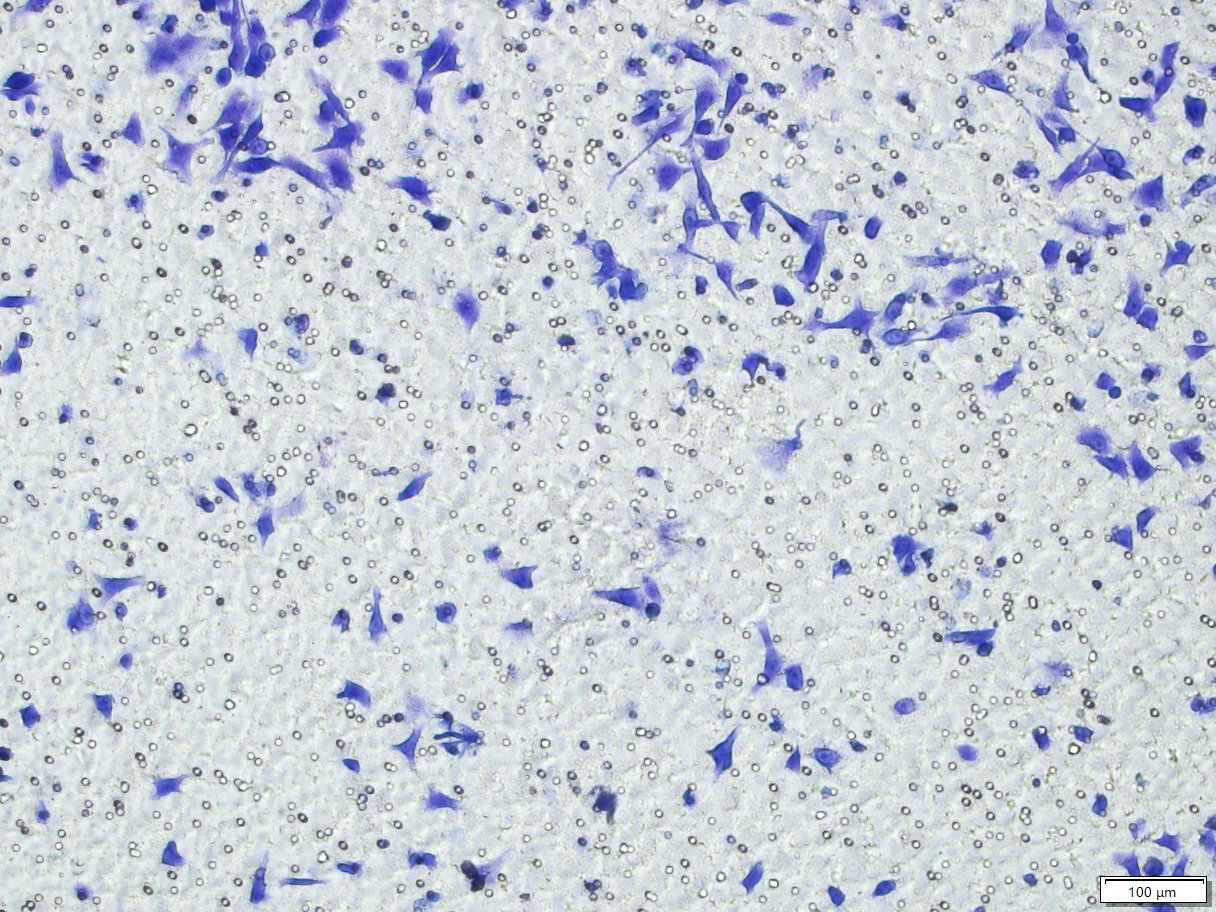

Supplement: Supplemental Information 2 [file peerj-cs-09-1651-s002.zip › Dataset 1/1-9.jpg]

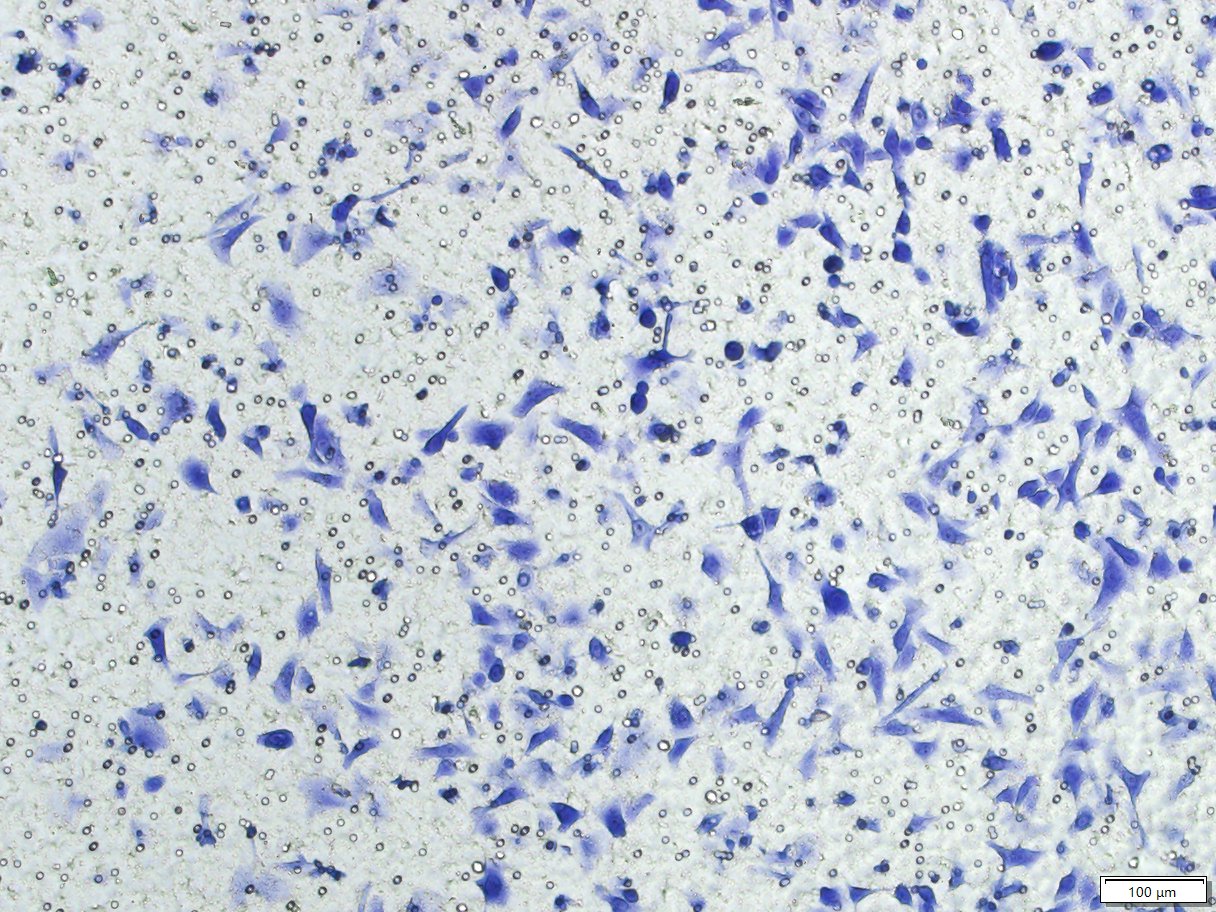

Supplement: Supplemental Information 2 [file peerj-cs-09-1651-s002.zip › Dataset 1/1=10.jpg]

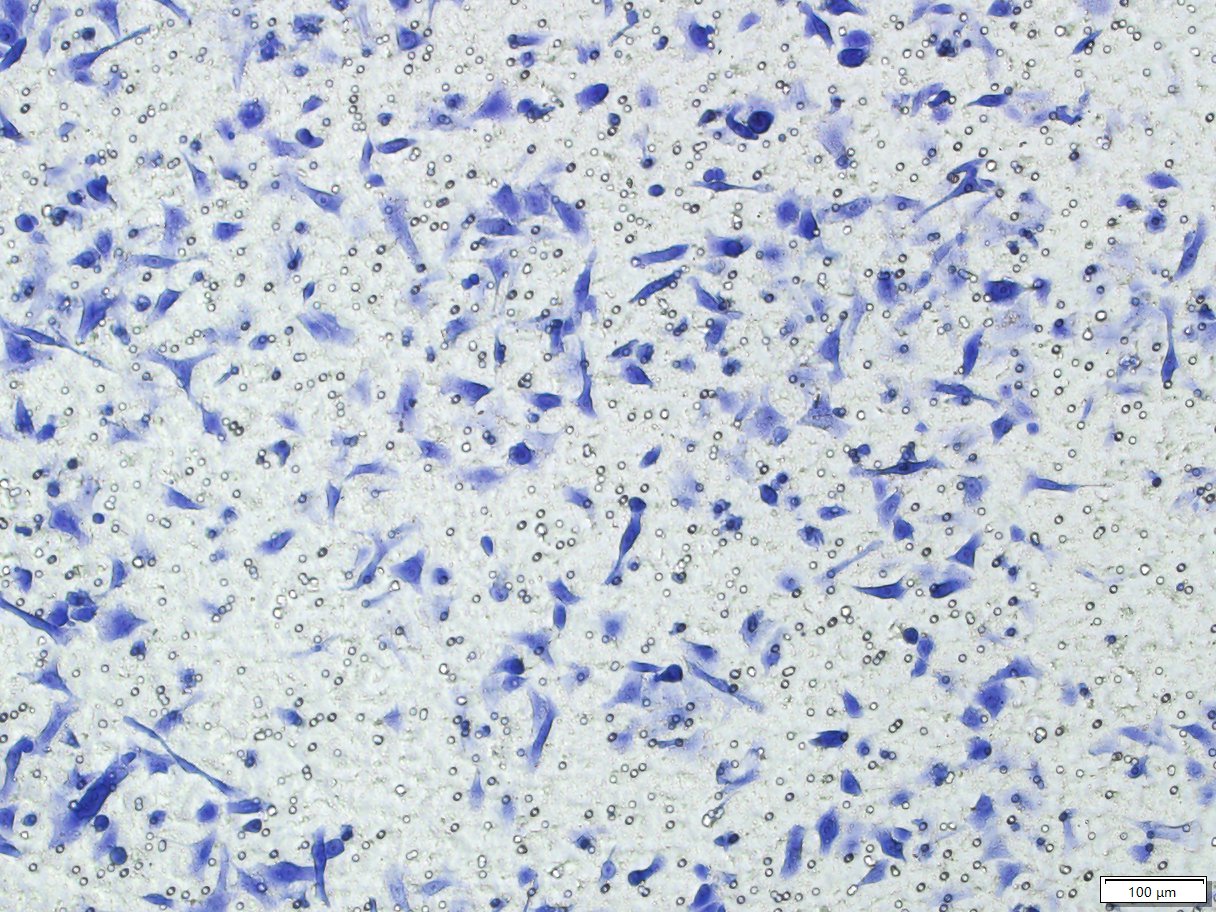

Supplement: Supplemental Information 2 [file peerj-cs-09-1651-s002.zip › Dataset 1/1=7.jpg]

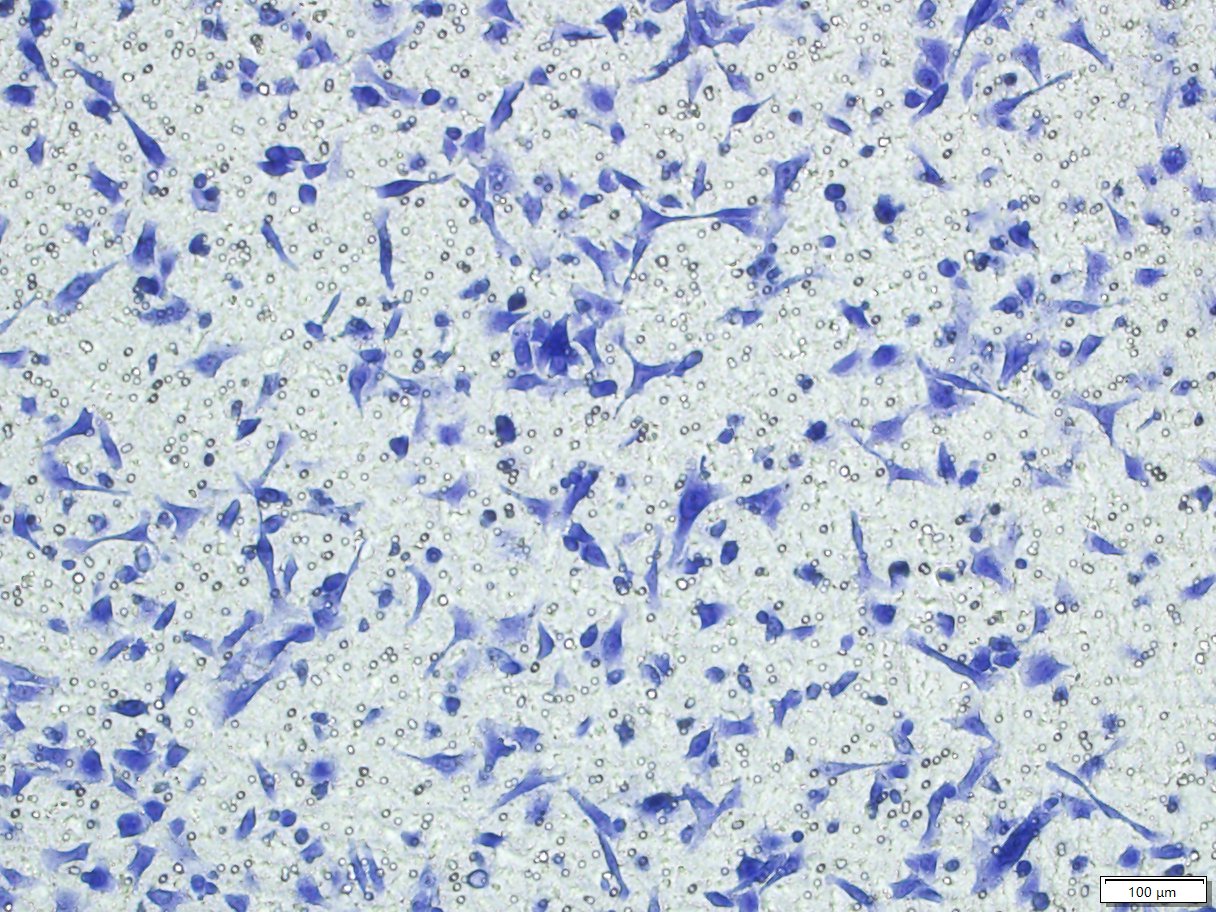

Supplement: Supplemental Information 2 [file peerj-cs-09-1651-s002.zip › Dataset 1/1=8.jpg]

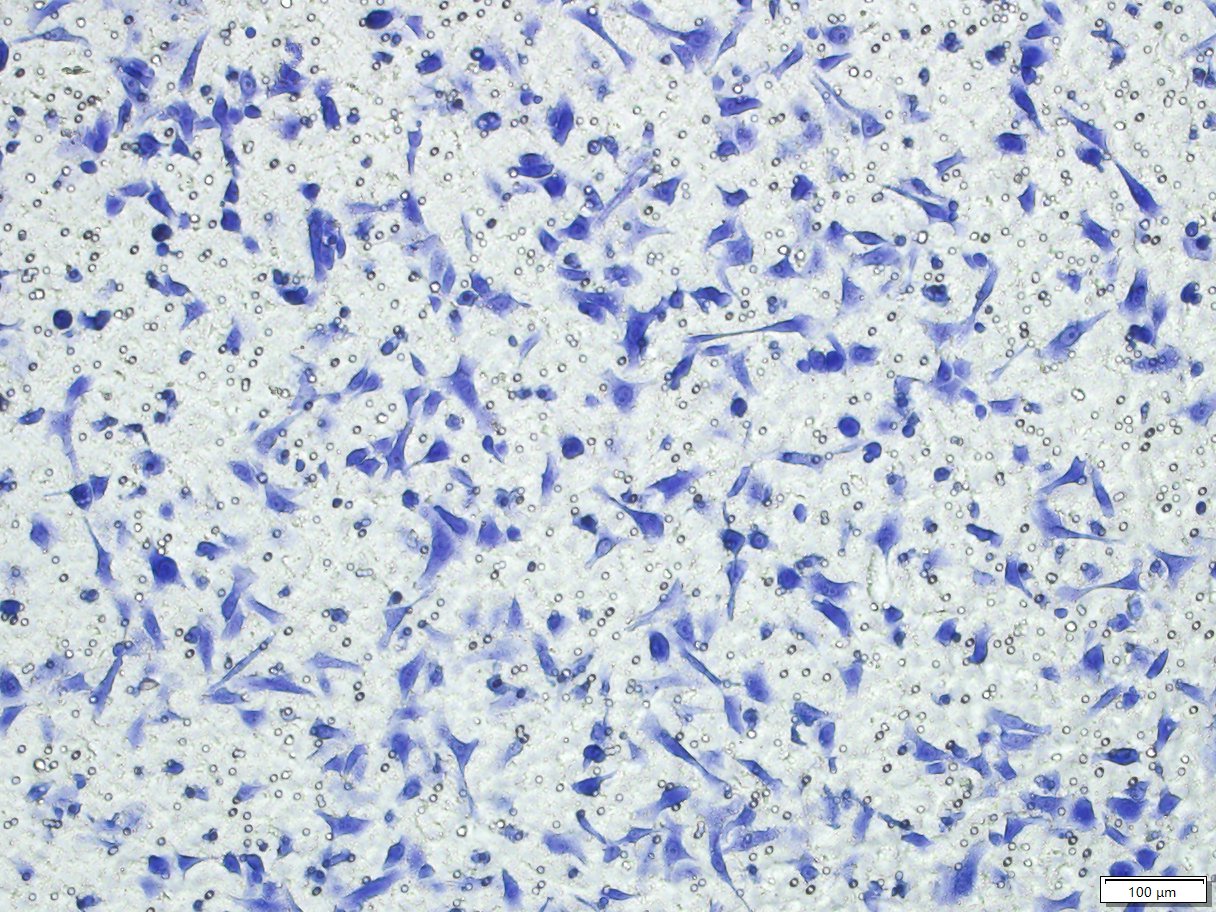

Supplement: Supplemental Information 2 [file peerj-cs-09-1651-s002.zip › Dataset 1/1=9.jpg]

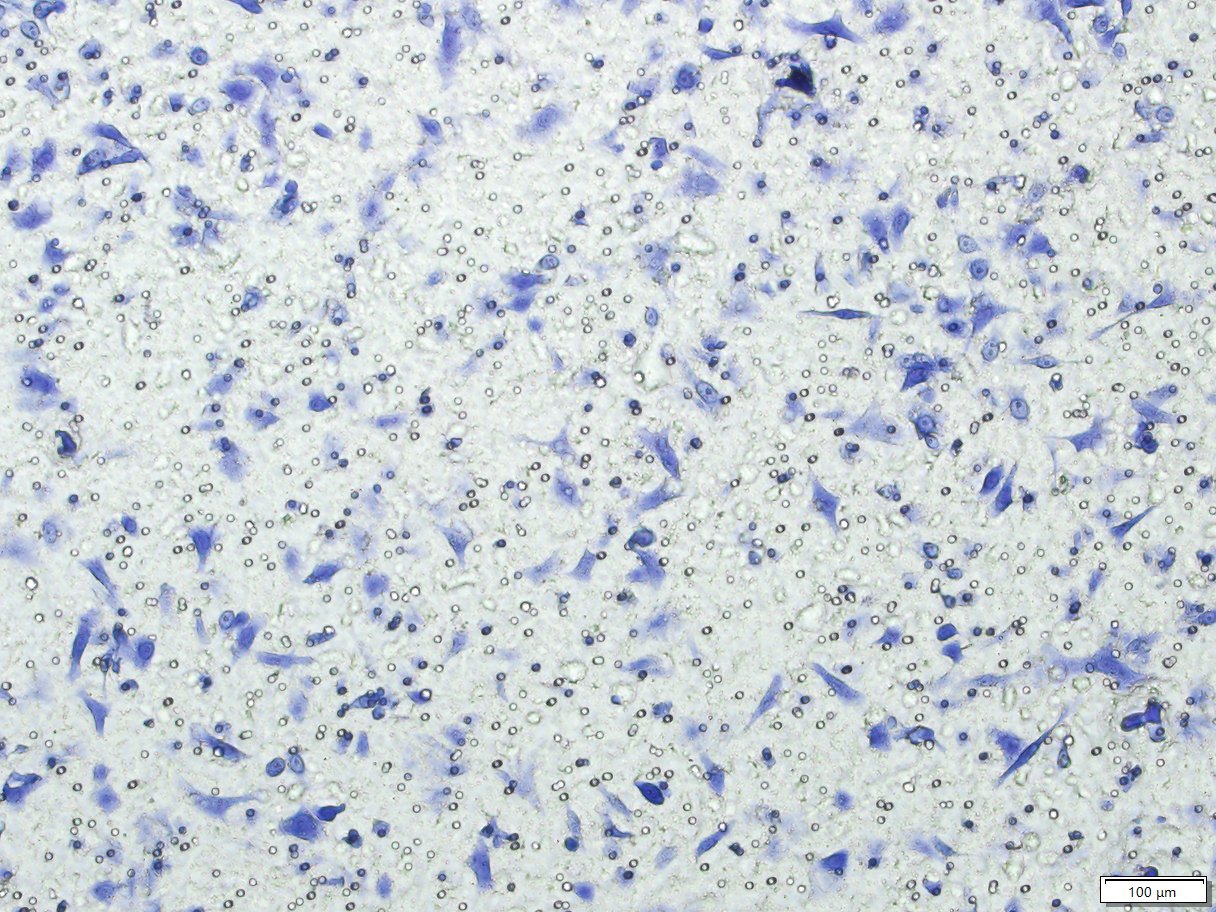

Supplement: Supplemental Information 2 [file peerj-cs-09-1651-s002.zip › Dataset 1/2+1.jpg]

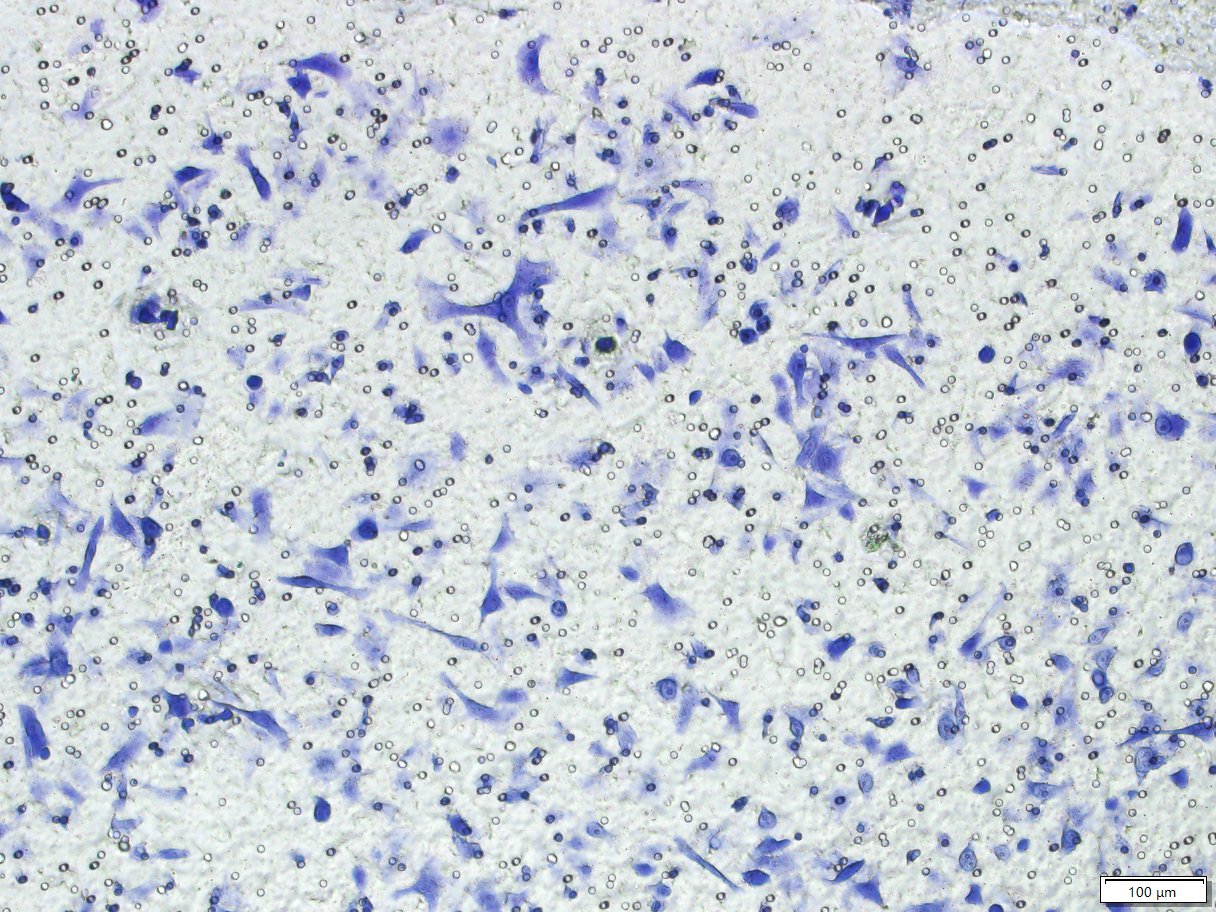

Supplement: Supplemental Information 2 [file peerj-cs-09-1651-s002.zip › Dataset 1/2+10.jpg]

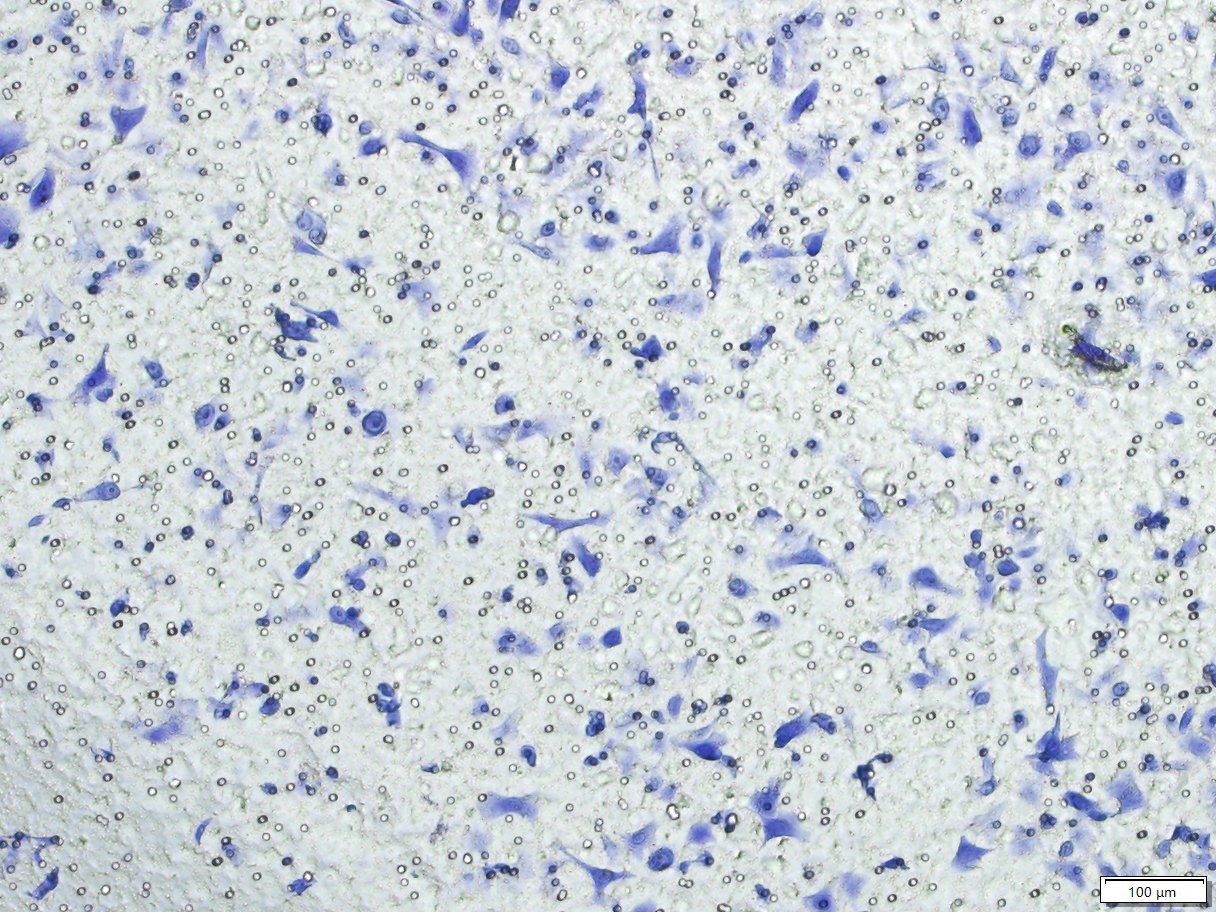

Supplement: Supplemental Information 2 [file peerj-cs-09-1651-s002.zip › Dataset 1/2+2.jpg]

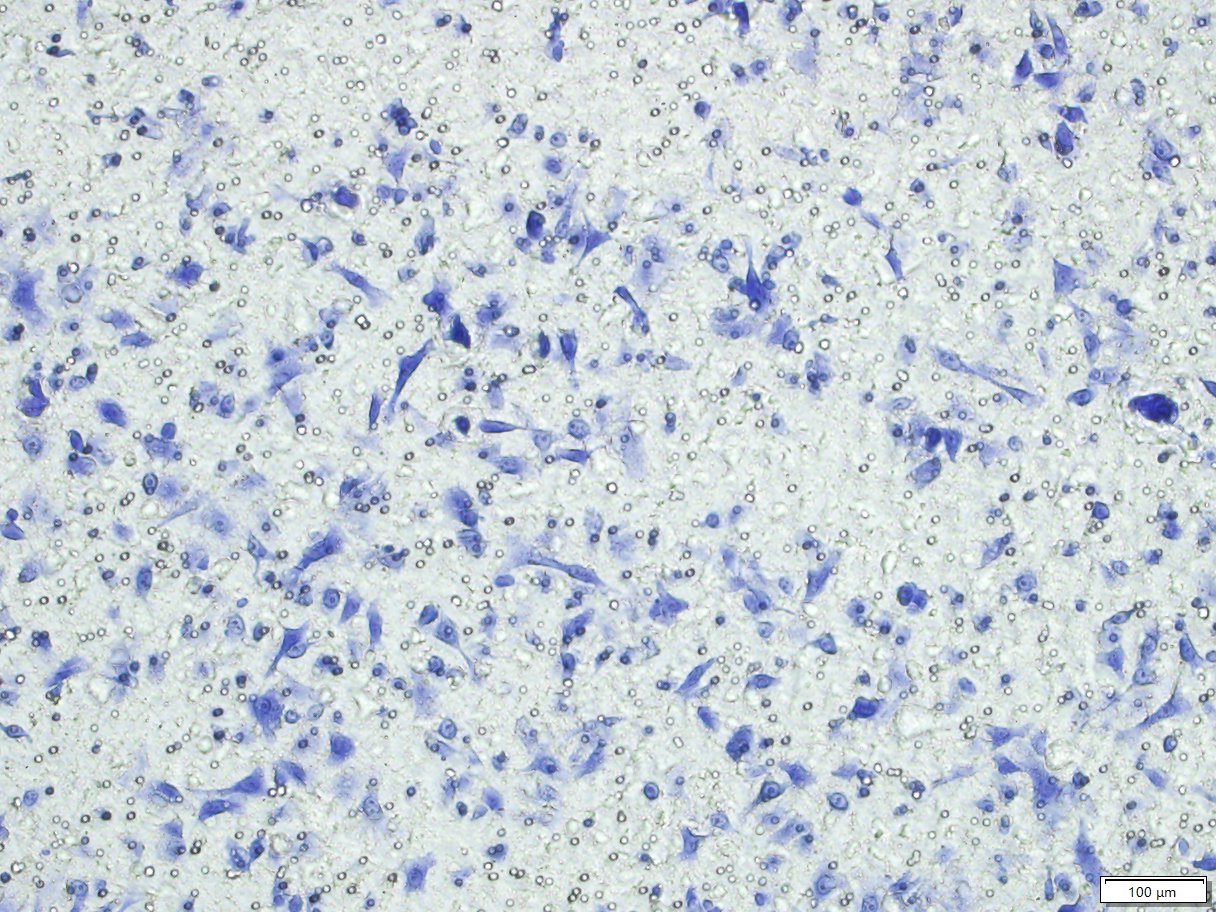

Supplement: Supplemental Information 2 [file peerj-cs-09-1651-s002.zip › Dataset 1/2+3.jpg]

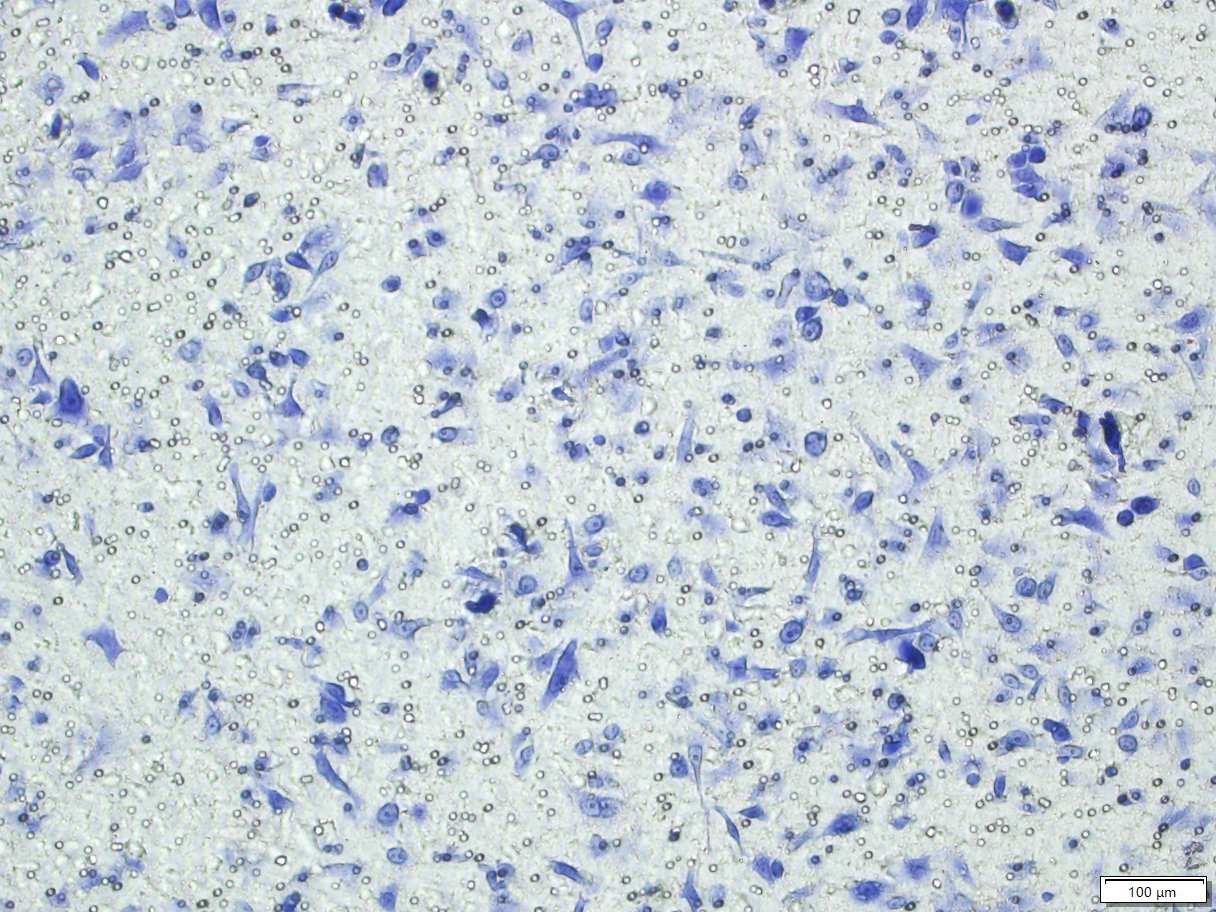

Supplement: Supplemental Information 2 [file peerj-cs-09-1651-s002.zip › Dataset 1/2+4.jpg]

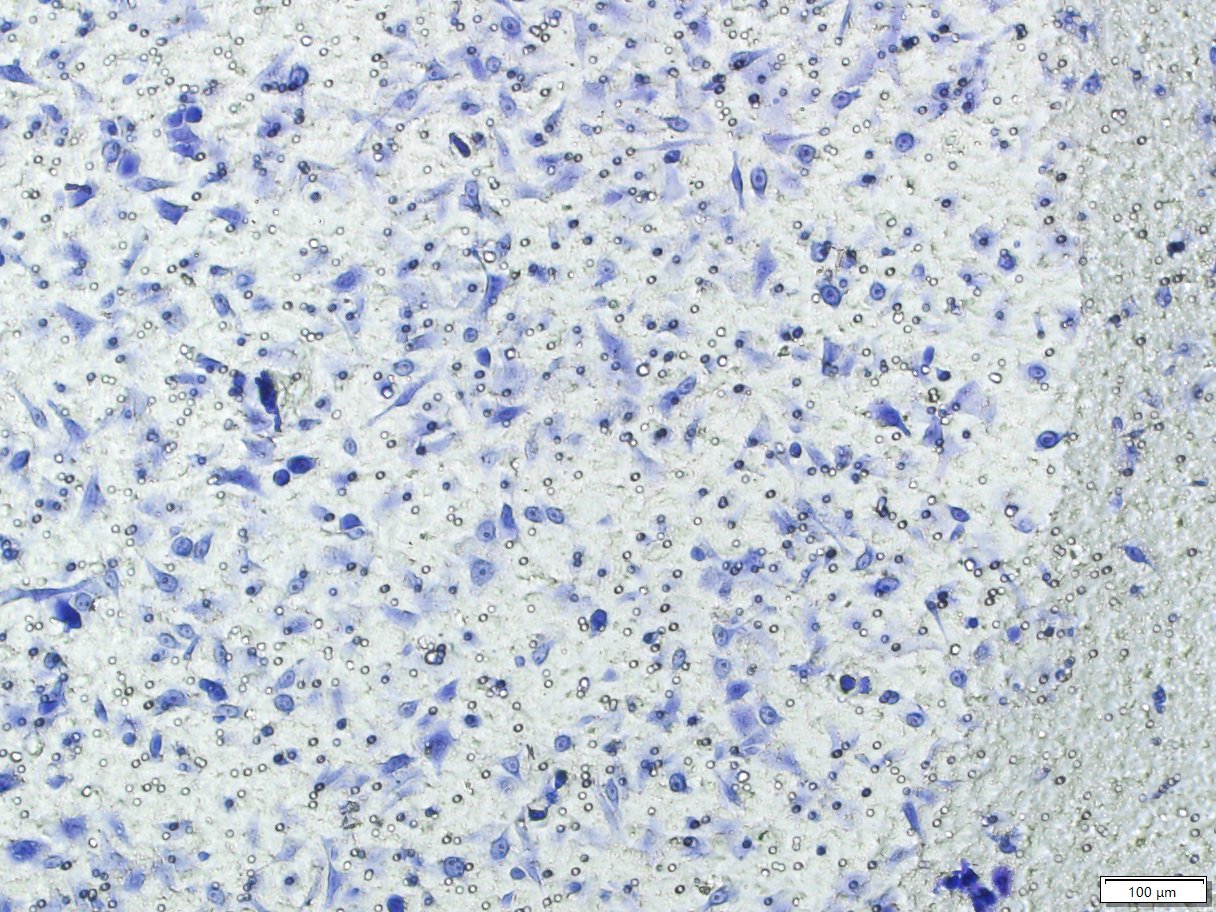

Supplement: Supplemental Information 2 [file peerj-cs-09-1651-s002.zip › Dataset 1/2+5.jpg]

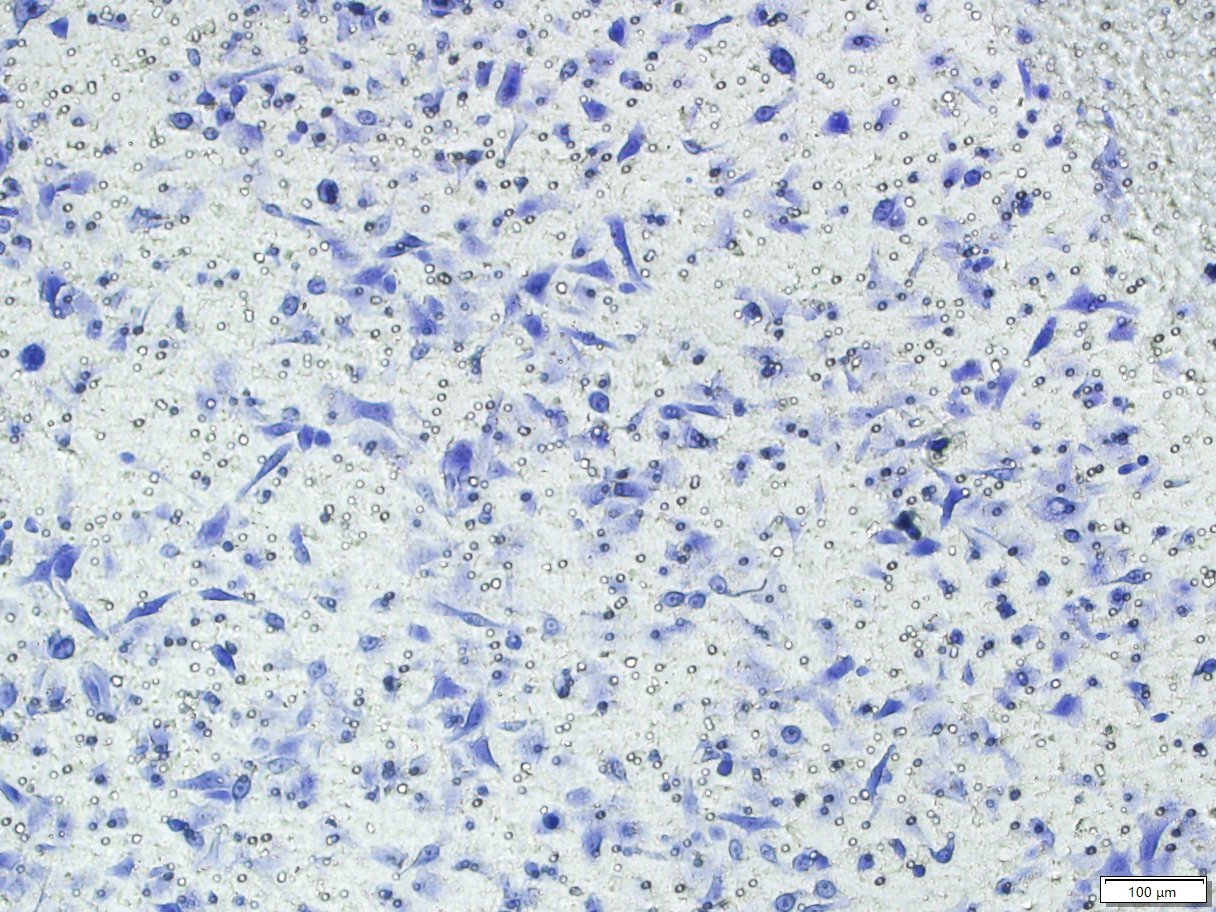

Supplement: Supplemental Information 2 [file peerj-cs-09-1651-s002.zip › Dataset 1/2+6.jpg]

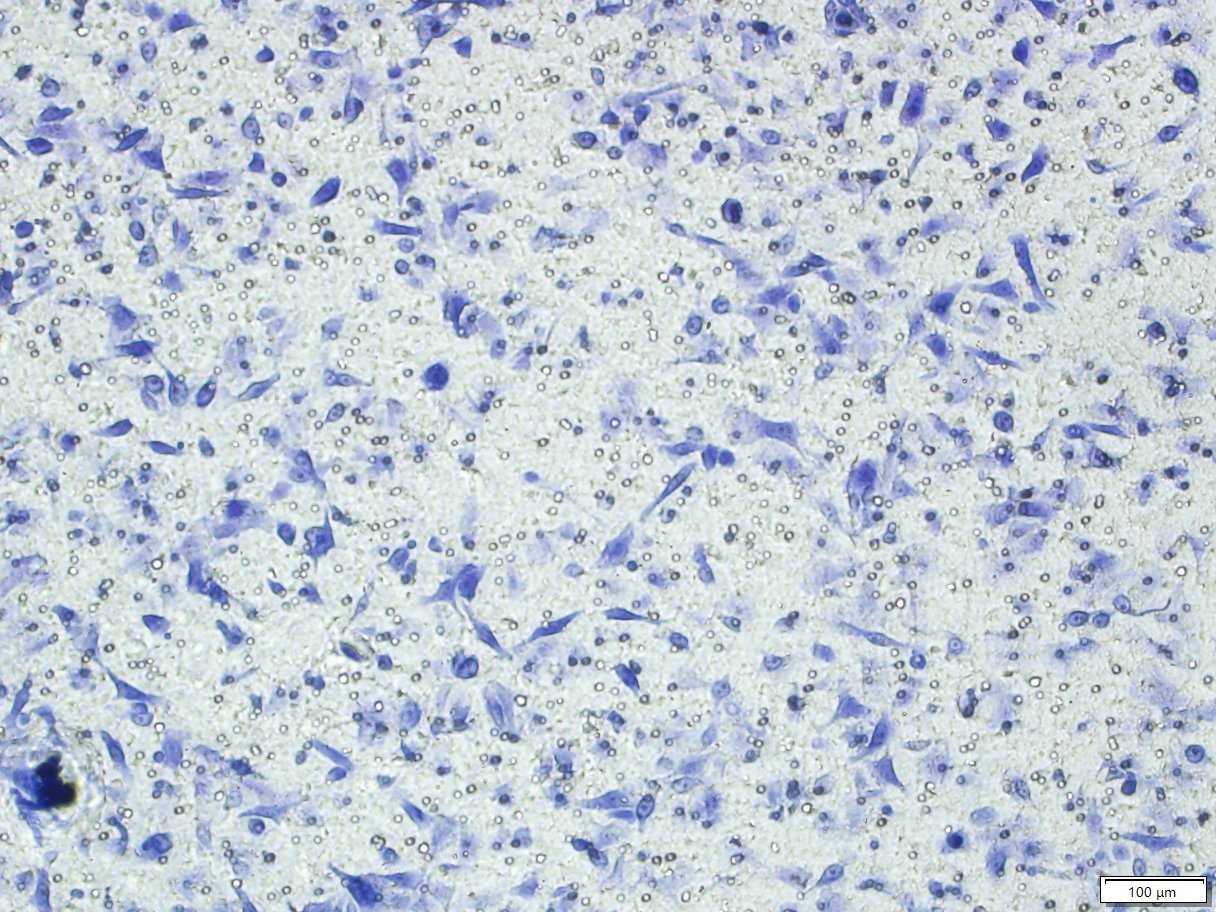

Supplement: Supplemental Information 2 [file peerj-cs-09-1651-s002.zip › Dataset 1/2+7.jpg]

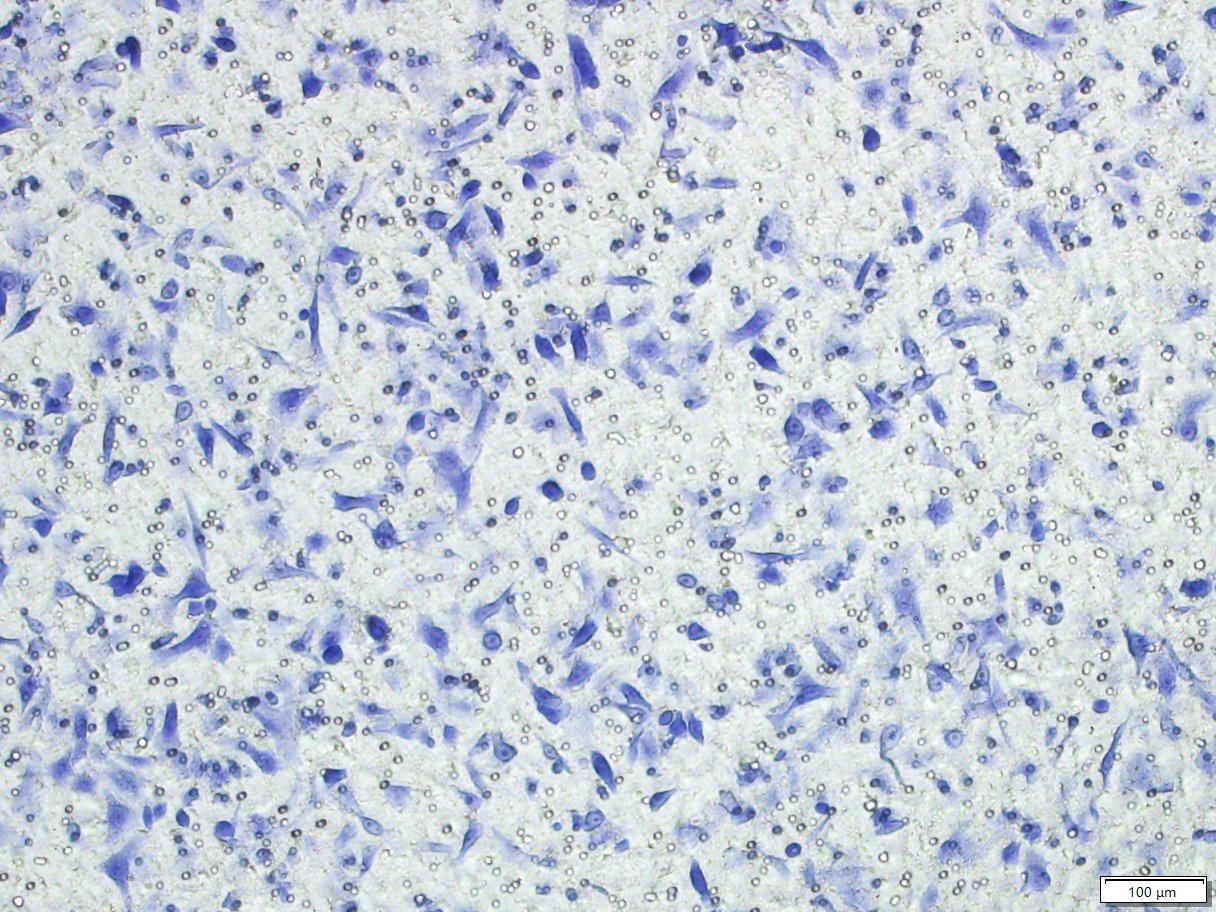

Supplement: Supplemental Information 2 [file peerj-cs-09-1651-s002.zip › Dataset 1/2+8.jpg]

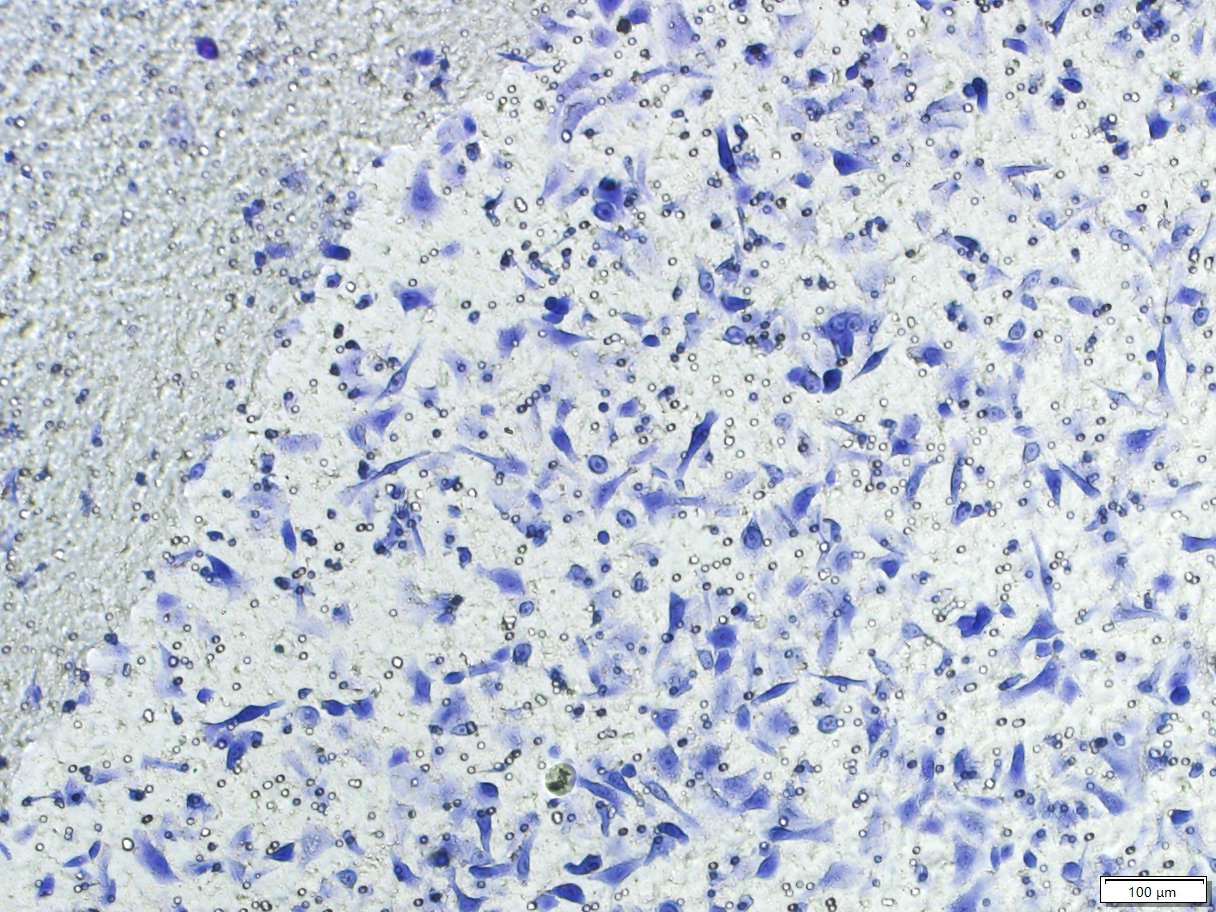

Supplement: Supplemental Information 2 [file peerj-cs-09-1651-s002.zip › Dataset 1/2+9.jpg]

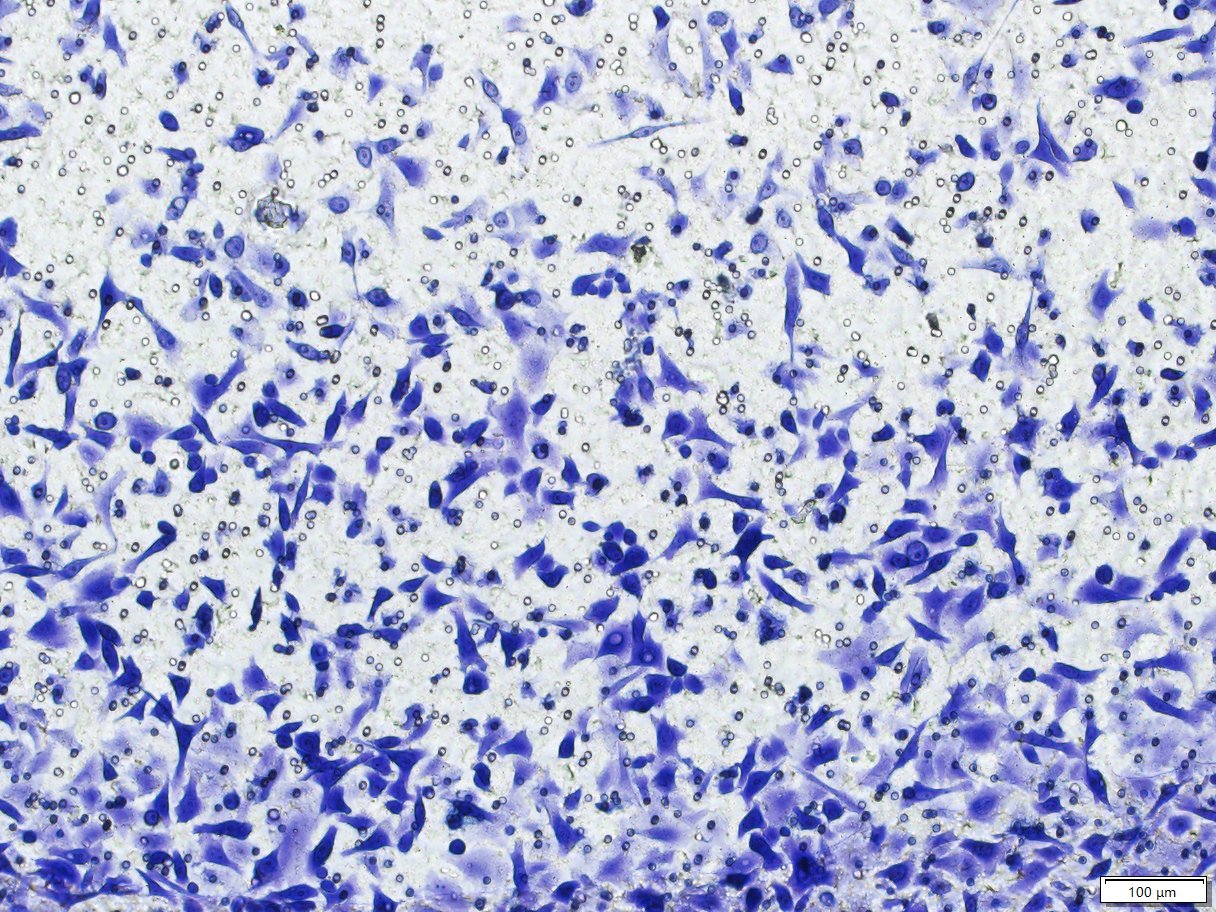

Supplement: Supplemental Information 2 [file peerj-cs-09-1651-s002.zip › Dataset 1/2-1.jpg]

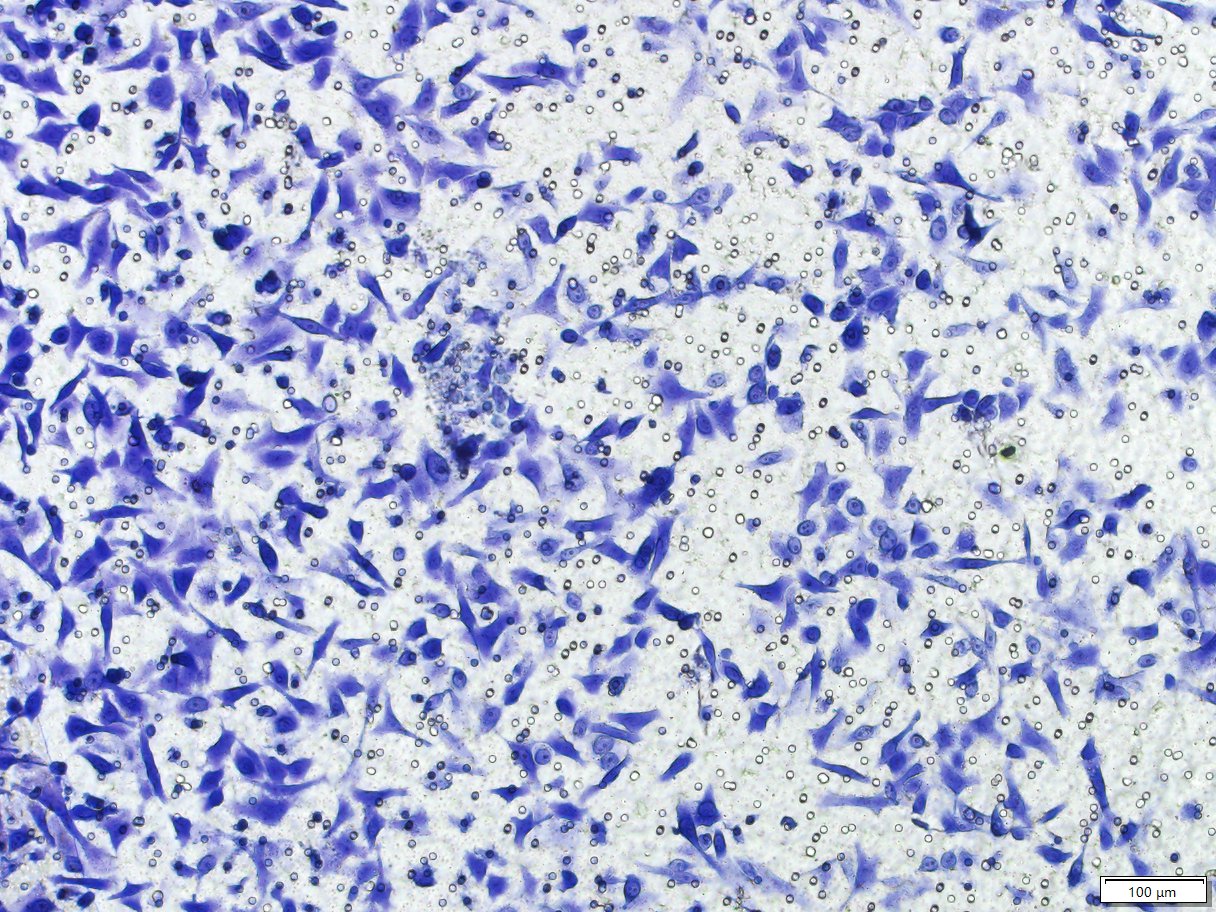

Supplement: Supplemental Information 2 [file peerj-cs-09-1651-s002.zip › Dataset 1/2-10.jpg]

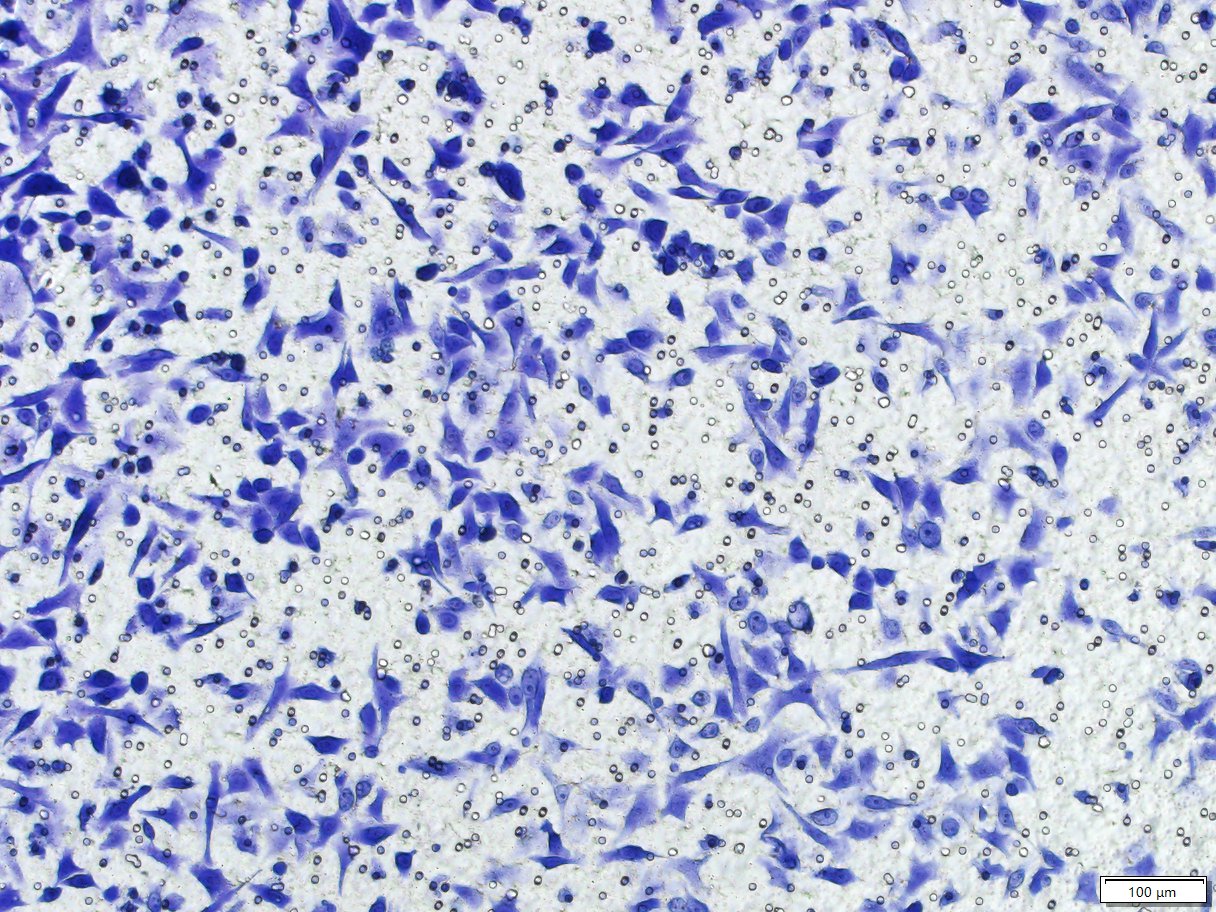

Supplement: Supplemental Information 2 [file peerj-cs-09-1651-s002.zip › Dataset 1/2-11.jpg]

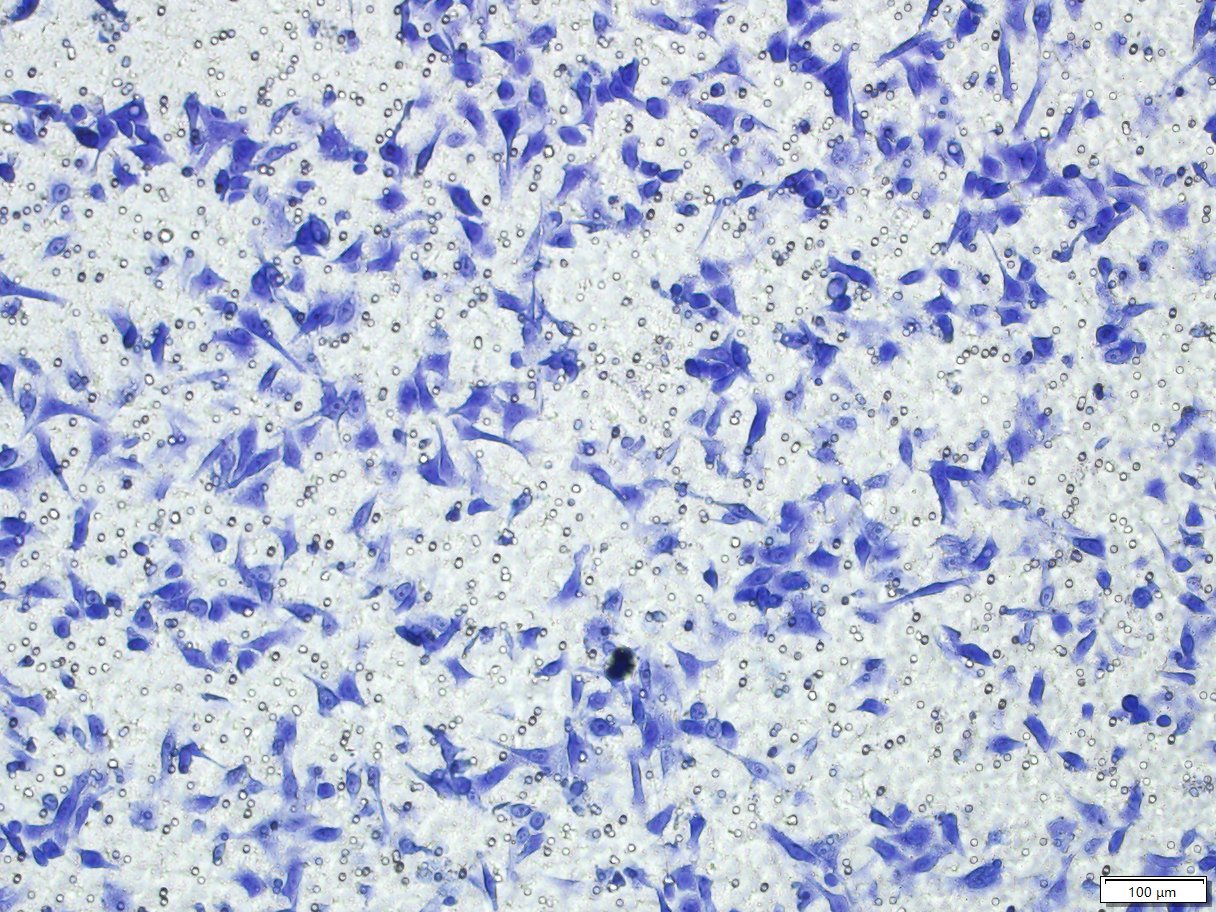

Supplement: Supplemental Information 2 [file peerj-cs-09-1651-s002.zip › Dataset 1/2-12.jpg]

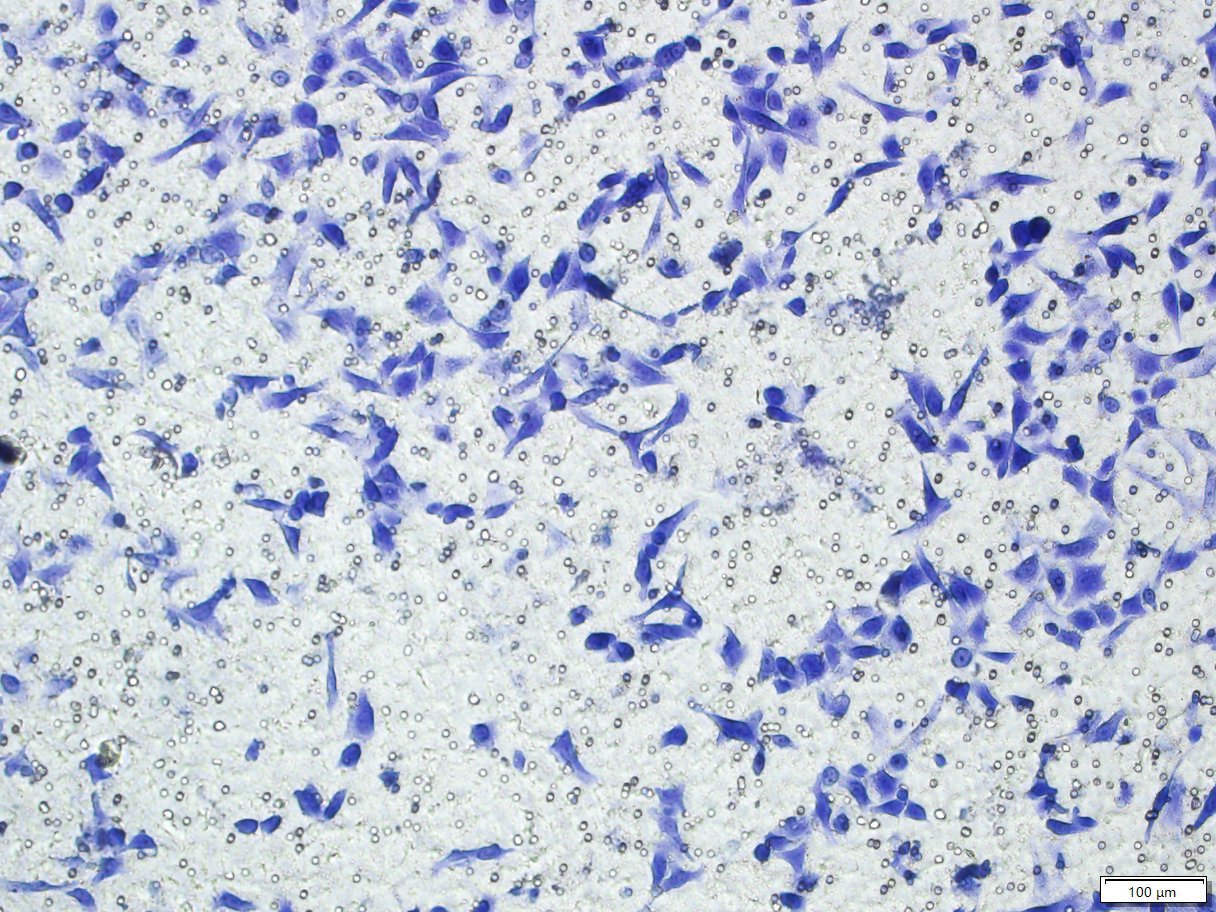

Supplement: Supplemental Information 2 [file peerj-cs-09-1651-s002.zip › Dataset 1/2-13.jpg]

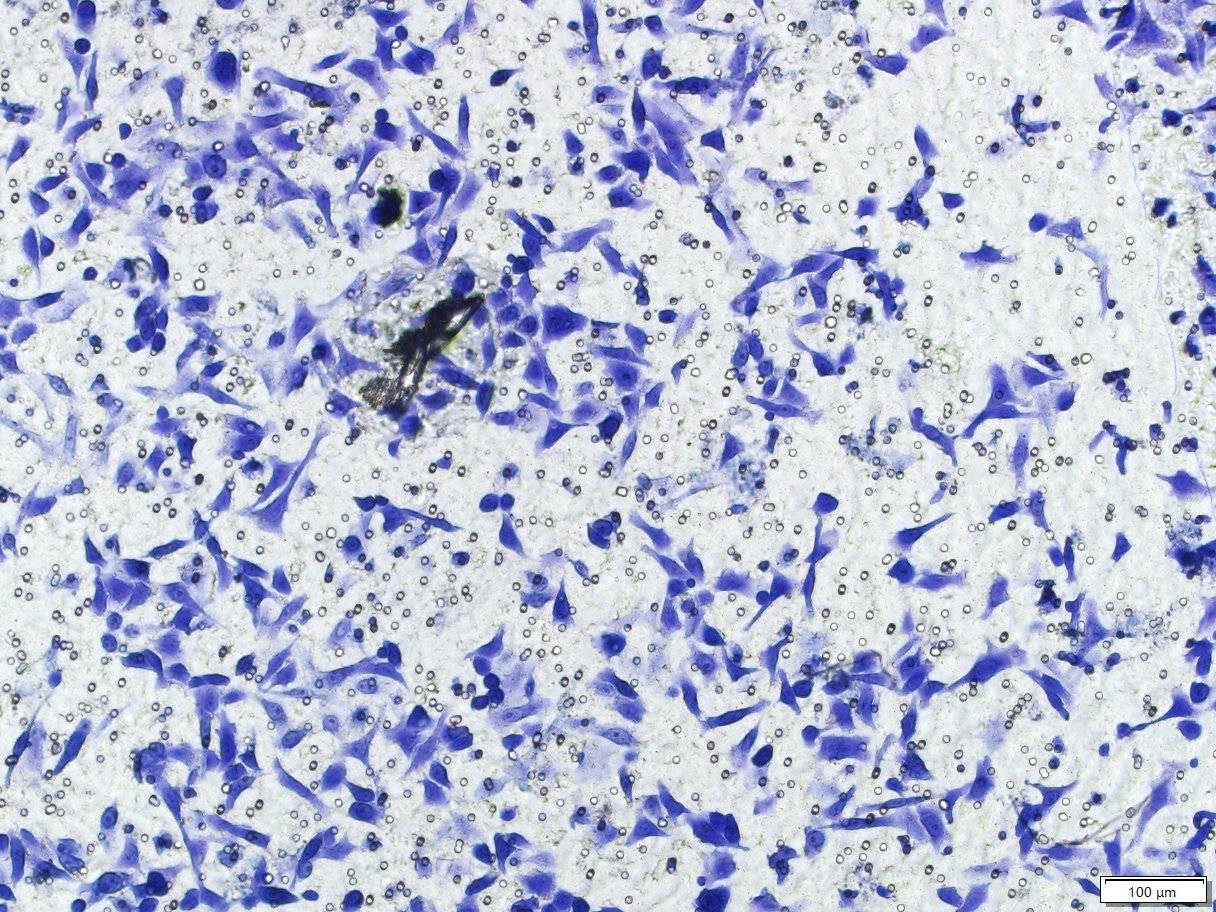

Supplement: Supplemental Information 2 [file peerj-cs-09-1651-s002.zip › Dataset 1/2-14.jpg]

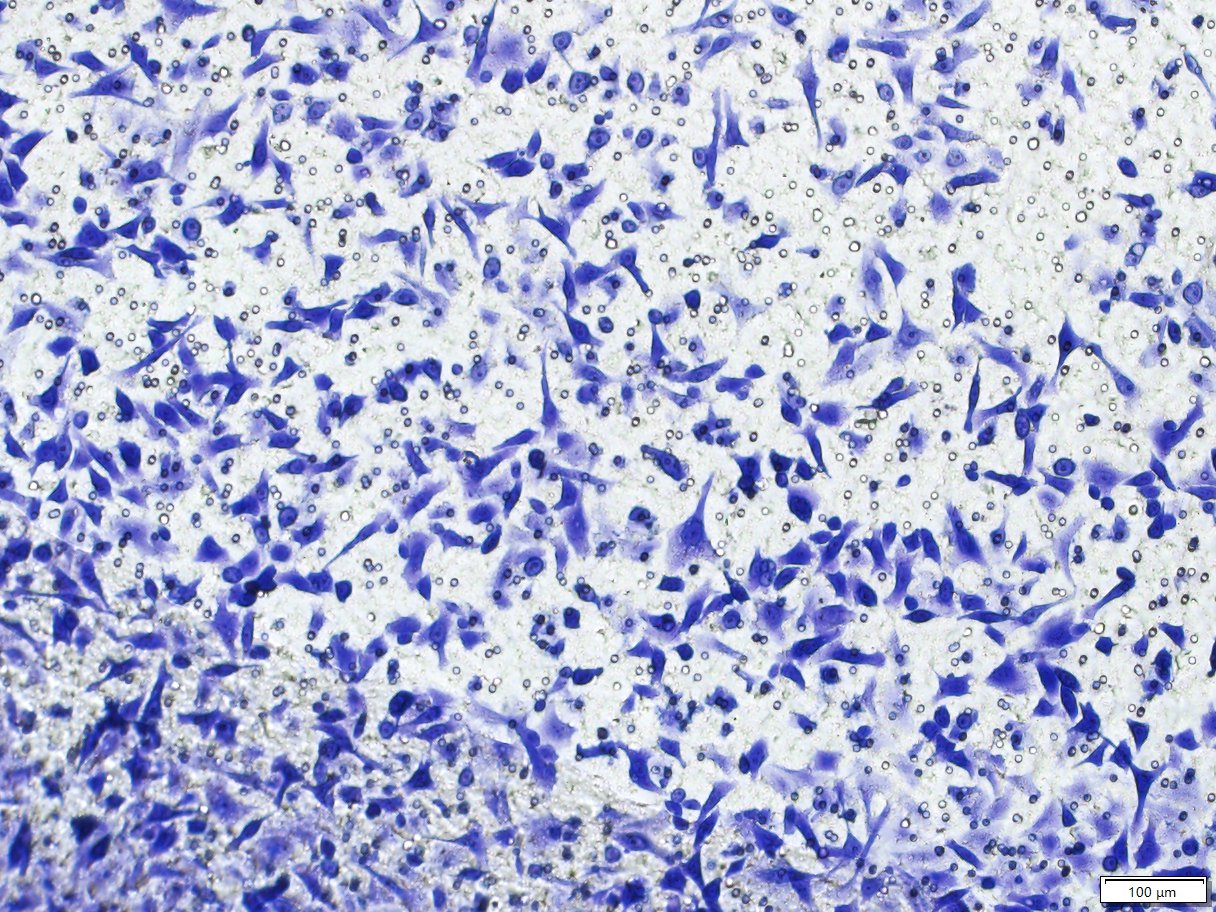

Supplement: Supplemental Information 2 [file peerj-cs-09-1651-s002.zip › Dataset 1/2-2.jpg]

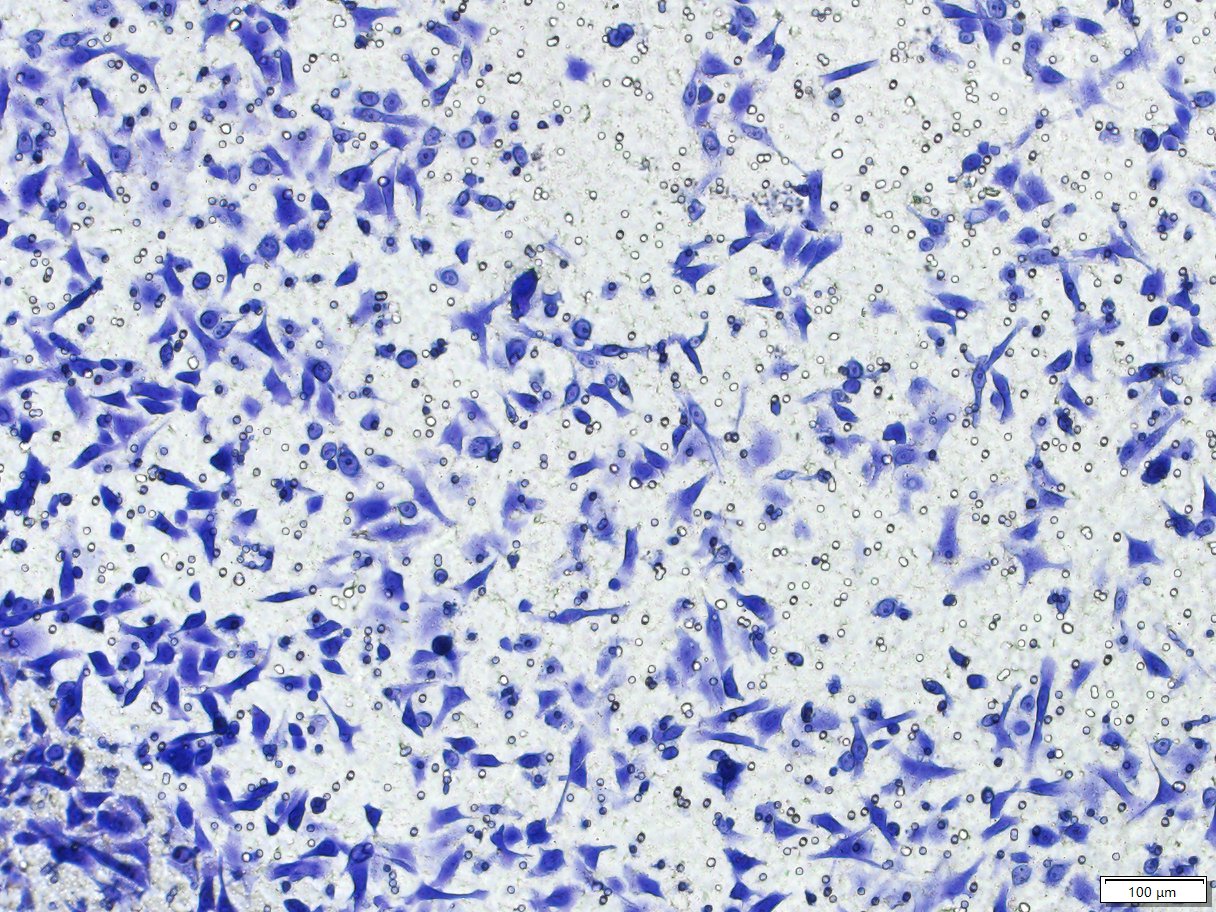

Supplement: Supplemental Information 2 [file peerj-cs-09-1651-s002.zip › Dataset 1/2-3.jpg]

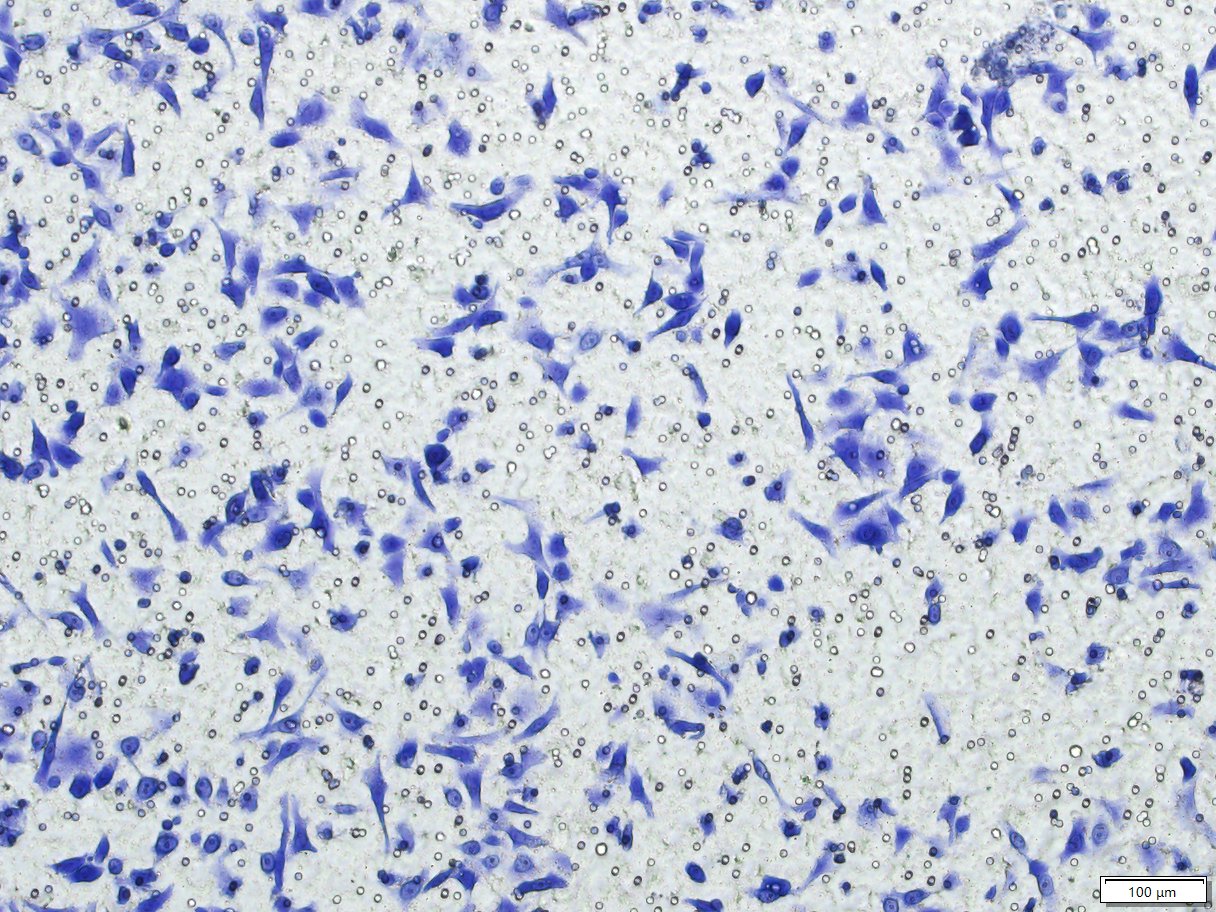

Supplement: Supplemental Information 2 [file peerj-cs-09-1651-s002.zip › Dataset 1/2-4.jpg]

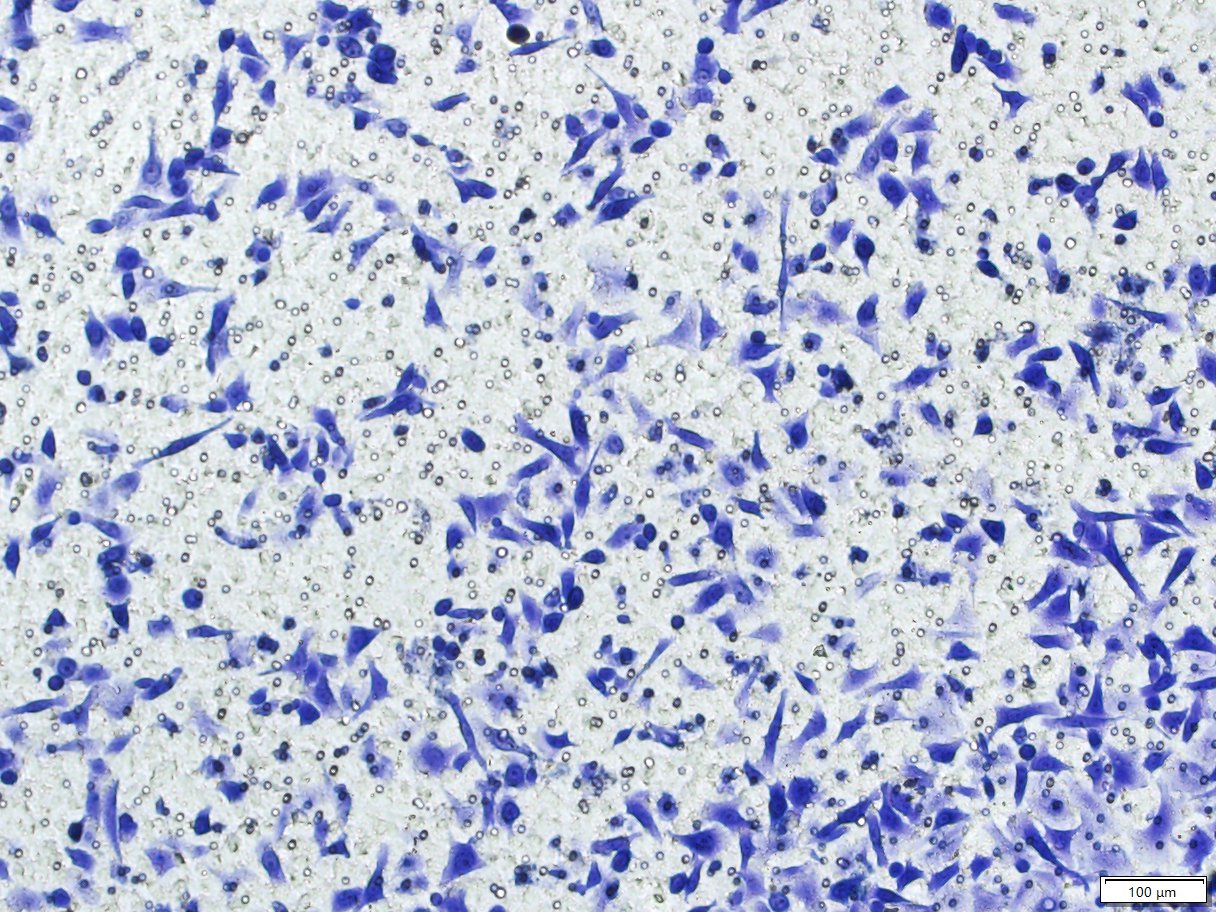

Supplement: Supplemental Information 2 [file peerj-cs-09-1651-s002.zip › Dataset 1/2-5.jpg]

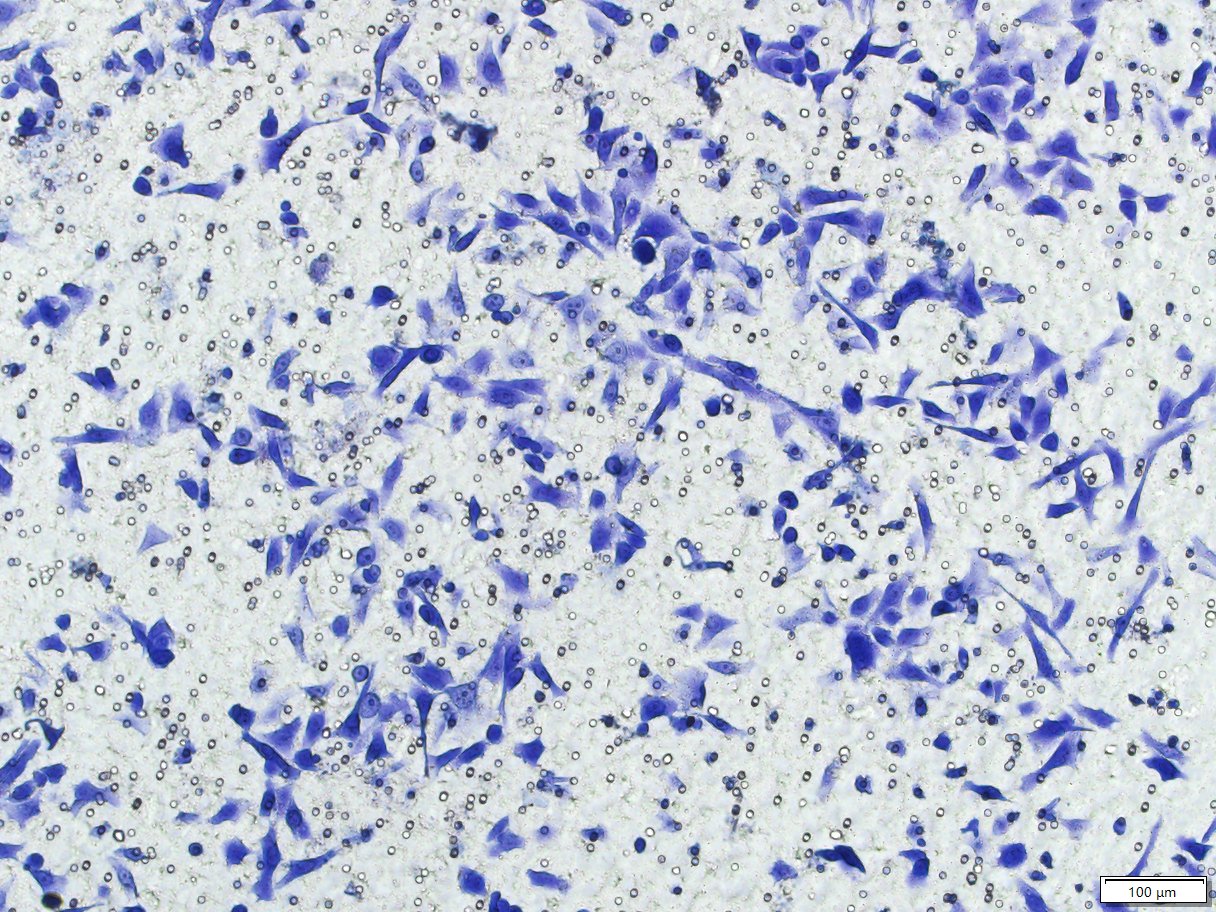

Supplement: Supplemental Information 2 [file peerj-cs-09-1651-s002.zip › Dataset 1/2-6.jpg]

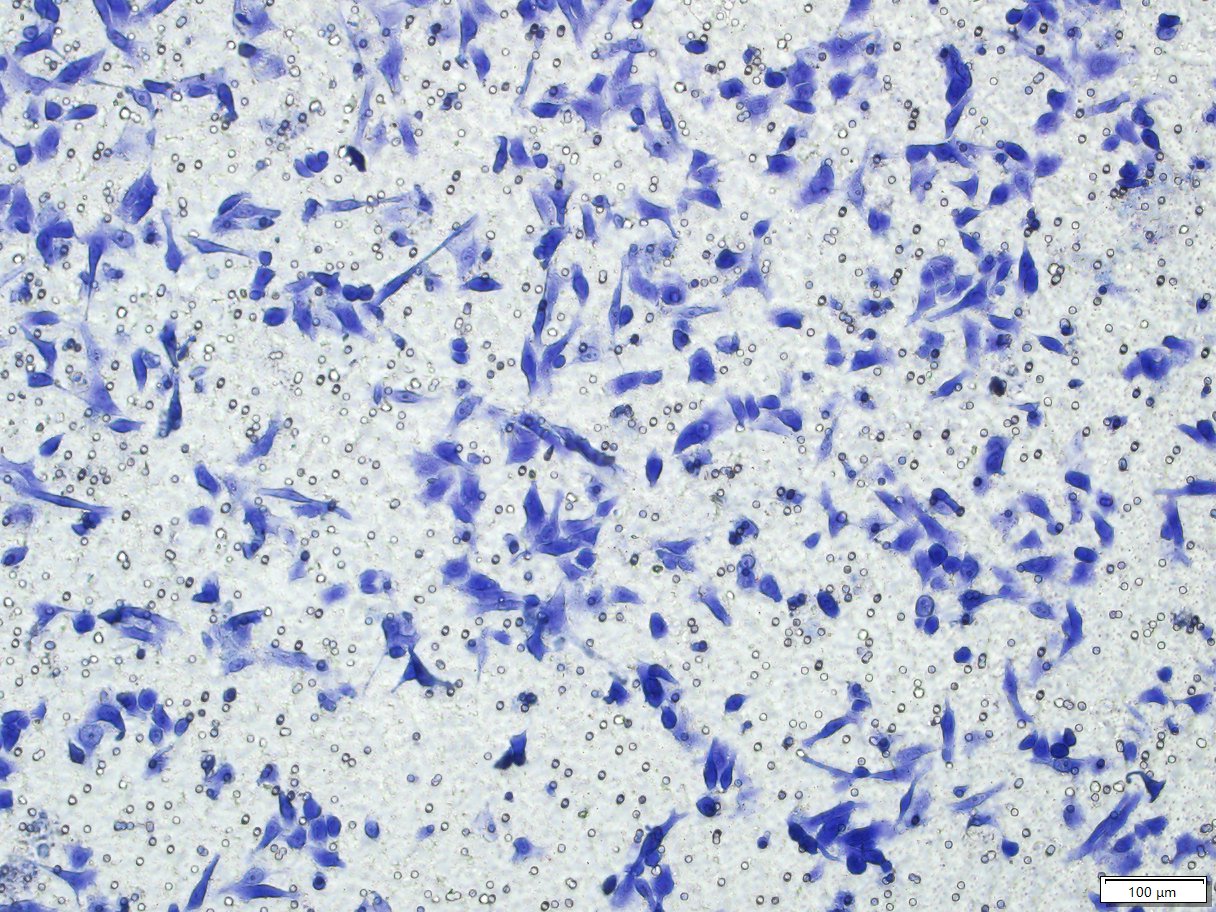

Supplement: Supplemental Information 2 [file peerj-cs-09-1651-s002.zip › Dataset 1/2-7.jpg]

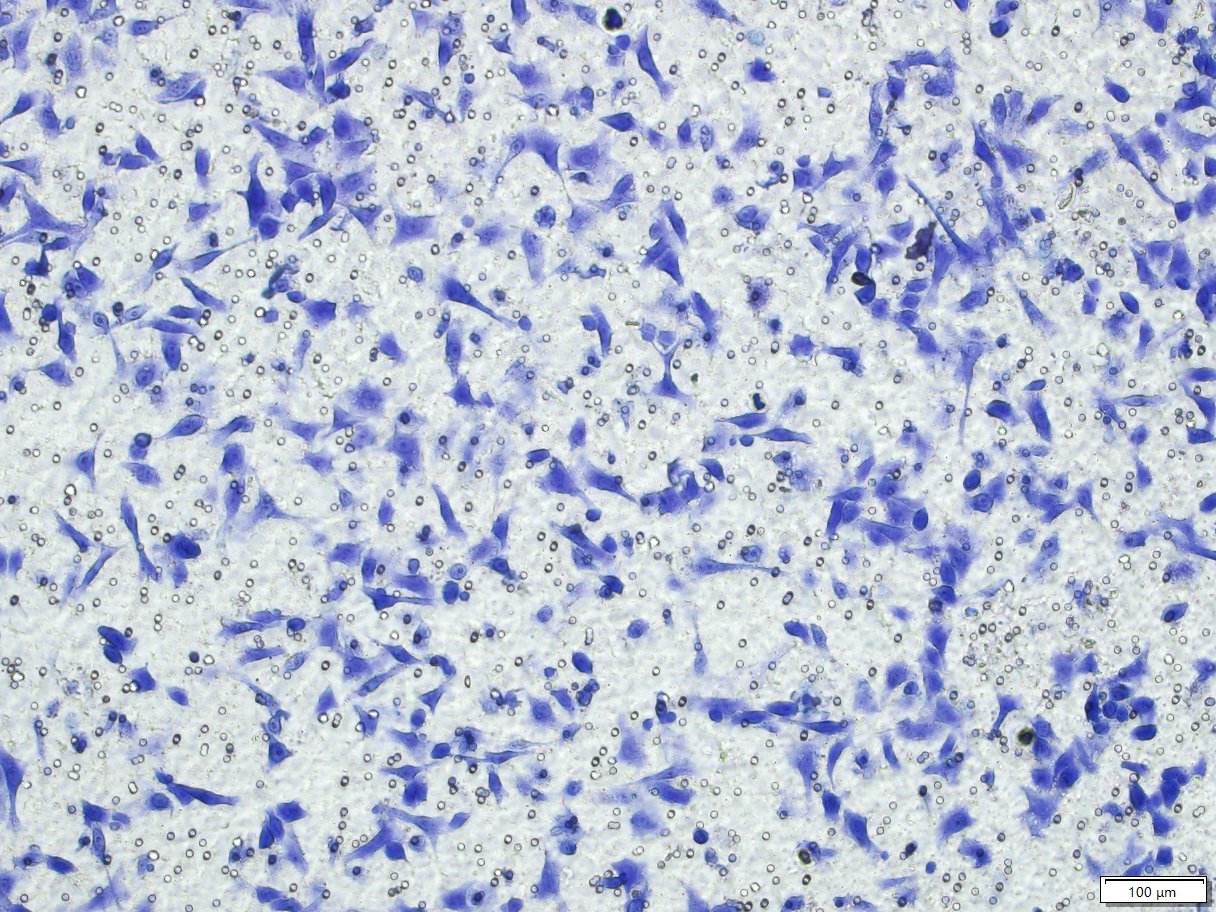

Supplement: Supplemental Information 2 [file peerj-cs-09-1651-s002.zip › Dataset 1/2-8.jpg]

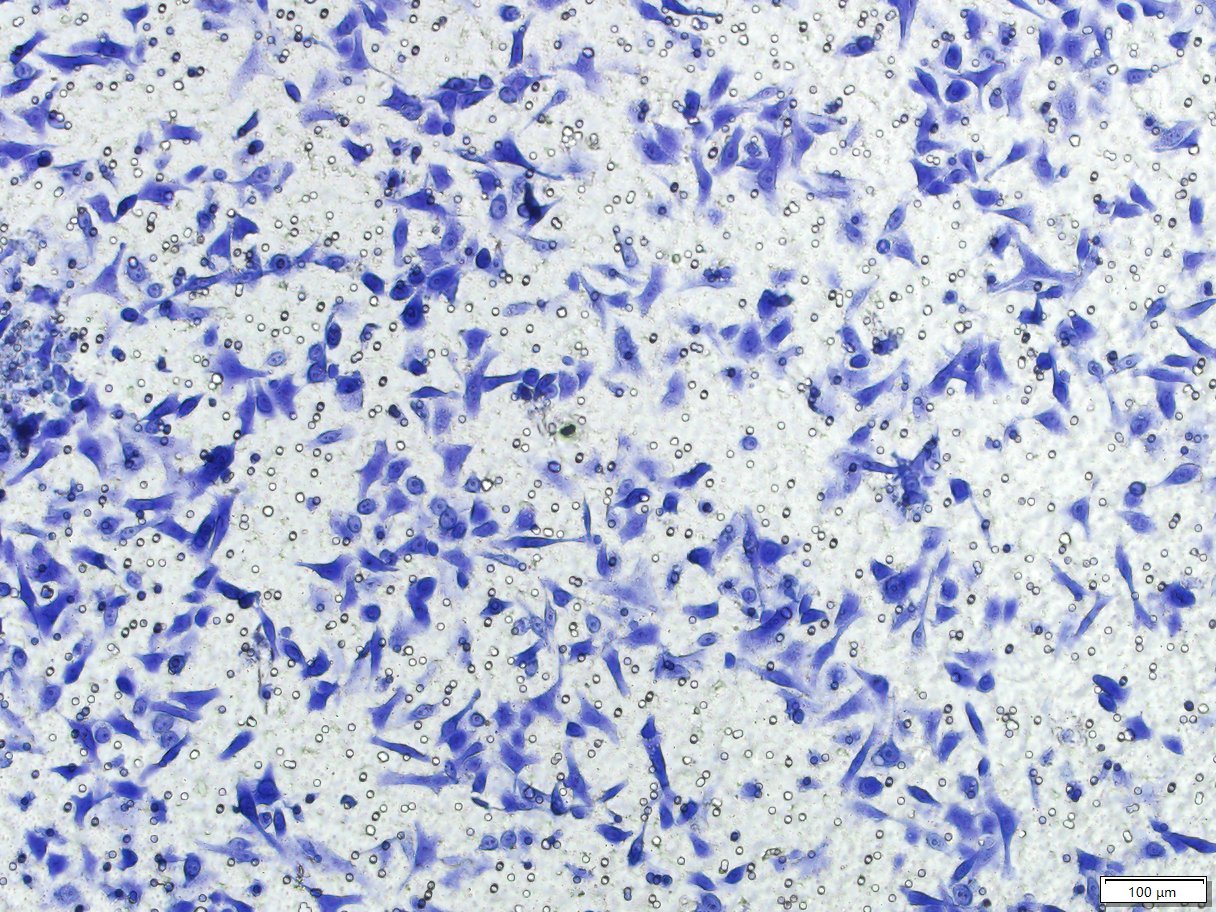

Supplement: Supplemental Information 2 [file peerj-cs-09-1651-s002.zip › Dataset 1/2-9.jpg]

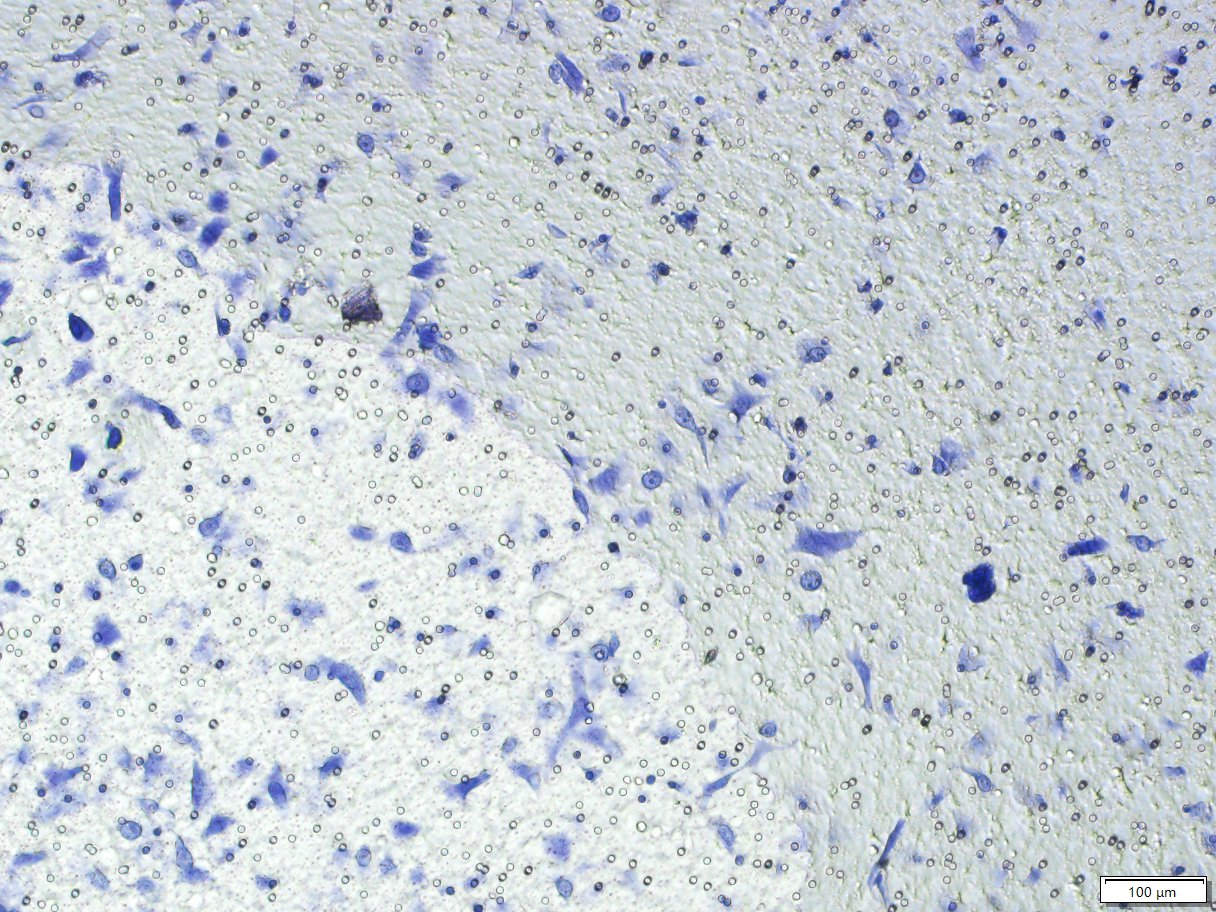

Supplement: Supplemental Information 3 [file peerj-cs-09-1651-s003.zip › Dataset 2/4+10.jpg]

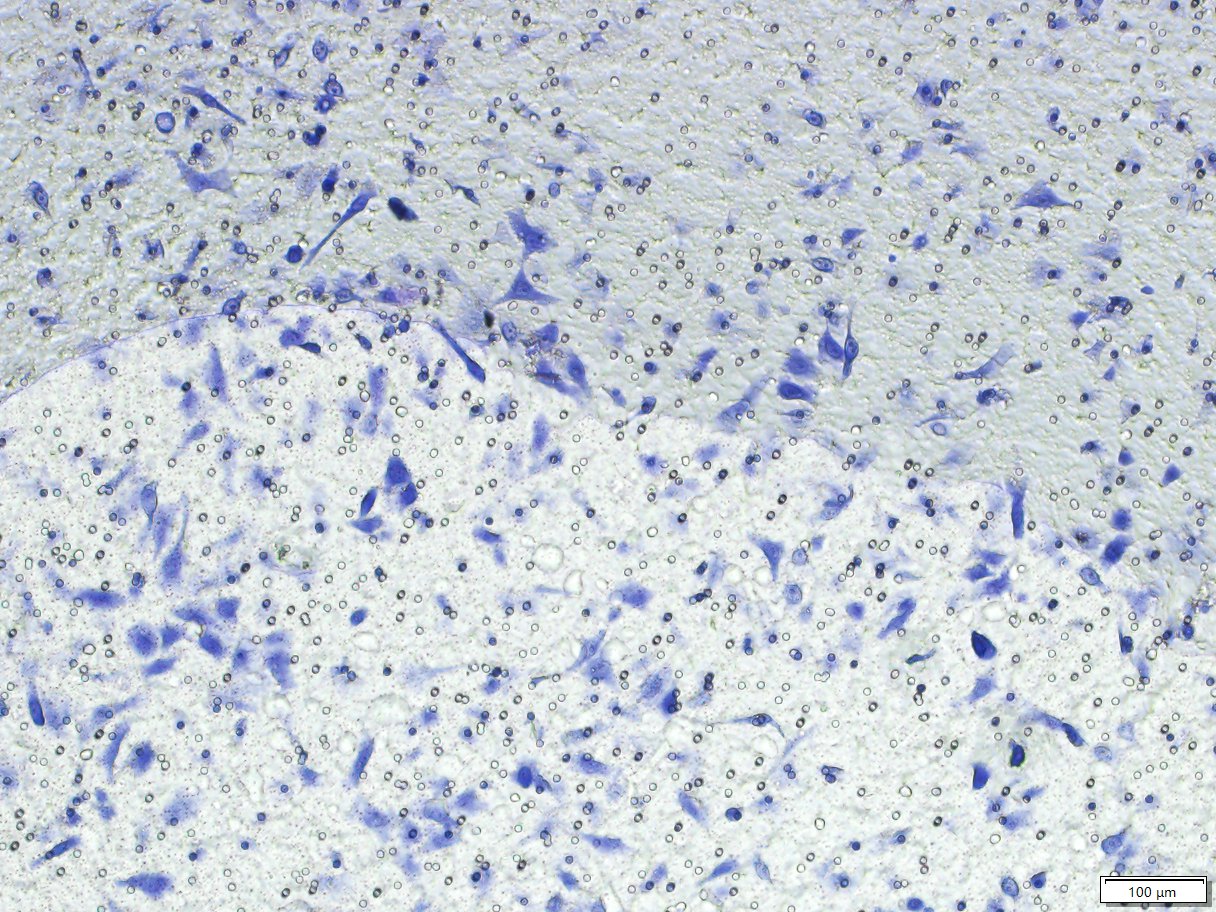

Supplement: Supplemental Information 3 [file peerj-cs-09-1651-s003.zip › Dataset 2/4+9.jpg]

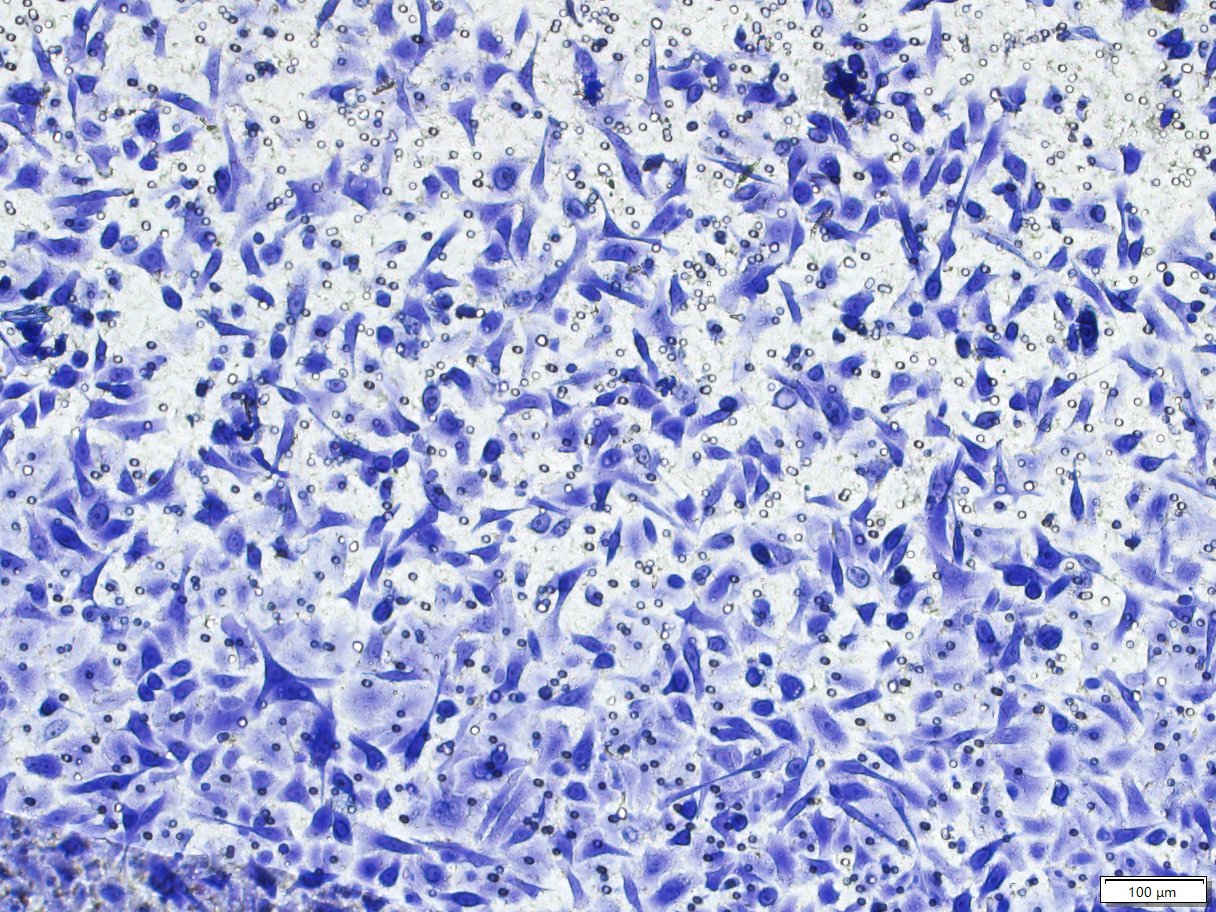

Supplement: Supplemental Information 3 [file peerj-cs-09-1651-s003.zip › Dataset 2/4-1.jpg]

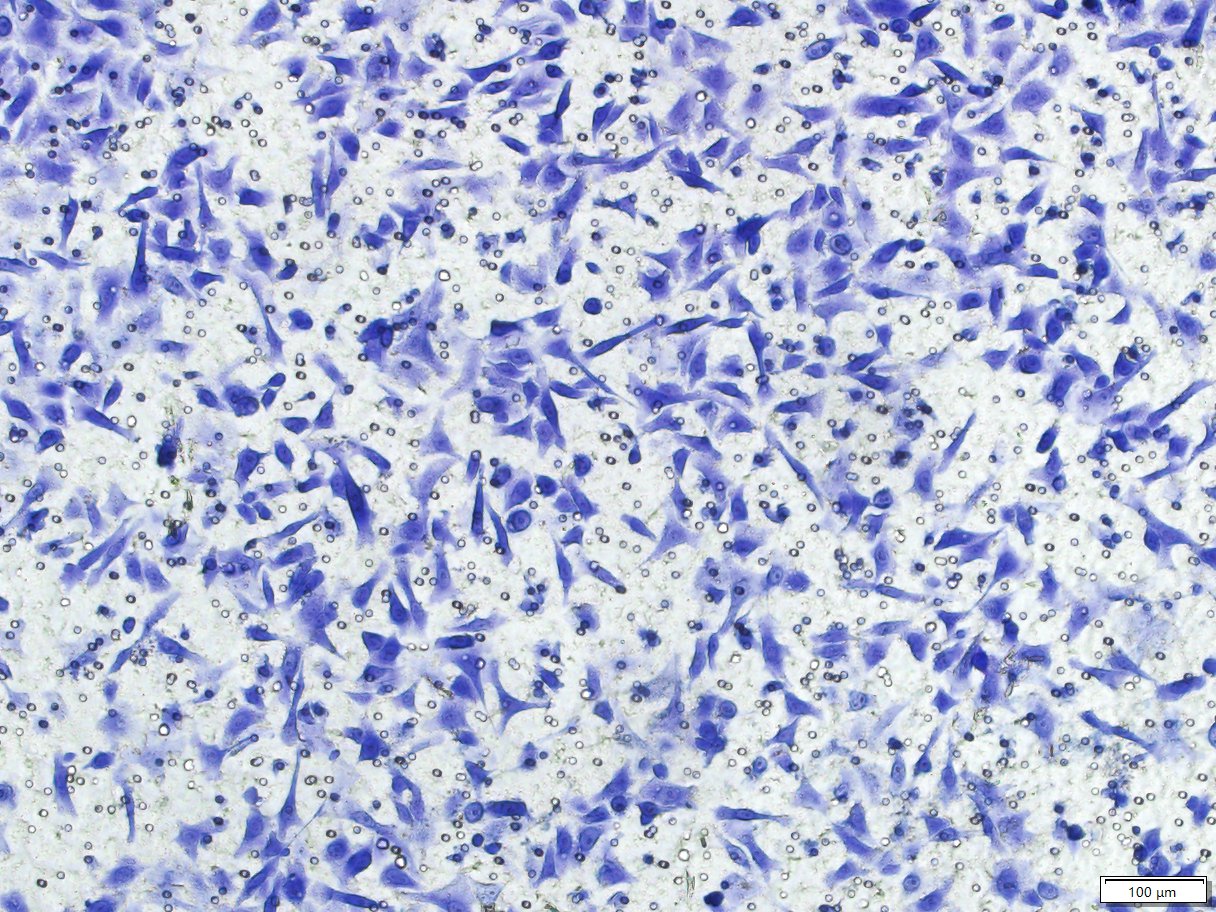

Supplement: Supplemental Information 3 [file peerj-cs-09-1651-s003.zip › Dataset 2/4-10.jpg]

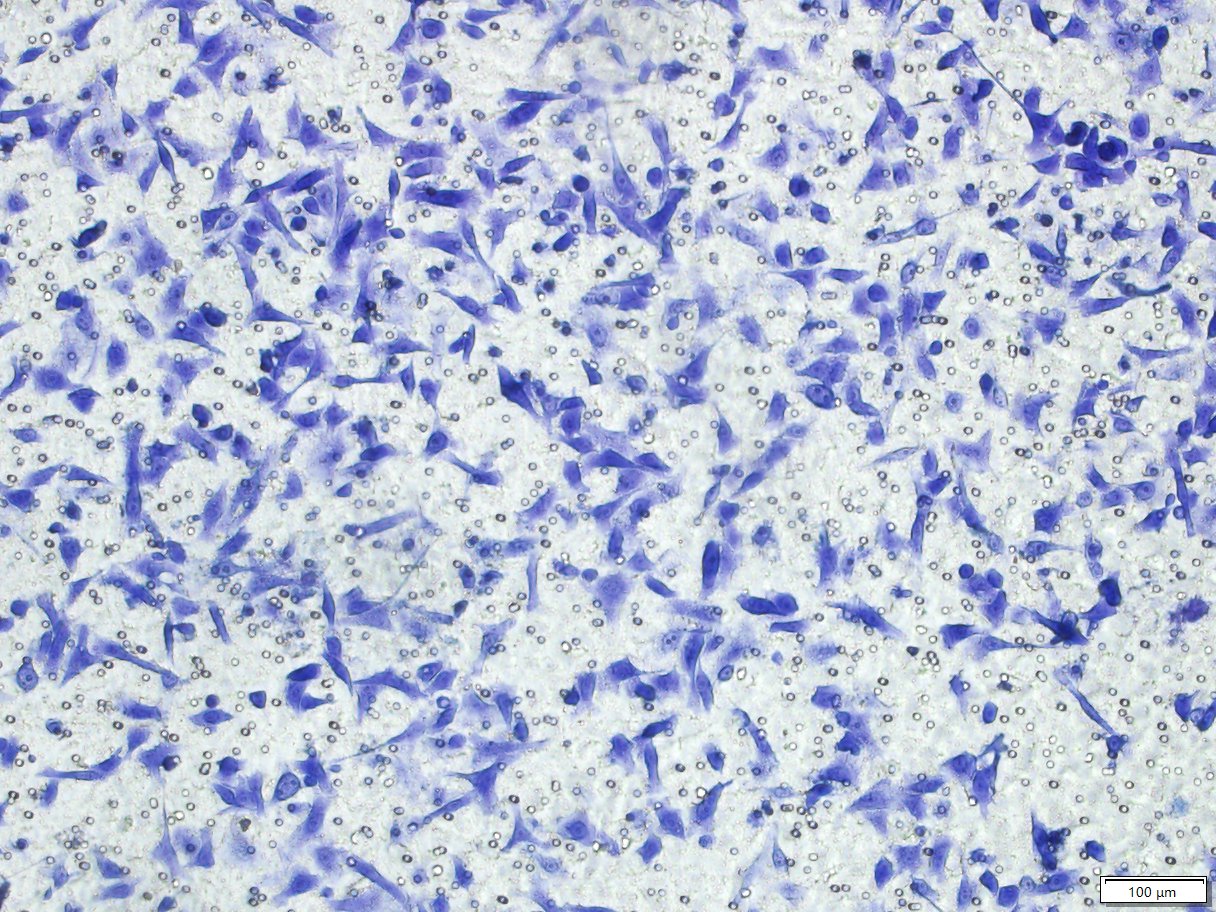

Supplement: Supplemental Information 3 [file peerj-cs-09-1651-s003.zip › Dataset 2/4-11.jpg]

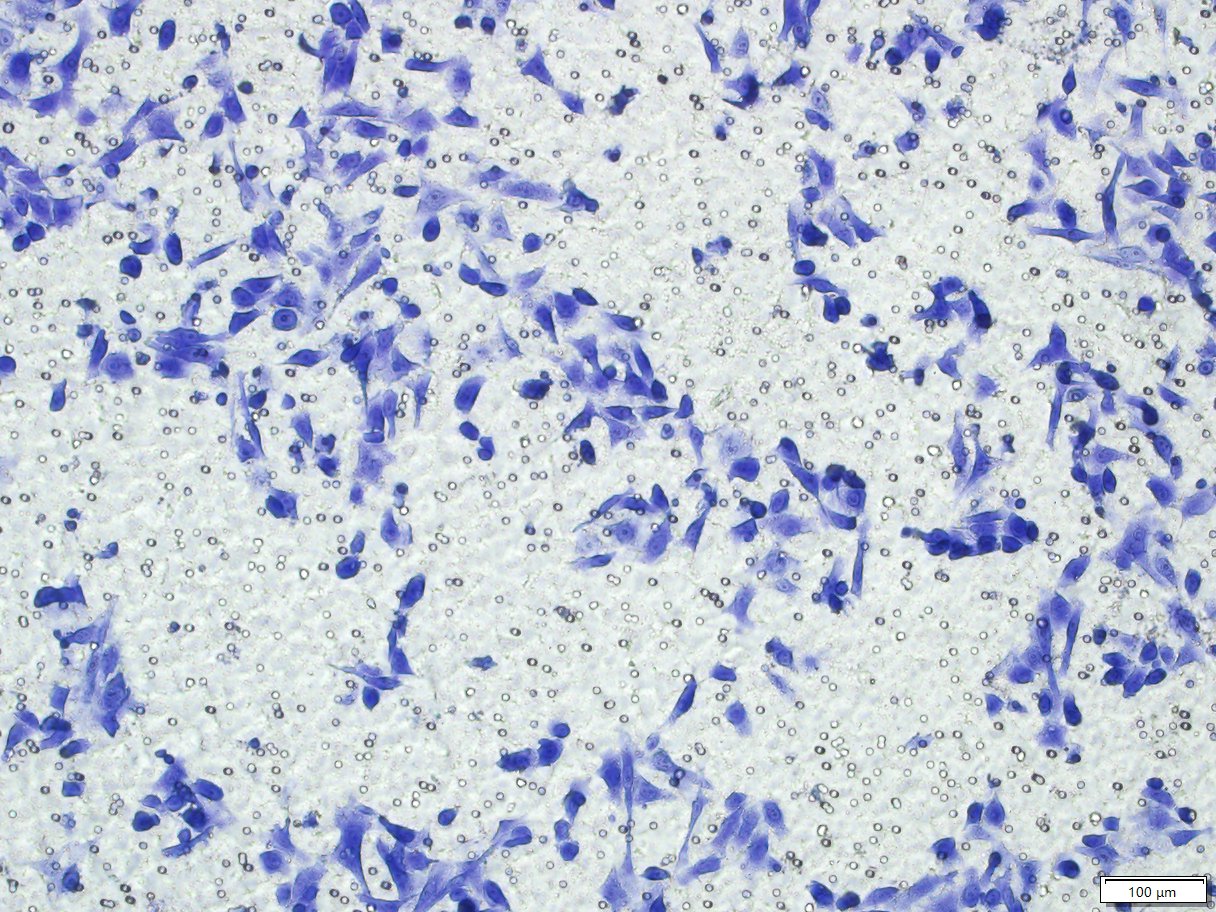

Supplement: Supplemental Information 3 [file peerj-cs-09-1651-s003.zip › Dataset 2/4-12.jpg]

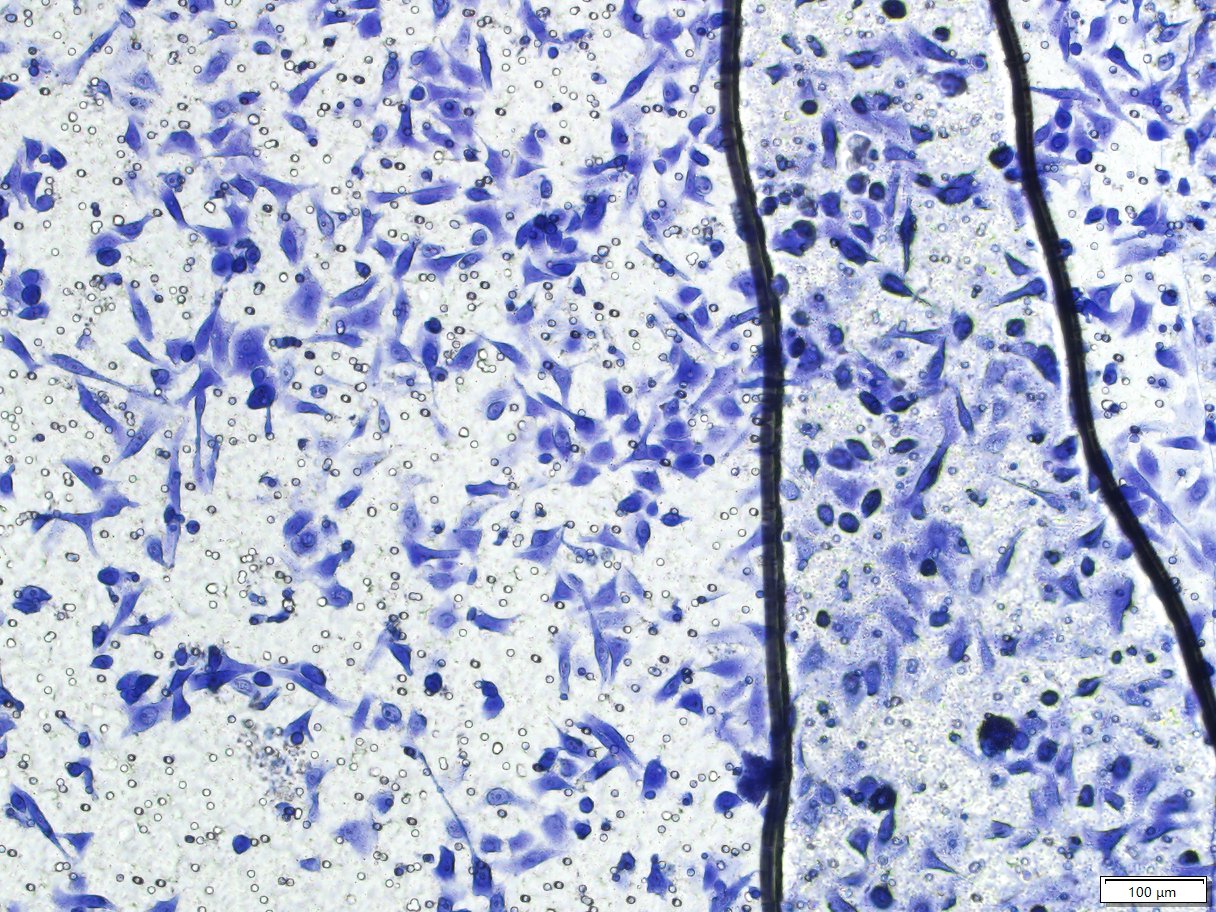

Supplement: Supplemental Information 3 [file peerj-cs-09-1651-s003.zip › Dataset 2/4-13.jpg]

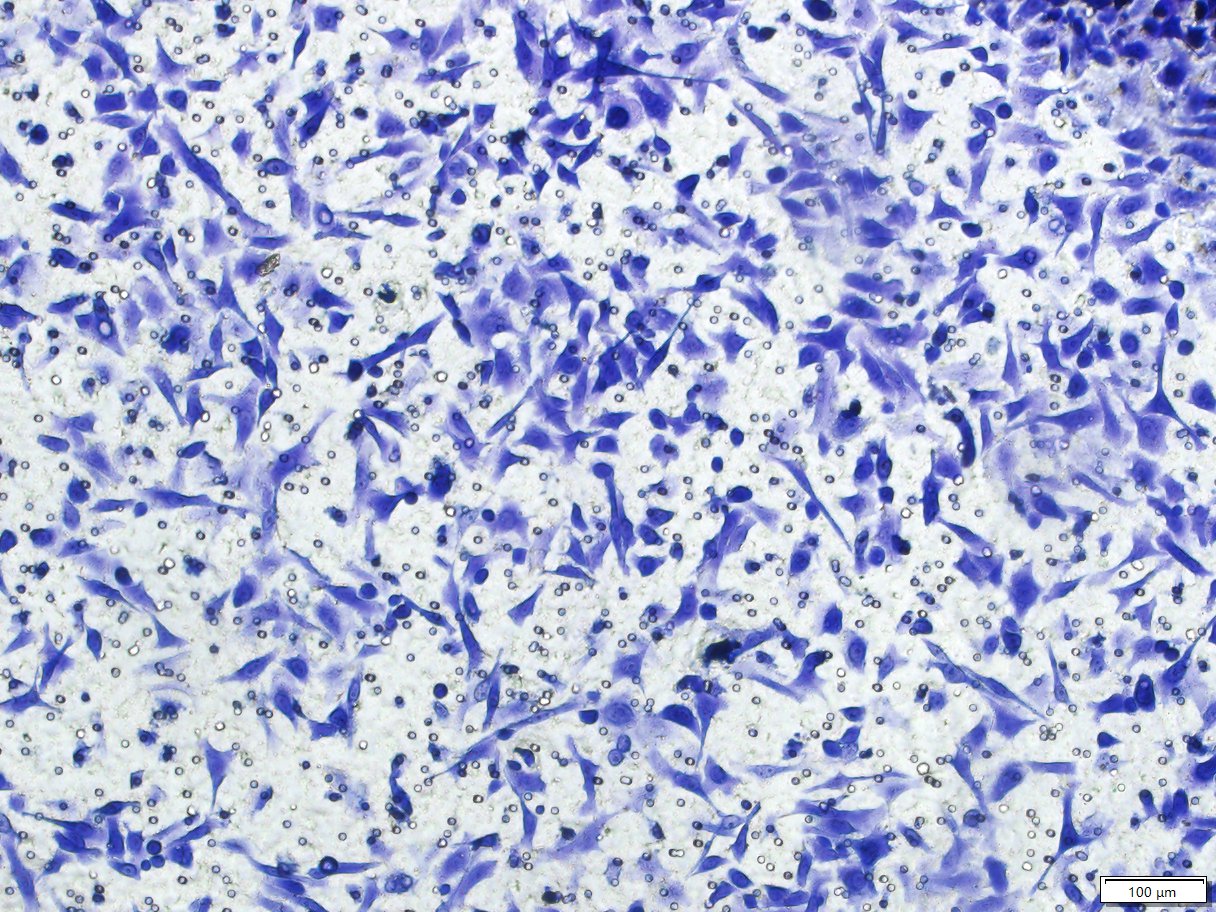

Supplement: Supplemental Information 3 [file peerj-cs-09-1651-s003.zip › Dataset 2/4-14.jpg]

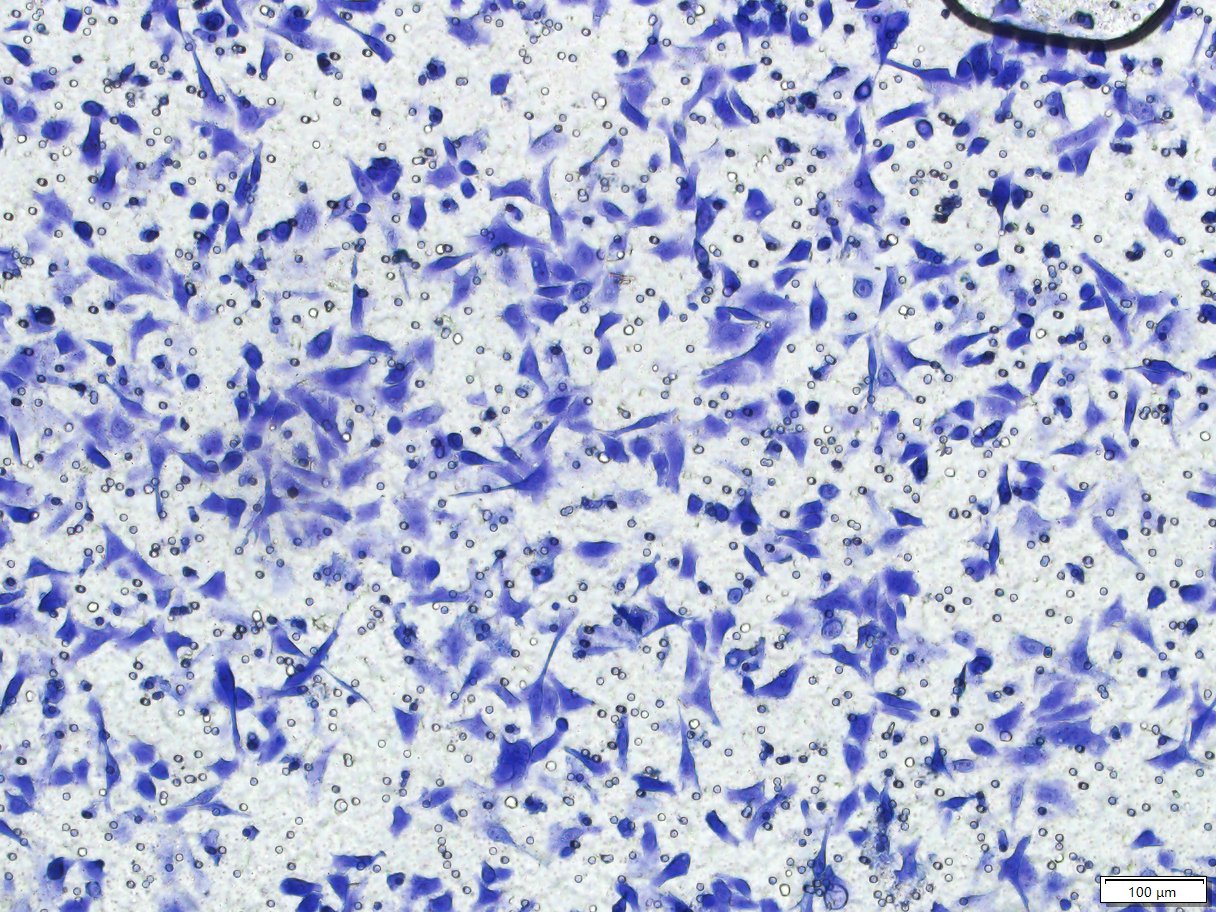

Supplement: Supplemental Information 3 [file peerj-cs-09-1651-s003.zip › Dataset 2/4-15.jpg]

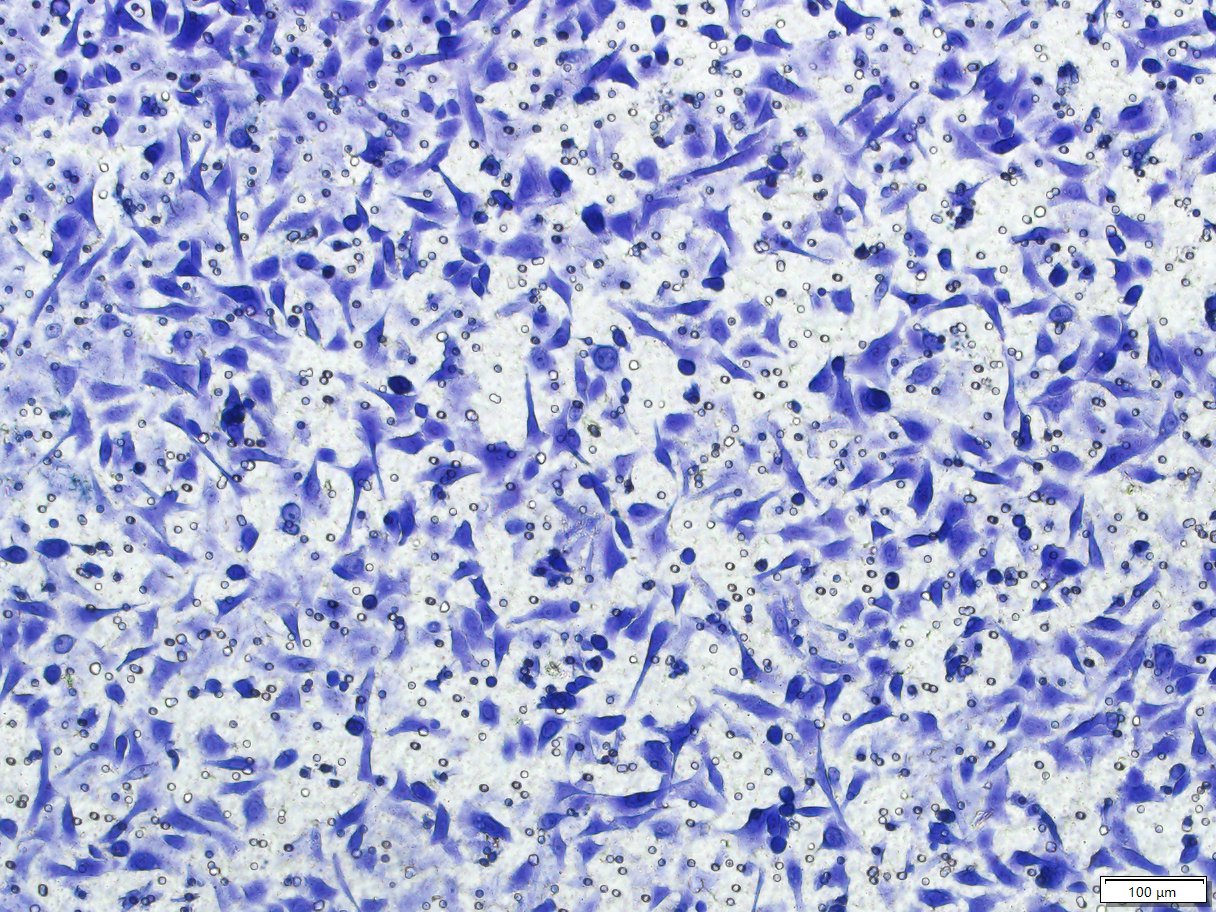

Supplement: Supplemental Information 3 [file peerj-cs-09-1651-s003.zip › Dataset 2/4-16.jpg]

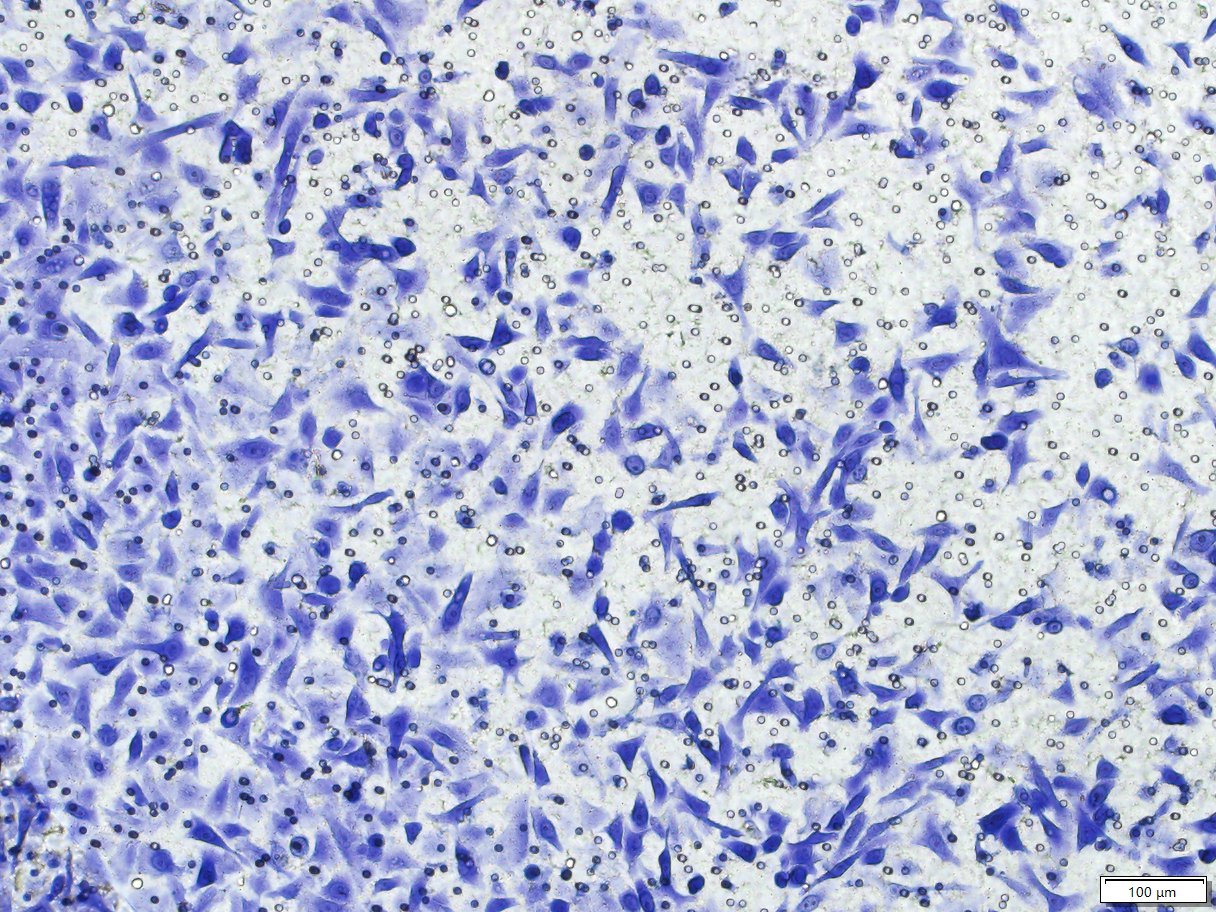

Supplement: Supplemental Information 3 [file peerj-cs-09-1651-s003.zip › Dataset 2/4-2.jpg]

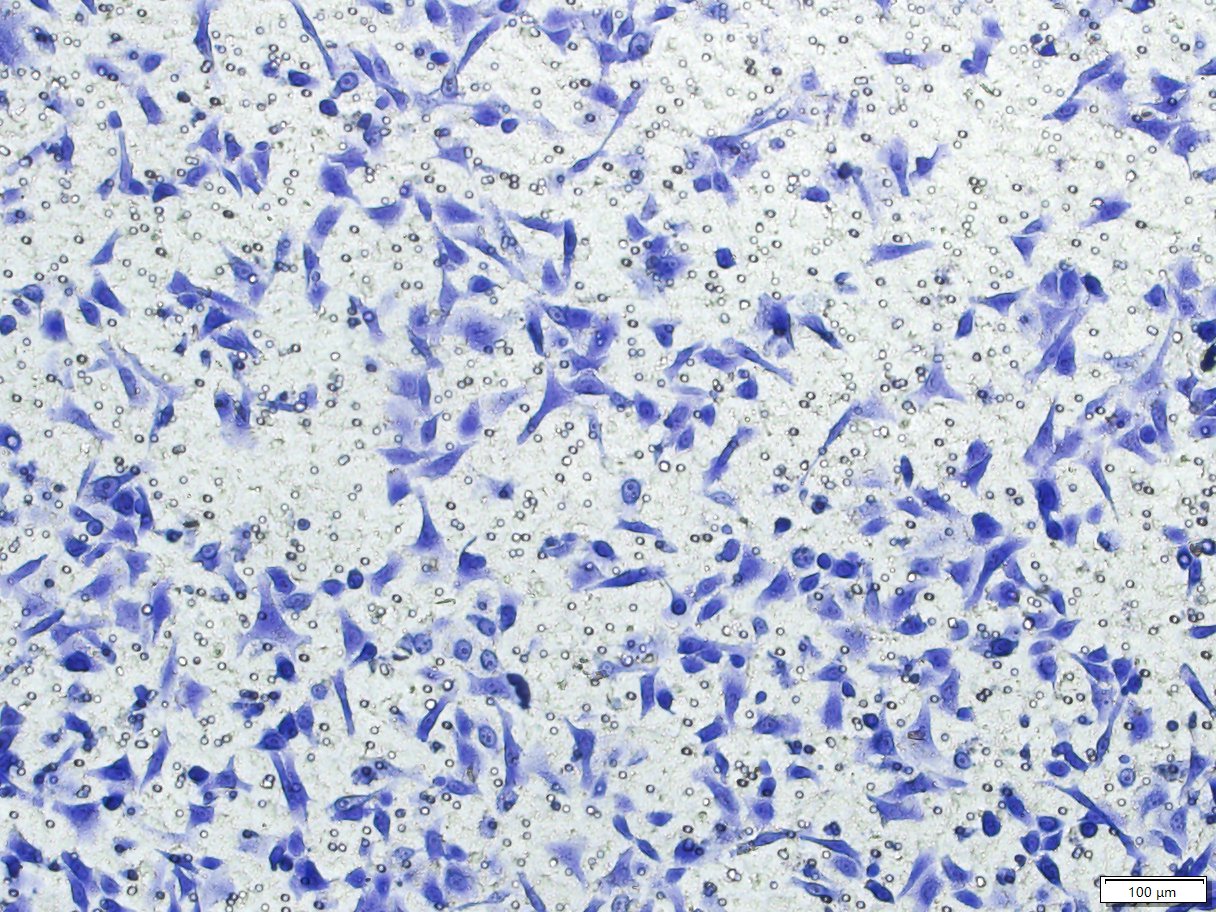

Supplement: Supplemental Information 3 [file peerj-cs-09-1651-s003.zip › Dataset 2/4-3.jpg]

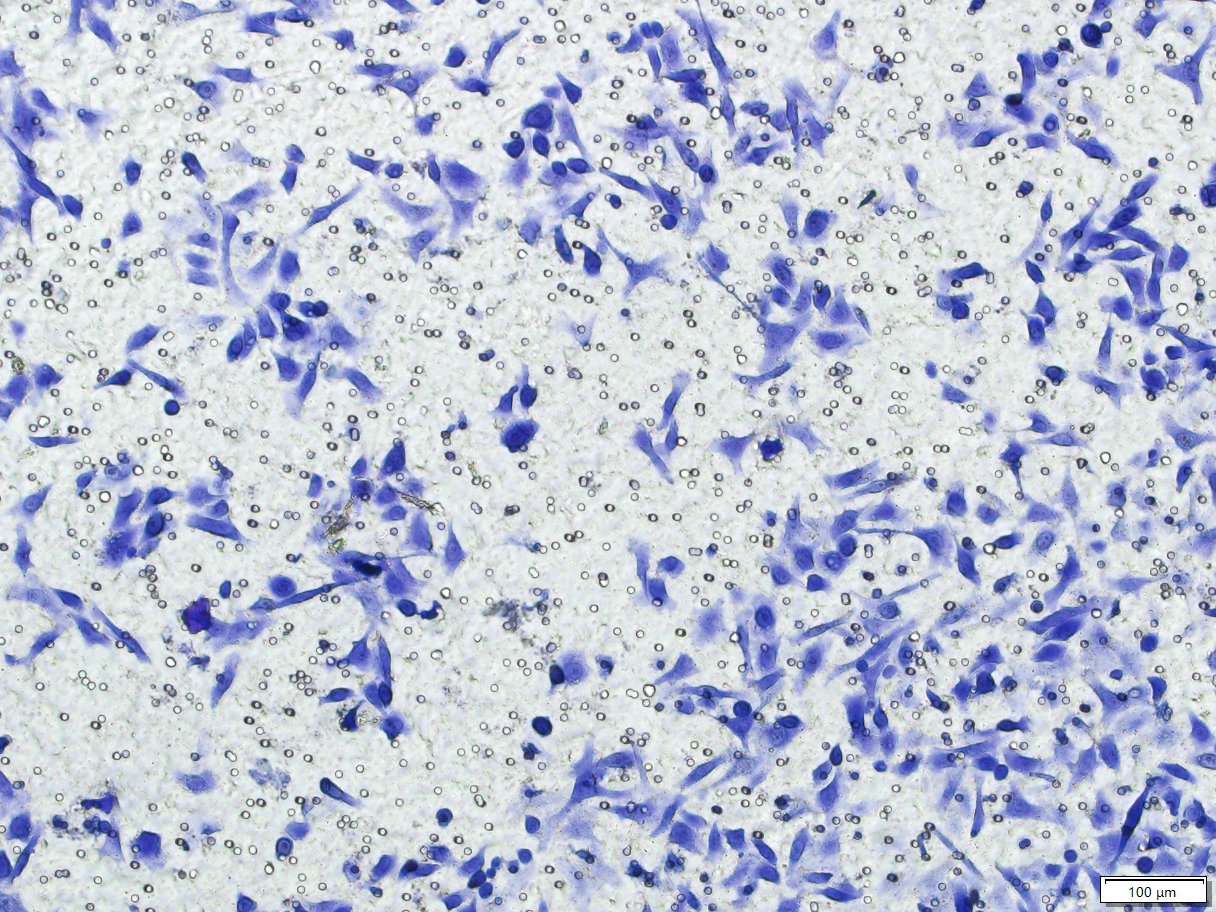

Supplement: Supplemental Information 3 [file peerj-cs-09-1651-s003.zip › Dataset 2/4-4.jpg]

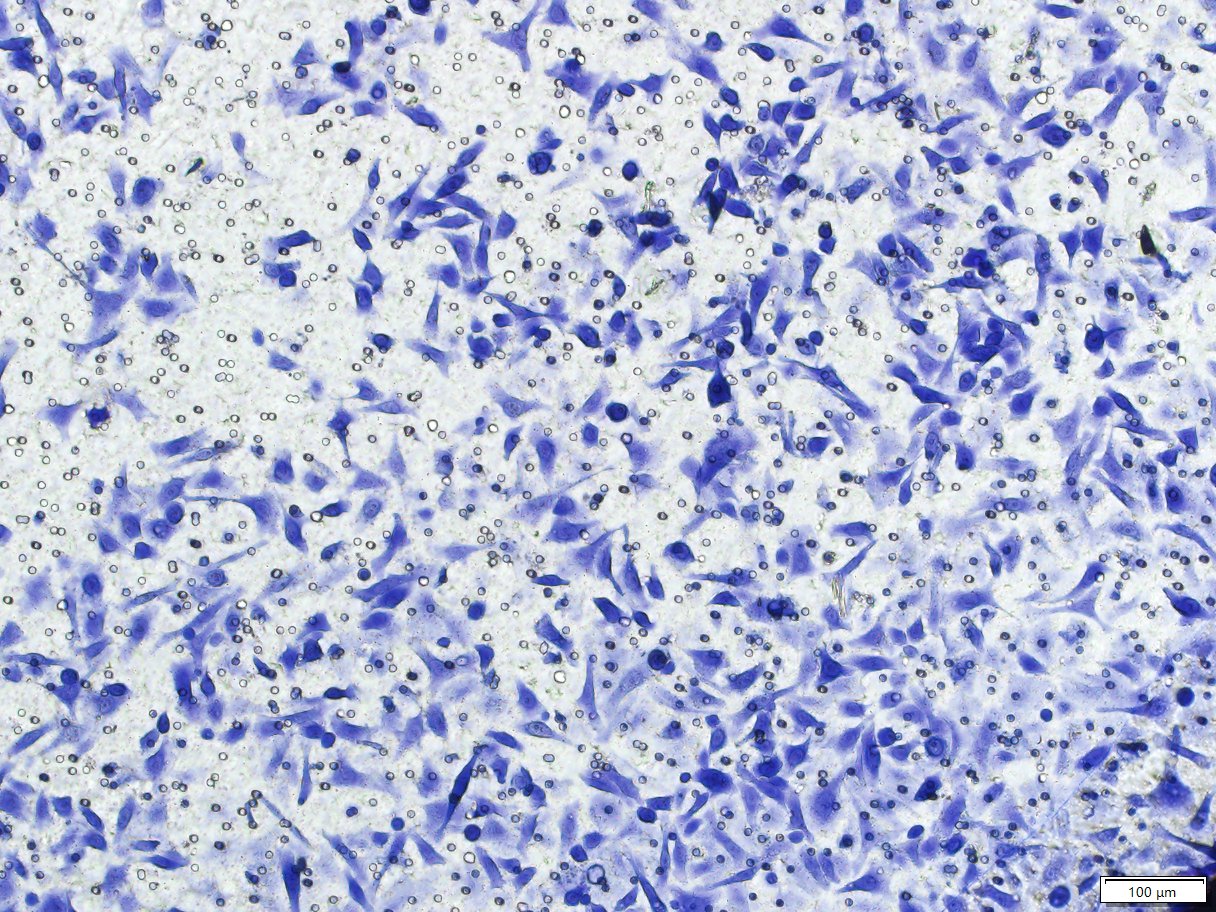

Supplement: Supplemental Information 3 [file peerj-cs-09-1651-s003.zip › Dataset 2/4-5.jpg]

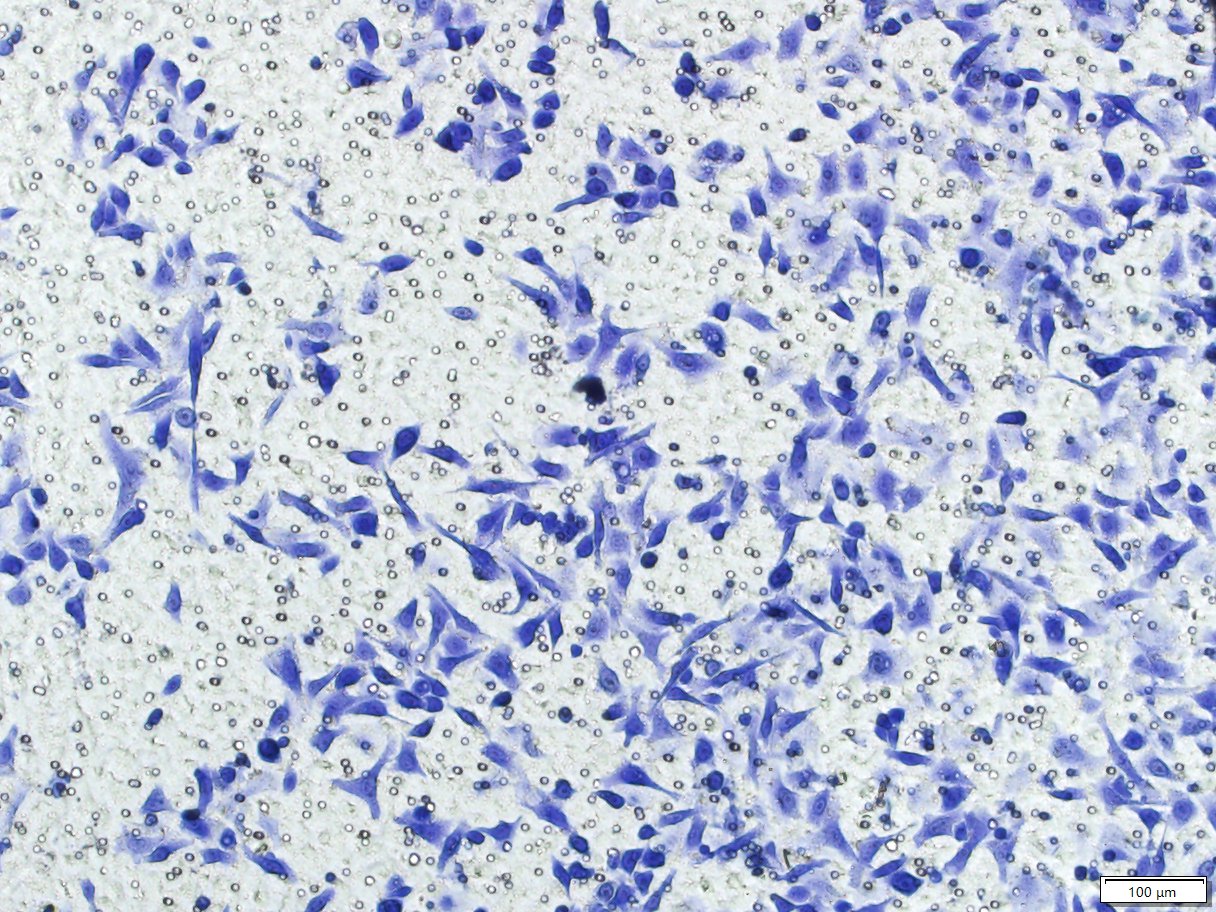

Supplement: Supplemental Information 3 [file peerj-cs-09-1651-s003.zip › Dataset 2/4-6.jpg]

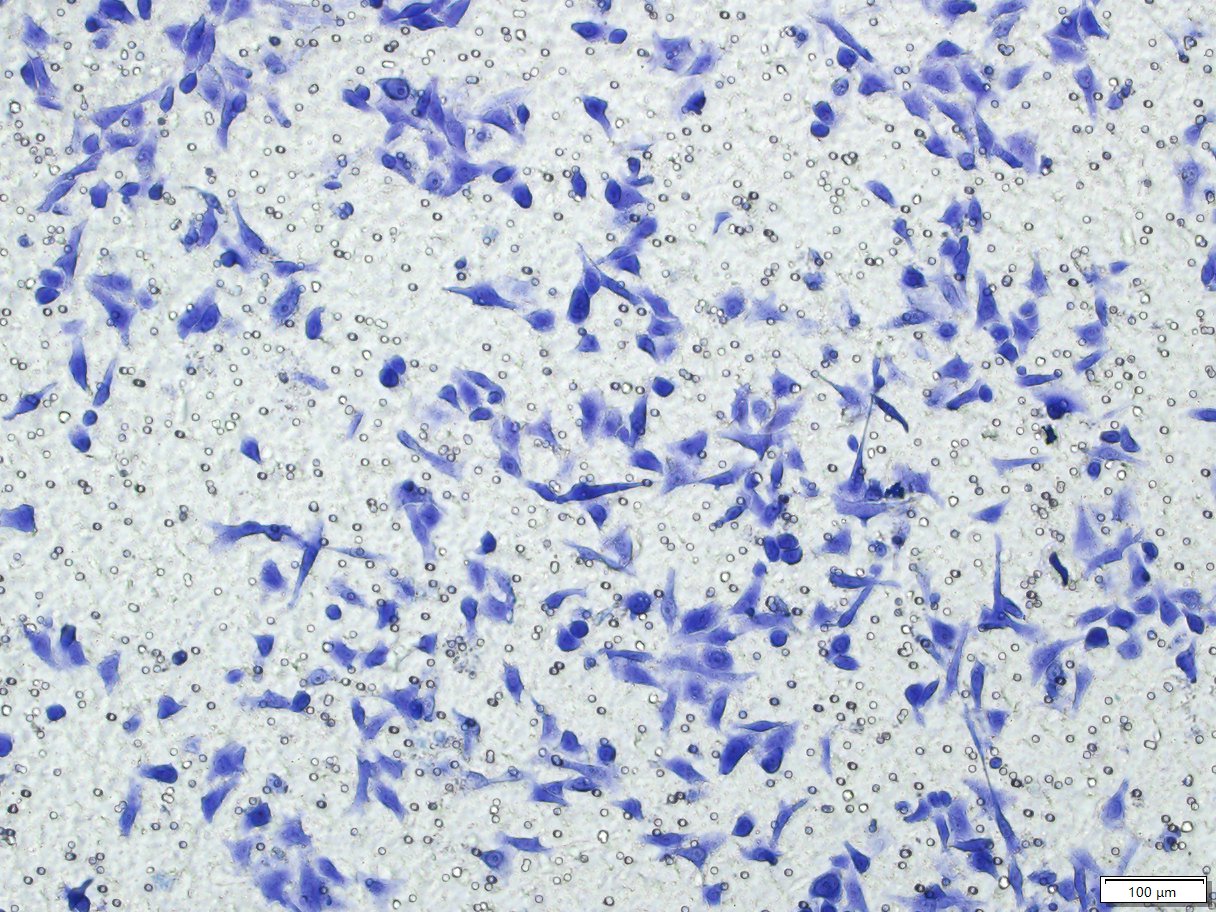

Supplement: Supplemental Information 3 [file peerj-cs-09-1651-s003.zip › Dataset 2/4-7.jpg]

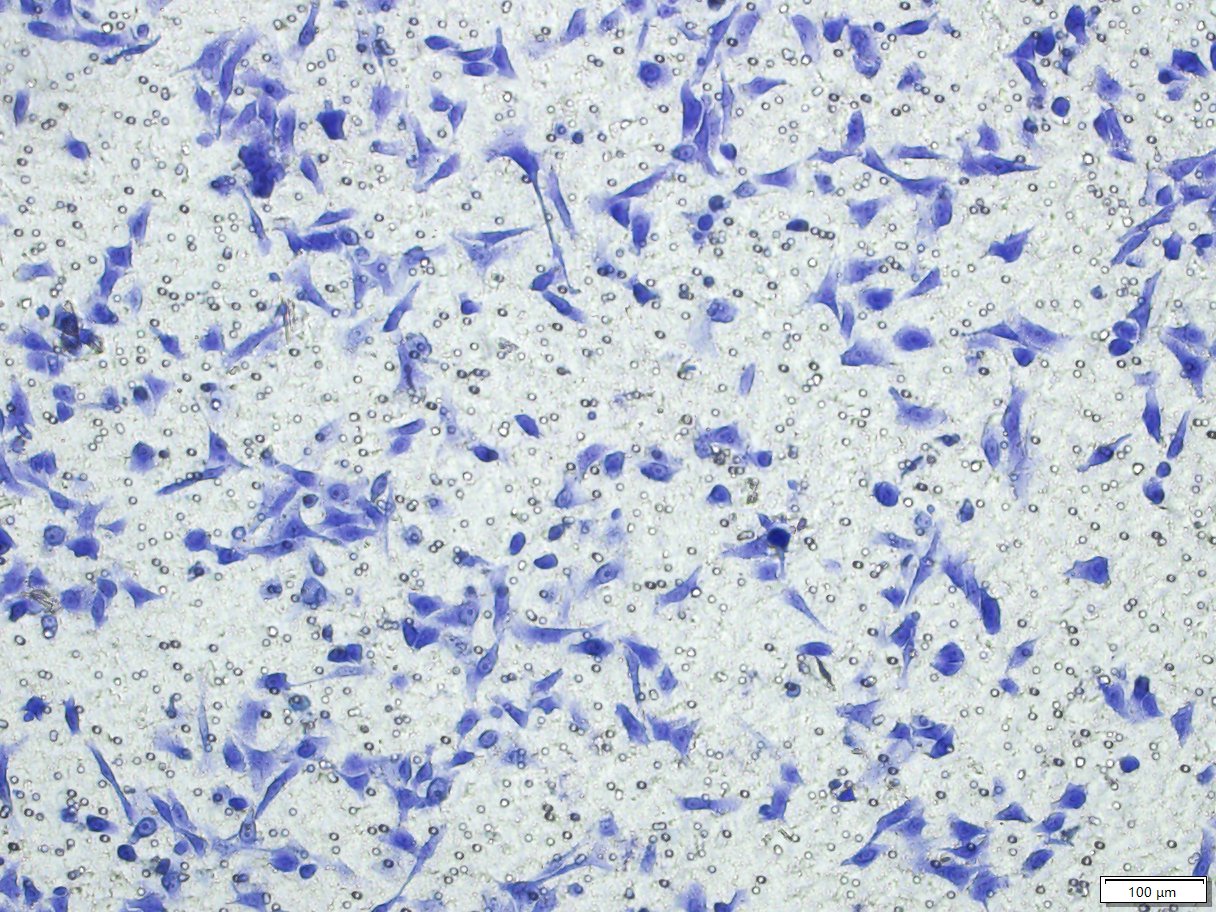

Supplement: Supplemental Information 3 [file peerj-cs-09-1651-s003.zip › Dataset 2/4-8.jpg]

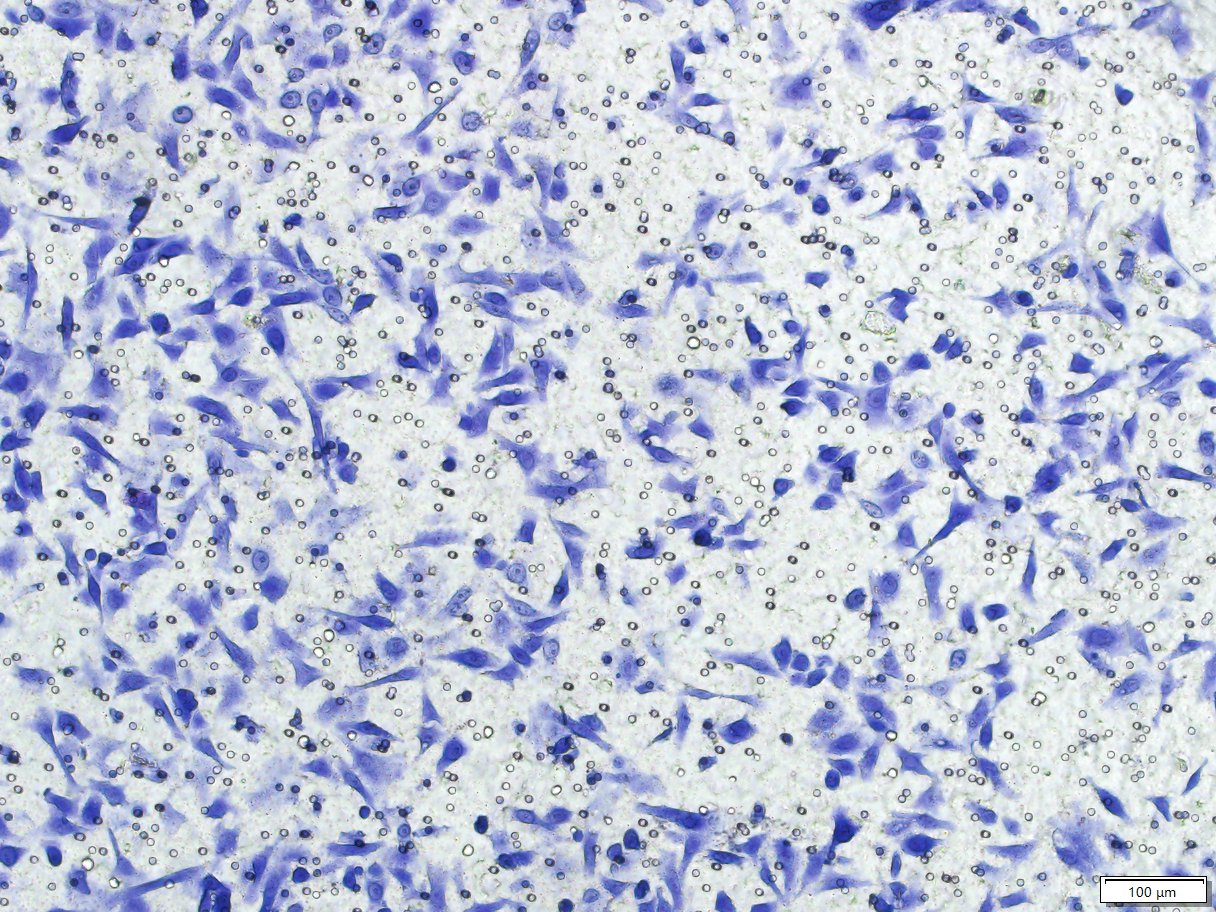

Supplement: Supplemental Information 3 [file peerj-cs-09-1651-s003.zip › Dataset 2/4-9.jpg]

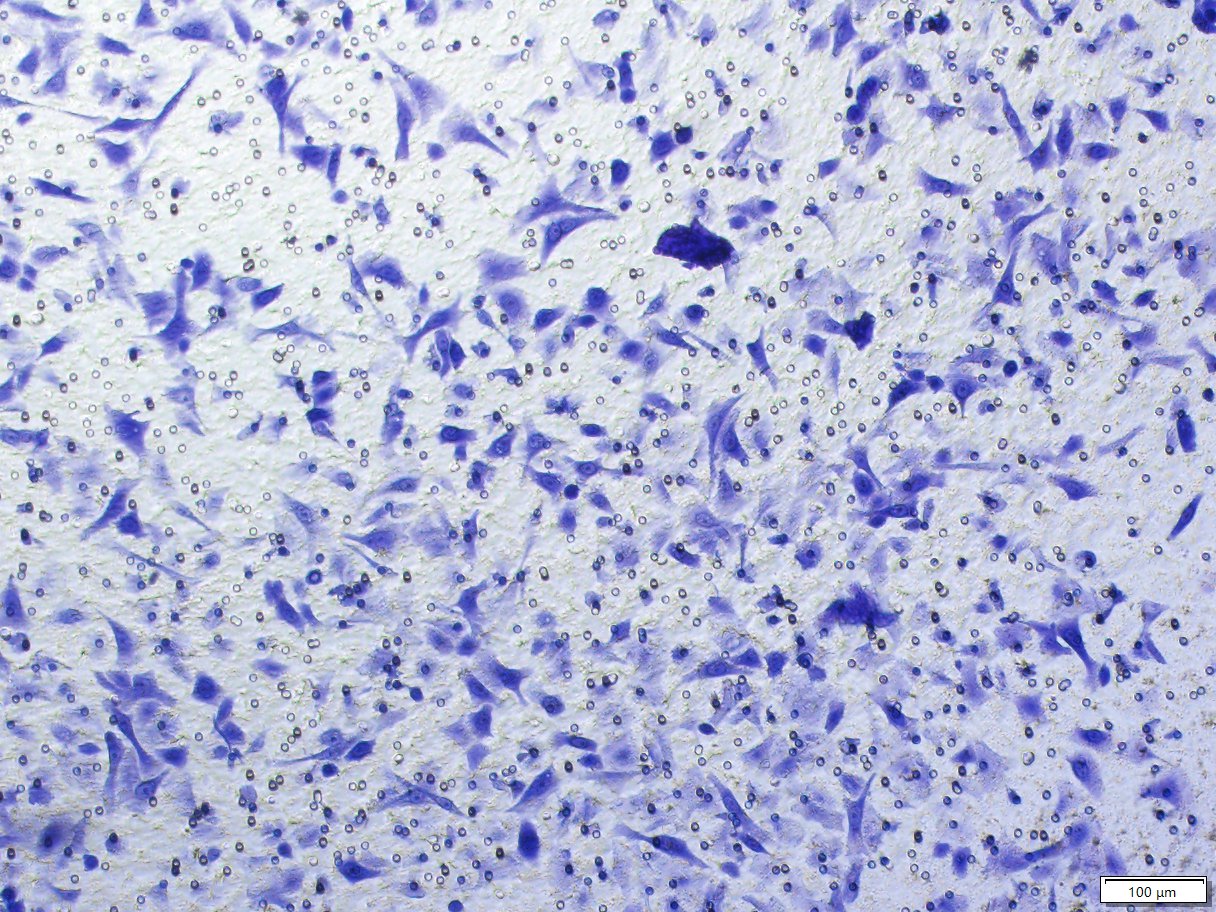

Supplement: Supplemental Information 3 [file peerj-cs-09-1651-s003.zip › Dataset 2/5+1.jpg]

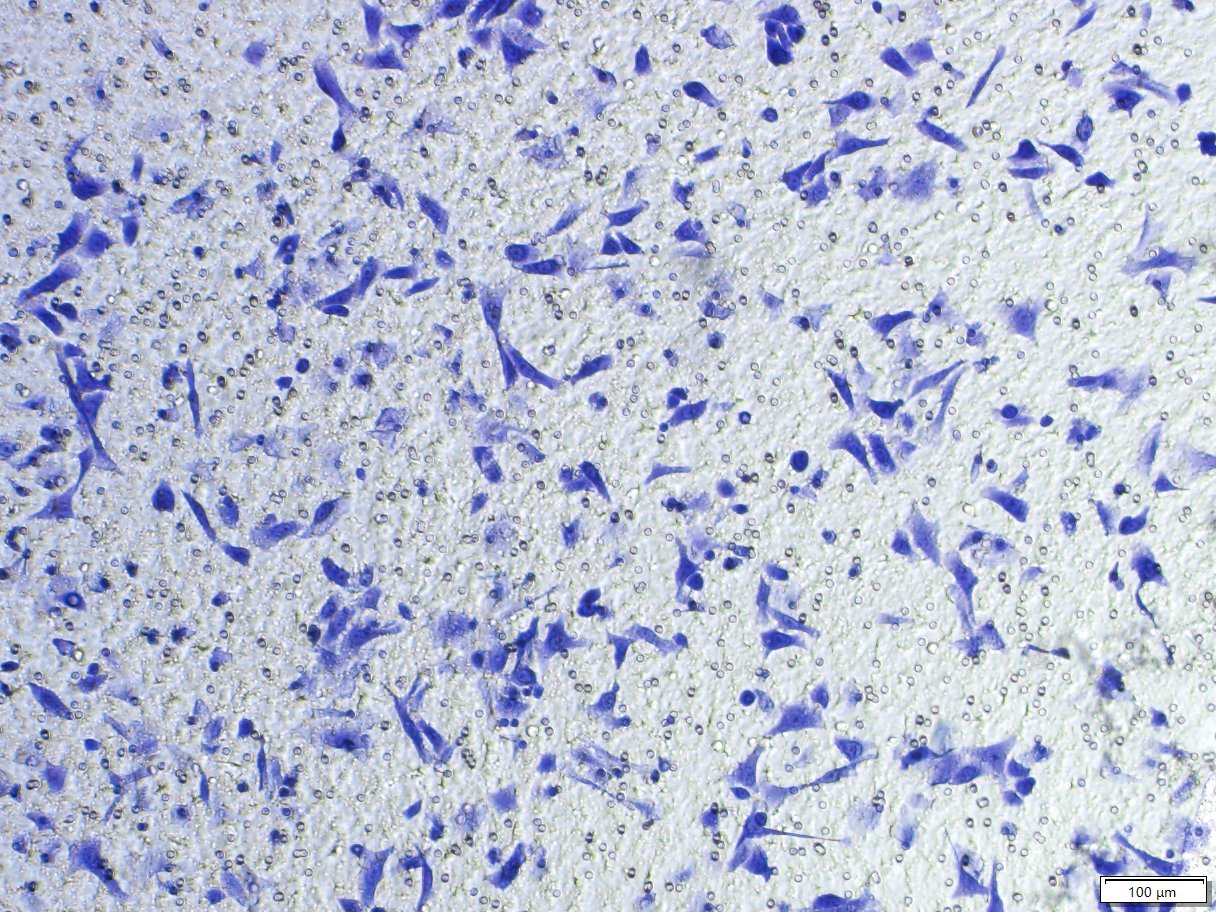

Supplement: Supplemental Information 3 [file peerj-cs-09-1651-s003.zip › Dataset 2/5+10.jpg]

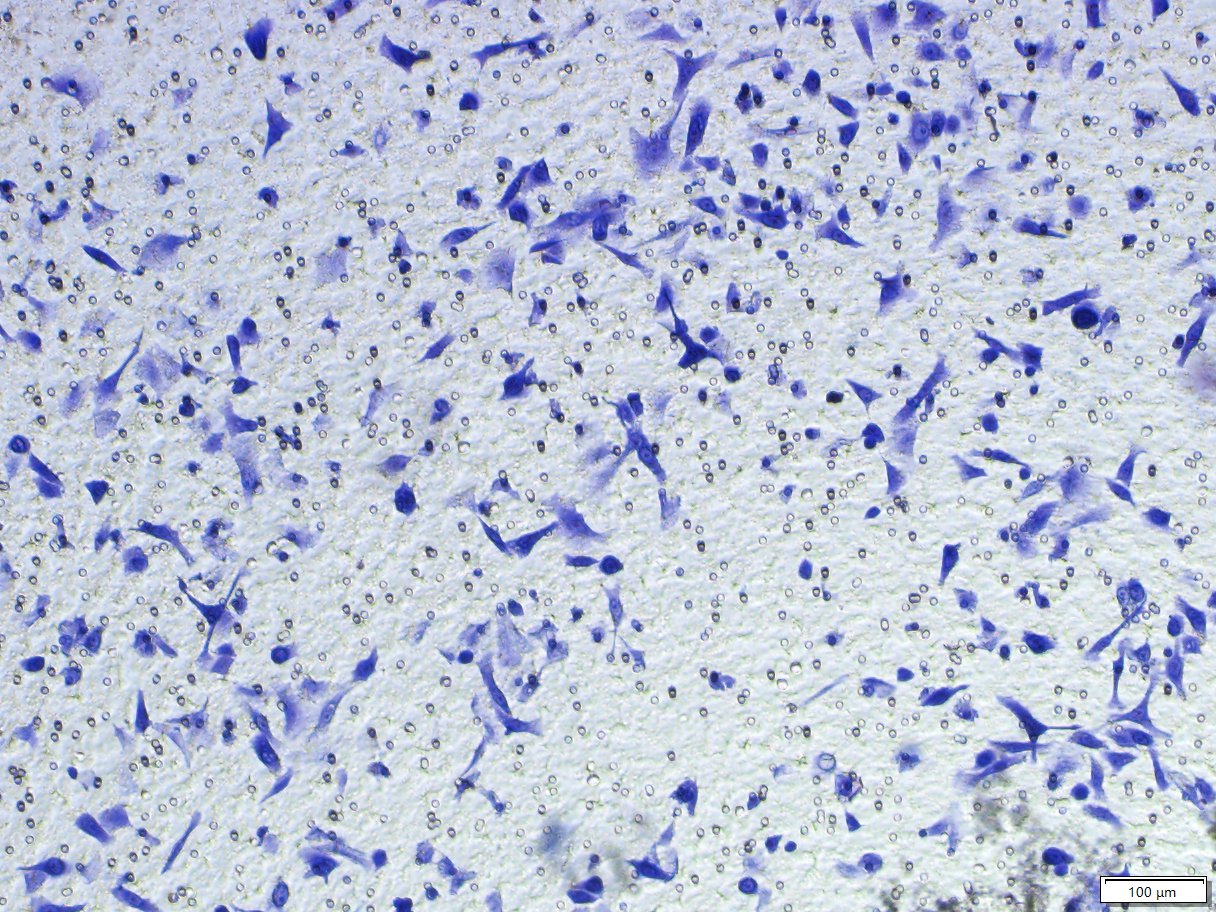

Supplement: Supplemental Information 3 [file peerj-cs-09-1651-s003.zip › Dataset 2/5+11.jpg]

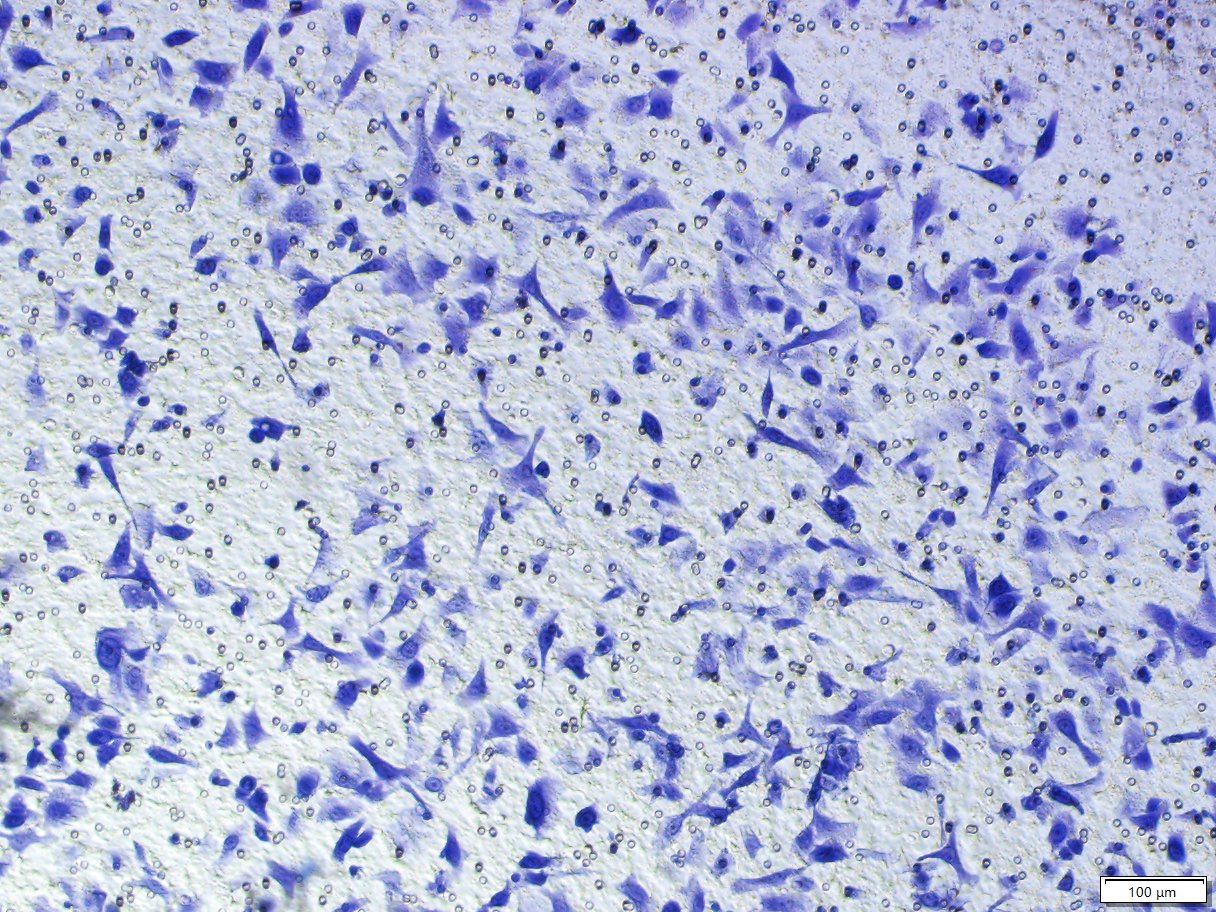

Supplement: Supplemental Information 3 [file peerj-cs-09-1651-s003.zip › Dataset 2/5+12.jpg]
