# Supplementary figures and images for: A deep learning approach for the detection and counting of colon cancer cells (HT-29 cells) bunches and impurities (part 2 of 6)
Source: PeerJ Comput Sci. 2023 Dec 5;9:e1651. doi: 10.7717/peerj-cs.1651 (PMC10773923; doi:10.7717/peerj-cs.1651)

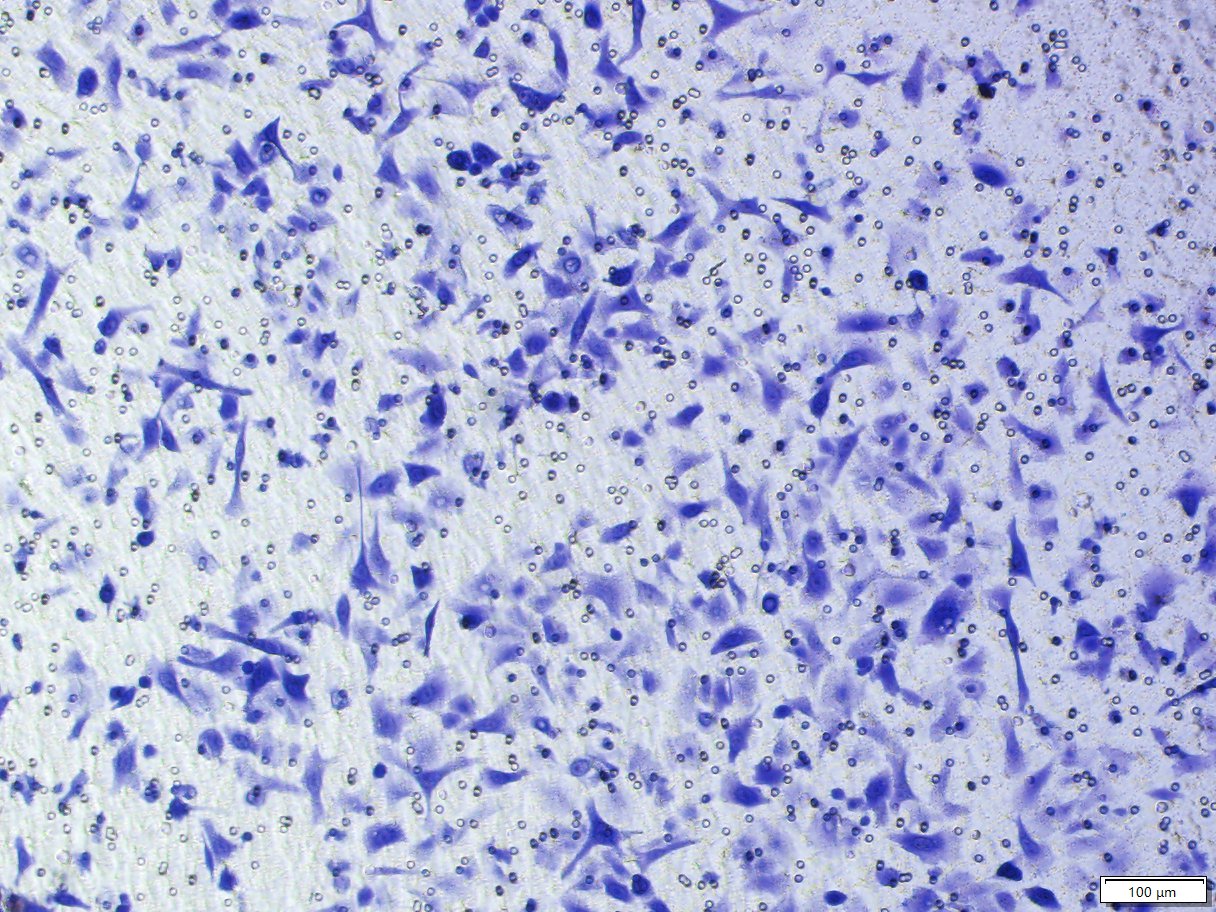

Supplement: Supplemental Information 3 [file peerj-cs-09-1651-s003.zip › Dataset 2/5+13.jpg]

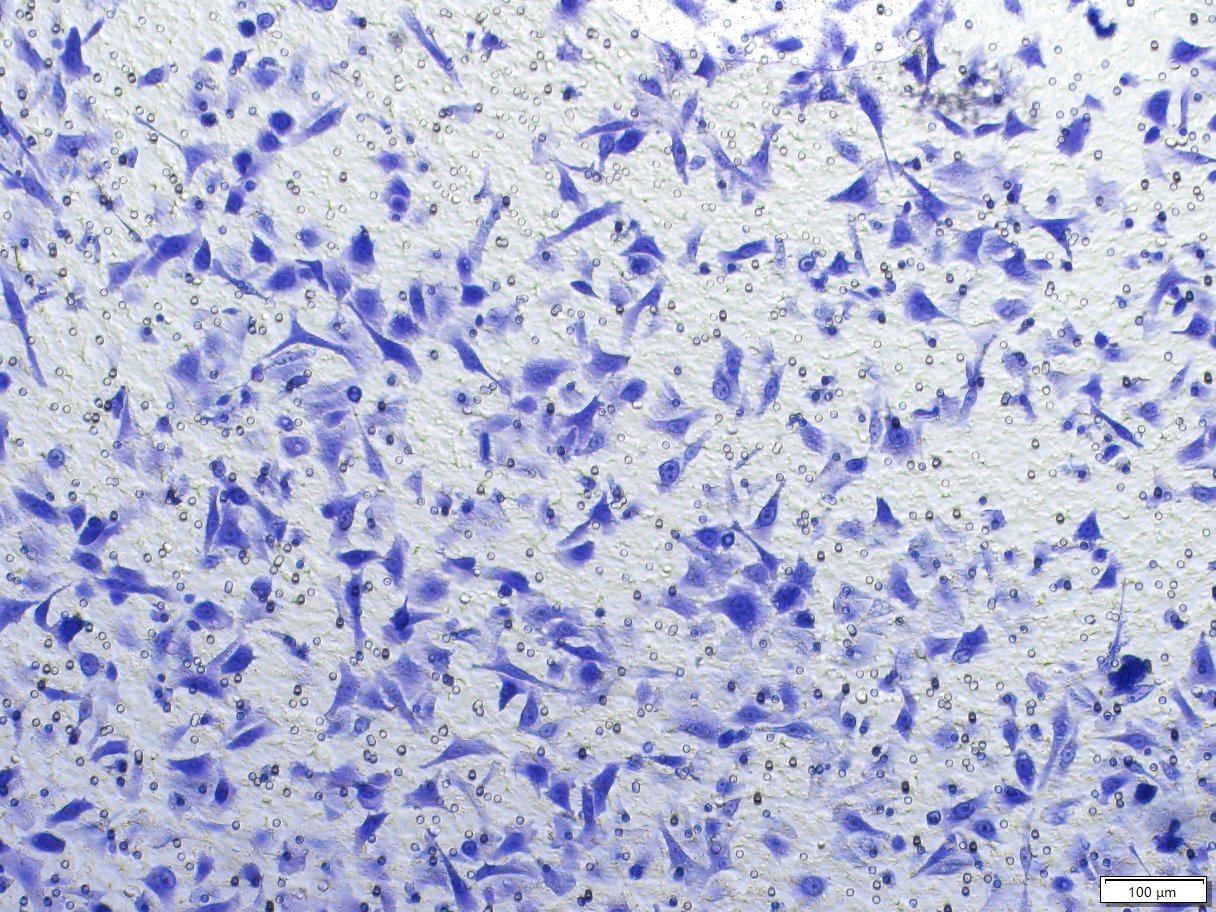

Supplement: Supplemental Information 3 [file peerj-cs-09-1651-s003.zip › Dataset 2/5+2.jpg]

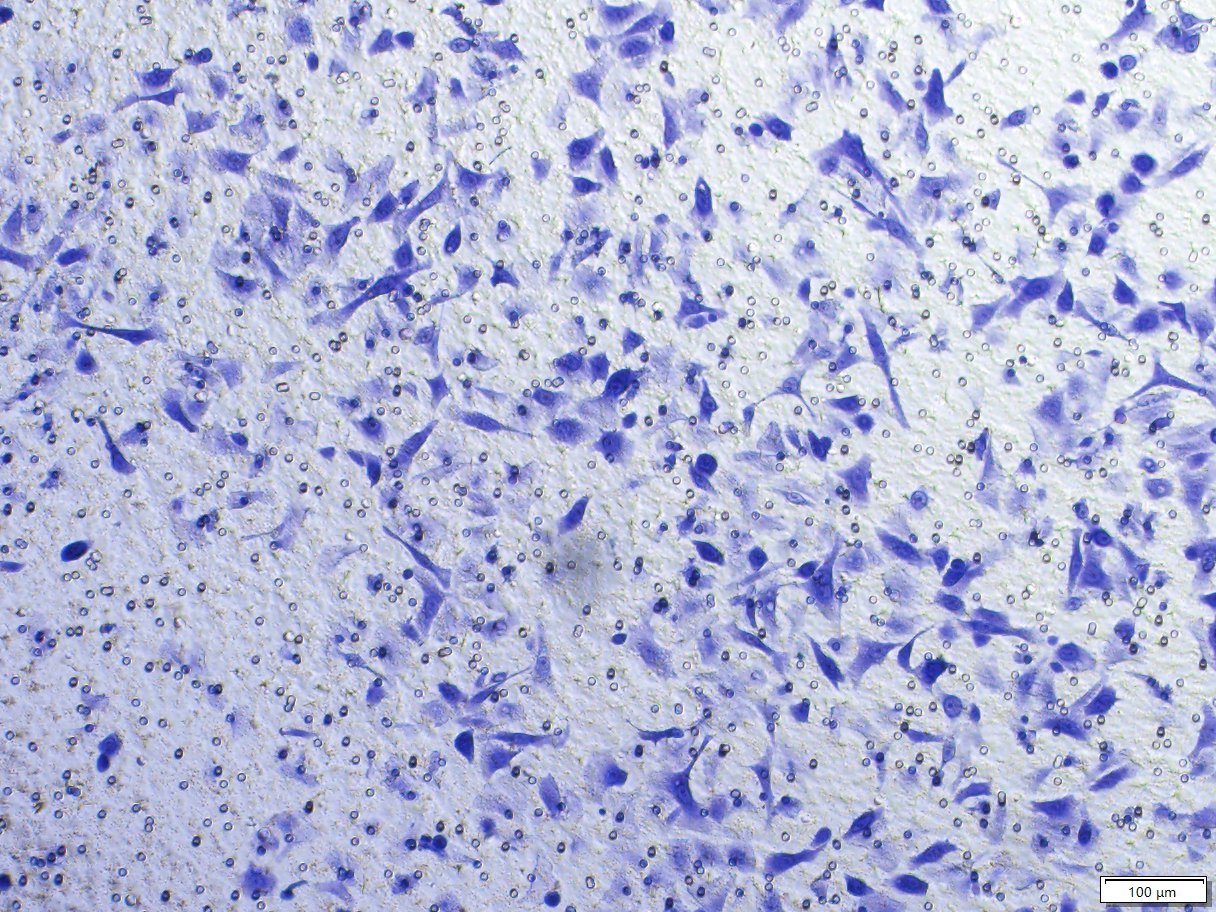

Supplement: Supplemental Information 3 [file peerj-cs-09-1651-s003.zip › Dataset 2/5+3.jpg]

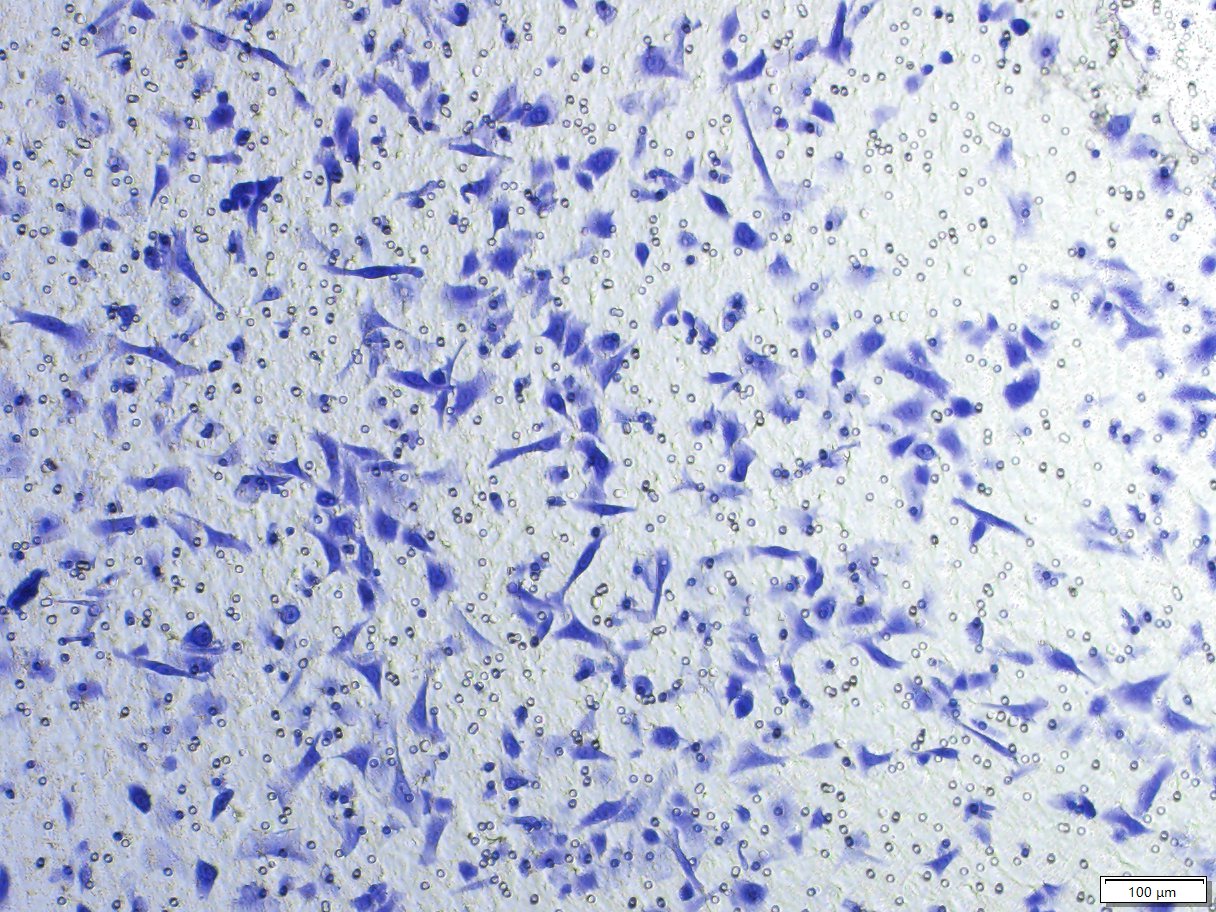

Supplement: Supplemental Information 3 [file peerj-cs-09-1651-s003.zip › Dataset 2/5+4.jpg]

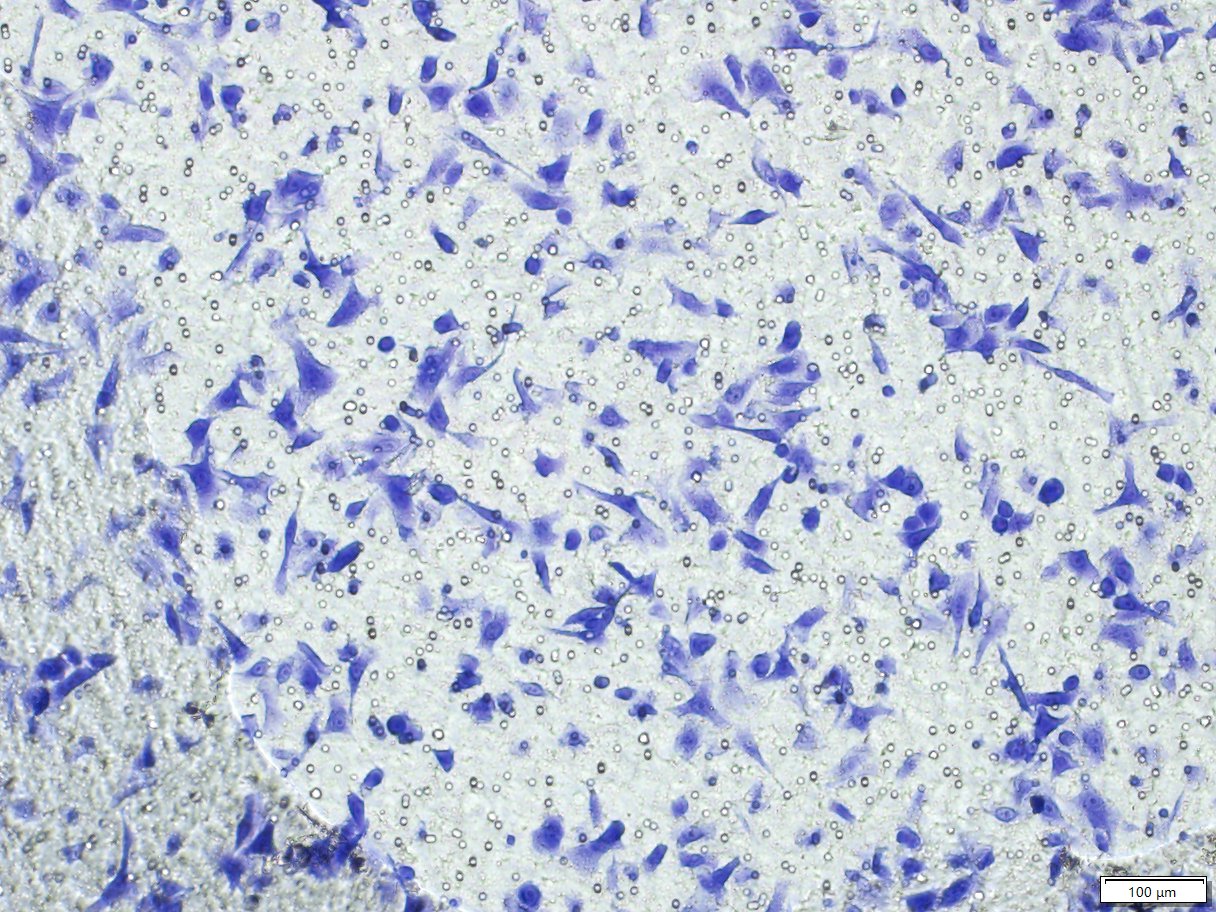

Supplement: Supplemental Information 3 [file peerj-cs-09-1651-s003.zip › Dataset 2/5+5.jpg]

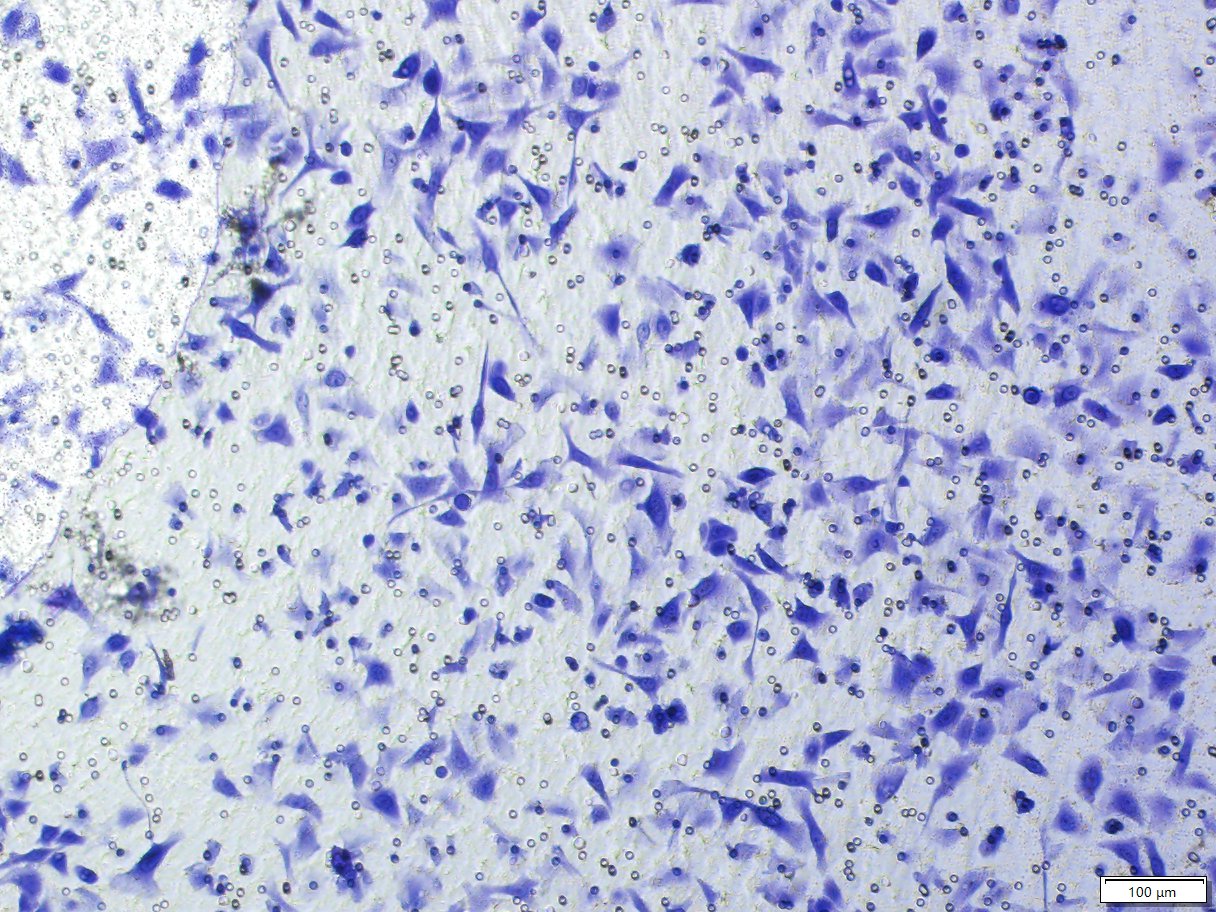

Supplement: Supplemental Information 3 [file peerj-cs-09-1651-s003.zip › Dataset 2/5+6.jpg]

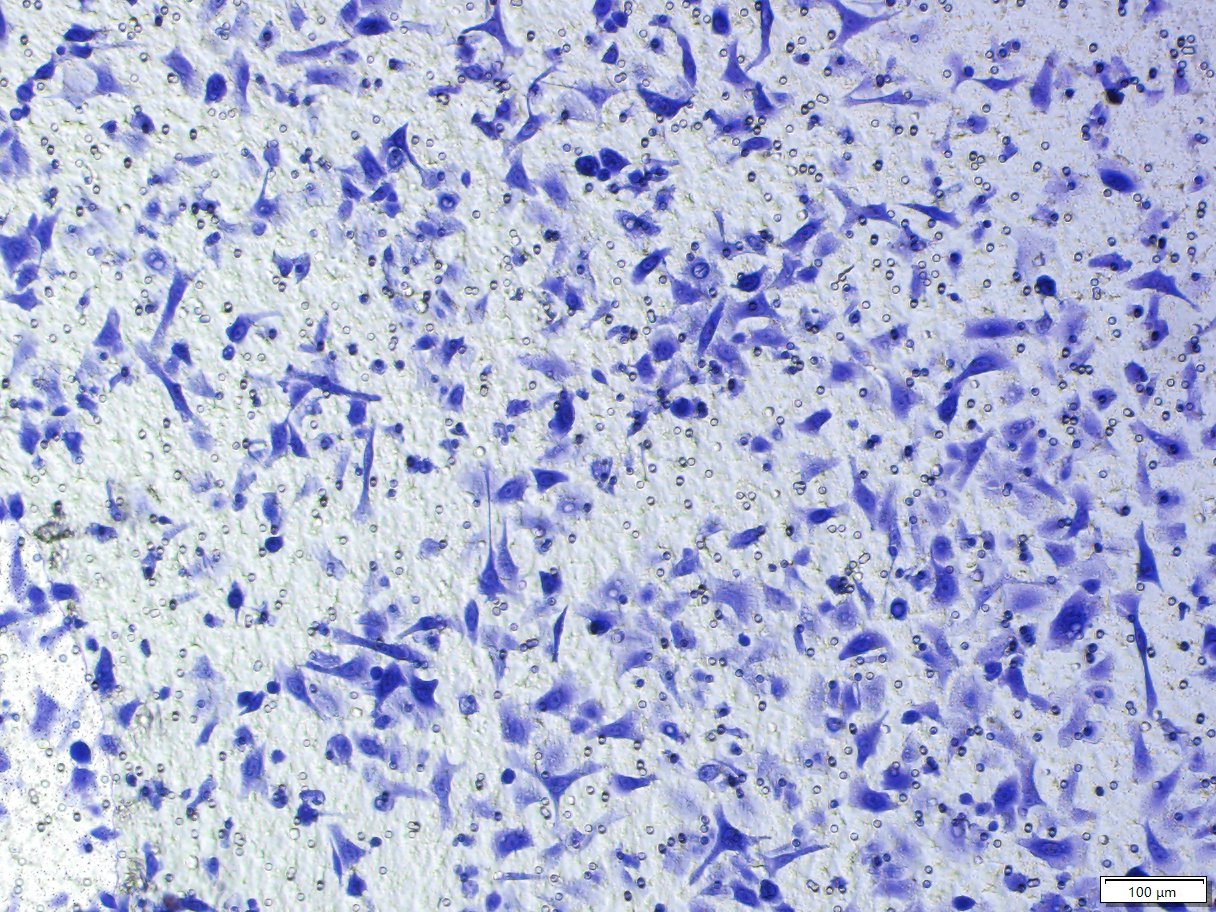

Supplement: Supplemental Information 3 [file peerj-cs-09-1651-s003.zip › Dataset 2/5+7.jpg]

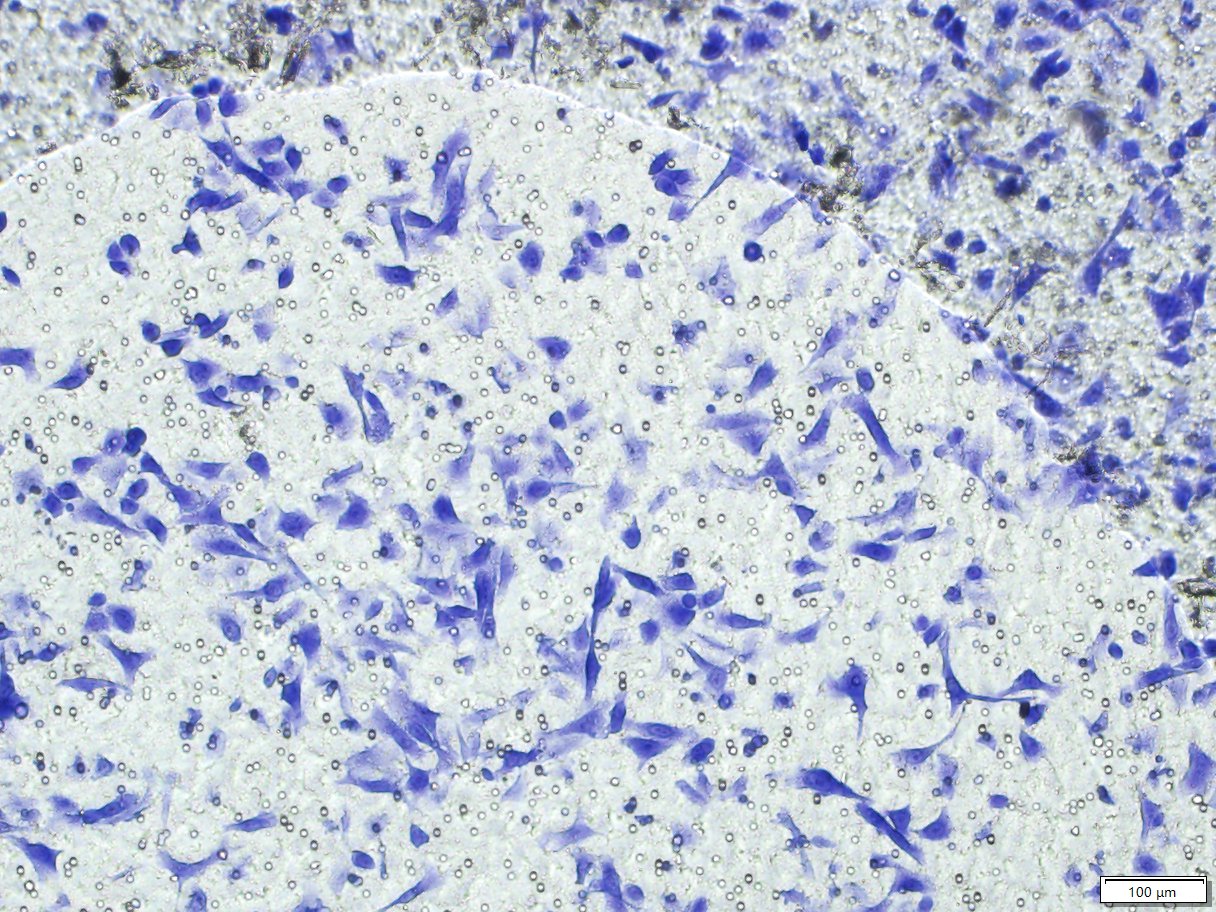

Supplement: Supplemental Information 3 [file peerj-cs-09-1651-s003.zip › Dataset 2/5+8.jpg]

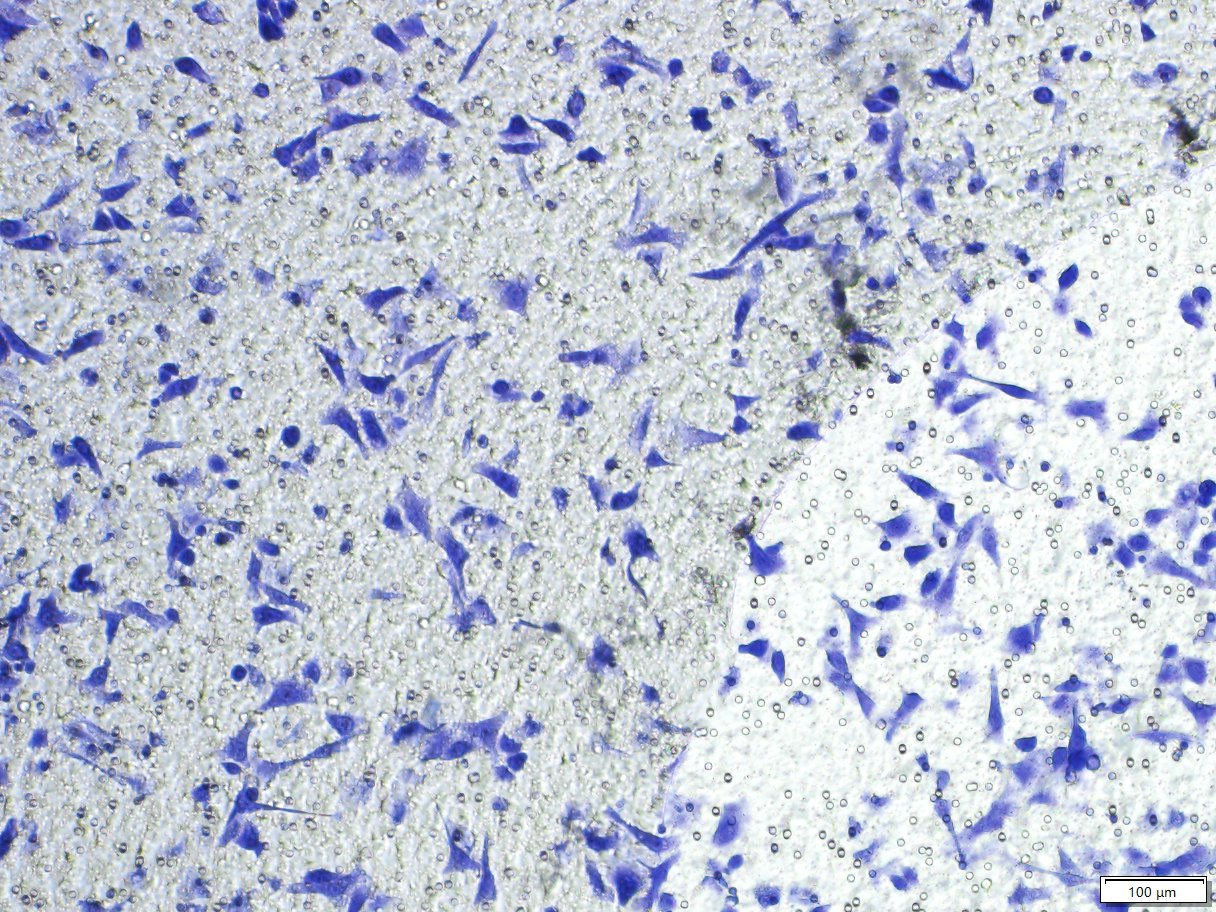

Supplement: Supplemental Information 3 [file peerj-cs-09-1651-s003.zip › Dataset 2/5+9.jpg]

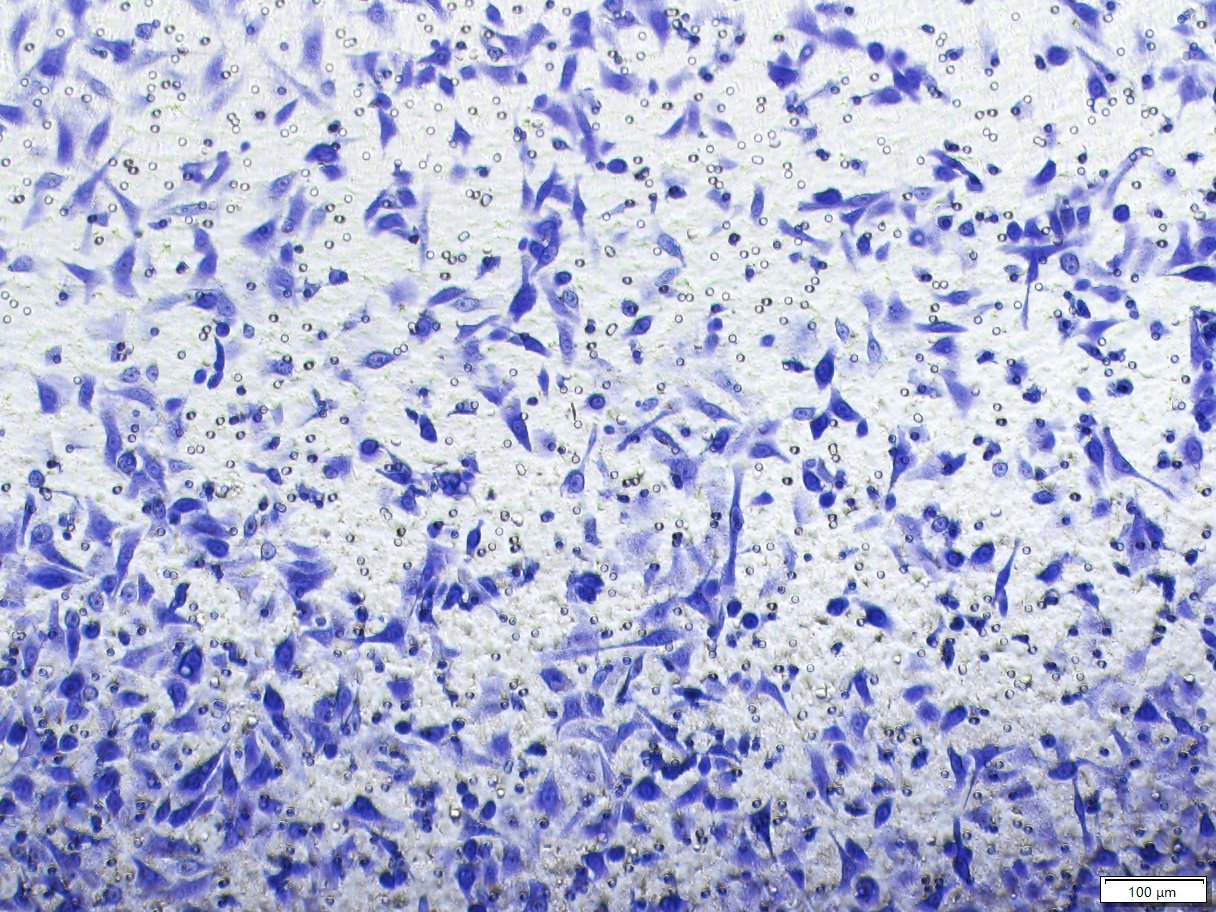

Supplement: Supplemental Information 3 [file peerj-cs-09-1651-s003.zip › Dataset 2/5-1.jpg]

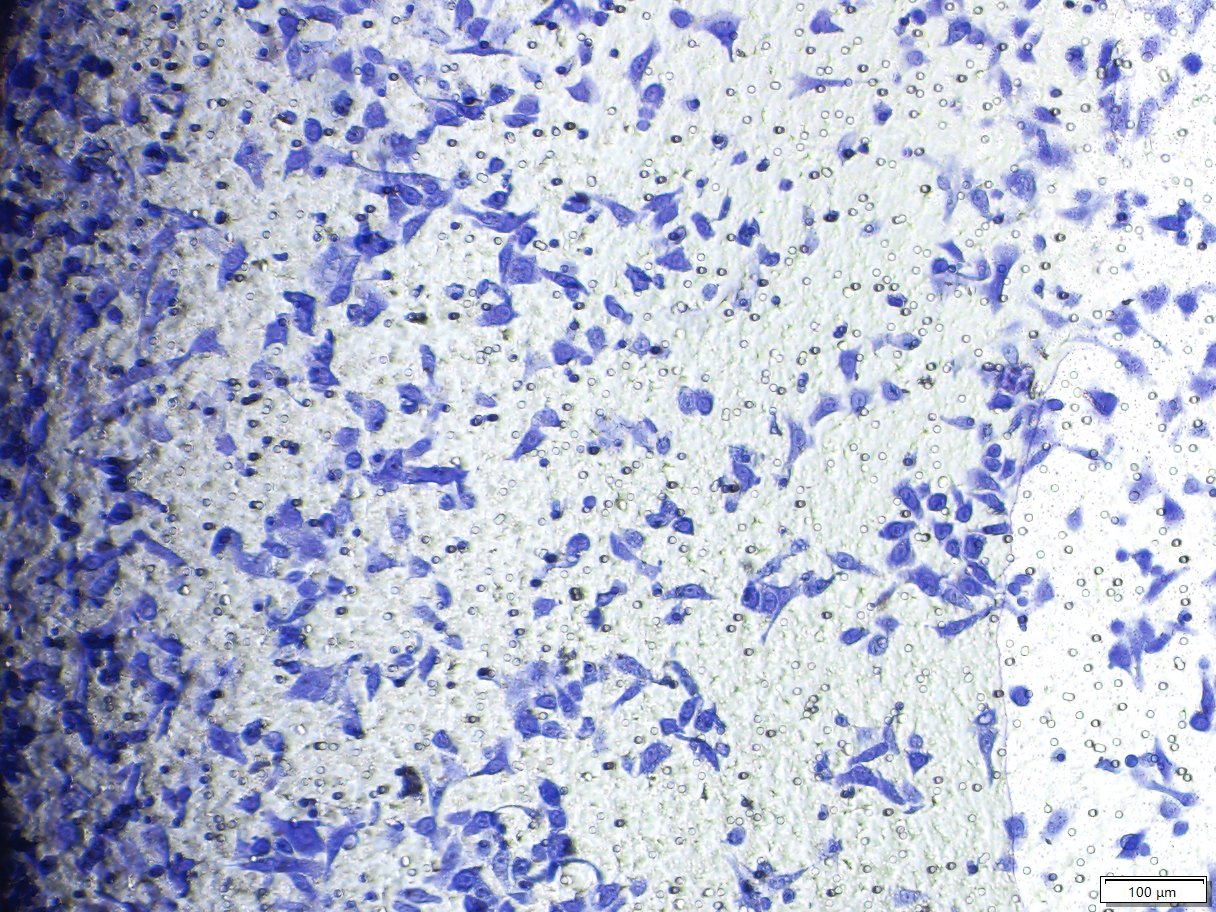

Supplement: Supplemental Information 3 [file peerj-cs-09-1651-s003.zip › Dataset 2/5-10.jpg]

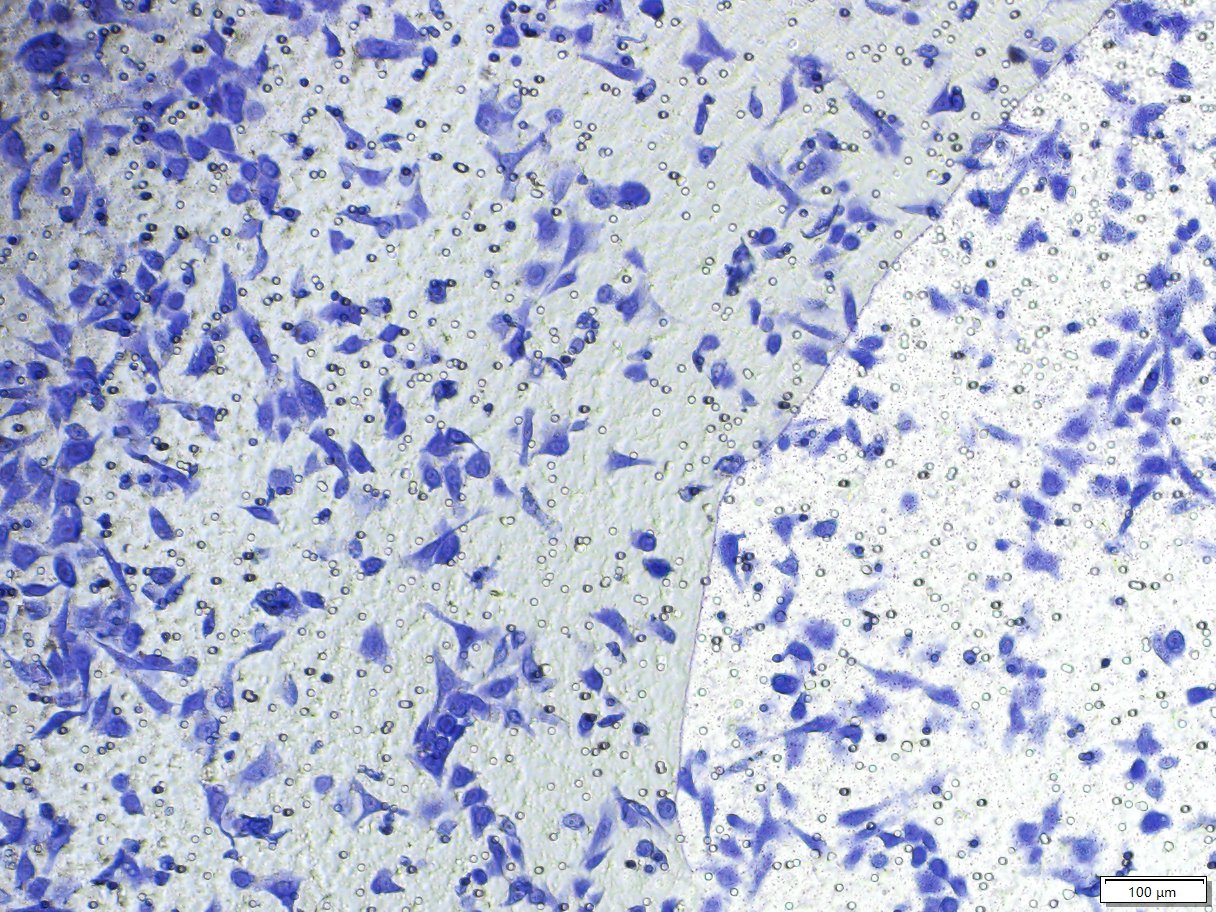

Supplement: Supplemental Information 3 [file peerj-cs-09-1651-s003.zip › Dataset 2/5-11.jpg]

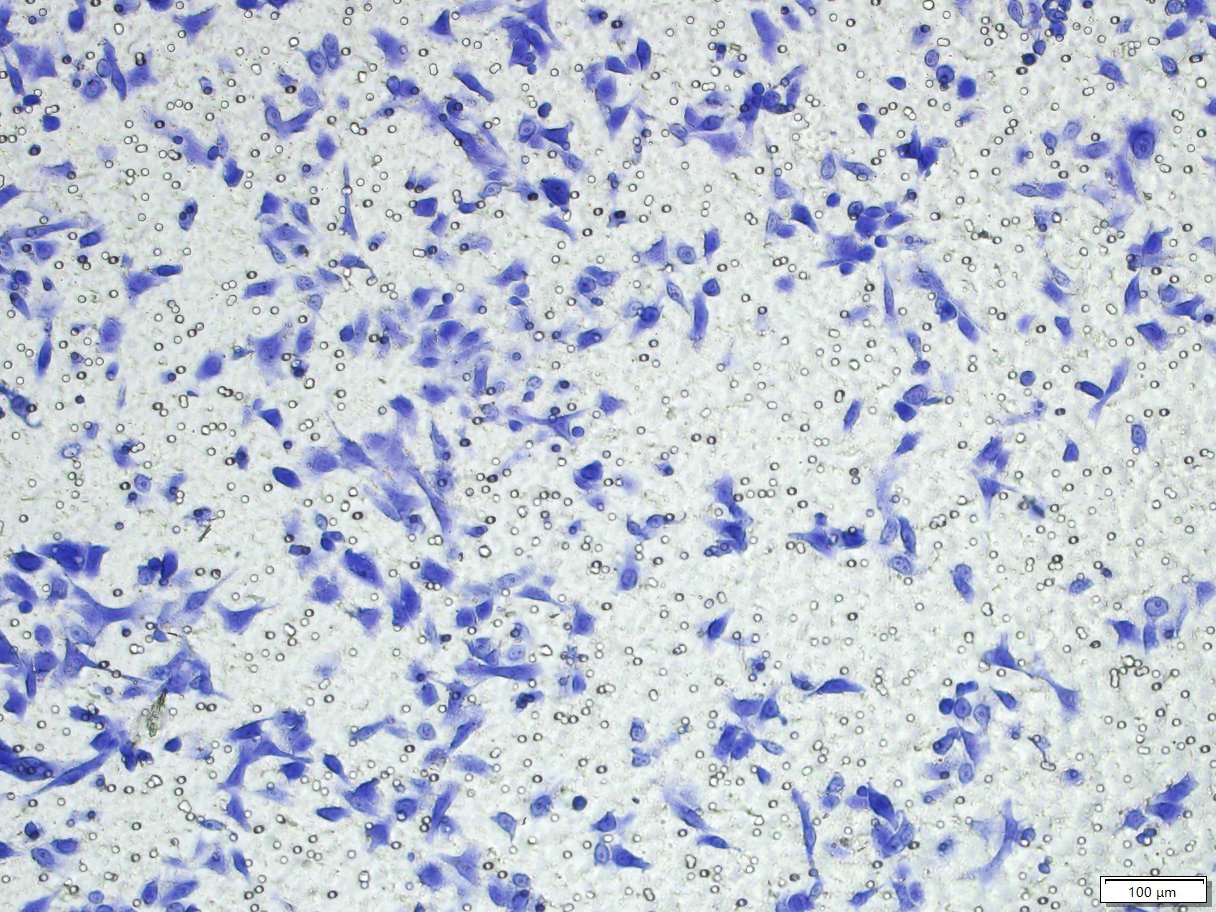

Supplement: Supplemental Information 3 [file peerj-cs-09-1651-s003.zip › Dataset 2/5-12.jpg]

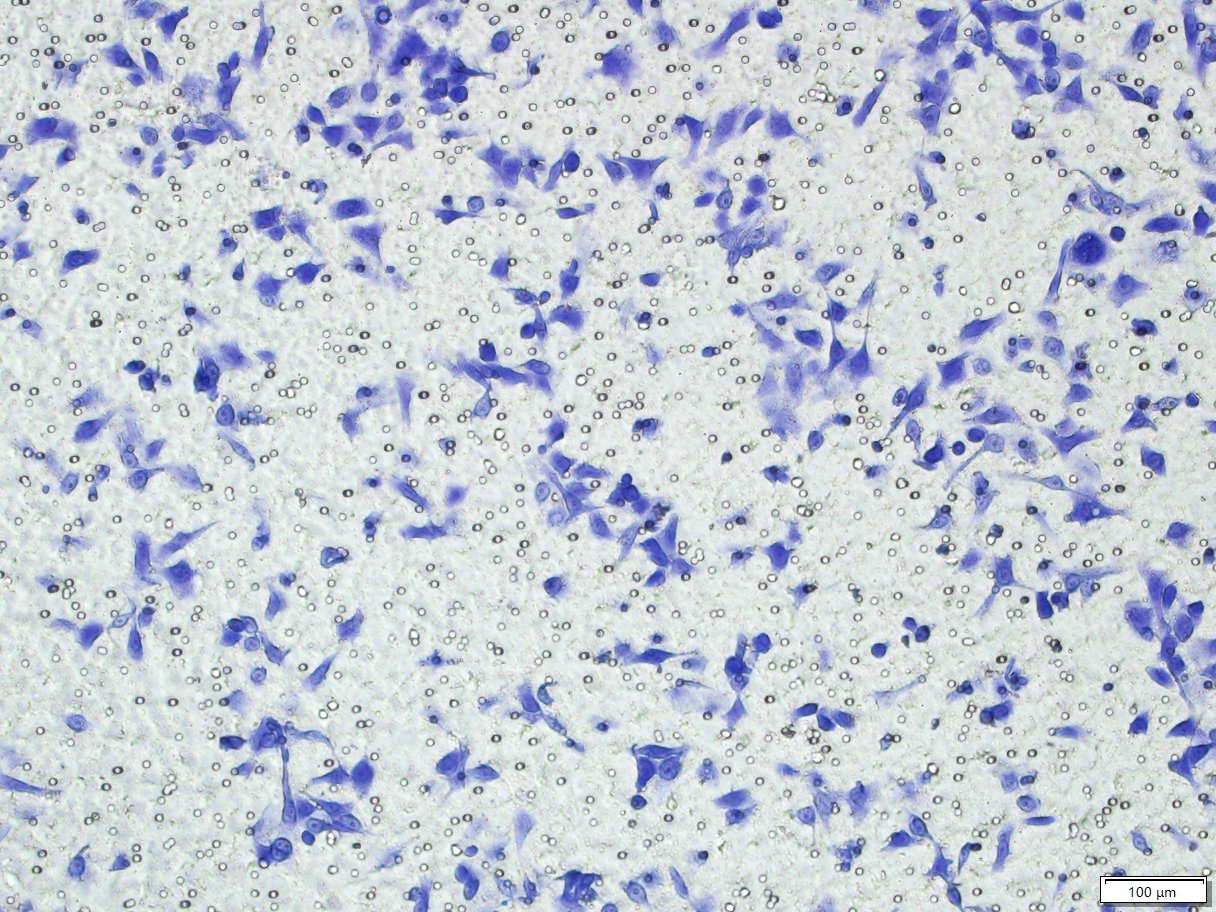

Supplement: Supplemental Information 3 [file peerj-cs-09-1651-s003.zip › Dataset 2/5-13.jpg]

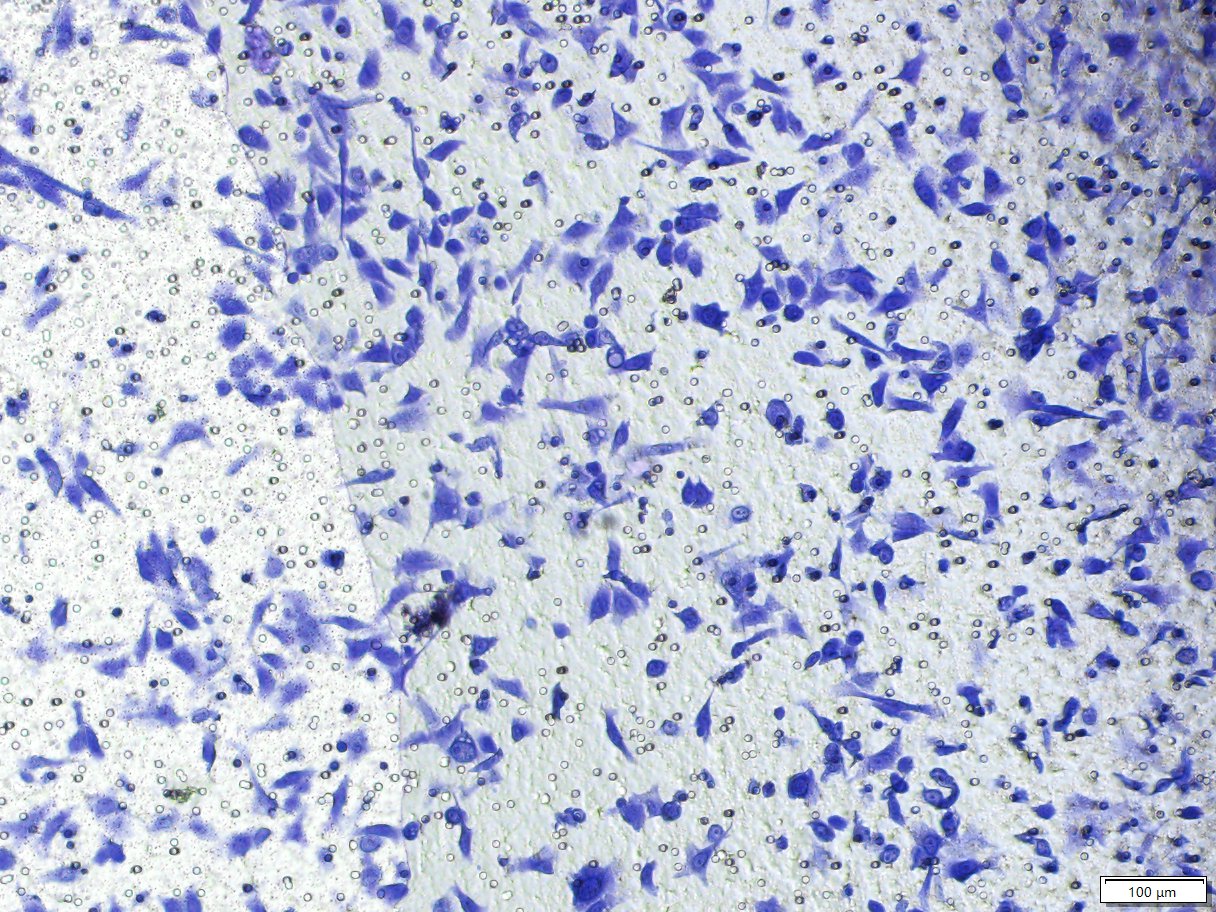

Supplement: Supplemental Information 3 [file peerj-cs-09-1651-s003.zip › Dataset 2/5-14.jpg]

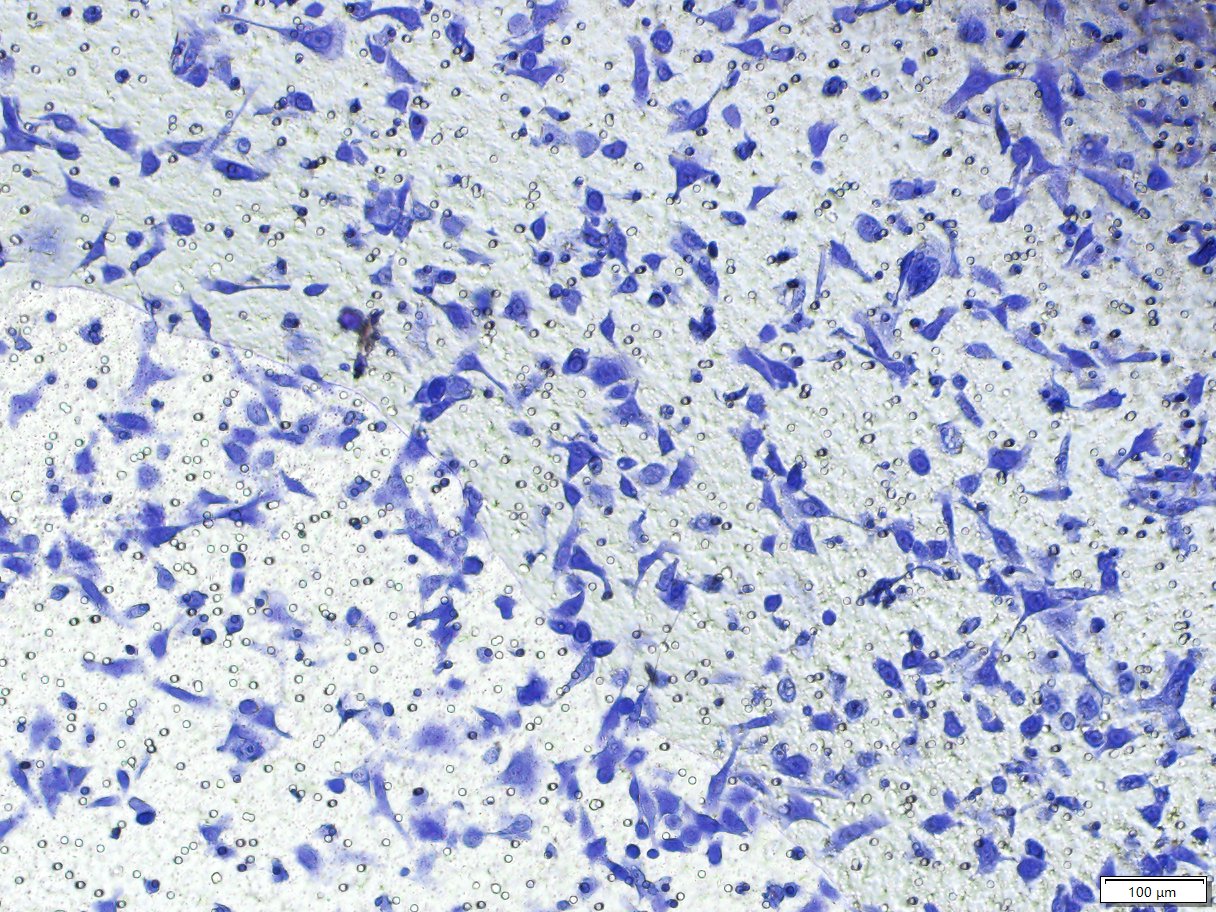

Supplement: Supplemental Information 3 [file peerj-cs-09-1651-s003.zip › Dataset 2/5-15.jpg]

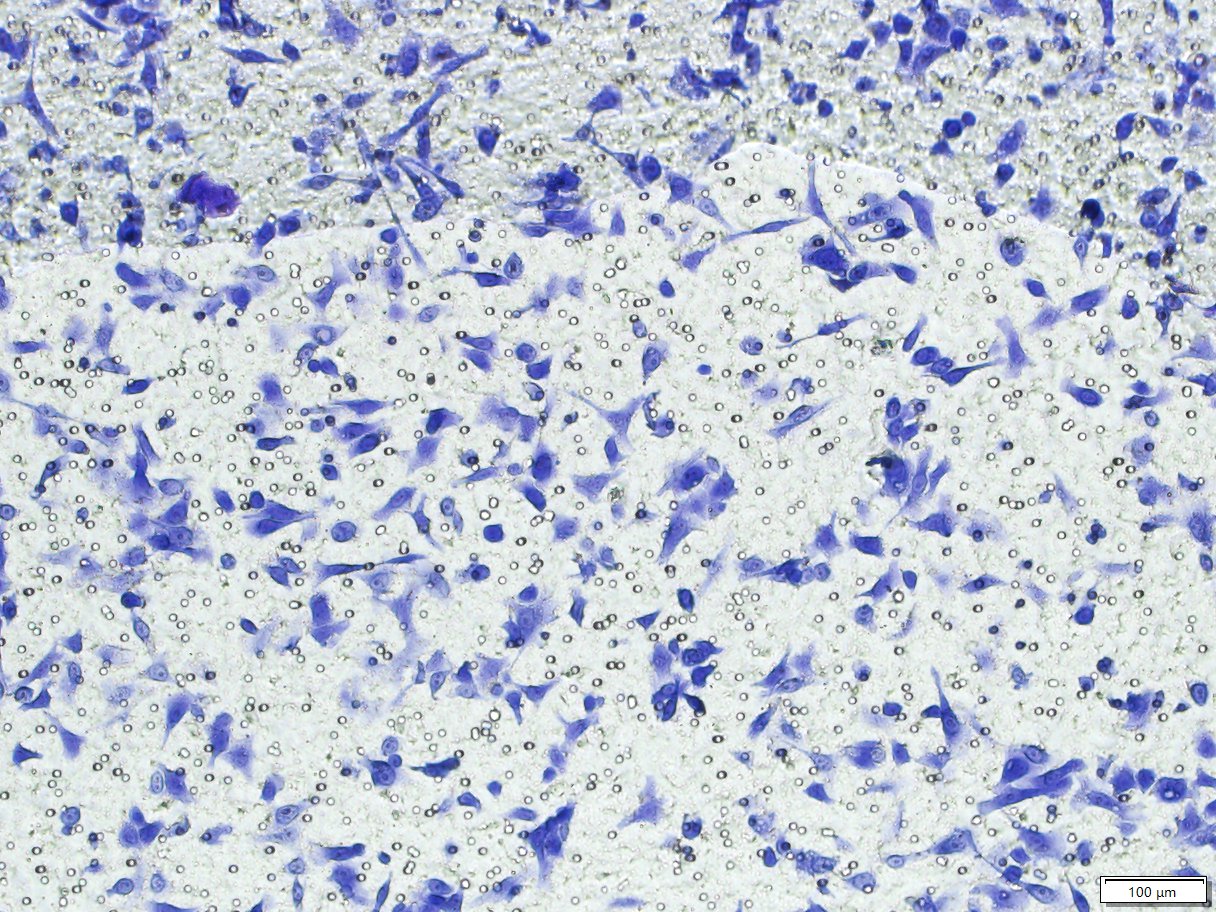

Supplement: Supplemental Information 3 [file peerj-cs-09-1651-s003.zip › Dataset 2/5-16.jpg]

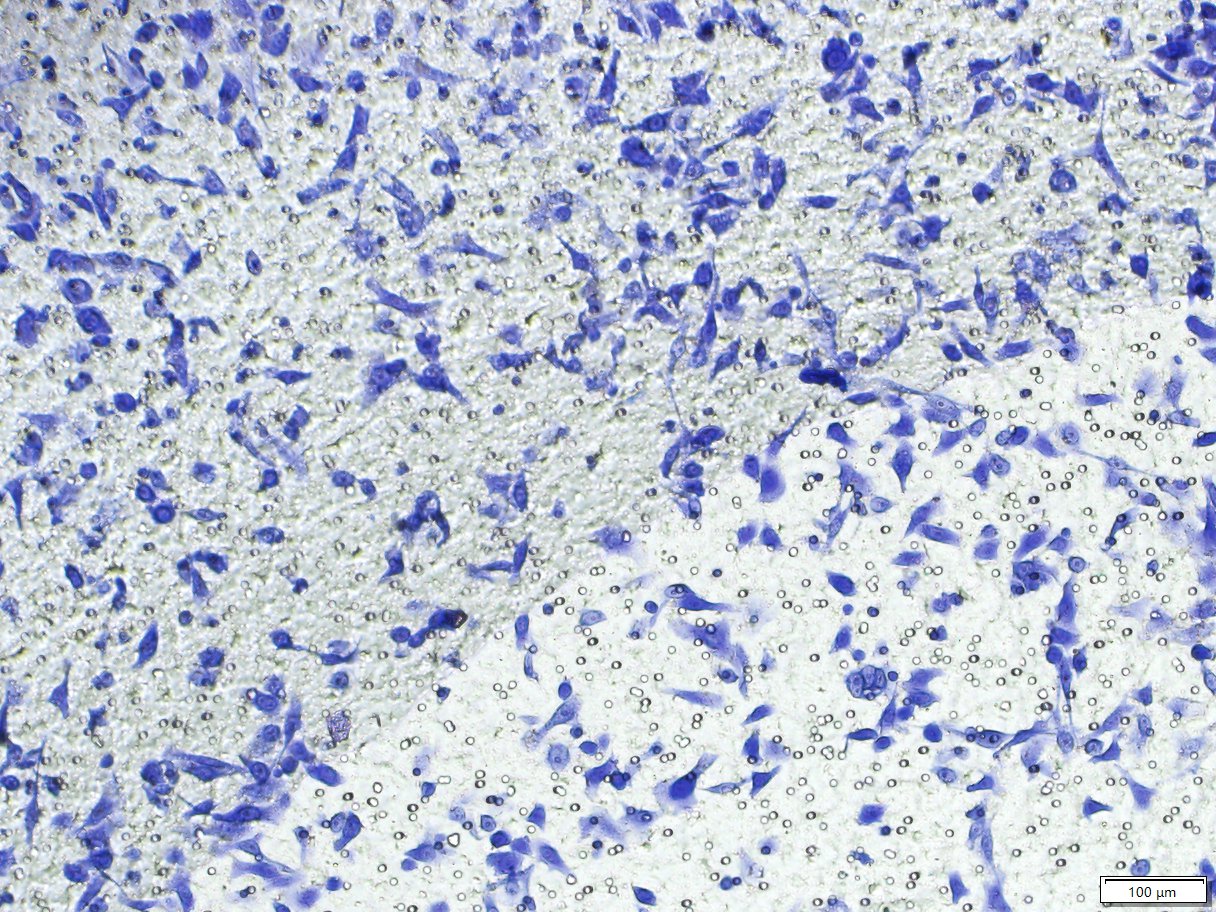

Supplement: Supplemental Information 3 [file peerj-cs-09-1651-s003.zip › Dataset 2/5-17.jpg]

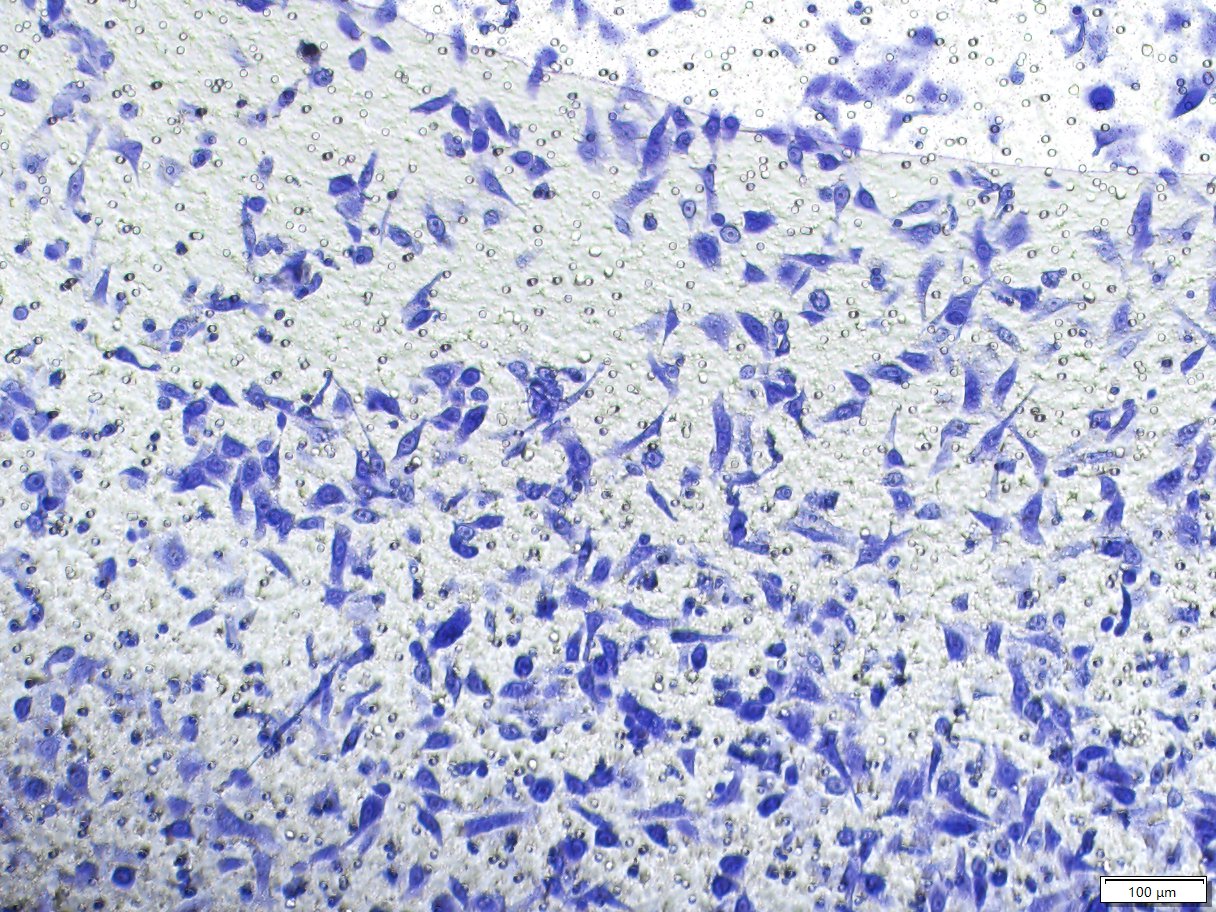

Supplement: Supplemental Information 3 [file peerj-cs-09-1651-s003.zip › Dataset 2/5-2.jpg]

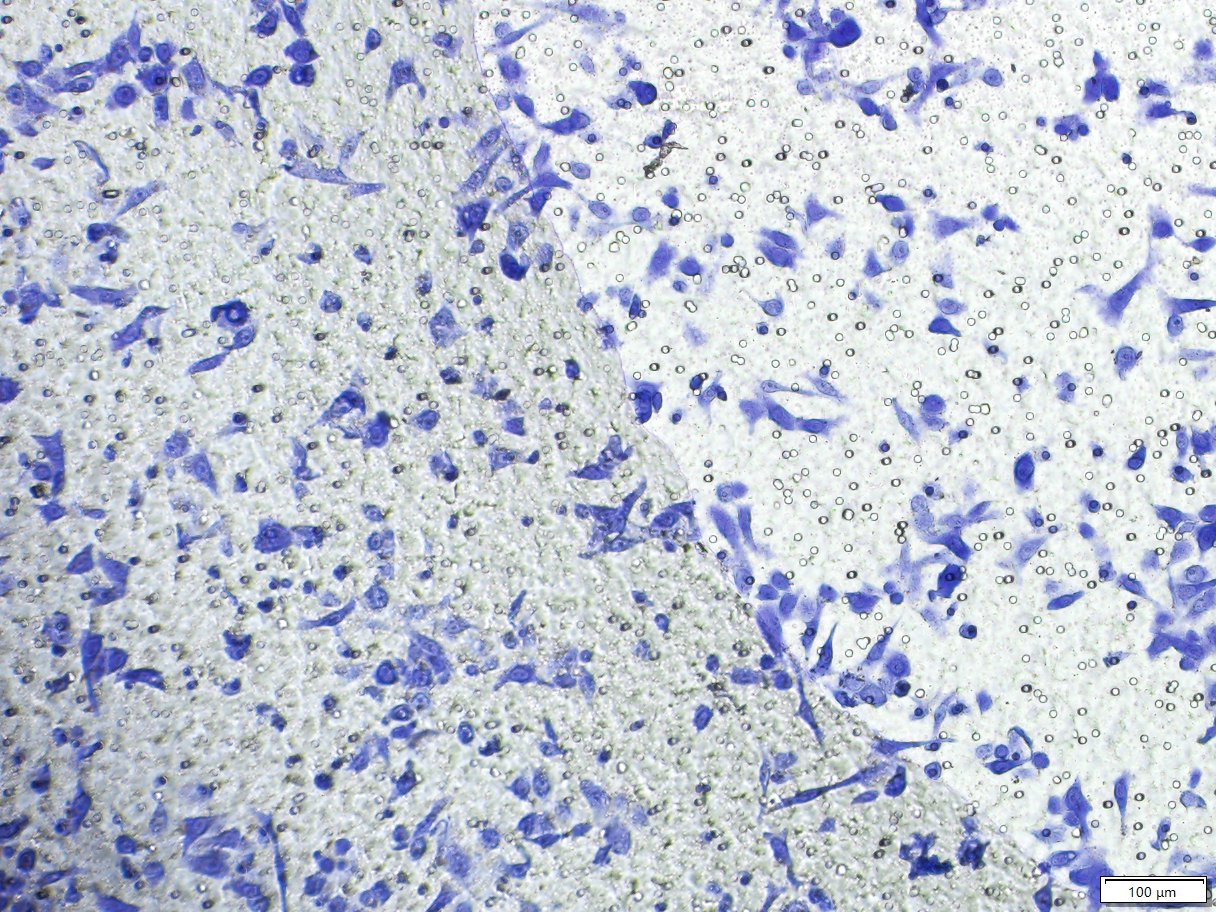

Supplement: Supplemental Information 3 [file peerj-cs-09-1651-s003.zip › Dataset 2/5-3.jpg]

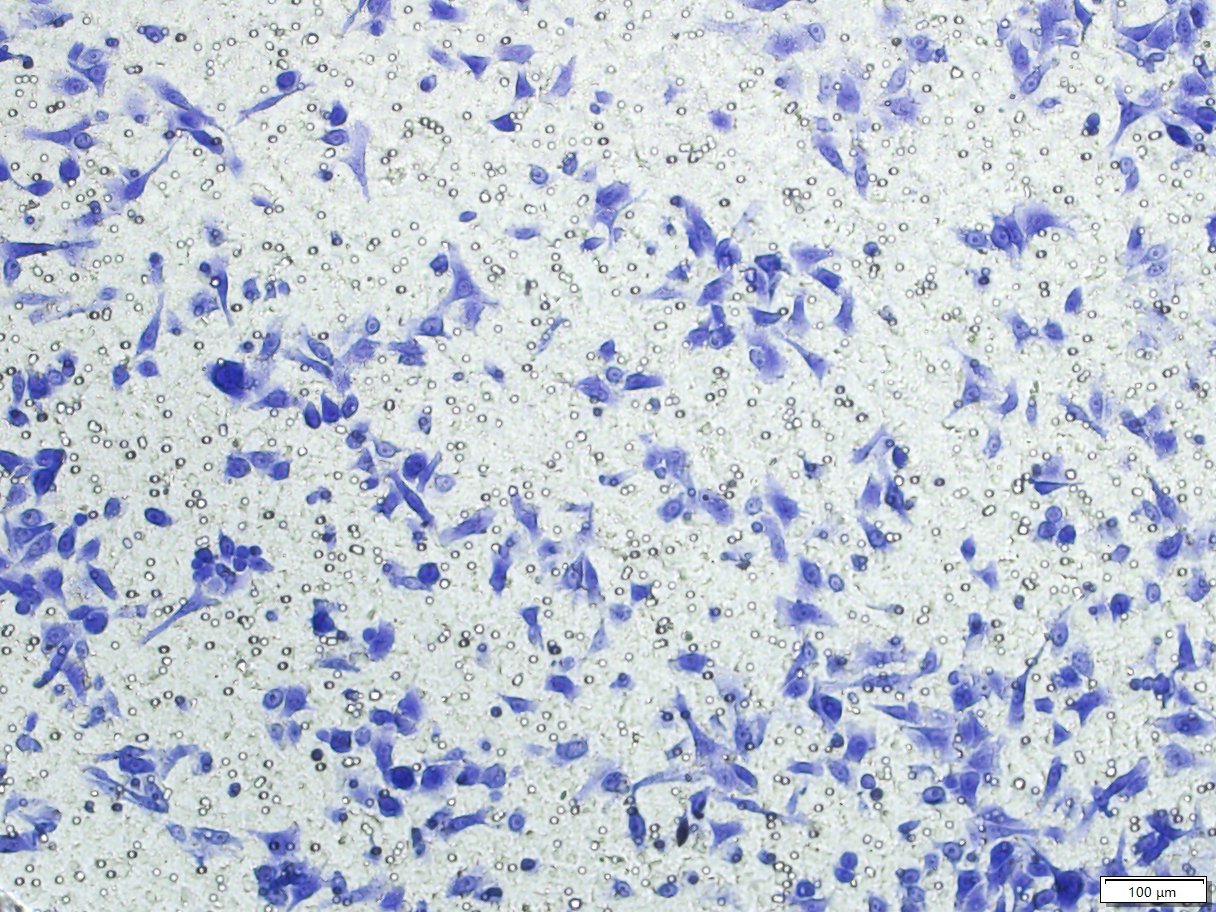

Supplement: Supplemental Information 3 [file peerj-cs-09-1651-s003.zip › Dataset 2/5-4.jpg]

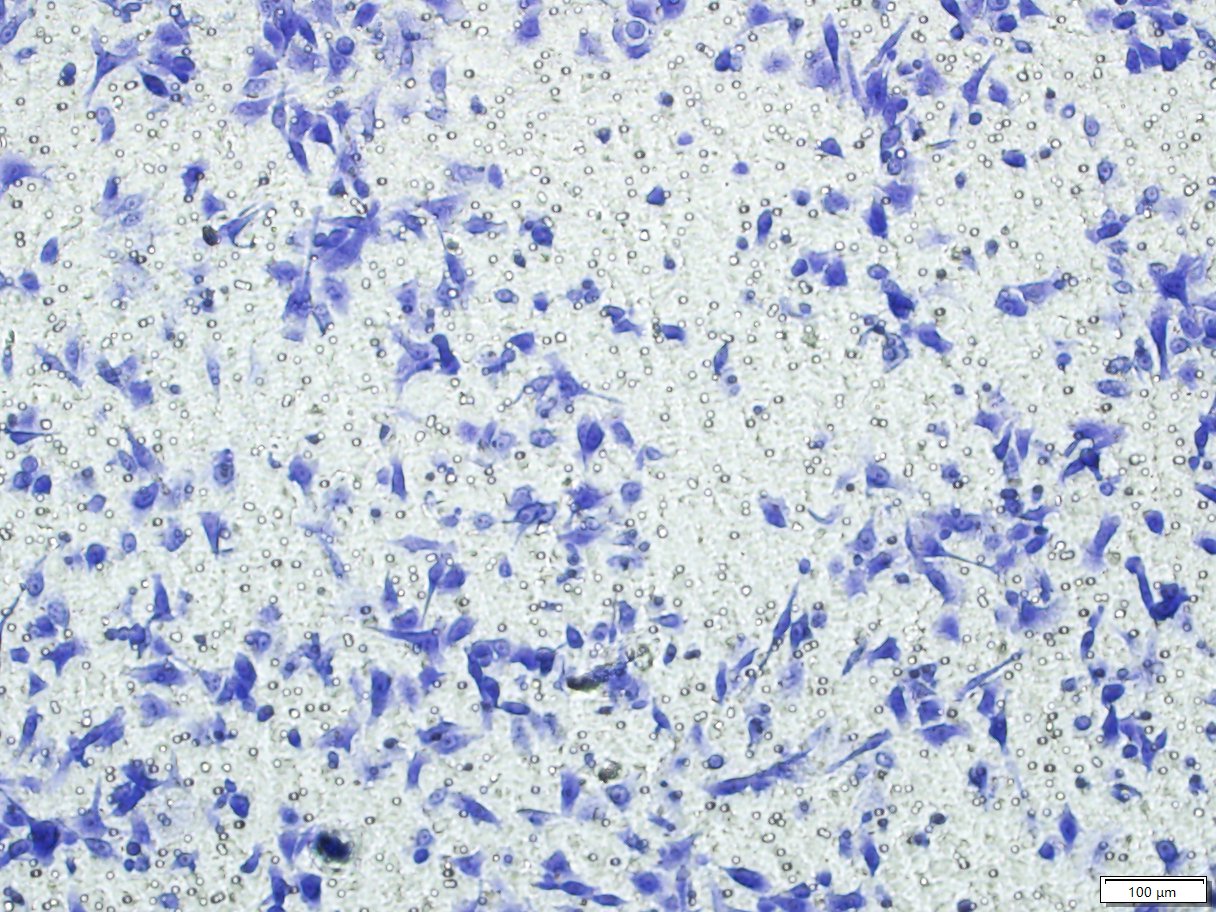

Supplement: Supplemental Information 3 [file peerj-cs-09-1651-s003.zip › Dataset 2/5-5.jpg]

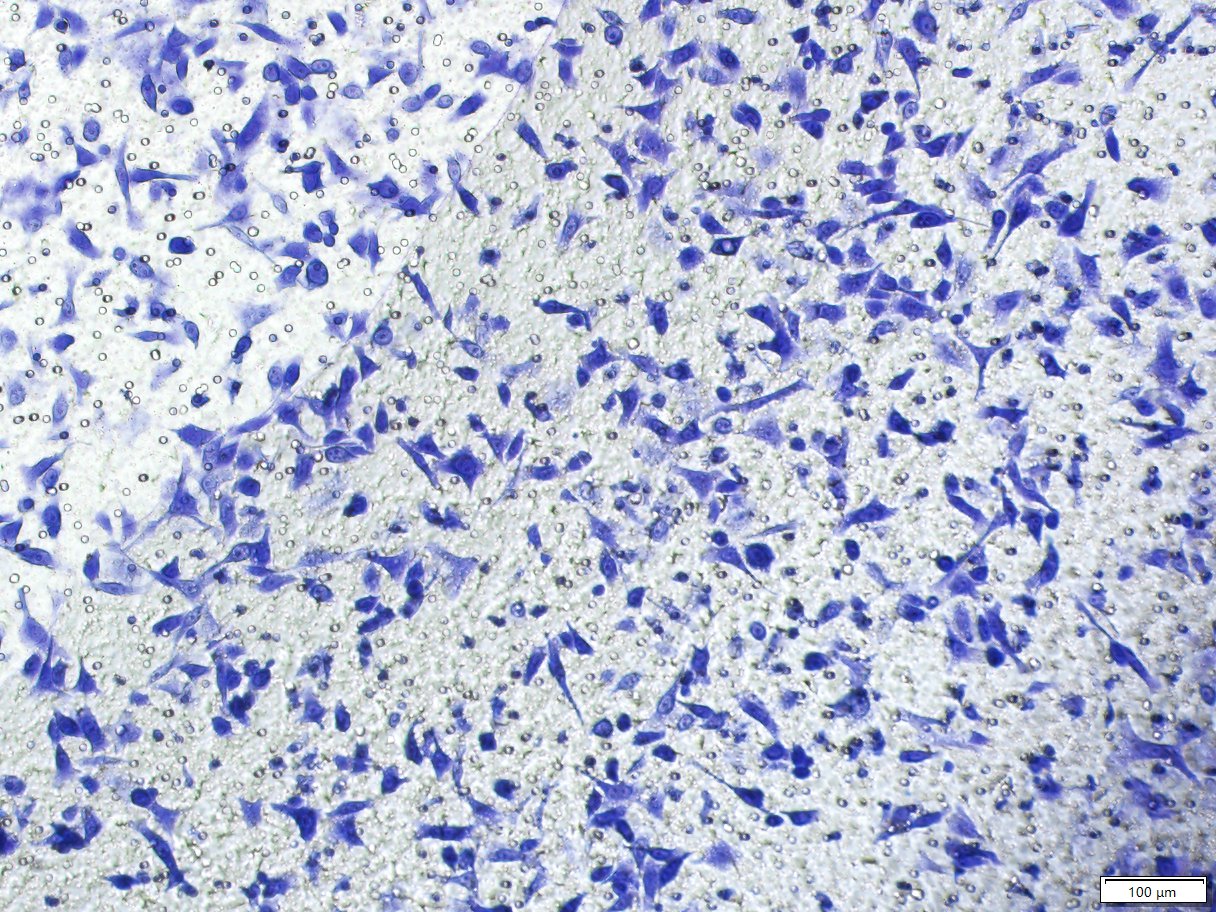

Supplement: Supplemental Information 3 [file peerj-cs-09-1651-s003.zip › Dataset 2/5-6.jpg]

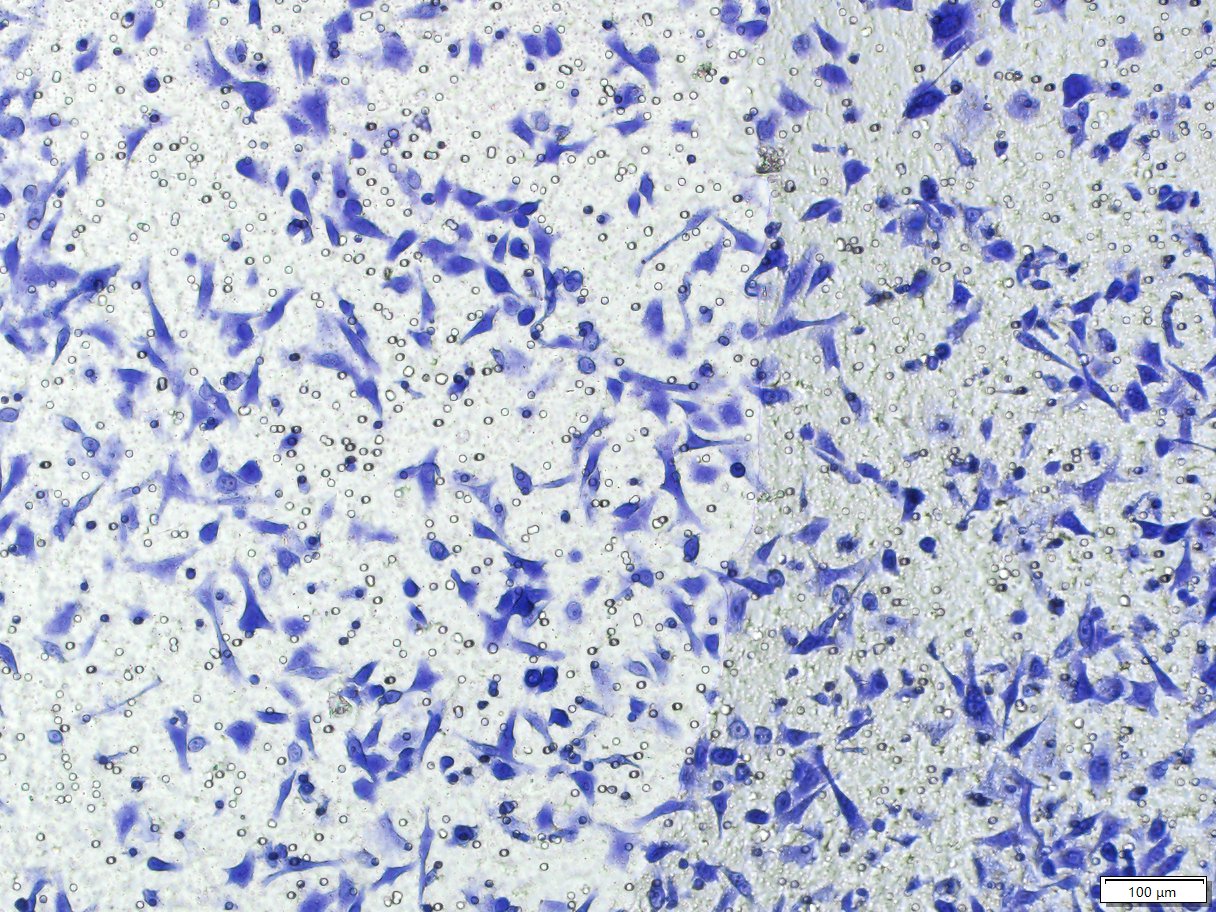

Supplement: Supplemental Information 3 [file peerj-cs-09-1651-s003.zip › Dataset 2/5-7.jpg]

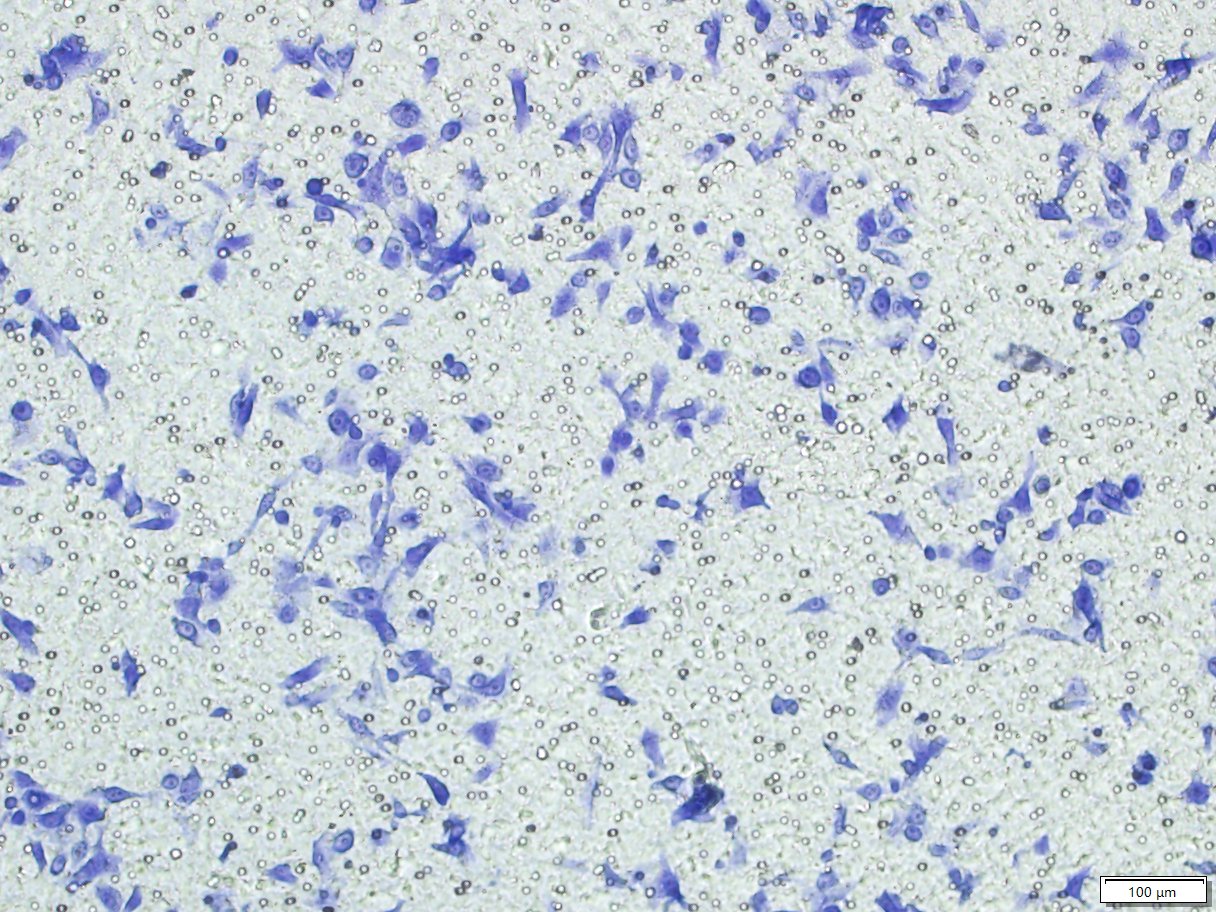

Supplement: Supplemental Information 3 [file peerj-cs-09-1651-s003.zip › Dataset 2/5-8.jpg]

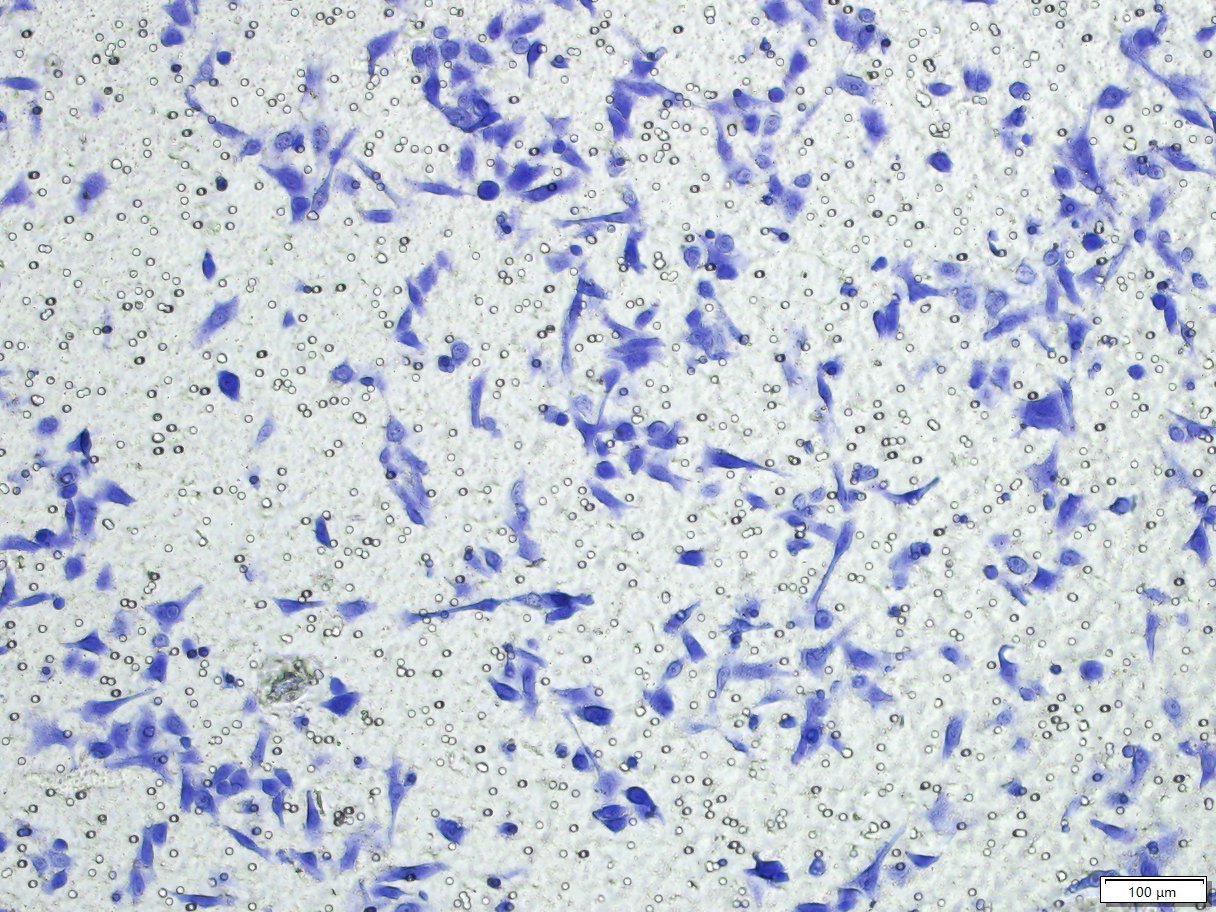

Supplement: Supplemental Information 3 [file peerj-cs-09-1651-s003.zip › Dataset 2/5-9.jpg]

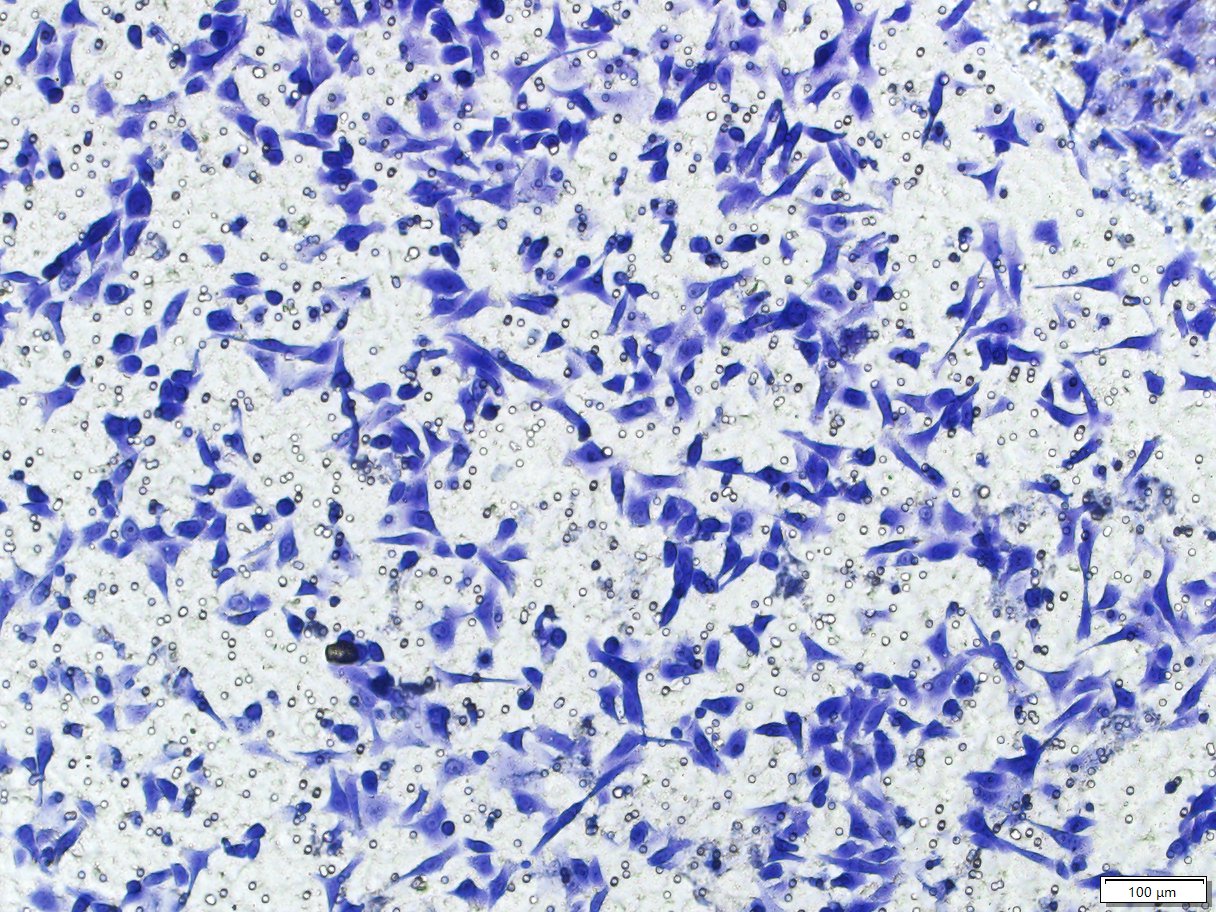

Supplement: Supplemental Information 4 [file peerj-cs-09-1651-s004.zip › Dataset 3/2-15.jpg]

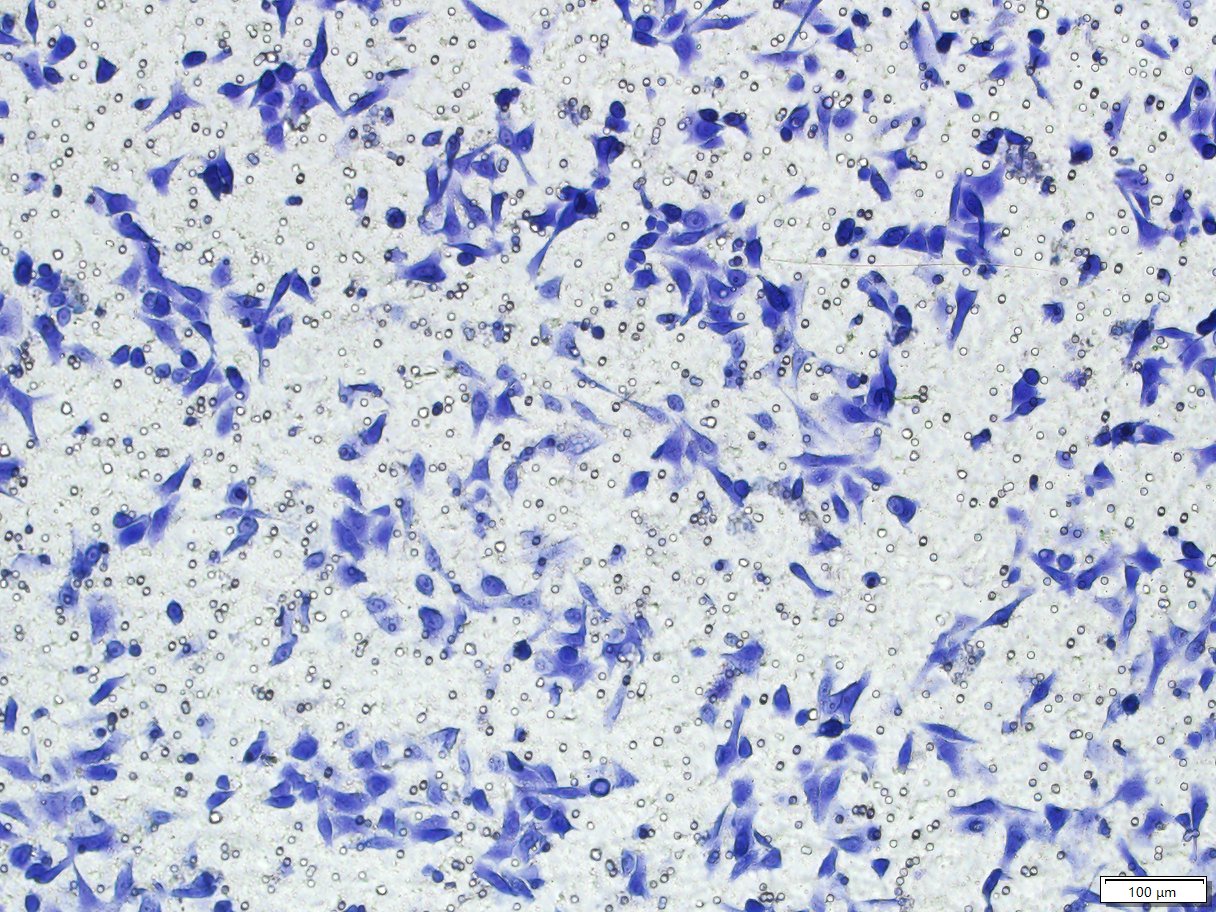

Supplement: Supplemental Information 4 [file peerj-cs-09-1651-s004.zip › Dataset 3/2-16.jpg]

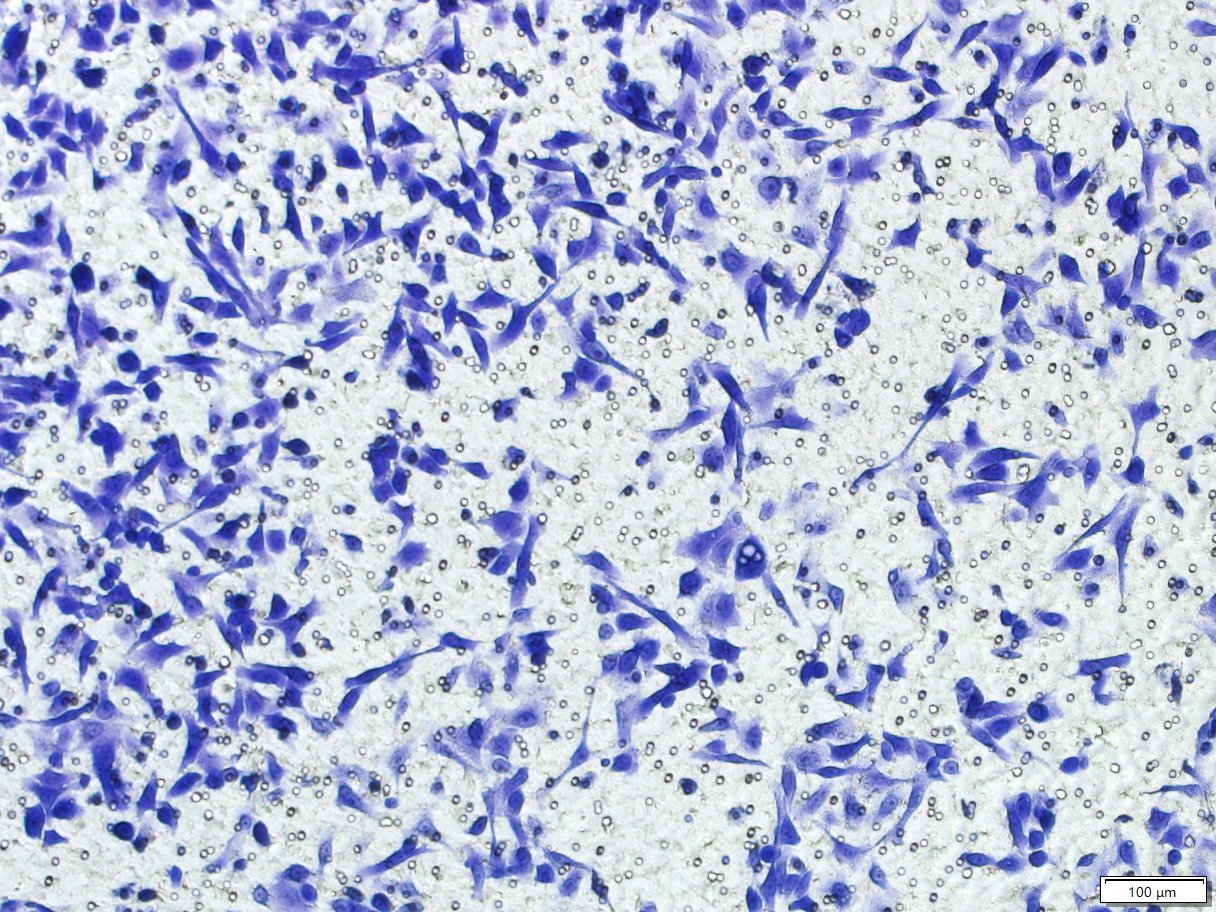

Supplement: Supplemental Information 4 [file peerj-cs-09-1651-s004.zip › Dataset 3/2-17.jpg]

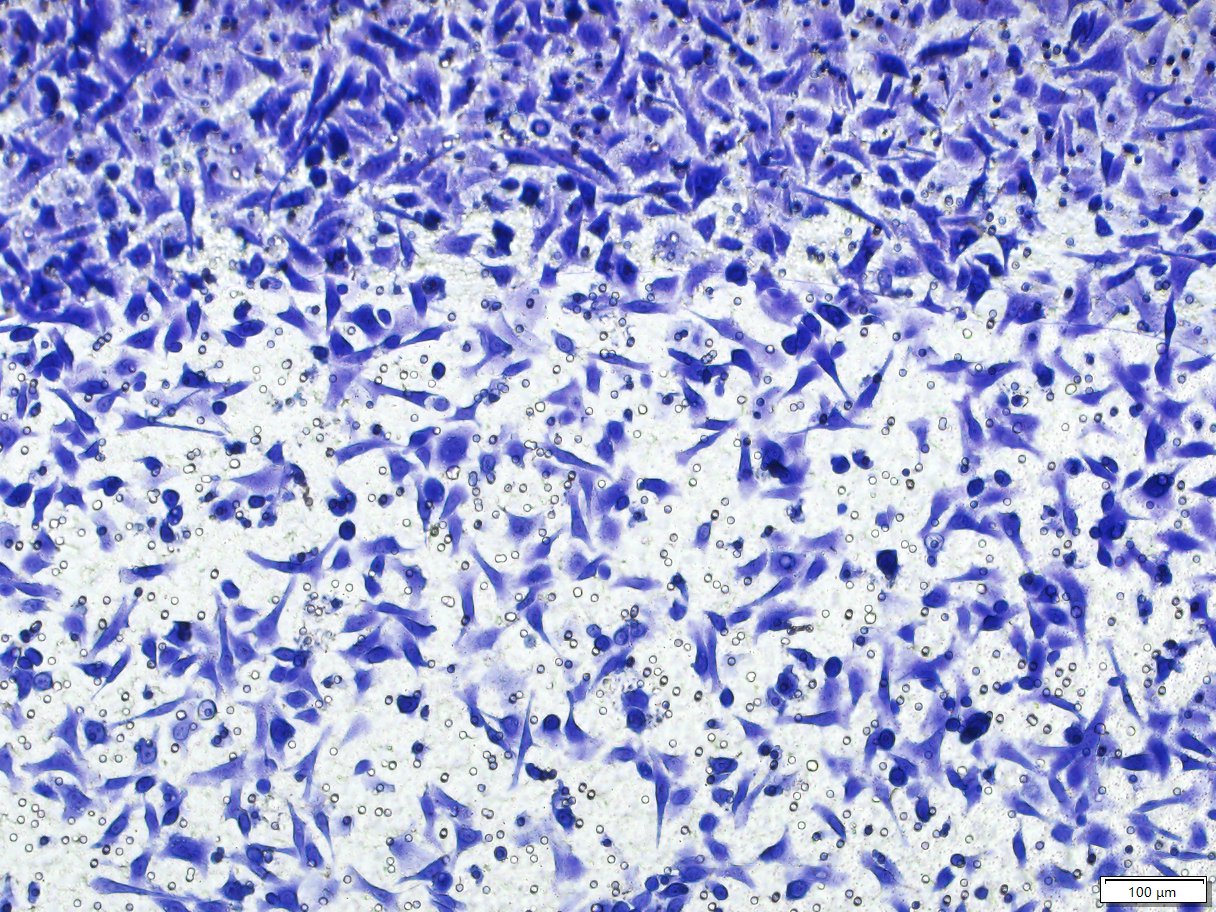

Supplement: Supplemental Information 4 [file peerj-cs-09-1651-s004.zip › Dataset 3/2-18.jpg]

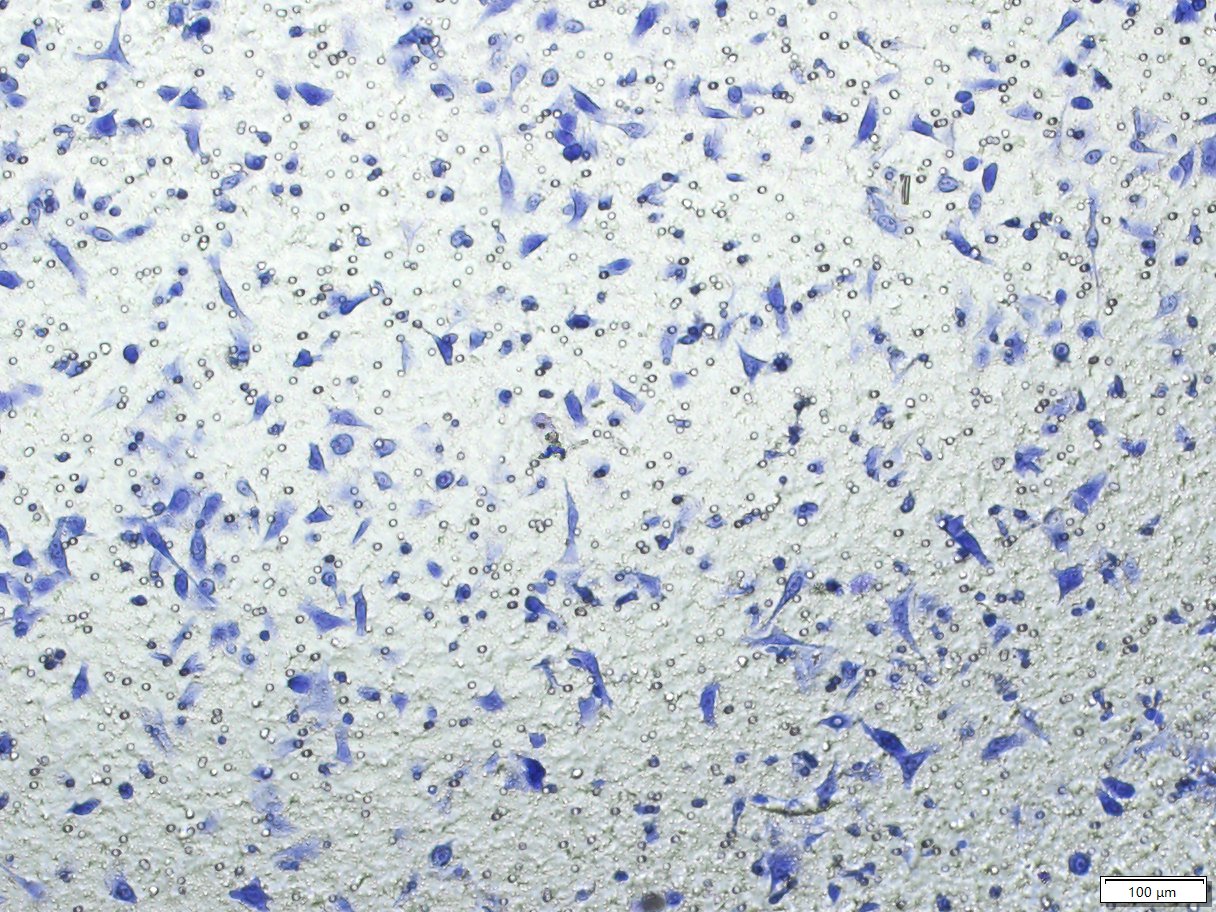

Supplement: Supplemental Information 4 [file peerj-cs-09-1651-s004.zip › Dataset 3/3+1.jpg]

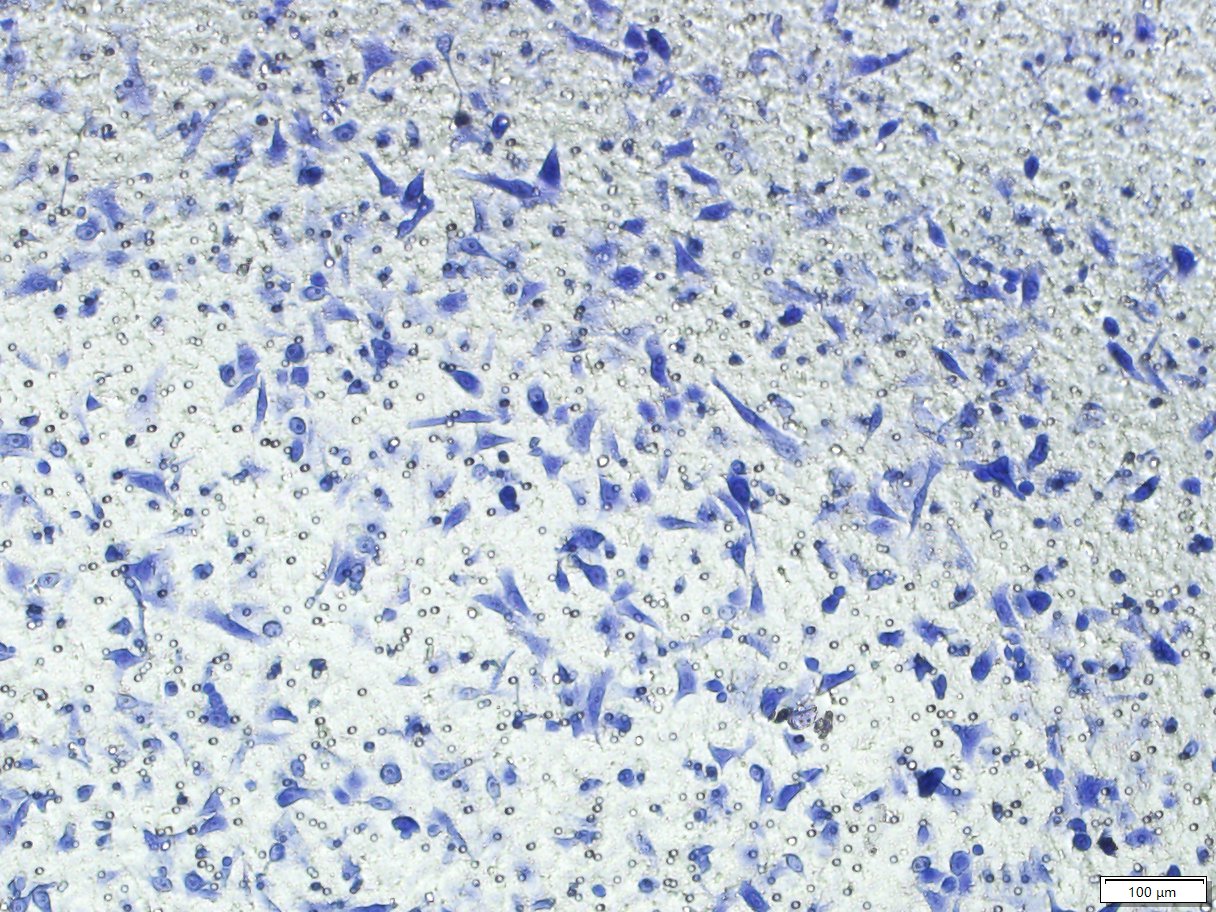

Supplement: Supplemental Information 4 [file peerj-cs-09-1651-s004.zip › Dataset 3/3+10.jpg]

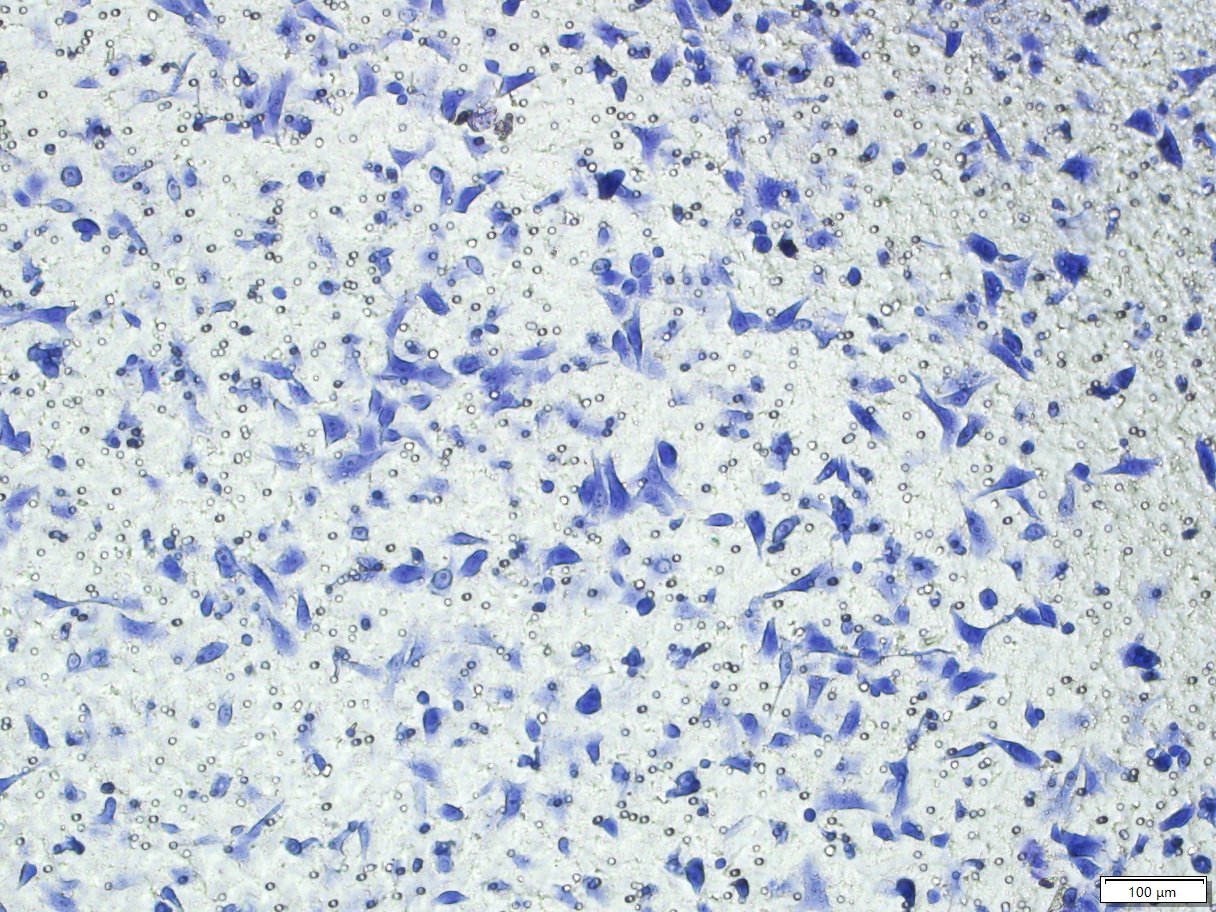

Supplement: Supplemental Information 4 [file peerj-cs-09-1651-s004.zip › Dataset 3/3+11.jpg]

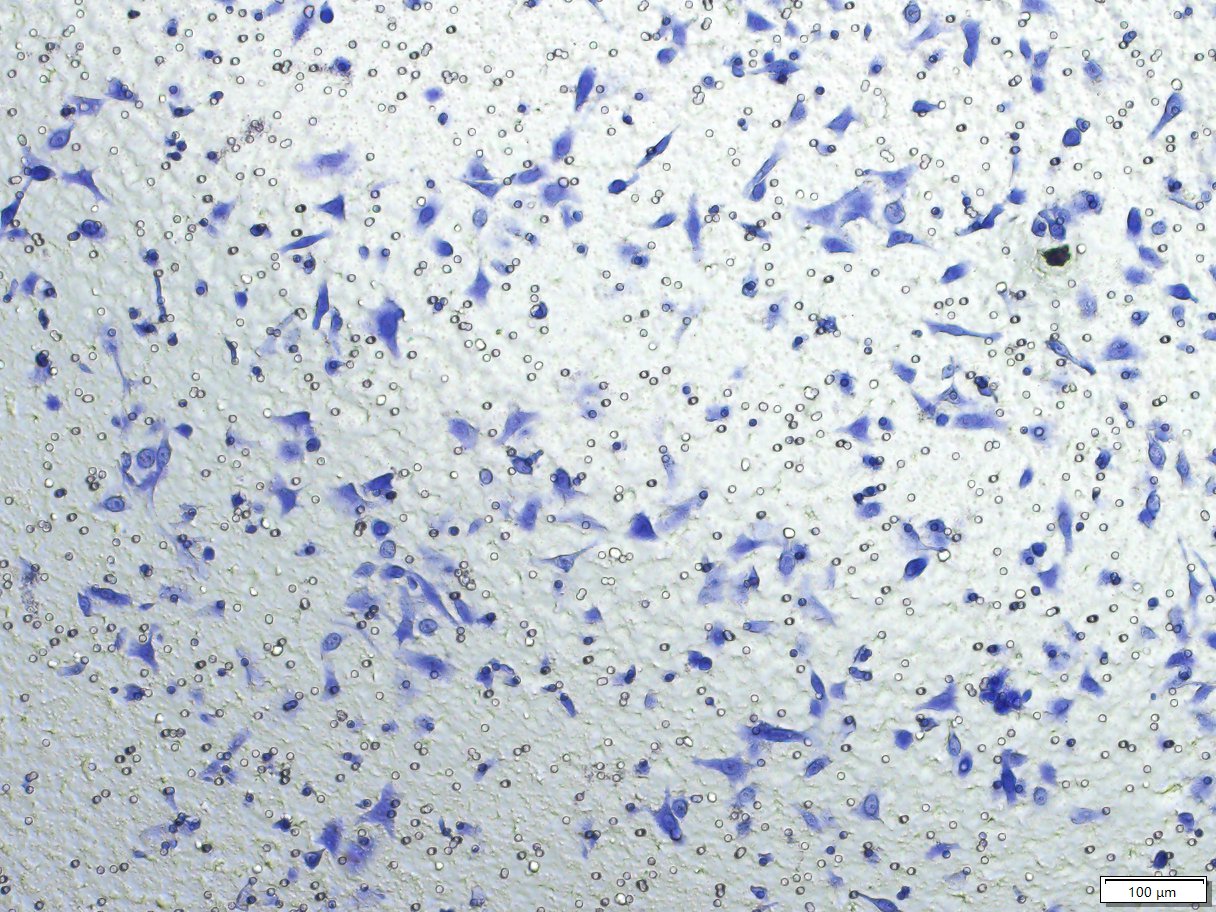

Supplement: Supplemental Information 4 [file peerj-cs-09-1651-s004.zip › Dataset 3/3+2.jpg]

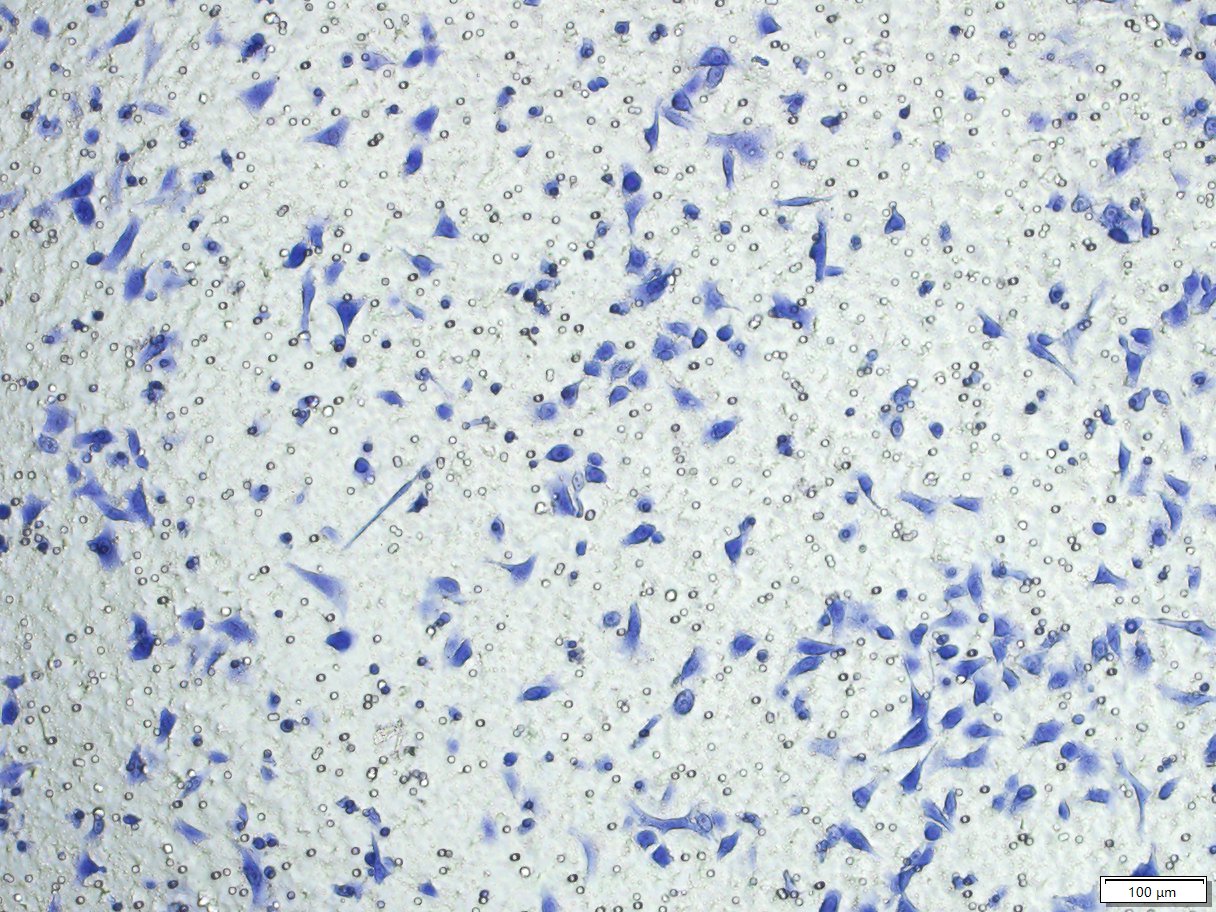

Supplement: Supplemental Information 4 [file peerj-cs-09-1651-s004.zip › Dataset 3/3+3.jpg]

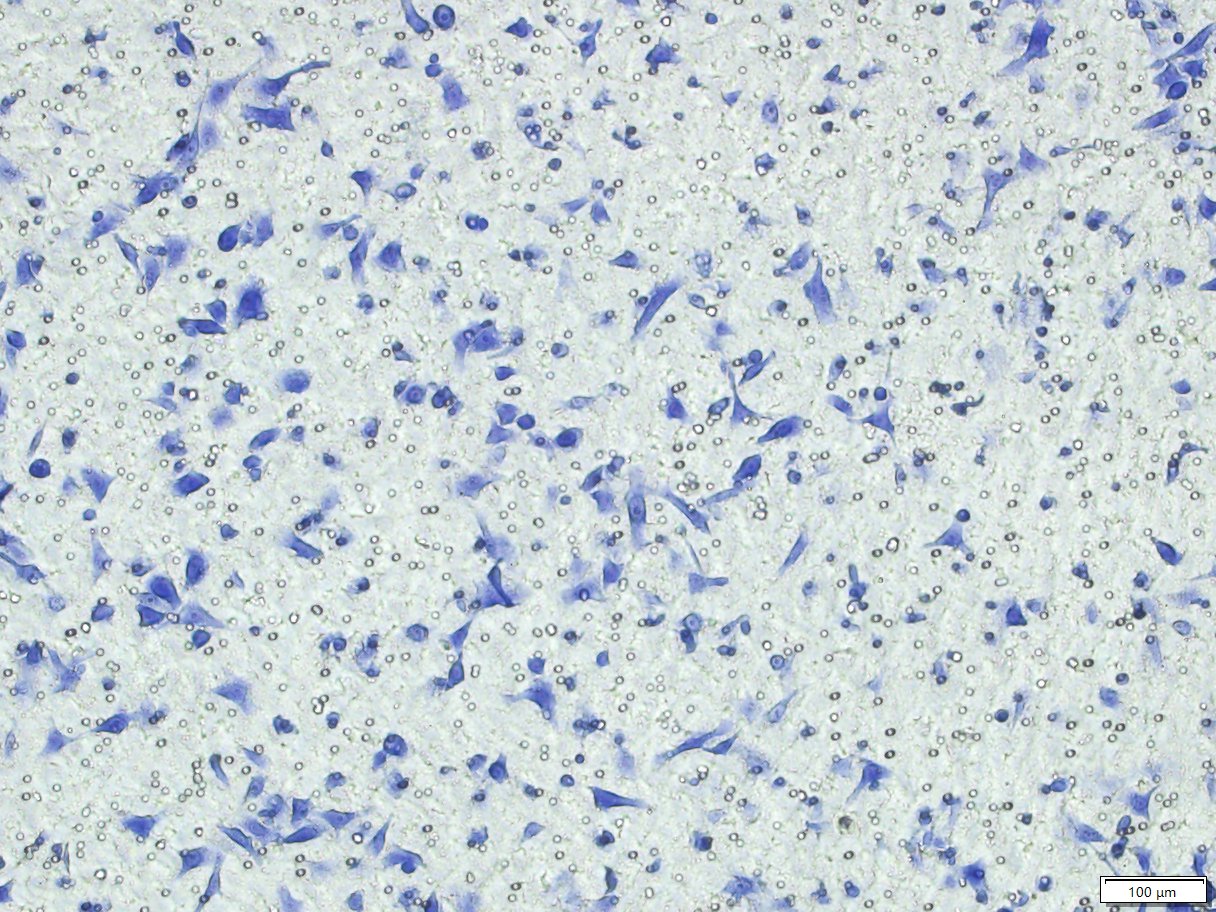

Supplement: Supplemental Information 4 [file peerj-cs-09-1651-s004.zip › Dataset 3/3+4.jpg]

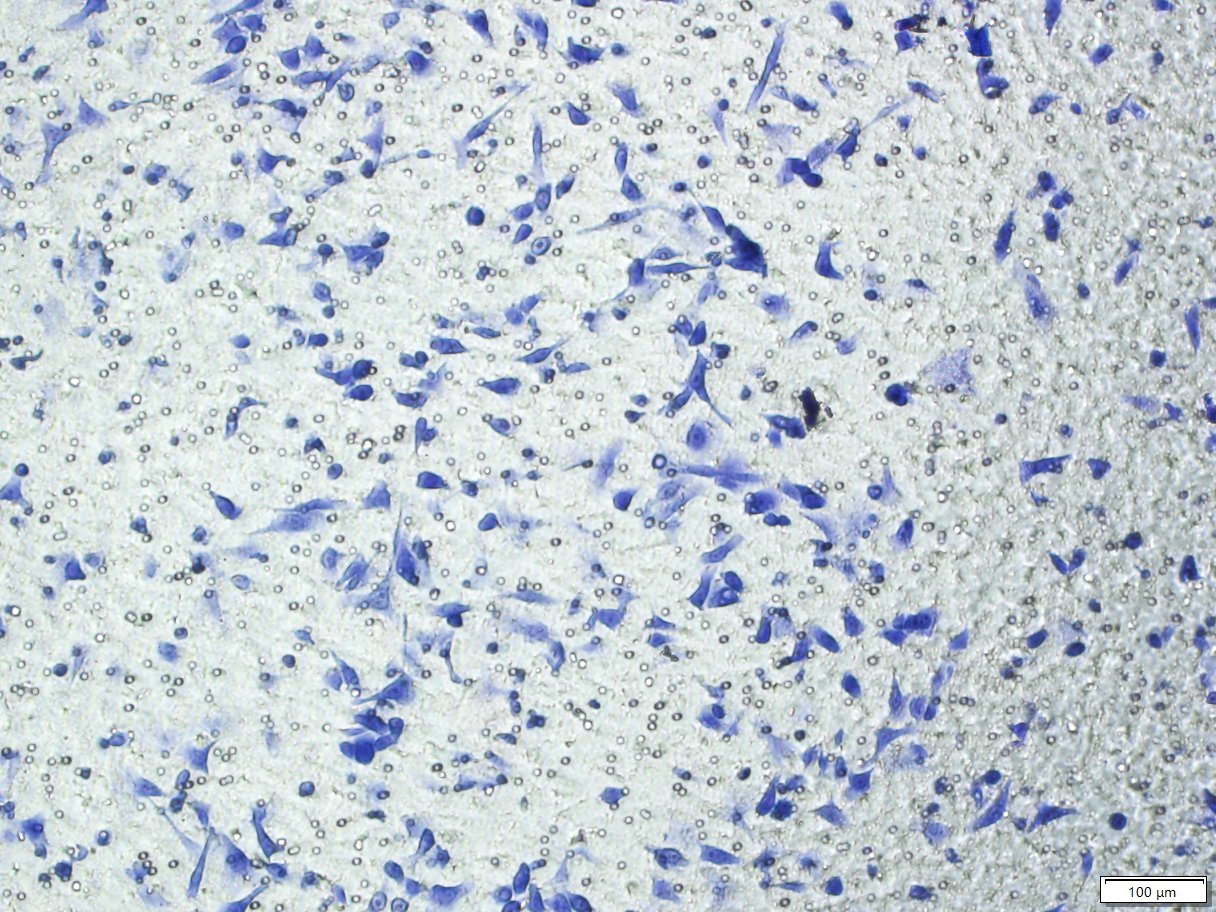

Supplement: Supplemental Information 4 [file peerj-cs-09-1651-s004.zip › Dataset 3/3+5.jpg]

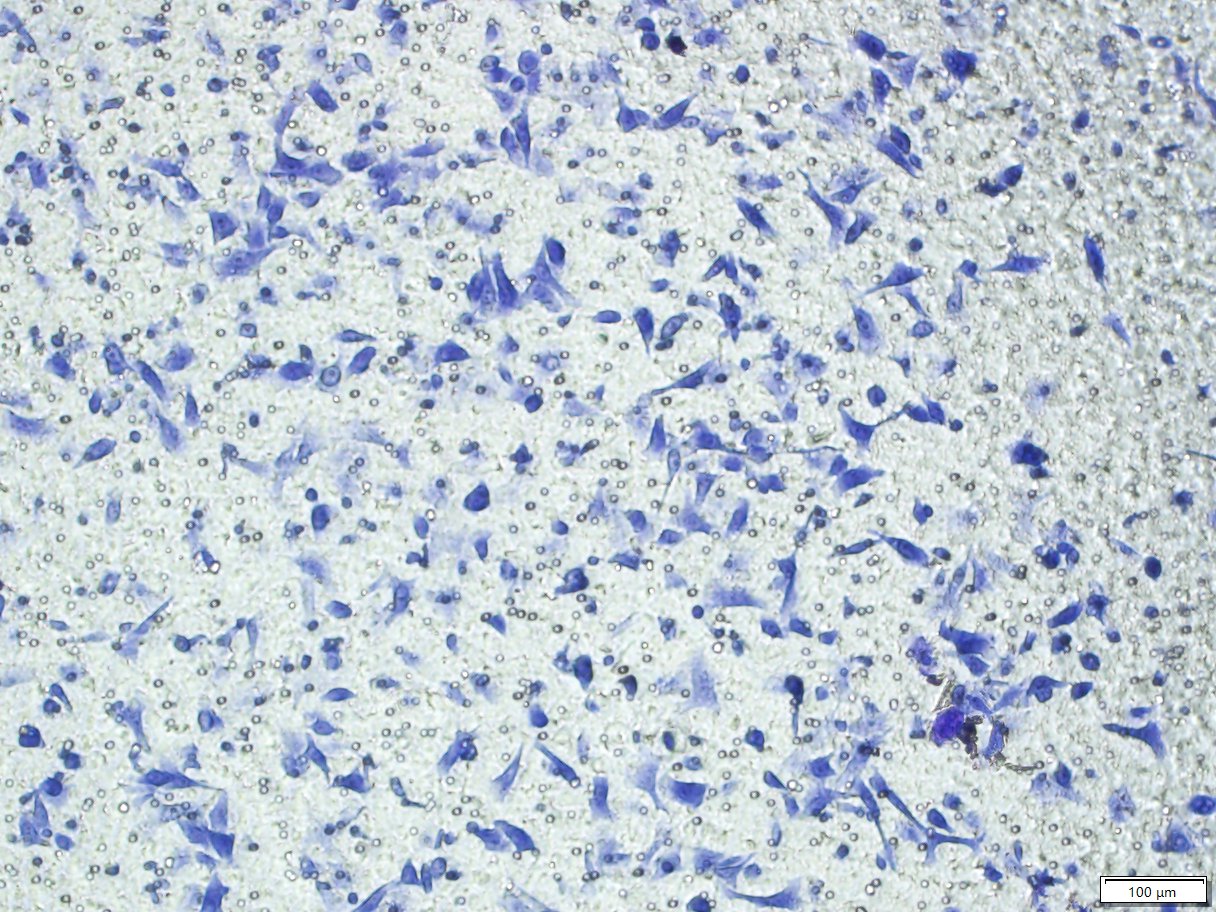

Supplement: Supplemental Information 4 [file peerj-cs-09-1651-s004.zip › Dataset 3/3+6.jpg]

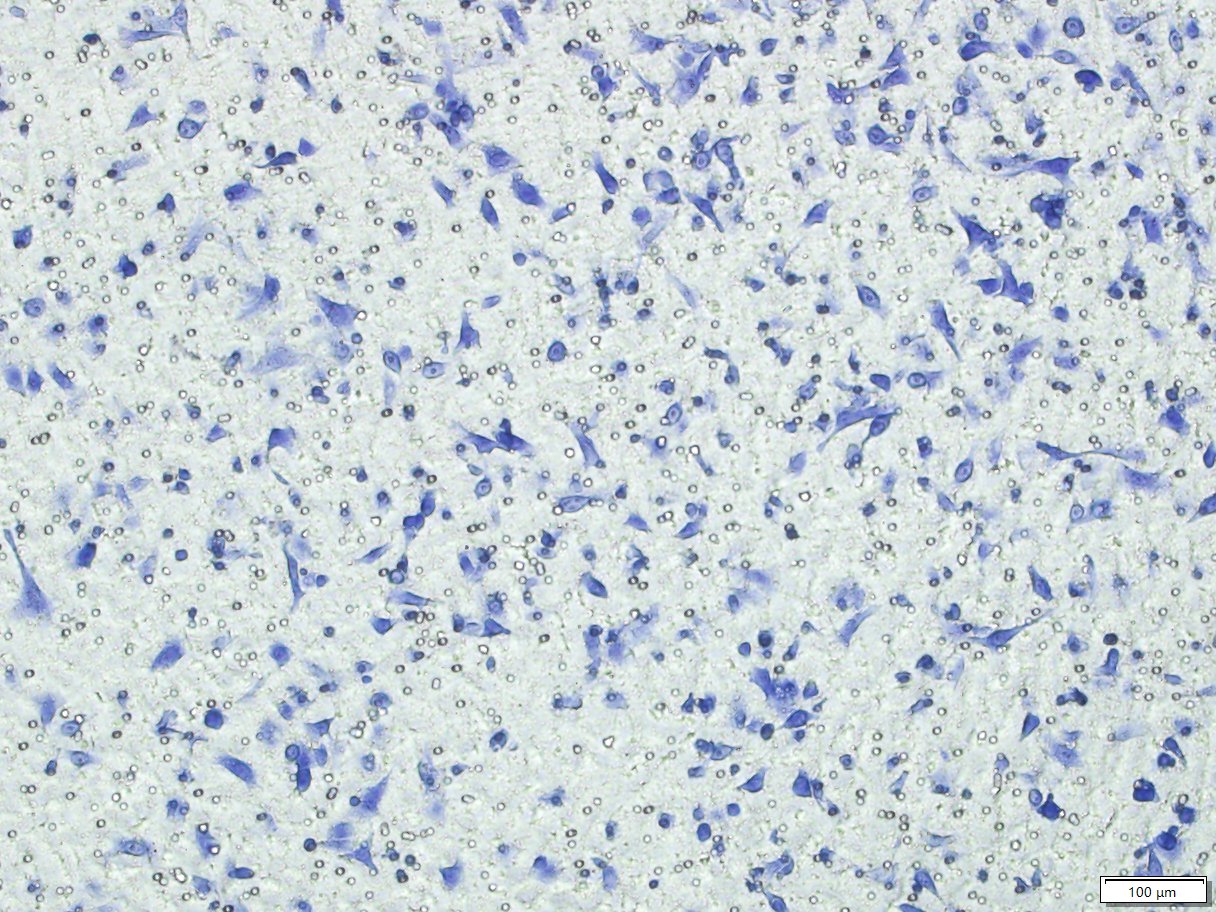

Supplement: Supplemental Information 4 [file peerj-cs-09-1651-s004.zip › Dataset 3/3+7.jpg]

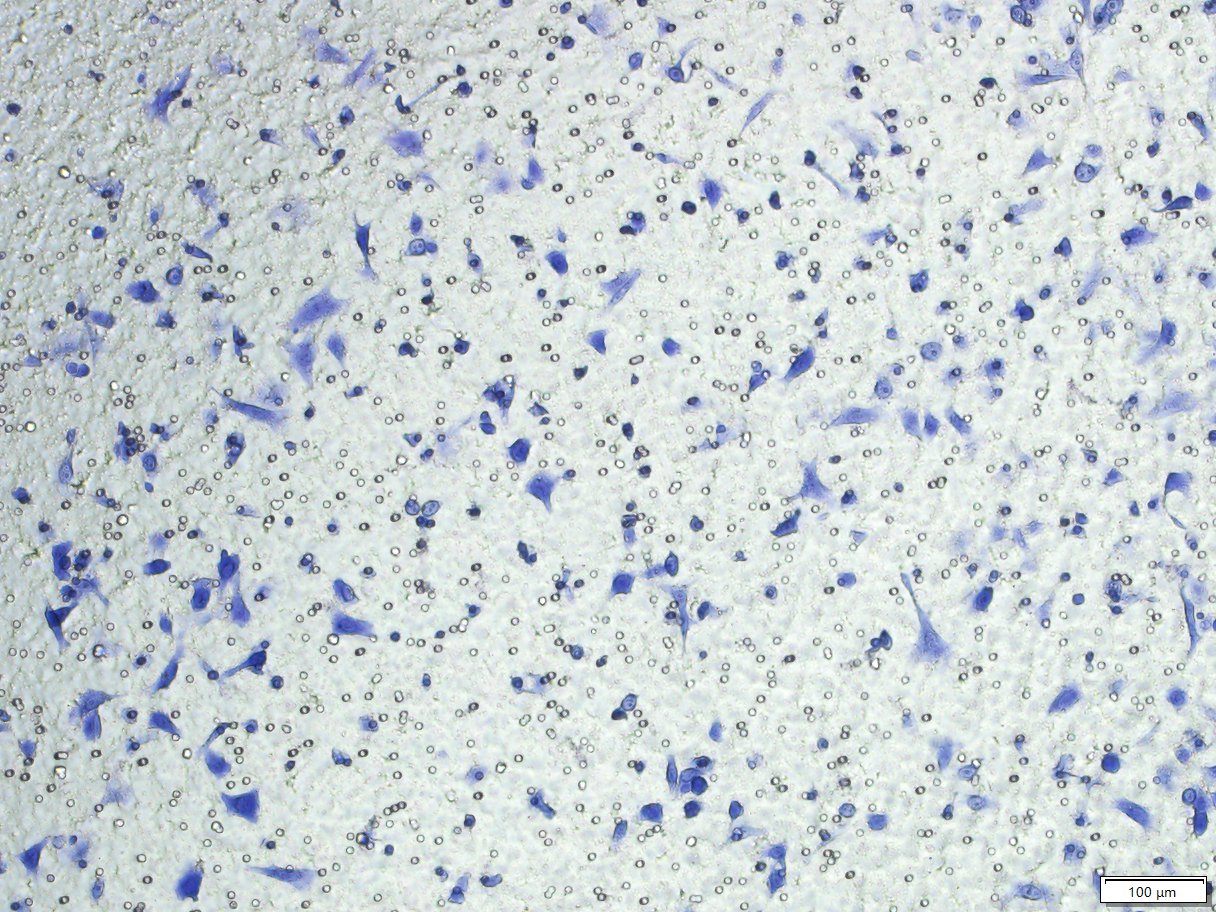

Supplement: Supplemental Information 4 [file peerj-cs-09-1651-s004.zip › Dataset 3/3+8.jpg]

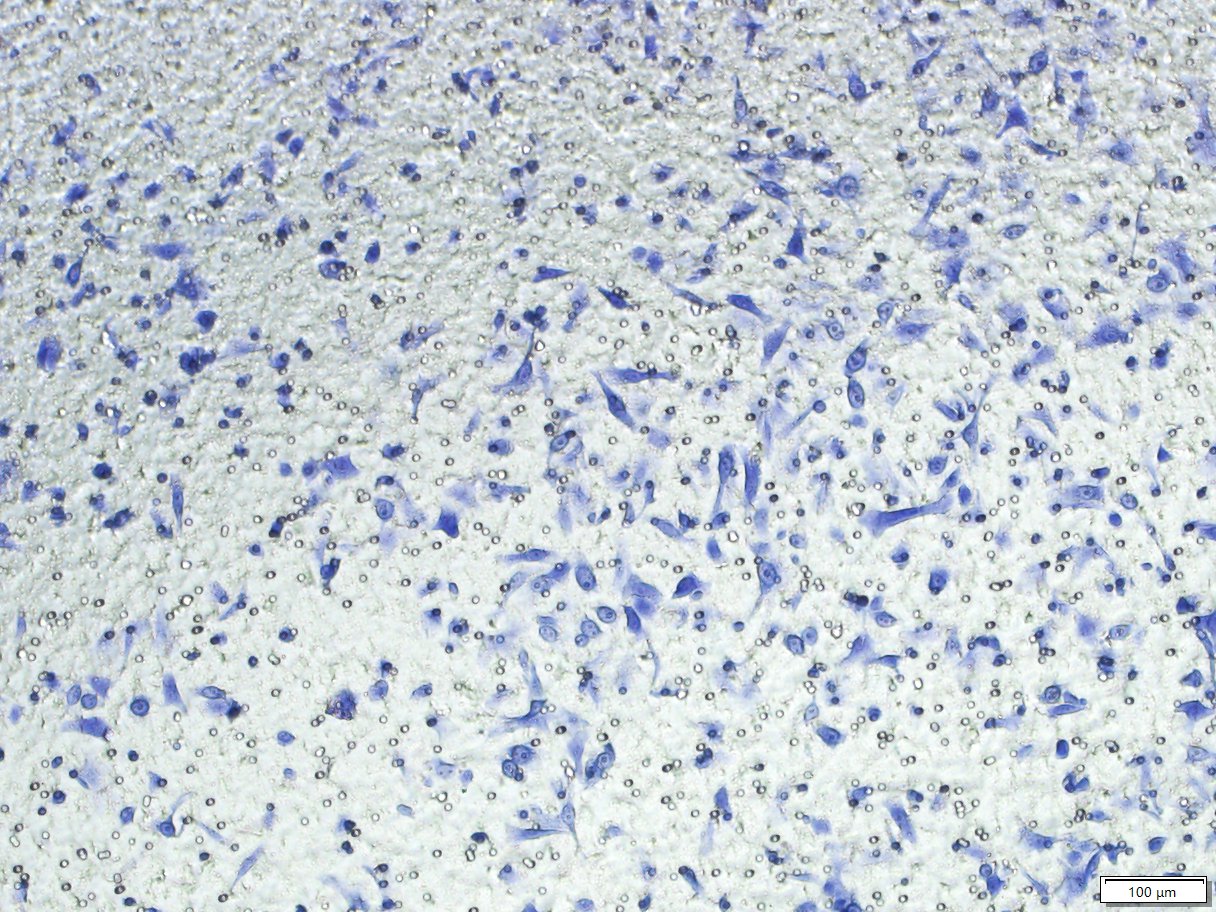

Supplement: Supplemental Information 4 [file peerj-cs-09-1651-s004.zip › Dataset 3/3+9.jpg]

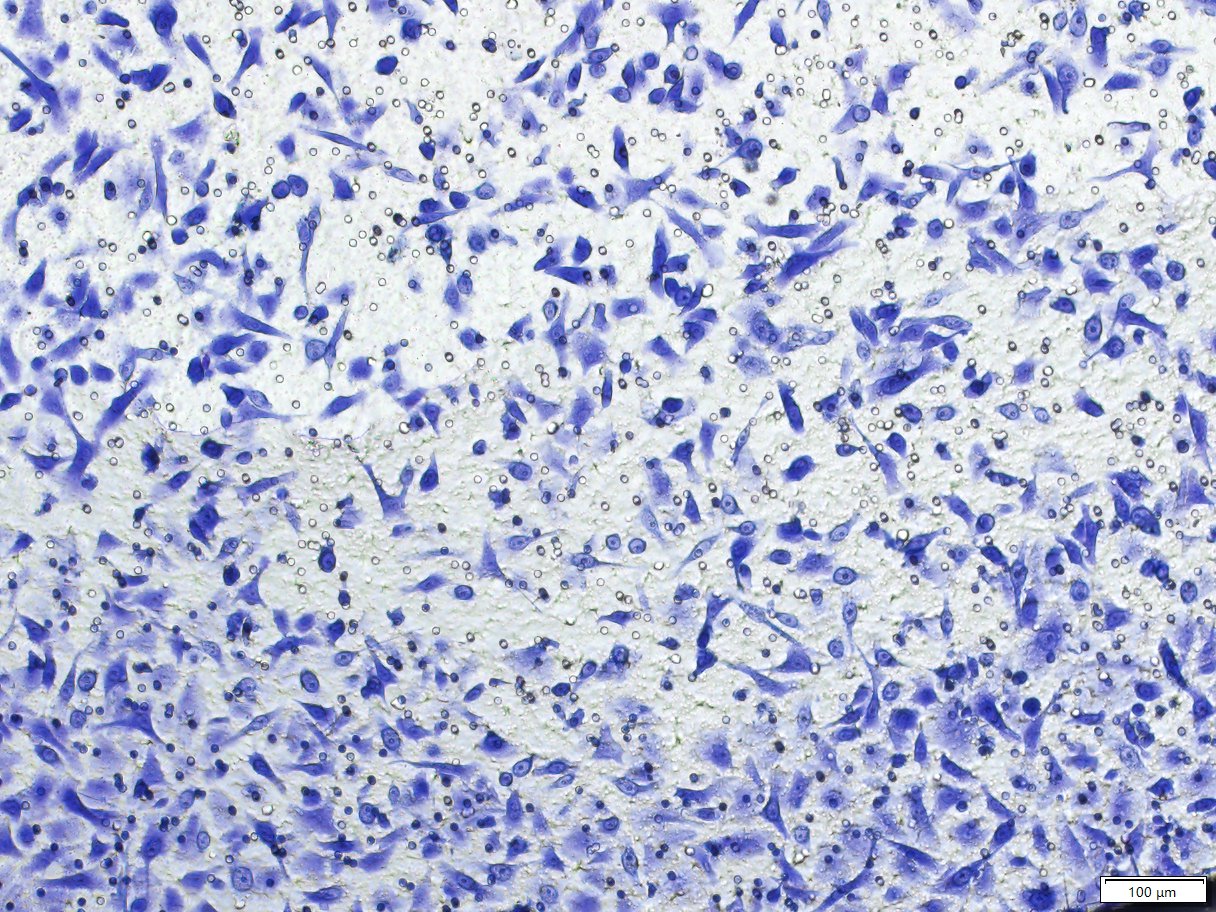

Supplement: Supplemental Information 4 [file peerj-cs-09-1651-s004.zip › Dataset 3/3-1.jpg]

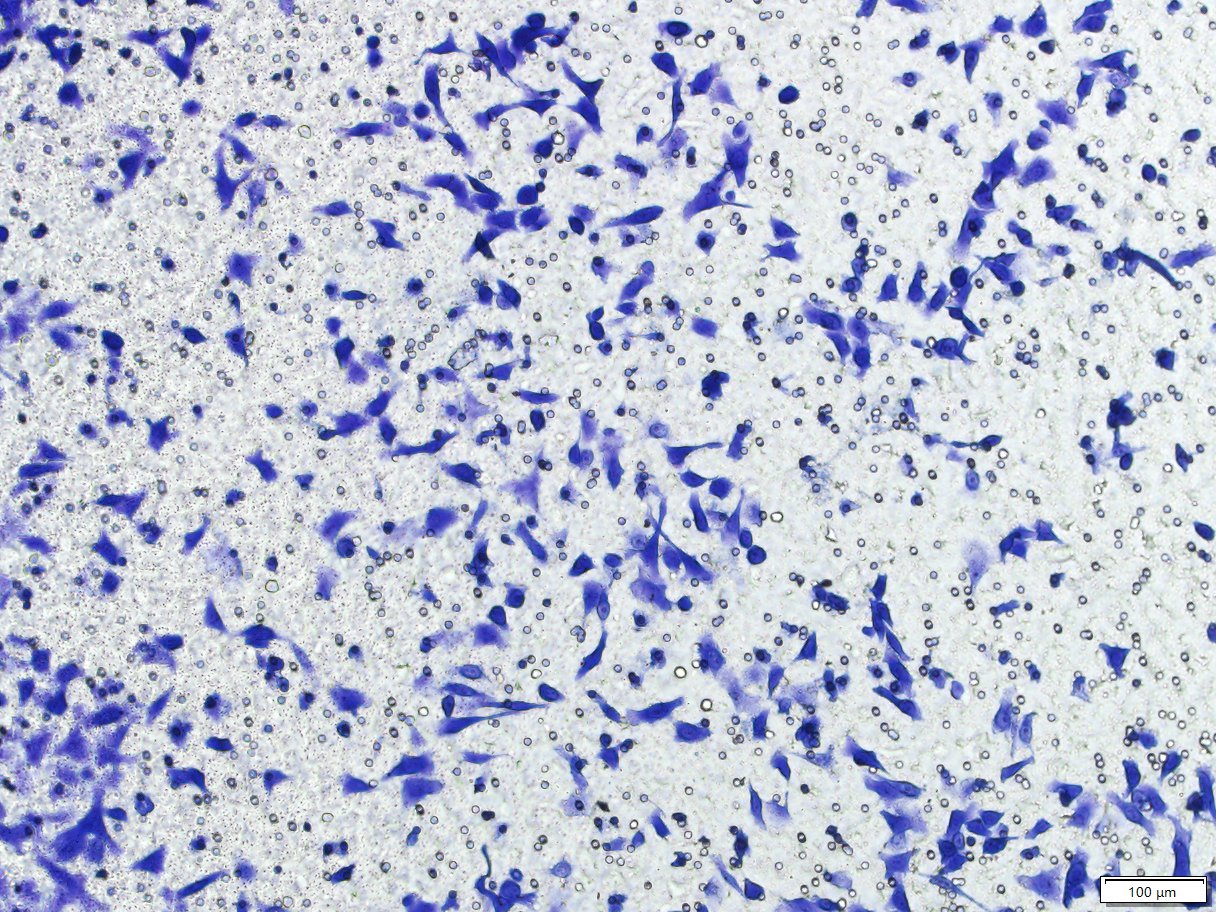

Supplement: Supplemental Information 4 [file peerj-cs-09-1651-s004.zip › Dataset 3/3-10.jpg]

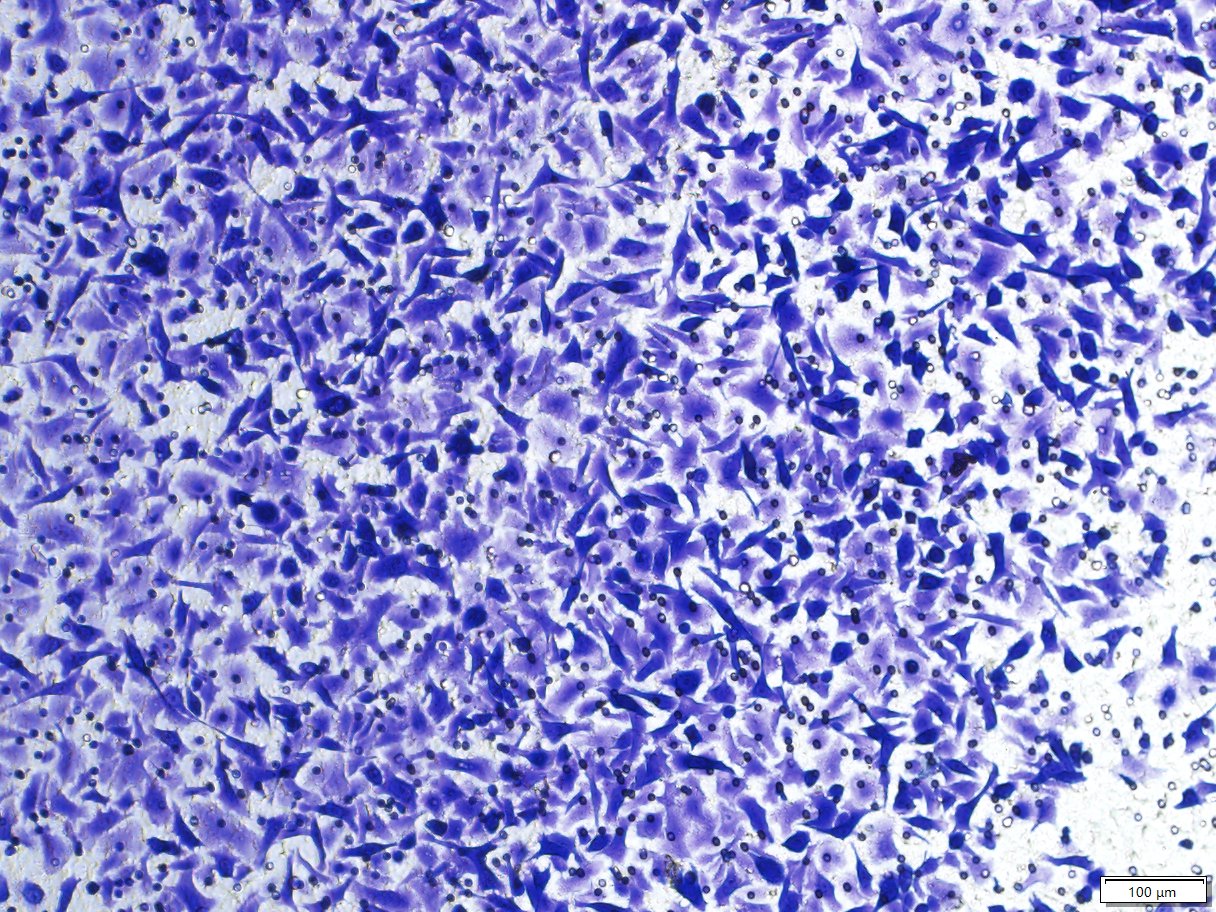

Supplement: Supplemental Information 4 [file peerj-cs-09-1651-s004.zip › Dataset 3/3-11.jpg]

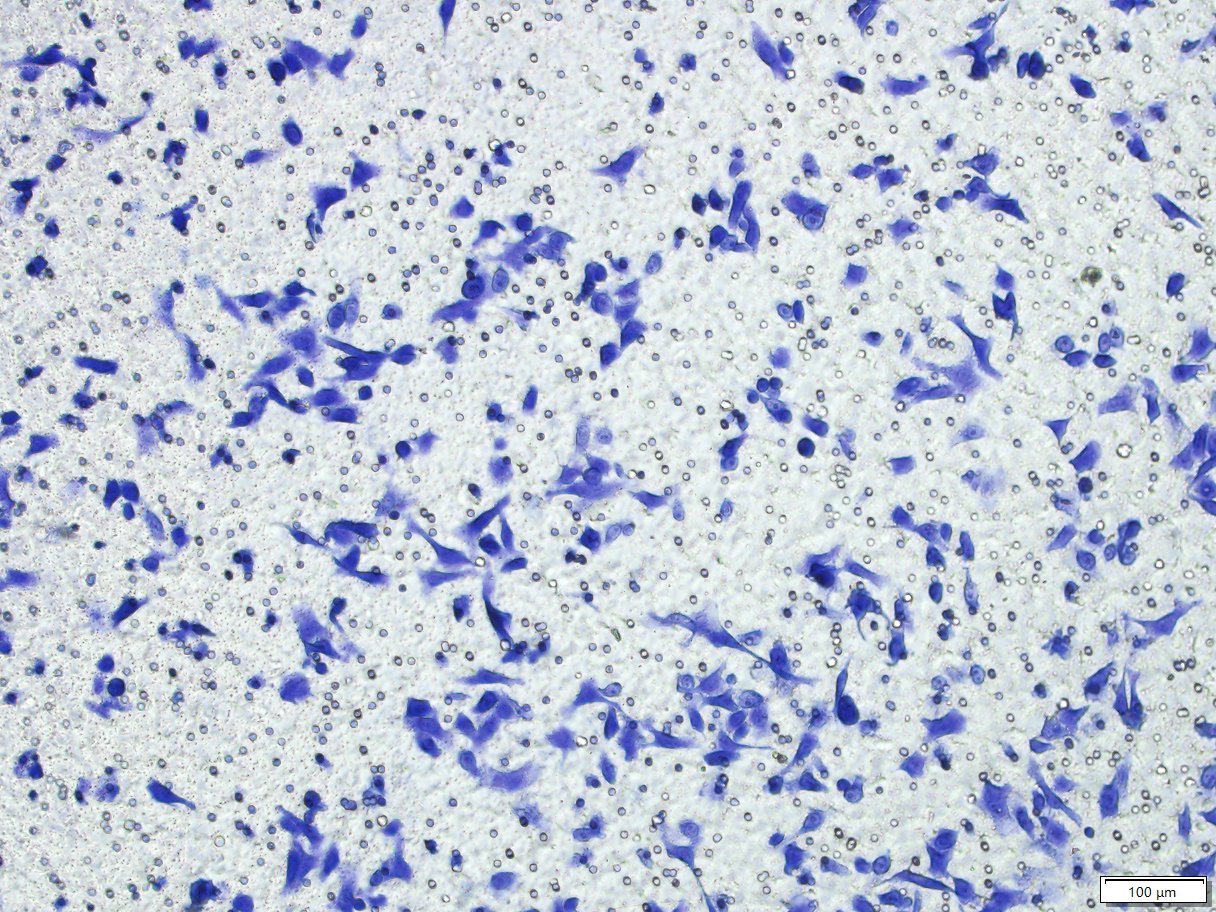

Supplement: Supplemental Information 4 [file peerj-cs-09-1651-s004.zip › Dataset 3/3-12.jpg]

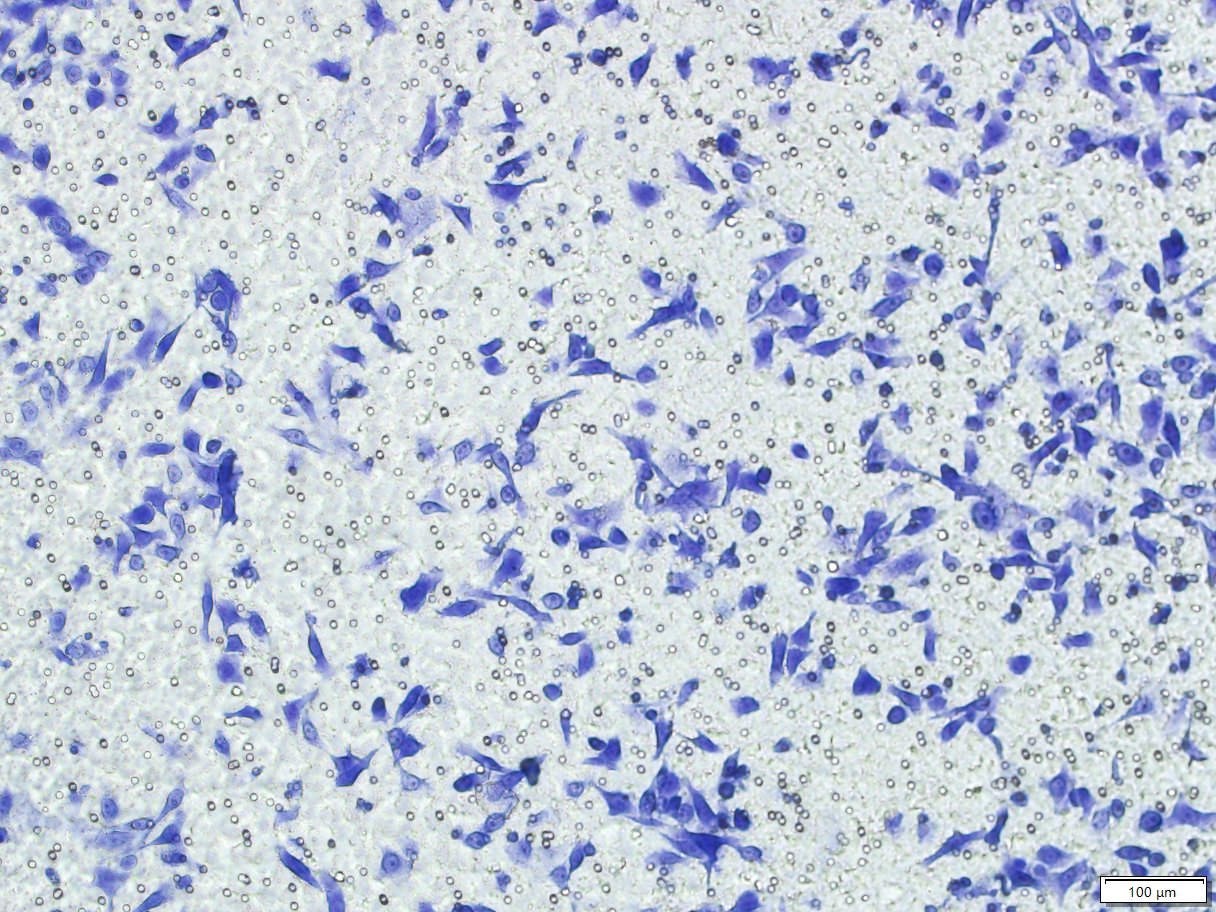

Supplement: Supplemental Information 4 [file peerj-cs-09-1651-s004.zip › Dataset 3/3-13.jpg]

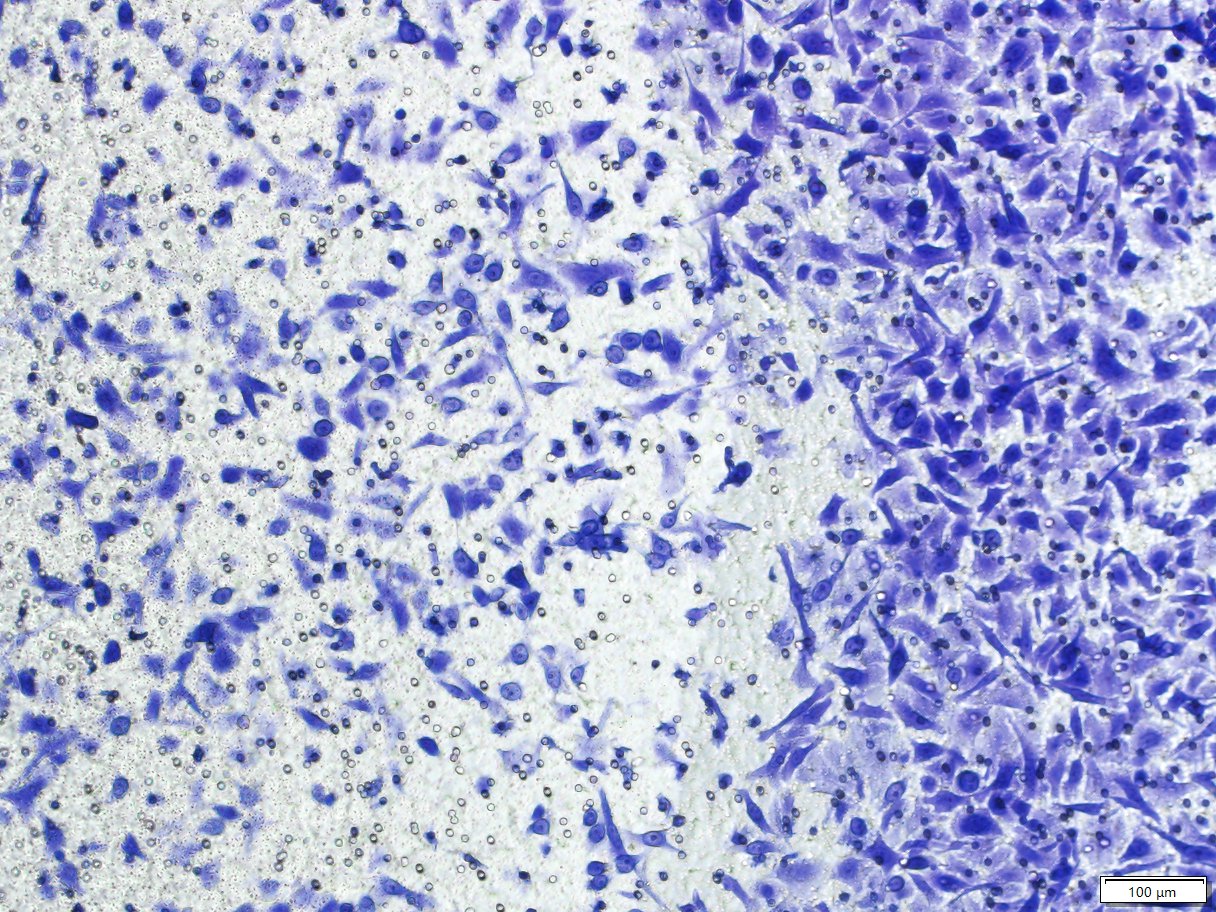

Supplement: Supplemental Information 4 [file peerj-cs-09-1651-s004.zip › Dataset 3/3-14.jpg]

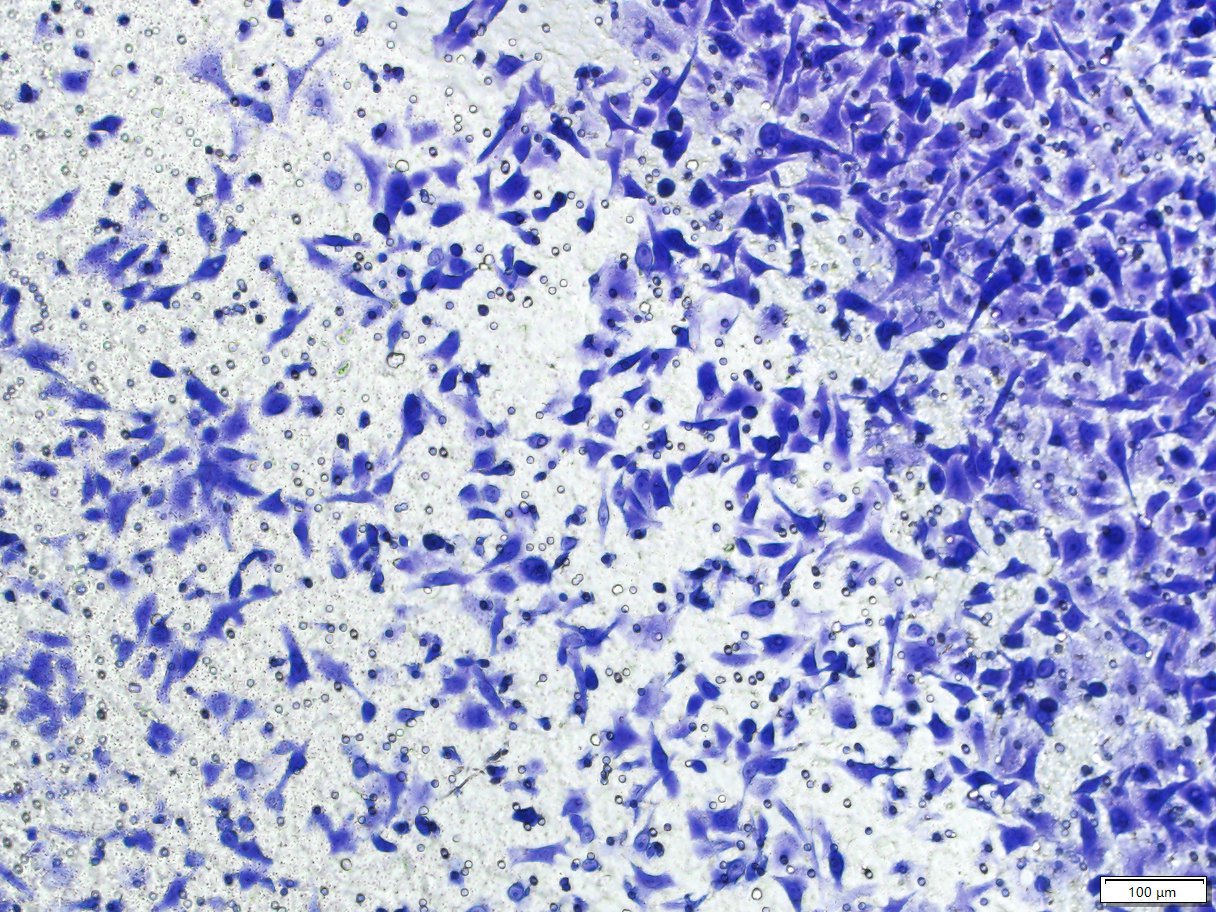

Supplement: Supplemental Information 4 [file peerj-cs-09-1651-s004.zip › Dataset 3/3-15.jpg]

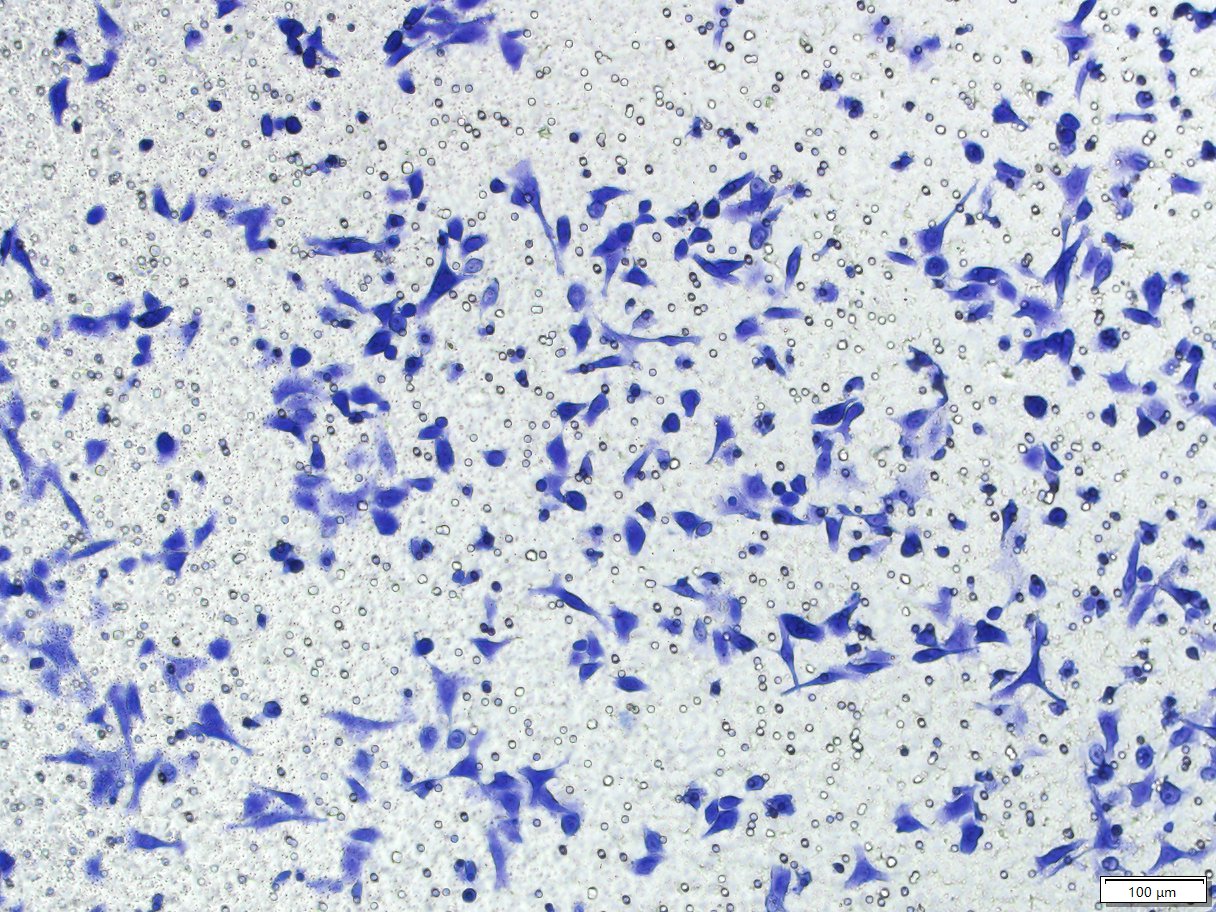

Supplement: Supplemental Information 4 [file peerj-cs-09-1651-s004.zip › Dataset 3/3-16.jpg]

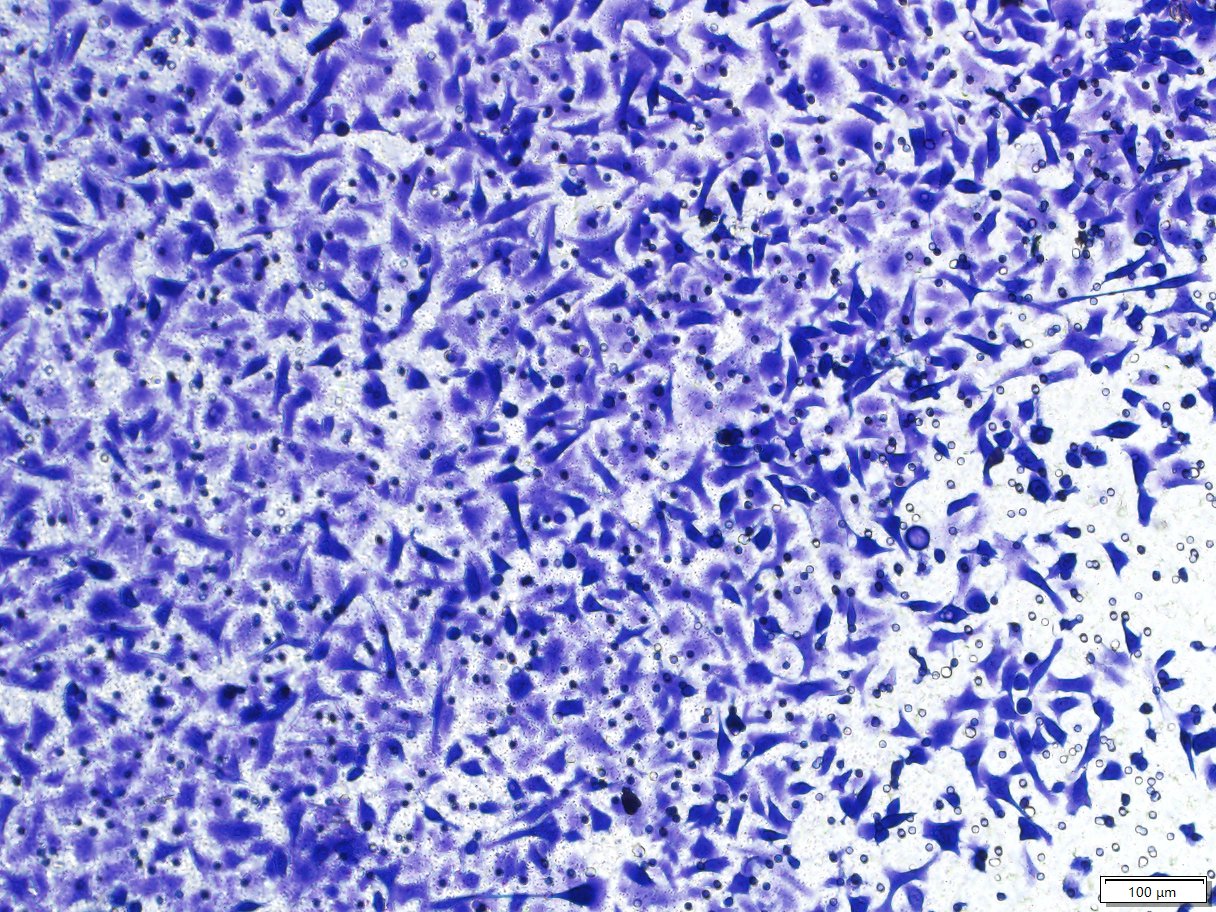

Supplement: Supplemental Information 4 [file peerj-cs-09-1651-s004.zip › Dataset 3/3-17.jpg]

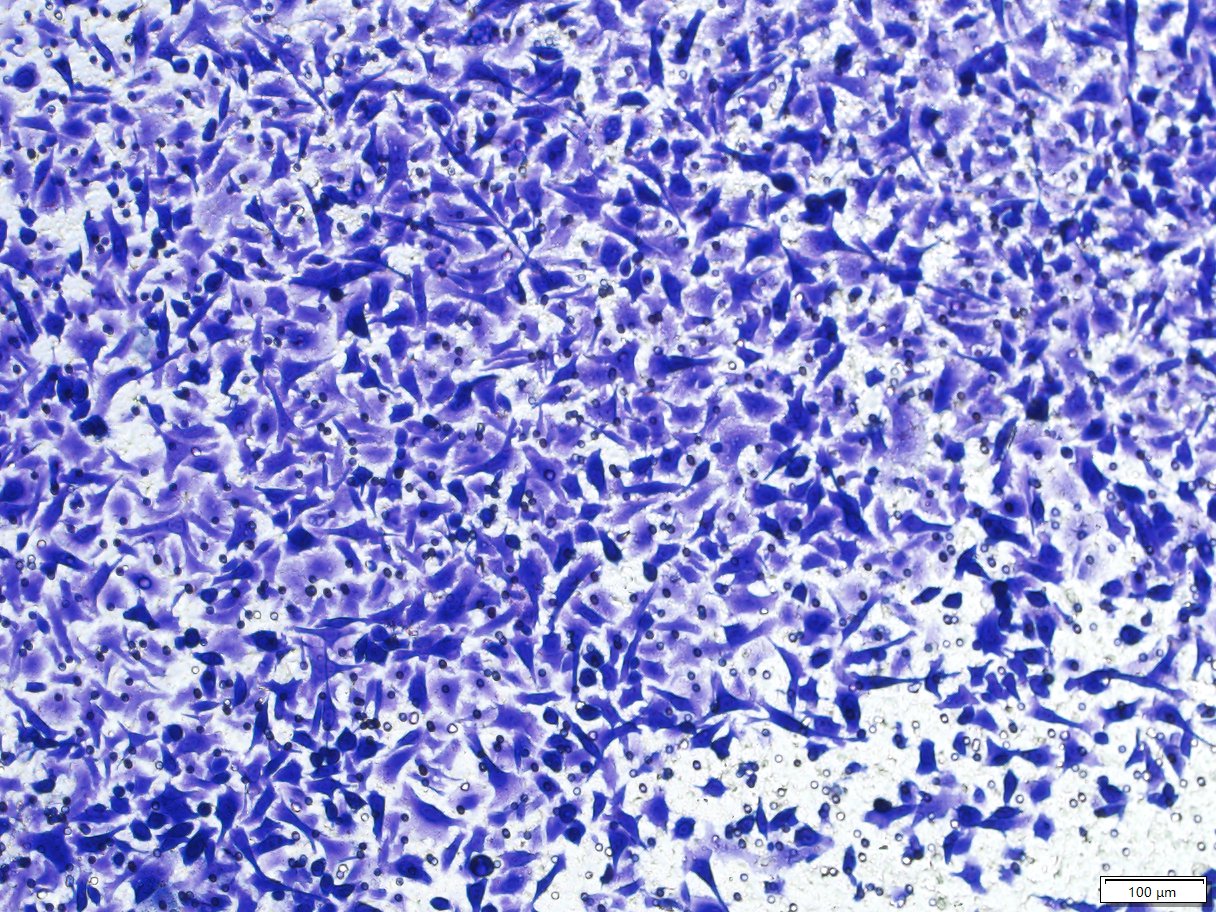

Supplement: Supplemental Information 4 [file peerj-cs-09-1651-s004.zip › Dataset 3/3-18.jpg]

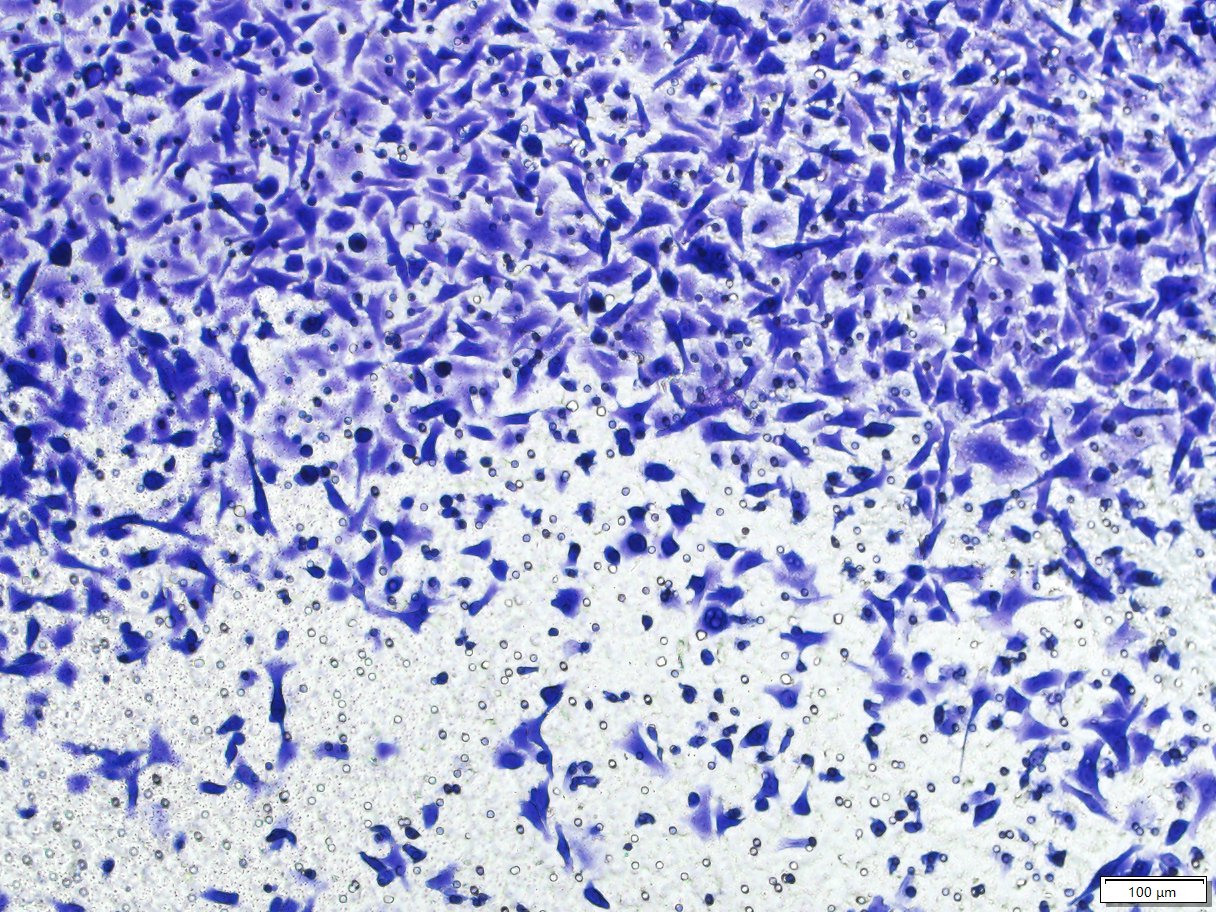

Supplement: Supplemental Information 4 [file peerj-cs-09-1651-s004.zip › Dataset 3/3-19.jpg]

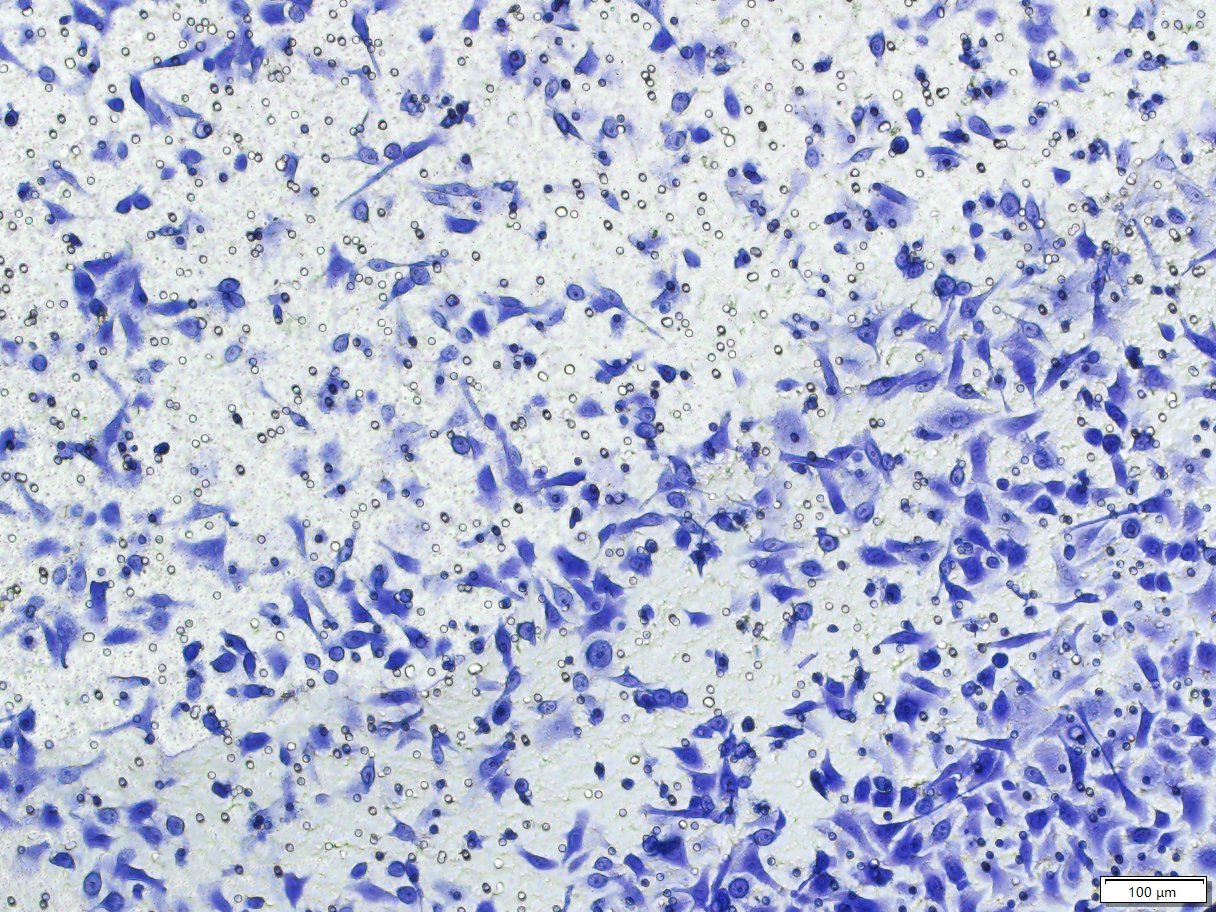

Supplement: Supplemental Information 4 [file peerj-cs-09-1651-s004.zip › Dataset 3/3-2.jpg]

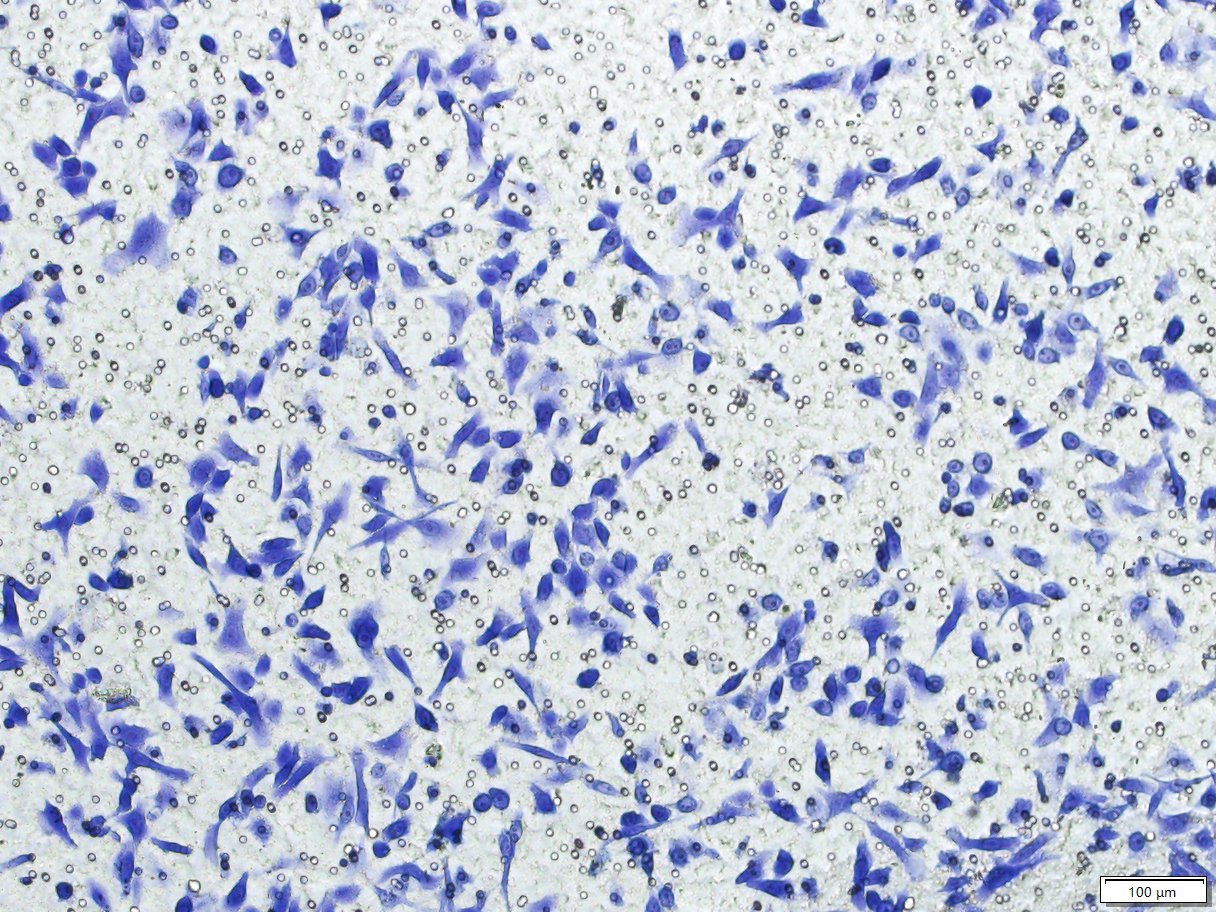

Supplement: Supplemental Information 4 [file peerj-cs-09-1651-s004.zip › Dataset 3/3-3.jpg]

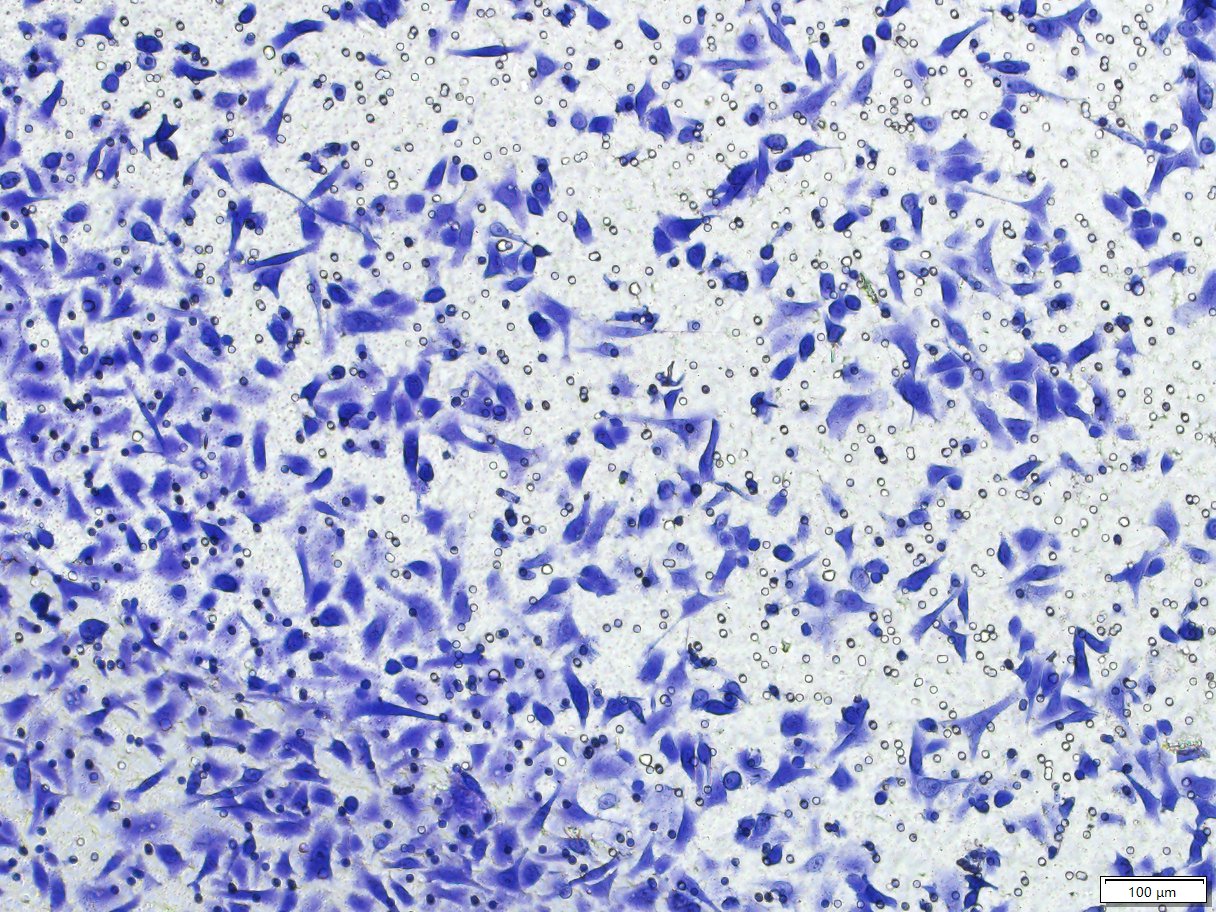

Supplement: Supplemental Information 4 [file peerj-cs-09-1651-s004.zip › Dataset 3/3-4.jpg]

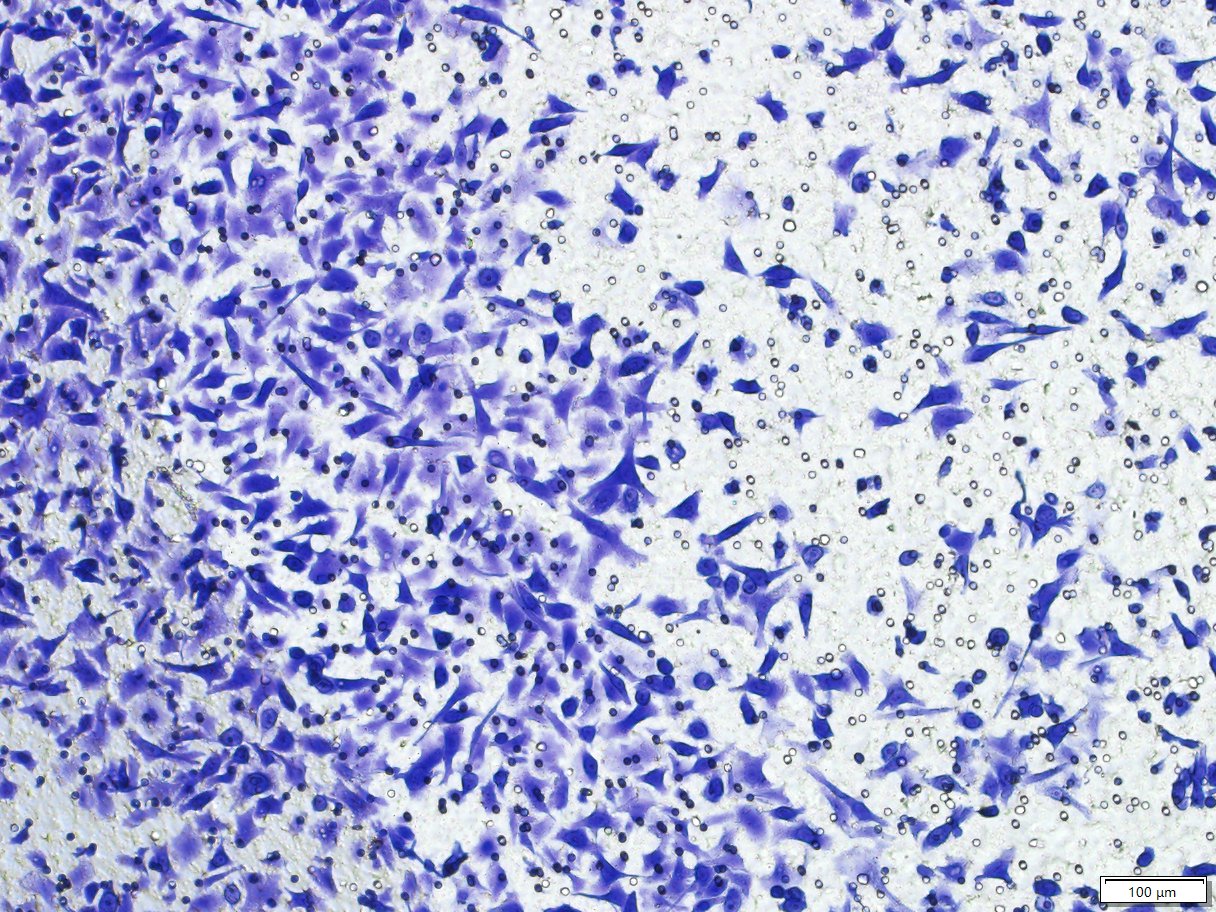

Supplement: Supplemental Information 4 [file peerj-cs-09-1651-s004.zip › Dataset 3/3-5.jpg]

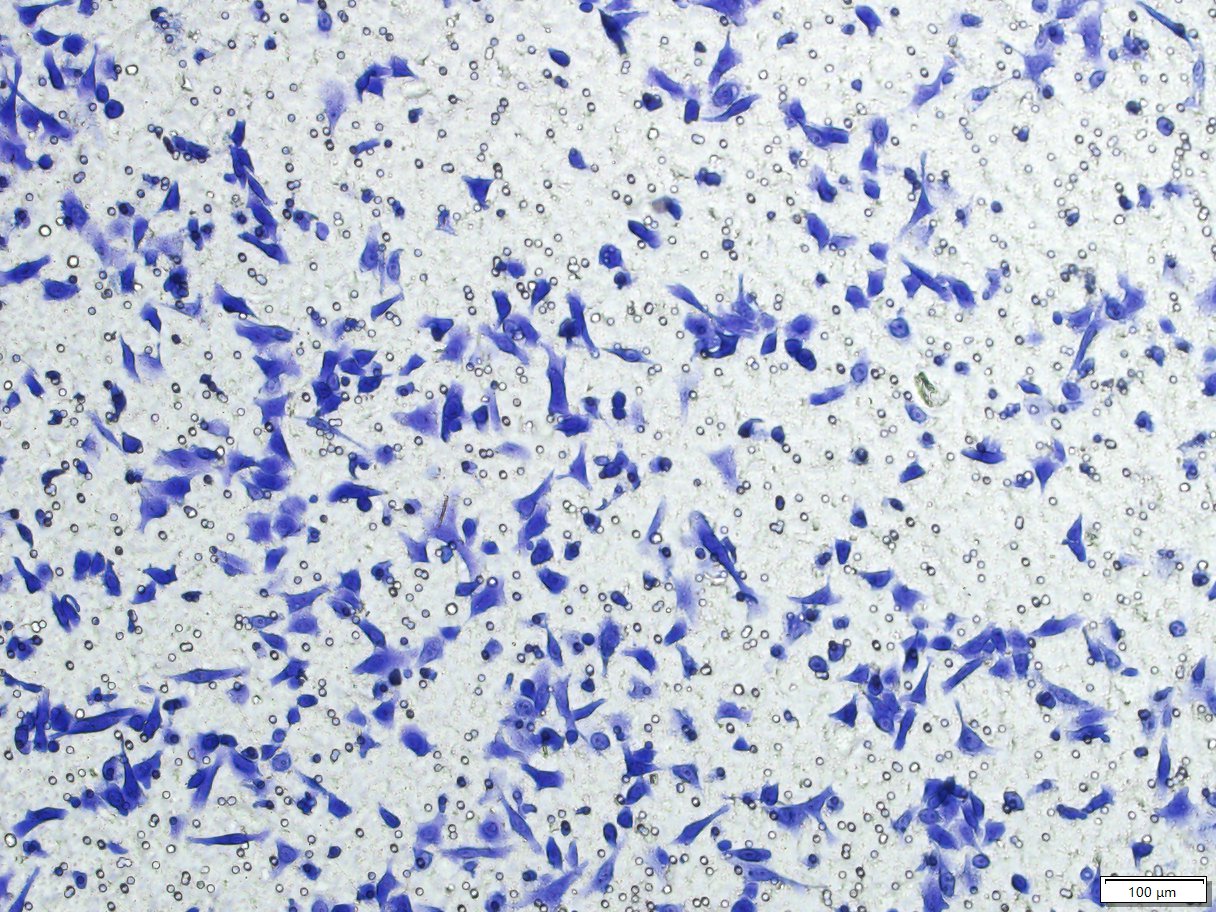

Supplement: Supplemental Information 4 [file peerj-cs-09-1651-s004.zip › Dataset 3/3-6.jpg]

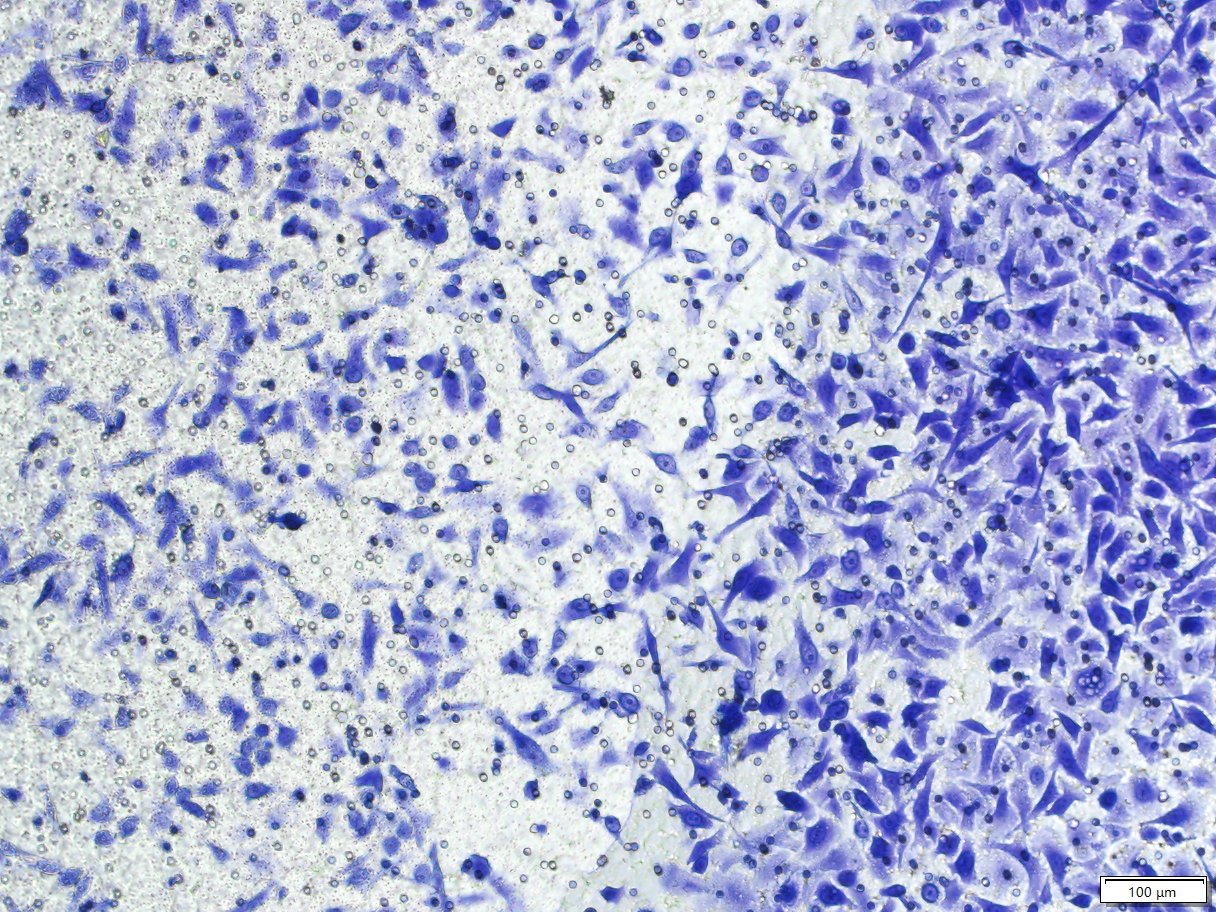

Supplement: Supplemental Information 4 [file peerj-cs-09-1651-s004.zip › Dataset 3/3-7.jpg]

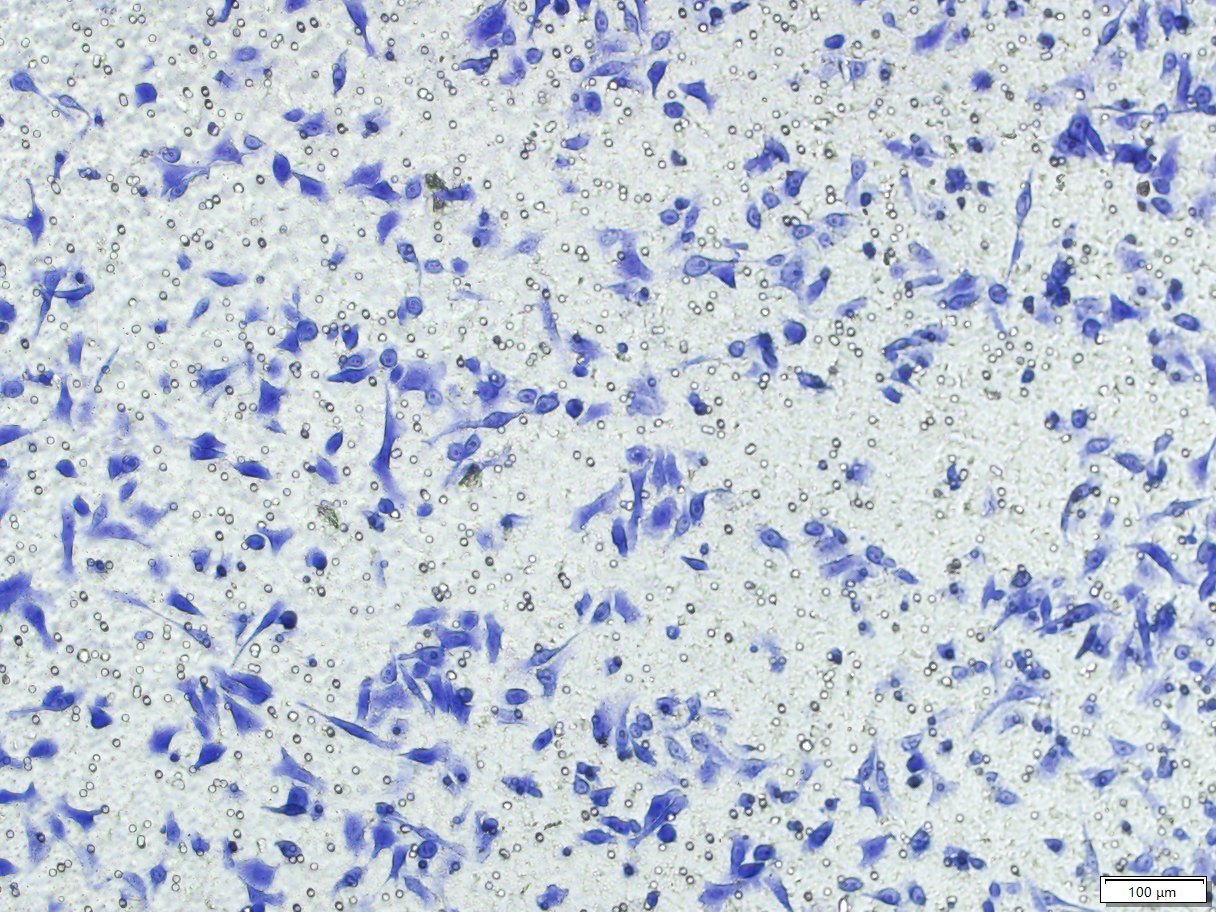

Supplement: Supplemental Information 4 [file peerj-cs-09-1651-s004.zip › Dataset 3/3-8.jpg]

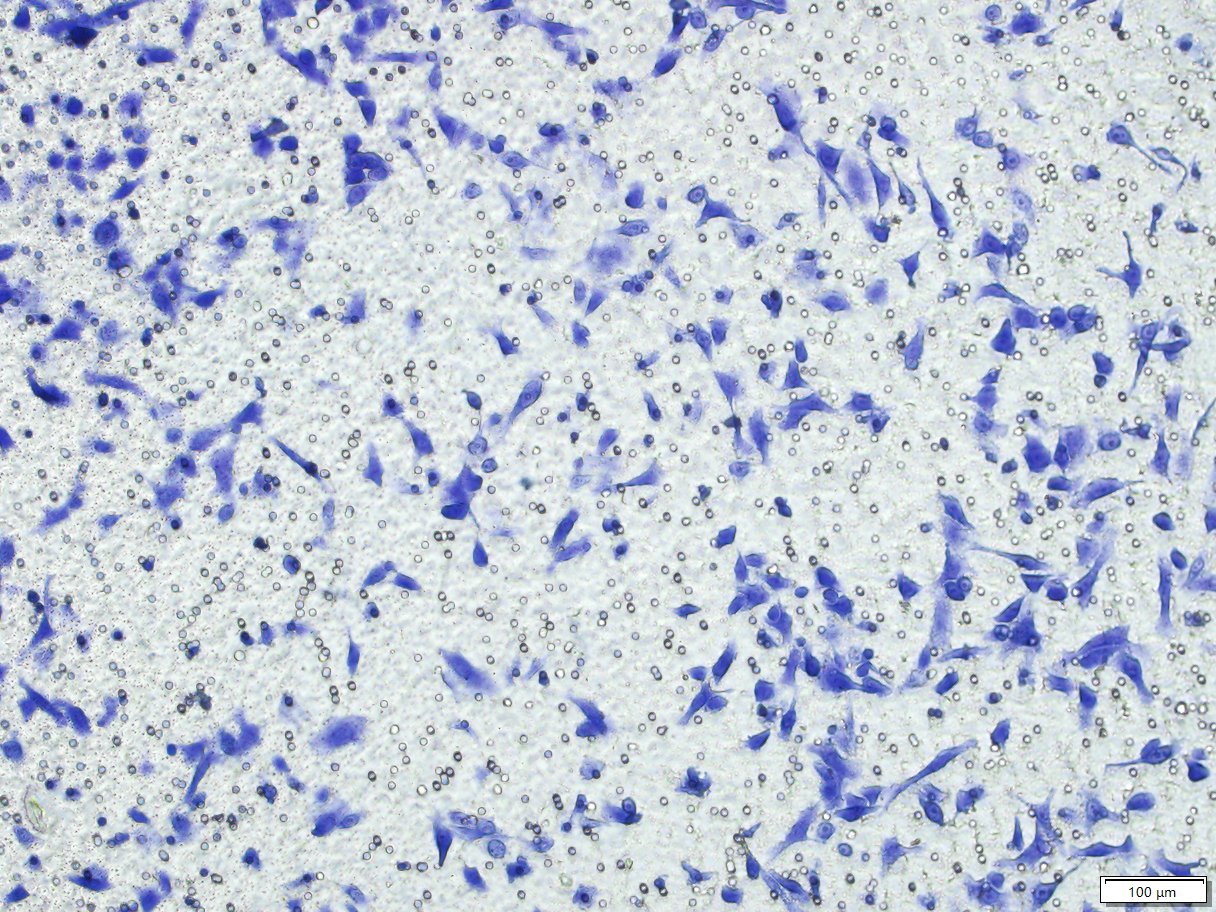

Supplement: Supplemental Information 4 [file peerj-cs-09-1651-s004.zip › Dataset 3/3-9.jpg]

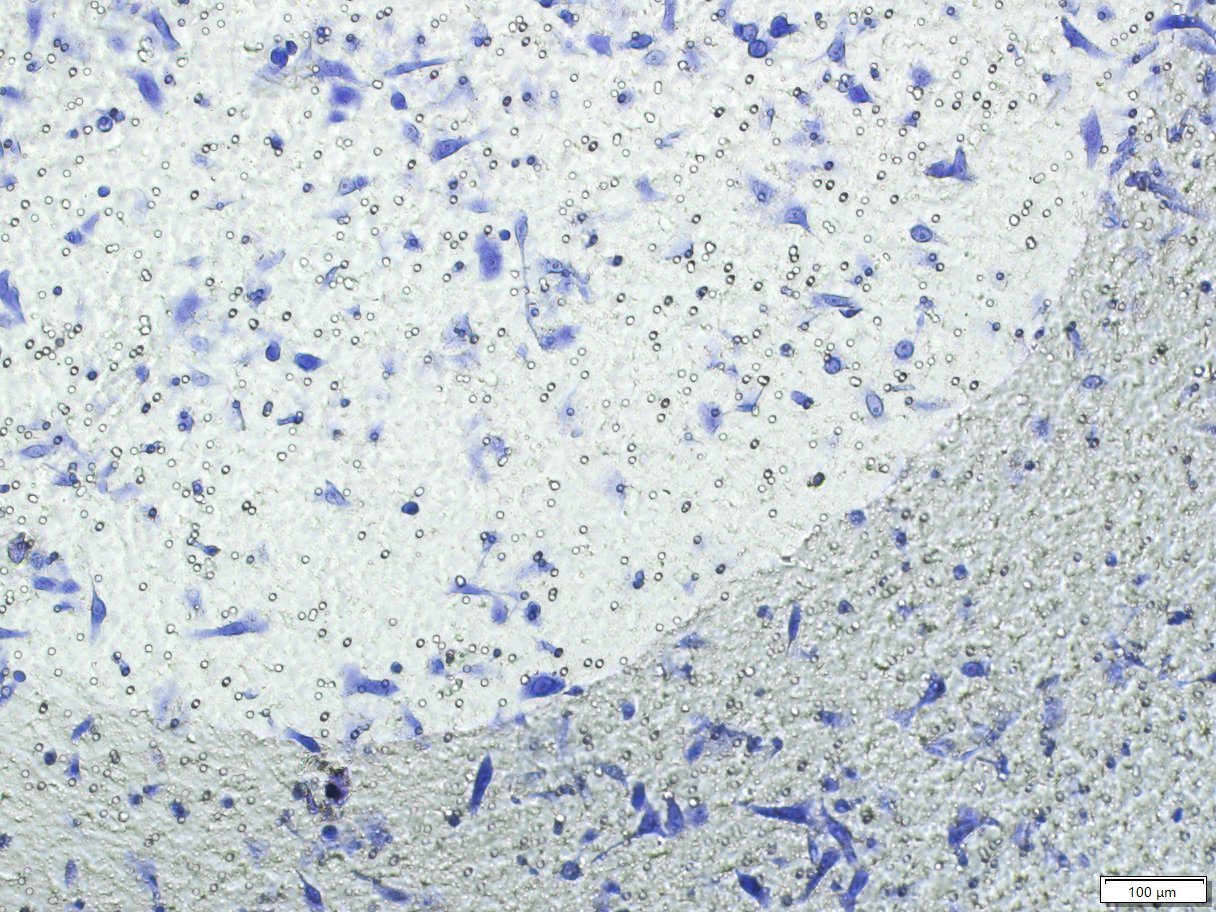

Supplement: Supplemental Information 4 [file peerj-cs-09-1651-s004.zip › Dataset 3/4+1.jpg]

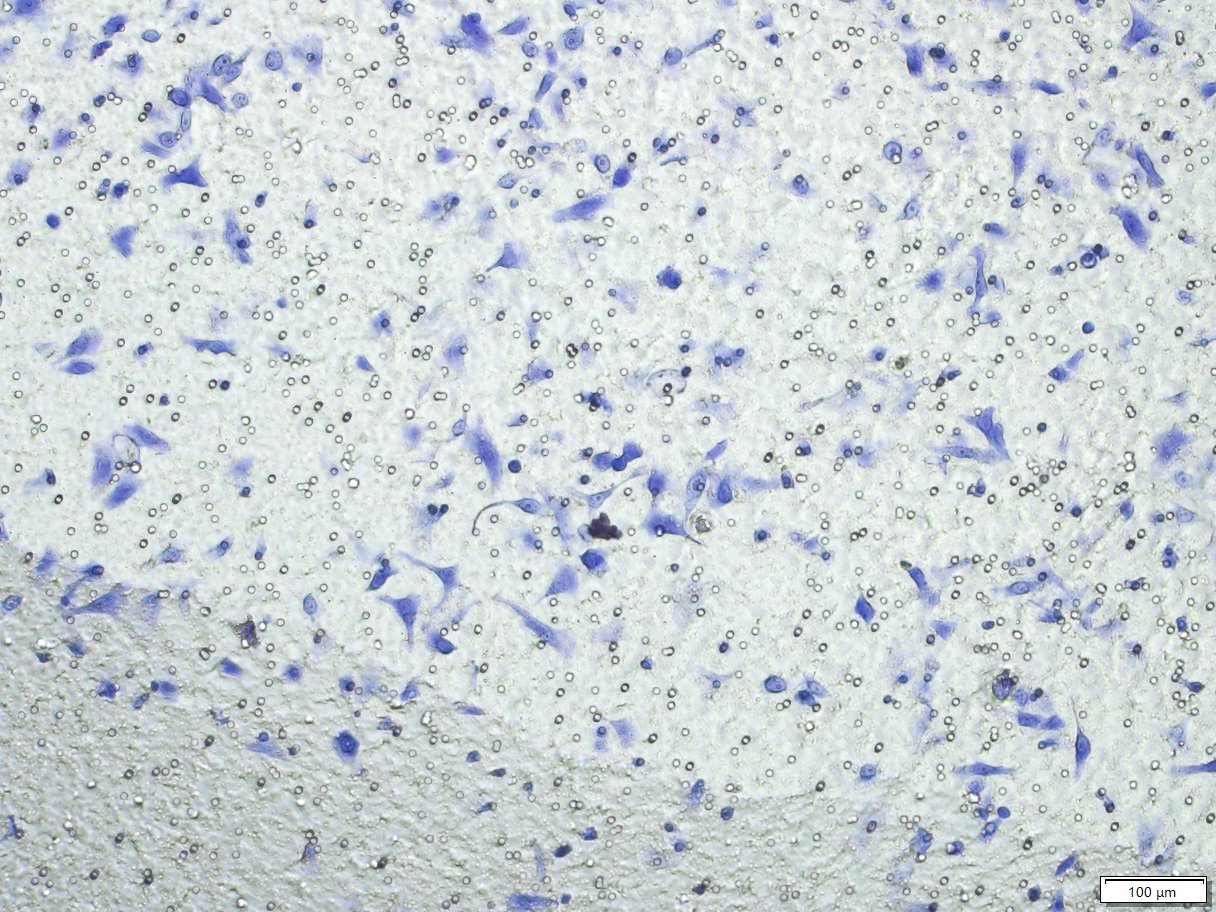

Supplement: Supplemental Information 4 [file peerj-cs-09-1651-s004.zip › Dataset 3/4+2.jpg]

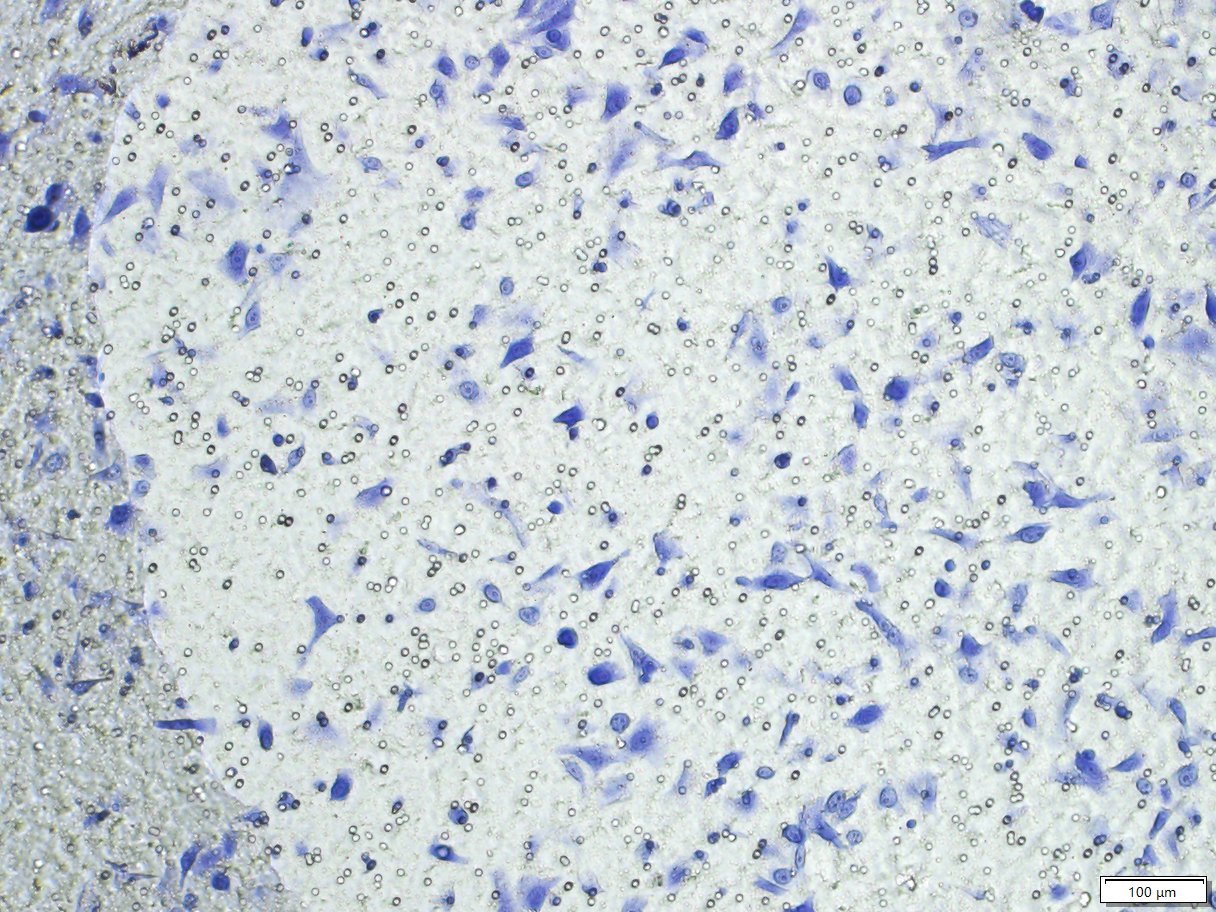

Supplement: Supplemental Information 4 [file peerj-cs-09-1651-s004.zip › Dataset 3/4+3.jpg]

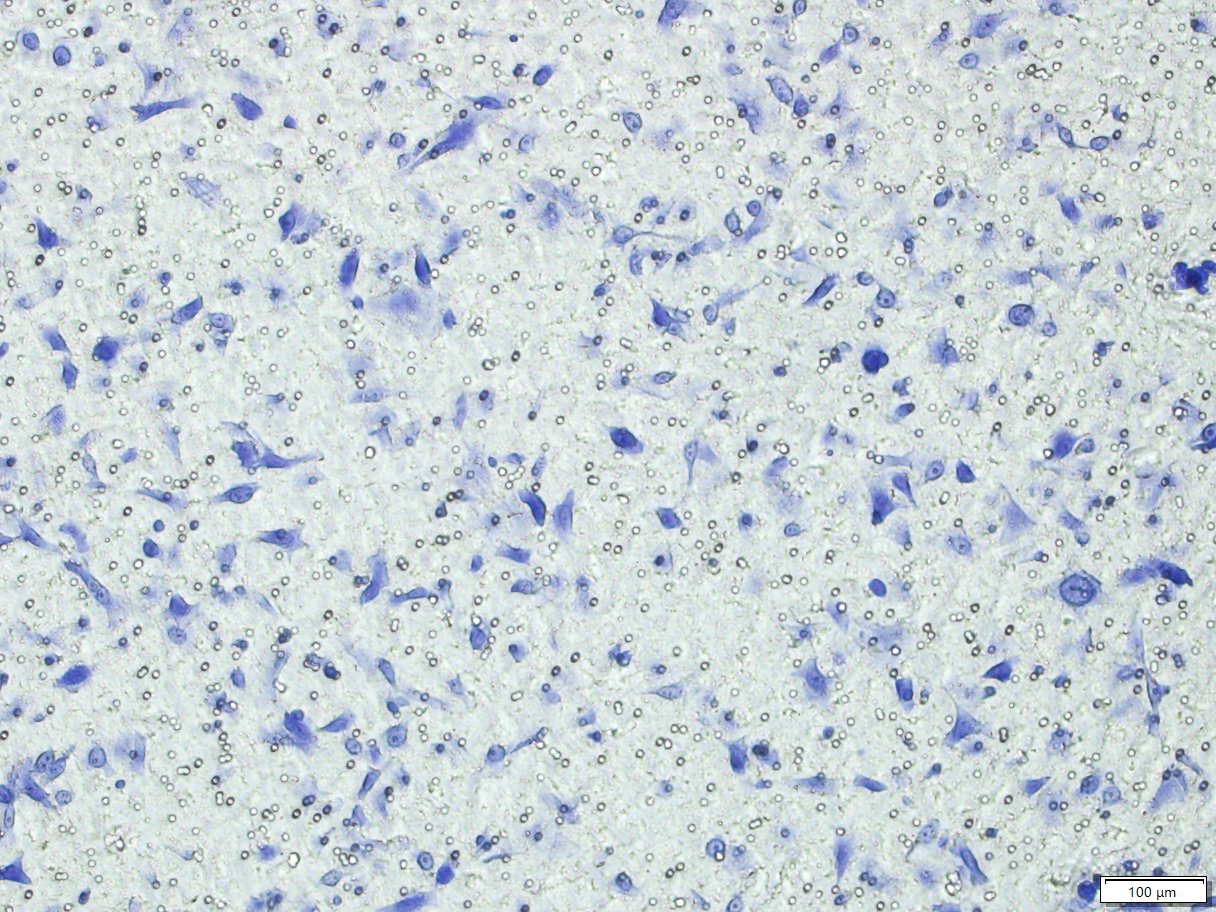

Supplement: Supplemental Information 4 [file peerj-cs-09-1651-s004.zip › Dataset 3/4+4.jpg]

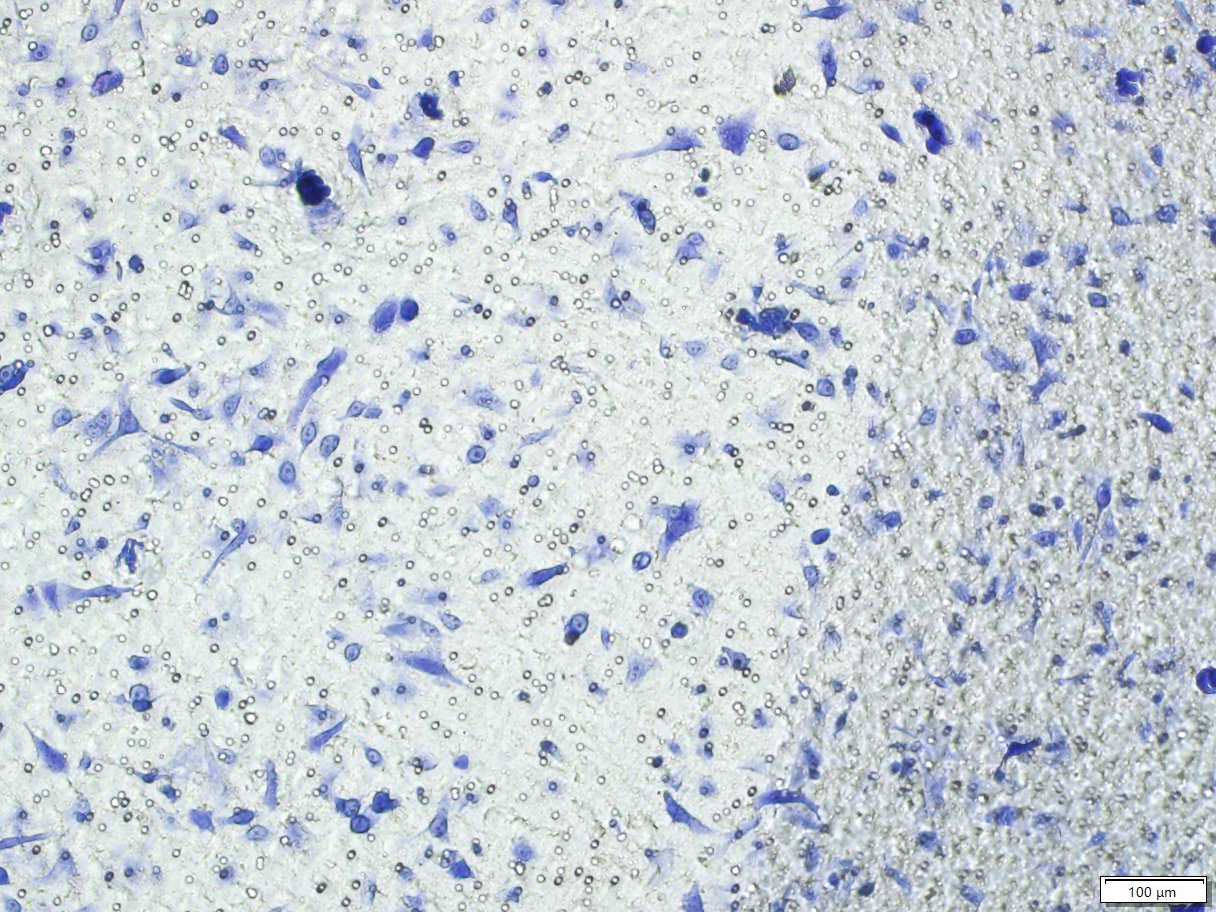

Supplement: Supplemental Information 4 [file peerj-cs-09-1651-s004.zip › Dataset 3/4+5.jpg]

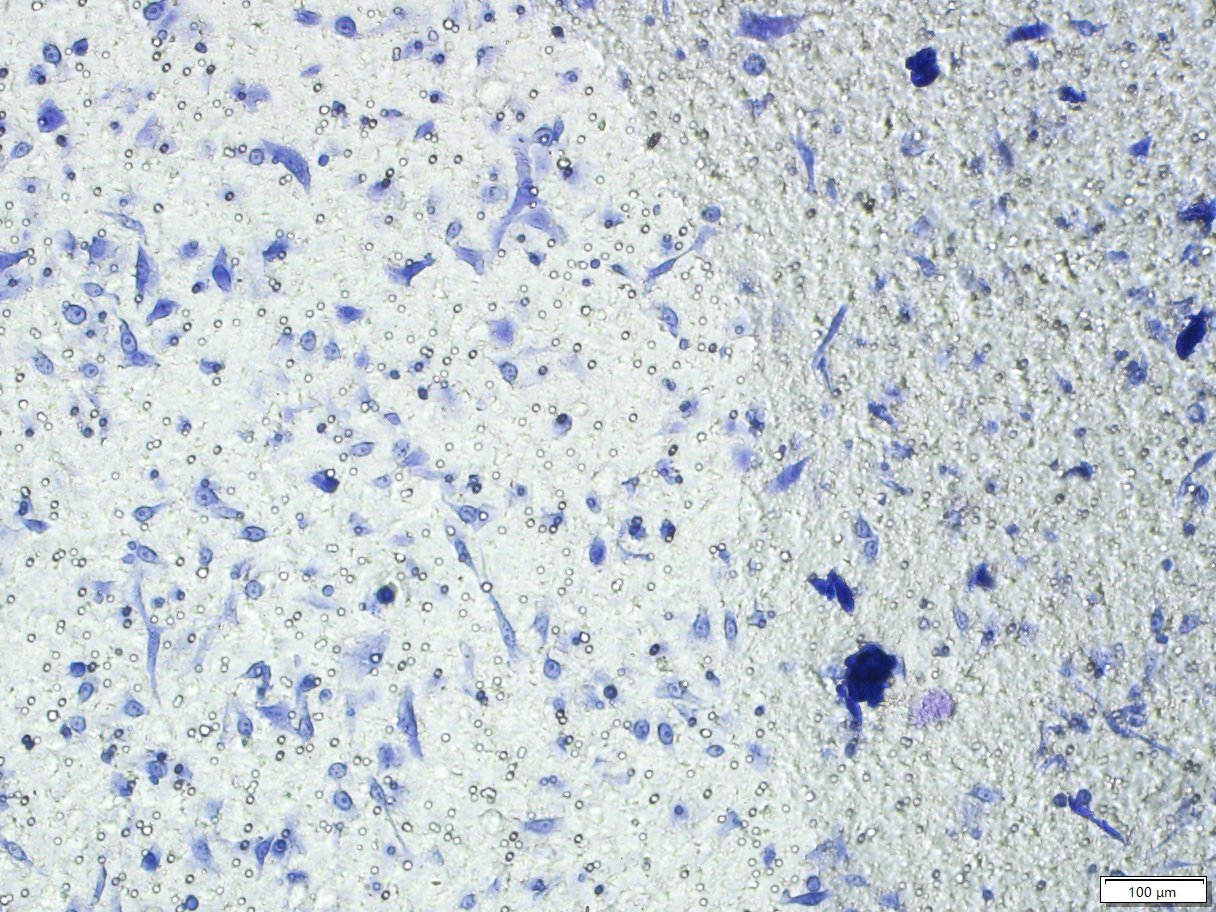

Supplement: Supplemental Information 4 [file peerj-cs-09-1651-s004.zip › Dataset 3/4+6.jpg]

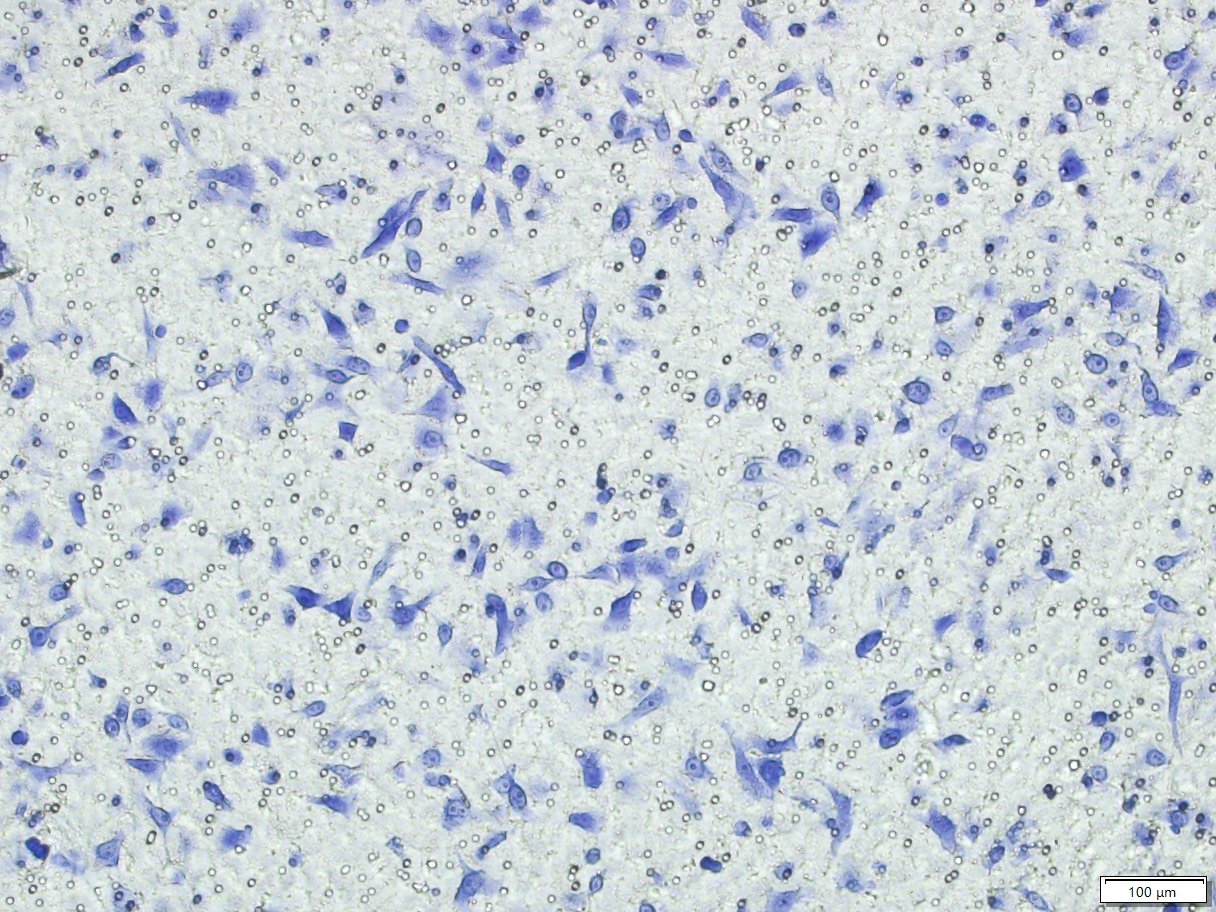

Supplement: Supplemental Information 4 [file peerj-cs-09-1651-s004.zip › Dataset 3/4+7.jpg]

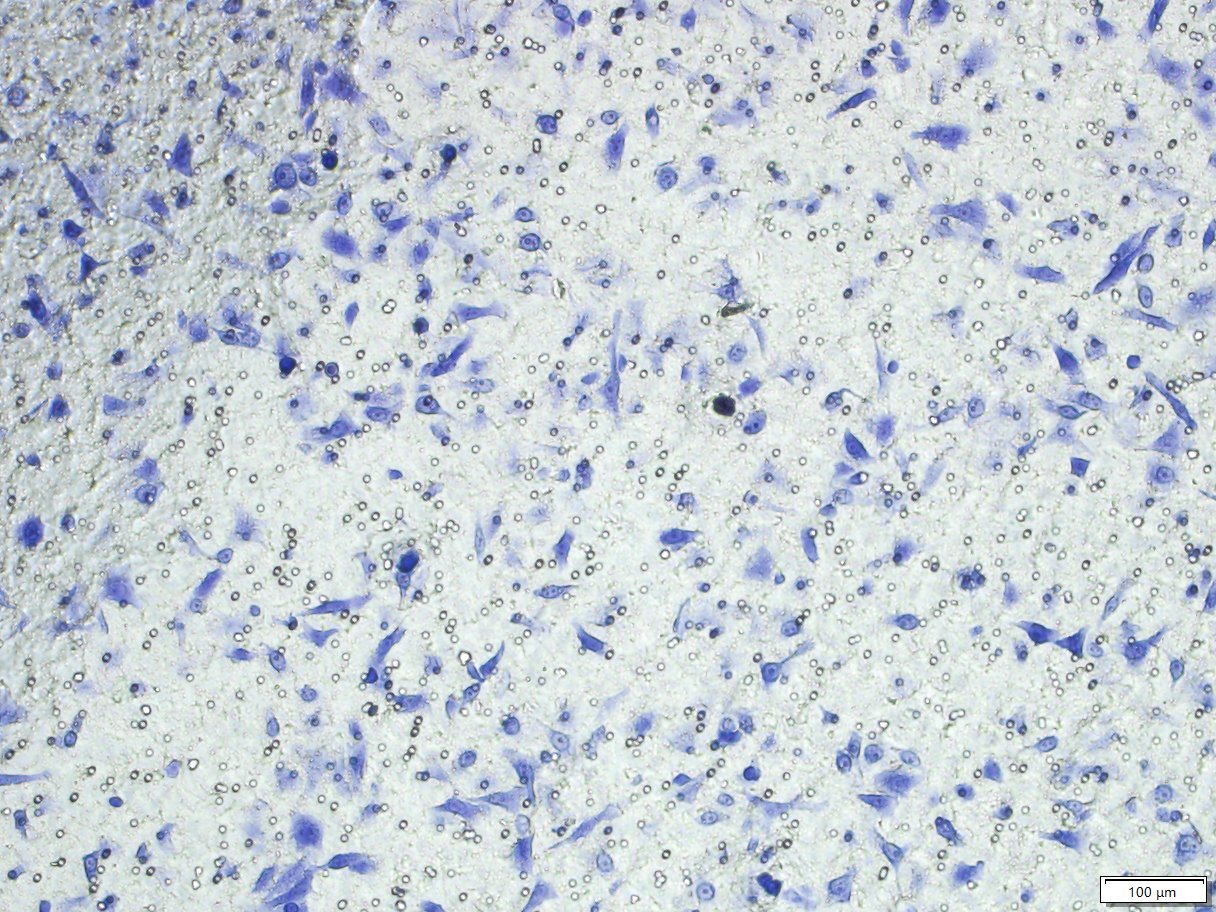

Supplement: Supplemental Information 4 [file peerj-cs-09-1651-s004.zip › Dataset 3/4+8.jpg]

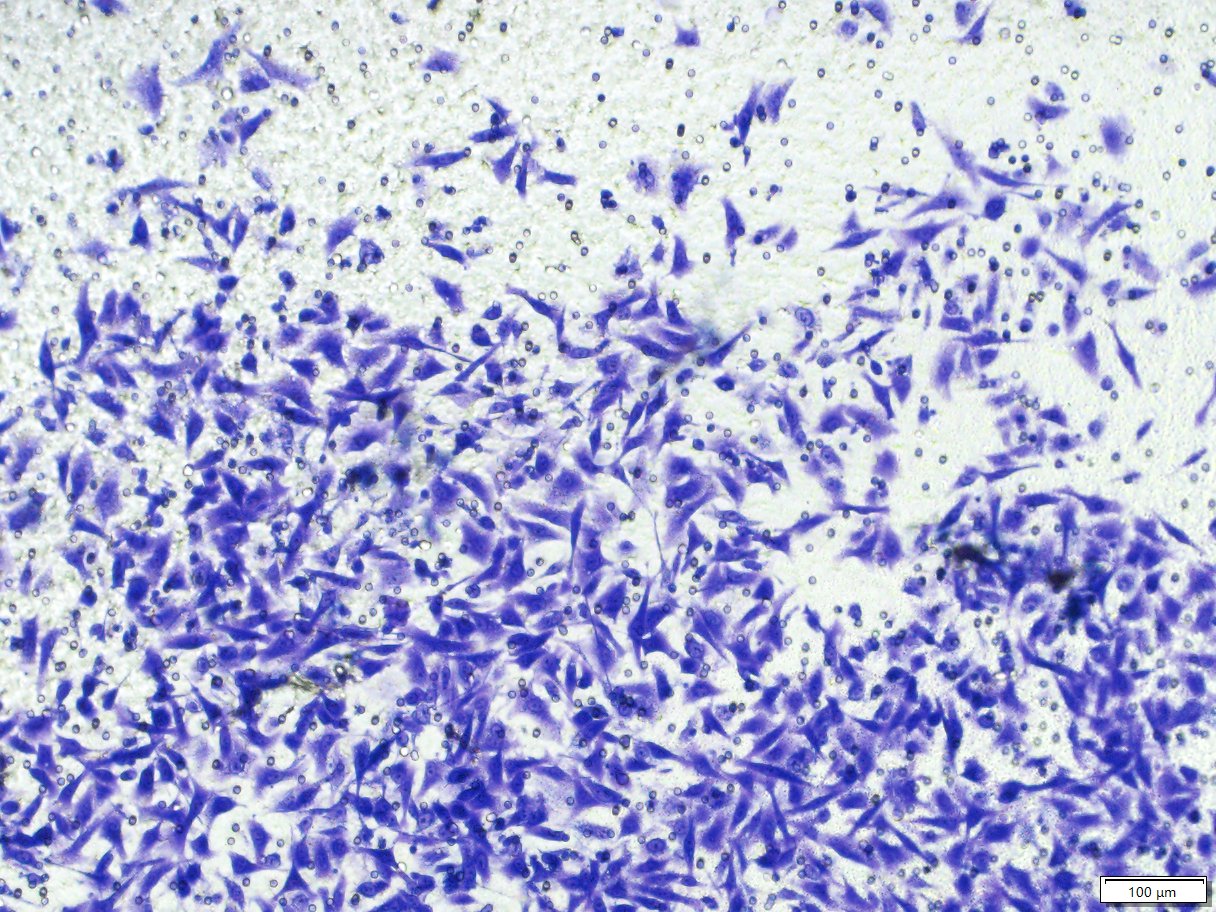

Supplement: Supplemental Information 5 [file peerj-cs-09-1651-s005.zip › Dataset 4/0+1.jpg]

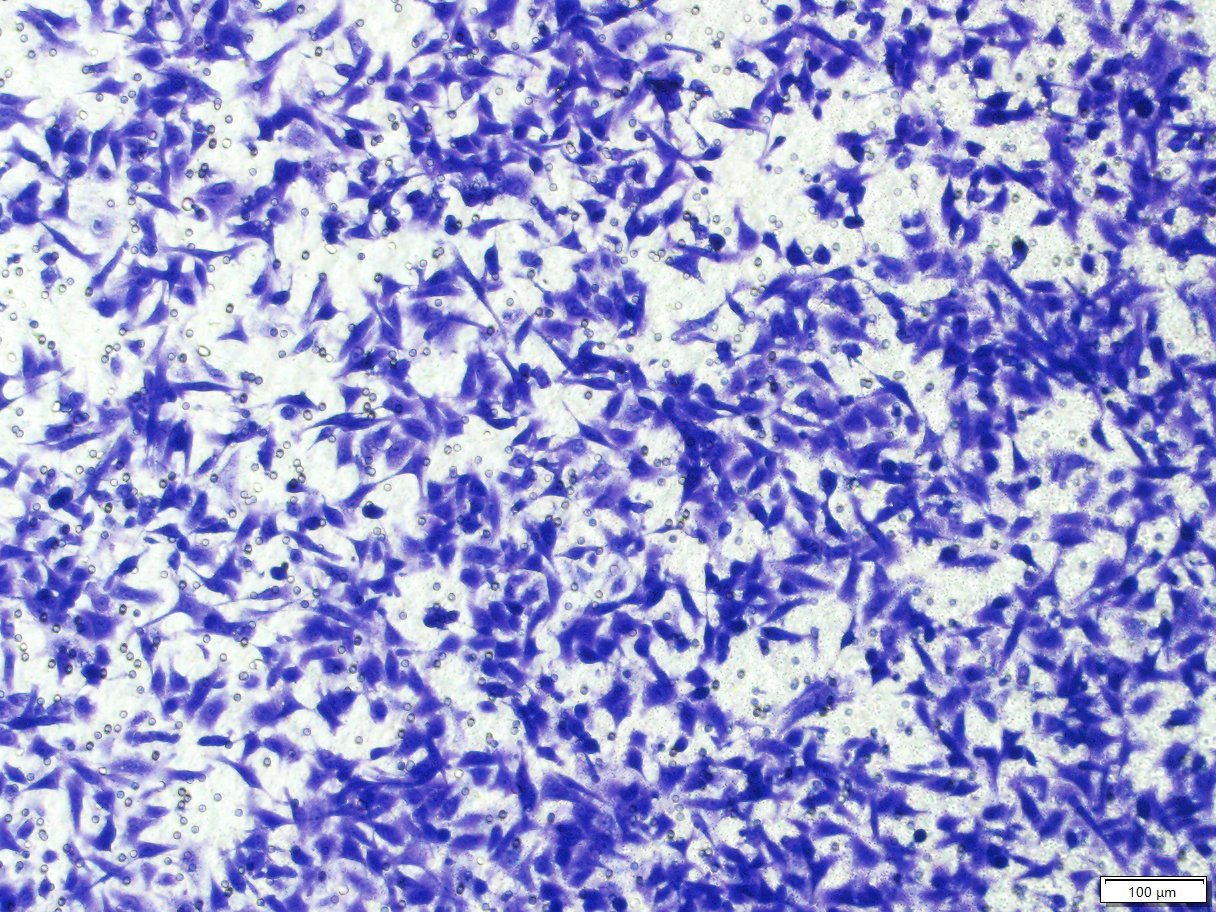

Supplement: Supplemental Information 5 [file peerj-cs-09-1651-s005.zip › Dataset 4/0+10.jpg]

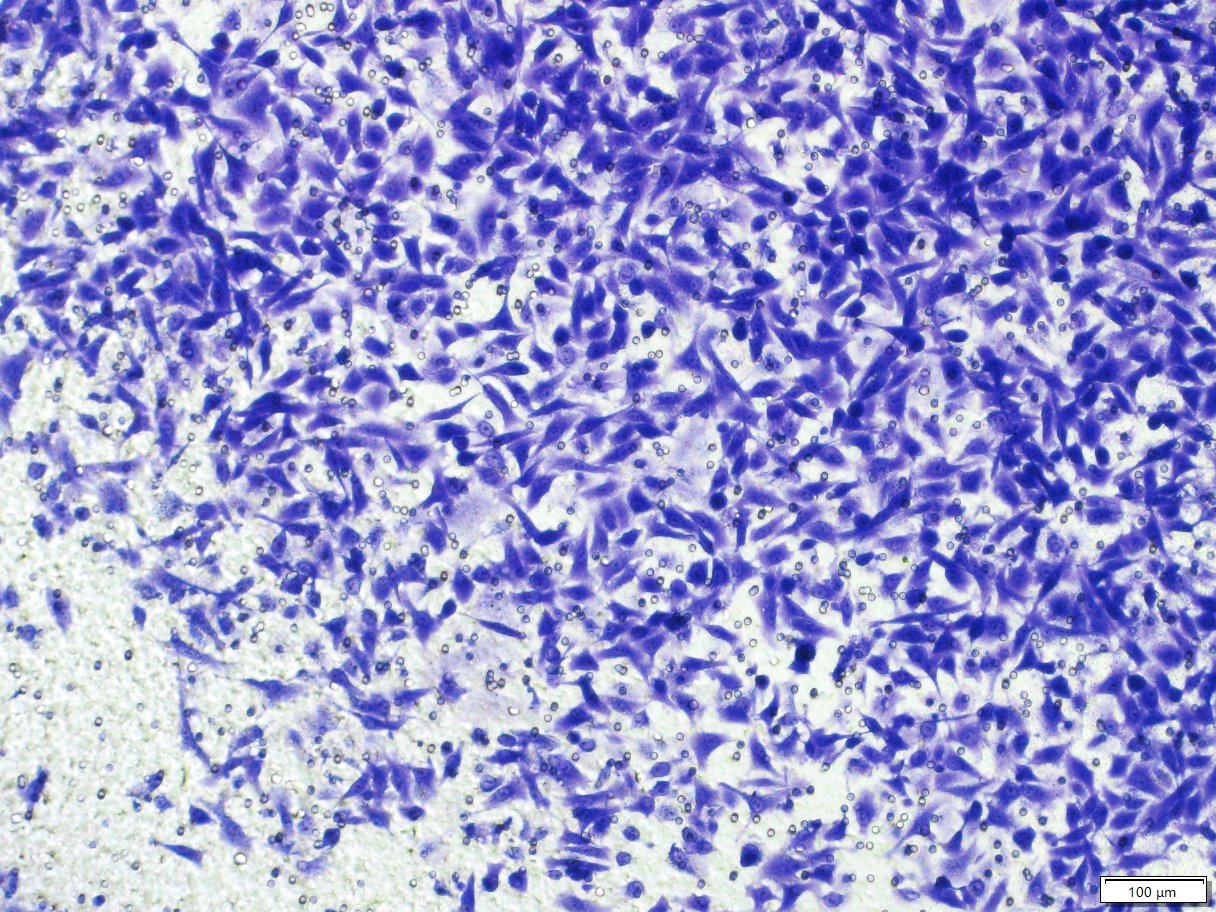

Supplement: Supplemental Information 5 [file peerj-cs-09-1651-s005.zip › Dataset 4/0+11.jpg]

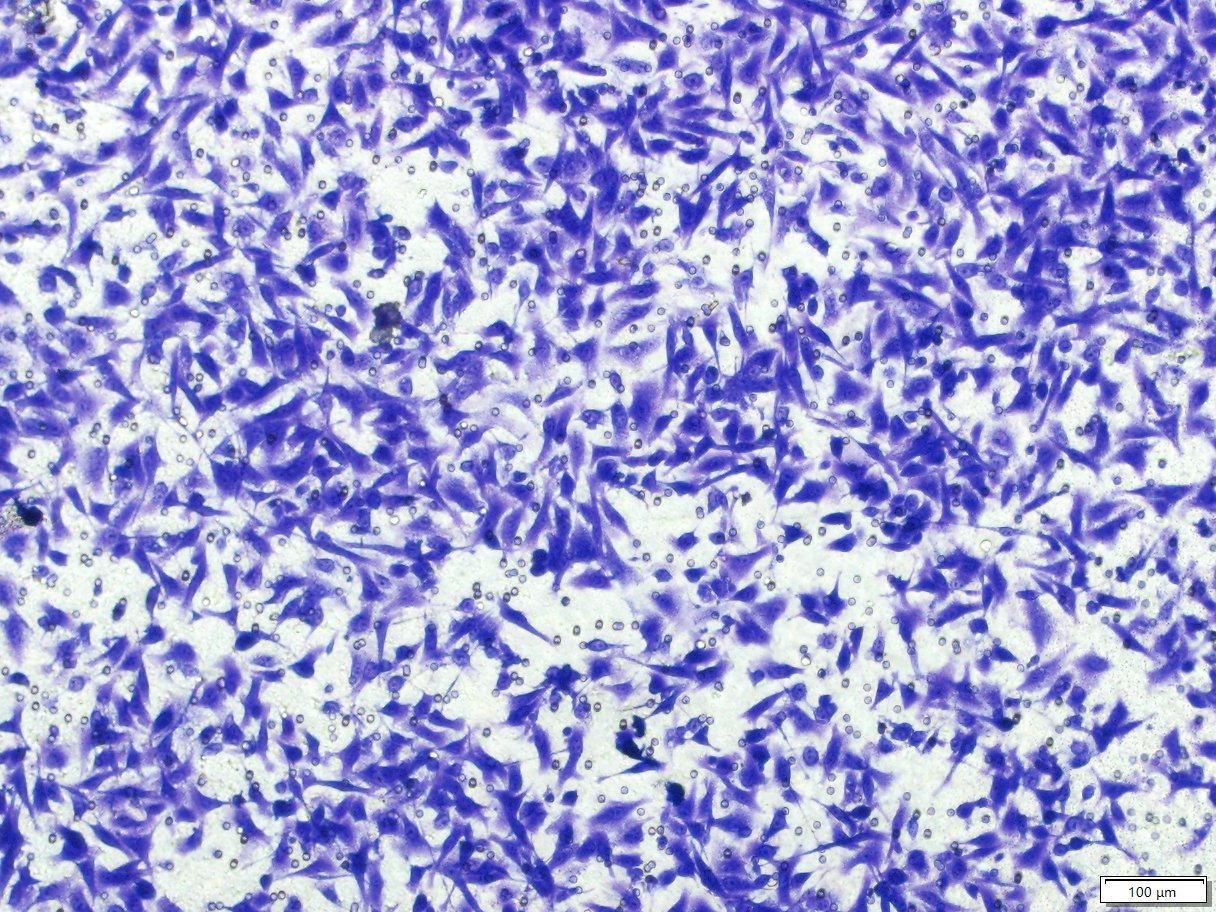

Supplement: Supplemental Information 5 [file peerj-cs-09-1651-s005.zip › Dataset 4/0+2.jpg]

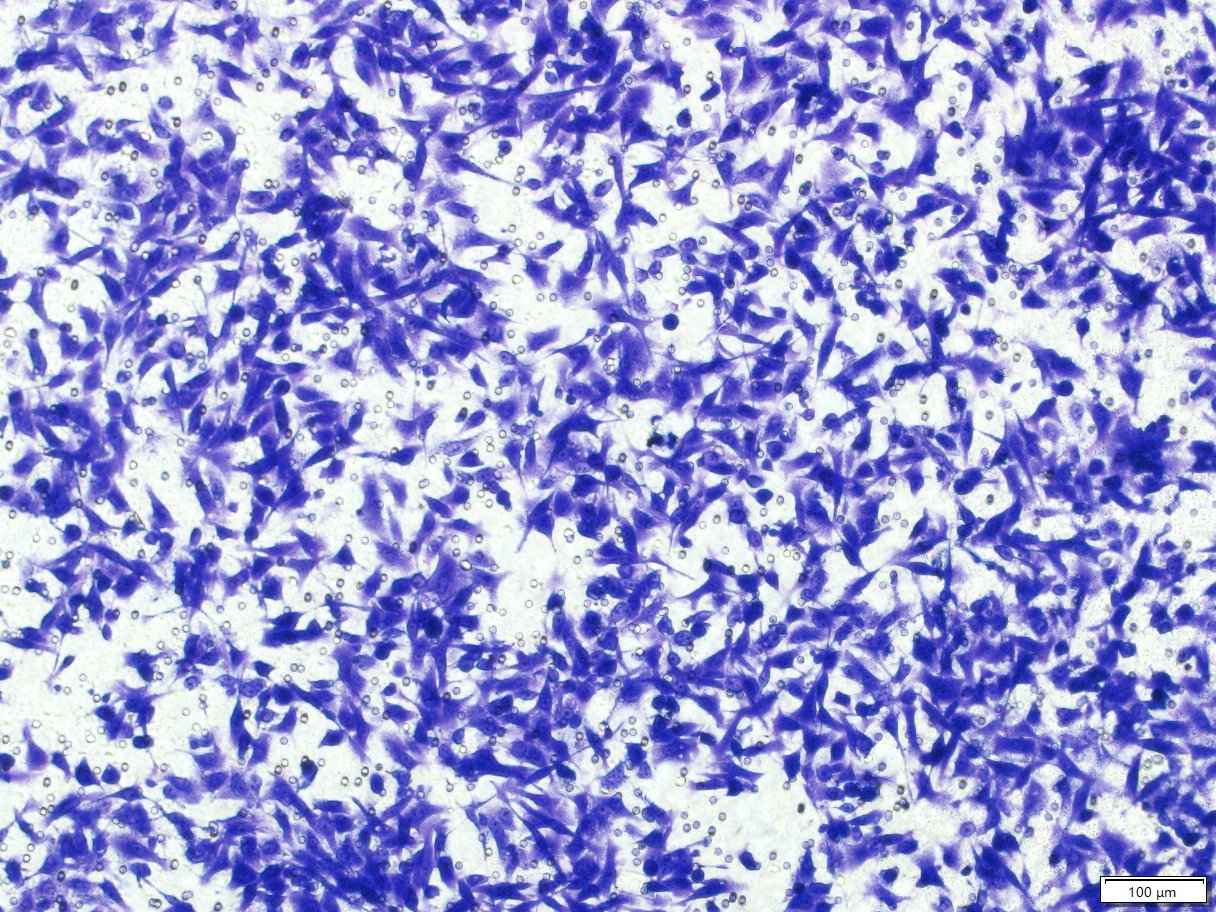

Supplement: Supplemental Information 5 [file peerj-cs-09-1651-s005.zip › Dataset 4/0+3.jpg]

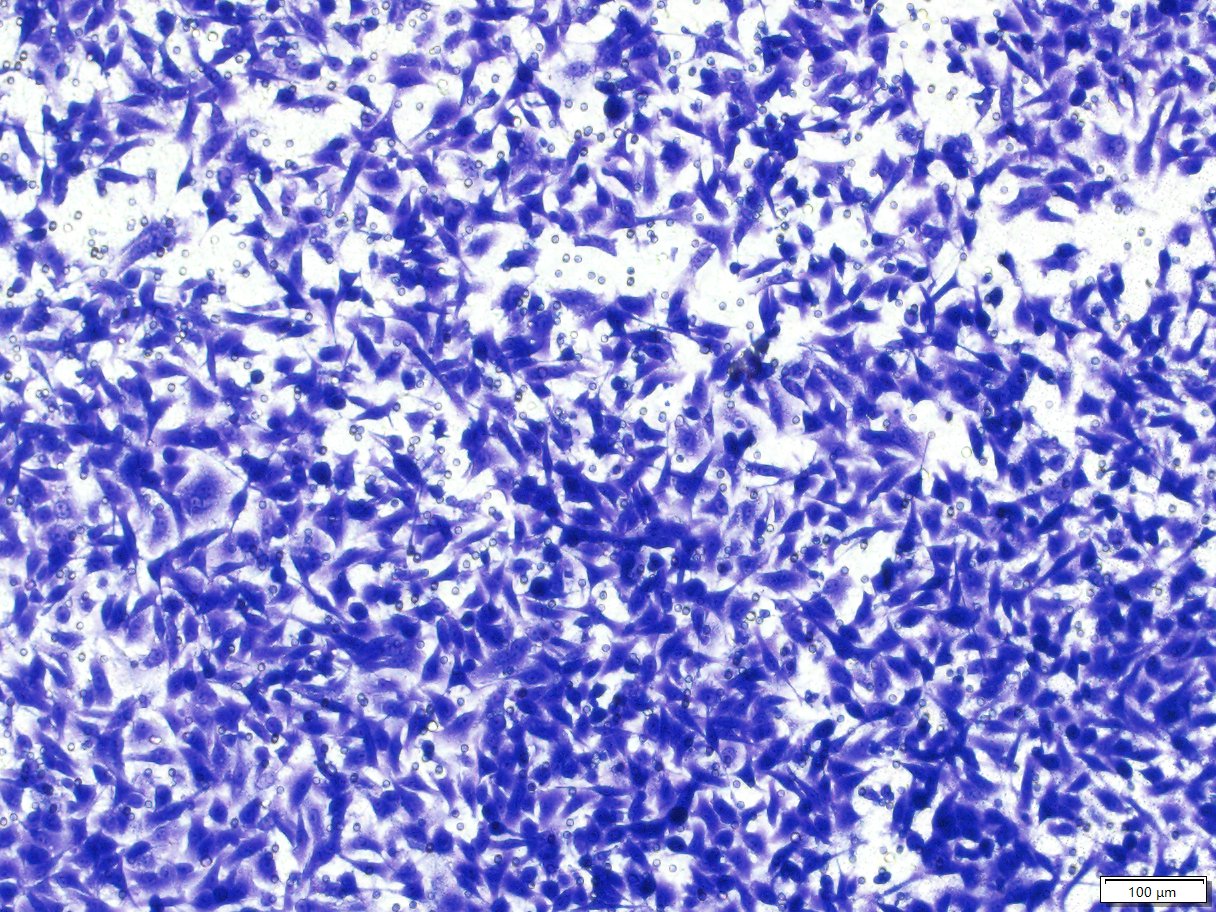

Supplement: Supplemental Information 5 [file peerj-cs-09-1651-s005.zip › Dataset 4/0+4.jpg]

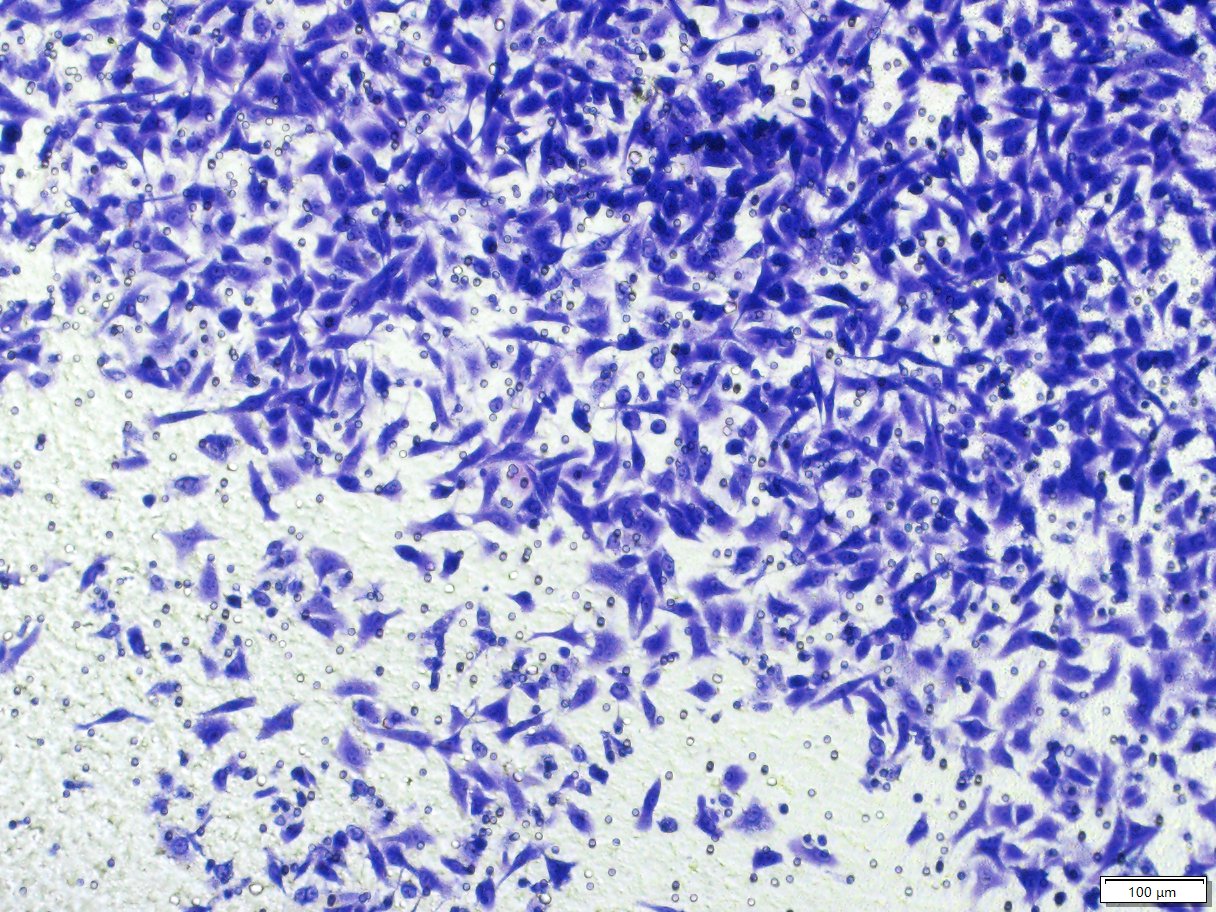

Supplement: Supplemental Information 5 [file peerj-cs-09-1651-s005.zip › Dataset 4/0+5.jpg]

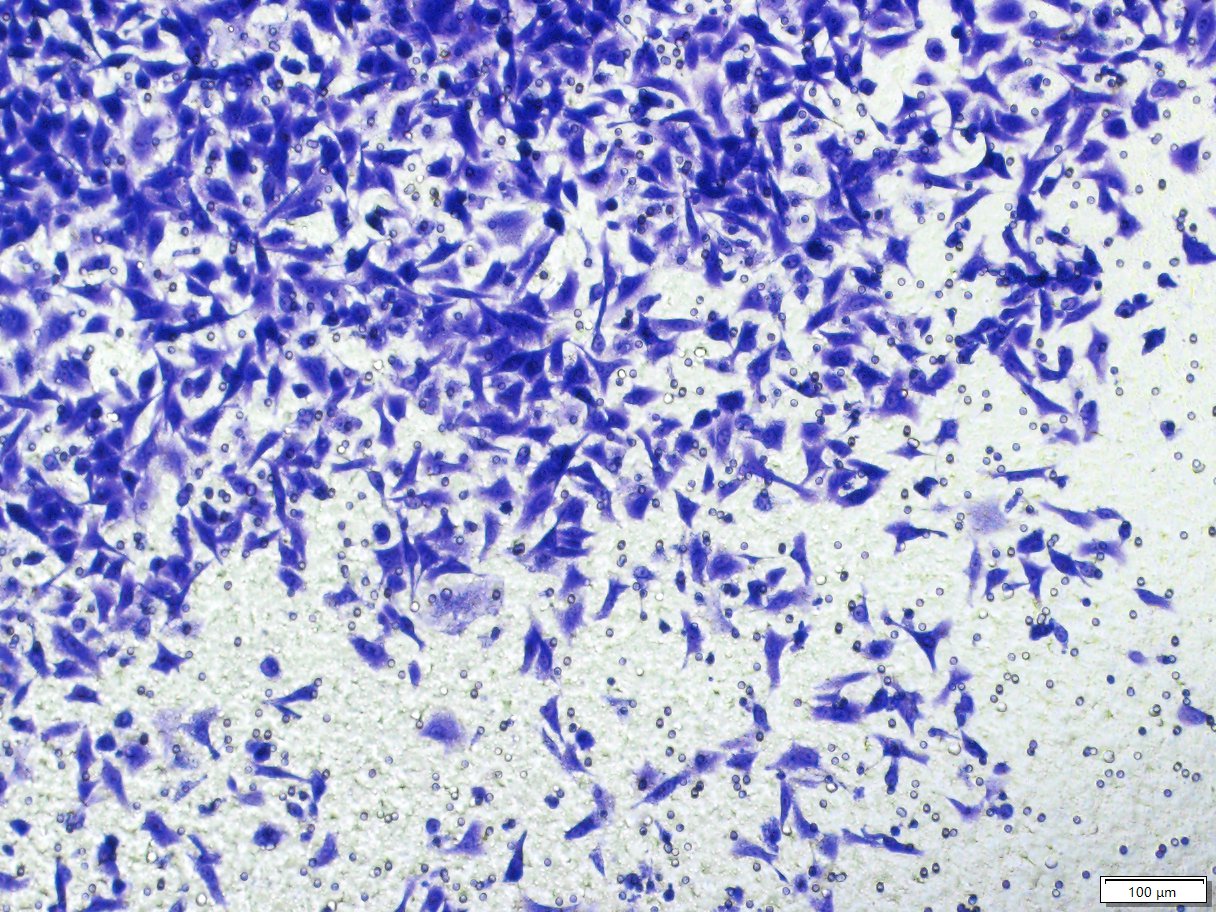

Supplement: Supplemental Information 5 [file peerj-cs-09-1651-s005.zip › Dataset 4/0+6.jpg]

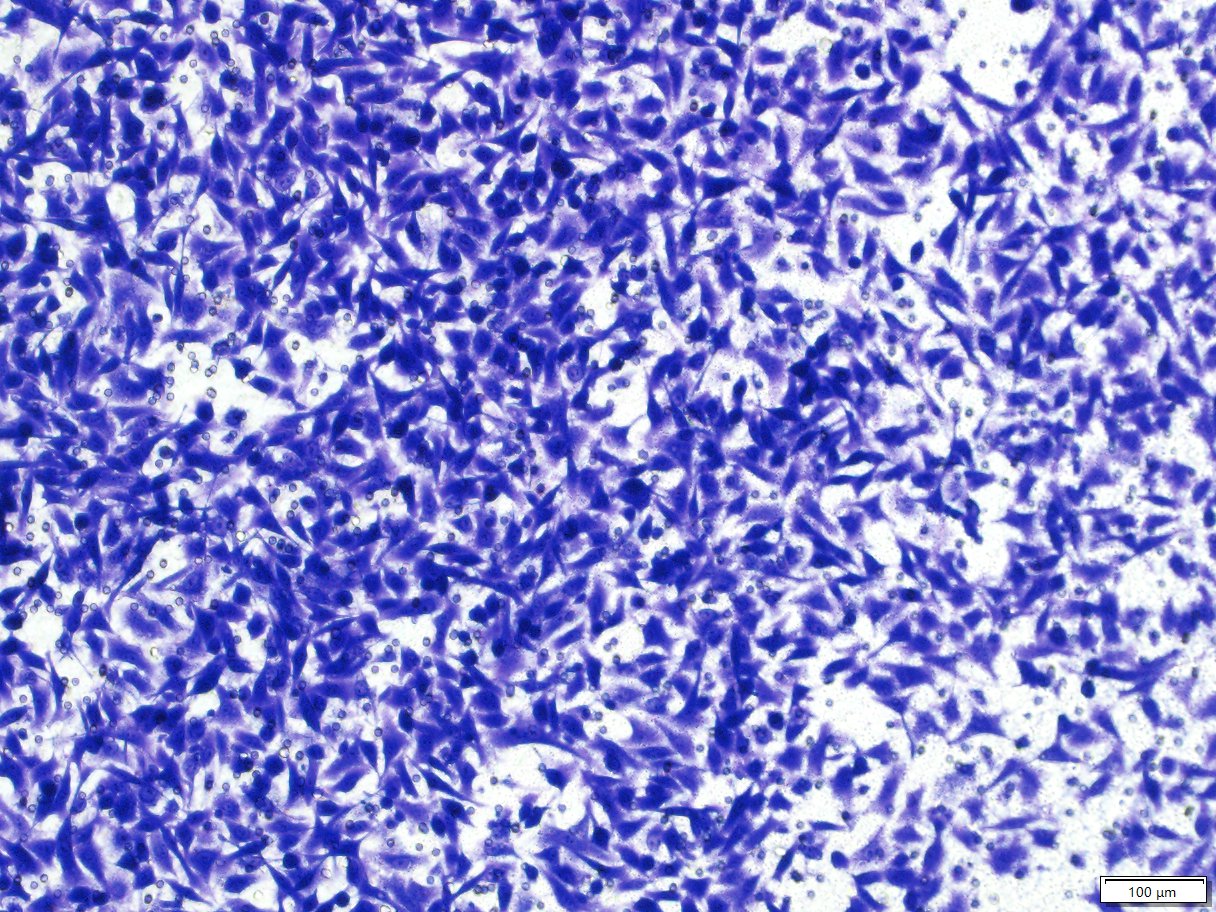

Supplement: Supplemental Information 5 [file peerj-cs-09-1651-s005.zip › Dataset 4/0+7.jpg]

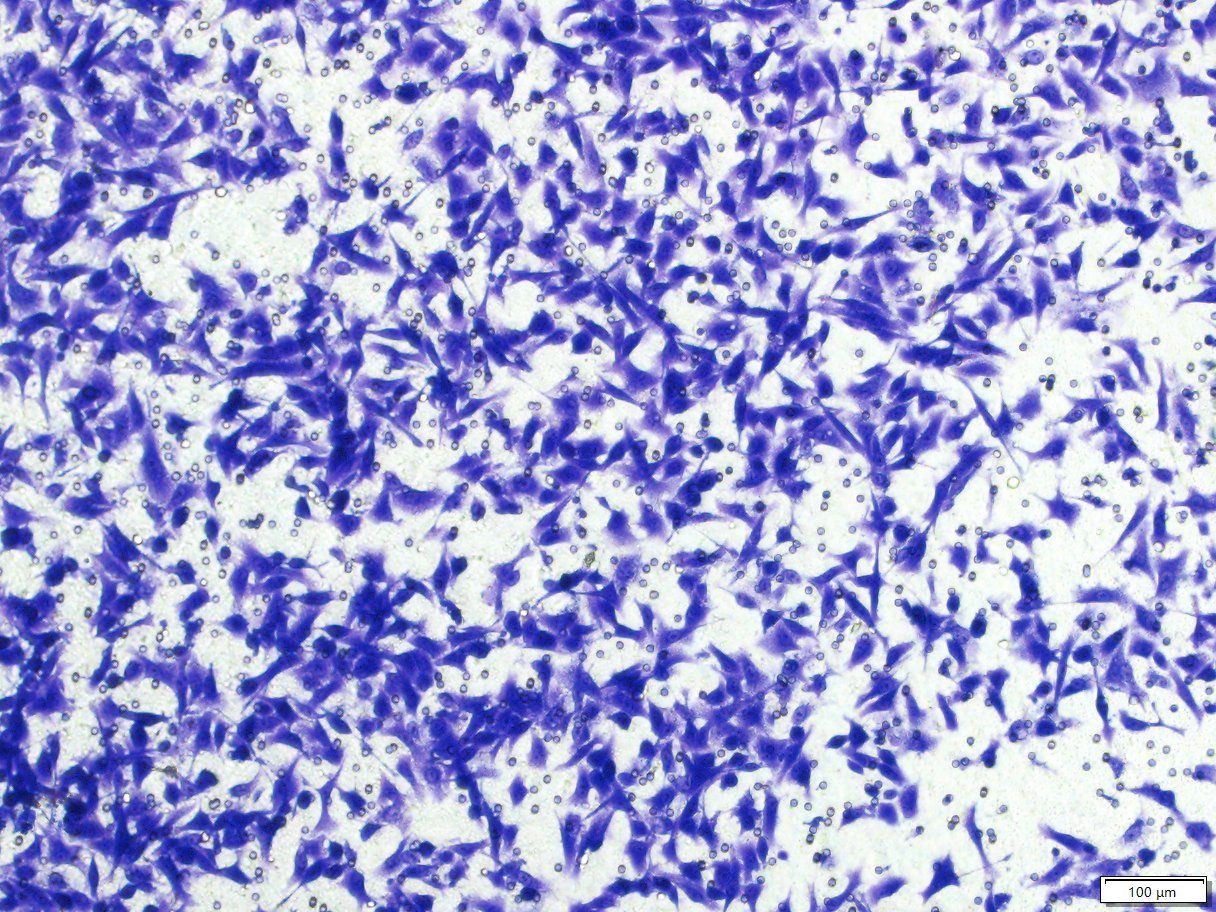

Supplement: Supplemental Information 5 [file peerj-cs-09-1651-s005.zip › Dataset 4/0+8.jpg]

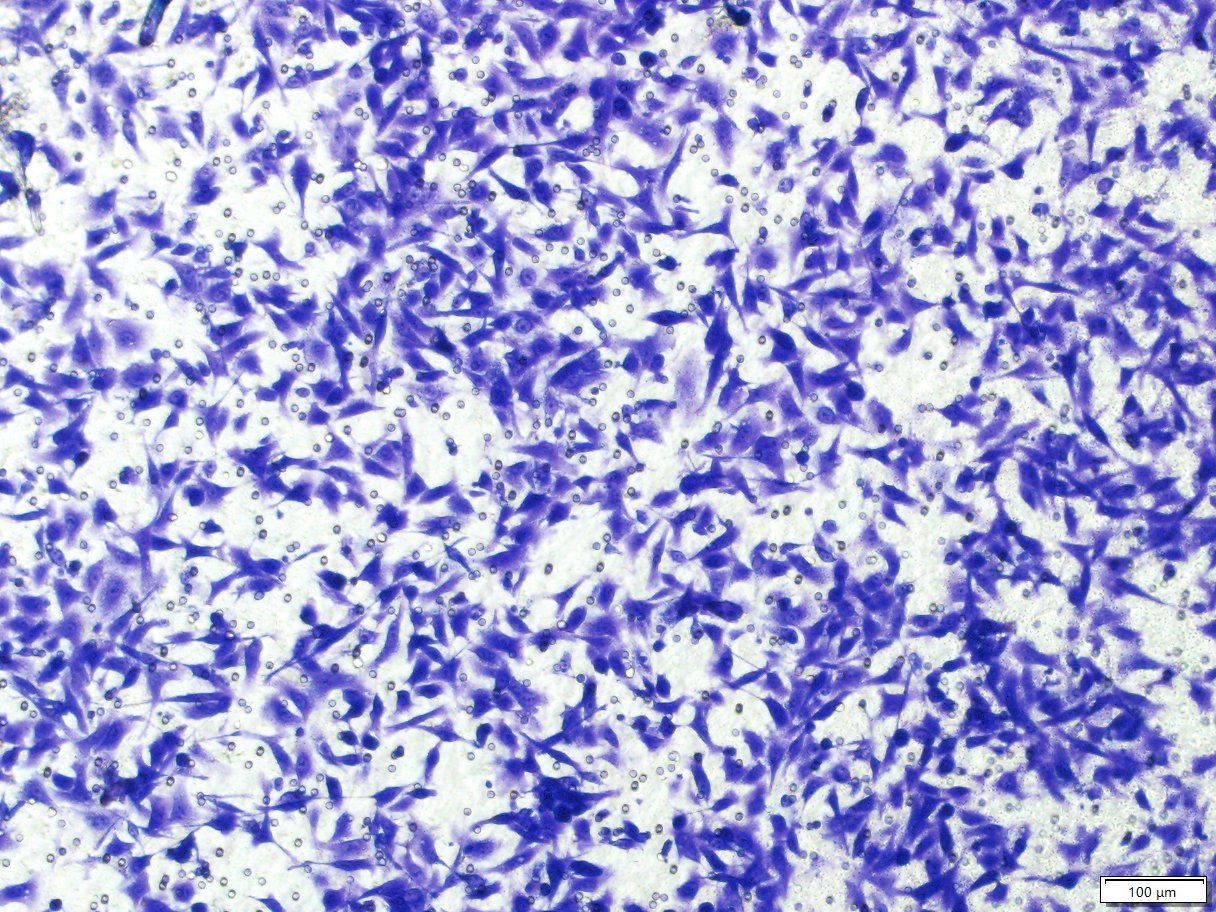

Supplement: Supplemental Information 5 [file peerj-cs-09-1651-s005.zip › Dataset 4/0+9.jpg]

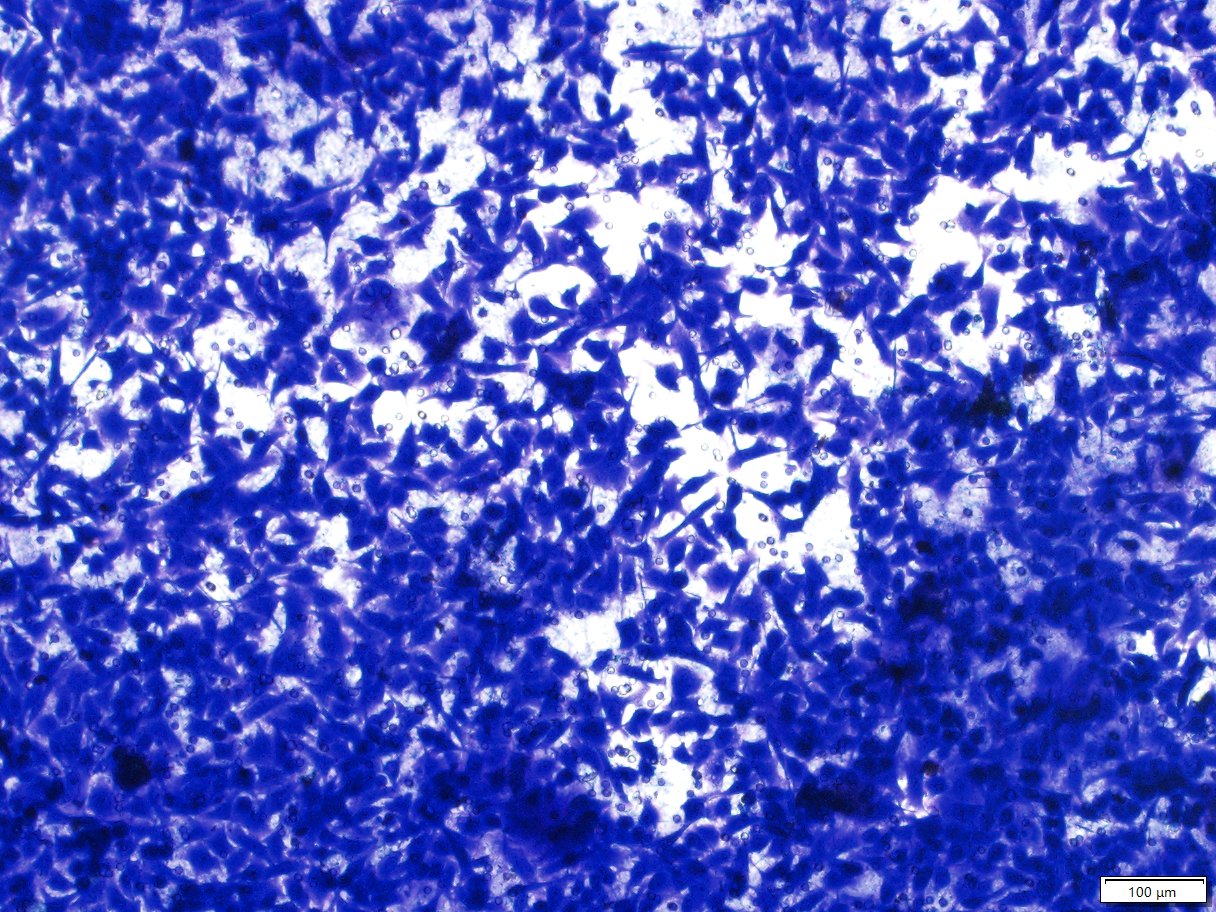

Supplement: Supplemental Information 5 [file peerj-cs-09-1651-s005.zip › Dataset 4/0-1.jpg]

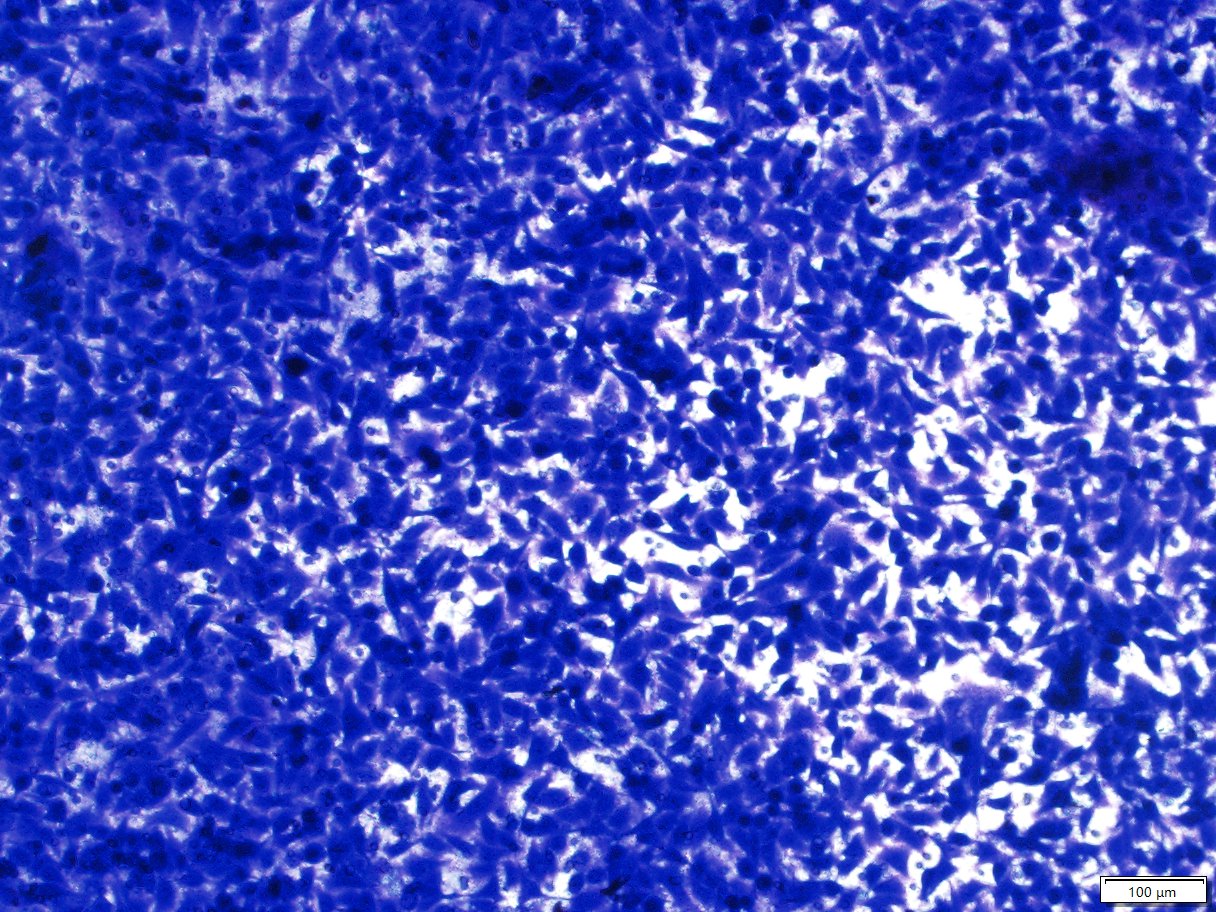

Supplement: Supplemental Information 5 [file peerj-cs-09-1651-s005.zip › Dataset 4/0-10.jpg]

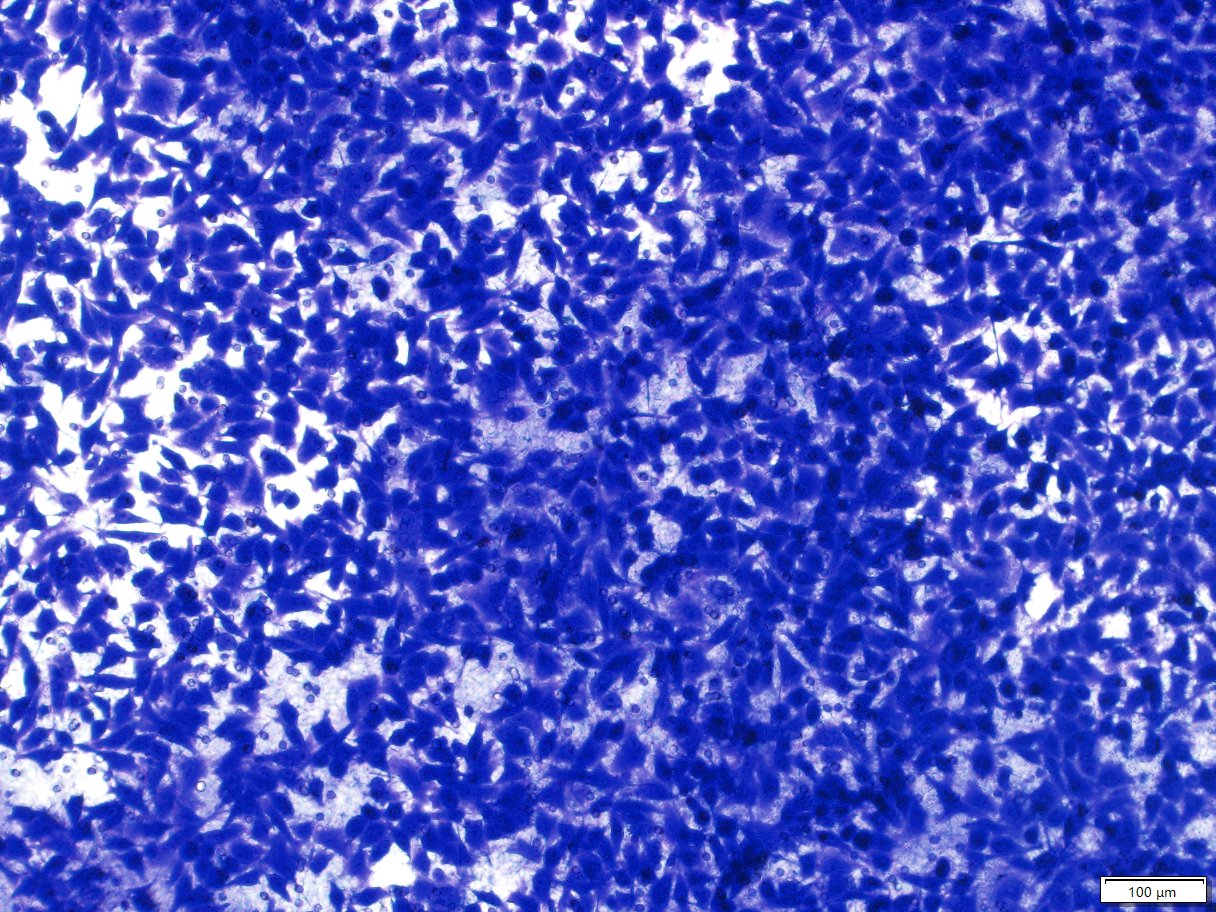

Supplement: Supplemental Information 5 [file peerj-cs-09-1651-s005.zip › Dataset 4/0-11.jpg]

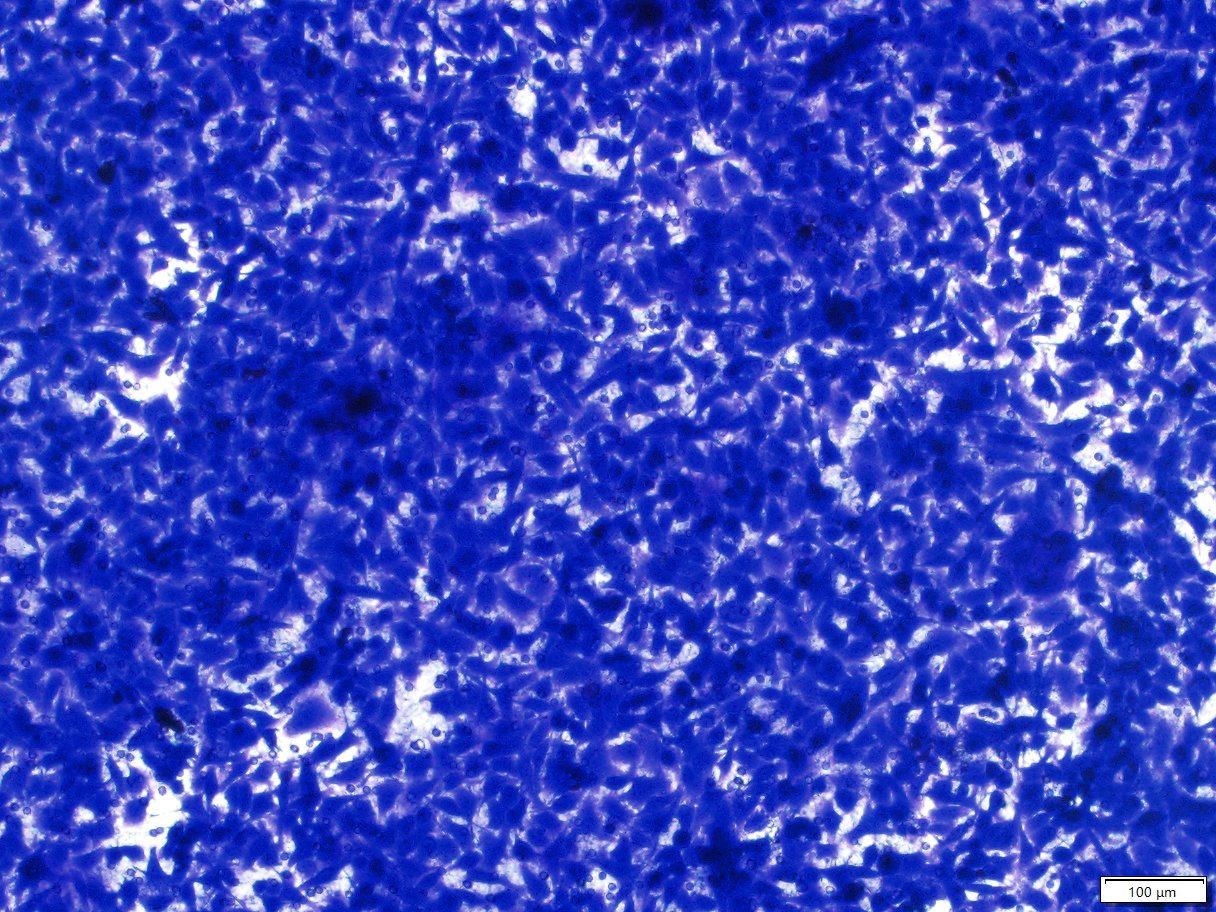

Supplement: Supplemental Information 5 [file peerj-cs-09-1651-s005.zip › Dataset 4/0-2.jpg]

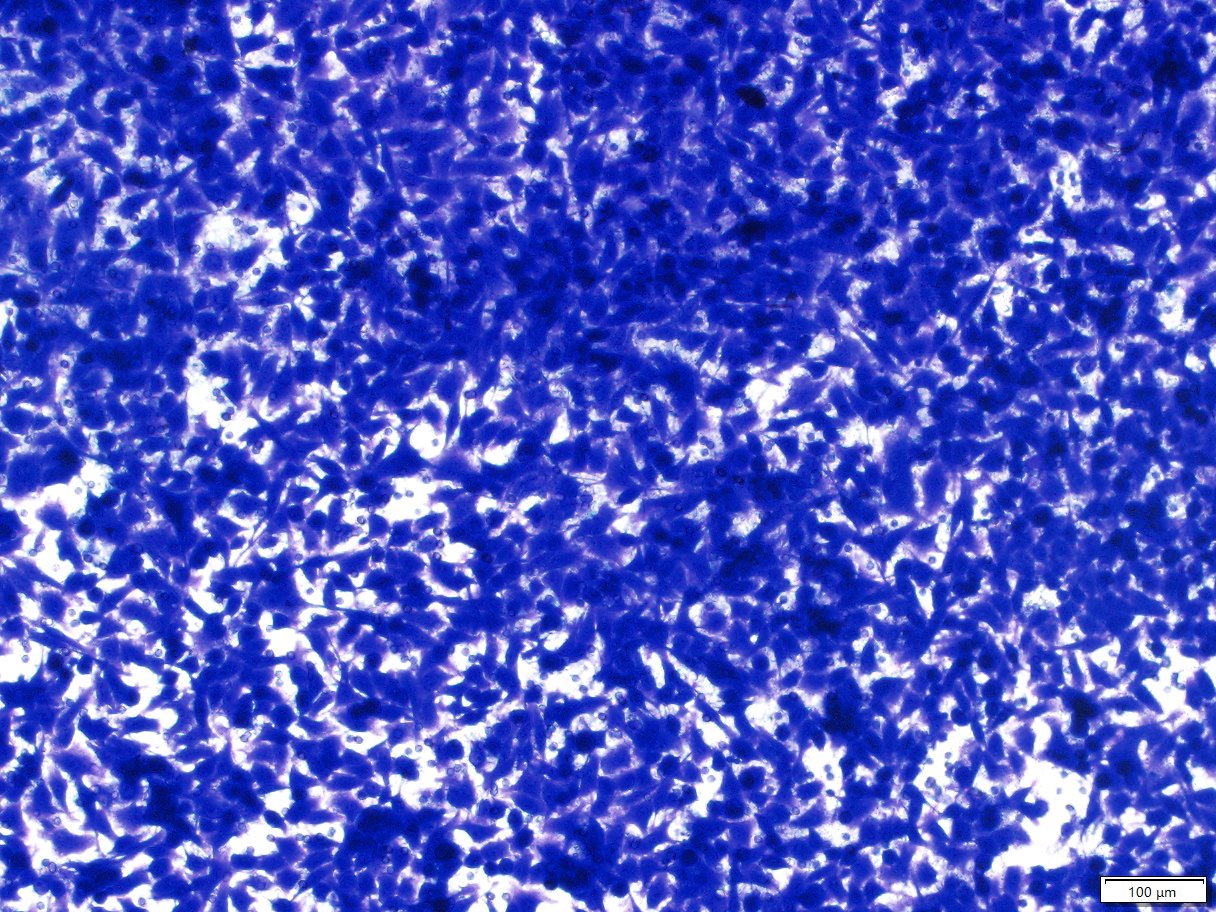

Supplement: Supplemental Information 5 [file peerj-cs-09-1651-s005.zip › Dataset 4/0-3.jpg]

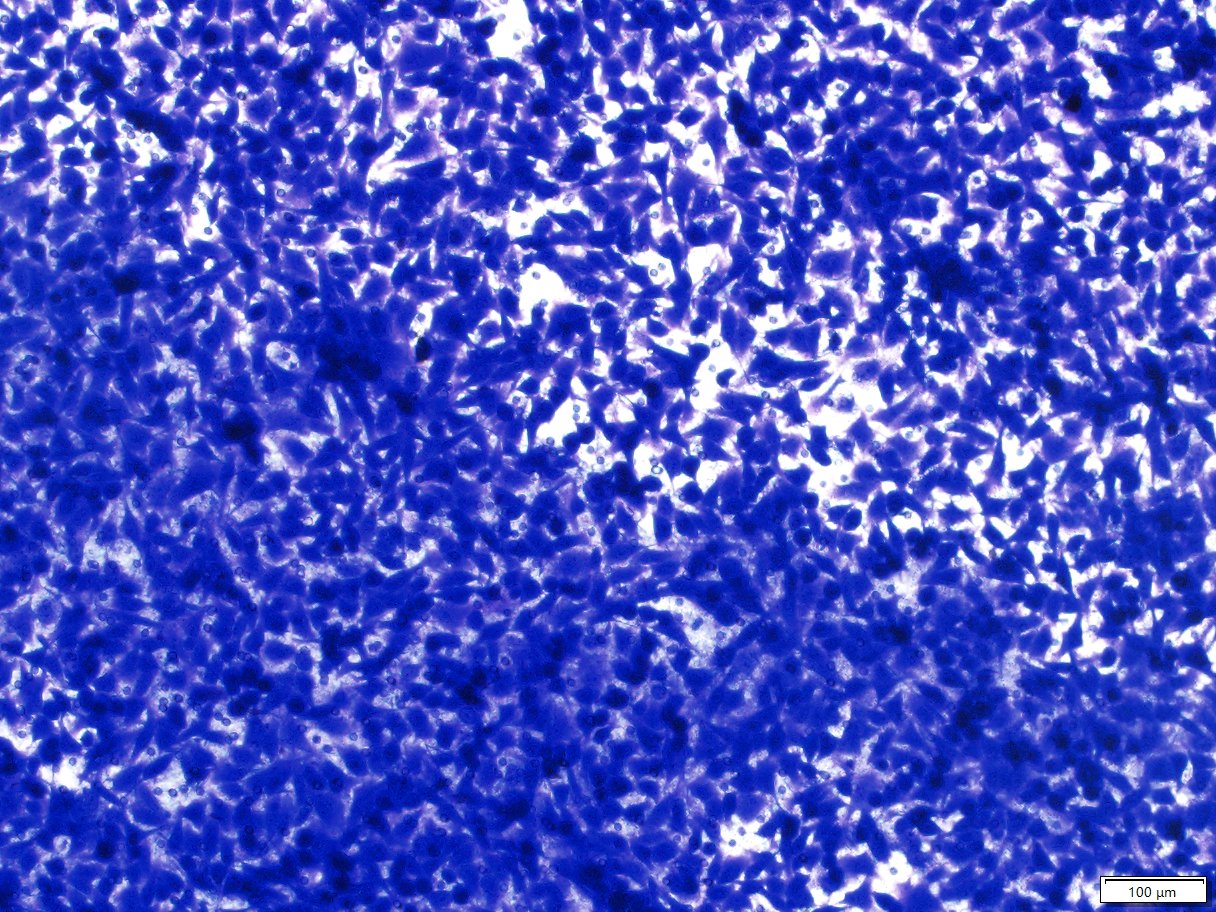

Supplement: Supplemental Information 5 [file peerj-cs-09-1651-s005.zip › Dataset 4/0-4.jpg]

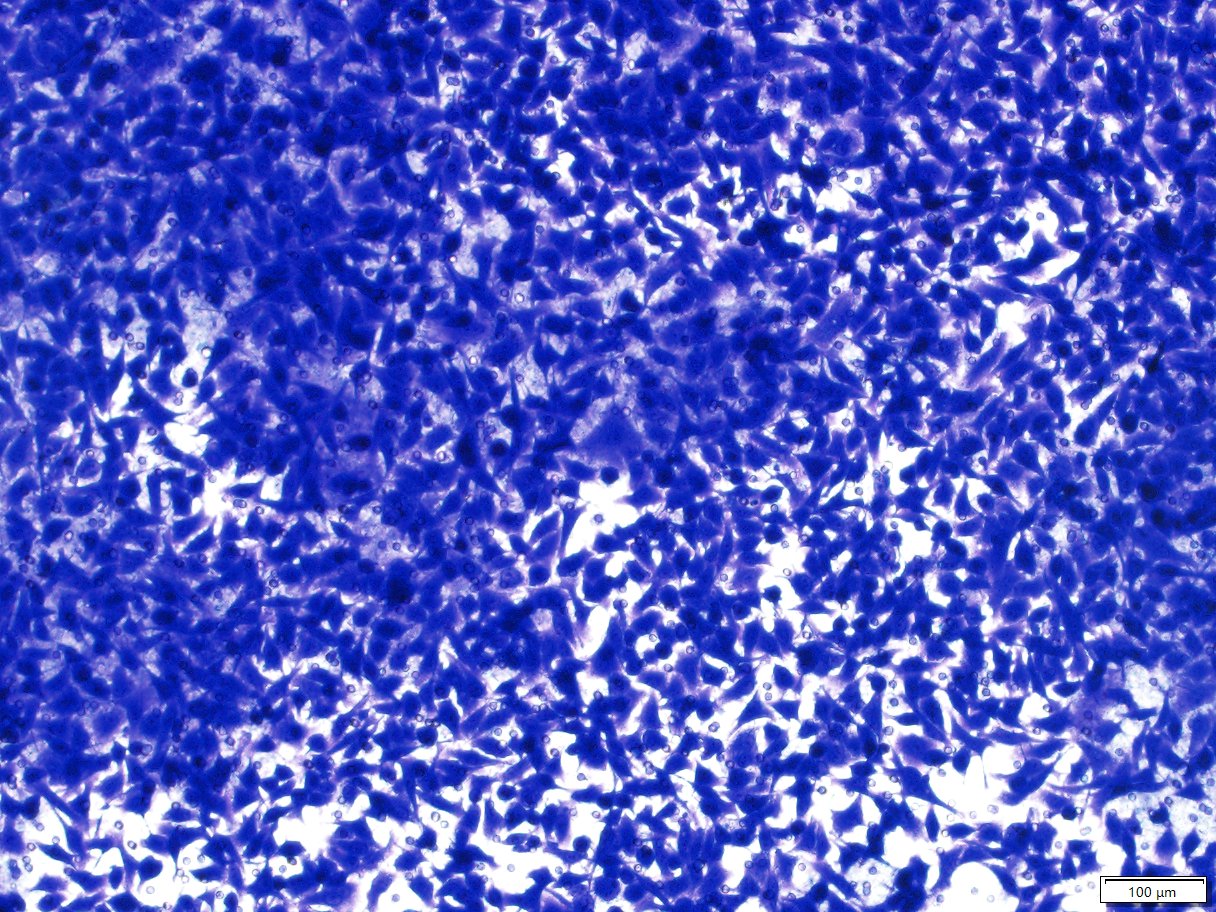

Supplement: Supplemental Information 5 [file peerj-cs-09-1651-s005.zip › Dataset 4/0-5.jpg]

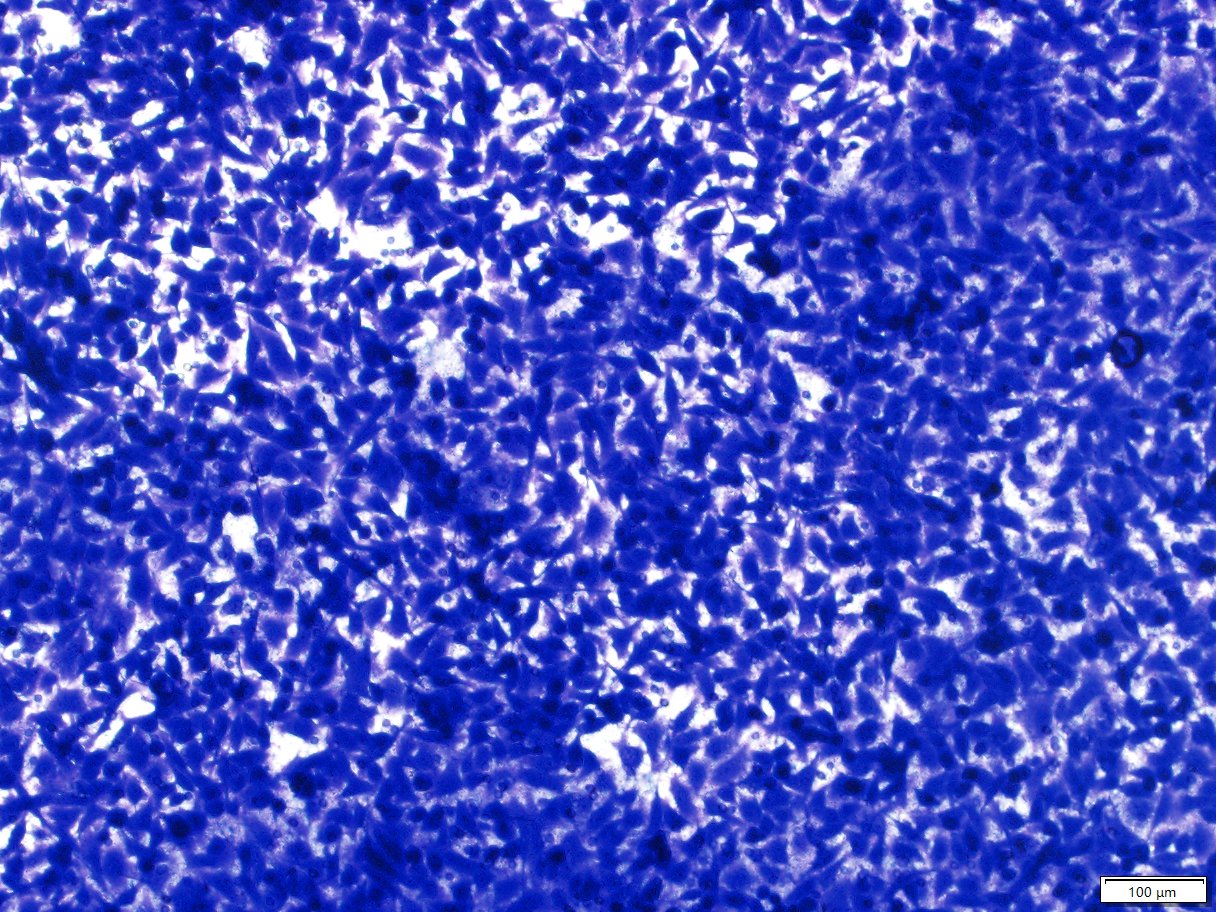

Supplement: Supplemental Information 5 [file peerj-cs-09-1651-s005.zip › Dataset 4/0-6.jpg]

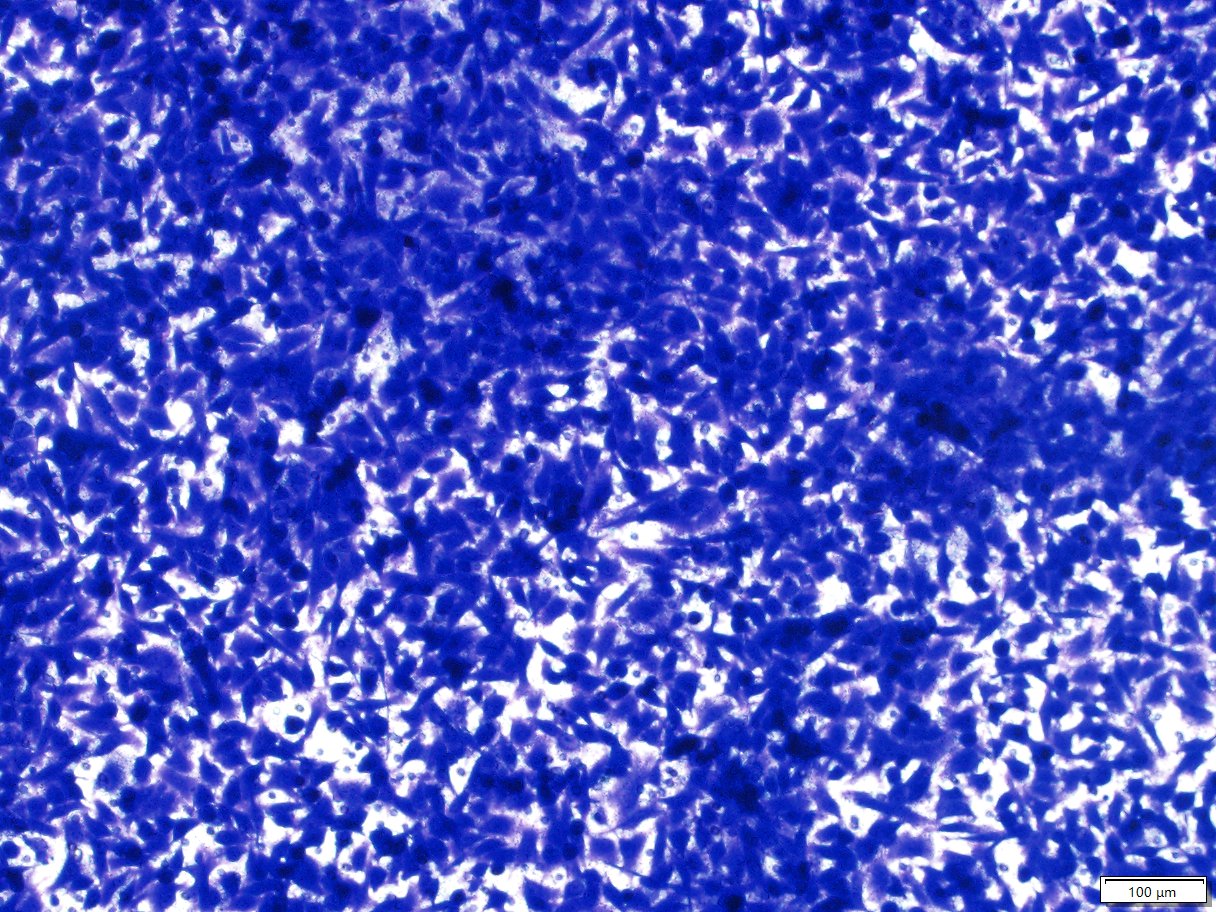

Supplement: Supplemental Information 5 [file peerj-cs-09-1651-s005.zip › Dataset 4/0-7.jpg]

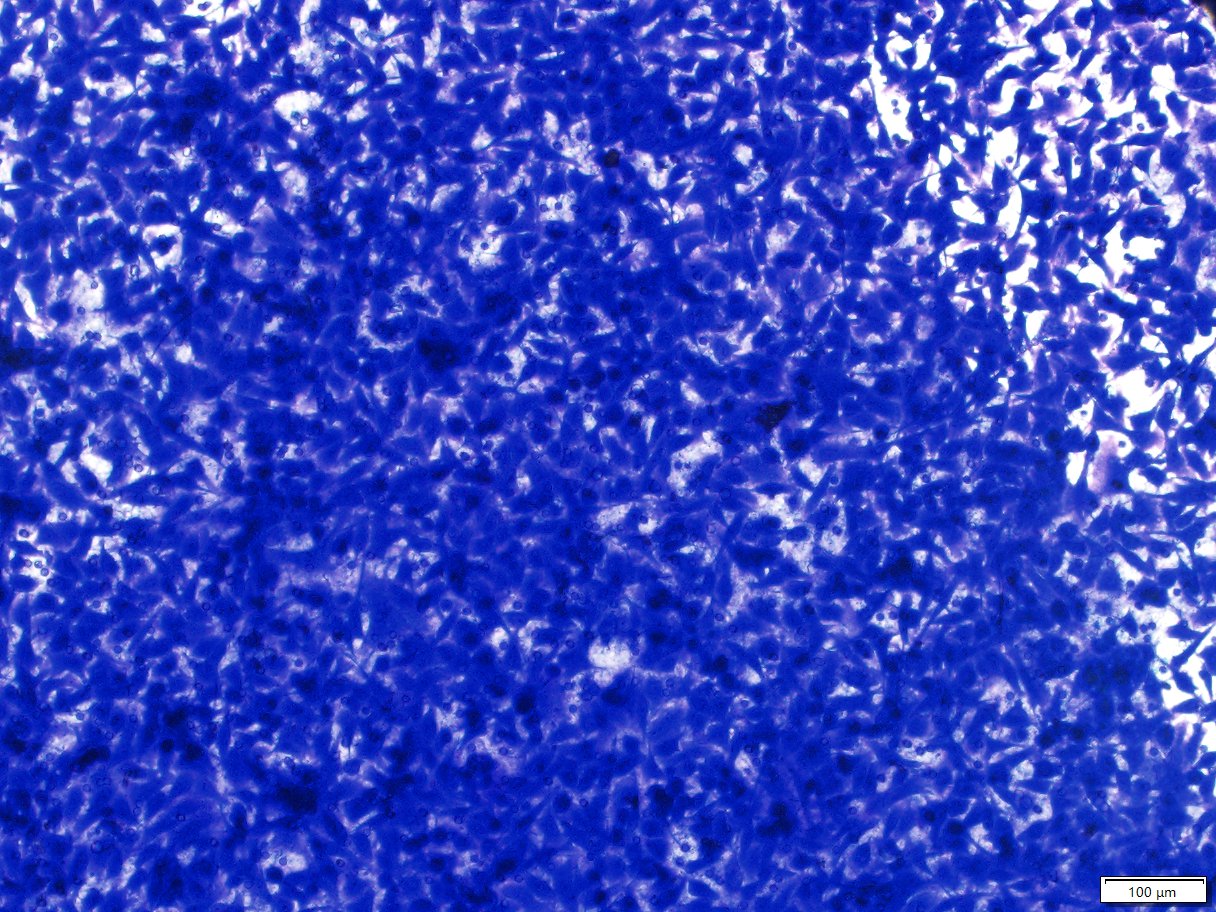

Supplement: Supplemental Information 5 [file peerj-cs-09-1651-s005.zip › Dataset 4/0-8.jpg]

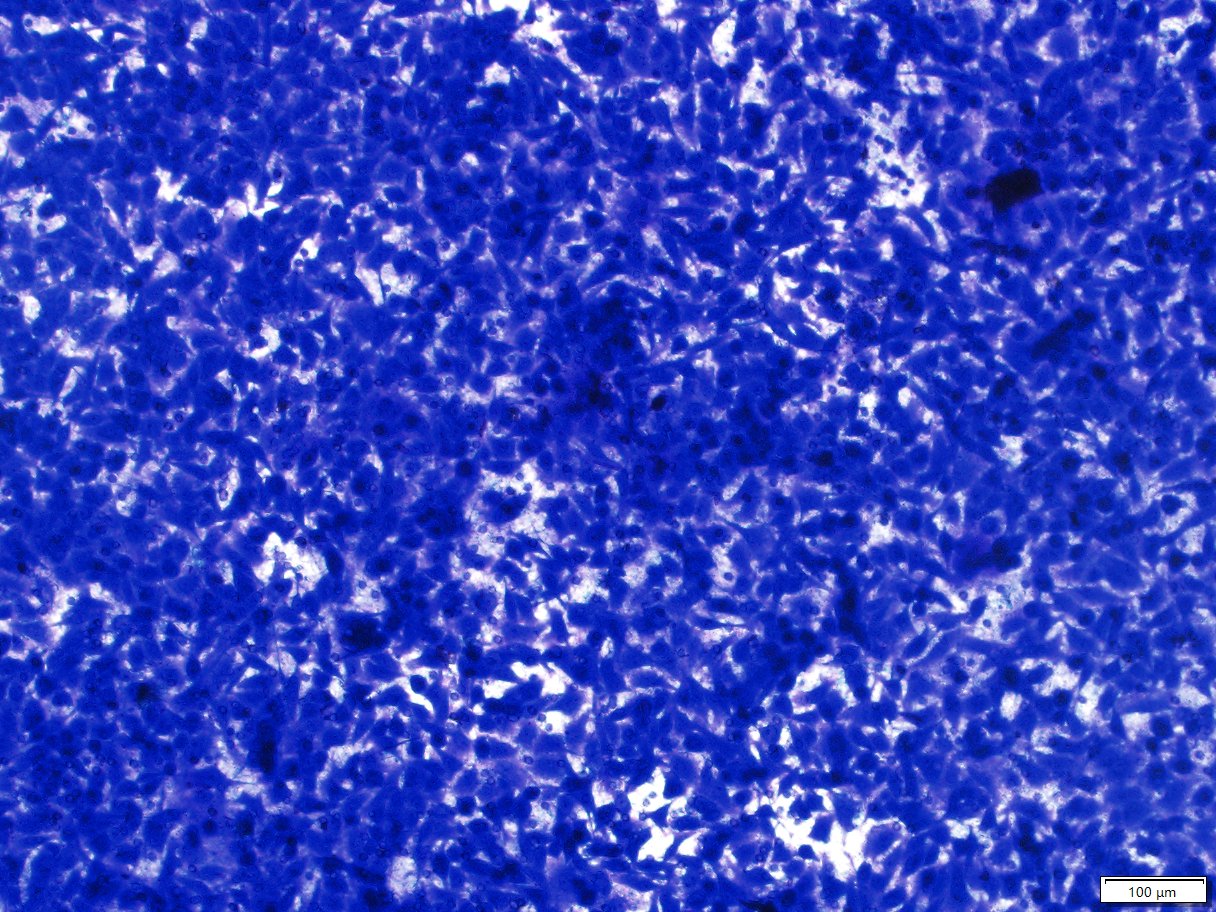

Supplement: Supplemental Information 5 [file peerj-cs-09-1651-s005.zip › Dataset 4/0-9.jpg]

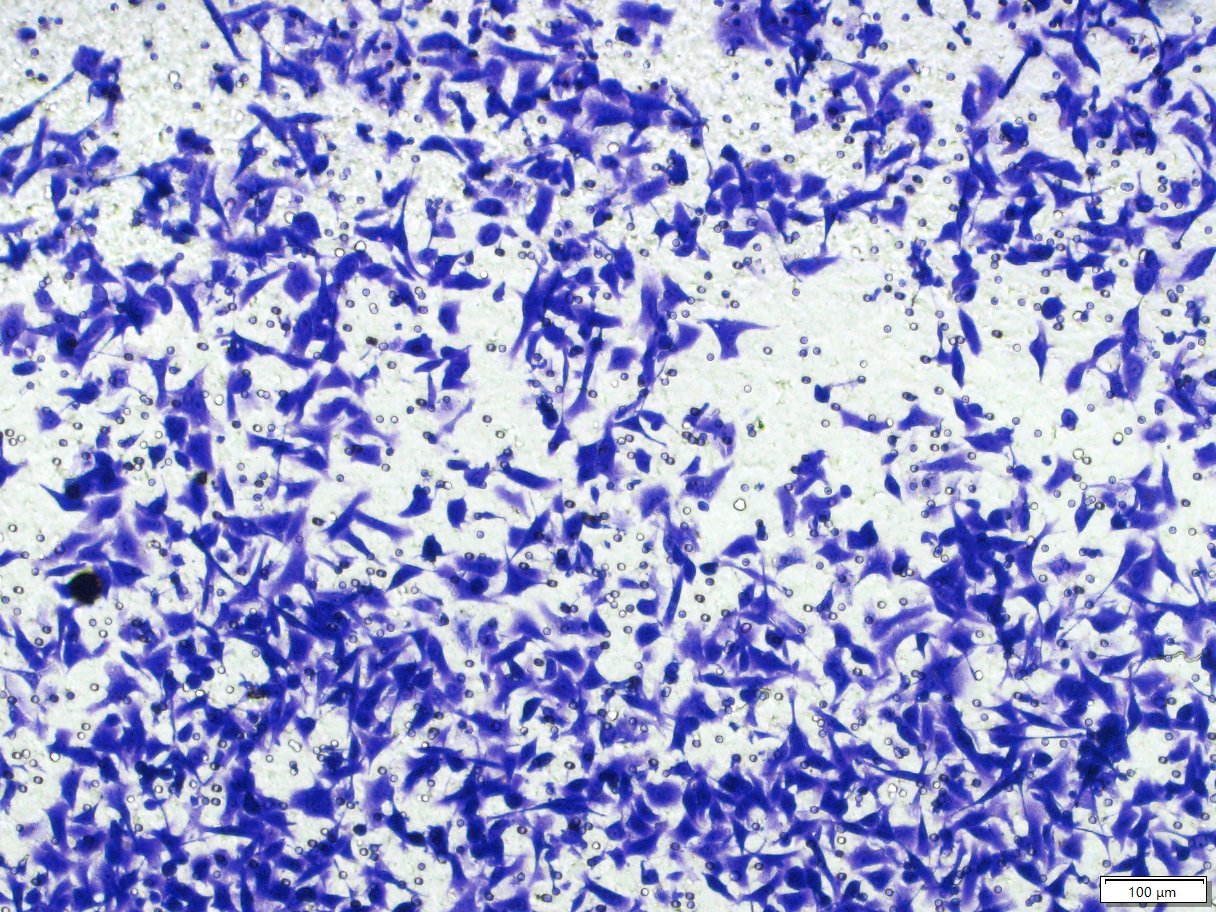

Supplement: Supplemental Information 5 [file peerj-cs-09-1651-s005.zip › Dataset 4/1+1.jpg]

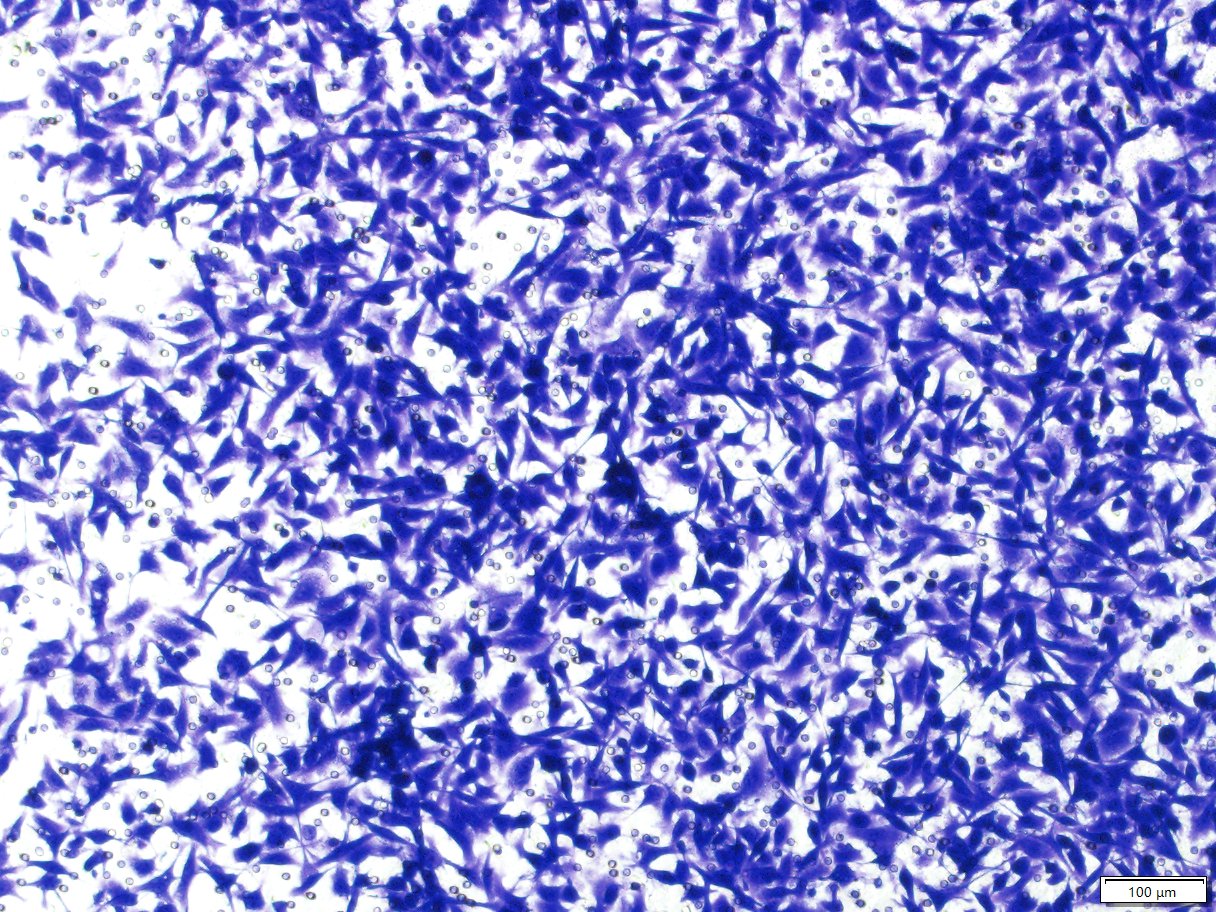

Supplement: Supplemental Information 5 [file peerj-cs-09-1651-s005.zip › Dataset 4/1+10.jpg]

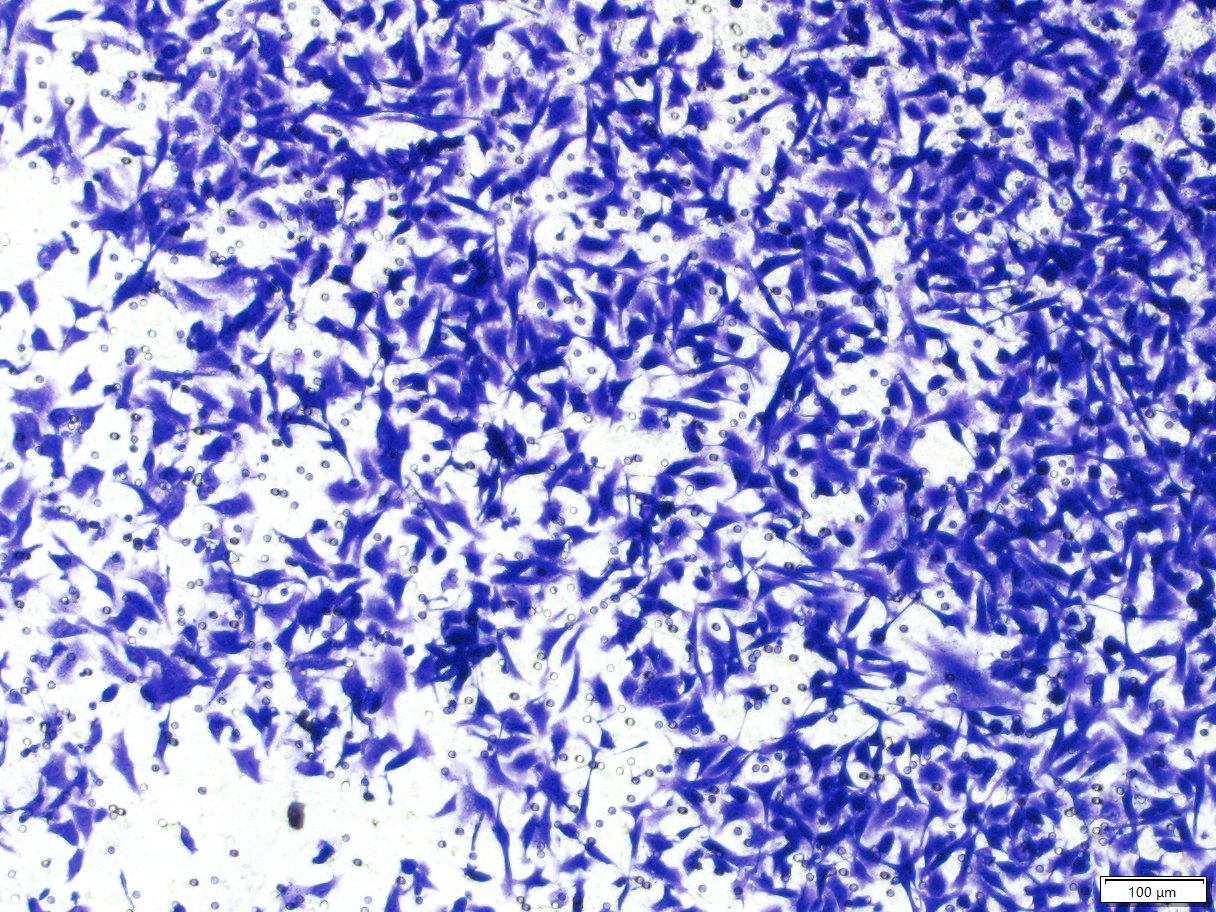

Supplement: Supplemental Information 5 [file peerj-cs-09-1651-s005.zip › Dataset 4/1+11.jpg]

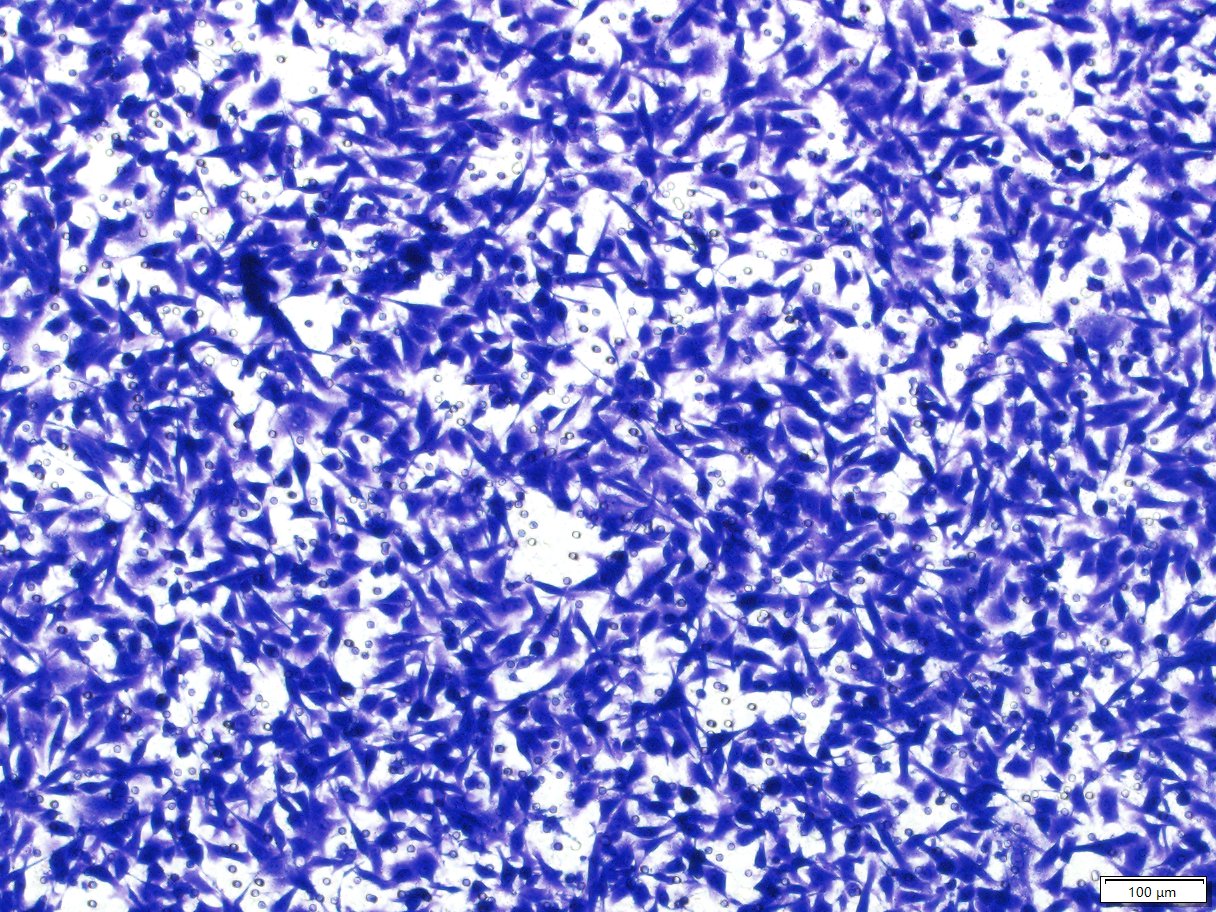

Supplement: Supplemental Information 5 [file peerj-cs-09-1651-s005.zip › Dataset 4/1+2.jpg]

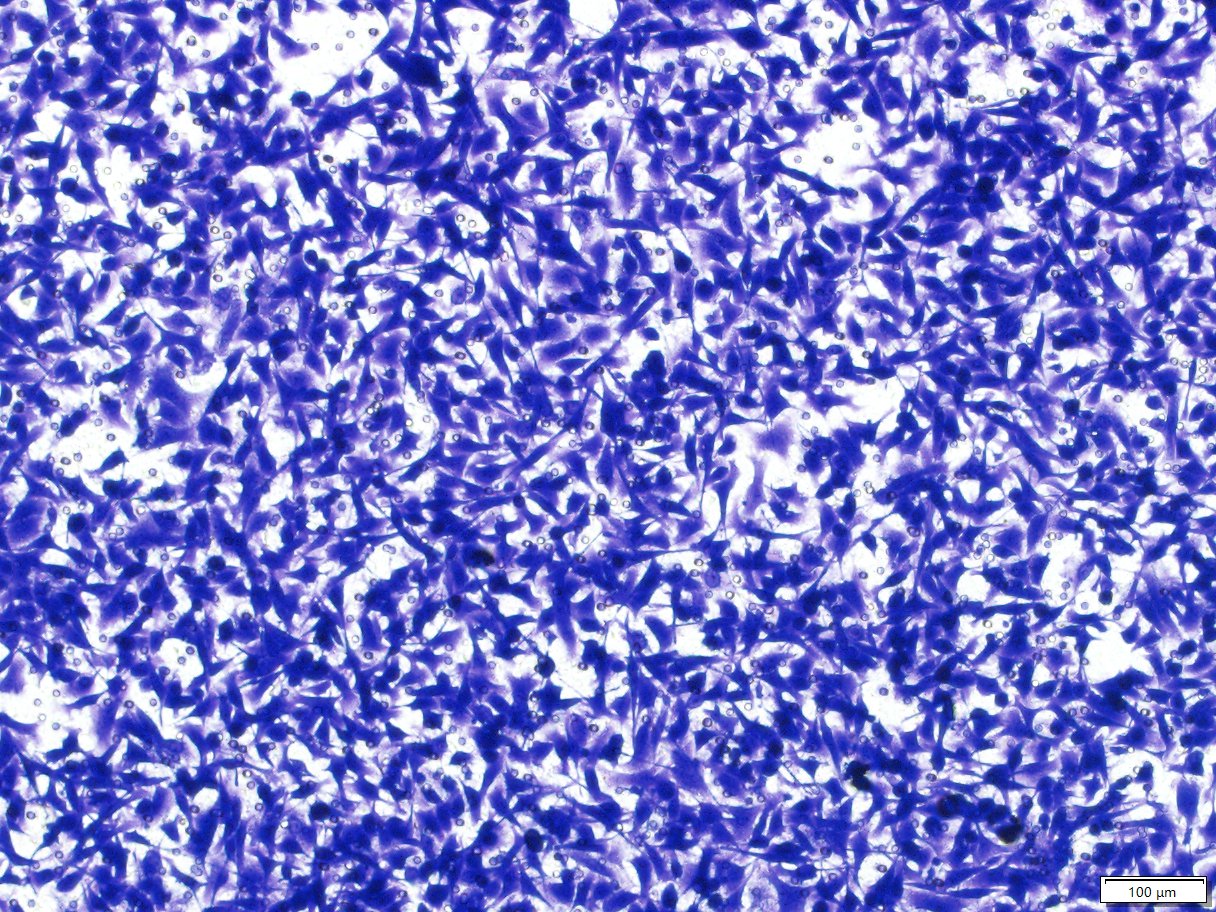

Supplement: Supplemental Information 5 [file peerj-cs-09-1651-s005.zip › Dataset 4/1+3.jpg]

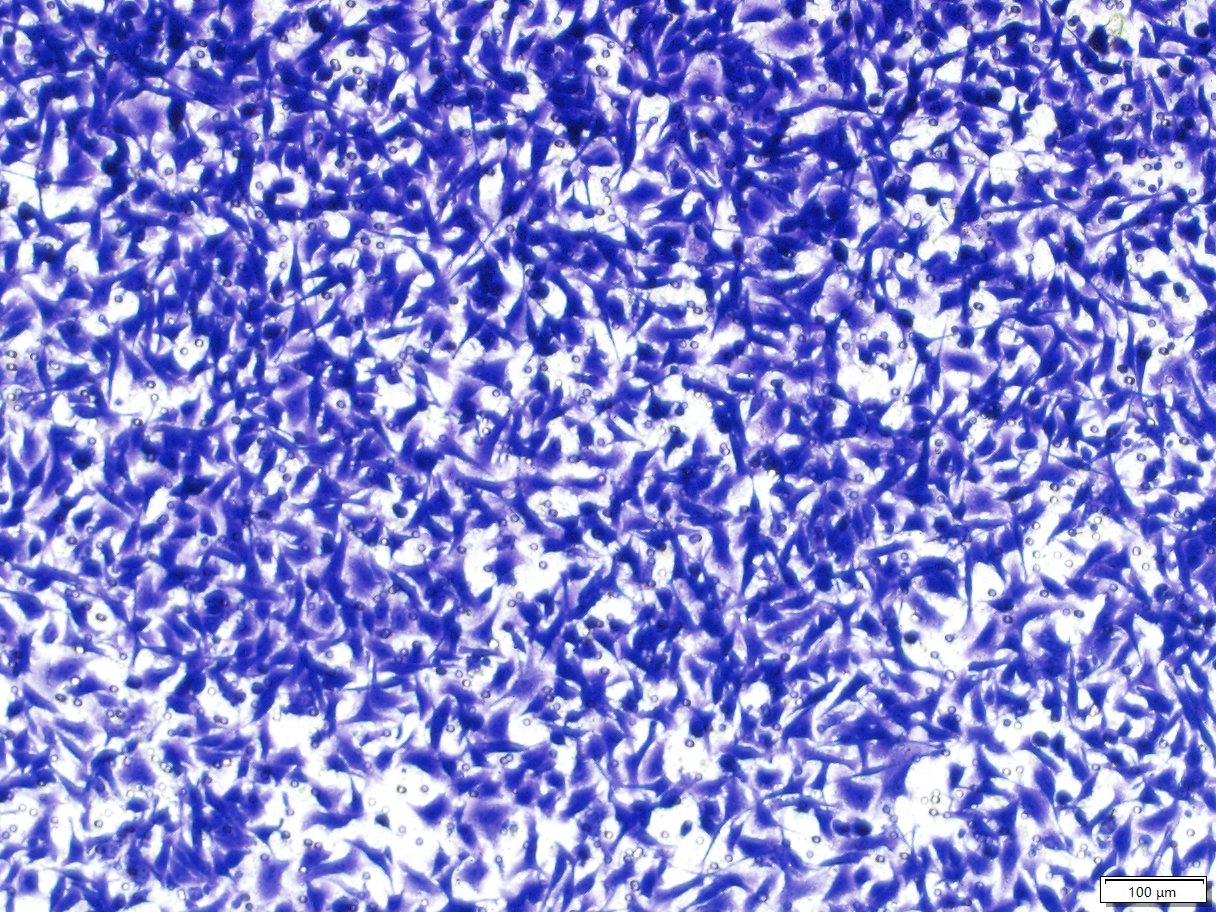

Supplement: Supplemental Information 5 [file peerj-cs-09-1651-s005.zip › Dataset 4/1+4.jpg]

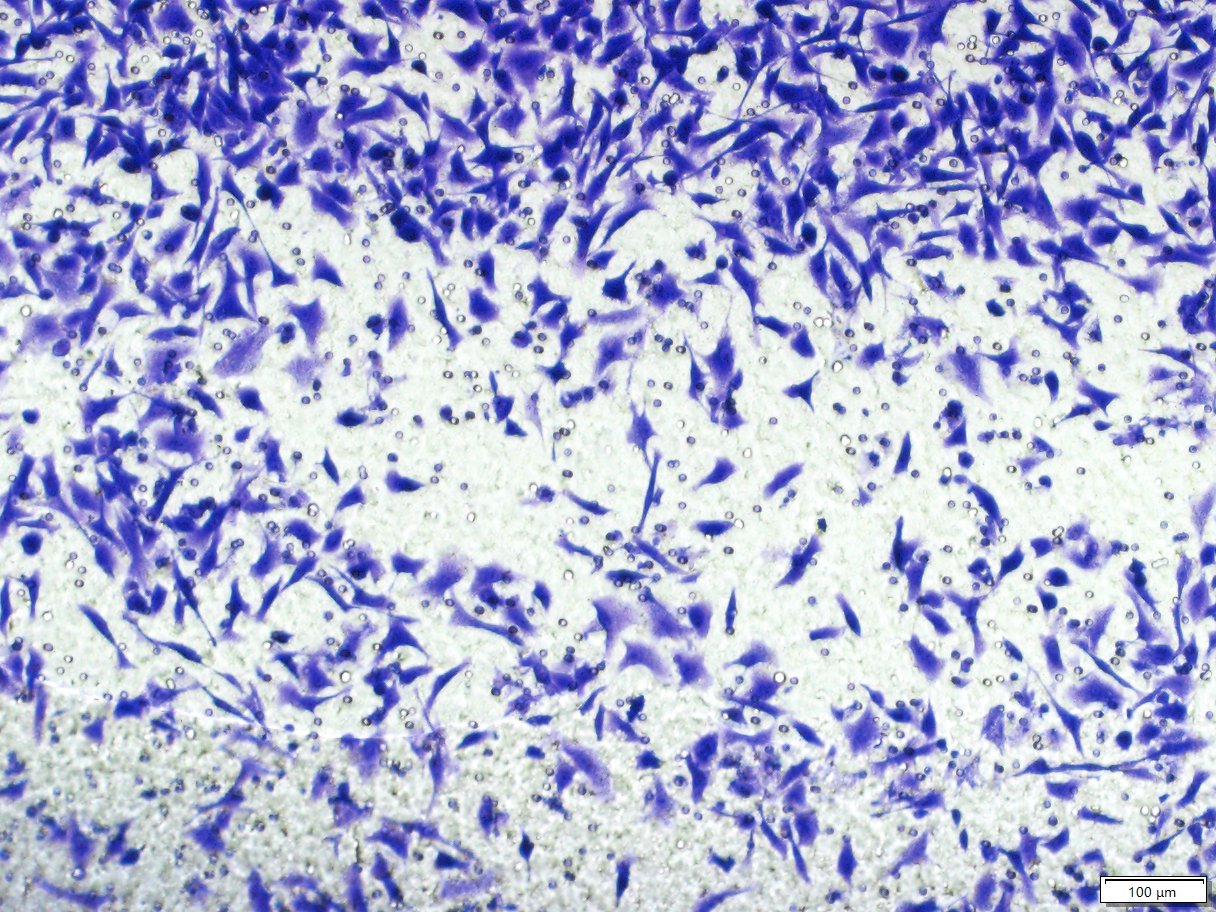

Supplement: Supplemental Information 5 [file peerj-cs-09-1651-s005.zip › Dataset 4/1+5.jpg]

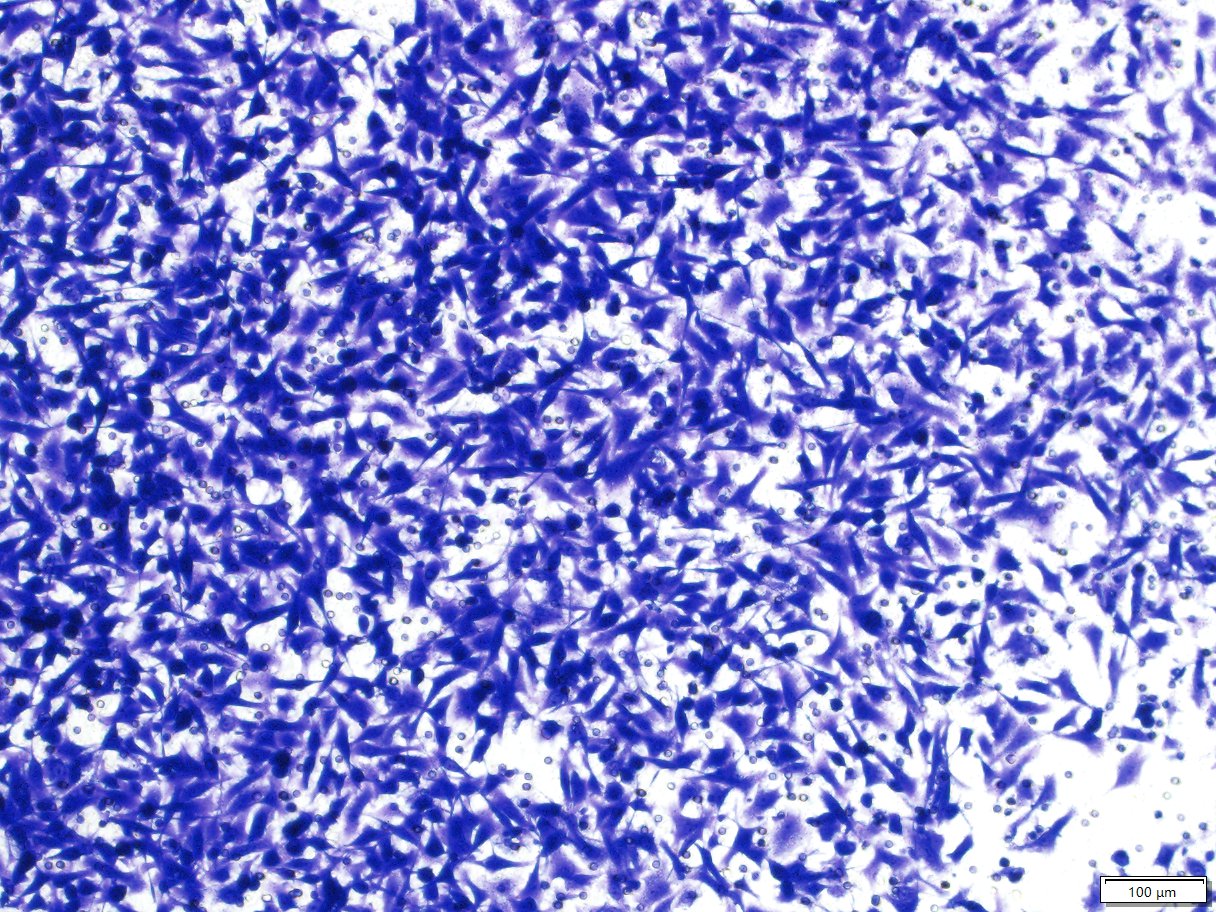

Supplement: Supplemental Information 5 [file peerj-cs-09-1651-s005.zip › Dataset 4/1+7.jpg]

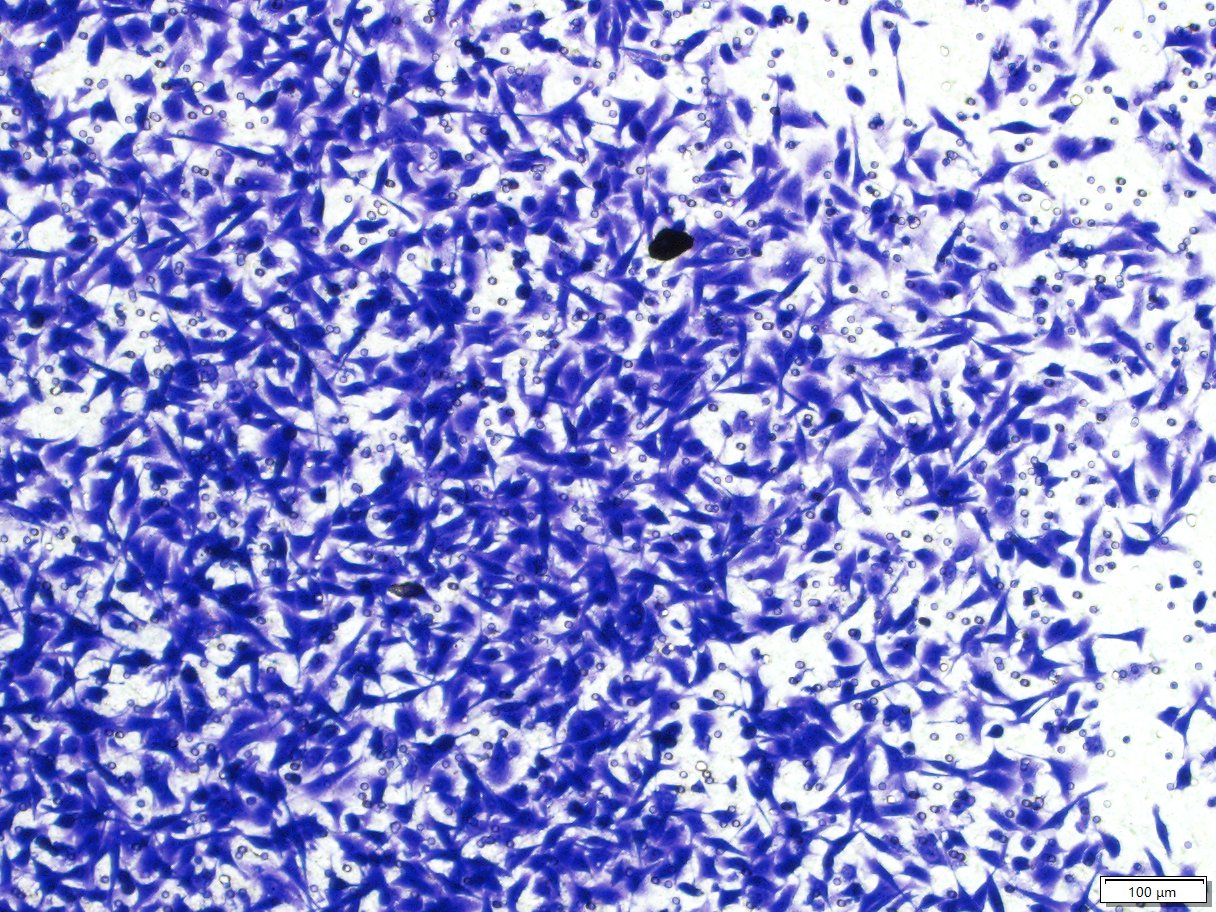

Supplement: Supplemental Information 5 [file peerj-cs-09-1651-s005.zip › Dataset 4/1+8.jpg]

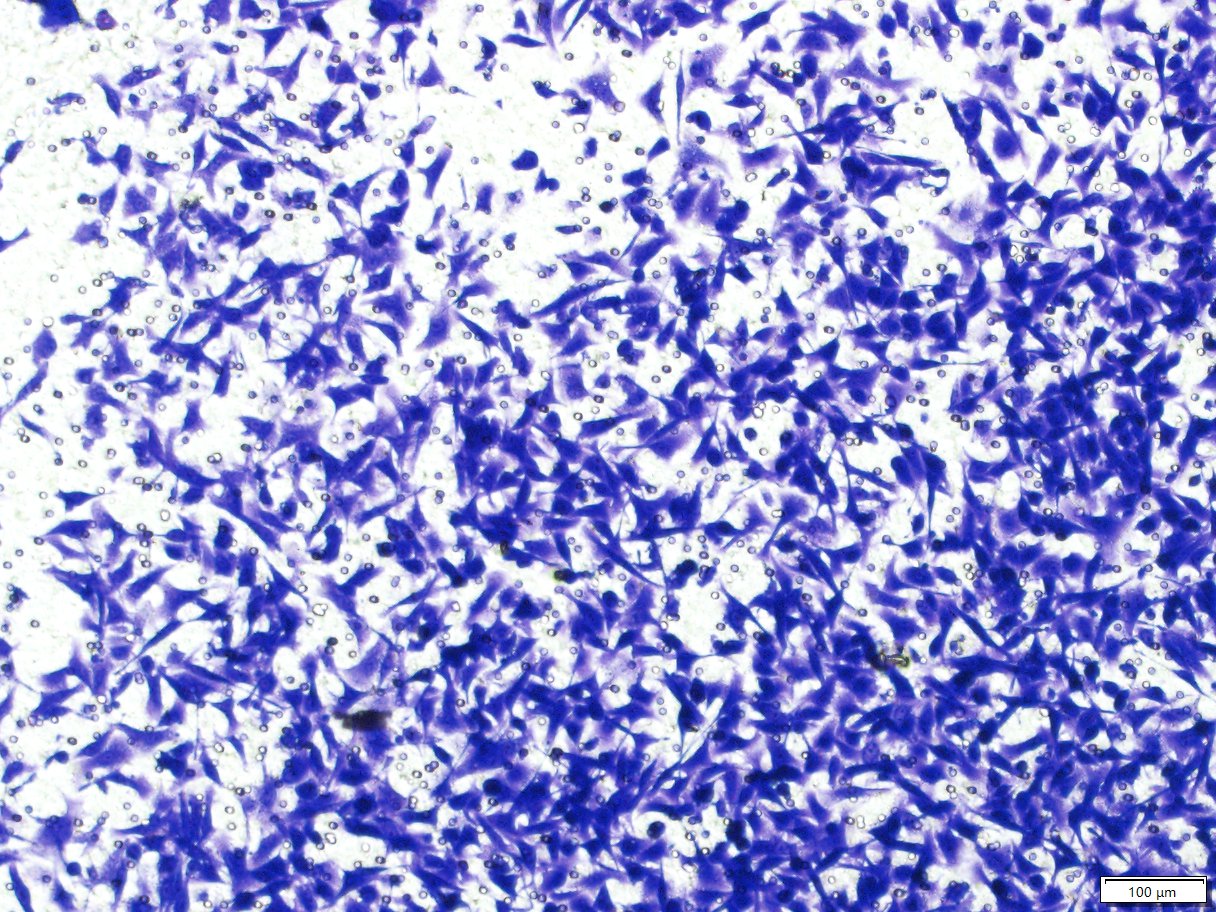

Supplement: Supplemental Information 5 [file peerj-cs-09-1651-s005.zip › Dataset 4/1+9.jpg]
