# Supplementary figures and images for: A deep learning approach for the detection and counting of colon cancer cells (HT-29 cells) bunches and impurities (part 3 of 6)
Source: PeerJ Comput Sci. 2023 Dec 5;9:e1651. doi: 10.7717/peerj-cs.1651 (PMC10773923; doi:10.7717/peerj-cs.1651)

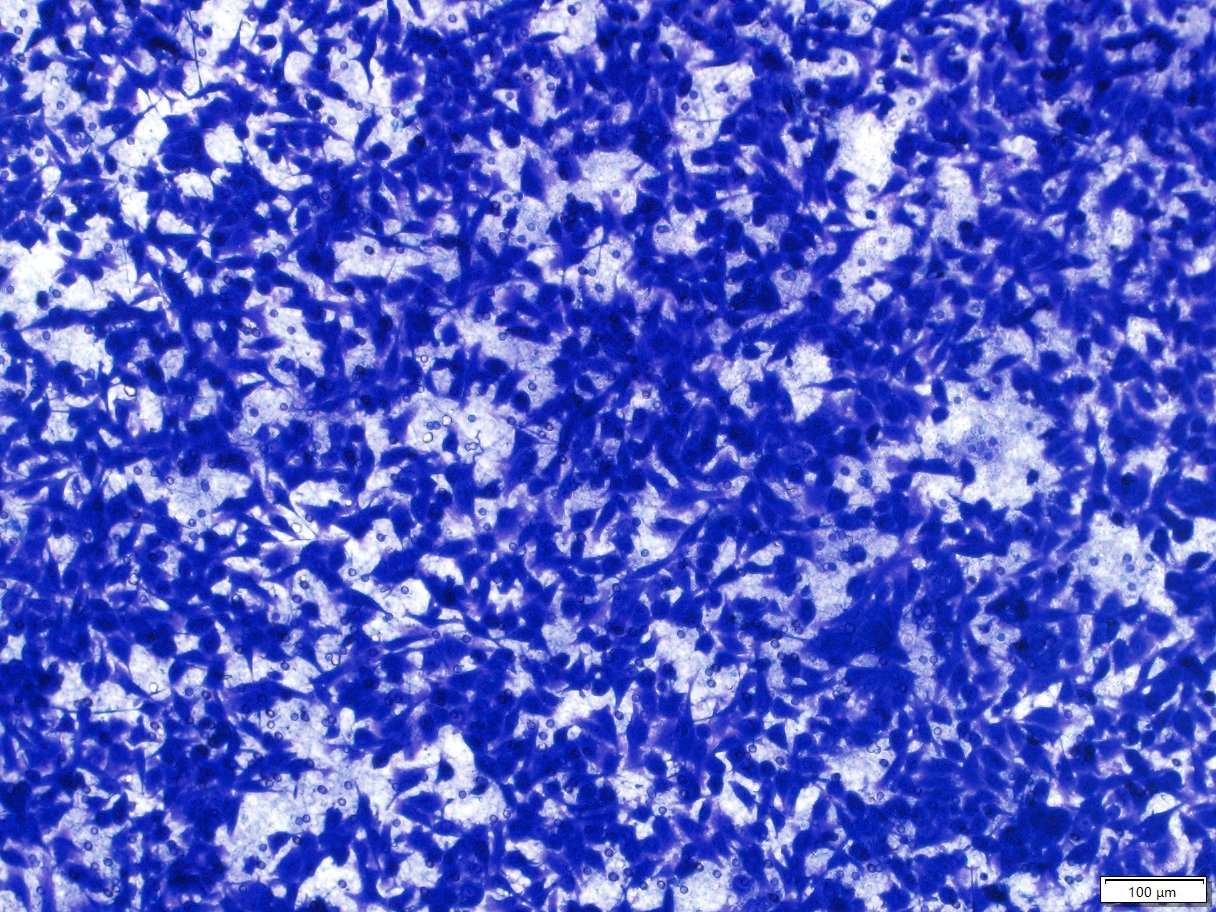

Supplement: Supplemental Information 5 [file peerj-cs-09-1651-s005.zip › Dataset 4/1-1.jpg]

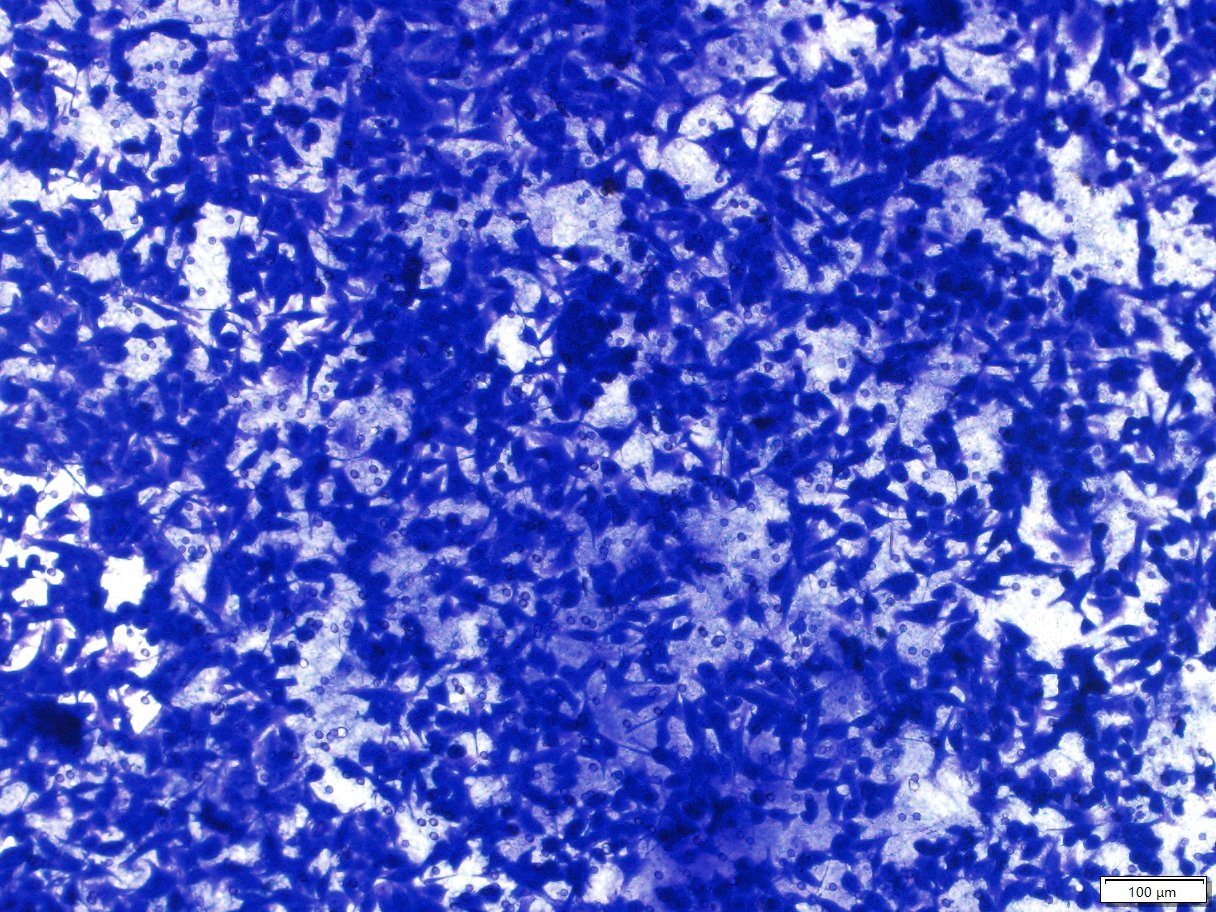

Supplement: Supplemental Information 5 [file peerj-cs-09-1651-s005.zip › Dataset 4/1-10.jpg]

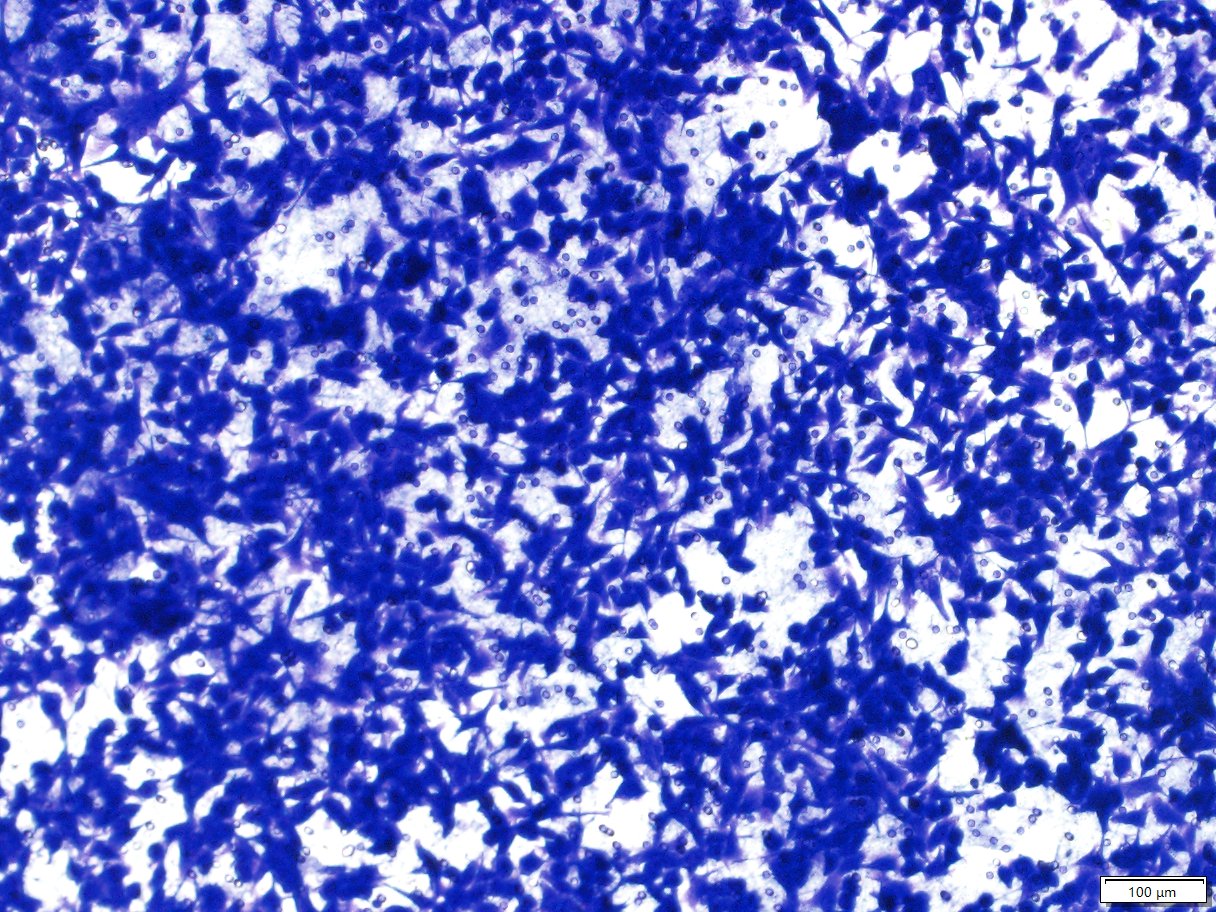

Supplement: Supplemental Information 5 [file peerj-cs-09-1651-s005.zip › Dataset 4/1-11.jpg]

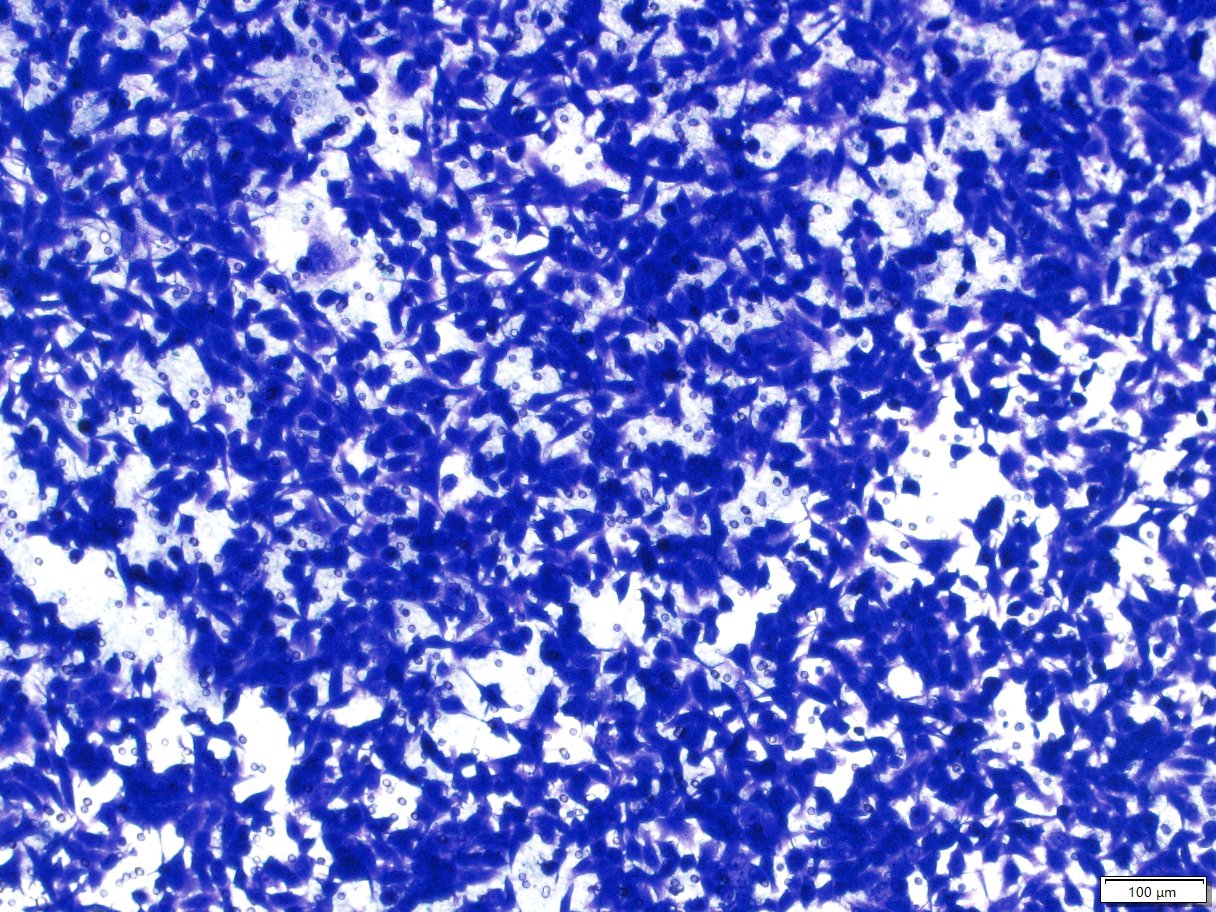

Supplement: Supplemental Information 5 [file peerj-cs-09-1651-s005.zip › Dataset 4/1-2.jpg]

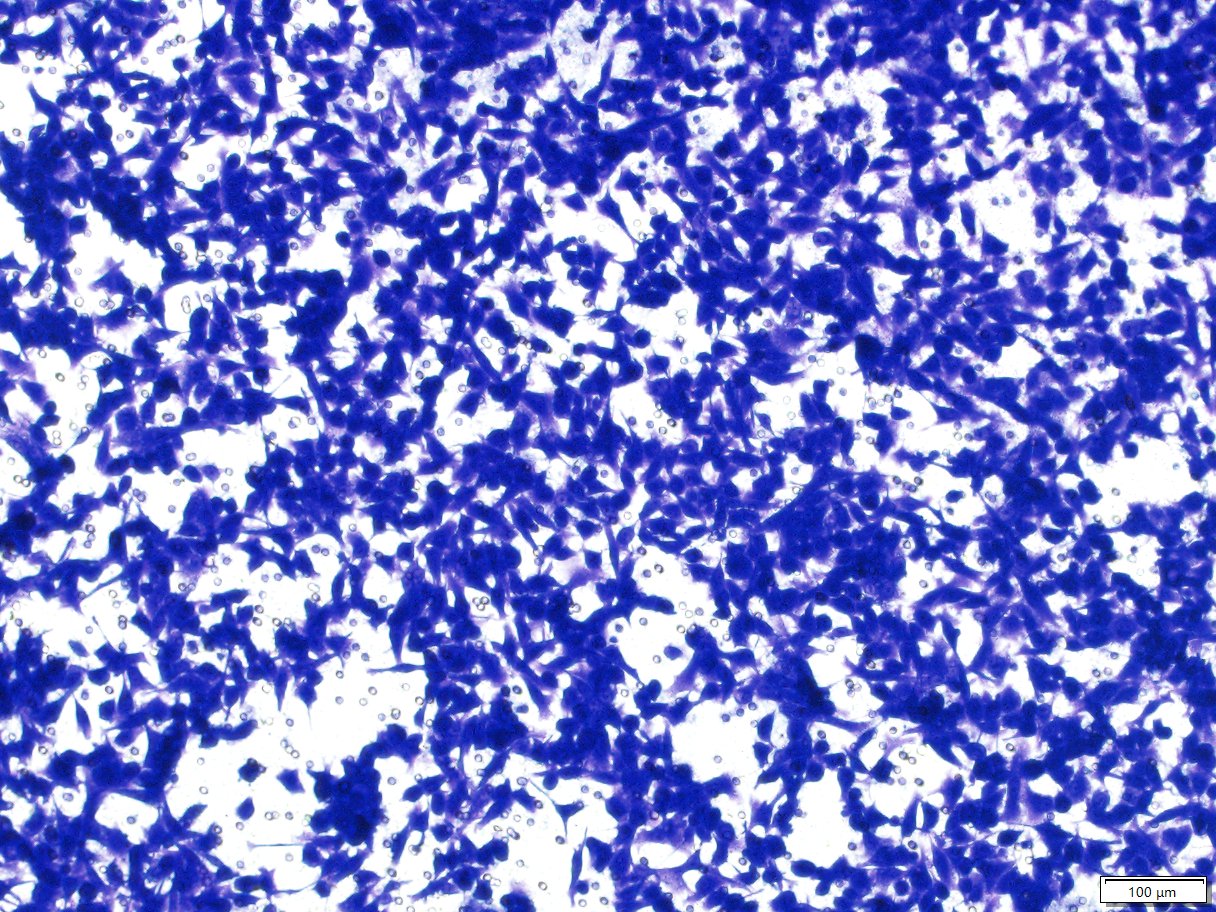

Supplement: Supplemental Information 5 [file peerj-cs-09-1651-s005.zip › Dataset 4/1-3.jpg]

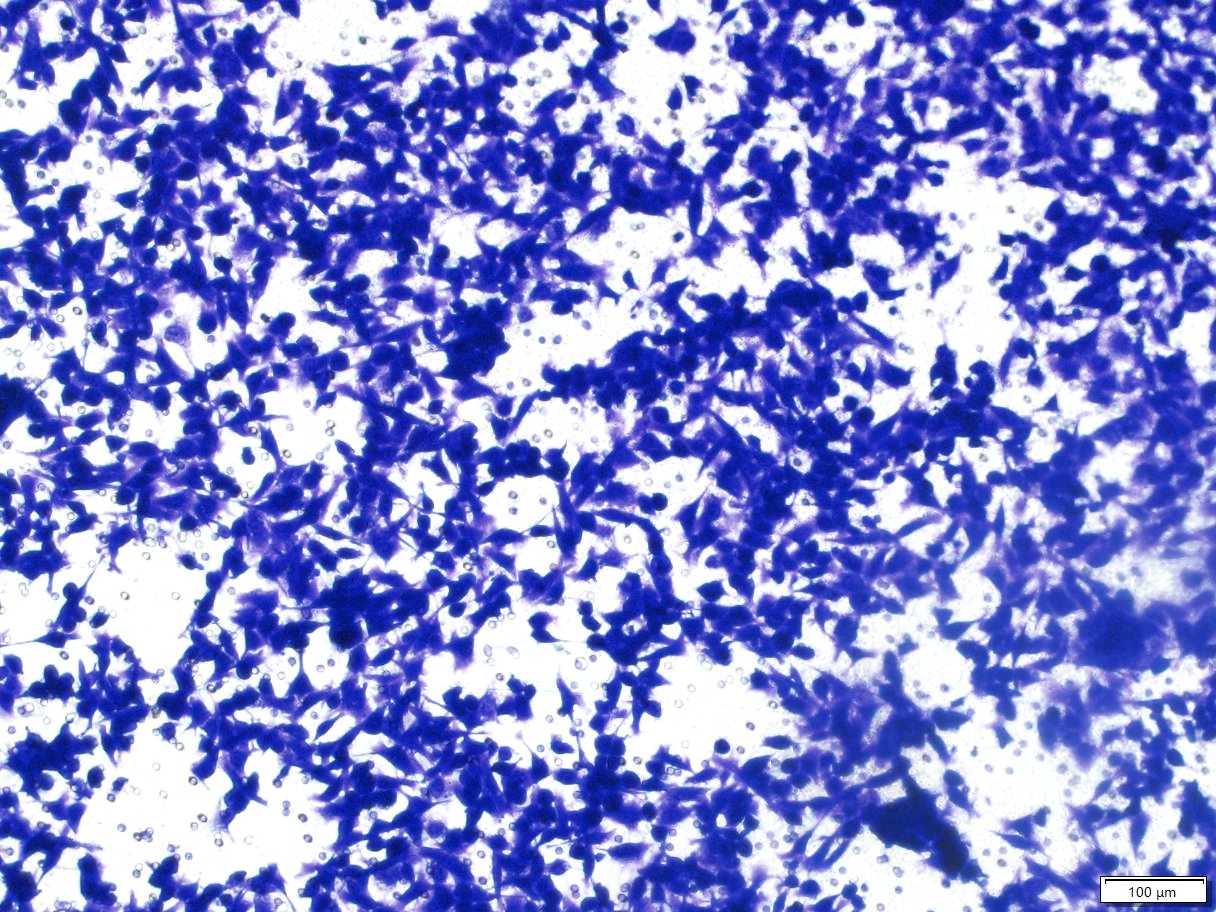

Supplement: Supplemental Information 5 [file peerj-cs-09-1651-s005.zip › Dataset 4/1-4.jpg]

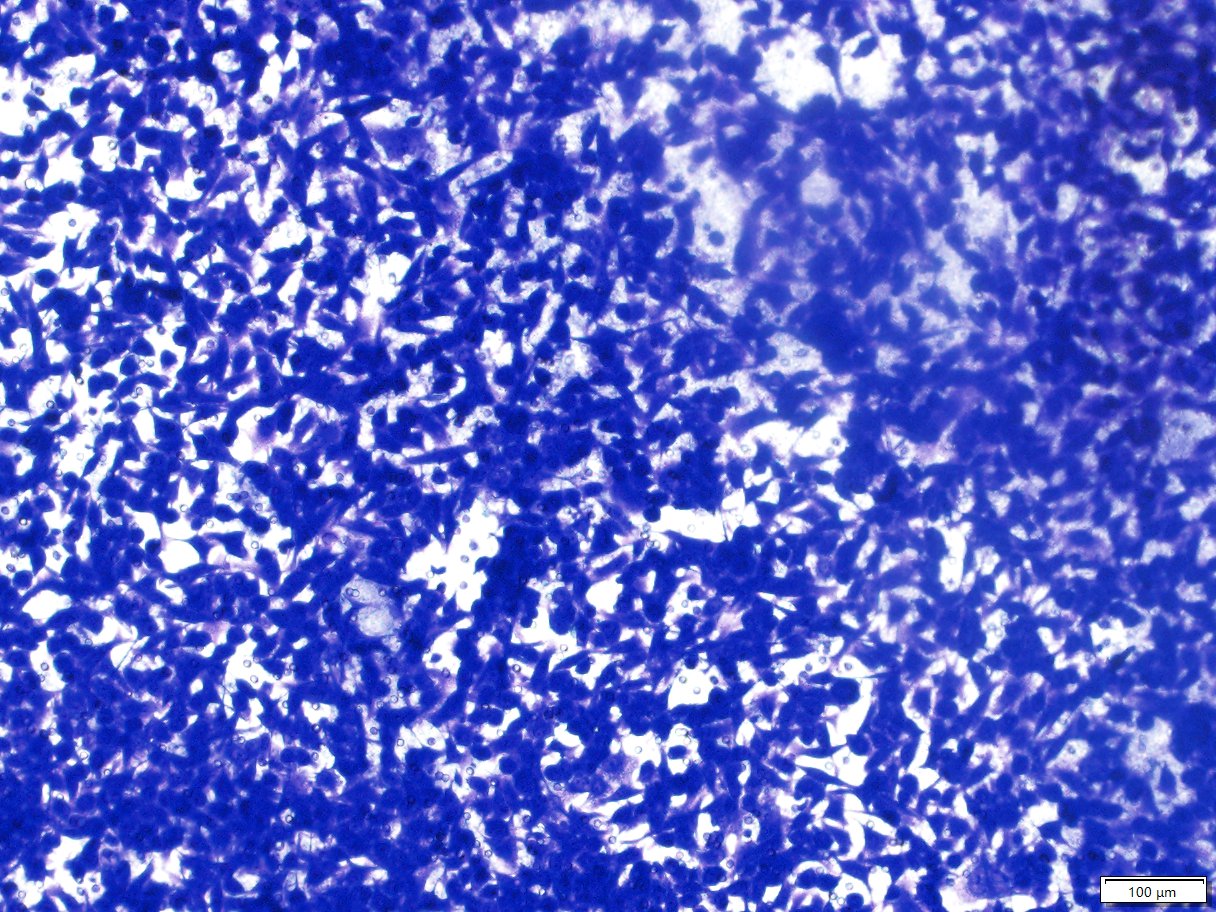

Supplement: Supplemental Information 5 [file peerj-cs-09-1651-s005.zip › Dataset 4/1-5.jpg]

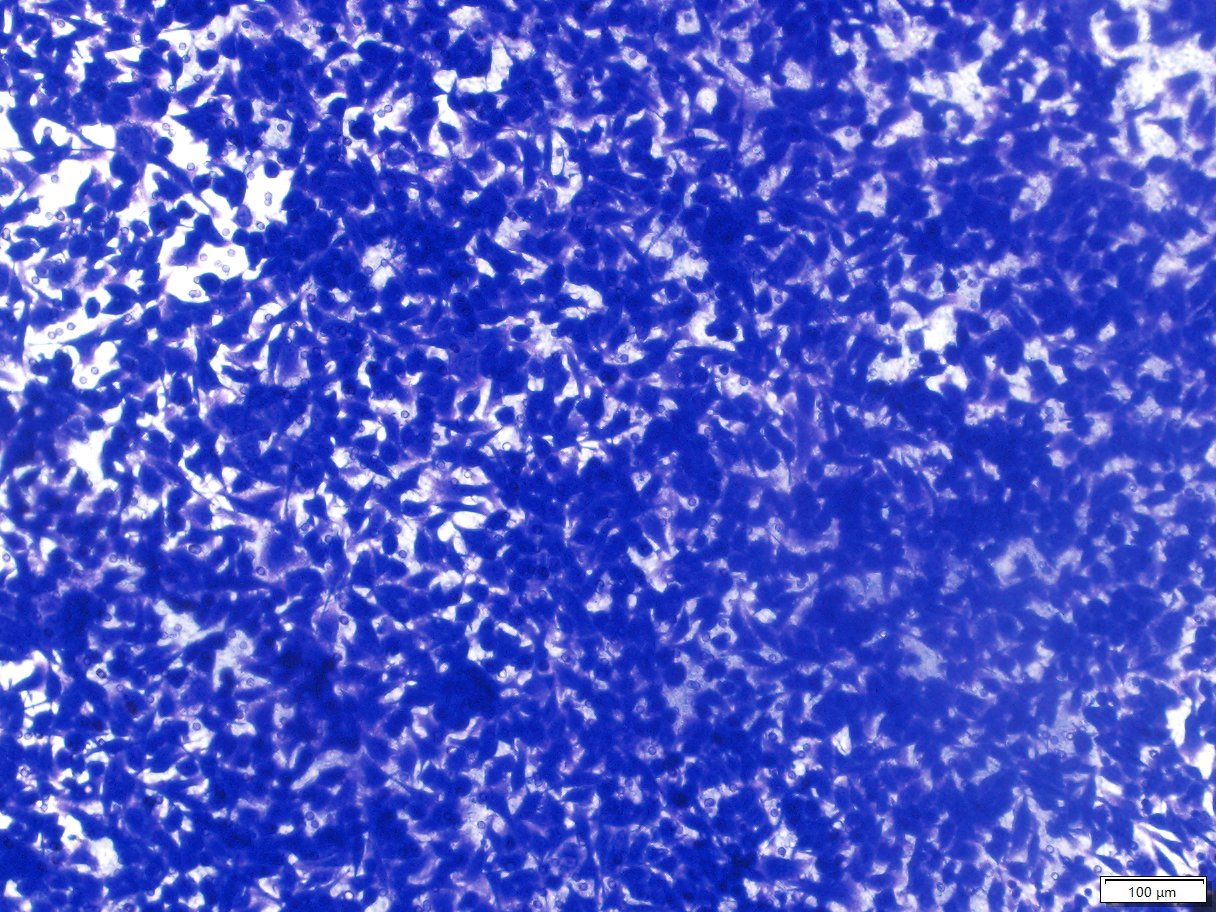

Supplement: Supplemental Information 5 [file peerj-cs-09-1651-s005.zip › Dataset 4/1-6.jpg]

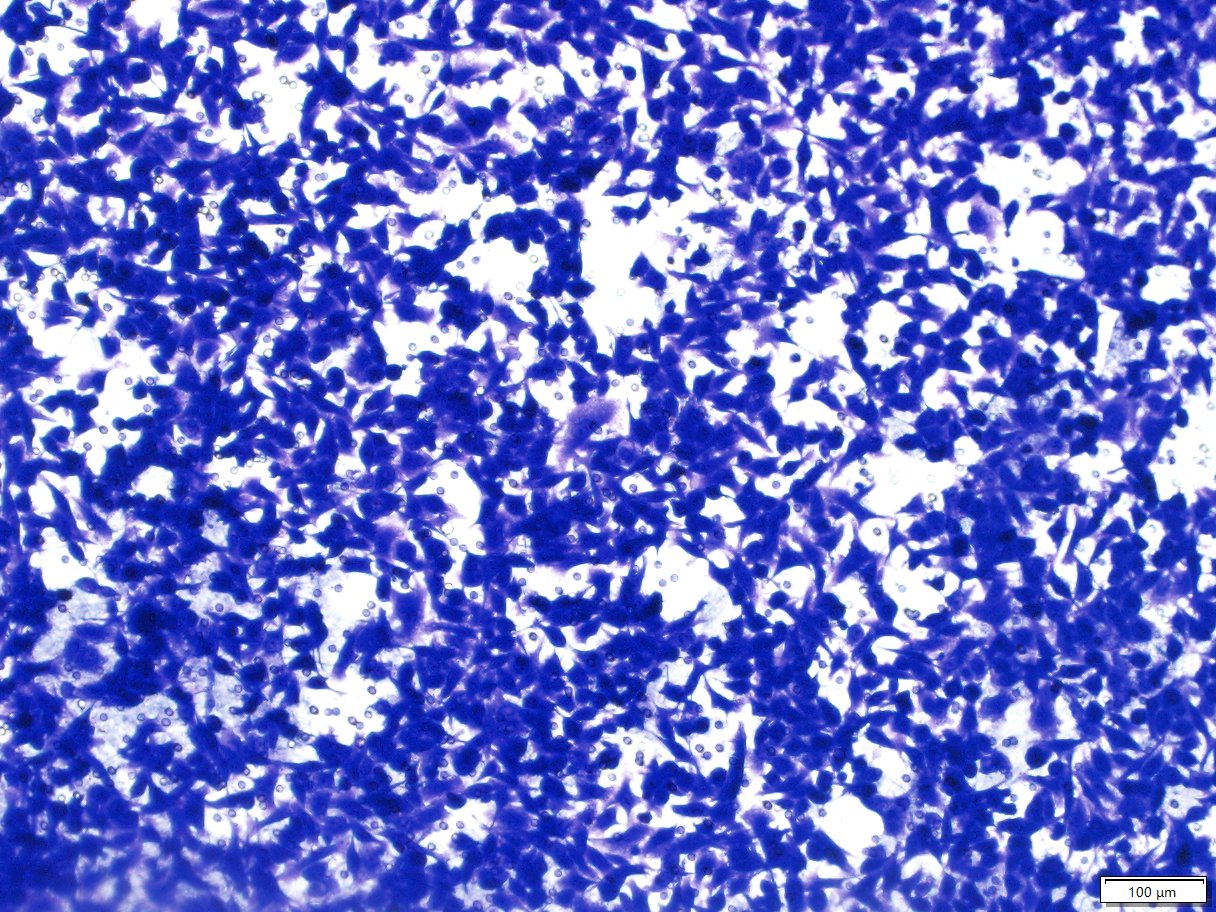

Supplement: Supplemental Information 5 [file peerj-cs-09-1651-s005.zip › Dataset 4/1-7.jpg]

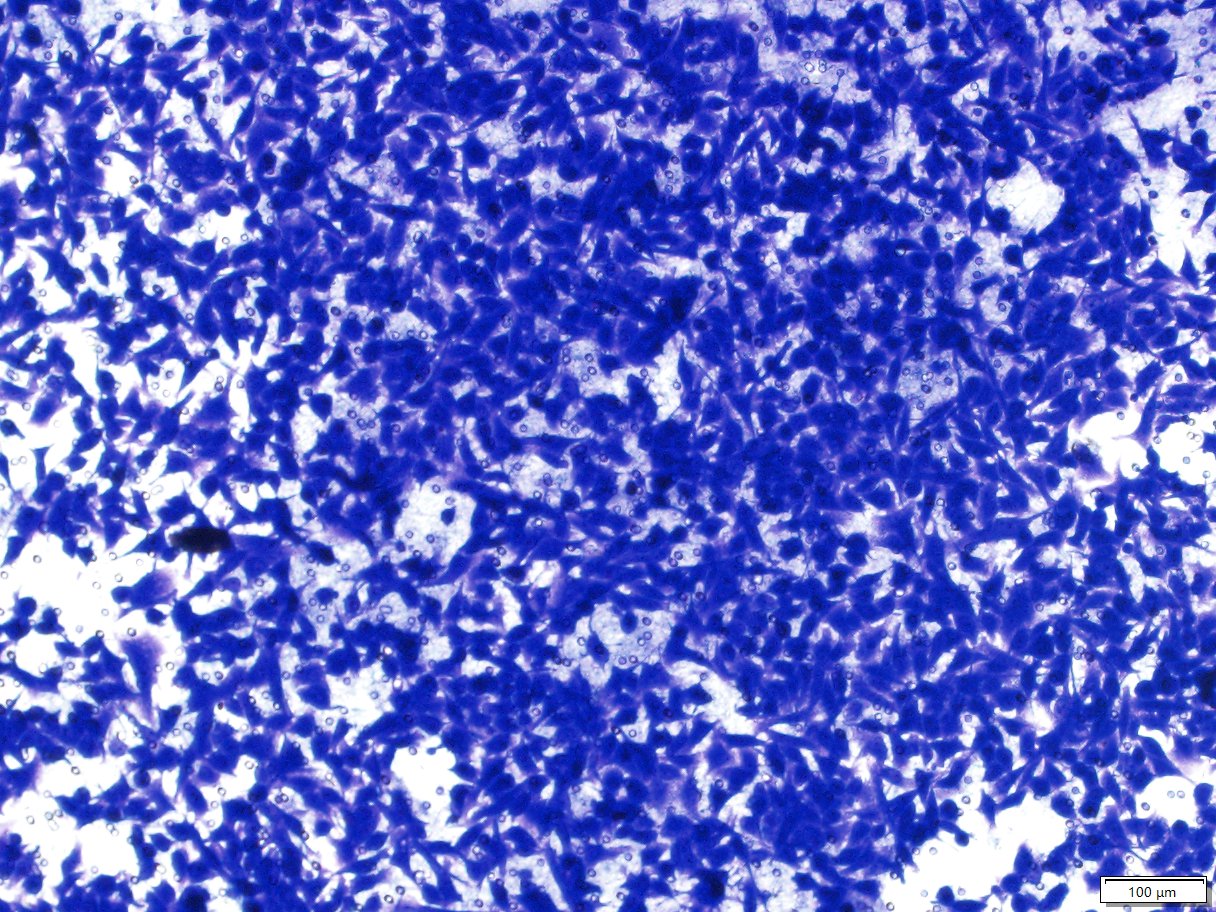

Supplement: Supplemental Information 5 [file peerj-cs-09-1651-s005.zip › Dataset 4/1-8.jpg]

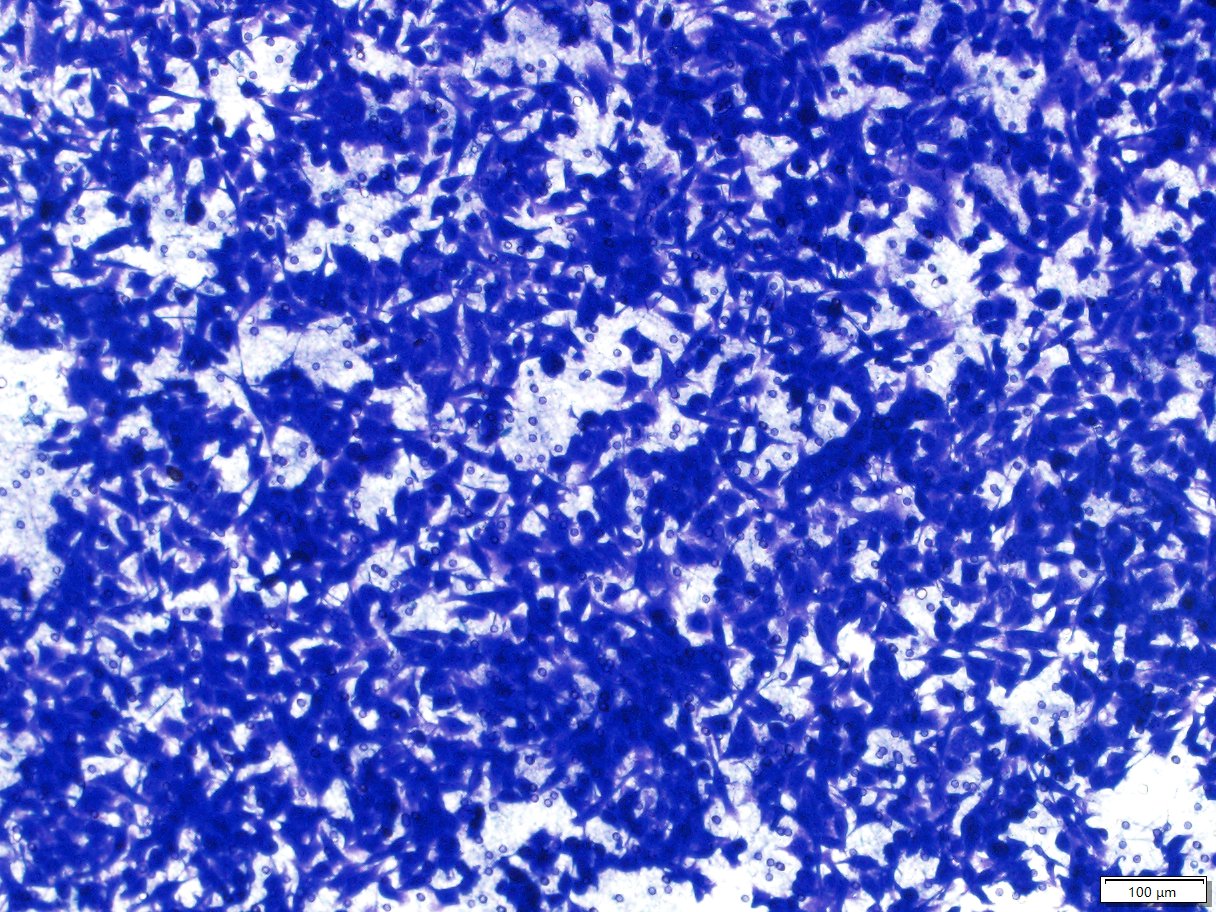

Supplement: Supplemental Information 5 [file peerj-cs-09-1651-s005.zip › Dataset 4/1-9.jpg]

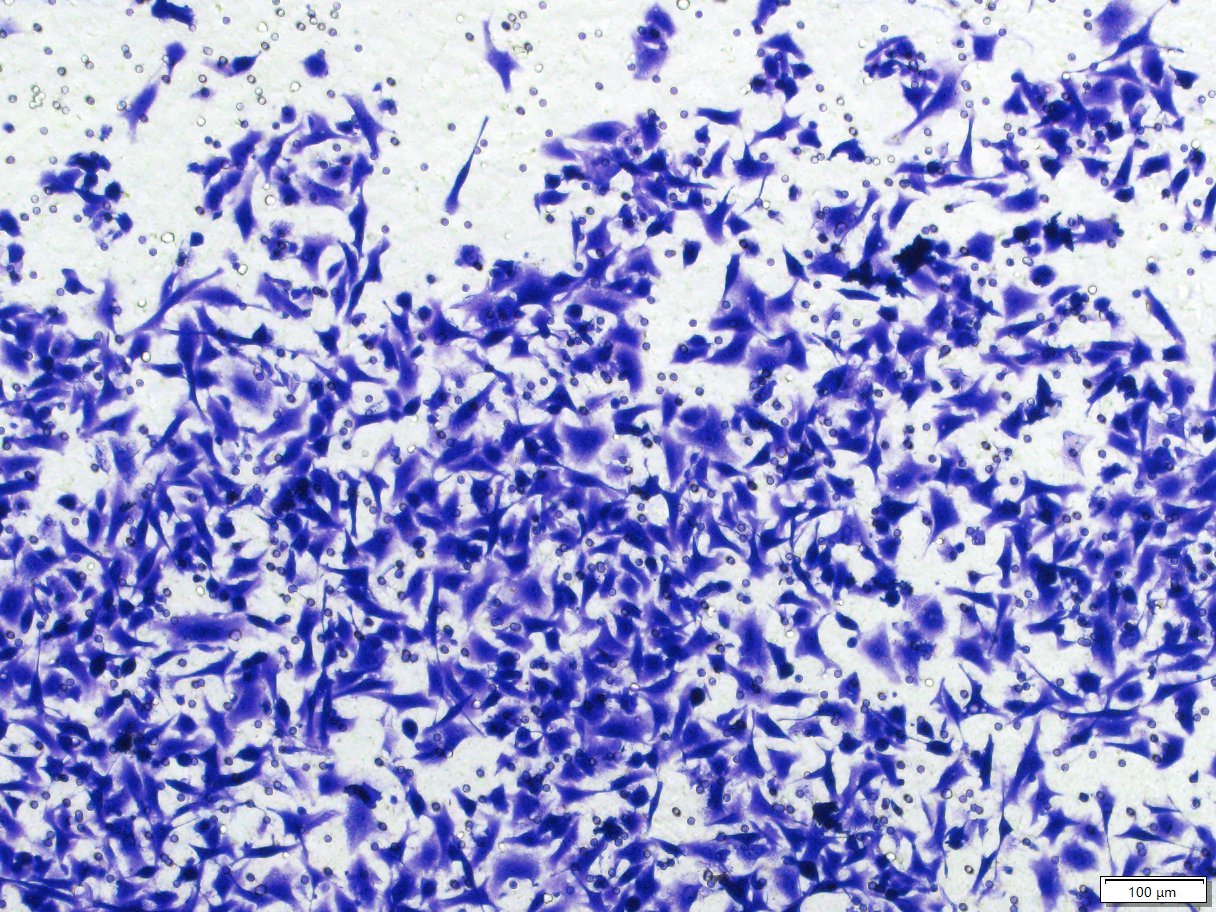

Supplement: Supplemental Information 5 [file peerj-cs-09-1651-s005.zip › Dataset 4/2+1.jpg]

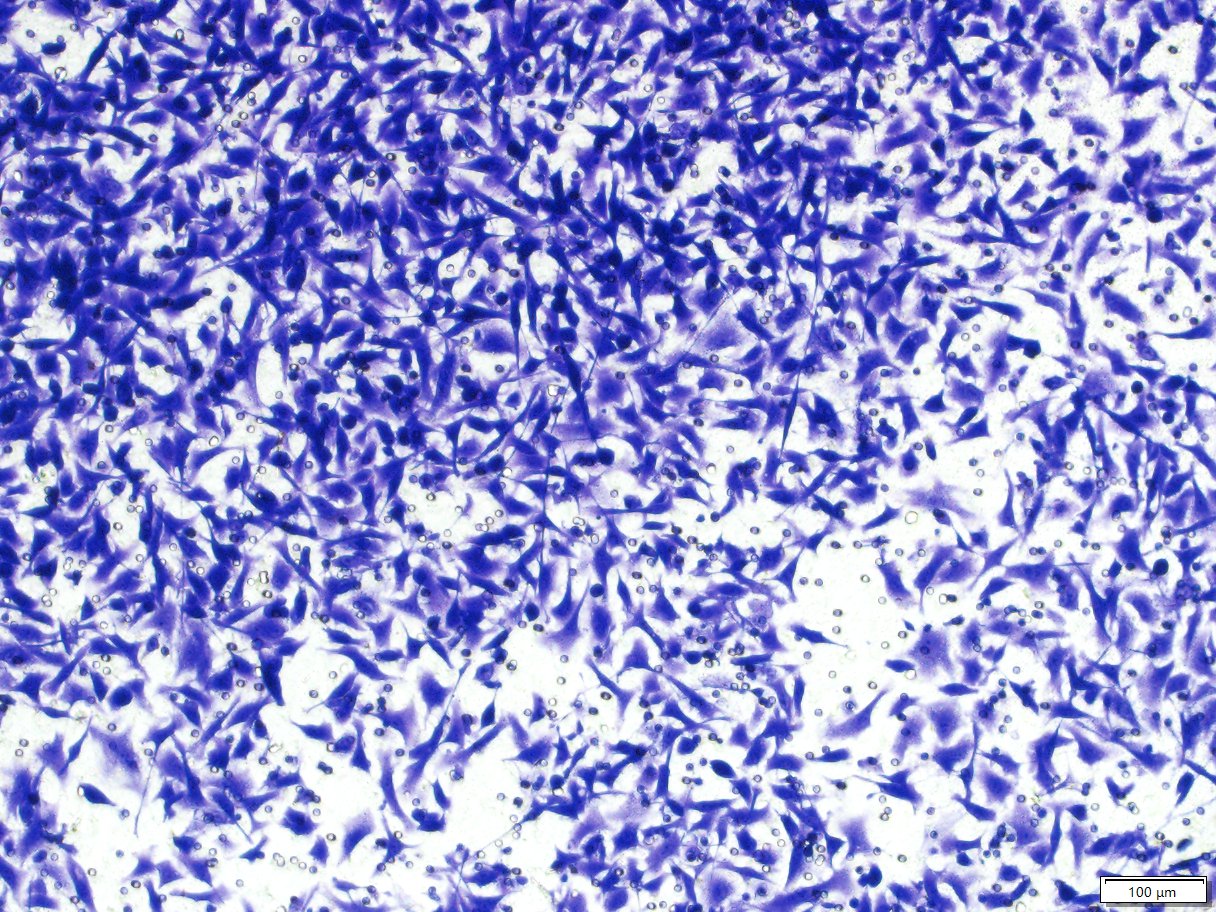

Supplement: Supplemental Information 6 [file peerj-cs-09-1651-s006.zip › Dataset 5/10+6.jpg]

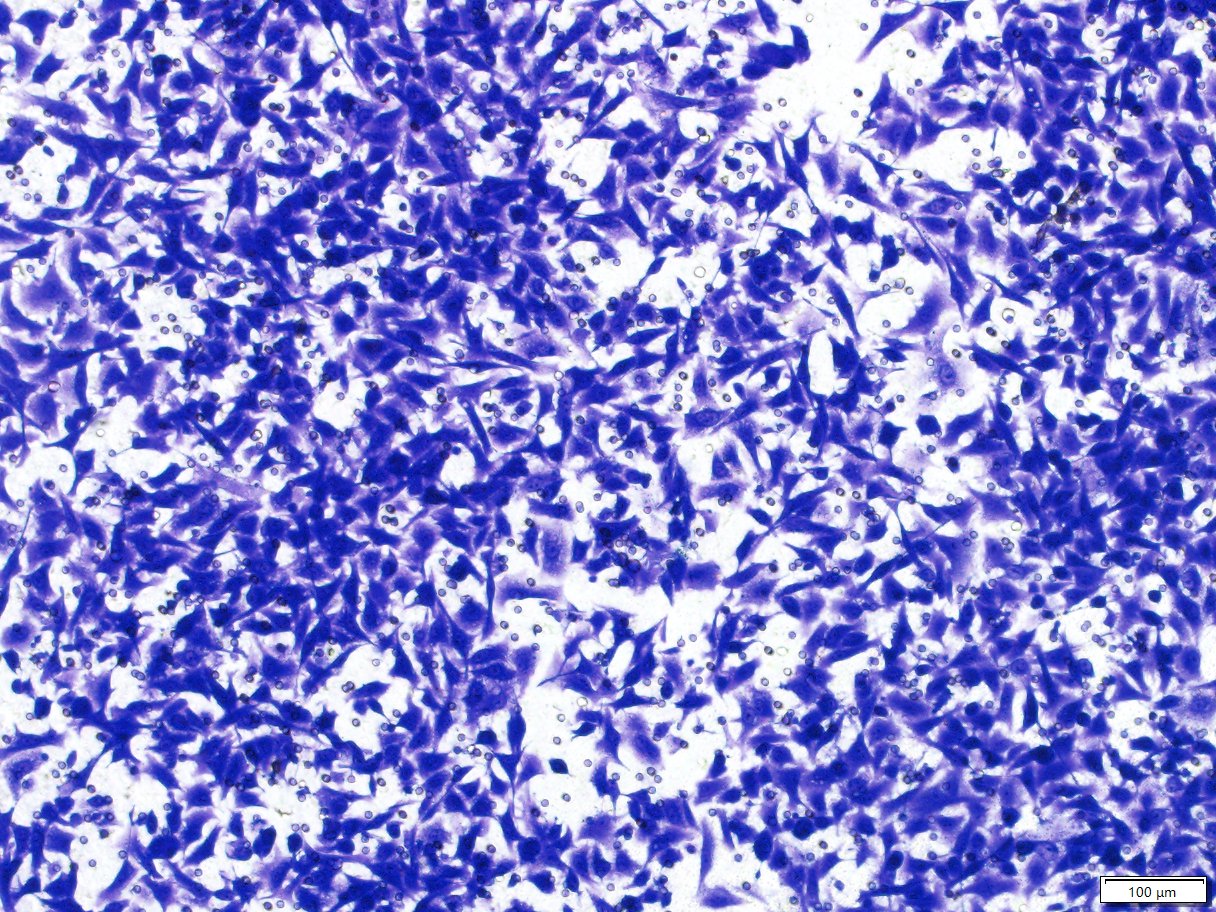

Supplement: Supplemental Information 6 [file peerj-cs-09-1651-s006.zip › Dataset 5/2+10.jpg]

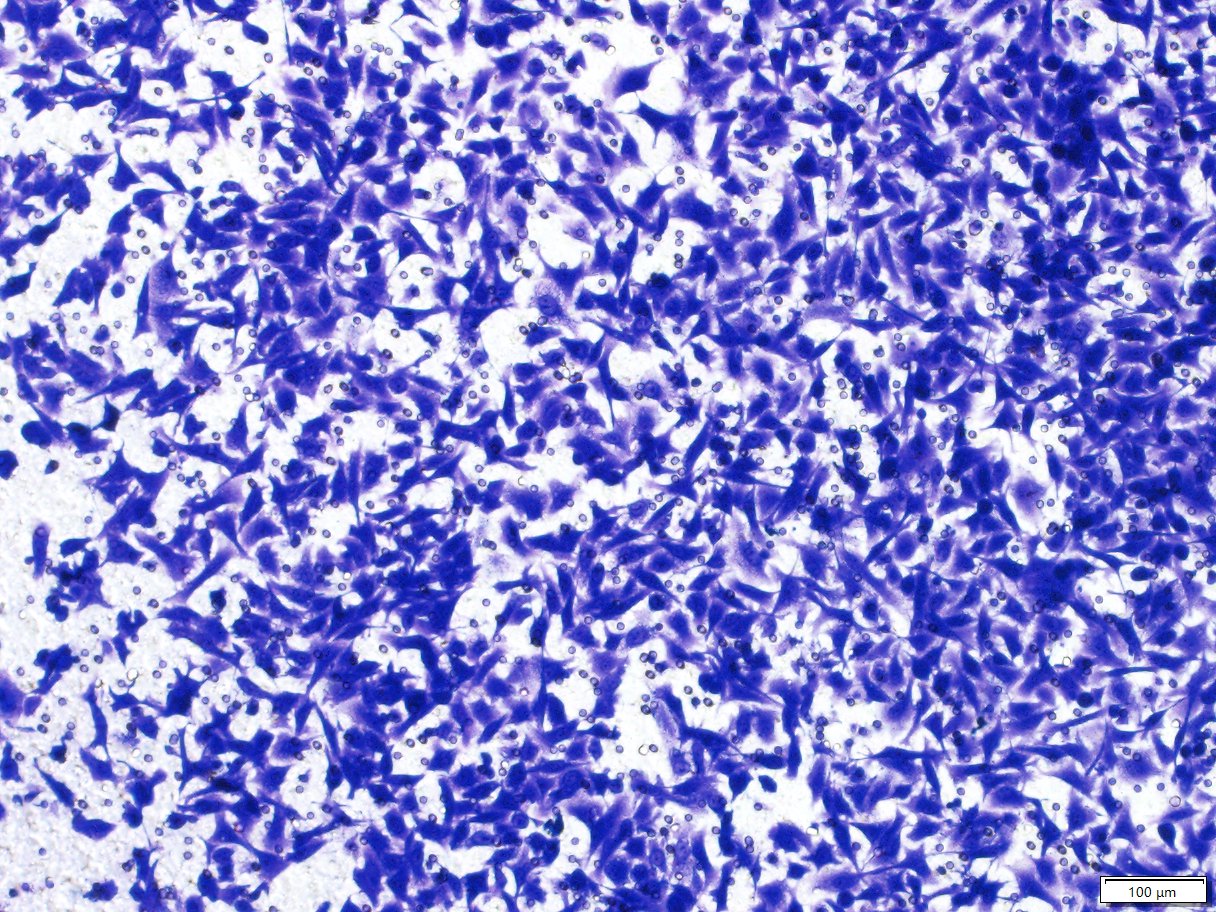

Supplement: Supplemental Information 6 [file peerj-cs-09-1651-s006.zip › Dataset 5/2+11.jpg]

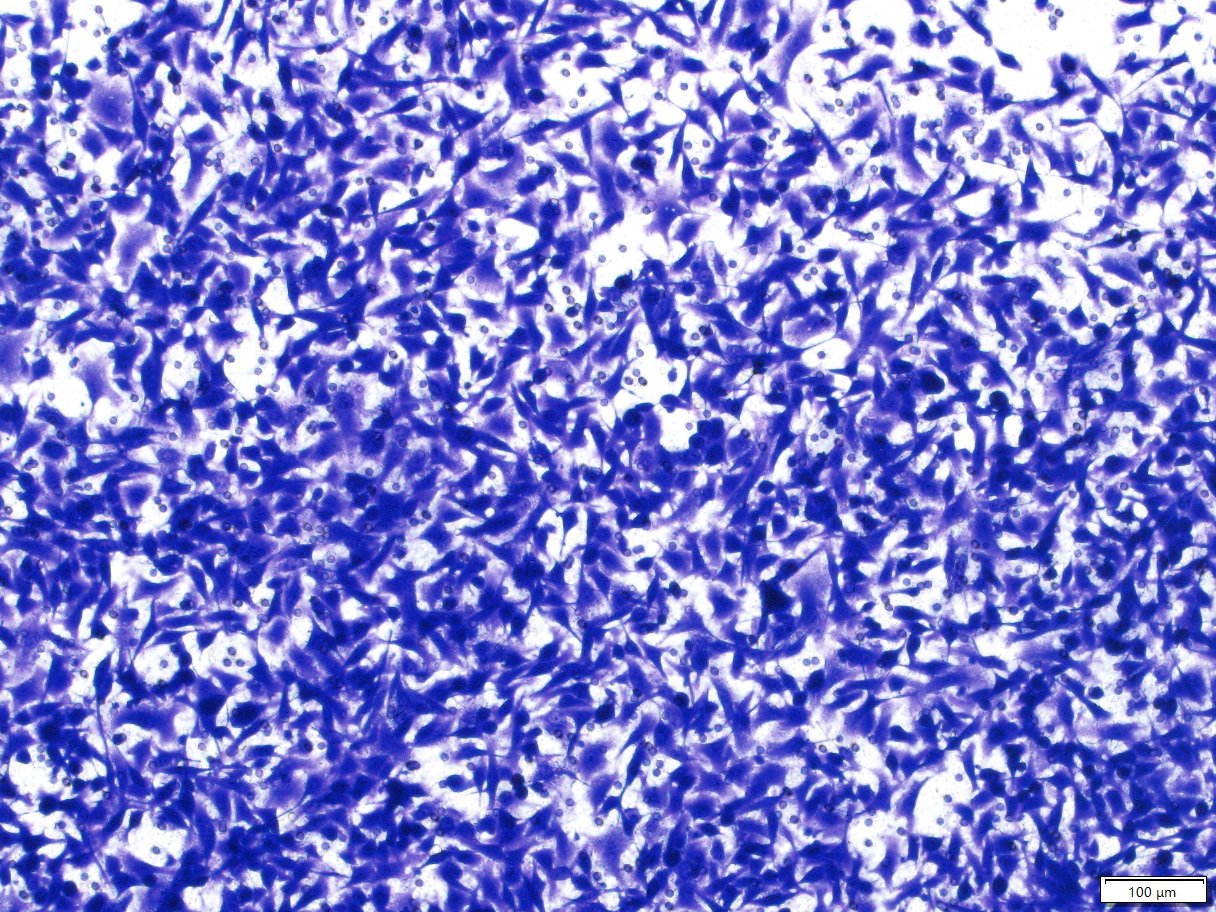

Supplement: Supplemental Information 6 [file peerj-cs-09-1651-s006.zip › Dataset 5/2+2.jpg]

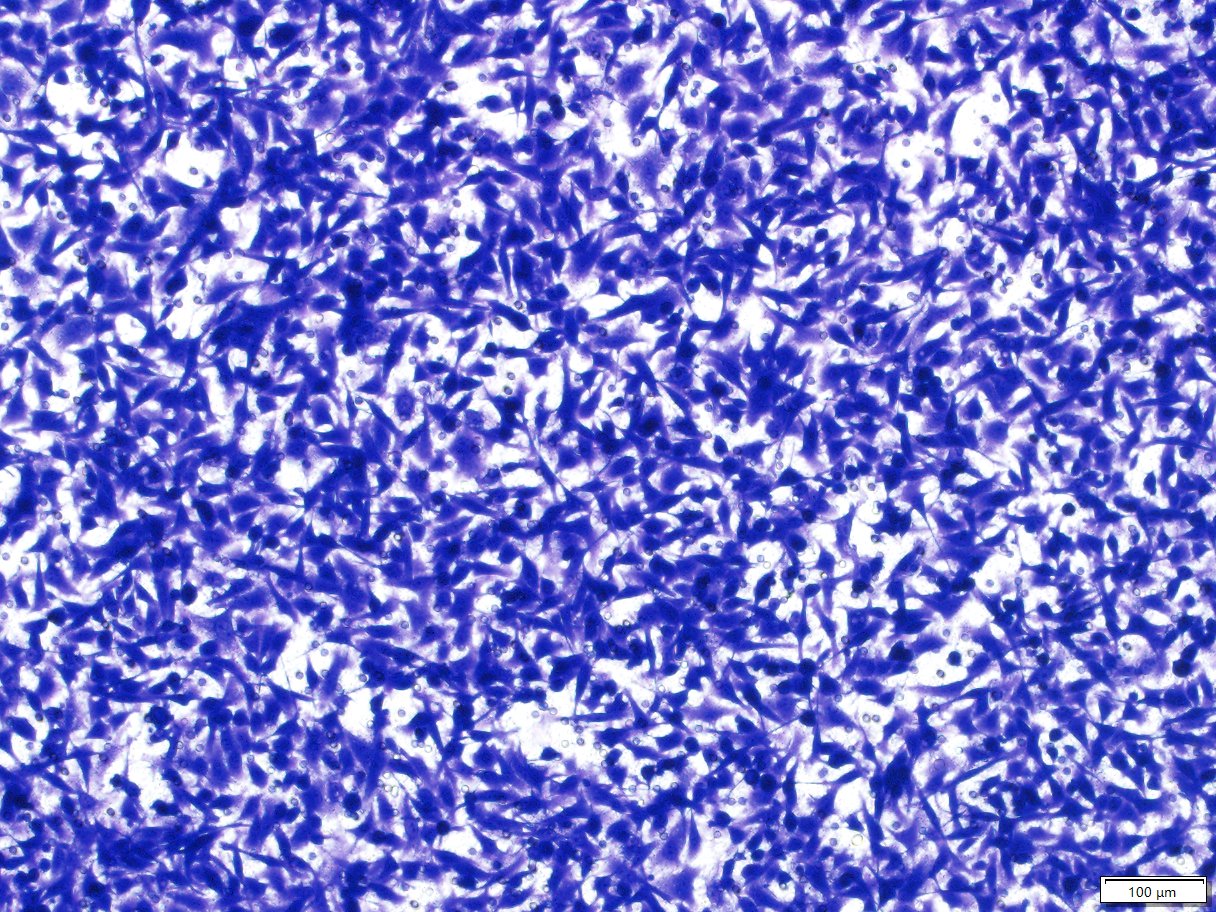

Supplement: Supplemental Information 6 [file peerj-cs-09-1651-s006.zip › Dataset 5/2+3.jpg]

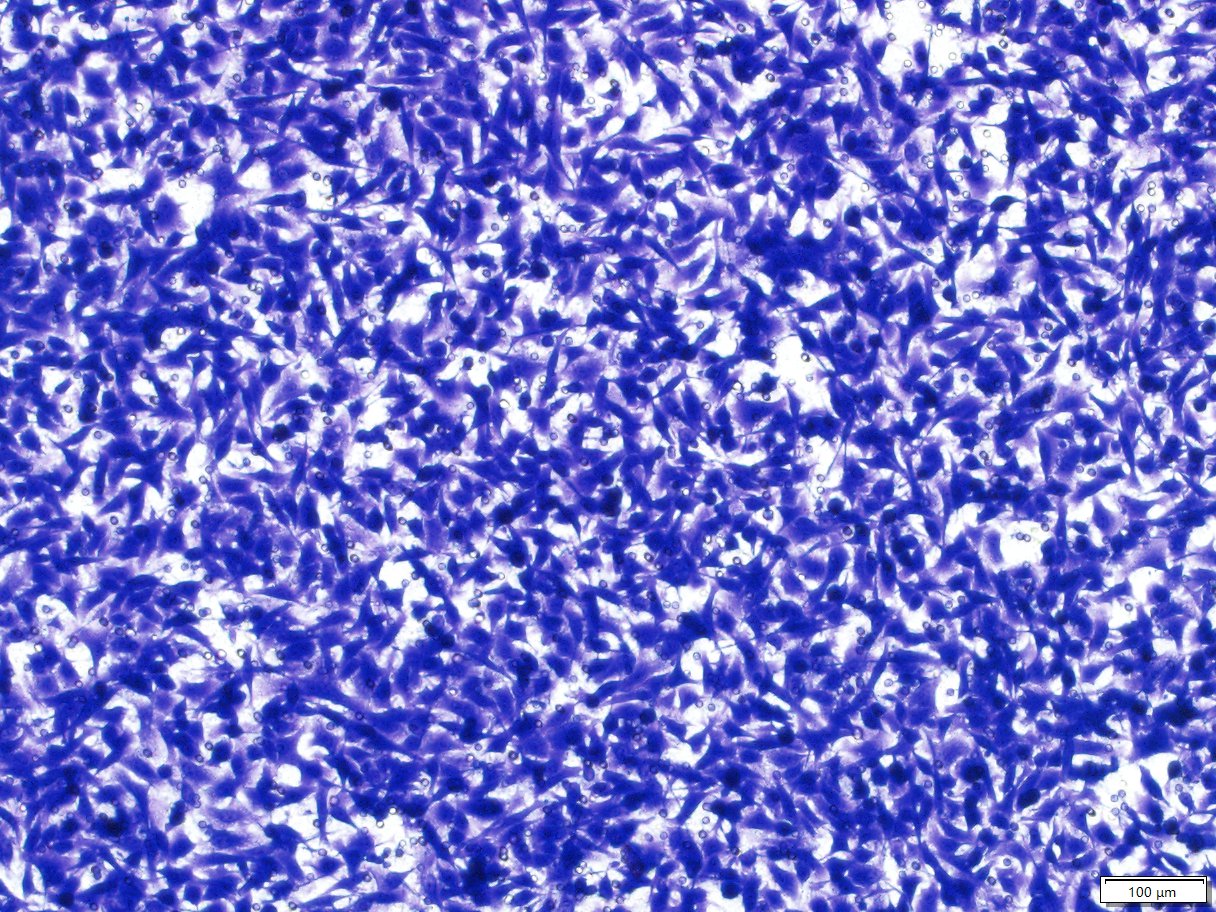

Supplement: Supplemental Information 6 [file peerj-cs-09-1651-s006.zip › Dataset 5/2+4.jpg]

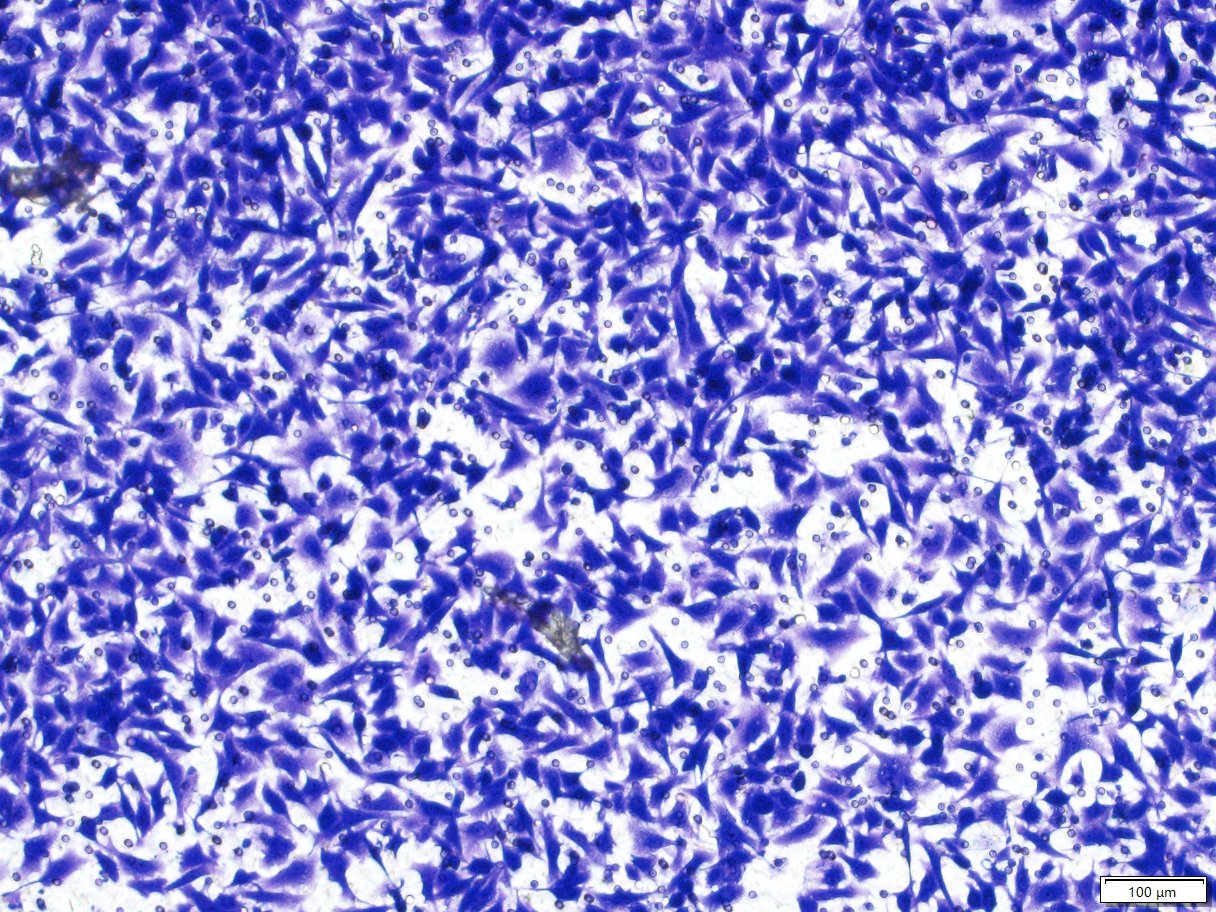

Supplement: Supplemental Information 6 [file peerj-cs-09-1651-s006.zip › Dataset 5/2+5.jpg]

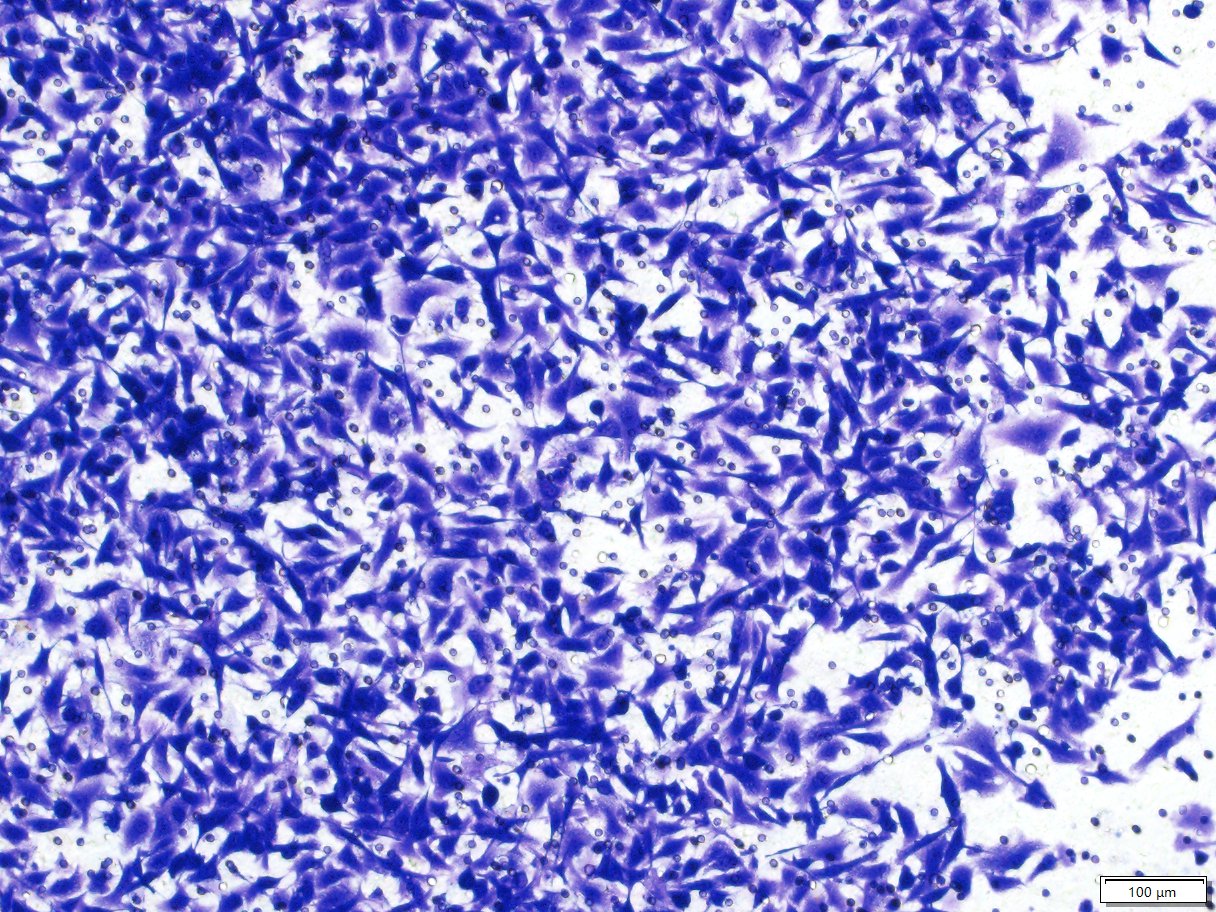

Supplement: Supplemental Information 6 [file peerj-cs-09-1651-s006.zip › Dataset 5/2+6.jpg]

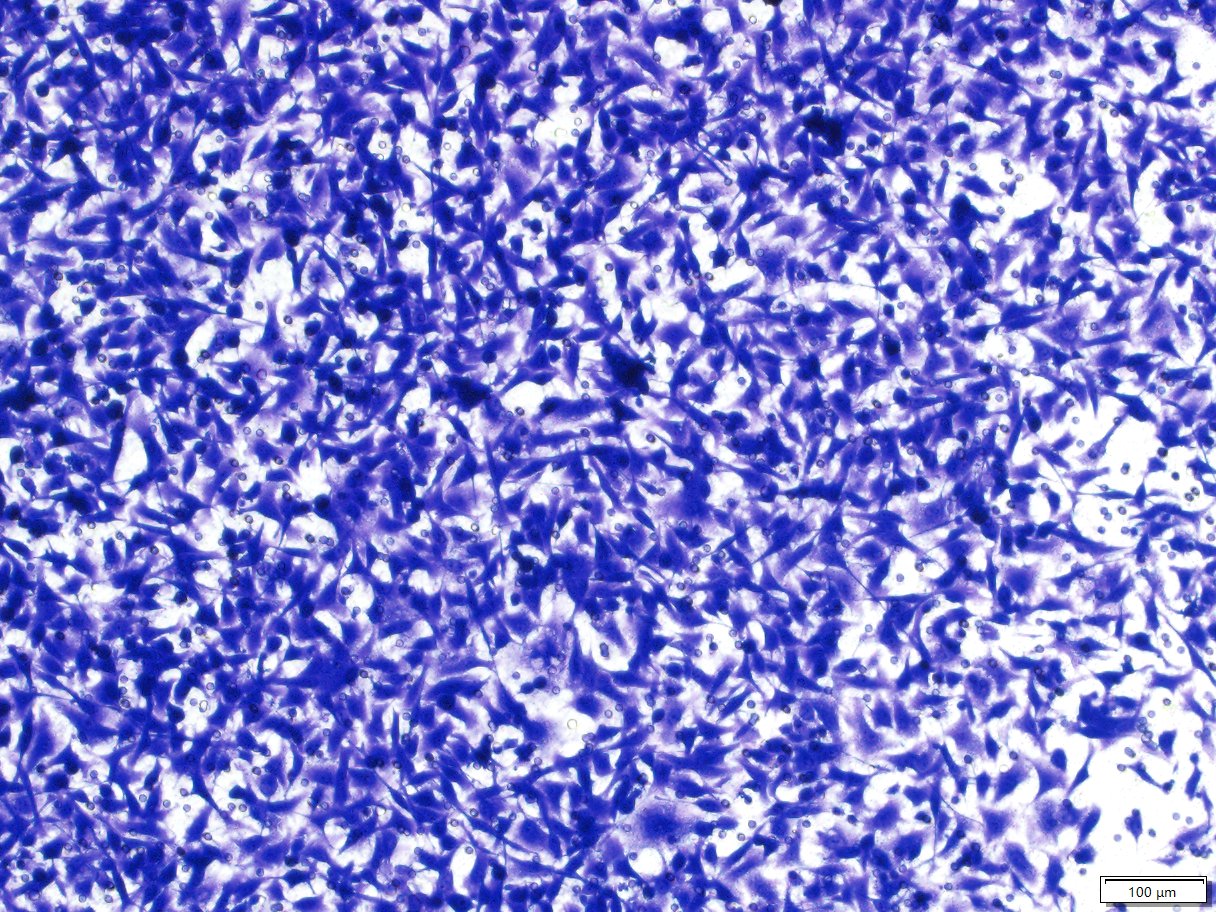

Supplement: Supplemental Information 6 [file peerj-cs-09-1651-s006.zip › Dataset 5/2+7.jpg]

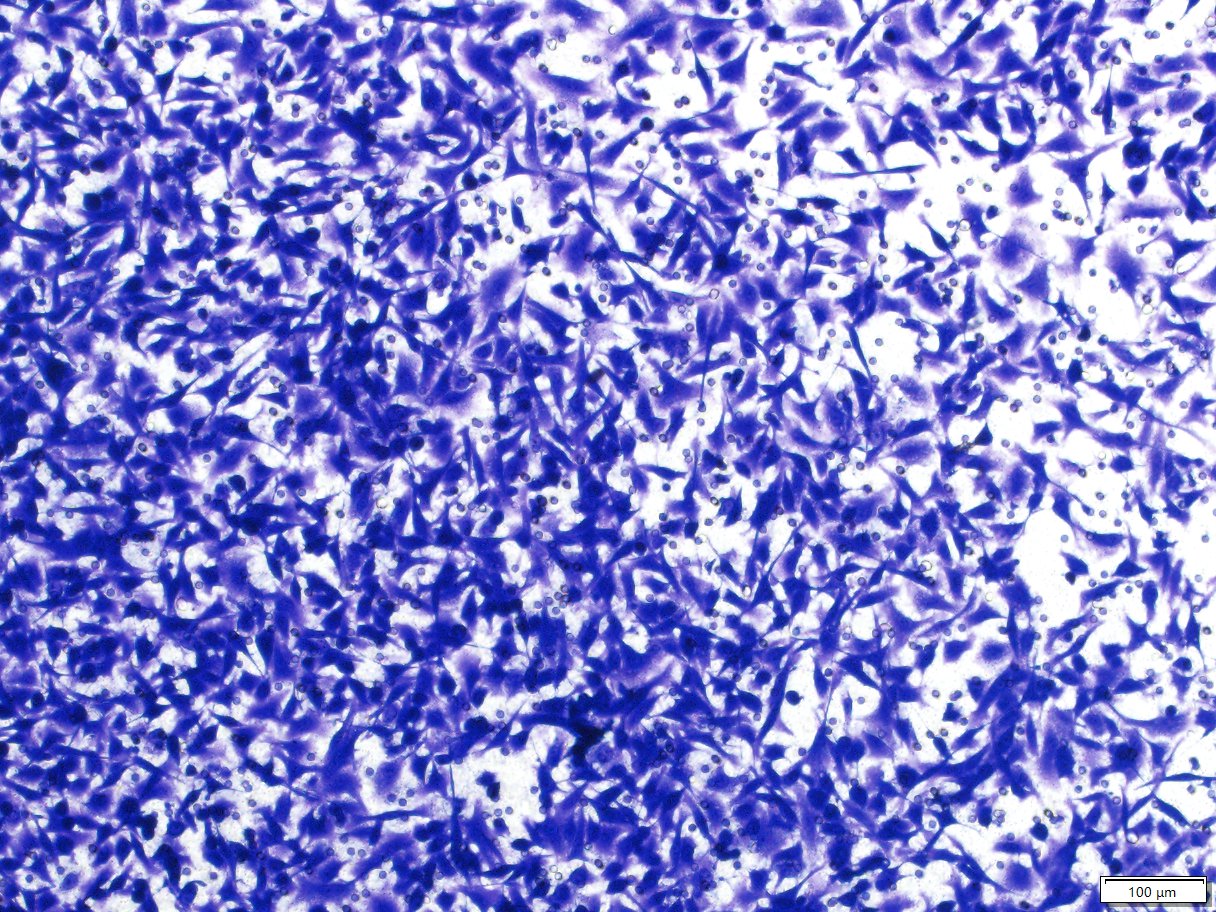

Supplement: Supplemental Information 6 [file peerj-cs-09-1651-s006.zip › Dataset 5/2+8.jpg]

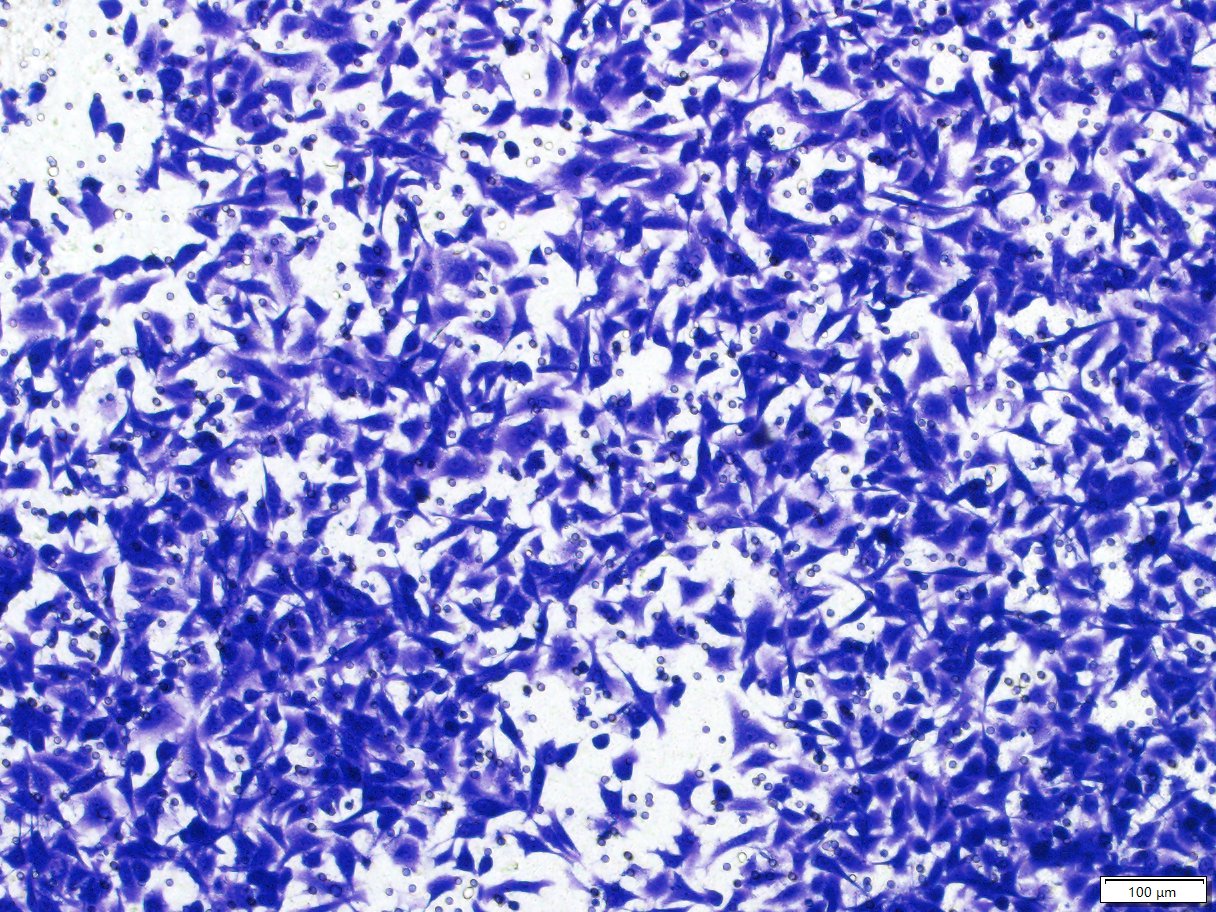

Supplement: Supplemental Information 6 [file peerj-cs-09-1651-s006.zip › Dataset 5/2+9.jpg]

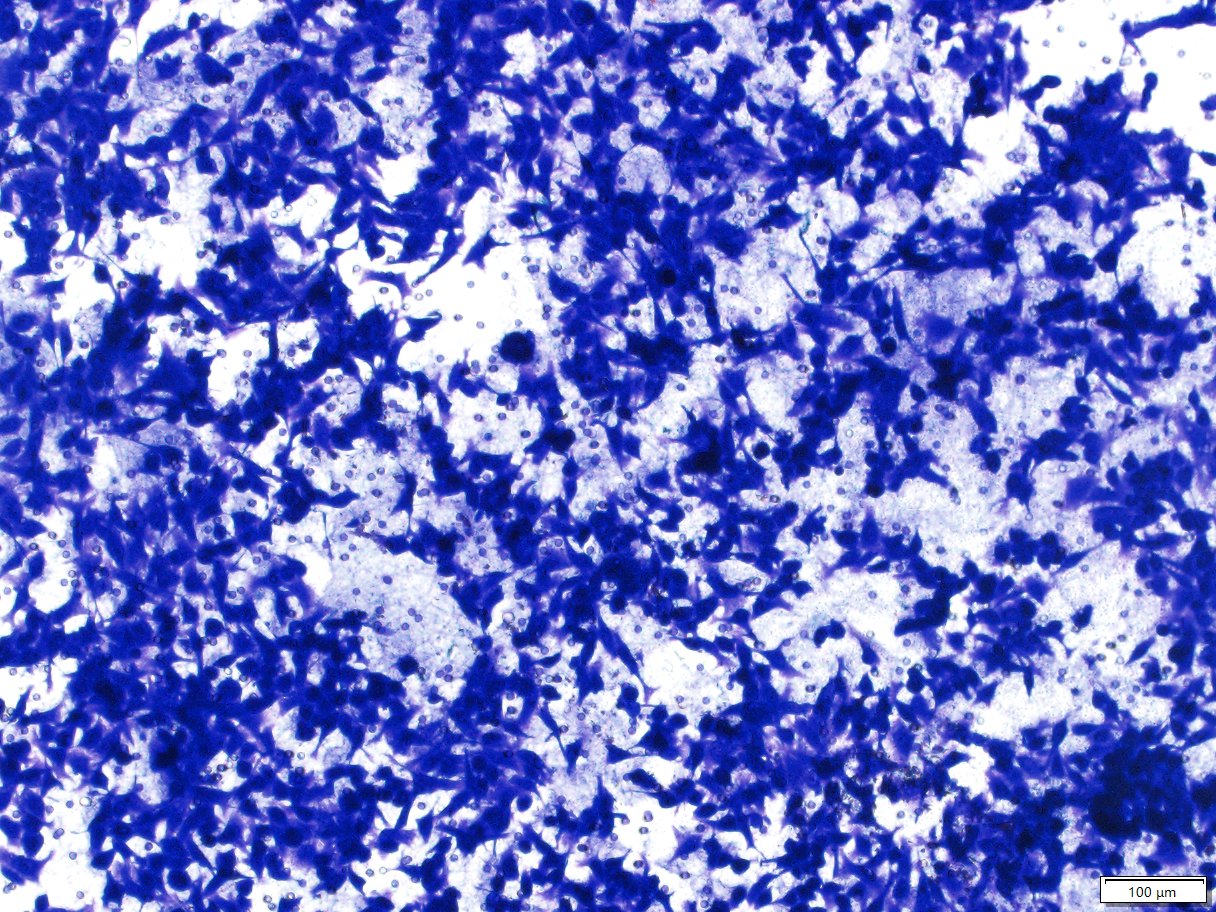

Supplement: Supplemental Information 6 [file peerj-cs-09-1651-s006.zip › Dataset 5/2-1.jpg]

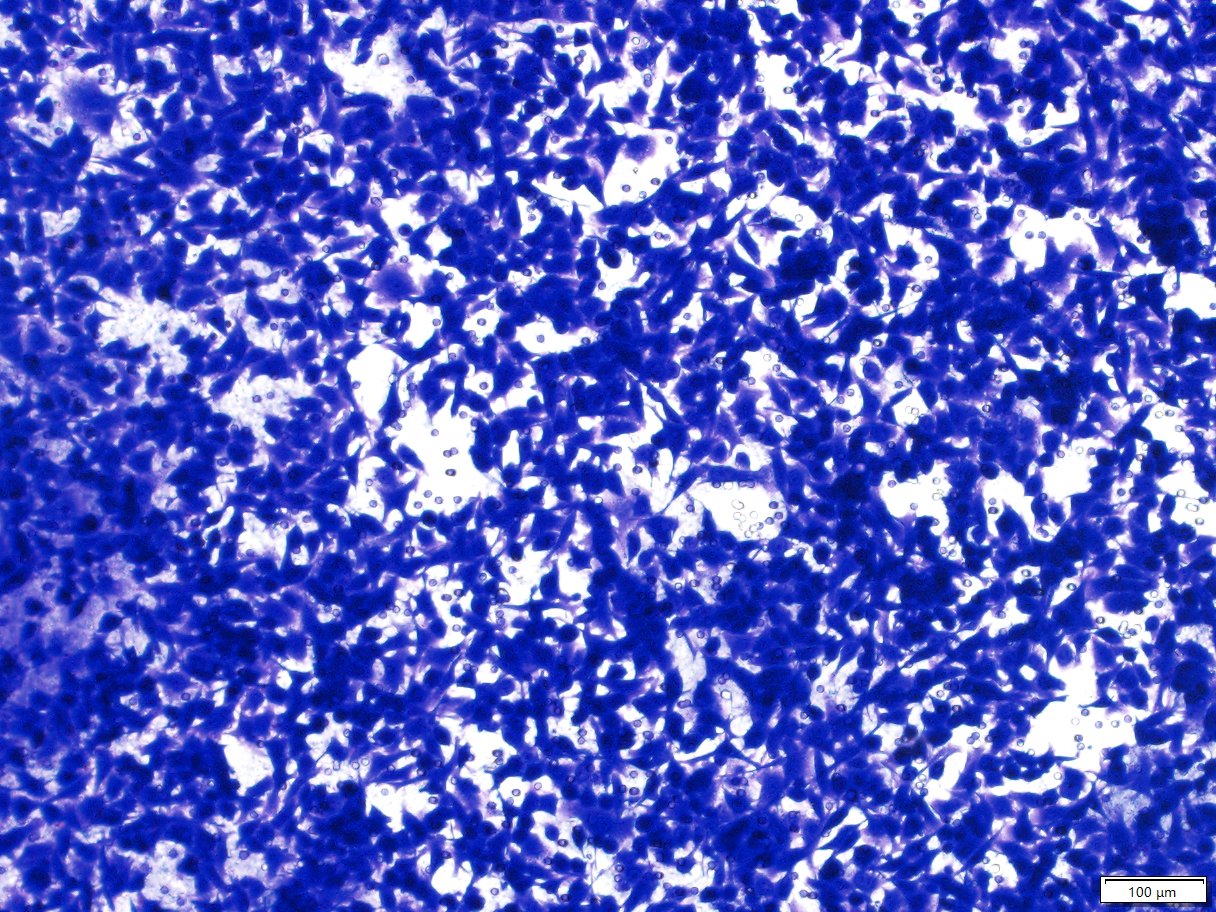

Supplement: Supplemental Information 6 [file peerj-cs-09-1651-s006.zip › Dataset 5/2-10.jpg]

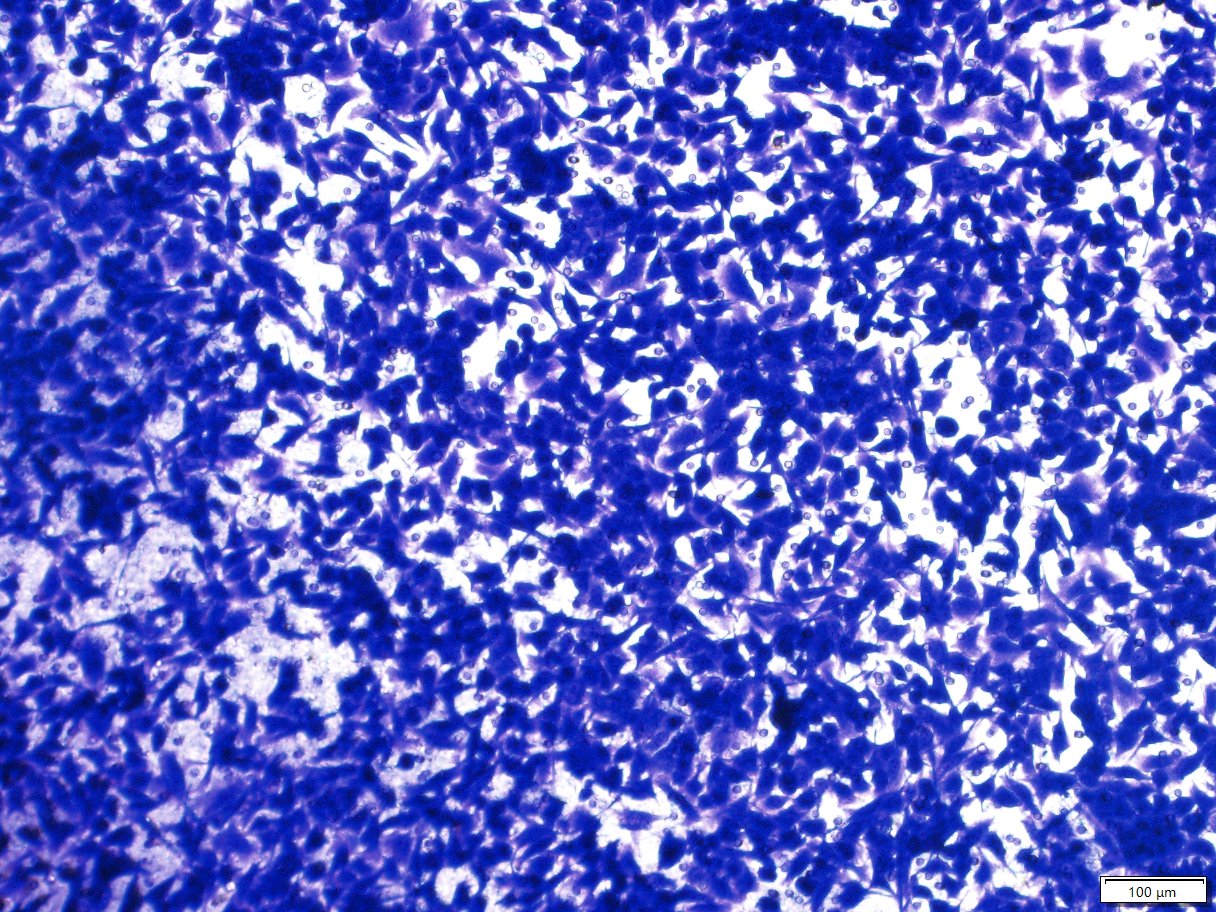

Supplement: Supplemental Information 6 [file peerj-cs-09-1651-s006.zip › Dataset 5/2-11.jpg]

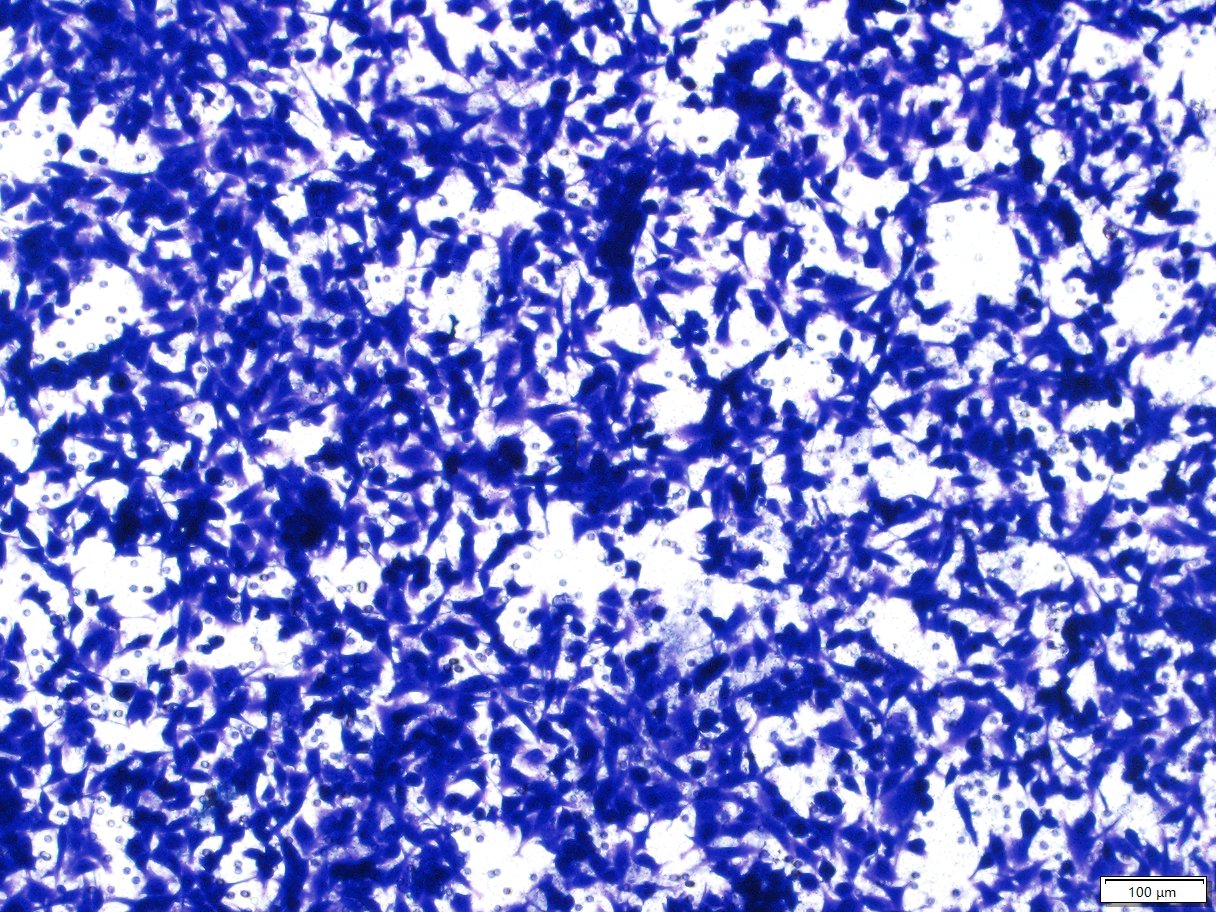

Supplement: Supplemental Information 6 [file peerj-cs-09-1651-s006.zip › Dataset 5/2-2.jpg]

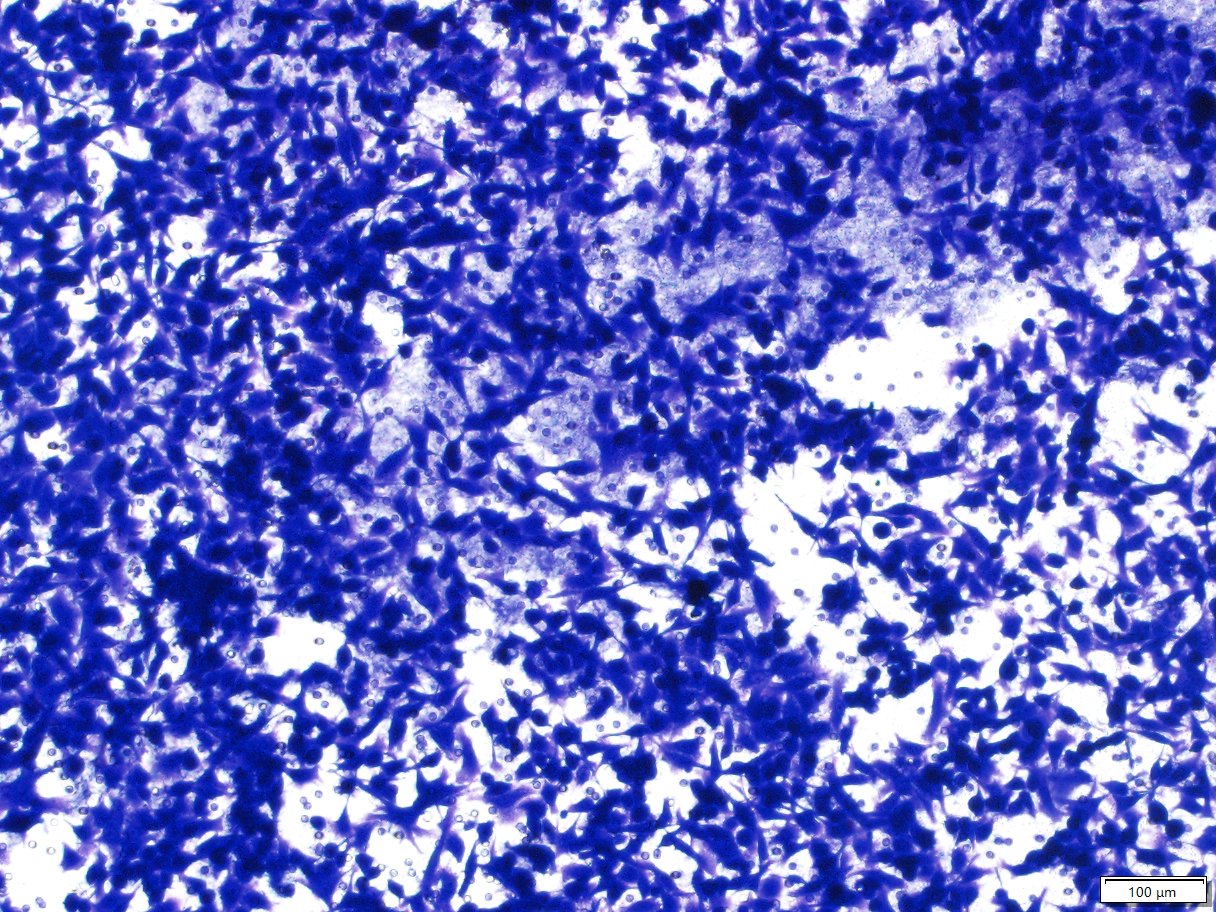

Supplement: Supplemental Information 6 [file peerj-cs-09-1651-s006.zip › Dataset 5/2-3.jpg]

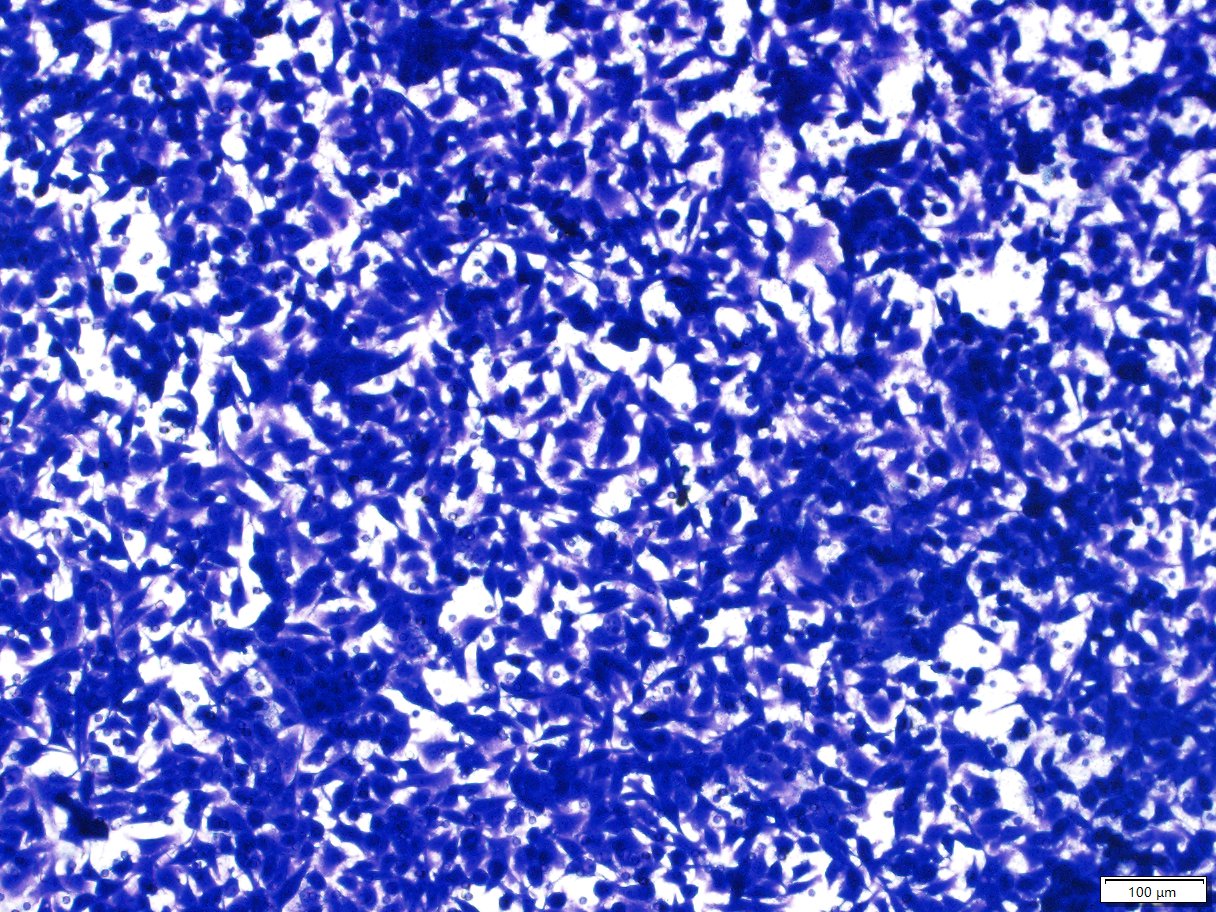

Supplement: Supplemental Information 6 [file peerj-cs-09-1651-s006.zip › Dataset 5/2-4.jpg]

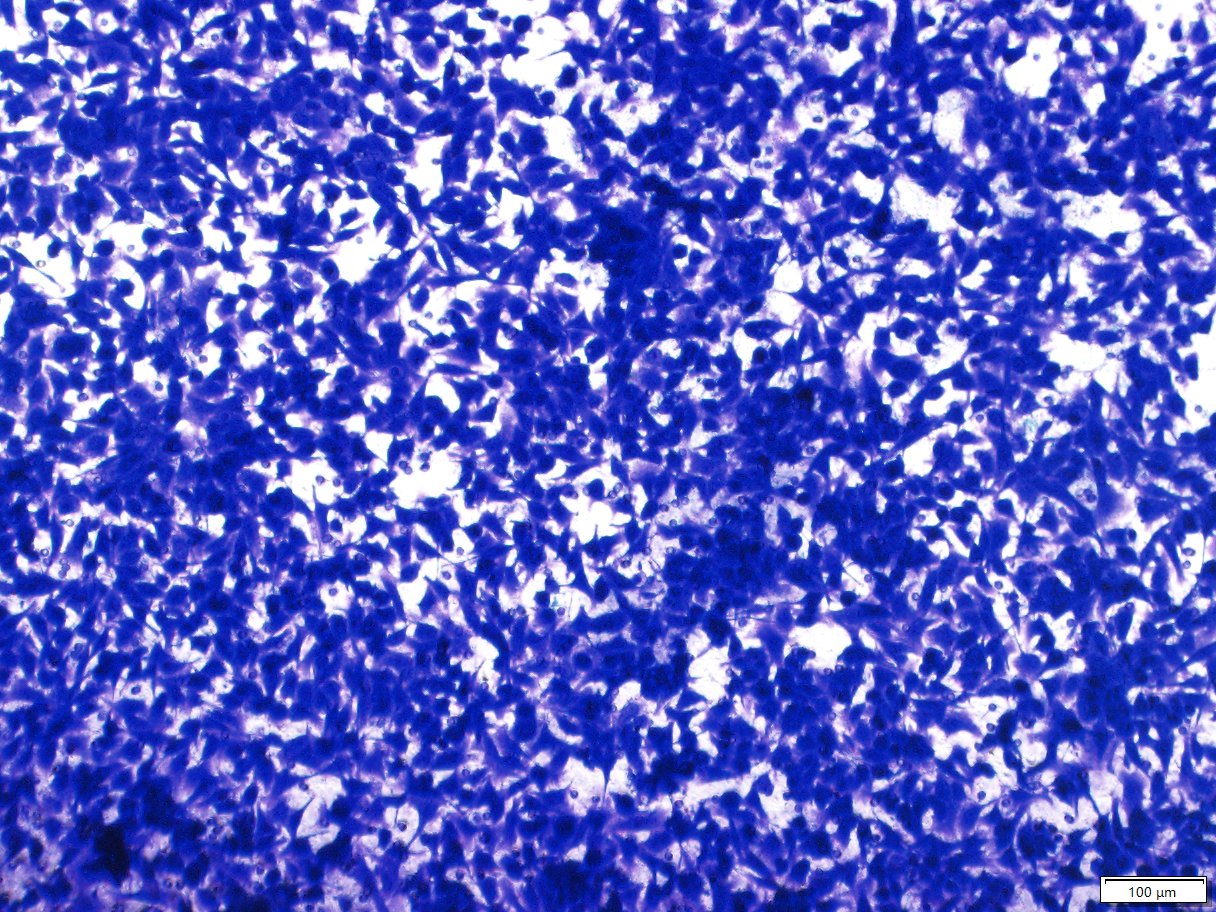

Supplement: Supplemental Information 6 [file peerj-cs-09-1651-s006.zip › Dataset 5/2-5.jpg]

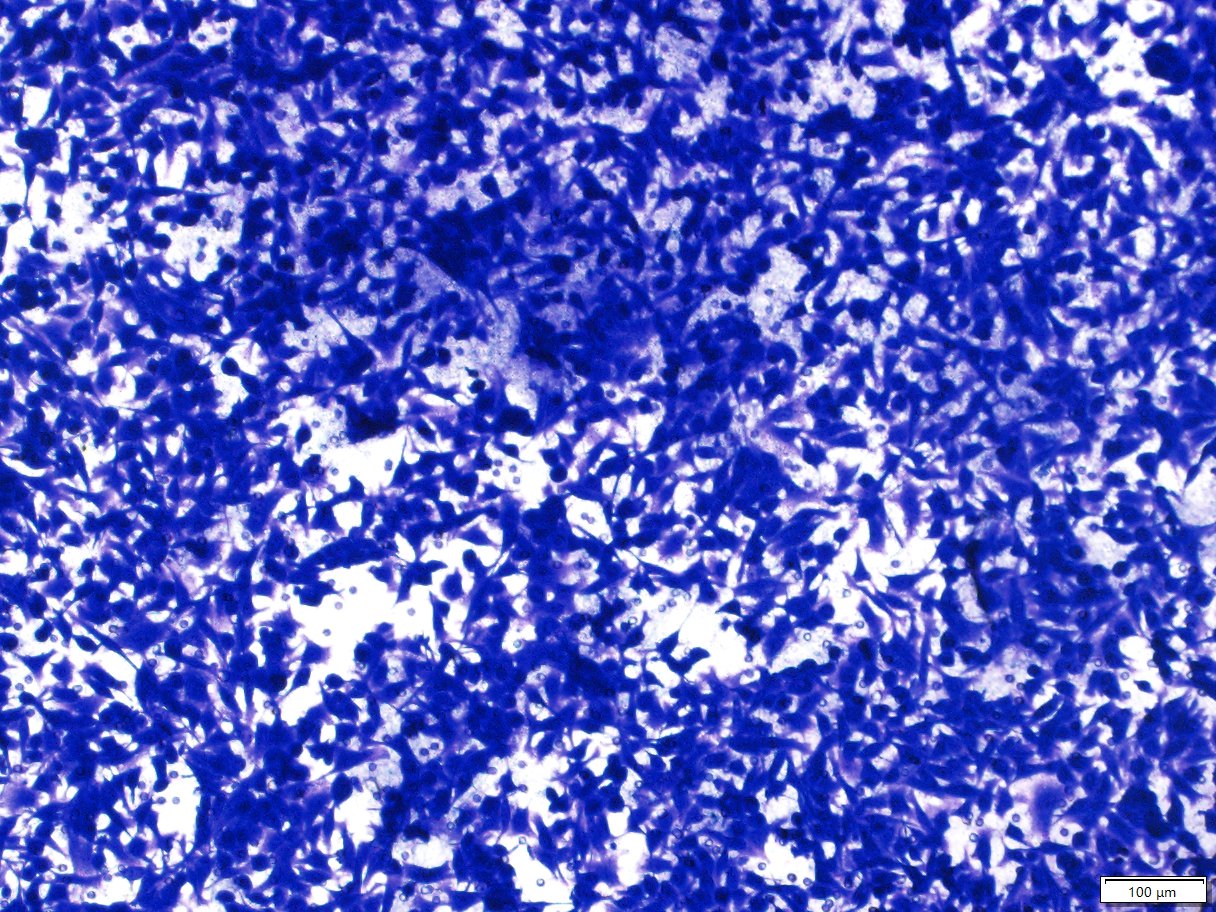

Supplement: Supplemental Information 6 [file peerj-cs-09-1651-s006.zip › Dataset 5/2-6.jpg]

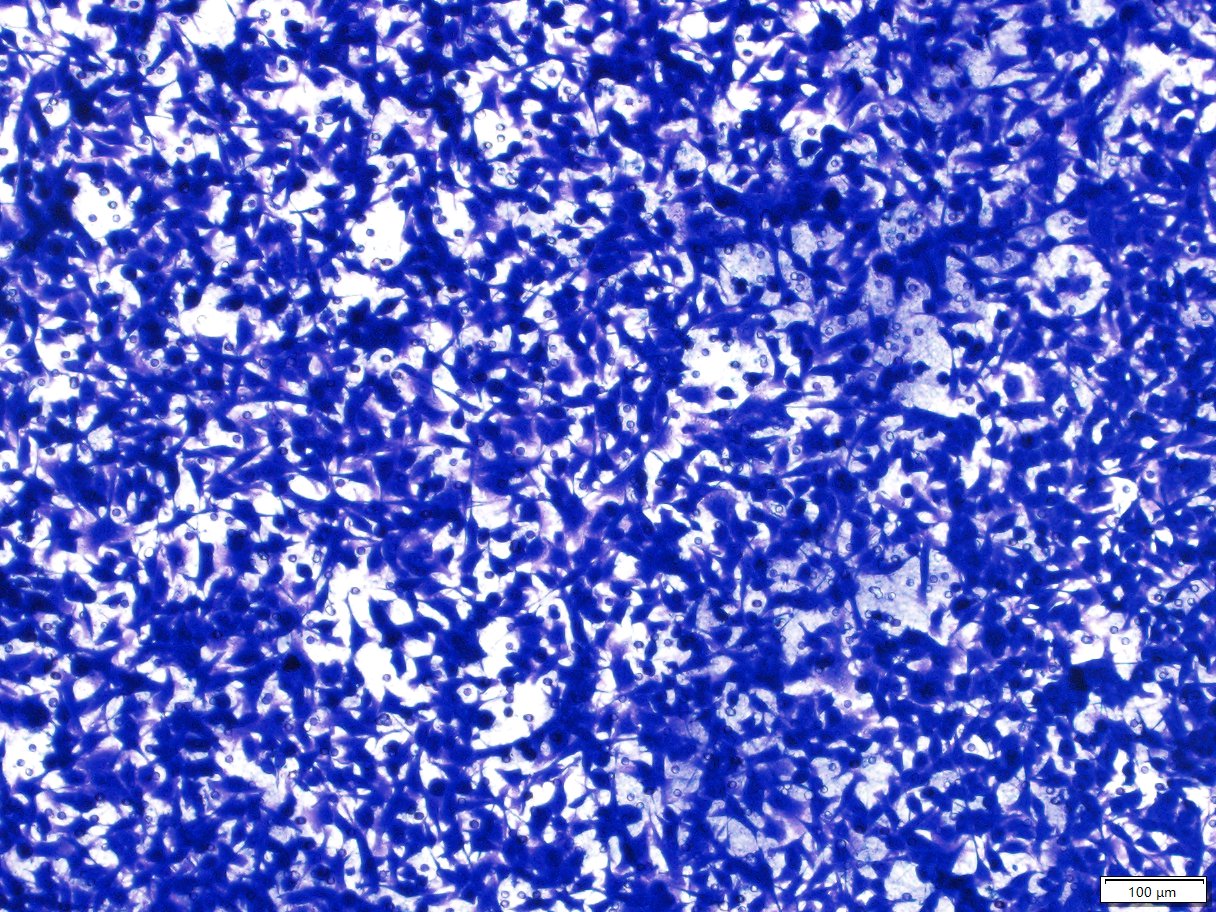

Supplement: Supplemental Information 6 [file peerj-cs-09-1651-s006.zip › Dataset 5/2-7.jpg]

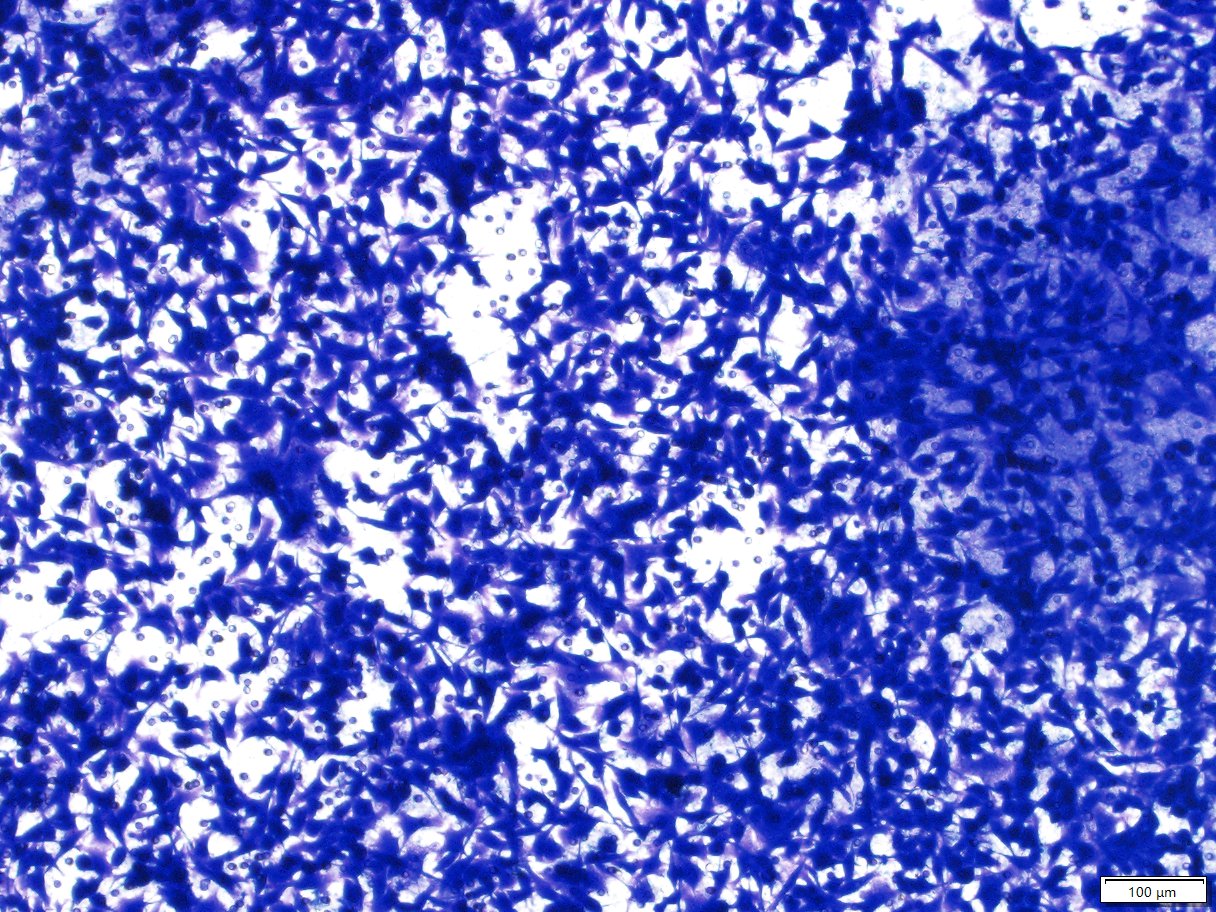

Supplement: Supplemental Information 6 [file peerj-cs-09-1651-s006.zip › Dataset 5/2-8.jpg]

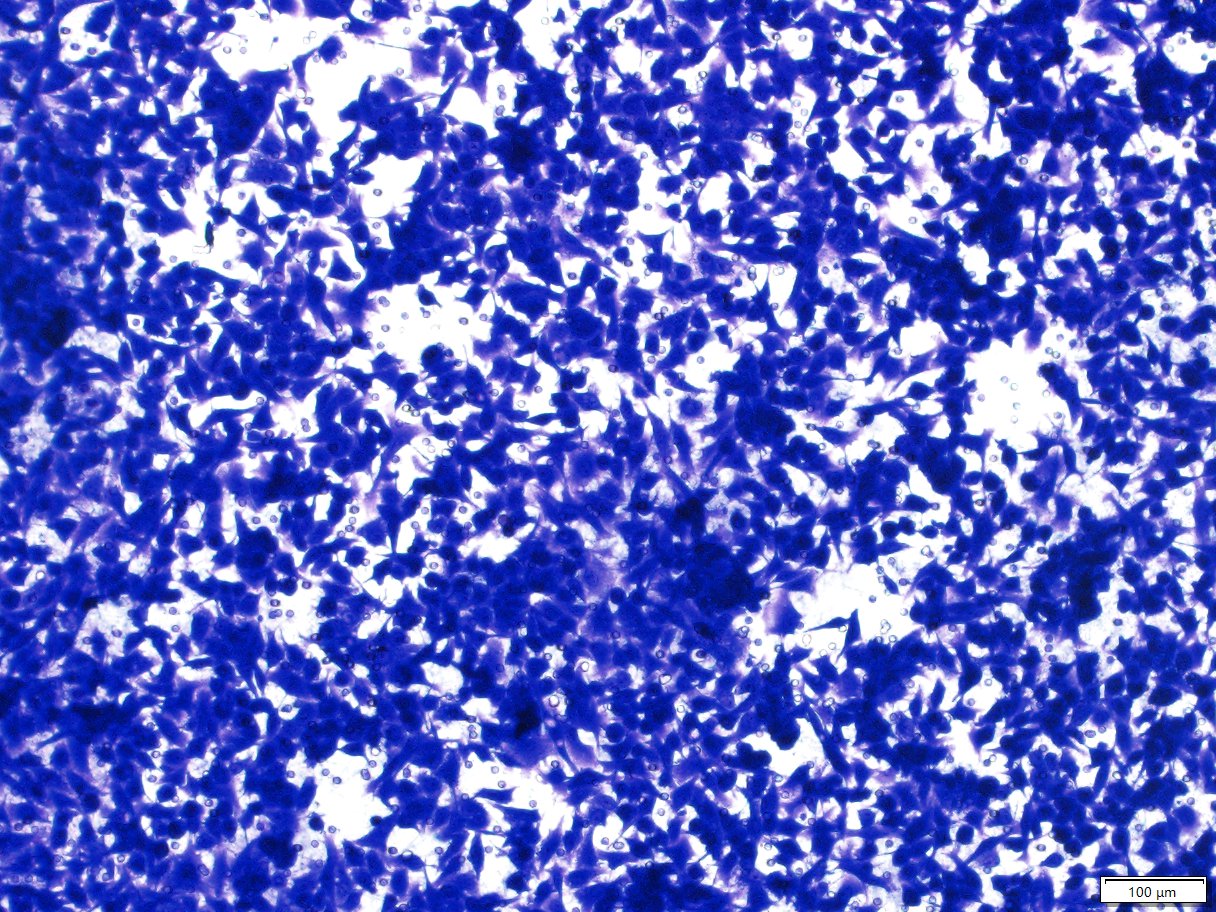

Supplement: Supplemental Information 6 [file peerj-cs-09-1651-s006.zip › Dataset 5/2-9.jpg]

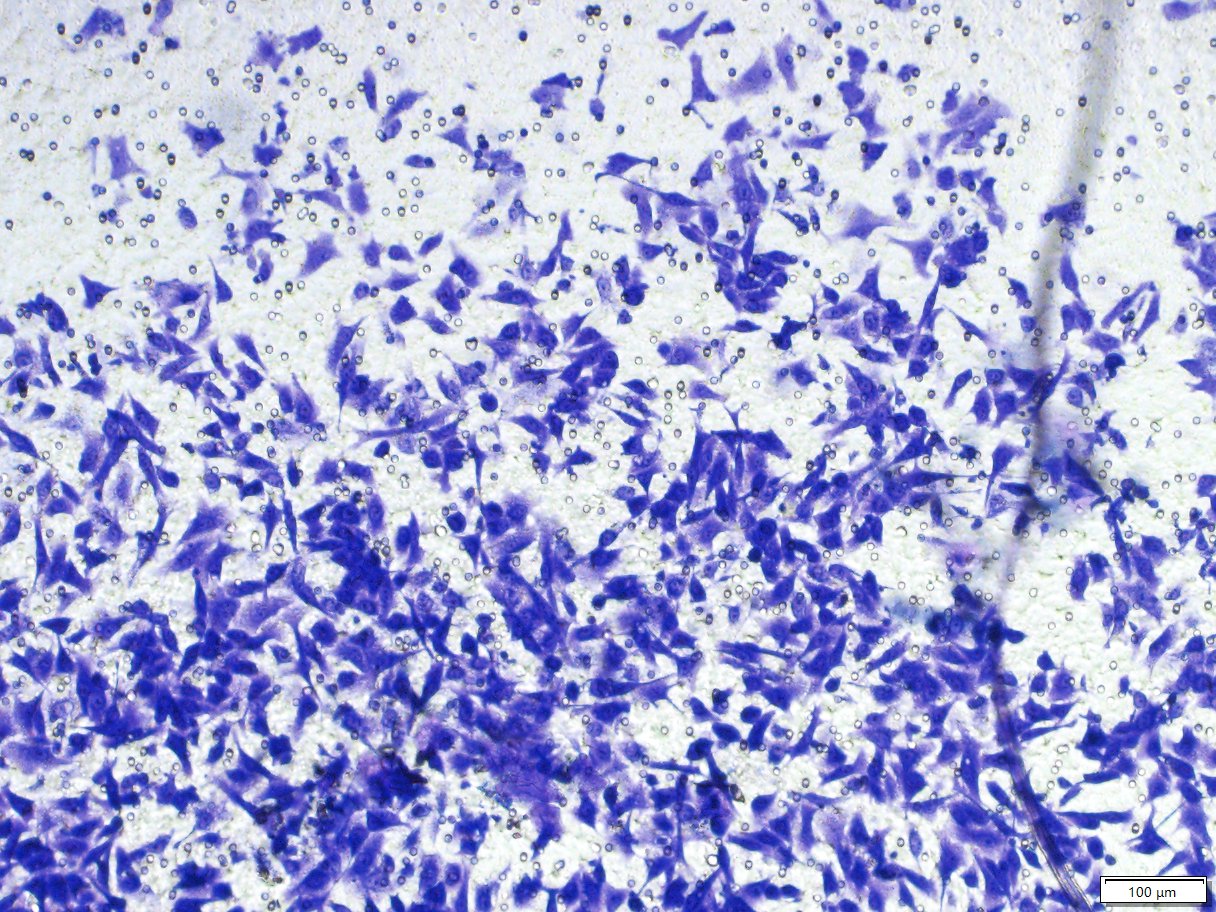

Supplement: Supplemental Information 6 [file peerj-cs-09-1651-s006.zip › Dataset 5/3+1.jpg]

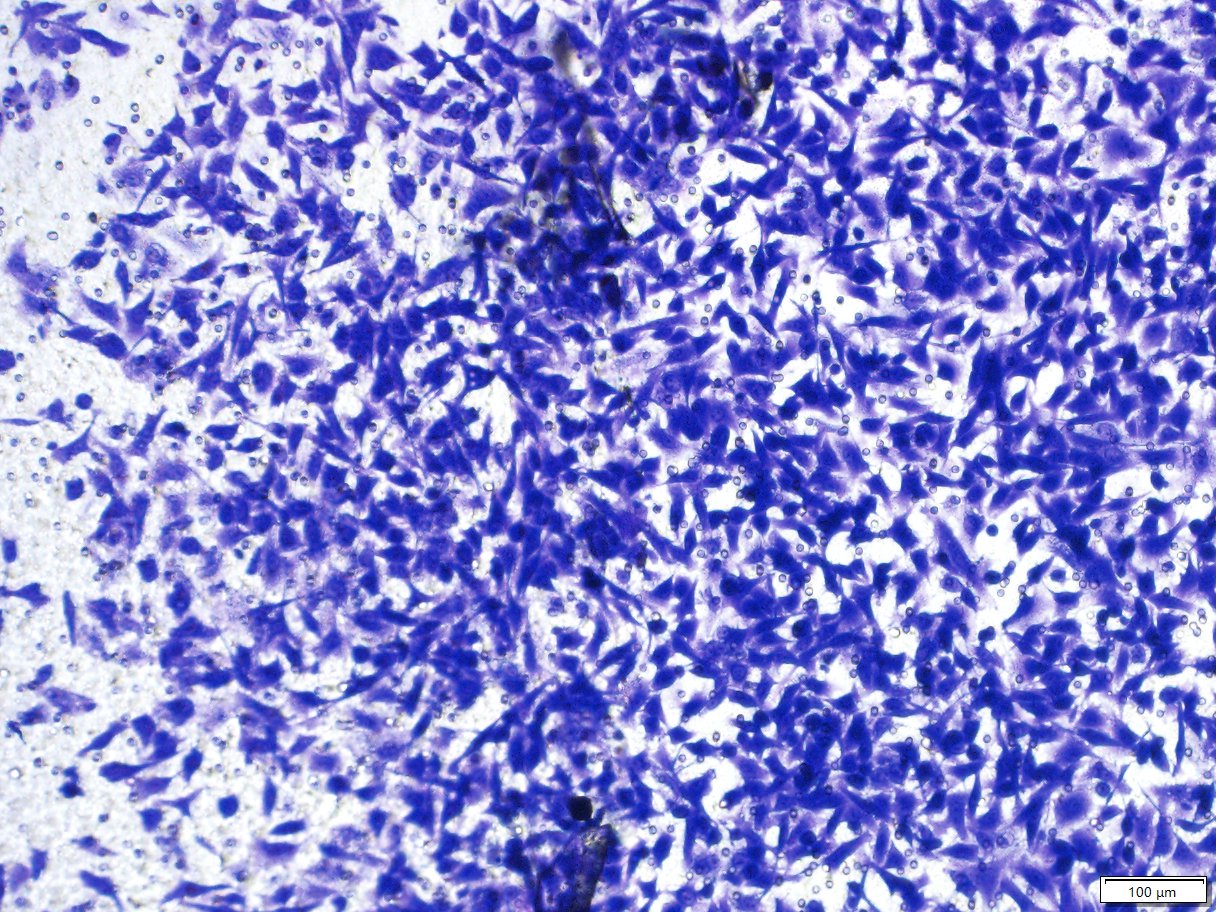

Supplement: Supplemental Information 6 [file peerj-cs-09-1651-s006.zip › Dataset 5/3+10.jpg]

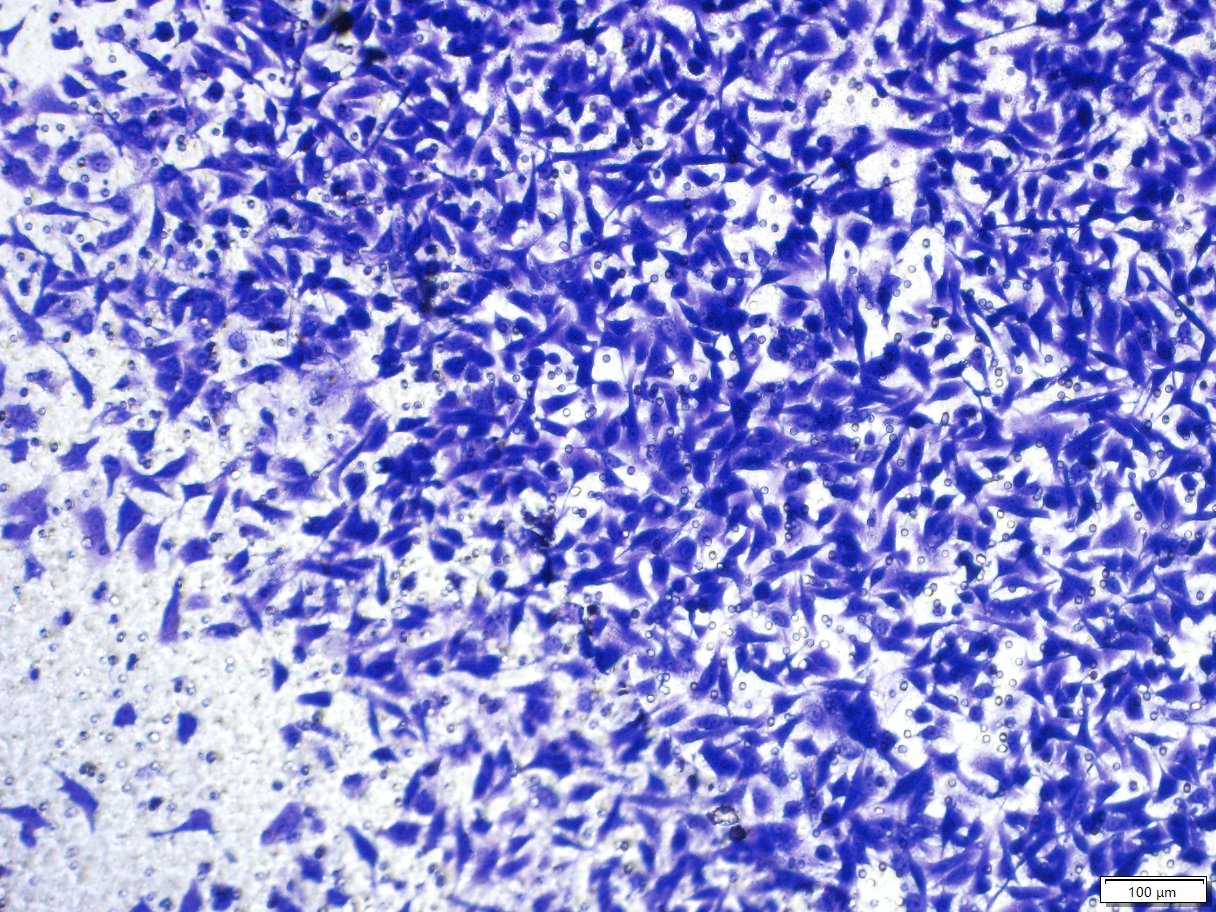

Supplement: Supplemental Information 6 [file peerj-cs-09-1651-s006.zip › Dataset 5/3+11.jpg]

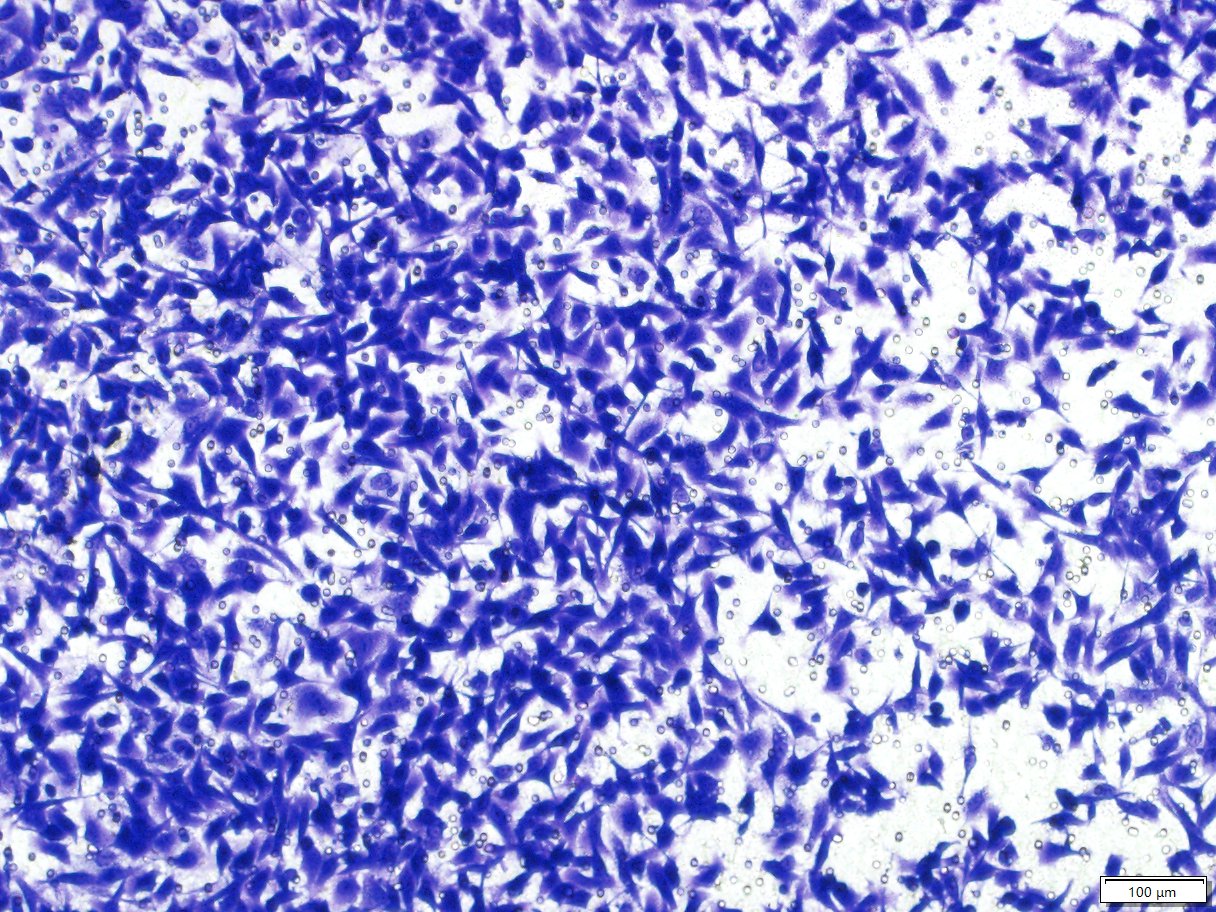

Supplement: Supplemental Information 6 [file peerj-cs-09-1651-s006.zip › Dataset 5/3+2.jpg]

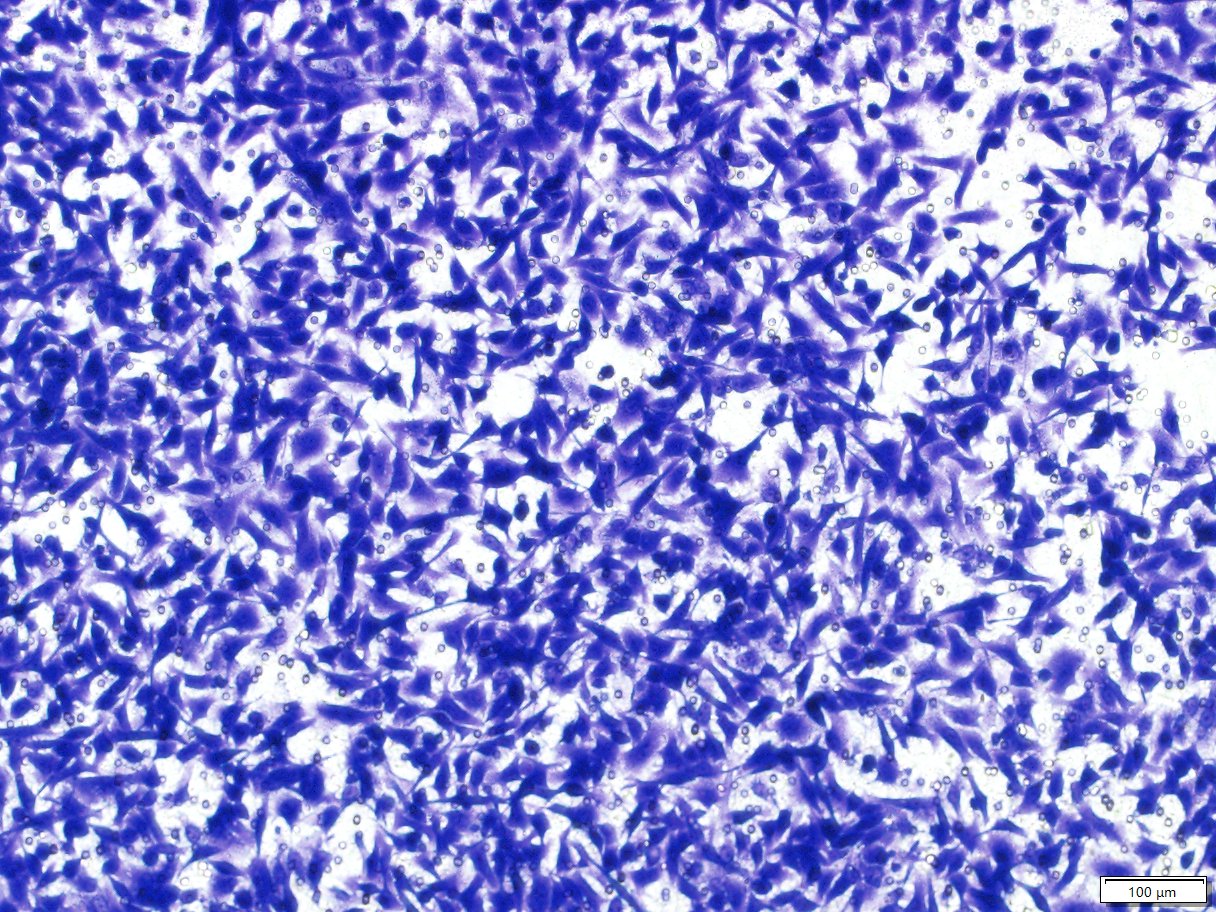

Supplement: Supplemental Information 6 [file peerj-cs-09-1651-s006.zip › Dataset 5/3+3.jpg]

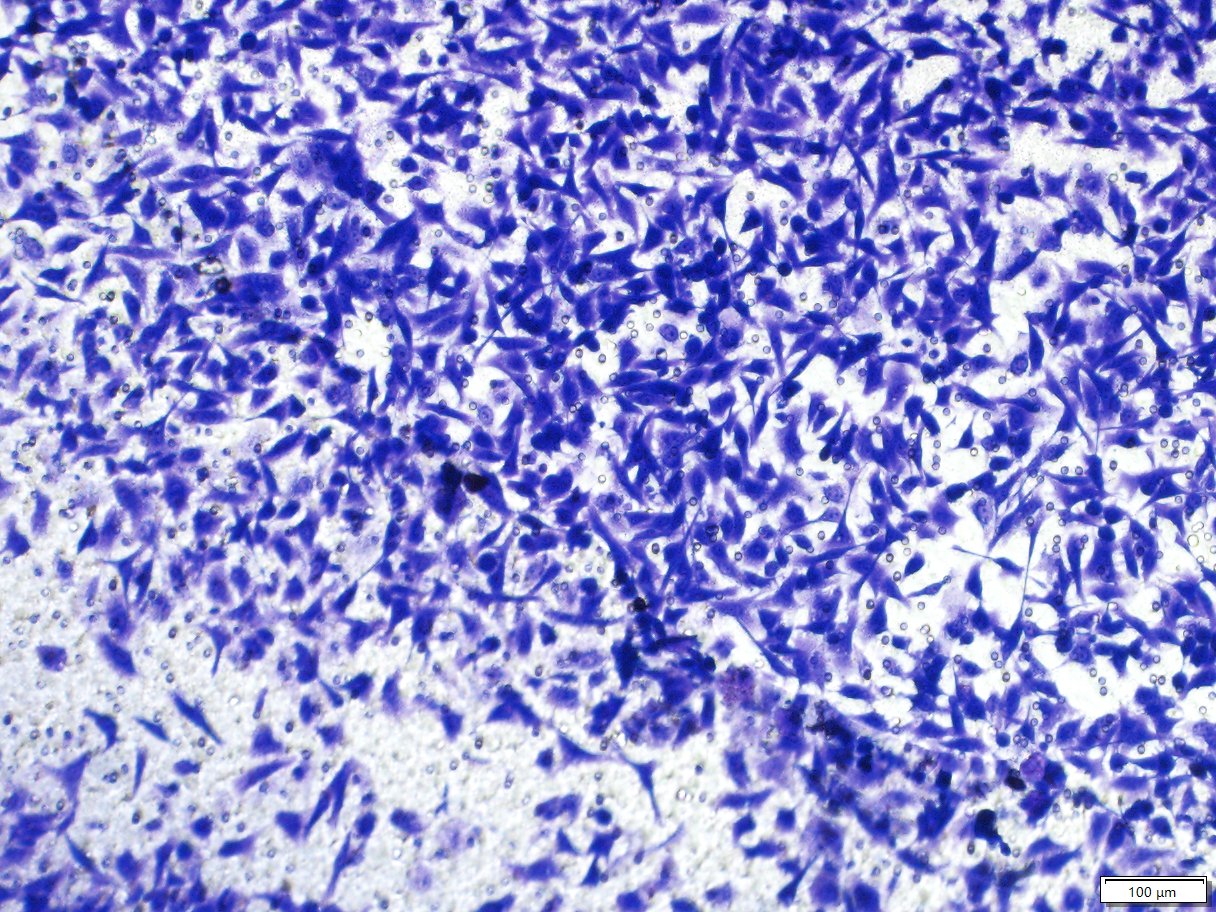

Supplement: Supplemental Information 6 [file peerj-cs-09-1651-s006.zip › Dataset 5/3+4.jpg]

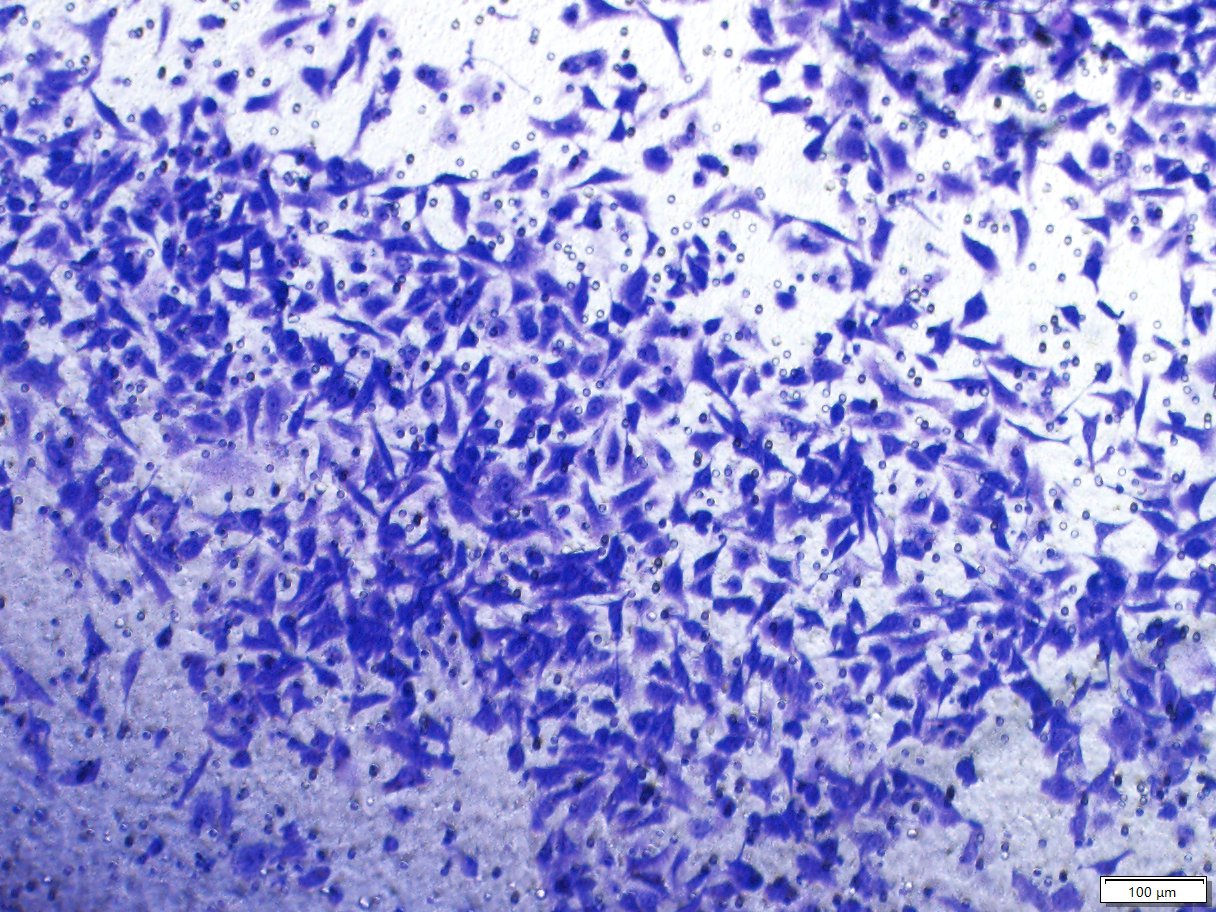

Supplement: Supplemental Information 6 [file peerj-cs-09-1651-s006.zip › Dataset 5/3+5.jpg]

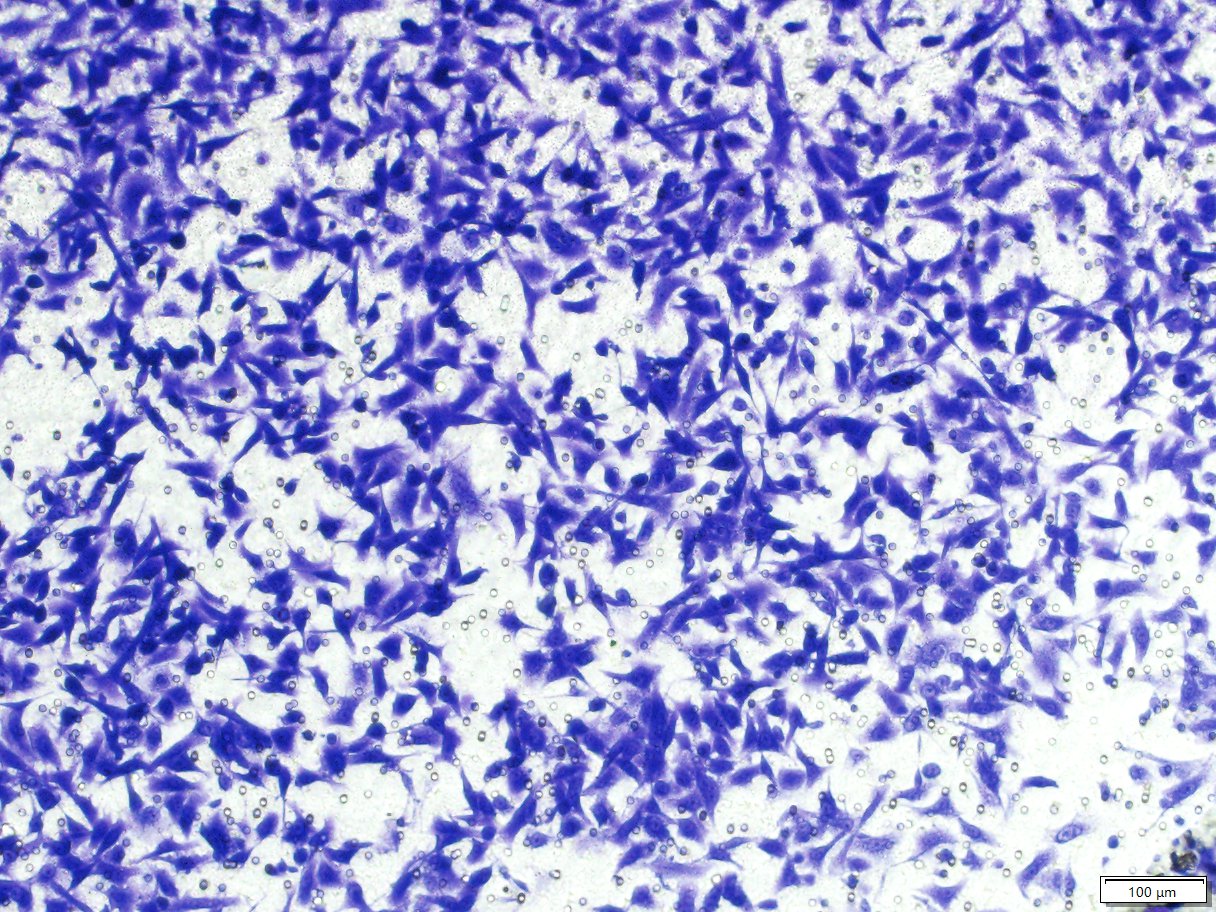

Supplement: Supplemental Information 6 [file peerj-cs-09-1651-s006.zip › Dataset 5/3+6.jpg]

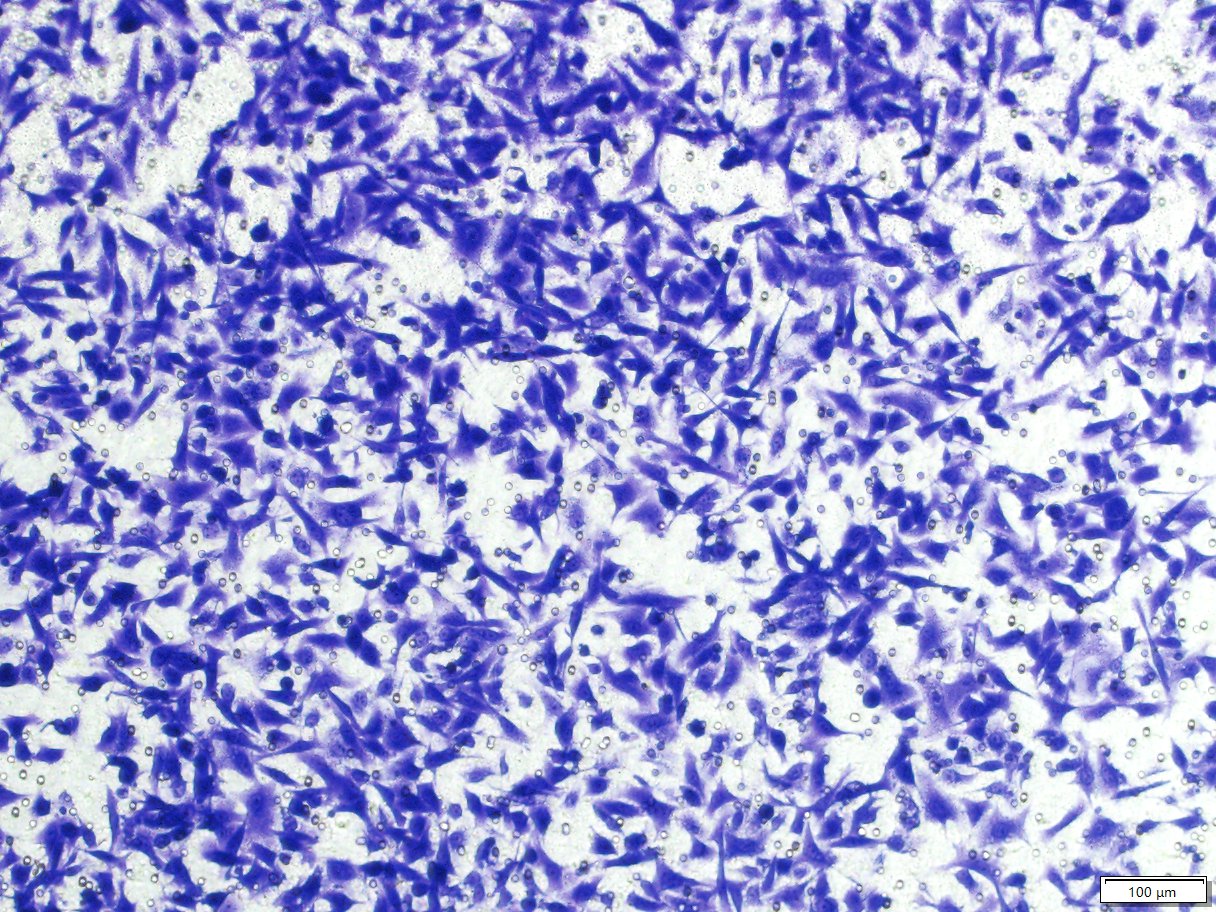

Supplement: Supplemental Information 6 [file peerj-cs-09-1651-s006.zip › Dataset 5/3+7.jpg]

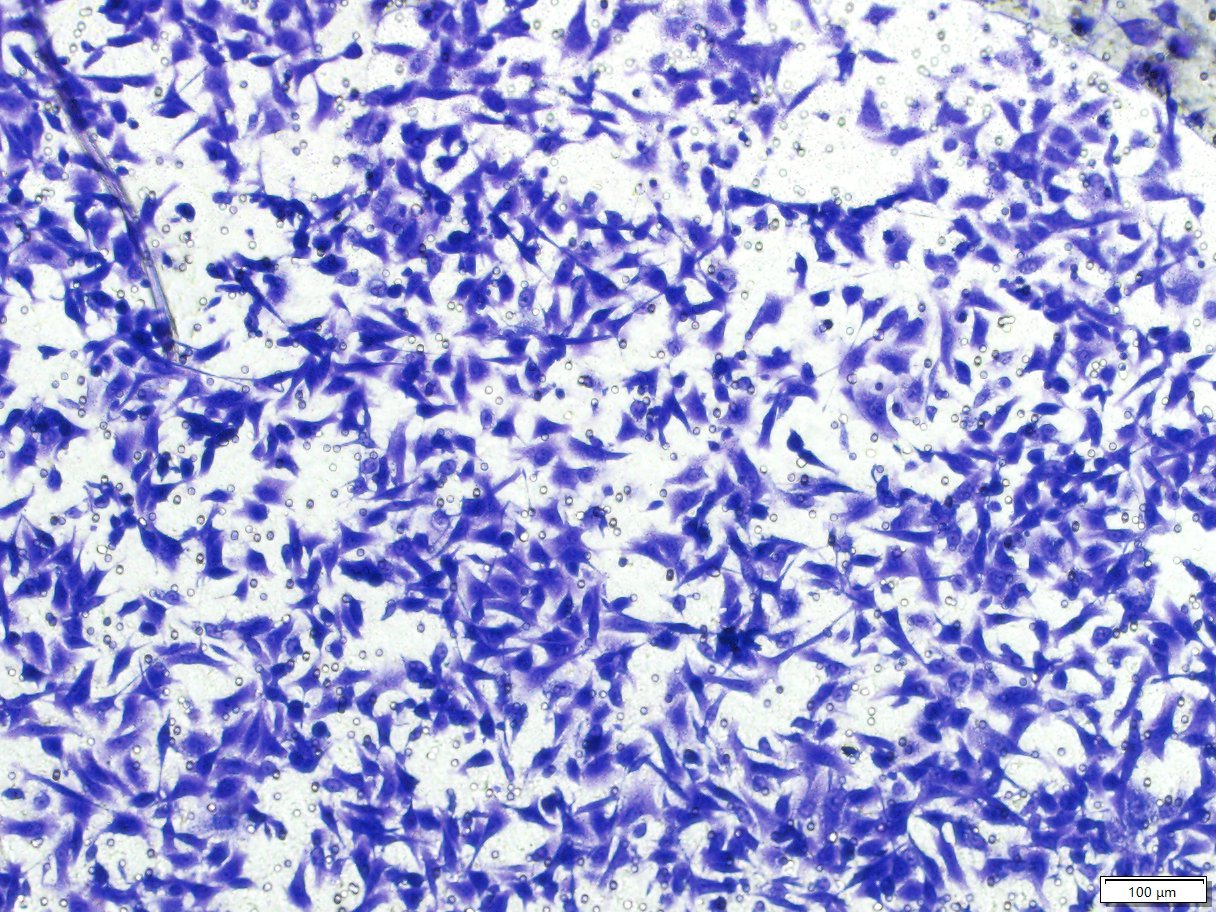

Supplement: Supplemental Information 6 [file peerj-cs-09-1651-s006.zip › Dataset 5/3+8.jpg]

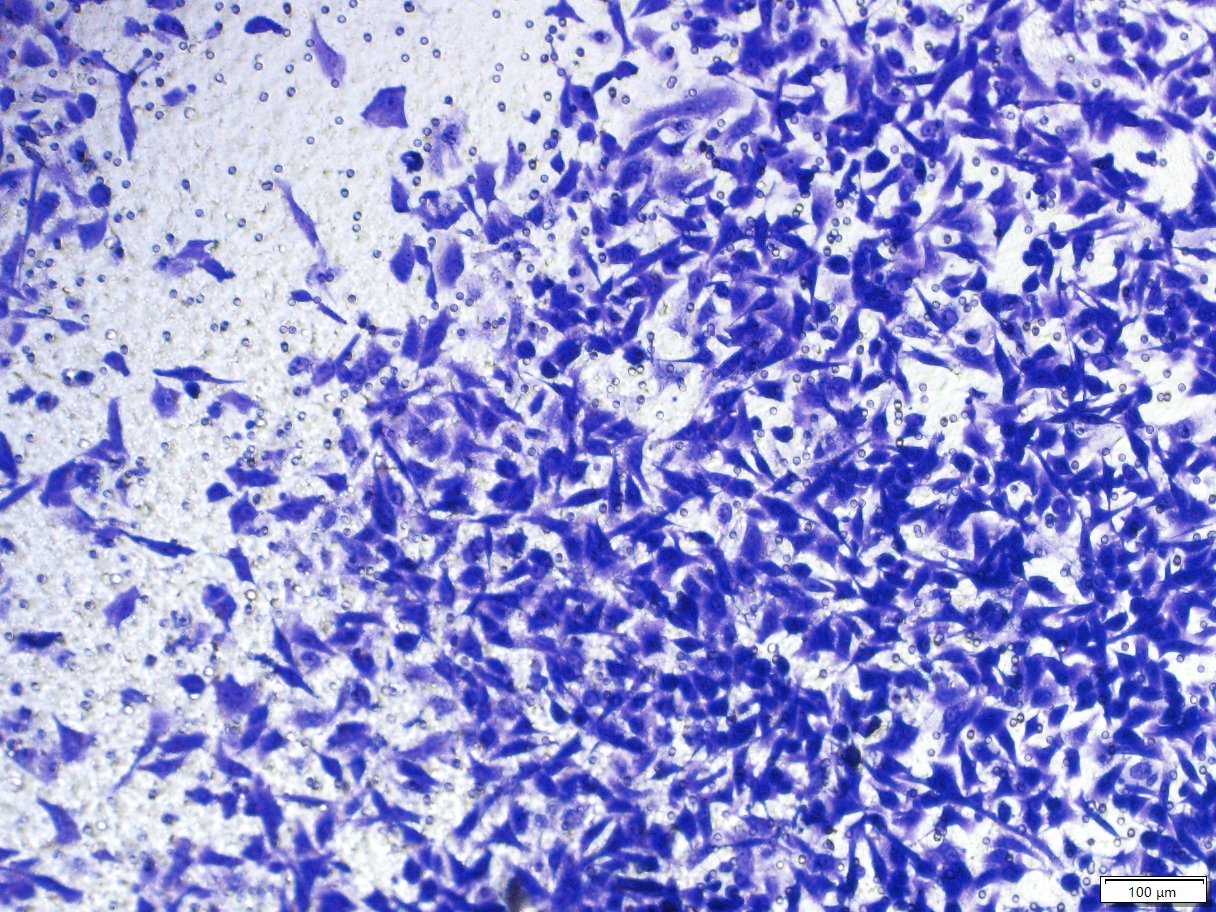

Supplement: Supplemental Information 6 [file peerj-cs-09-1651-s006.zip › Dataset 5/3+9.jpg]

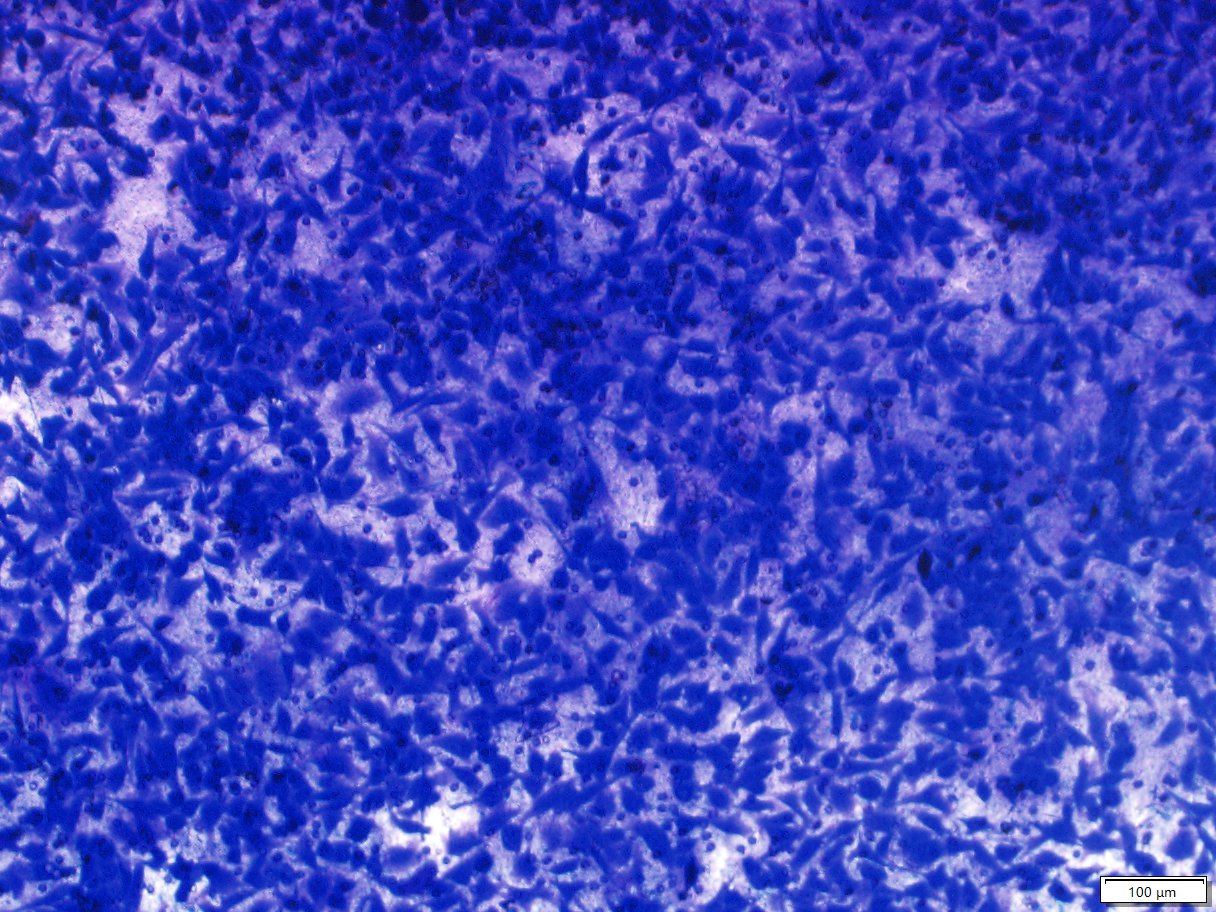

Supplement: Supplemental Information 6 [file peerj-cs-09-1651-s006.zip › Dataset 5/3-1.jpg]

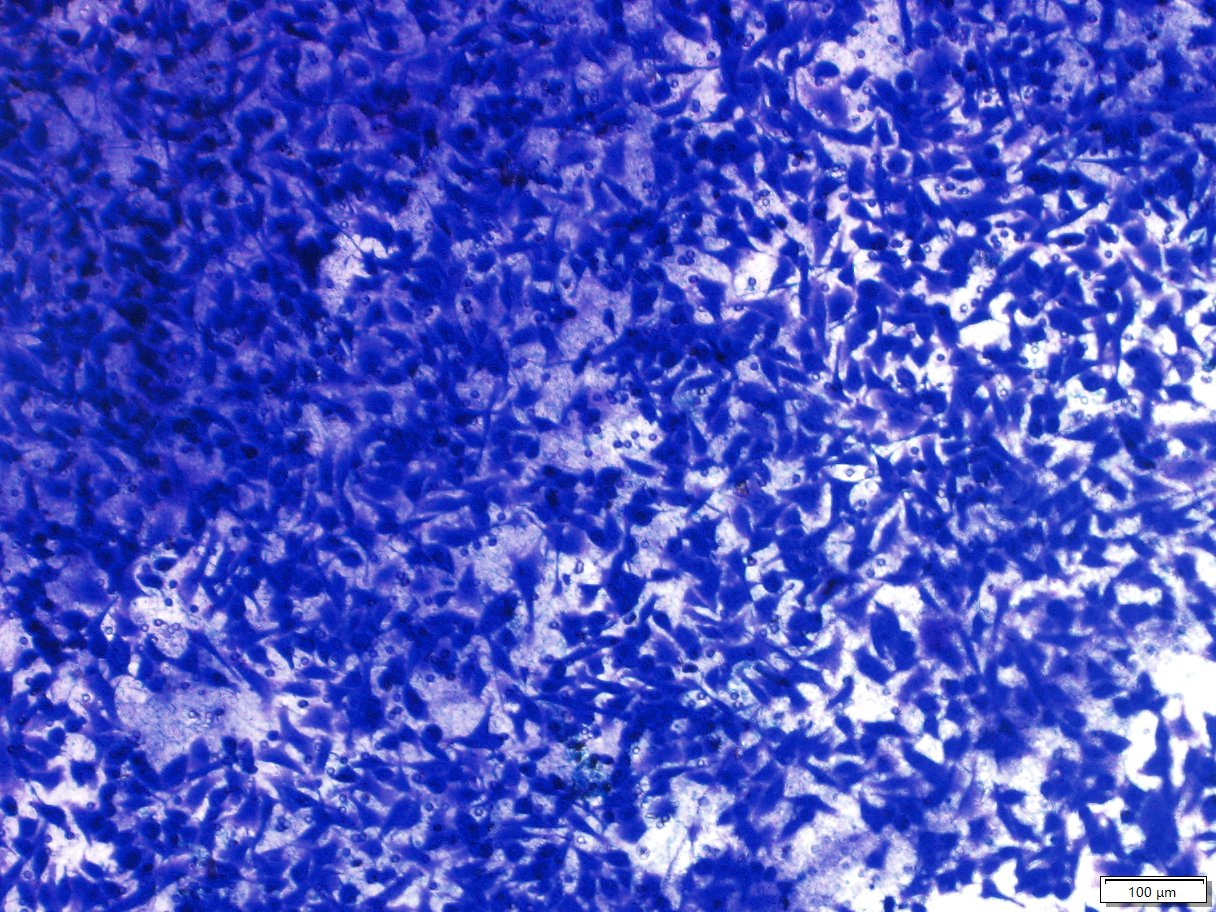

Supplement: Supplemental Information 6 [file peerj-cs-09-1651-s006.zip › Dataset 5/3-10.jpg]

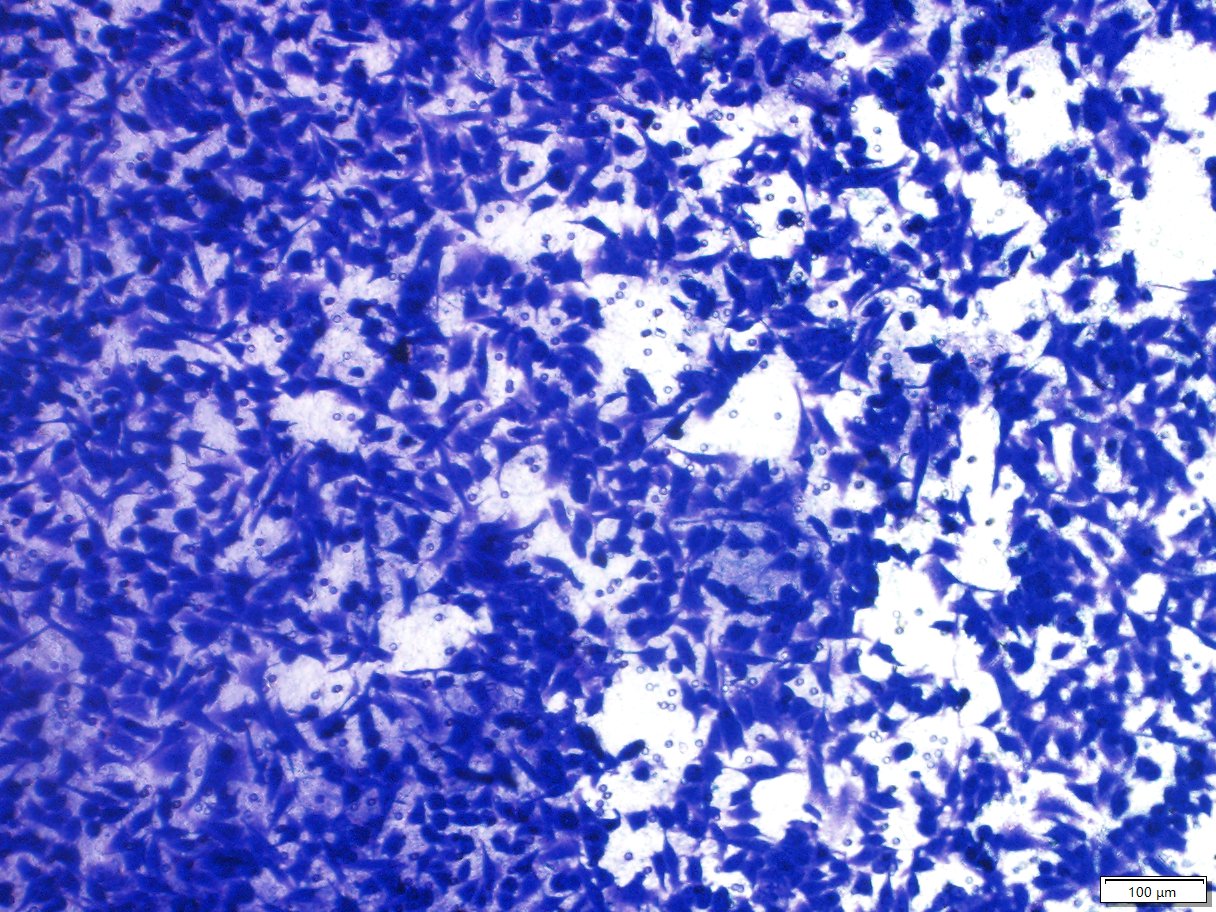

Supplement: Supplemental Information 6 [file peerj-cs-09-1651-s006.zip › Dataset 5/3-11.jpg]

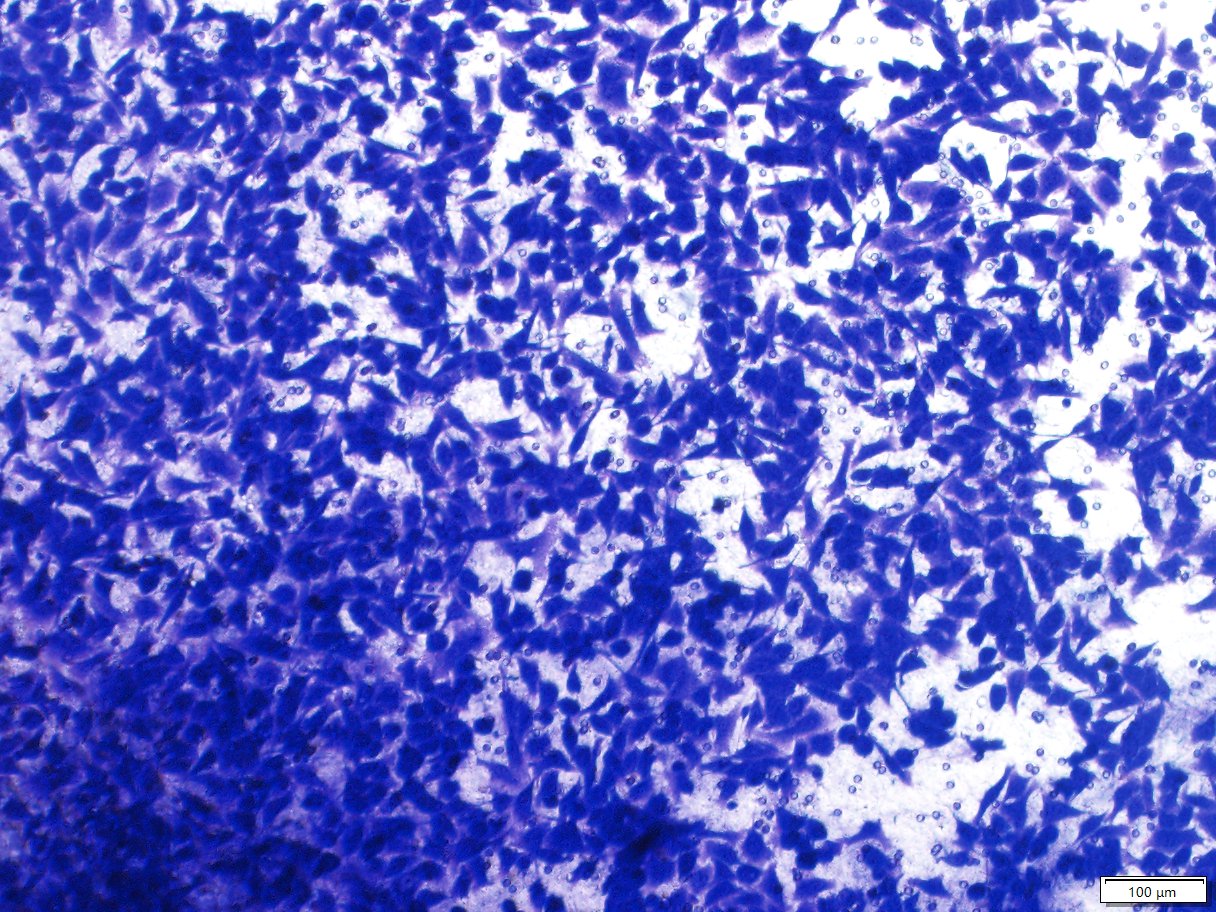

Supplement: Supplemental Information 6 [file peerj-cs-09-1651-s006.zip › Dataset 5/3-12.jpg]

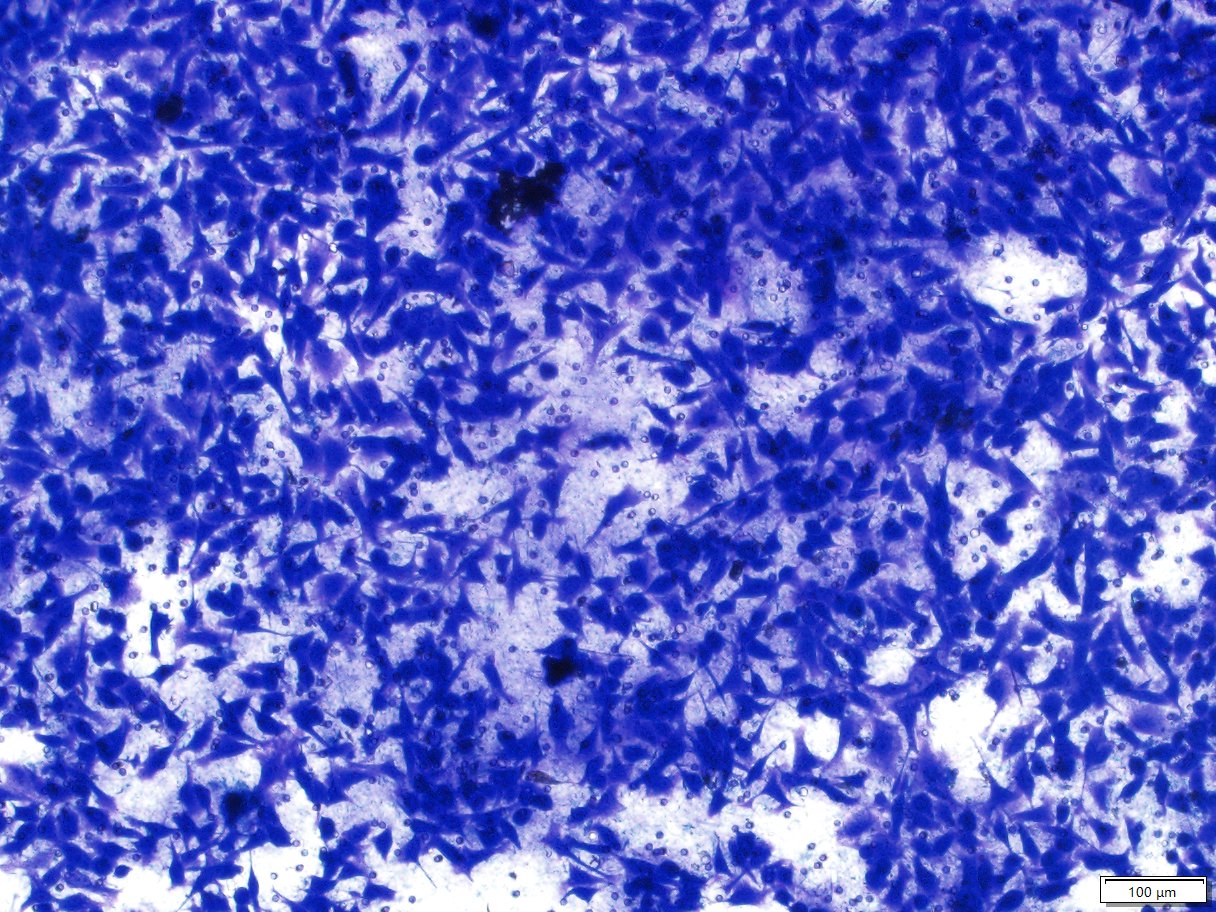

Supplement: Supplemental Information 6 [file peerj-cs-09-1651-s006.zip › Dataset 5/3-2.jpg]

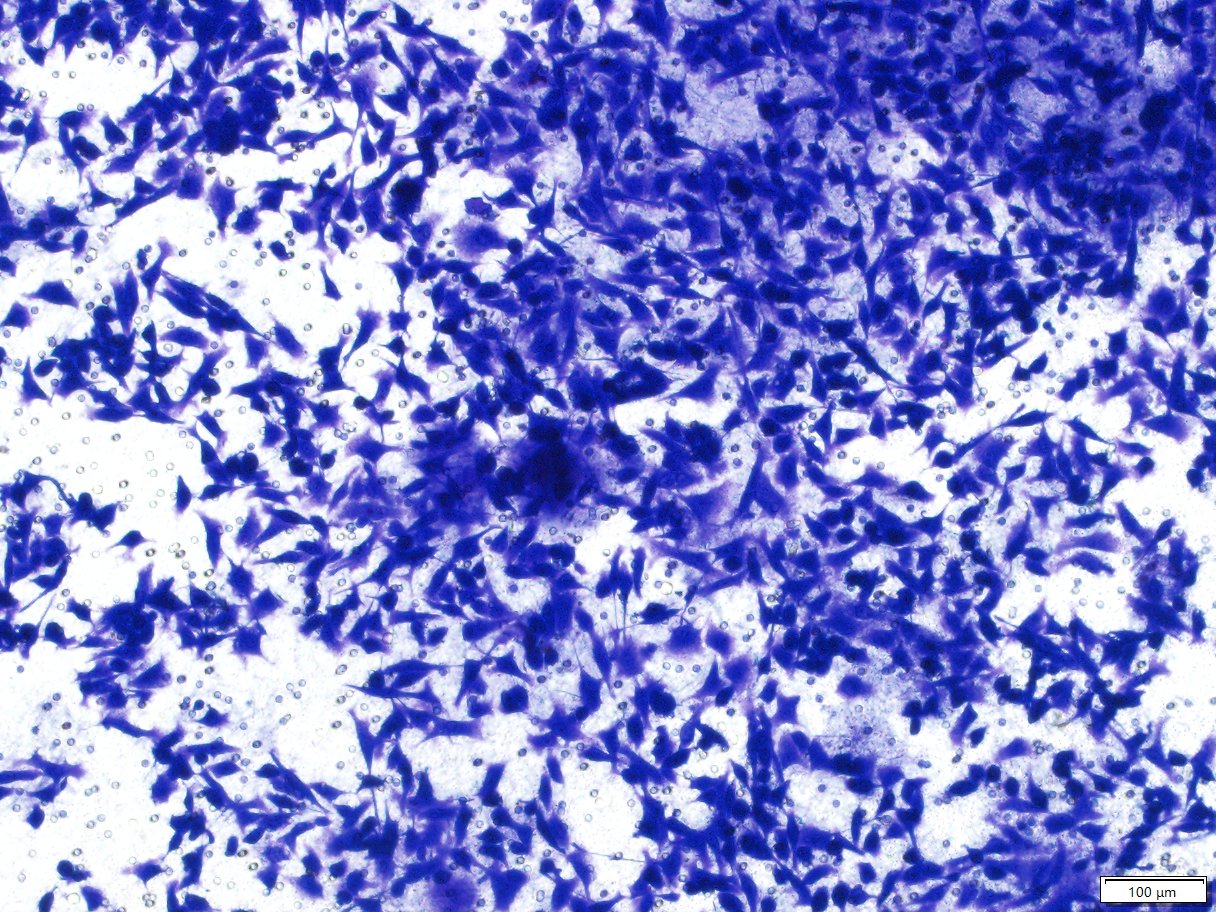

Supplement: Supplemental Information 6 [file peerj-cs-09-1651-s006.zip › Dataset 5/3-3.jpg]

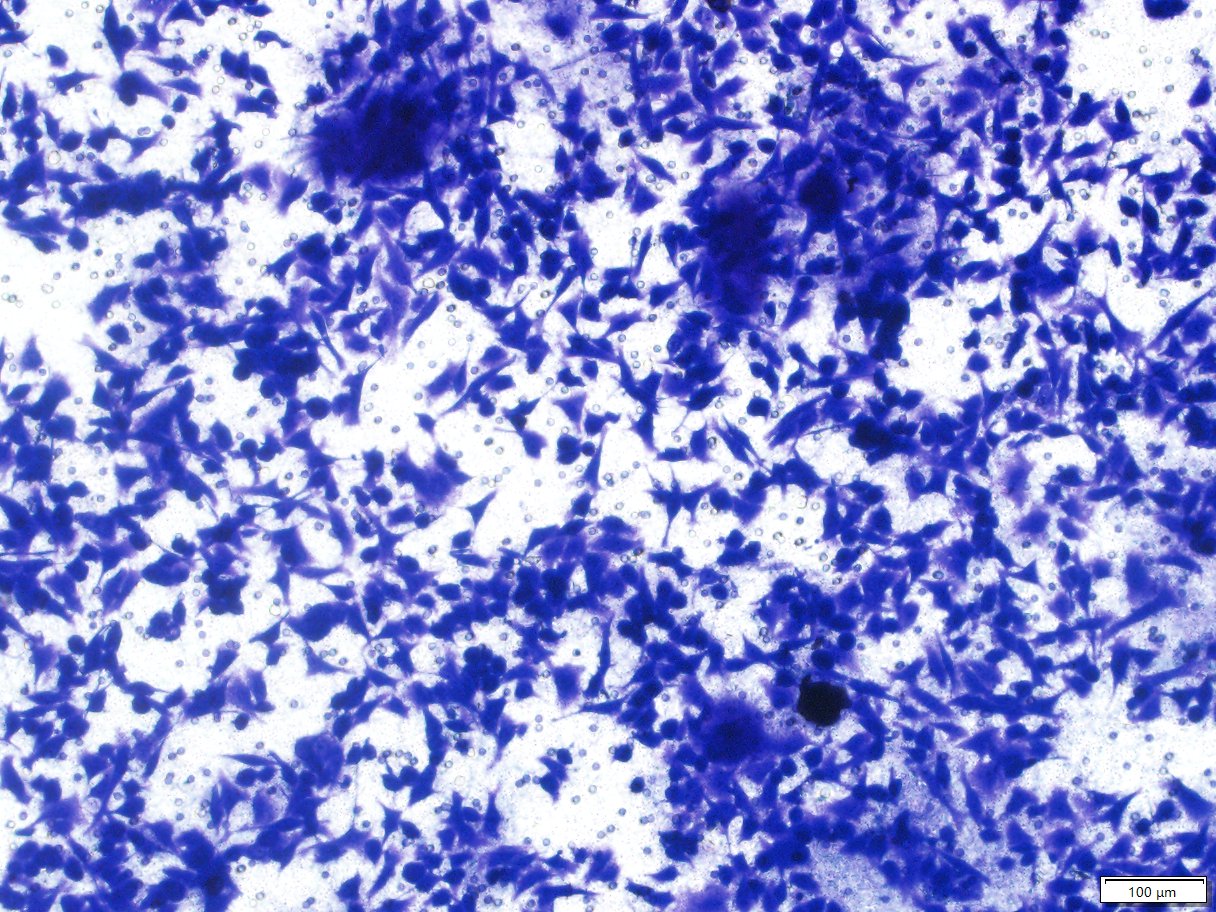

Supplement: Supplemental Information 6 [file peerj-cs-09-1651-s006.zip › Dataset 5/3-4.jpg]

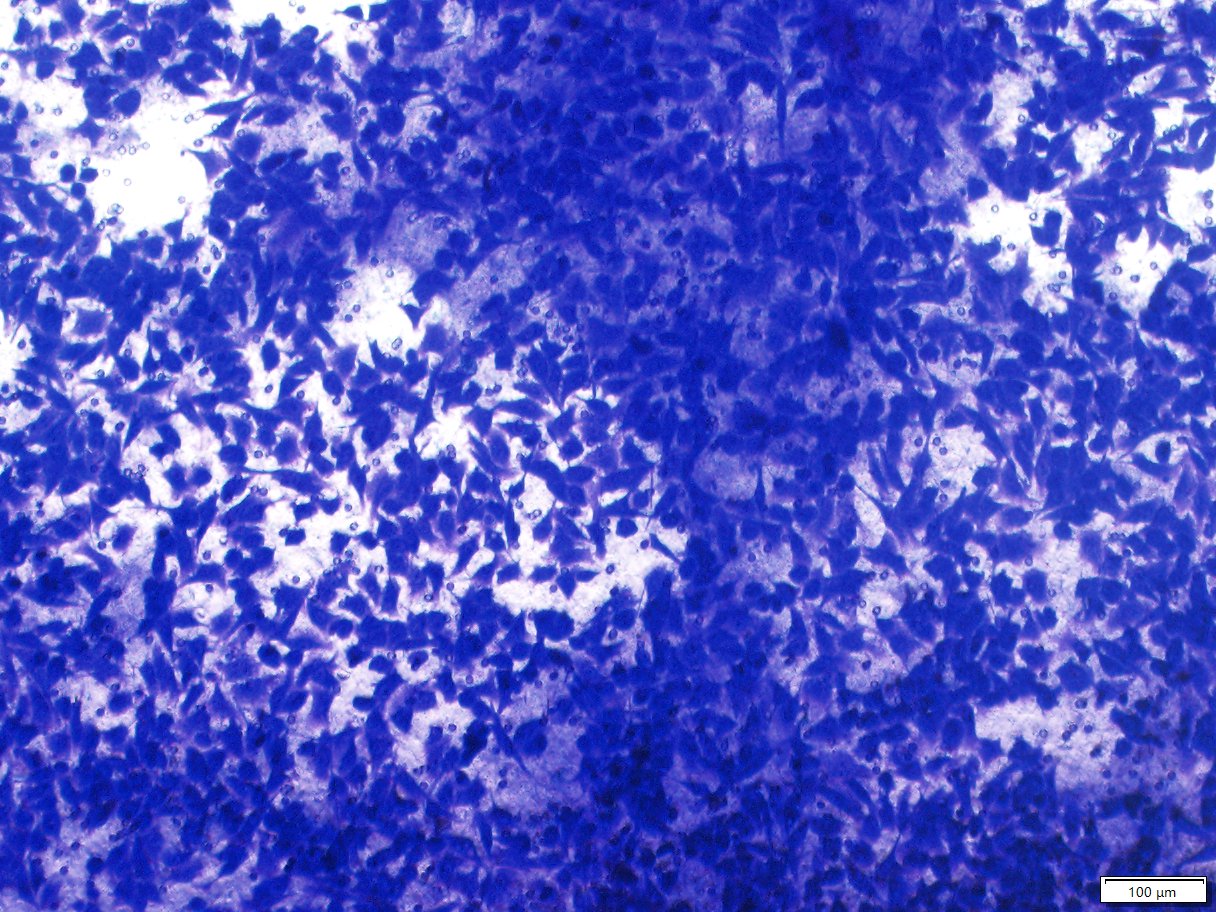

Supplement: Supplemental Information 6 [file peerj-cs-09-1651-s006.zip › Dataset 5/3-5.jpg]

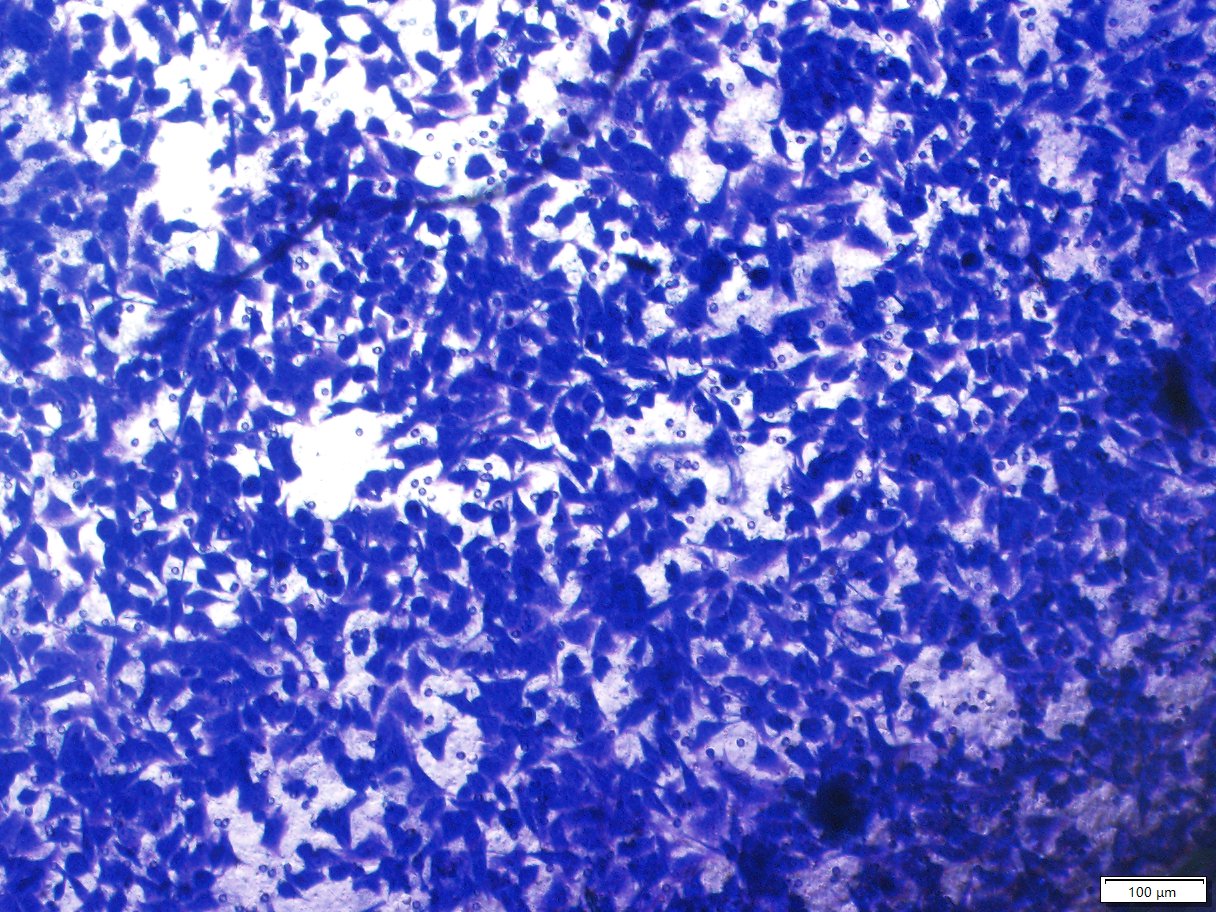

Supplement: Supplemental Information 6 [file peerj-cs-09-1651-s006.zip › Dataset 5/3-6.jpg]

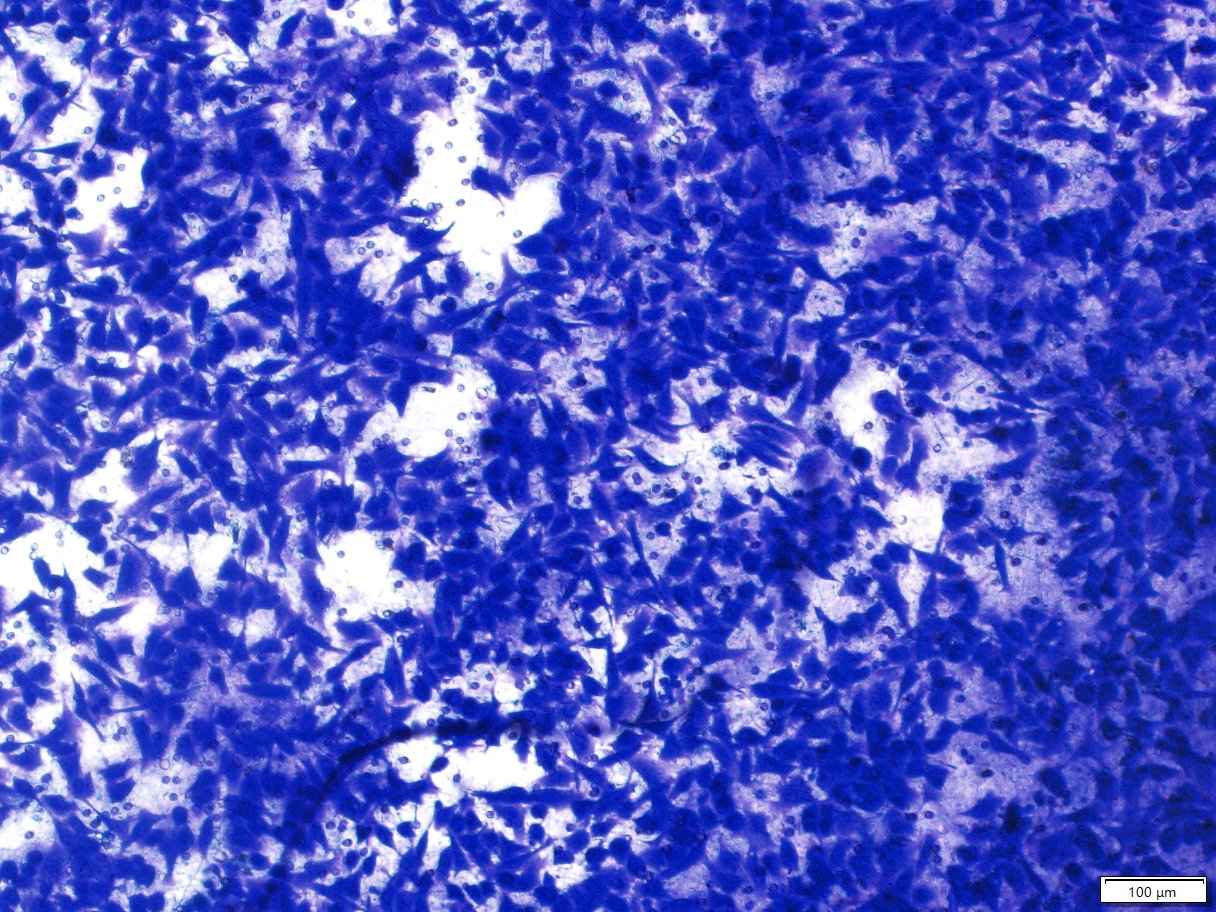

Supplement: Supplemental Information 6 [file peerj-cs-09-1651-s006.zip › Dataset 5/3-7.jpg]

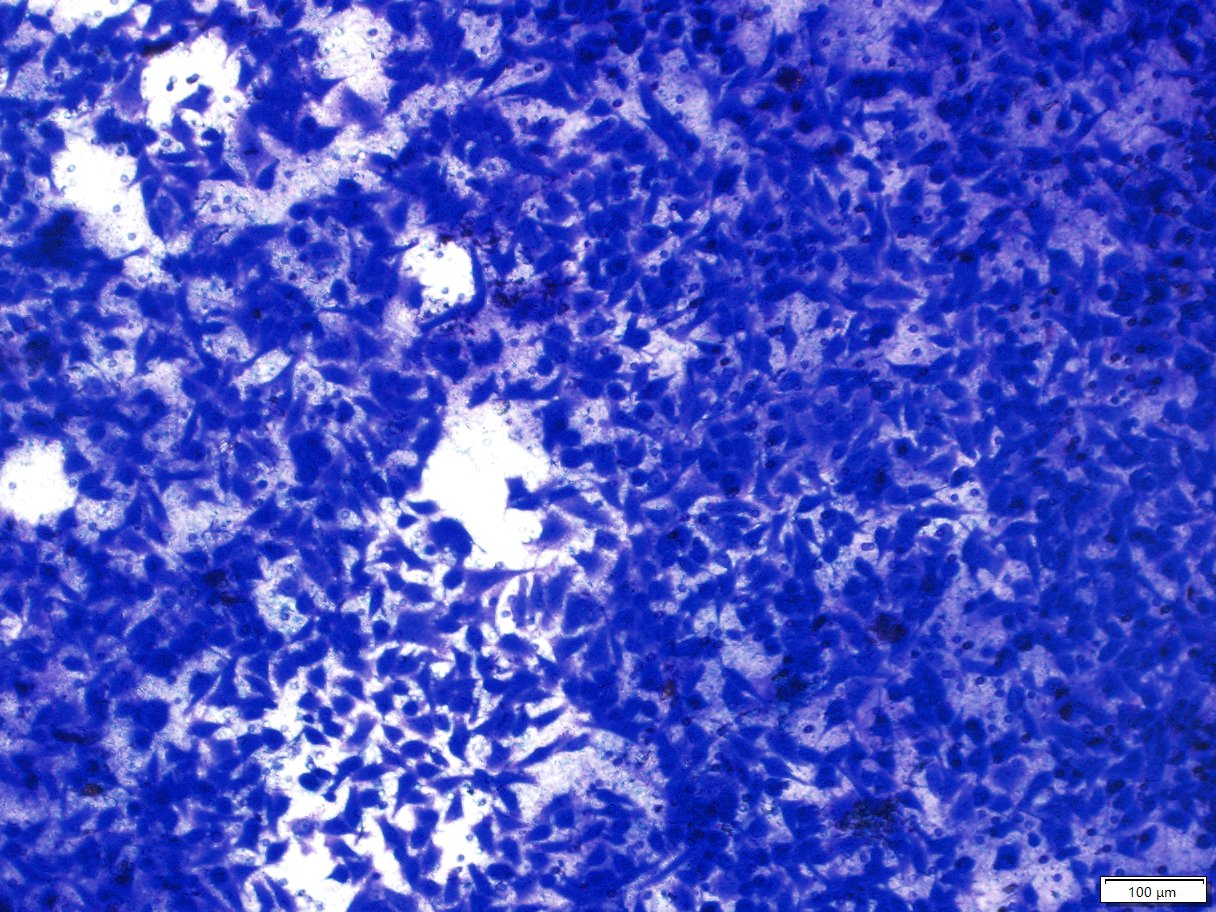

Supplement: Supplemental Information 6 [file peerj-cs-09-1651-s006.zip › Dataset 5/3-8.jpg]

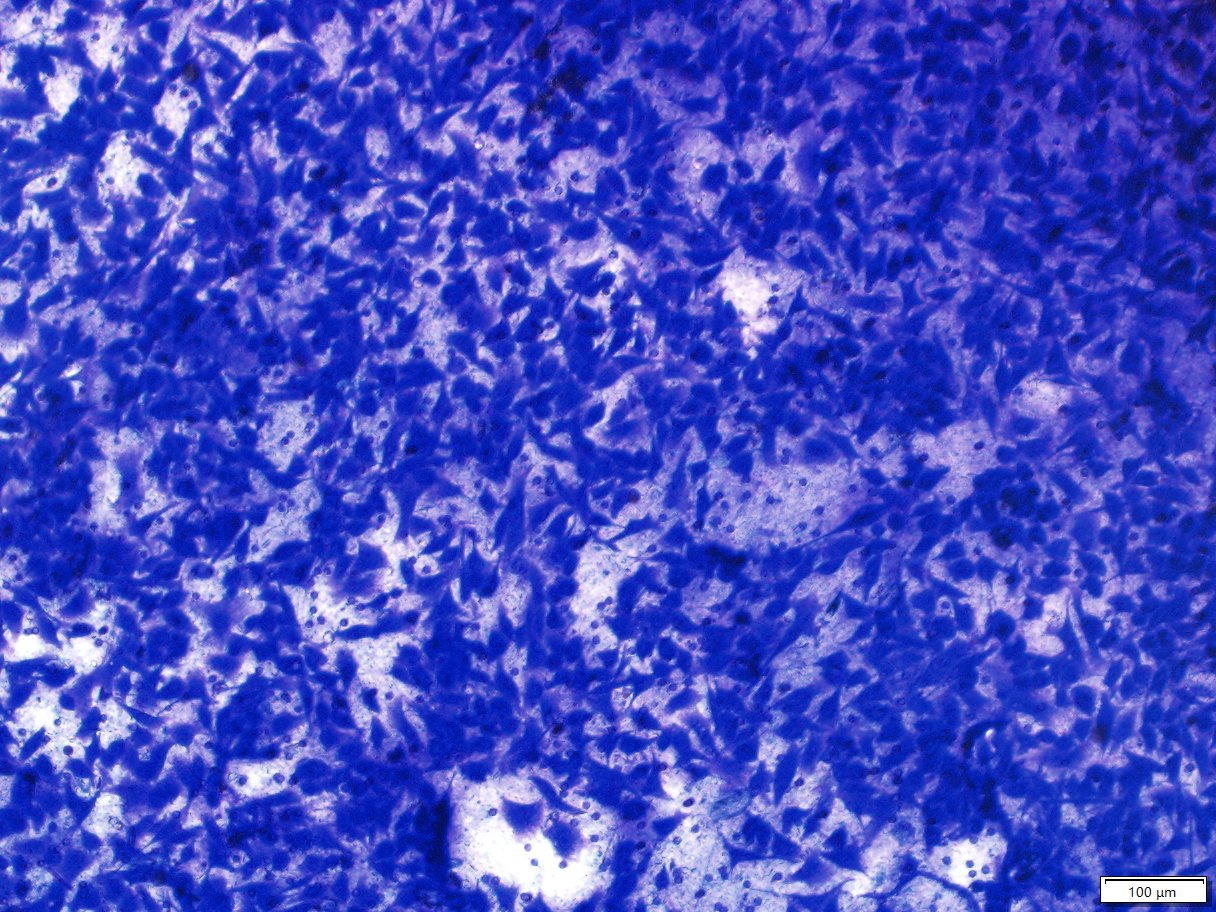

Supplement: Supplemental Information 6 [file peerj-cs-09-1651-s006.zip › Dataset 5/3-9.jpg]

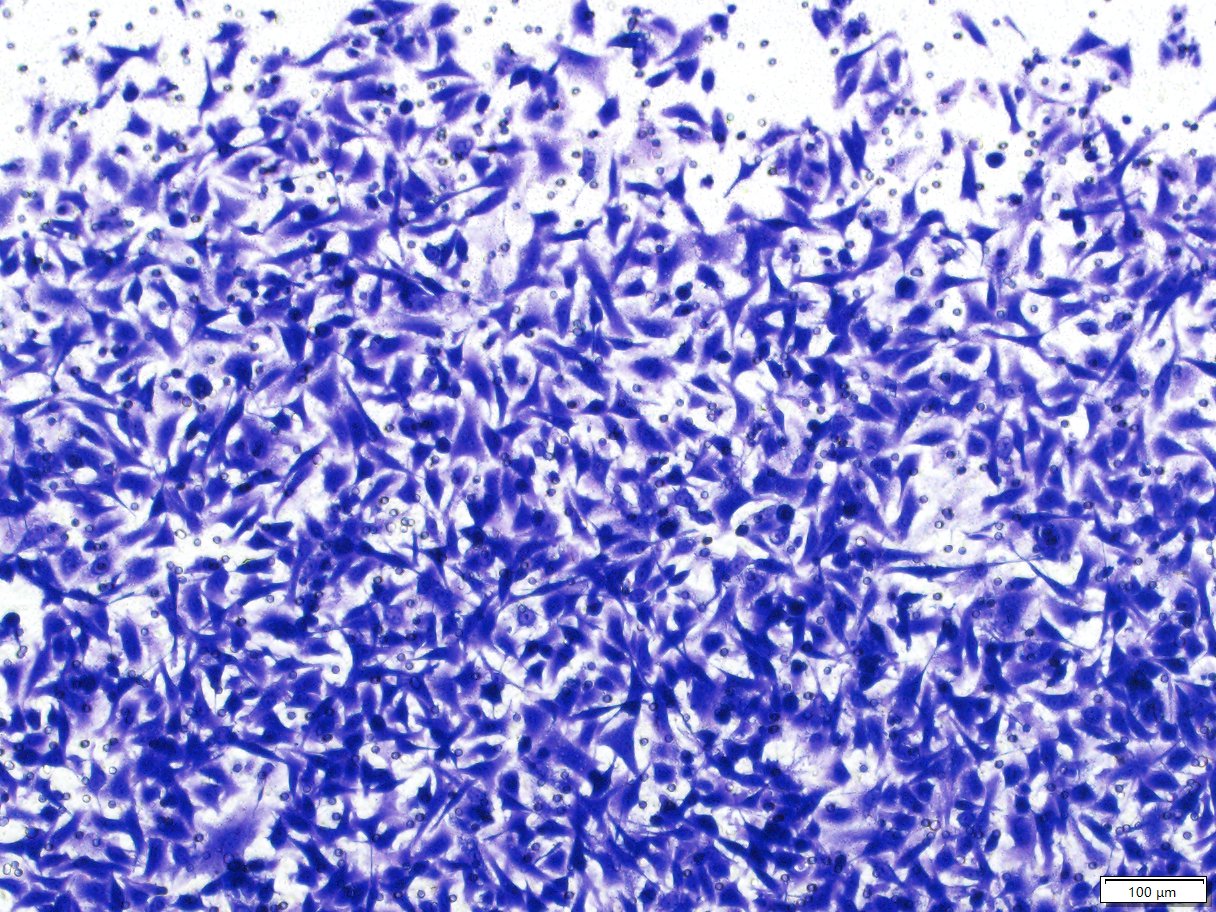

Supplement: Supplemental Information 6 [file peerj-cs-09-1651-s006.zip › Dataset 5/4+1.jpg]

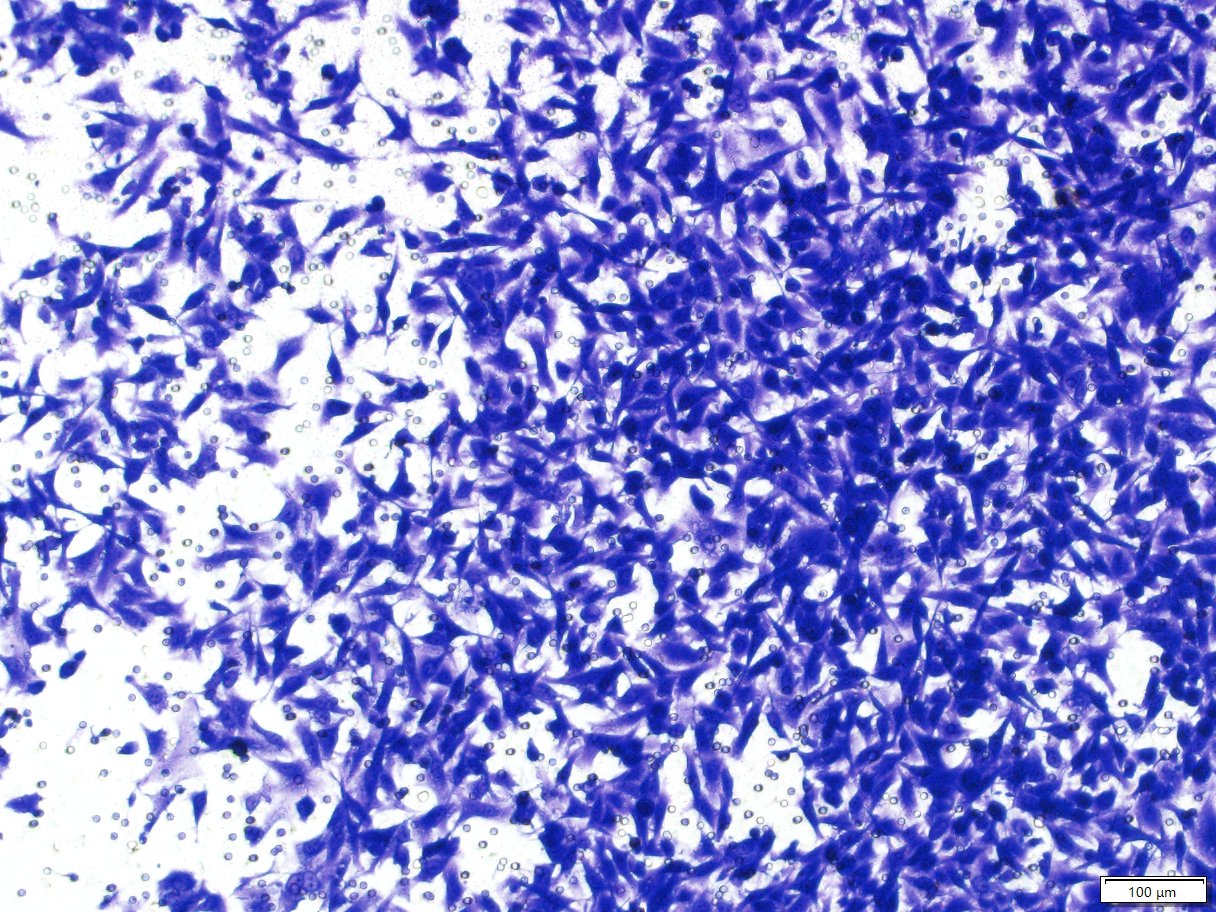

Supplement: Supplemental Information 6 [file peerj-cs-09-1651-s006.zip › Dataset 5/4+10.jpg]

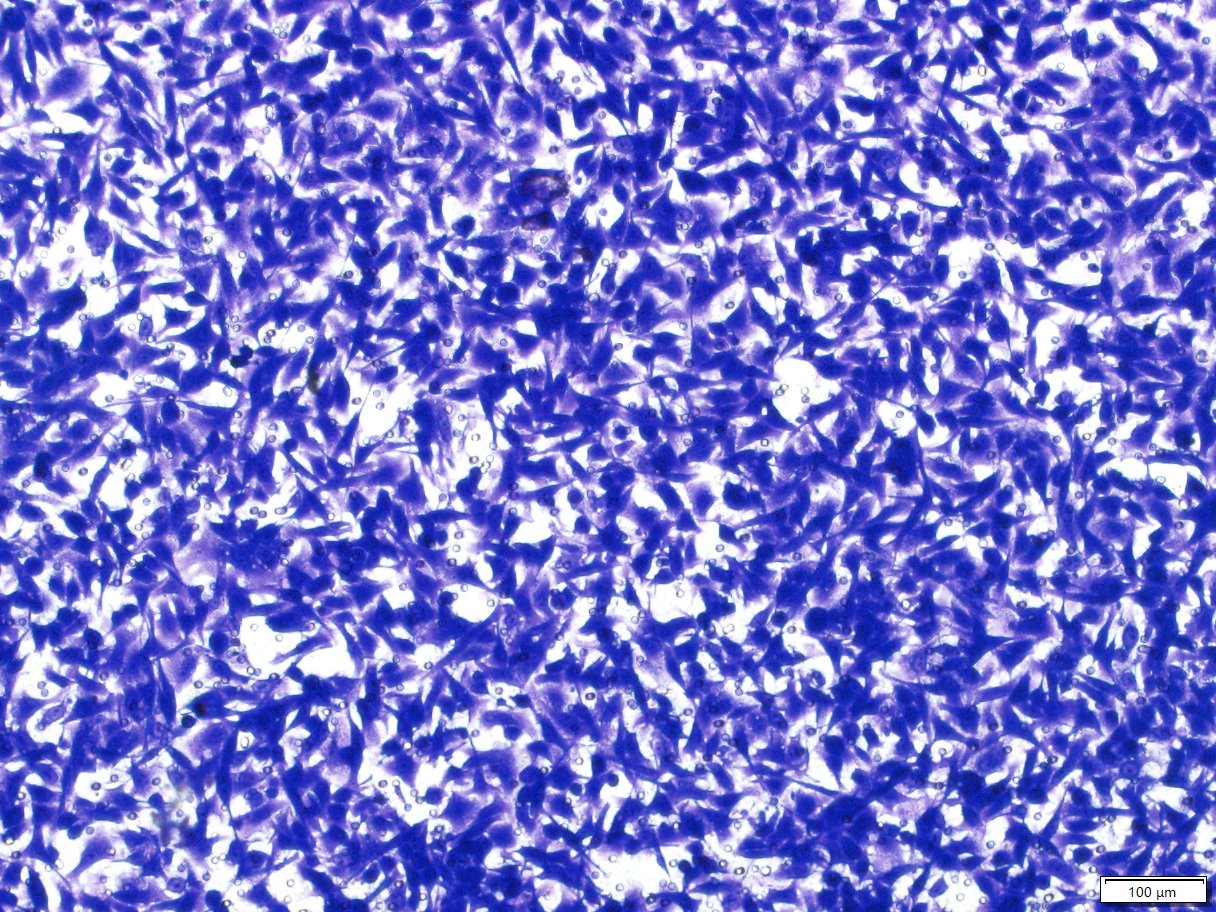

Supplement: Supplemental Information 6 [file peerj-cs-09-1651-s006.zip › Dataset 5/4+2.jpg]

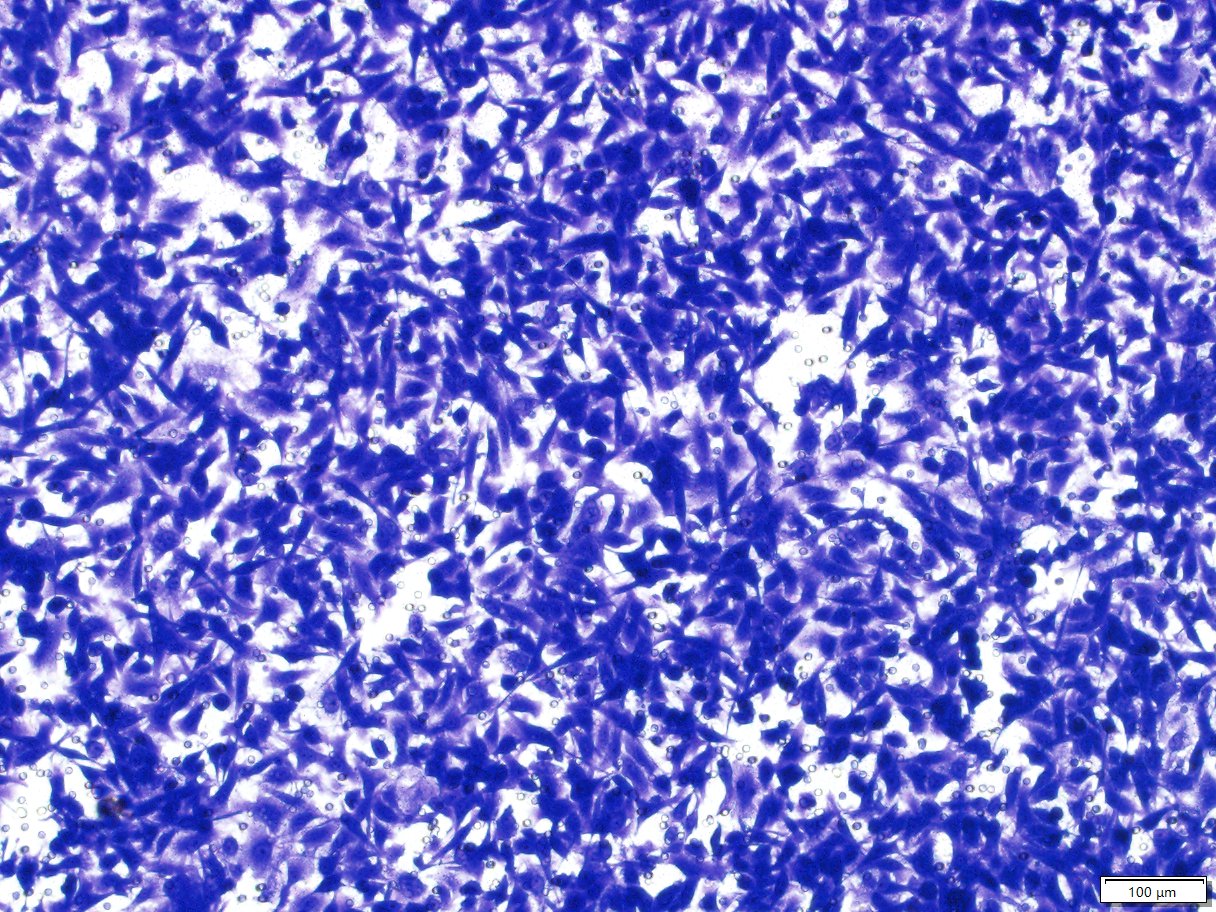

Supplement: Supplemental Information 6 [file peerj-cs-09-1651-s006.zip › Dataset 5/4+3.jpg]

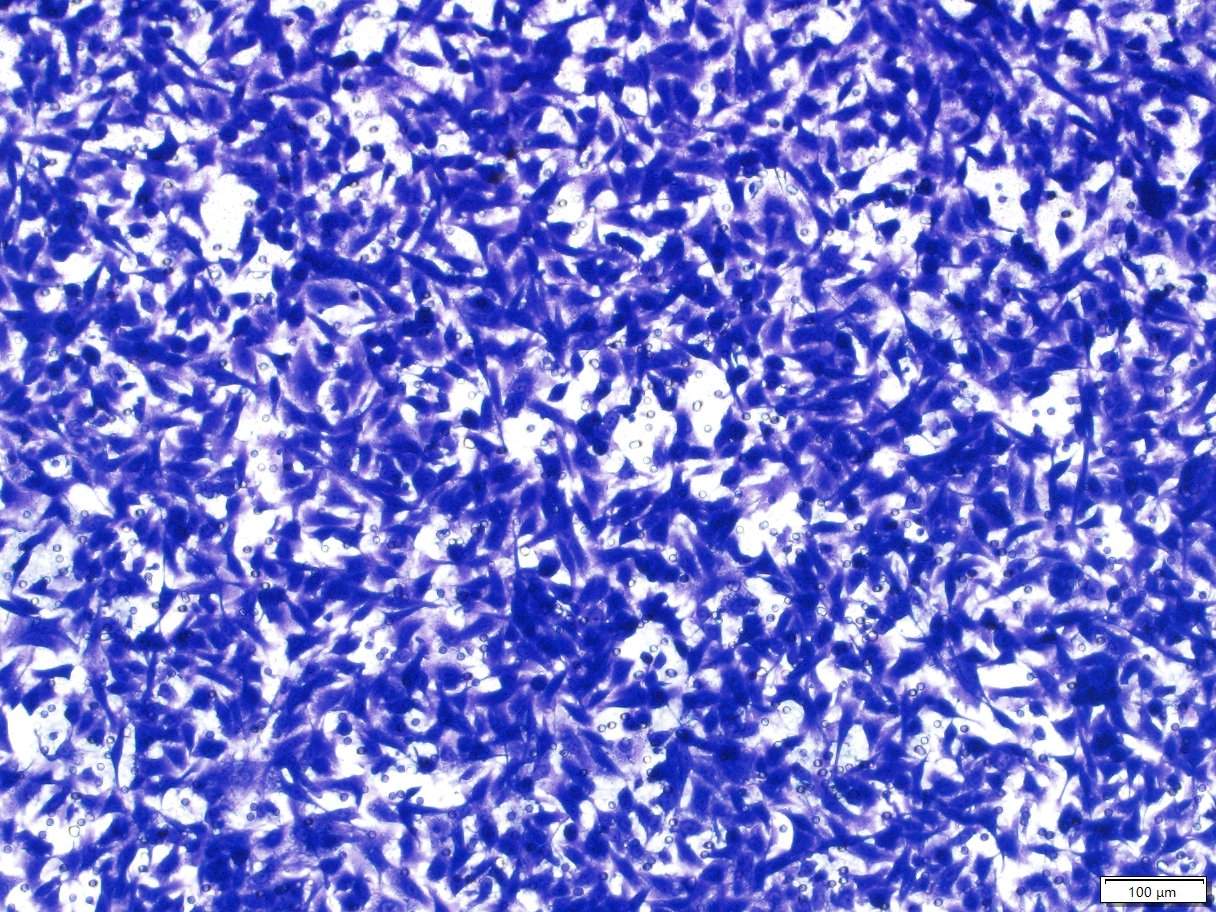

Supplement: Supplemental Information 6 [file peerj-cs-09-1651-s006.zip › Dataset 5/4+4.jpg]

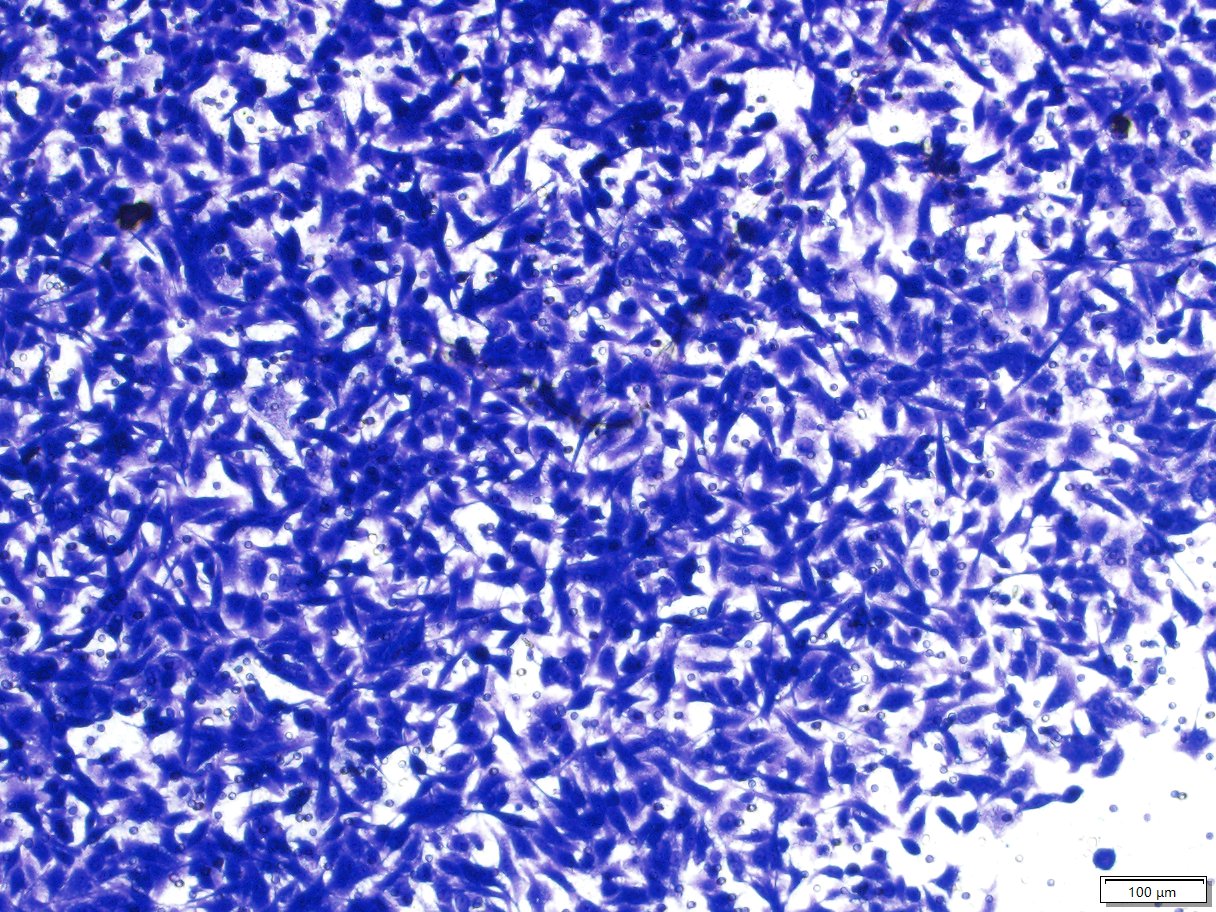

Supplement: Supplemental Information 6 [file peerj-cs-09-1651-s006.zip › Dataset 5/4+5.jpg]

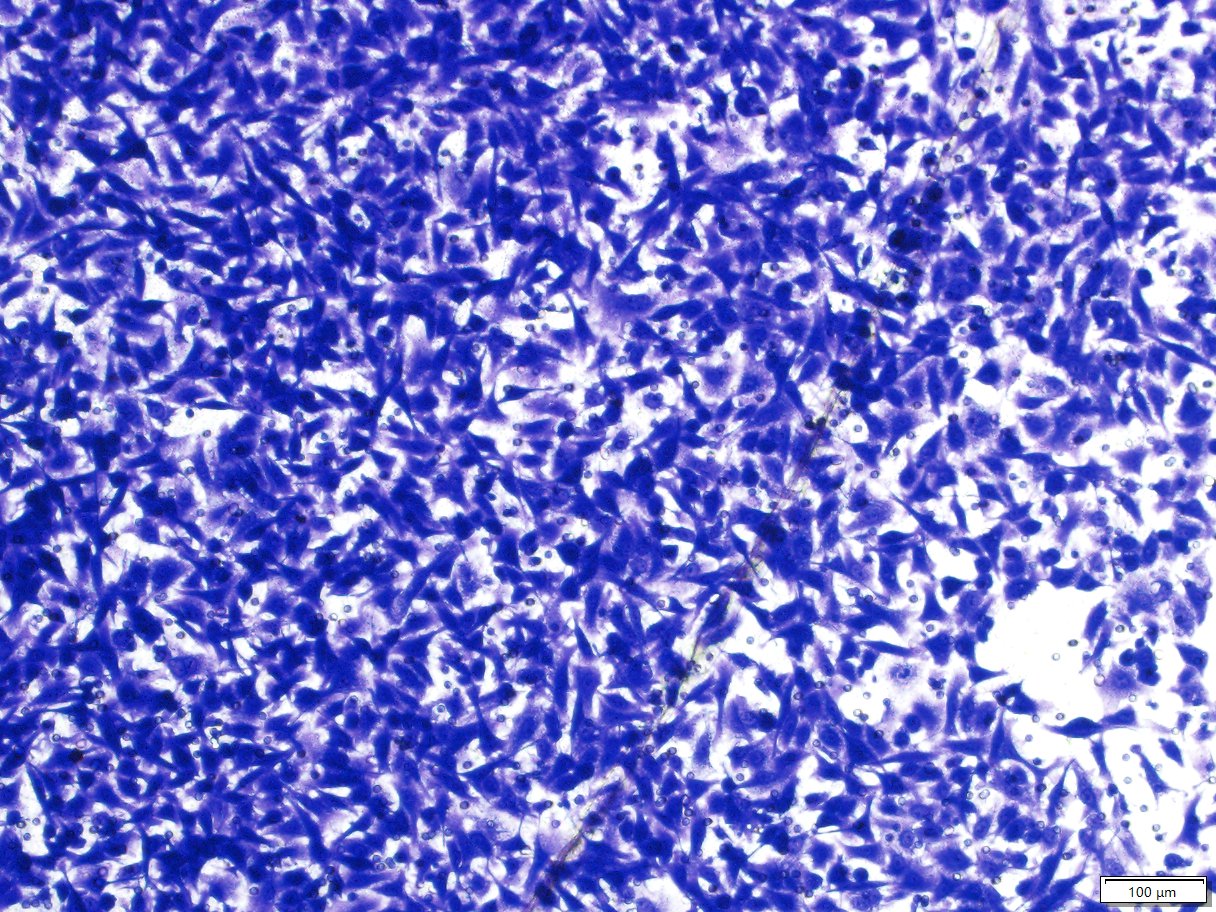

Supplement: Supplemental Information 6 [file peerj-cs-09-1651-s006.zip › Dataset 5/4+6.jpg]

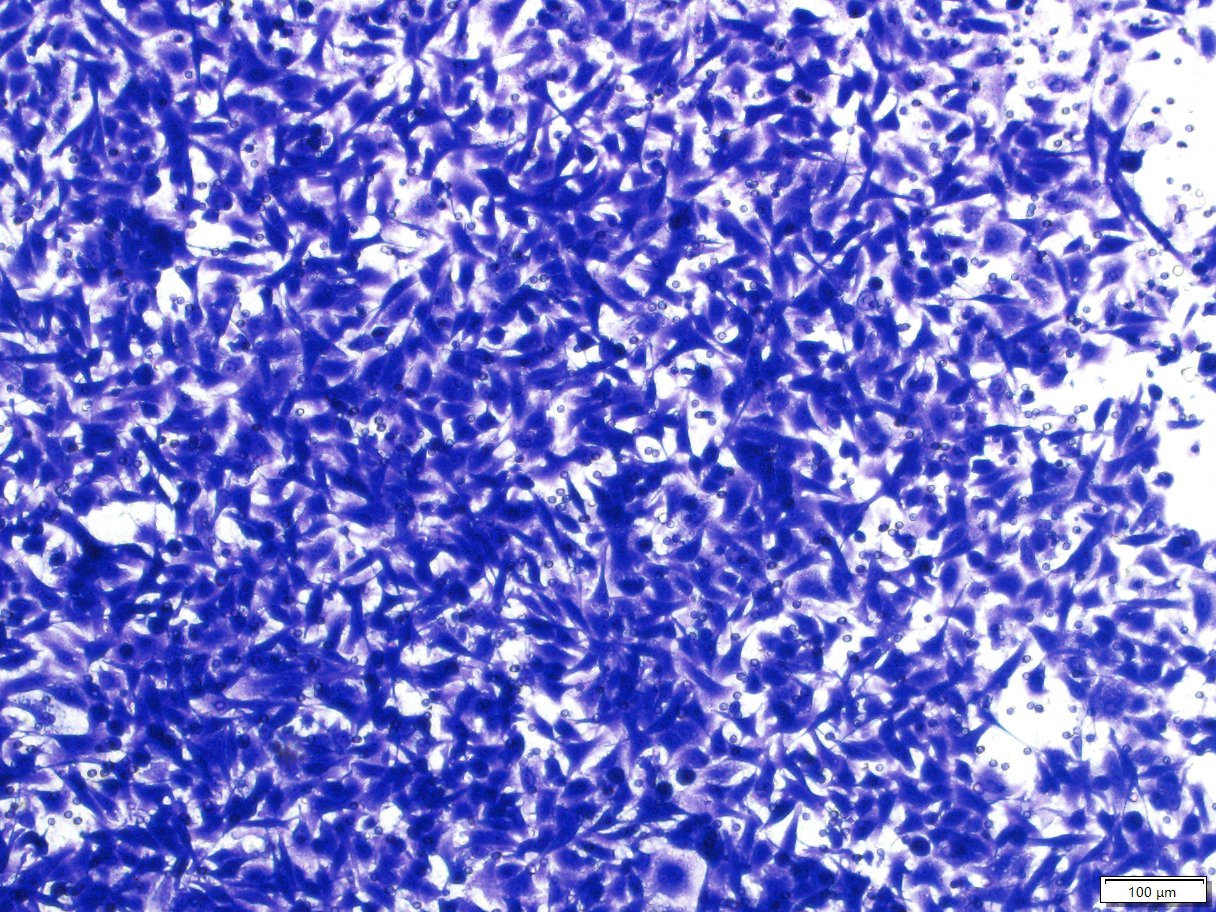

Supplement: Supplemental Information 6 [file peerj-cs-09-1651-s006.zip › Dataset 5/4+7.jpg]

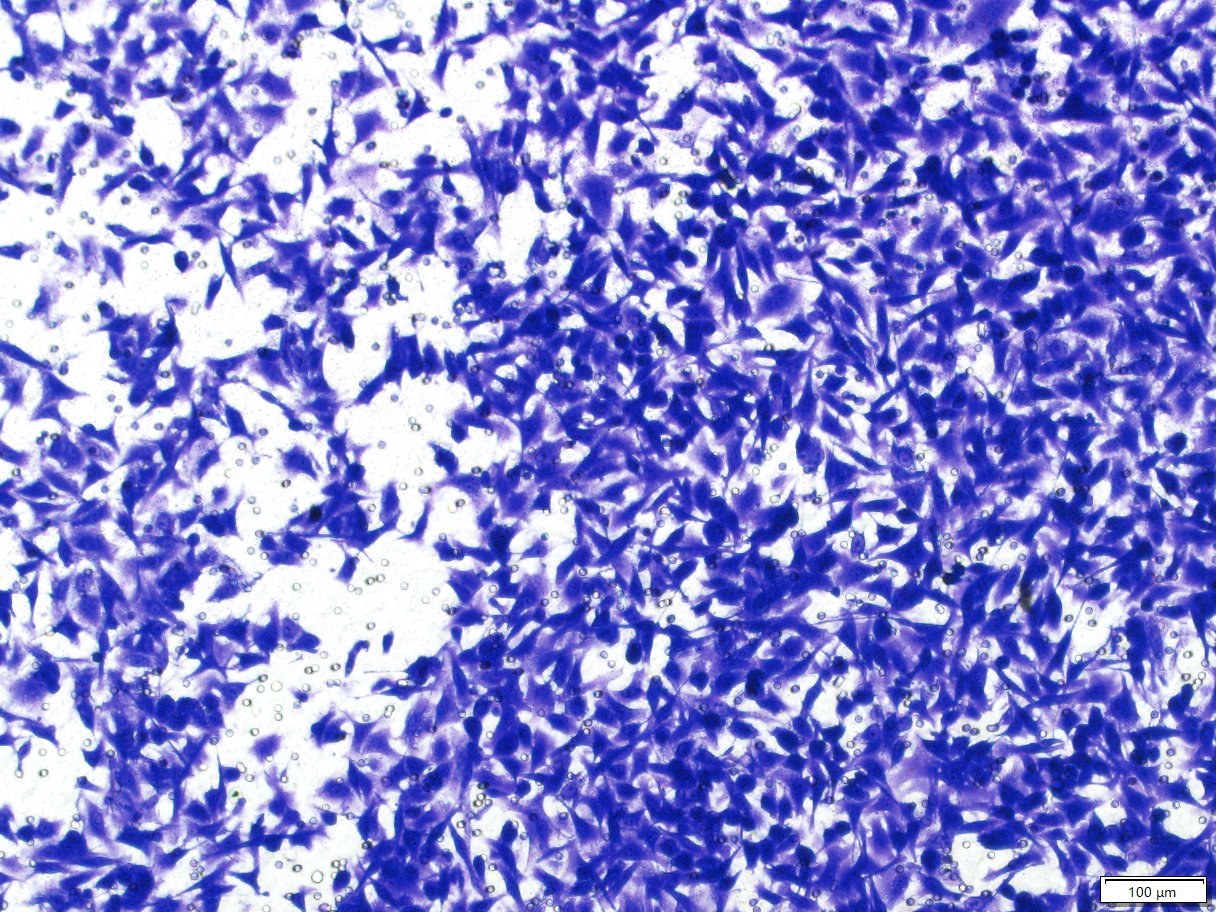

Supplement: Supplemental Information 6 [file peerj-cs-09-1651-s006.zip › Dataset 5/4+8.jpg]

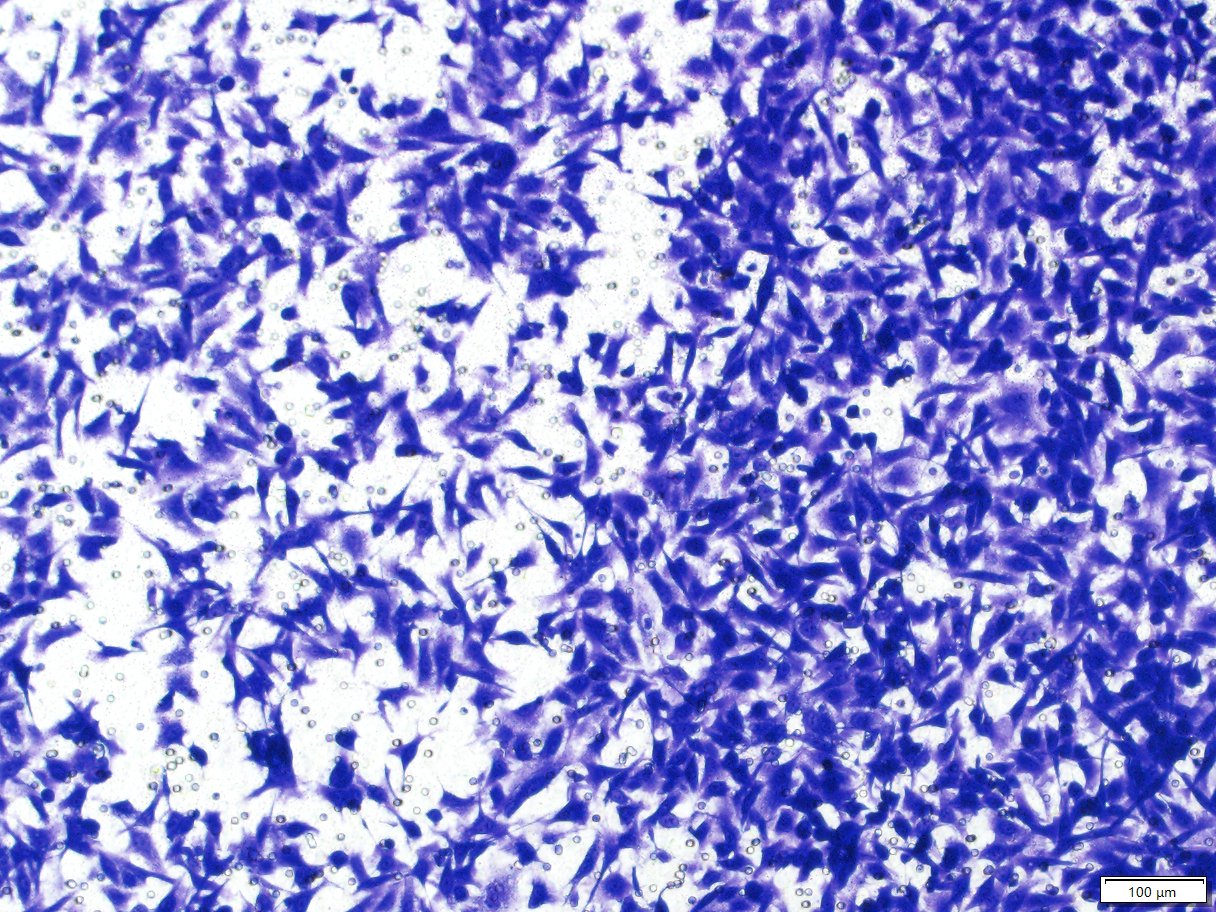

Supplement: Supplemental Information 6 [file peerj-cs-09-1651-s006.zip › Dataset 5/4+9.jpg]

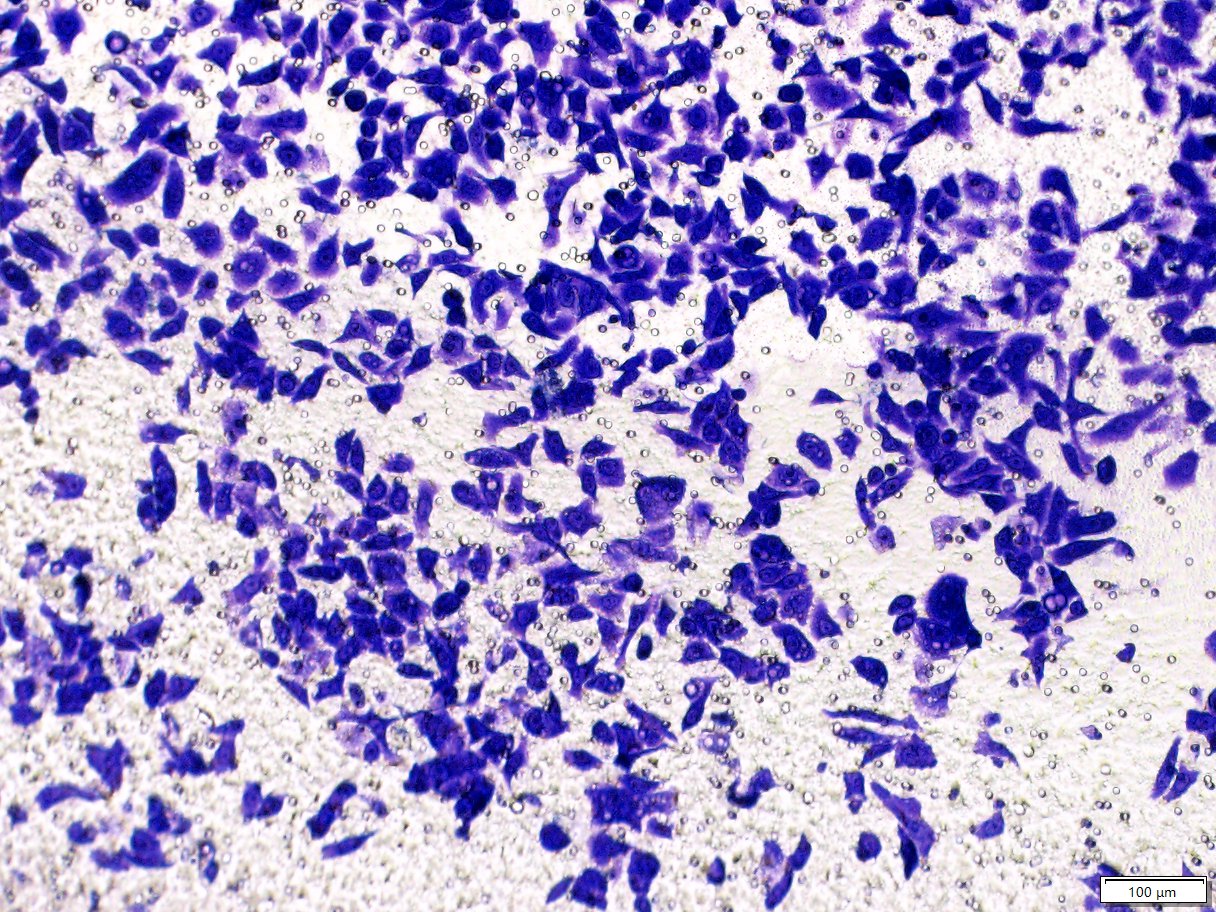

Supplement: Supplemental Information 7 [file peerj-cs-09-1651-s007.zip › Dataset 6/0+1.jpg]

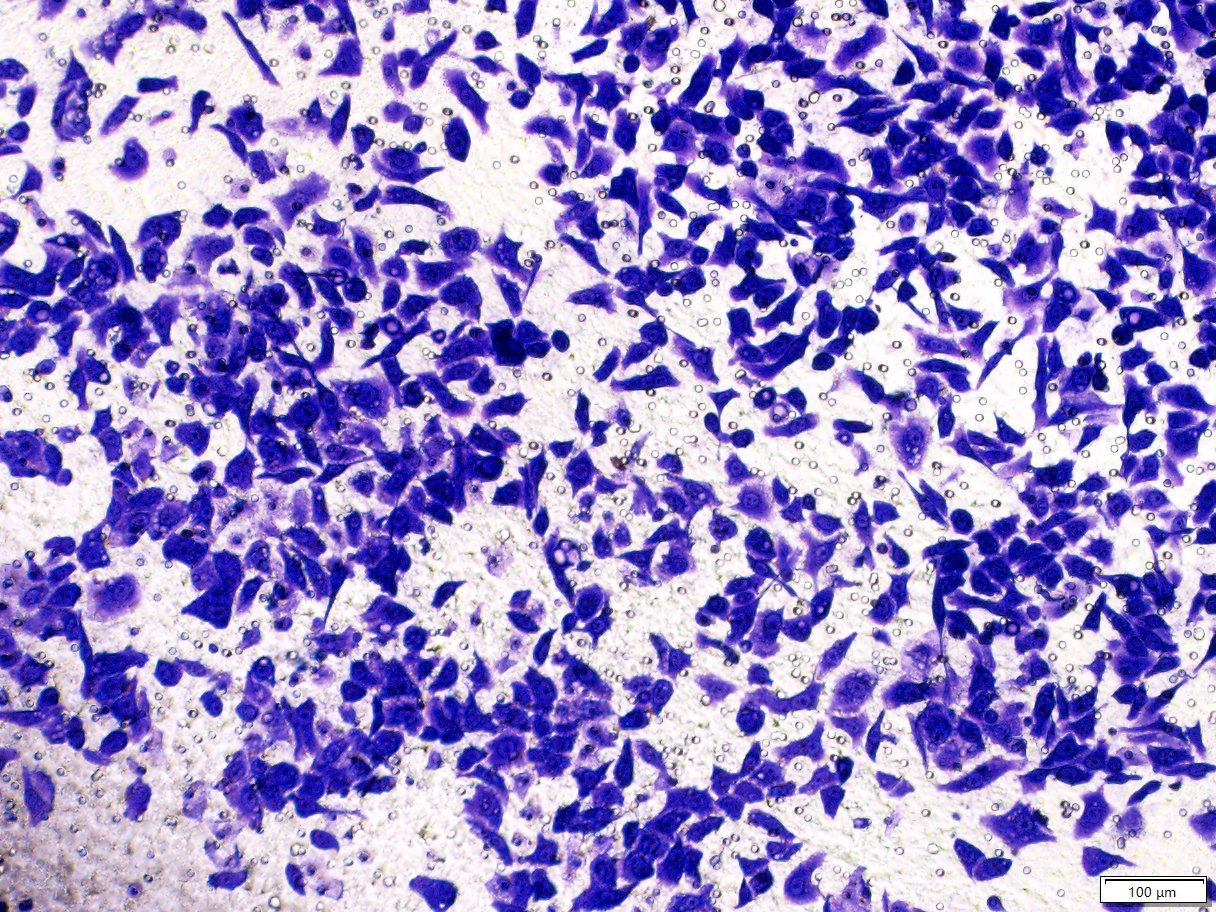

Supplement: Supplemental Information 7 [file peerj-cs-09-1651-s007.zip › Dataset 6/0+10.jpg]

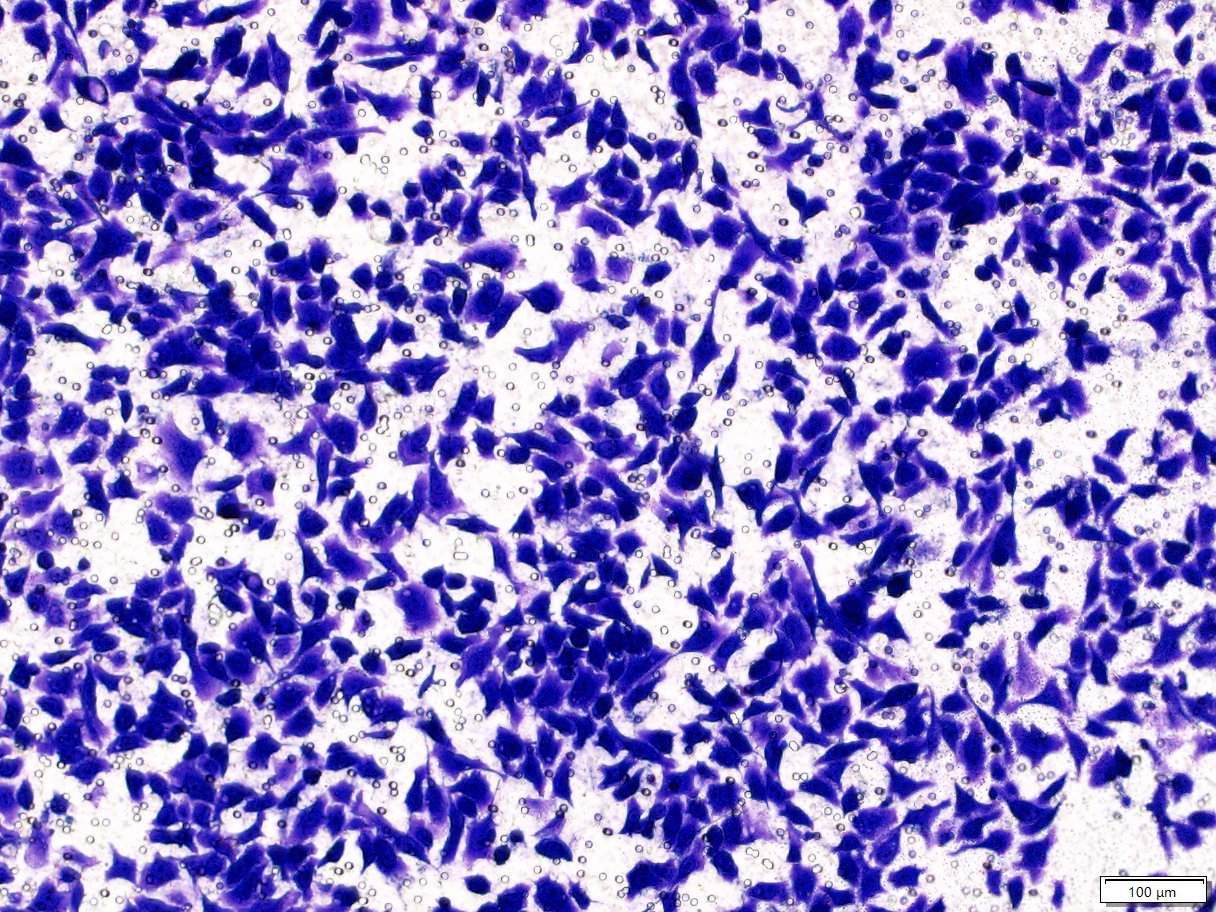

Supplement: Supplemental Information 7 [file peerj-cs-09-1651-s007.zip › Dataset 6/0+11.jpg]

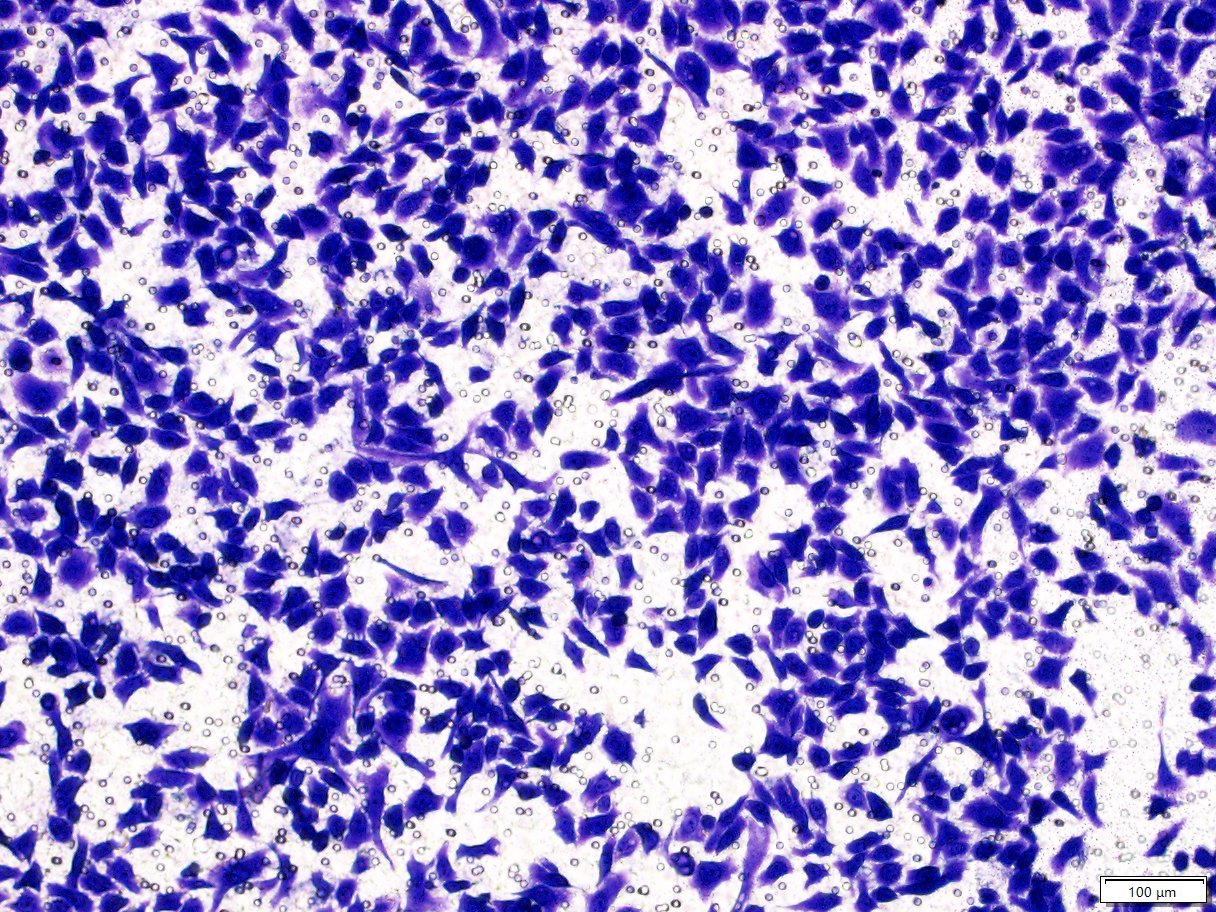

Supplement: Supplemental Information 7 [file peerj-cs-09-1651-s007.zip › Dataset 6/0+12.jpg]

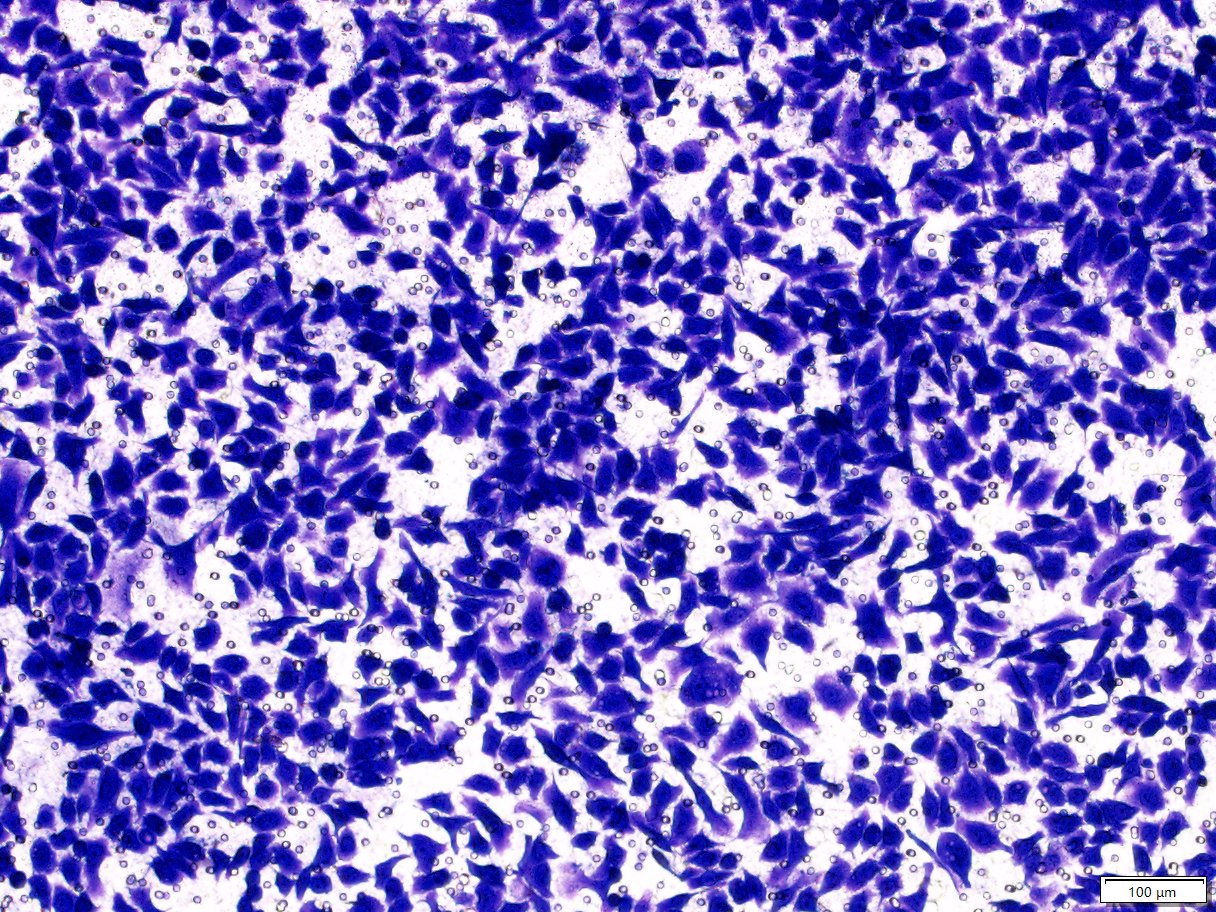

Supplement: Supplemental Information 7 [file peerj-cs-09-1651-s007.zip › Dataset 6/0+13.jpg]

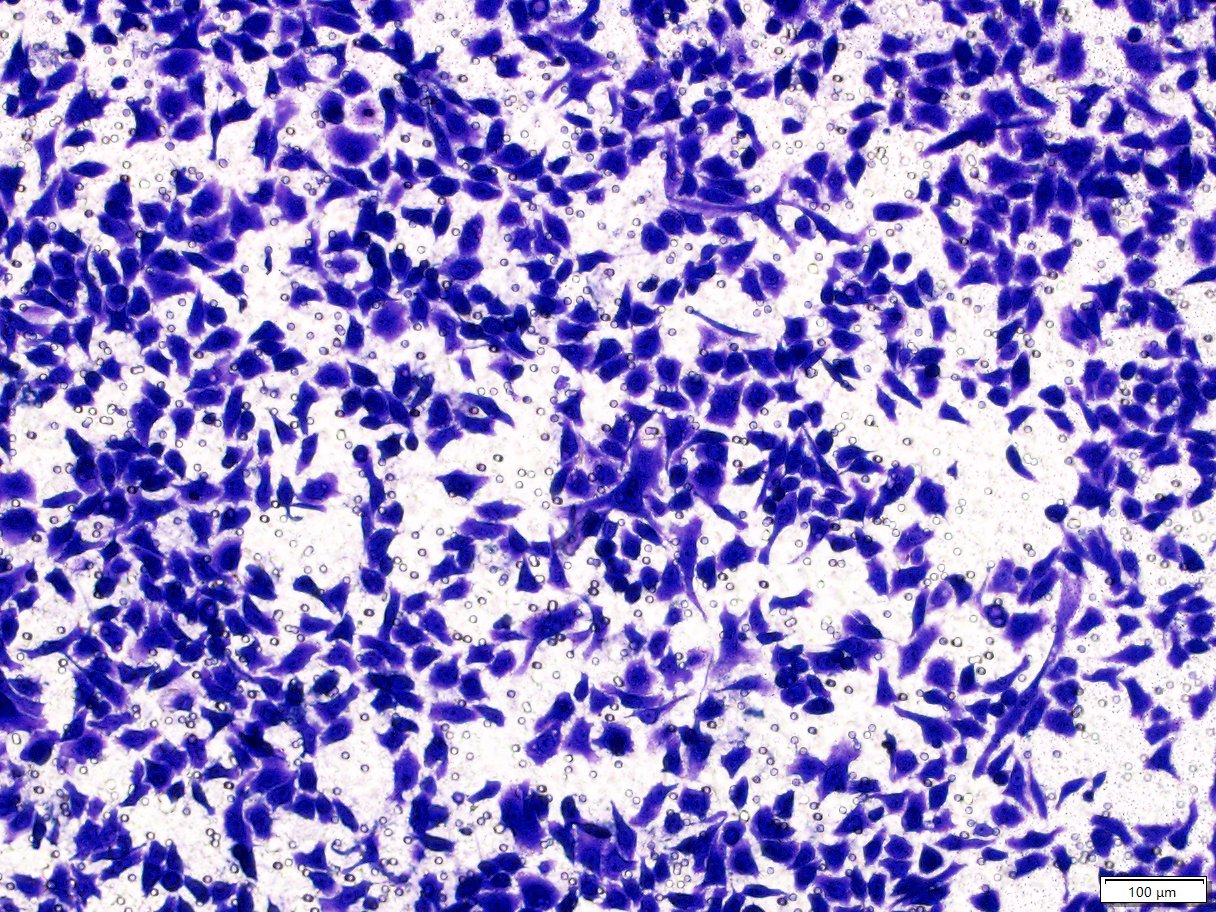

Supplement: Supplemental Information 7 [file peerj-cs-09-1651-s007.zip › Dataset 6/0+14.jpg]

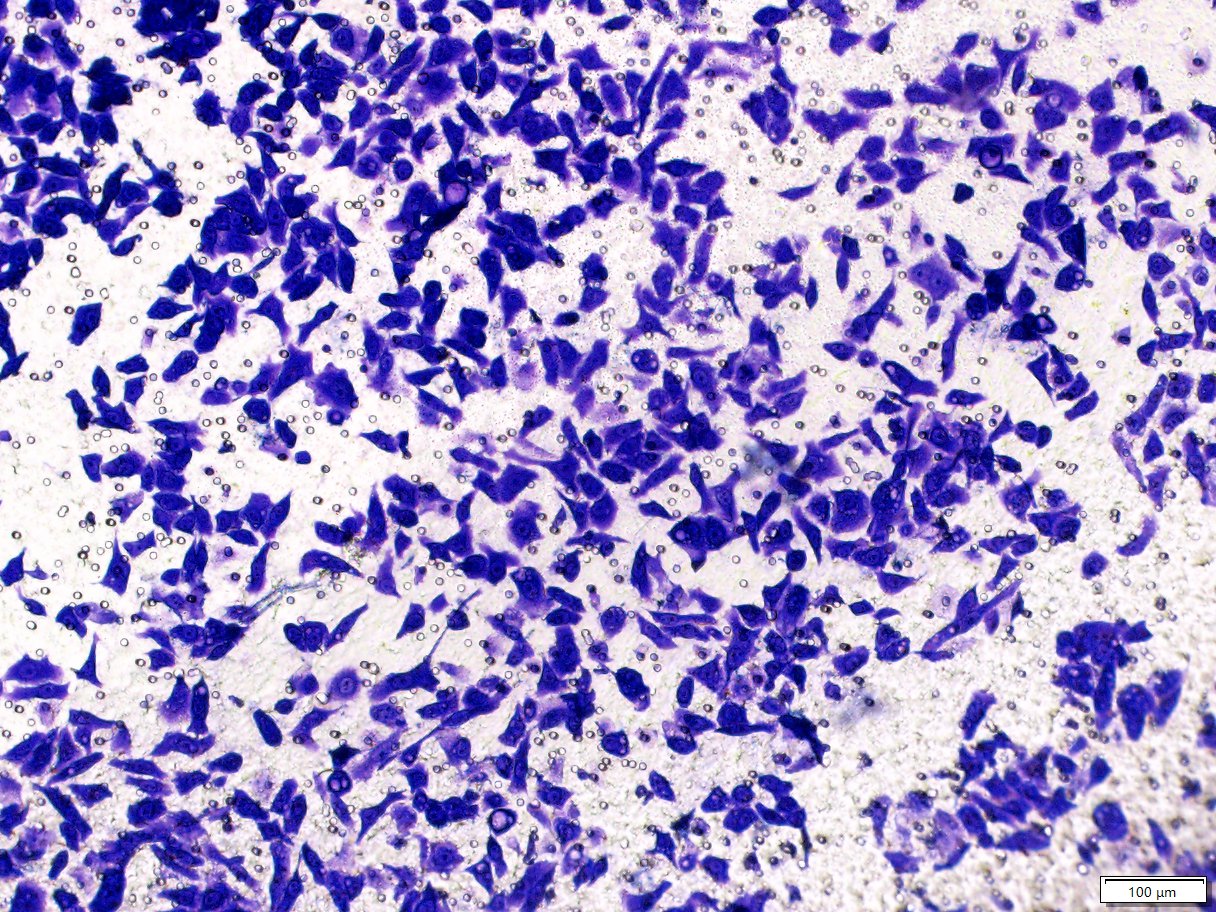

Supplement: Supplemental Information 7 [file peerj-cs-09-1651-s007.zip › Dataset 6/0+2.jpg]

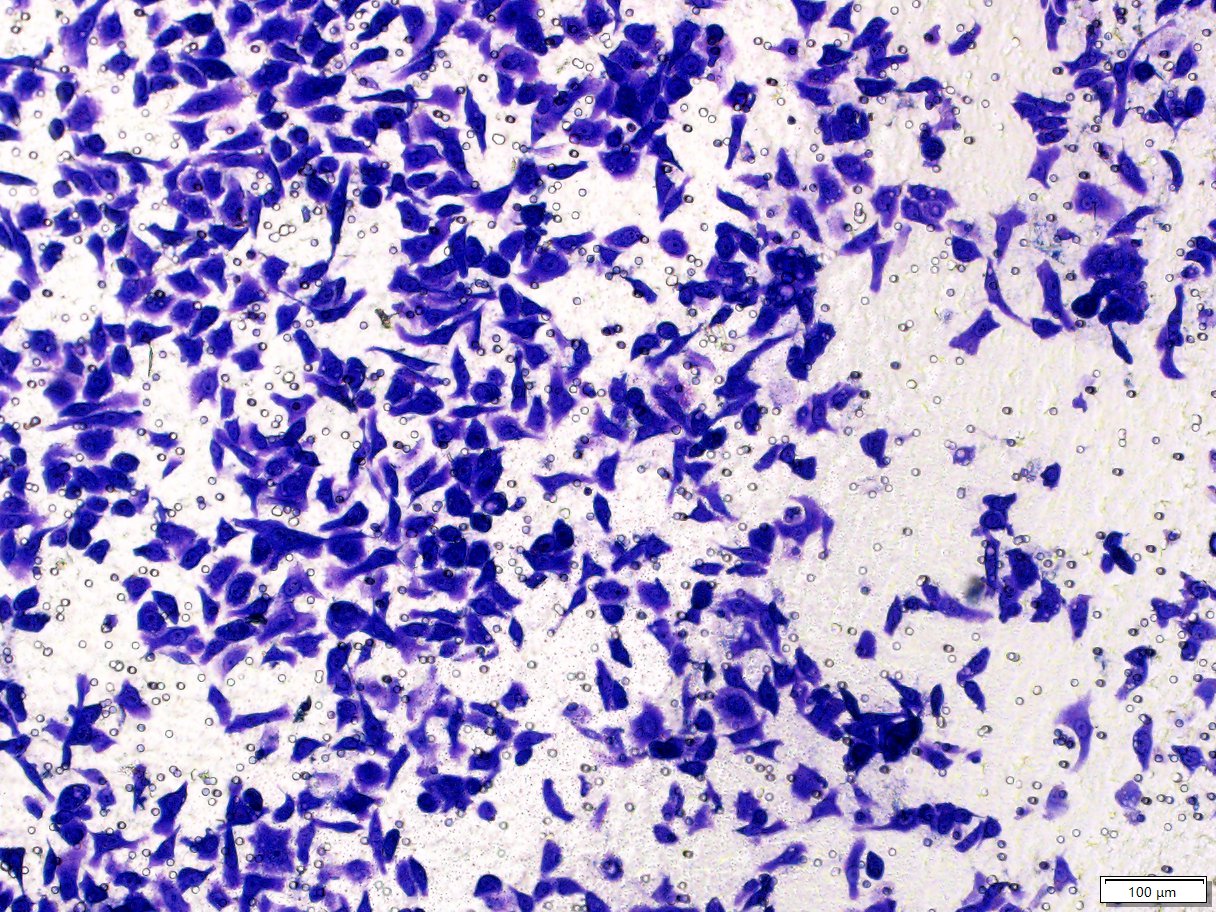

Supplement: Supplemental Information 7 [file peerj-cs-09-1651-s007.zip › Dataset 6/0+3.jpg]

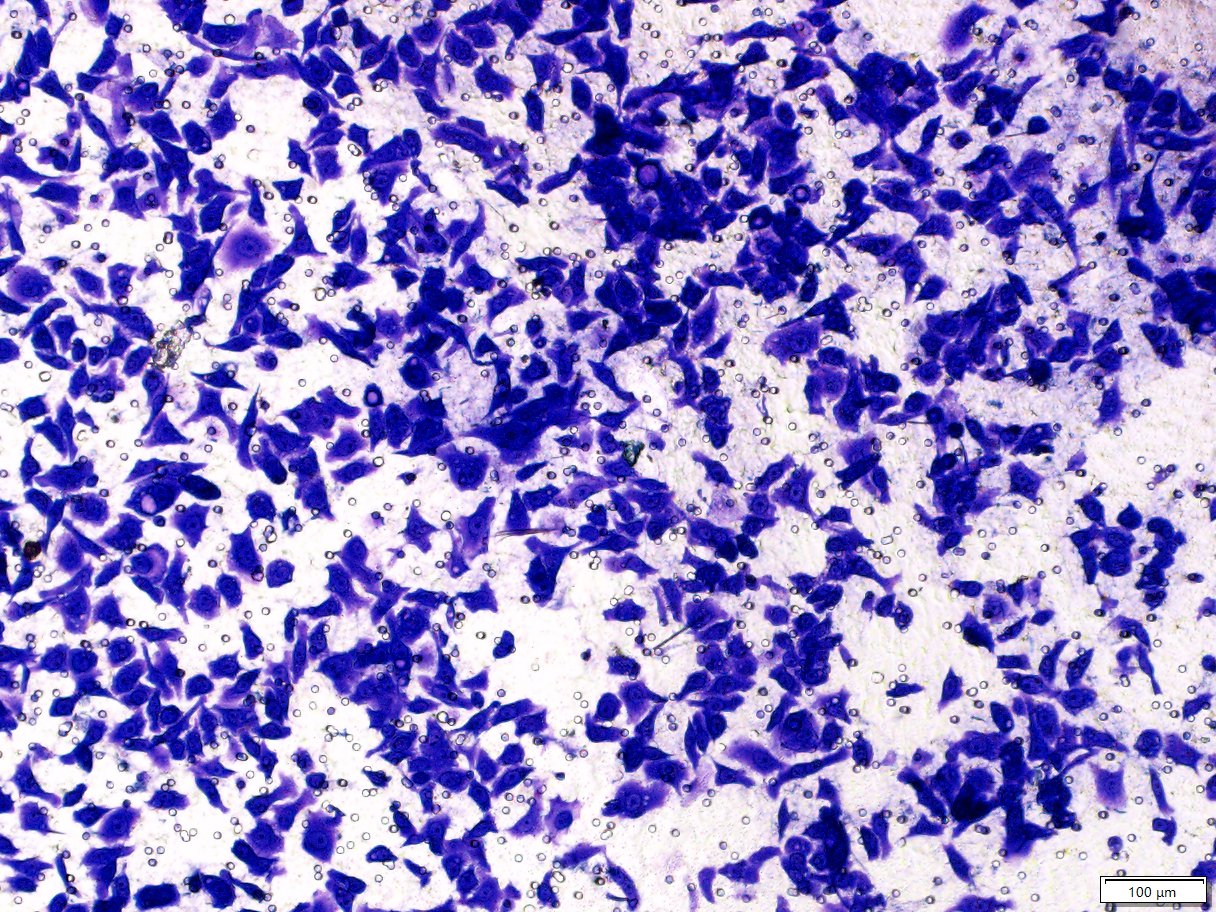

Supplement: Supplemental Information 7 [file peerj-cs-09-1651-s007.zip › Dataset 6/0+4.jpg]

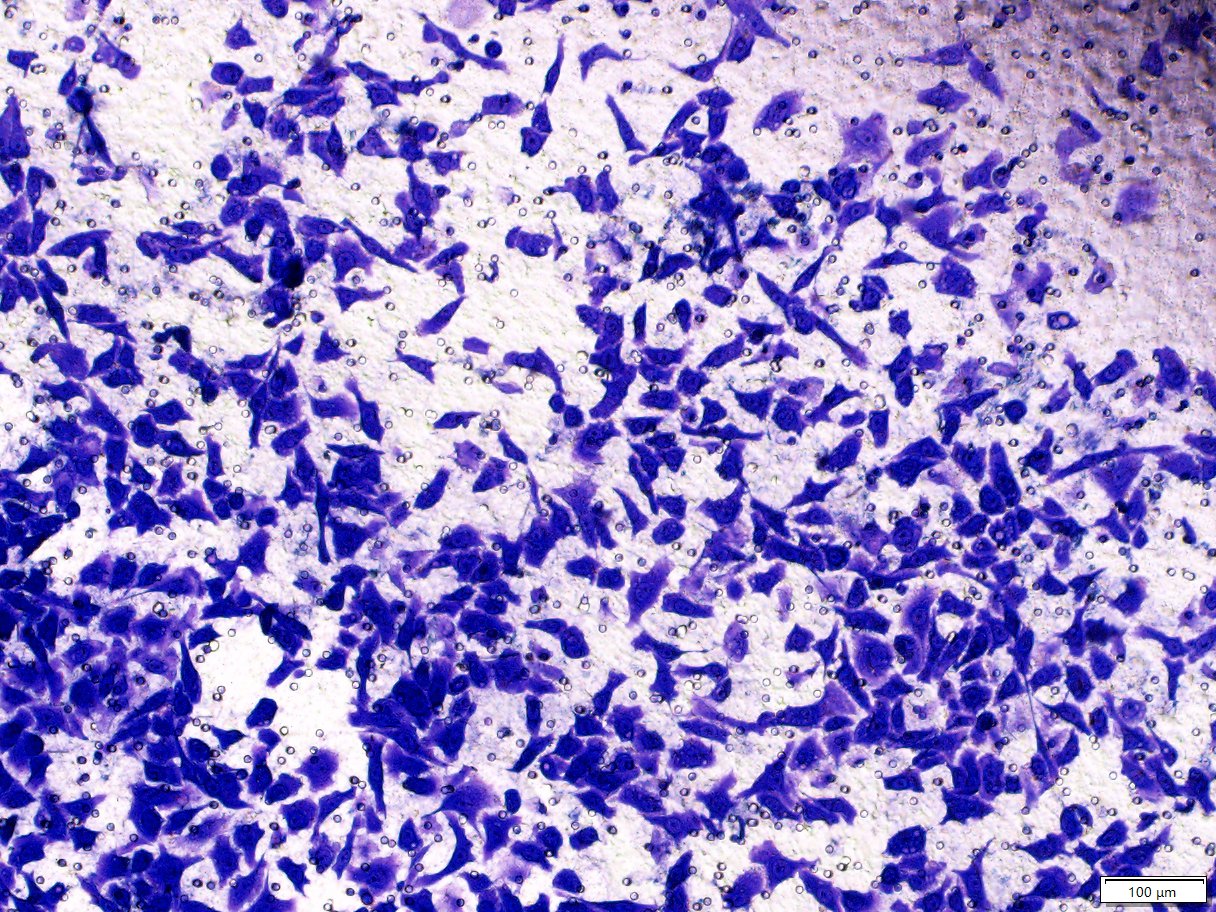

Supplement: Supplemental Information 7 [file peerj-cs-09-1651-s007.zip › Dataset 6/0+5.jpg]

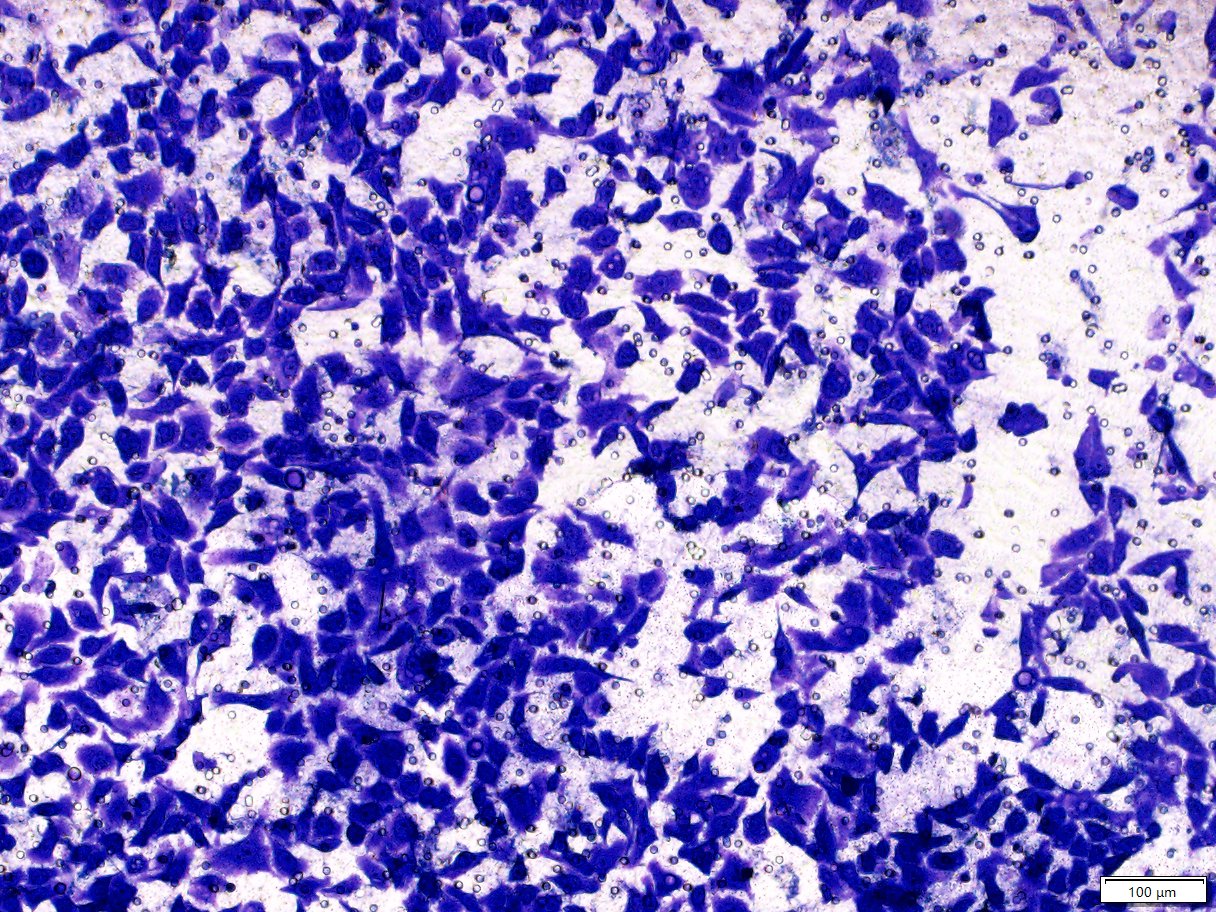

Supplement: Supplemental Information 7 [file peerj-cs-09-1651-s007.zip › Dataset 6/0+6.jpg]

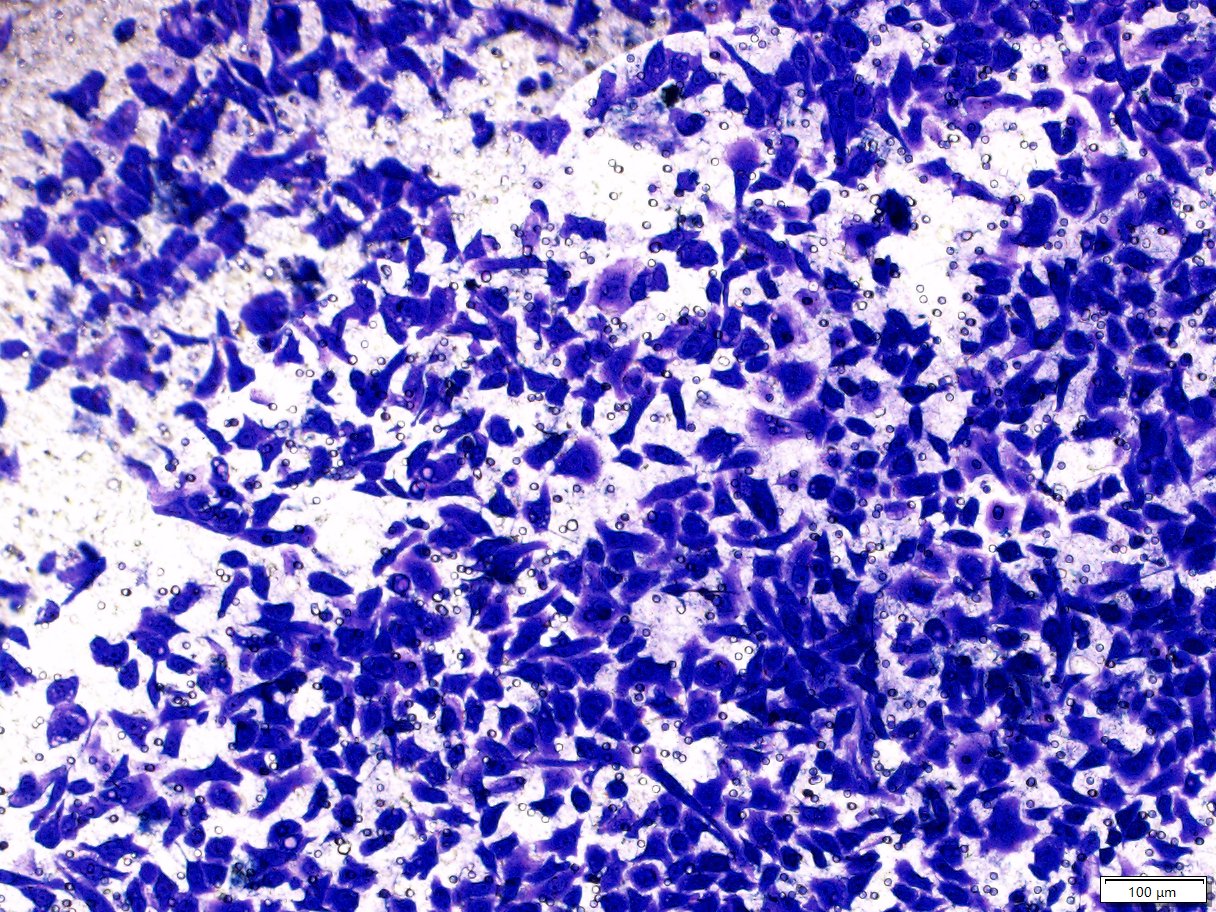

Supplement: Supplemental Information 7 [file peerj-cs-09-1651-s007.zip › Dataset 6/0+7.jpg]

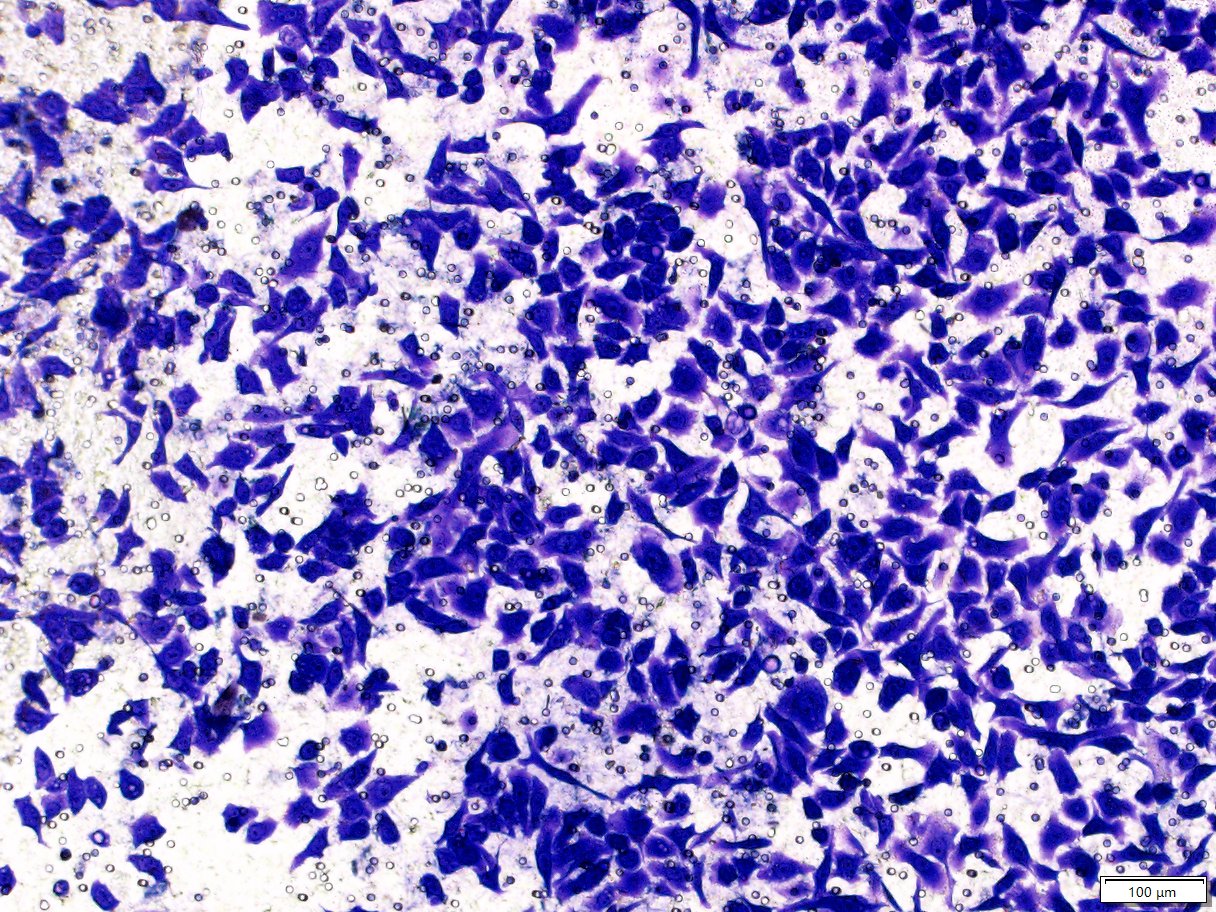

Supplement: Supplemental Information 7 [file peerj-cs-09-1651-s007.zip › Dataset 6/0+8.jpg]

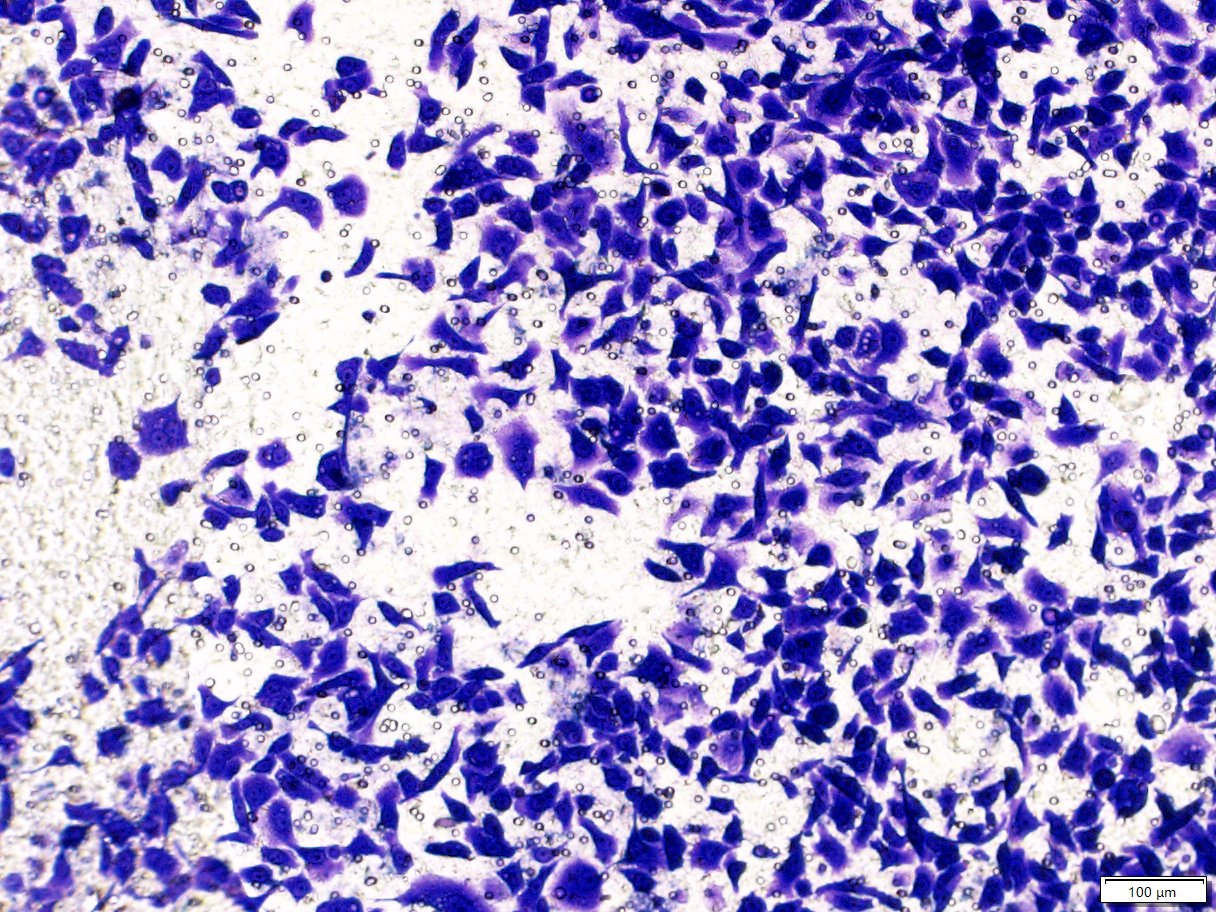

Supplement: Supplemental Information 7 [file peerj-cs-09-1651-s007.zip › Dataset 6/0+9.jpg]

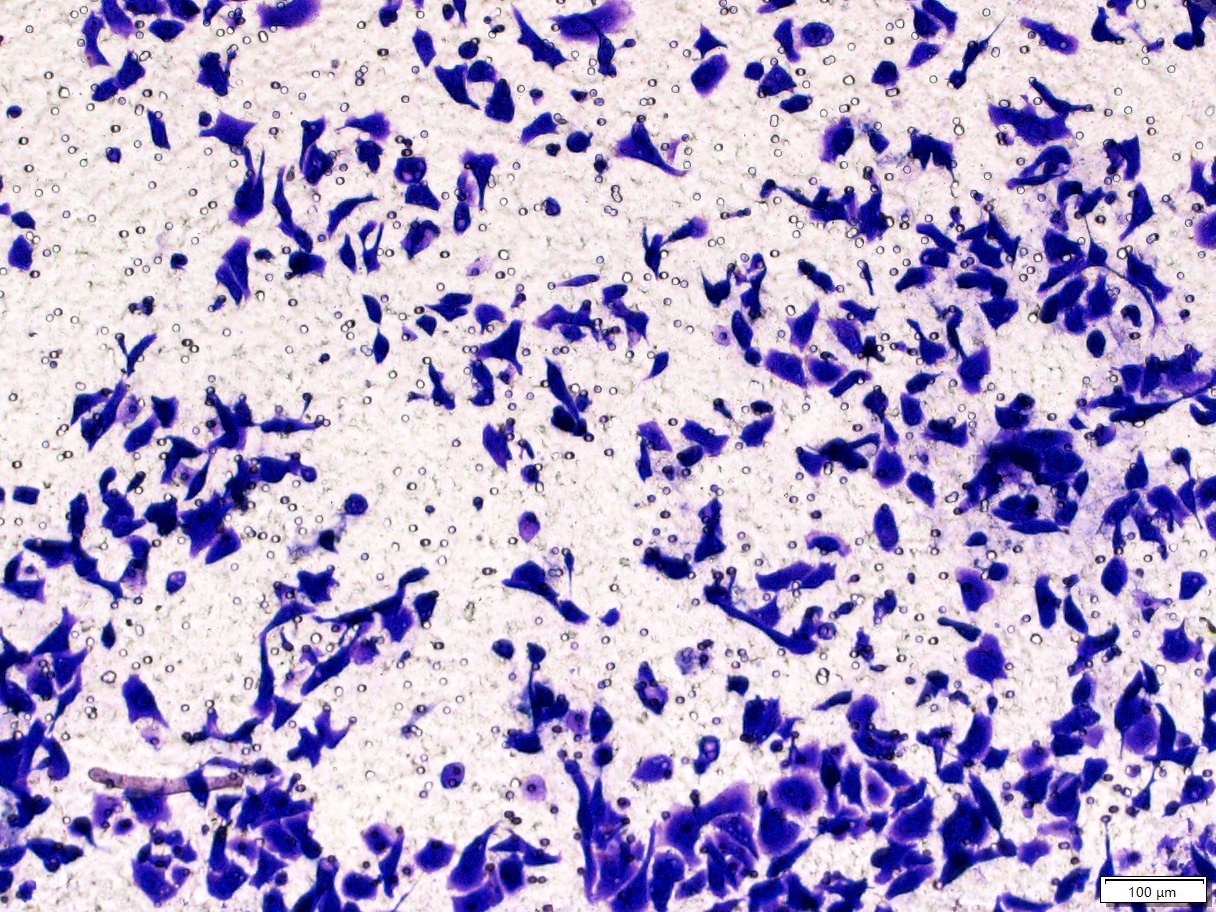

Supplement: Supplemental Information 7 [file peerj-cs-09-1651-s007.zip › Dataset 6/0-1.jpg]

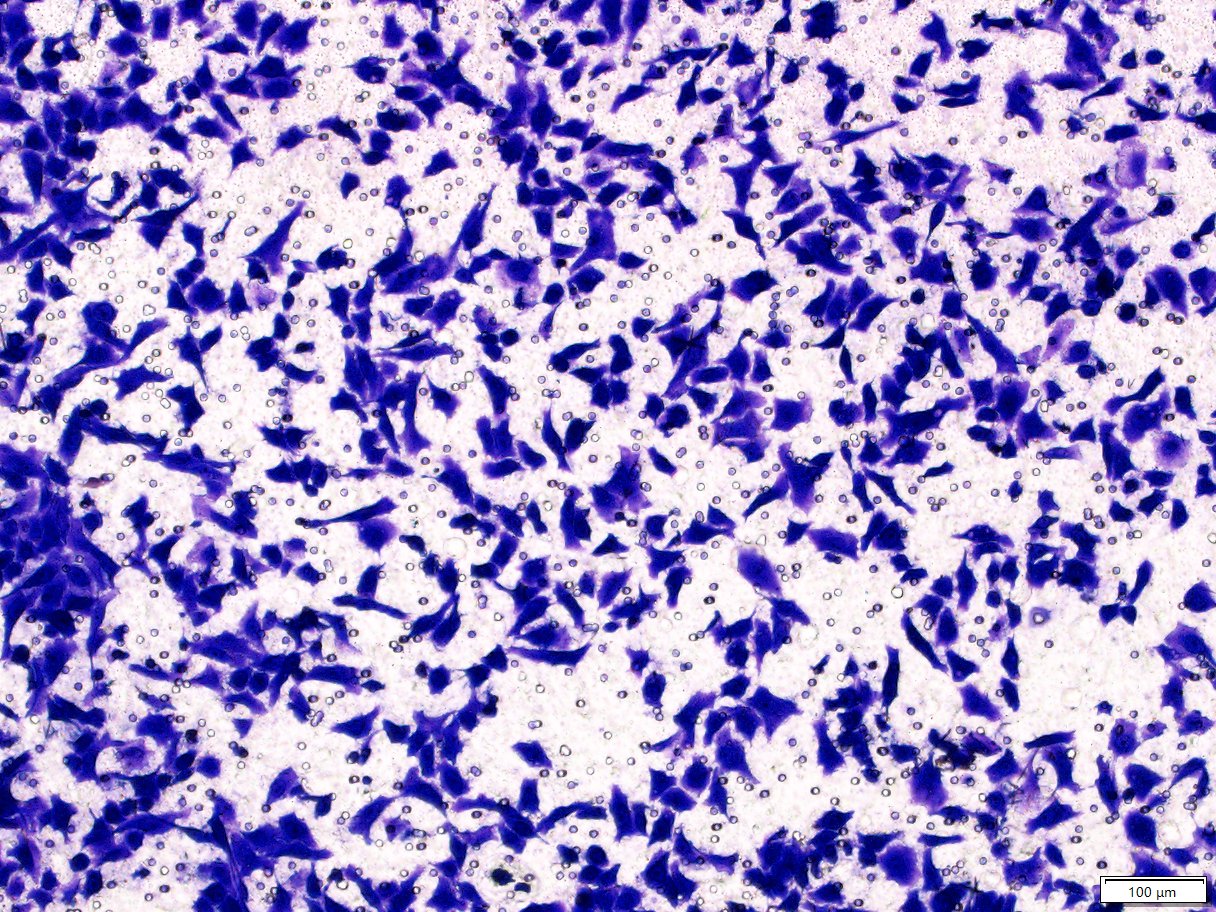

Supplement: Supplemental Information 7 [file peerj-cs-09-1651-s007.zip › Dataset 6/0-10.jpg]

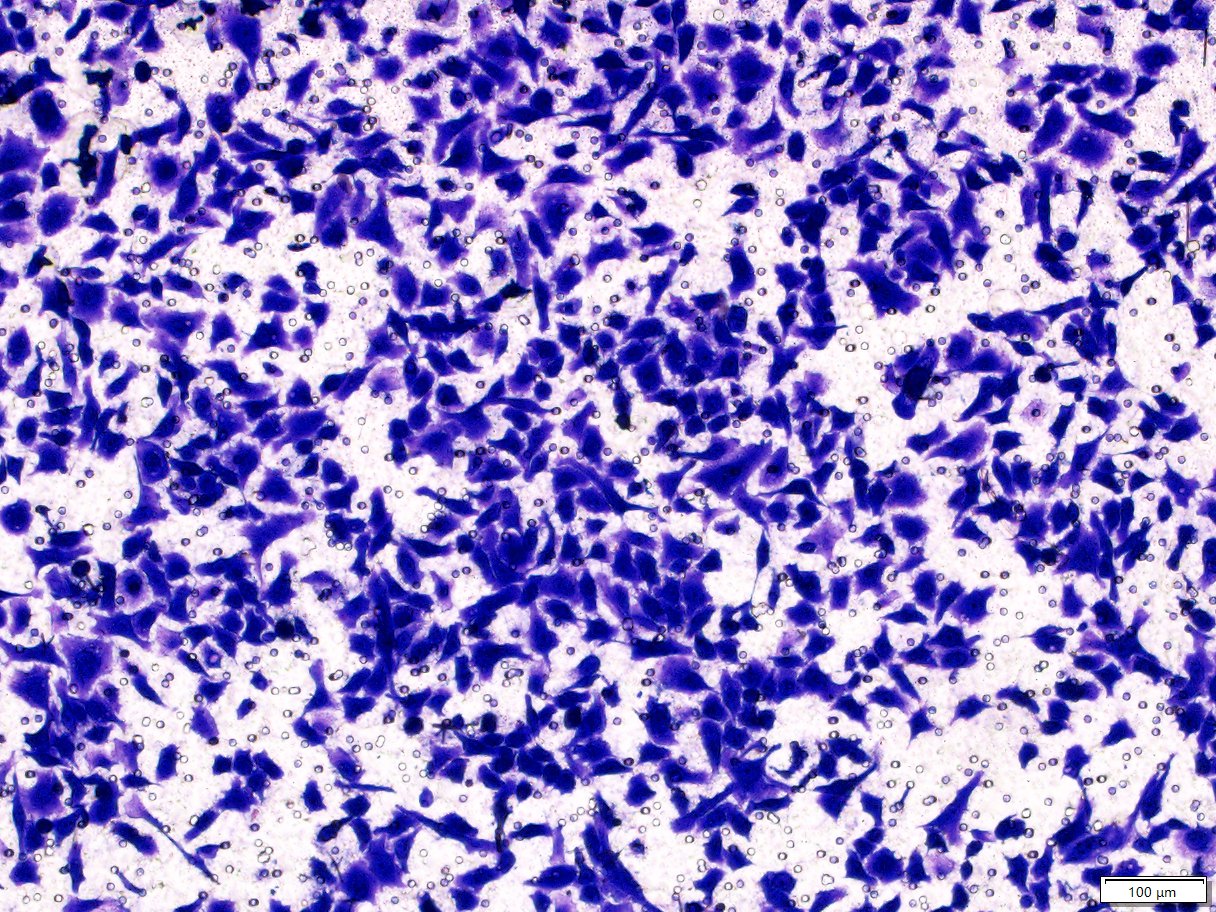

Supplement: Supplemental Information 7 [file peerj-cs-09-1651-s007.zip › Dataset 6/0-11.jpg]

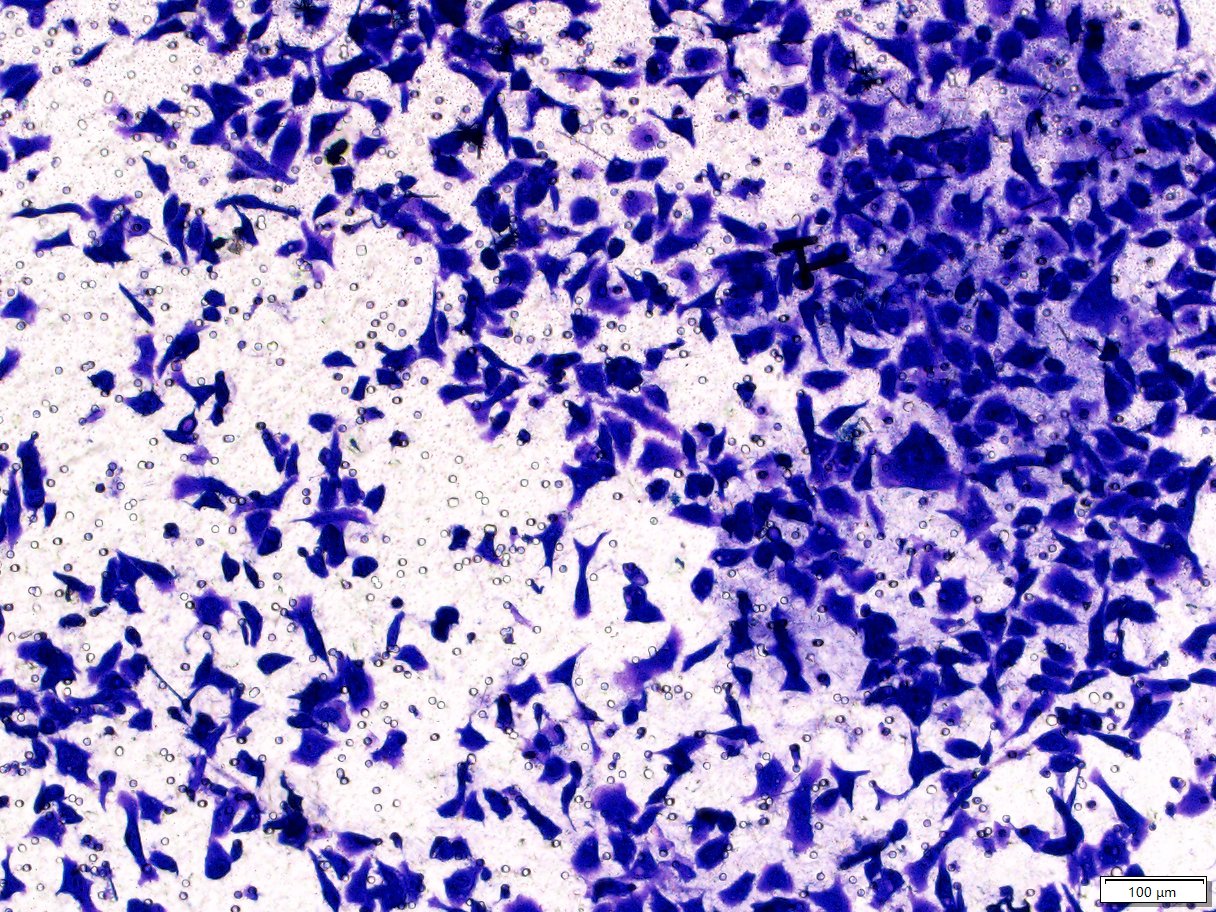

Supplement: Supplemental Information 7 [file peerj-cs-09-1651-s007.zip › Dataset 6/0-2.jpg]

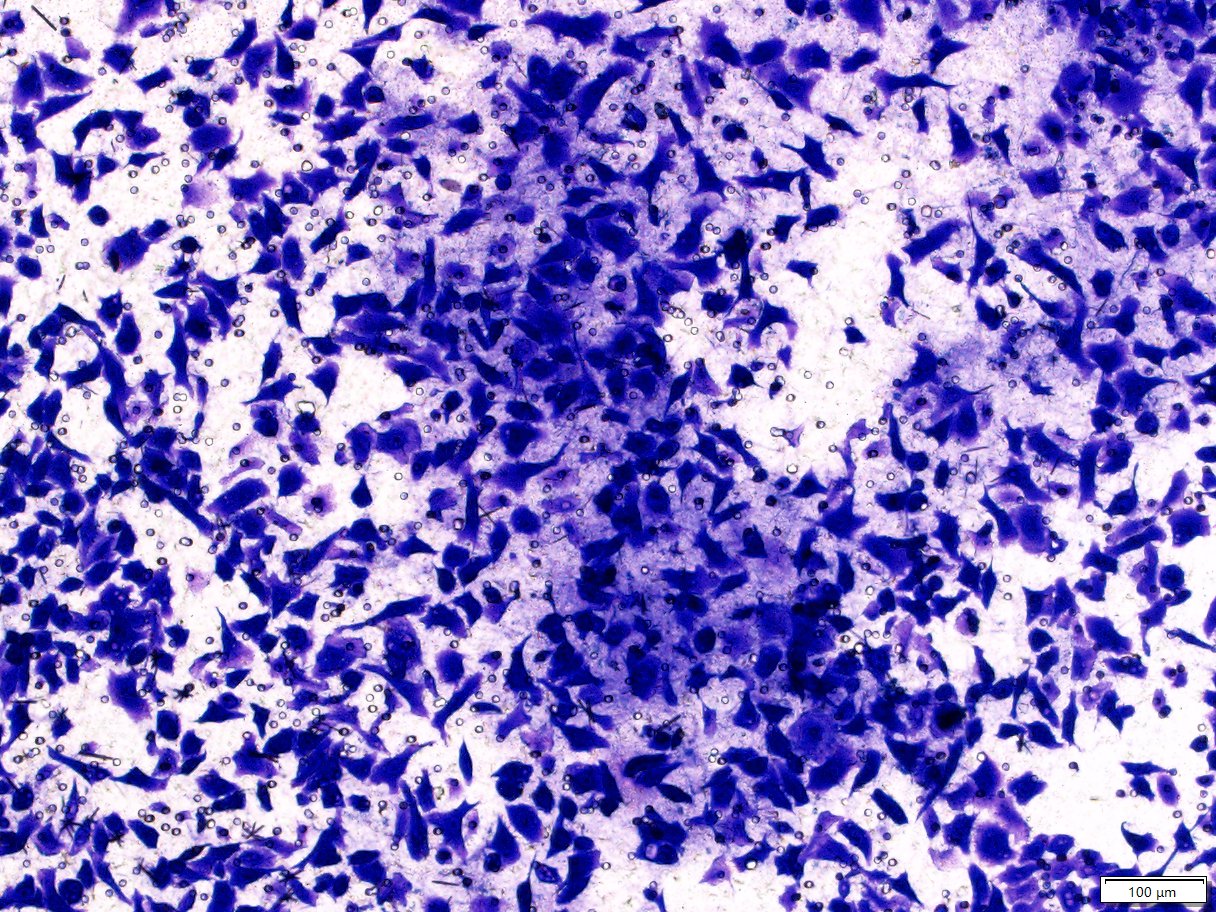

Supplement: Supplemental Information 7 [file peerj-cs-09-1651-s007.zip › Dataset 6/0-3.jpg]

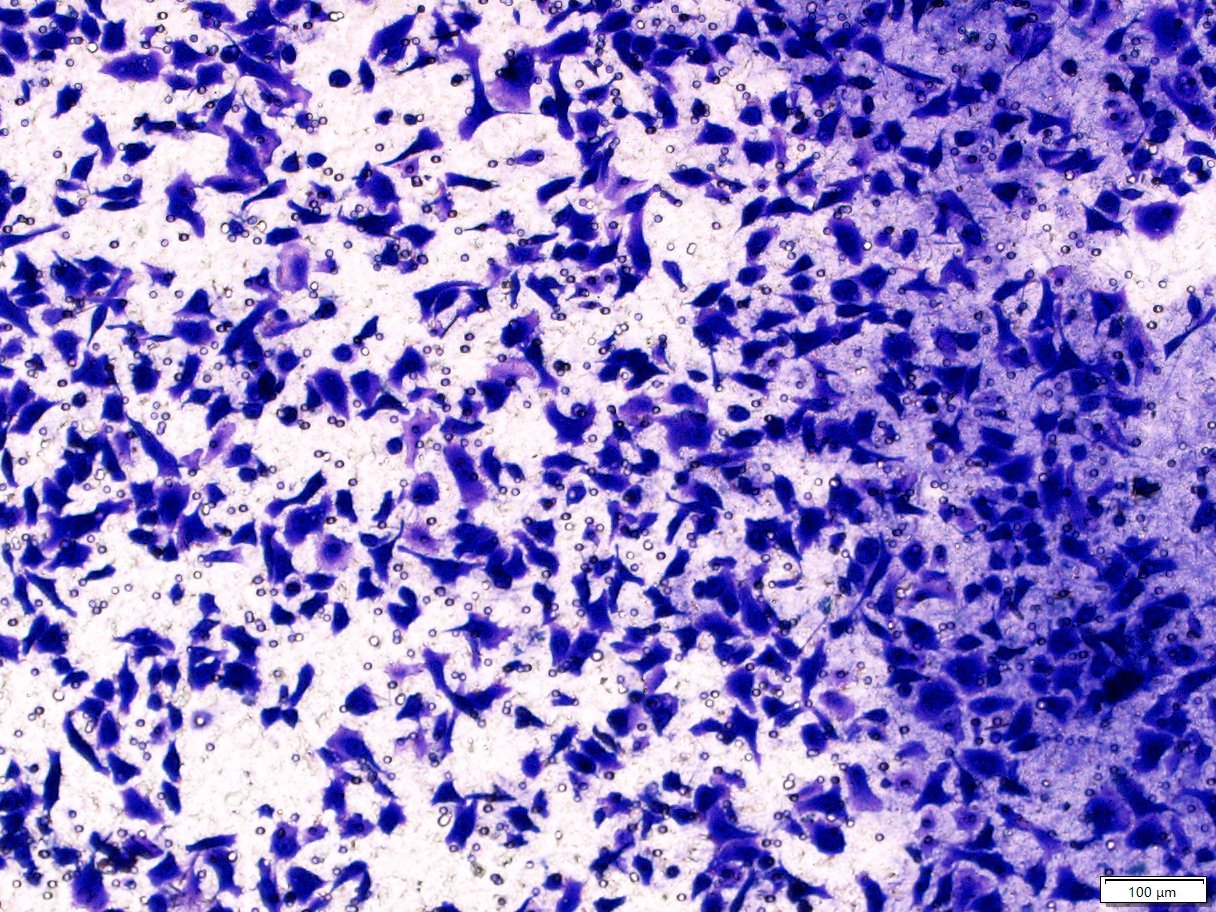

Supplement: Supplemental Information 7 [file peerj-cs-09-1651-s007.zip › Dataset 6/0-4.jpg]

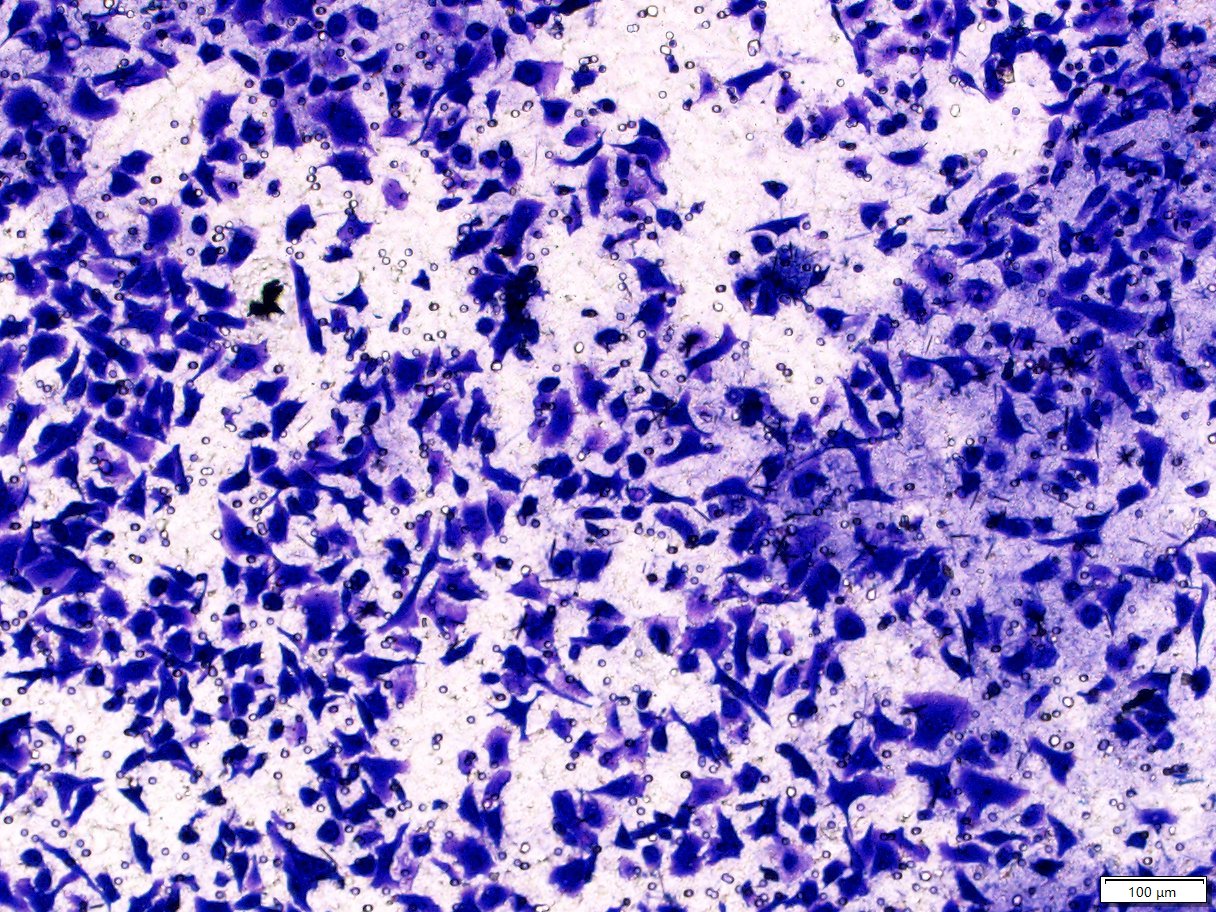

Supplement: Supplemental Information 7 [file peerj-cs-09-1651-s007.zip › Dataset 6/0-5.jpg]

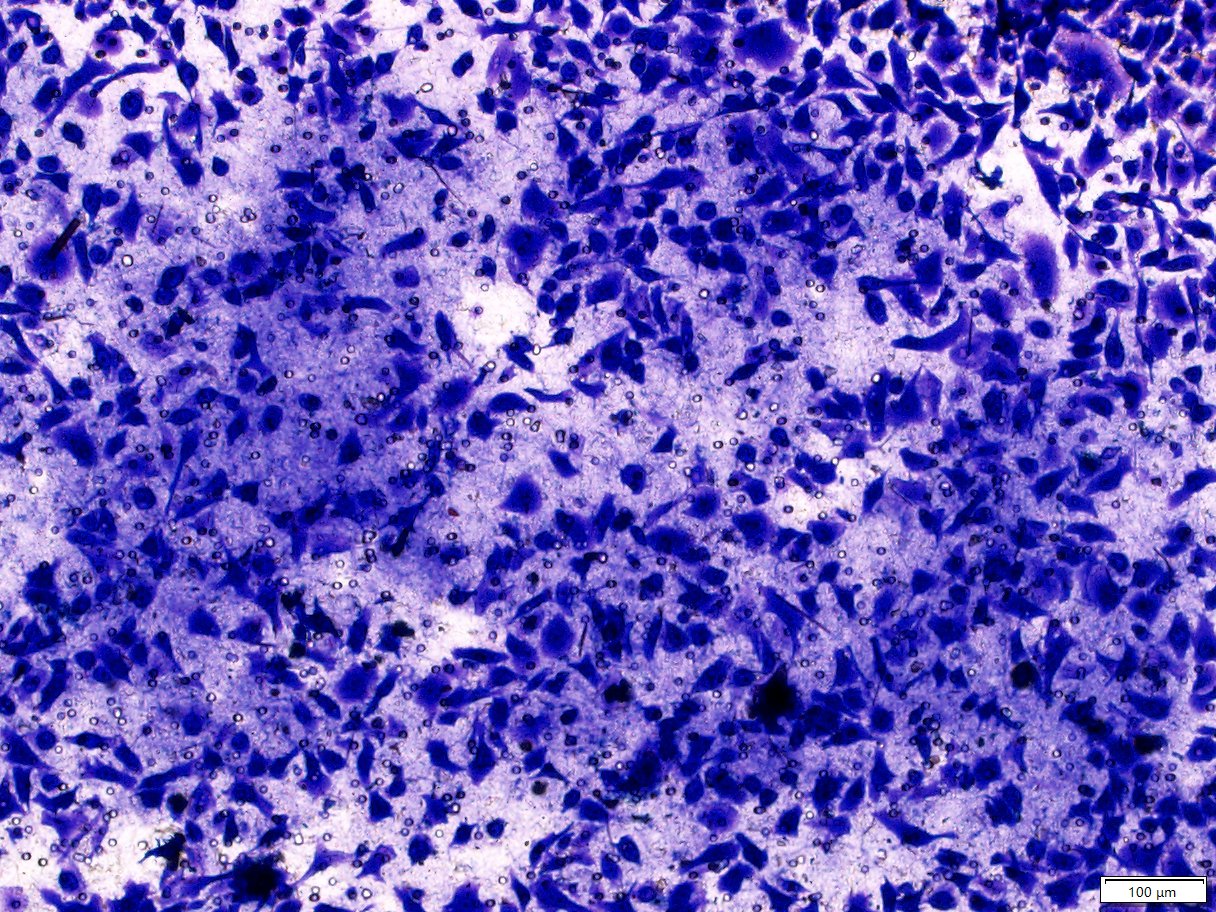

Supplement: Supplemental Information 7 [file peerj-cs-09-1651-s007.zip › Dataset 6/0-6.jpg]

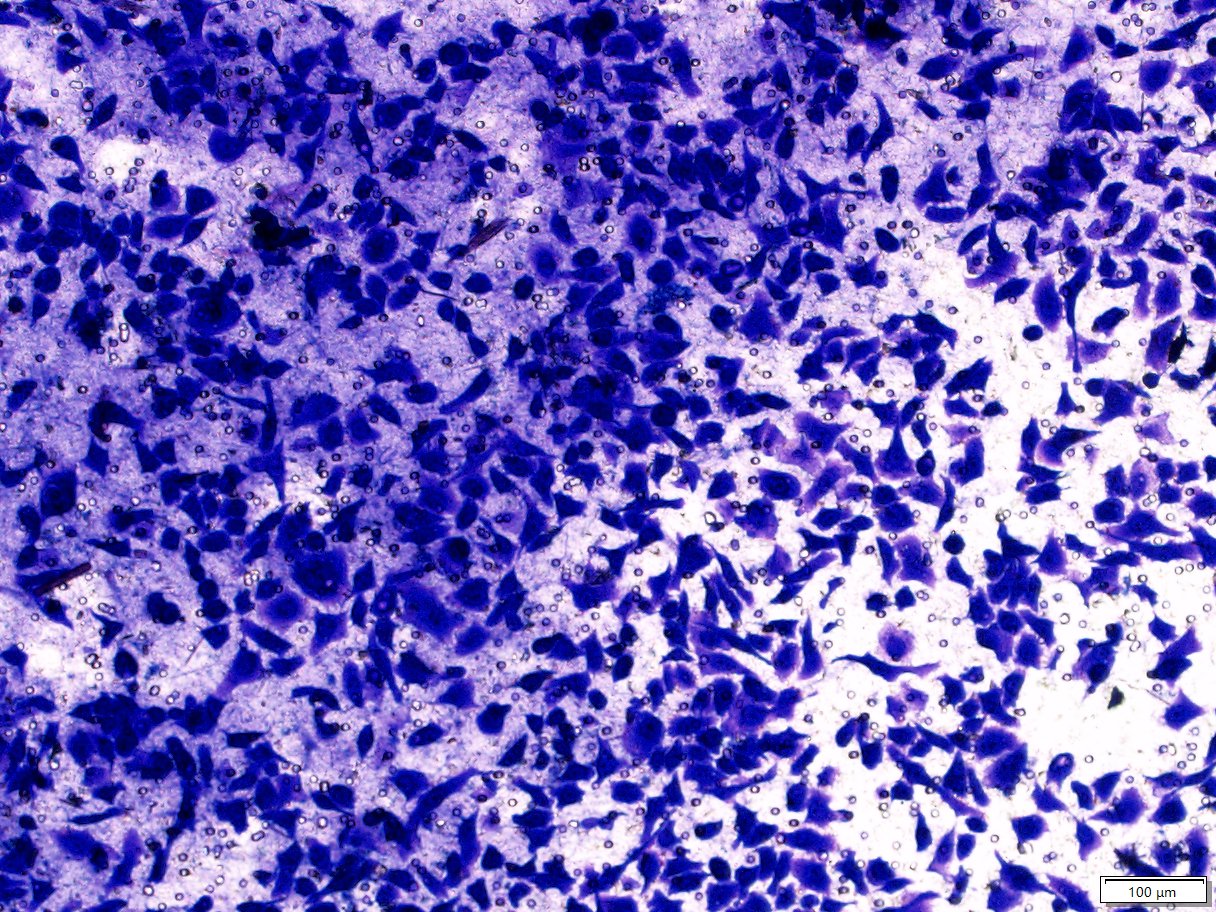

Supplement: Supplemental Information 7 [file peerj-cs-09-1651-s007.zip › Dataset 6/0-7.jpg]

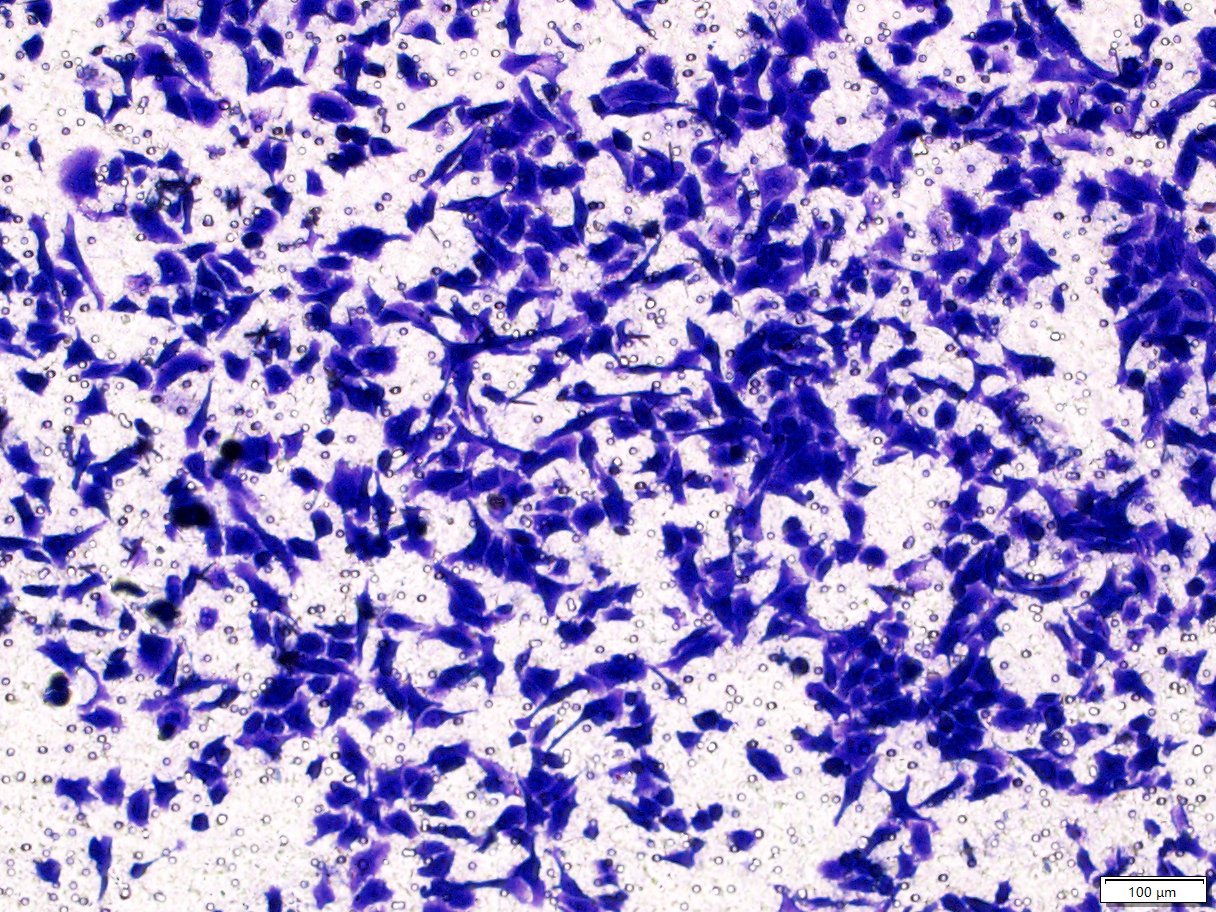

Supplement: Supplemental Information 7 [file peerj-cs-09-1651-s007.zip › Dataset 6/0-8.jpg]

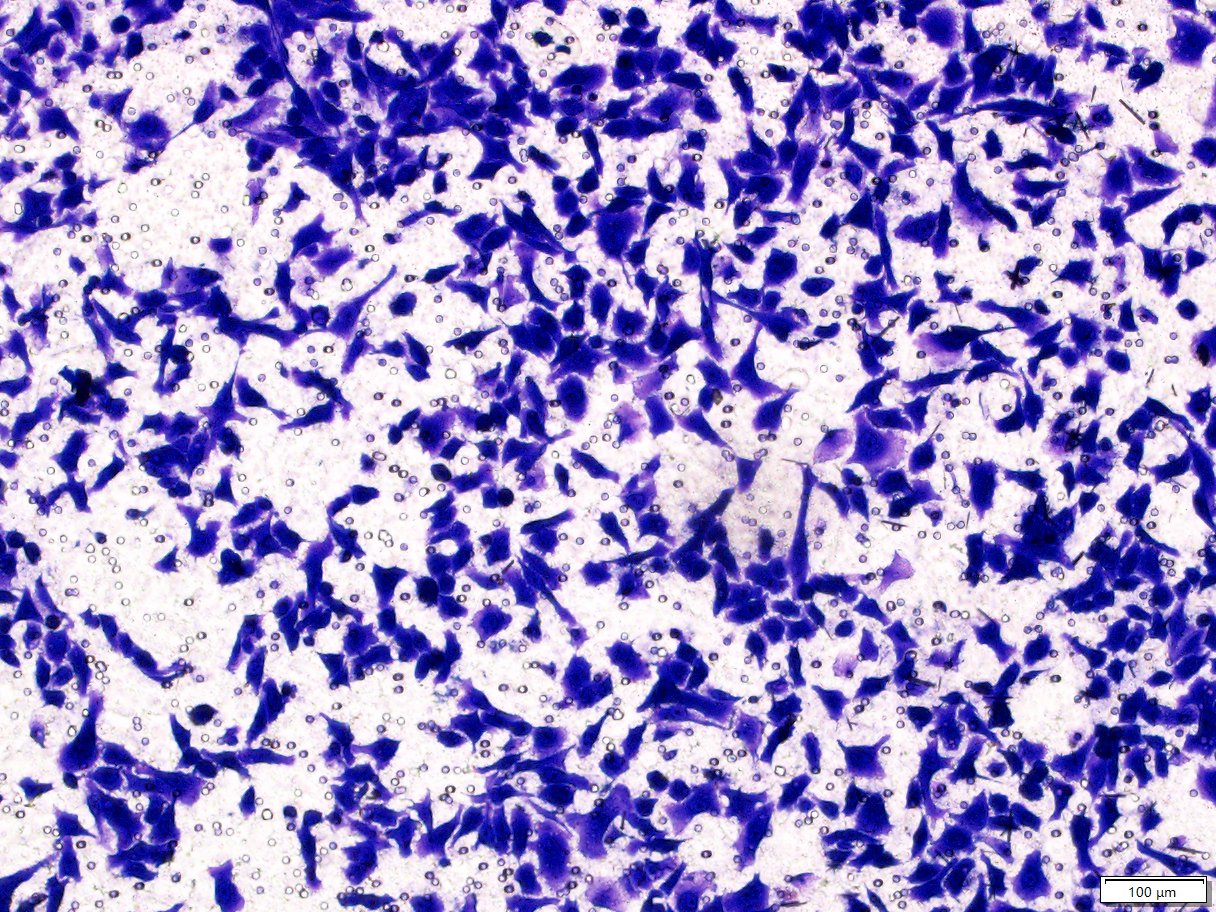

Supplement: Supplemental Information 7 [file peerj-cs-09-1651-s007.zip › Dataset 6/0-9.jpg]

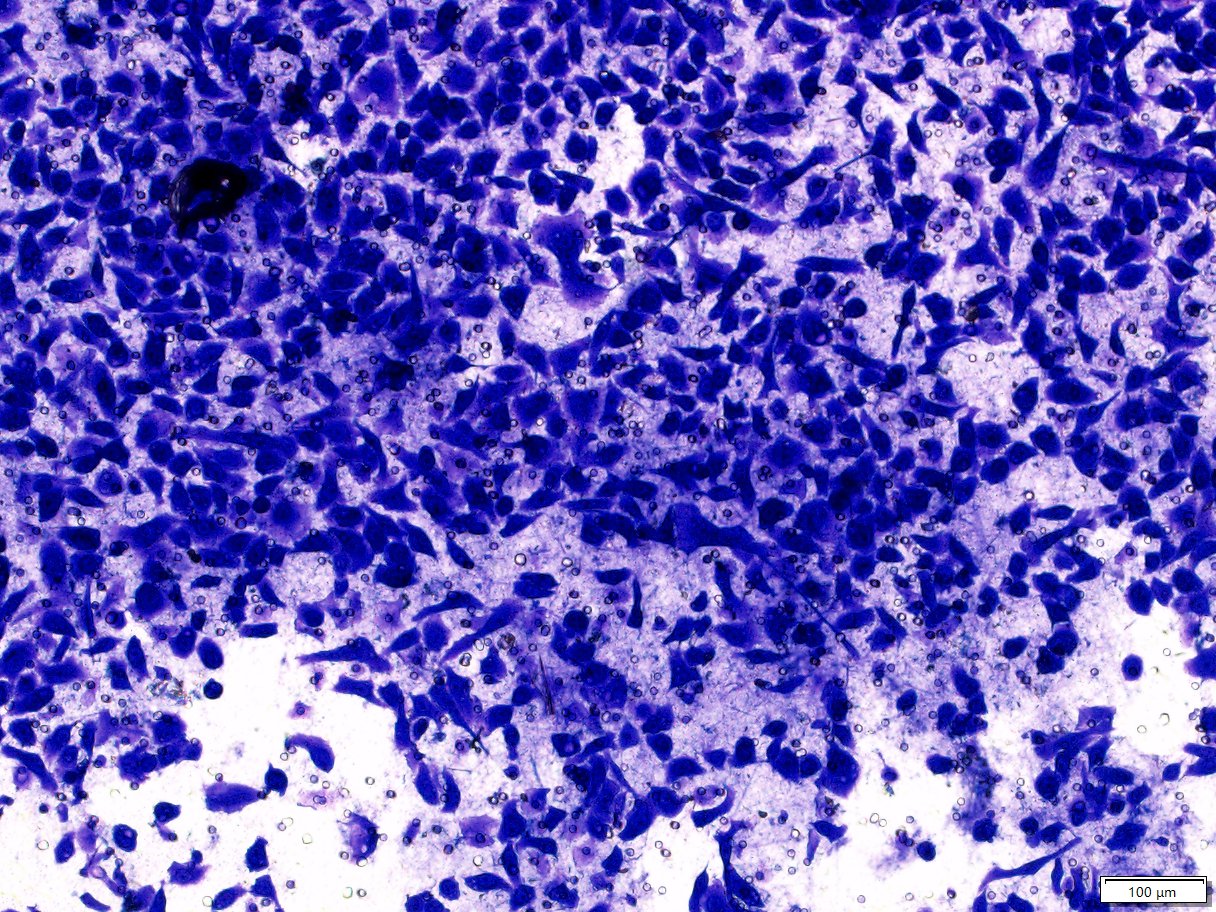

Supplement: Supplemental Information 7 [file peerj-cs-09-1651-s007.zip › Dataset 6/1+1.jpg]

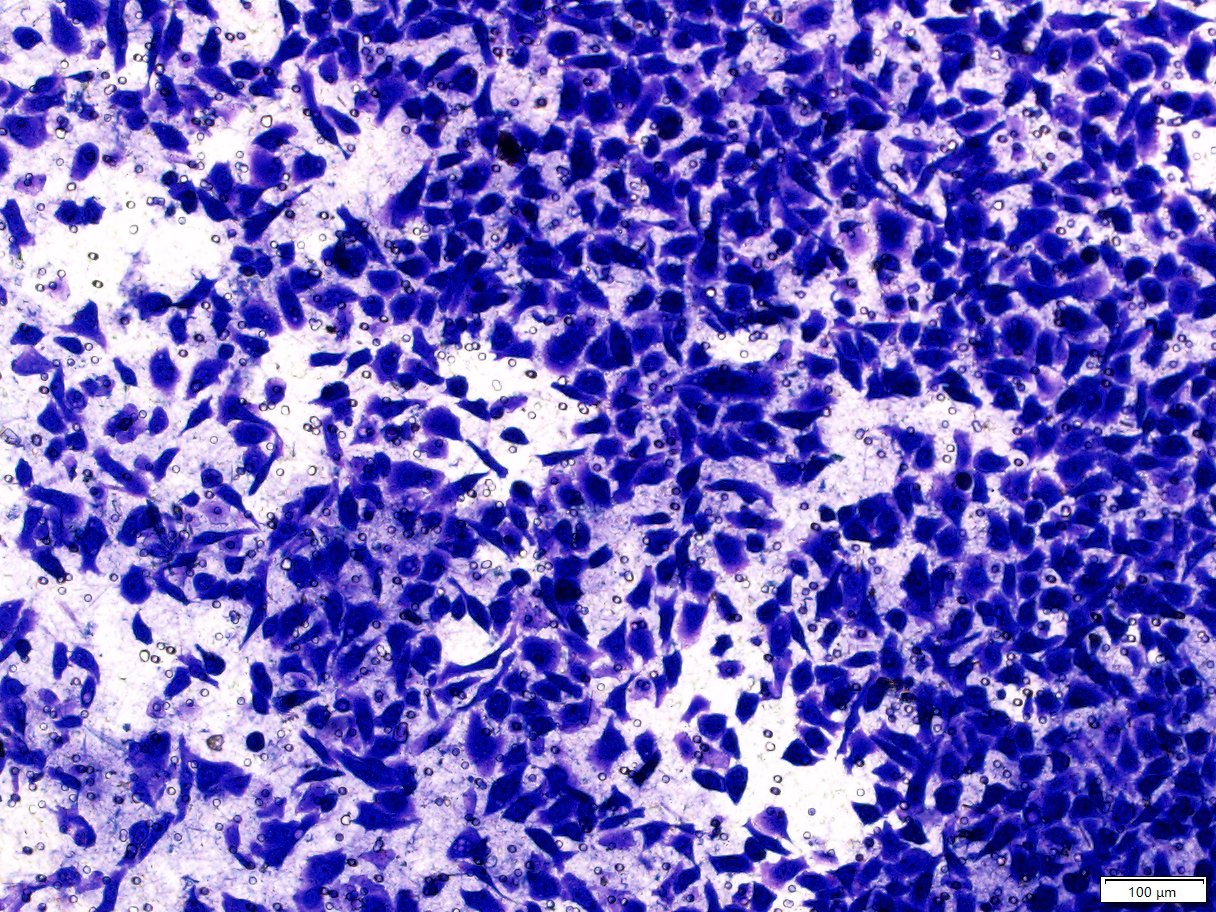

Supplement: Supplemental Information 7 [file peerj-cs-09-1651-s007.zip › Dataset 6/1+10.jpg]

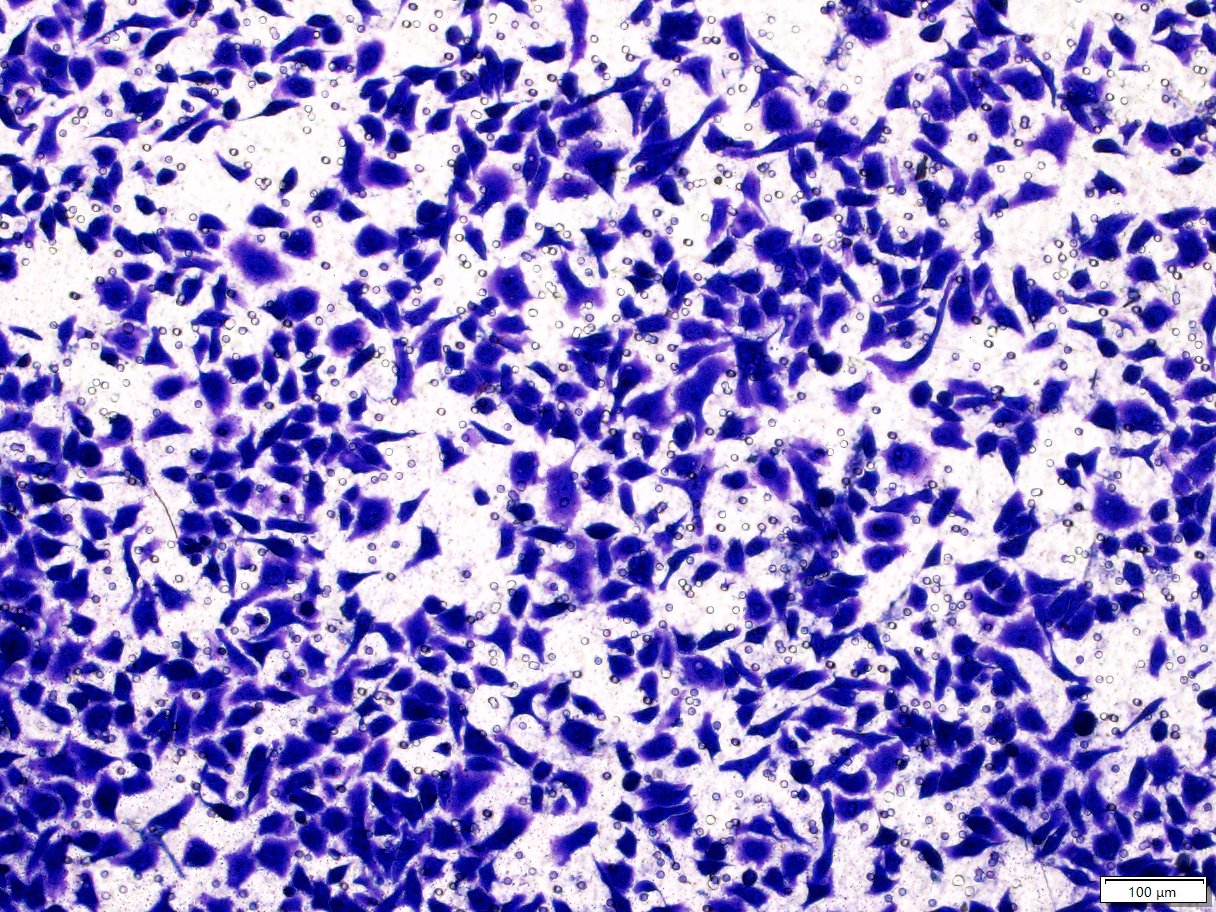

Supplement: Supplemental Information 7 [file peerj-cs-09-1651-s007.zip › Dataset 6/1+11.jpg]

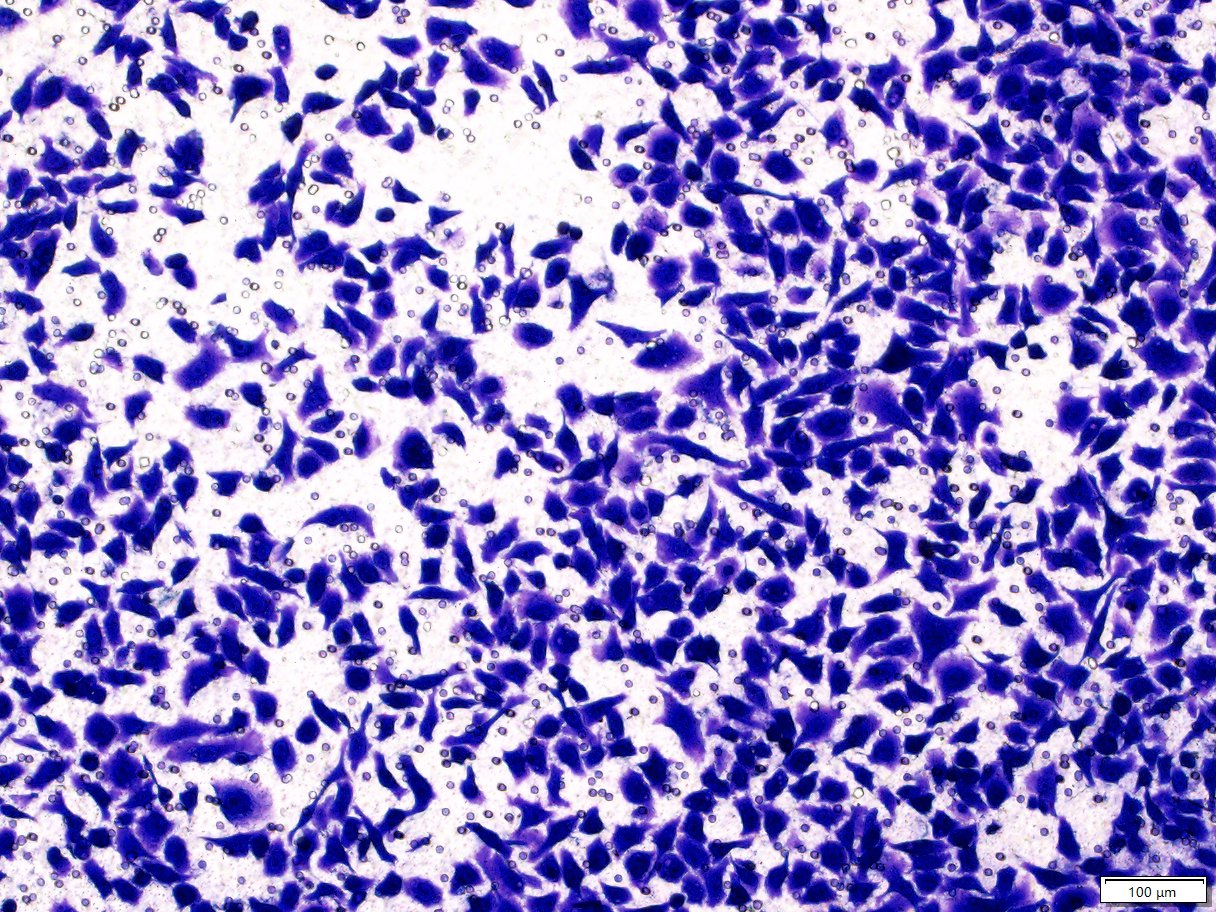

Supplement: Supplemental Information 7 [file peerj-cs-09-1651-s007.zip › Dataset 6/1+12.jpg]

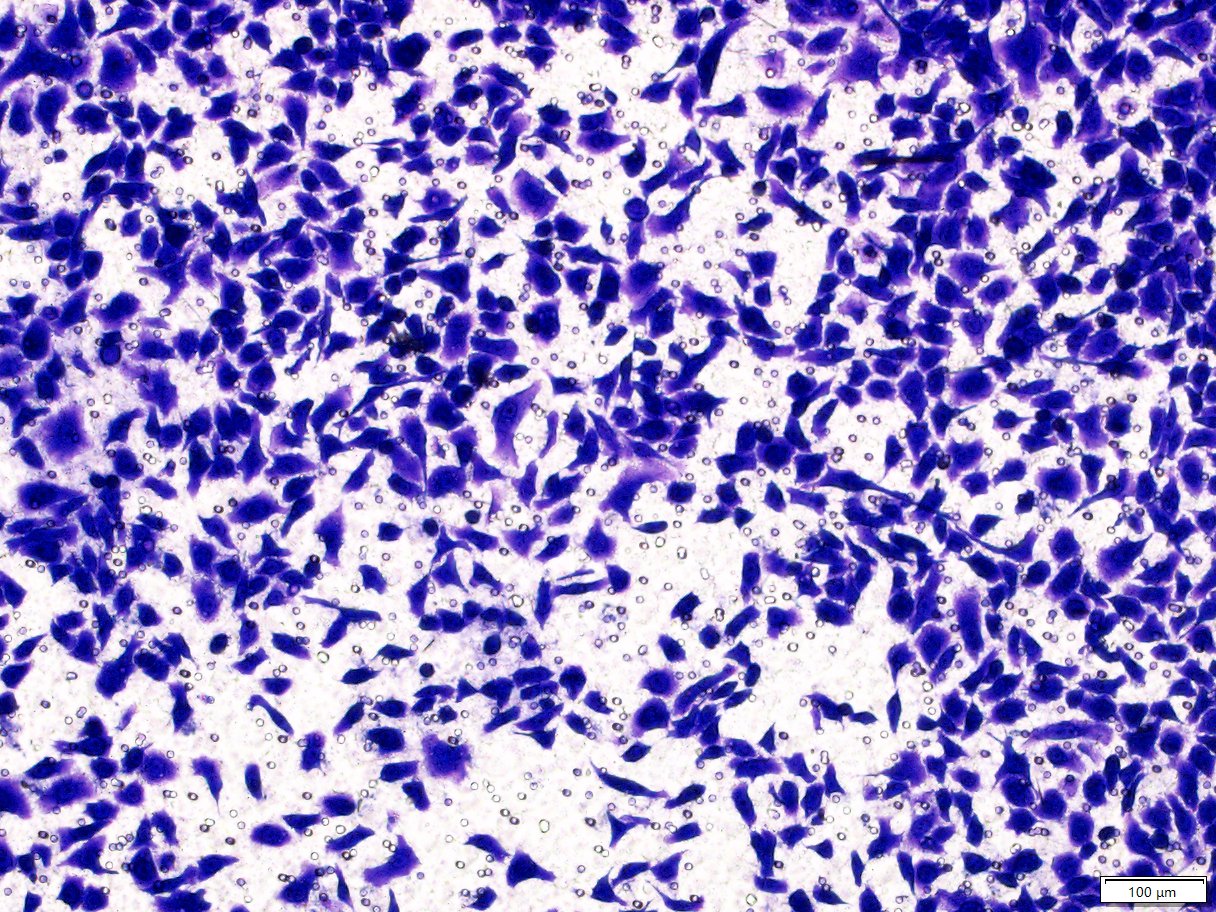

Supplement: Supplemental Information 7 [file peerj-cs-09-1651-s007.zip › Dataset 6/1+13.jpg]

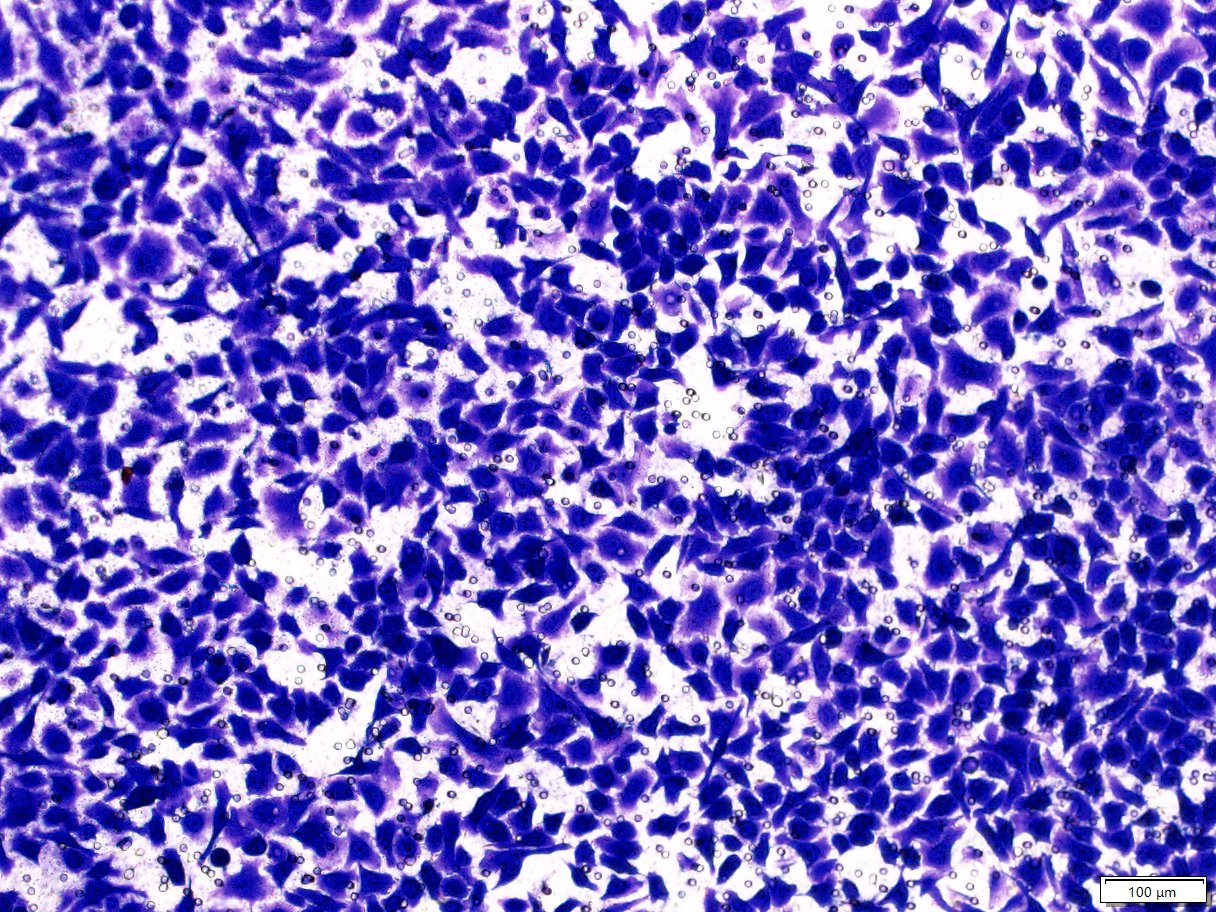

Supplement: Supplemental Information 7 [file peerj-cs-09-1651-s007.zip › Dataset 6/1+14.jpg]

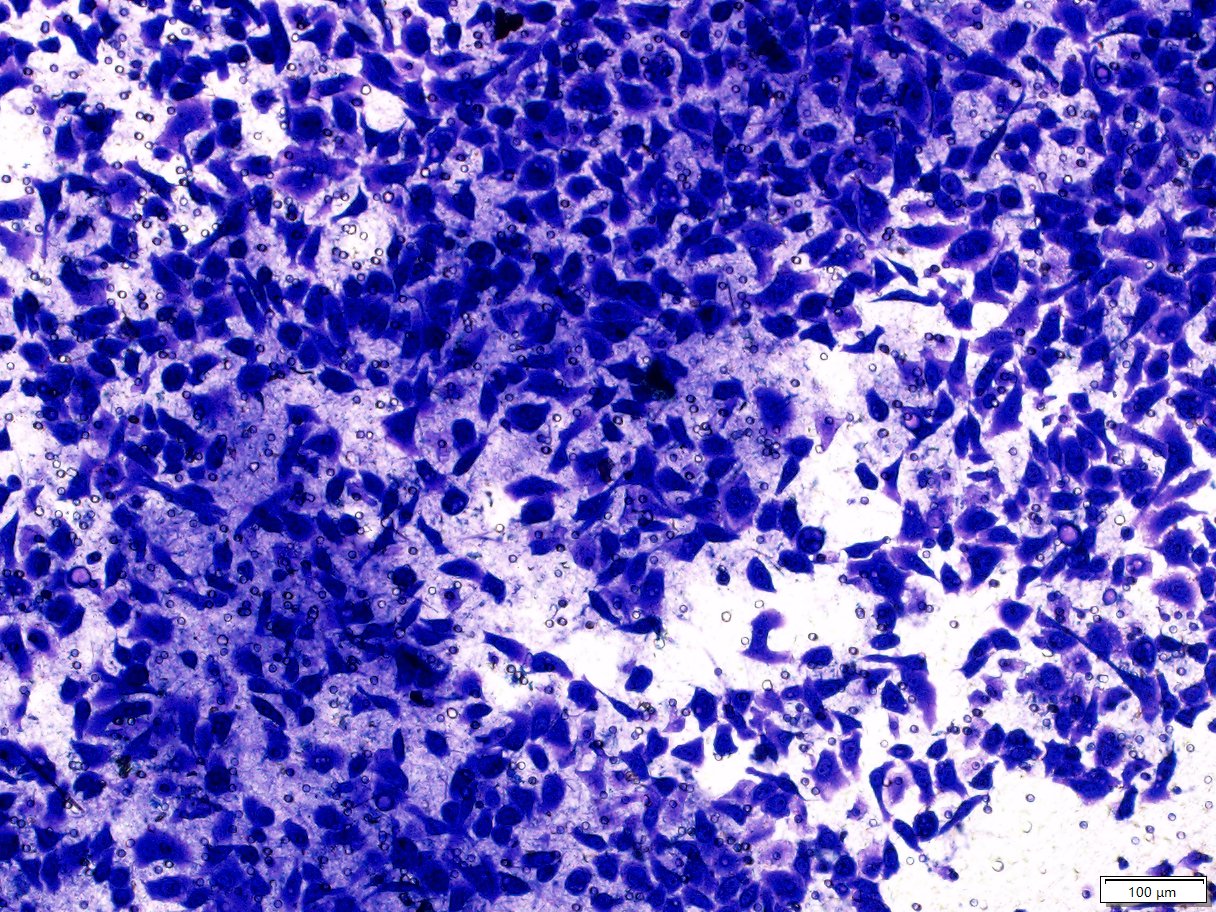

Supplement: Supplemental Information 7 [file peerj-cs-09-1651-s007.zip › Dataset 6/1+2.jpg]

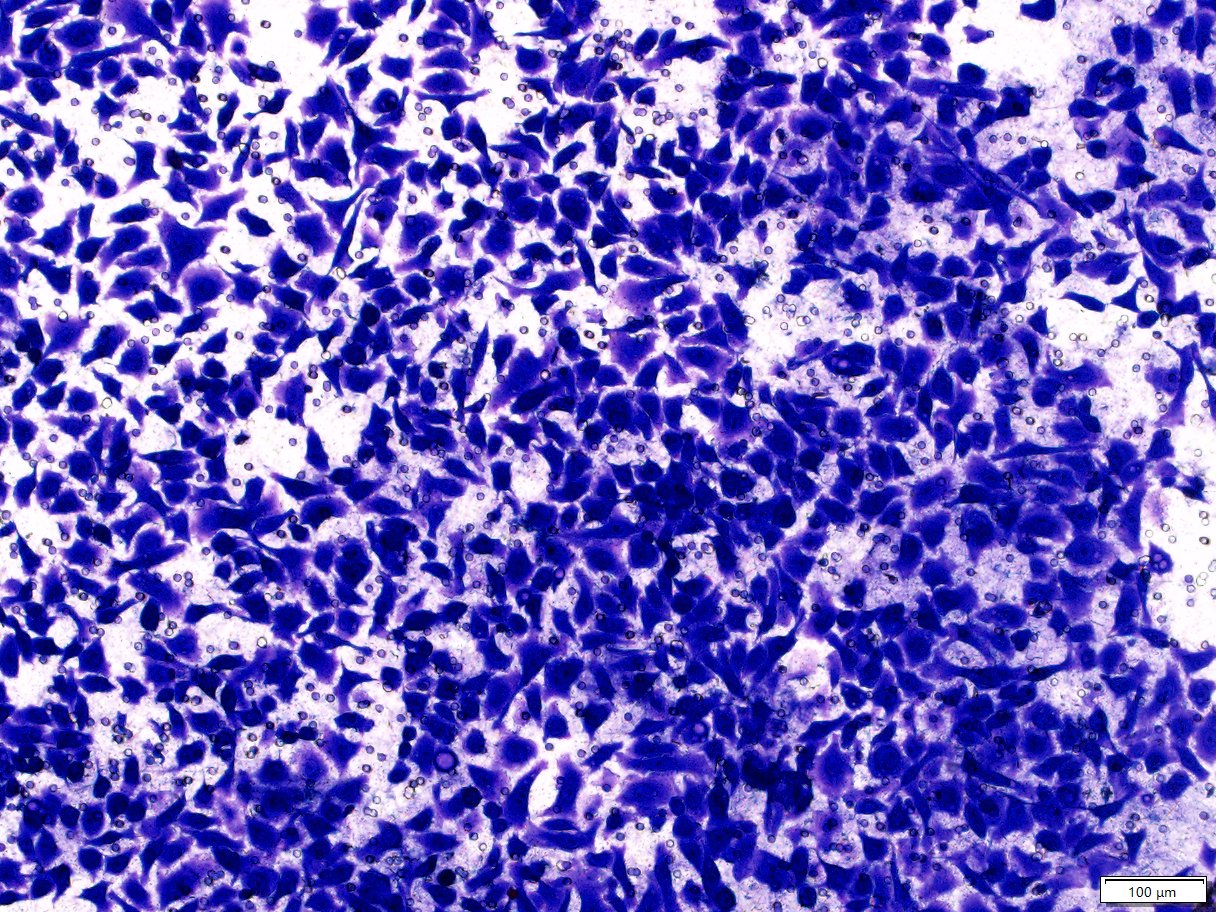

Supplement: Supplemental Information 7 [file peerj-cs-09-1651-s007.zip › Dataset 6/1+3.jpg]
