# Supplementary figures and images for: A deep learning approach for the detection and counting of colon cancer cells (HT-29 cells) bunches and impurities (part 4 of 6)
Source: PeerJ Comput Sci. 2023 Dec 5;9:e1651. doi: 10.7717/peerj-cs.1651 (PMC10773923; doi:10.7717/peerj-cs.1651)

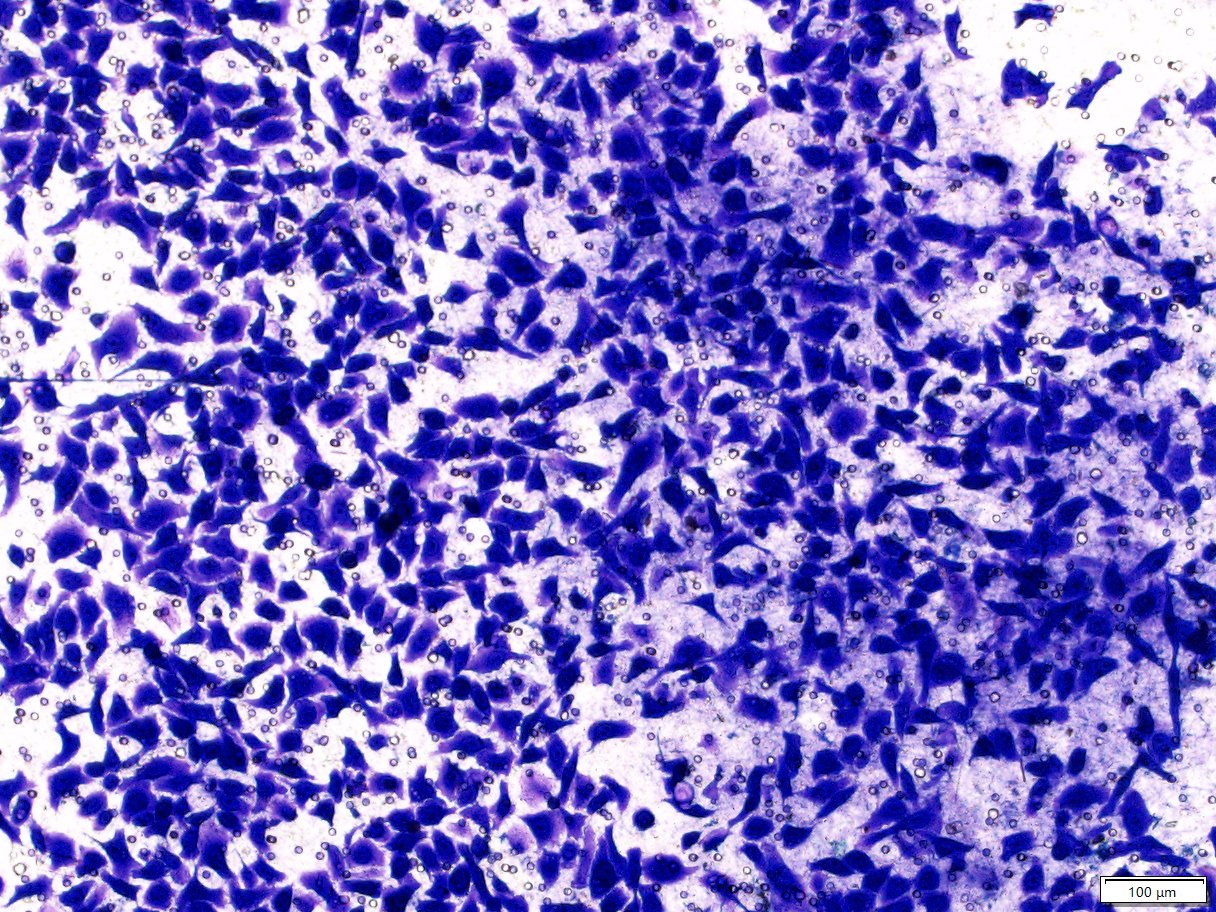

Supplement: Supplemental Information 7 [file peerj-cs-09-1651-s007.zip › Dataset 6/1+4.jpg]

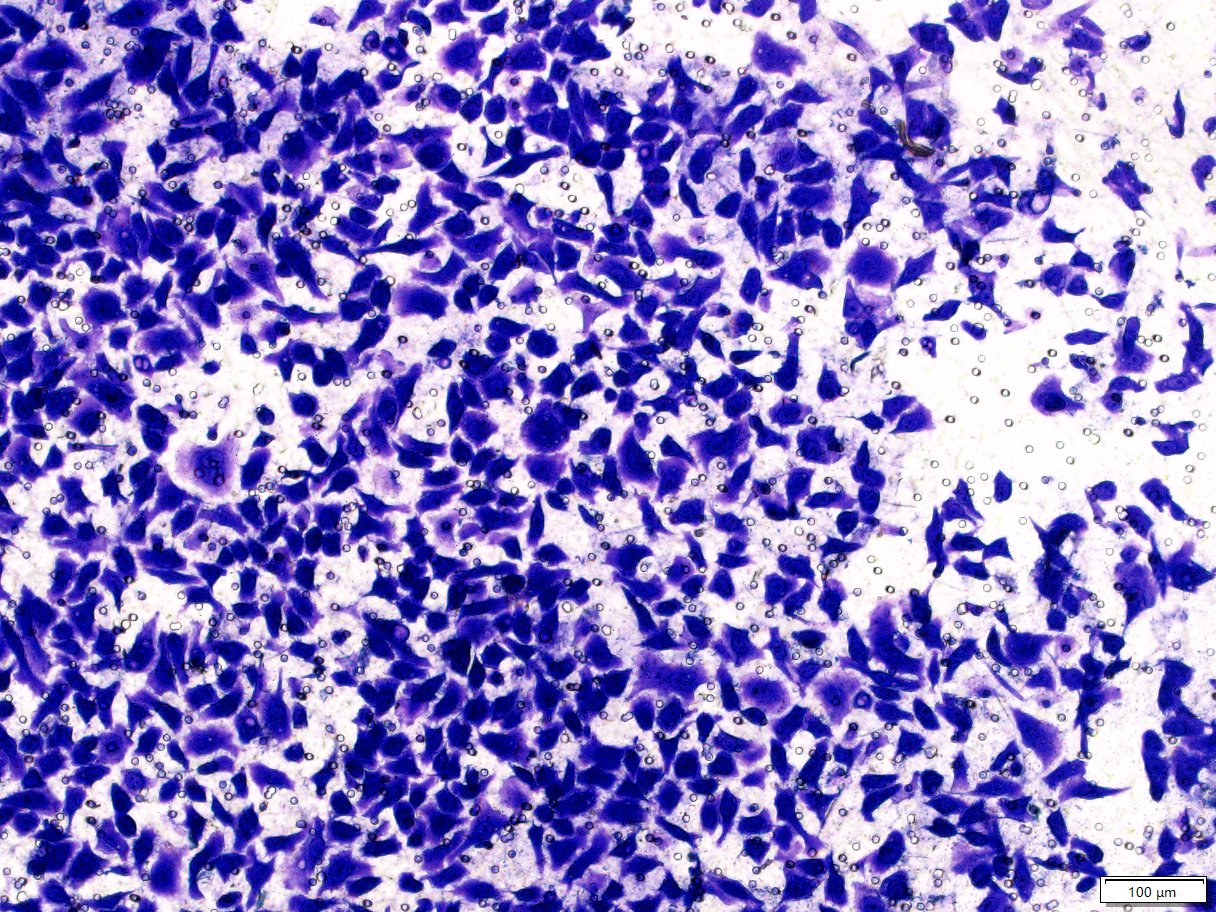

Supplement: Supplemental Information 7 [file peerj-cs-09-1651-s007.zip › Dataset 6/1+5.jpg]

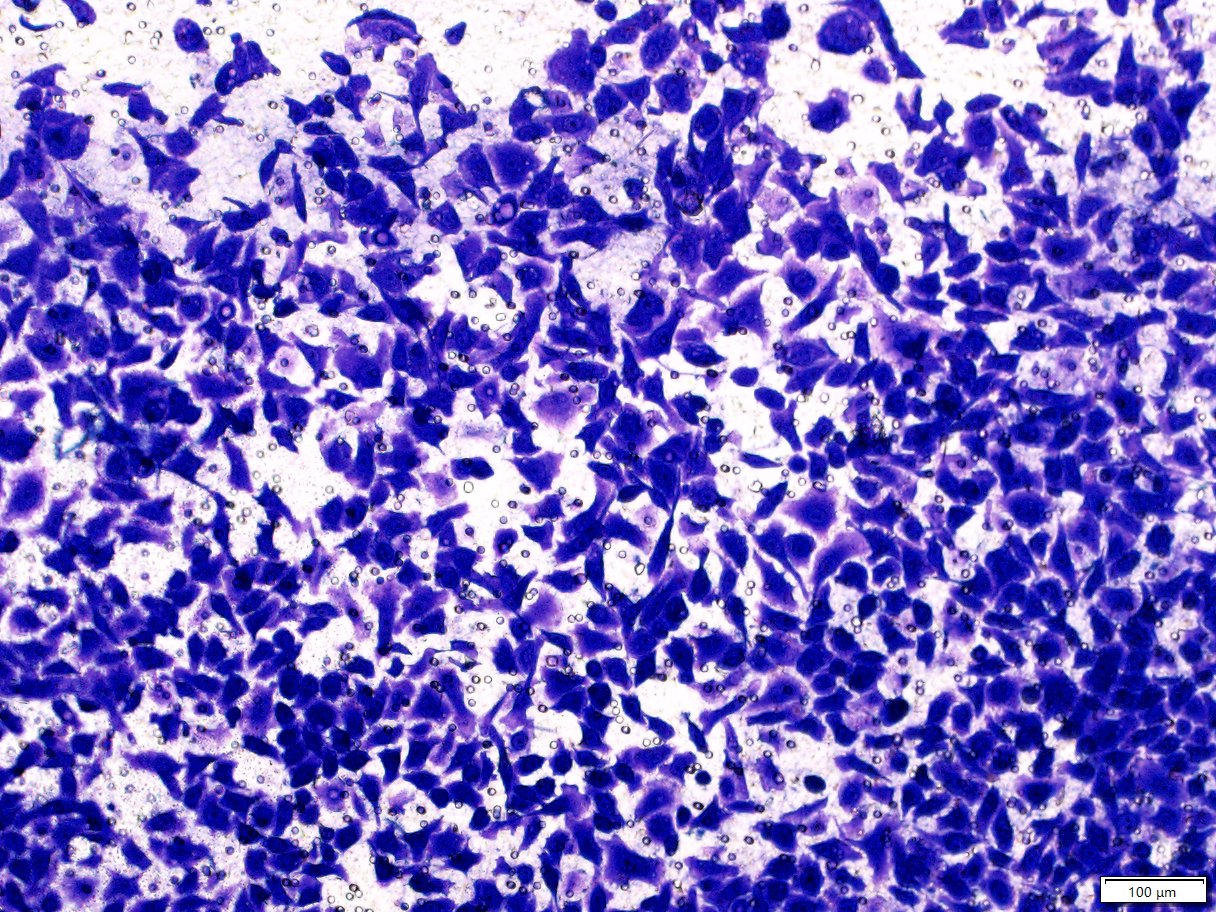

Supplement: Supplemental Information 7 [file peerj-cs-09-1651-s007.zip › Dataset 6/1+6.jpg]

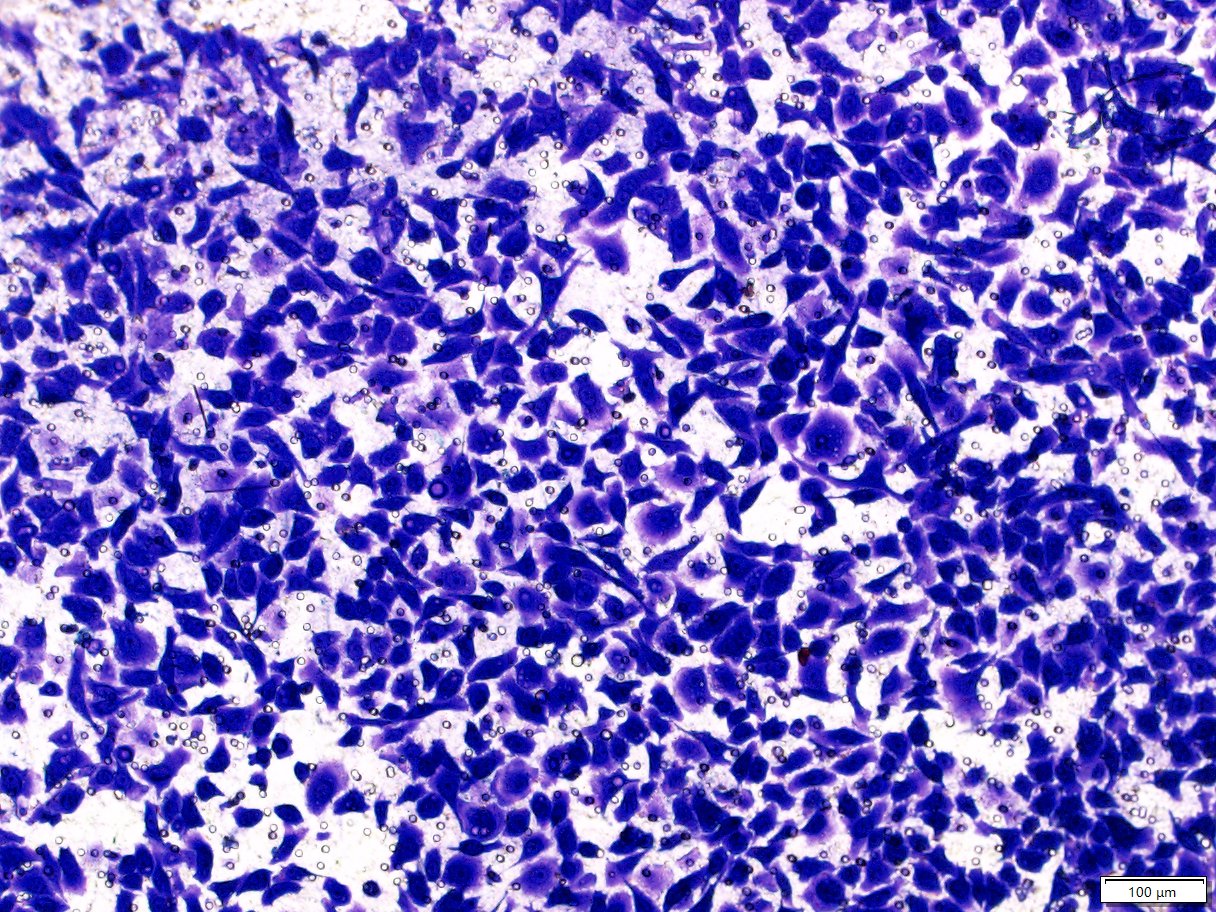

Supplement: Supplemental Information 7 [file peerj-cs-09-1651-s007.zip › Dataset 6/1+7.jpg]

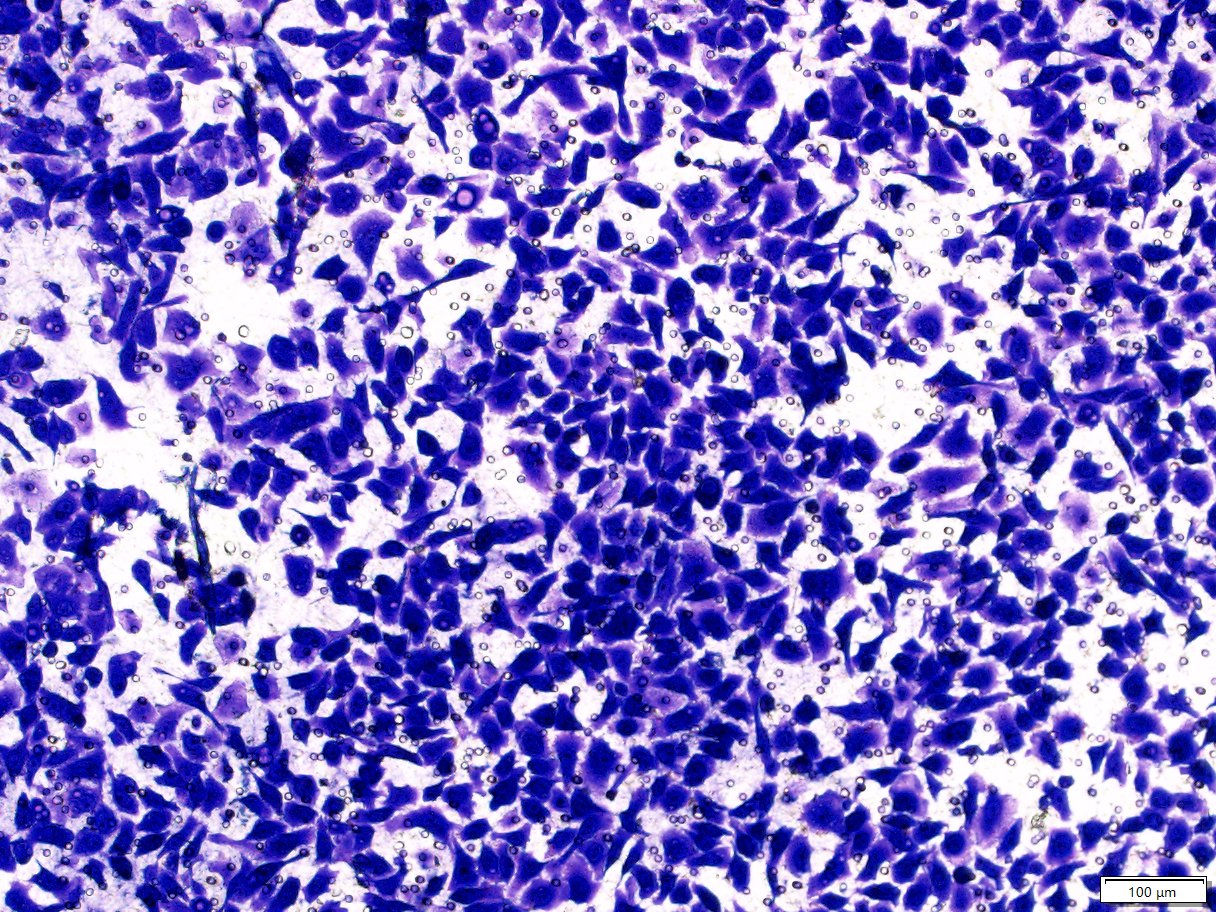

Supplement: Supplemental Information 7 [file peerj-cs-09-1651-s007.zip › Dataset 6/1+8.jpg]

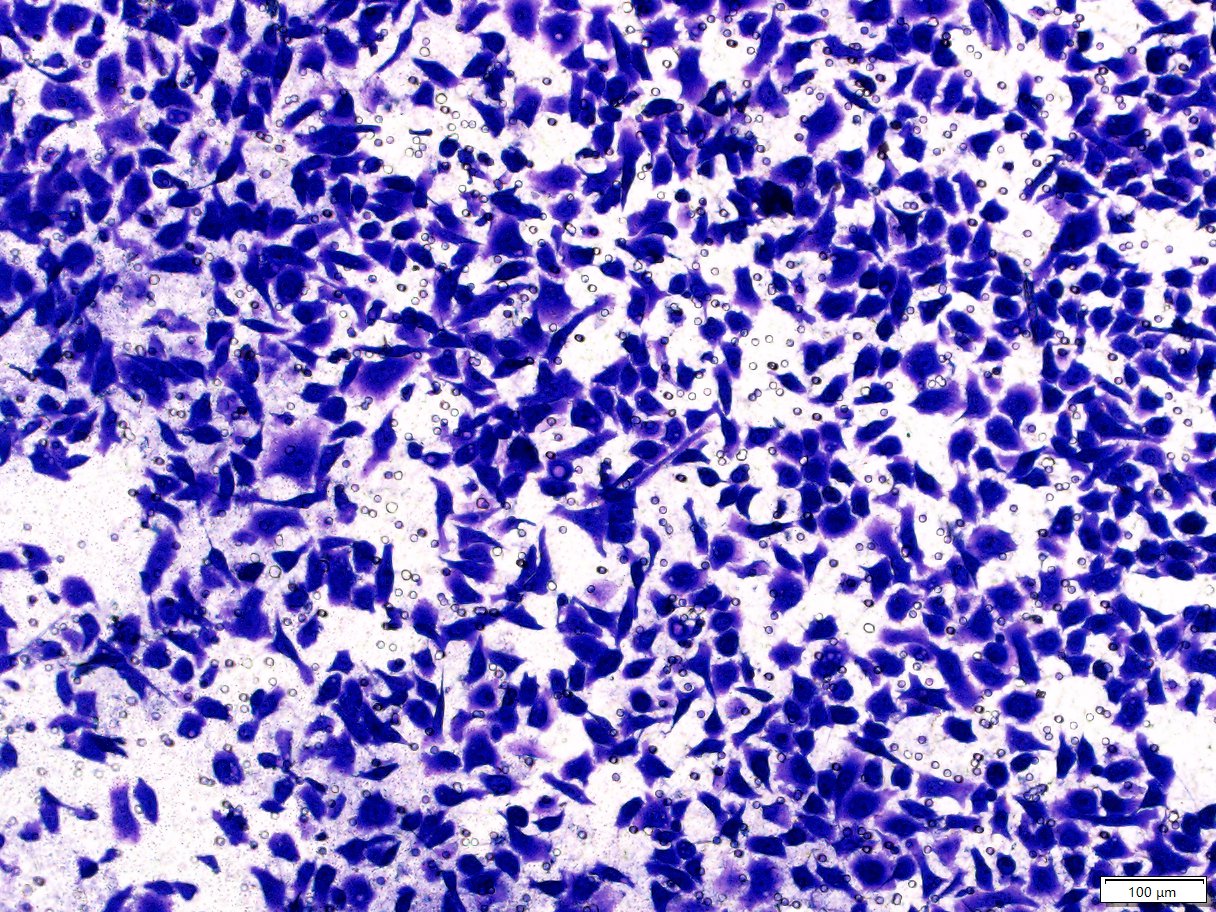

Supplement: Supplemental Information 7 [file peerj-cs-09-1651-s007.zip › Dataset 6/1+9.jpg]

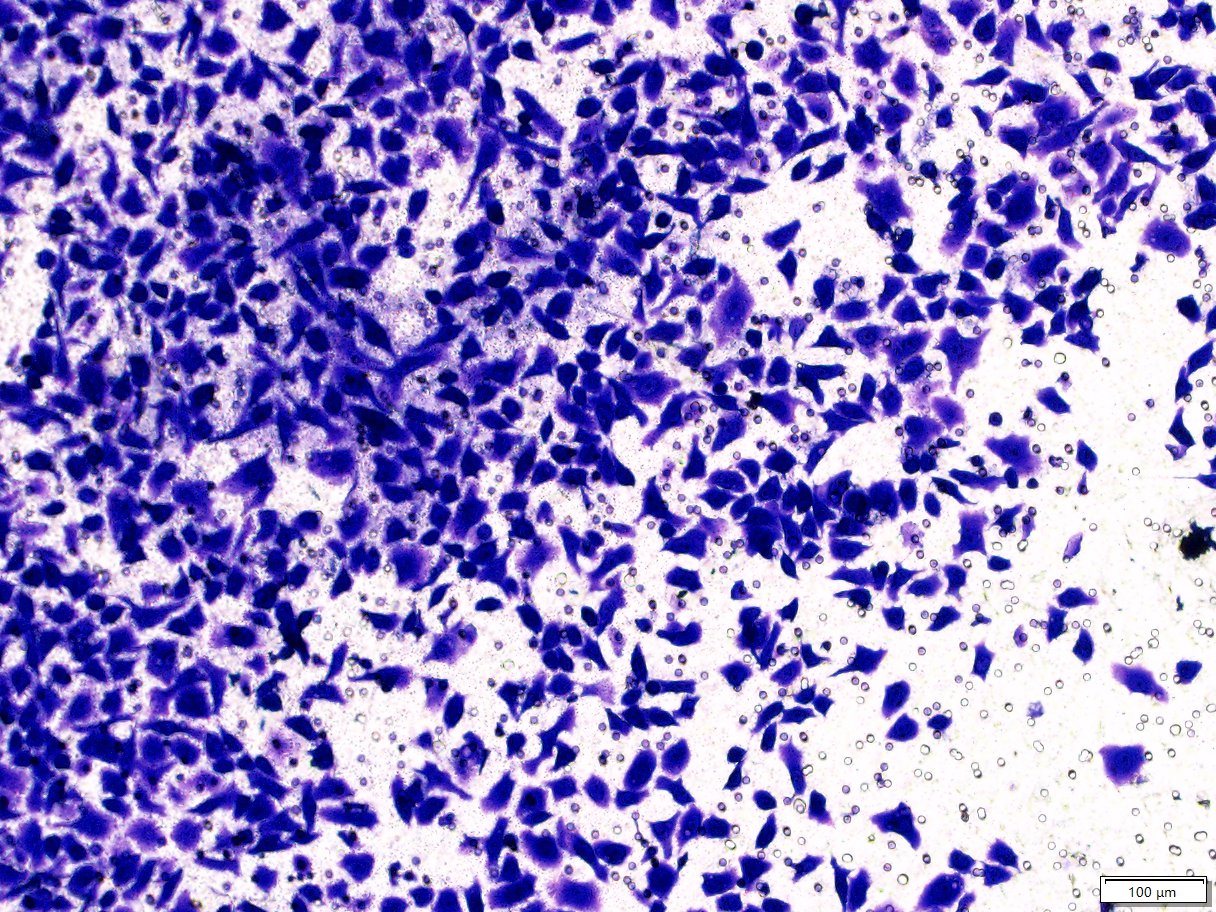

Supplement: Supplemental Information 7 [file peerj-cs-09-1651-s007.zip › Dataset 6/1-1.jpg]

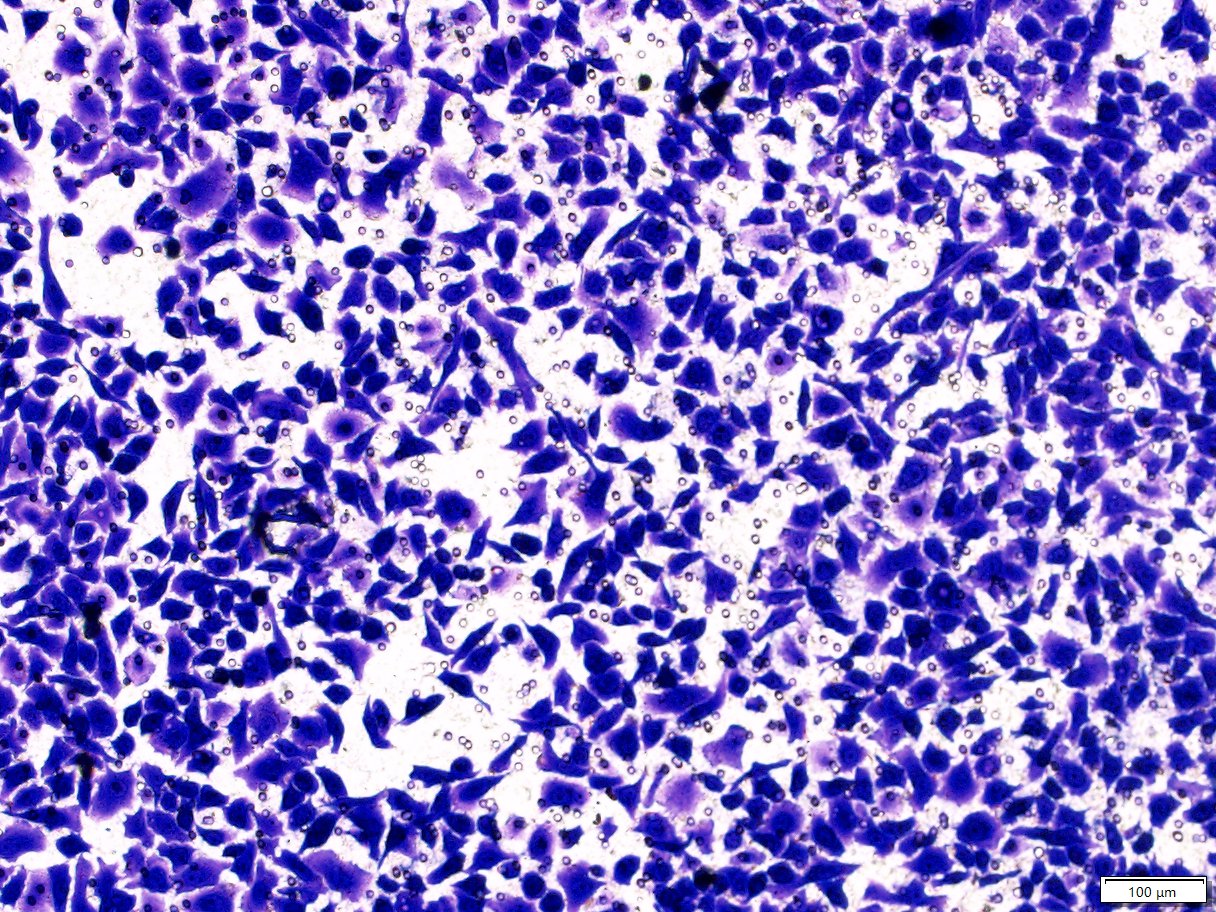

Supplement: Supplemental Information 7 [file peerj-cs-09-1651-s007.zip › Dataset 6/1-10.jpg]

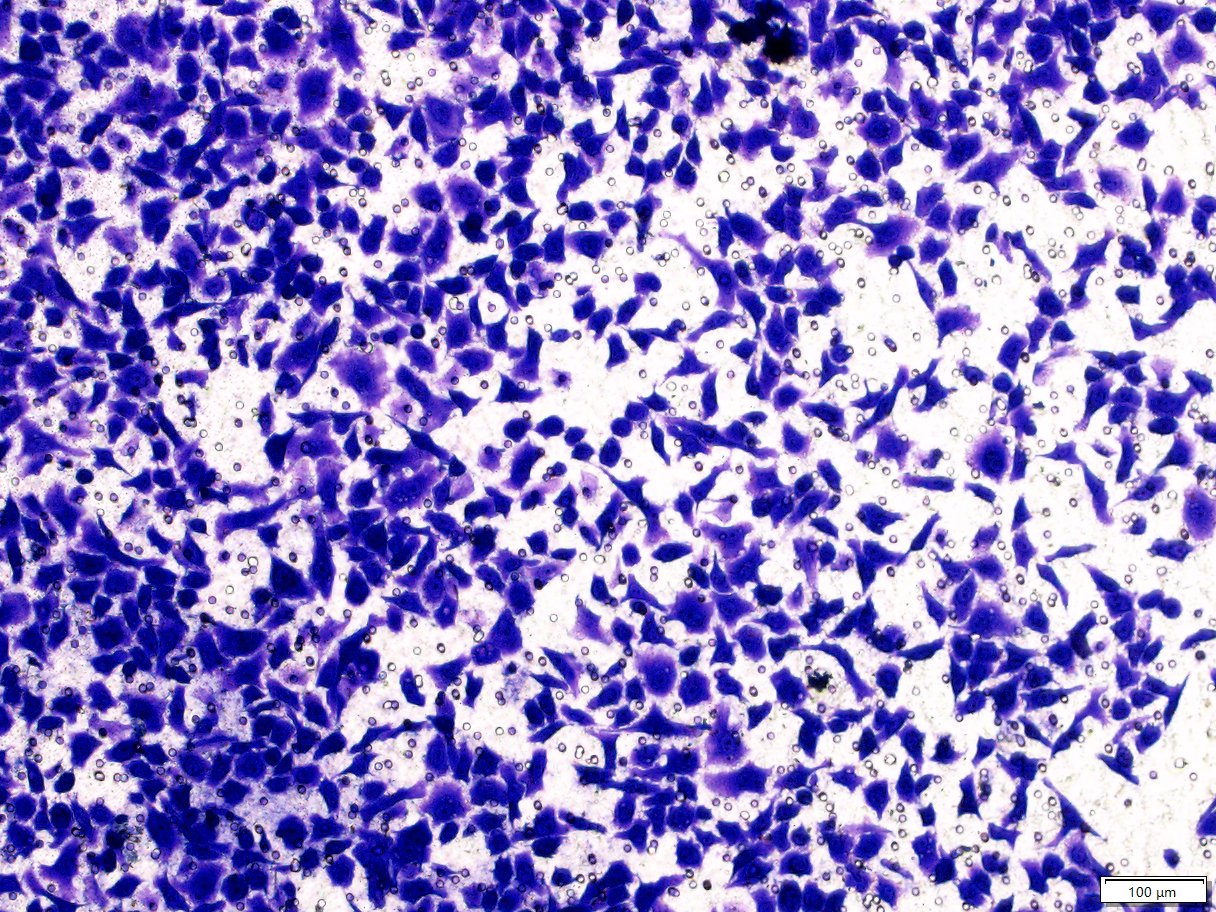

Supplement: Supplemental Information 7 [file peerj-cs-09-1651-s007.zip › Dataset 6/1-11.jpg]

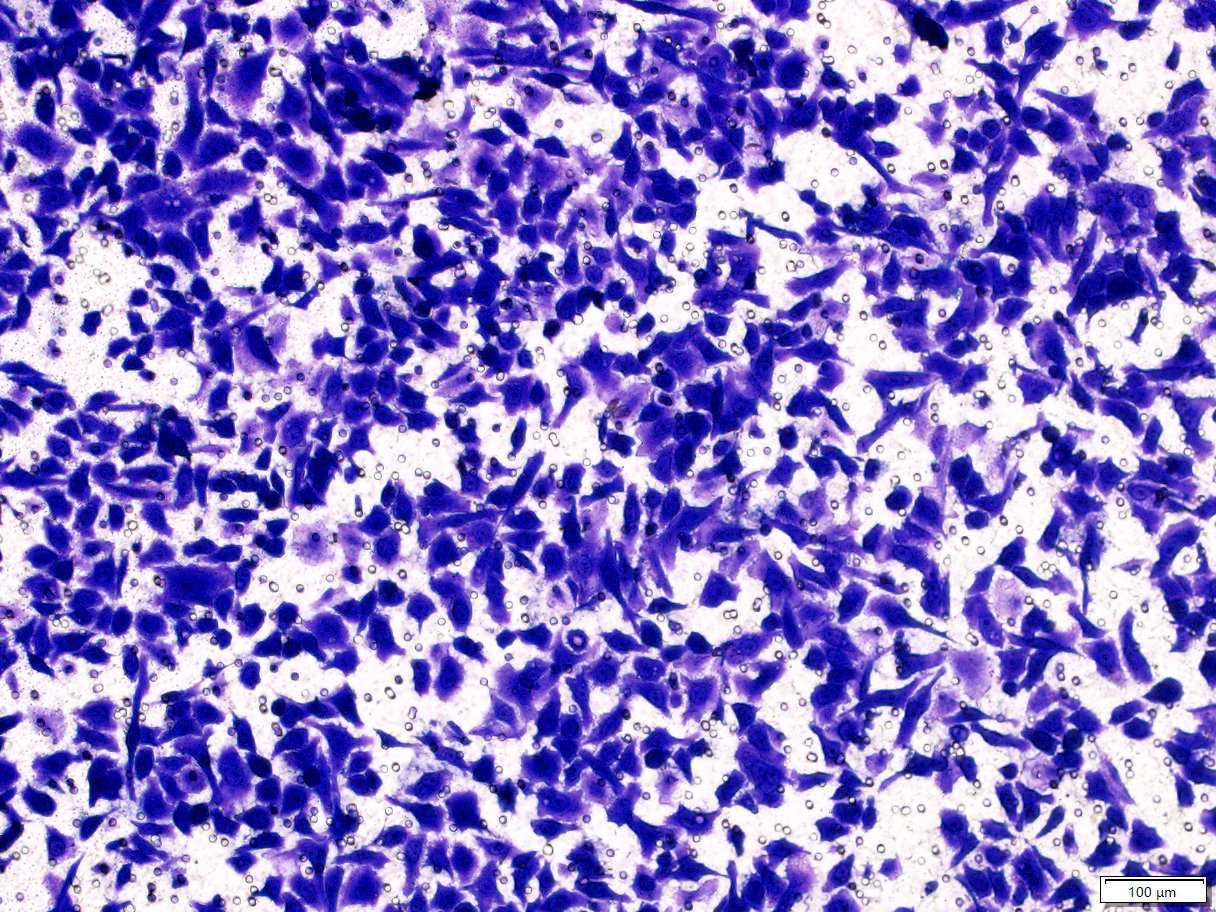

Supplement: Supplemental Information 7 [file peerj-cs-09-1651-s007.zip › Dataset 6/1-12.jpg]

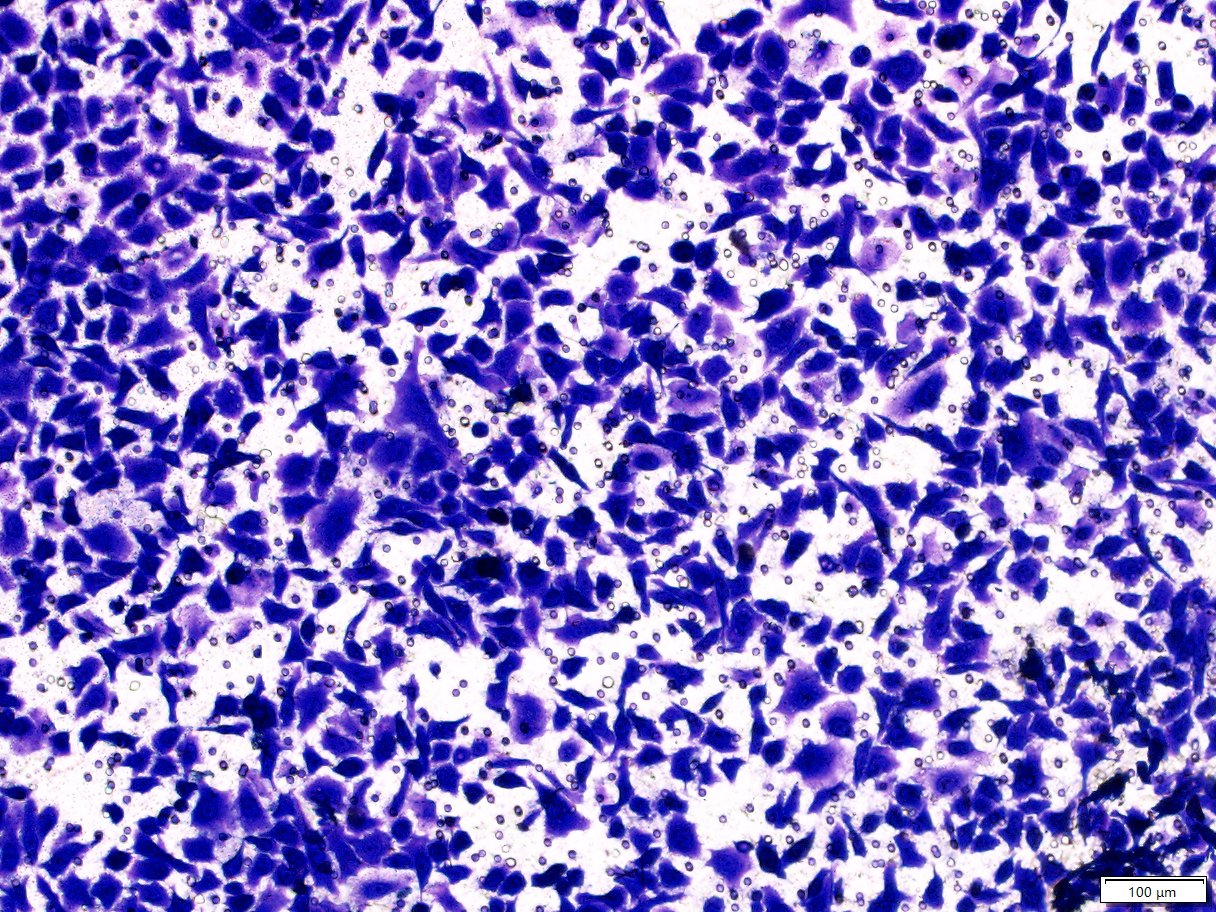

Supplement: Supplemental Information 7 [file peerj-cs-09-1651-s007.zip › Dataset 6/1-13.jpg]

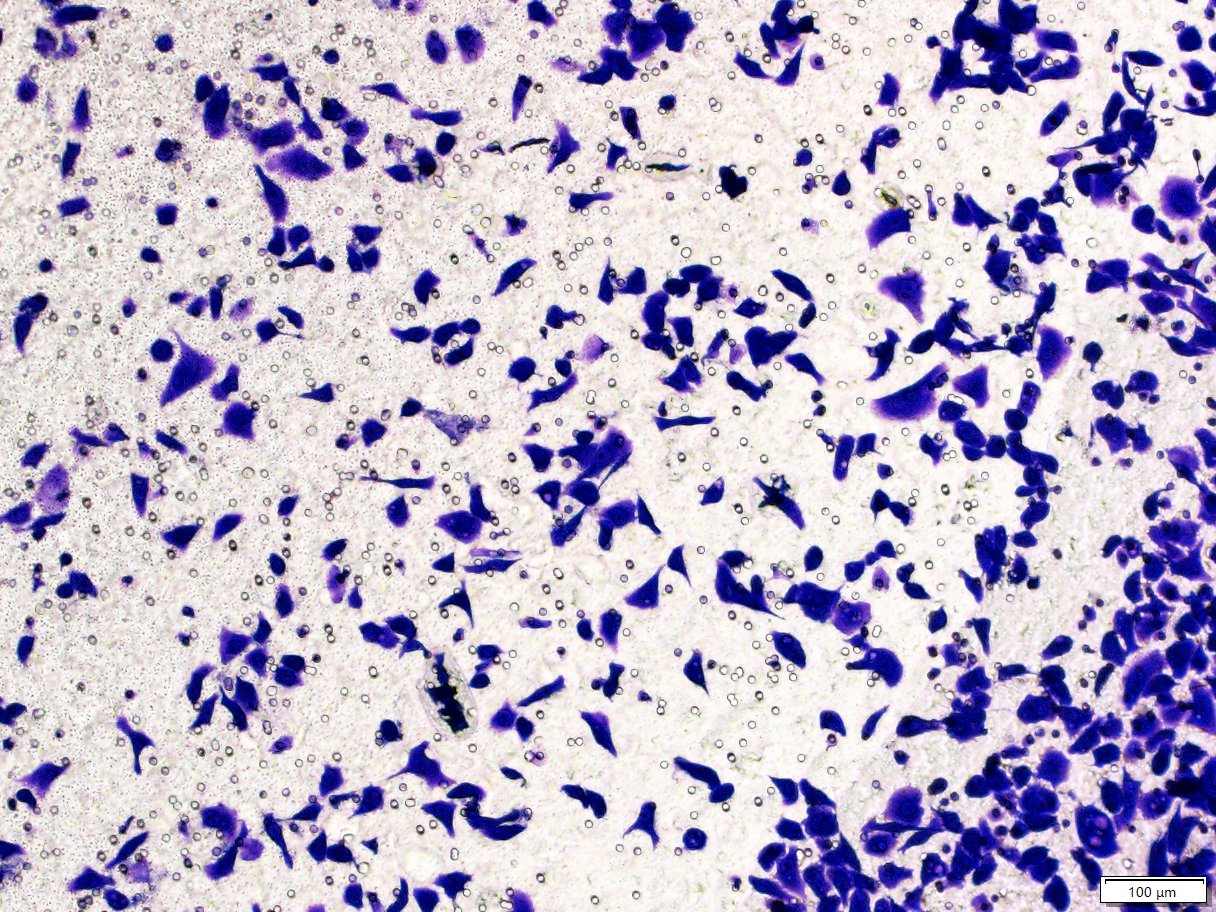

Supplement: Supplemental Information 7 [file peerj-cs-09-1651-s007.zip › Dataset 6/1-2.jpg]

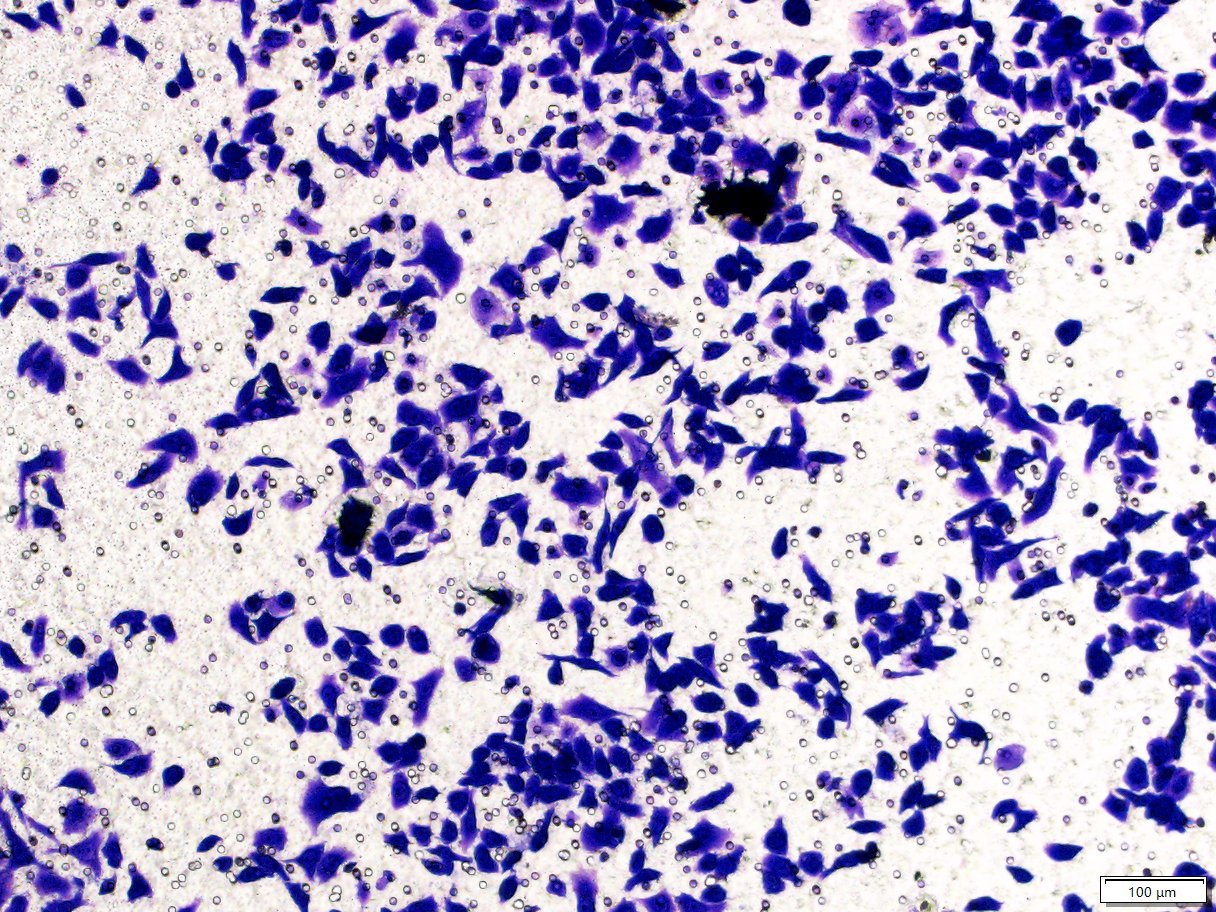

Supplement: Supplemental Information 7 [file peerj-cs-09-1651-s007.zip › Dataset 6/1-3.jpg]

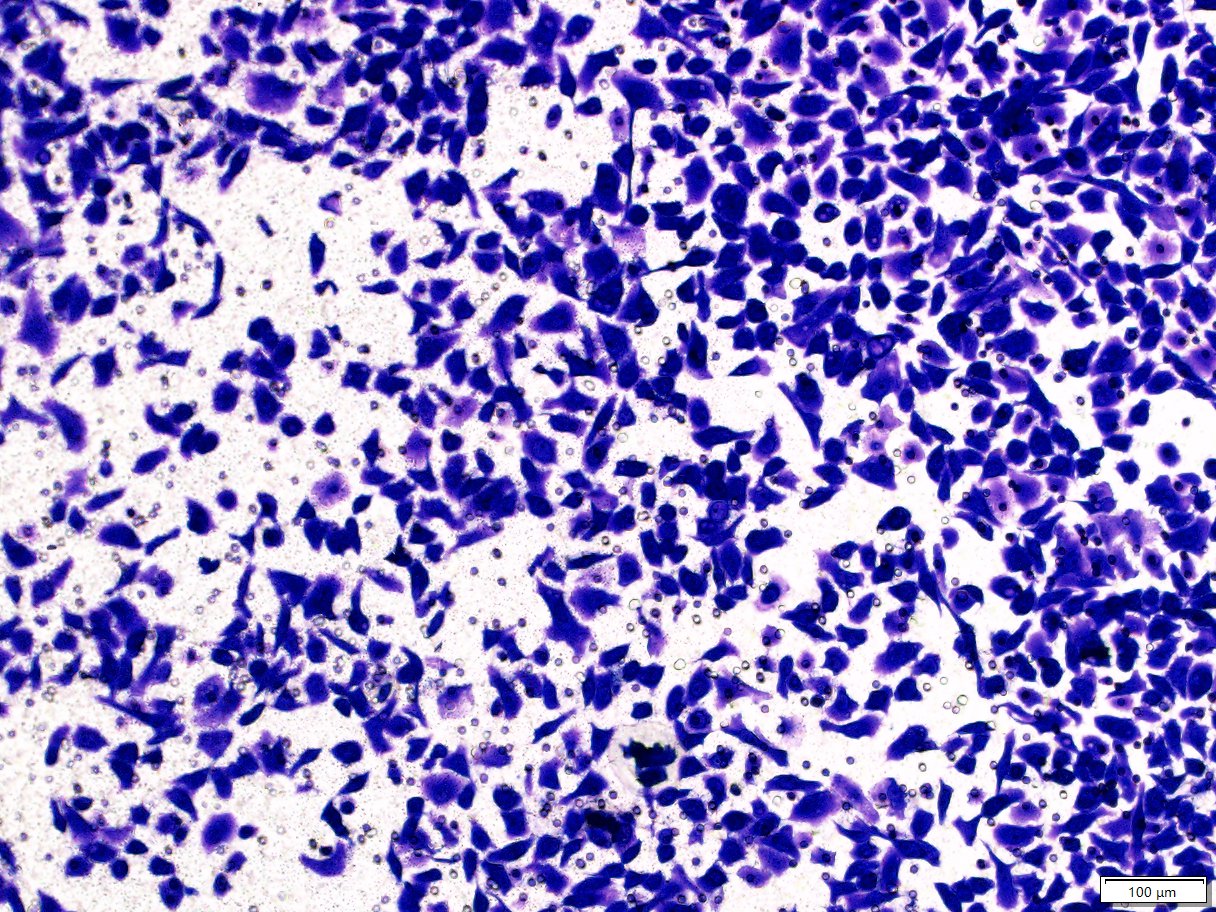

Supplement: Supplemental Information 7 [file peerj-cs-09-1651-s007.zip › Dataset 6/1-4.jpg]

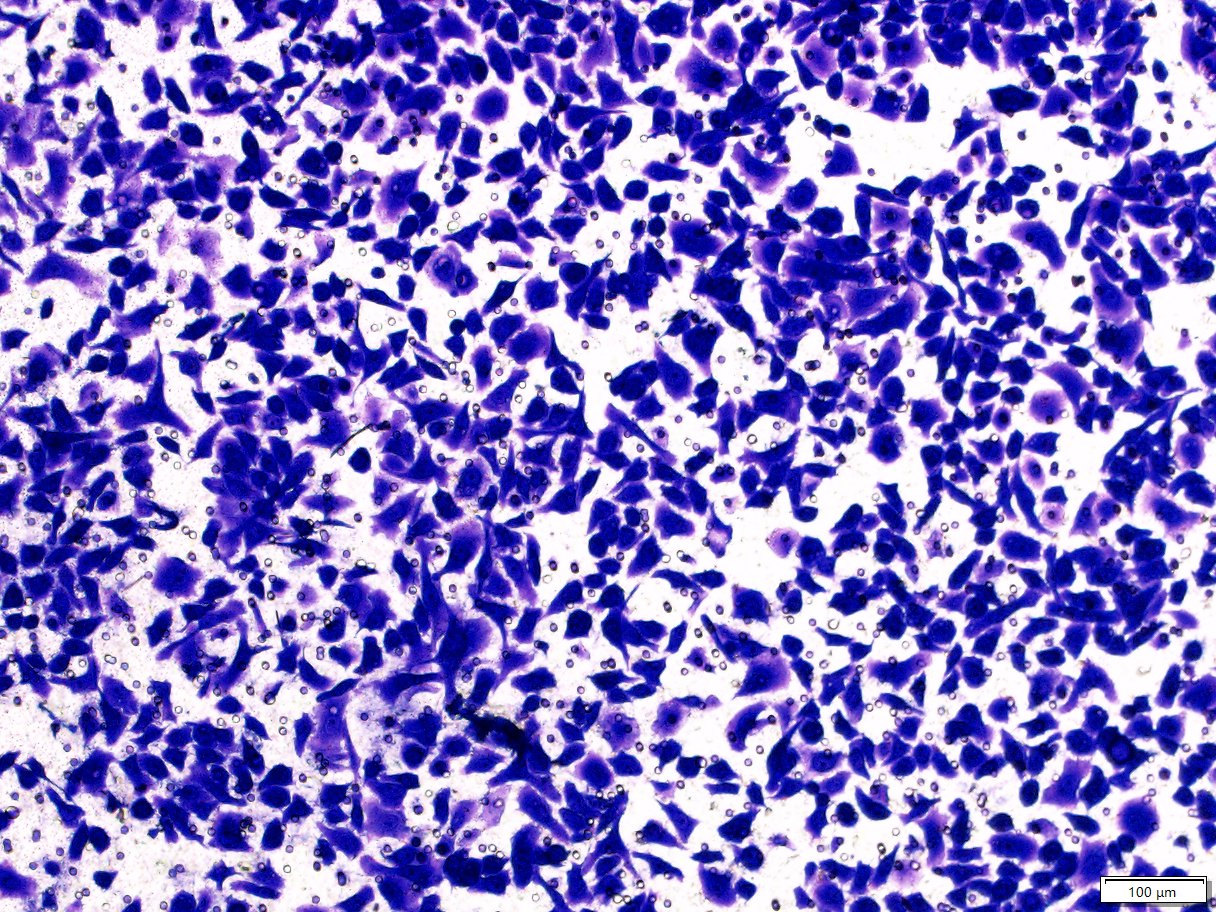

Supplement: Supplemental Information 7 [file peerj-cs-09-1651-s007.zip › Dataset 6/1-5.jpg]

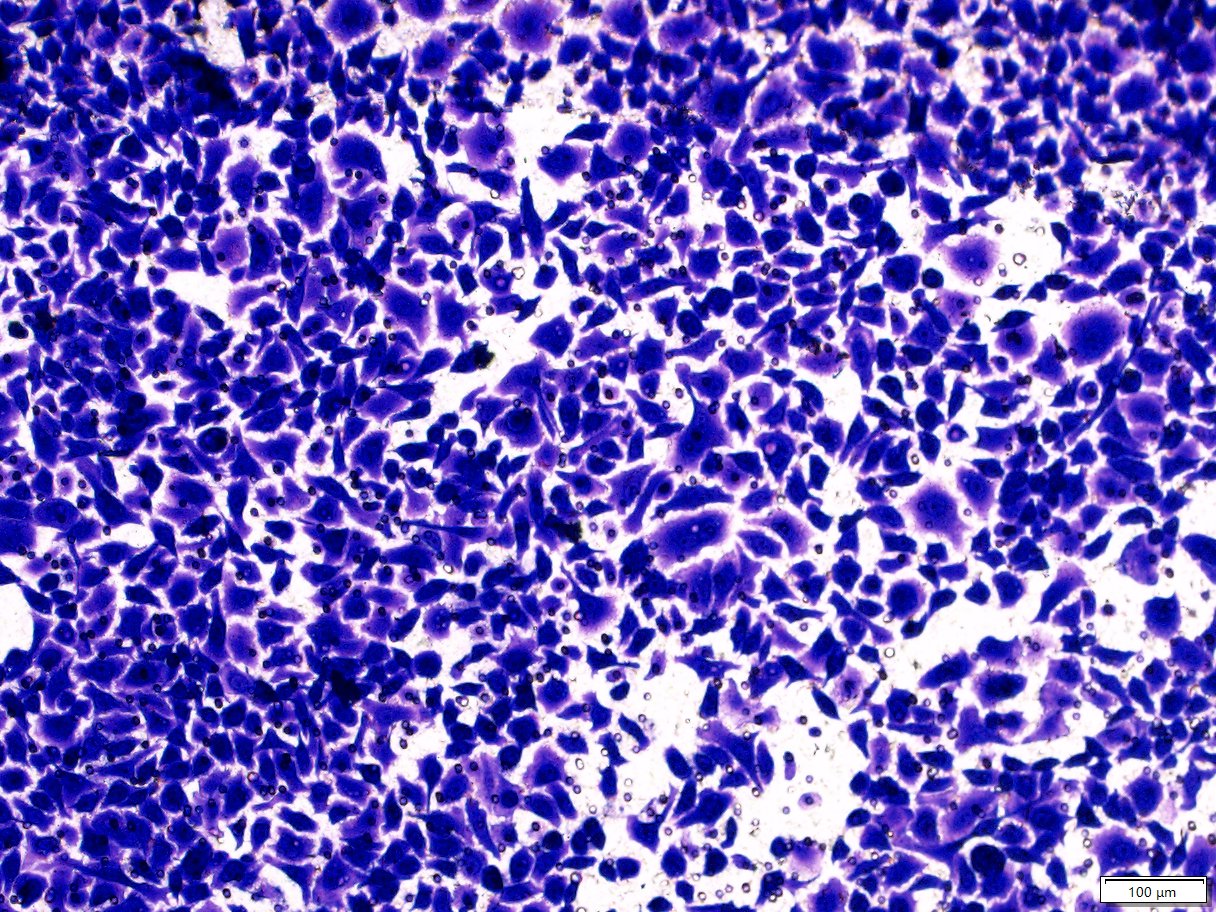

Supplement: Supplemental Information 7 [file peerj-cs-09-1651-s007.zip › Dataset 6/1-6.jpg]

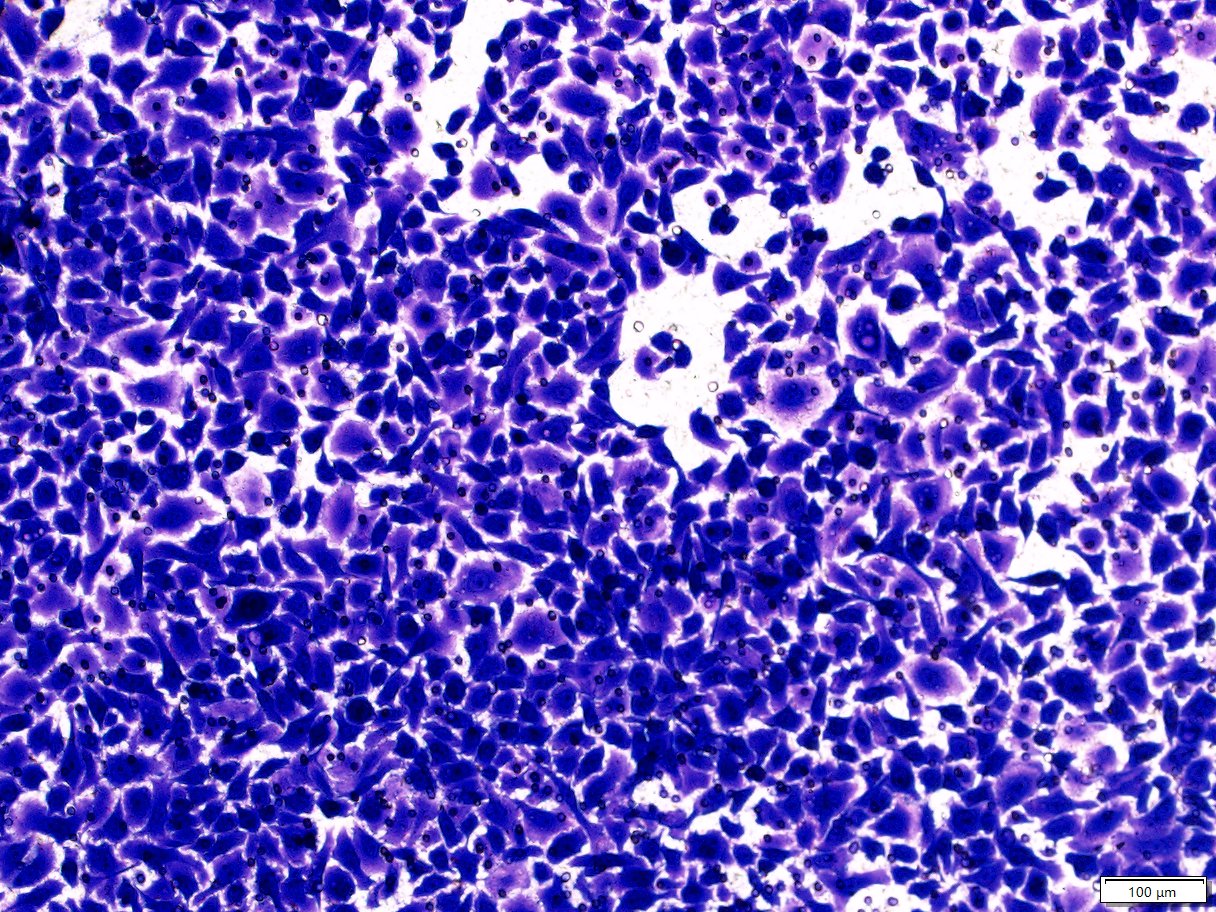

Supplement: Supplemental Information 7 [file peerj-cs-09-1651-s007.zip › Dataset 6/1-7.jpg]

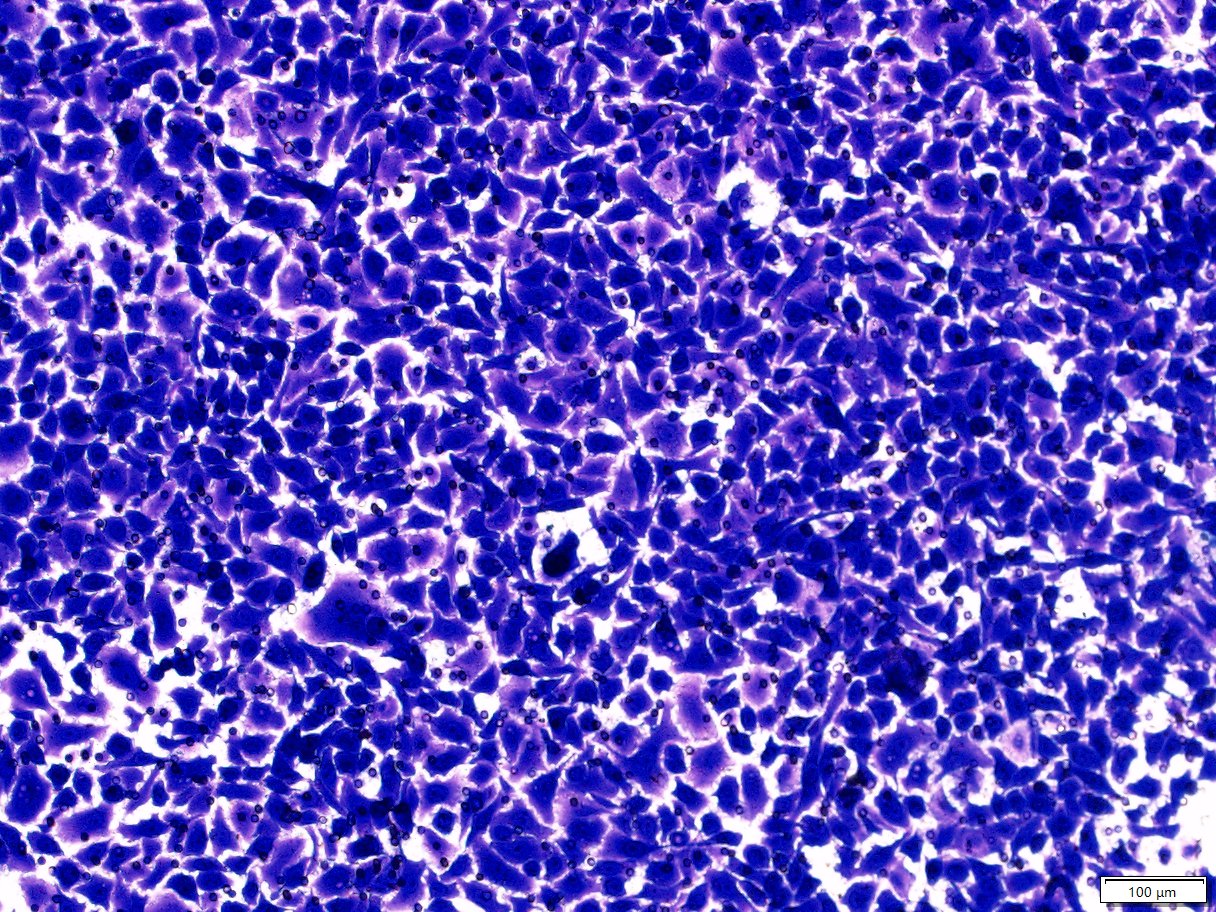

Supplement: Supplemental Information 7 [file peerj-cs-09-1651-s007.zip › Dataset 6/1-8.jpg]

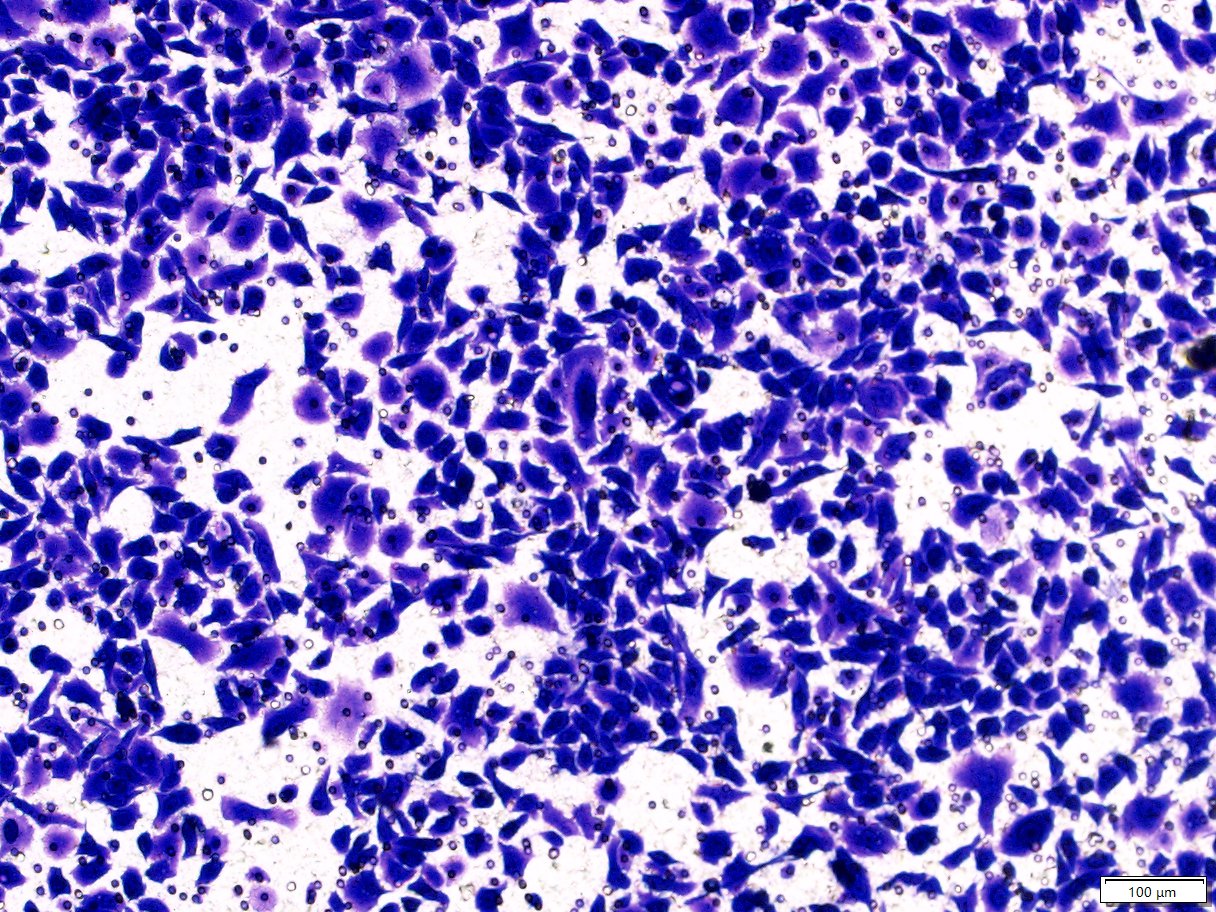

Supplement: Supplemental Information 7 [file peerj-cs-09-1651-s007.zip › Dataset 6/1-9.jpg]

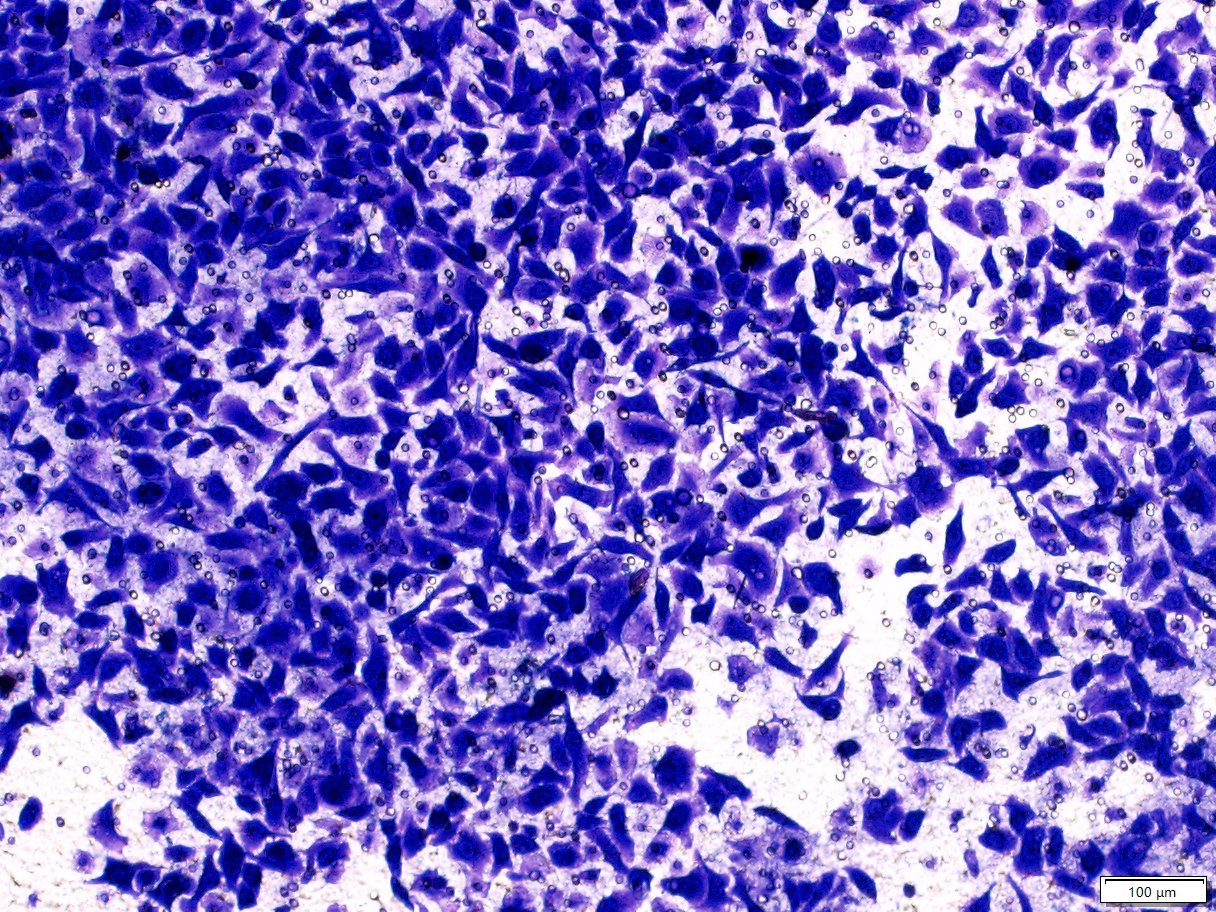

Supplement: Supplemental Information 7 [file peerj-cs-09-1651-s007.zip › Dataset 6/2+1.jpg]

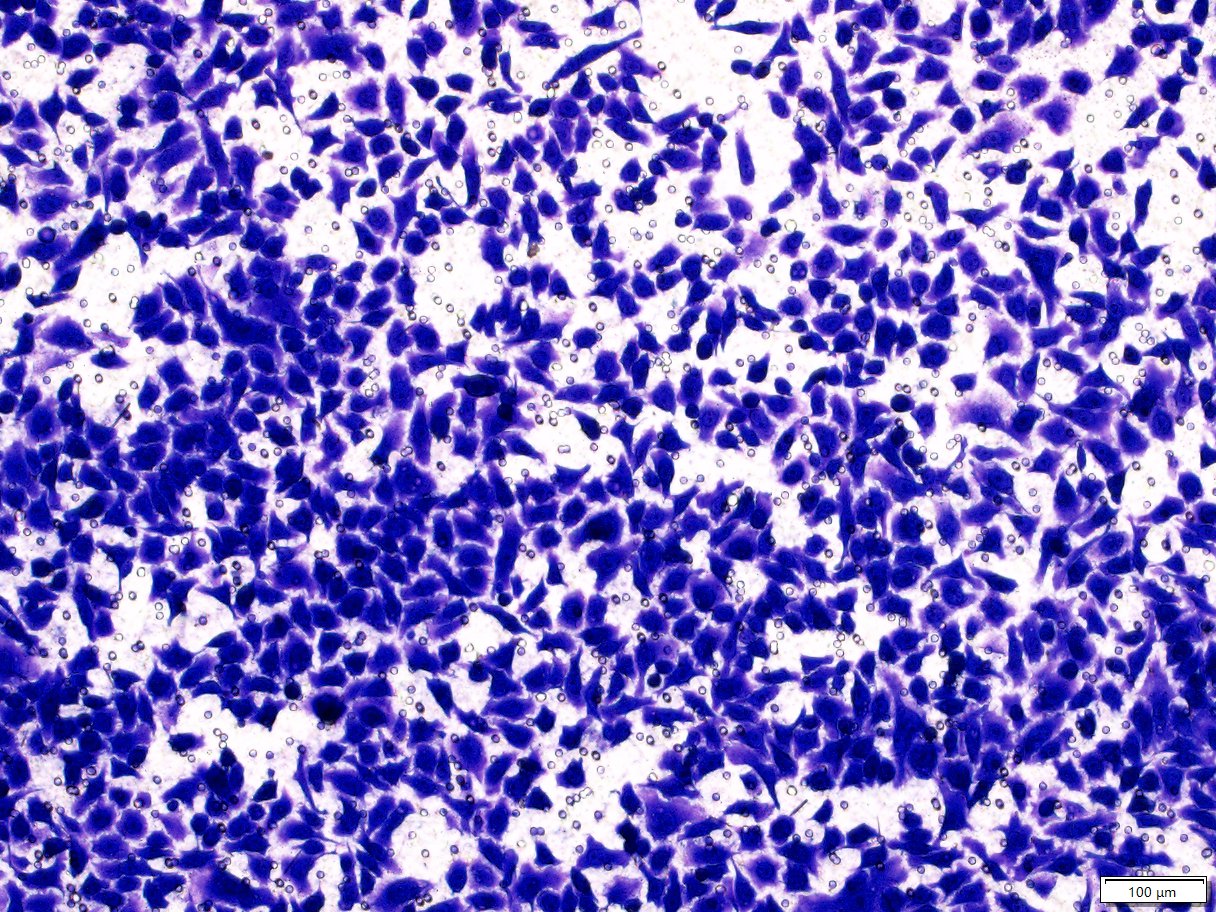

Supplement: Supplemental Information 7 [file peerj-cs-09-1651-s007.zip › Dataset 6/2+10.jpg]

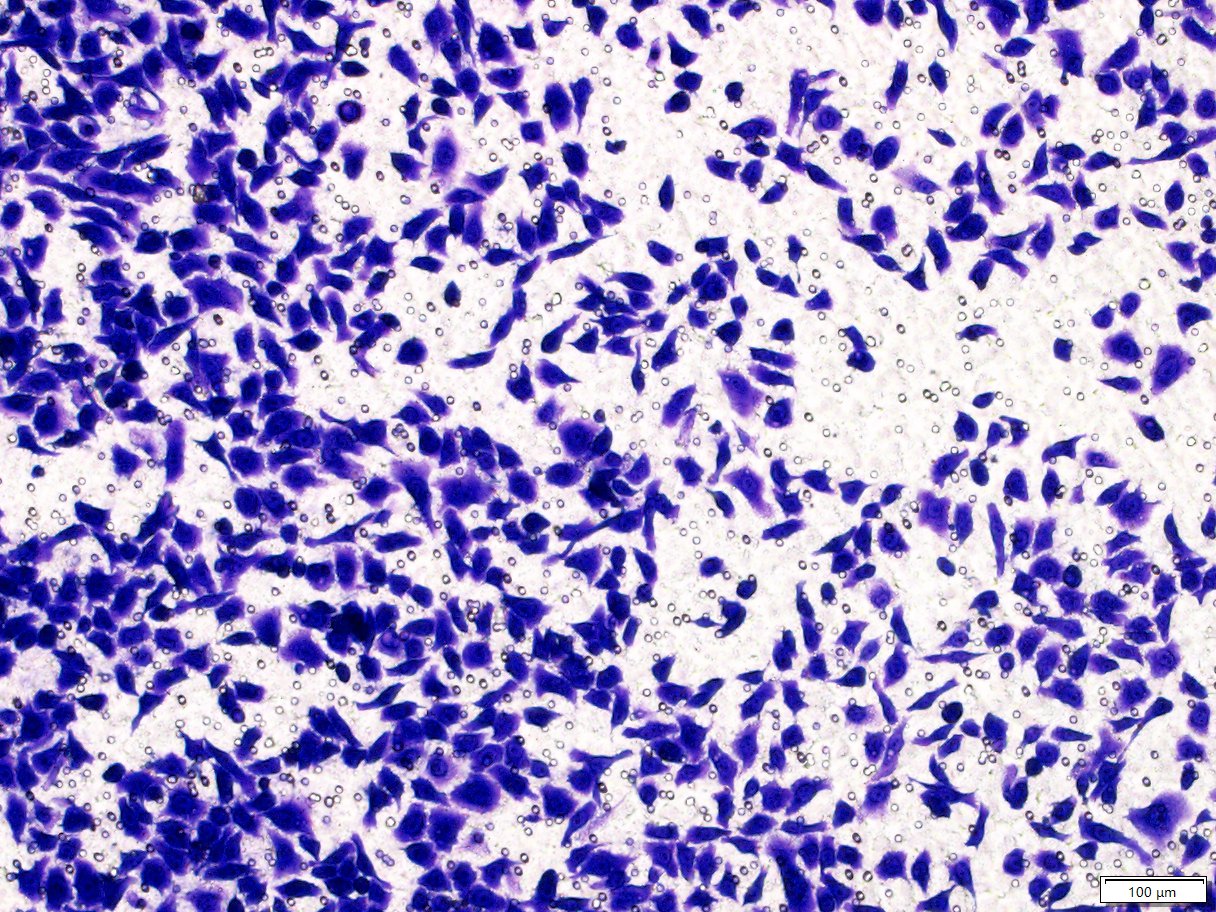

Supplement: Supplemental Information 7 [file peerj-cs-09-1651-s007.zip › Dataset 6/2+11.jpg]

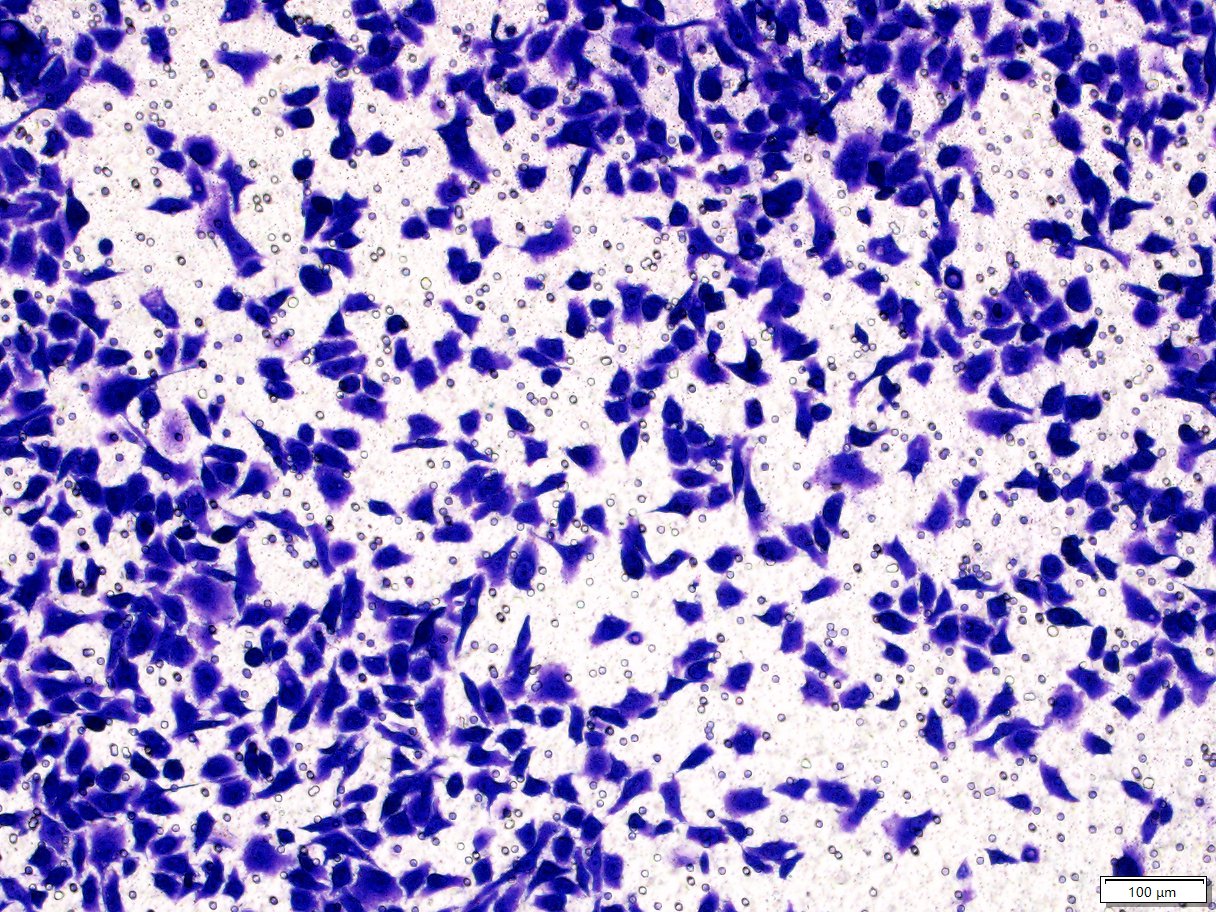

Supplement: Supplemental Information 7 [file peerj-cs-09-1651-s007.zip › Dataset 6/2+12.jpg]

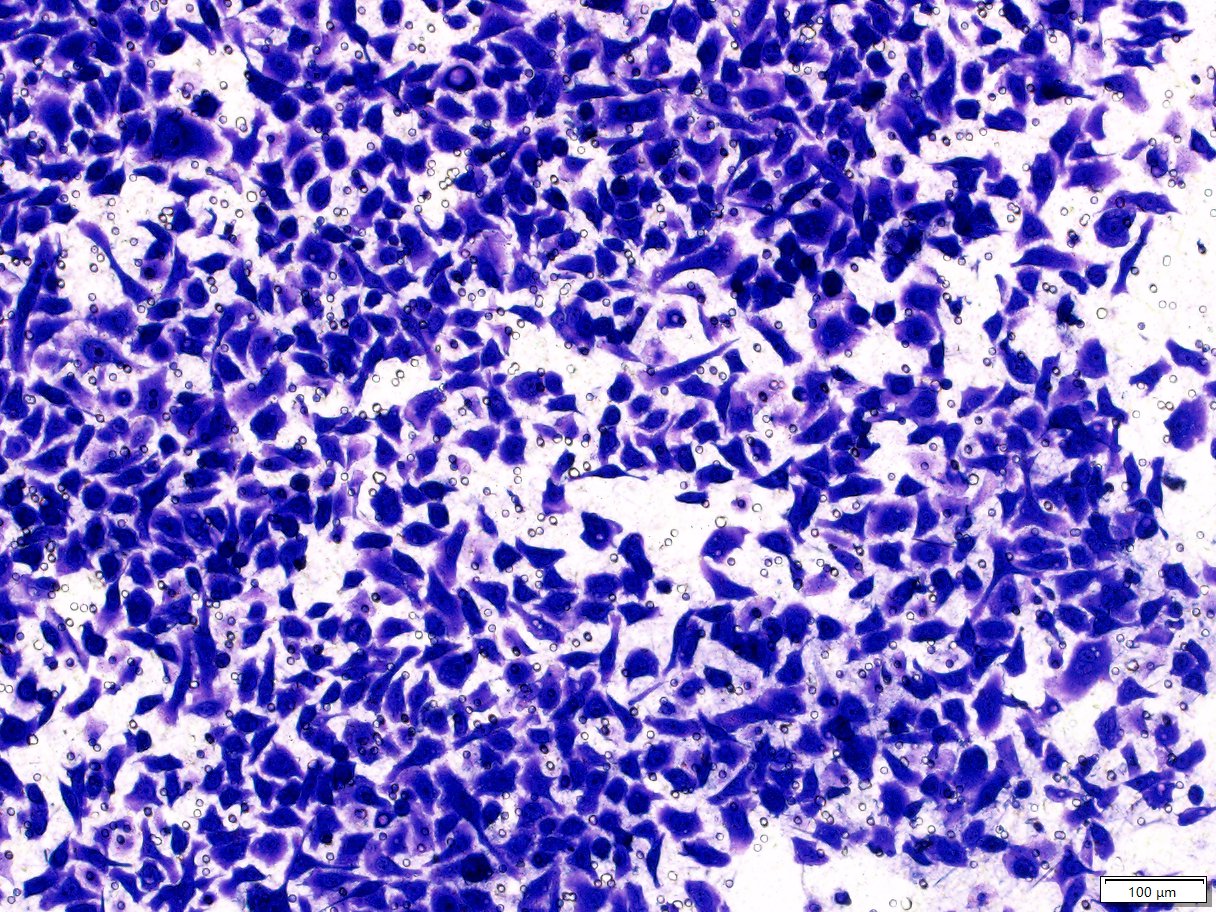

Supplement: Supplemental Information 7 [file peerj-cs-09-1651-s007.zip › Dataset 6/2+2.jpg]

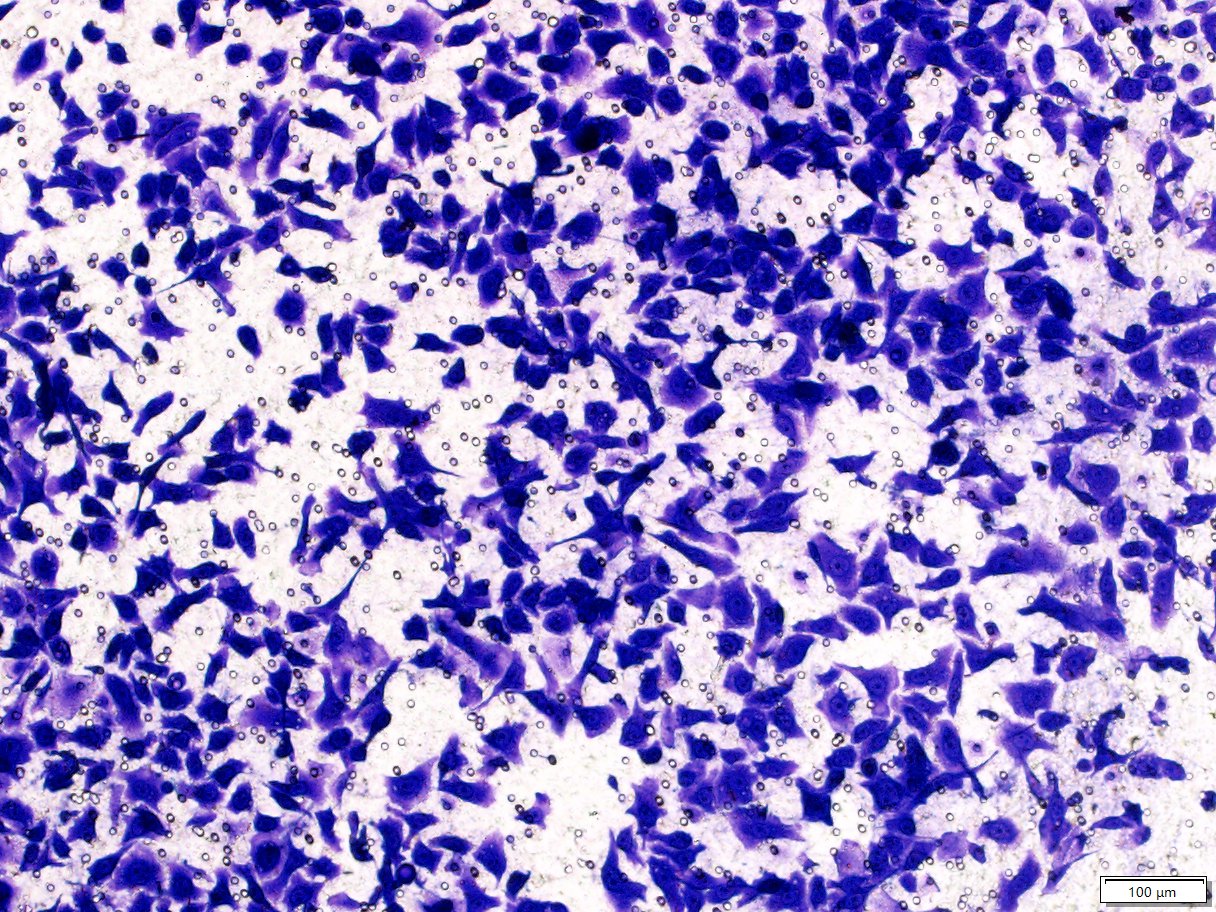

Supplement: Supplemental Information 7 [file peerj-cs-09-1651-s007.zip › Dataset 6/2+36.jpg]

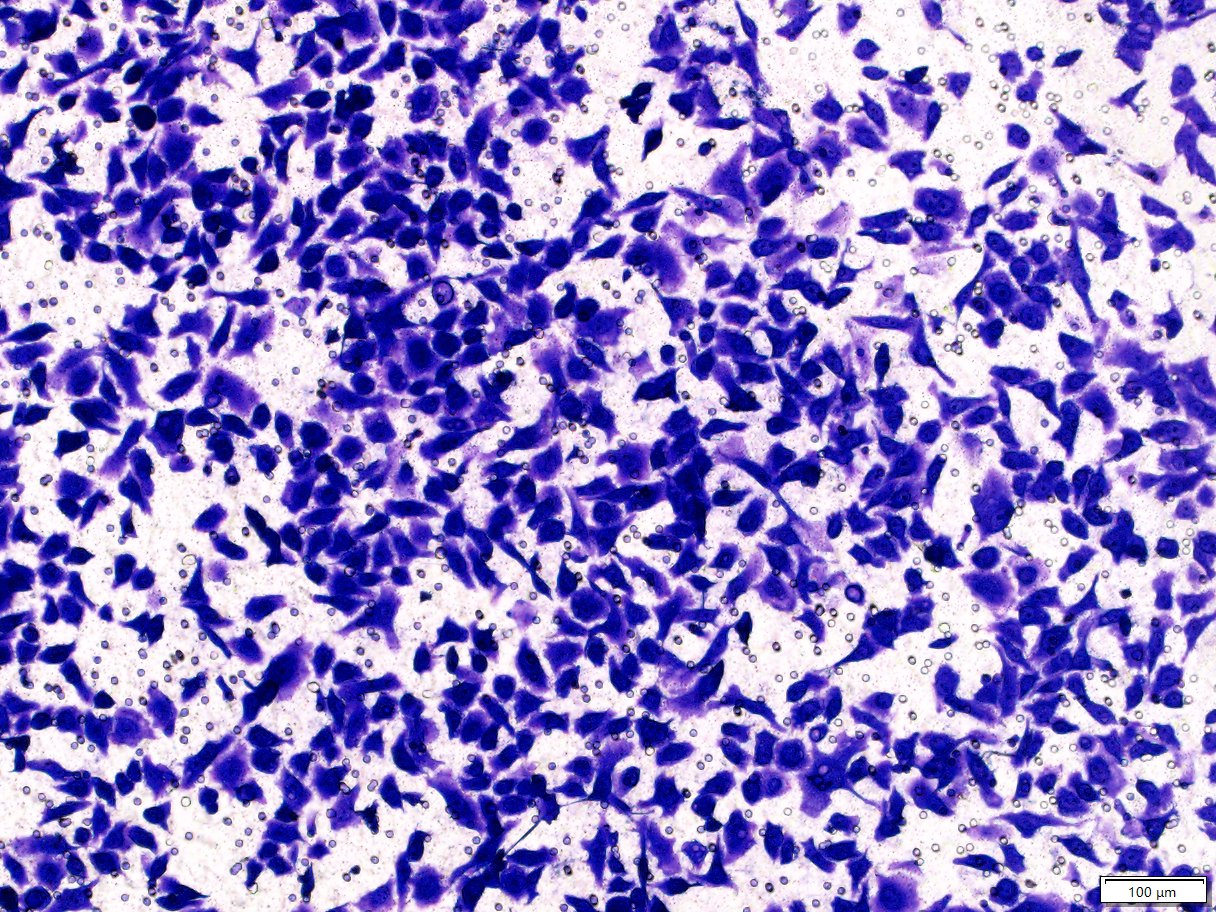

Supplement: Supplemental Information 7 [file peerj-cs-09-1651-s007.zip › Dataset 6/2+4.jpg]

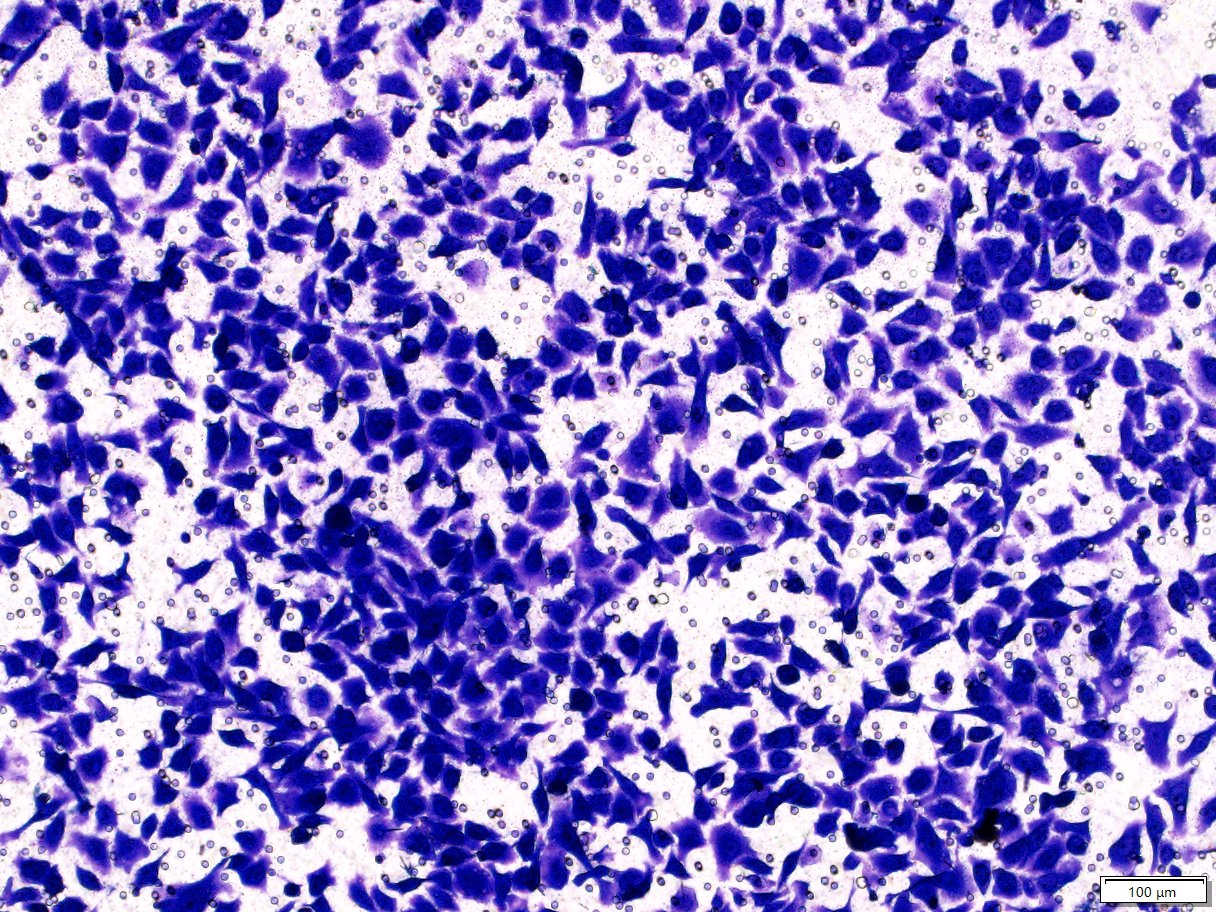

Supplement: Supplemental Information 7 [file peerj-cs-09-1651-s007.zip › Dataset 6/2+5.jpg]

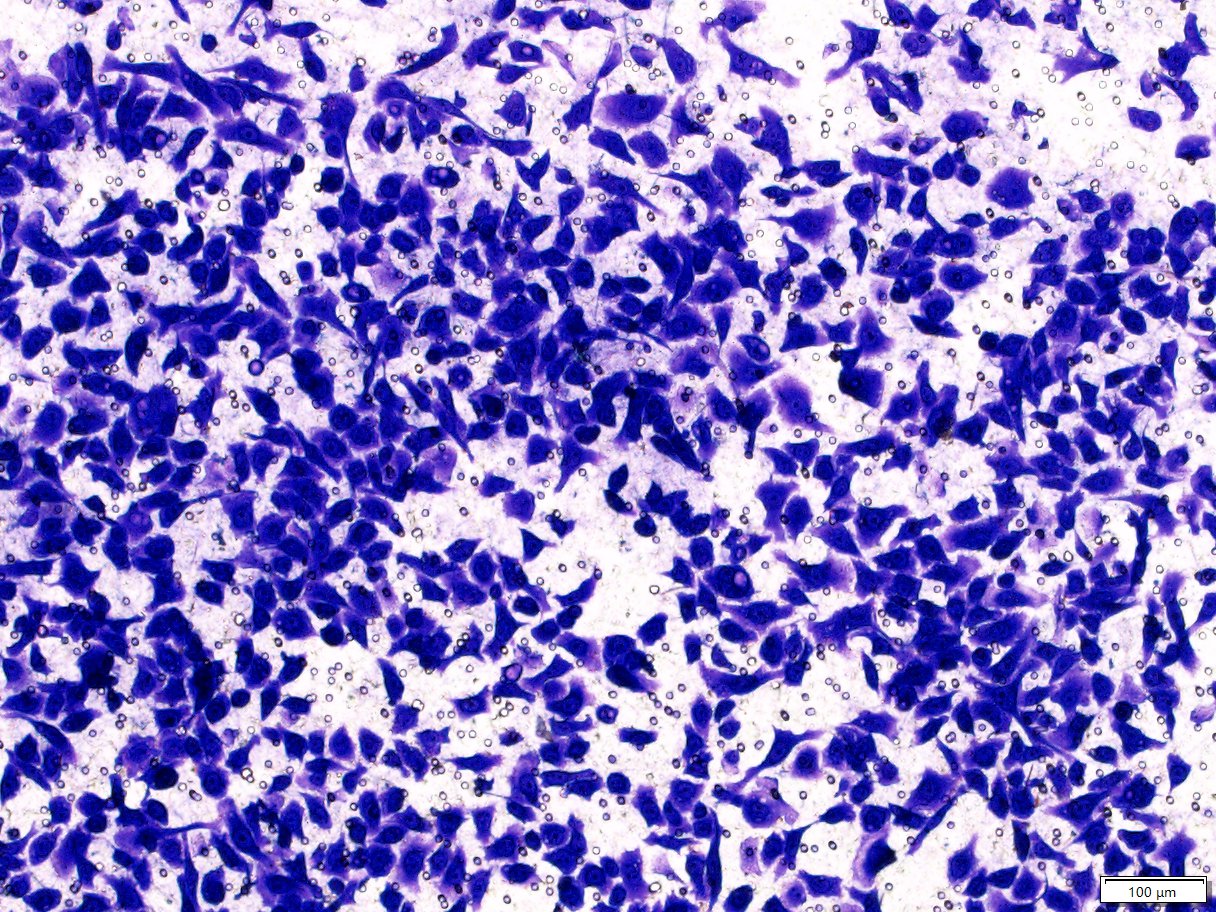

Supplement: Supplemental Information 7 [file peerj-cs-09-1651-s007.zip › Dataset 6/2+6.jpg]

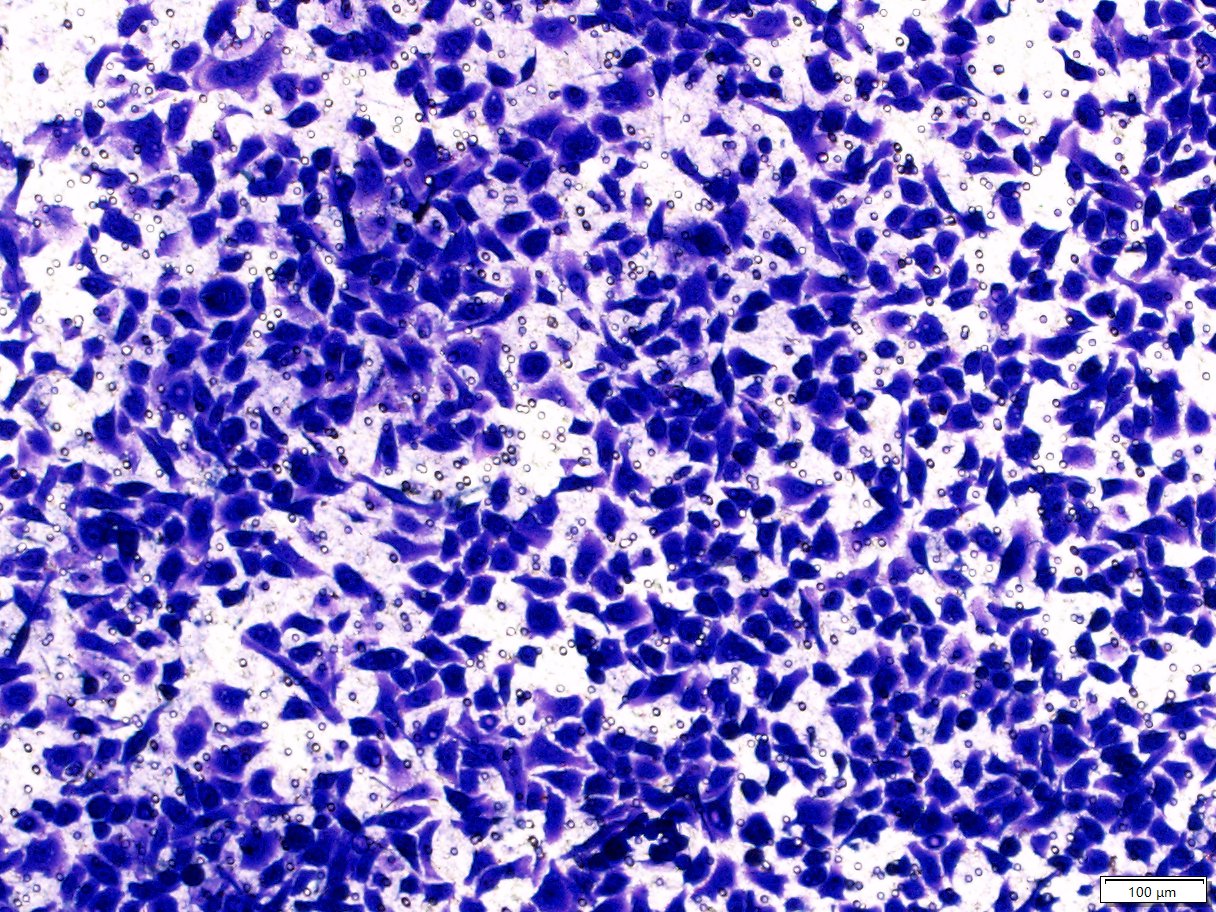

Supplement: Supplemental Information 7 [file peerj-cs-09-1651-s007.zip › Dataset 6/2+7.jpg]

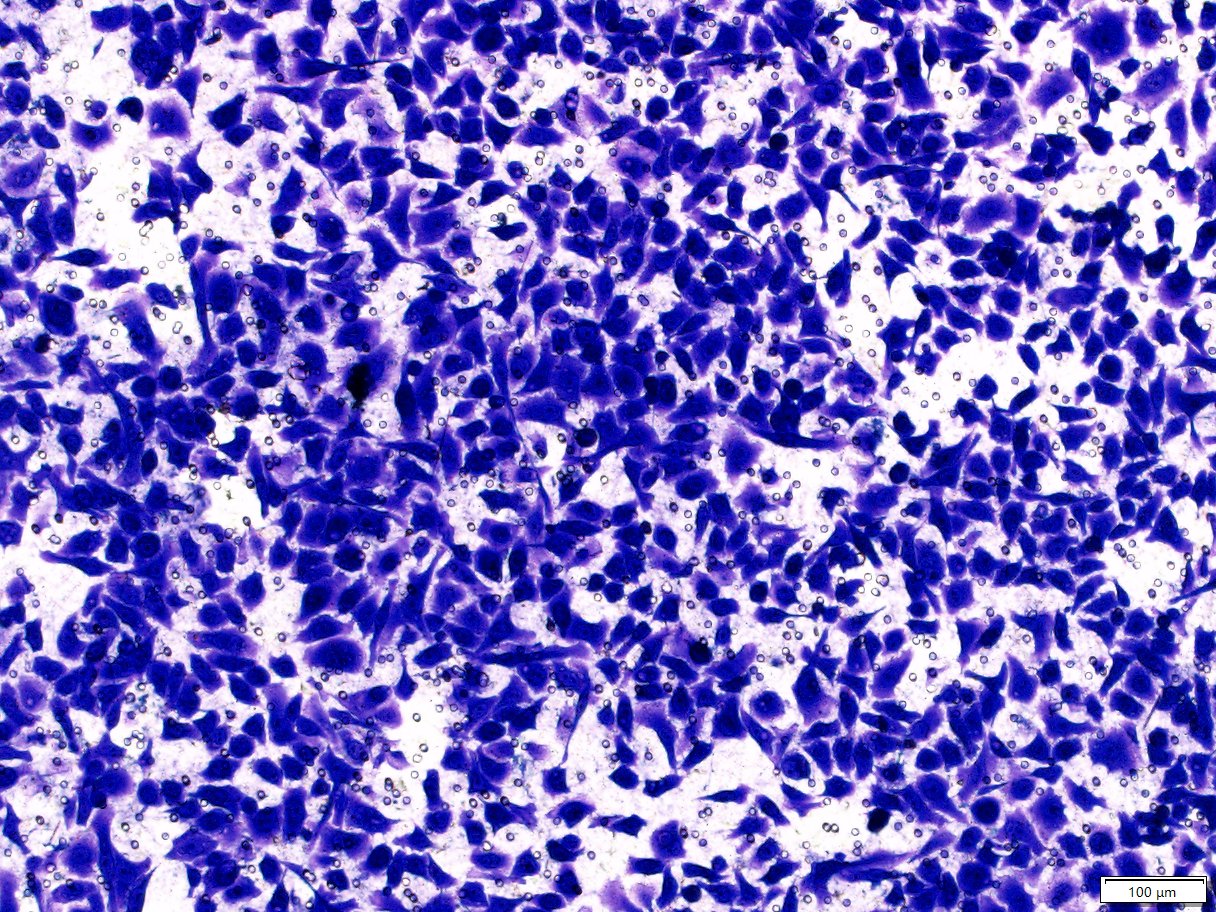

Supplement: Supplemental Information 7 [file peerj-cs-09-1651-s007.zip › Dataset 6/2+8.jpg]

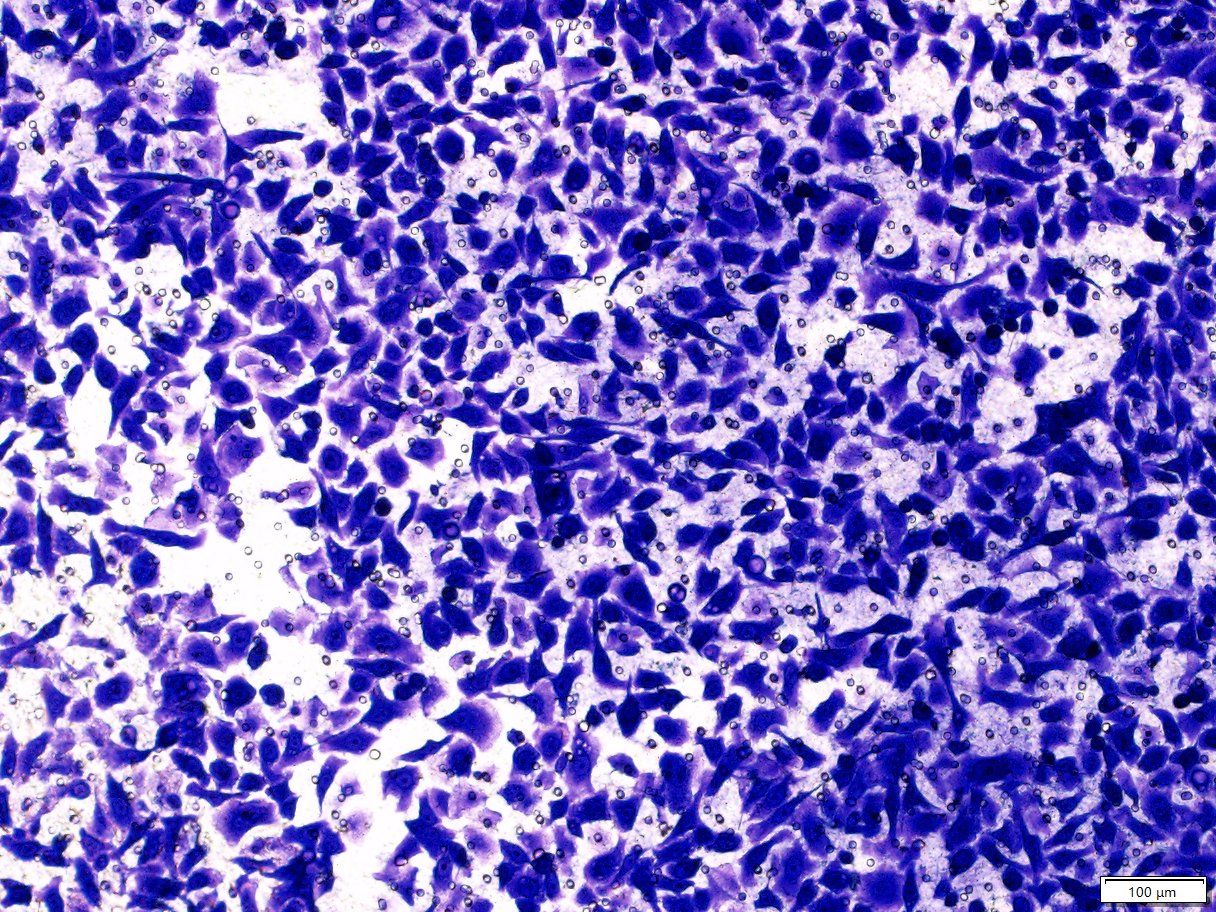

Supplement: Supplemental Information 7 [file peerj-cs-09-1651-s007.zip › Dataset 6/2+9.jpg]

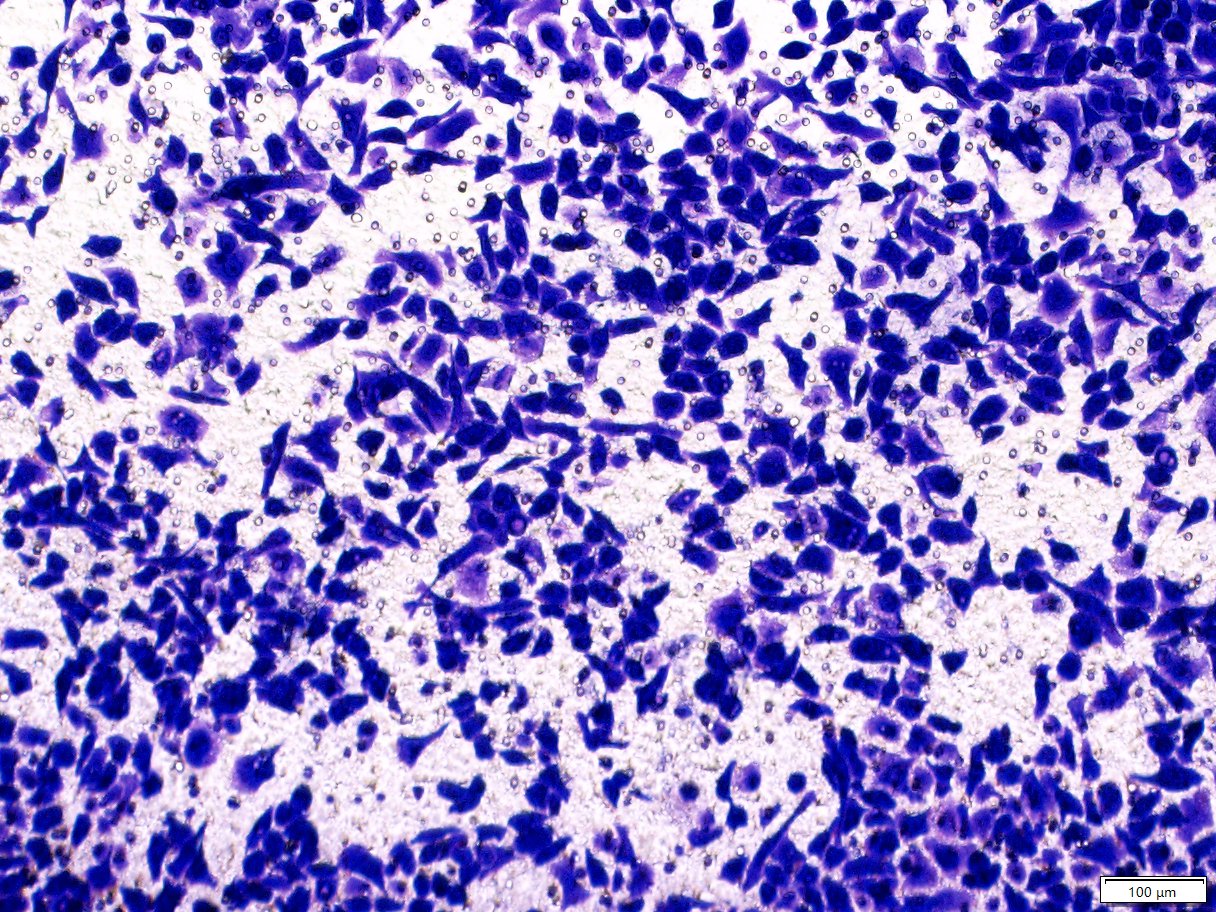

Supplement: Supplemental Information 7 [file peerj-cs-09-1651-s007.zip › Dataset 6/2-1.jpg]

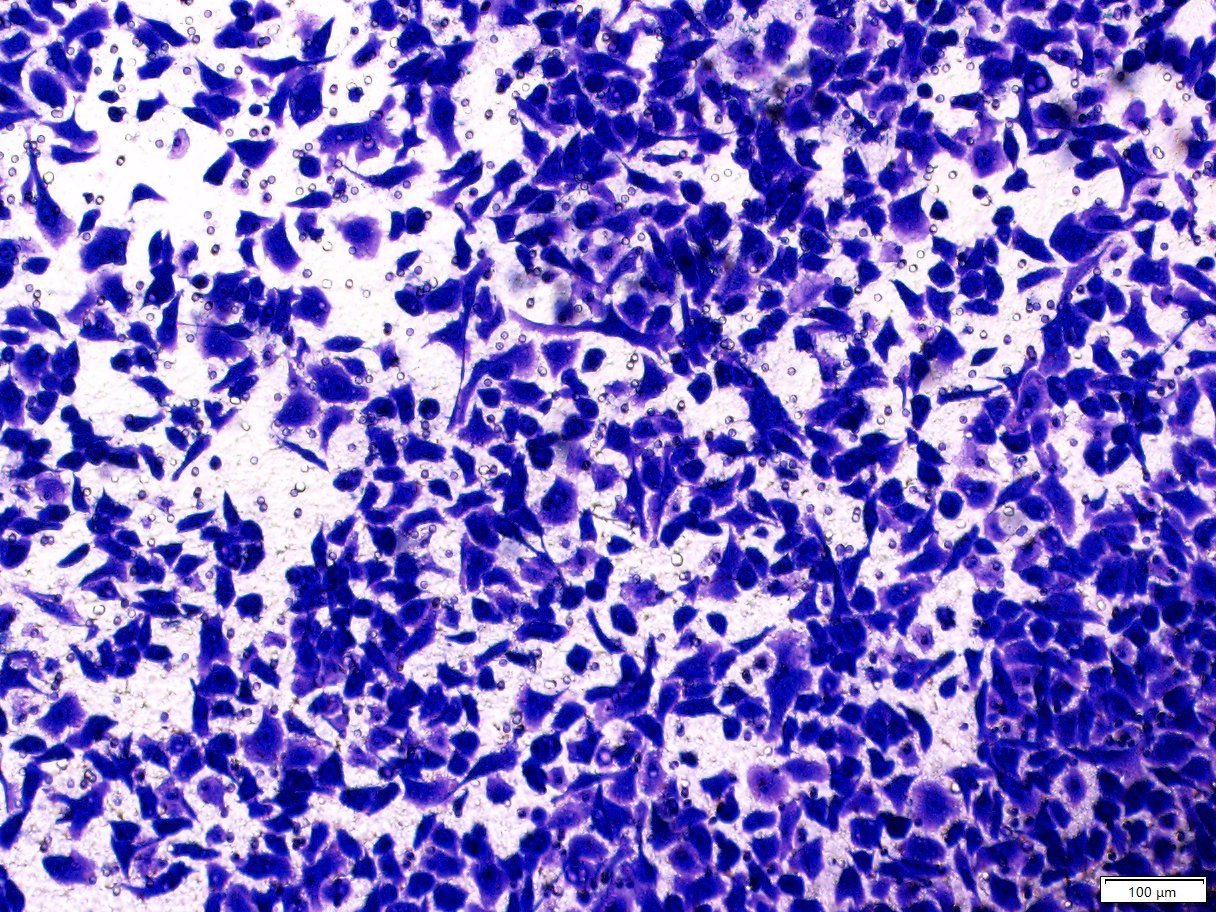

Supplement: Supplemental Information 7 [file peerj-cs-09-1651-s007.zip › Dataset 6/2-2.jpg]

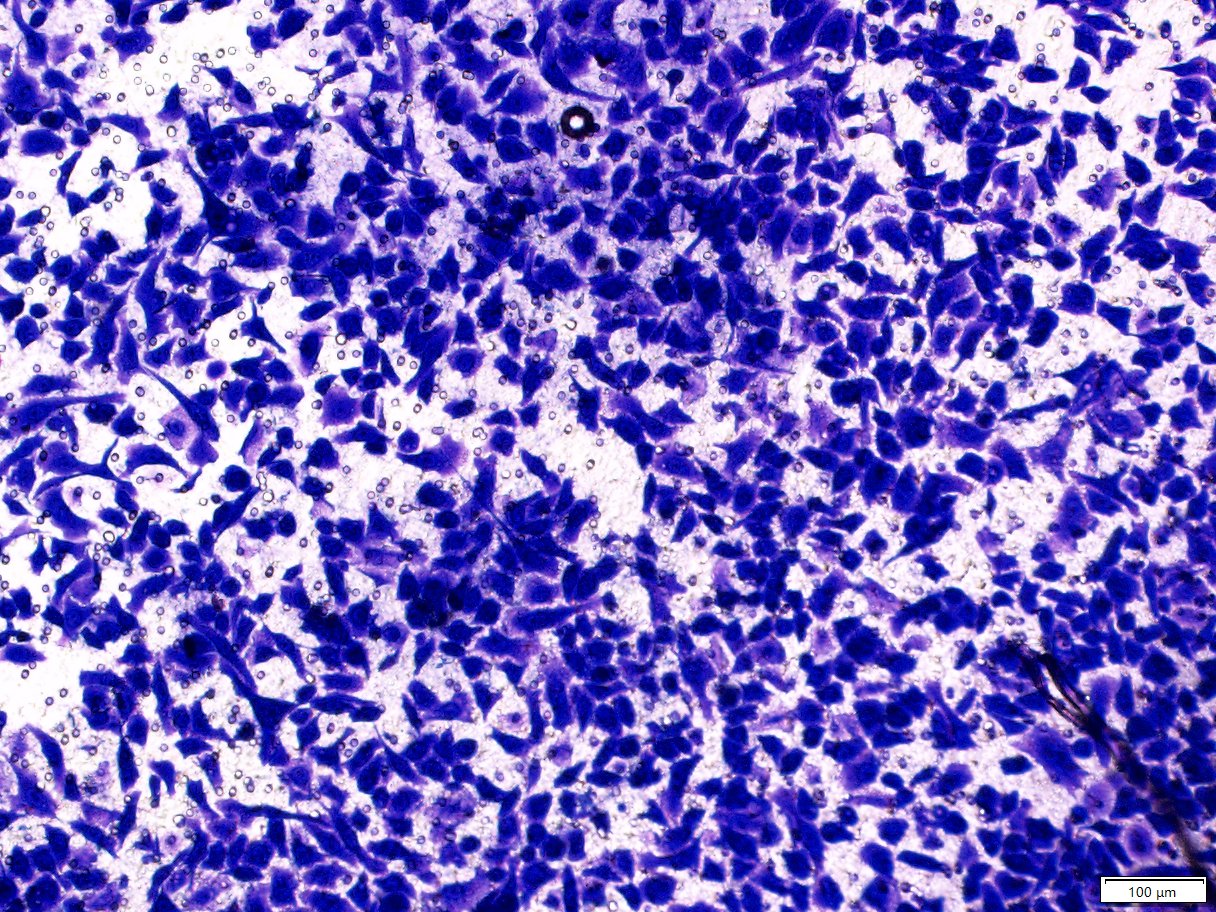

Supplement: Supplemental Information 7 [file peerj-cs-09-1651-s007.zip › Dataset 6/2-3.jpg]

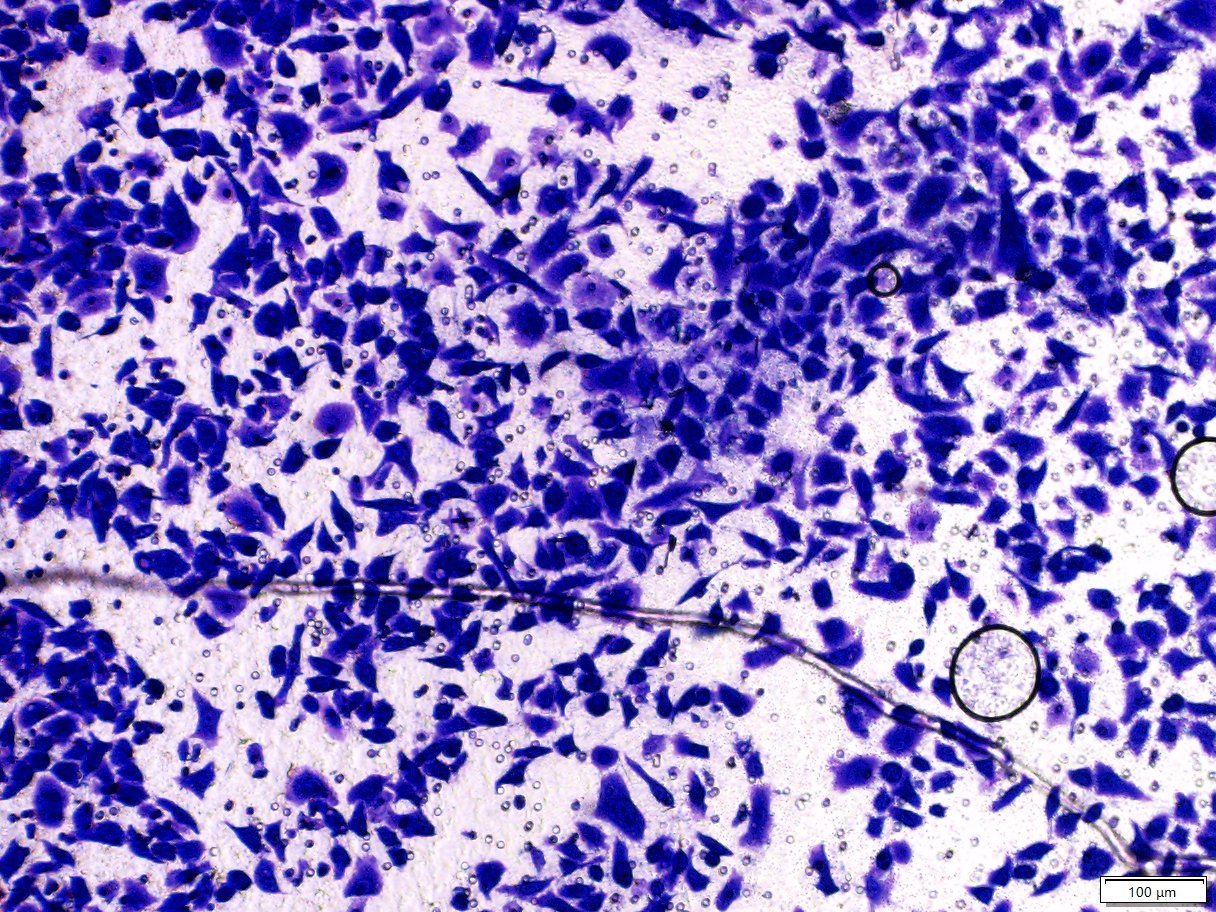

Supplement: Supplemental Information 8 [file peerj-cs-09-1651-s008.zip › Dataset 7/2-10.jpg]

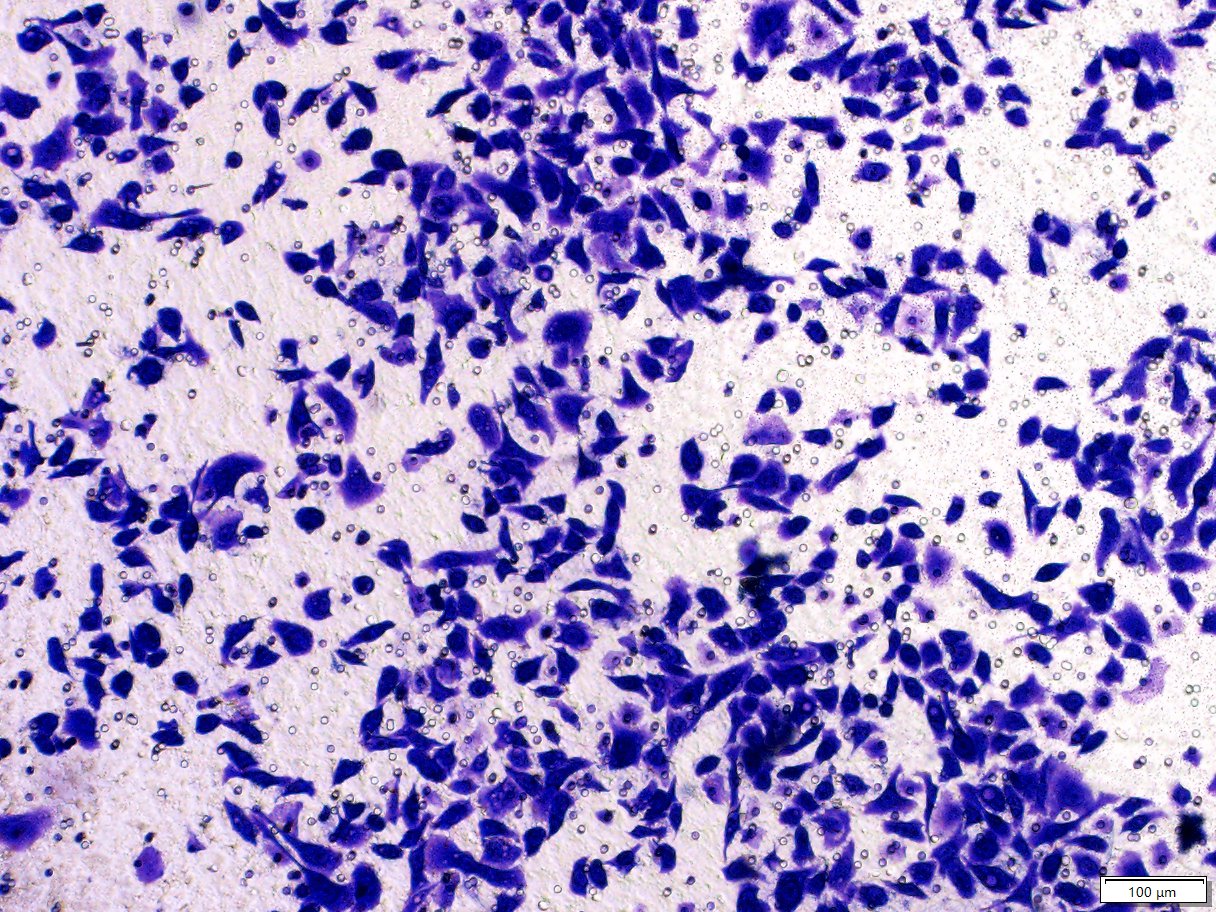

Supplement: Supplemental Information 8 [file peerj-cs-09-1651-s008.zip › Dataset 7/2-11.jpg]

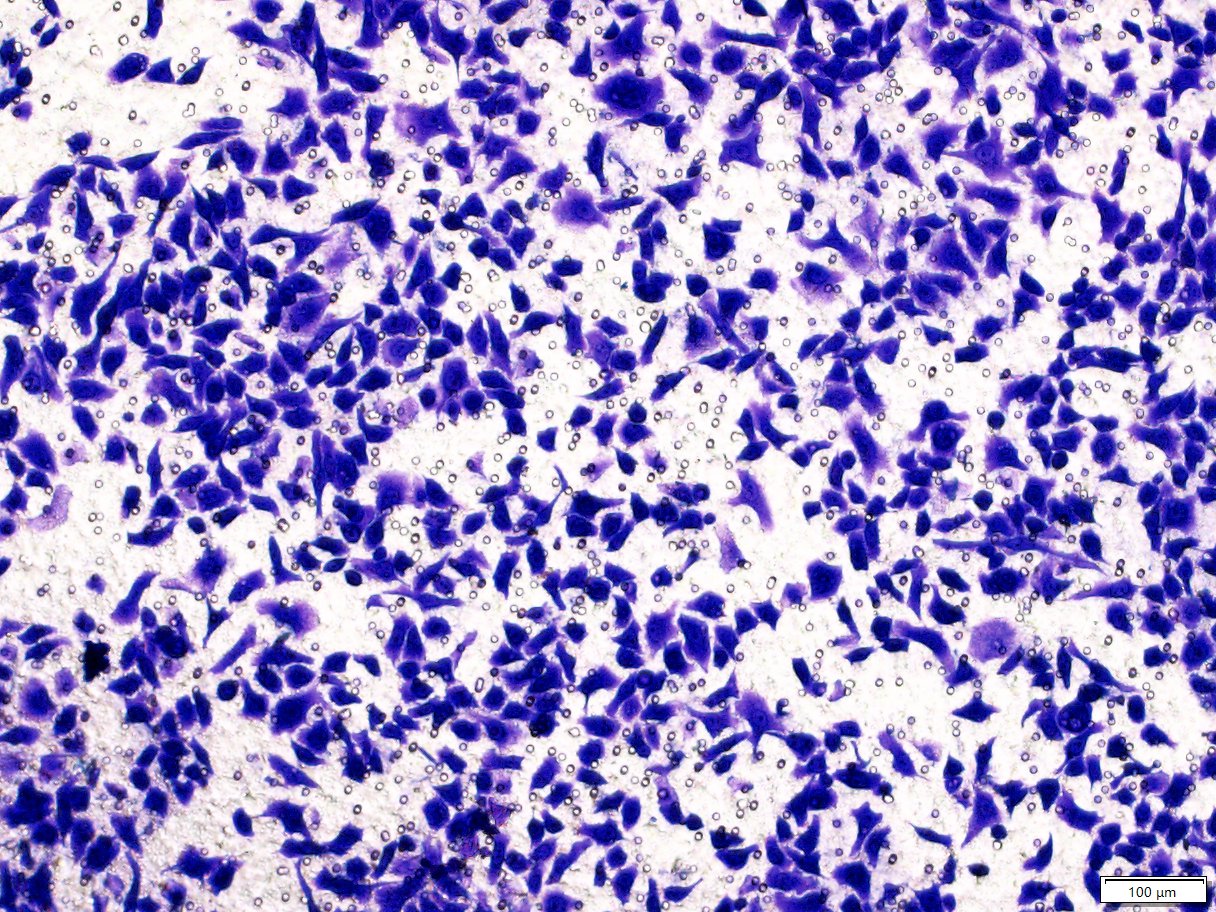

Supplement: Supplemental Information 8 [file peerj-cs-09-1651-s008.zip › Dataset 7/2-12.jpg]

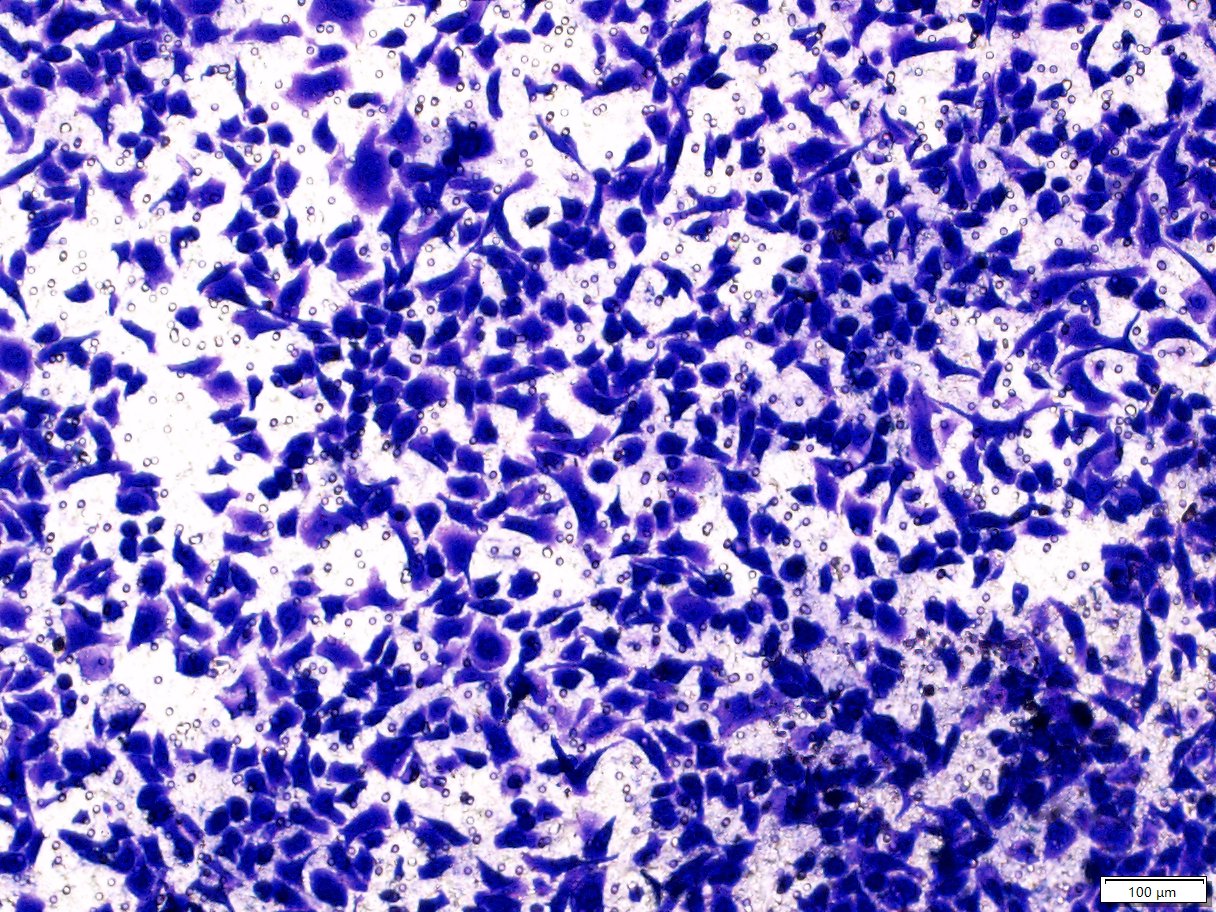

Supplement: Supplemental Information 8 [file peerj-cs-09-1651-s008.zip › Dataset 7/2-13.jpg]

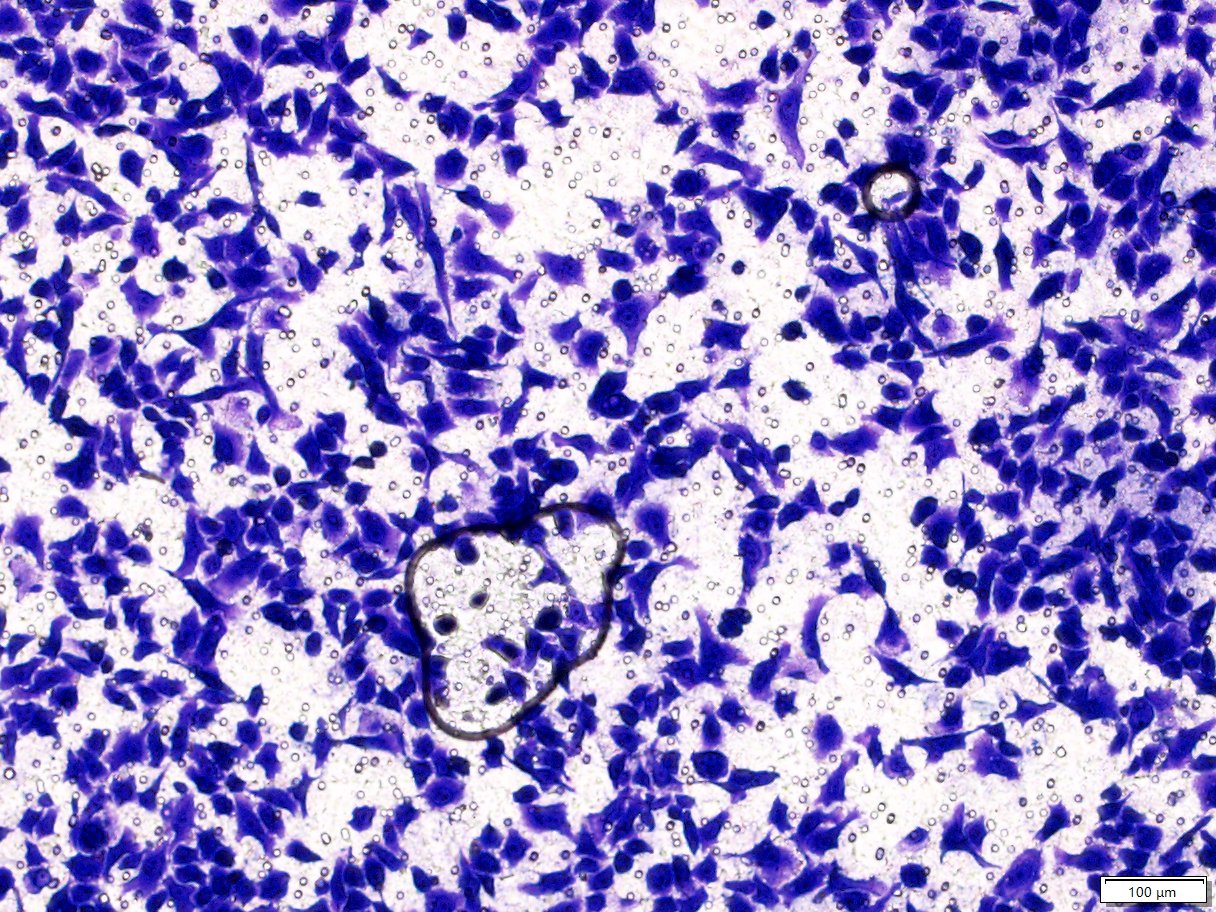

Supplement: Supplemental Information 8 [file peerj-cs-09-1651-s008.zip › Dataset 7/2-14.jpg]

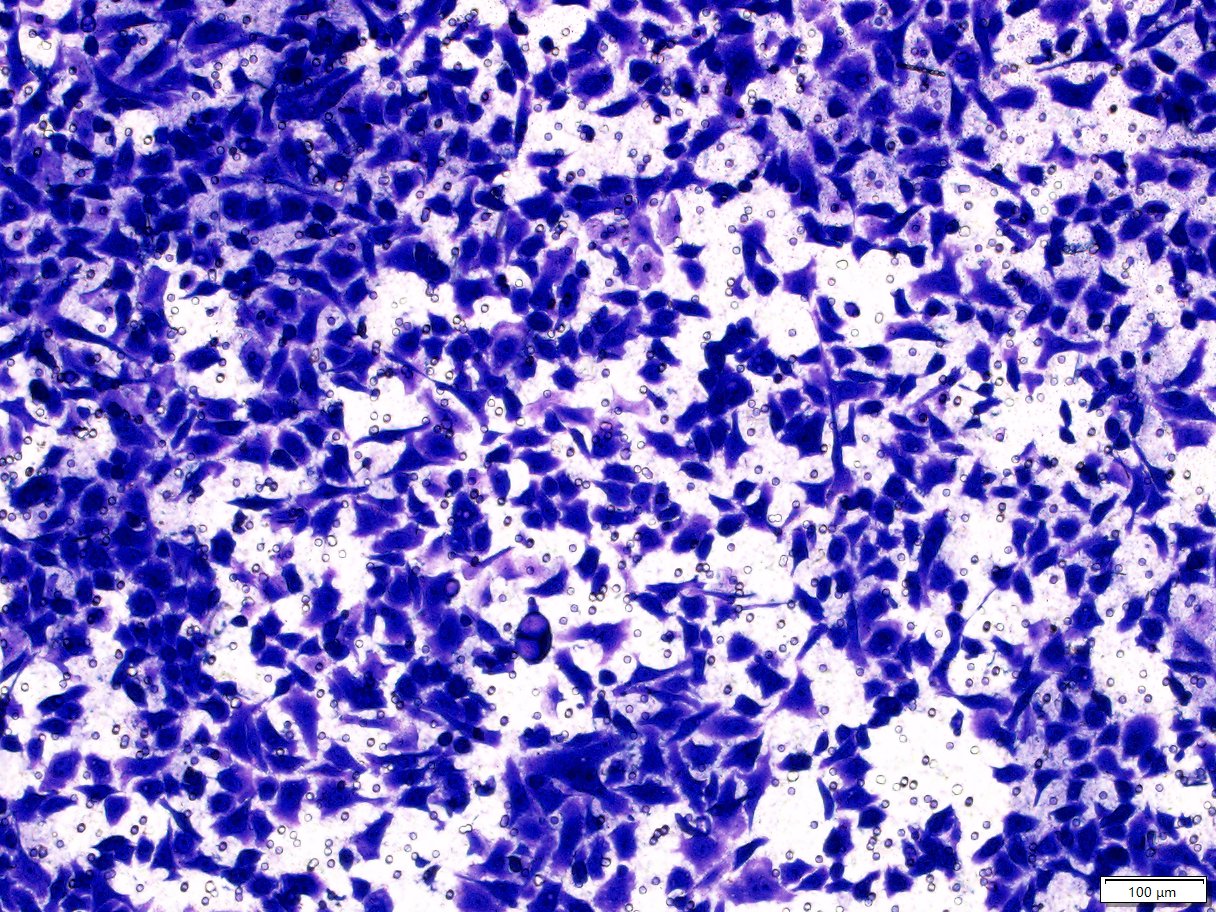

Supplement: Supplemental Information 8 [file peerj-cs-09-1651-s008.zip › Dataset 7/2-15.jpg]

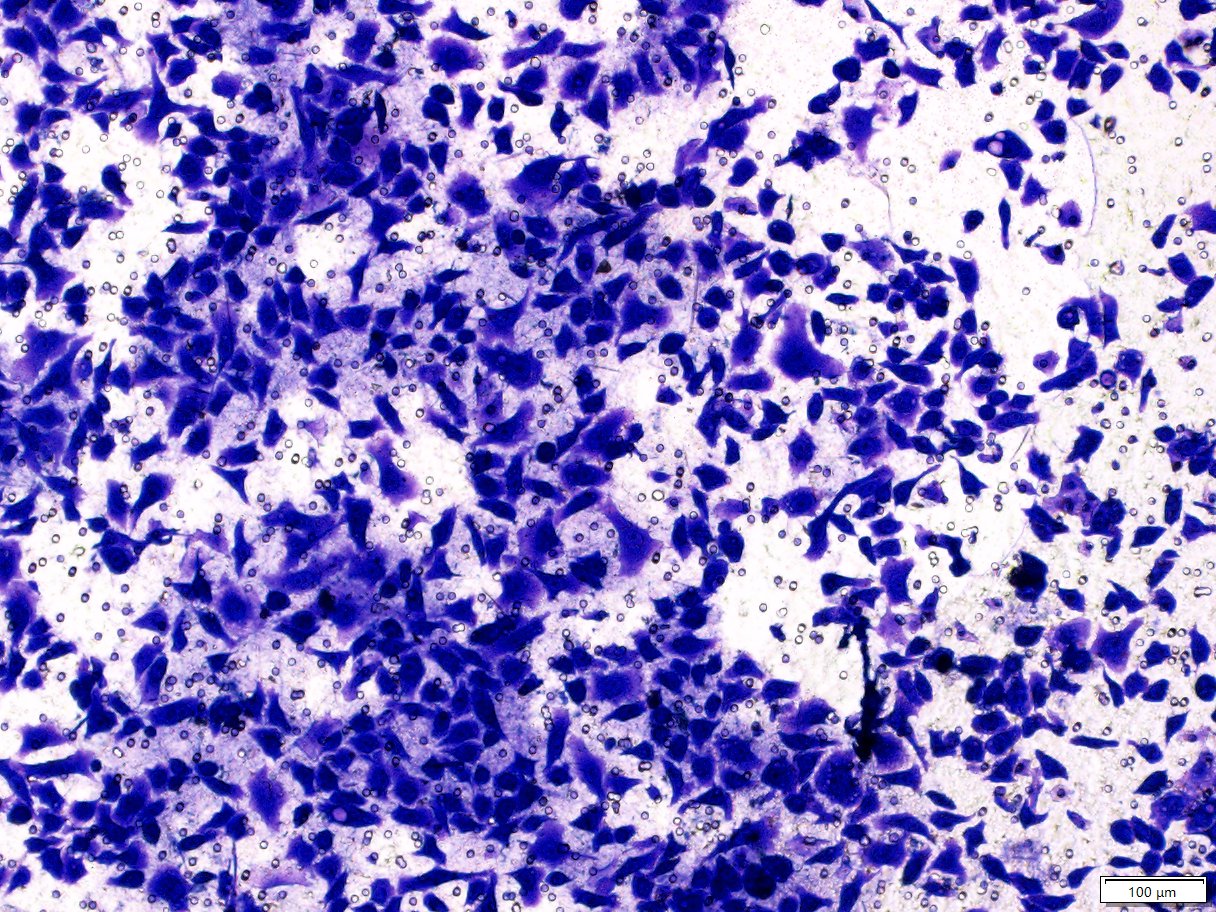

Supplement: Supplemental Information 8 [file peerj-cs-09-1651-s008.zip › Dataset 7/2-4.jpg]

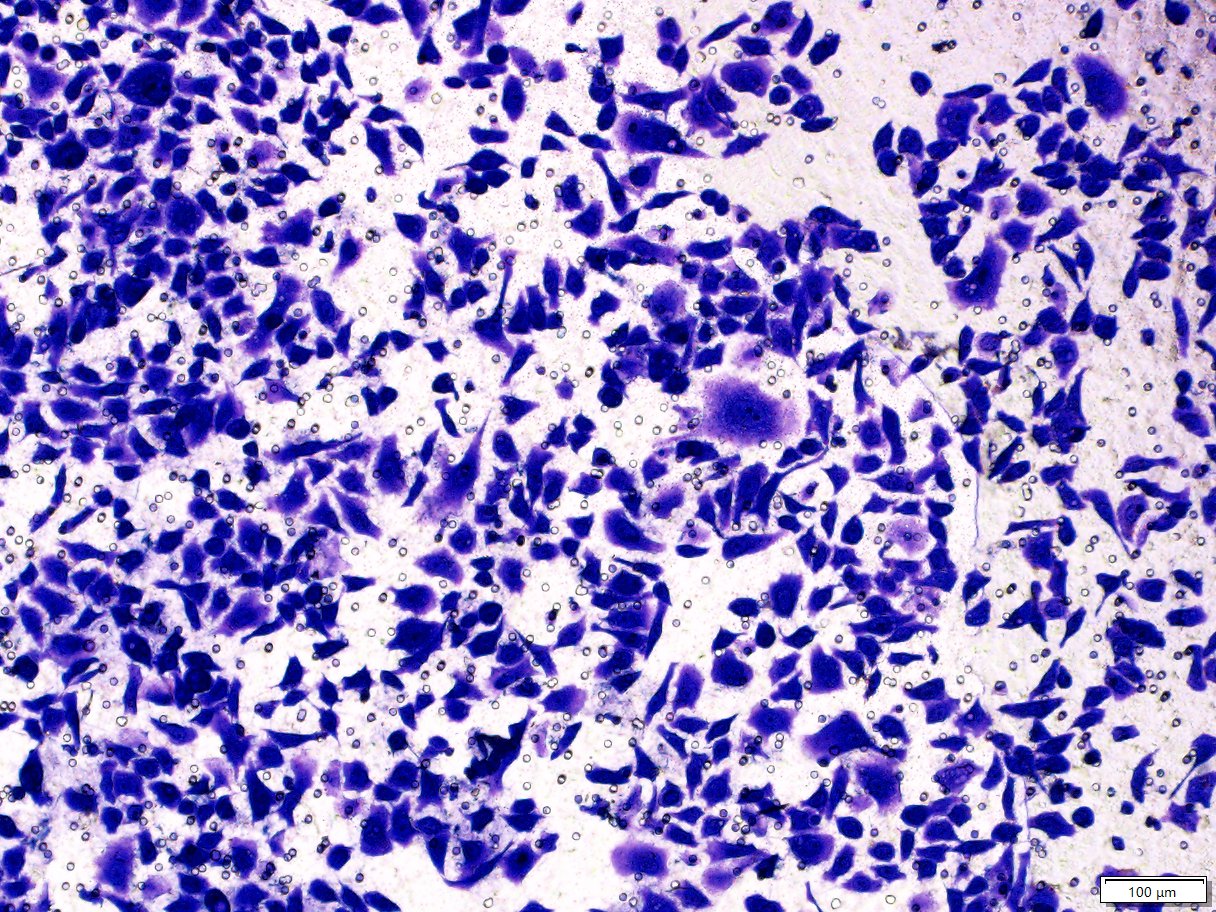

Supplement: Supplemental Information 8 [file peerj-cs-09-1651-s008.zip › Dataset 7/2-5.jpg]

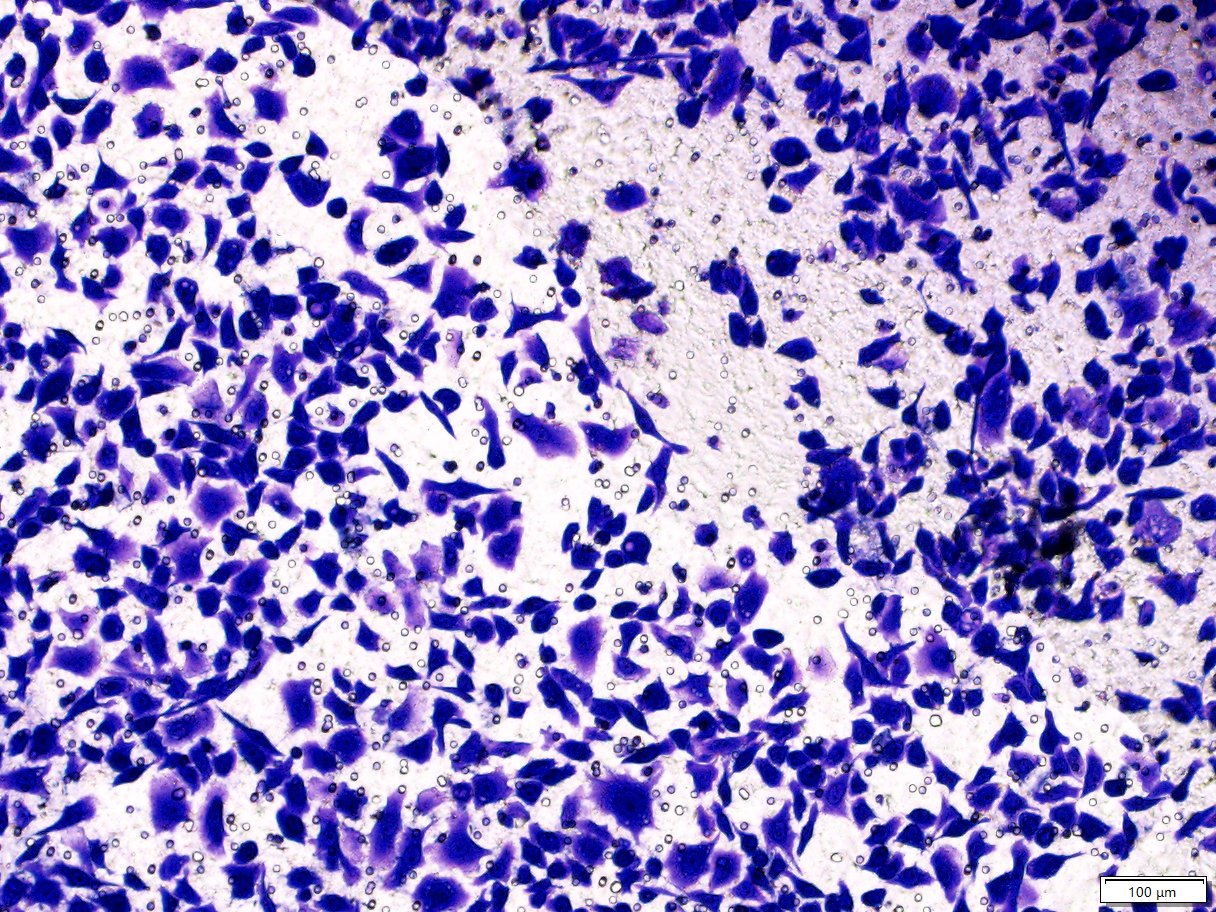

Supplement: Supplemental Information 8 [file peerj-cs-09-1651-s008.zip › Dataset 7/2-6.jpg]

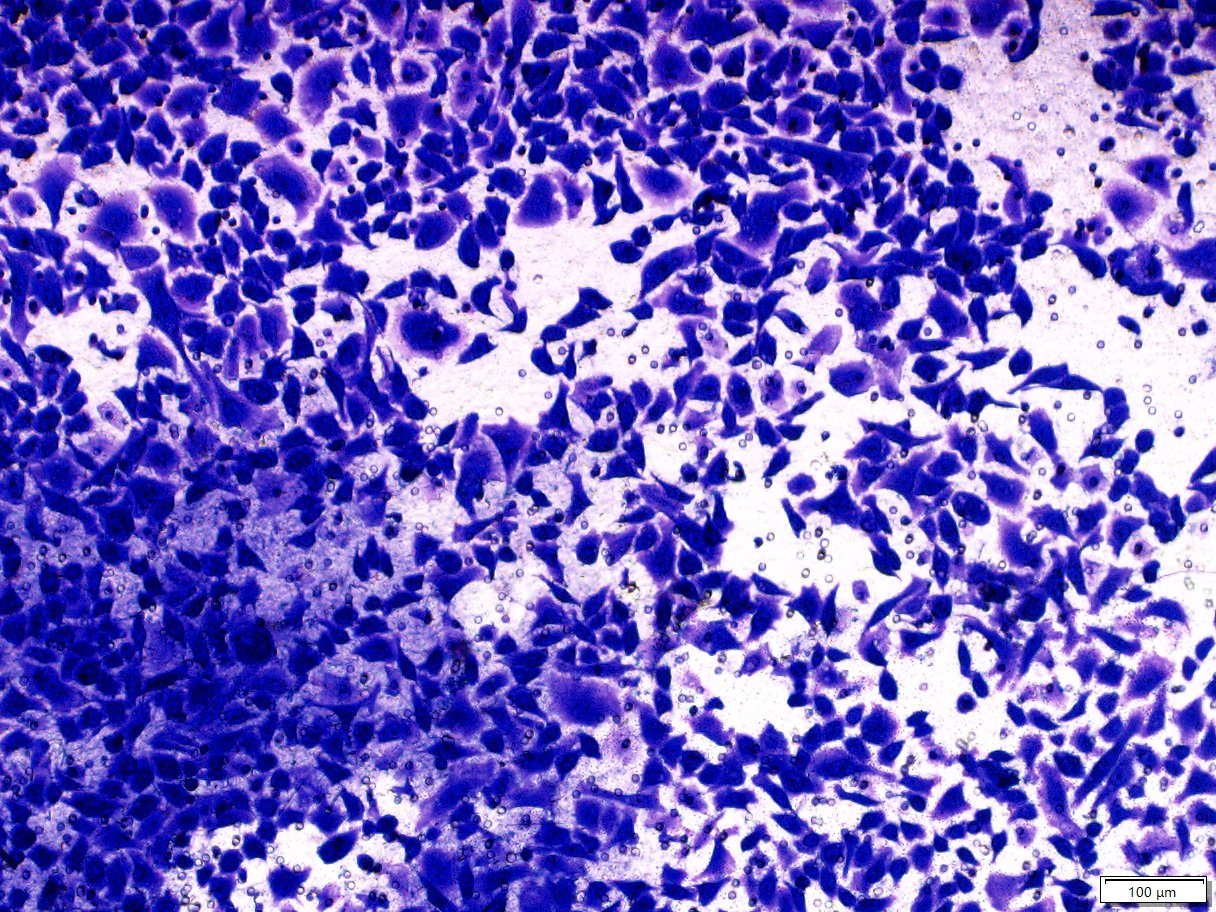

Supplement: Supplemental Information 8 [file peerj-cs-09-1651-s008.zip › Dataset 7/2-7.jpg]

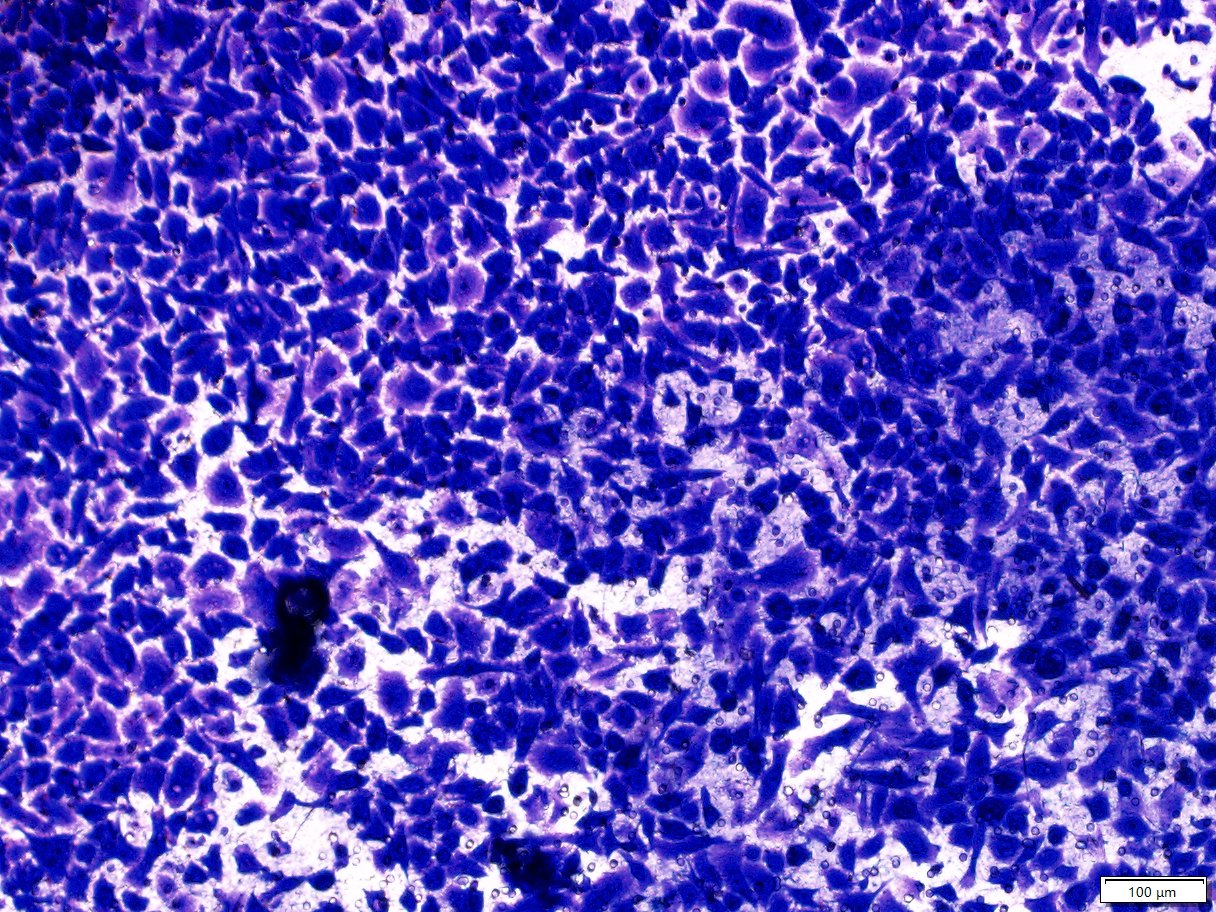

Supplement: Supplemental Information 8 [file peerj-cs-09-1651-s008.zip › Dataset 7/2-8.jpg]

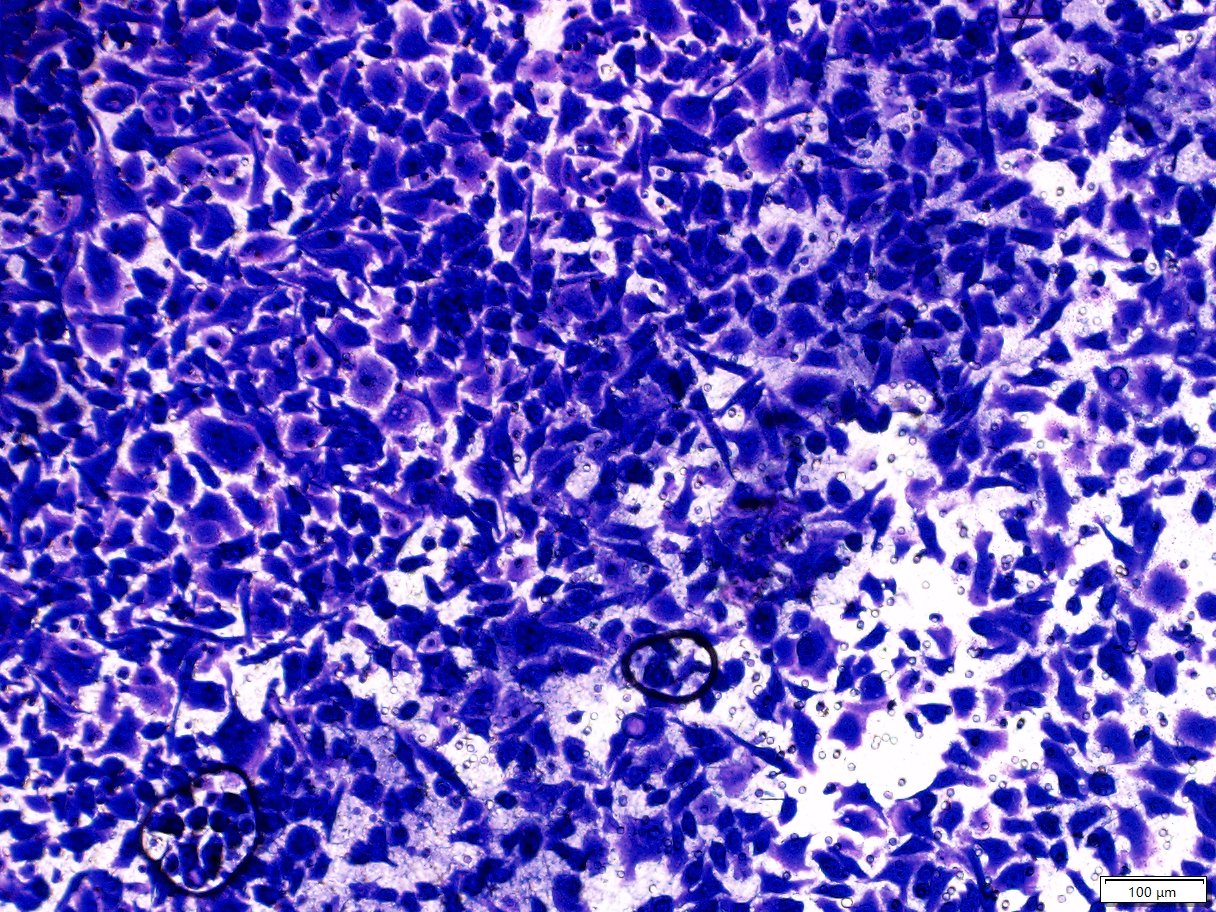

Supplement: Supplemental Information 8 [file peerj-cs-09-1651-s008.zip › Dataset 7/2-9.jpg]

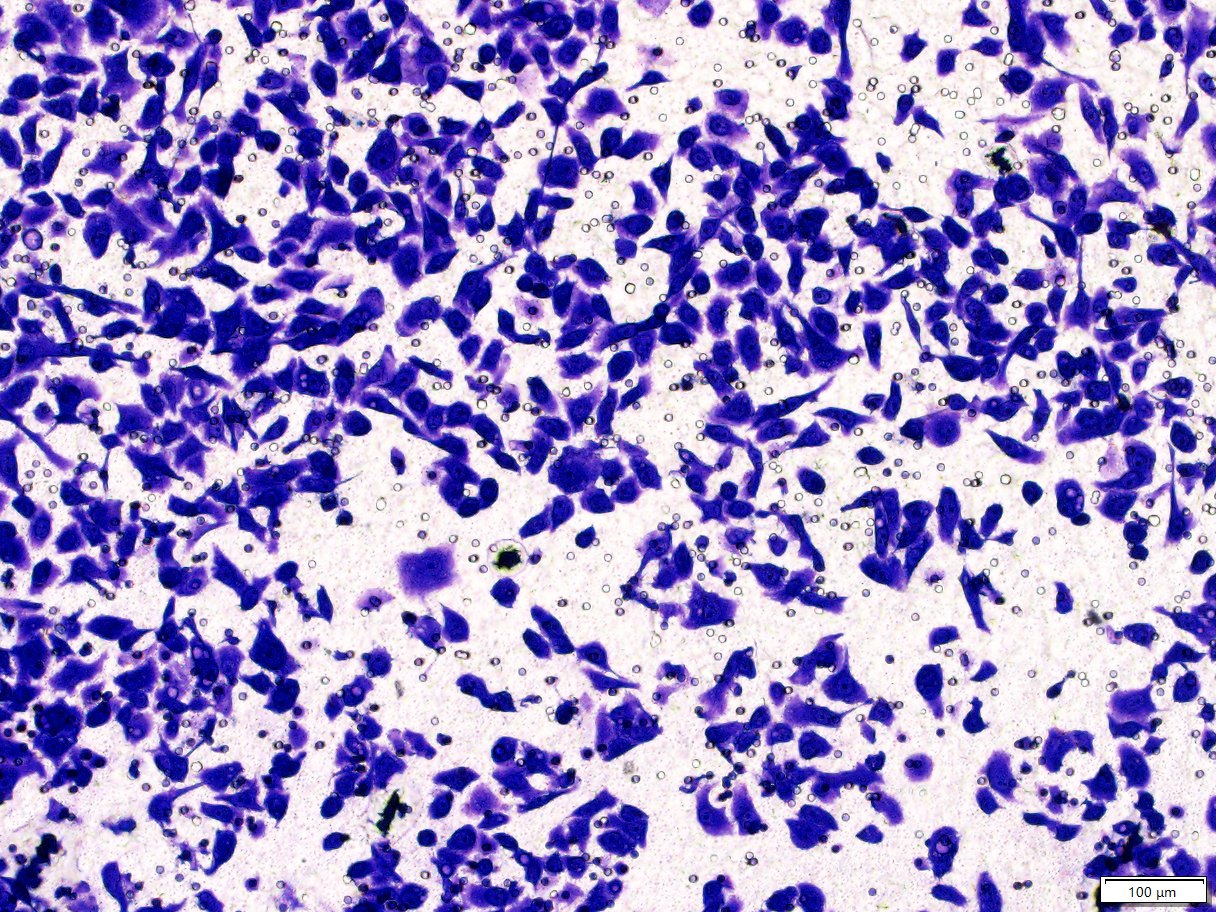

Supplement: Supplemental Information 8 [file peerj-cs-09-1651-s008.zip › Dataset 7/3+1.jpg]

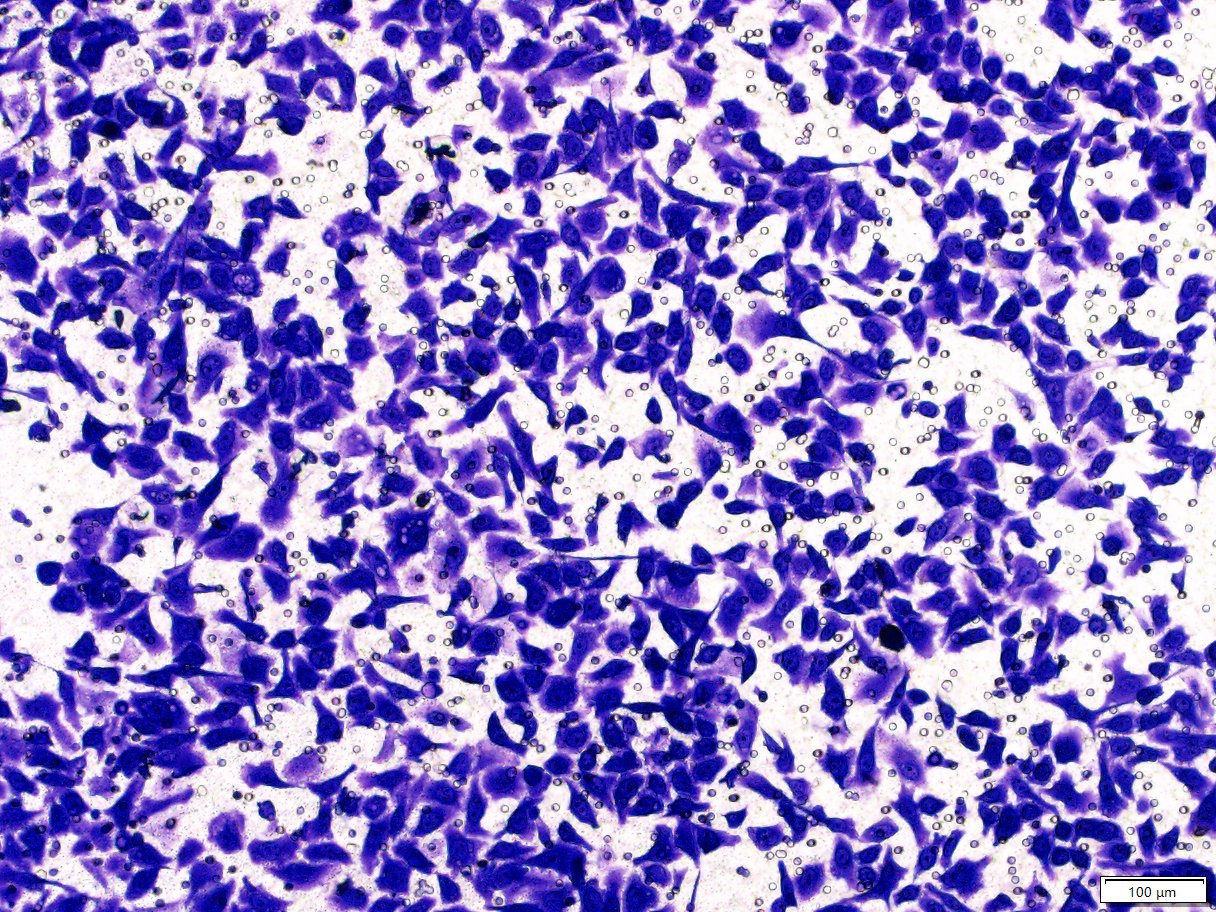

Supplement: Supplemental Information 8 [file peerj-cs-09-1651-s008.zip › Dataset 7/3+10.jpg]

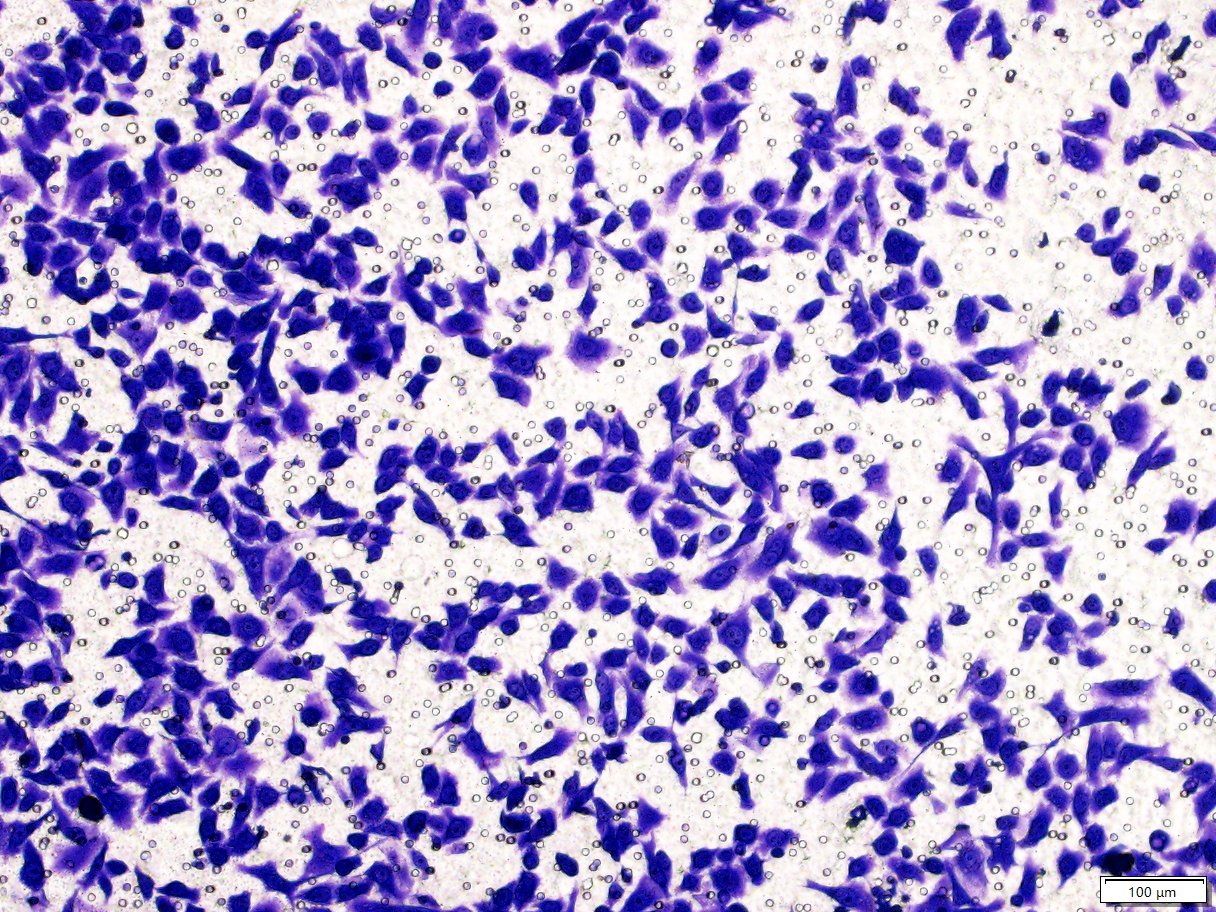

Supplement: Supplemental Information 8 [file peerj-cs-09-1651-s008.zip › Dataset 7/3+11.jpg]

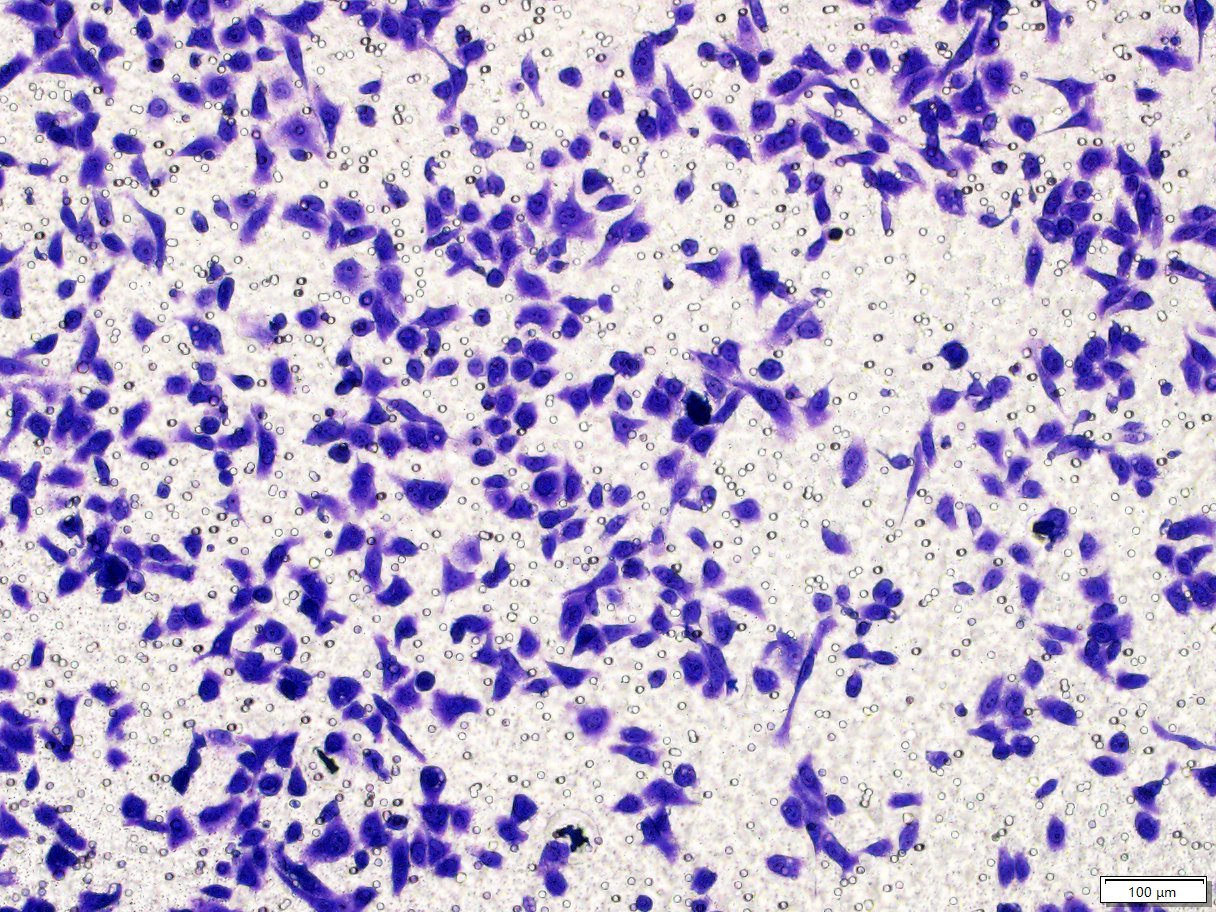

Supplement: Supplemental Information 8 [file peerj-cs-09-1651-s008.zip › Dataset 7/3+12.jpg]

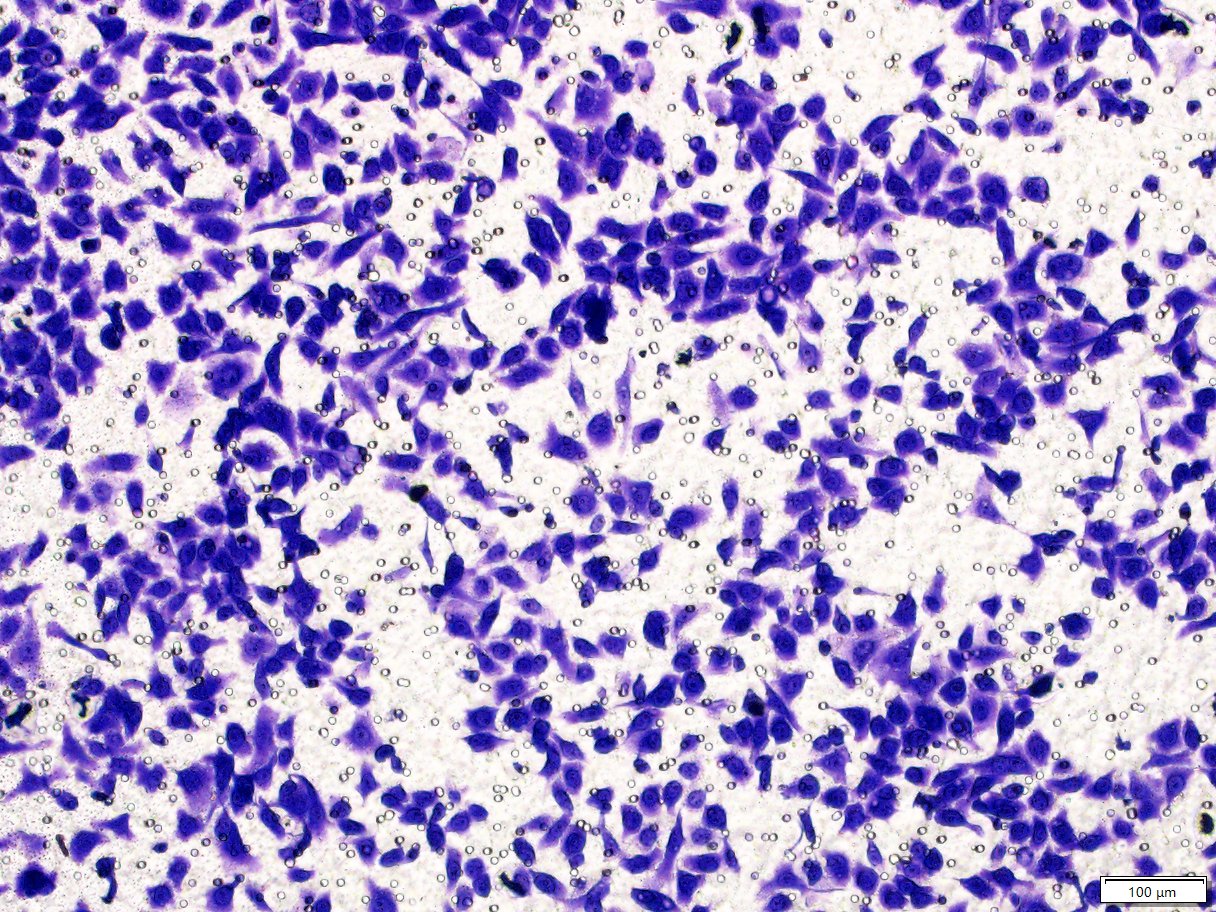

Supplement: Supplemental Information 8 [file peerj-cs-09-1651-s008.zip › Dataset 7/3+13.jpg]

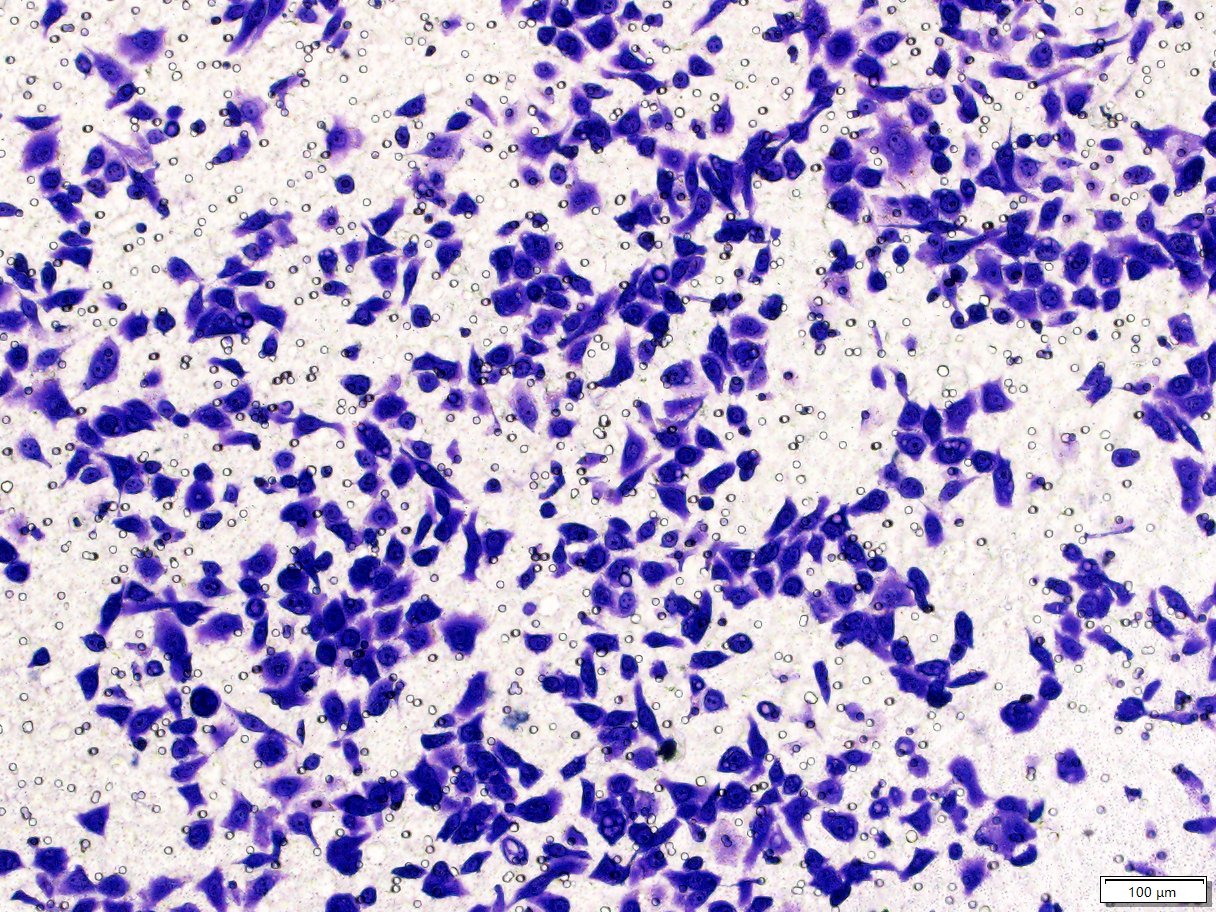

Supplement: Supplemental Information 8 [file peerj-cs-09-1651-s008.zip › Dataset 7/3+2.jpg]

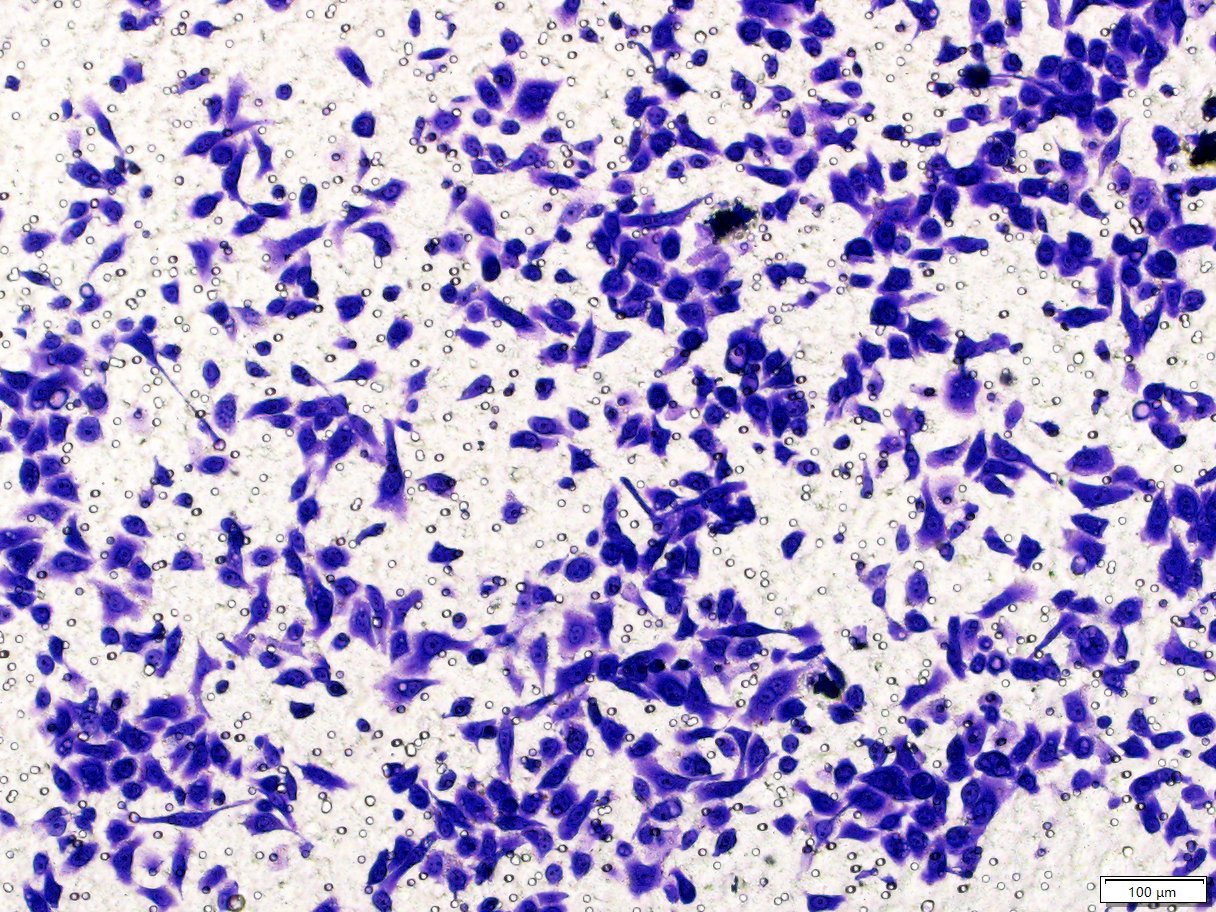

Supplement: Supplemental Information 8 [file peerj-cs-09-1651-s008.zip › Dataset 7/3+3.jpg]

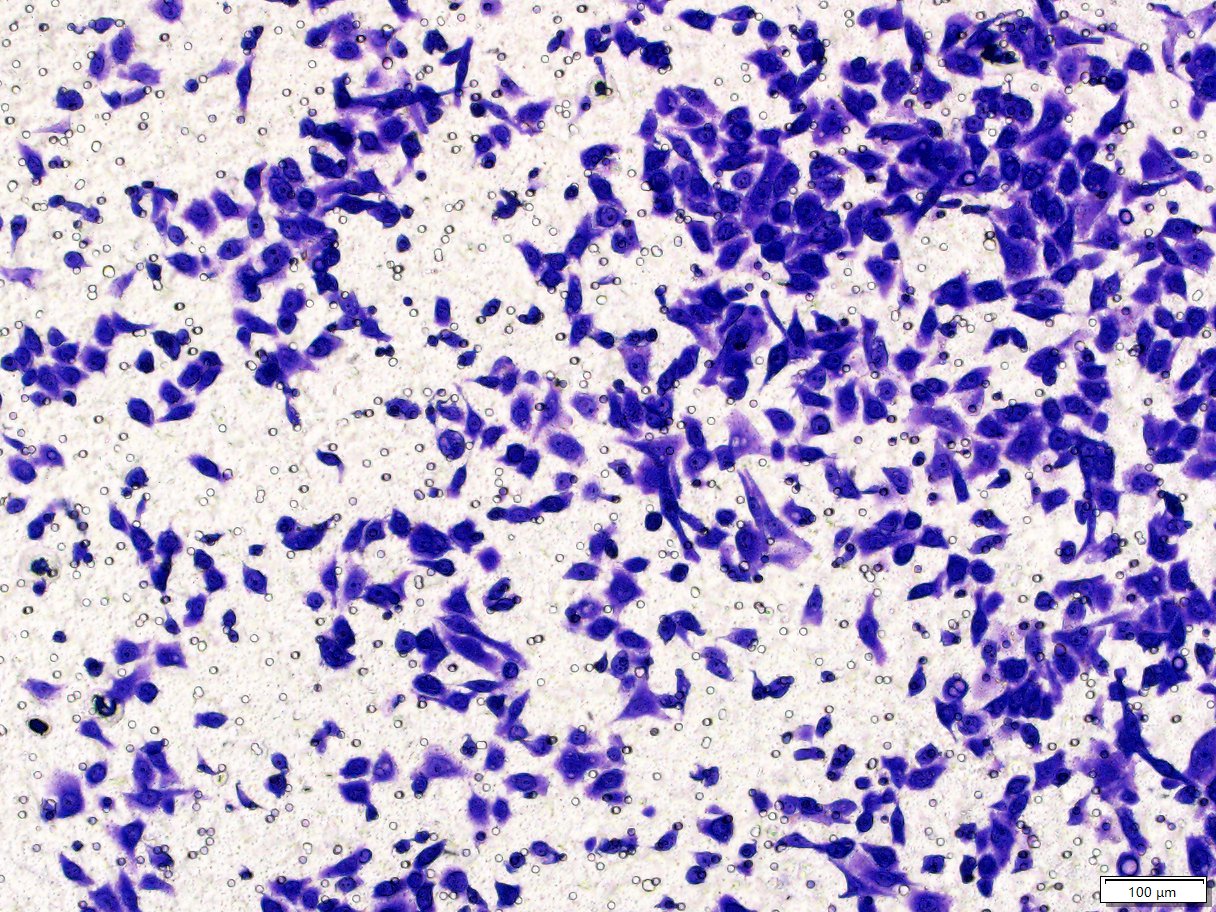

Supplement: Supplemental Information 8 [file peerj-cs-09-1651-s008.zip › Dataset 7/3+4.jpg]

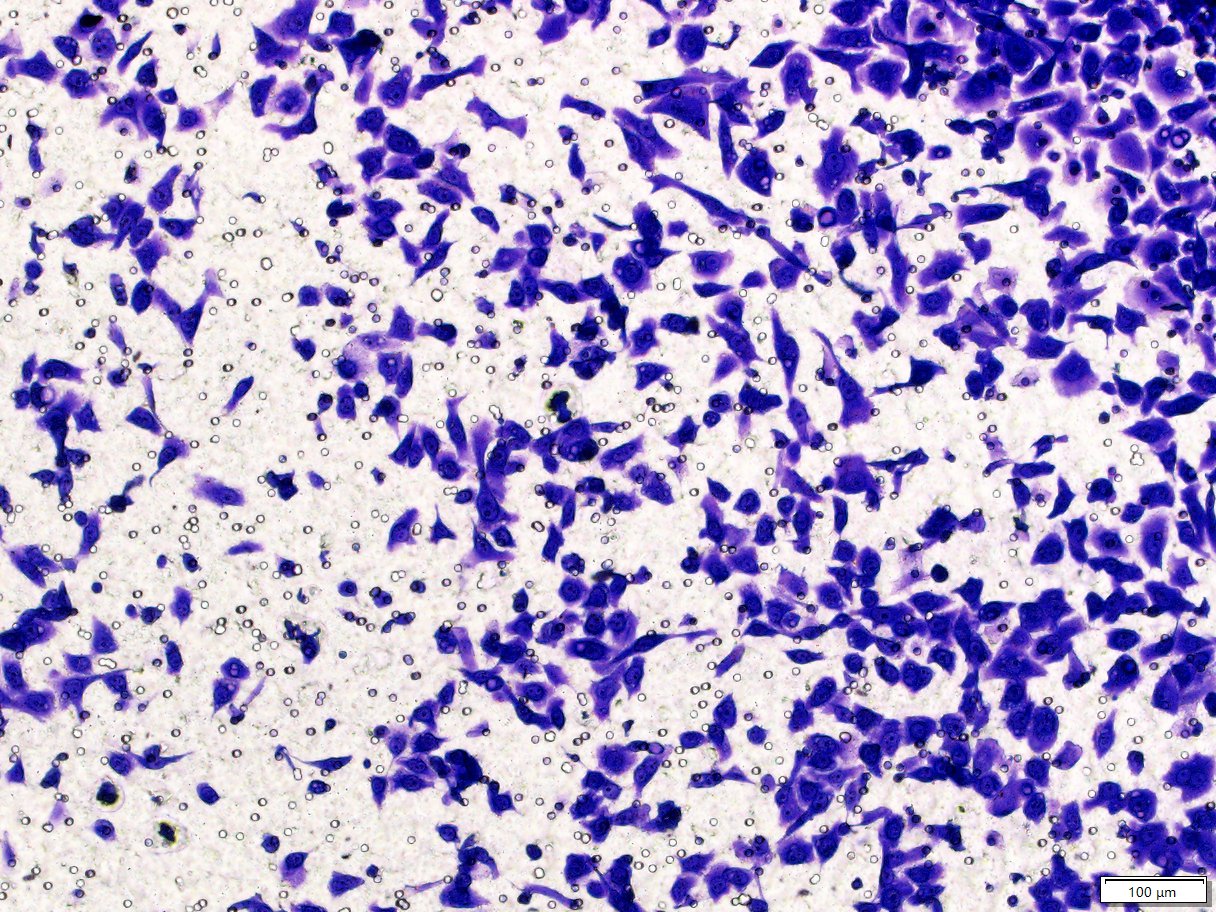

Supplement: Supplemental Information 8 [file peerj-cs-09-1651-s008.zip › Dataset 7/3+5.jpg]

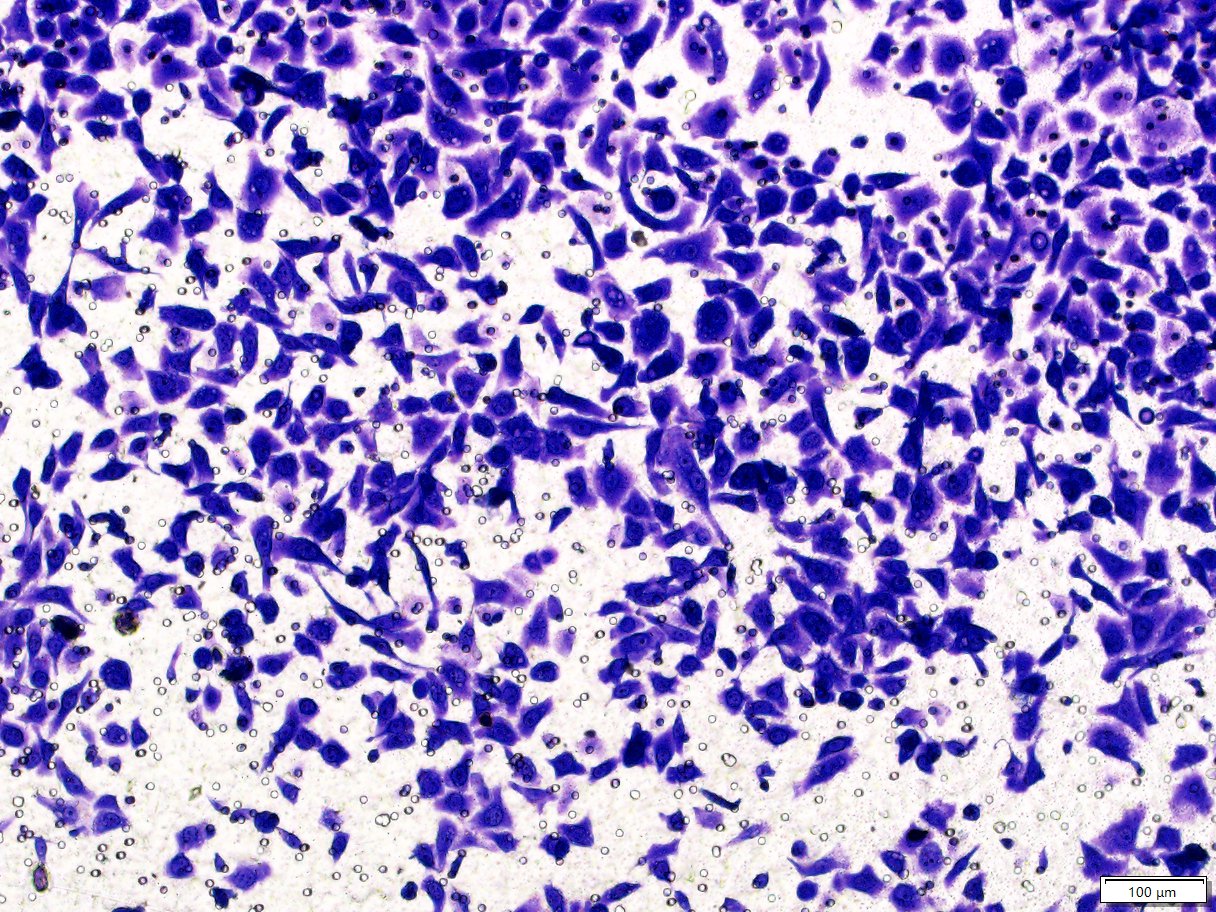

Supplement: Supplemental Information 8 [file peerj-cs-09-1651-s008.zip › Dataset 7/3+6.jpg]

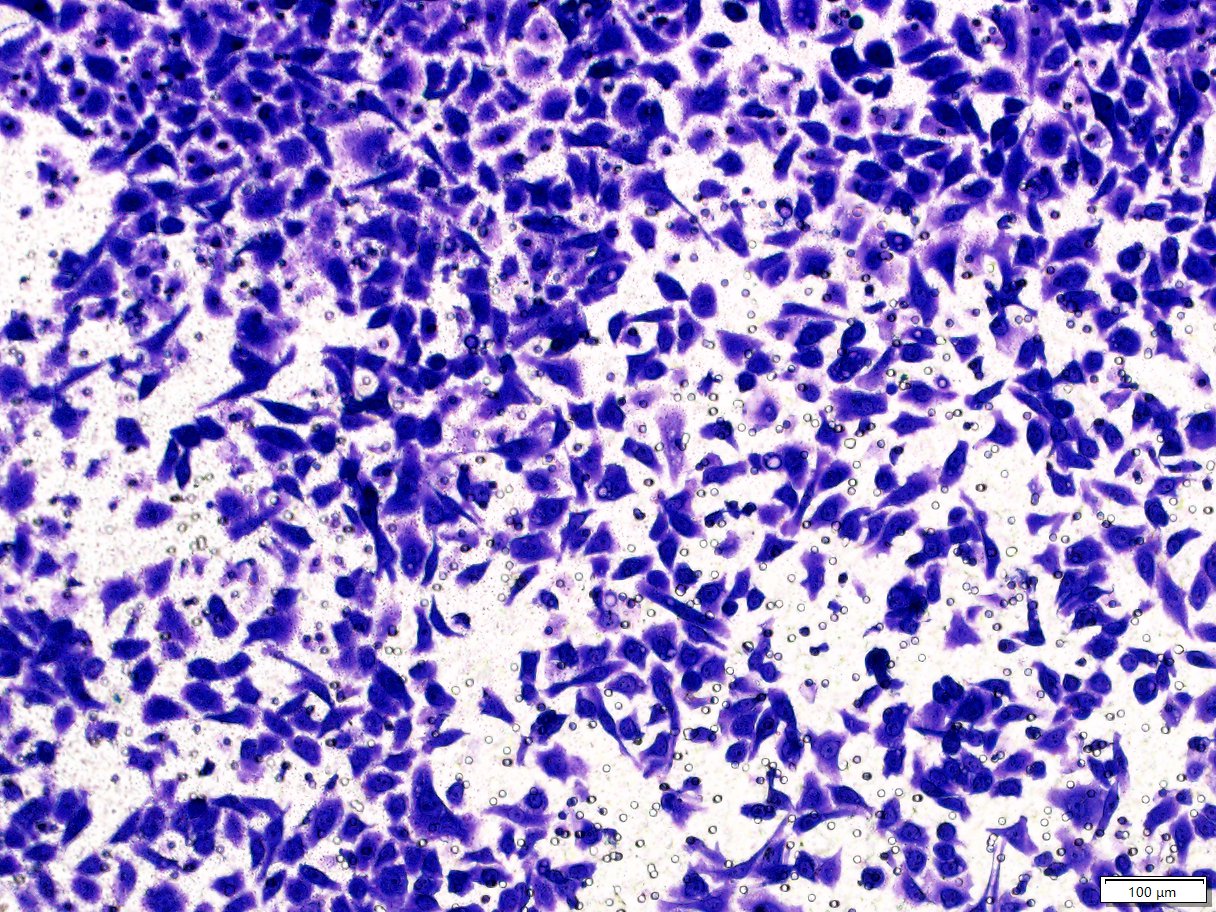

Supplement: Supplemental Information 8 [file peerj-cs-09-1651-s008.zip › Dataset 7/3+7.jpg]

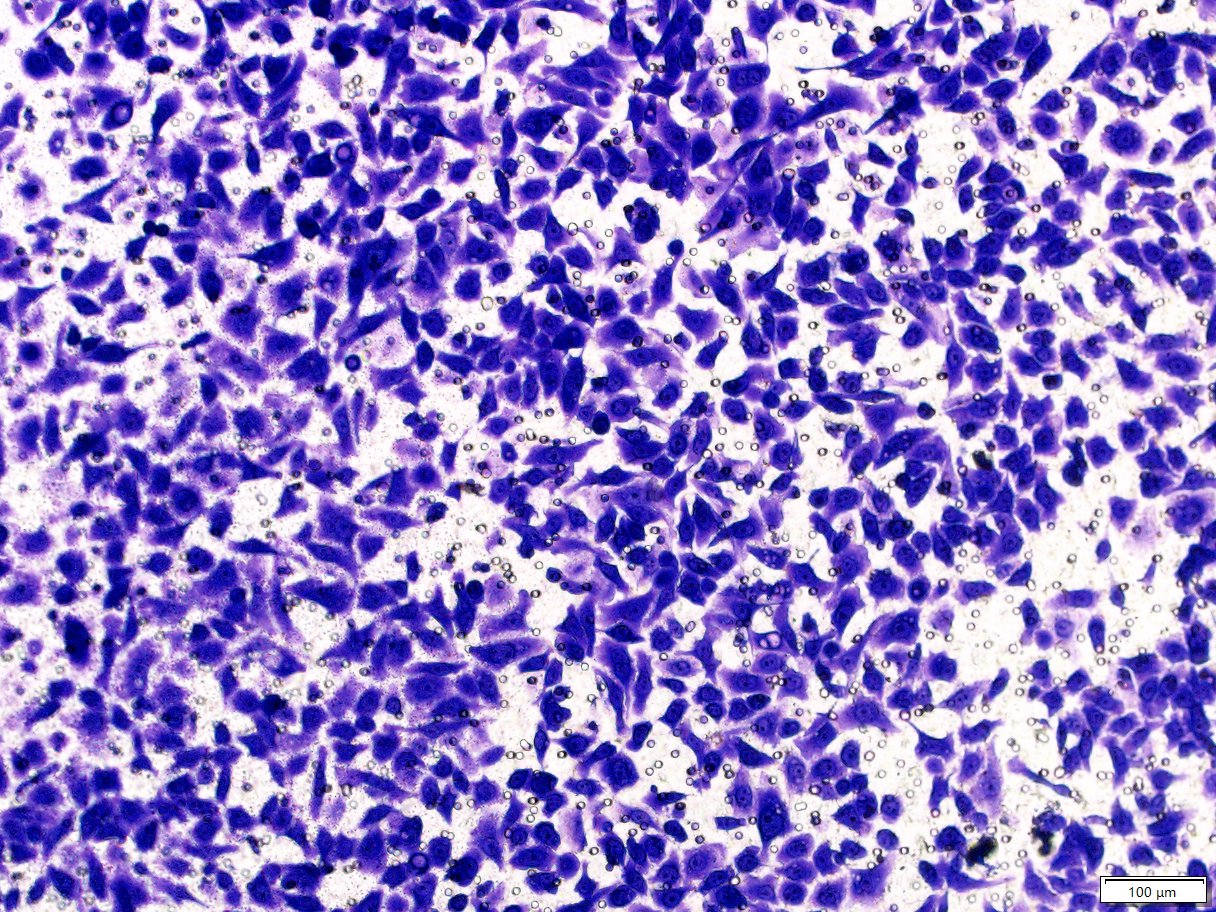

Supplement: Supplemental Information 8 [file peerj-cs-09-1651-s008.zip › Dataset 7/3+8.jpg]

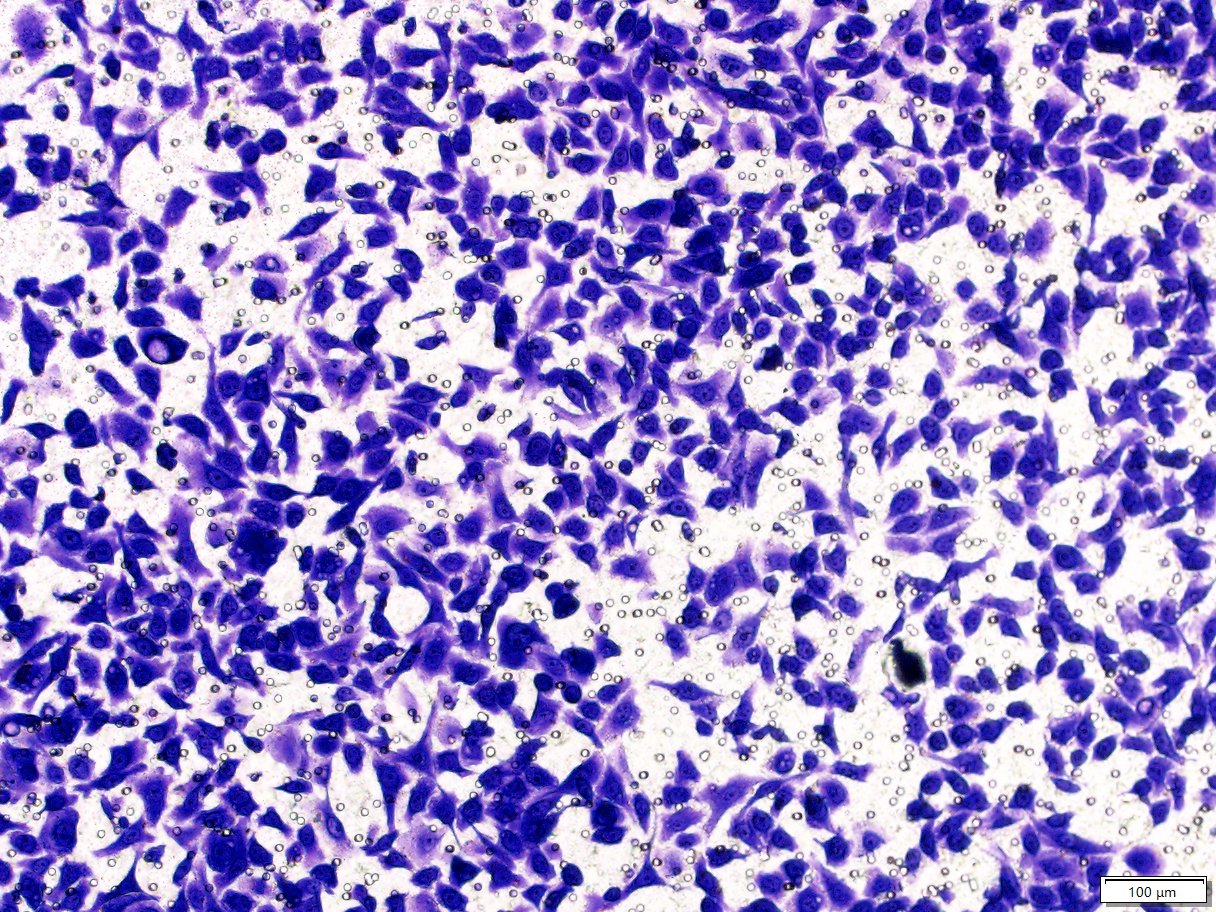

Supplement: Supplemental Information 8 [file peerj-cs-09-1651-s008.zip › Dataset 7/3+9.jpg]

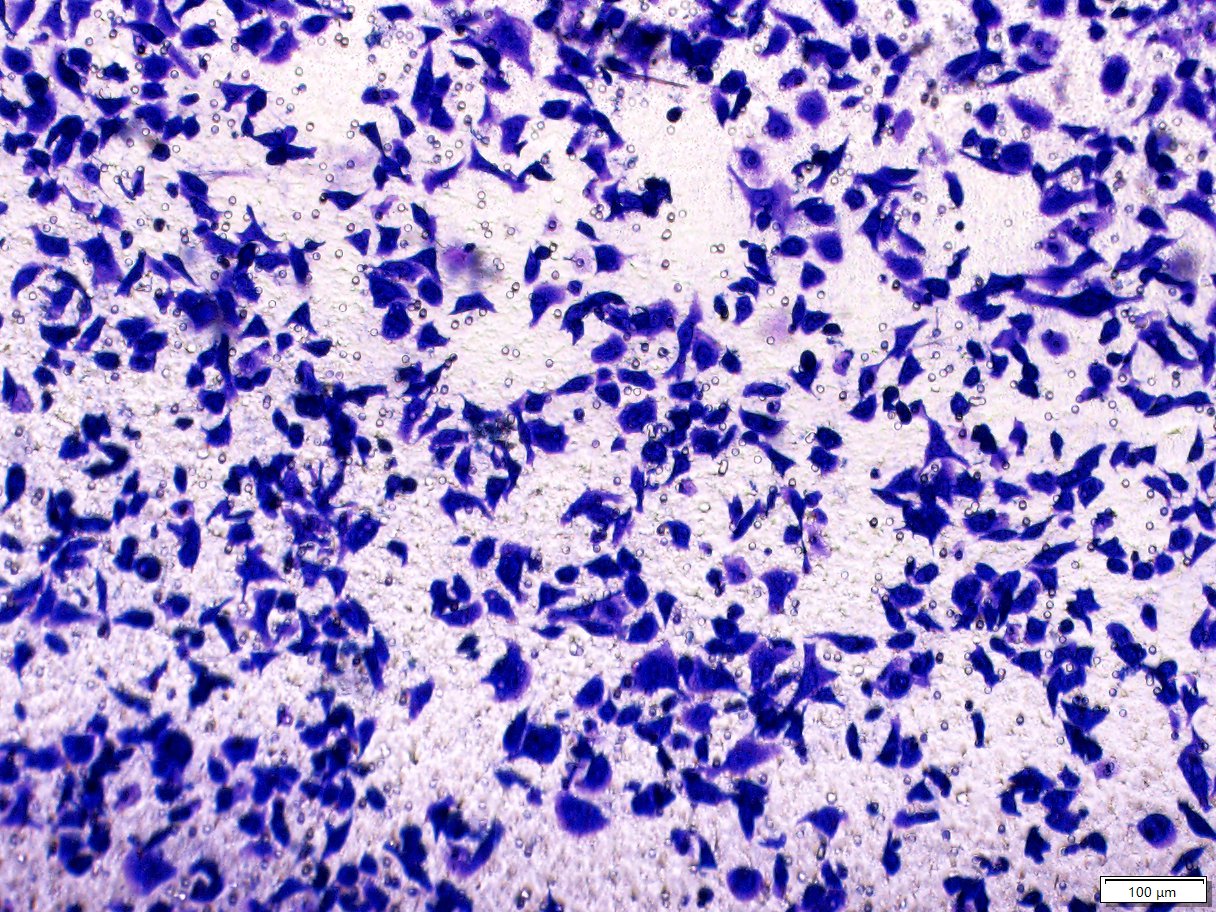

Supplement: Supplemental Information 8 [file peerj-cs-09-1651-s008.zip › Dataset 7/3-1.jpg]

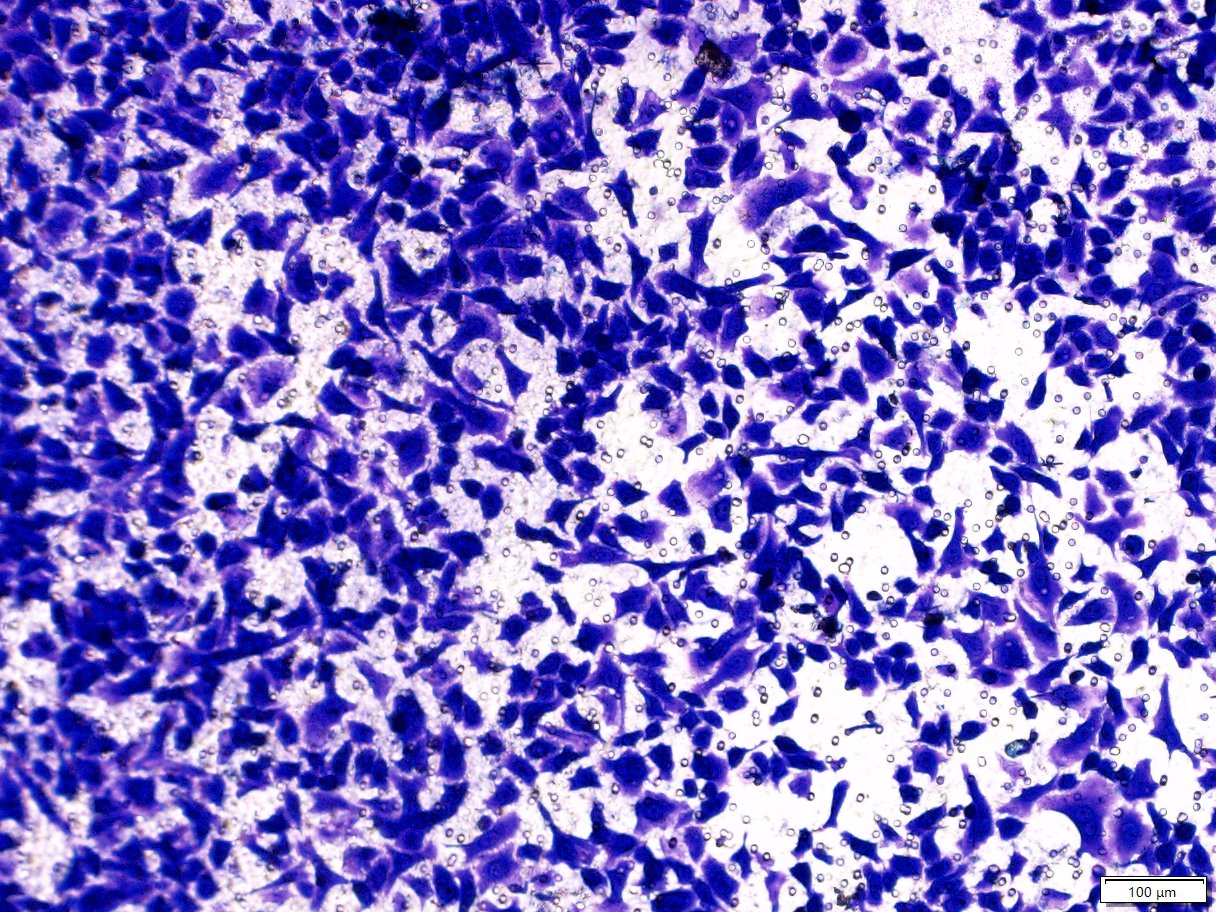

Supplement: Supplemental Information 8 [file peerj-cs-09-1651-s008.zip › Dataset 7/3-10.jpg]

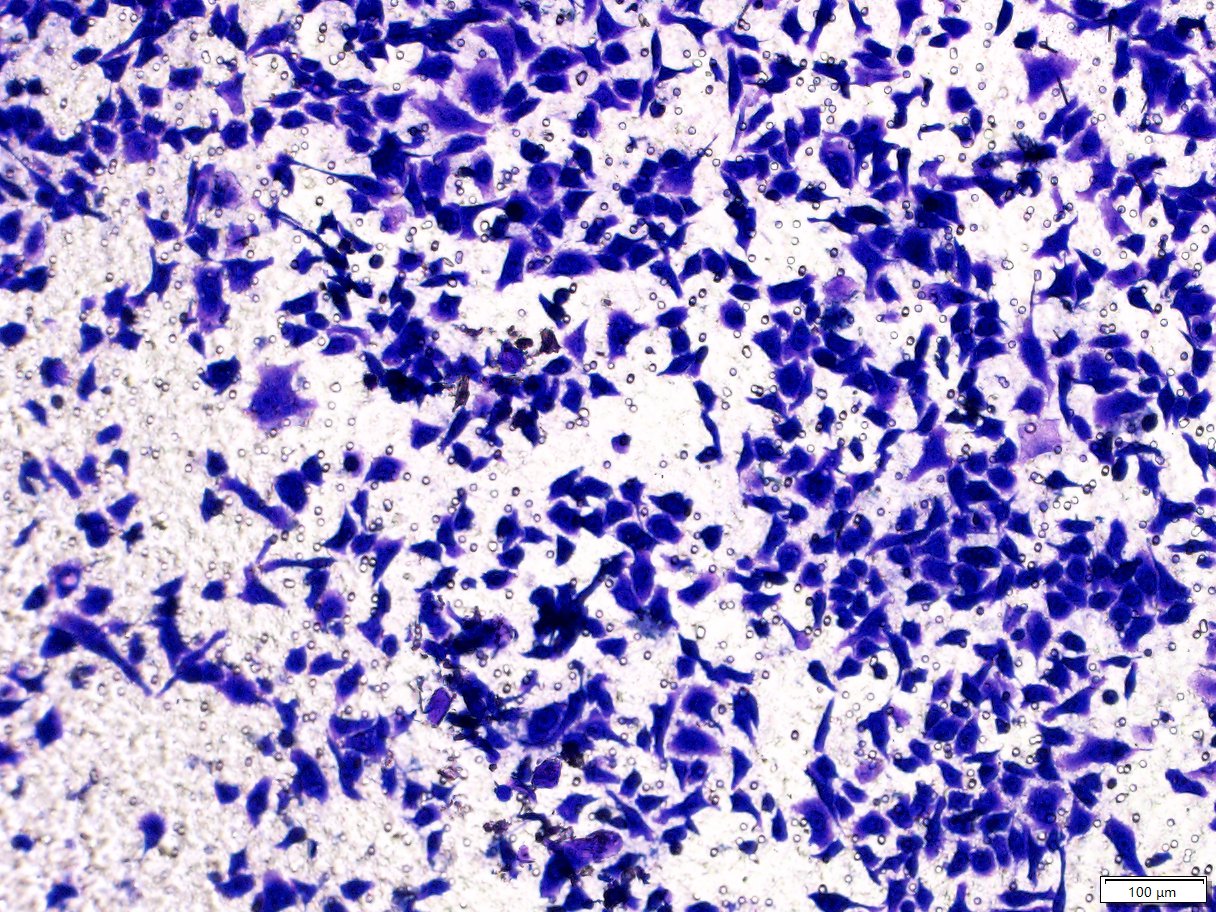

Supplement: Supplemental Information 8 [file peerj-cs-09-1651-s008.zip › Dataset 7/3-11.jpg]

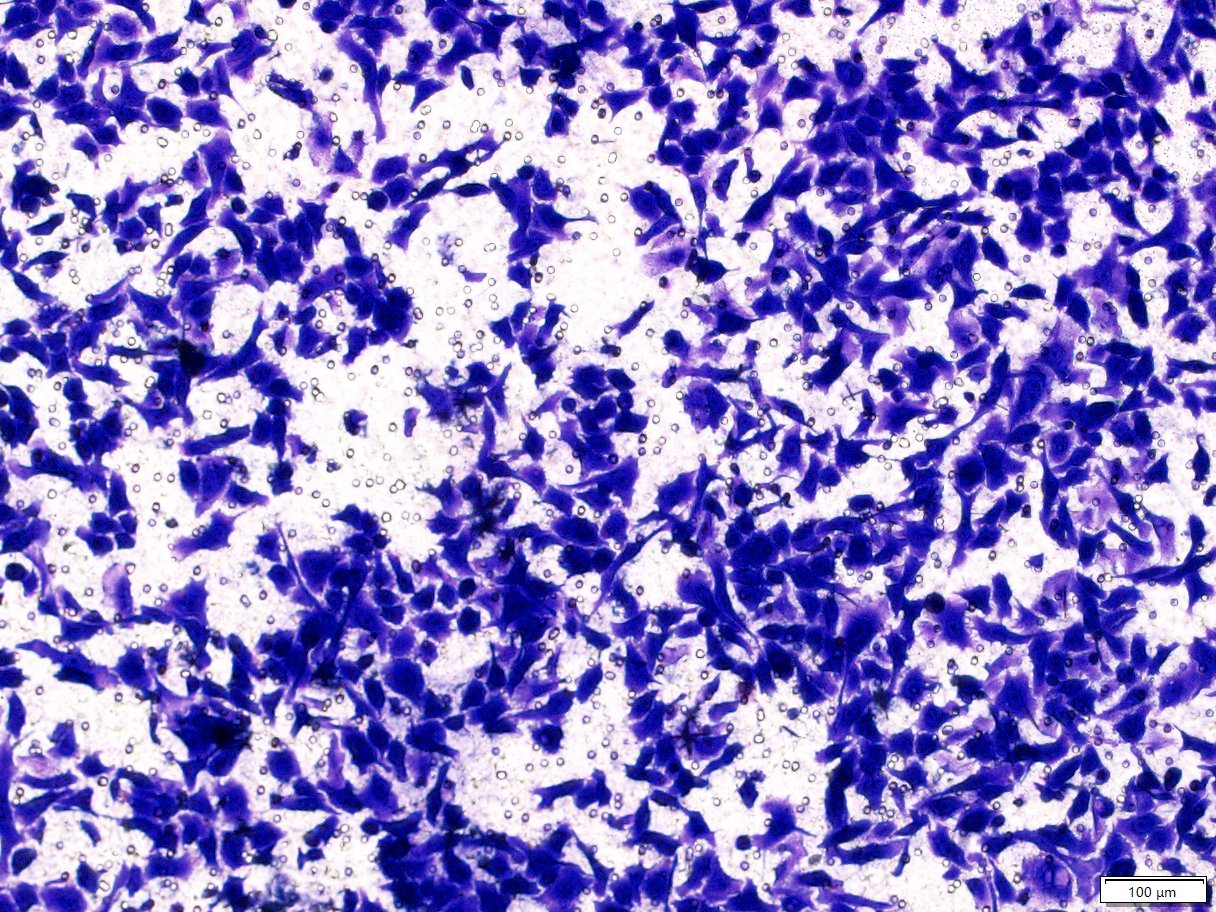

Supplement: Supplemental Information 8 [file peerj-cs-09-1651-s008.zip › Dataset 7/3-12.jpg]

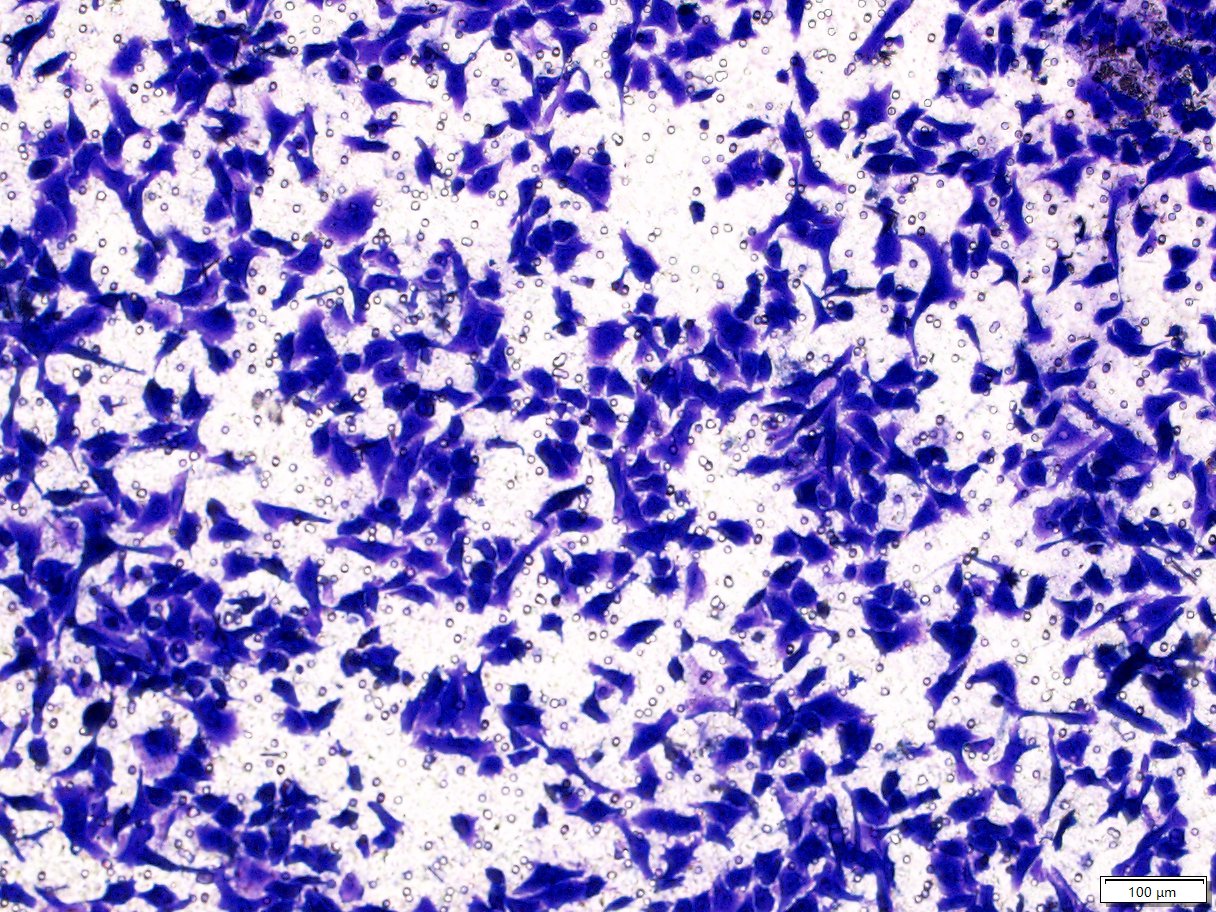

Supplement: Supplemental Information 8 [file peerj-cs-09-1651-s008.zip › Dataset 7/3-13.jpg]

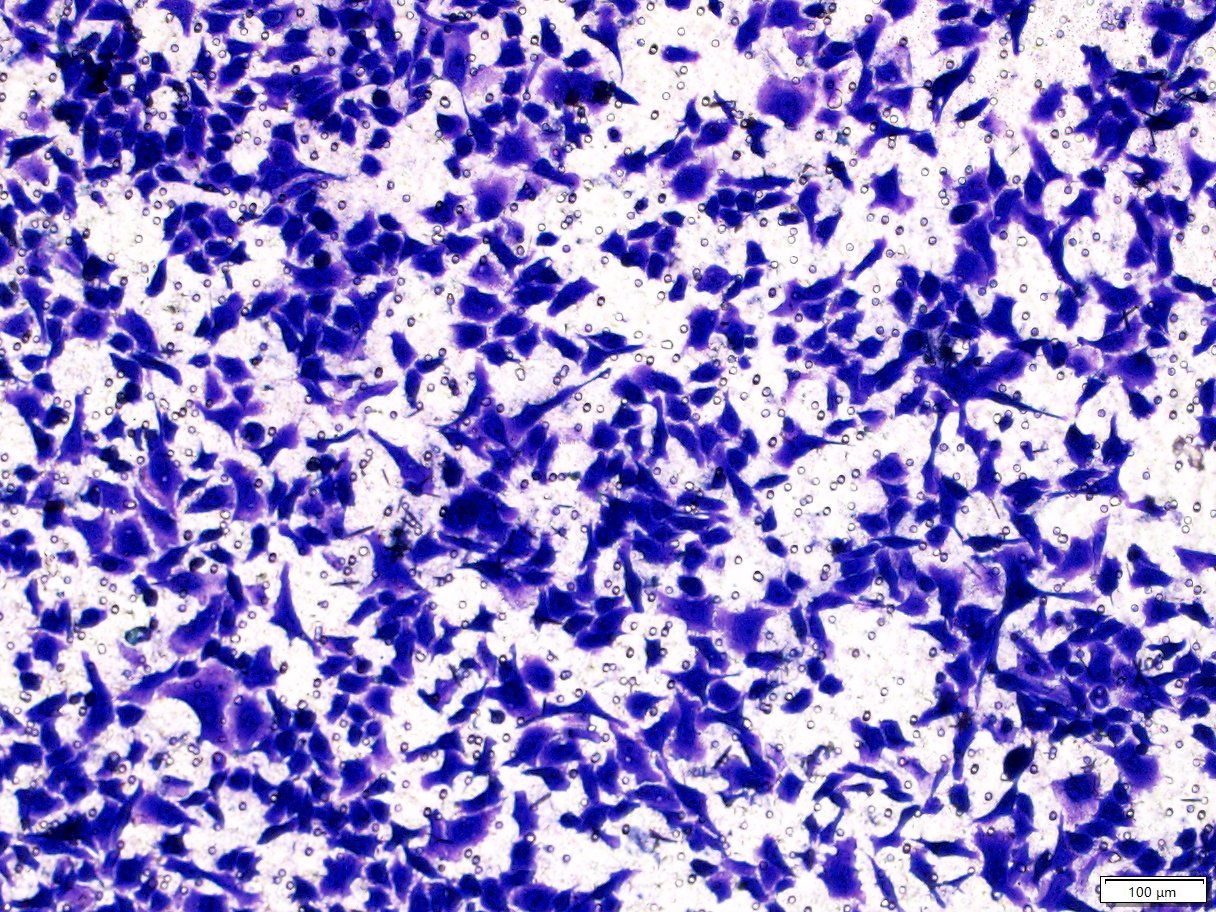

Supplement: Supplemental Information 8 [file peerj-cs-09-1651-s008.zip › Dataset 7/3-14.jpg]

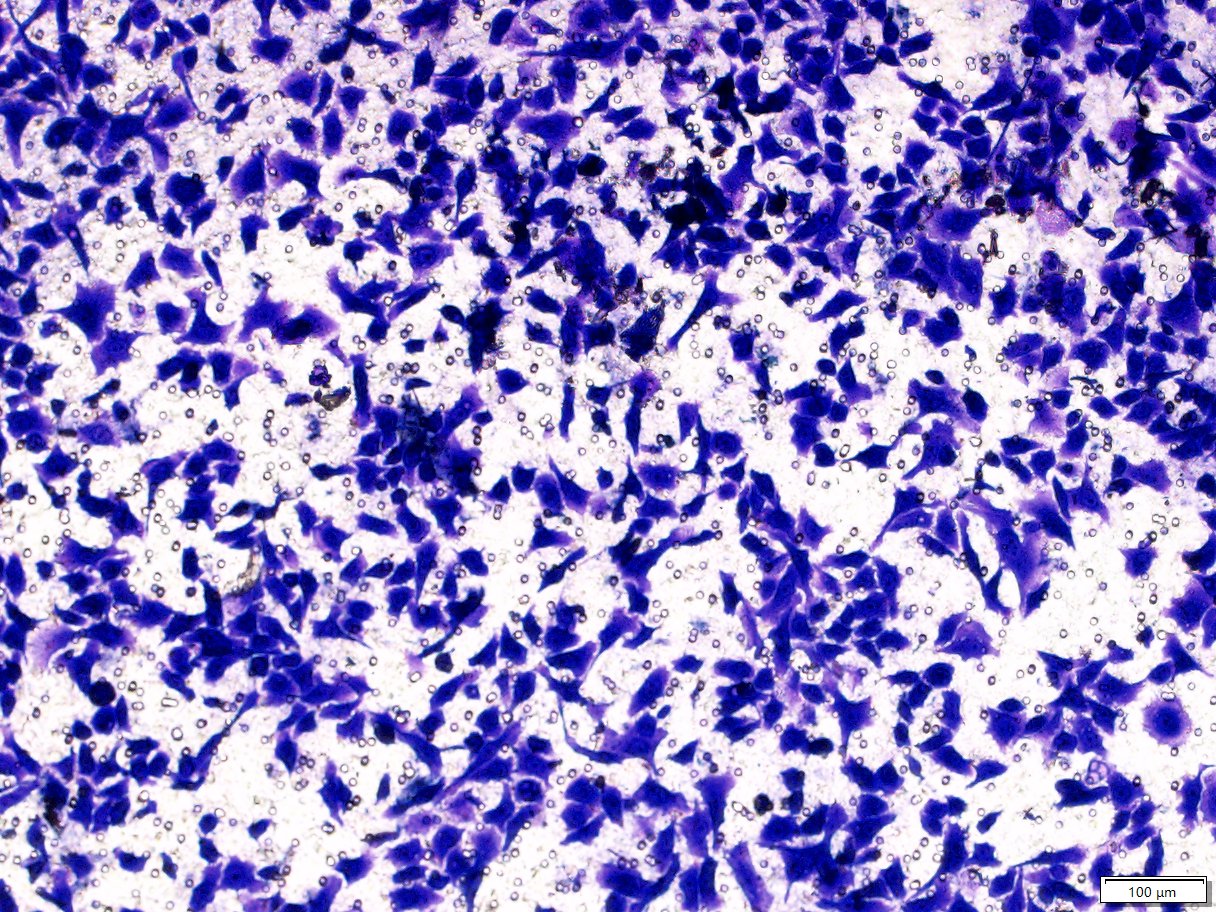

Supplement: Supplemental Information 8 [file peerj-cs-09-1651-s008.zip › Dataset 7/3-15.jpg]

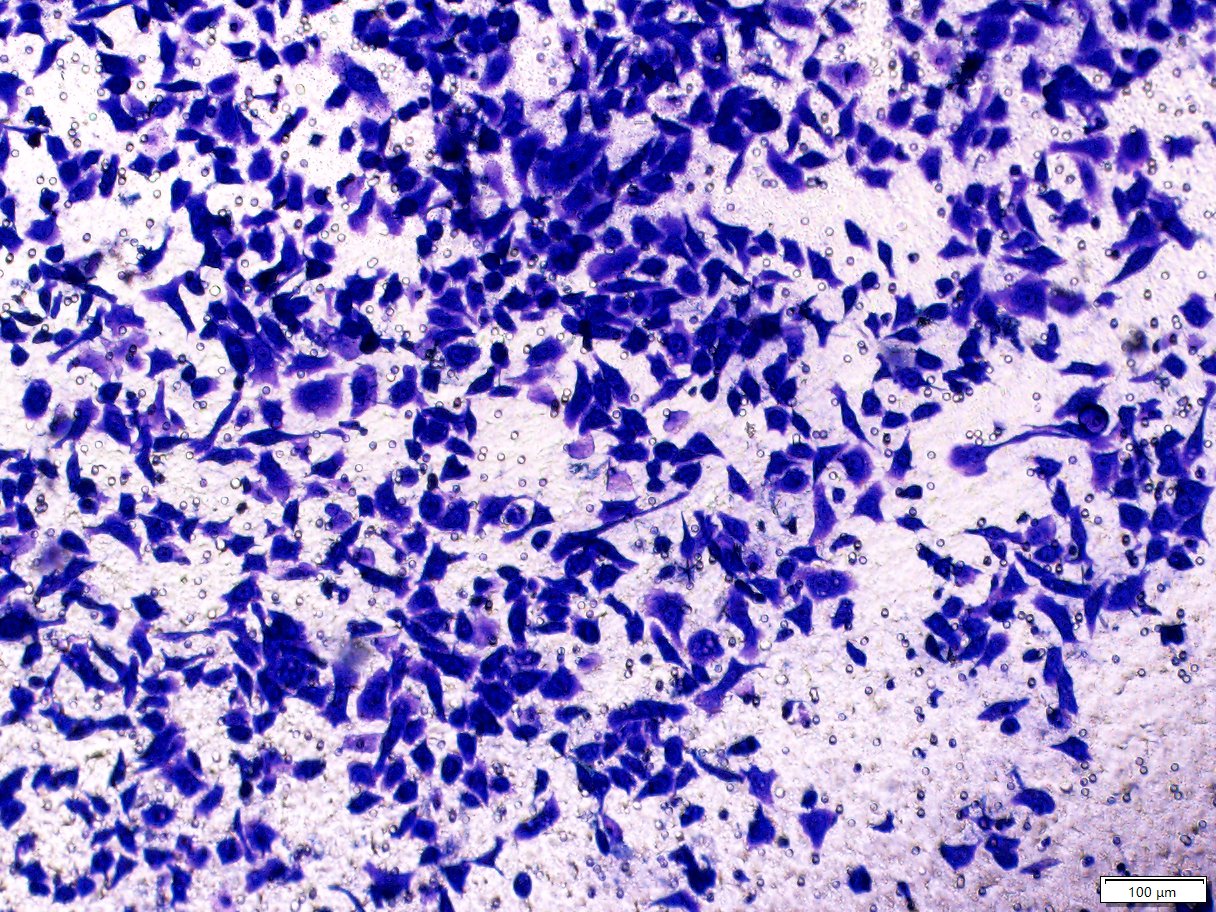

Supplement: Supplemental Information 8 [file peerj-cs-09-1651-s008.zip › Dataset 7/3-2.jpg]

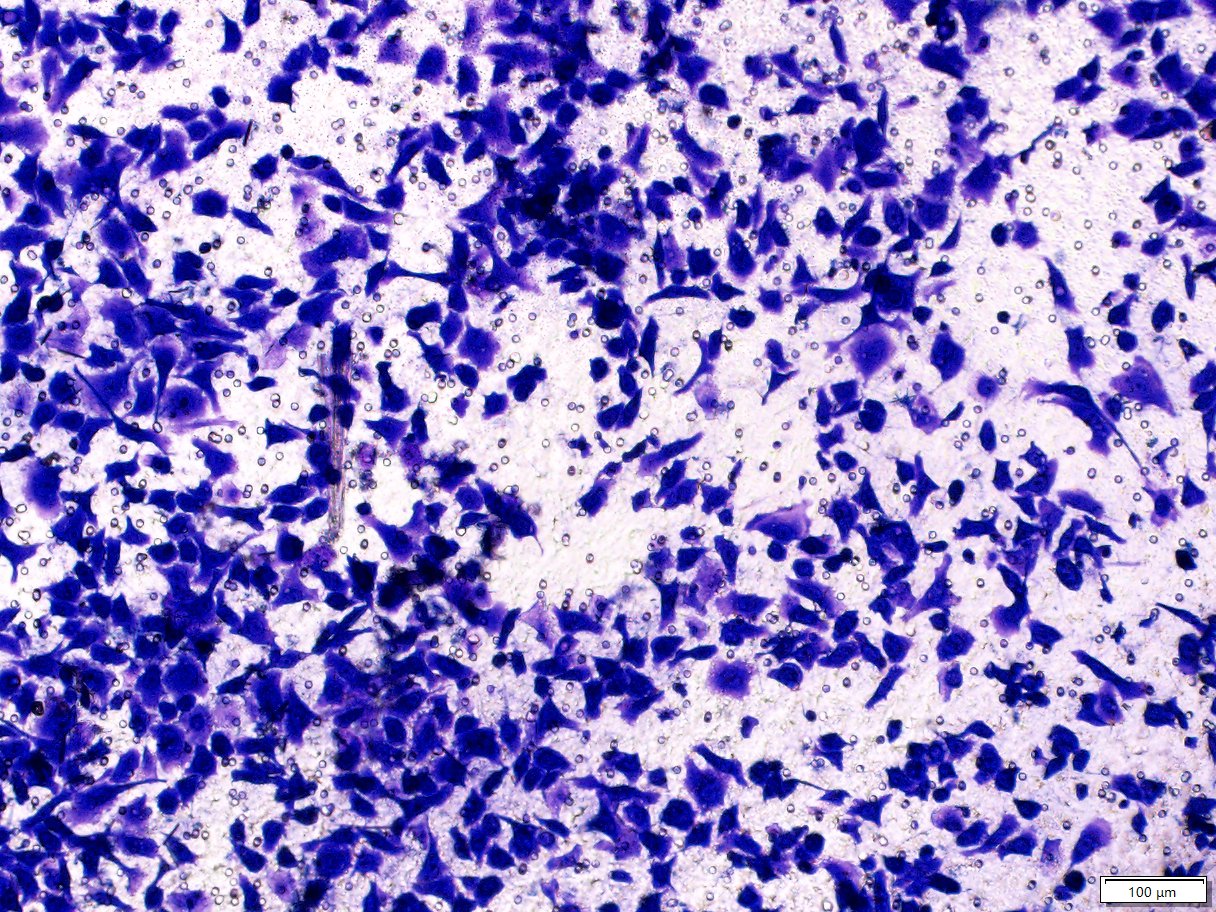

Supplement: Supplemental Information 8 [file peerj-cs-09-1651-s008.zip › Dataset 7/3-3.jpg]

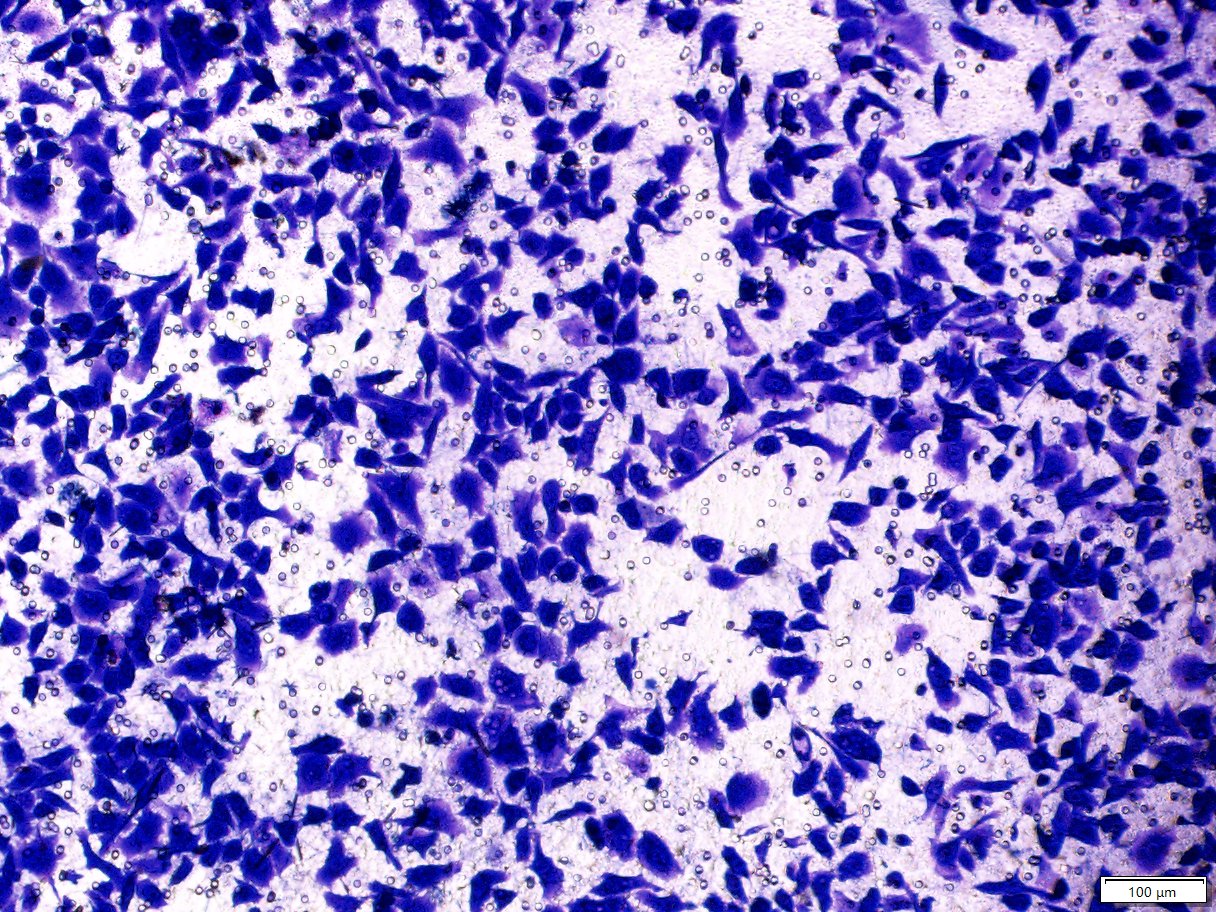

Supplement: Supplemental Information 8 [file peerj-cs-09-1651-s008.zip › Dataset 7/3-4.jpg]

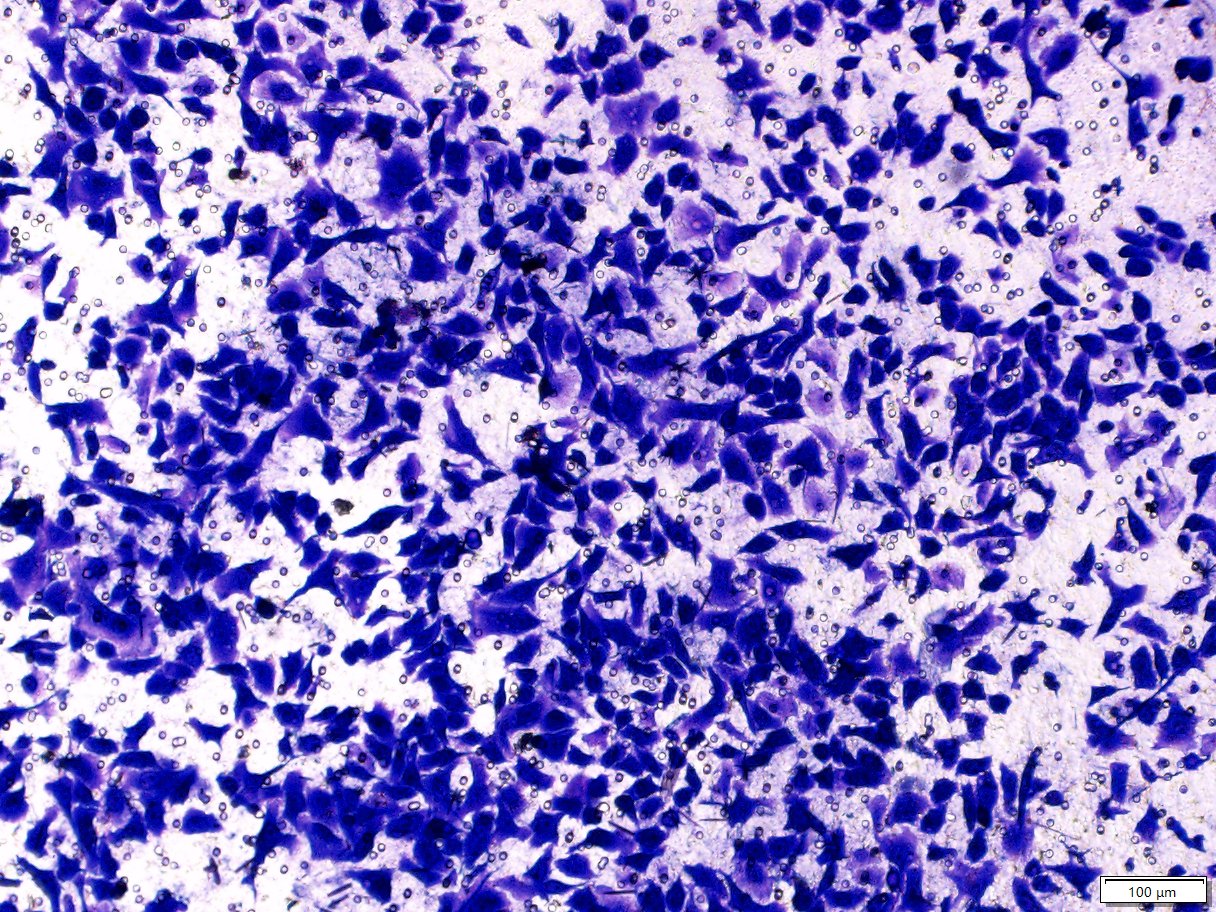

Supplement: Supplemental Information 8 [file peerj-cs-09-1651-s008.zip › Dataset 7/3-5.jpg]

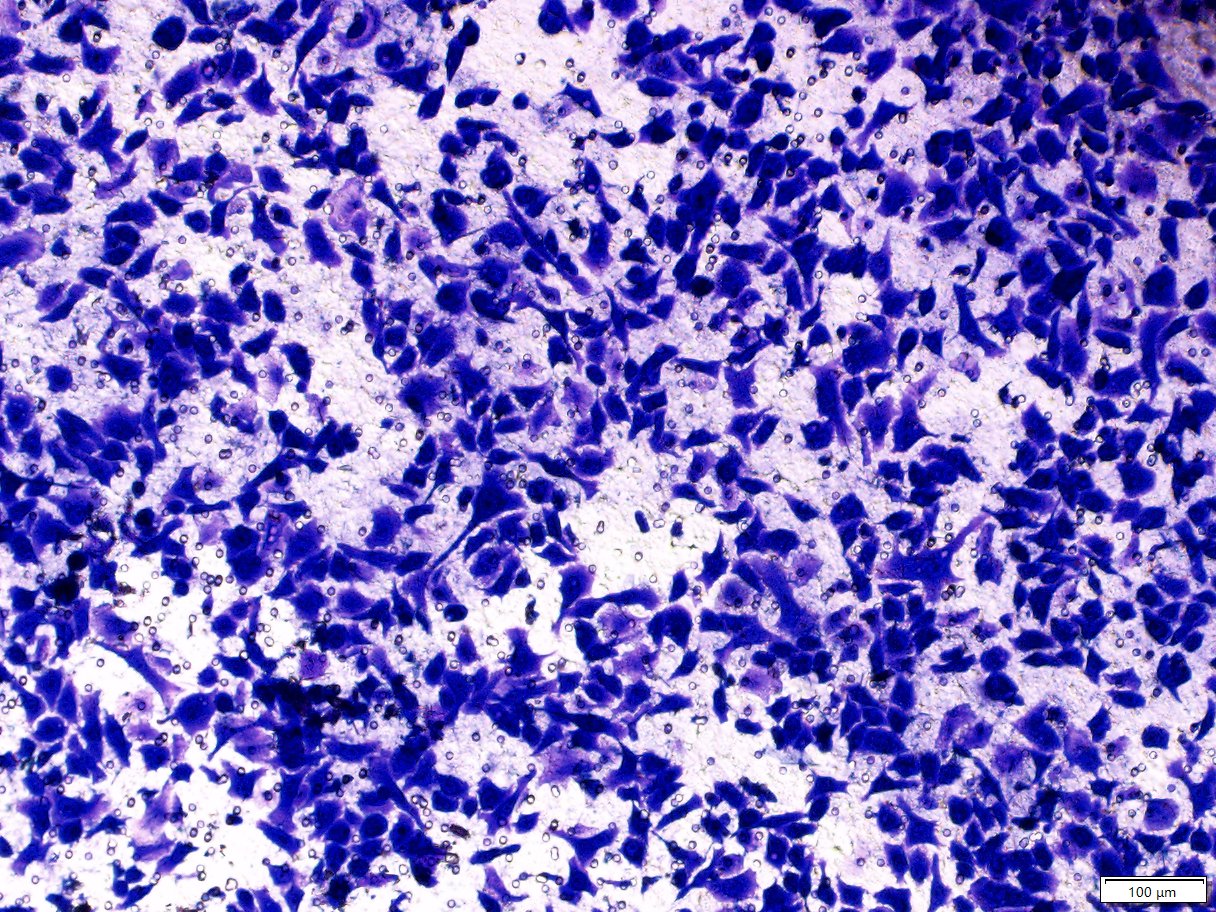

Supplement: Supplemental Information 8 [file peerj-cs-09-1651-s008.zip › Dataset 7/3-6.jpg]

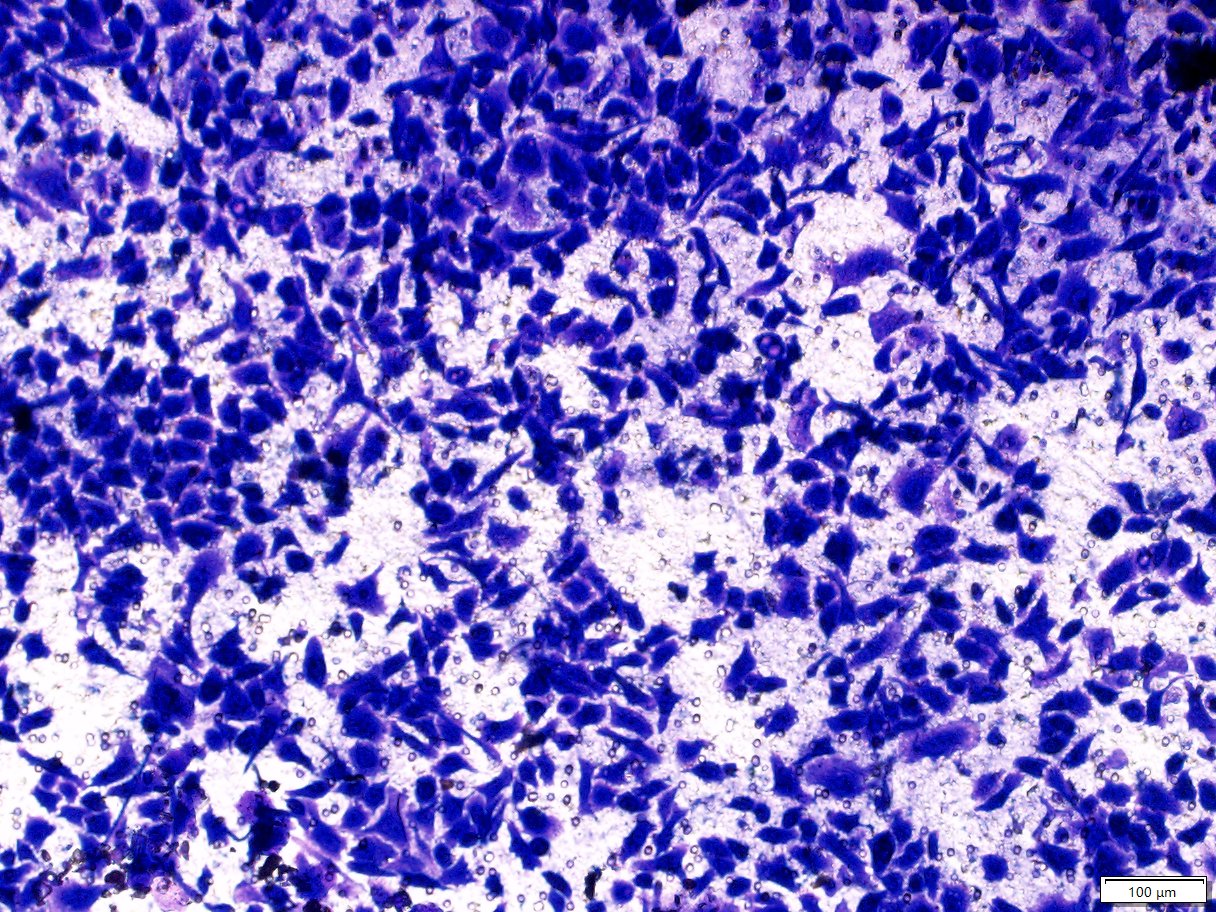

Supplement: Supplemental Information 8 [file peerj-cs-09-1651-s008.zip › Dataset 7/3-7.jpg]

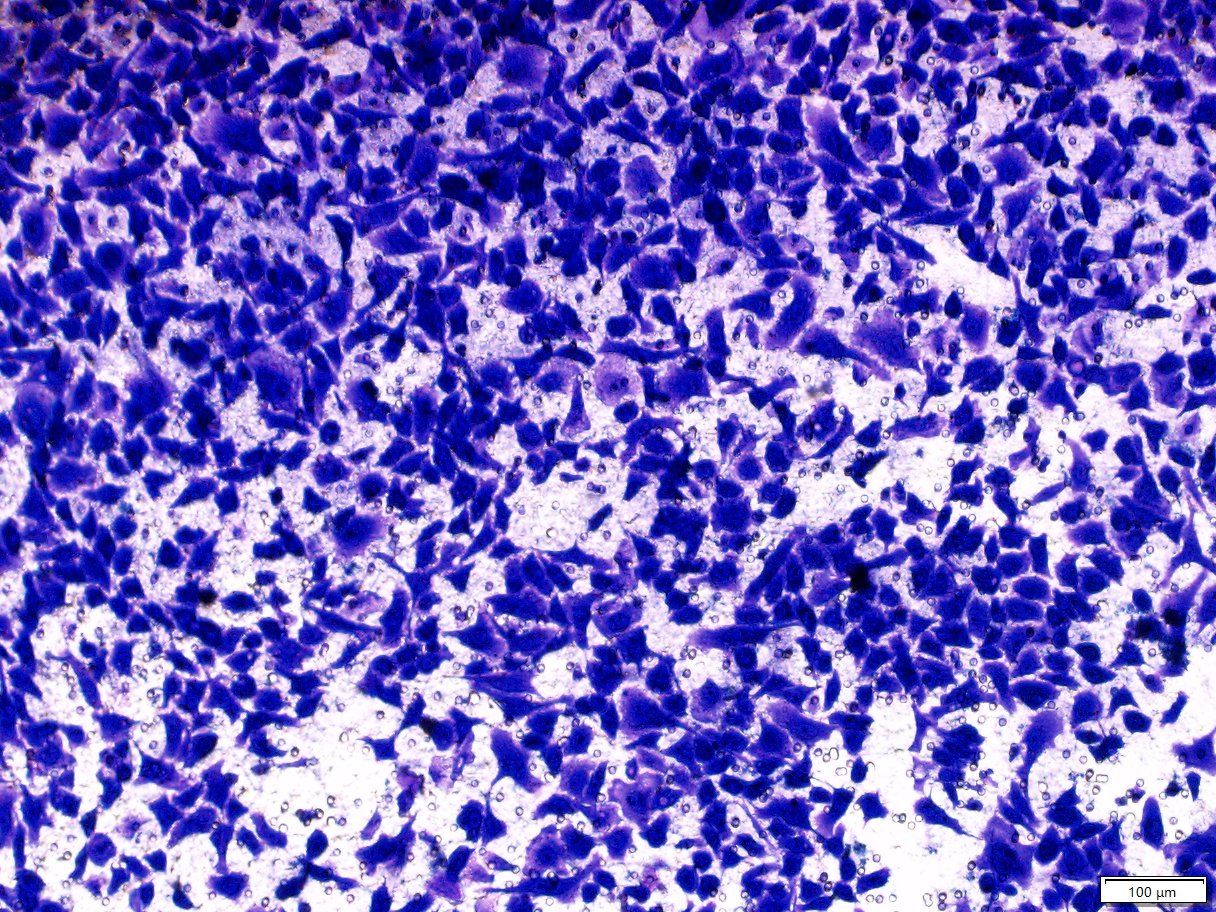

Supplement: Supplemental Information 8 [file peerj-cs-09-1651-s008.zip › Dataset 7/3-8.jpg]

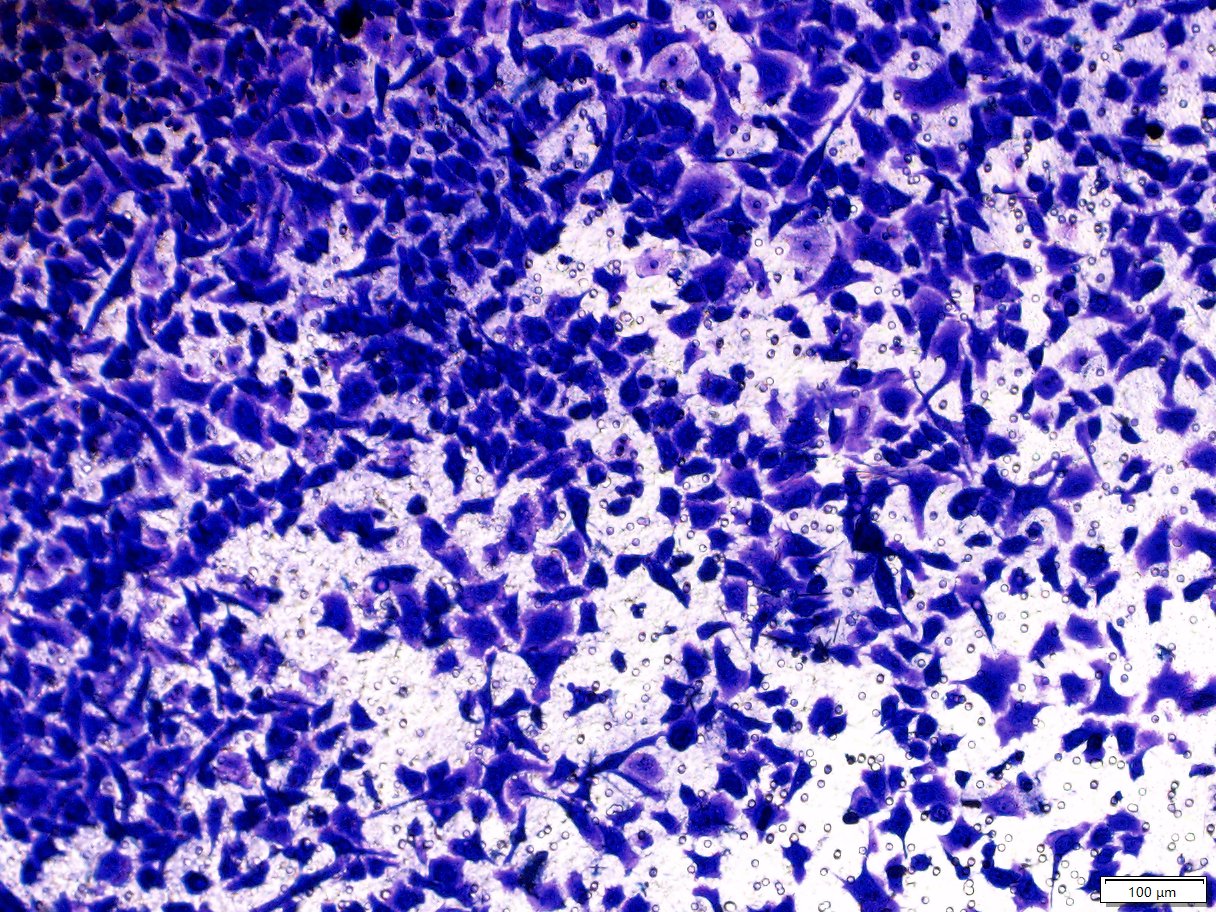

Supplement: Supplemental Information 8 [file peerj-cs-09-1651-s008.zip › Dataset 7/3-9.jpg]

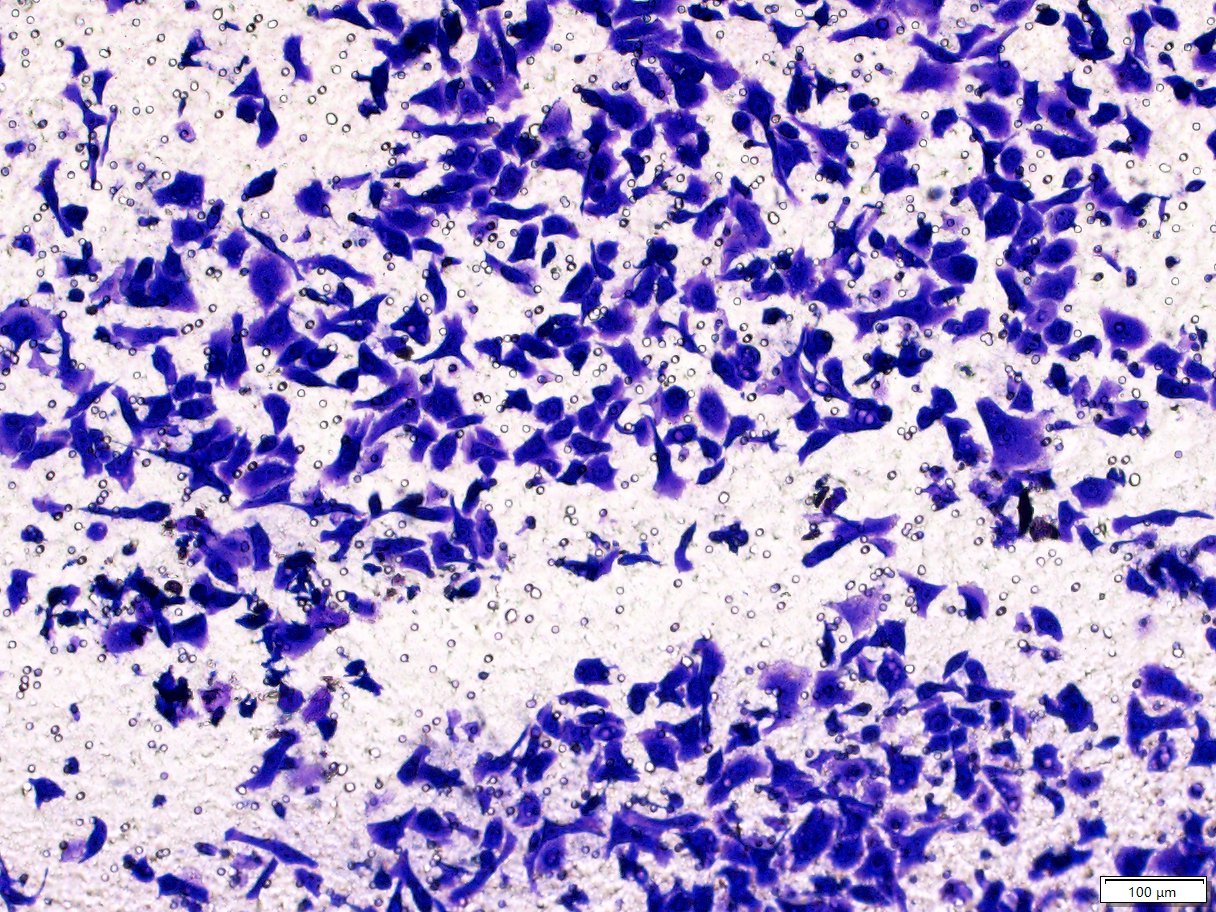

Supplement: Supplemental Information 8 [file peerj-cs-09-1651-s008.zip › Dataset 7/4+1.jpg]

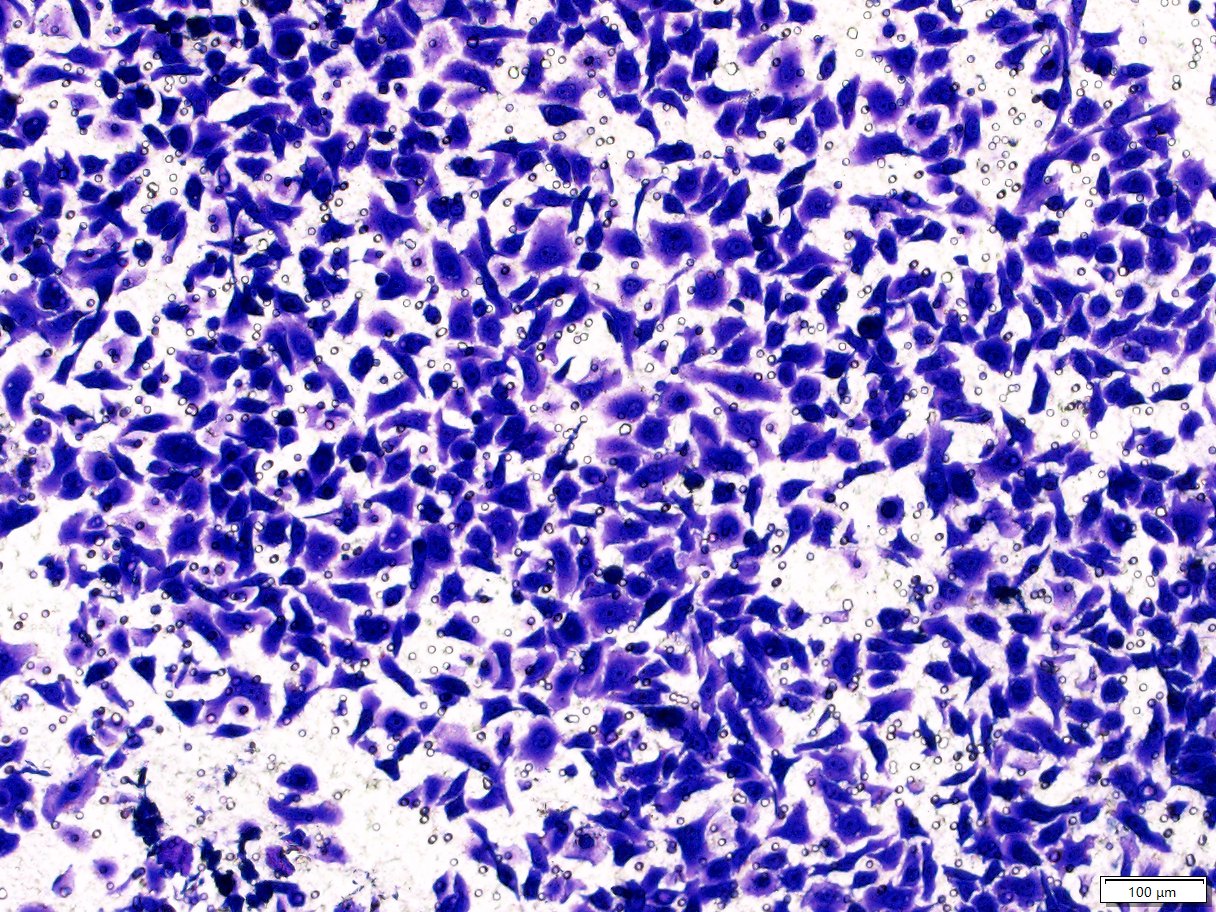

Supplement: Supplemental Information 8 [file peerj-cs-09-1651-s008.zip › Dataset 7/4+10.jpg]

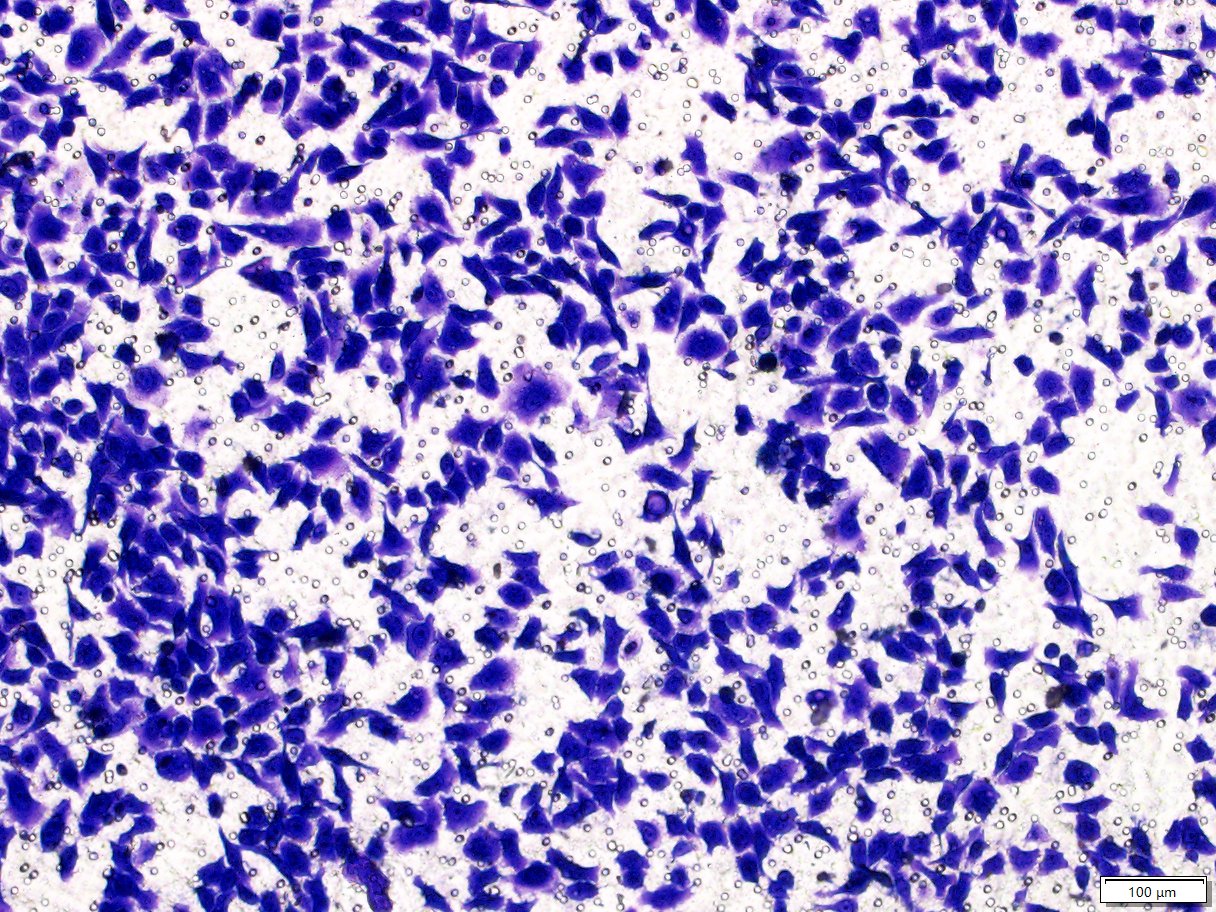

Supplement: Supplemental Information 8 [file peerj-cs-09-1651-s008.zip › Dataset 7/4+11.jpg]

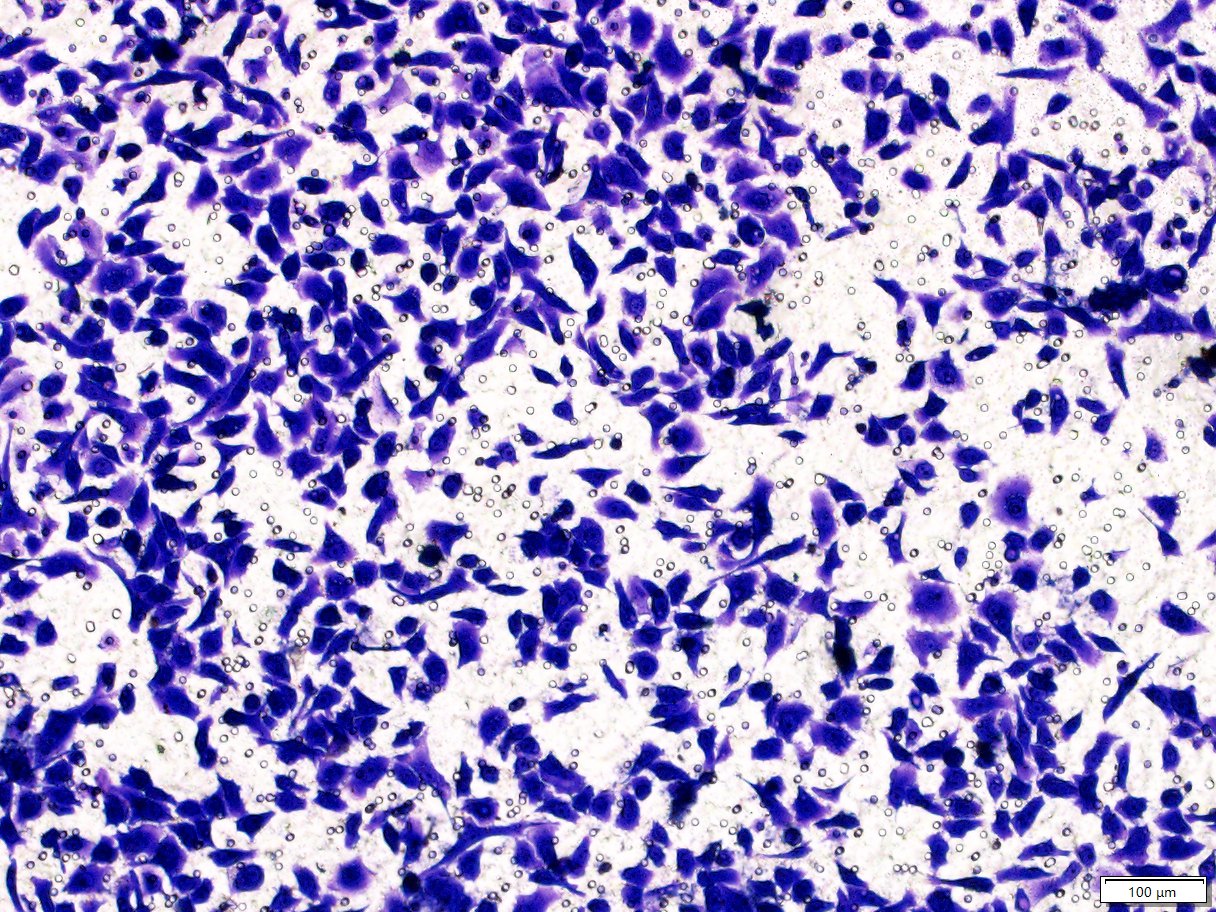

Supplement: Supplemental Information 8 [file peerj-cs-09-1651-s008.zip › Dataset 7/4+12.jpg]

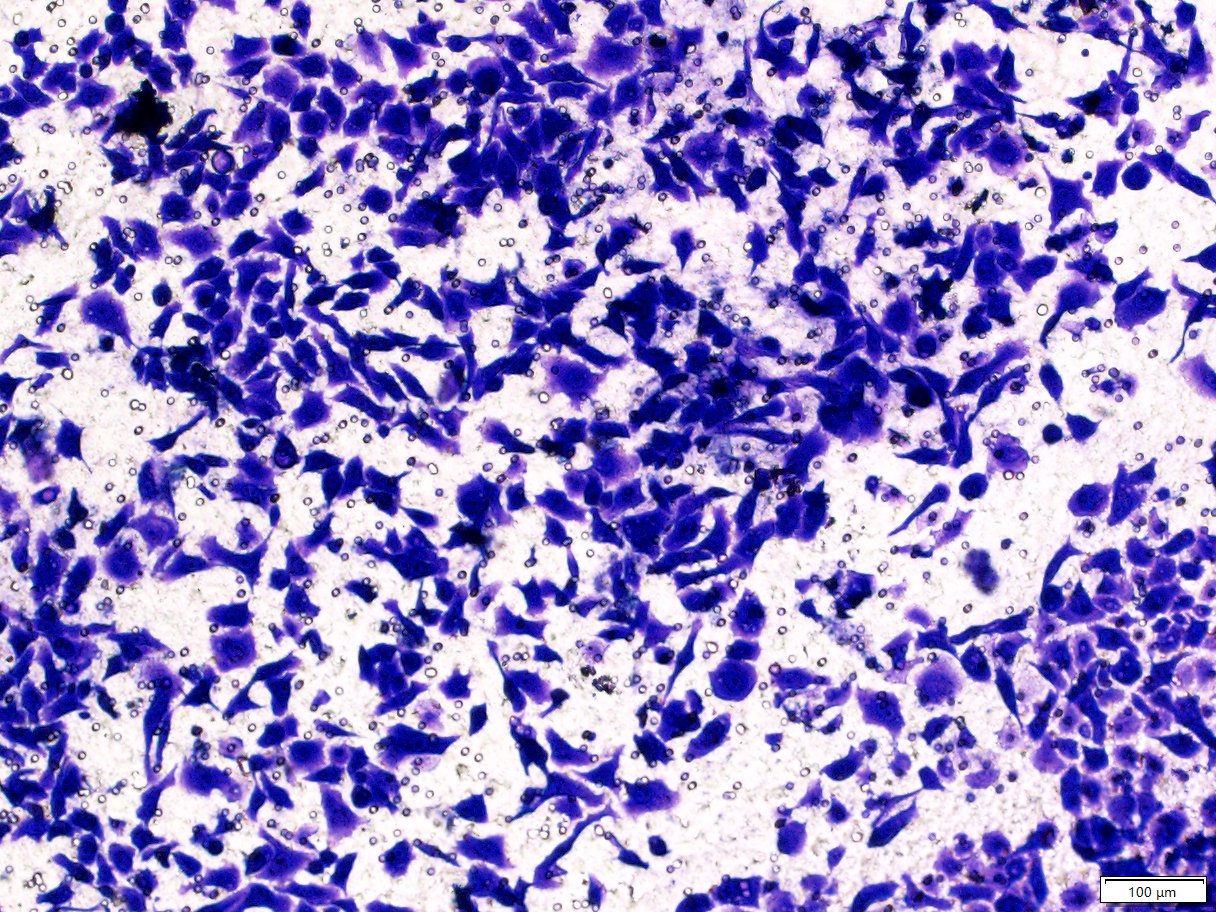

Supplement: Supplemental Information 8 [file peerj-cs-09-1651-s008.zip › Dataset 7/4+2.jpg]

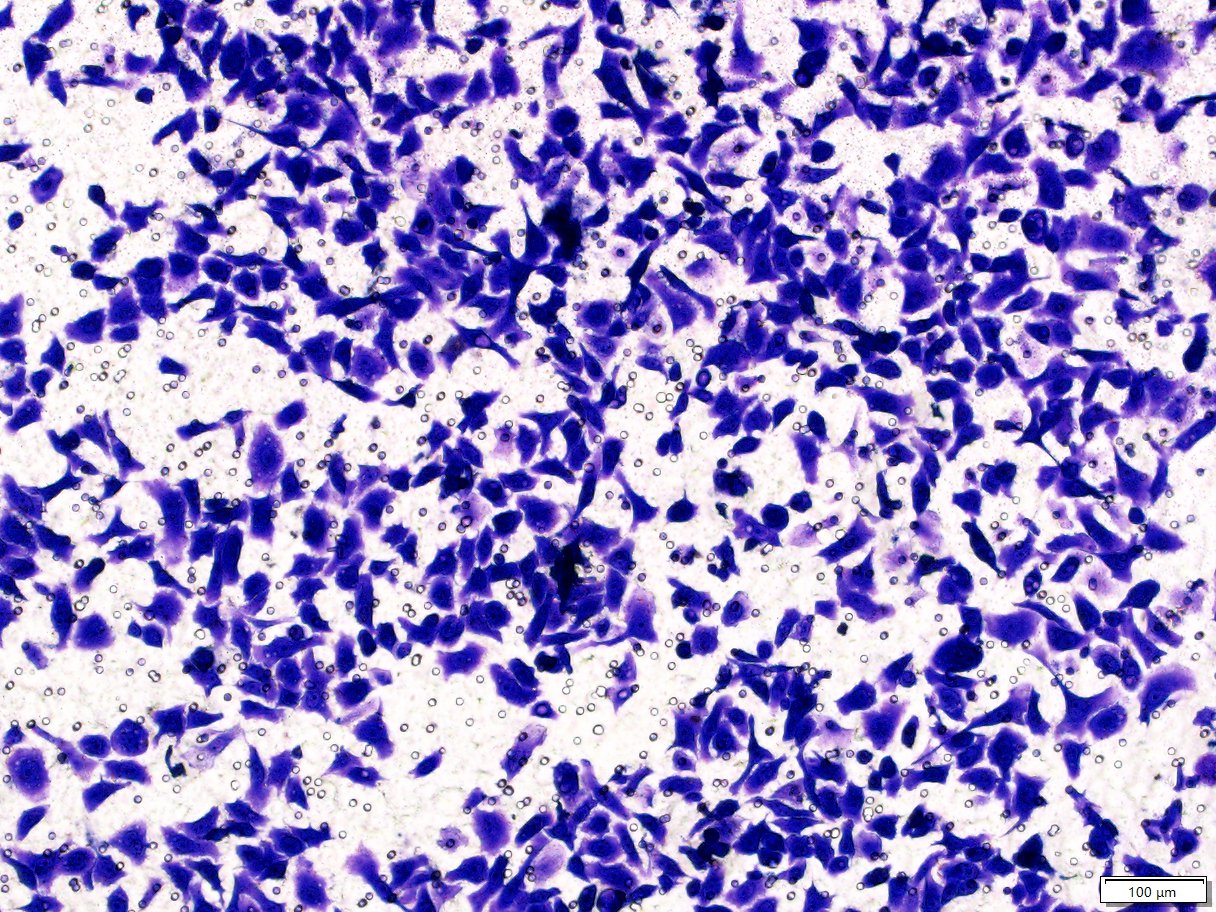

Supplement: Supplemental Information 8 [file peerj-cs-09-1651-s008.zip › Dataset 7/4+3.jpg]

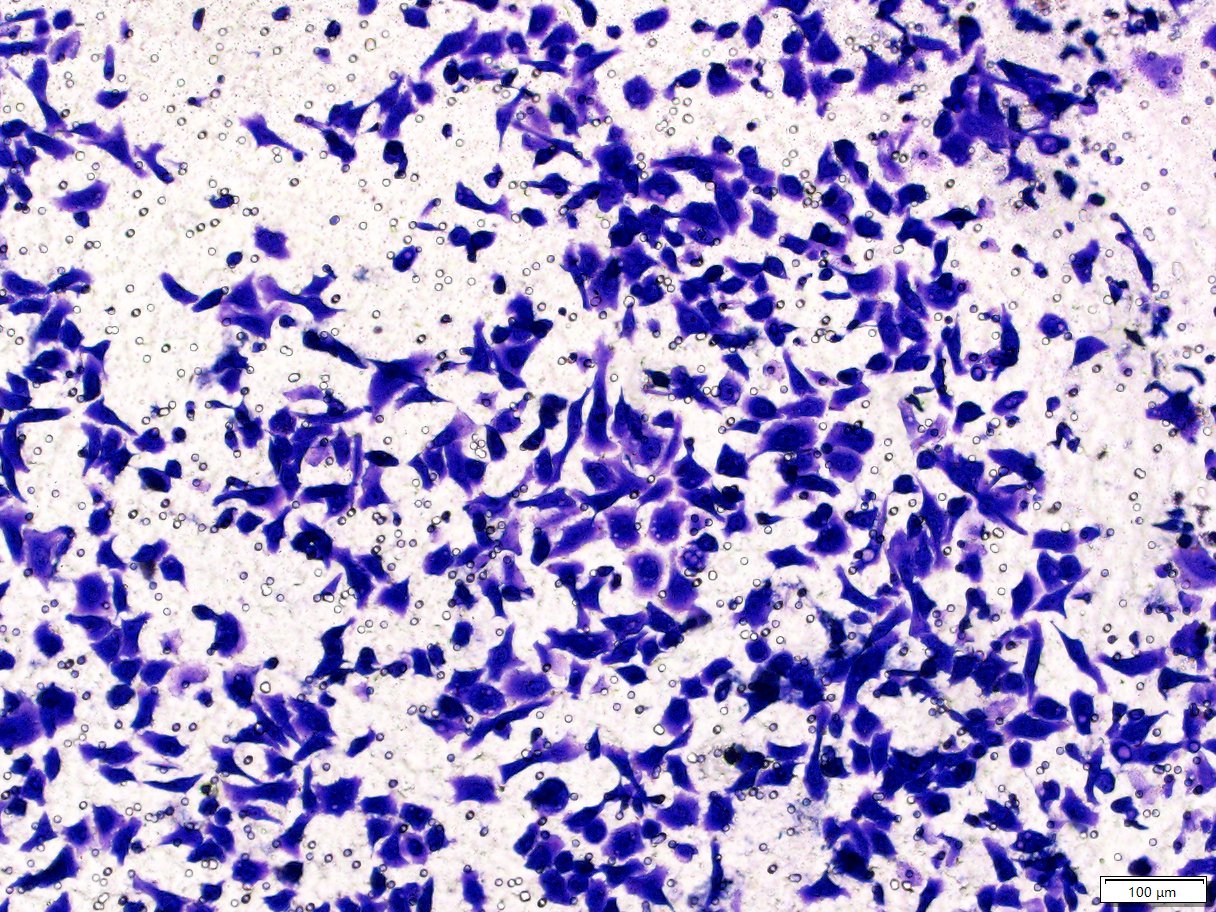

Supplement: Supplemental Information 8 [file peerj-cs-09-1651-s008.zip › Dataset 7/4+4.jpg]

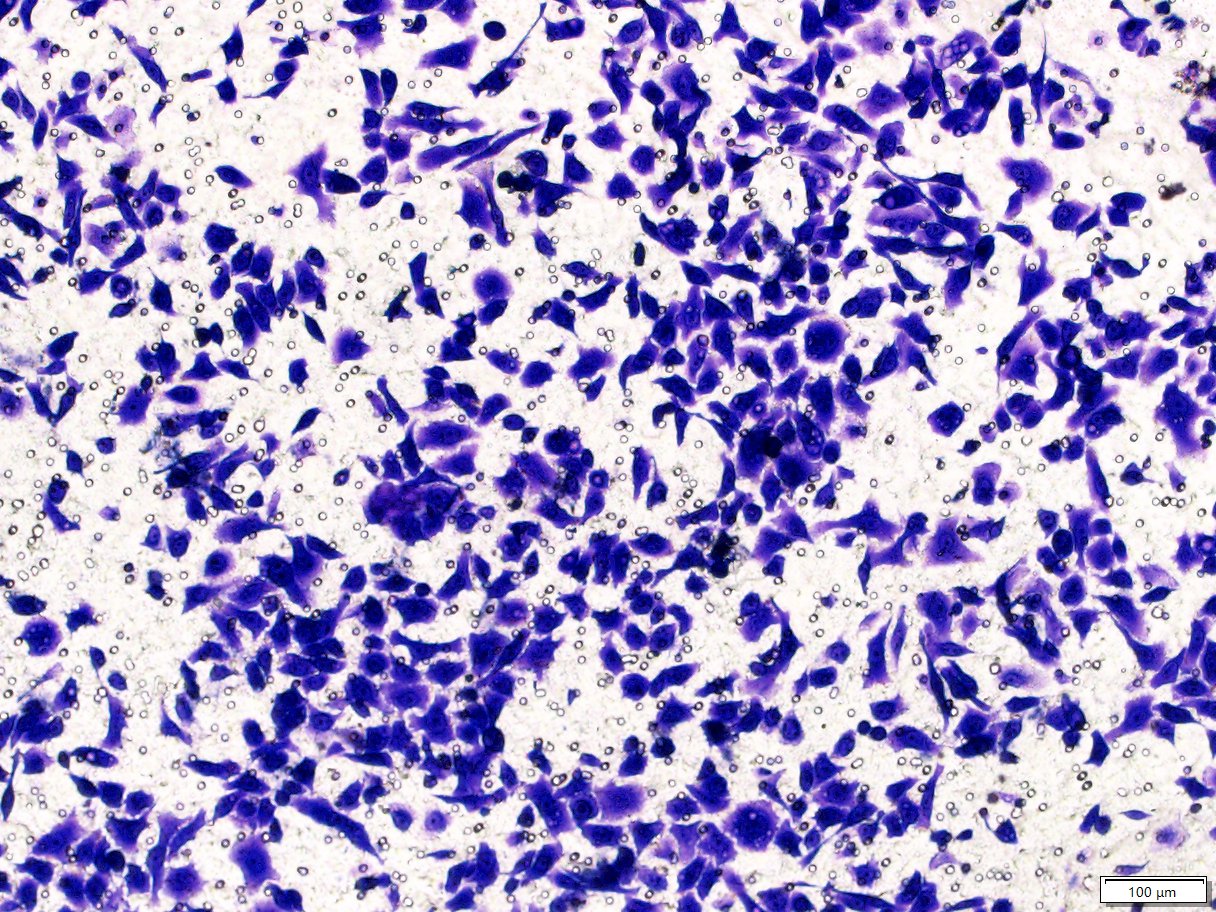

Supplement: Supplemental Information 8 [file peerj-cs-09-1651-s008.zip › Dataset 7/4+5.jpg]

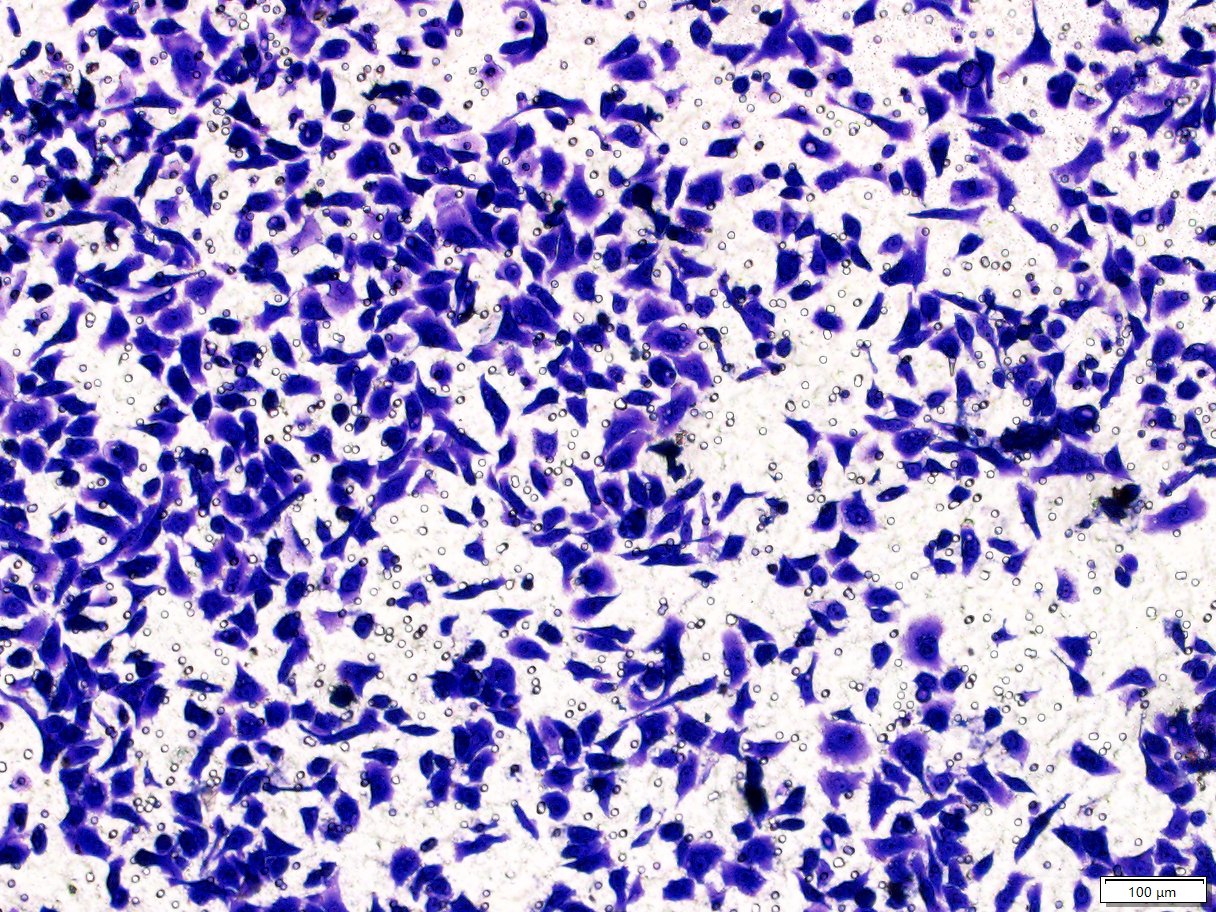

Supplement: Supplemental Information 8 [file peerj-cs-09-1651-s008.zip › Dataset 7/4+6.jpg]

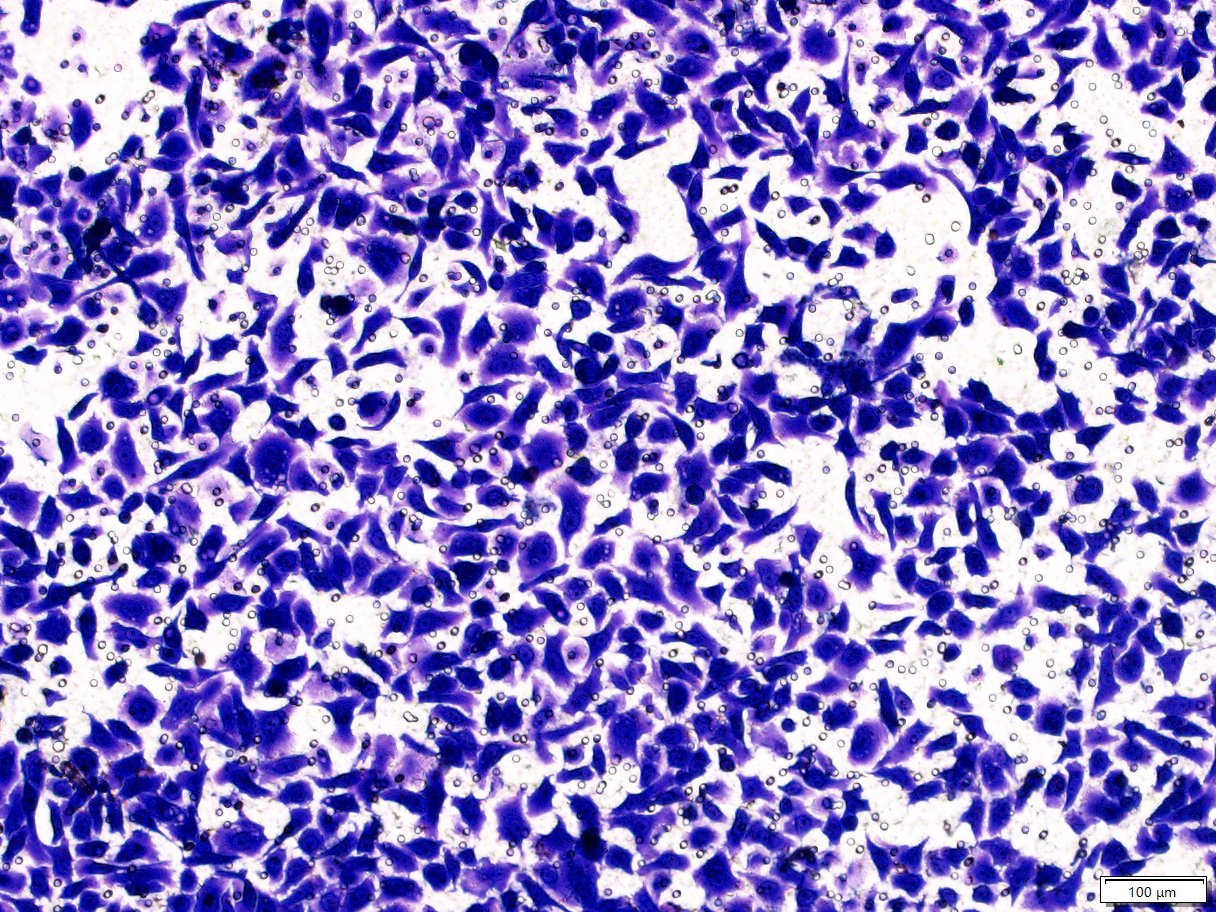

Supplement: Supplemental Information 8 [file peerj-cs-09-1651-s008.zip › Dataset 7/4+7.jpg]

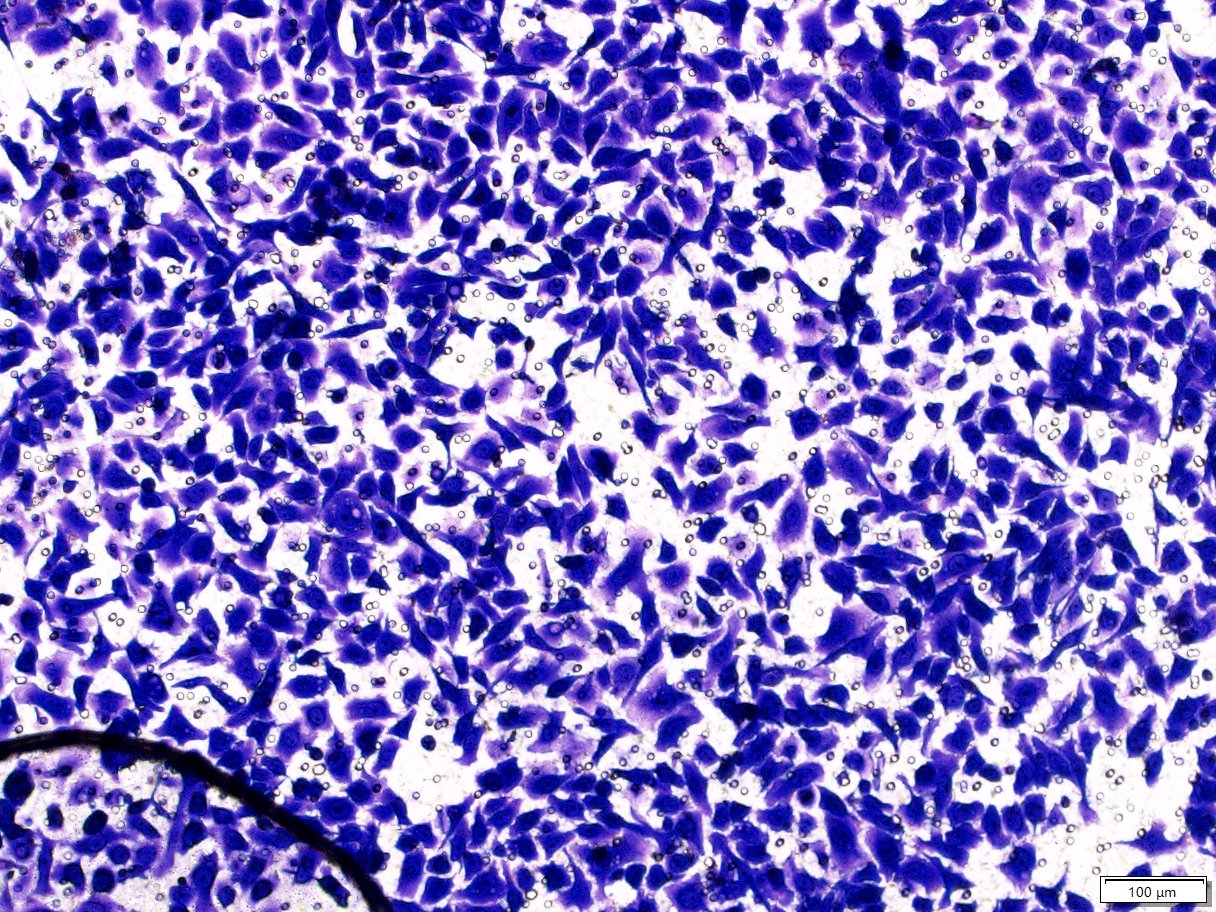

Supplement: Supplemental Information 8 [file peerj-cs-09-1651-s008.zip › Dataset 7/4+8.jpg]

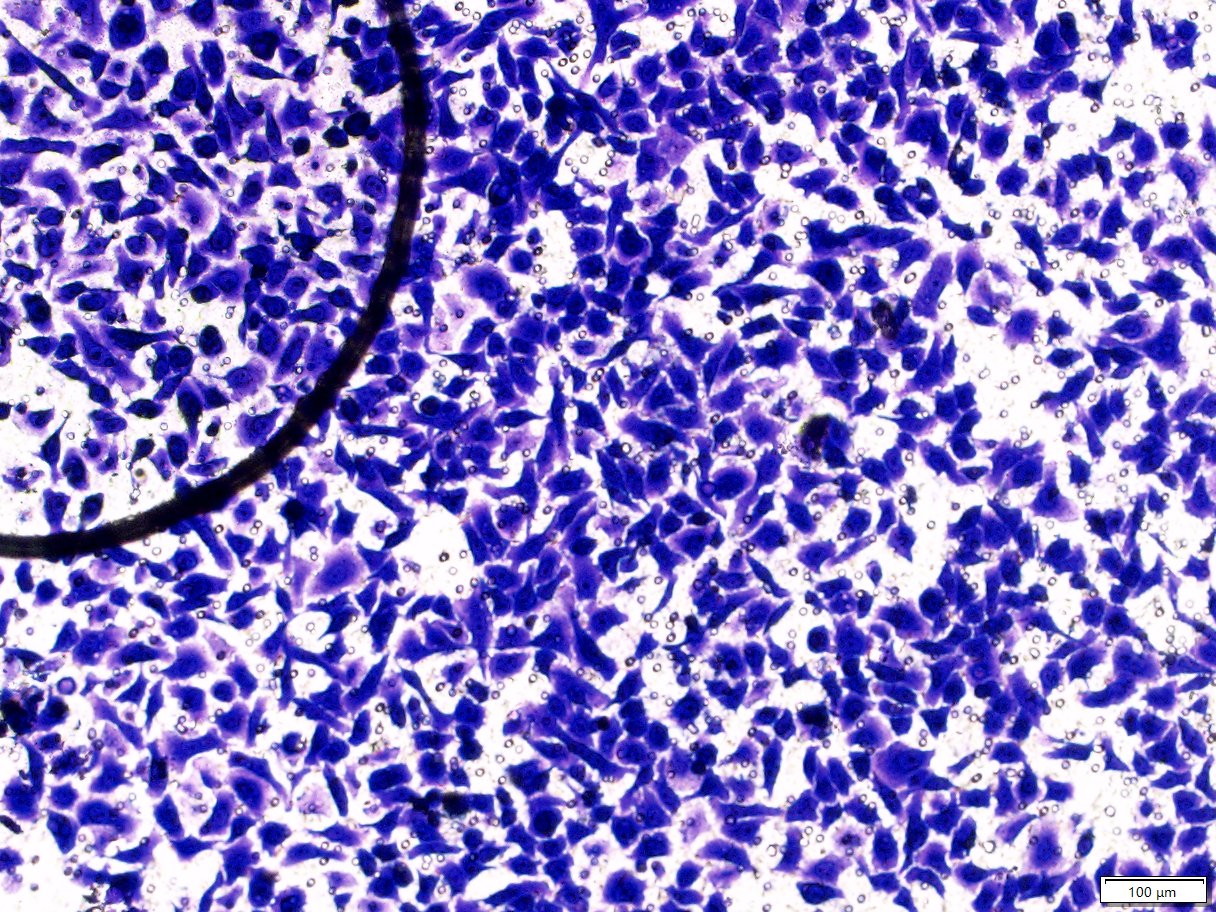

Supplement: Supplemental Information 8 [file peerj-cs-09-1651-s008.zip › Dataset 7/4+9.jpg]

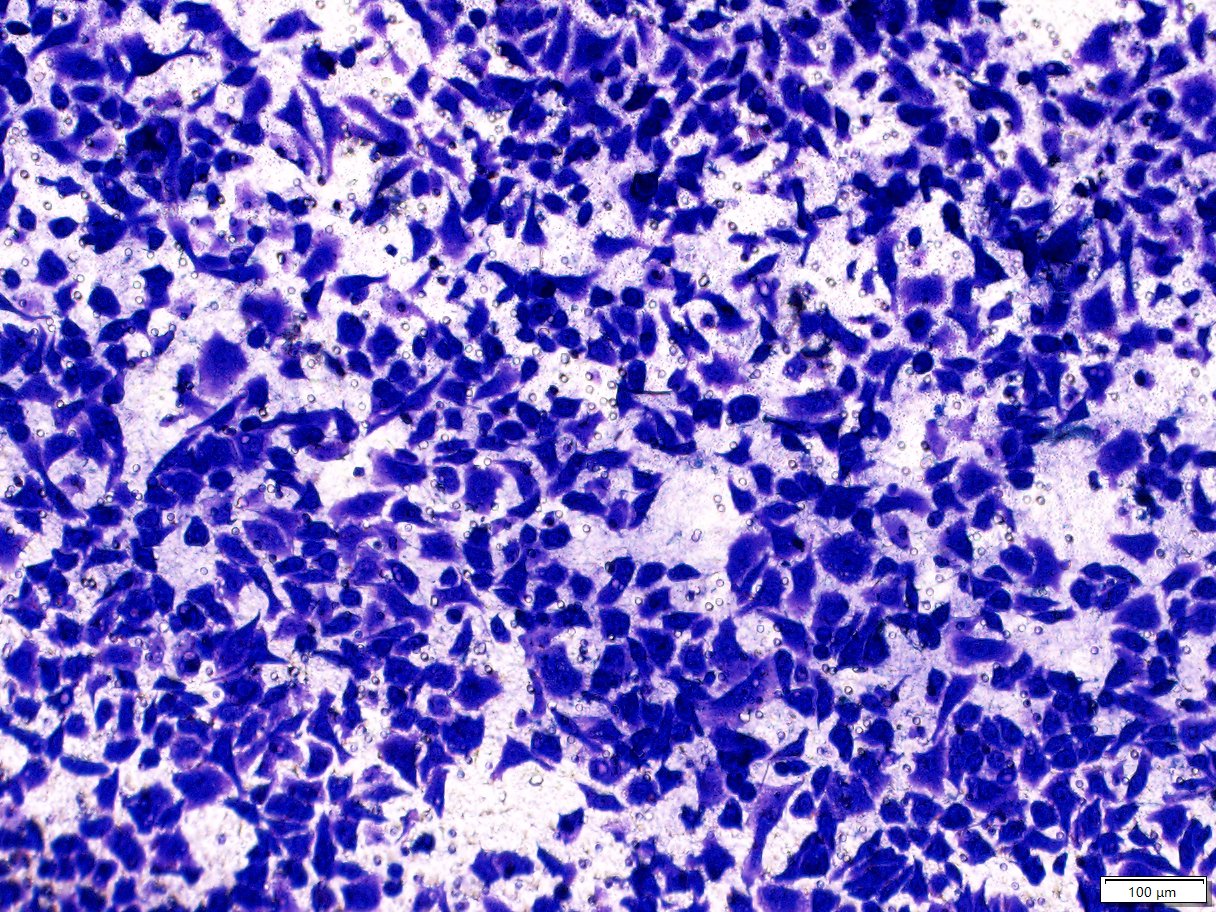

Supplement: Supplemental Information 8 [file peerj-cs-09-1651-s008.zip › Dataset 7/4-1.jpg]

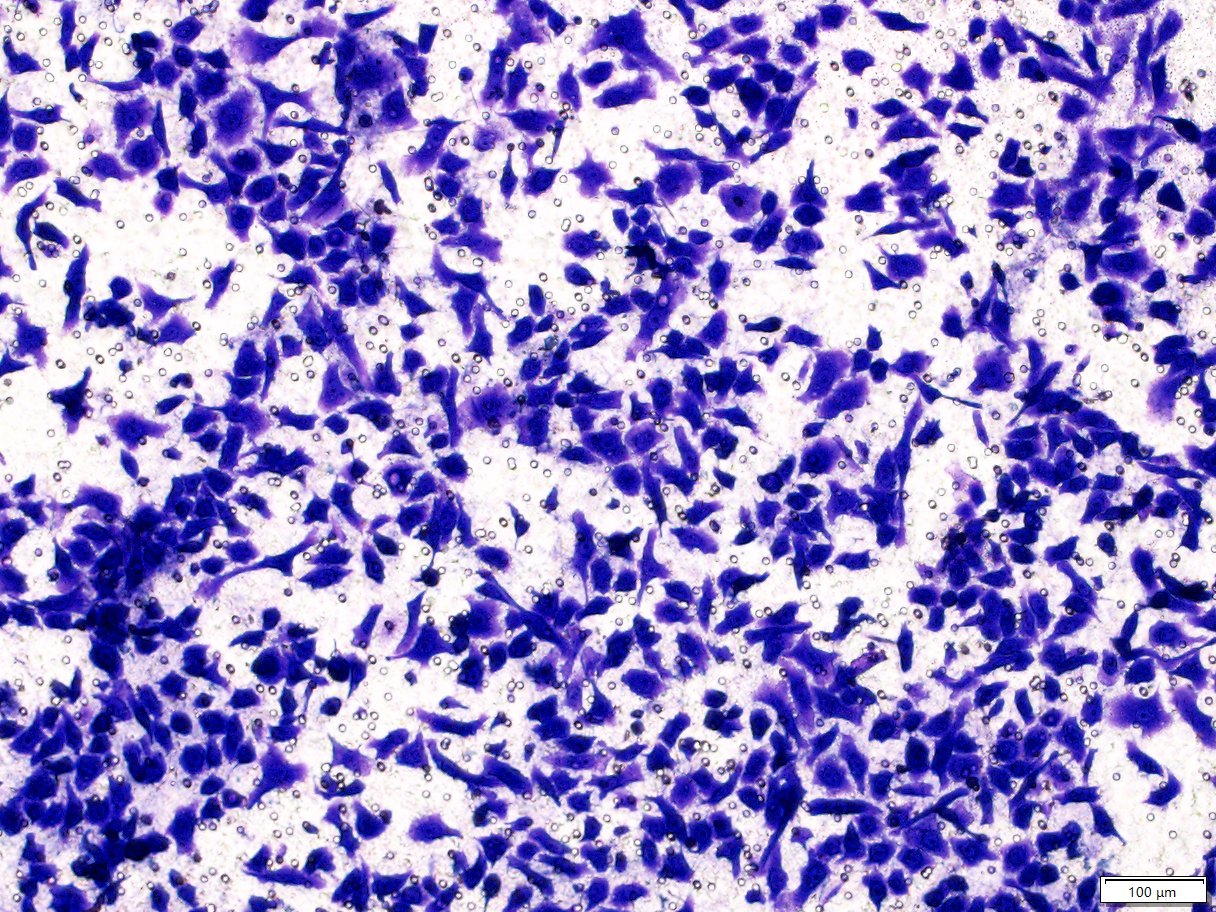

Supplement: Supplemental Information 8 [file peerj-cs-09-1651-s008.zip › Dataset 7/4-10.jpg]

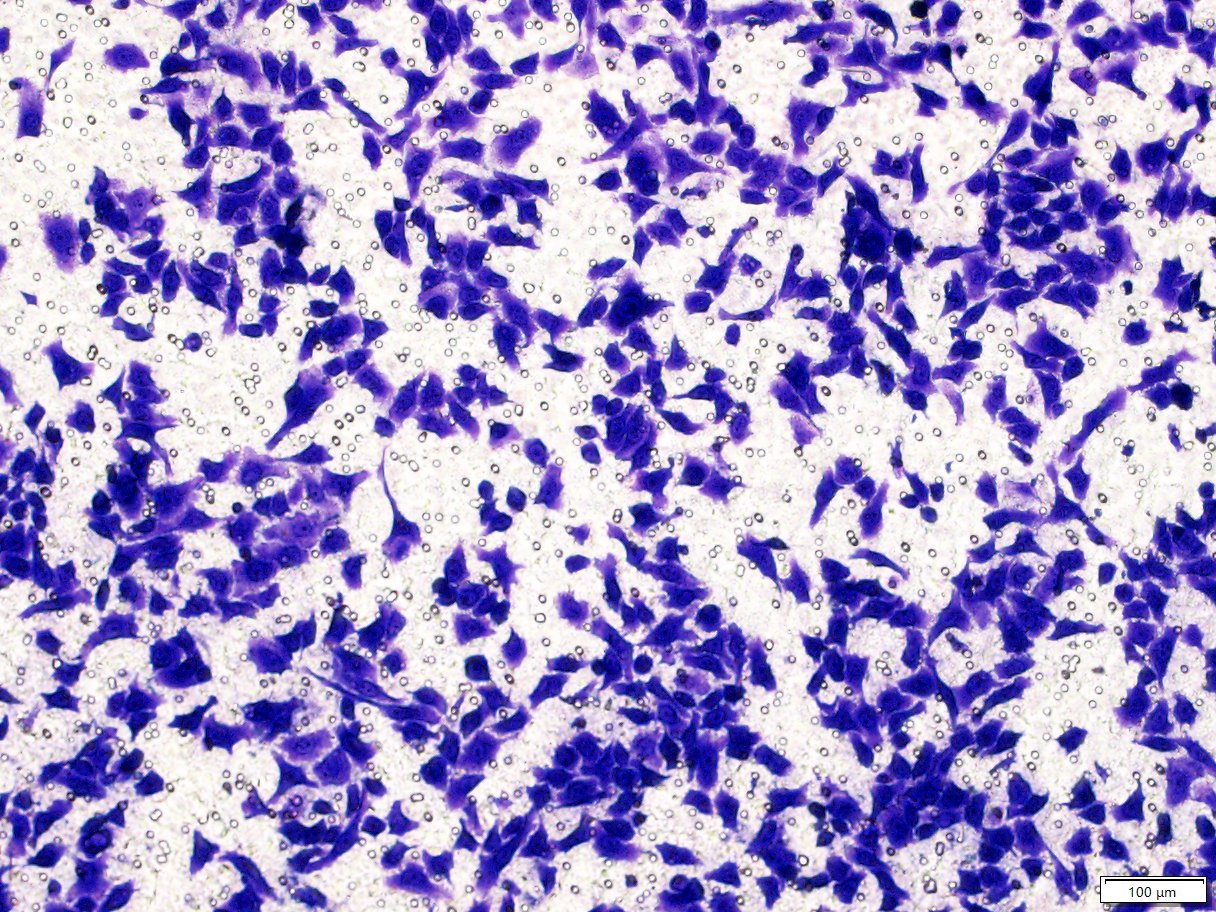

Supplement: Supplemental Information 8 [file peerj-cs-09-1651-s008.zip › Dataset 7/4-11.jpg]

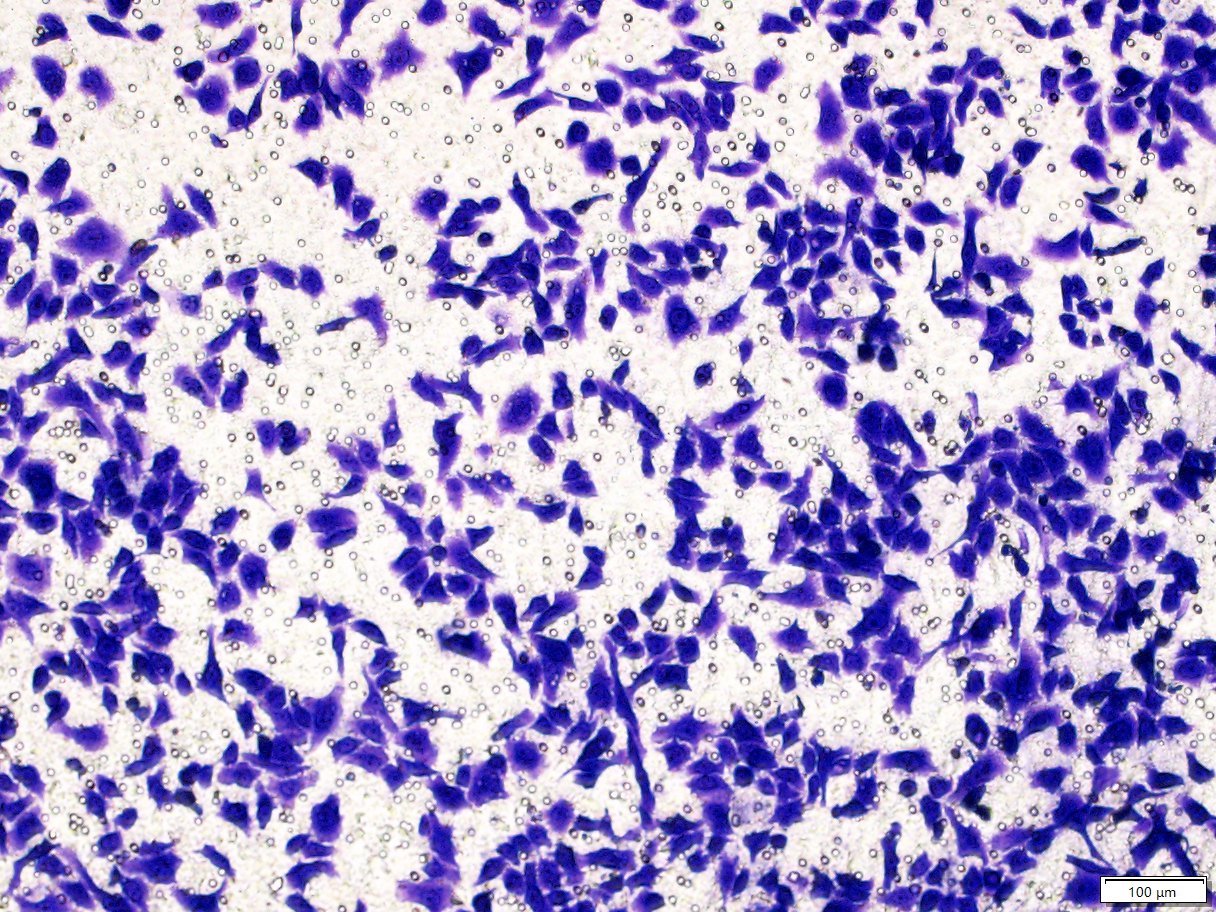

Supplement: Supplemental Information 8 [file peerj-cs-09-1651-s008.zip › Dataset 7/4-12.jpg]

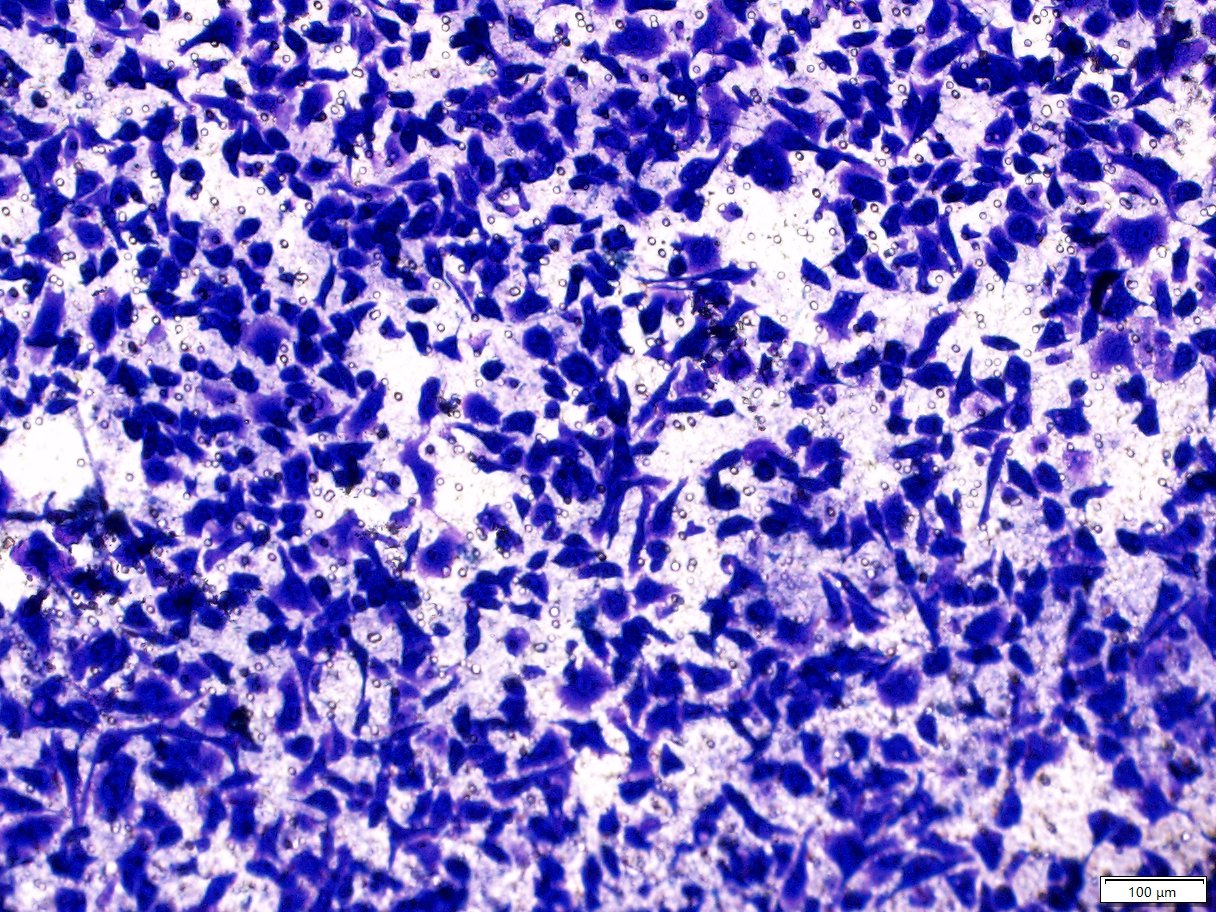

Supplement: Supplemental Information 8 [file peerj-cs-09-1651-s008.zip › Dataset 7/4-2.jpg]

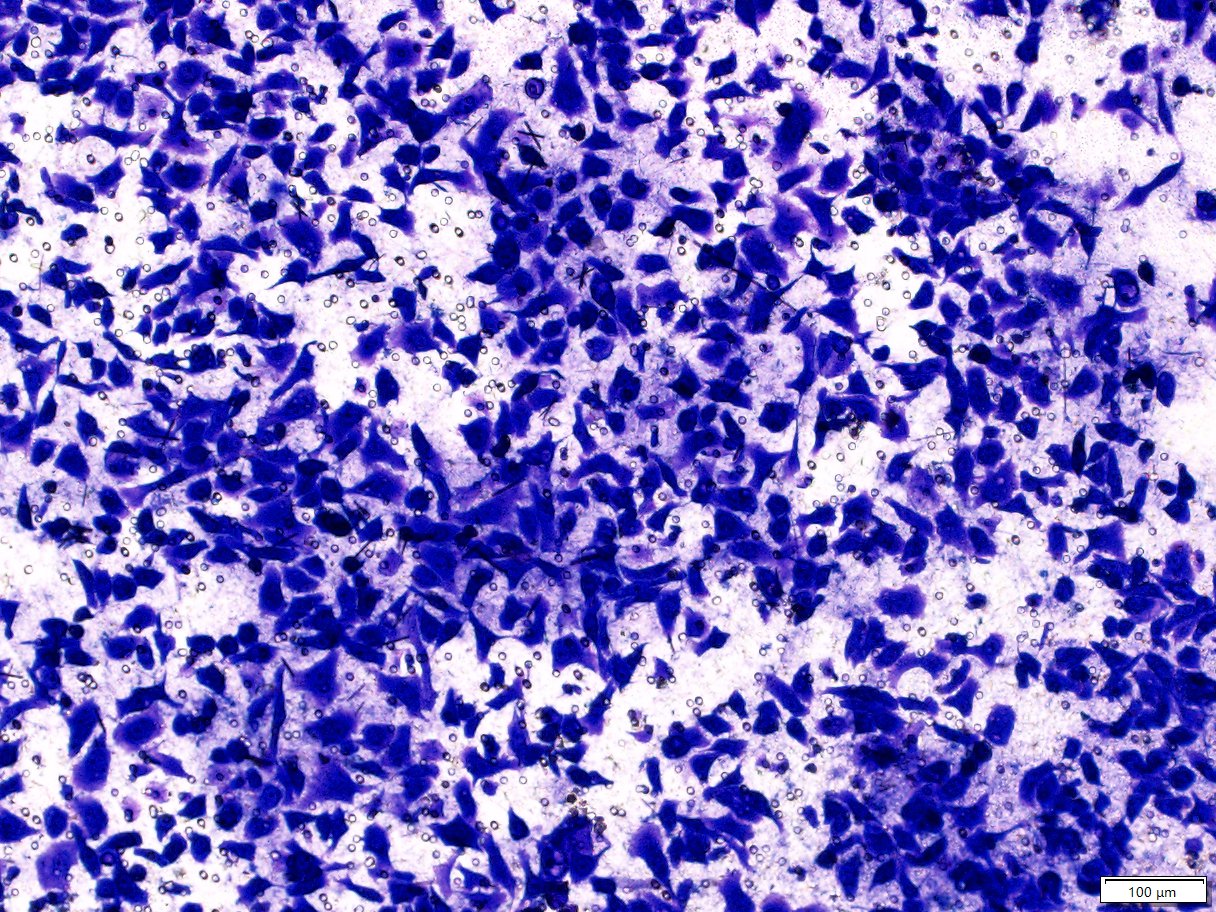

Supplement: Supplemental Information 8 [file peerj-cs-09-1651-s008.zip › Dataset 7/4-3.jpg]

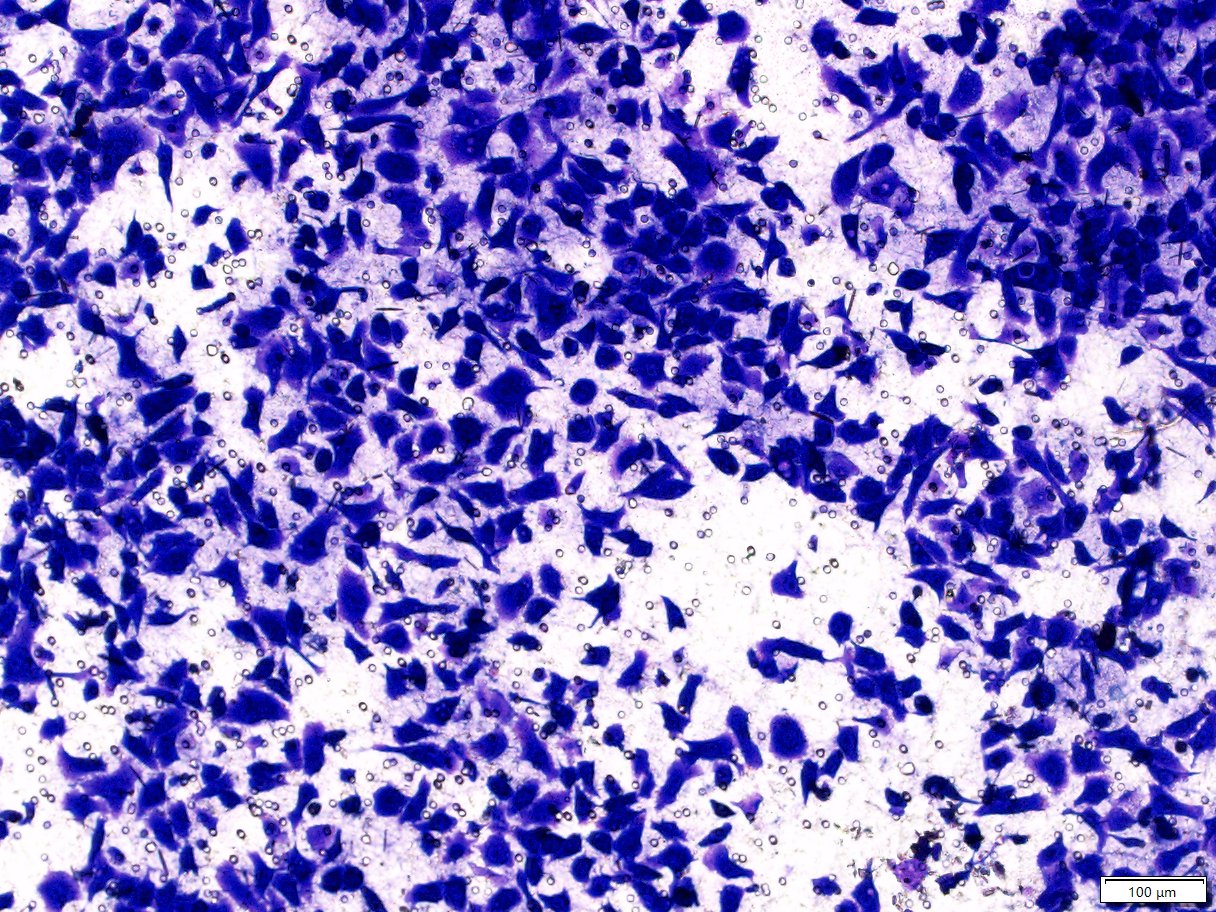

Supplement: Supplemental Information 8 [file peerj-cs-09-1651-s008.zip › Dataset 7/4-4.jpg]

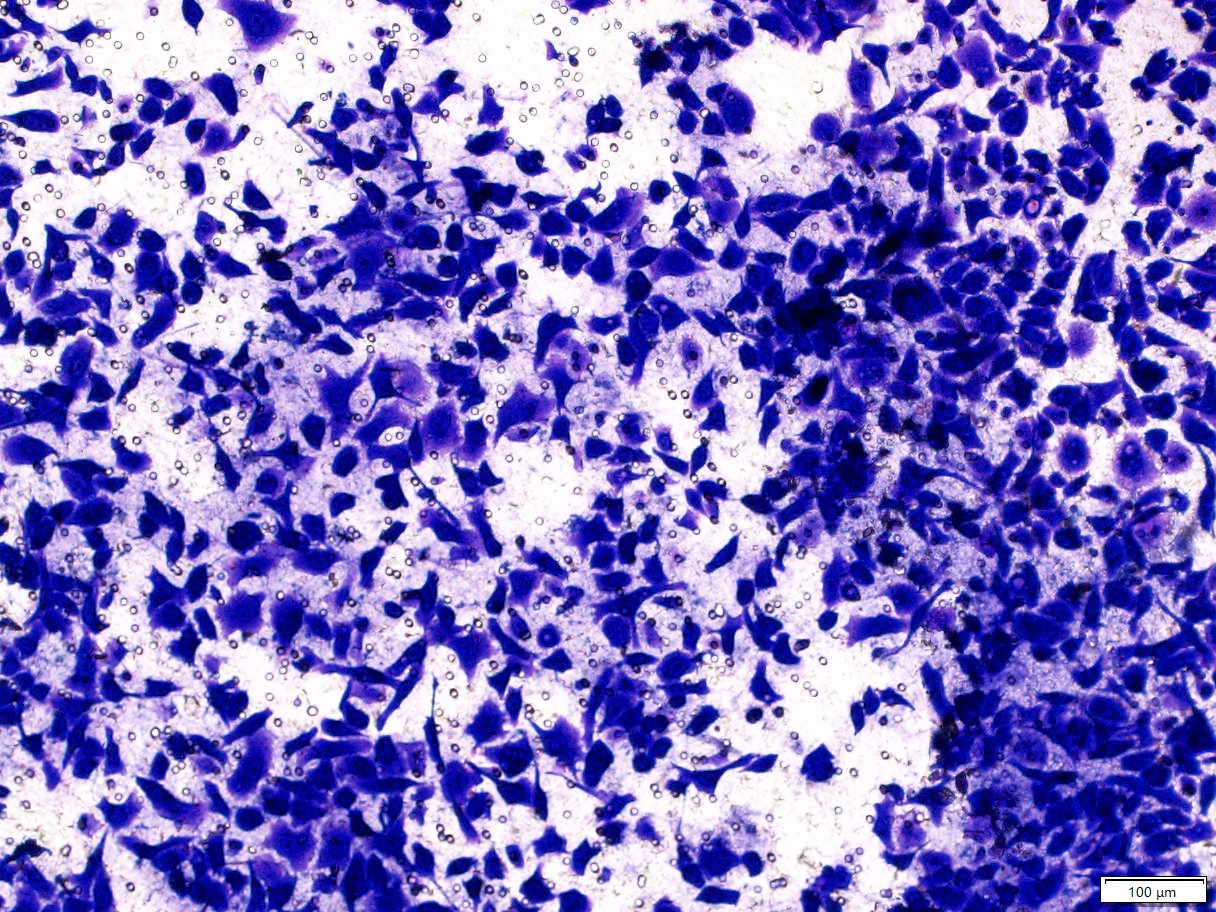

Supplement: Supplemental Information 8 [file peerj-cs-09-1651-s008.zip › Dataset 7/4-5.jpg]

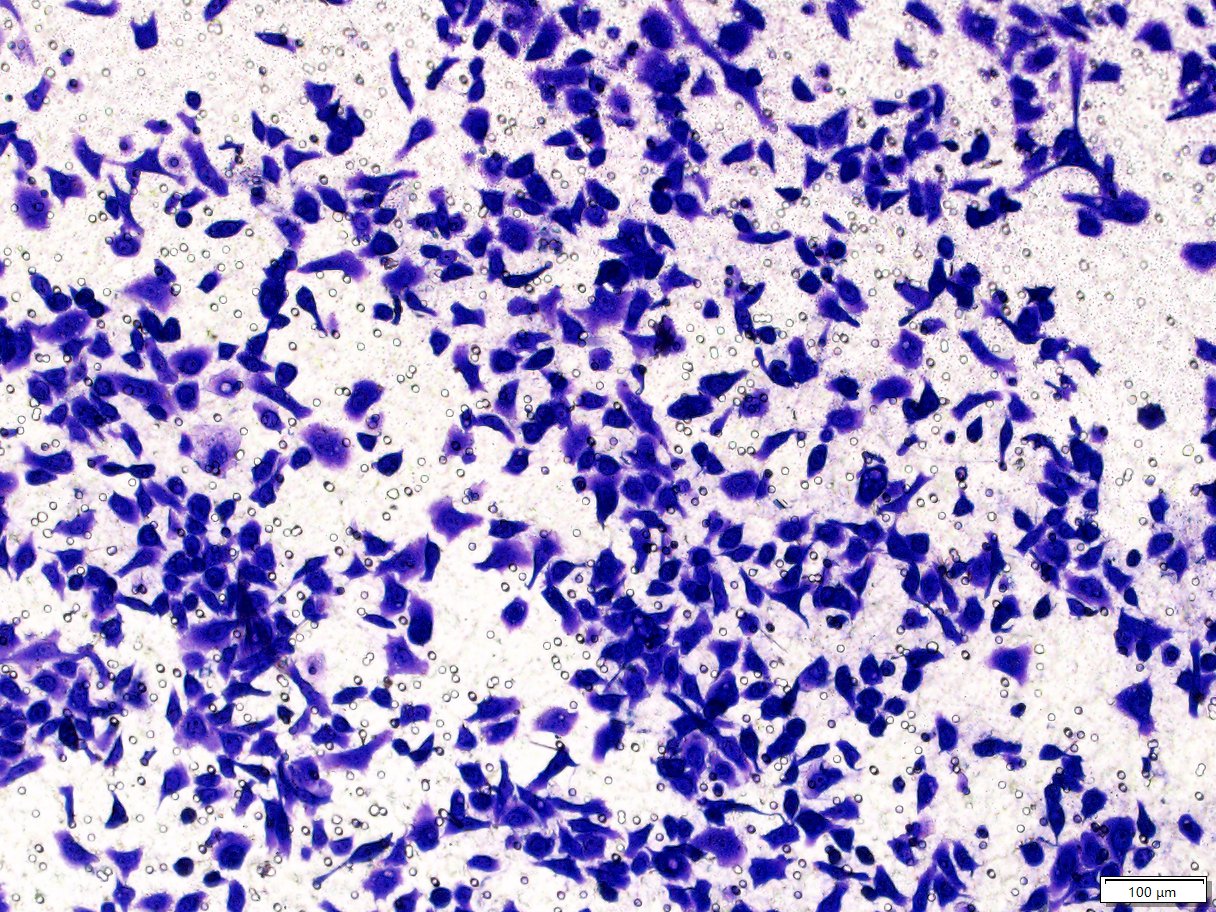

Supplement: Supplemental Information 8 [file peerj-cs-09-1651-s008.zip › Dataset 7/4-6.jpg]

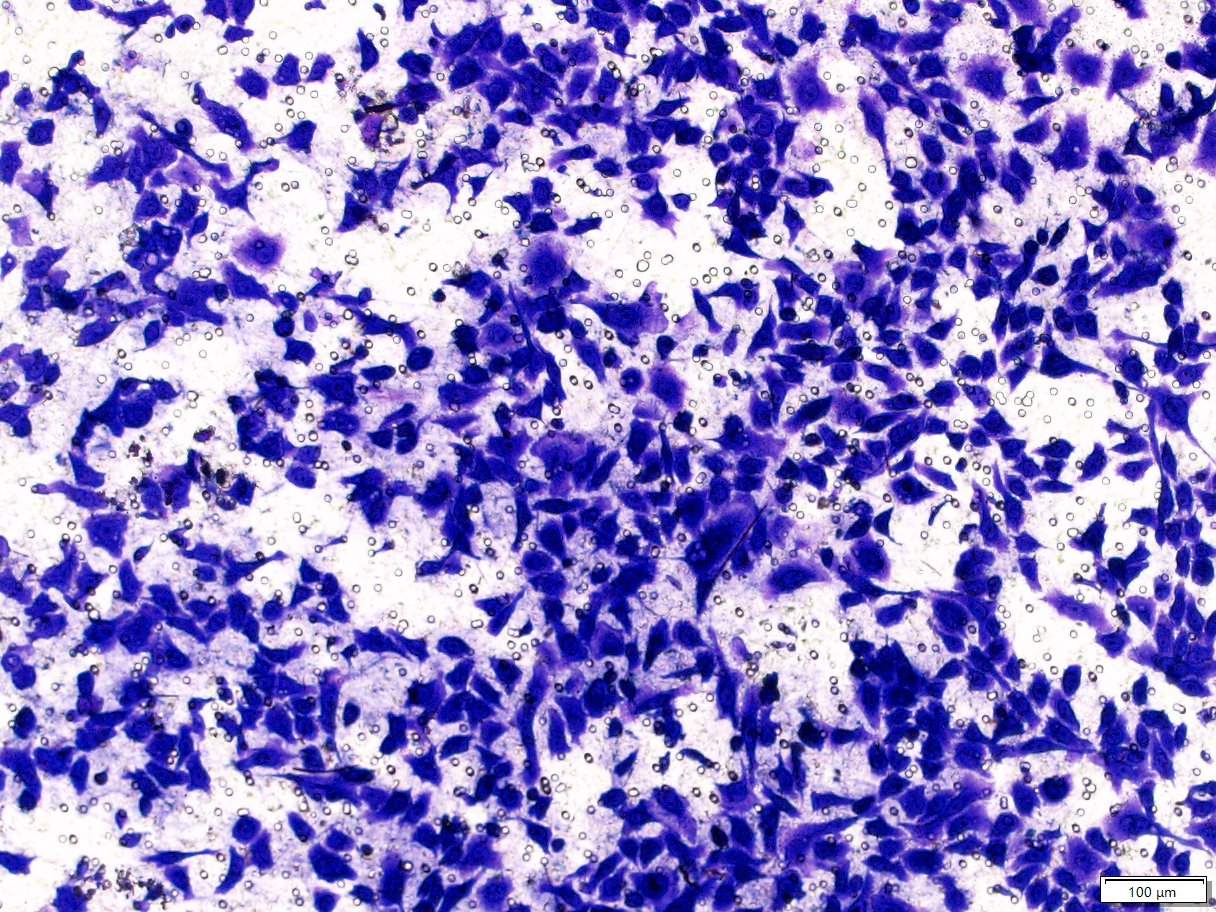

Supplement: Supplemental Information 8 [file peerj-cs-09-1651-s008.zip › Dataset 7/4-7.jpg]

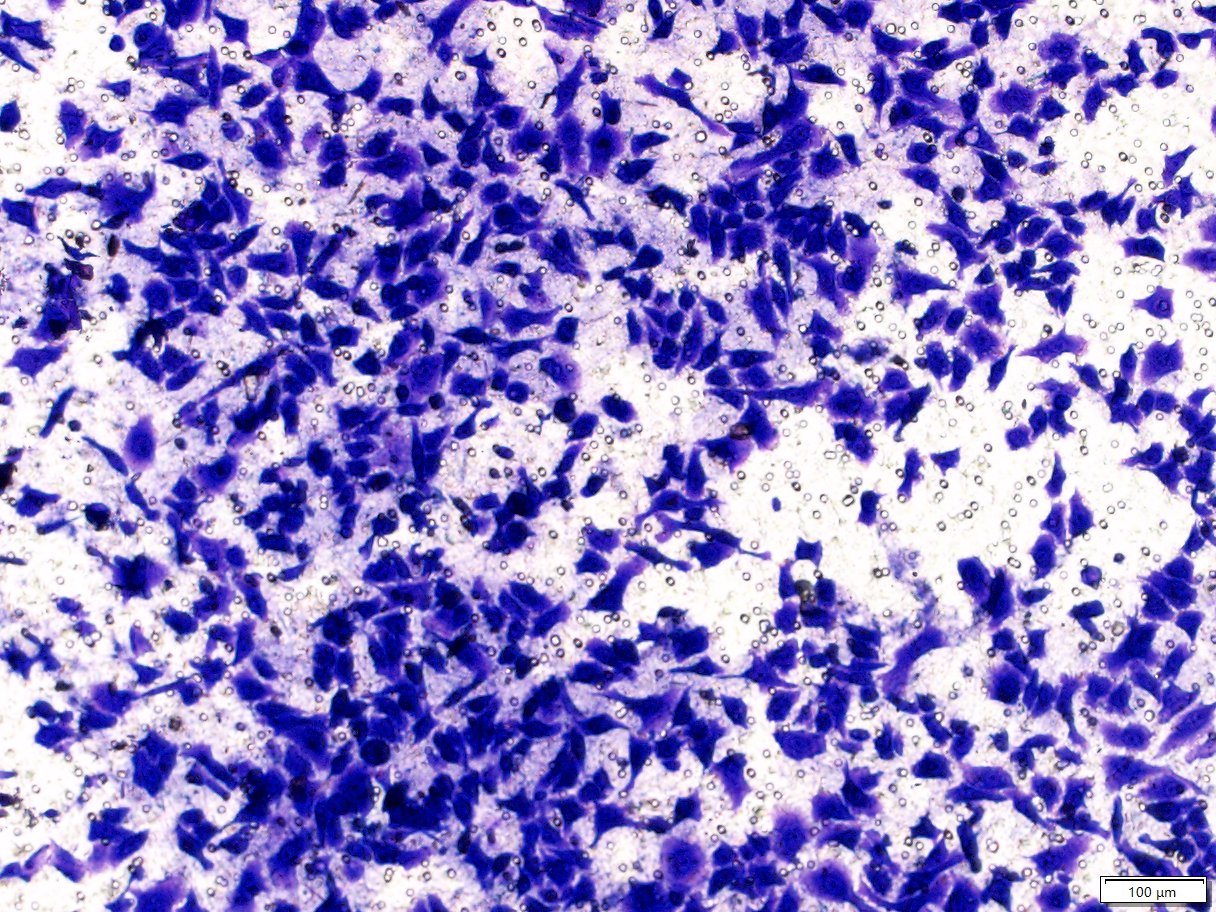

Supplement: Supplemental Information 8 [file peerj-cs-09-1651-s008.zip › Dataset 7/4-8.jpg]

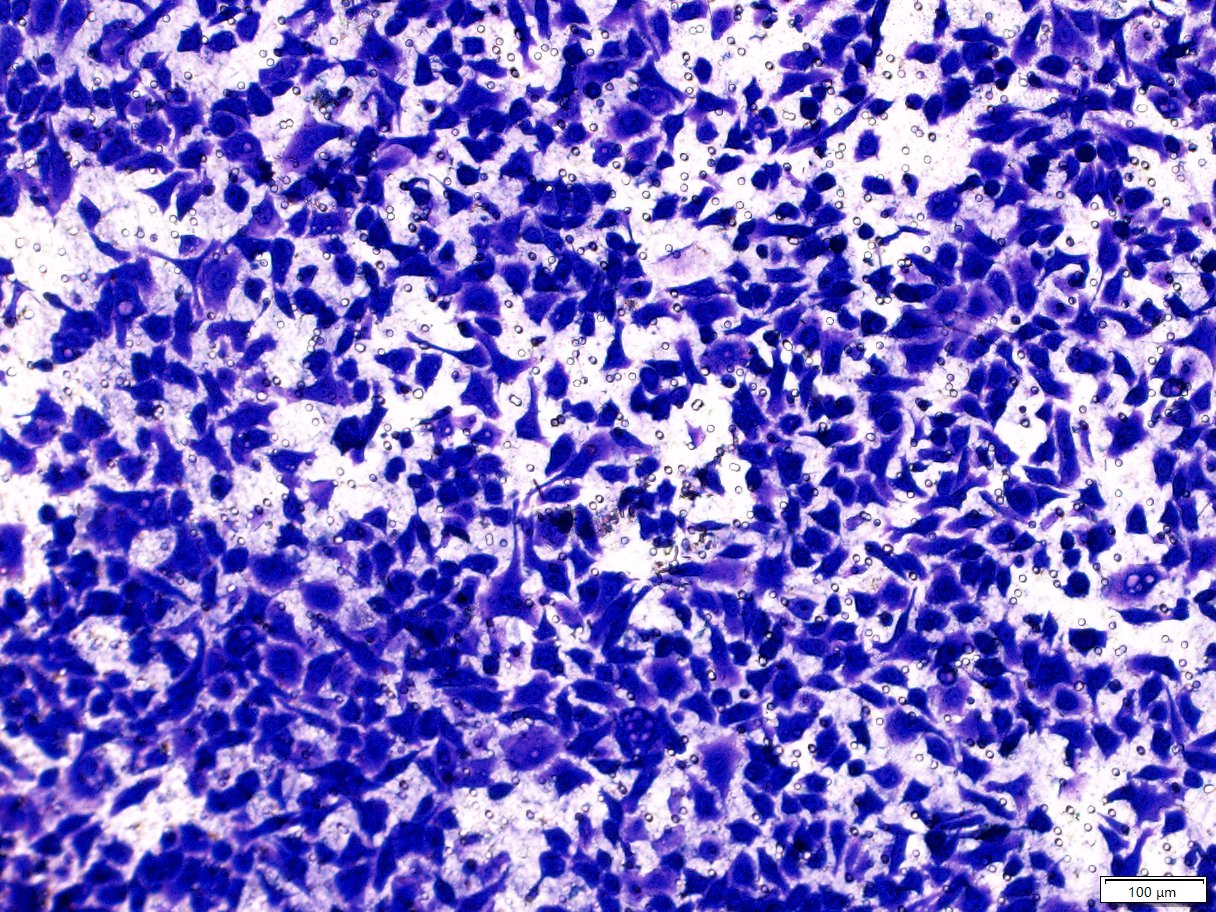

Supplement: Supplemental Information 8 [file peerj-cs-09-1651-s008.zip › Dataset 7/4-9.jpg]

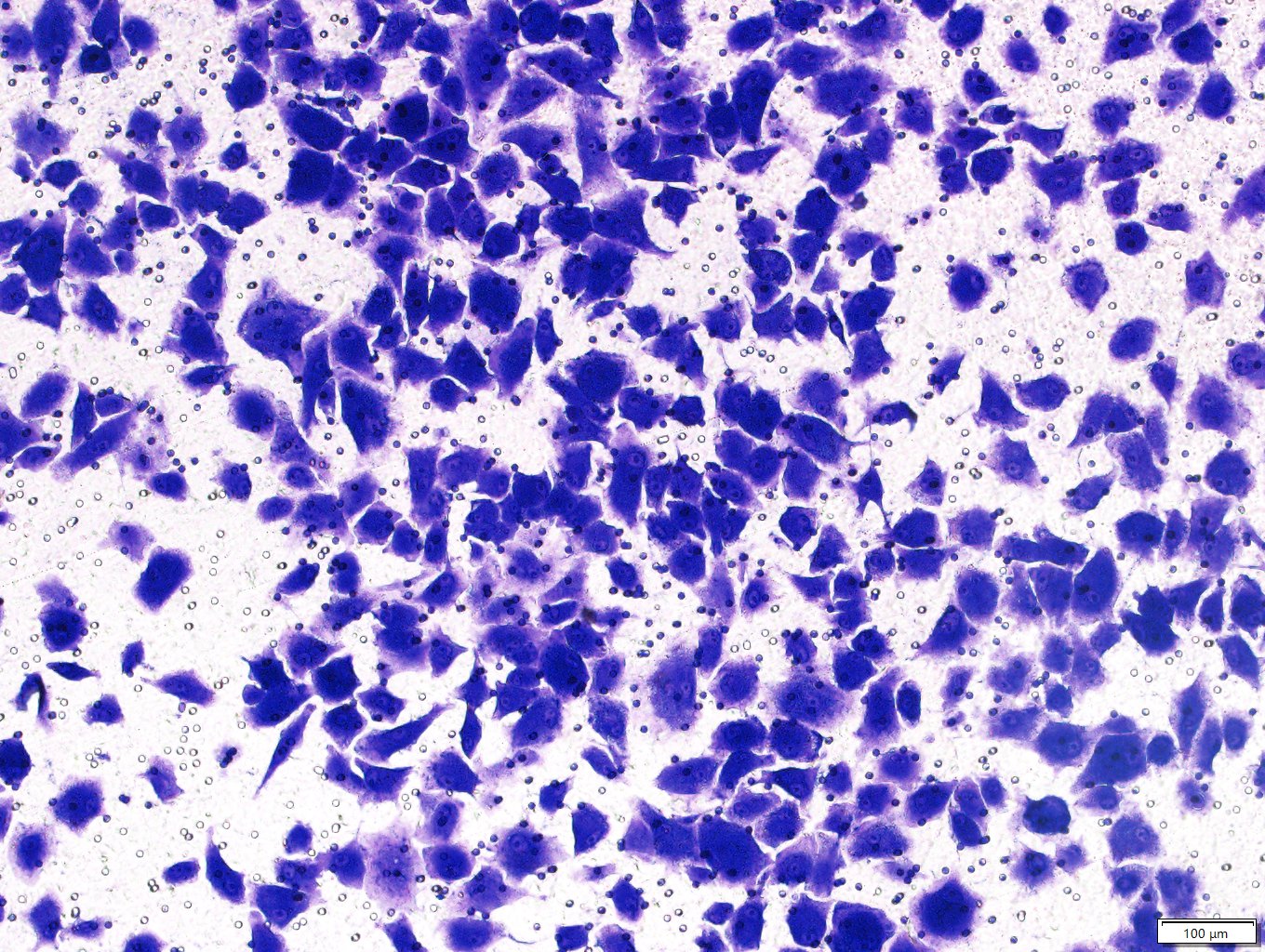

Supplement: Supplemental Information 9 [file peerj-cs-09-1651-s009.zip › Dataset 8/图像_1178.jpg]

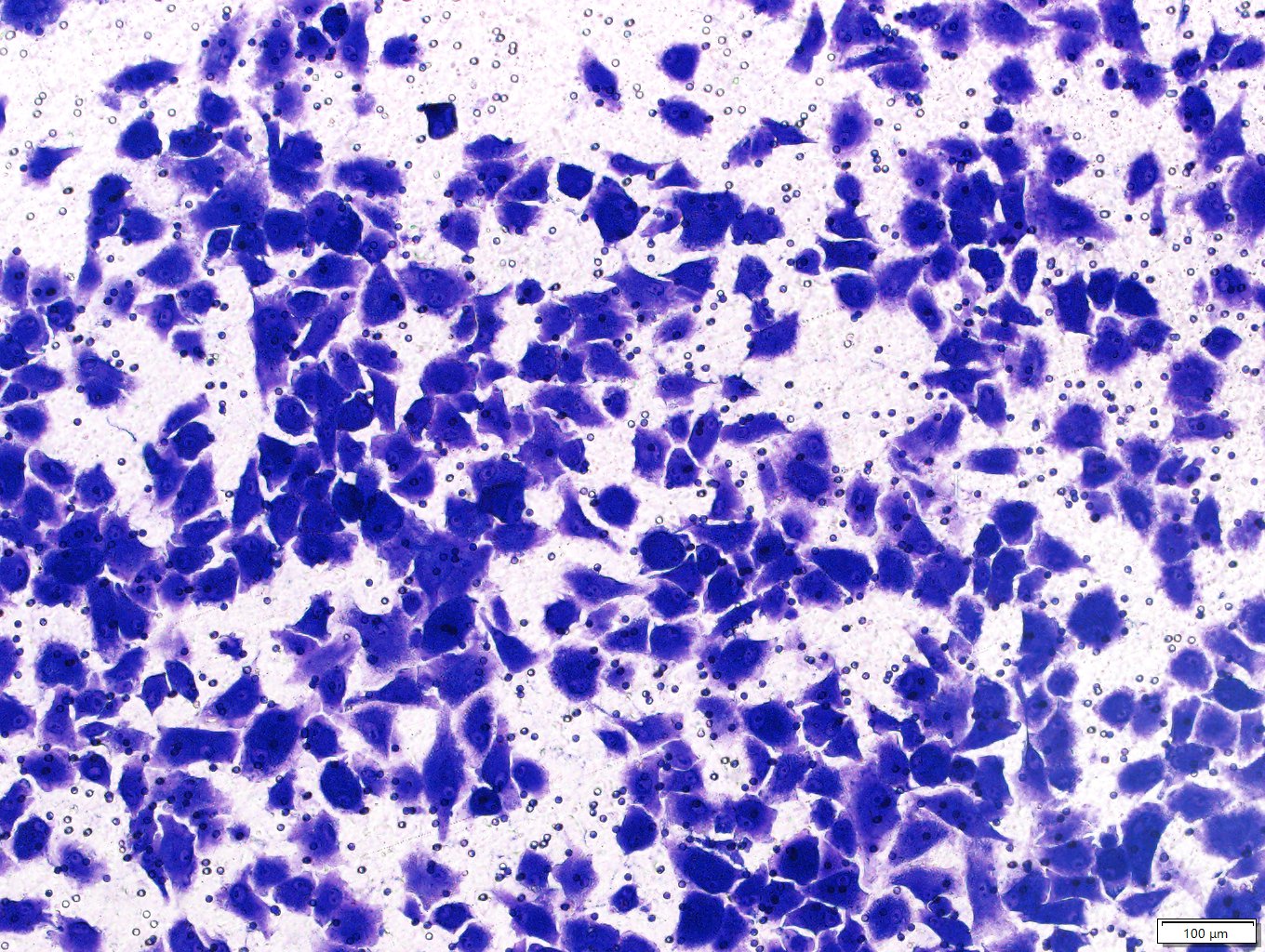

Supplement: Supplemental Information 9 [file peerj-cs-09-1651-s009.zip › Dataset 8/图像_1179.jpg]
