# Supplementary figures and images for: A deep learning approach for the detection and counting of colon cancer cells (HT-29 cells) bunches and impurities (part 5 of 6)
Source: PeerJ Comput Sci. 2023 Dec 5;9:e1651. doi: 10.7717/peerj-cs.1651 (PMC10773923; doi:10.7717/peerj-cs.1651)

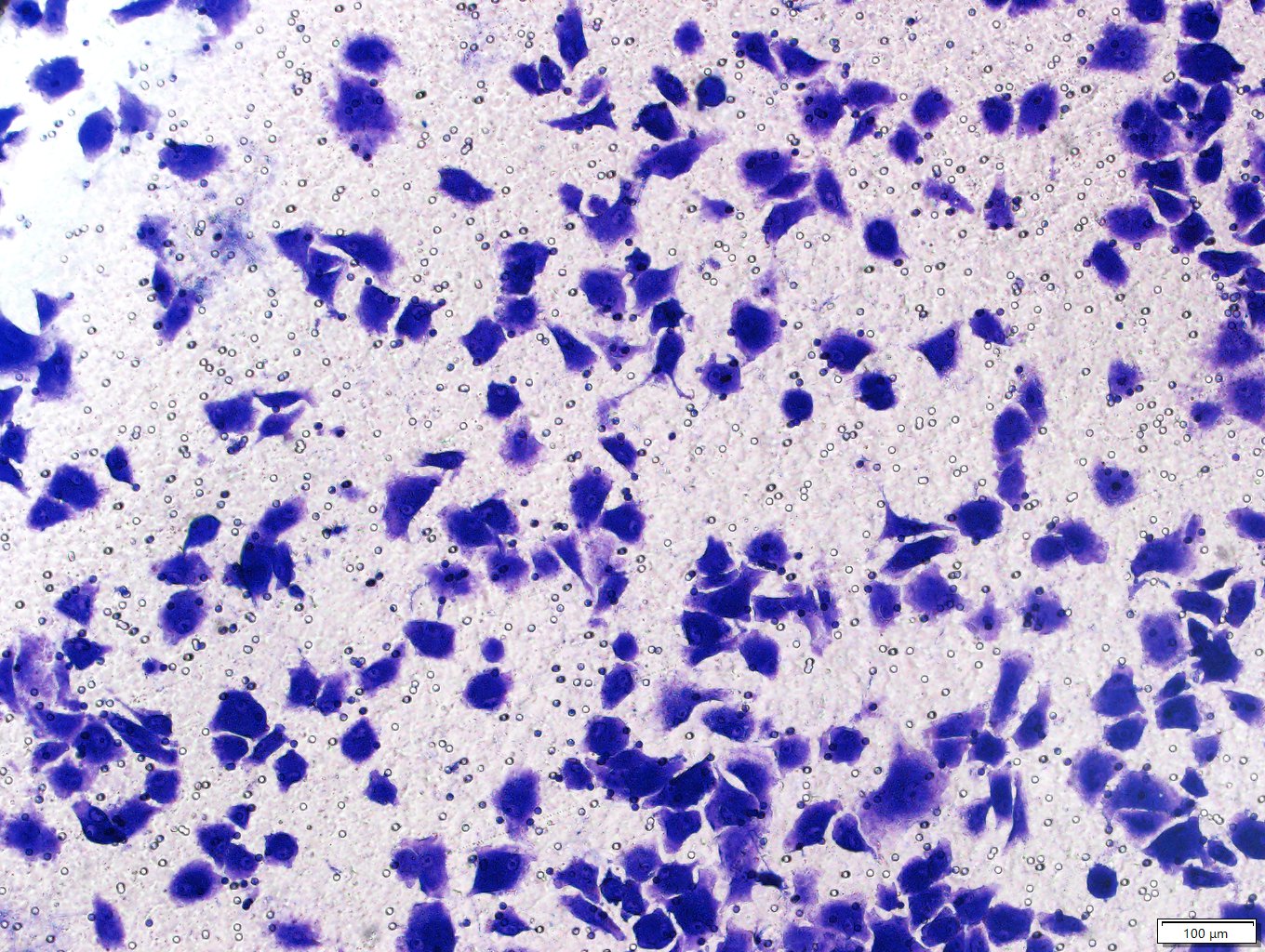

Supplement: Supplemental Information 9 [file peerj-cs-09-1651-s009.zip › Dataset 8/图像_1180.jpg]

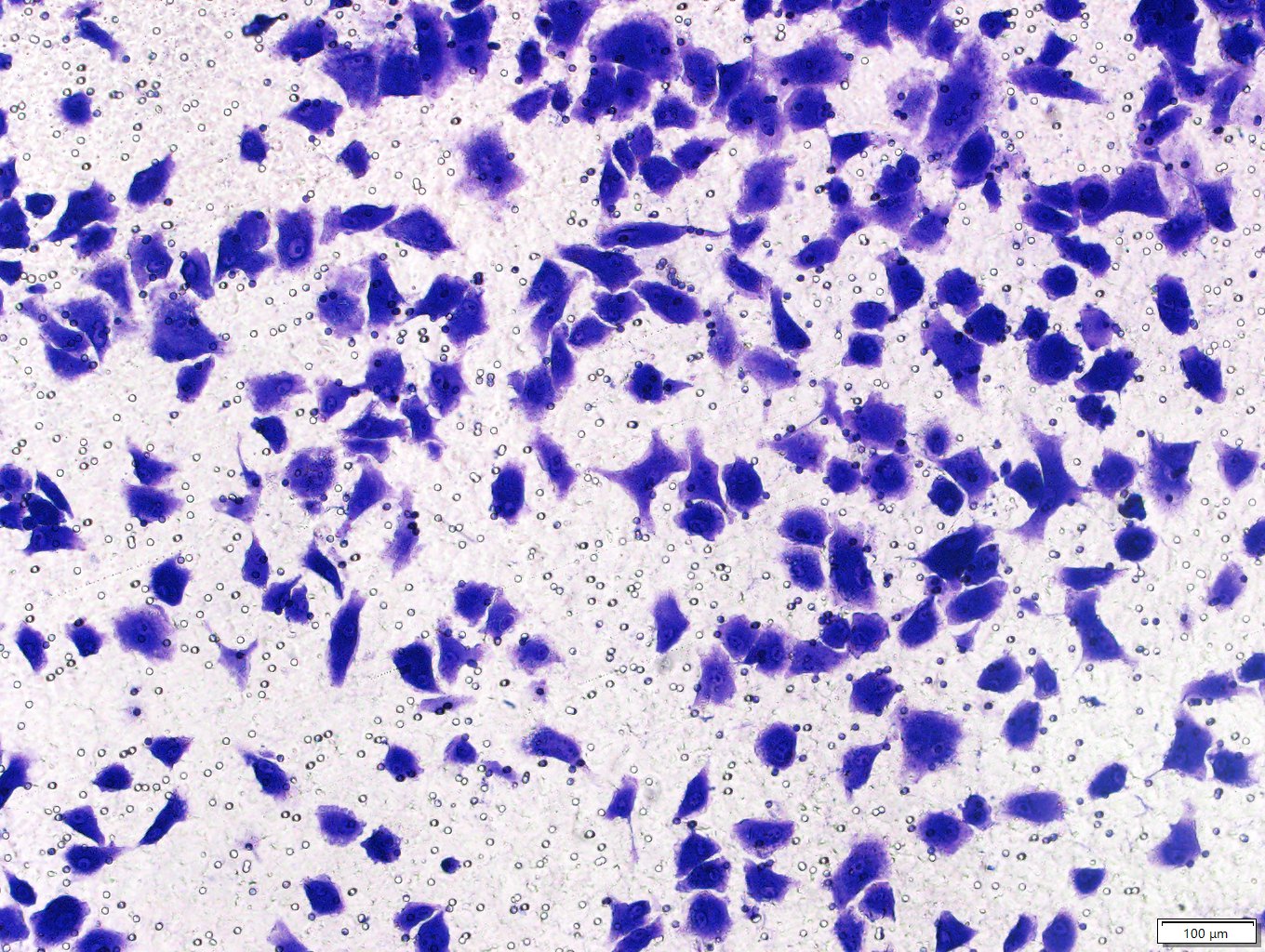

Supplement: Supplemental Information 9 [file peerj-cs-09-1651-s009.zip › Dataset 8/图像_1181.jpg]

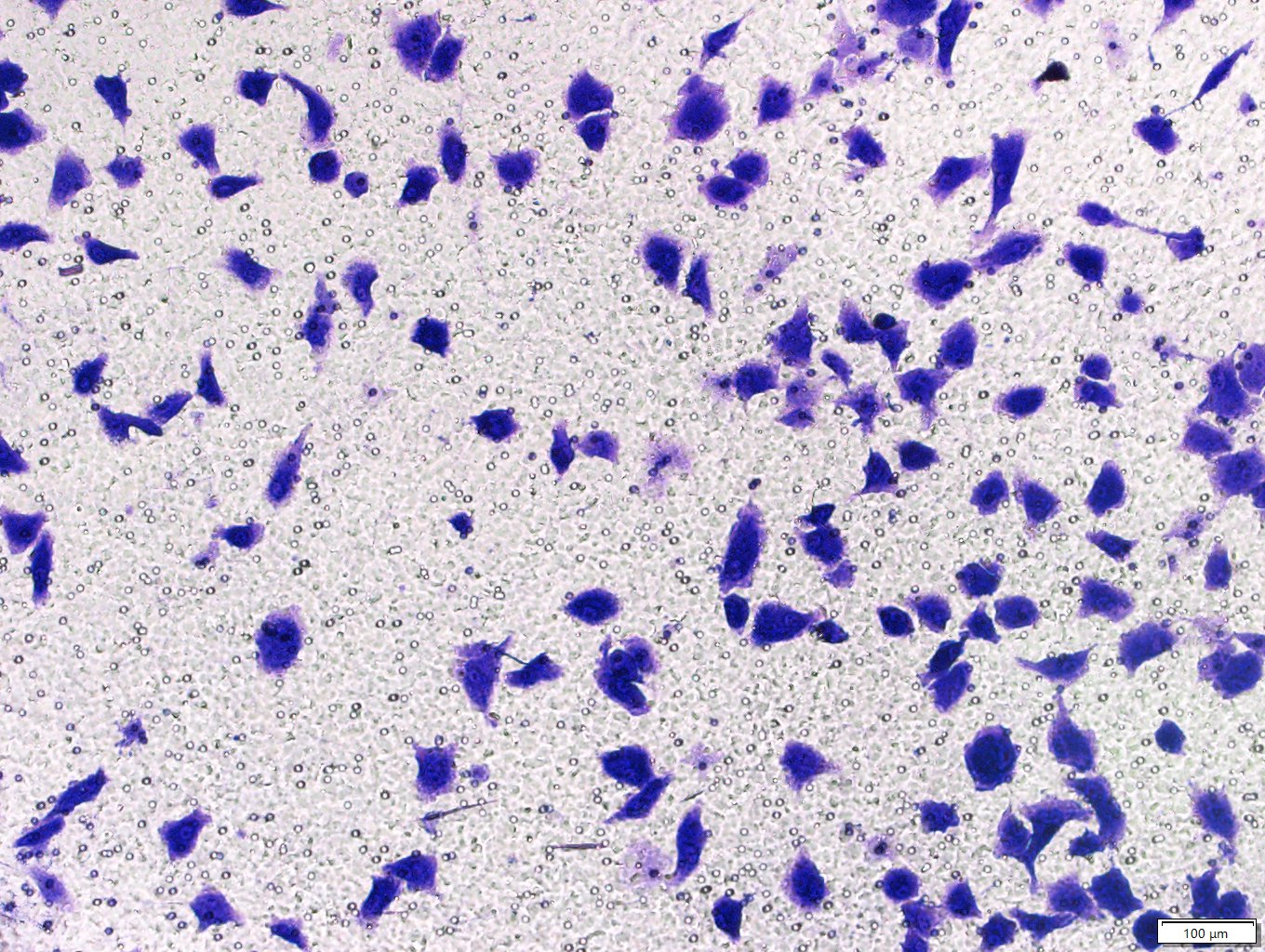

Supplement: Supplemental Information 9 [file peerj-cs-09-1651-s009.zip › Dataset 8/图像_1182.jpg]

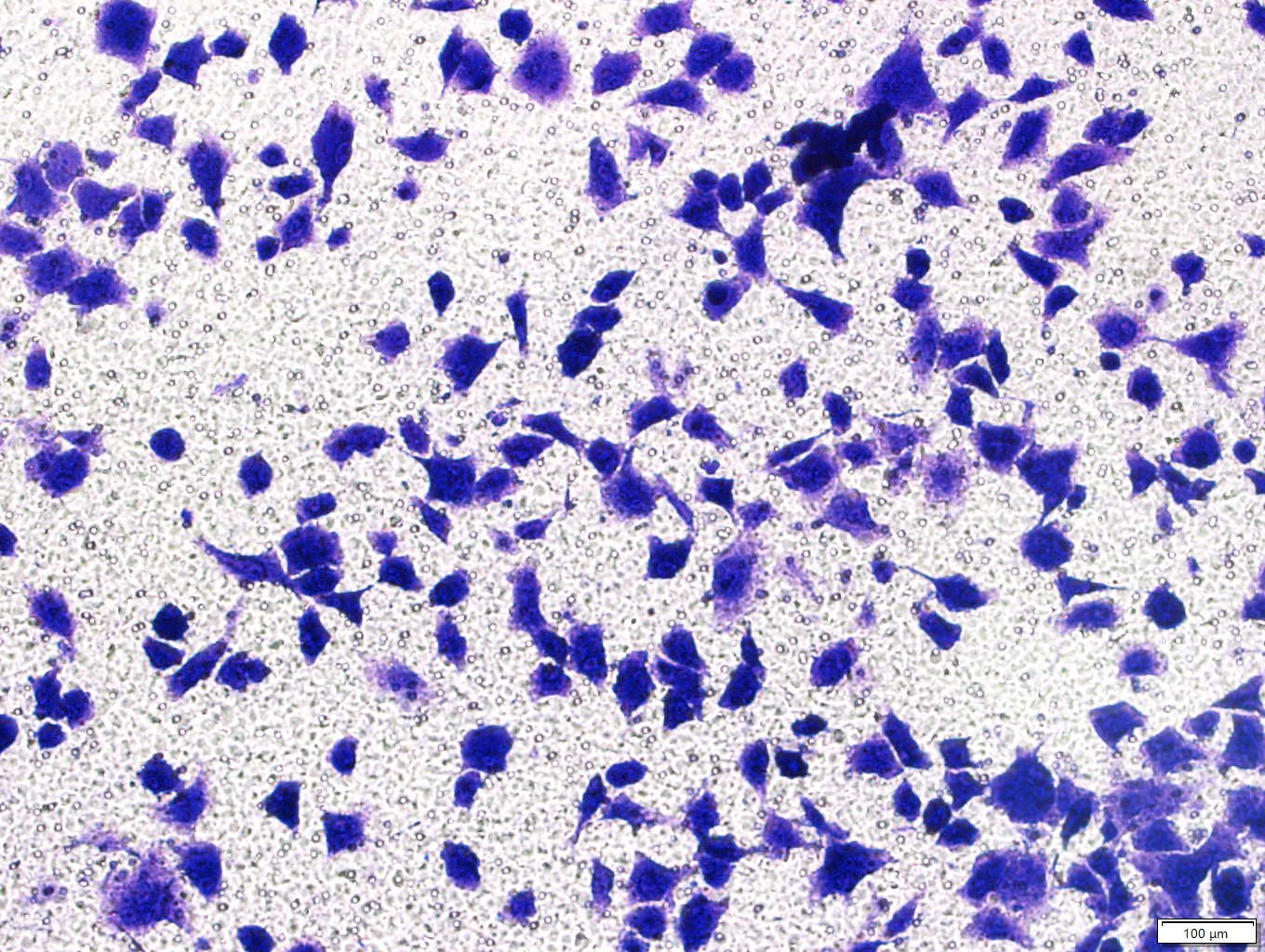

Supplement: Supplemental Information 9 [file peerj-cs-09-1651-s009.zip › Dataset 8/图像_1183.jpg]

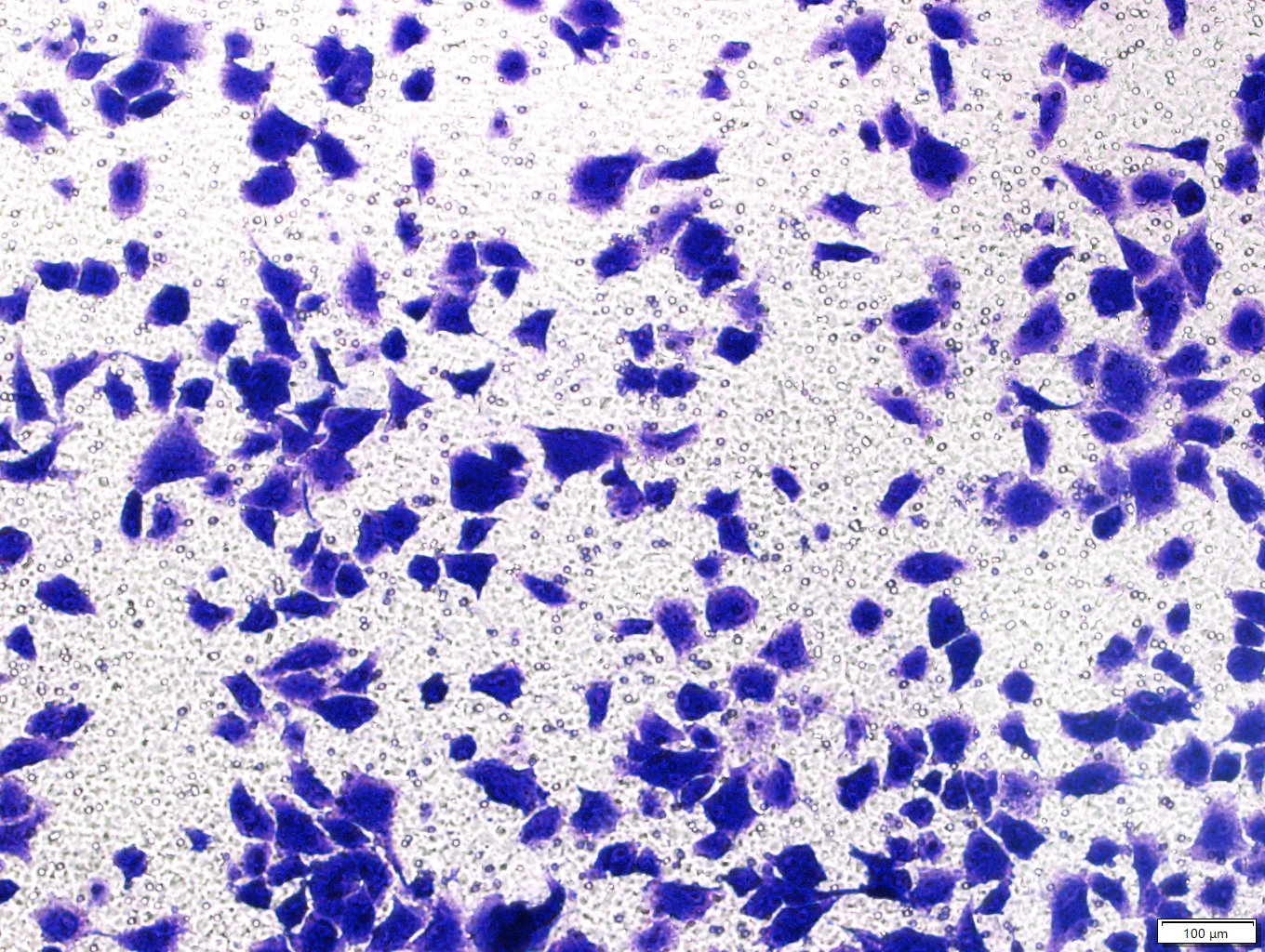

Supplement: Supplemental Information 9 [file peerj-cs-09-1651-s009.zip › Dataset 8/图像_1184.jpg]

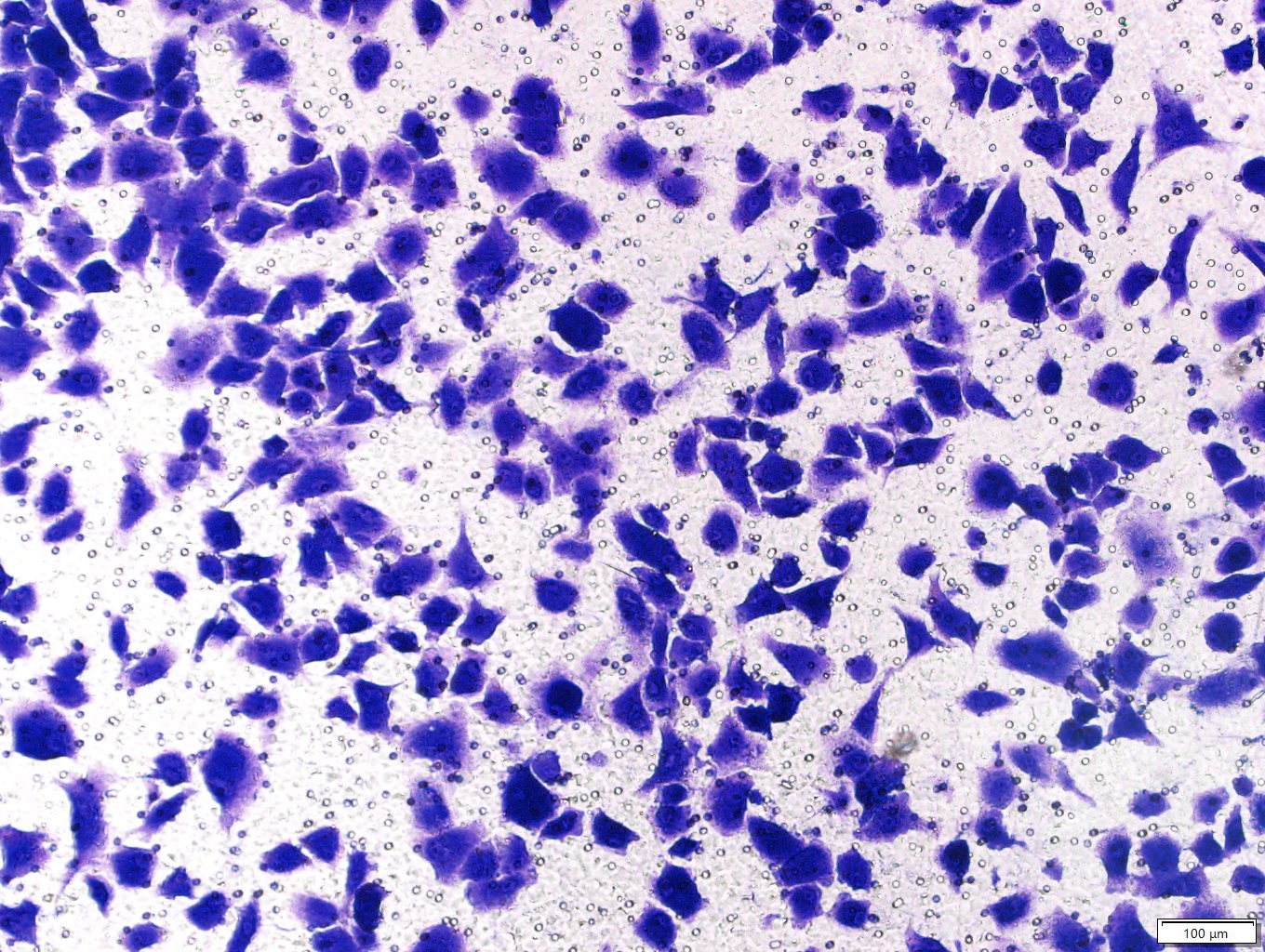

Supplement: Supplemental Information 9 [file peerj-cs-09-1651-s009.zip › Dataset 8/图像_1185.jpg]

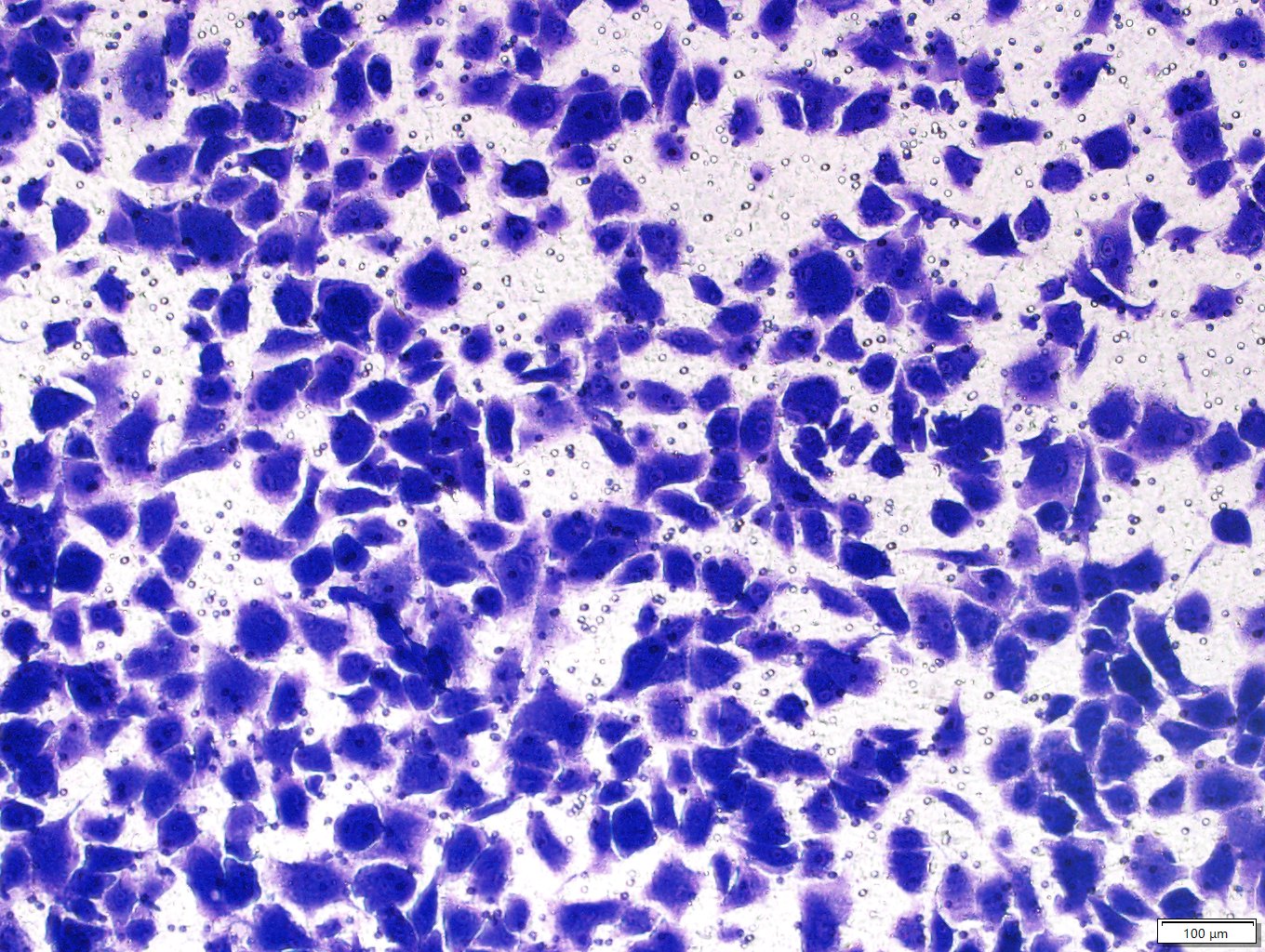

Supplement: Supplemental Information 9 [file peerj-cs-09-1651-s009.zip › Dataset 8/图像_1186.jpg]

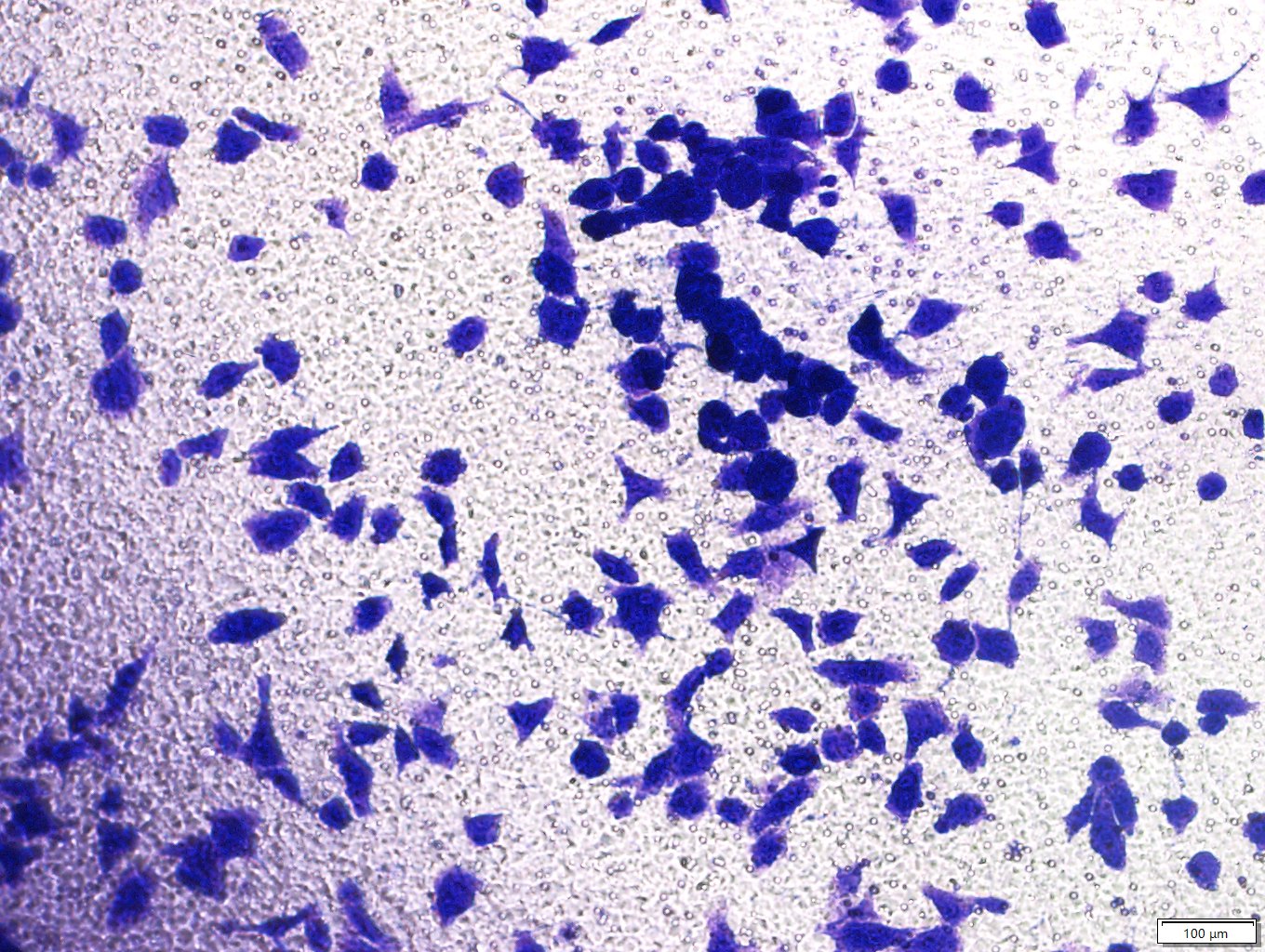

Supplement: Supplemental Information 9 [file peerj-cs-09-1651-s009.zip › Dataset 8/图像_1229.jpg]

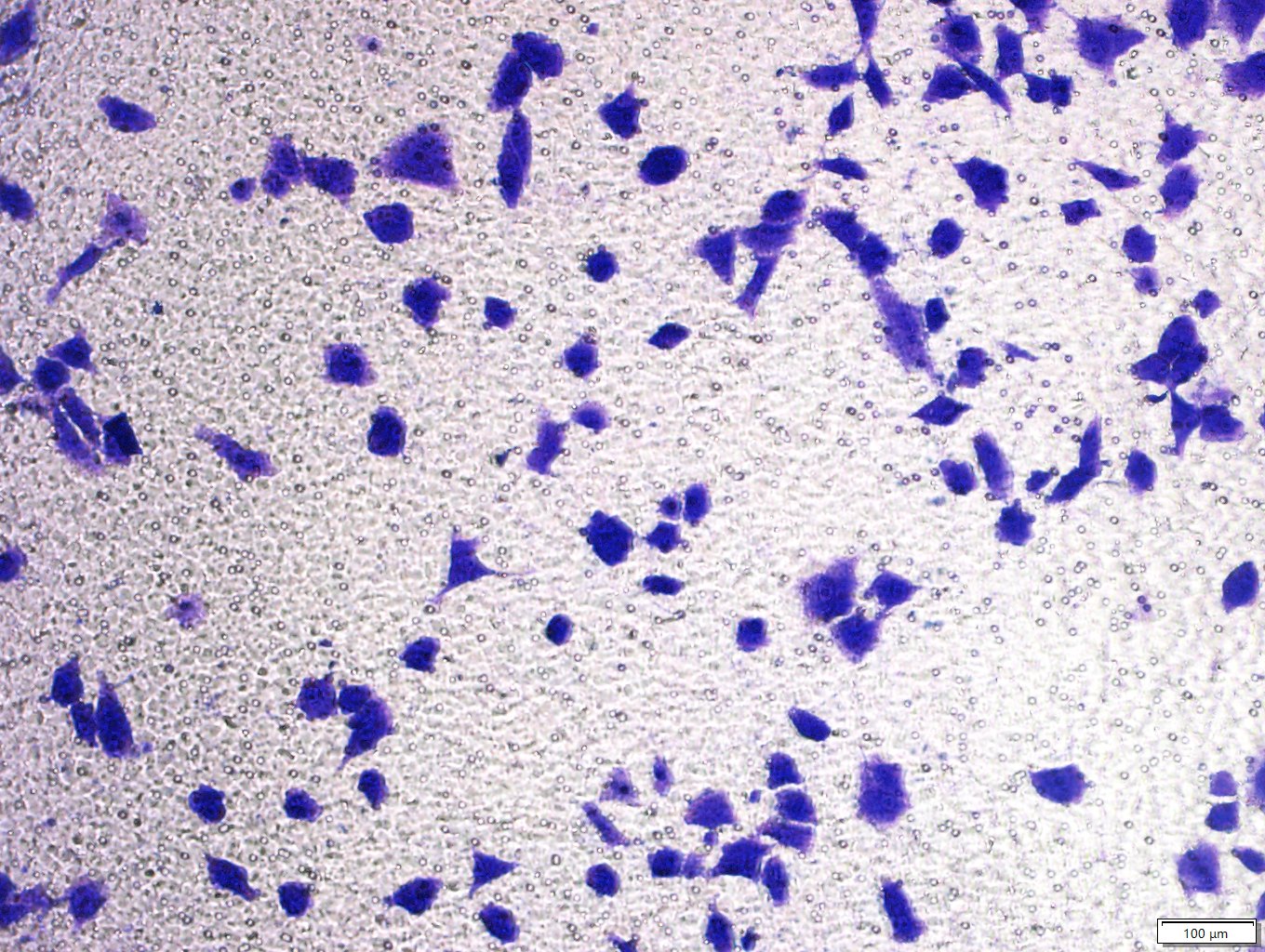

Supplement: Supplemental Information 9 [file peerj-cs-09-1651-s009.zip › Dataset 8/图像_1230.jpg]

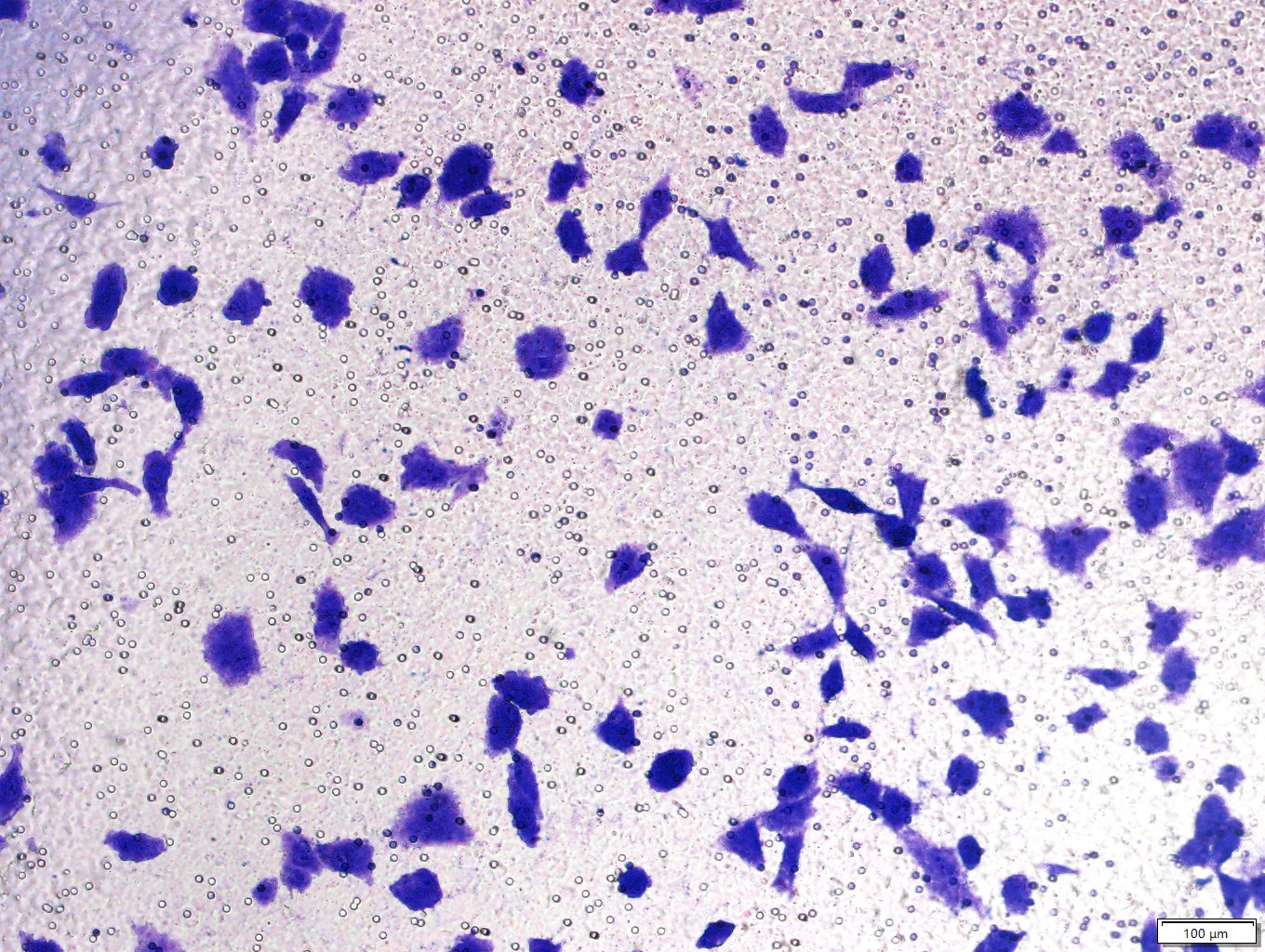

Supplement: Supplemental Information 9 [file peerj-cs-09-1651-s009.zip › Dataset 8/图像_1231.jpg]

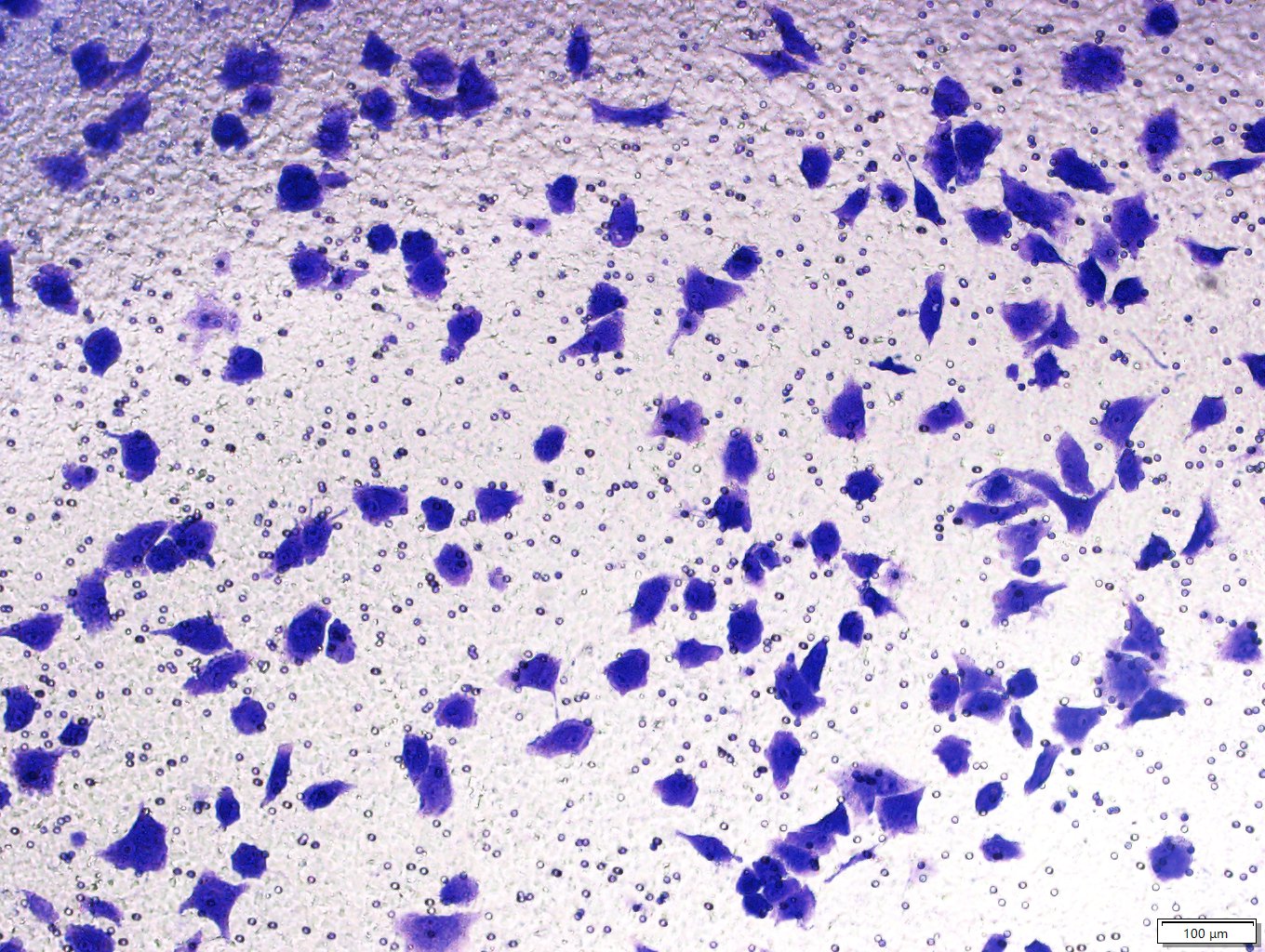

Supplement: Supplemental Information 9 [file peerj-cs-09-1651-s009.zip › Dataset 8/图像_1232.jpg]

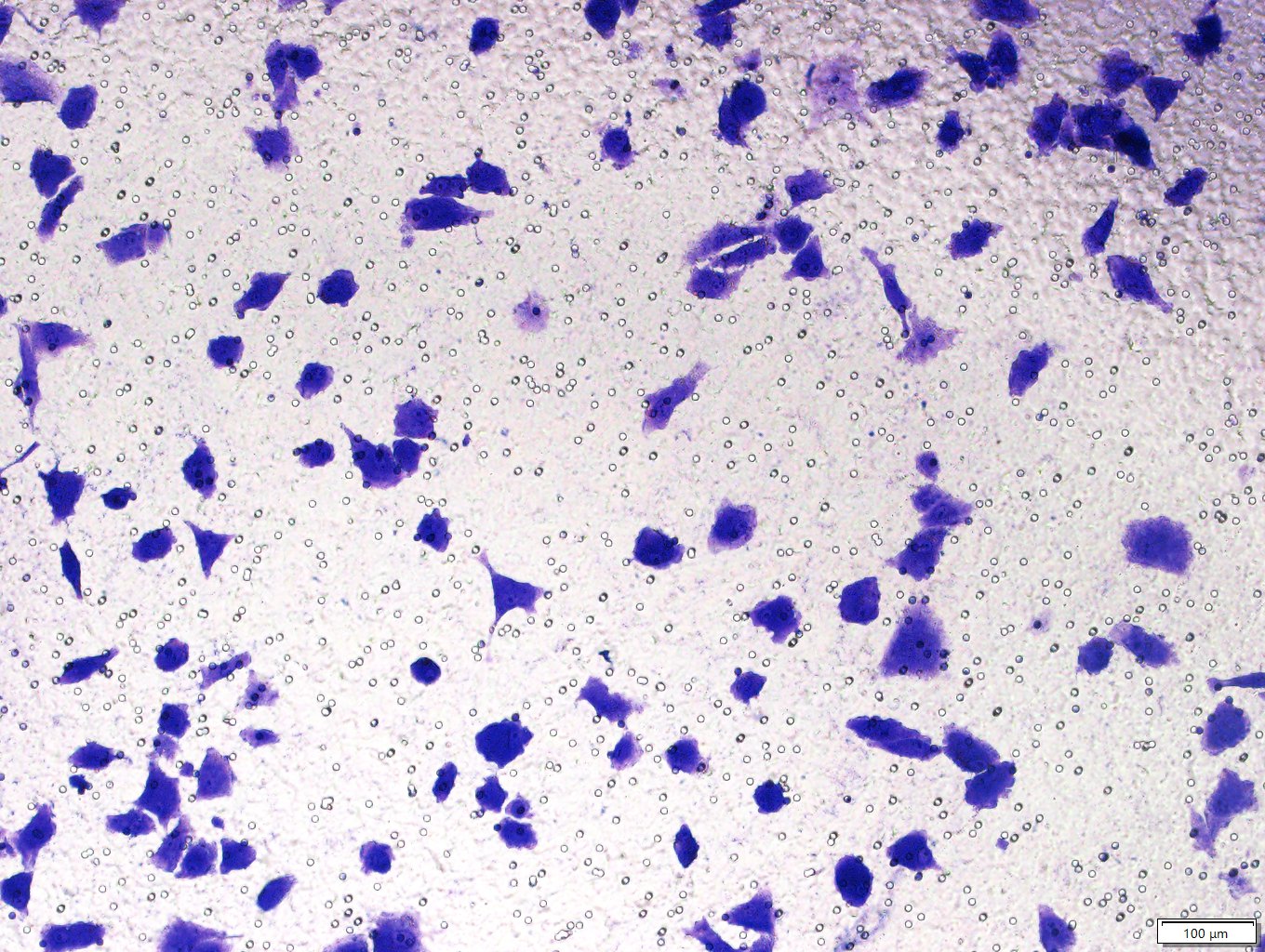

Supplement: Supplemental Information 9 [file peerj-cs-09-1651-s009.zip › Dataset 8/图像_1233.jpg]

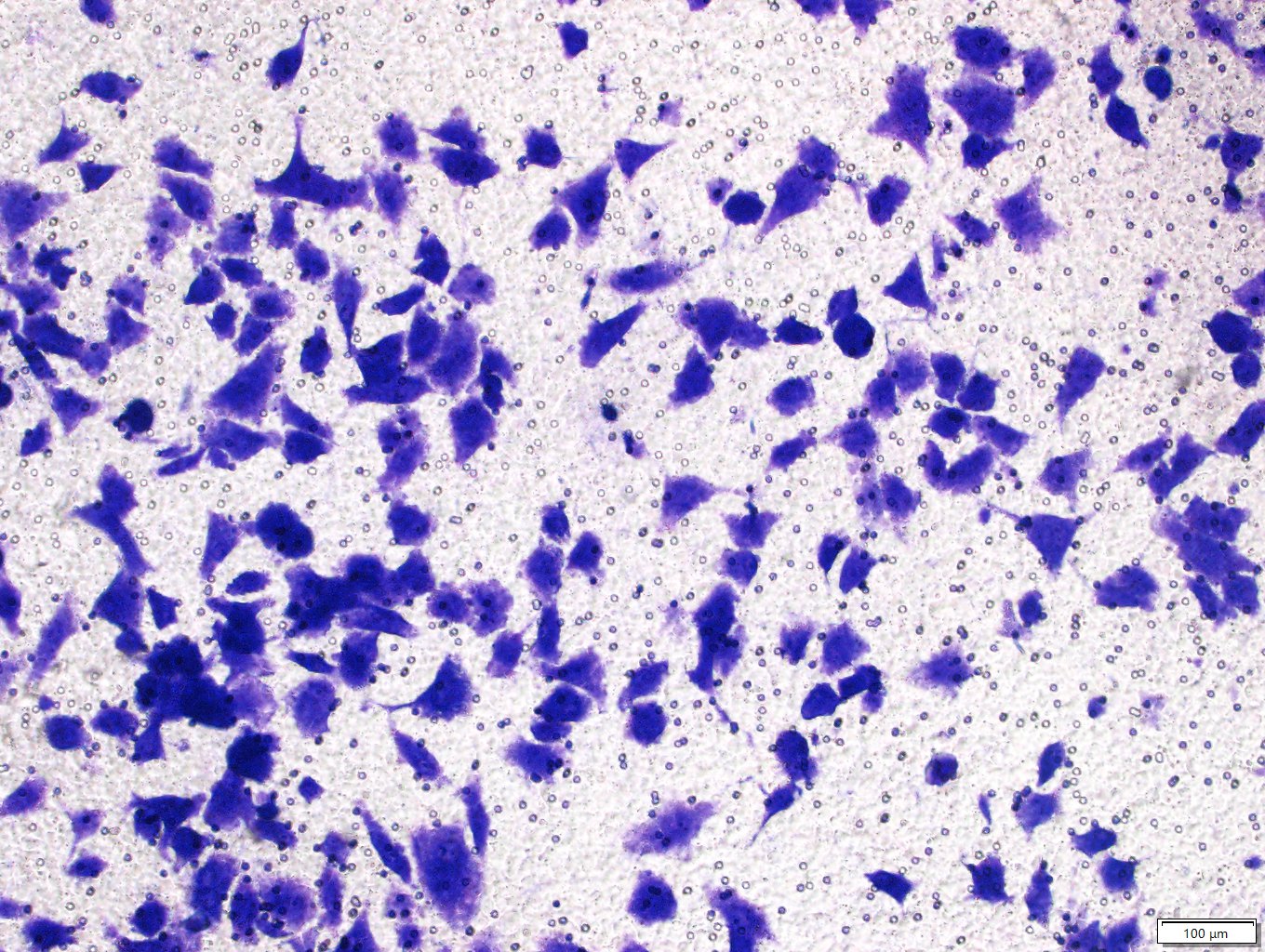

Supplement: Supplemental Information 9 [file peerj-cs-09-1651-s009.zip › Dataset 8/图像_1234.jpg]

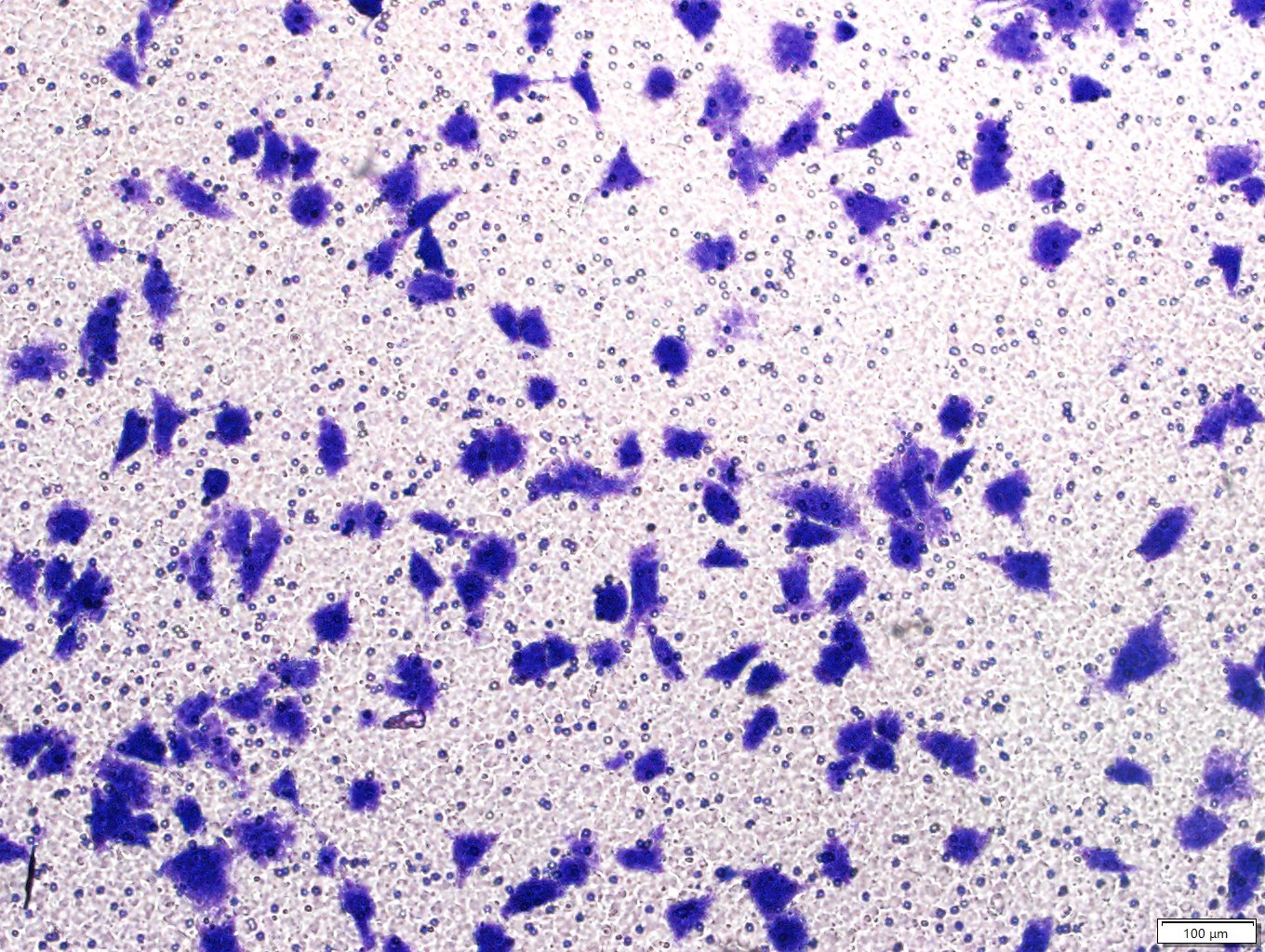

Supplement: Supplemental Information 9 [file peerj-cs-09-1651-s009.zip › Dataset 8/图像_1235.jpg]

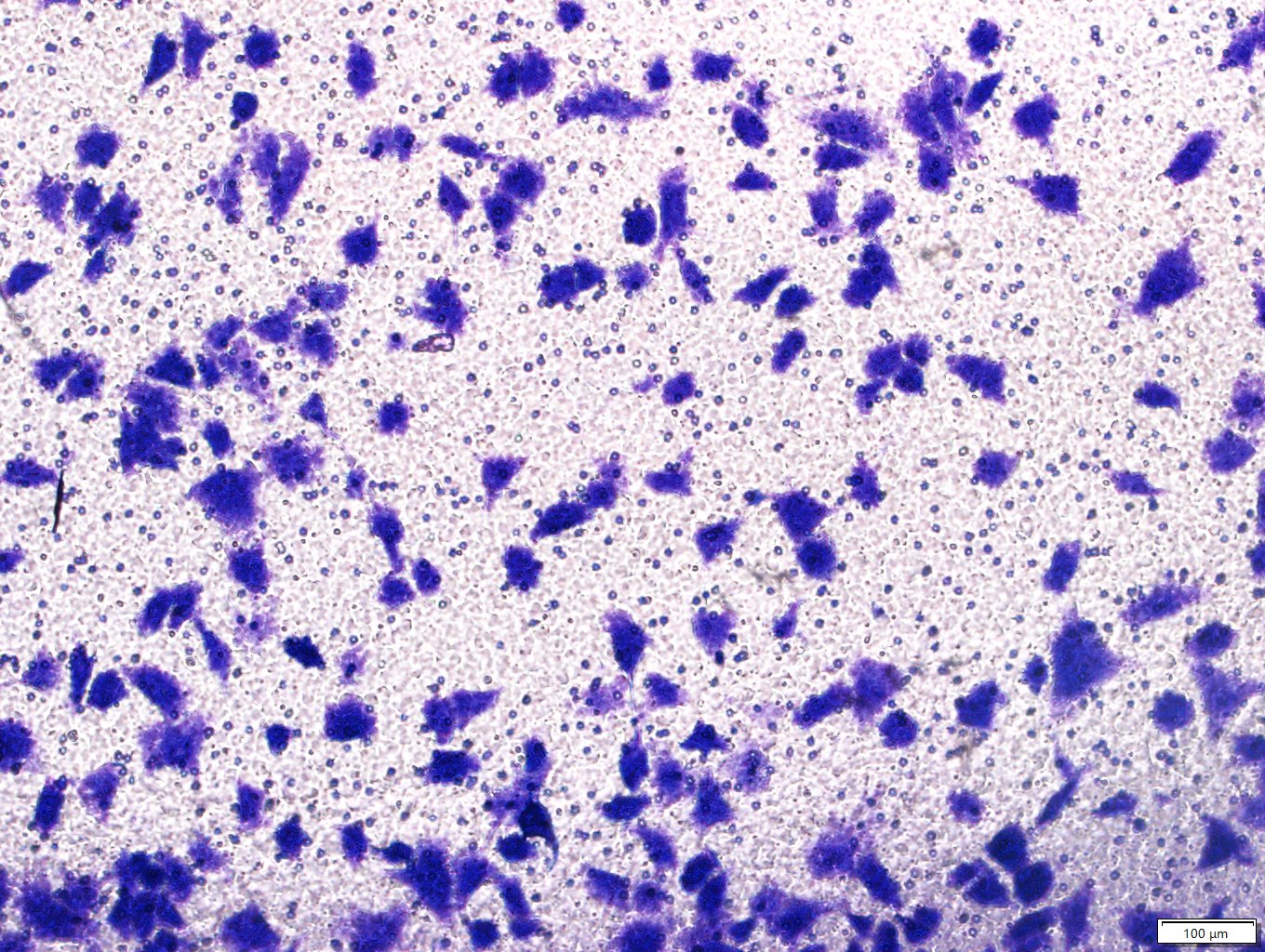

Supplement: Supplemental Information 9 [file peerj-cs-09-1651-s009.zip › Dataset 8/图像_1236.jpg]

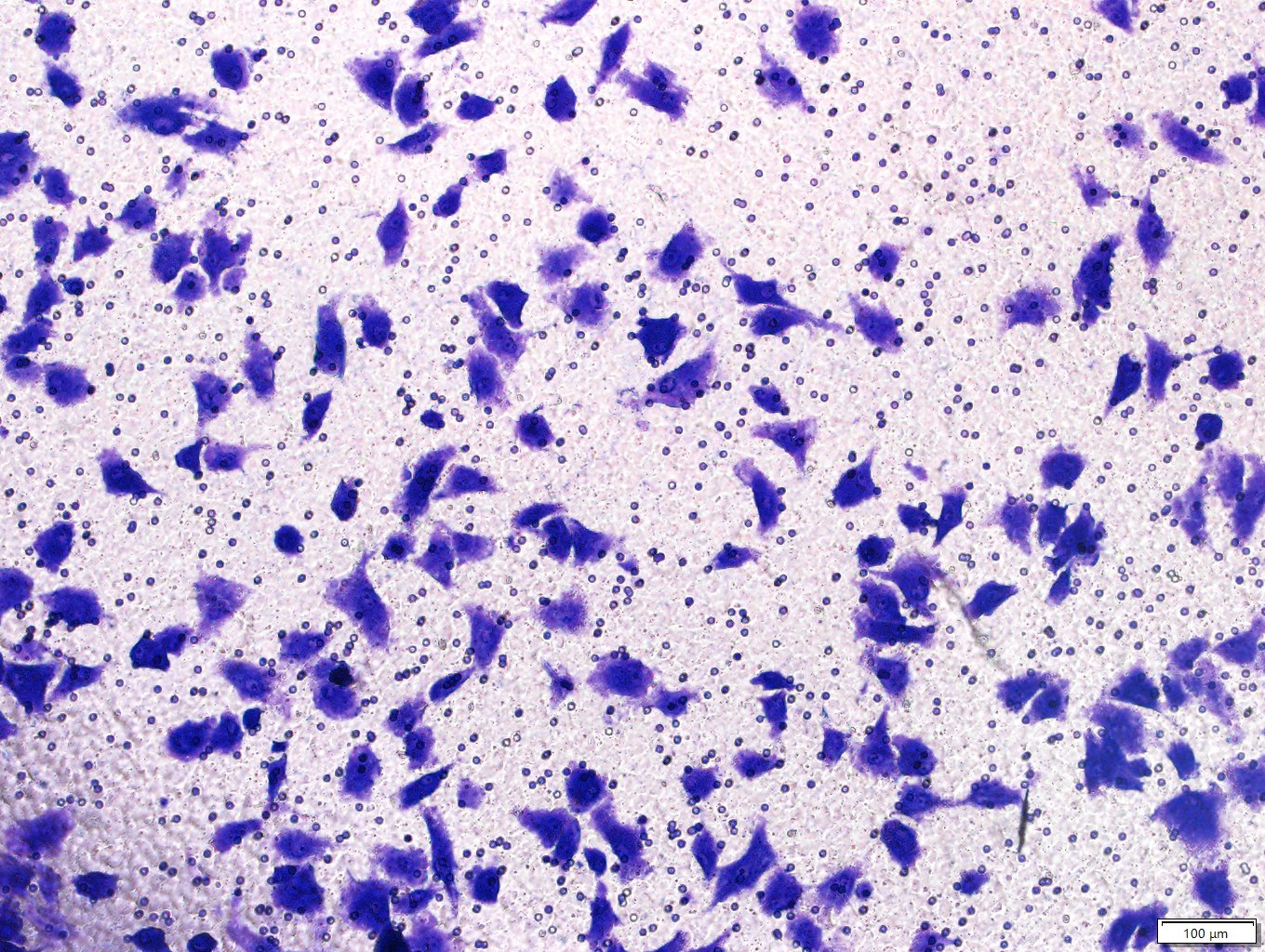

Supplement: Supplemental Information 9 [file peerj-cs-09-1651-s009.zip › Dataset 8/图像_1237.jpg]

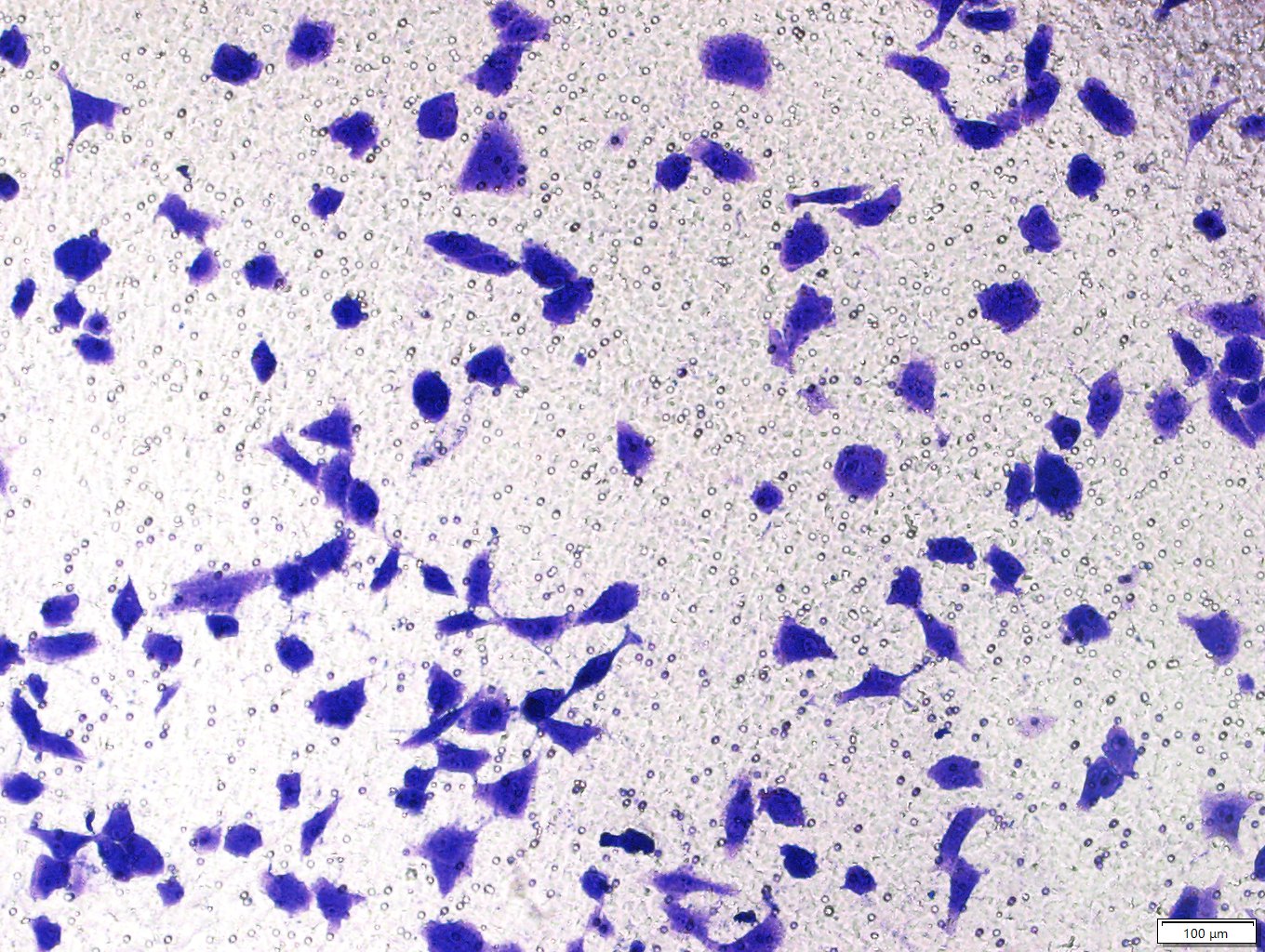

Supplement: Supplemental Information 9 [file peerj-cs-09-1651-s009.zip › Dataset 8/图像_1238.jpg]

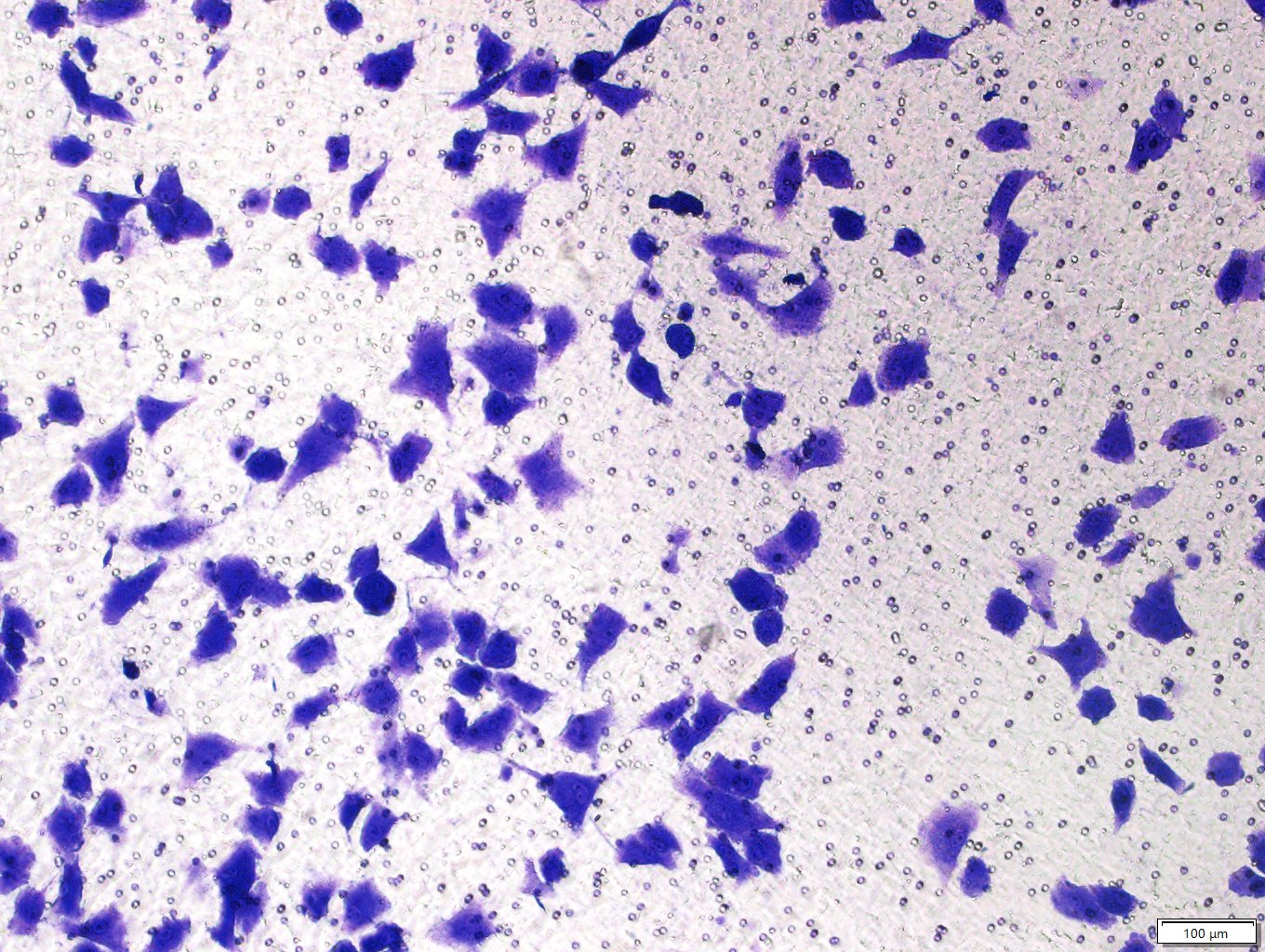

Supplement: Supplemental Information 9 [file peerj-cs-09-1651-s009.zip › Dataset 8/图像_1239.jpg]

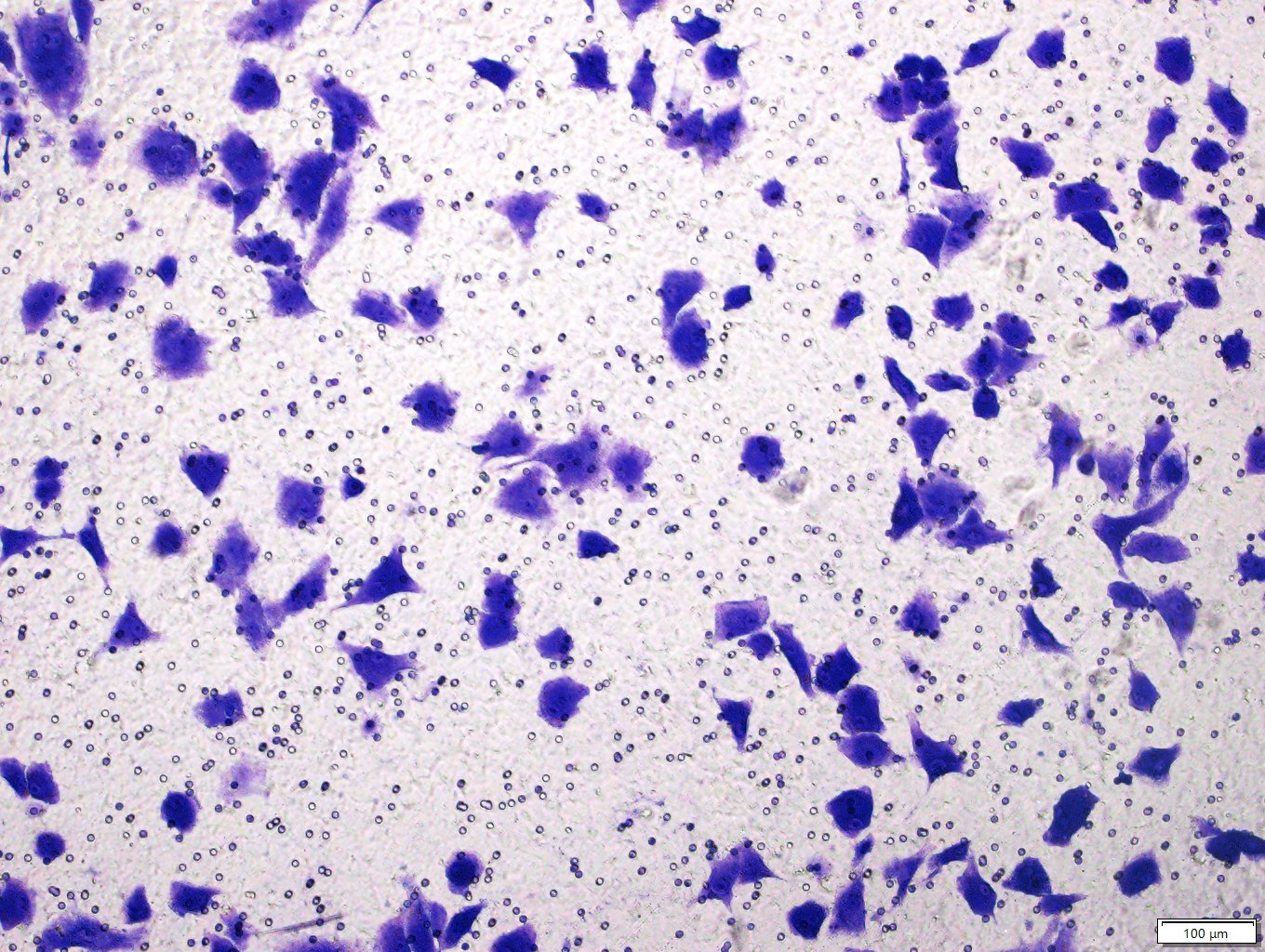

Supplement: Supplemental Information 9 [file peerj-cs-09-1651-s009.zip › Dataset 8/图像_1240.jpg]

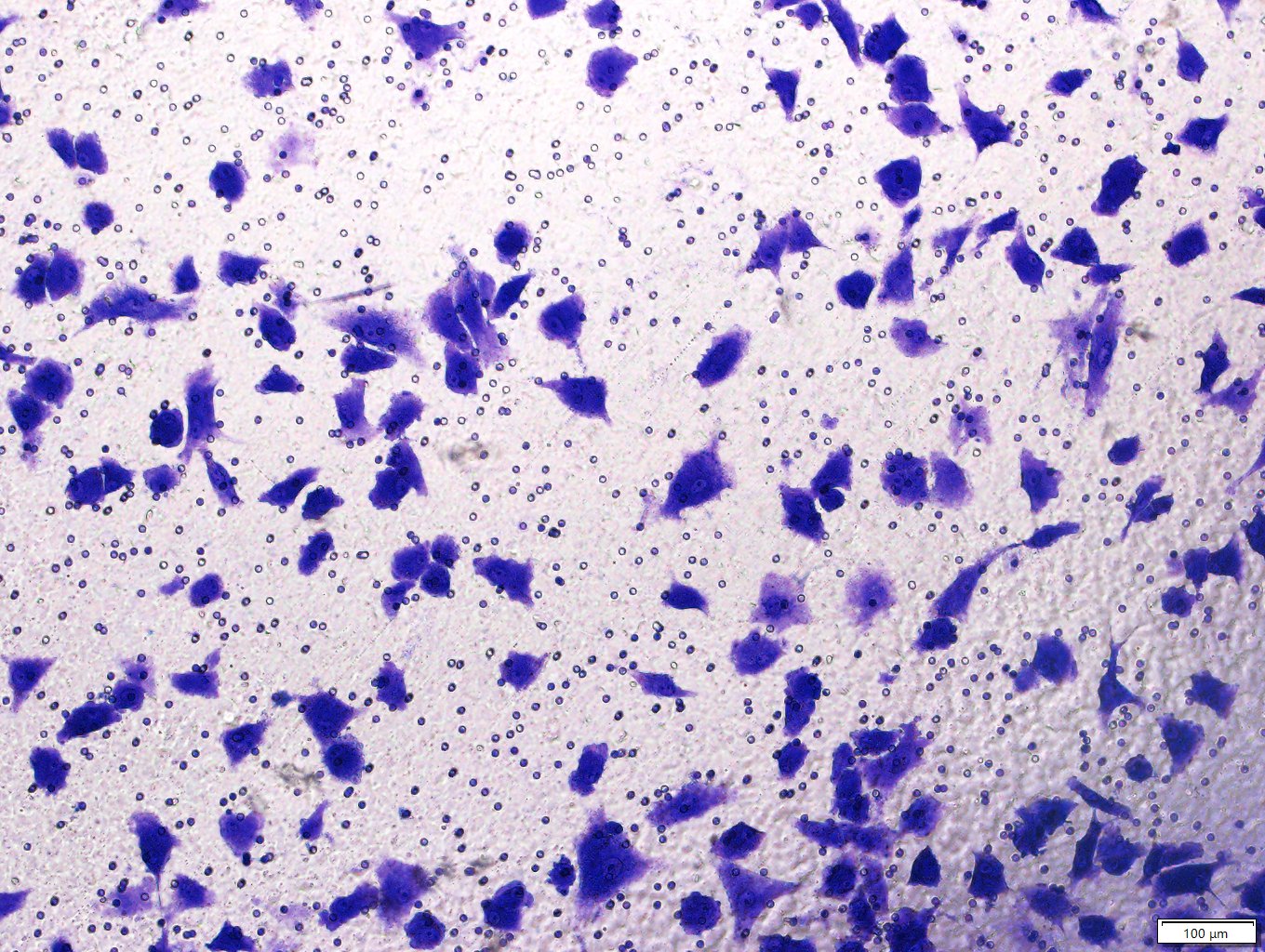

Supplement: Supplemental Information 9 [file peerj-cs-09-1651-s009.zip › Dataset 8/图像_1241.jpg]

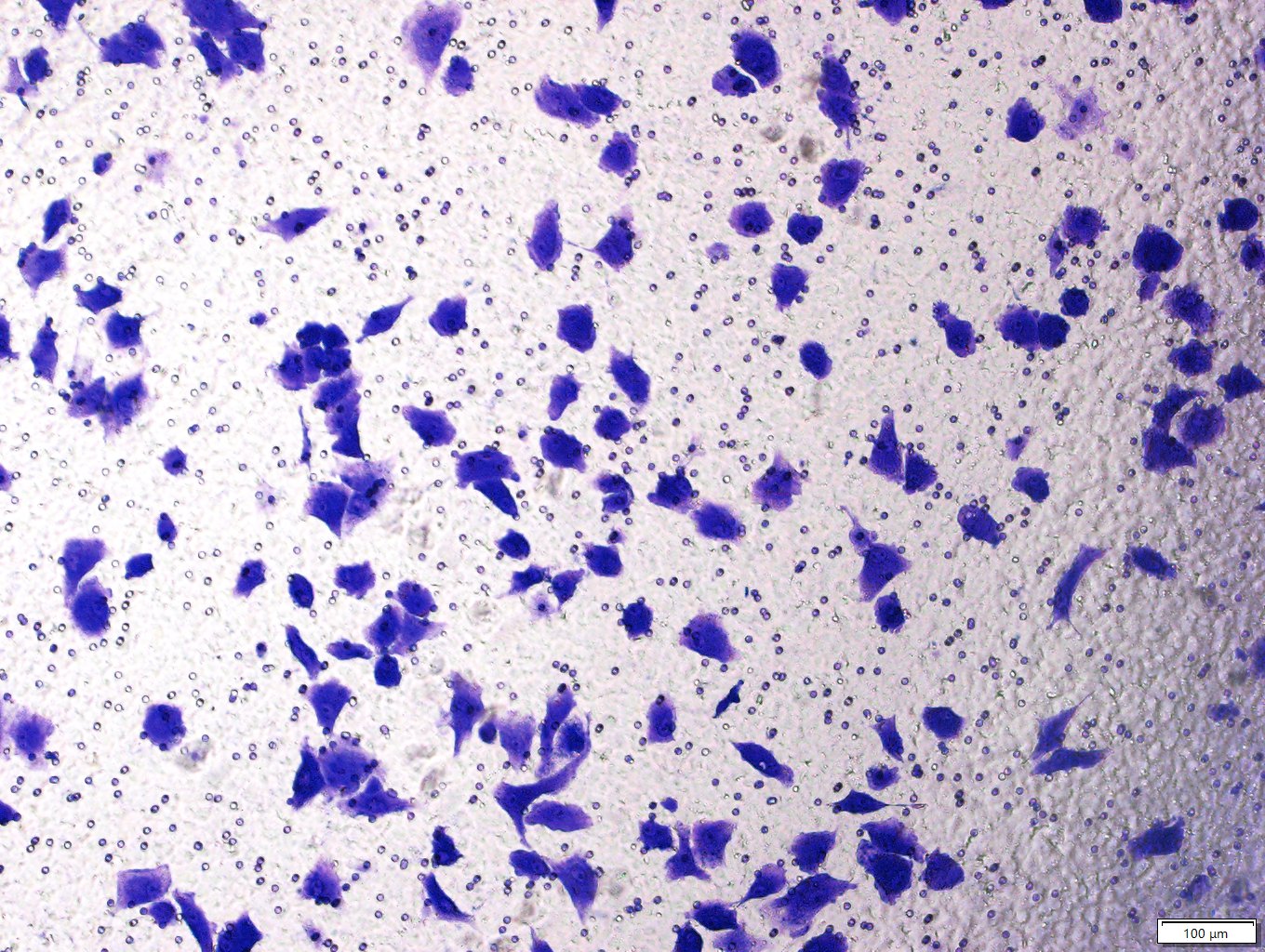

Supplement: Supplemental Information 9 [file peerj-cs-09-1651-s009.zip › Dataset 8/图像_1242.jpg]

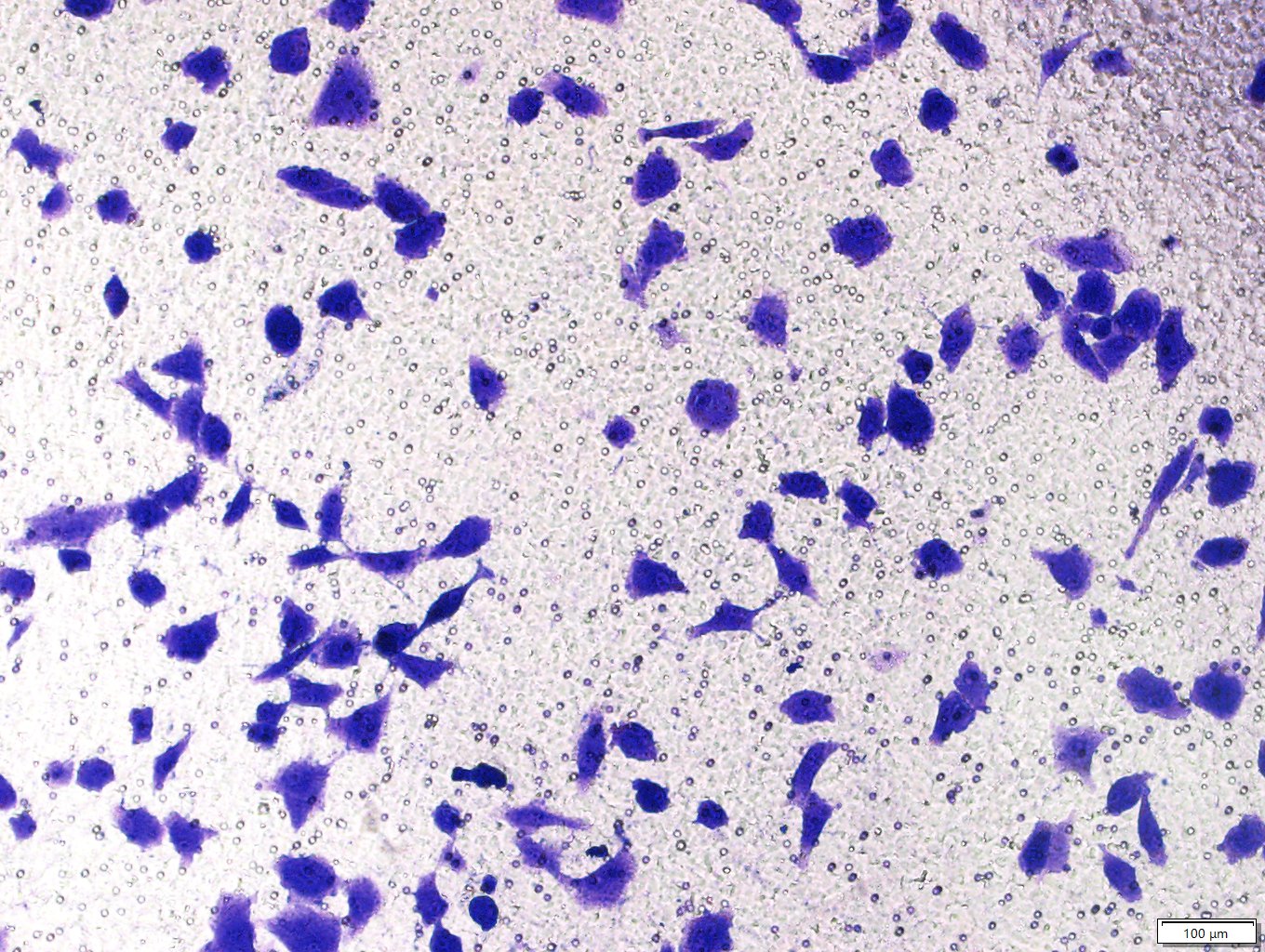

Supplement: Supplemental Information 9 [file peerj-cs-09-1651-s009.zip › Dataset 8/图像_1243.jpg]

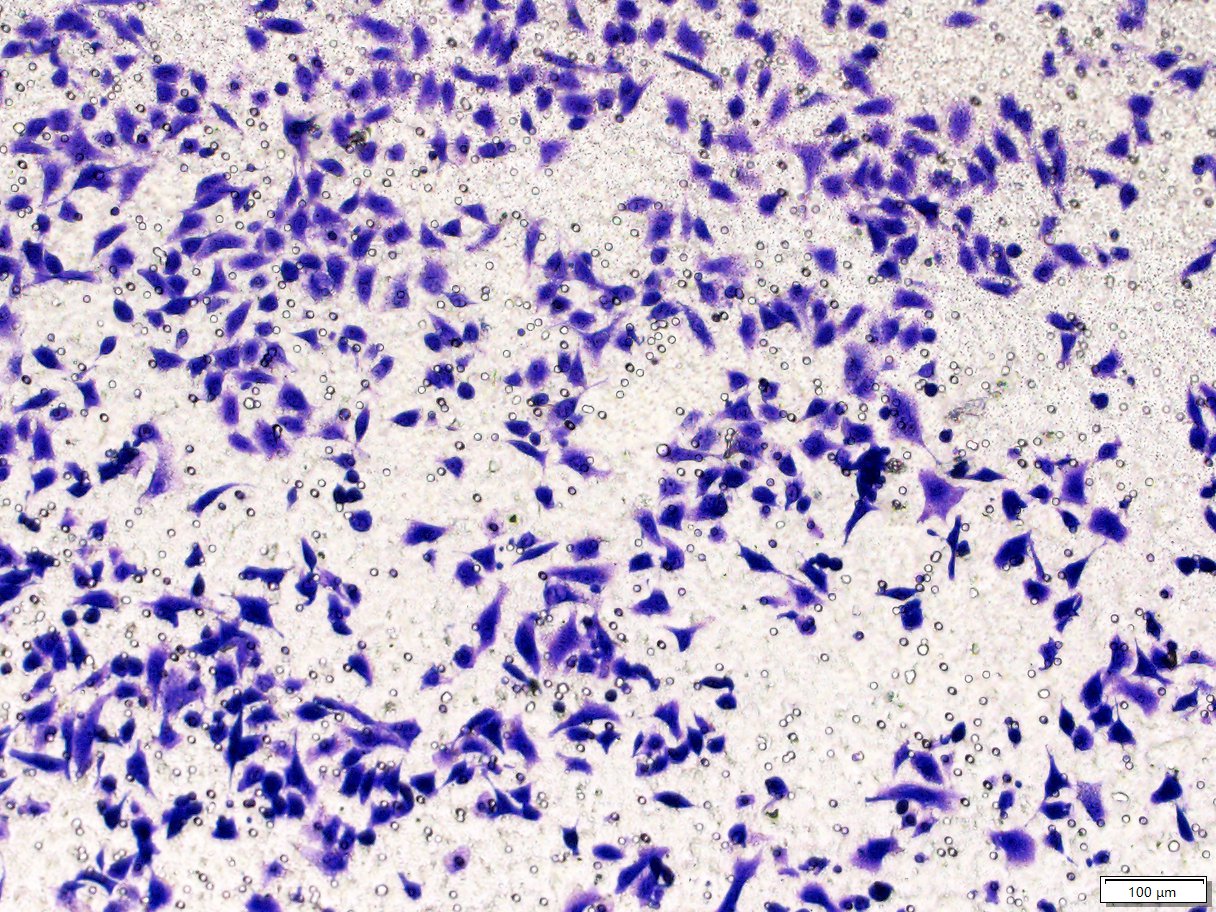

Supplement: Supplemental Information 10 [file peerj-cs-09-1651-s010.zip › Dataset 9/0+1.jpg]

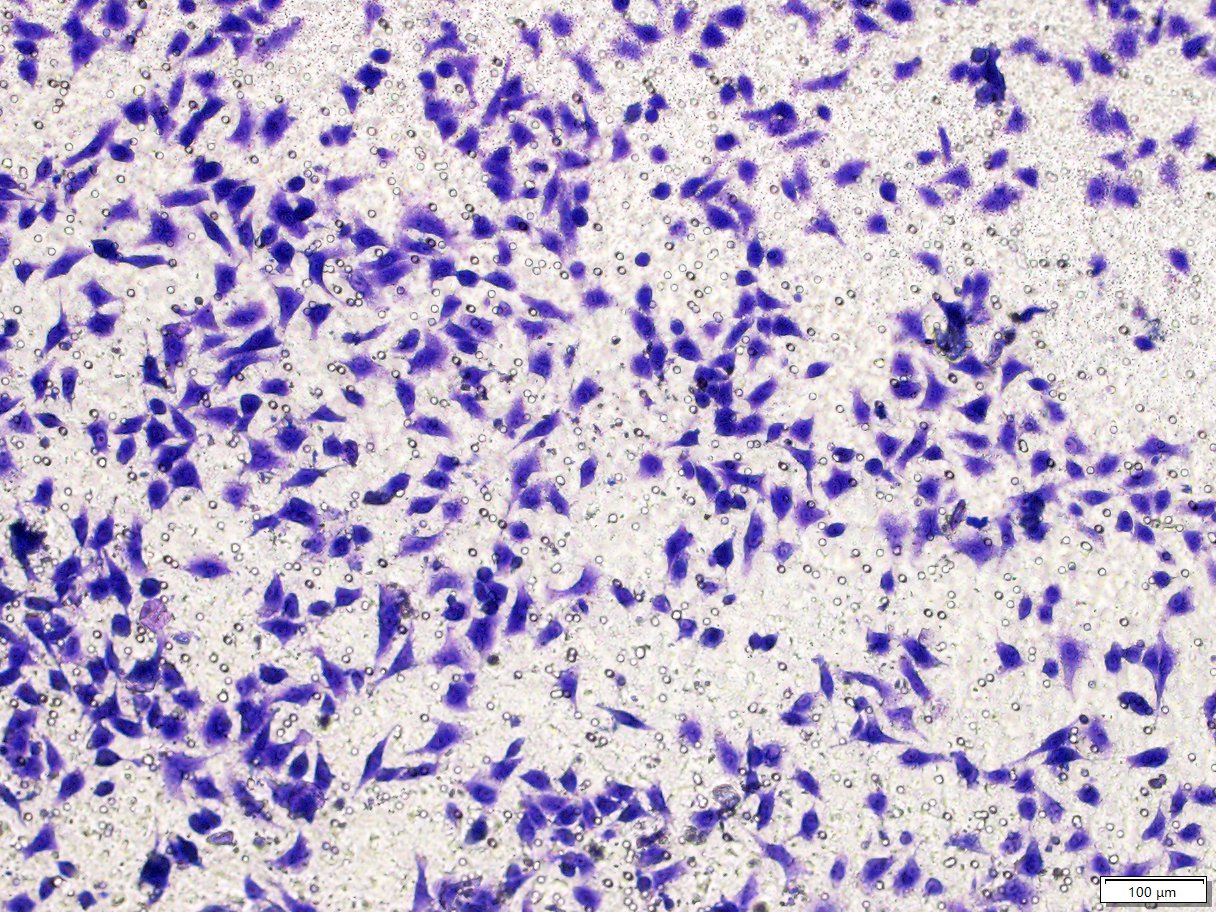

Supplement: Supplemental Information 10 [file peerj-cs-09-1651-s010.zip › Dataset 9/0+10.jpg]

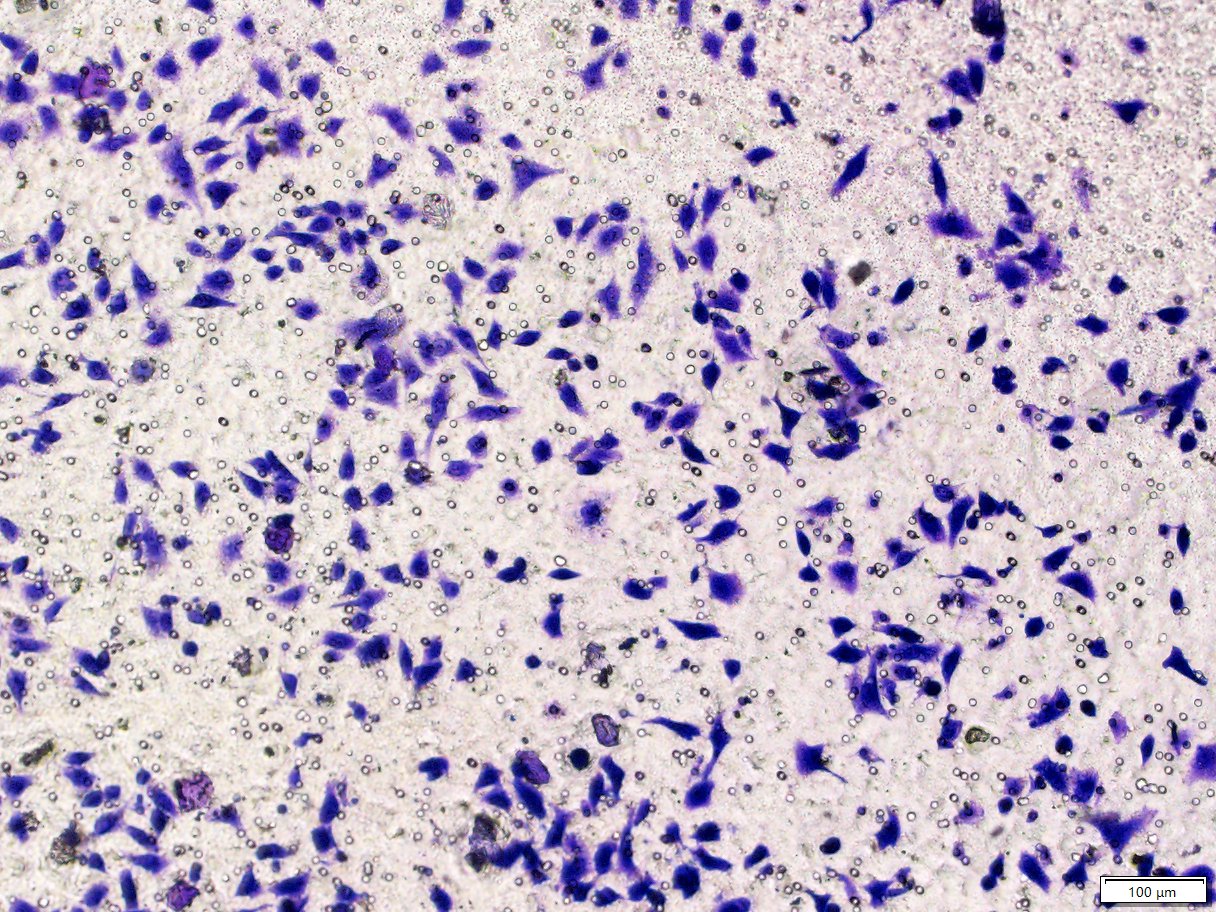

Supplement: Supplemental Information 10 [file peerj-cs-09-1651-s010.zip › Dataset 9/0+2.jpg]

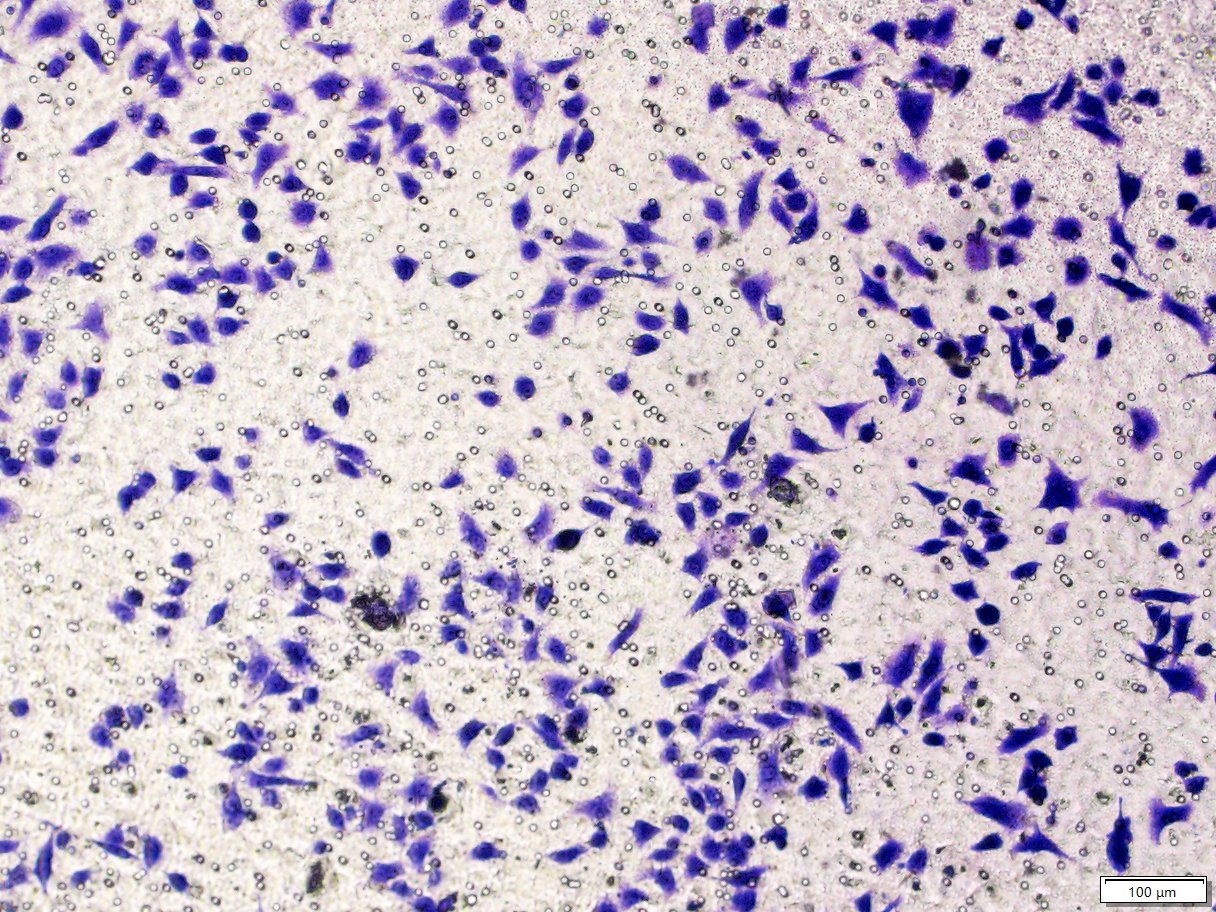

Supplement: Supplemental Information 10 [file peerj-cs-09-1651-s010.zip › Dataset 9/0+3.jpg]

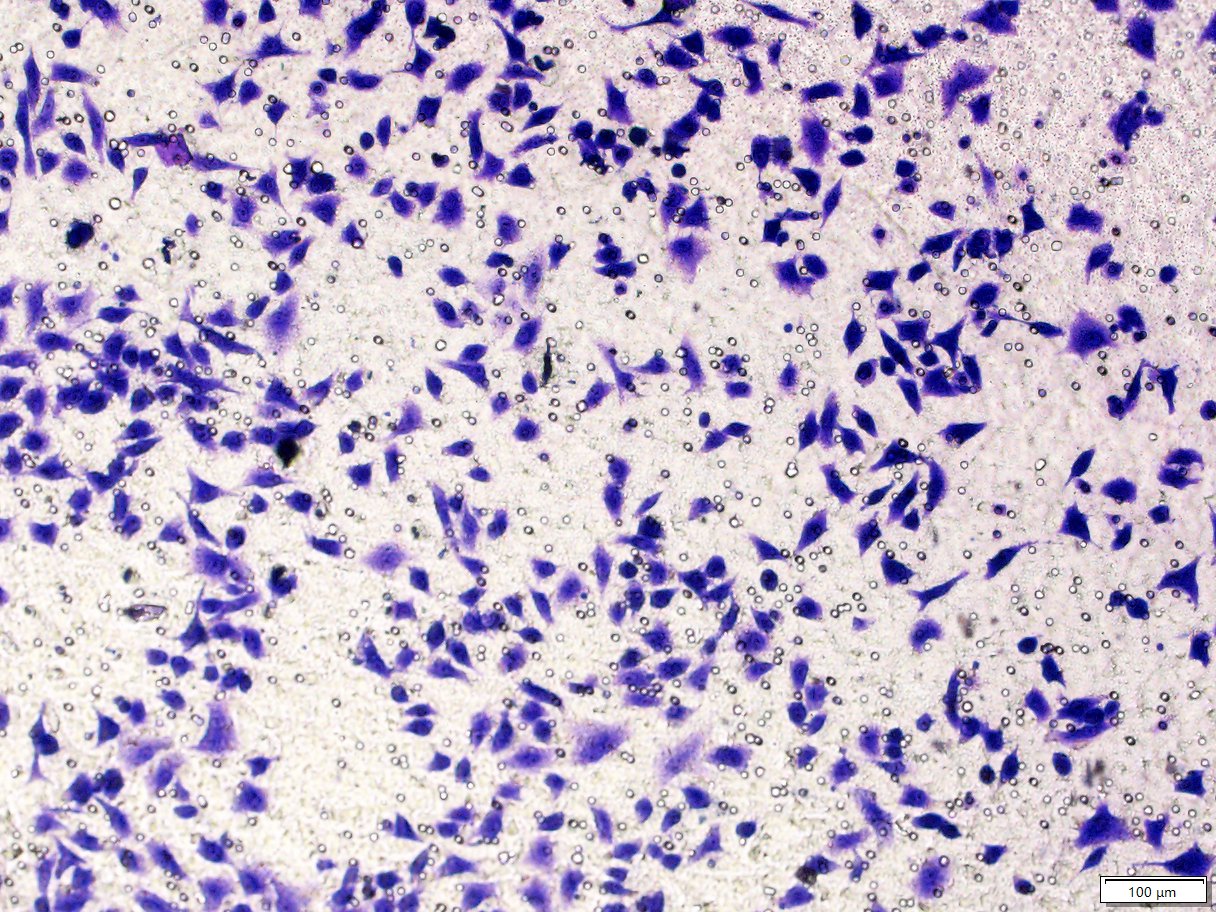

Supplement: Supplemental Information 10 [file peerj-cs-09-1651-s010.zip › Dataset 9/0+4.jpg]

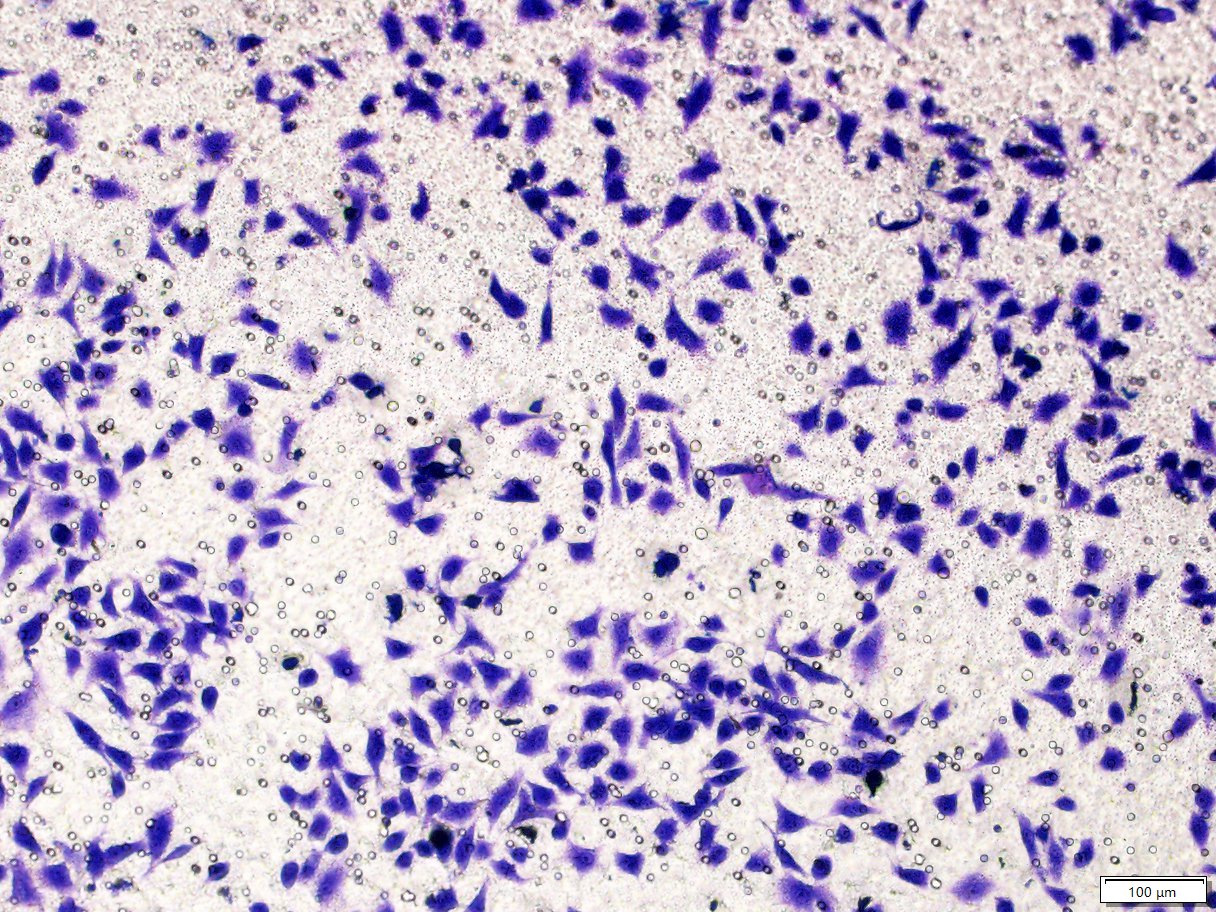

Supplement: Supplemental Information 10 [file peerj-cs-09-1651-s010.zip › Dataset 9/0+5.jpg]

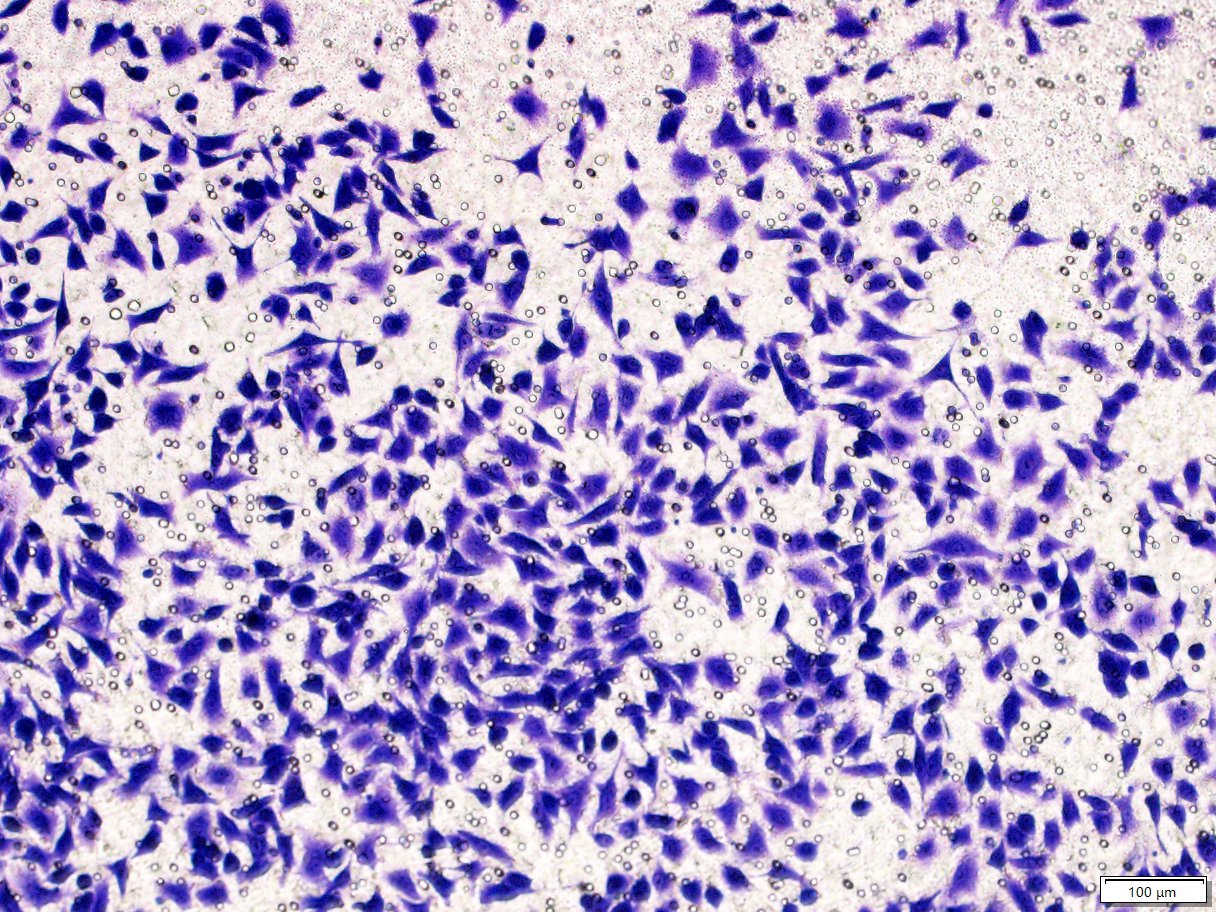

Supplement: Supplemental Information 10 [file peerj-cs-09-1651-s010.zip › Dataset 9/0+6.jpg]

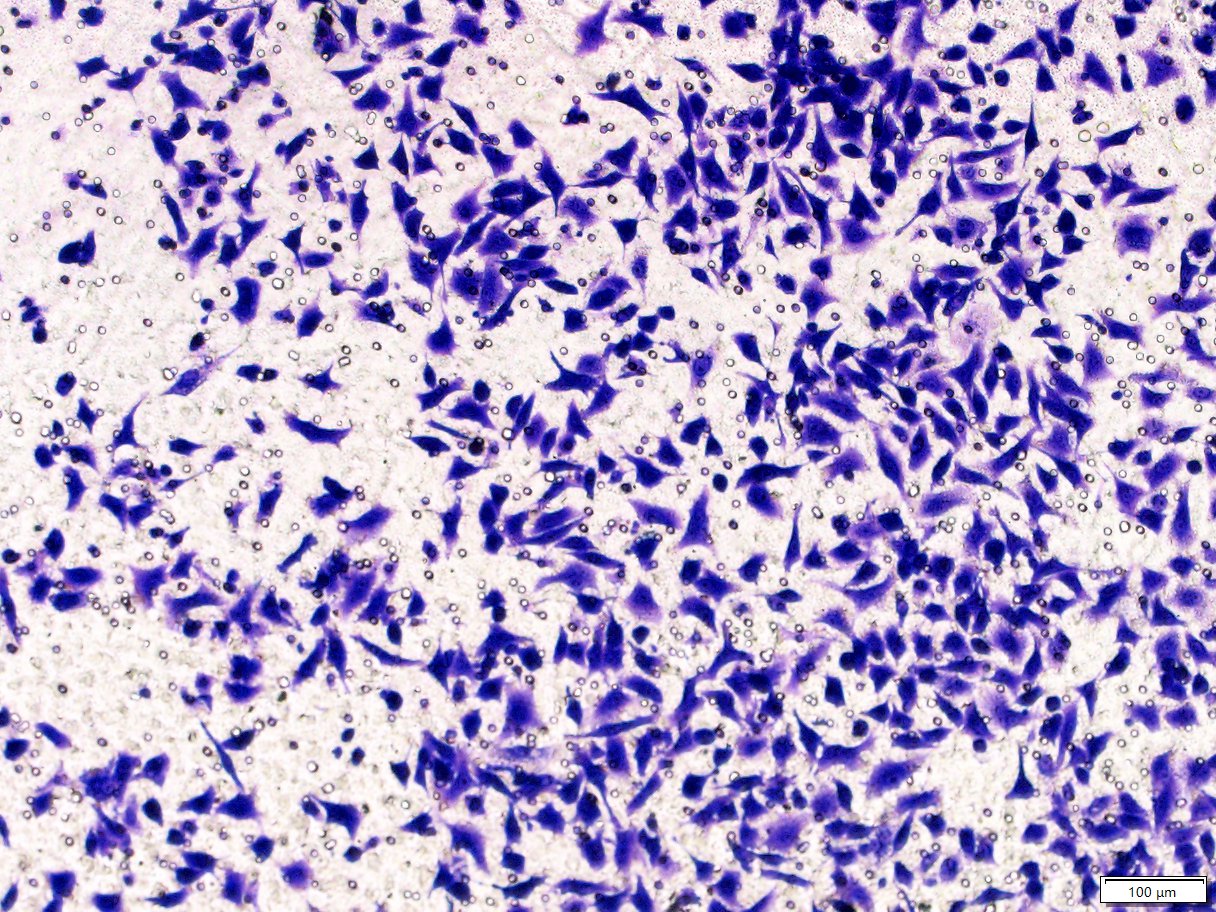

Supplement: Supplemental Information 10 [file peerj-cs-09-1651-s010.zip › Dataset 9/0+7.jpg]

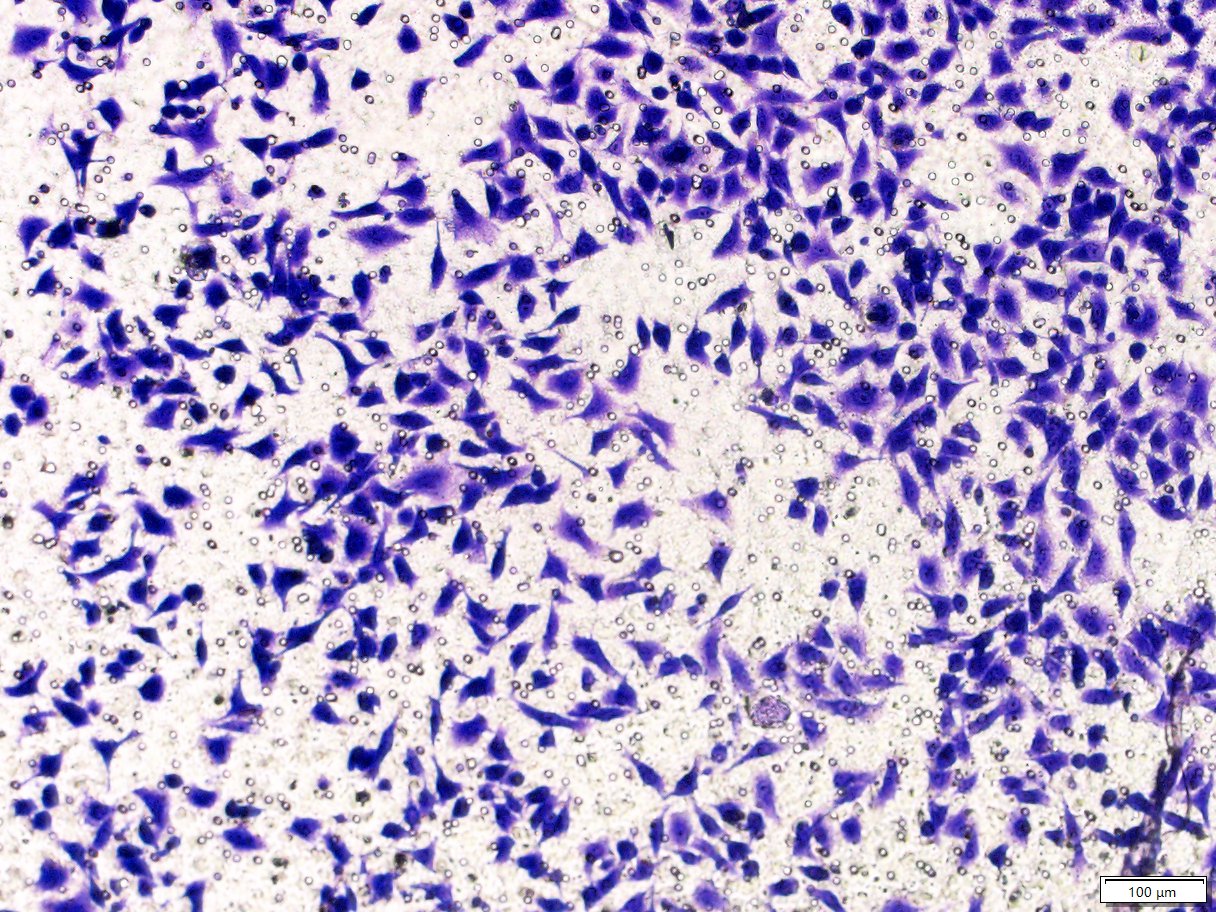

Supplement: Supplemental Information 10 [file peerj-cs-09-1651-s010.zip › Dataset 9/0+8.jpg]

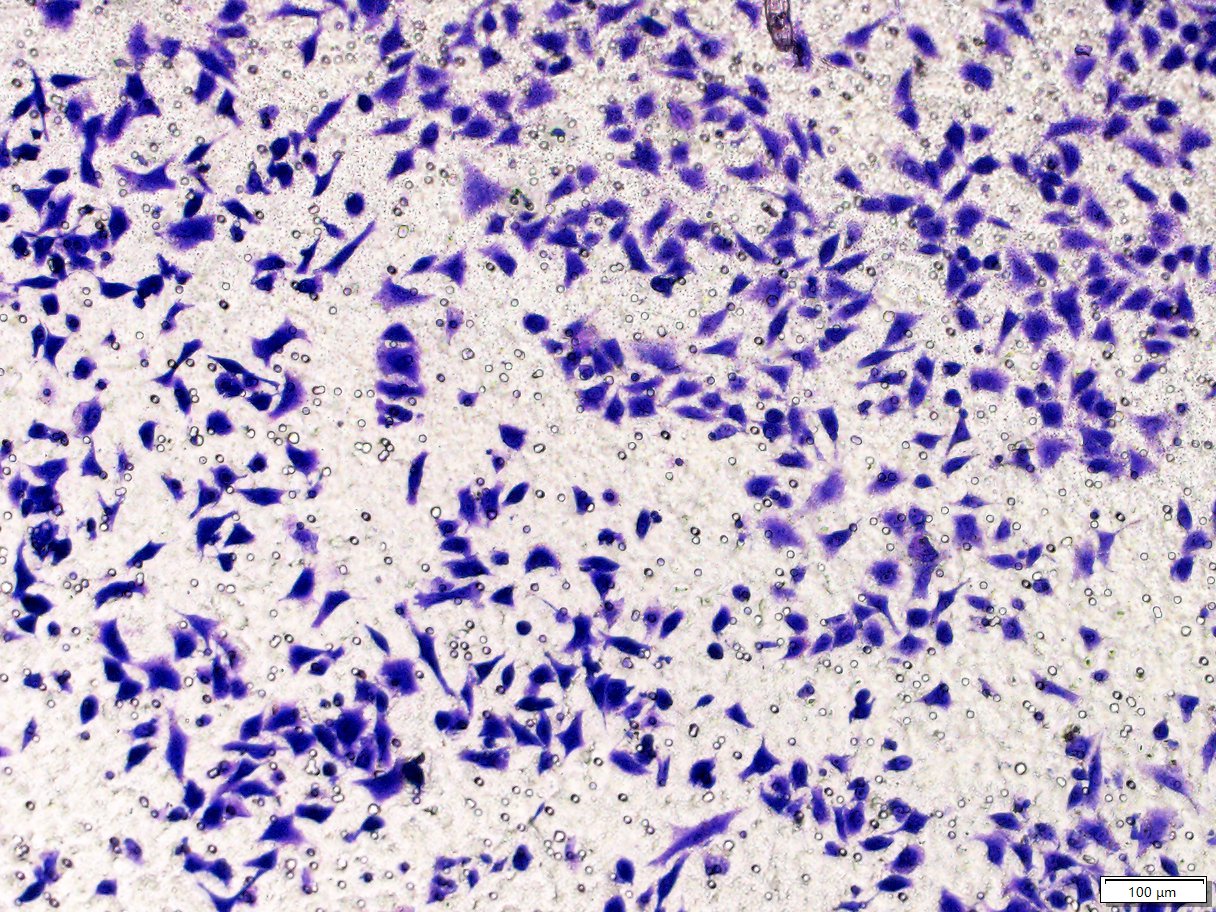

Supplement: Supplemental Information 10 [file peerj-cs-09-1651-s010.zip › Dataset 9/0+9.jpg]

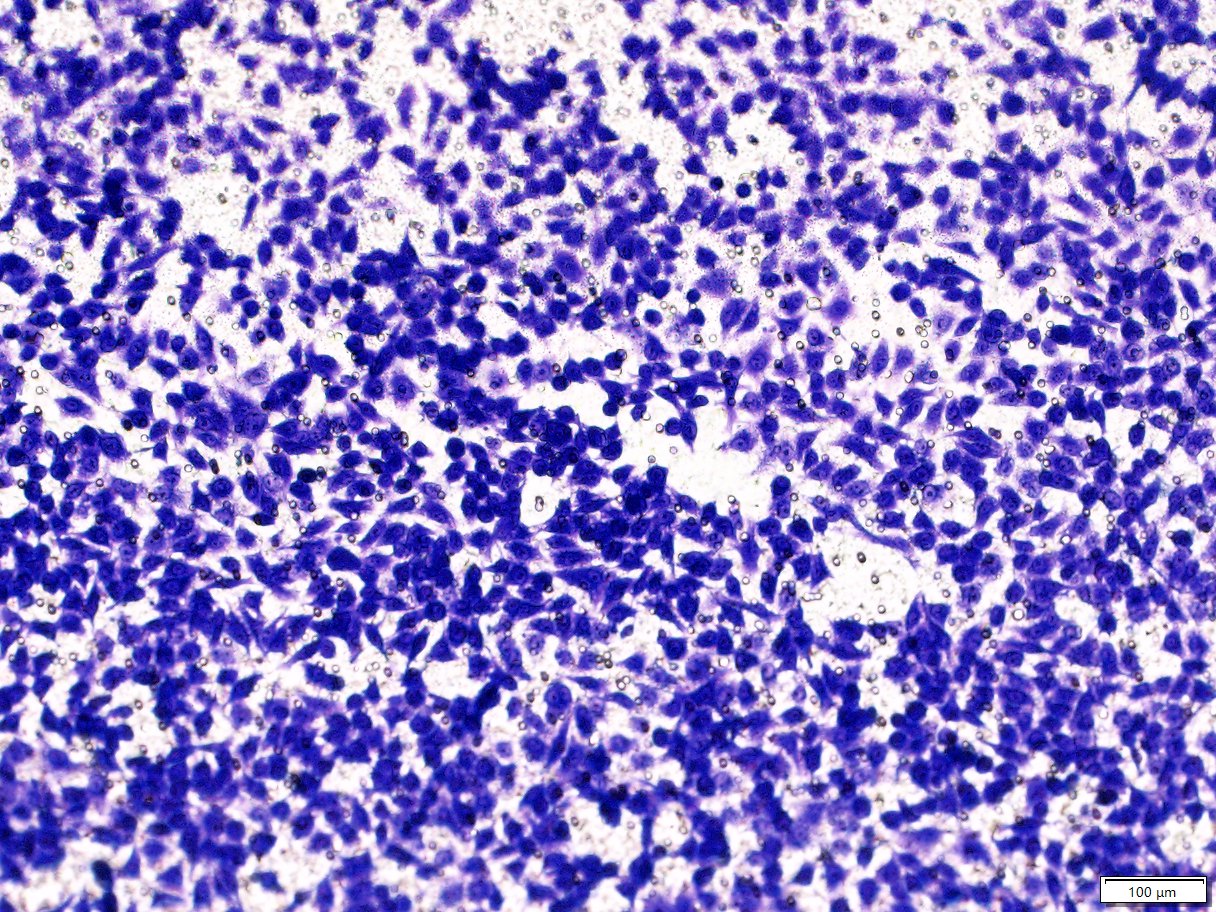

Supplement: Supplemental Information 10 [file peerj-cs-09-1651-s010.zip › Dataset 9/0-1.jpg]

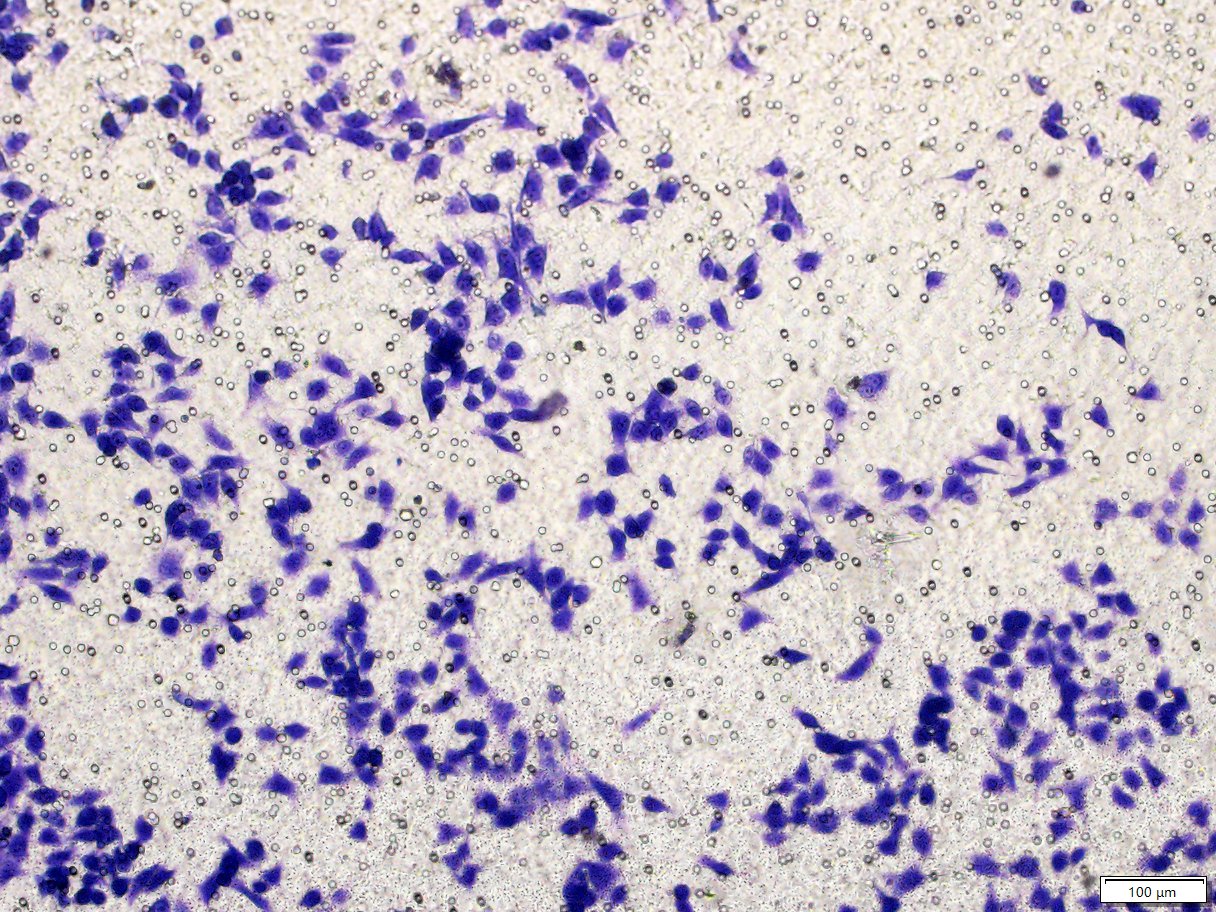

Supplement: Supplemental Information 10 [file peerj-cs-09-1651-s010.zip › Dataset 9/0-1íñ0.jpg]

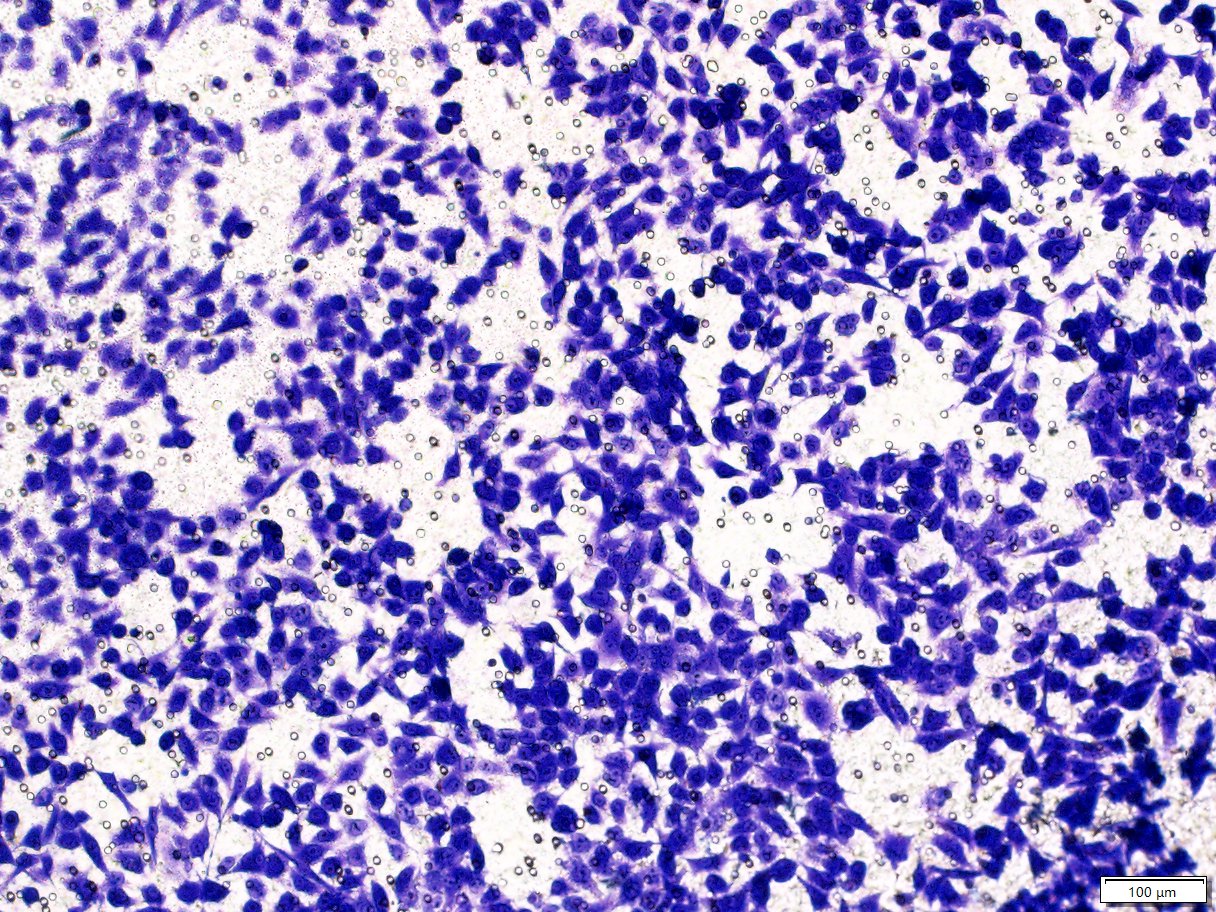

Supplement: Supplemental Information 10 [file peerj-cs-09-1651-s010.zip › Dataset 9/0-2.jpg]

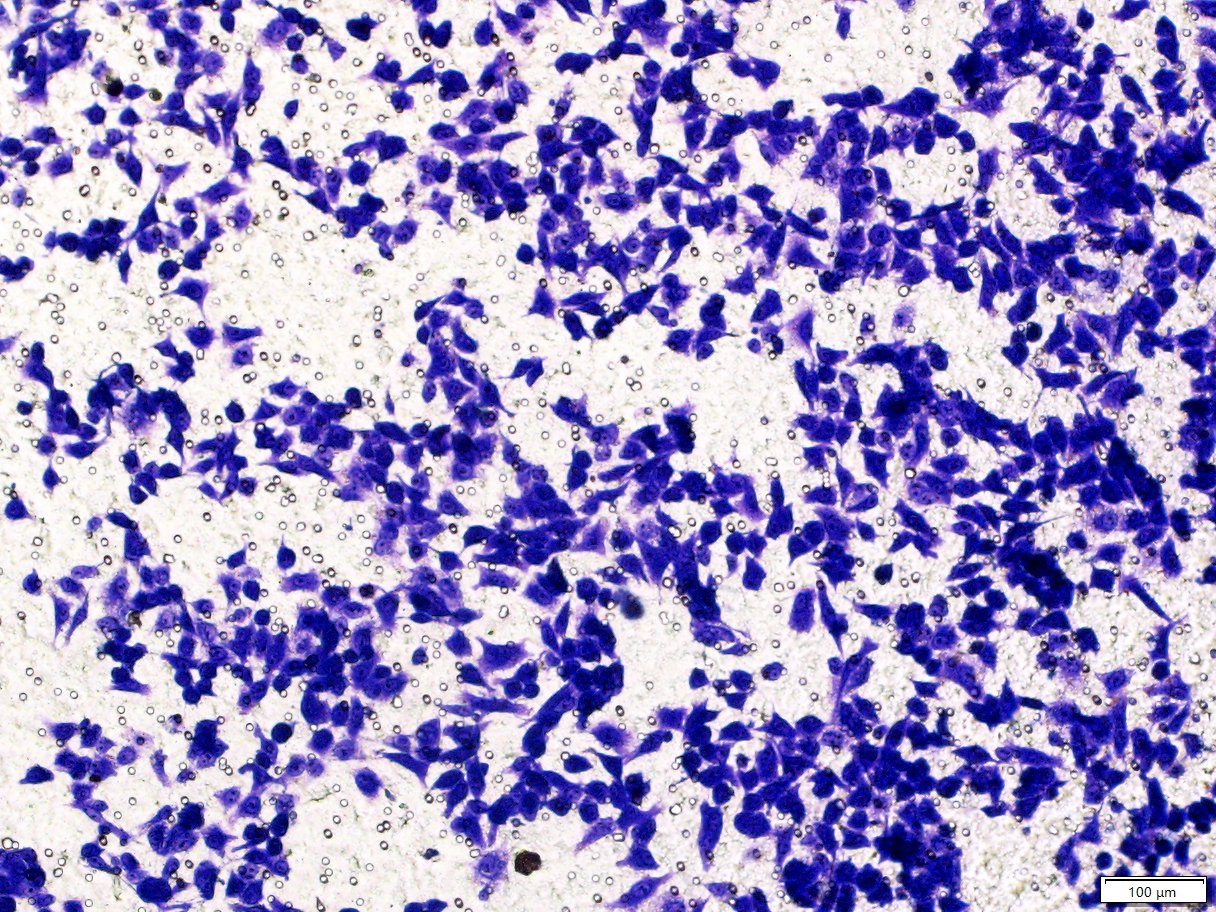

Supplement: Supplemental Information 10 [file peerj-cs-09-1651-s010.zip › Dataset 9/0-3.jpg]

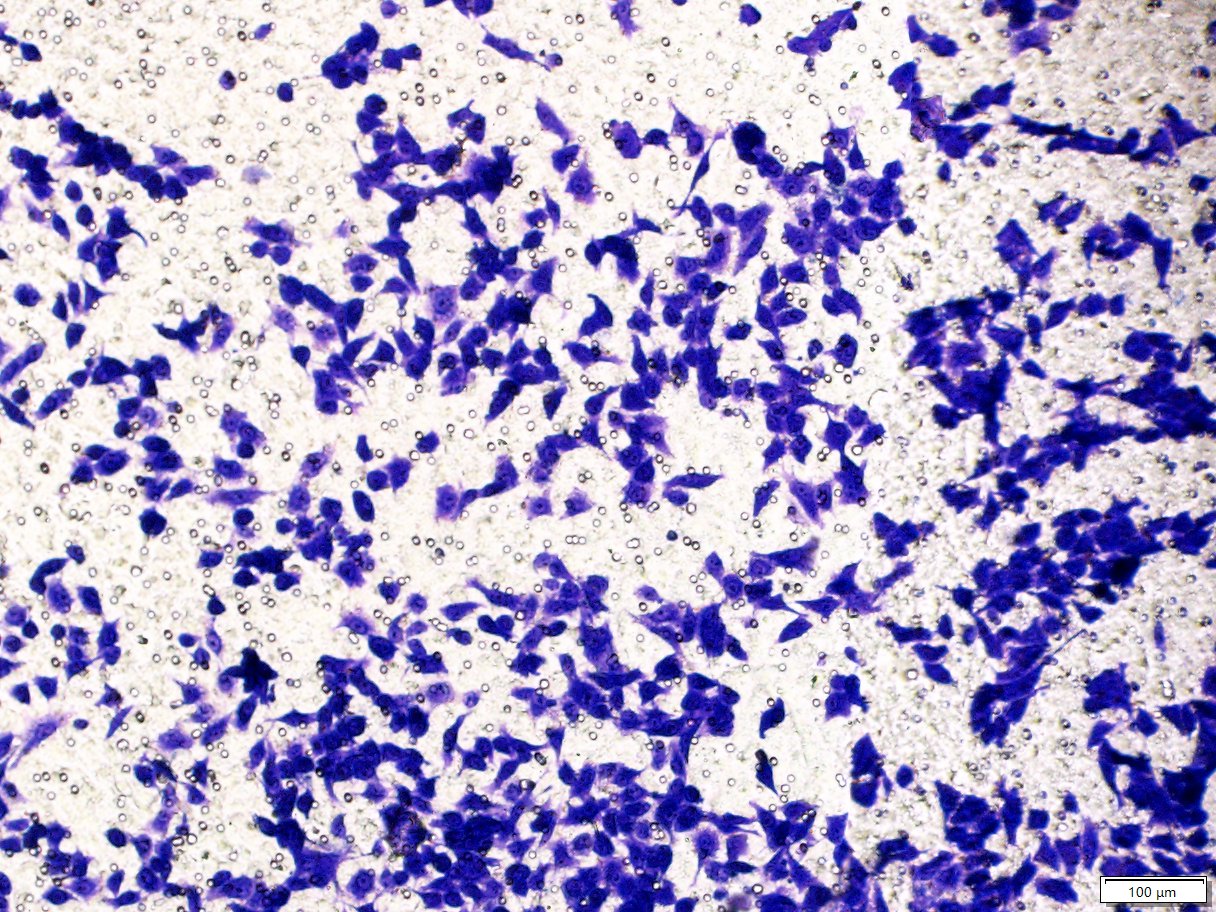

Supplement: Supplemental Information 10 [file peerj-cs-09-1651-s010.zip › Dataset 9/0-4.jpg]

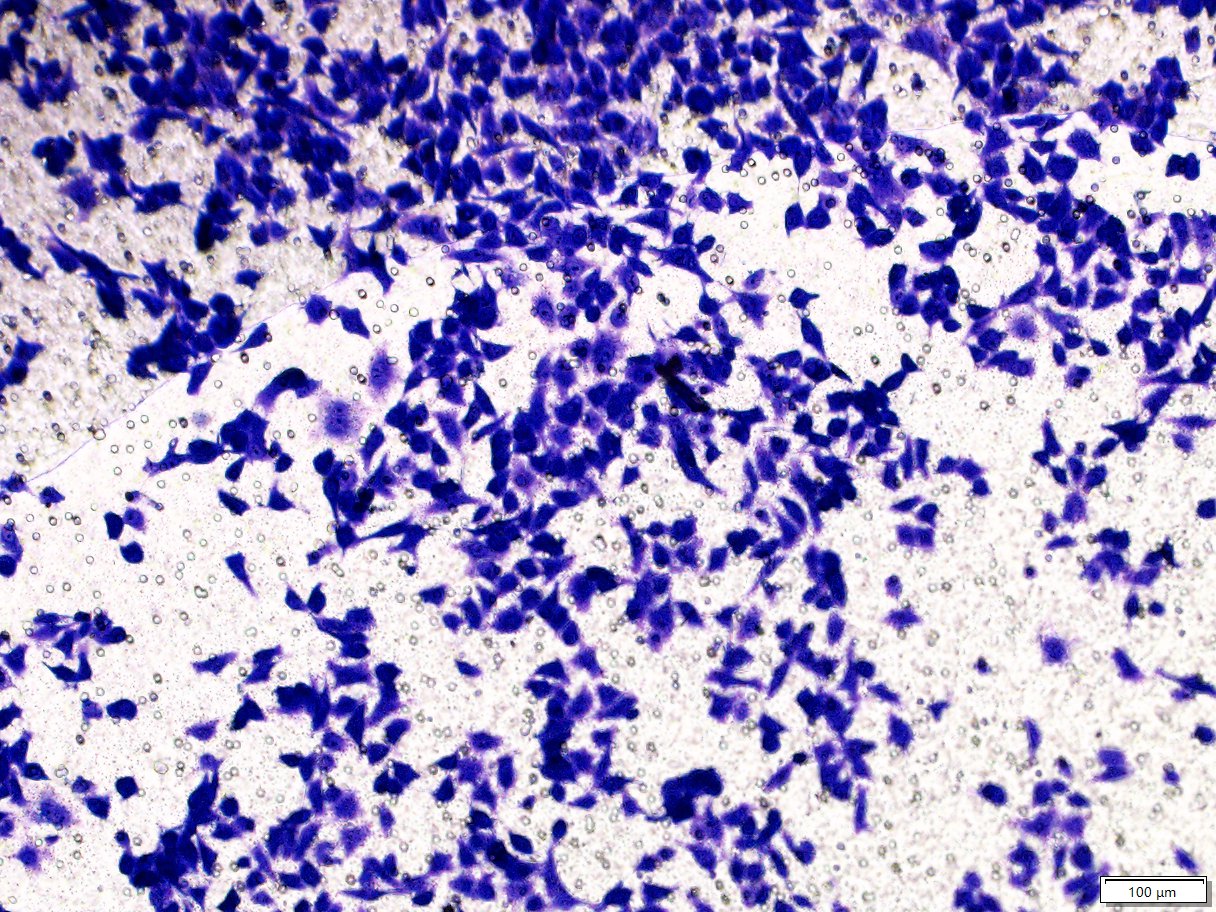

Supplement: Supplemental Information 10 [file peerj-cs-09-1651-s010.zip › Dataset 9/0-5.jpg]

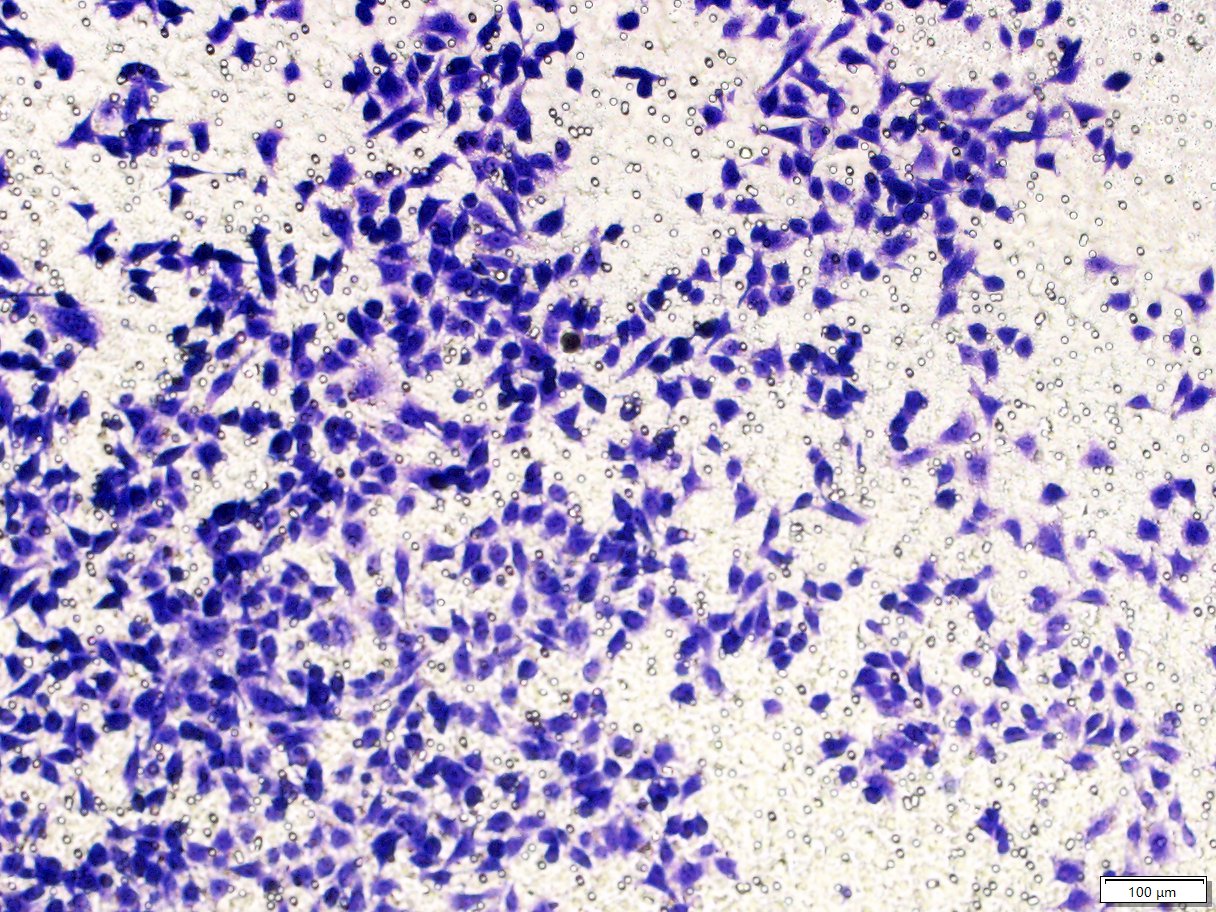

Supplement: Supplemental Information 10 [file peerj-cs-09-1651-s010.zip › Dataset 9/0-6.jpg]

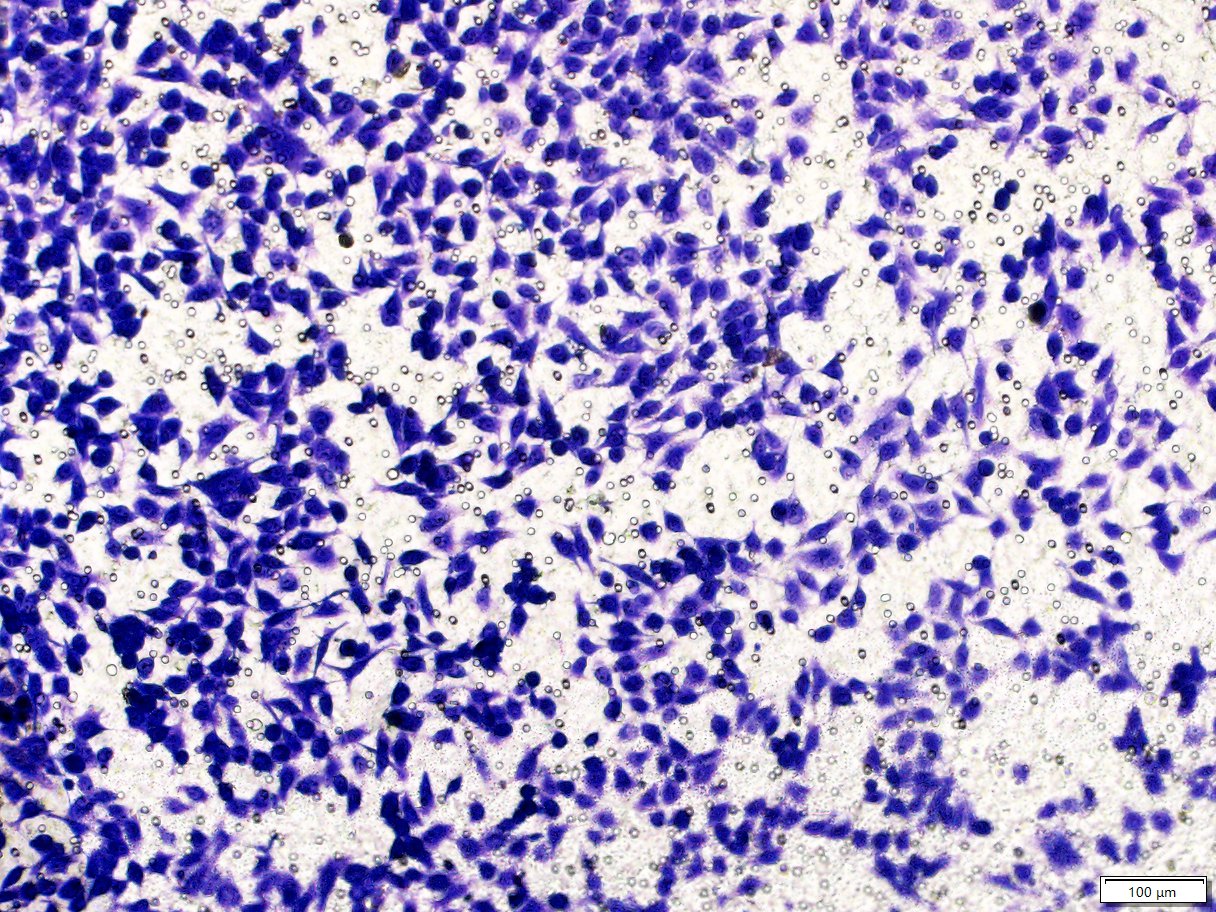

Supplement: Supplemental Information 10 [file peerj-cs-09-1651-s010.zip › Dataset 9/0-7.jpg]

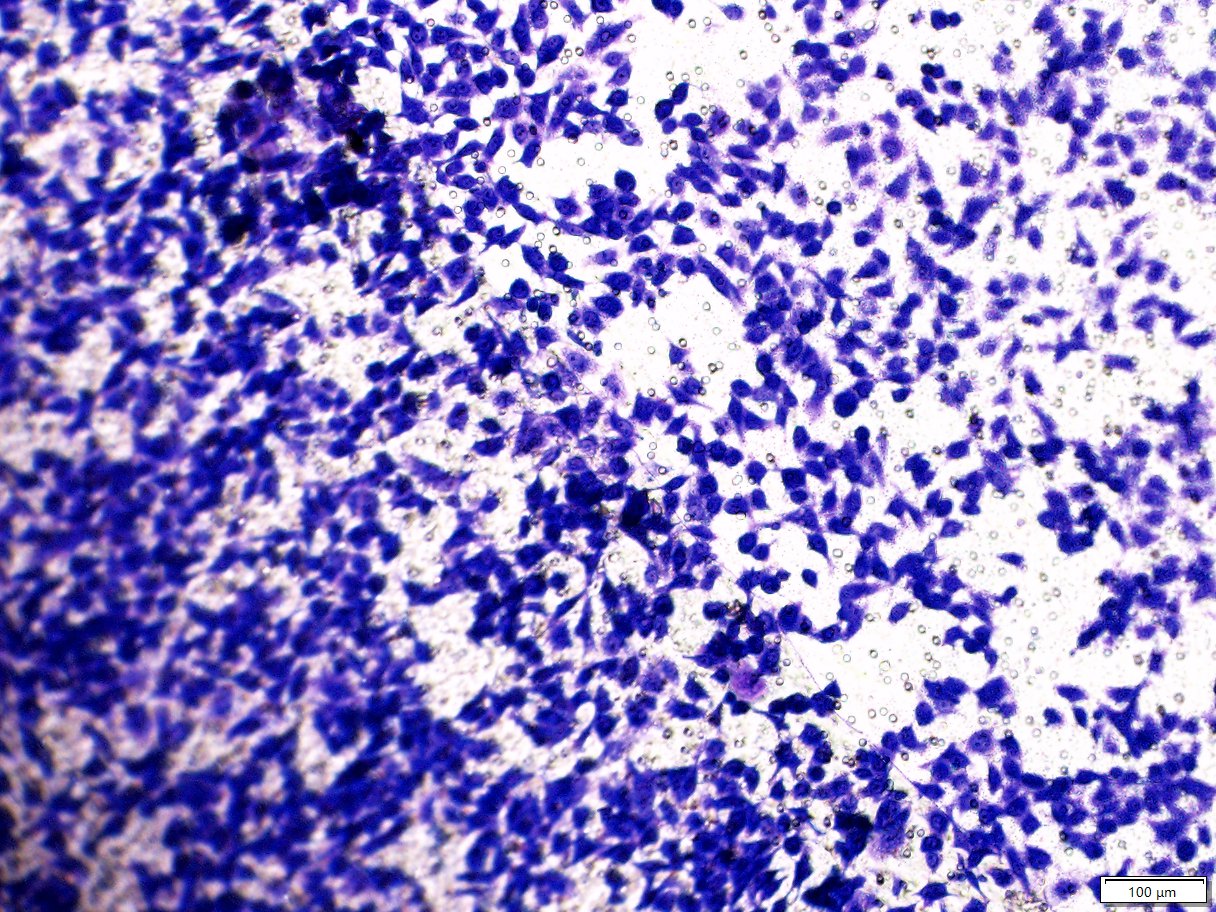

Supplement: Supplemental Information 10 [file peerj-cs-09-1651-s010.zip › Dataset 9/0-8.jpg]

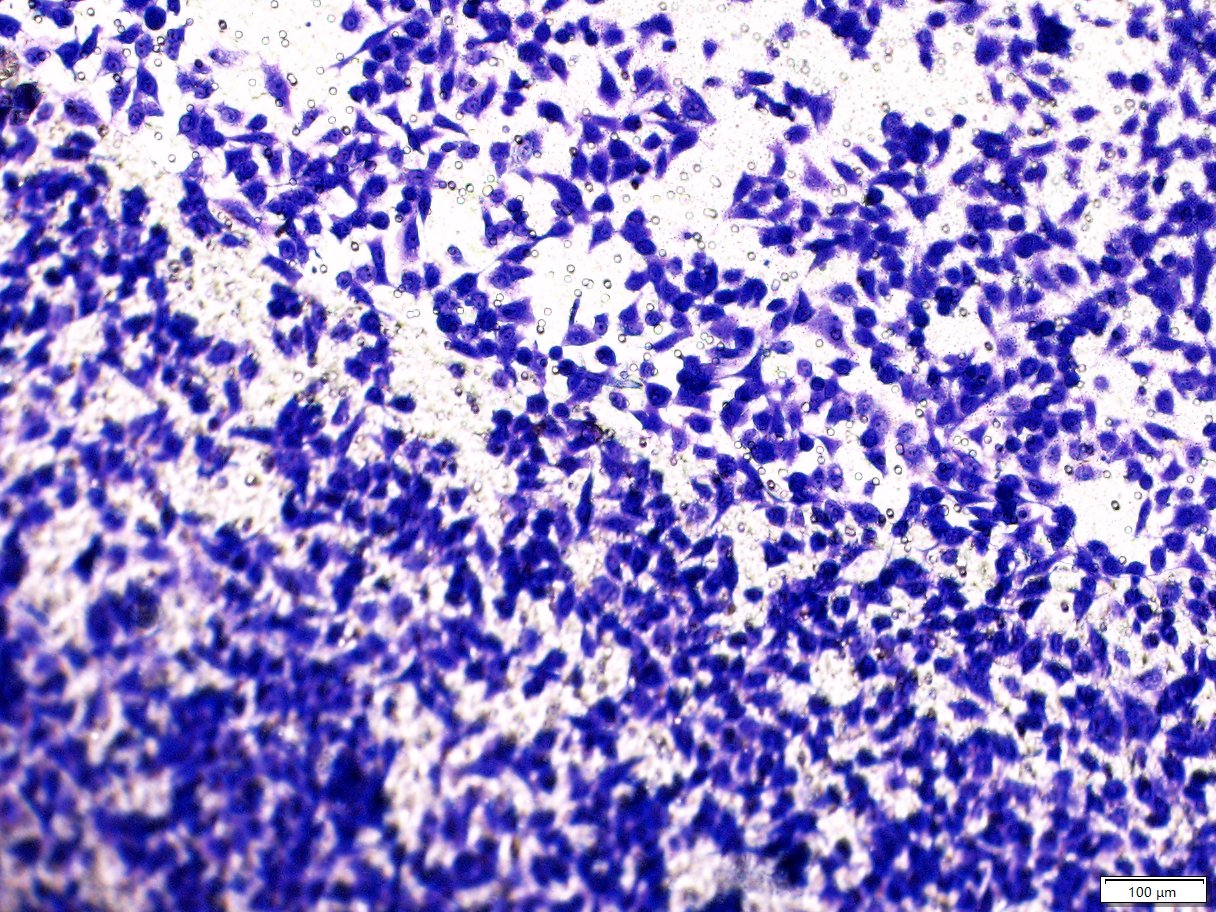

Supplement: Supplemental Information 10 [file peerj-cs-09-1651-s010.zip › Dataset 9/0-9.jpg]

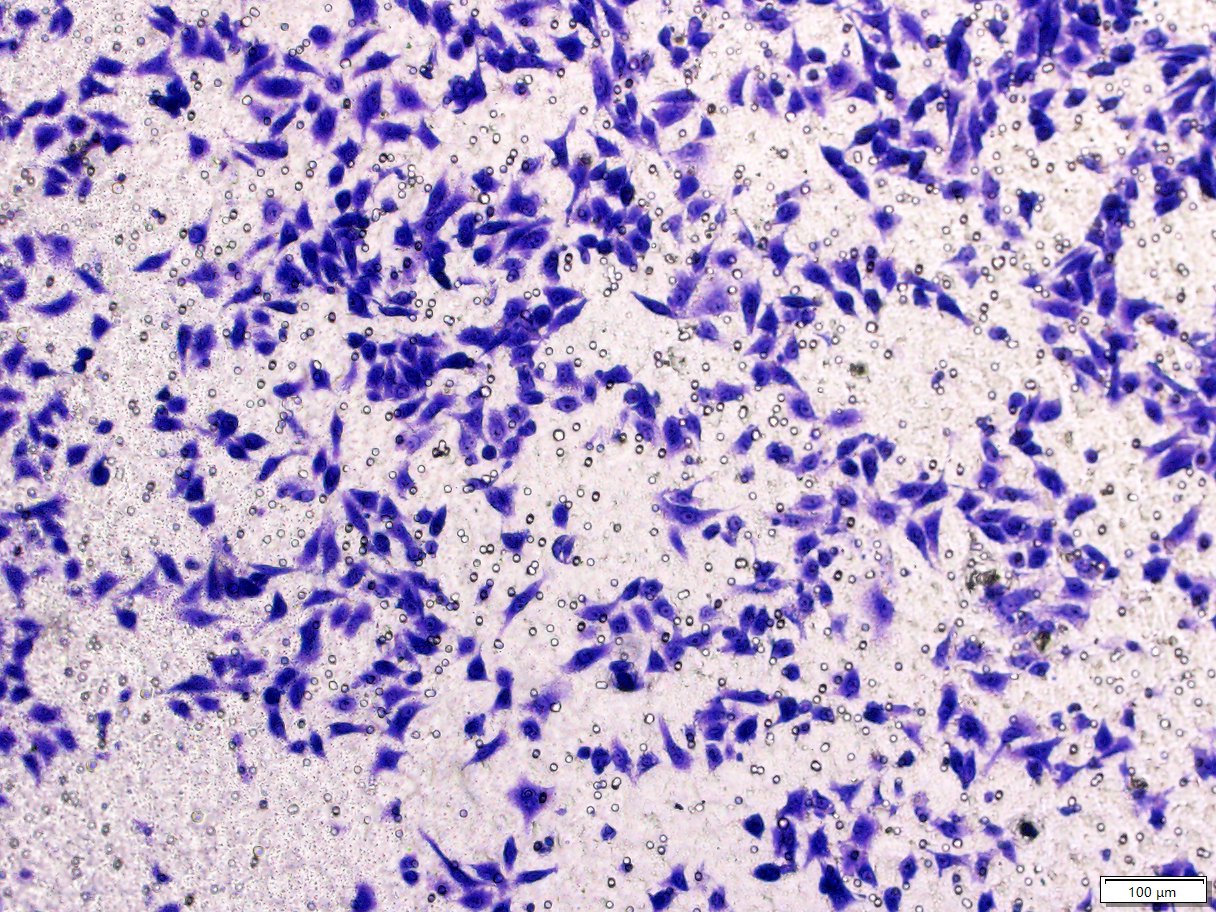

Supplement: Supplemental Information 10 [file peerj-cs-09-1651-s010.zip › Dataset 9/1+1.jpg]

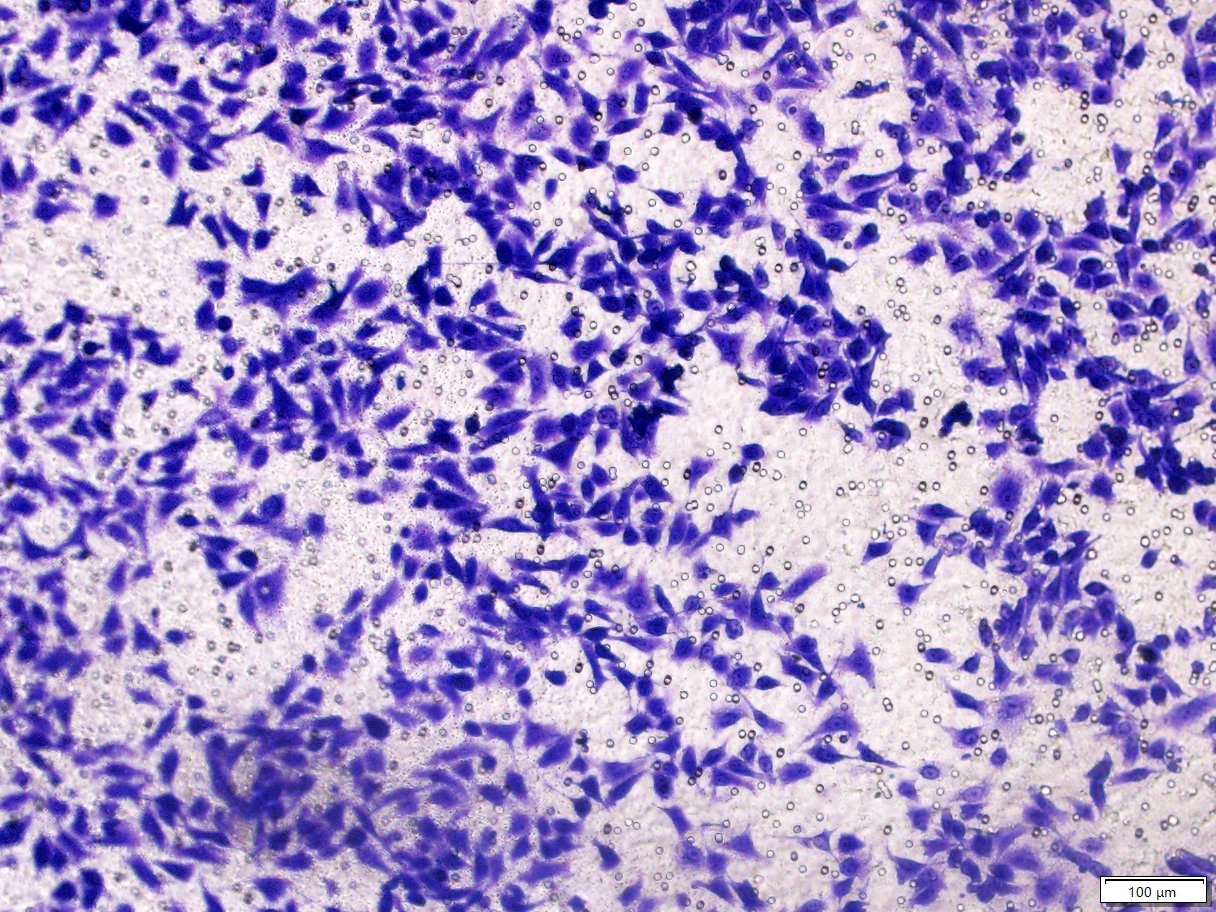

Supplement: Supplemental Information 10 [file peerj-cs-09-1651-s010.zip › Dataset 9/1+10.jpg]

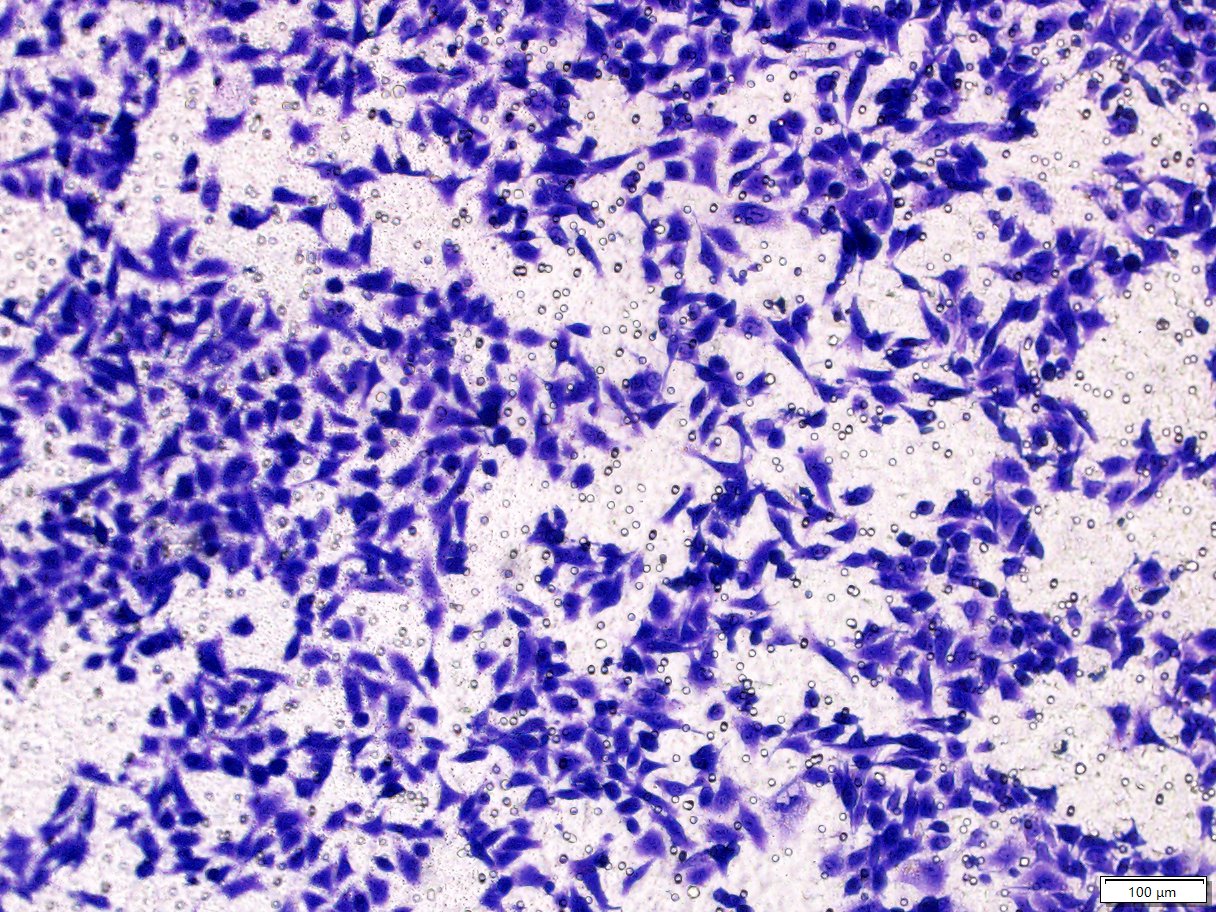

Supplement: Supplemental Information 10 [file peerj-cs-09-1651-s010.zip › Dataset 9/1+11.jpg]

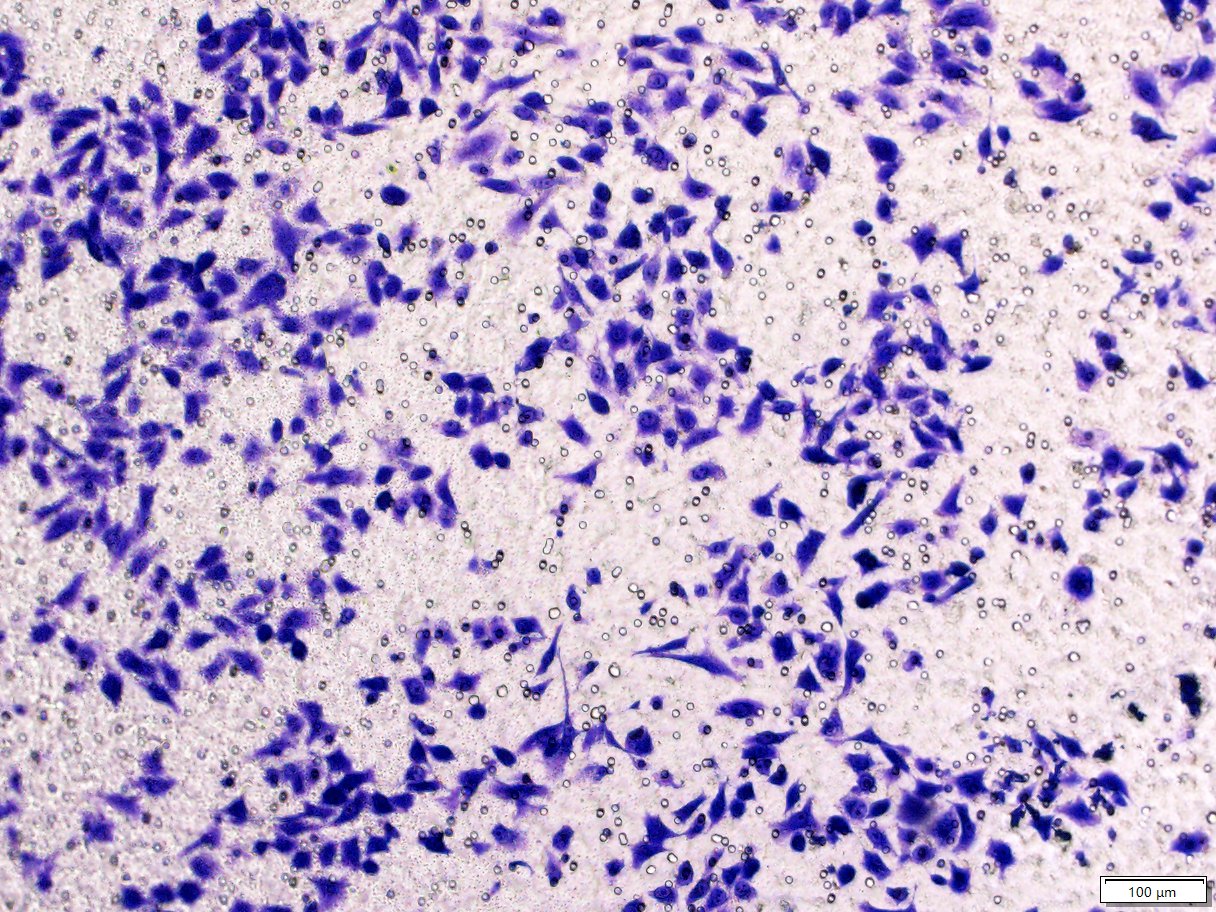

Supplement: Supplemental Information 10 [file peerj-cs-09-1651-s010.zip › Dataset 9/1+2.jpg]

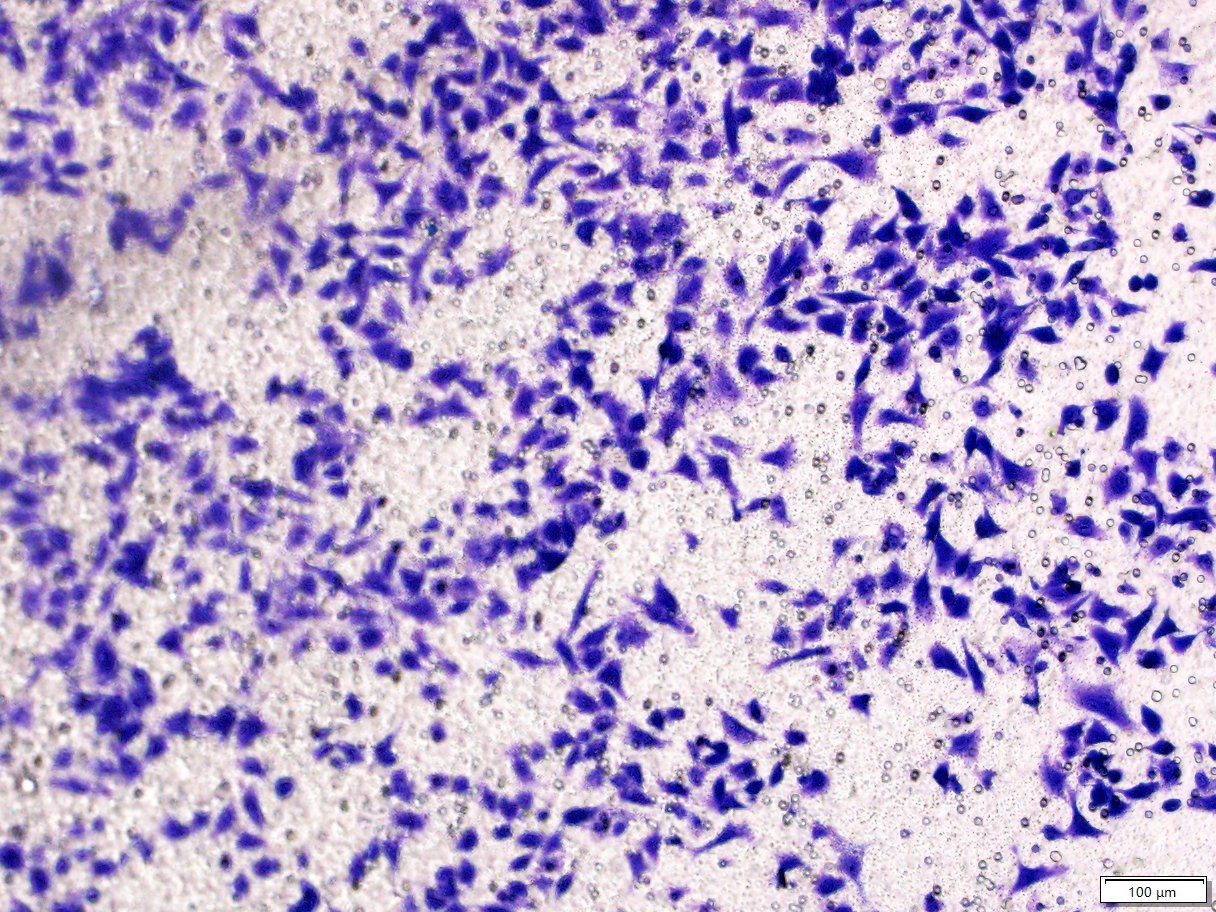

Supplement: Supplemental Information 10 [file peerj-cs-09-1651-s010.zip › Dataset 9/1+3.jpg]

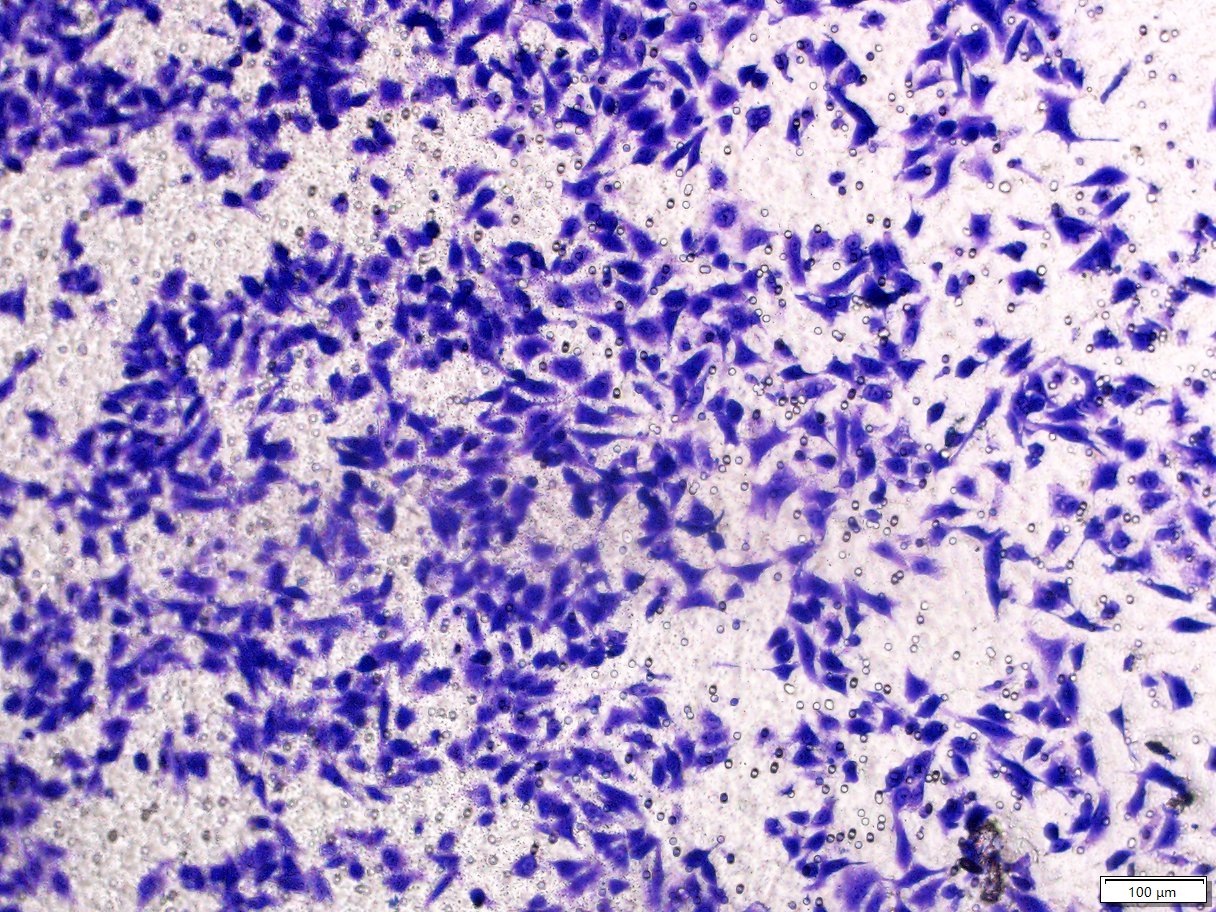

Supplement: Supplemental Information 10 [file peerj-cs-09-1651-s010.zip › Dataset 9/1+4.jpg]

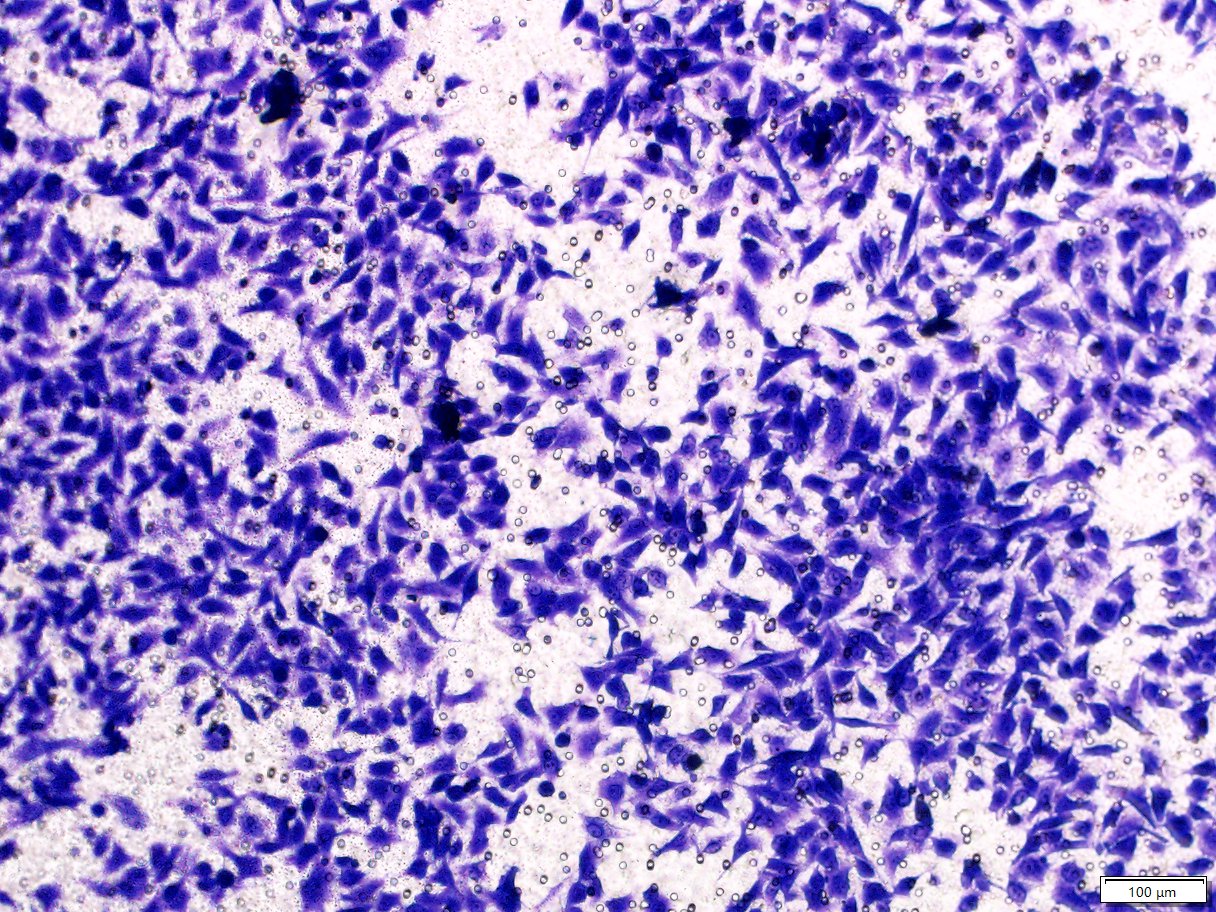

Supplement: Supplemental Information 10 [file peerj-cs-09-1651-s010.zip › Dataset 9/1+5.jpg]

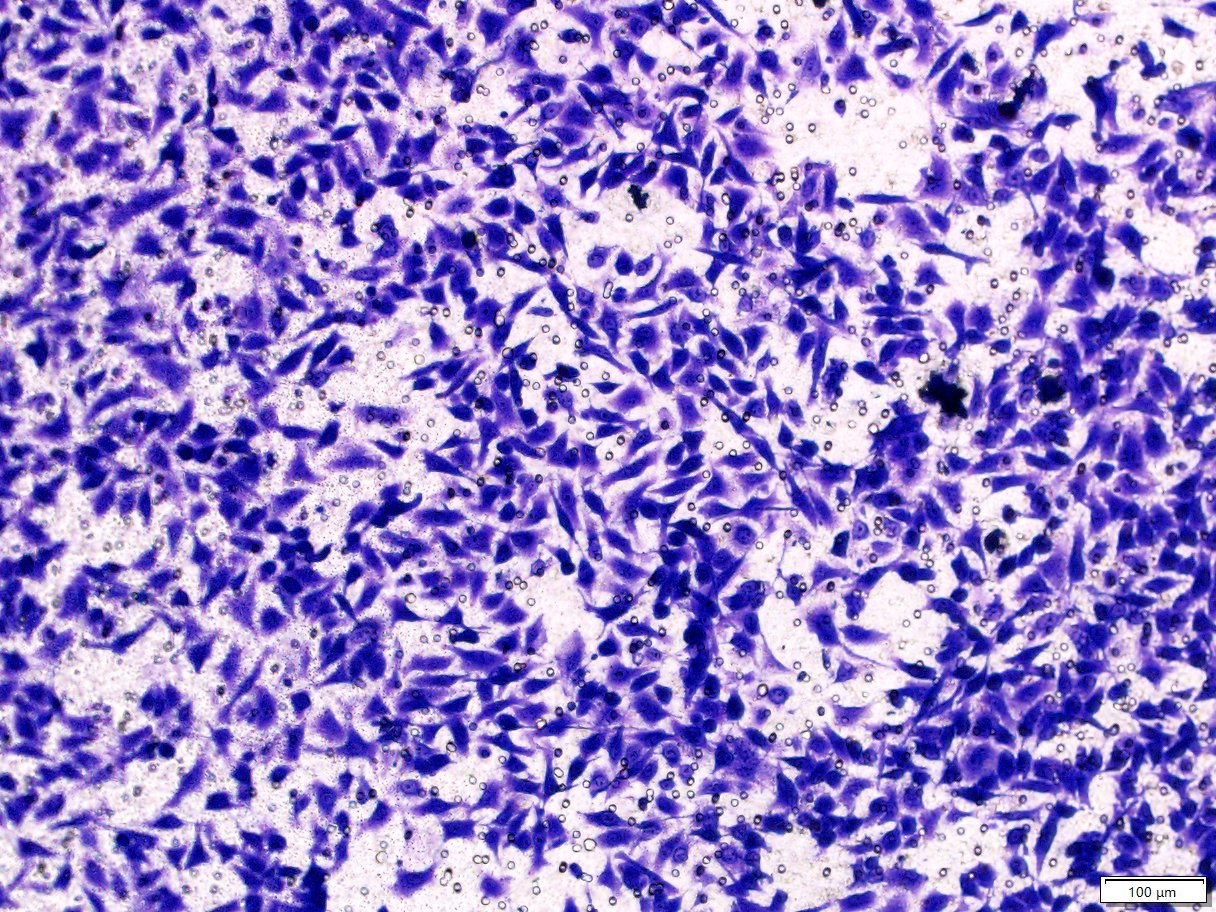

Supplement: Supplemental Information 10 [file peerj-cs-09-1651-s010.zip › Dataset 9/1+6.jpg]

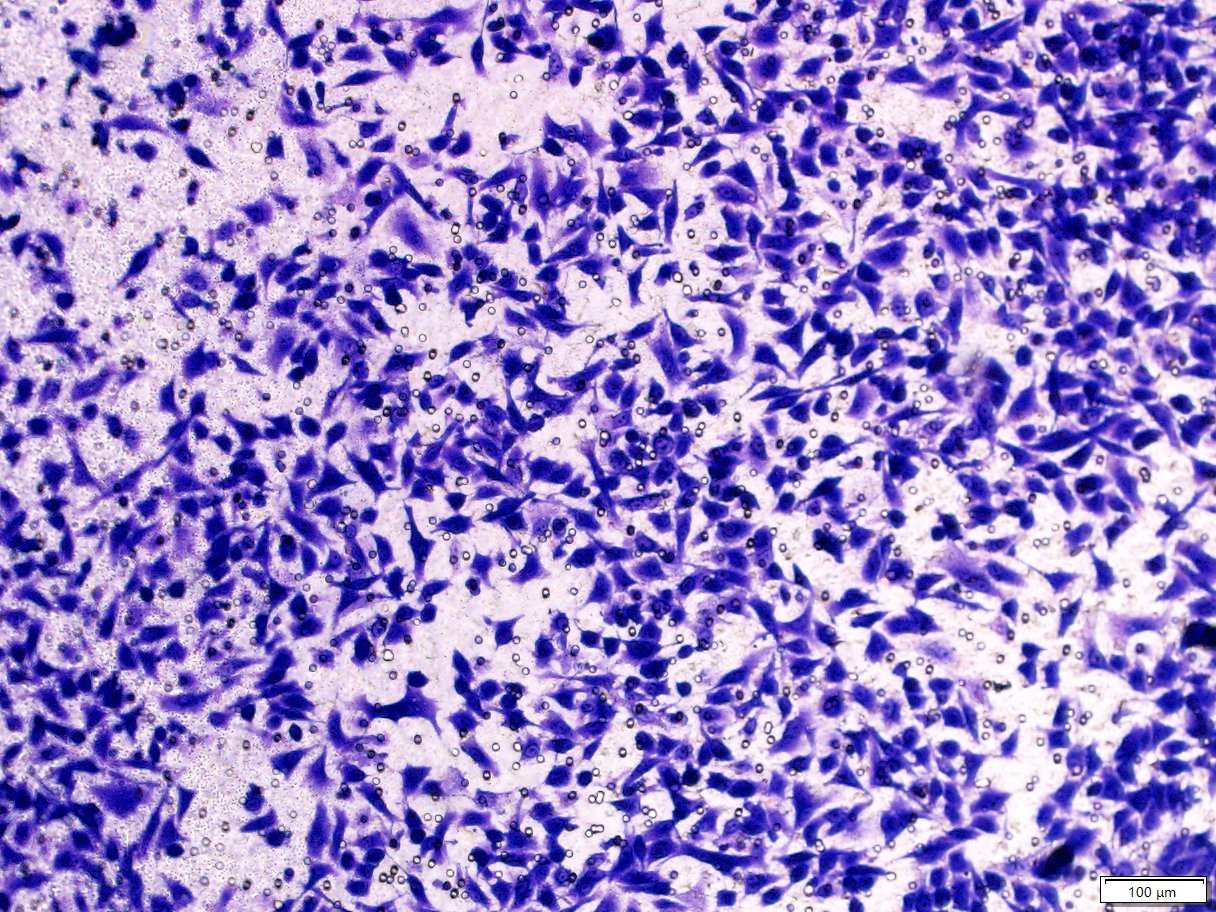

Supplement: Supplemental Information 10 [file peerj-cs-09-1651-s010.zip › Dataset 9/1+7.jpg]

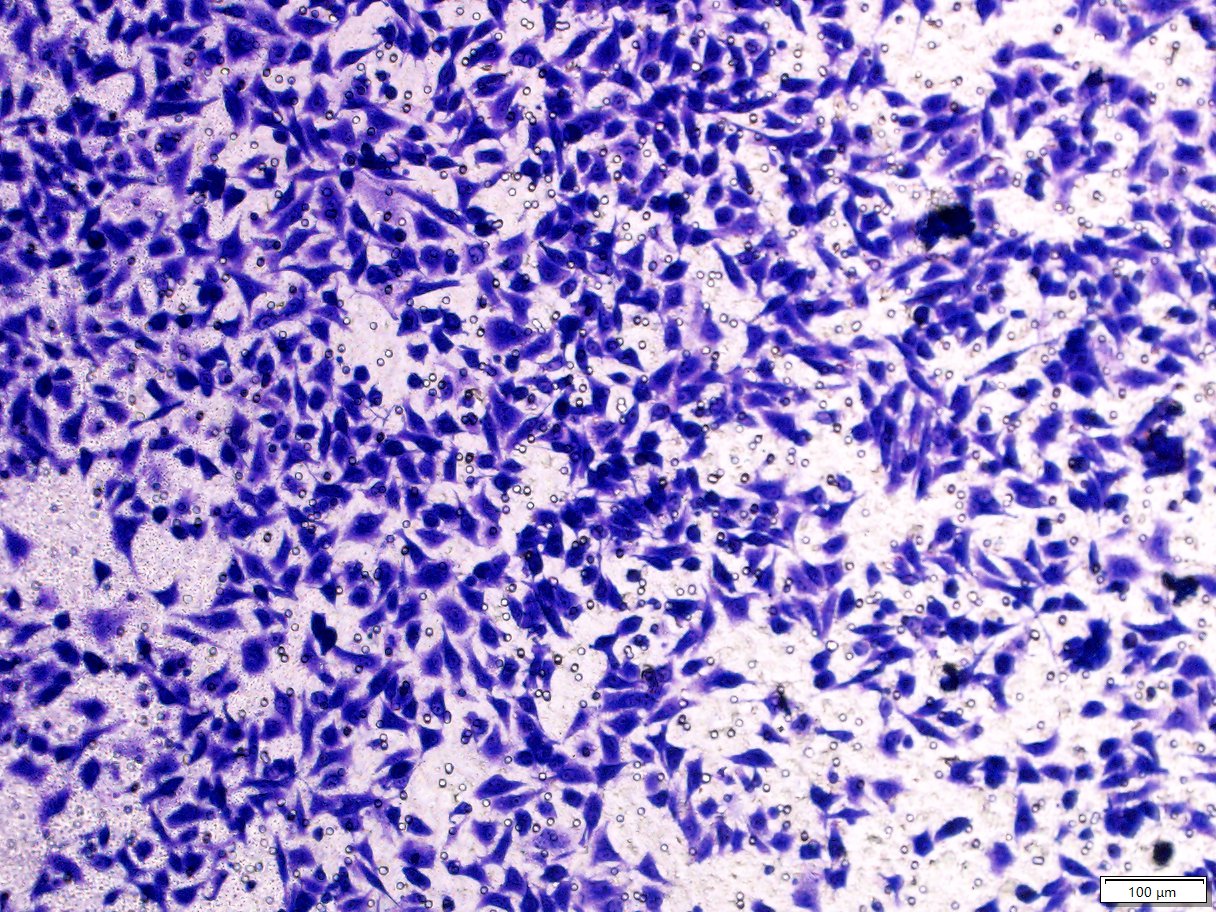

Supplement: Supplemental Information 10 [file peerj-cs-09-1651-s010.zip › Dataset 9/1+8.jpg]

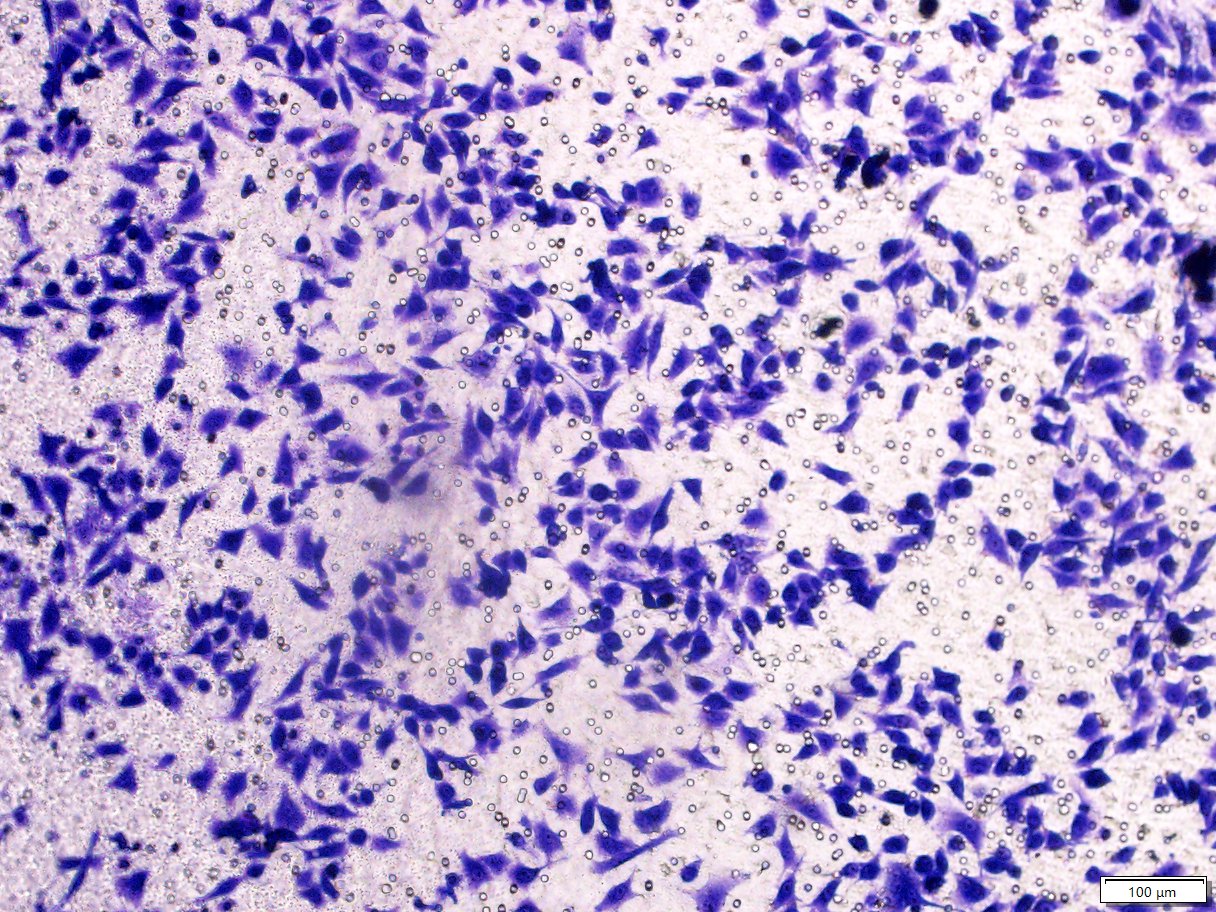

Supplement: Supplemental Information 10 [file peerj-cs-09-1651-s010.zip › Dataset 9/1+9.jpg]

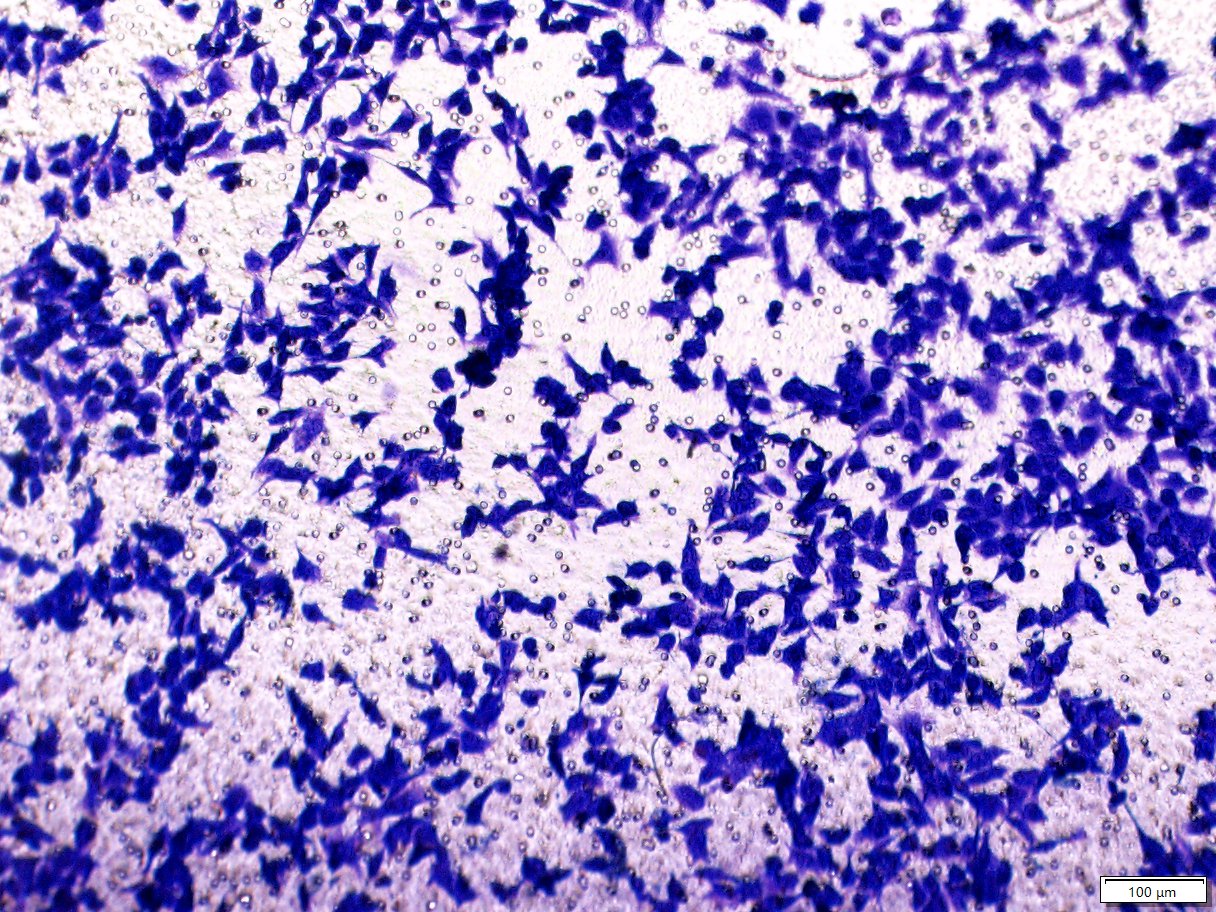

Supplement: Supplemental Information 10 [file peerj-cs-09-1651-s010.zip › Dataset 9/1-1.jpg]

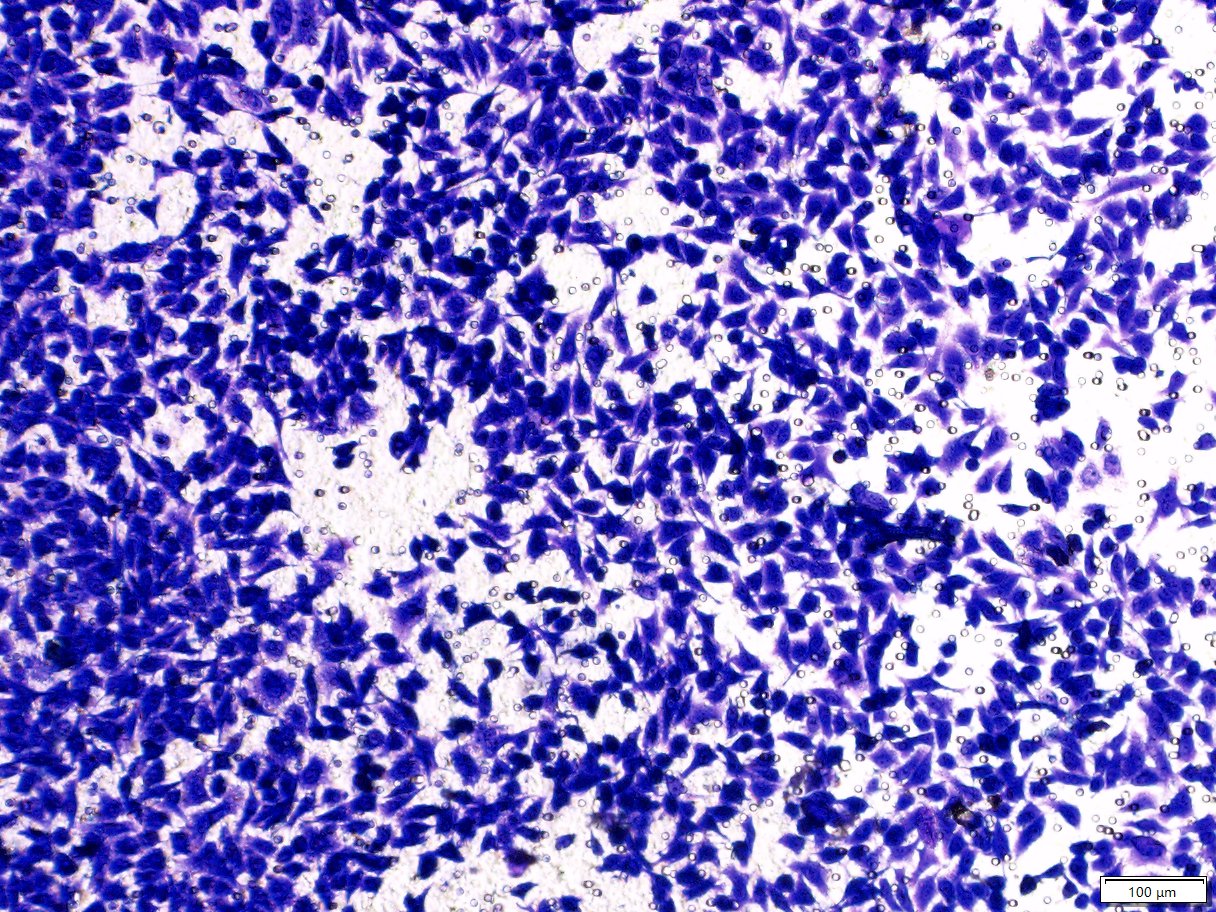

Supplement: Supplemental Information 10 [file peerj-cs-09-1651-s010.zip › Dataset 9/1-10.jpg]

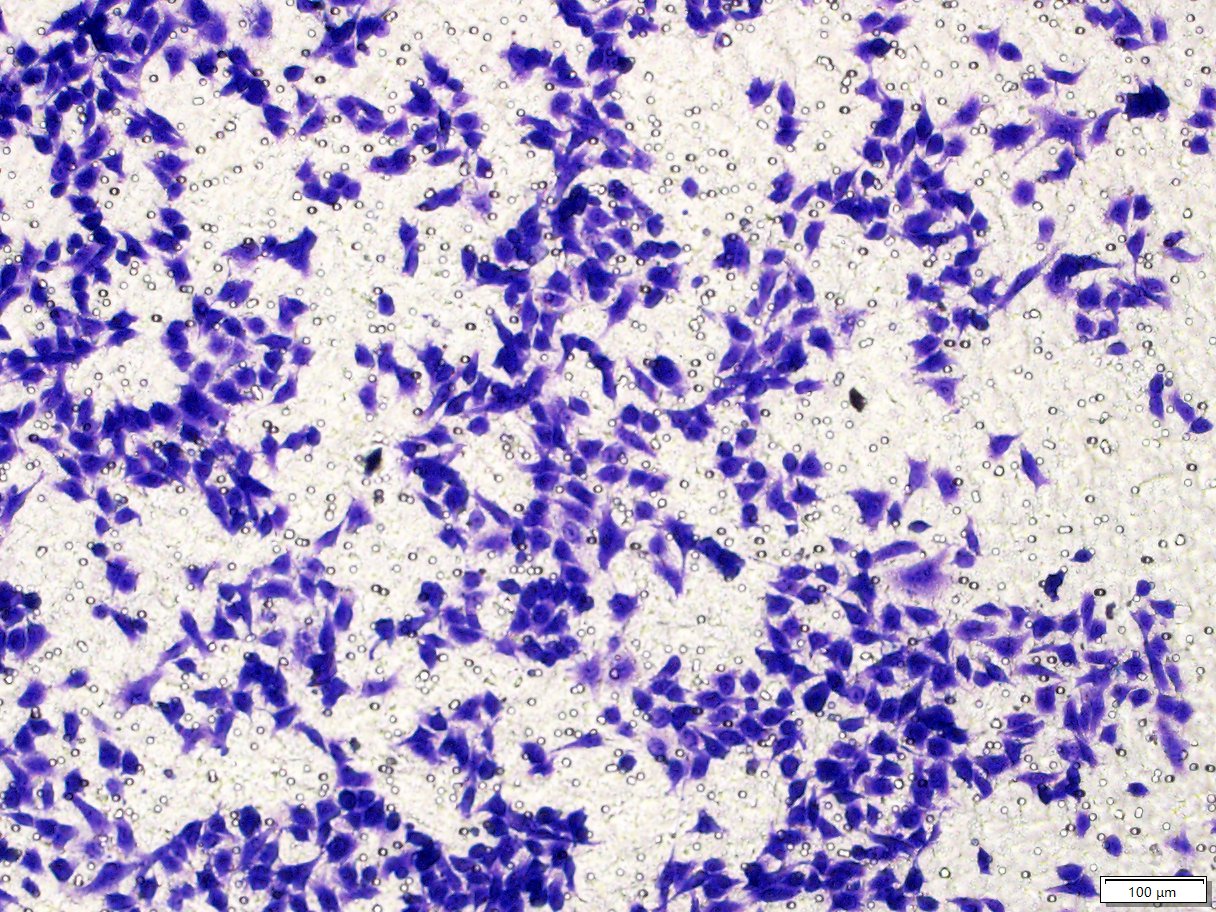

Supplement: Supplemental Information 10 [file peerj-cs-09-1651-s010.zip › Dataset 9/1-112.jpg]

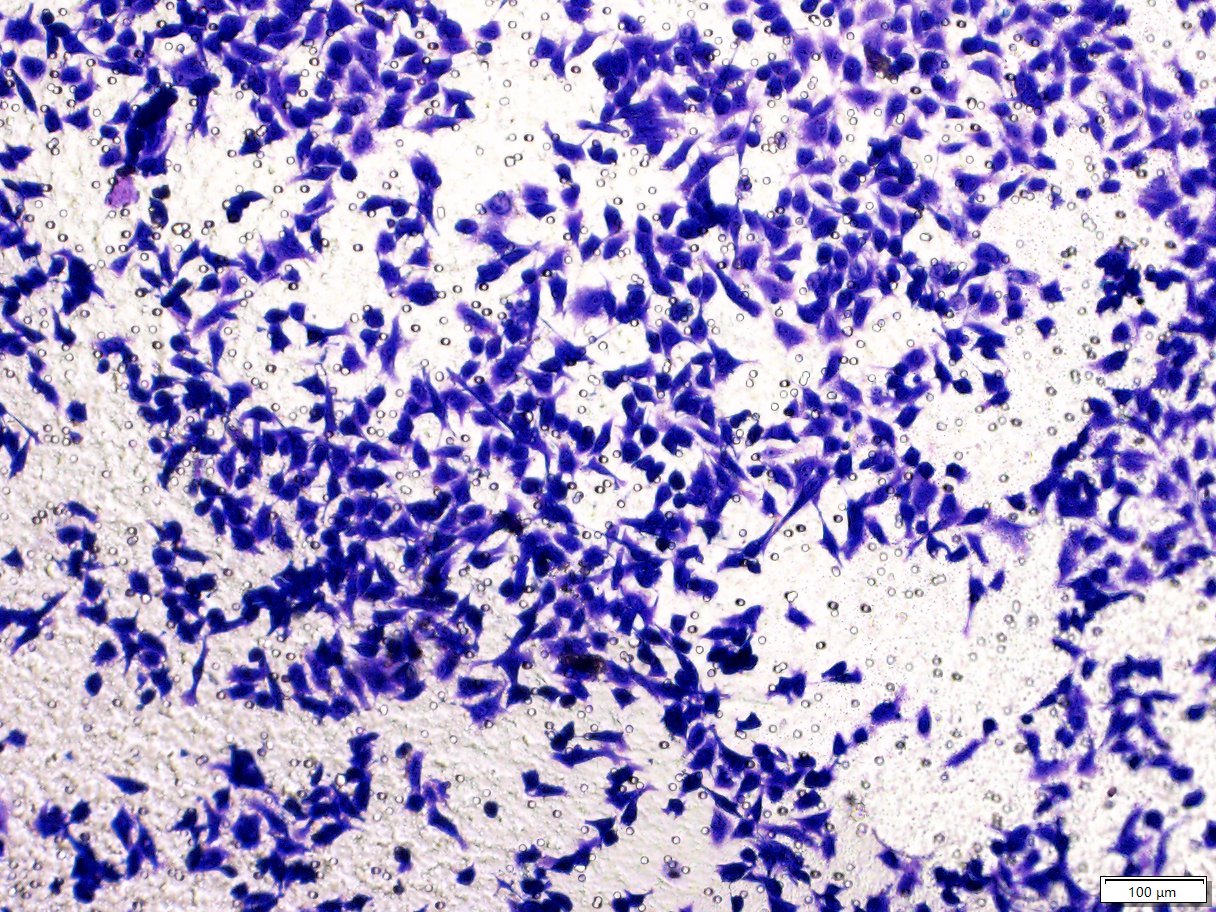

Supplement: Supplemental Information 10 [file peerj-cs-09-1651-s010.zip › Dataset 9/1-12.jpg]

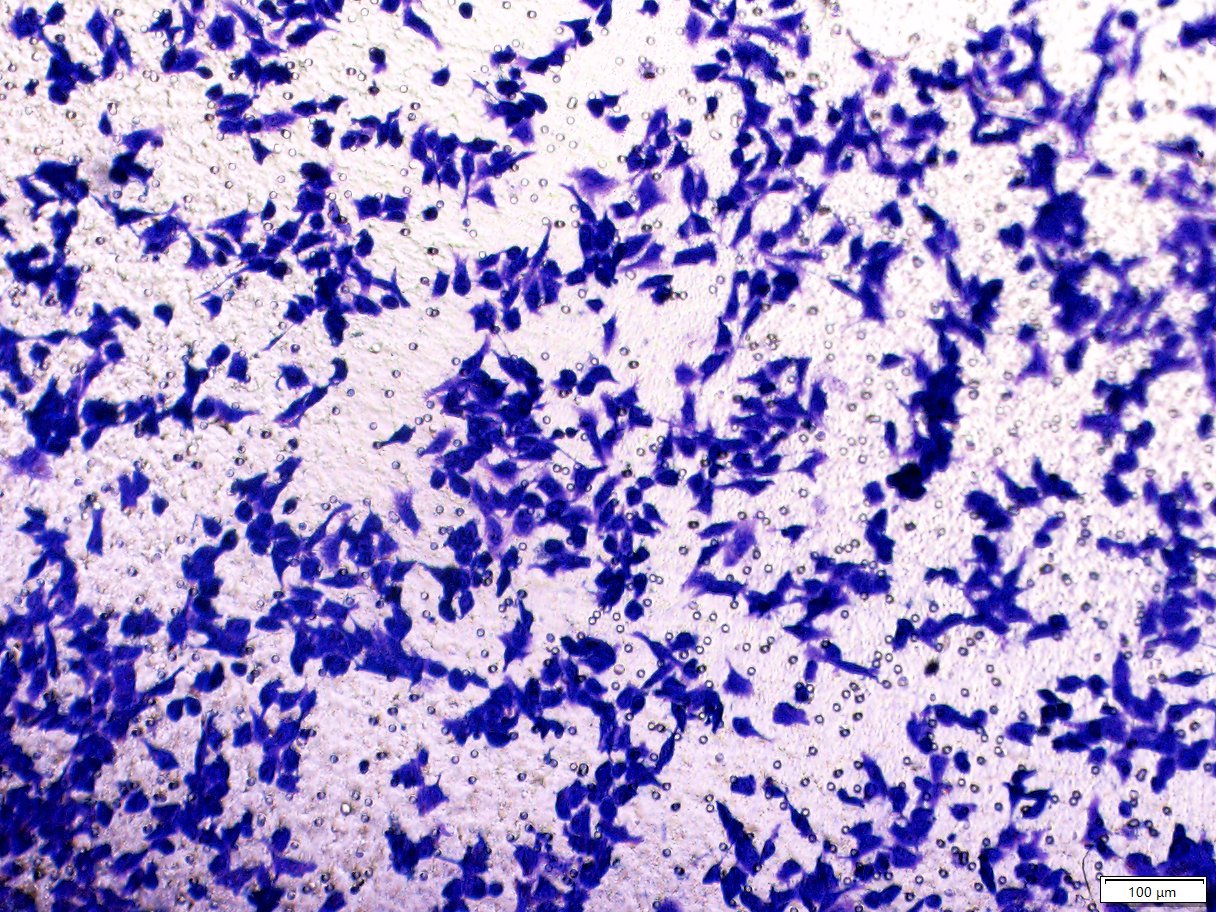

Supplement: Supplemental Information 10 [file peerj-cs-09-1651-s010.zip › Dataset 9/1-13.jpg]

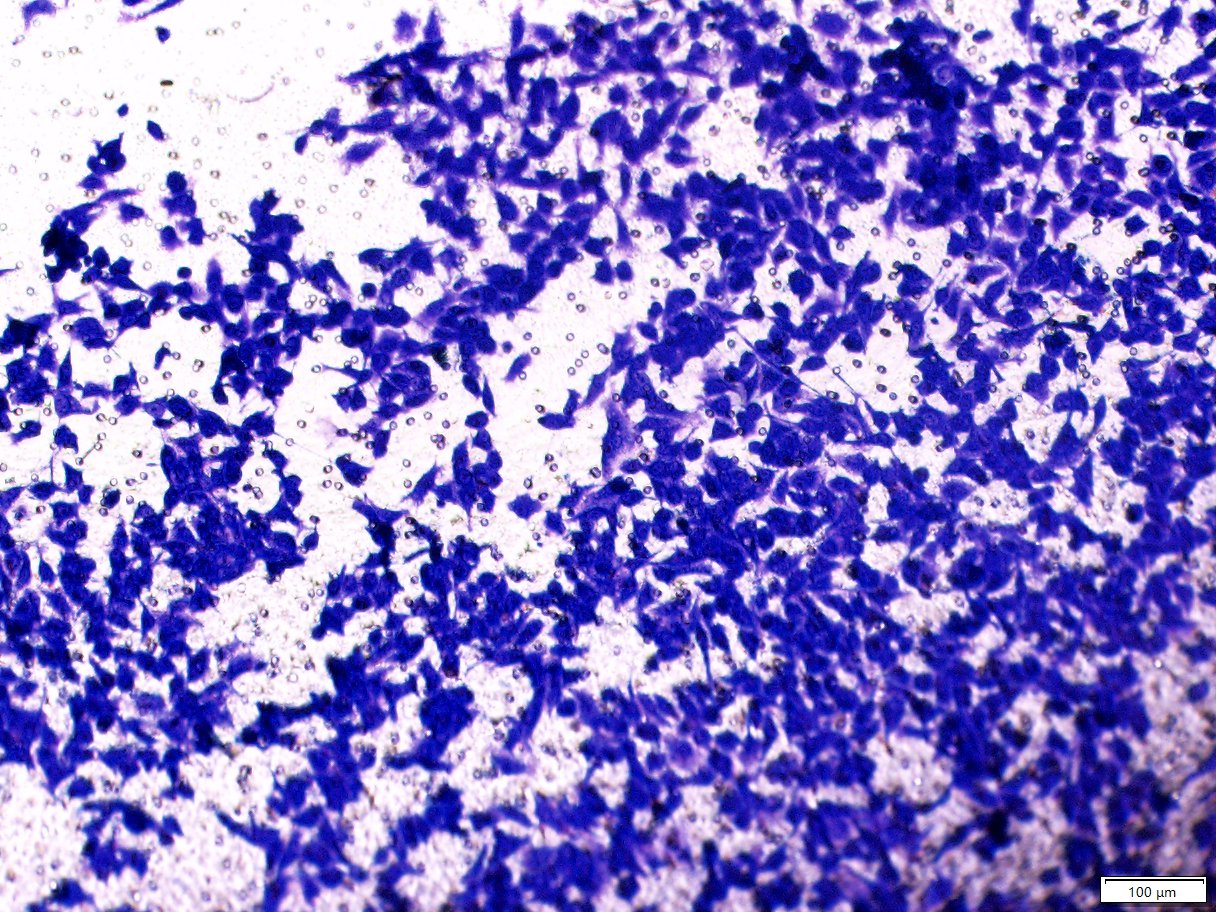

Supplement: Supplemental Information 10 [file peerj-cs-09-1651-s010.zip › Dataset 9/1-2.jpg]

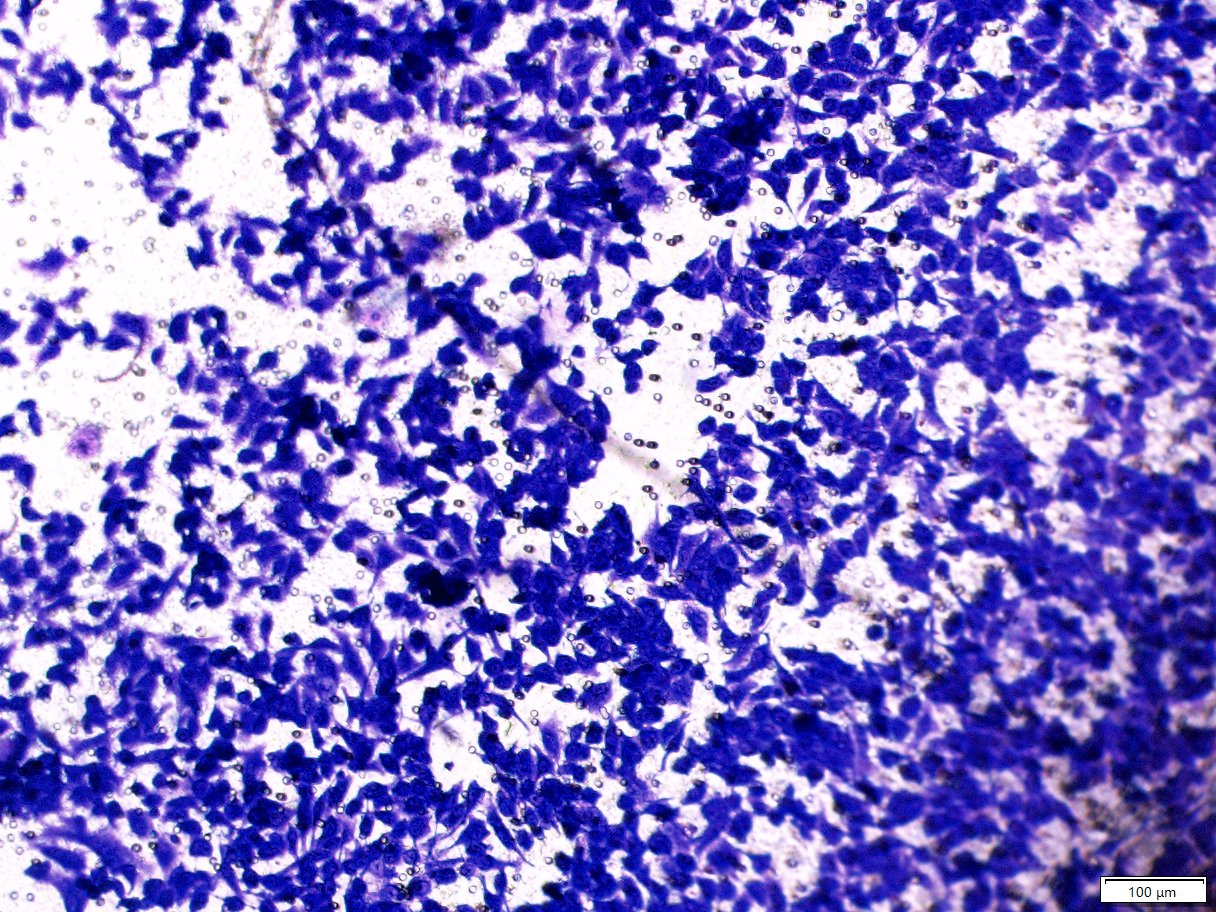

Supplement: Supplemental Information 10 [file peerj-cs-09-1651-s010.zip › Dataset 9/1-3.jpg]

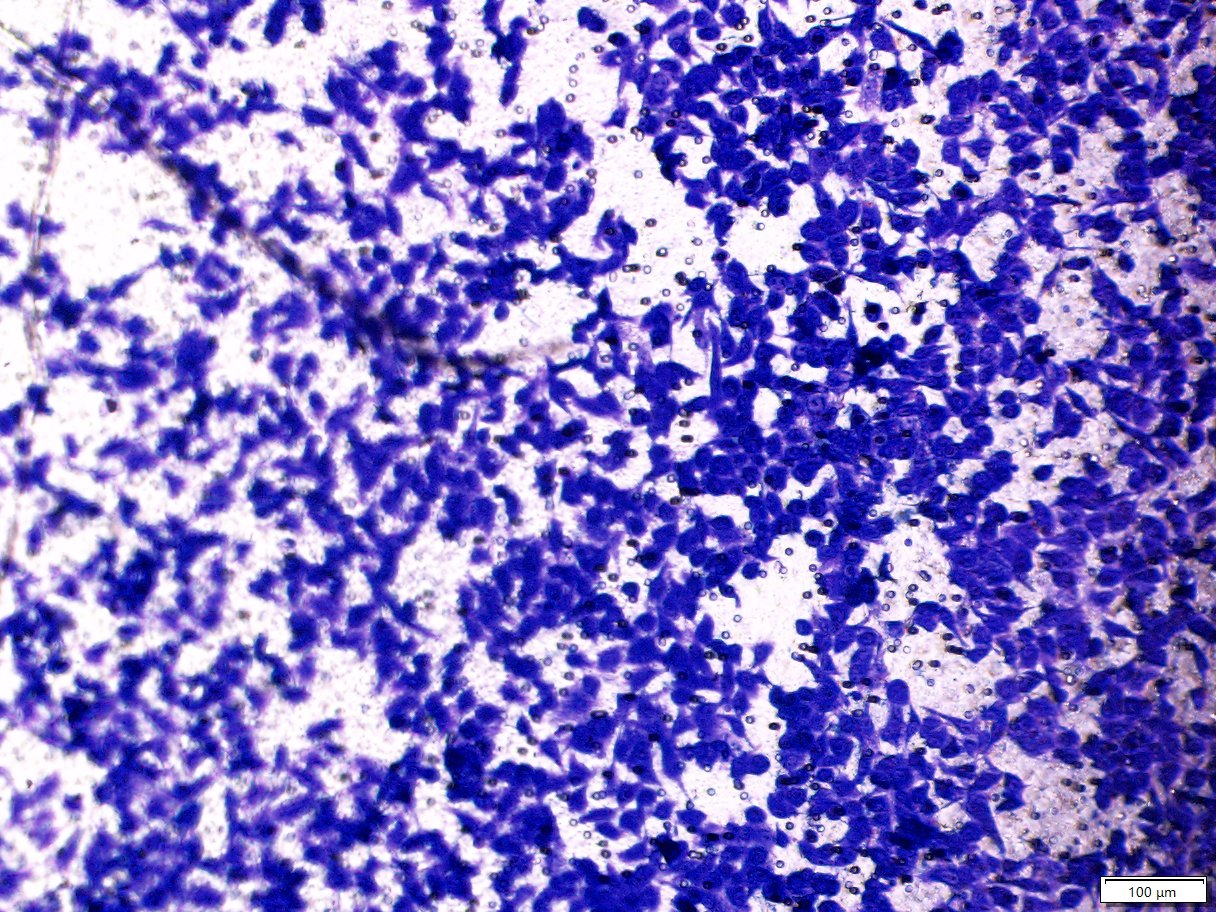

Supplement: Supplemental Information 10 [file peerj-cs-09-1651-s010.zip › Dataset 9/1-4.jpg]

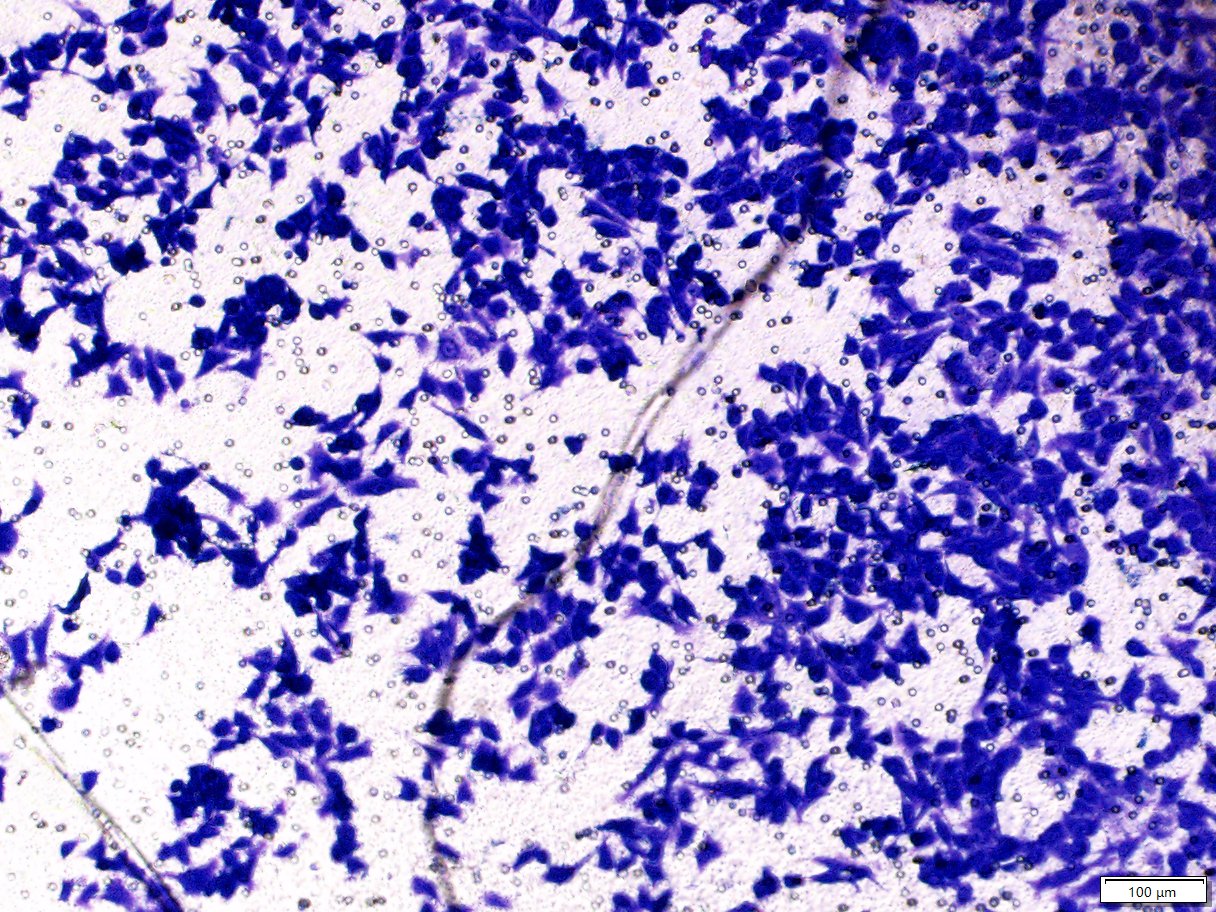

Supplement: Supplemental Information 10 [file peerj-cs-09-1651-s010.zip › Dataset 9/1-56.jpg]

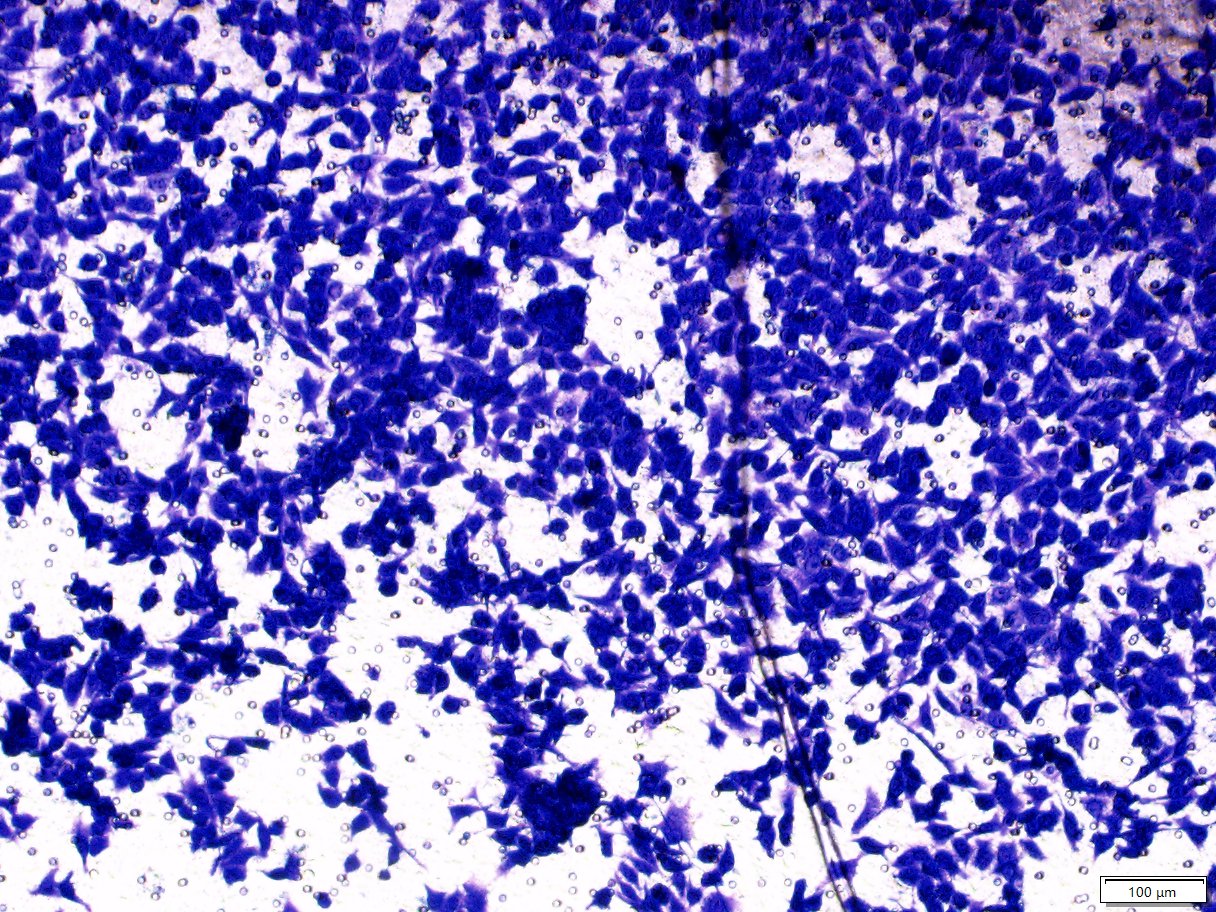

Supplement: Supplemental Information 10 [file peerj-cs-09-1651-s010.zip › Dataset 9/1-6.jpg]

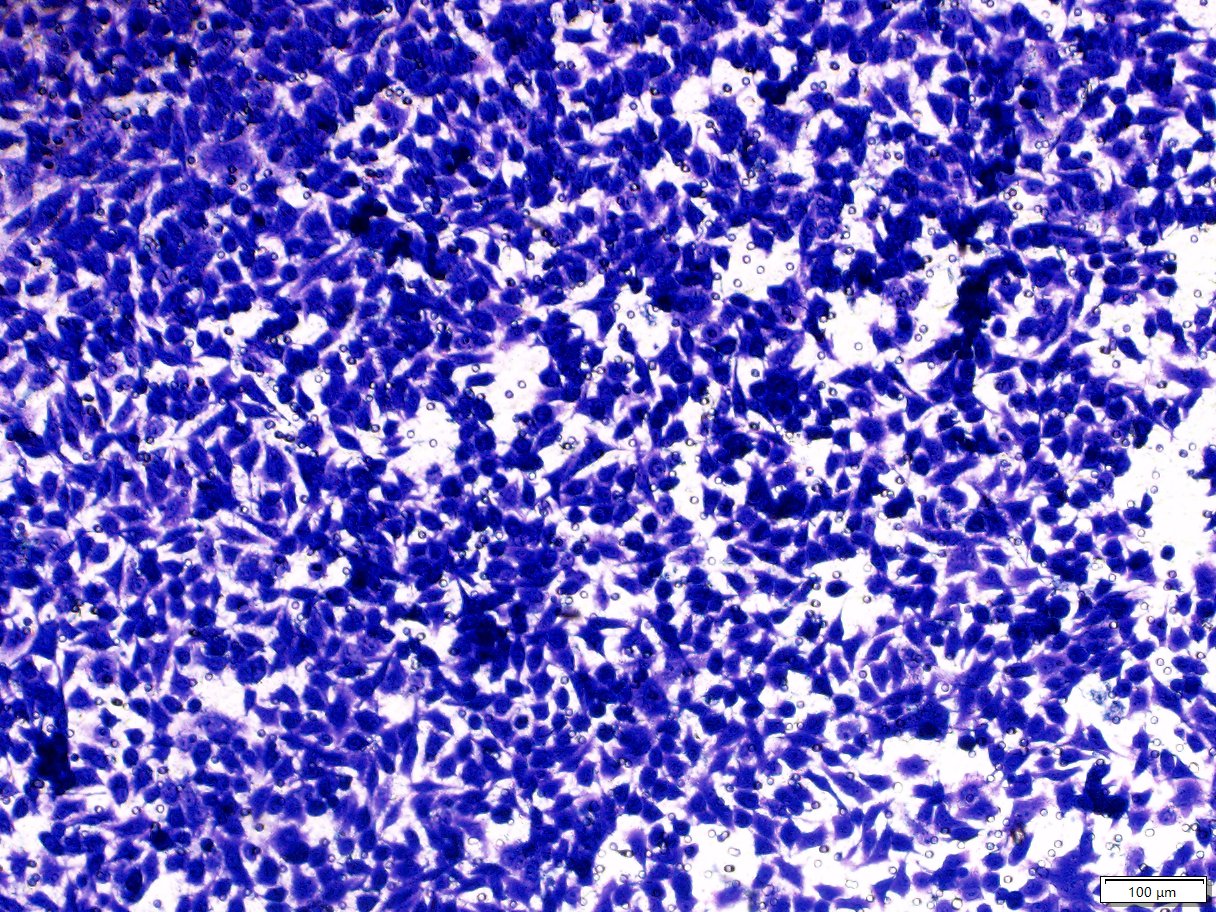

Supplement: Supplemental Information 10 [file peerj-cs-09-1651-s010.zip › Dataset 9/1-7.jpg]

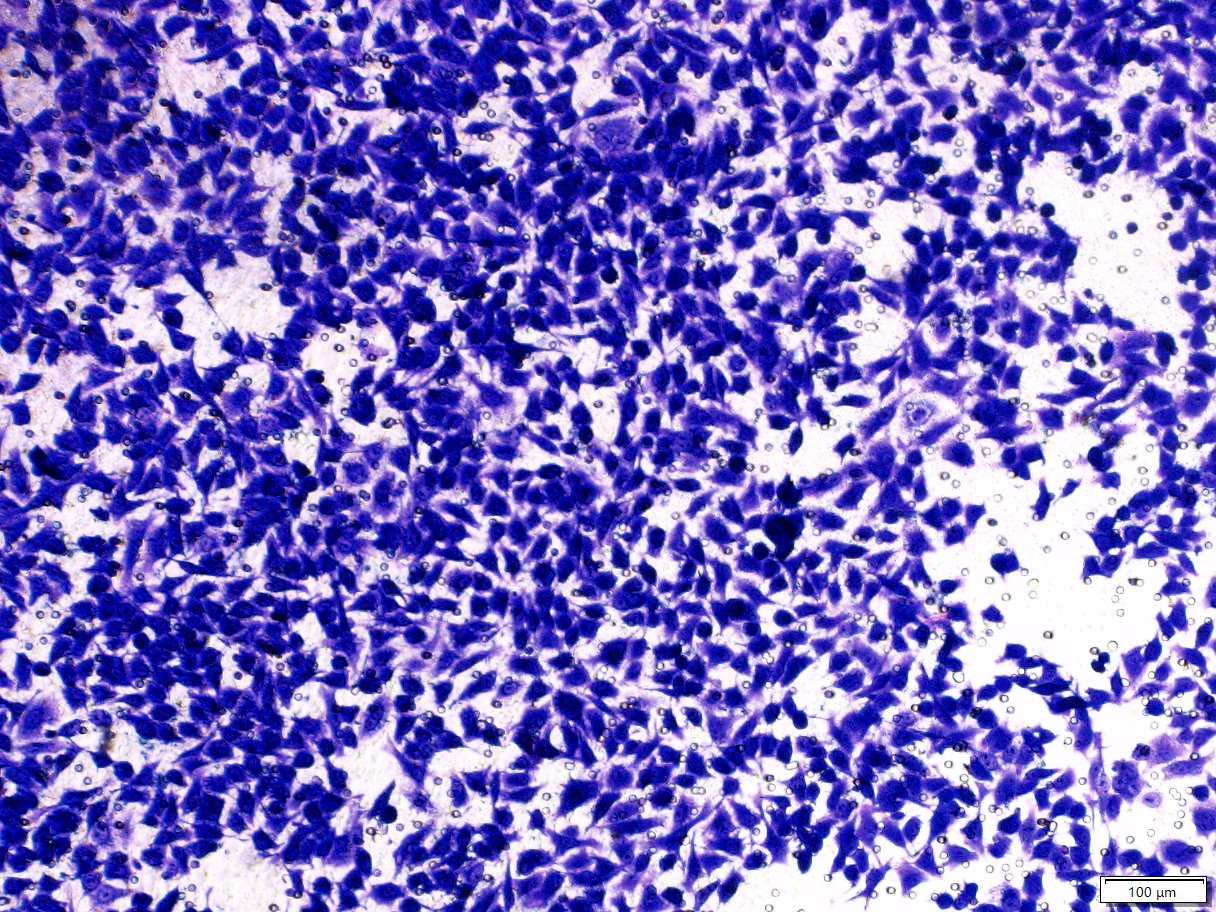

Supplement: Supplemental Information 10 [file peerj-cs-09-1651-s010.zip › Dataset 9/1-8.jpg]

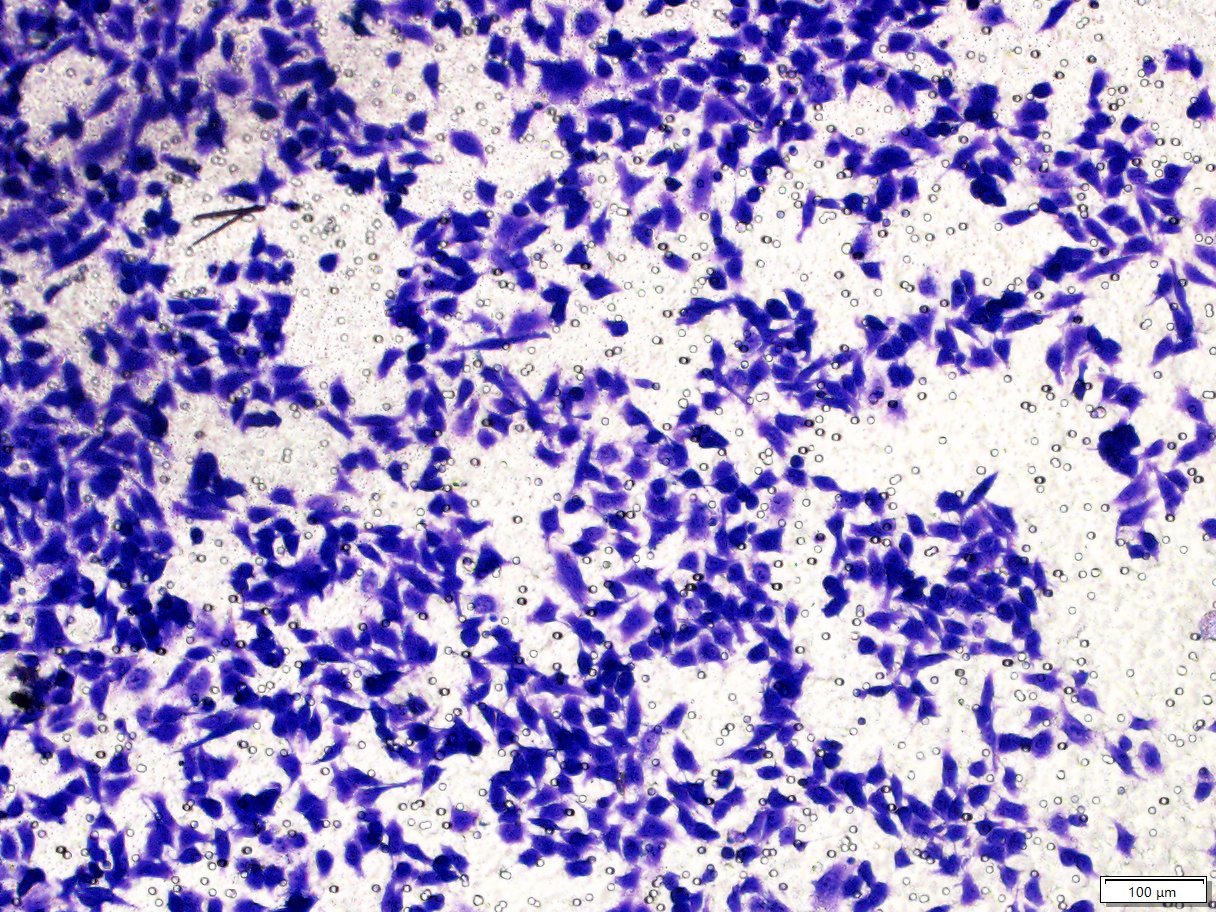

Supplement: Supplemental Information 10 [file peerj-cs-09-1651-s010.zip › Dataset 9/1-9.jpg]

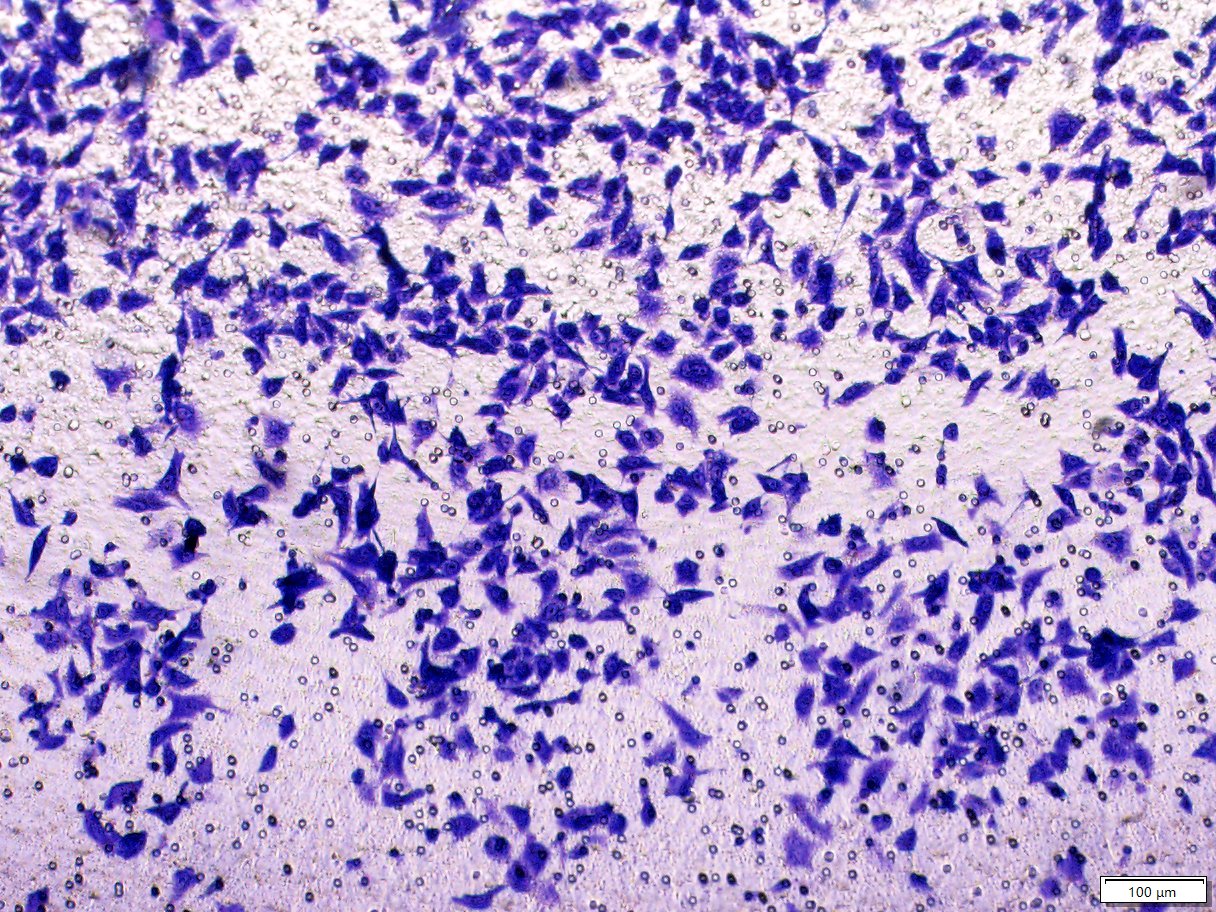

Supplement: Supplemental Information 10 [file peerj-cs-09-1651-s010.zip › Dataset 9/2+1.jpg]

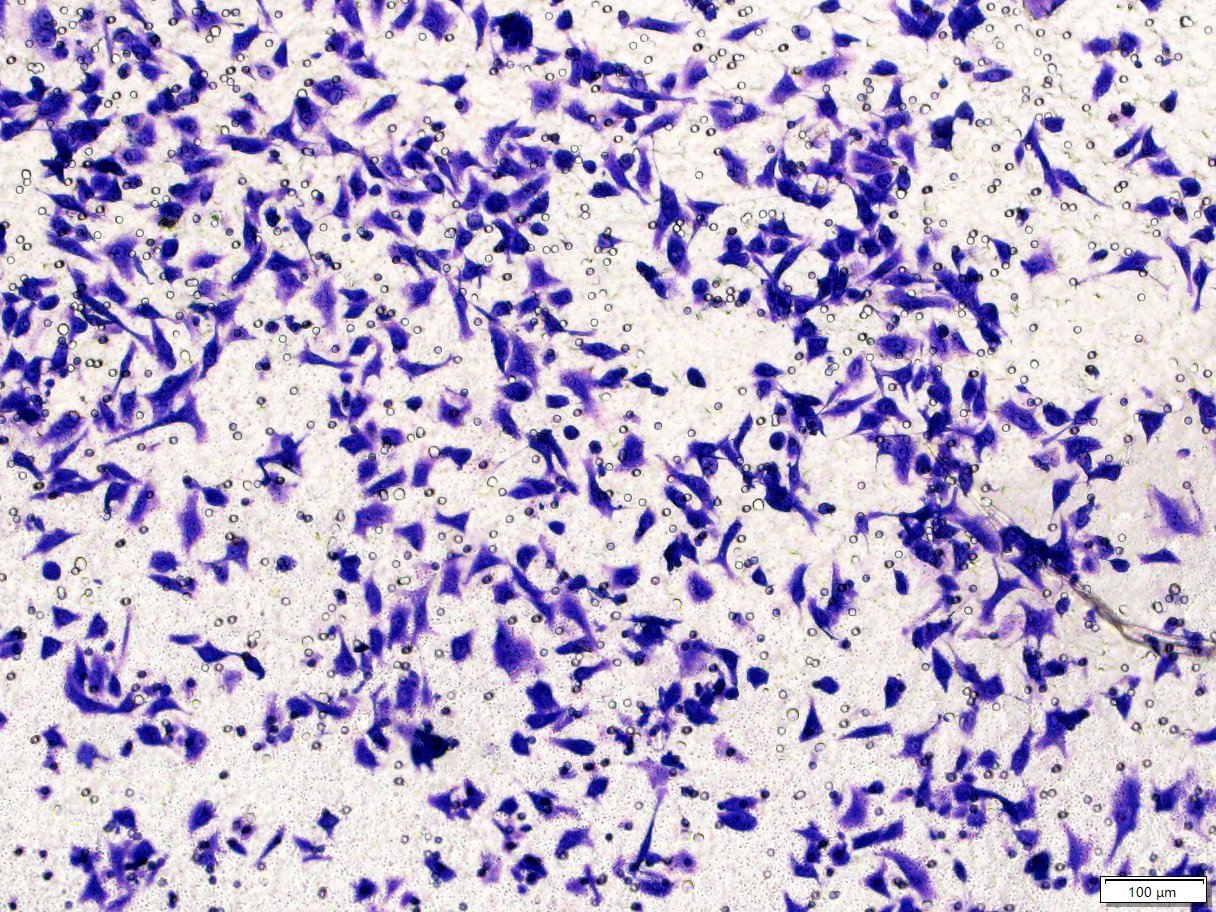

Supplement: Supplemental Information 10 [file peerj-cs-09-1651-s010.zip › Dataset 9/2+2.jpg]

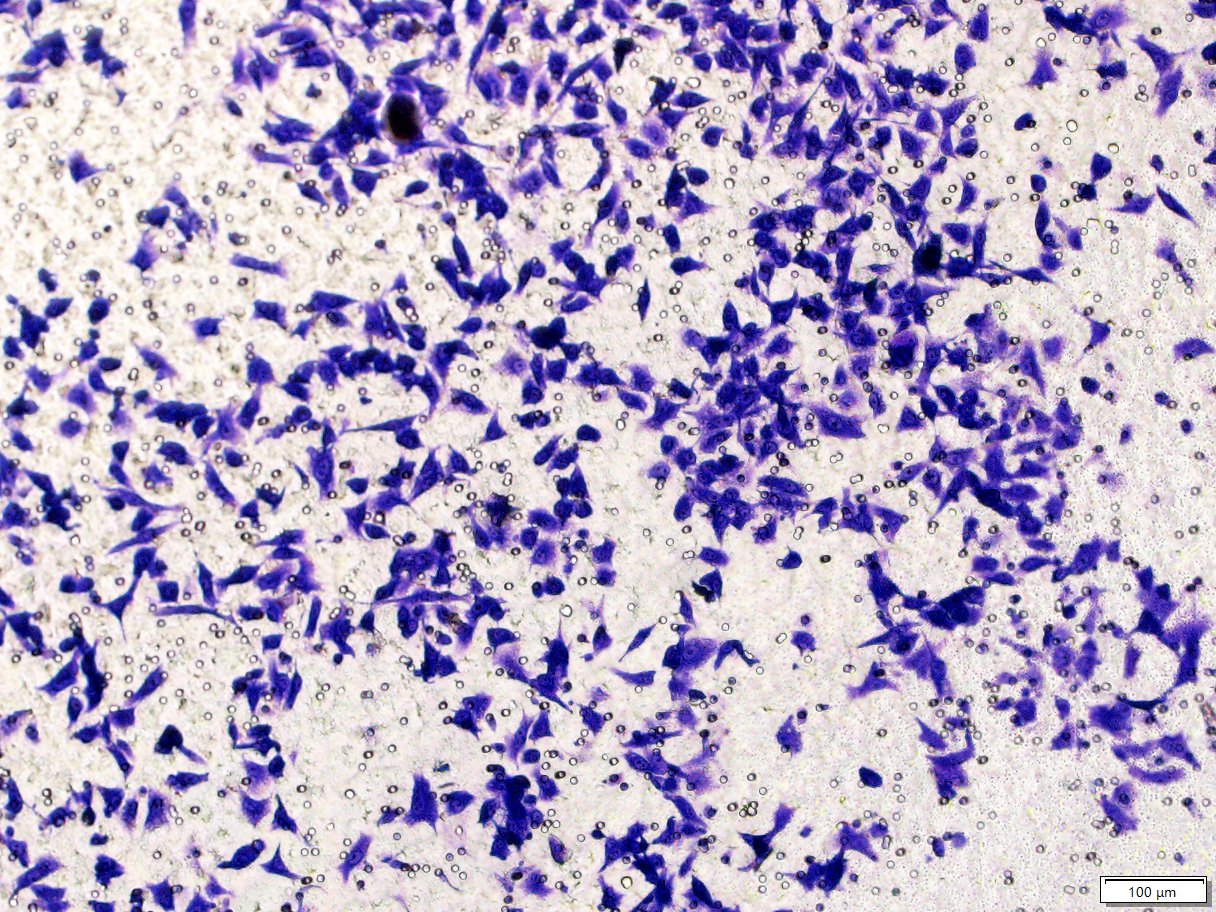

Supplement: Supplemental Information 10 [file peerj-cs-09-1651-s010.zip › Dataset 9/2+3.jpg]

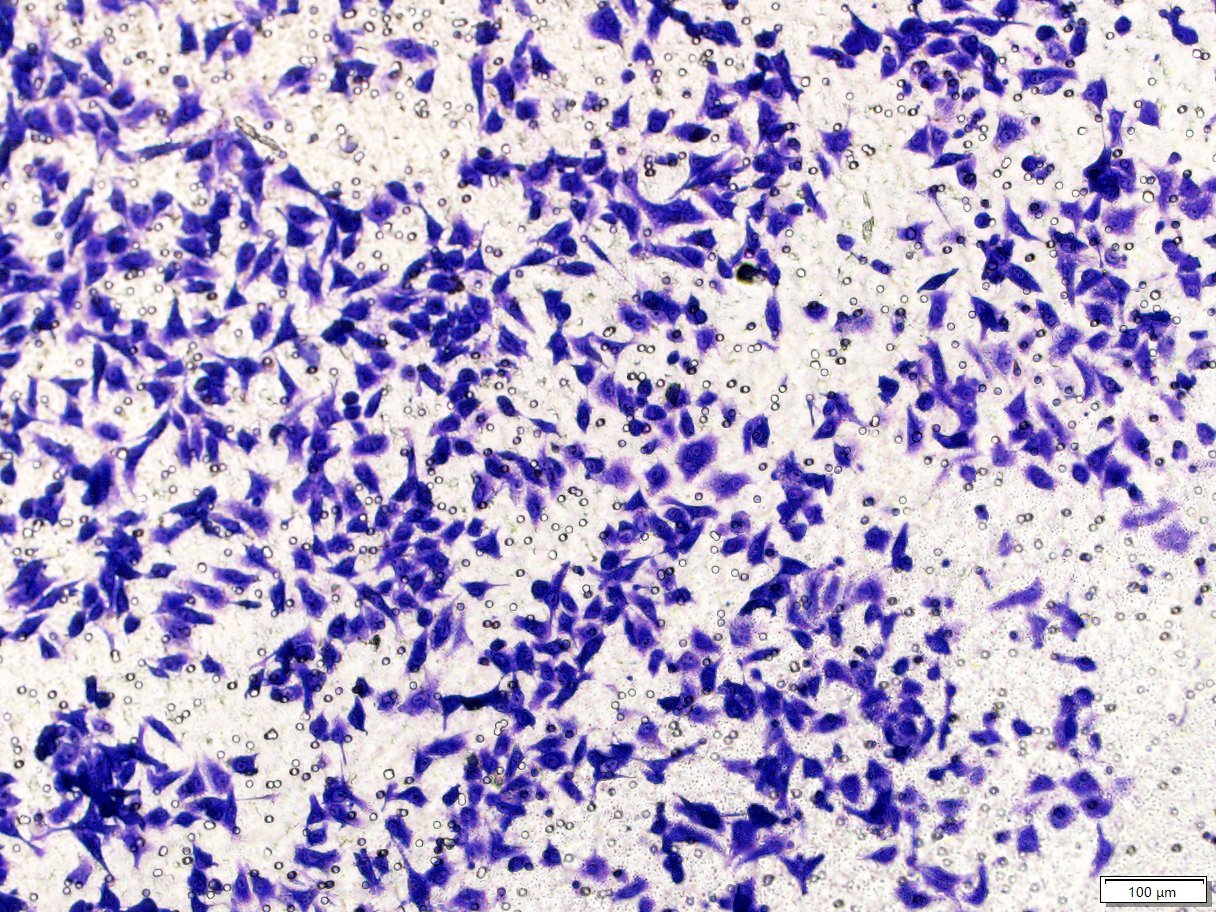

Supplement: Supplemental Information 10 [file peerj-cs-09-1651-s010.zip › Dataset 9/2+4.jpg]

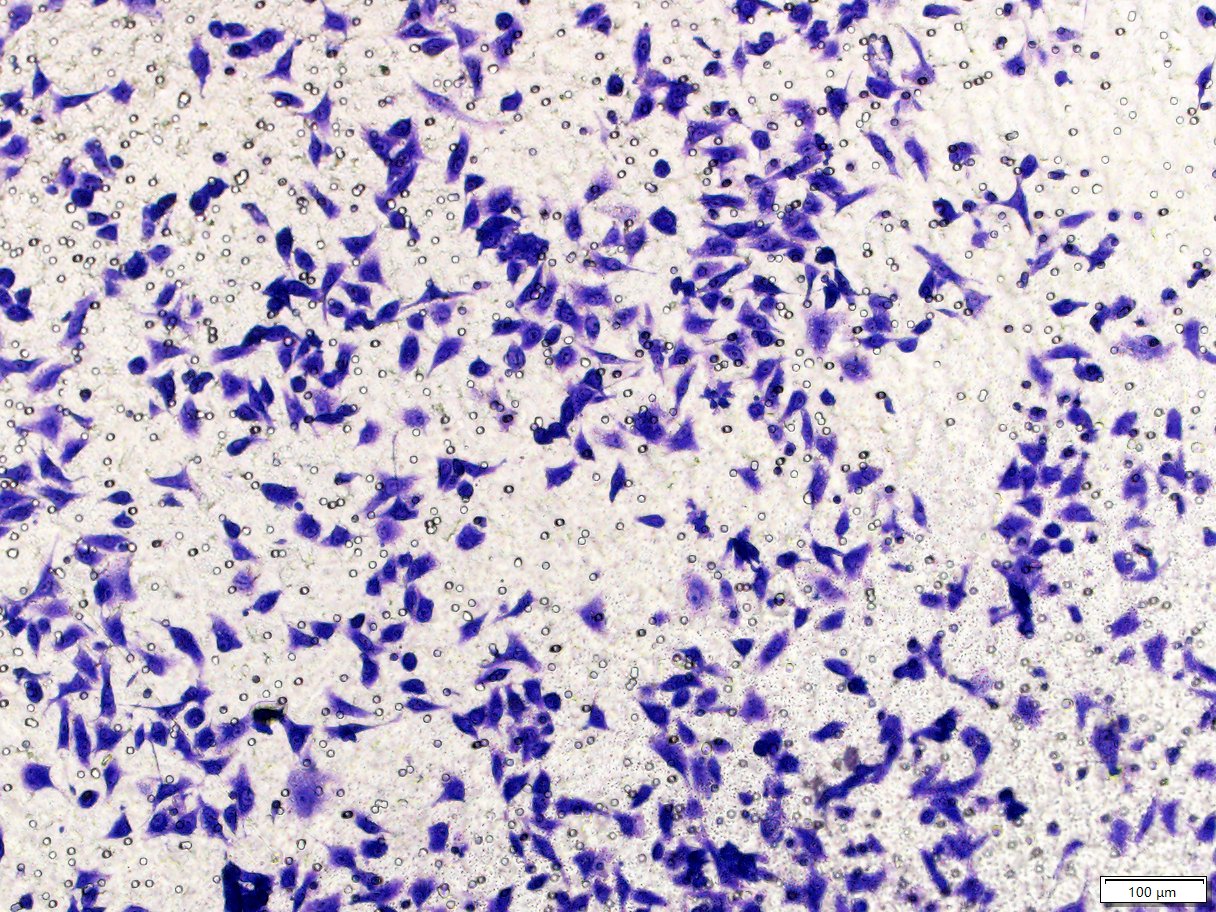

Supplement: Supplemental Information 10 [file peerj-cs-09-1651-s010.zip › Dataset 9/2+5.jpg]

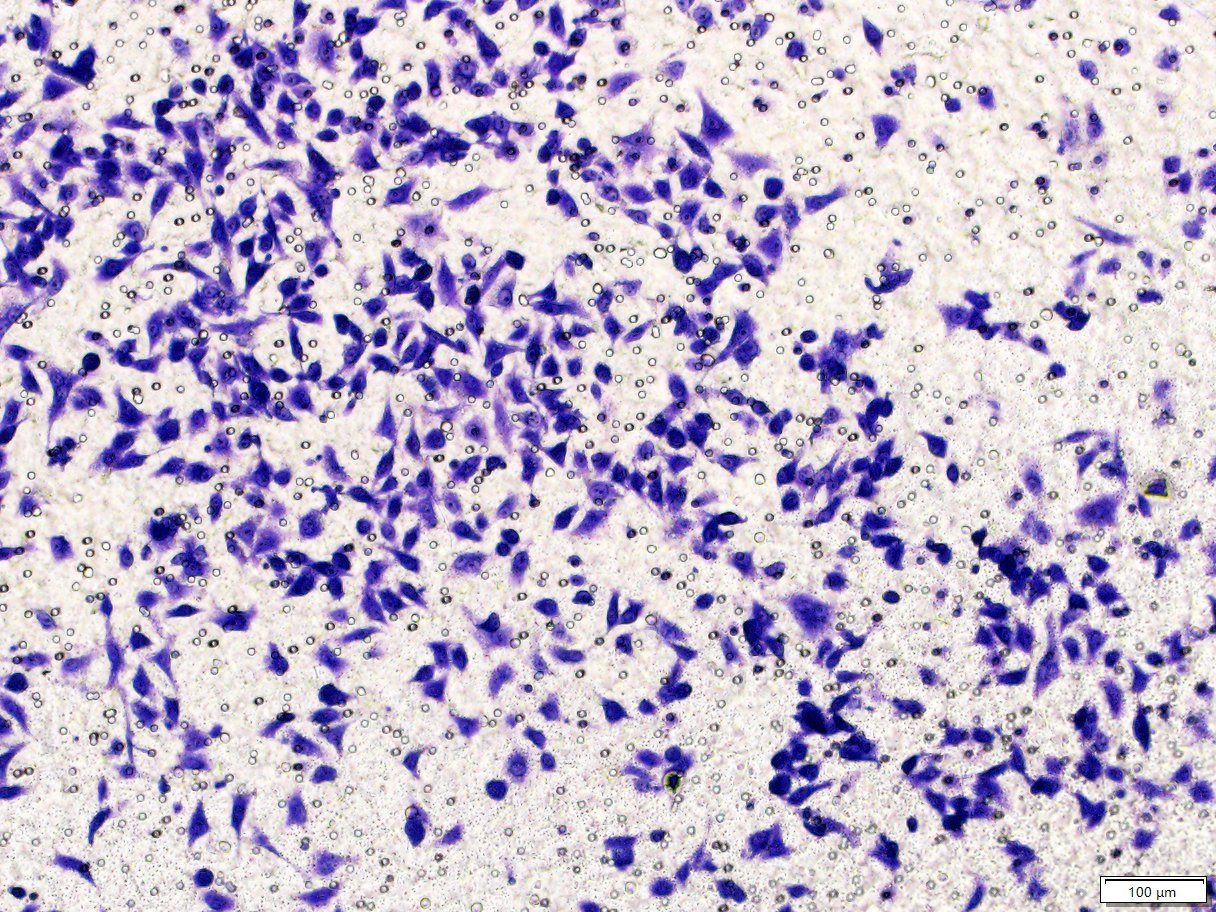

Supplement: Supplemental Information 10 [file peerj-cs-09-1651-s010.zip › Dataset 9/2+6.jpg]

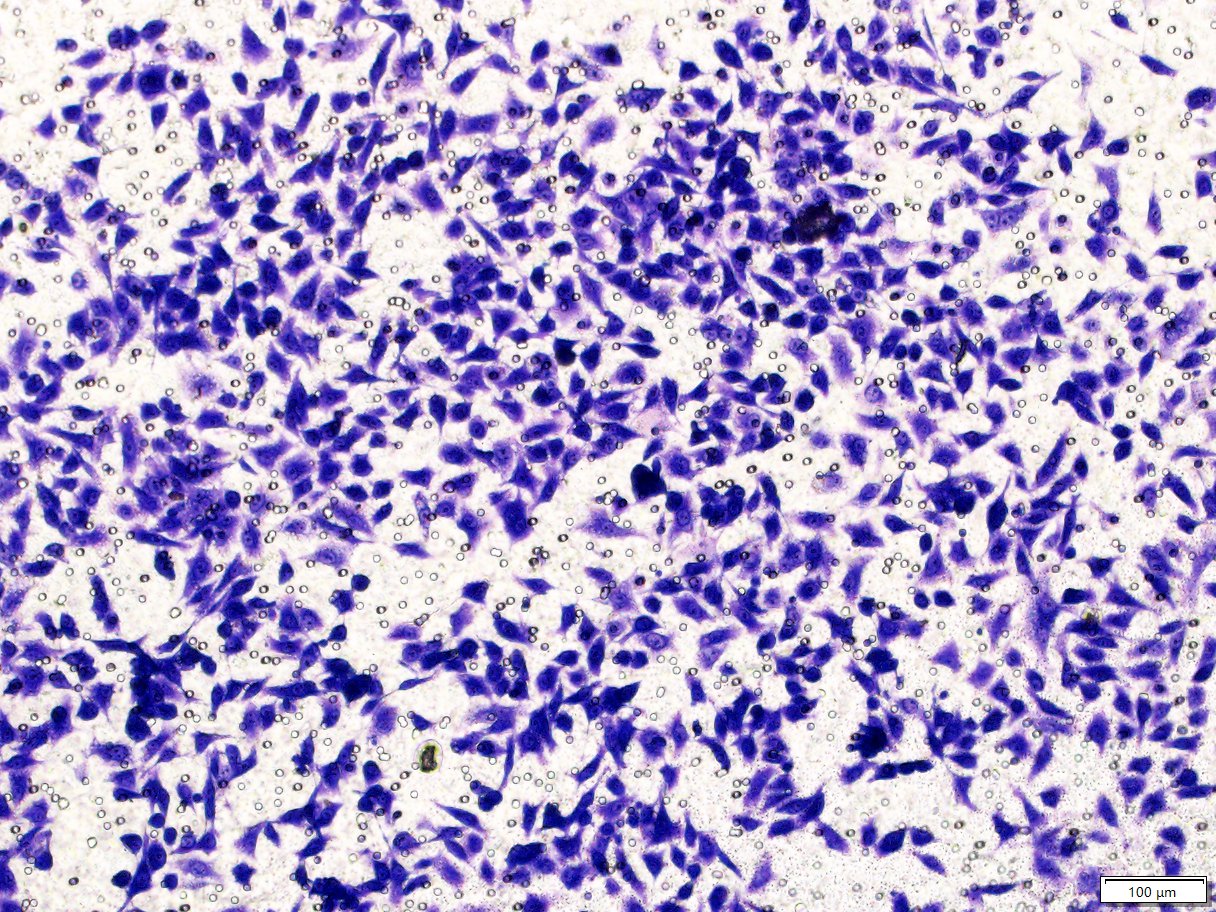

Supplement: Supplemental Information 10 [file peerj-cs-09-1651-s010.zip › Dataset 9/2+7.jpg]

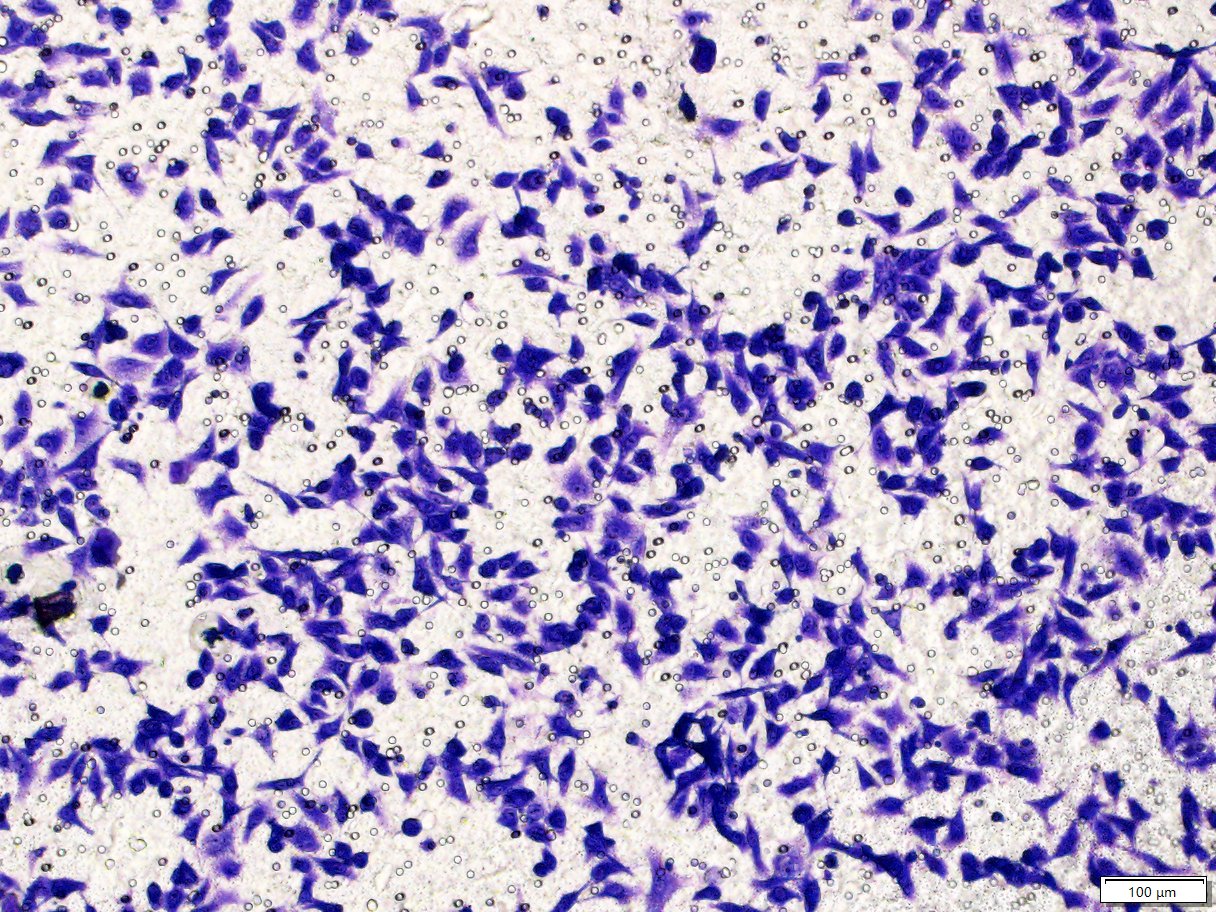

Supplement: Supplemental Information 10 [file peerj-cs-09-1651-s010.zip › Dataset 9/2+8.jpg]

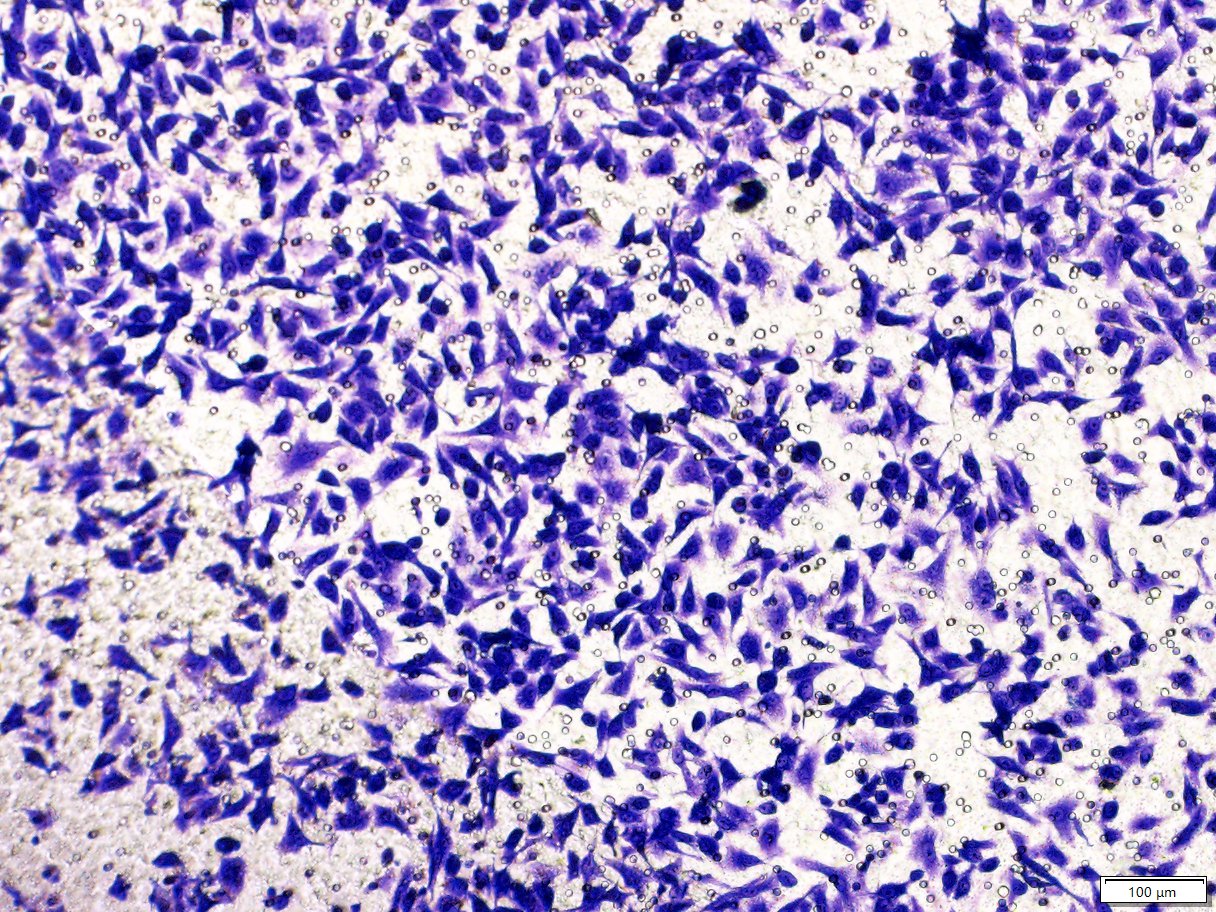

Supplement: Supplemental Information 11 [file peerj-cs-09-1651-s011.zip › Dataset 10/2+10.jpg]

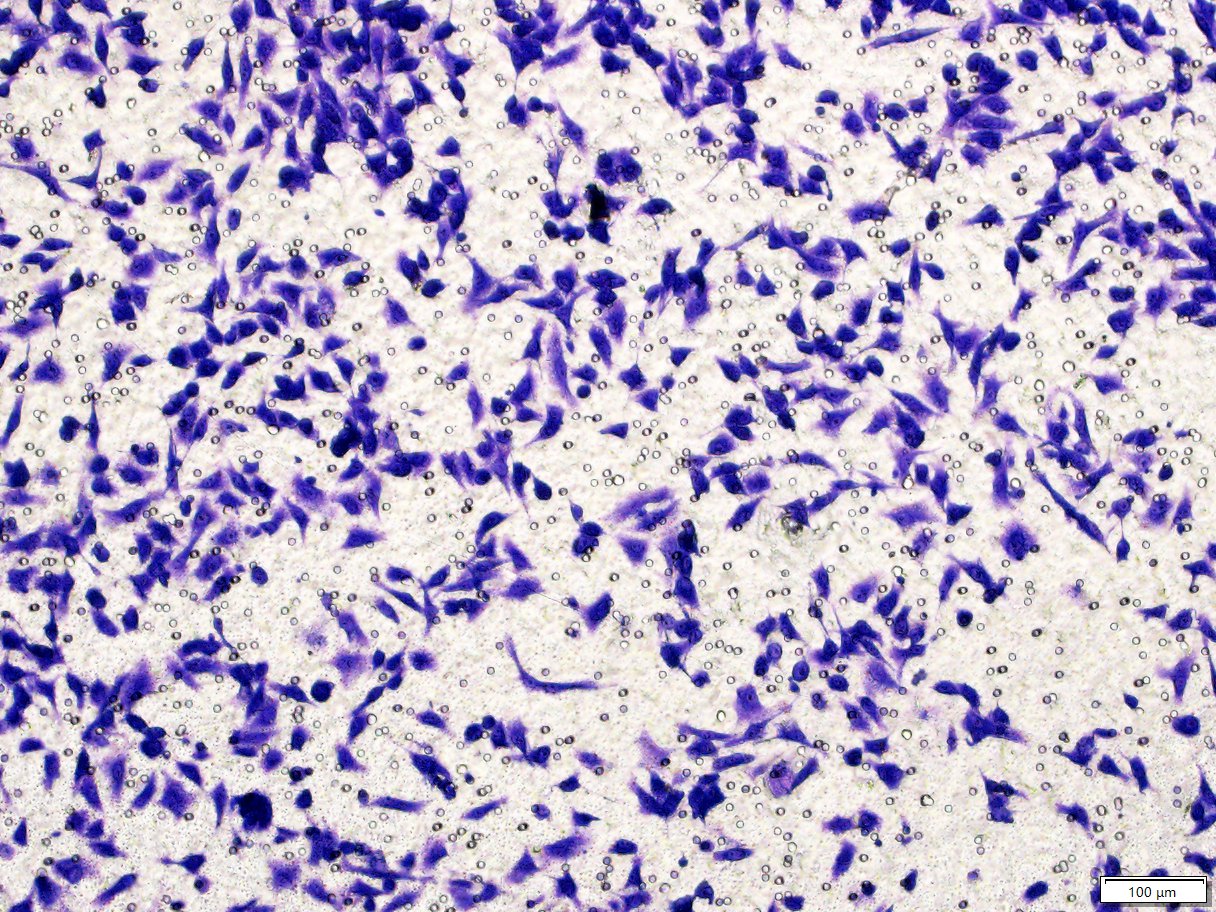

Supplement: Supplemental Information 11 [file peerj-cs-09-1651-s011.zip › Dataset 10/2+11.jpg]

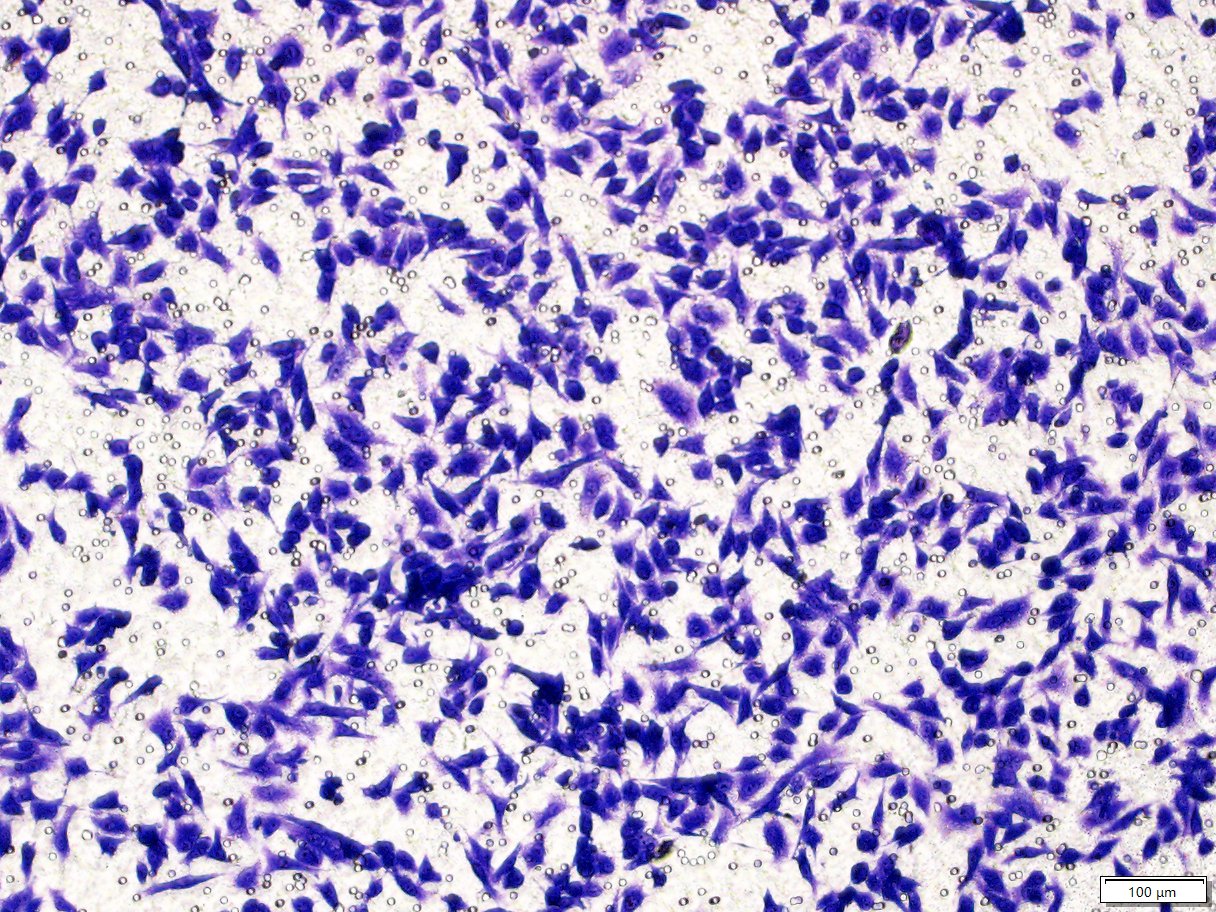

Supplement: Supplemental Information 11 [file peerj-cs-09-1651-s011.zip › Dataset 10/2+12.jpg]

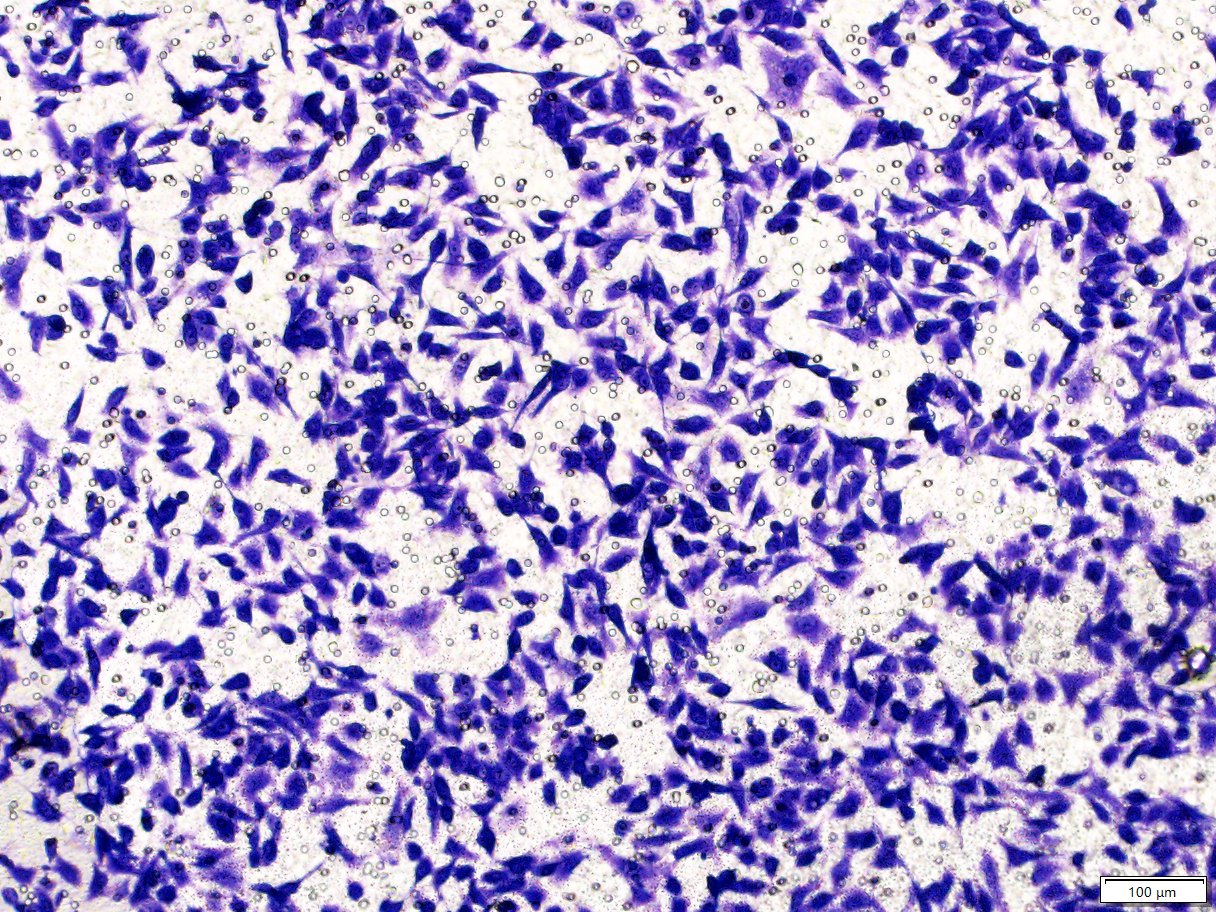

Supplement: Supplemental Information 11 [file peerj-cs-09-1651-s011.zip › Dataset 10/2+9.jpg]

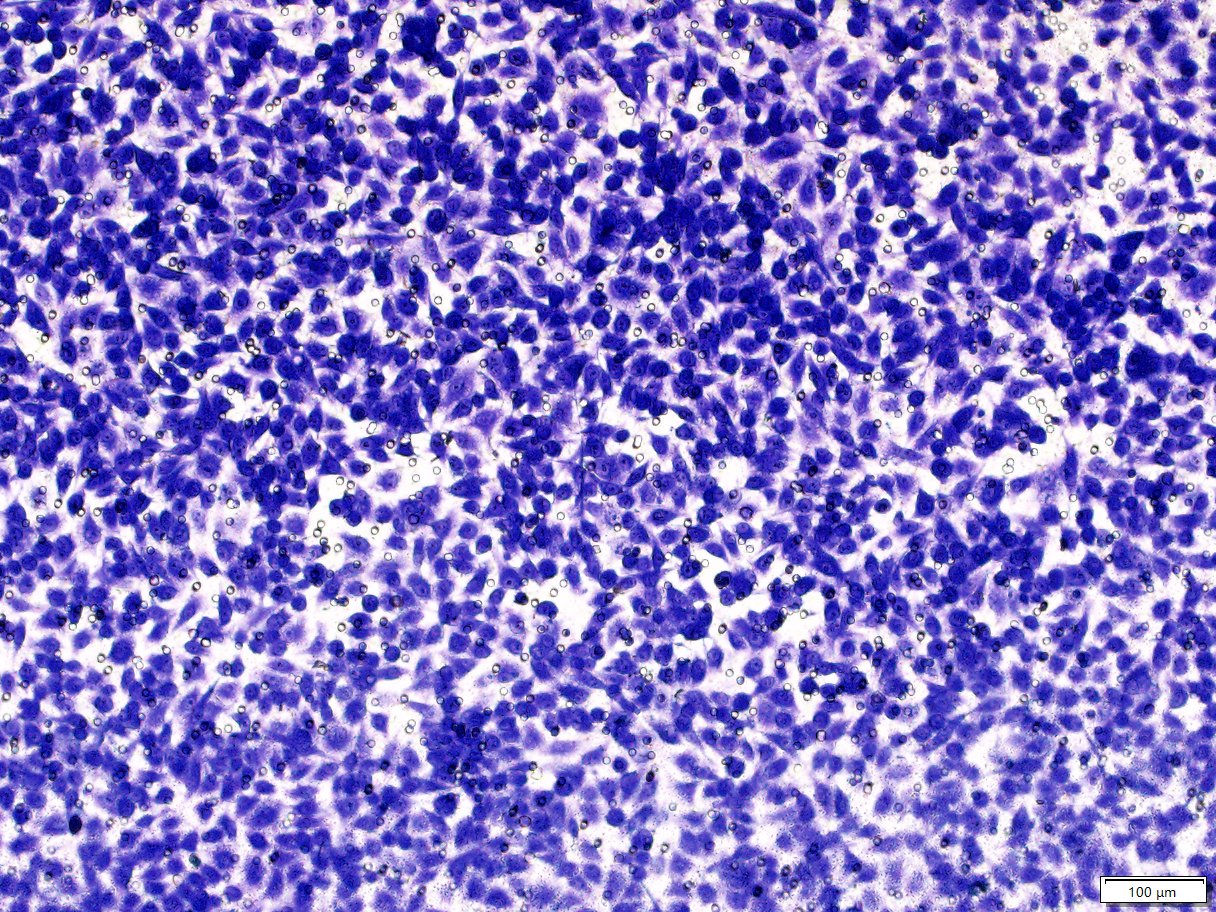

Supplement: Supplemental Information 11 [file peerj-cs-09-1651-s011.zip › Dataset 10/2-1.jpg]

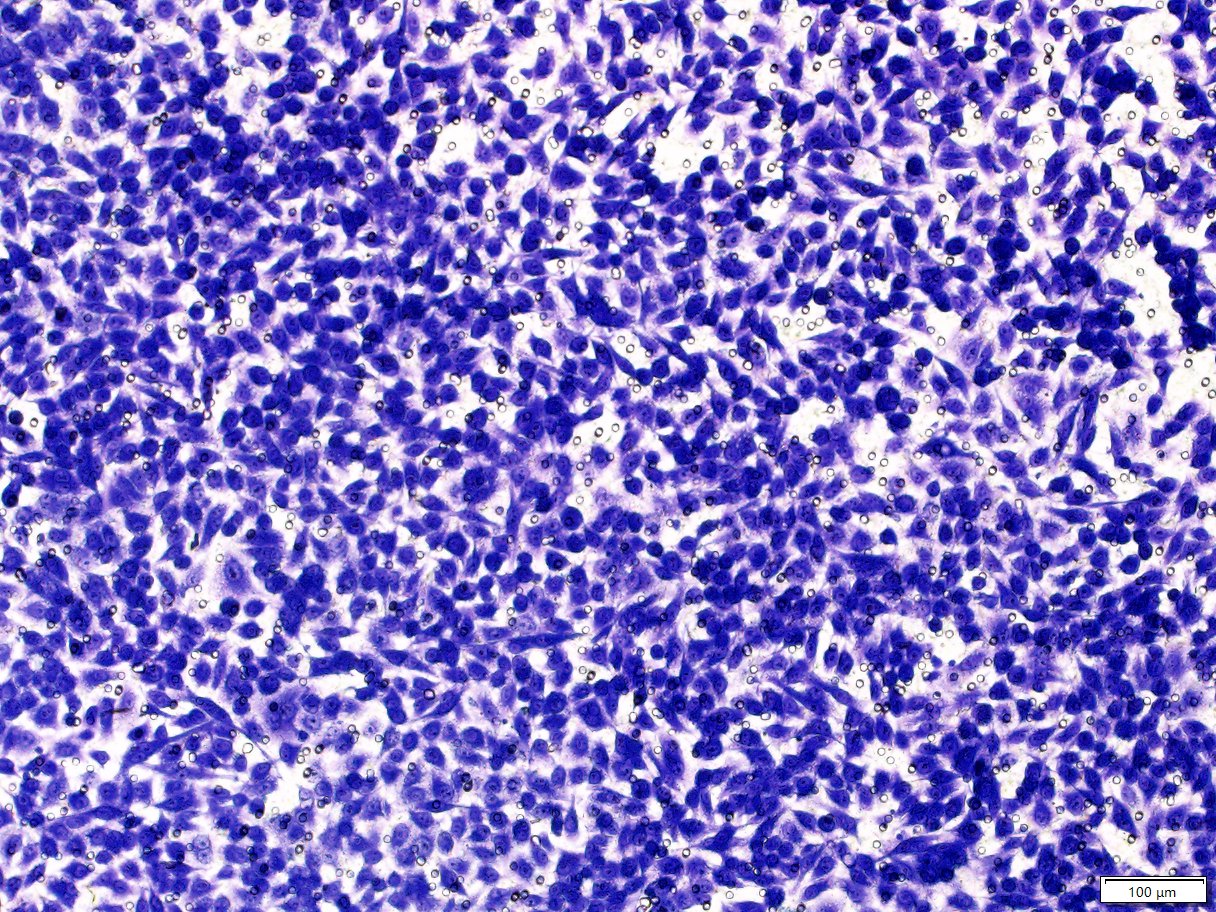

Supplement: Supplemental Information 11 [file peerj-cs-09-1651-s011.zip › Dataset 10/2-10.jpg]

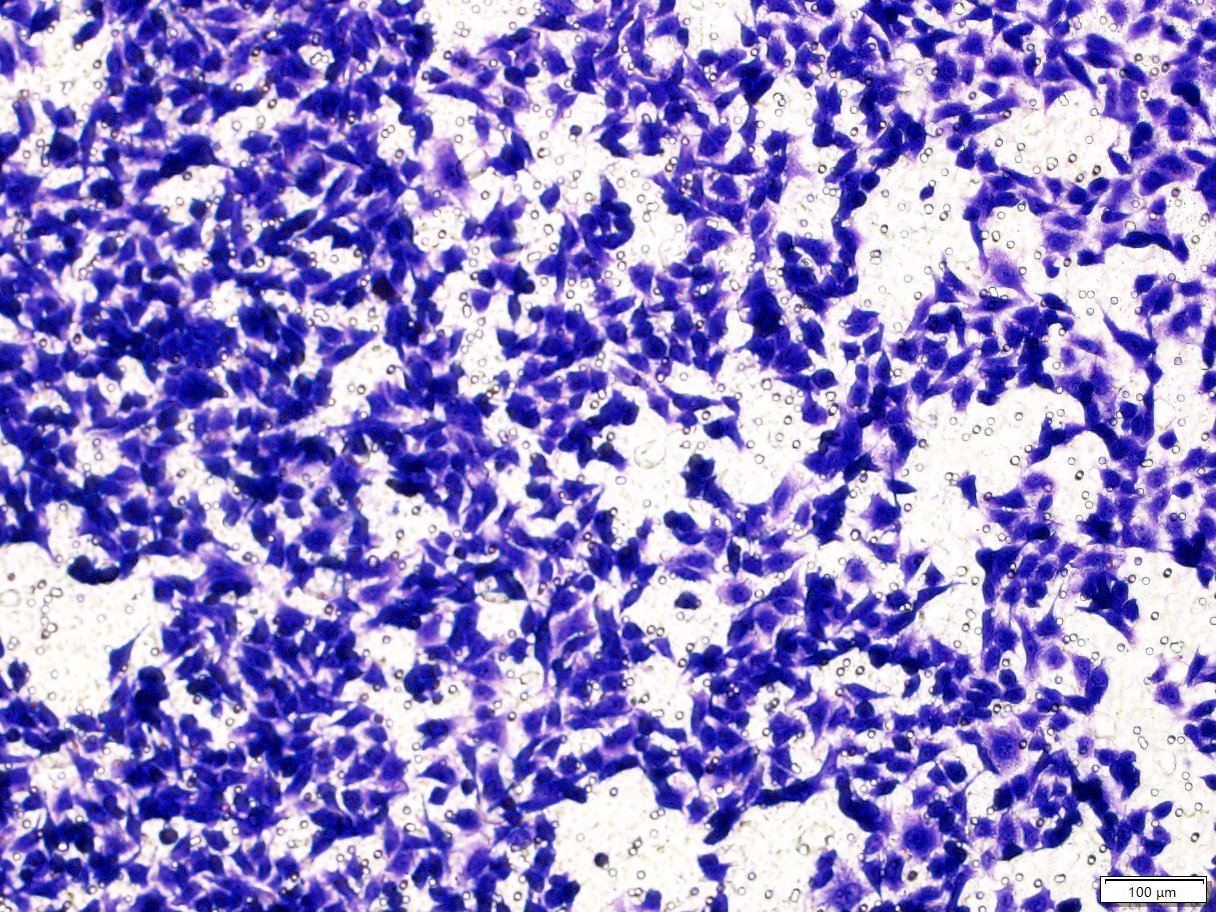

Supplement: Supplemental Information 11 [file peerj-cs-09-1651-s011.zip › Dataset 10/2-11.jpg]

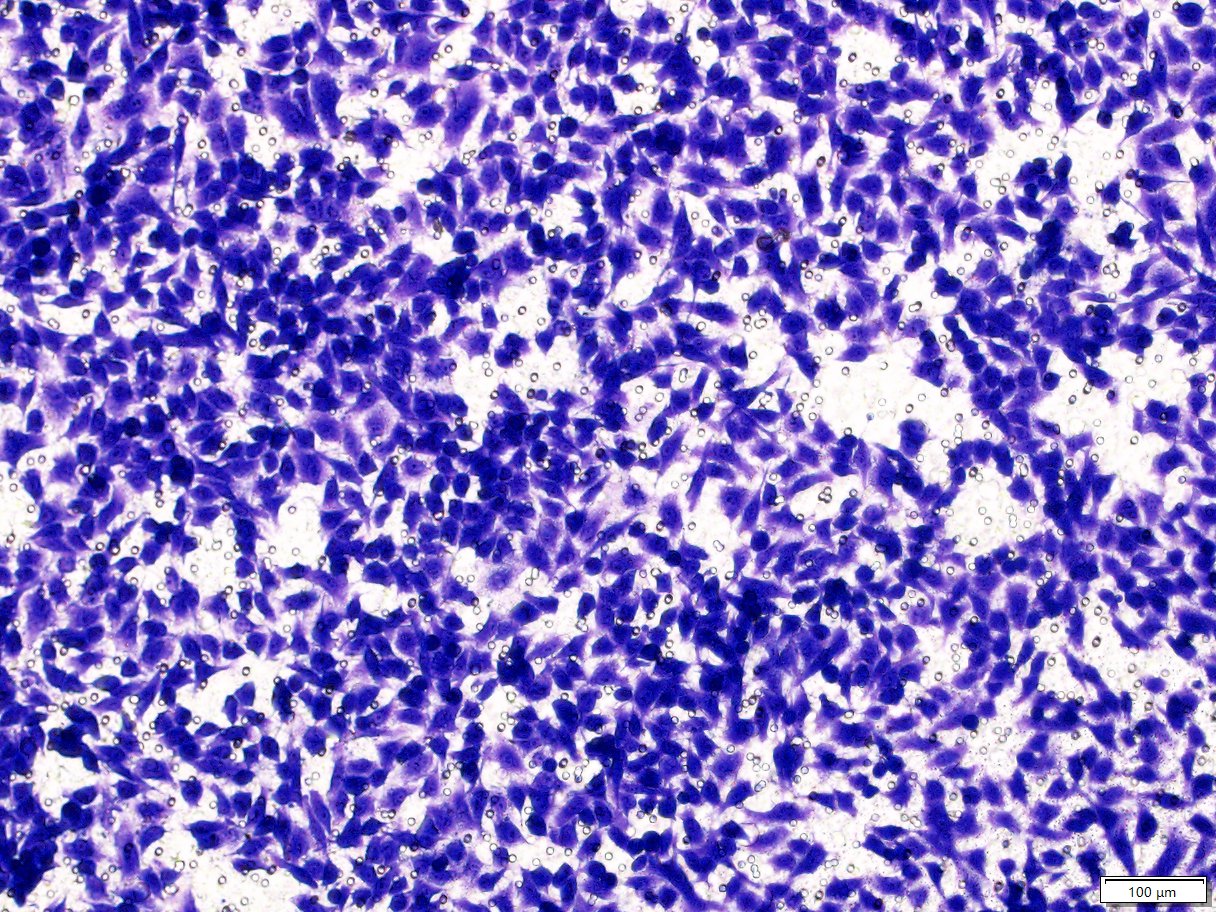

Supplement: Supplemental Information 11 [file peerj-cs-09-1651-s011.zip › Dataset 10/2-12.jpg]

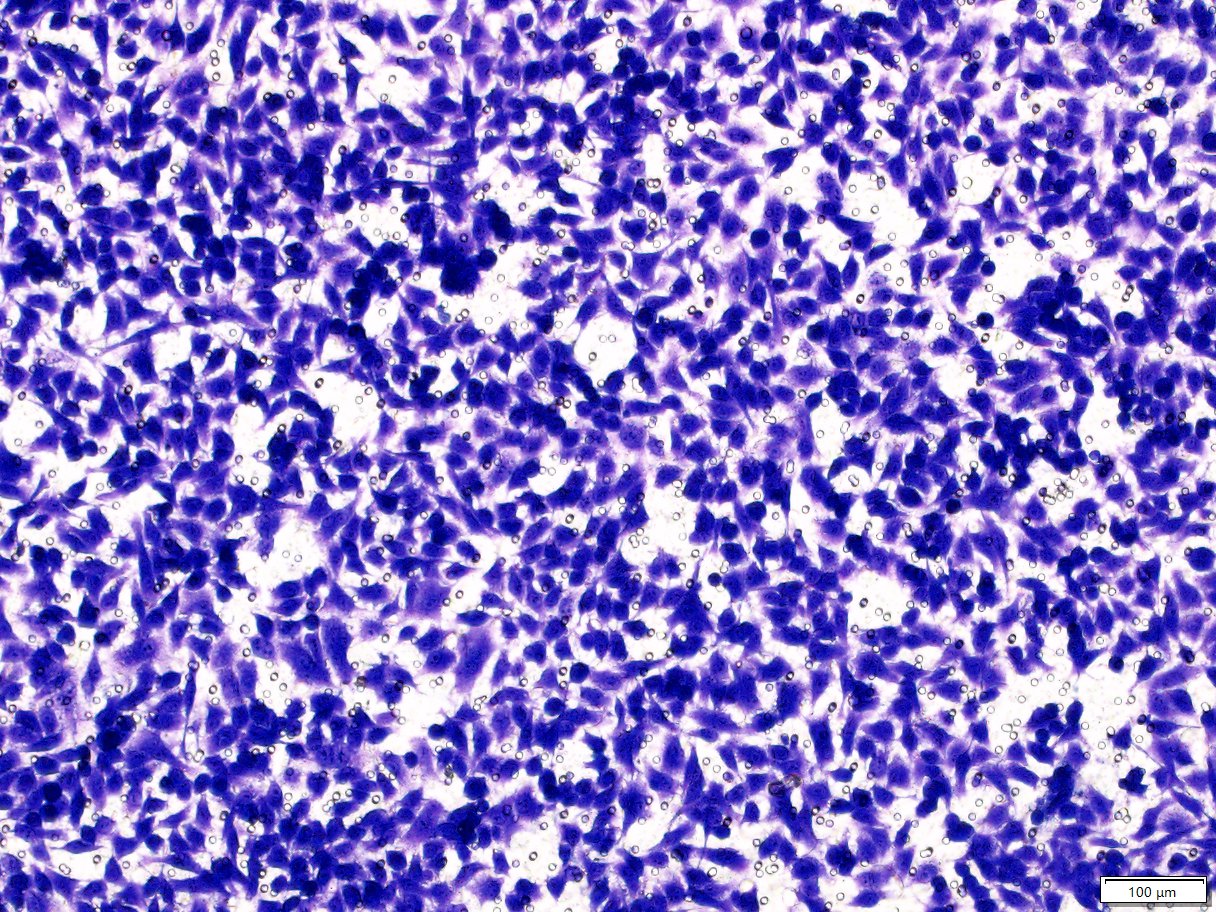

Supplement: Supplemental Information 11 [file peerj-cs-09-1651-s011.zip › Dataset 10/2-13.jpg]

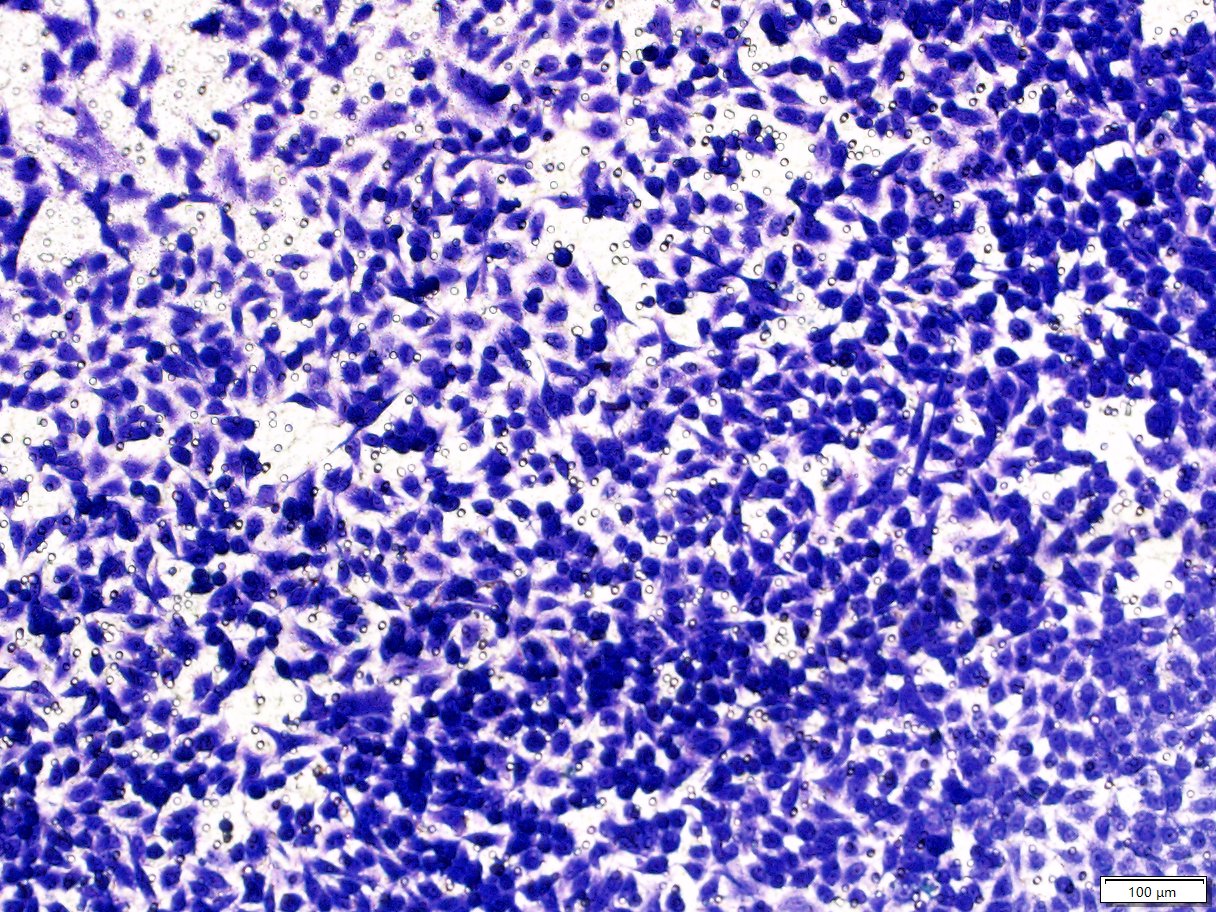

Supplement: Supplemental Information 11 [file peerj-cs-09-1651-s011.zip › Dataset 10/2-2.jpg]

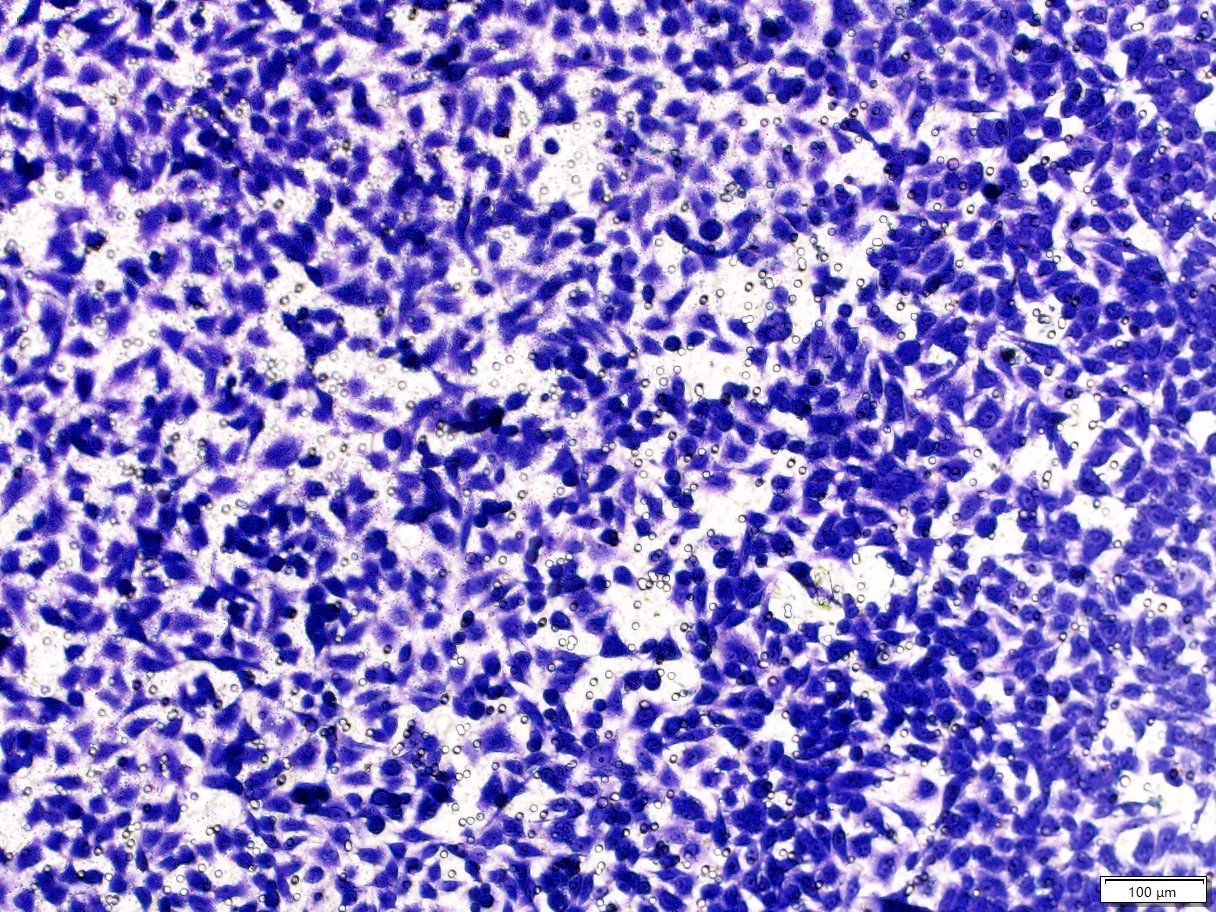

Supplement: Supplemental Information 11 [file peerj-cs-09-1651-s011.zip › Dataset 10/2-3.jpg]

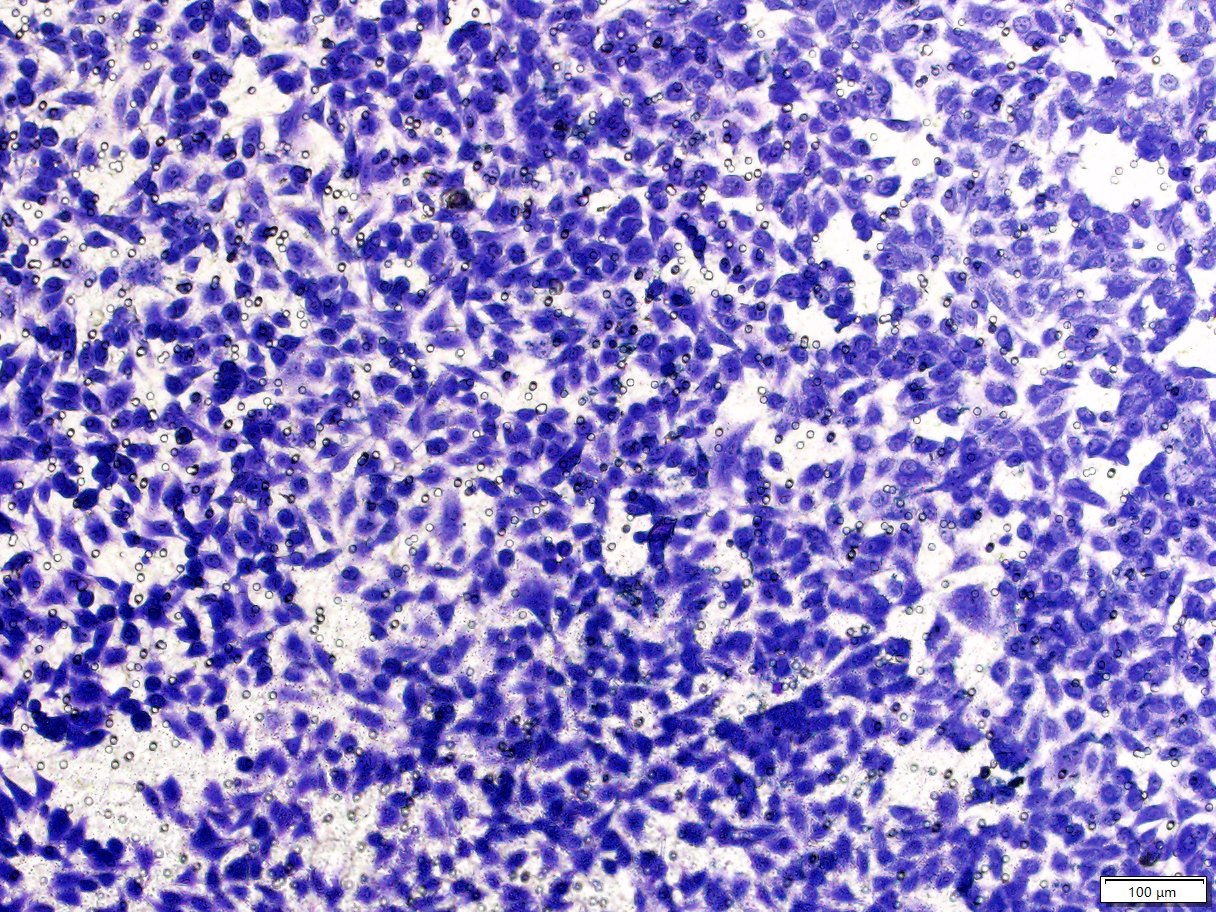

Supplement: Supplemental Information 11 [file peerj-cs-09-1651-s011.zip › Dataset 10/2-4.jpg]

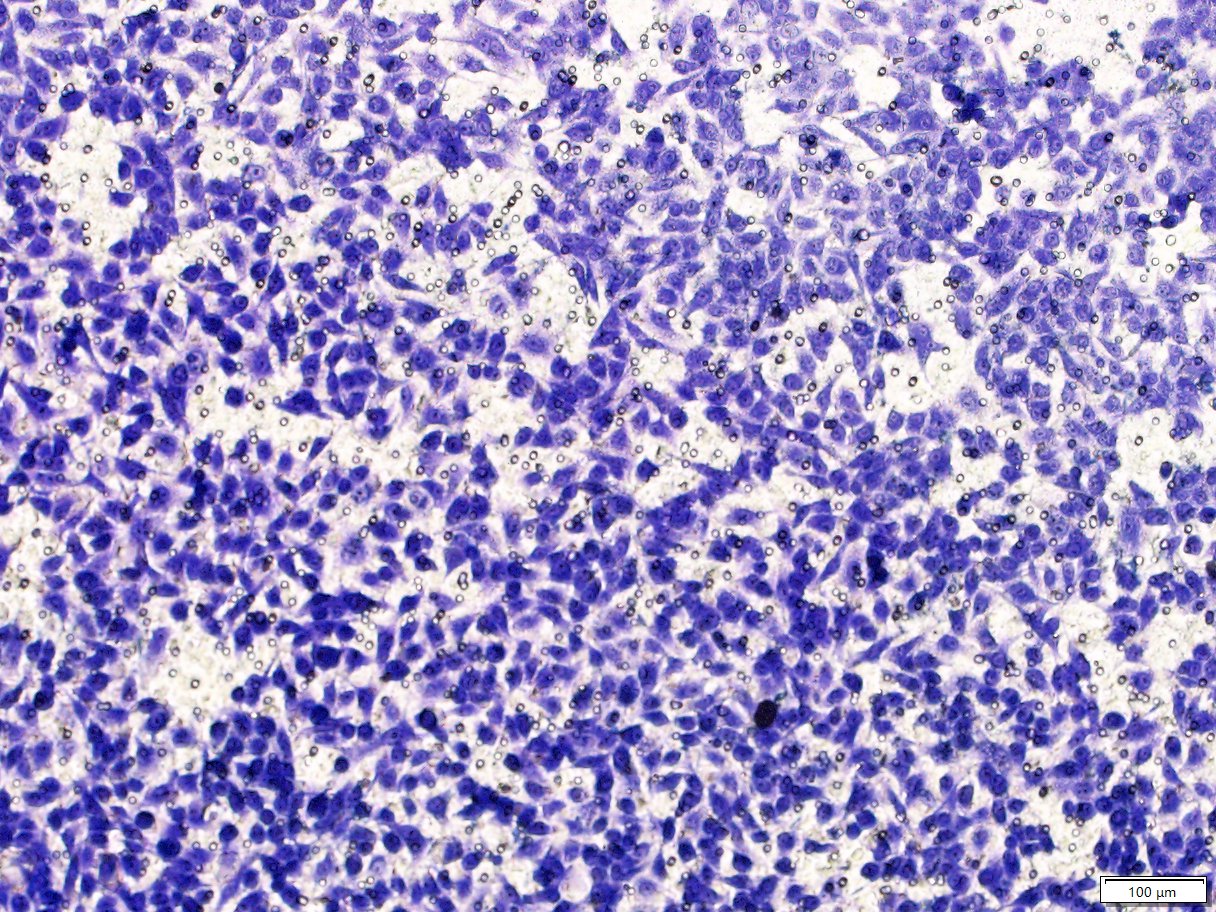

Supplement: Supplemental Information 11 [file peerj-cs-09-1651-s011.zip › Dataset 10/2-5.jpg]

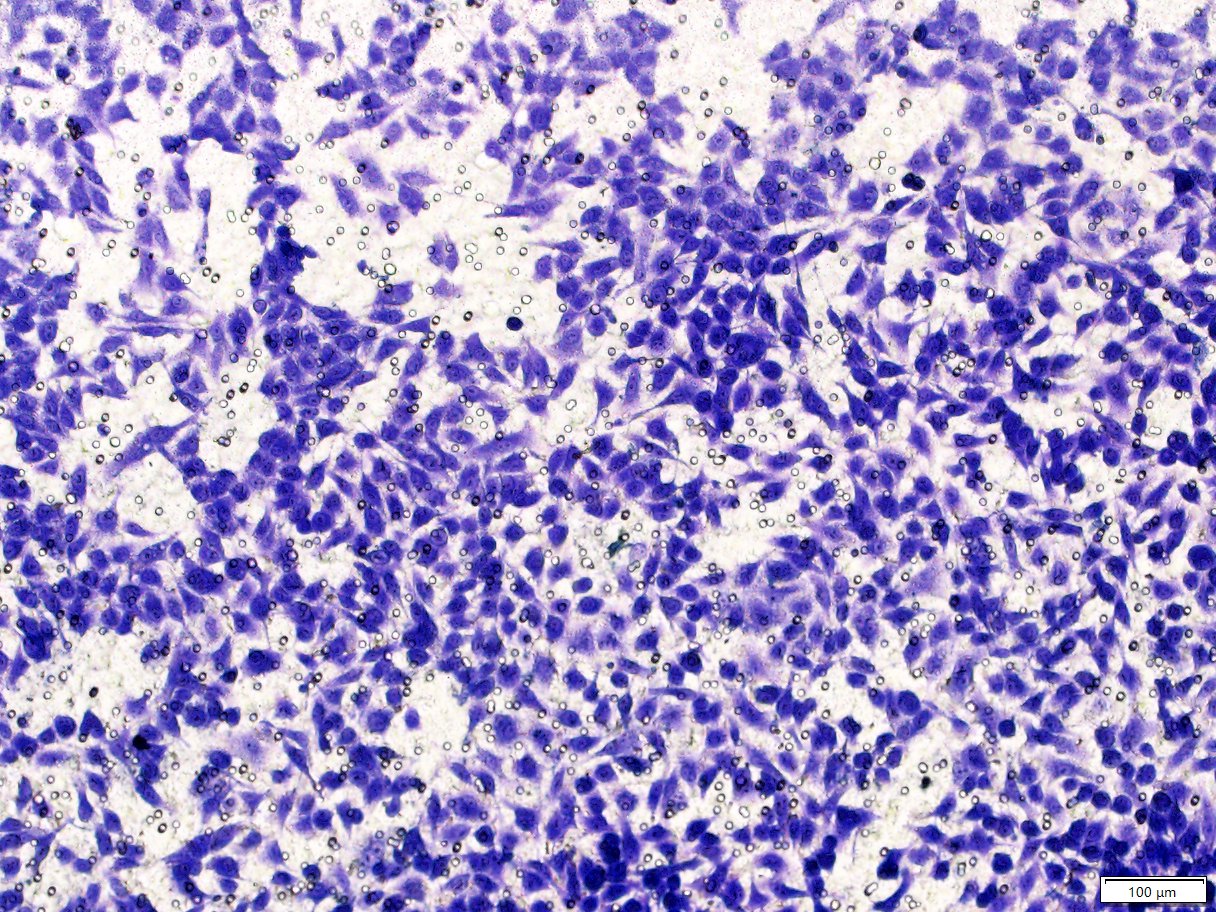

Supplement: Supplemental Information 11 [file peerj-cs-09-1651-s011.zip › Dataset 10/2-6.jpg]

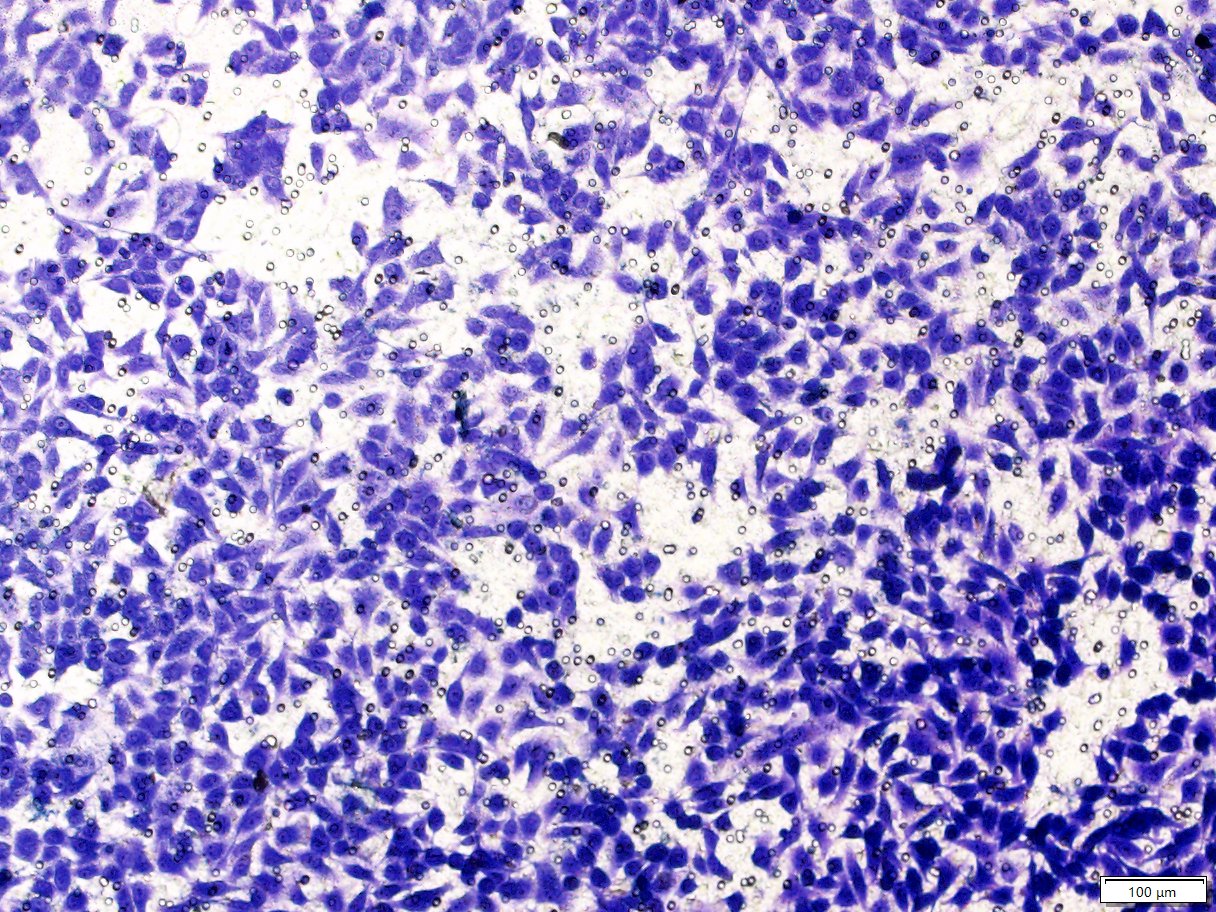

Supplement: Supplemental Information 11 [file peerj-cs-09-1651-s011.zip › Dataset 10/2-7.jpg]

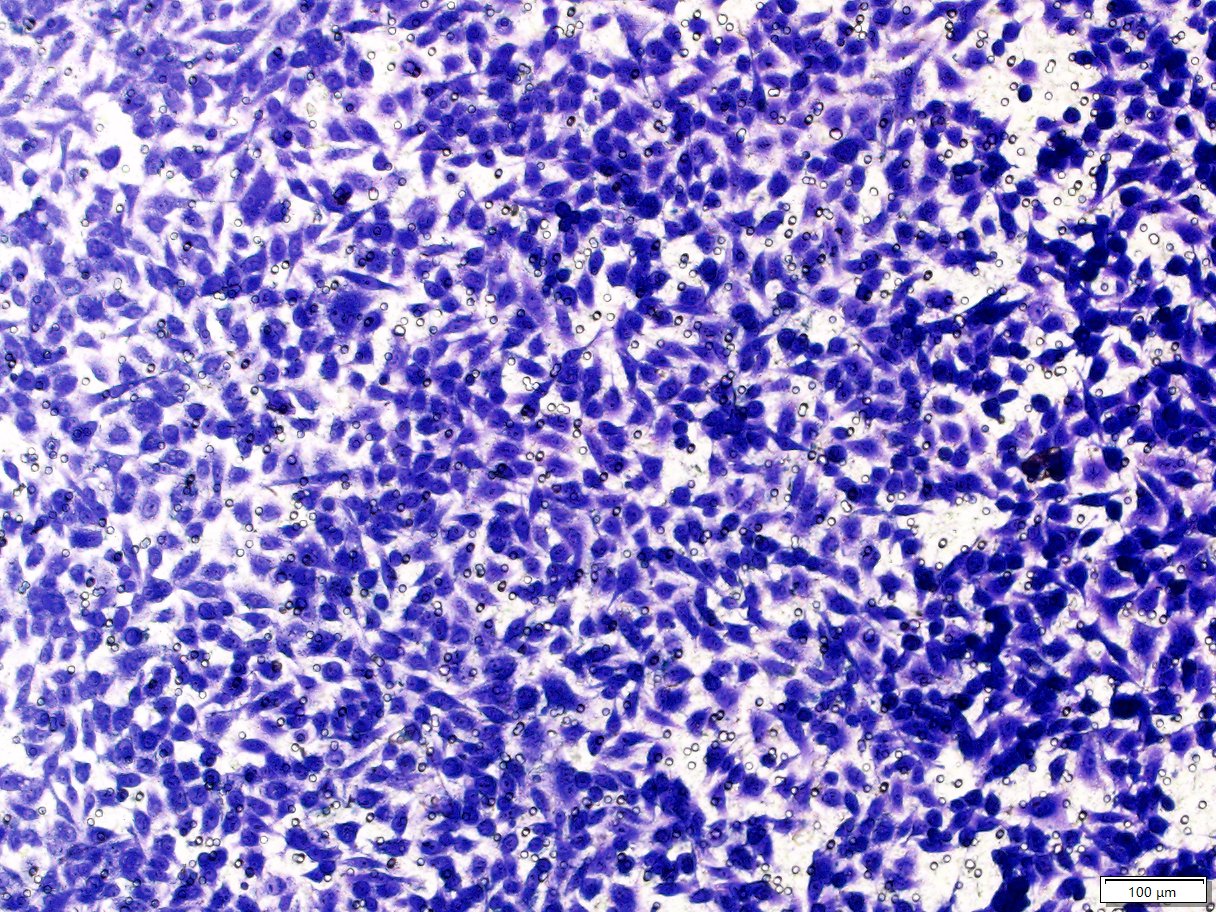

Supplement: Supplemental Information 11 [file peerj-cs-09-1651-s011.zip › Dataset 10/2-8.jpg]

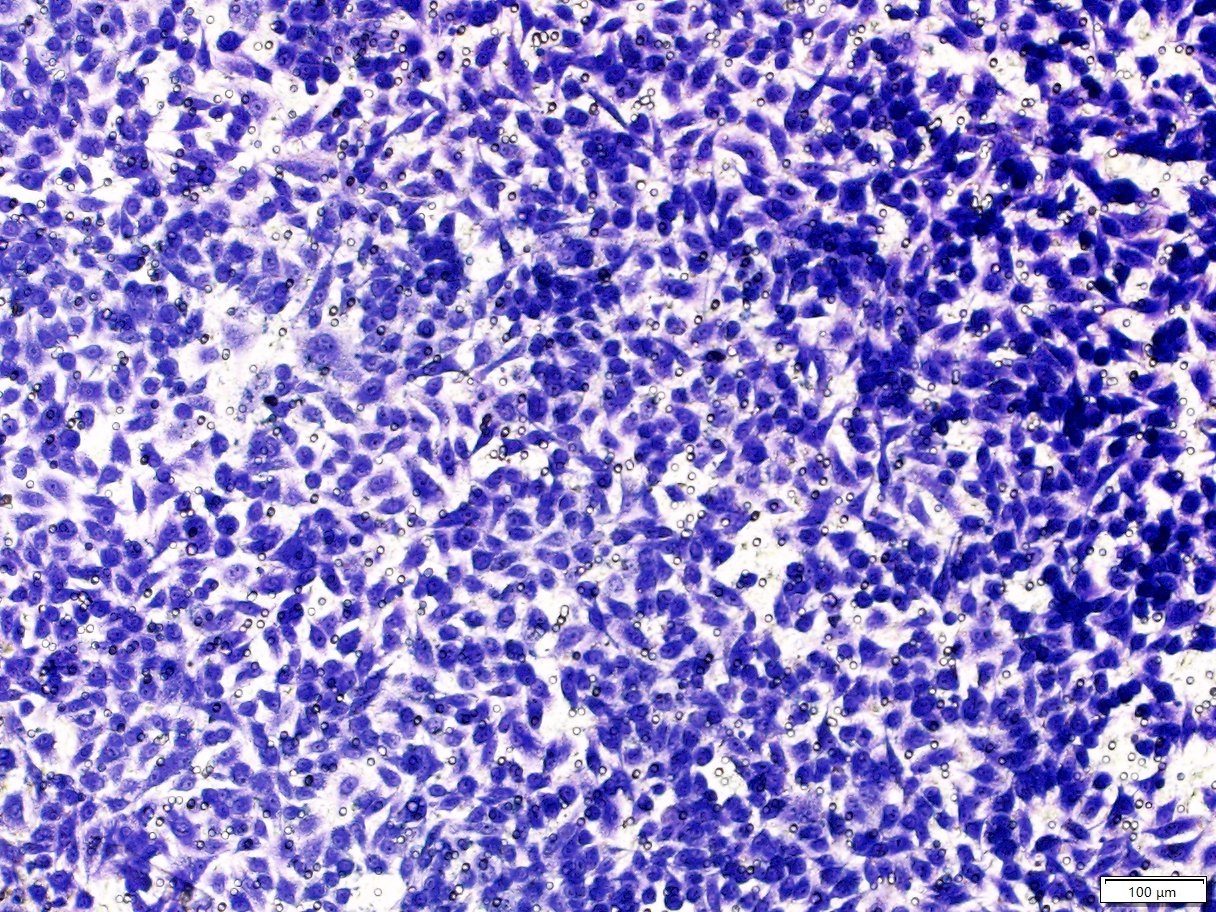

Supplement: Supplemental Information 11 [file peerj-cs-09-1651-s011.zip › Dataset 10/2-9.jpg]

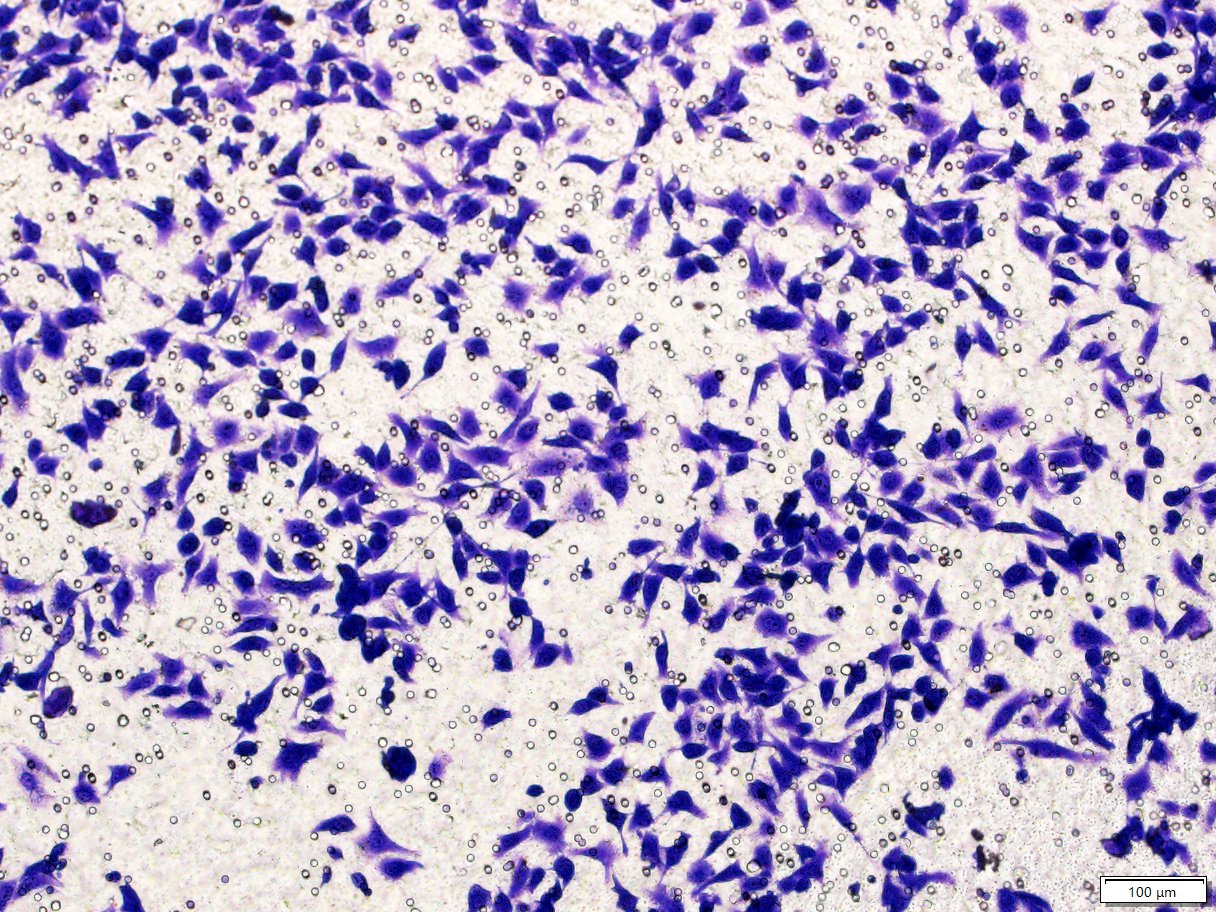

Supplement: Supplemental Information 11 [file peerj-cs-09-1651-s011.zip › Dataset 10/3+1.jpg]

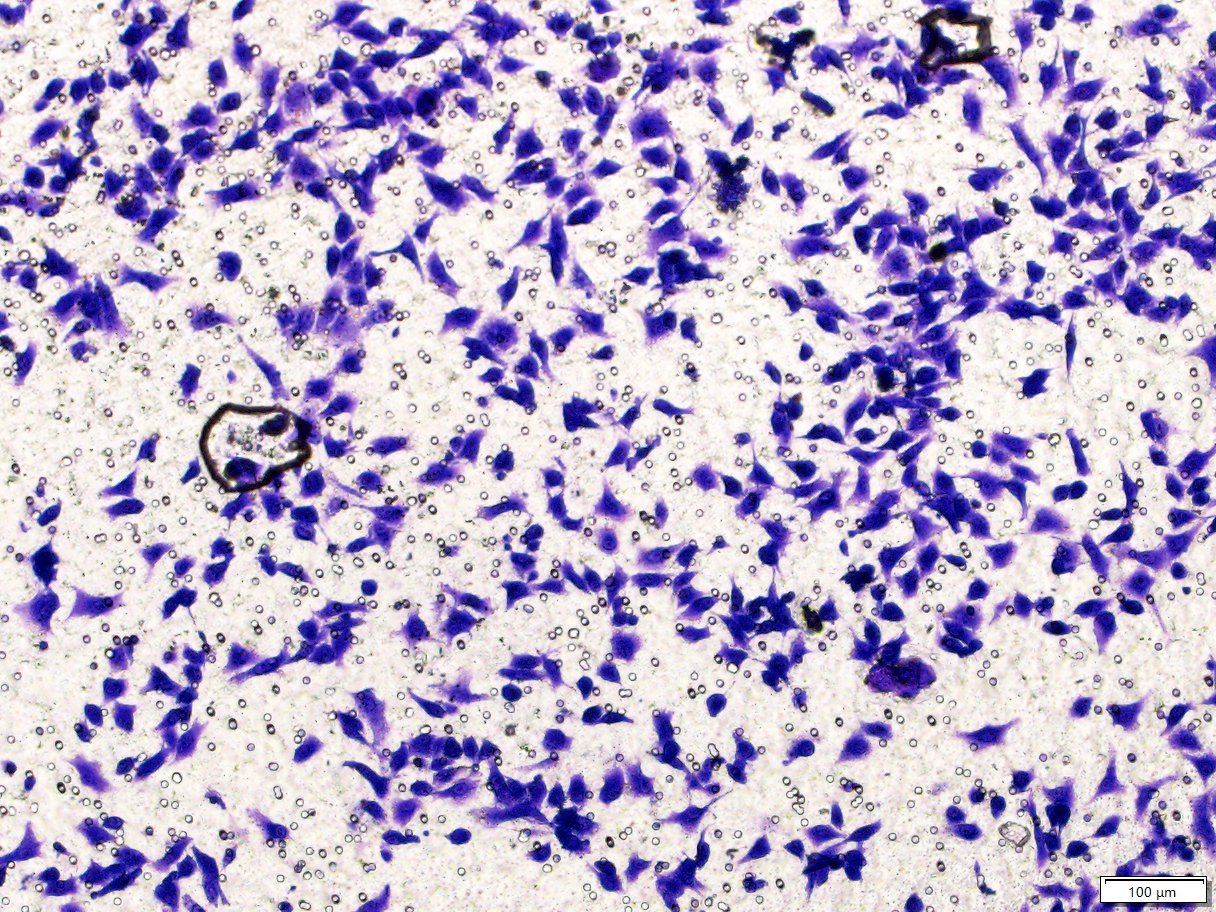

Supplement: Supplemental Information 11 [file peerj-cs-09-1651-s011.zip › Dataset 10/3+10.jpg]

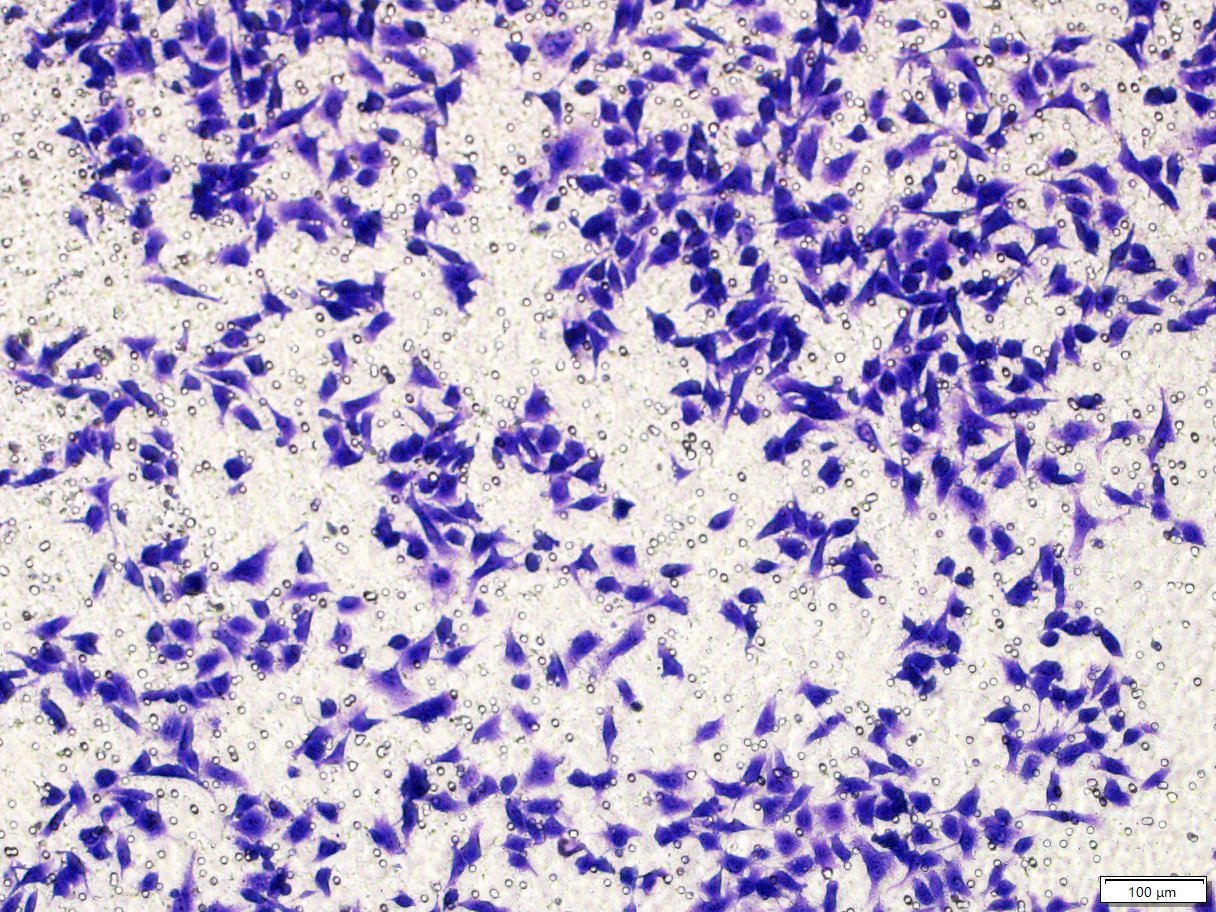

Supplement: Supplemental Information 11 [file peerj-cs-09-1651-s011.zip › Dataset 10/3+11.jpg]

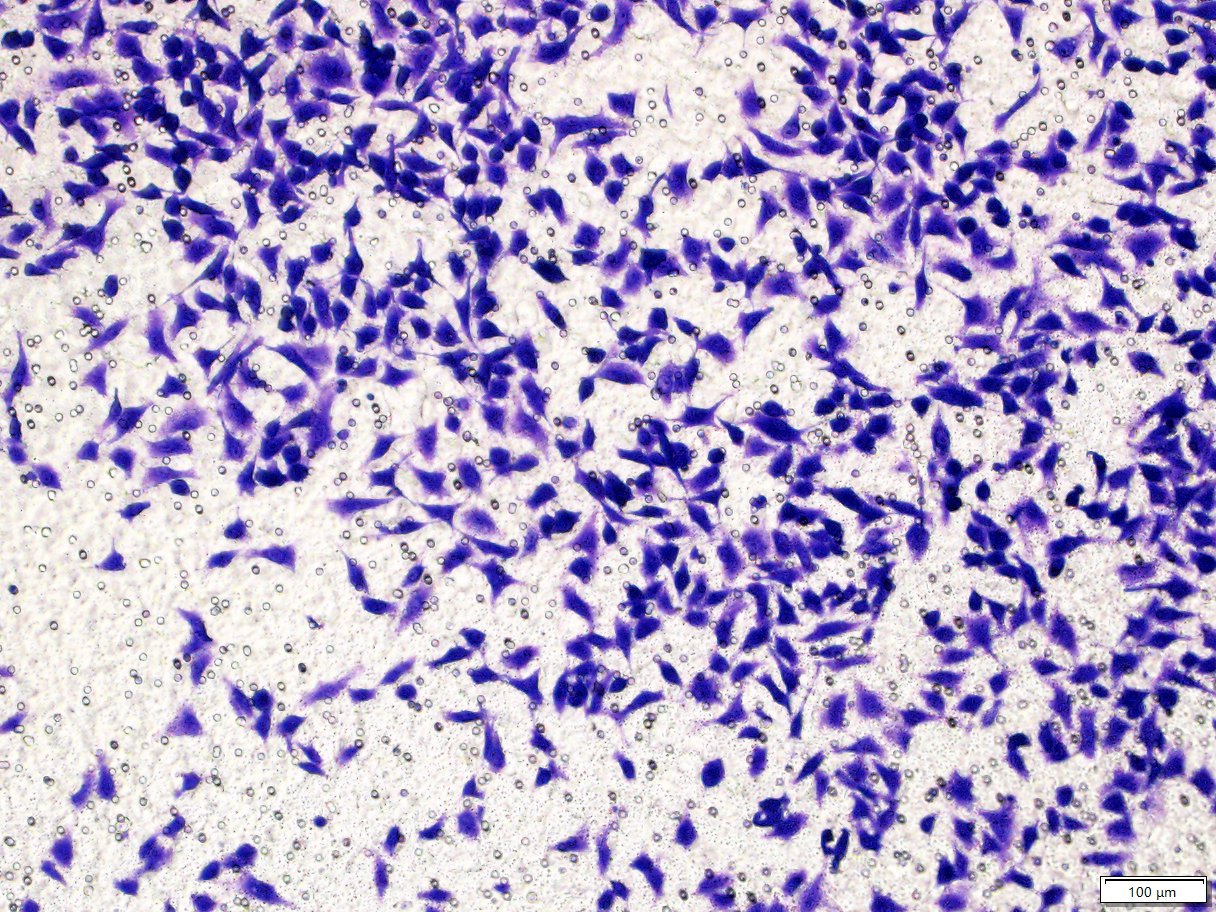

Supplement: Supplemental Information 11 [file peerj-cs-09-1651-s011.zip › Dataset 10/3+12.jpg]

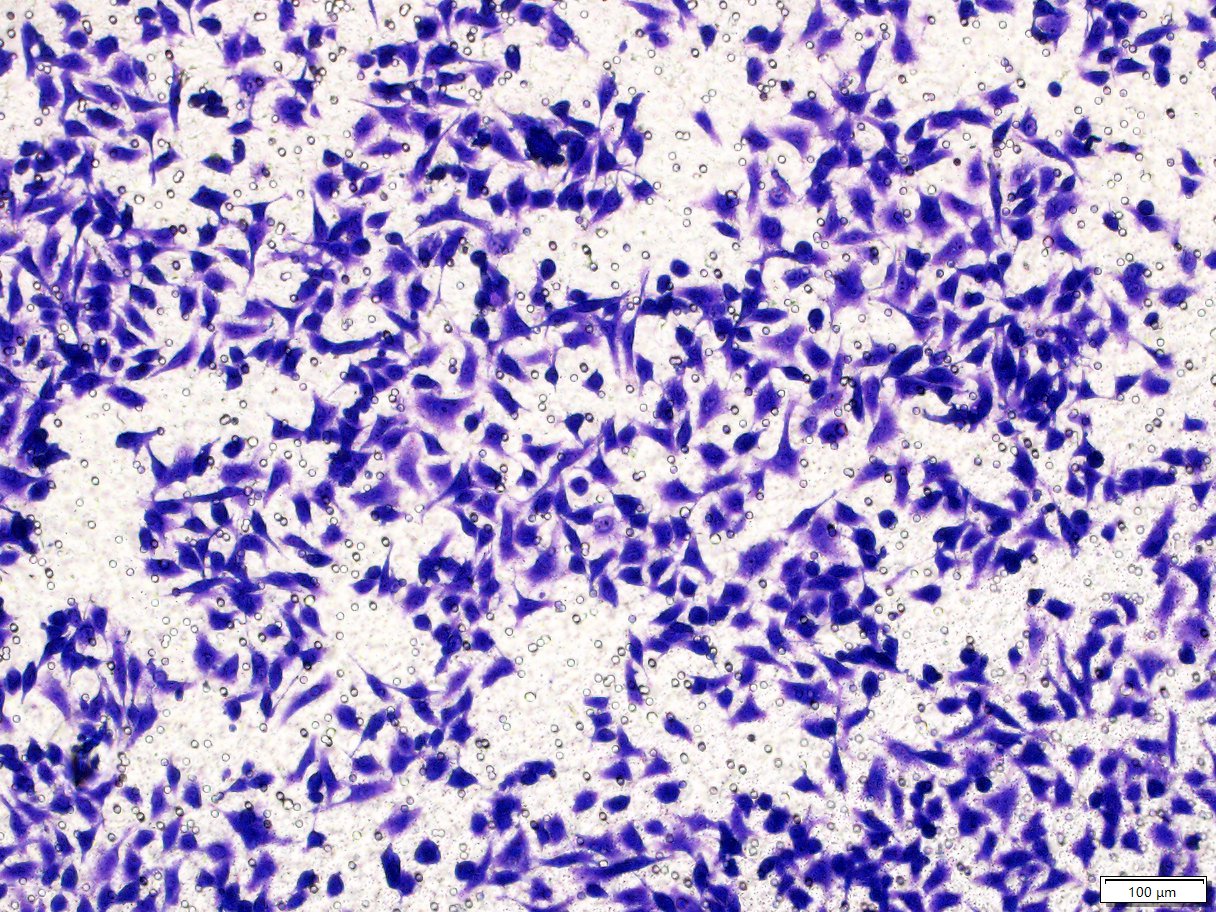

Supplement: Supplemental Information 11 [file peerj-cs-09-1651-s011.zip › Dataset 10/3+13.jpg]

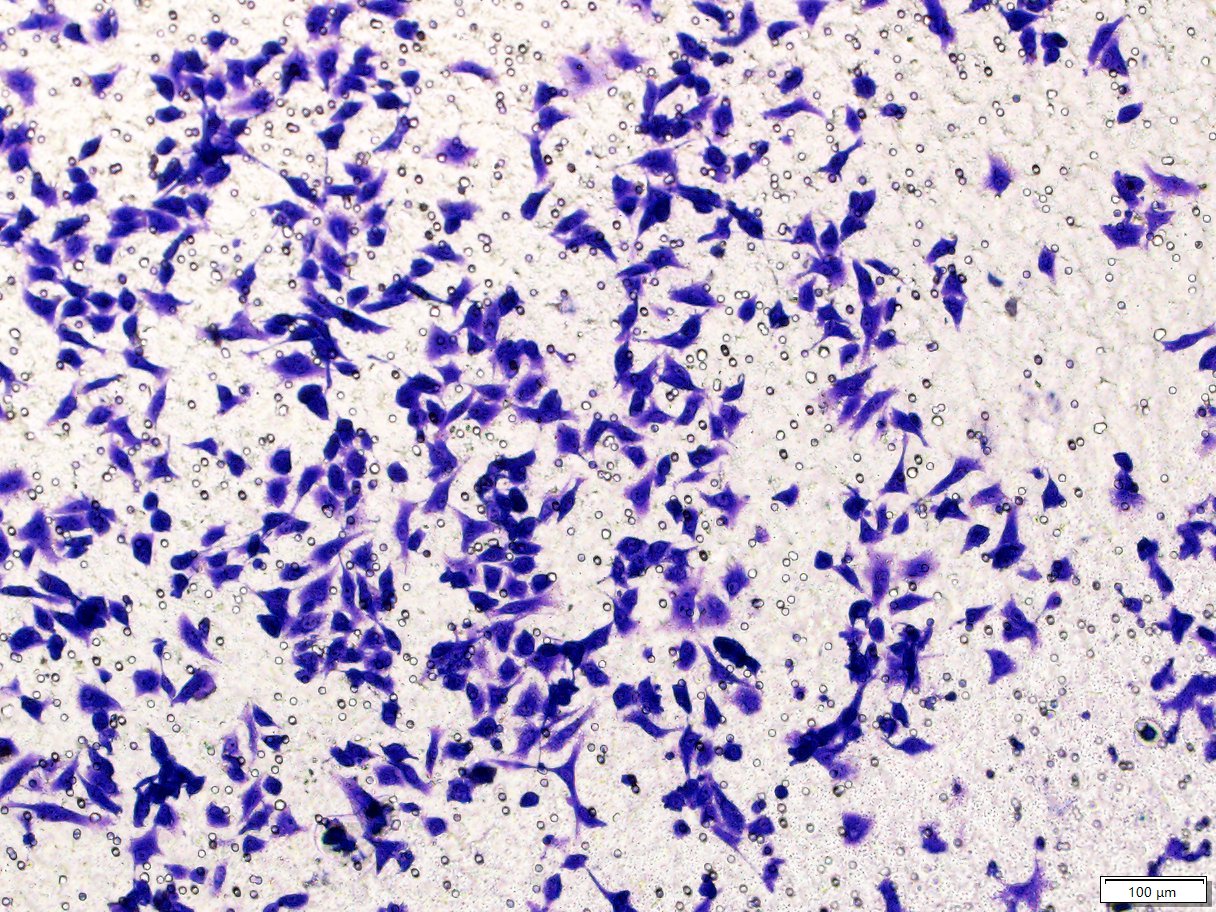

Supplement: Supplemental Information 11 [file peerj-cs-09-1651-s011.zip › Dataset 10/3+2.jpg]

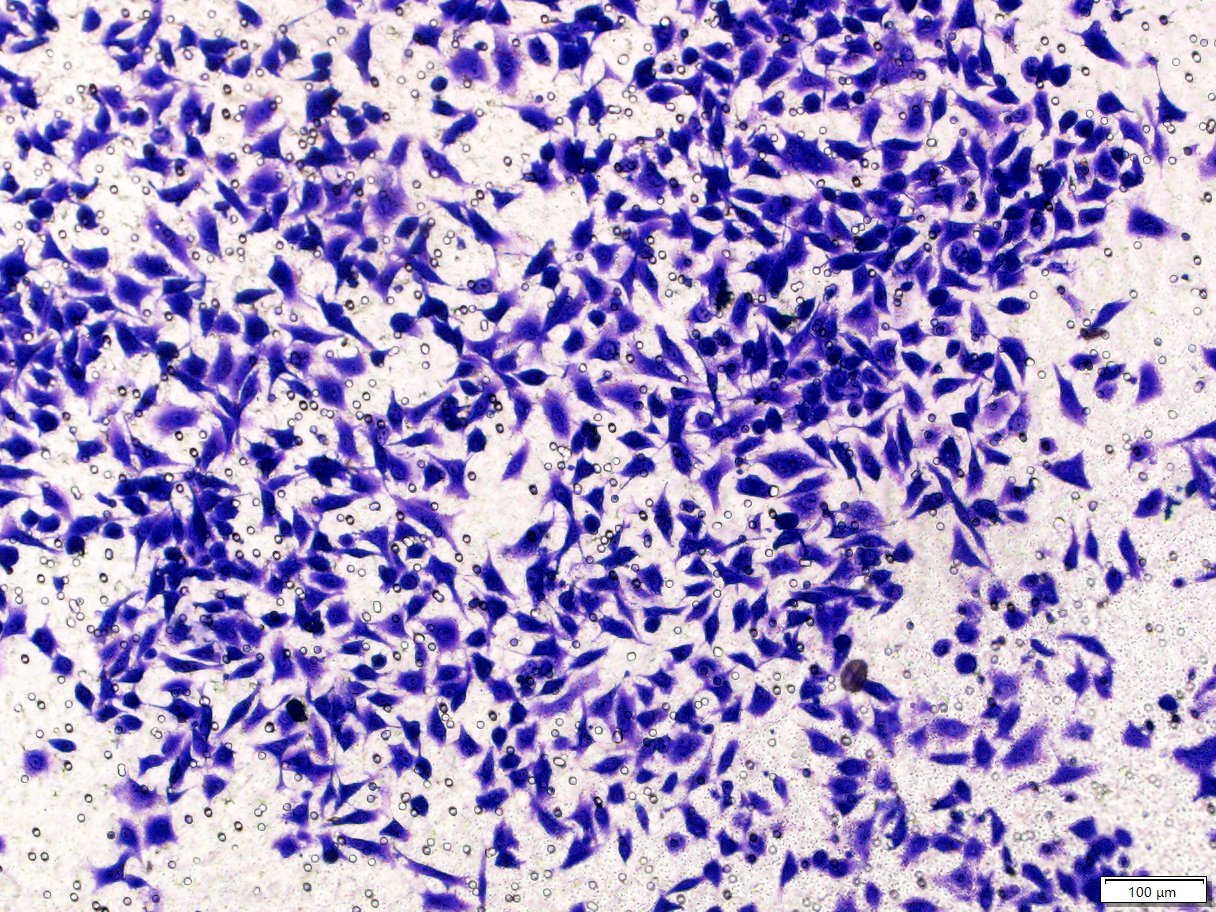

Supplement: Supplemental Information 11 [file peerj-cs-09-1651-s011.zip › Dataset 10/3+3.jpg]

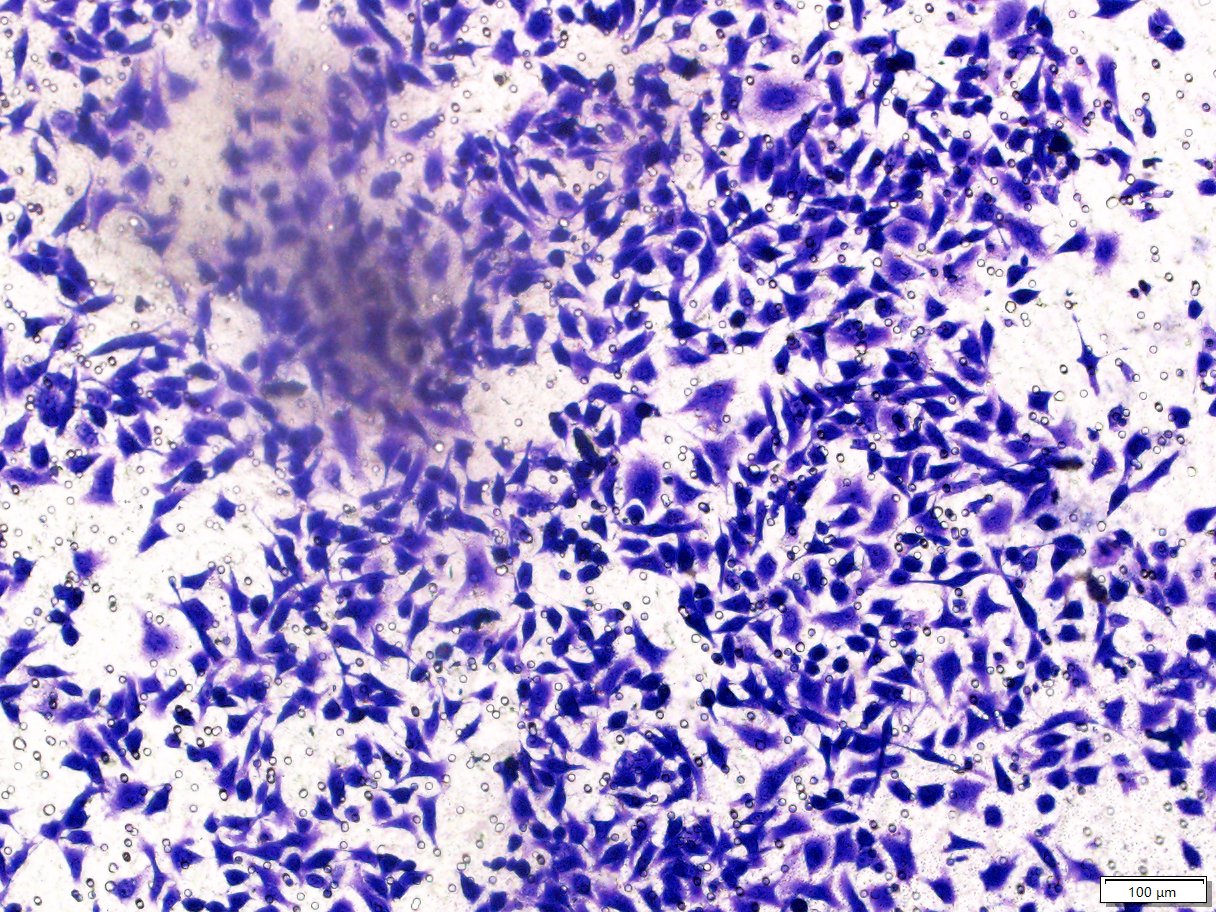

Supplement: Supplemental Information 11 [file peerj-cs-09-1651-s011.zip › Dataset 10/3+4.jpg]

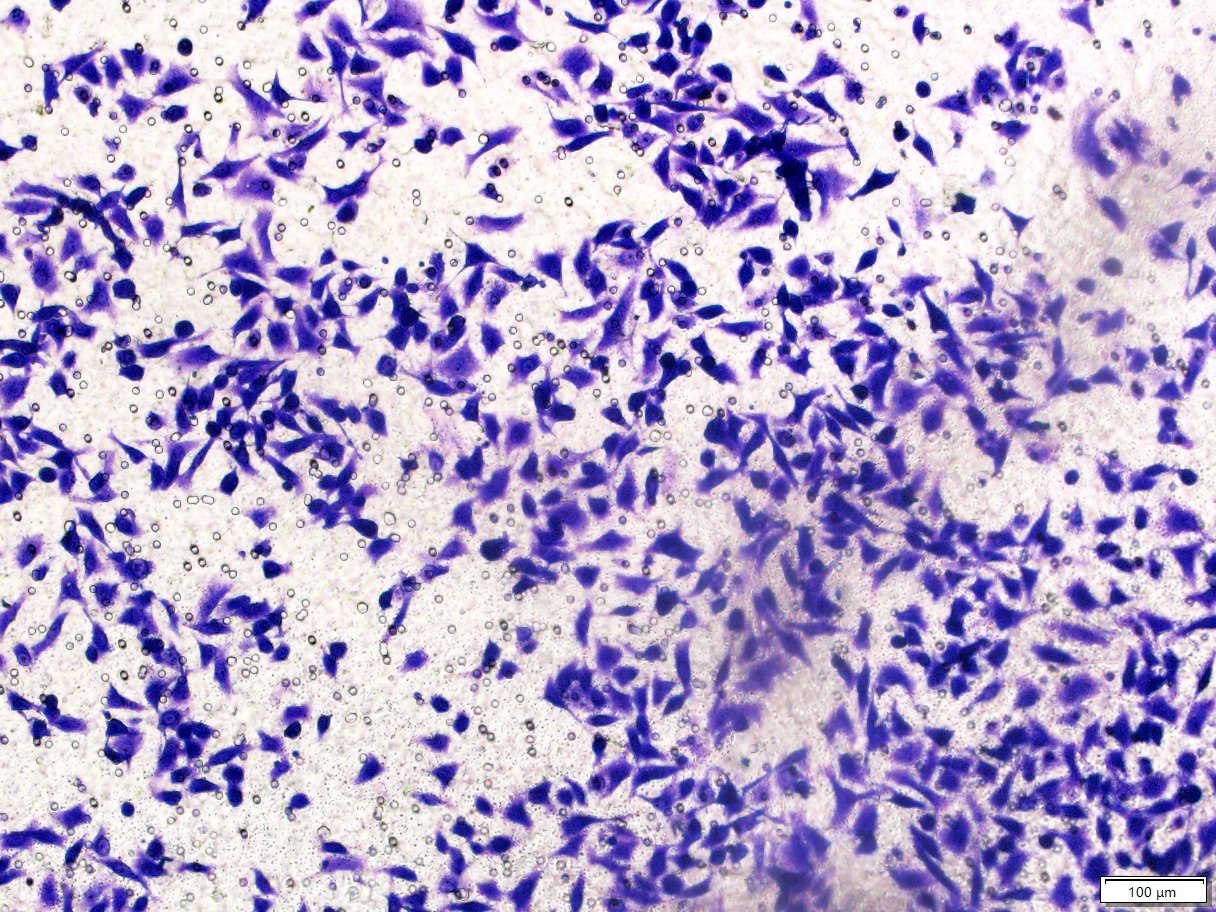

Supplement: Supplemental Information 11 [file peerj-cs-09-1651-s011.zip › Dataset 10/3+5.jpg]
