# Supplementary figures and images for: A deep learning approach for the detection and counting of colon cancer cells (HT-29 cells) bunches and impurities (part 6 of 6)
Source: PeerJ Comput Sci. 2023 Dec 5;9:e1651. doi: 10.7717/peerj-cs.1651 (PMC10773923; doi:10.7717/peerj-cs.1651)

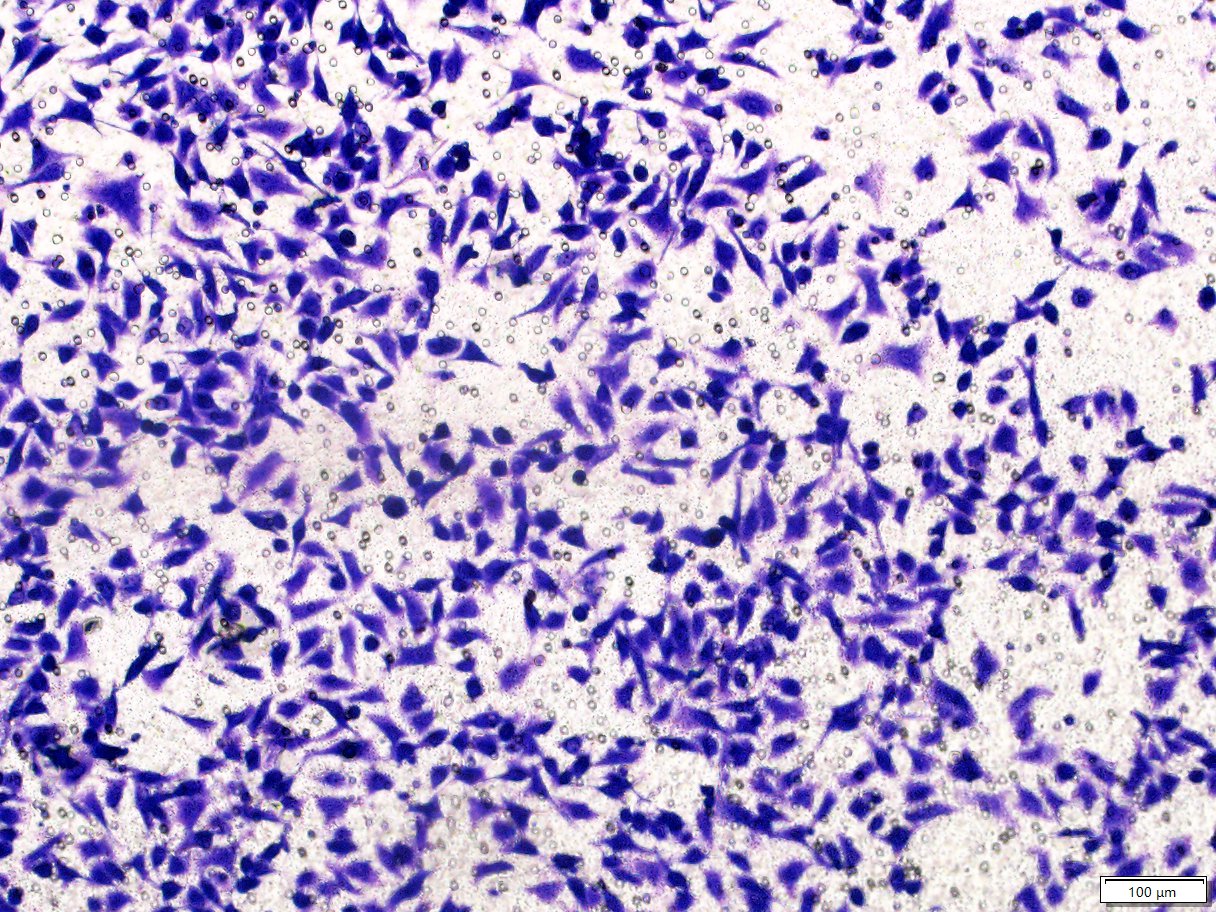

Supplement: Supplemental Information 11 [file peerj-cs-09-1651-s011.zip › Dataset 10/3+6.jpg]

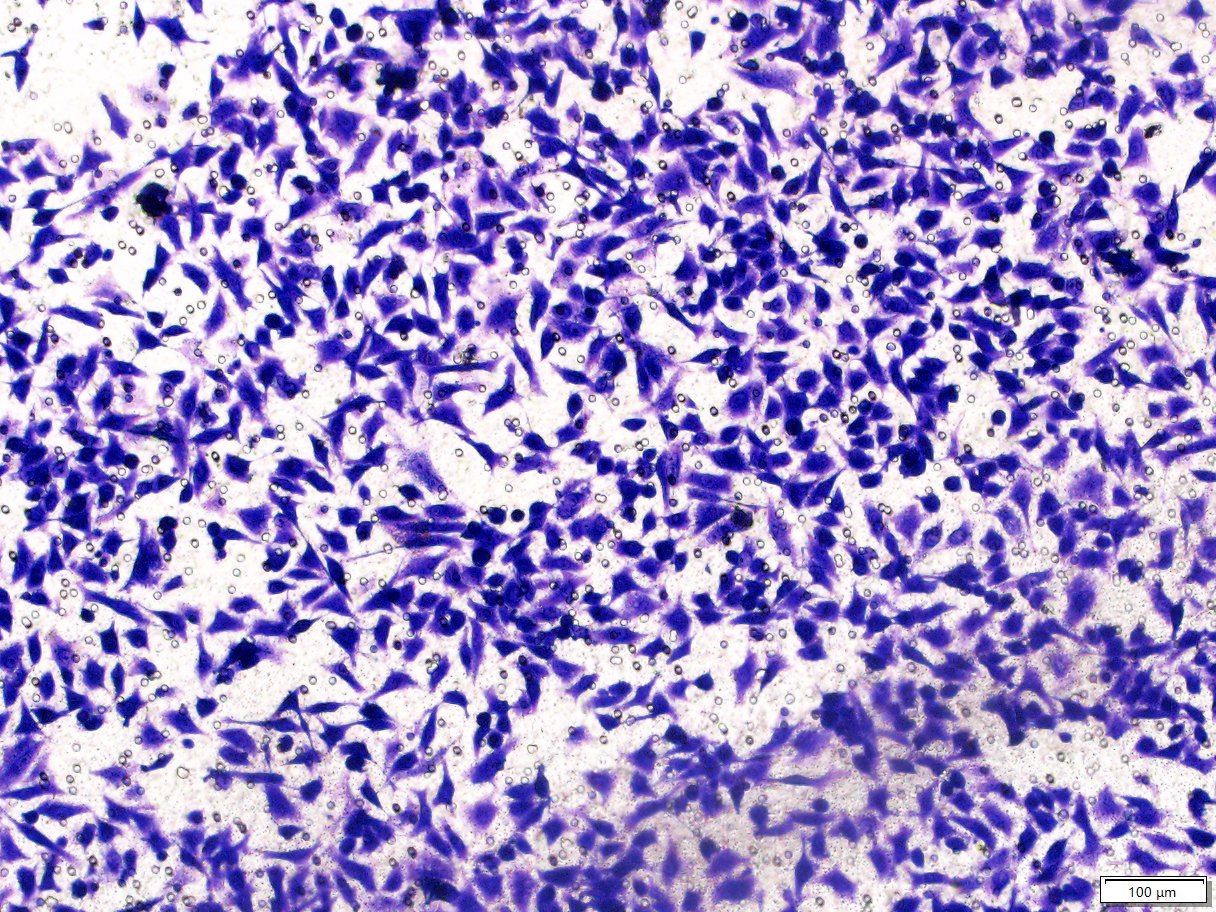

Supplement: Supplemental Information 11 [file peerj-cs-09-1651-s011.zip › Dataset 10/3+7.jpg]

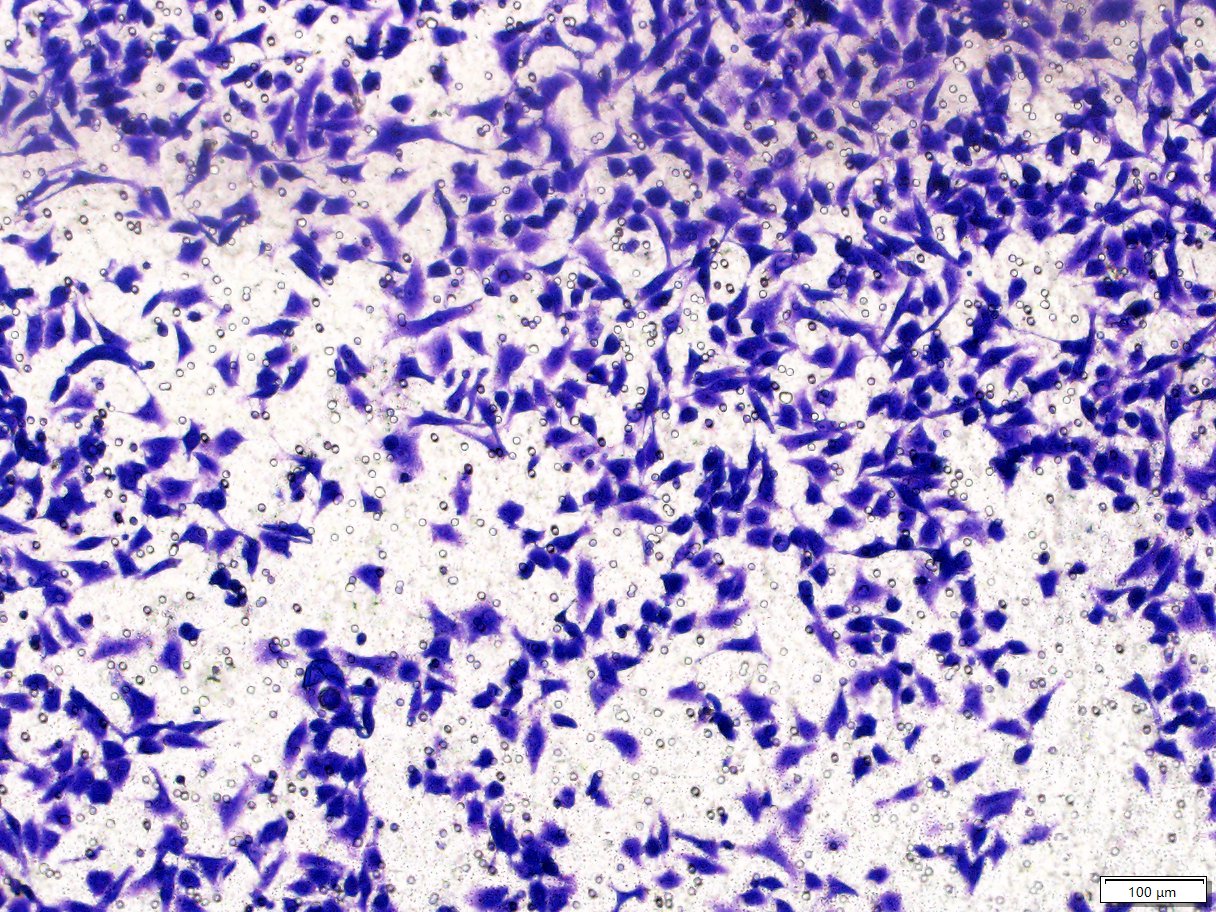

Supplement: Supplemental Information 11 [file peerj-cs-09-1651-s011.zip › Dataset 10/3+8.jpg]

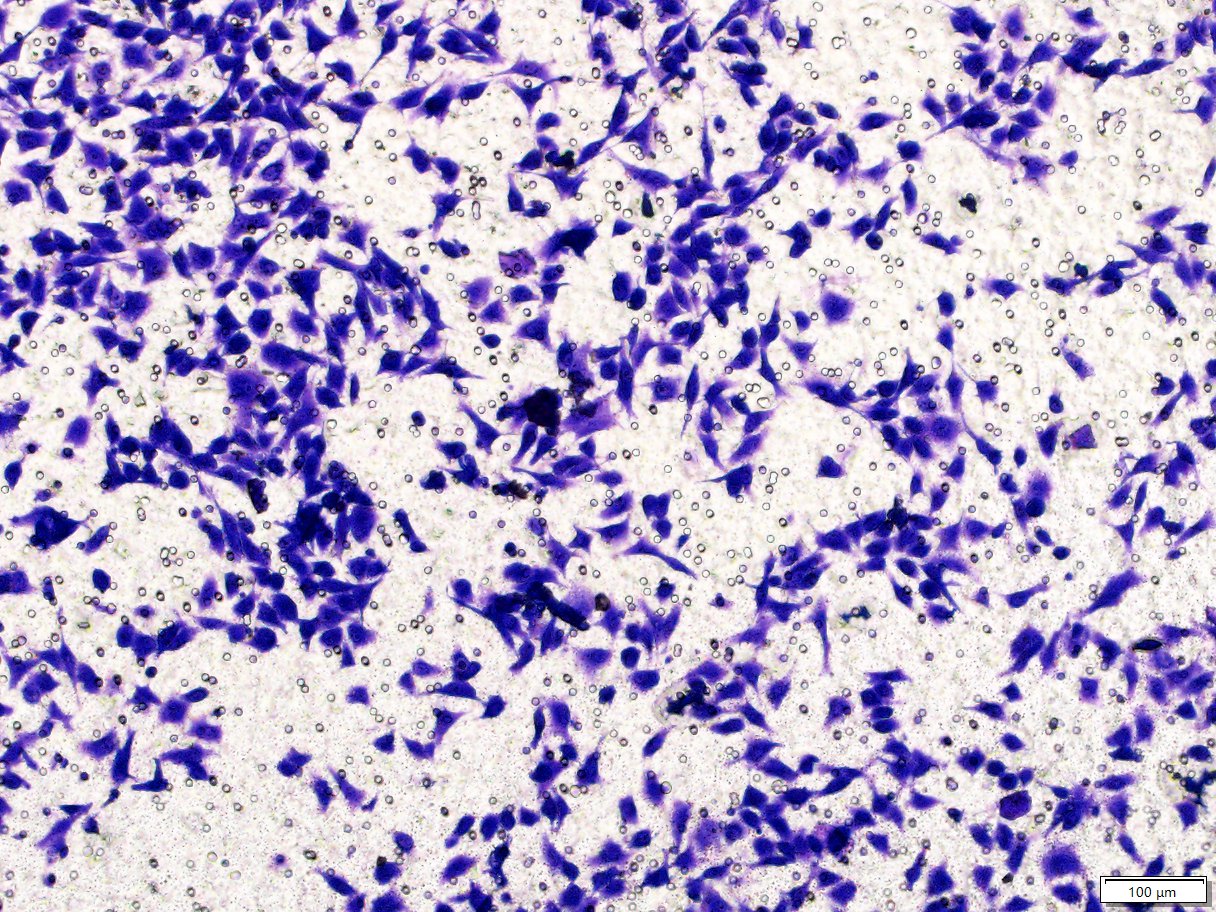

Supplement: Supplemental Information 11 [file peerj-cs-09-1651-s011.zip › Dataset 10/3+9.jpg]

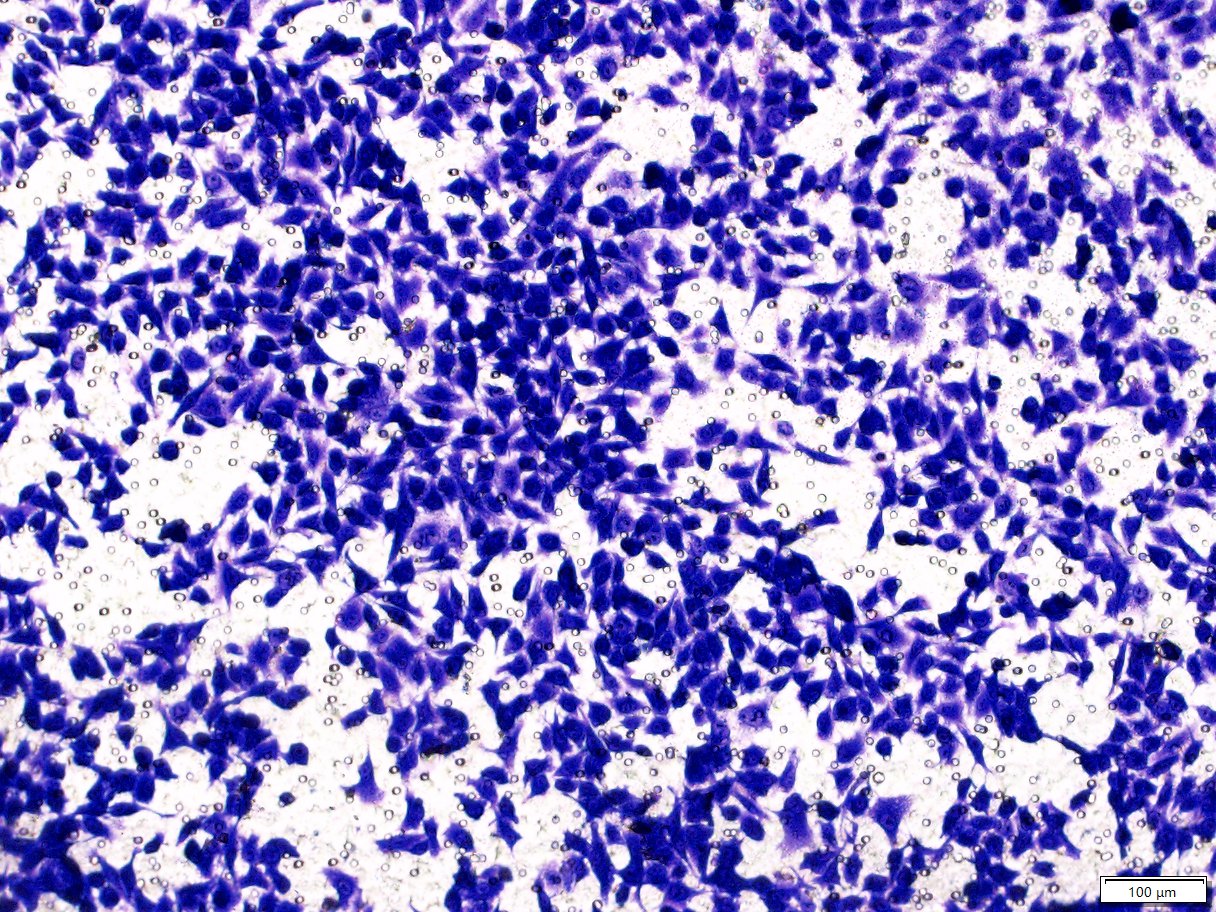

Supplement: Supplemental Information 11 [file peerj-cs-09-1651-s011.zip › Dataset 10/3-1.jpg]

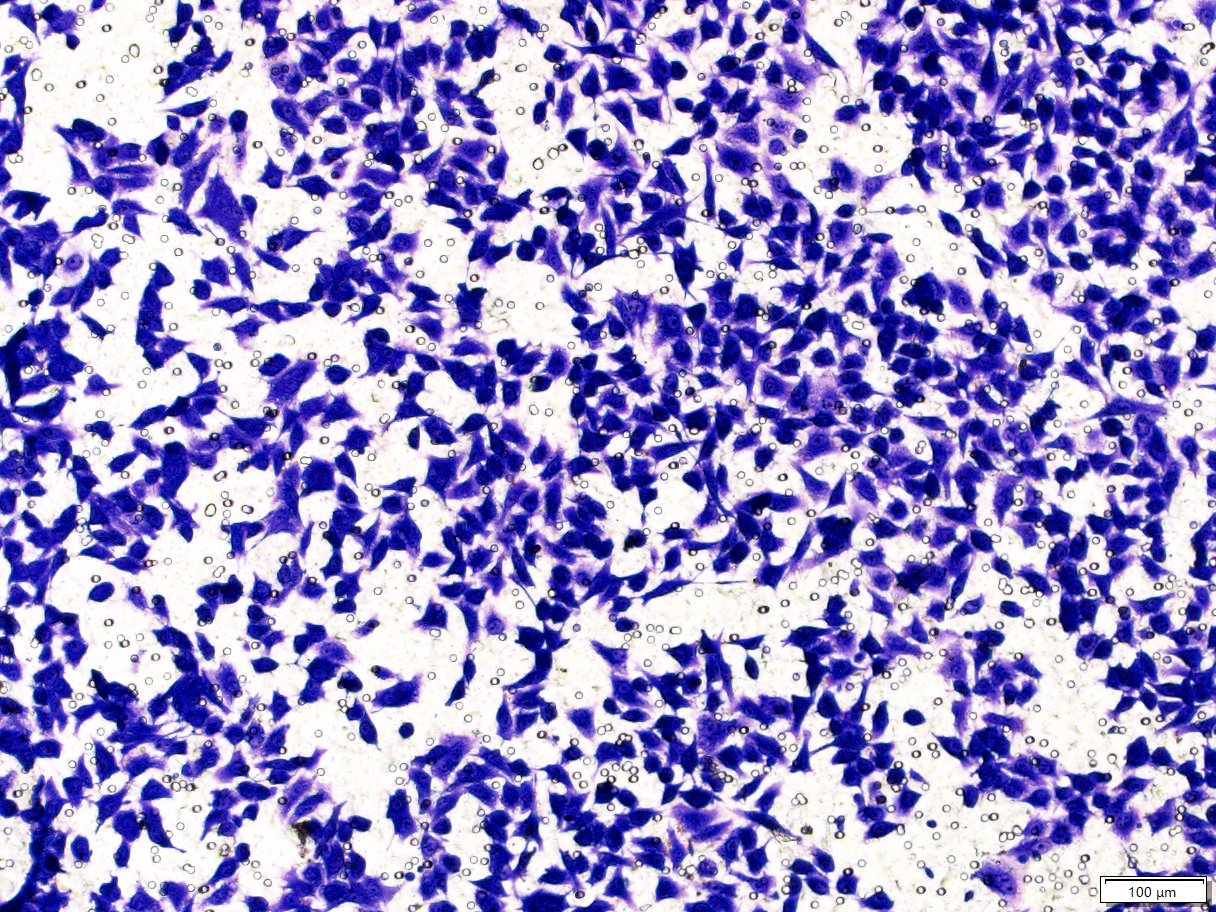

Supplement: Supplemental Information 11 [file peerj-cs-09-1651-s011.zip › Dataset 10/3-10.jpg]

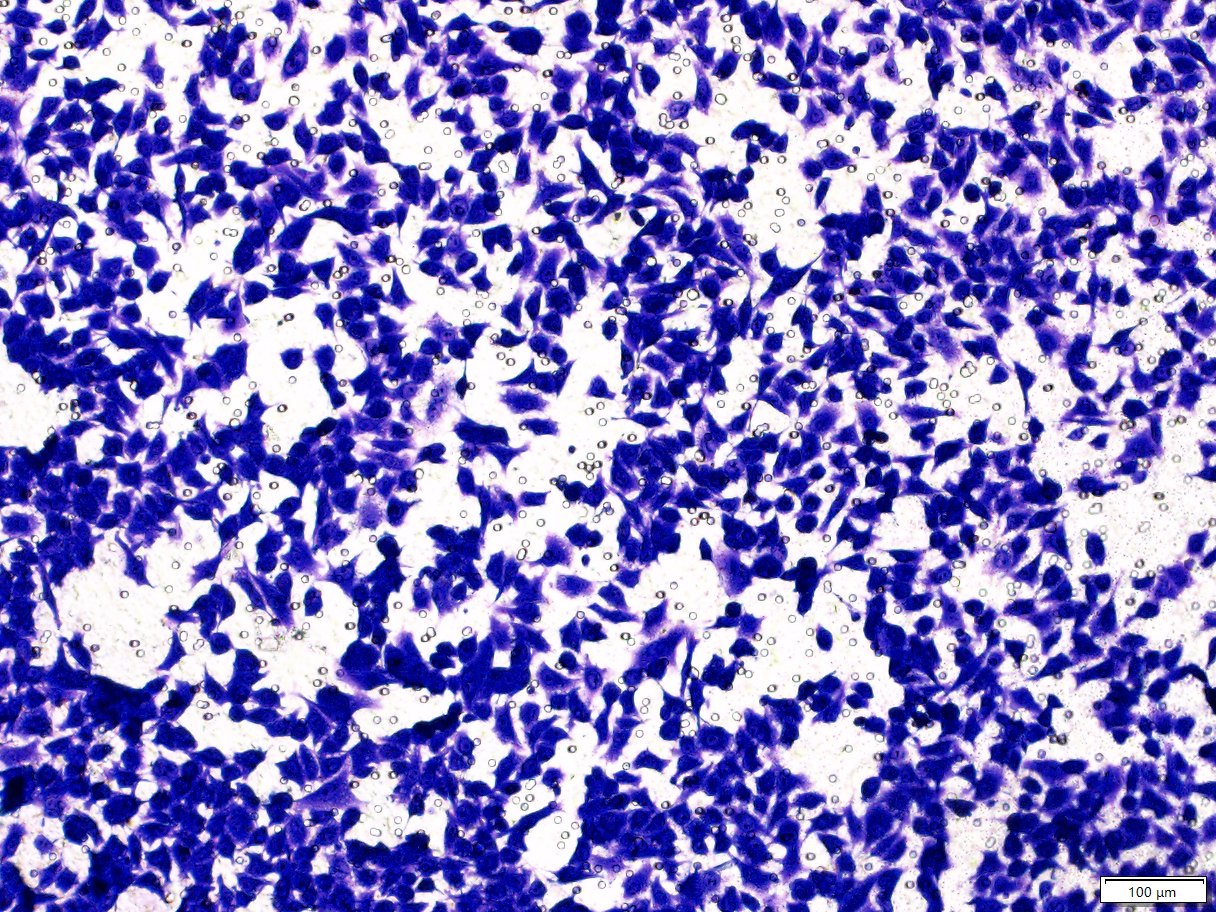

Supplement: Supplemental Information 11 [file peerj-cs-09-1651-s011.zip › Dataset 10/3-11.jpg]

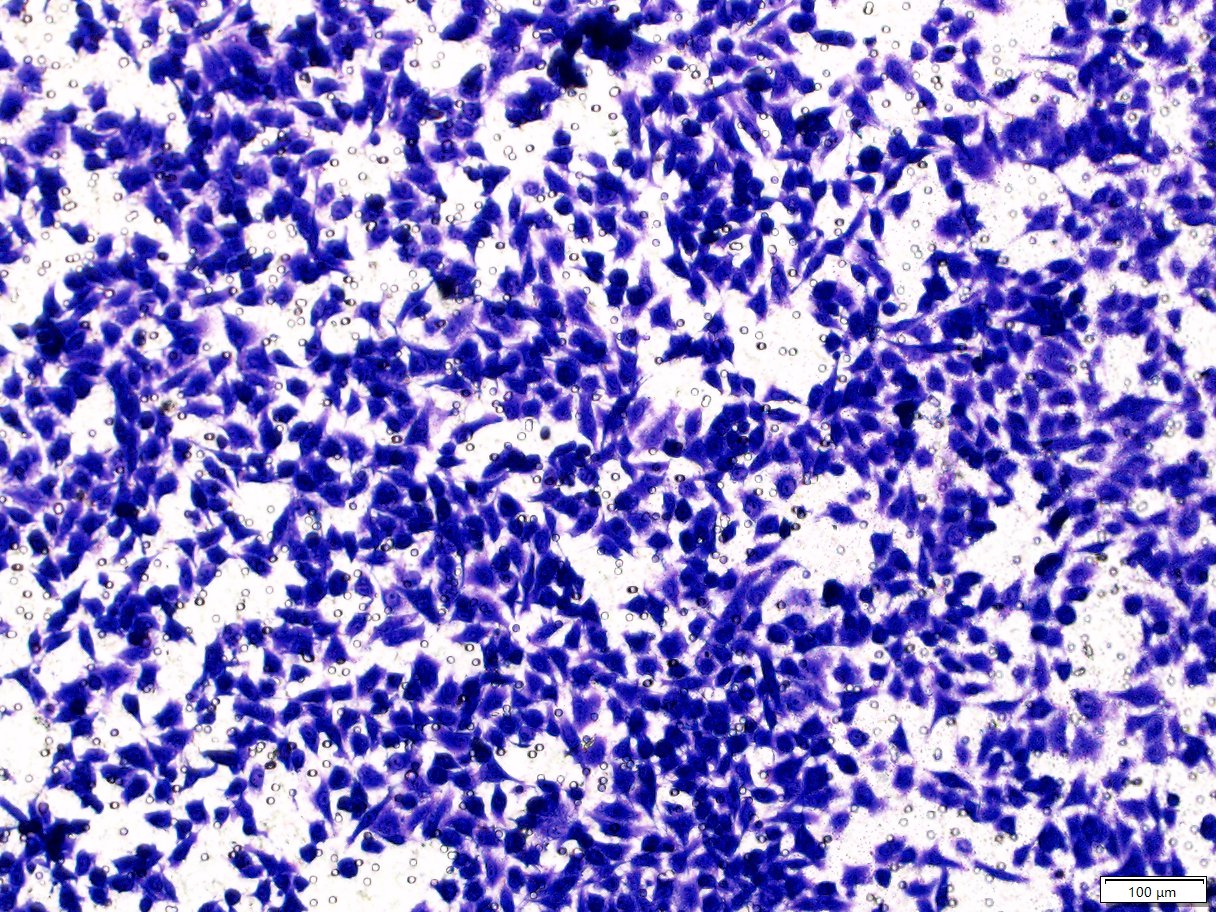

Supplement: Supplemental Information 11 [file peerj-cs-09-1651-s011.zip › Dataset 10/3-12.jpg]

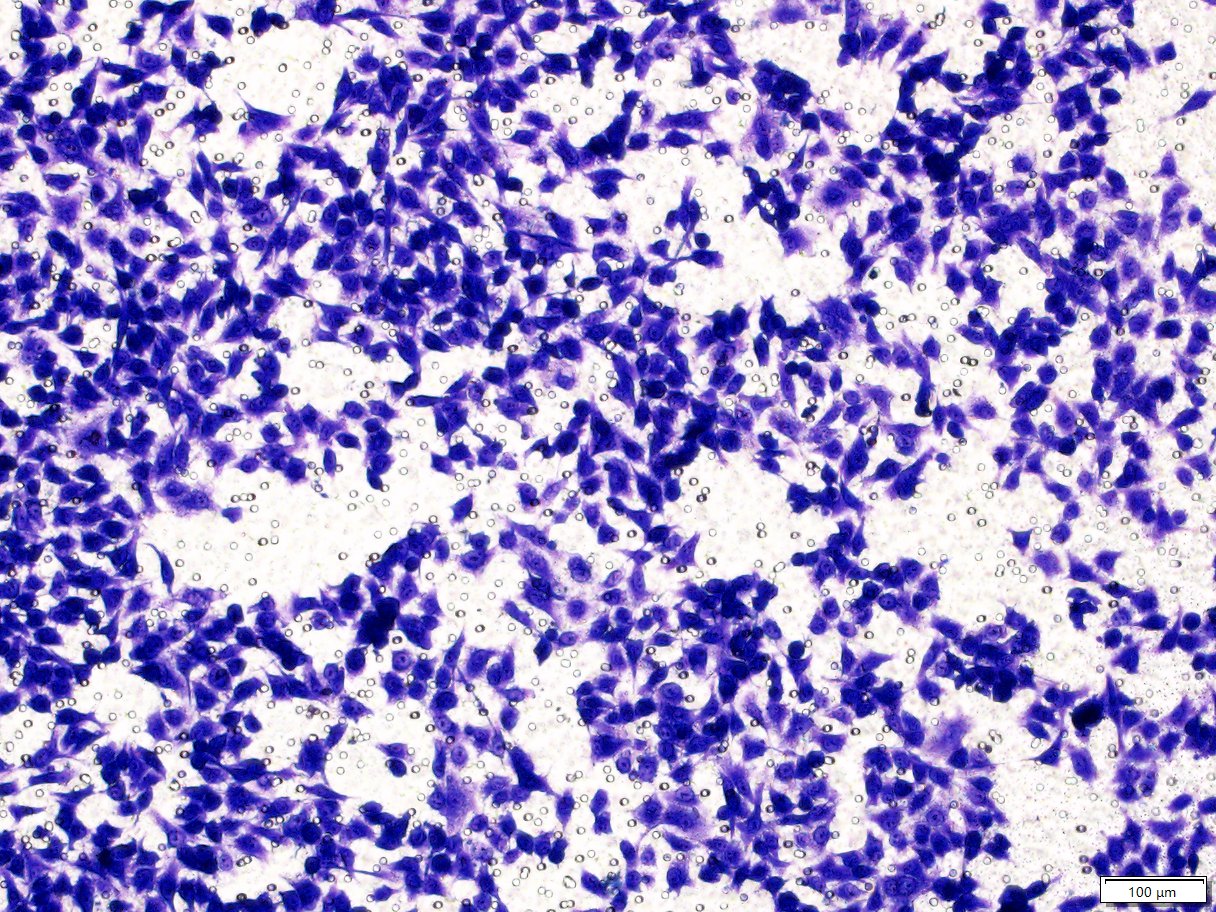

Supplement: Supplemental Information 11 [file peerj-cs-09-1651-s011.zip › Dataset 10/3-13.jpg]

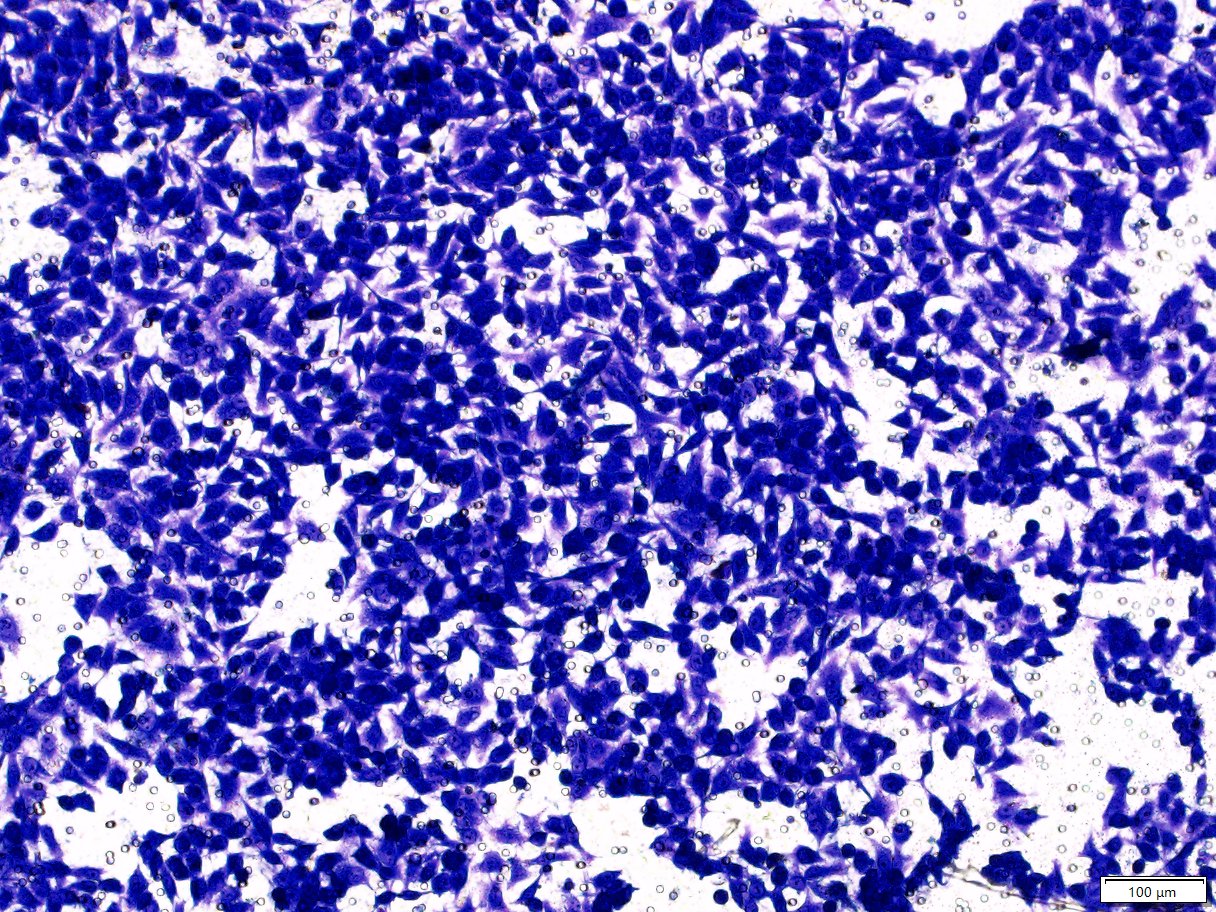

Supplement: Supplemental Information 11 [file peerj-cs-09-1651-s011.zip › Dataset 10/3-14.jpg]

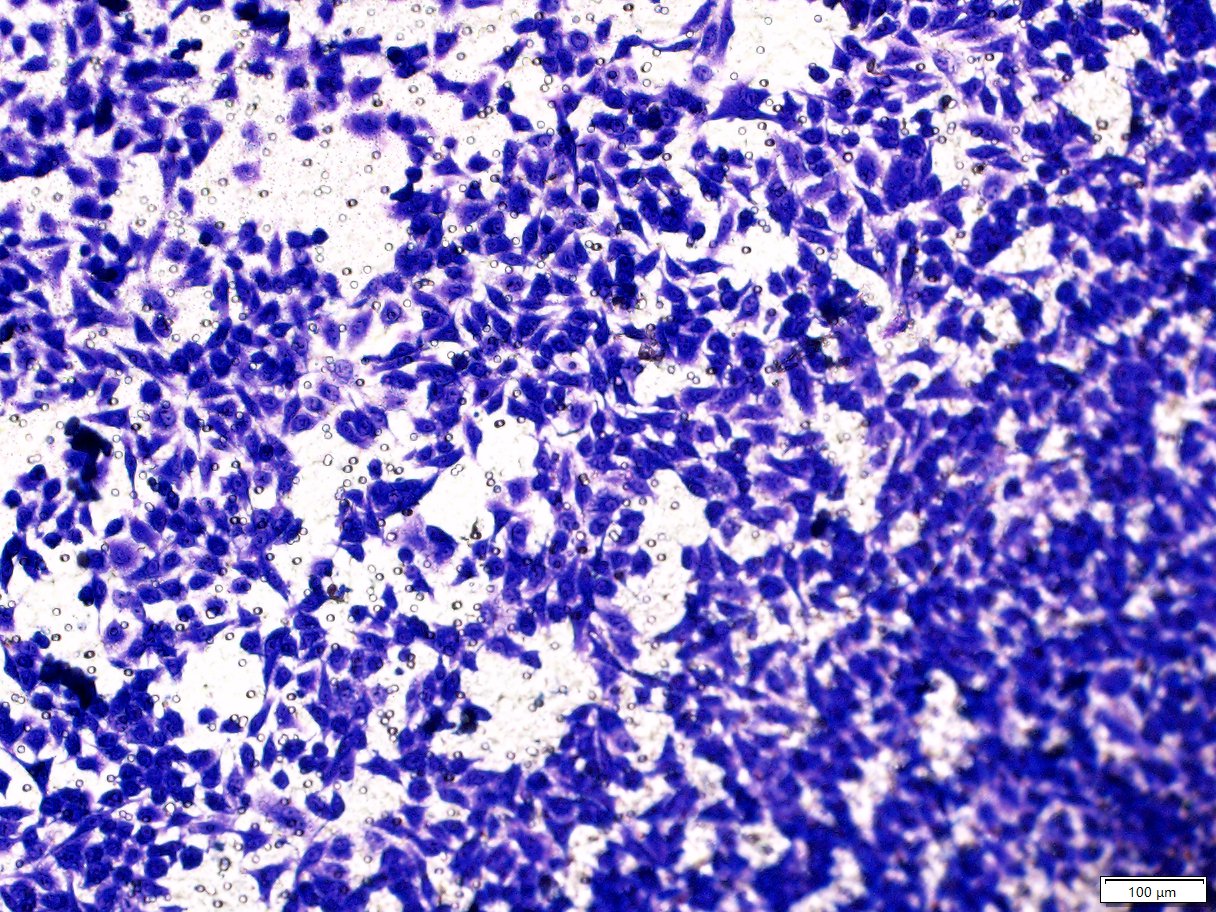

Supplement: Supplemental Information 11 [file peerj-cs-09-1651-s011.zip › Dataset 10/3-2.jpg]

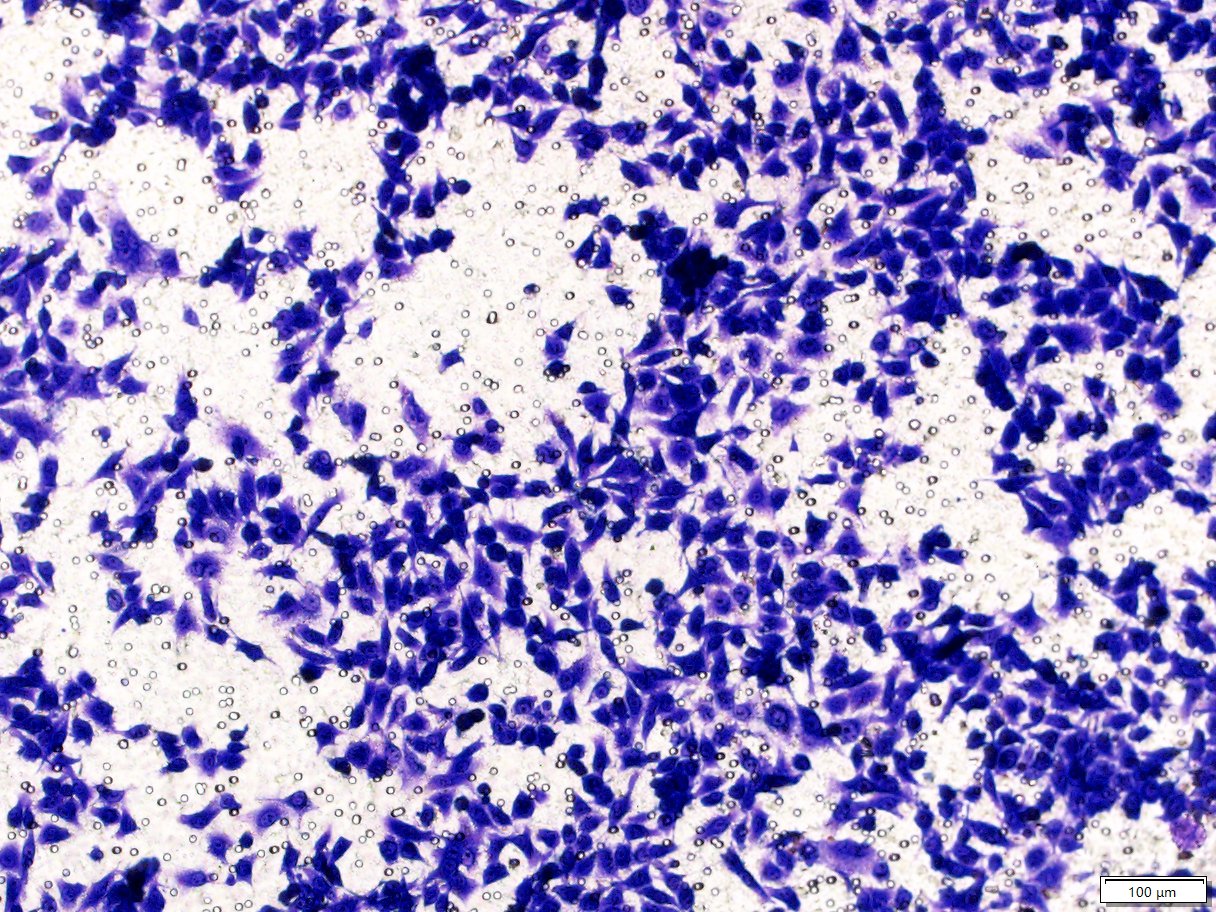

Supplement: Supplemental Information 11 [file peerj-cs-09-1651-s011.zip › Dataset 10/3-3.jpg]

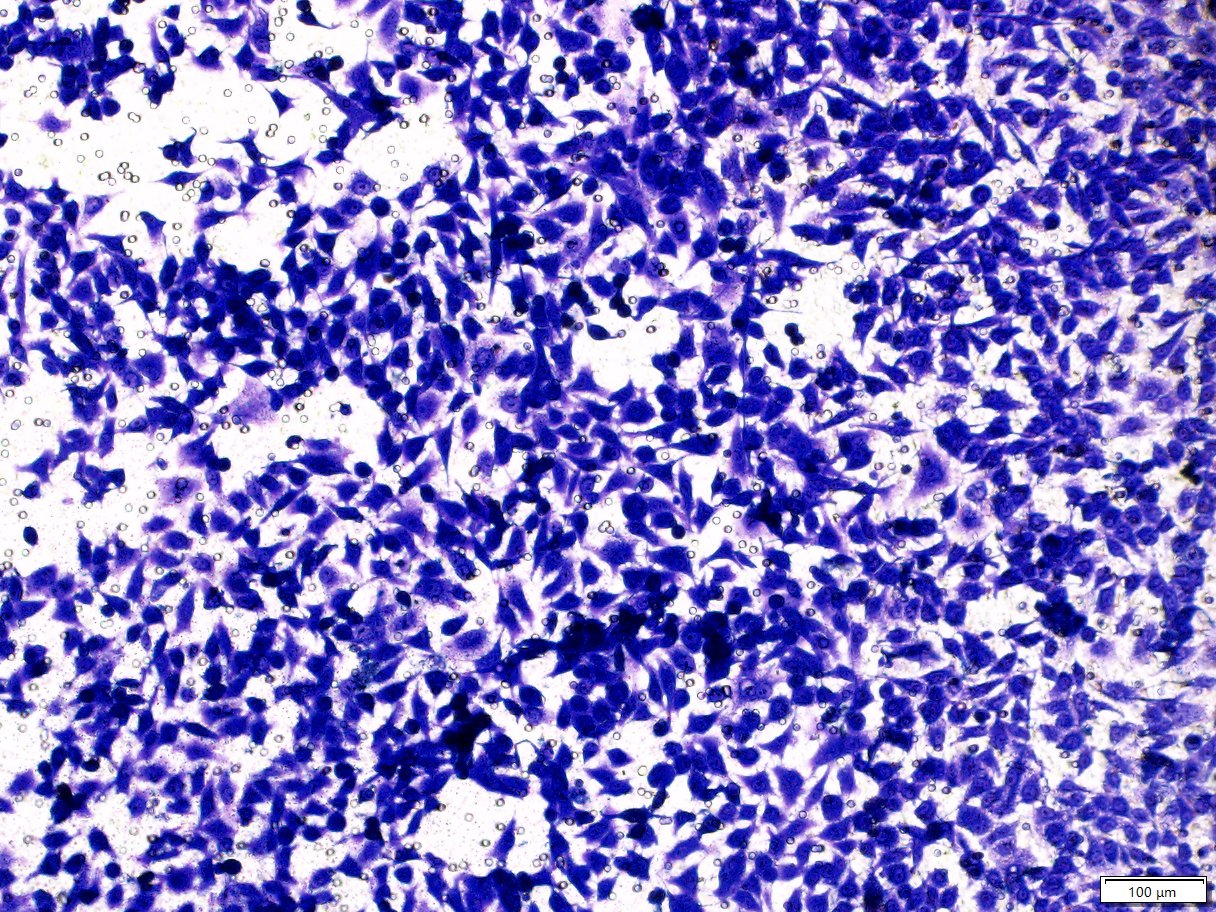

Supplement: Supplemental Information 11 [file peerj-cs-09-1651-s011.zip › Dataset 10/3-4.jpg]

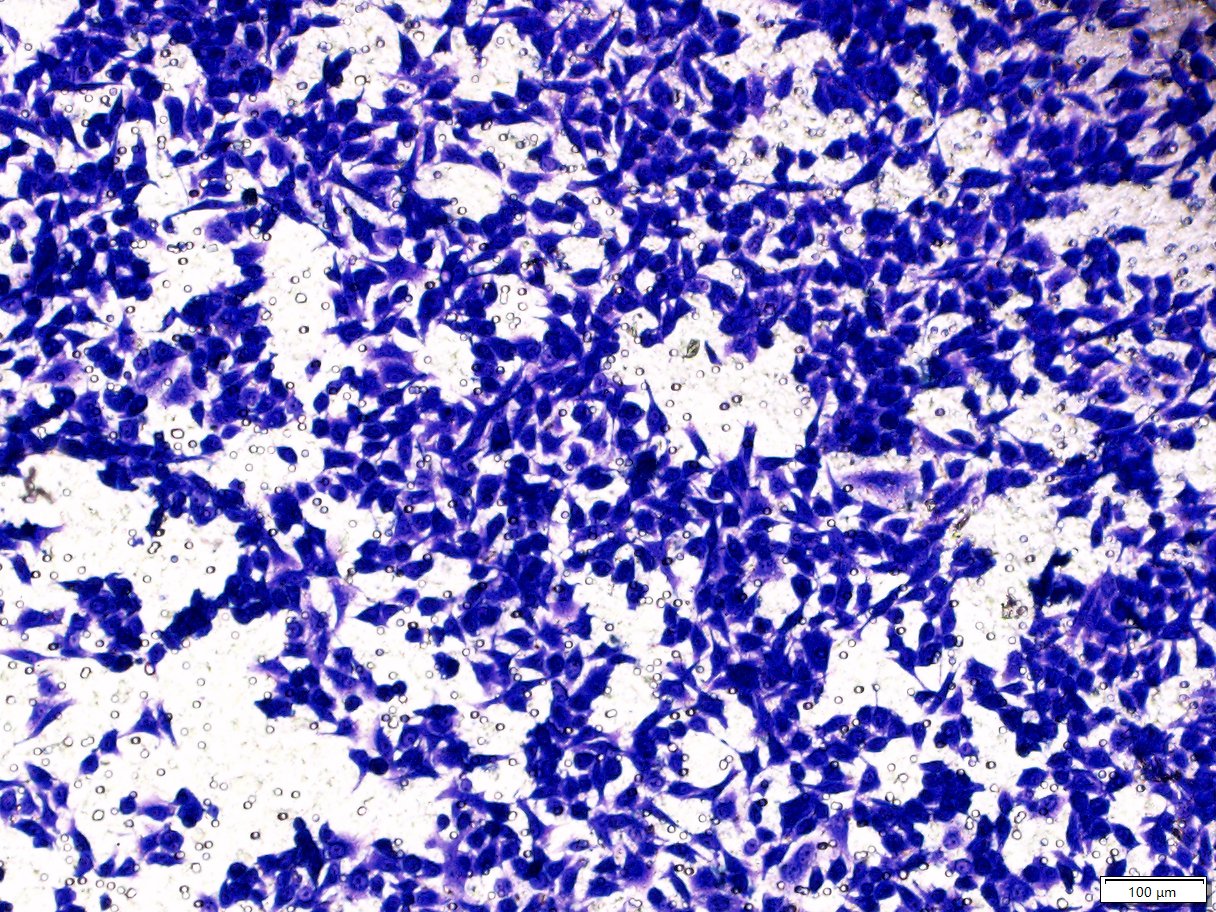

Supplement: Supplemental Information 11 [file peerj-cs-09-1651-s011.zip › Dataset 10/3-5.jpg]

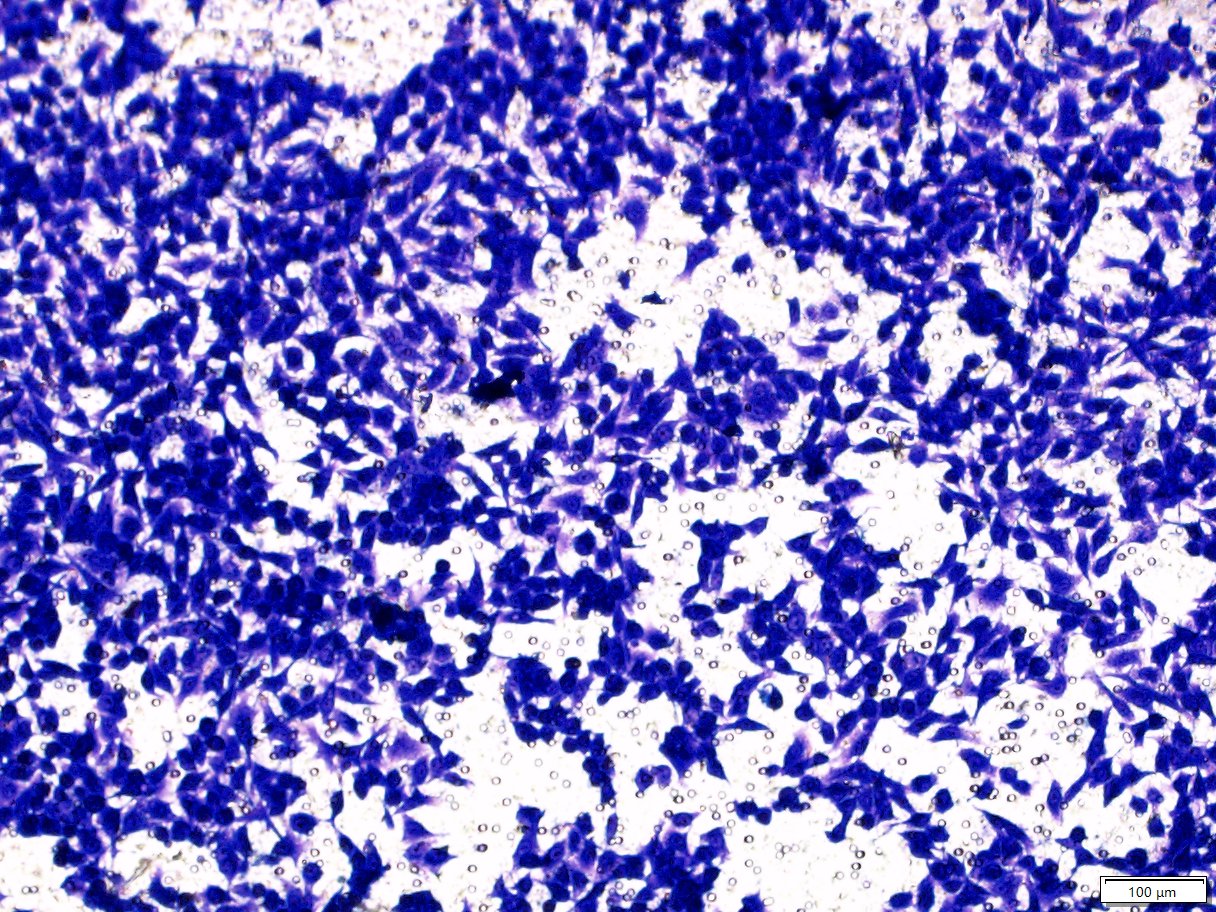

Supplement: Supplemental Information 11 [file peerj-cs-09-1651-s011.zip › Dataset 10/3-6.jpg]

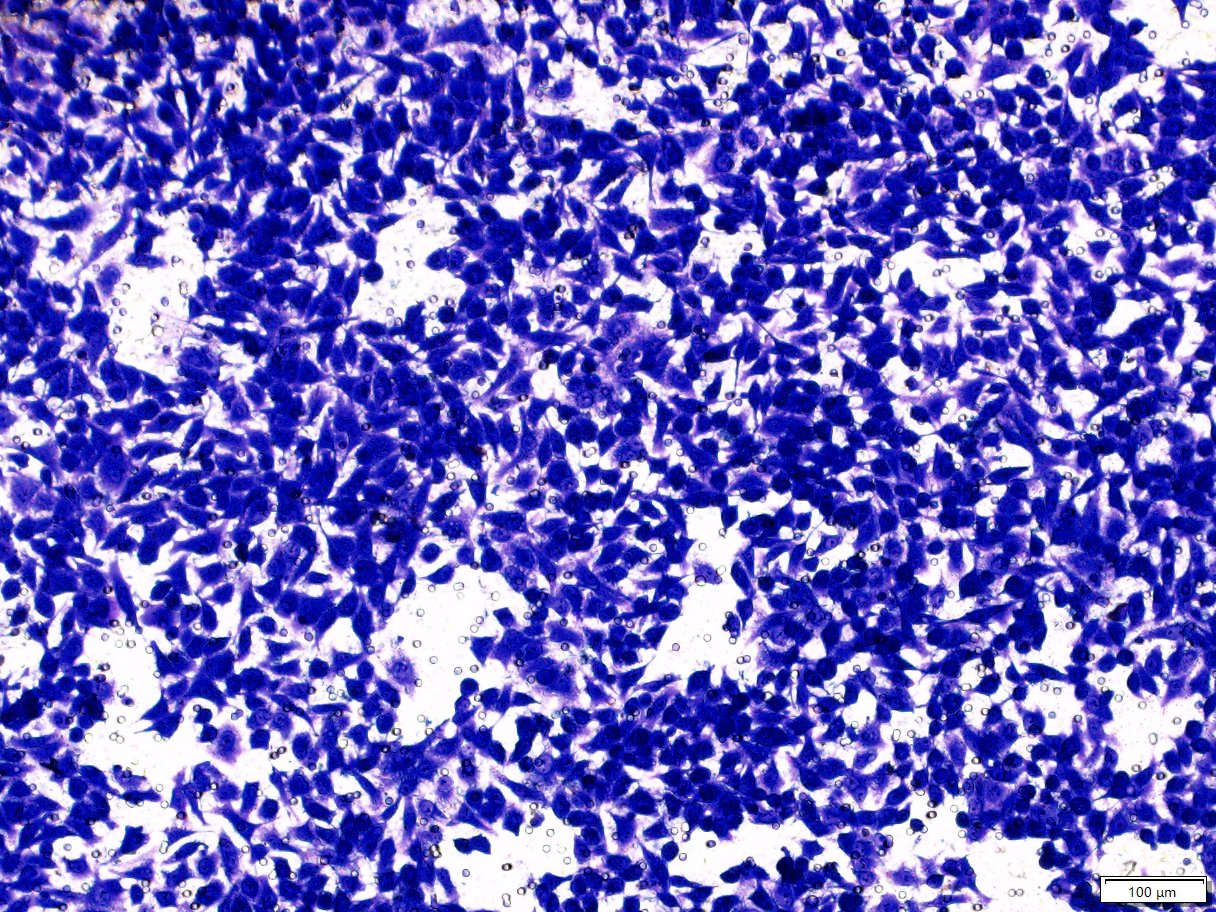

Supplement: Supplemental Information 11 [file peerj-cs-09-1651-s011.zip › Dataset 10/3-7.jpg]

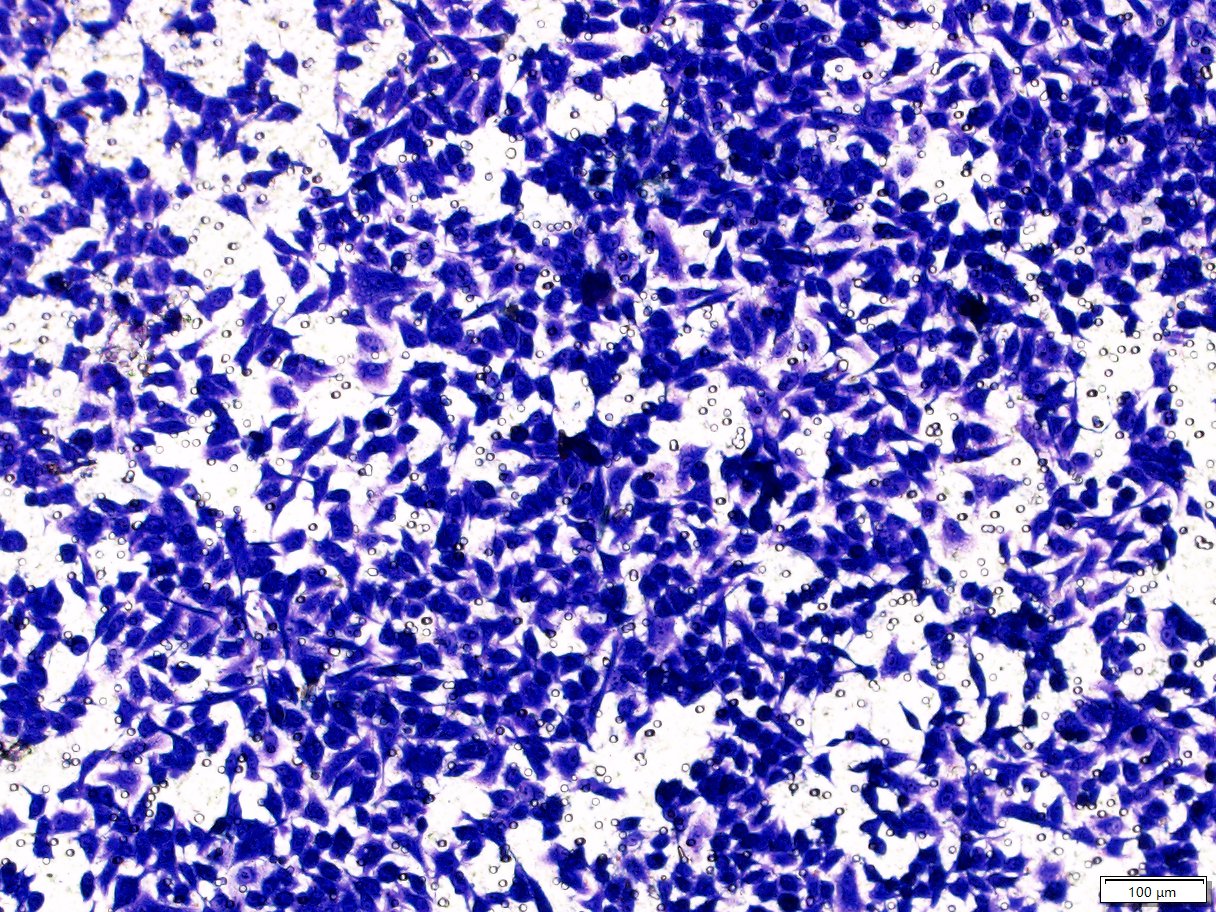

Supplement: Supplemental Information 11 [file peerj-cs-09-1651-s011.zip › Dataset 10/3-8.jpg]

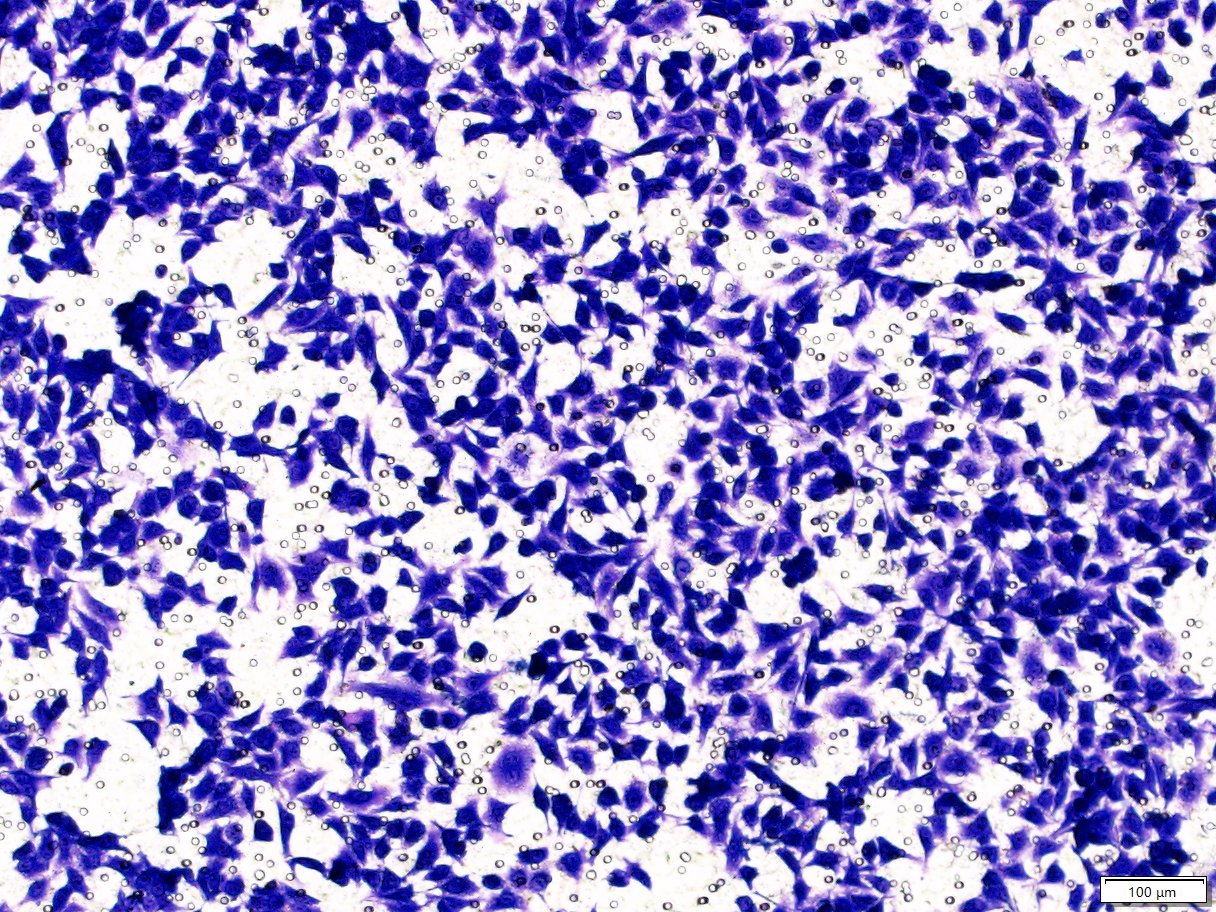

Supplement: Supplemental Information 11 [file peerj-cs-09-1651-s011.zip › Dataset 10/3-9.jpg]

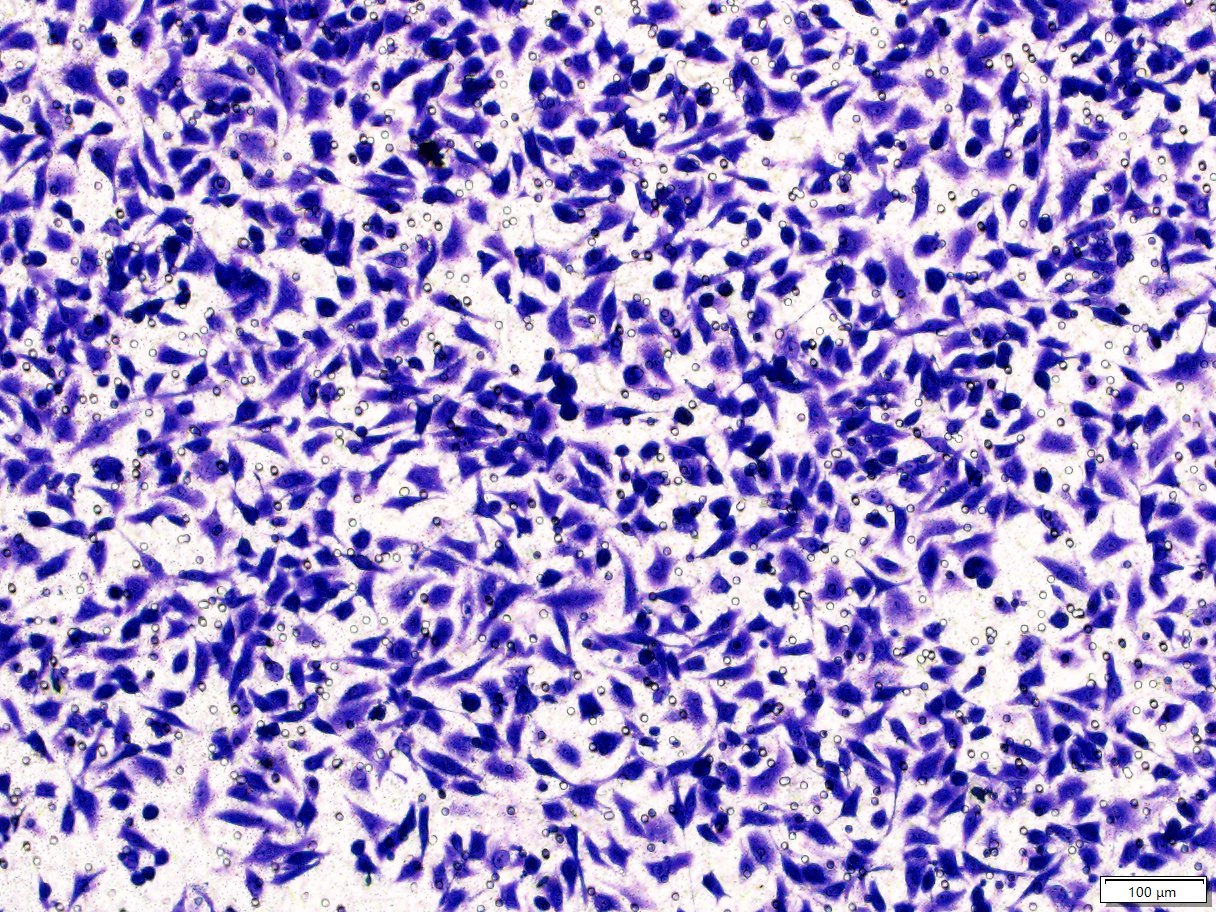

Supplement: Supplemental Information 11 [file peerj-cs-09-1651-s011.zip › Dataset 10/4+1.jpg]

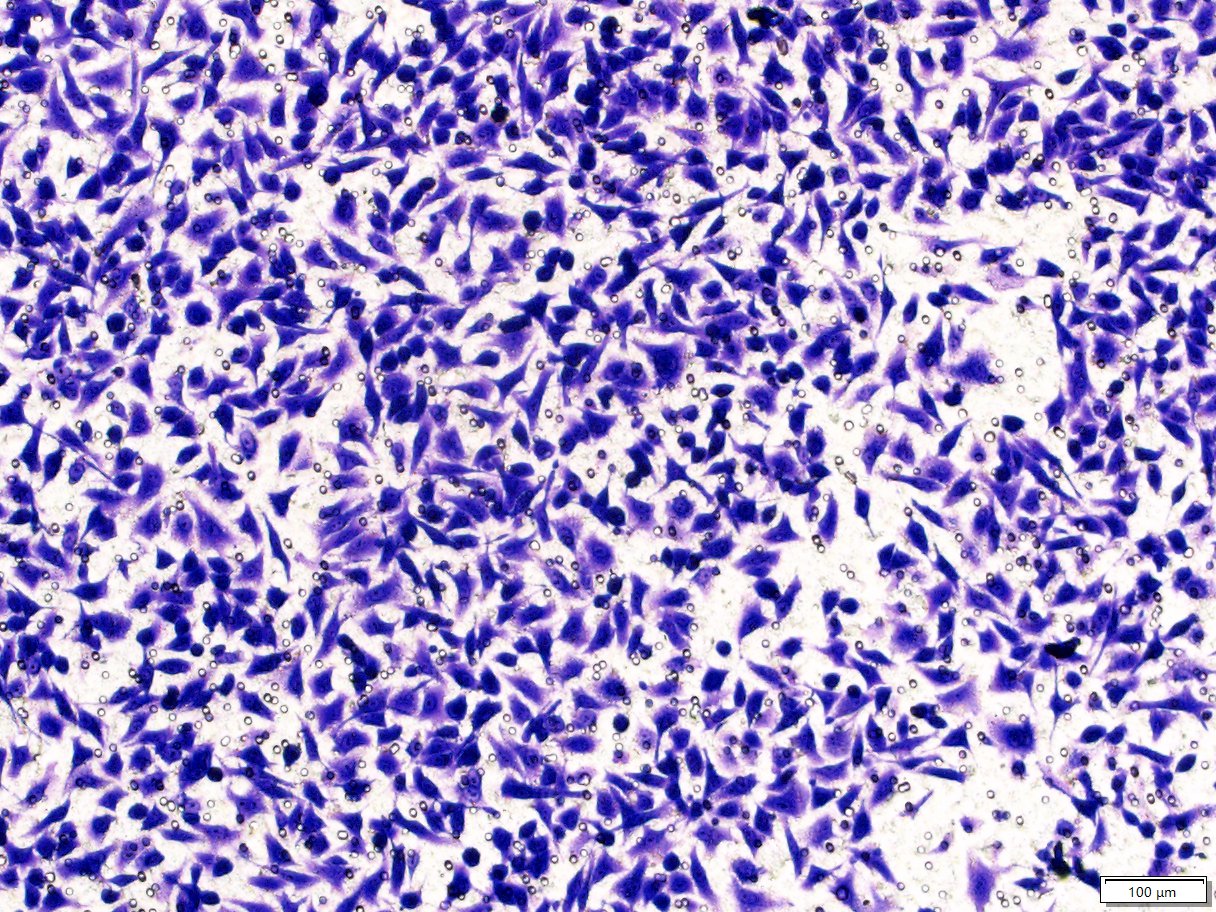

Supplement: Supplemental Information 11 [file peerj-cs-09-1651-s011.zip › Dataset 10/4+2.jpg]

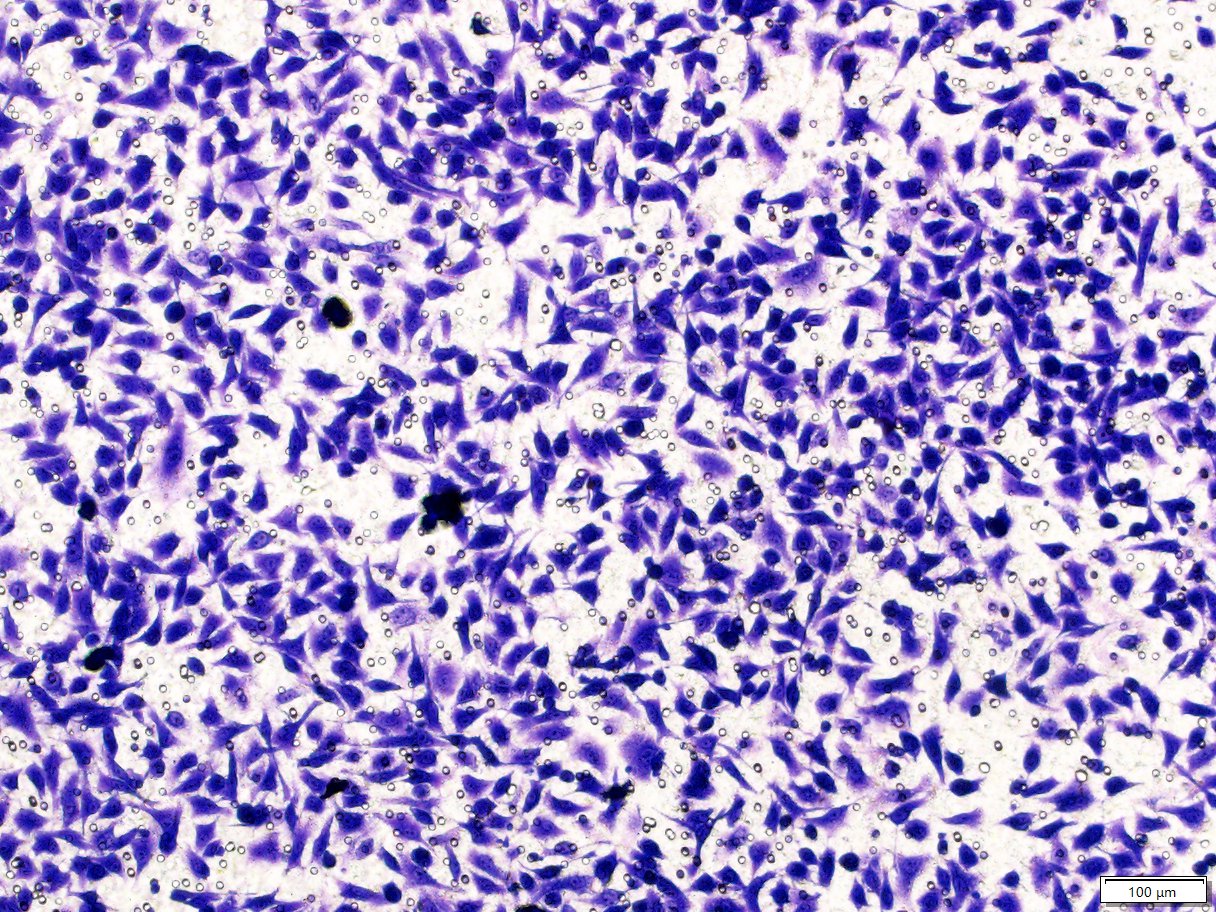

Supplement: Supplemental Information 11 [file peerj-cs-09-1651-s011.zip › Dataset 10/4+3.jpg]

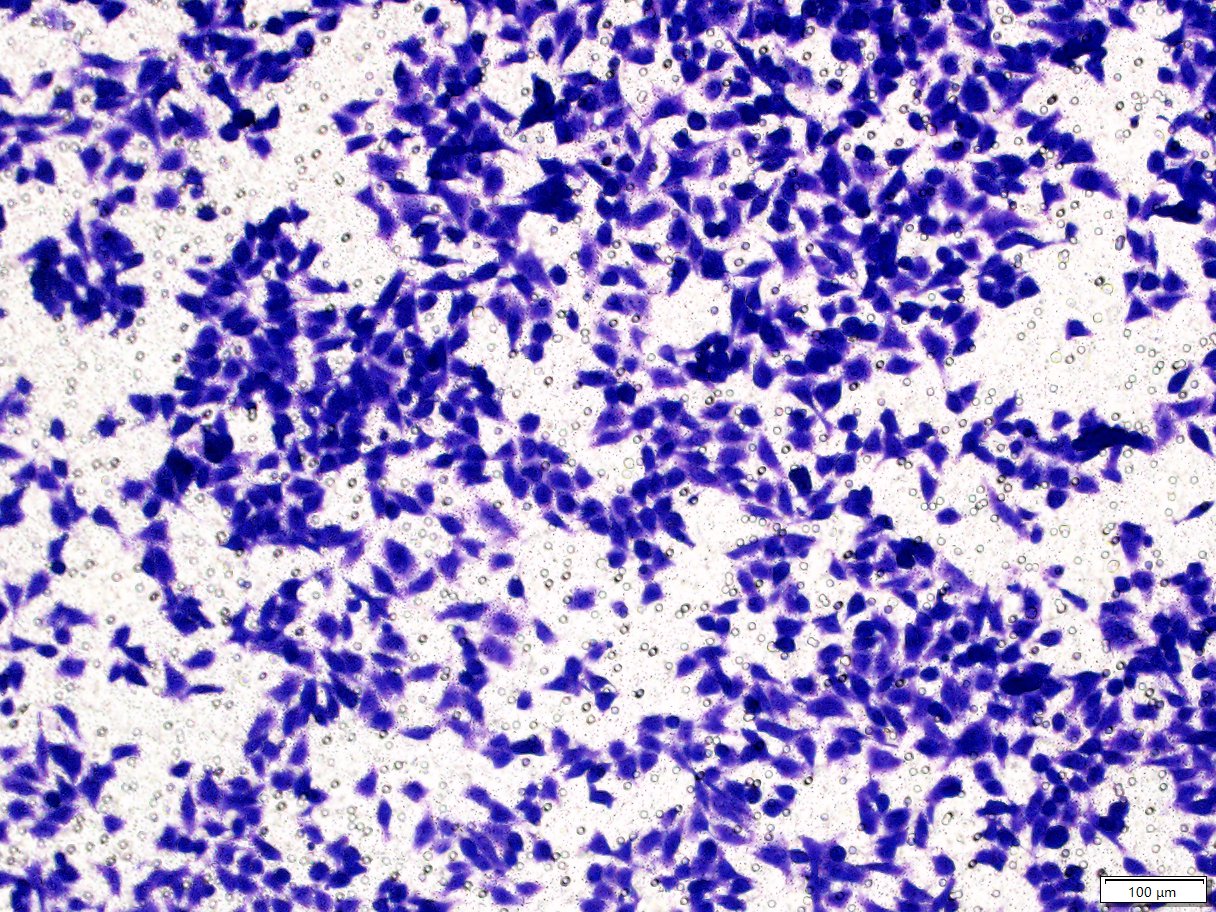

Supplement: Supplemental Information 11 [file peerj-cs-09-1651-s011.zip › Dataset 10/5-15.jpg]

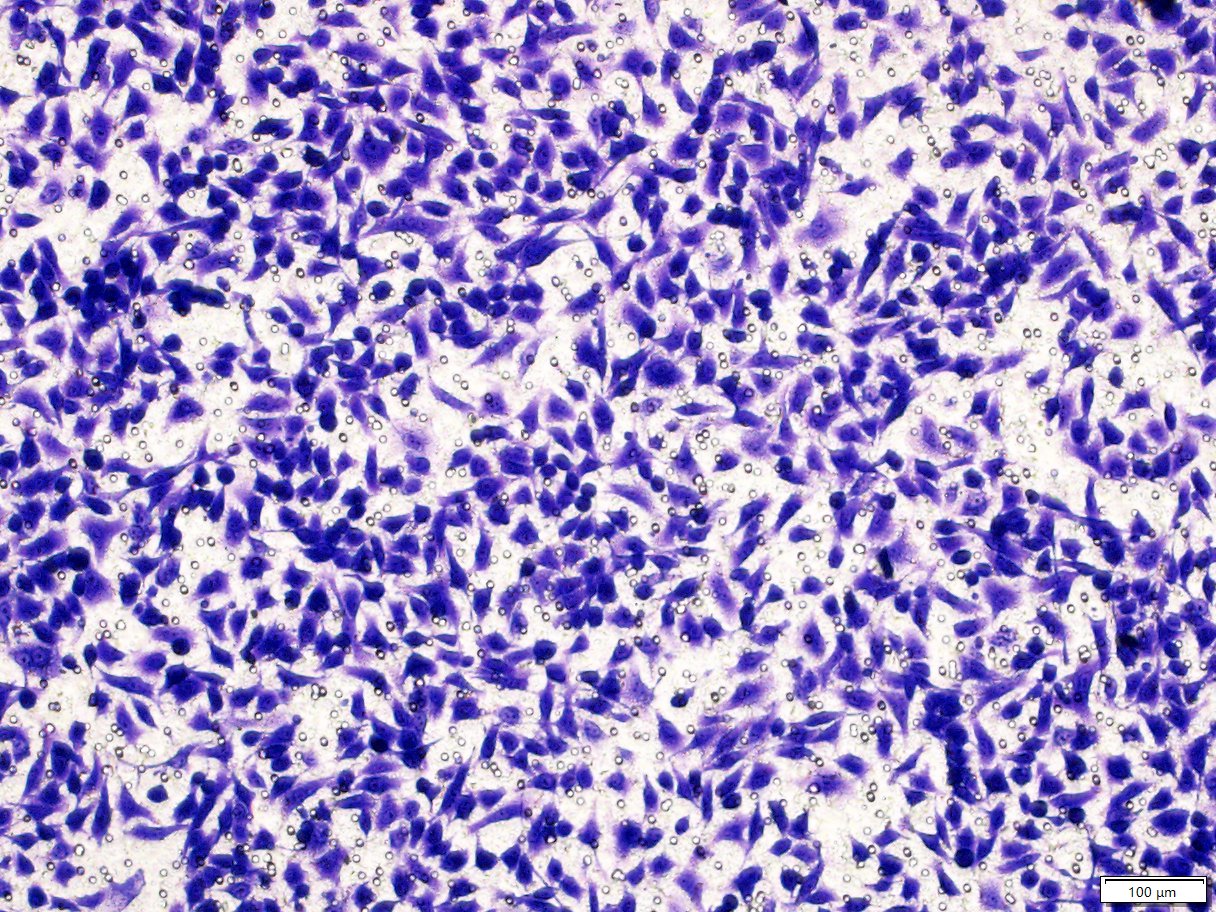

Supplement: Supplemental Information 12 [file peerj-cs-09-1651-s012.zip › Dataset 11/4+10.jpg]

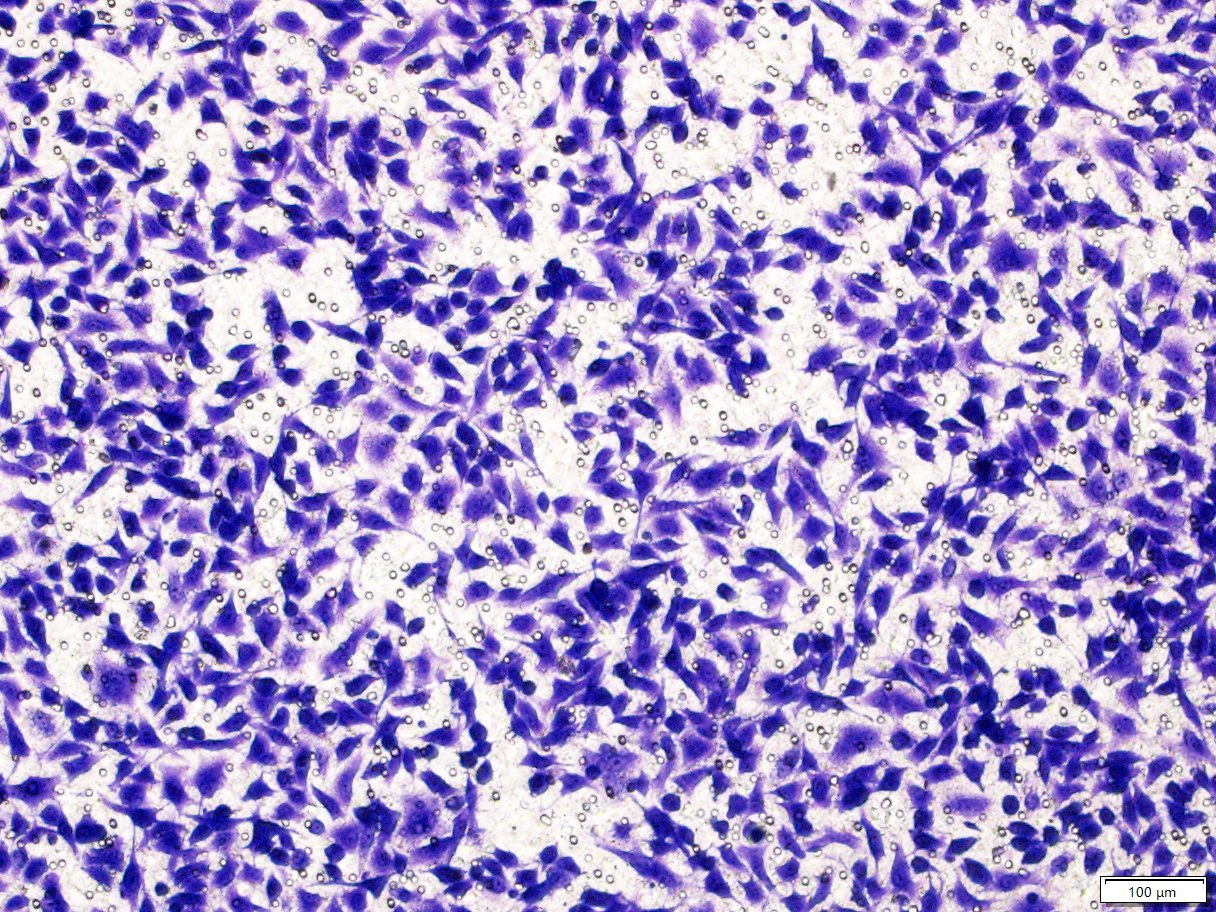

Supplement: Supplemental Information 12 [file peerj-cs-09-1651-s012.zip › Dataset 11/4+11.jpg]

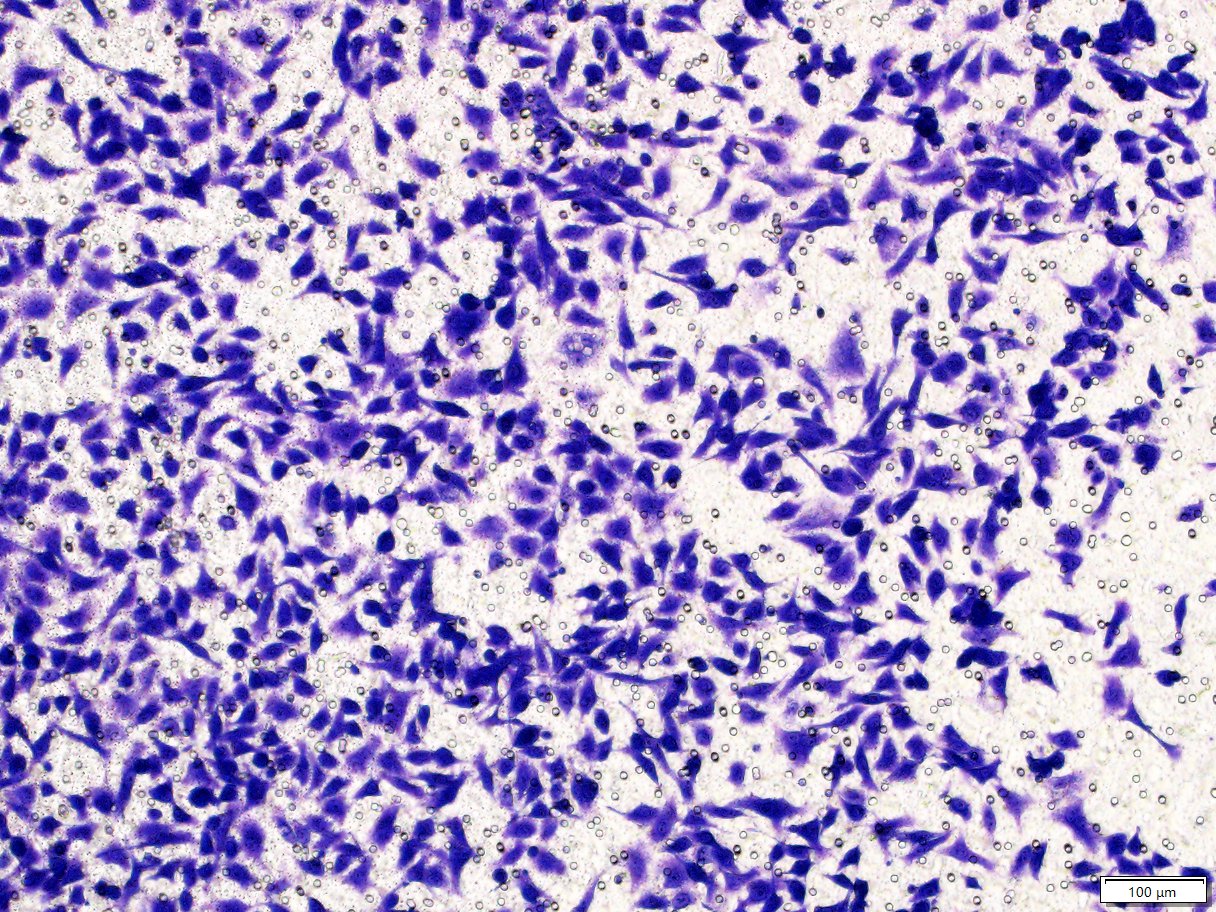

Supplement: Supplemental Information 12 [file peerj-cs-09-1651-s012.zip › Dataset 11/4+4.jpg]

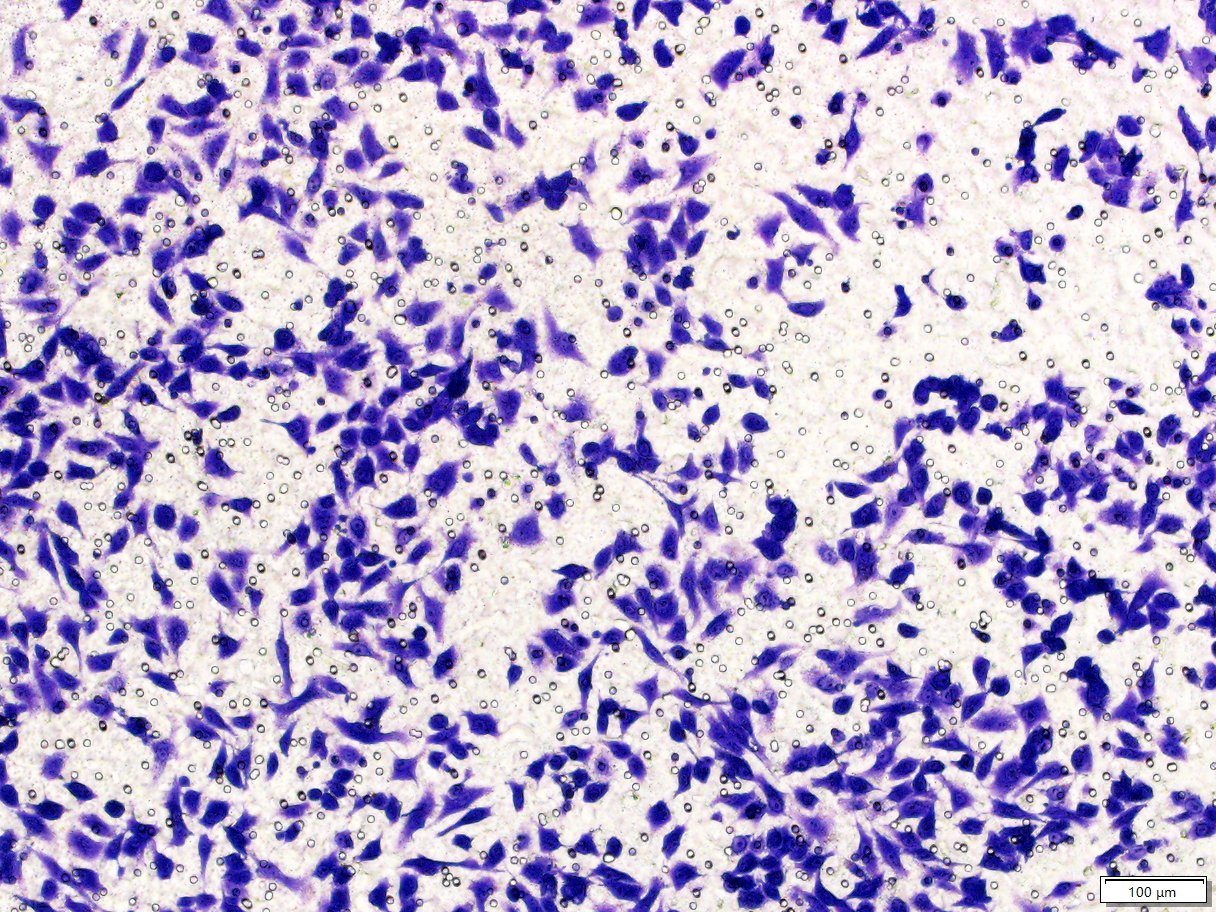

Supplement: Supplemental Information 12 [file peerj-cs-09-1651-s012.zip › Dataset 11/4+5.jpg]

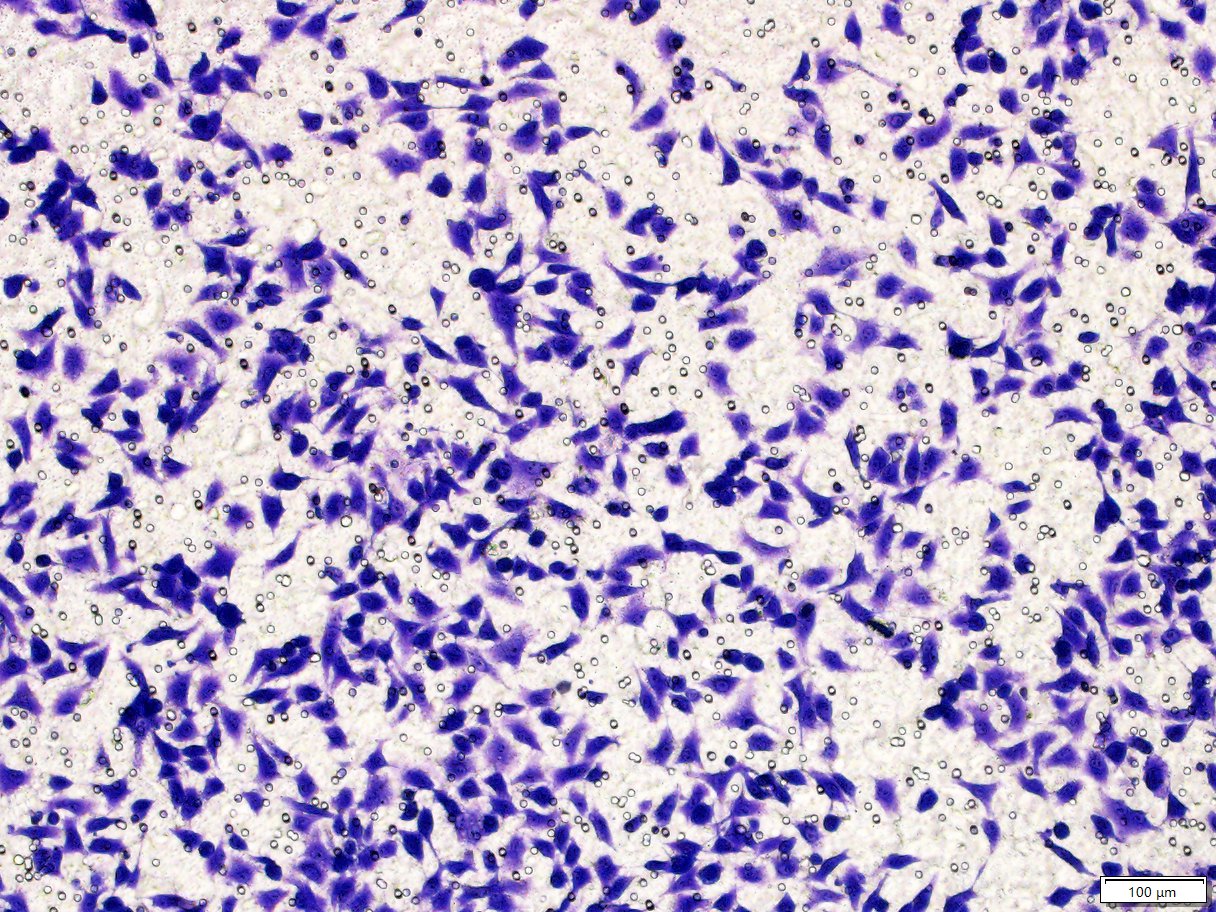

Supplement: Supplemental Information 12 [file peerj-cs-09-1651-s012.zip › Dataset 11/4+6.jpg]

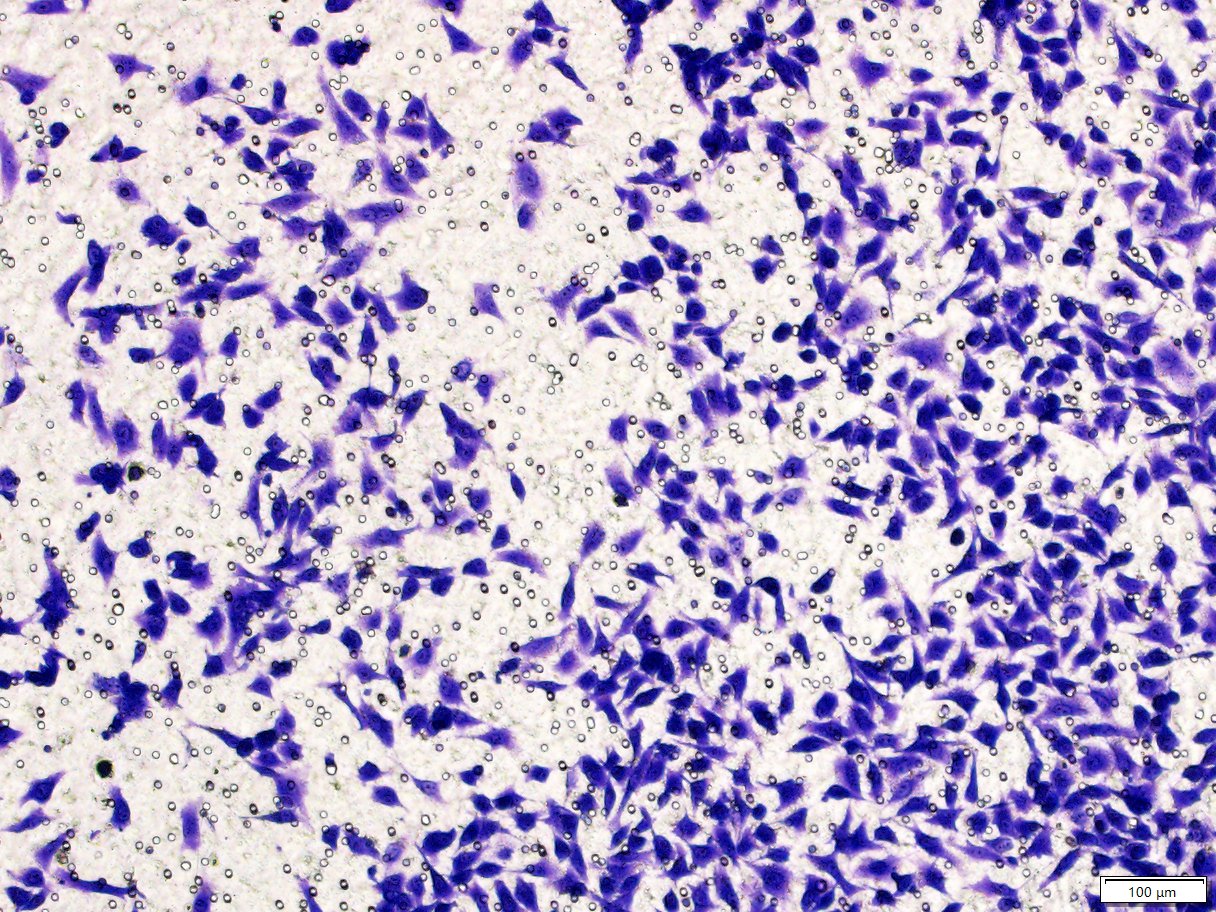

Supplement: Supplemental Information 12 [file peerj-cs-09-1651-s012.zip › Dataset 11/4+7.jpg]

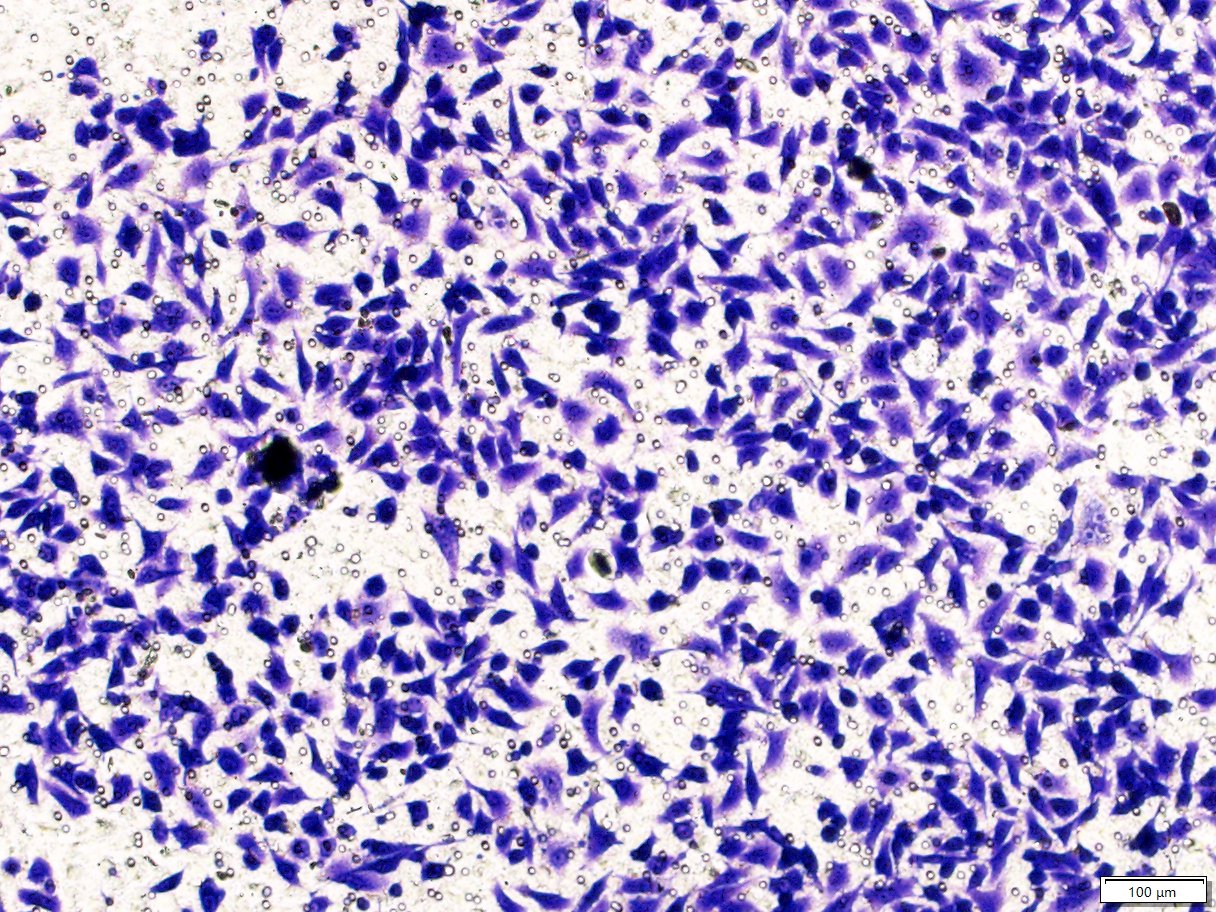

Supplement: Supplemental Information 12 [file peerj-cs-09-1651-s012.zip › Dataset 11/4+8.jpg]

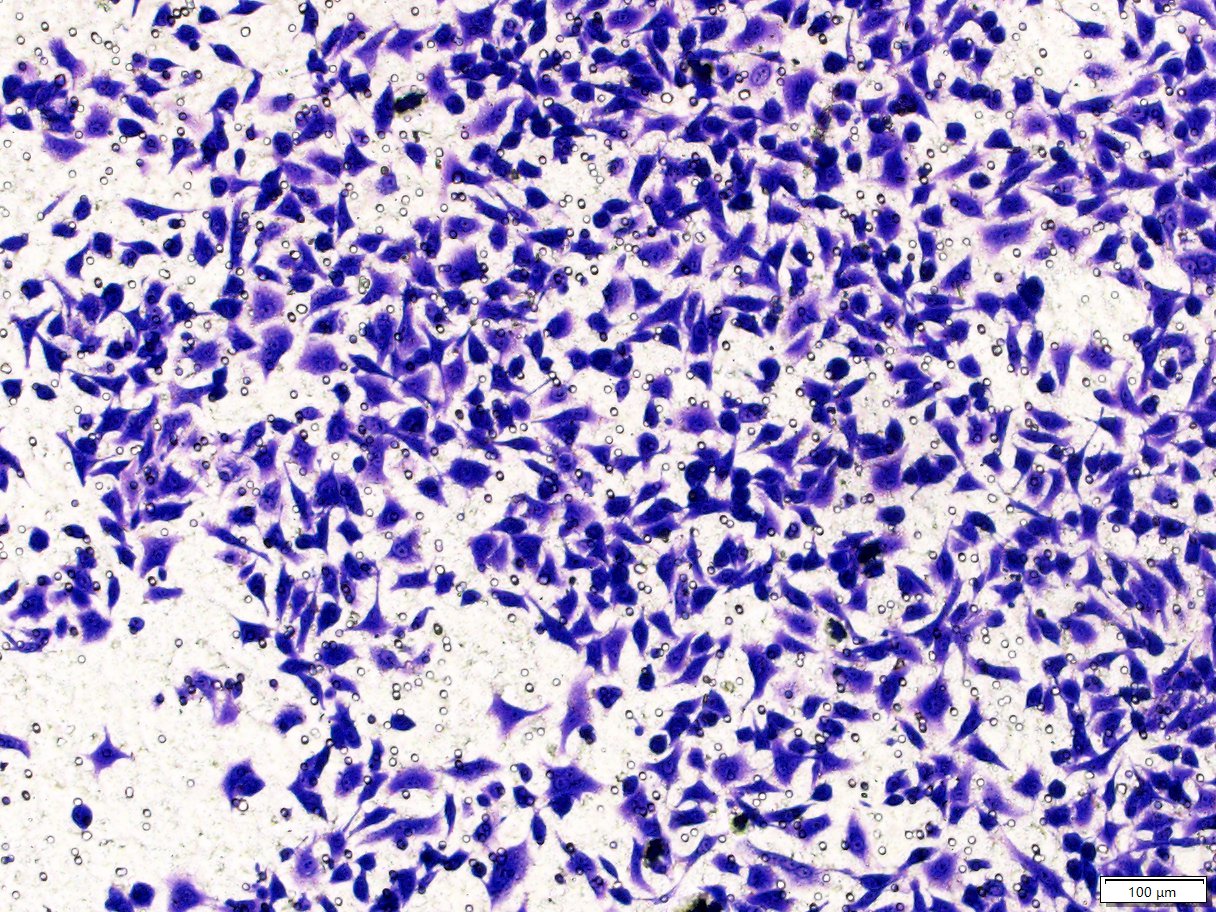

Supplement: Supplemental Information 12 [file peerj-cs-09-1651-s012.zip › Dataset 11/4+9.jpg]

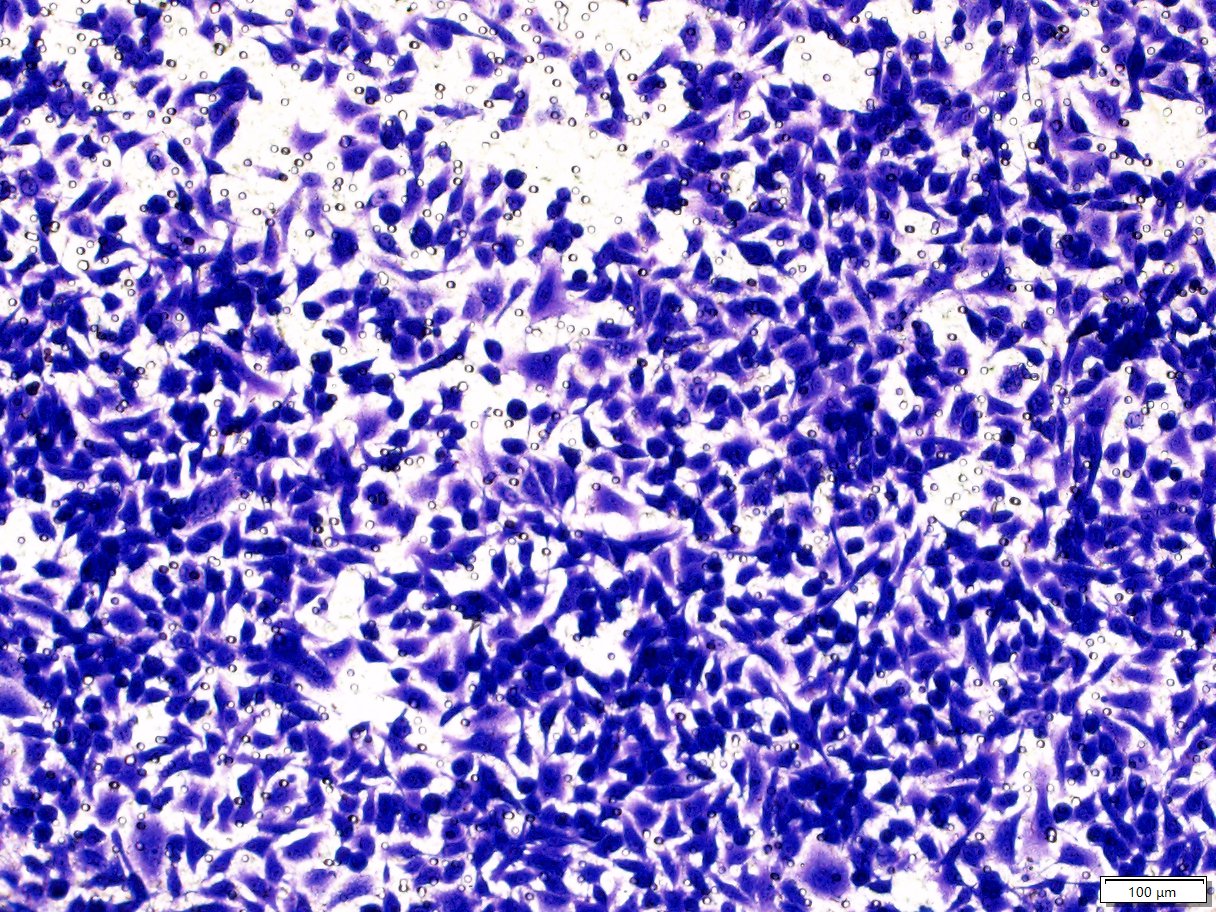

Supplement: Supplemental Information 12 [file peerj-cs-09-1651-s012.zip › Dataset 11/4-1.jpg]

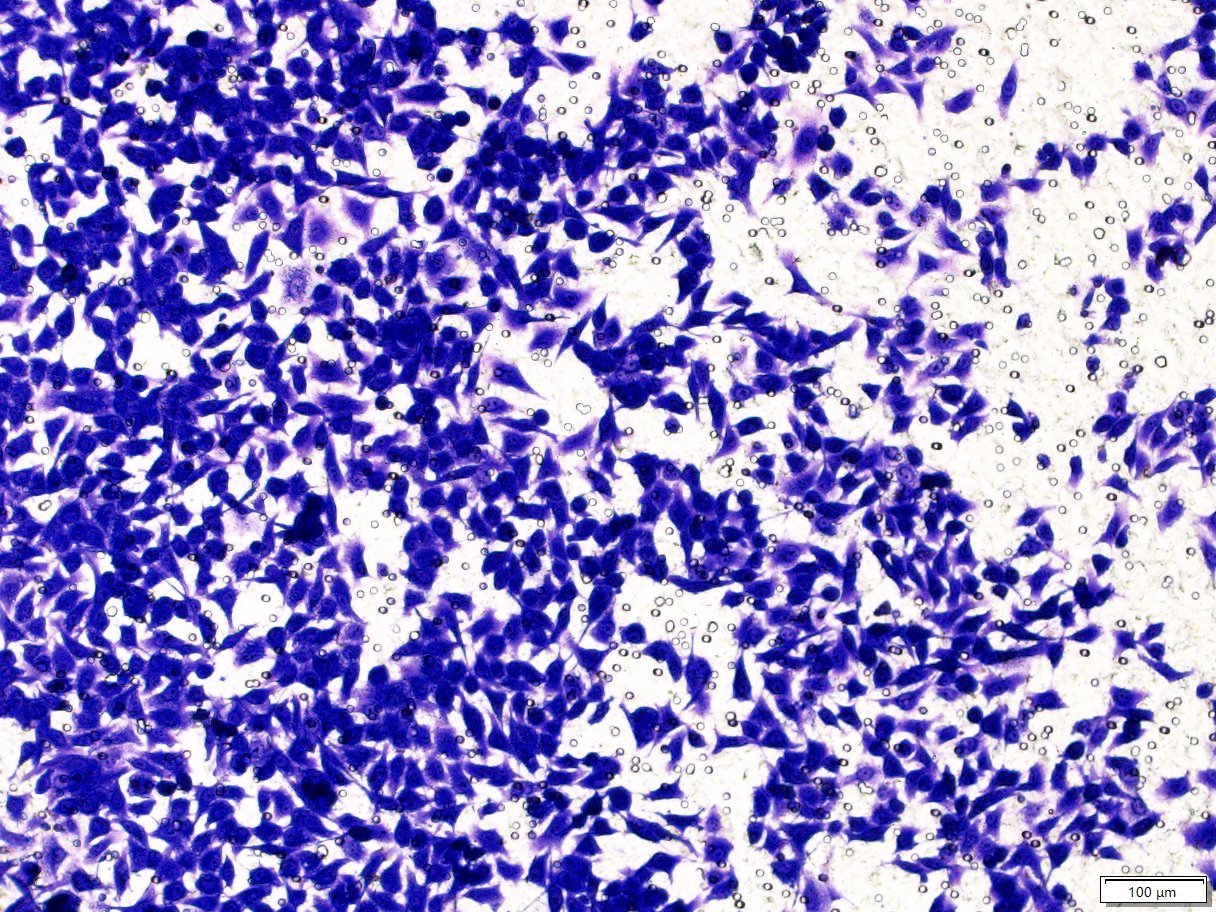

Supplement: Supplemental Information 12 [file peerj-cs-09-1651-s012.zip › Dataset 11/4-10.jpg]

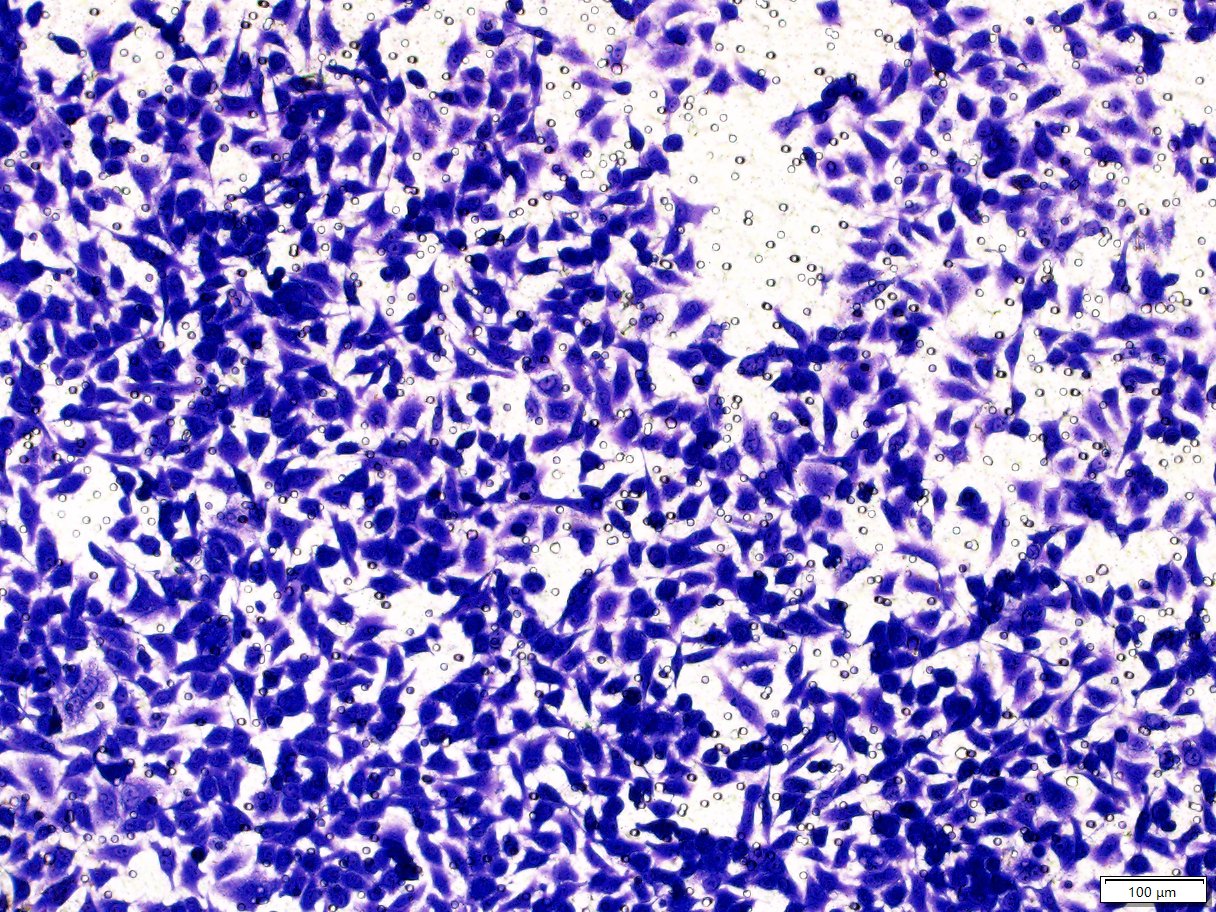

Supplement: Supplemental Information 12 [file peerj-cs-09-1651-s012.zip › Dataset 11/4-11.jpg]

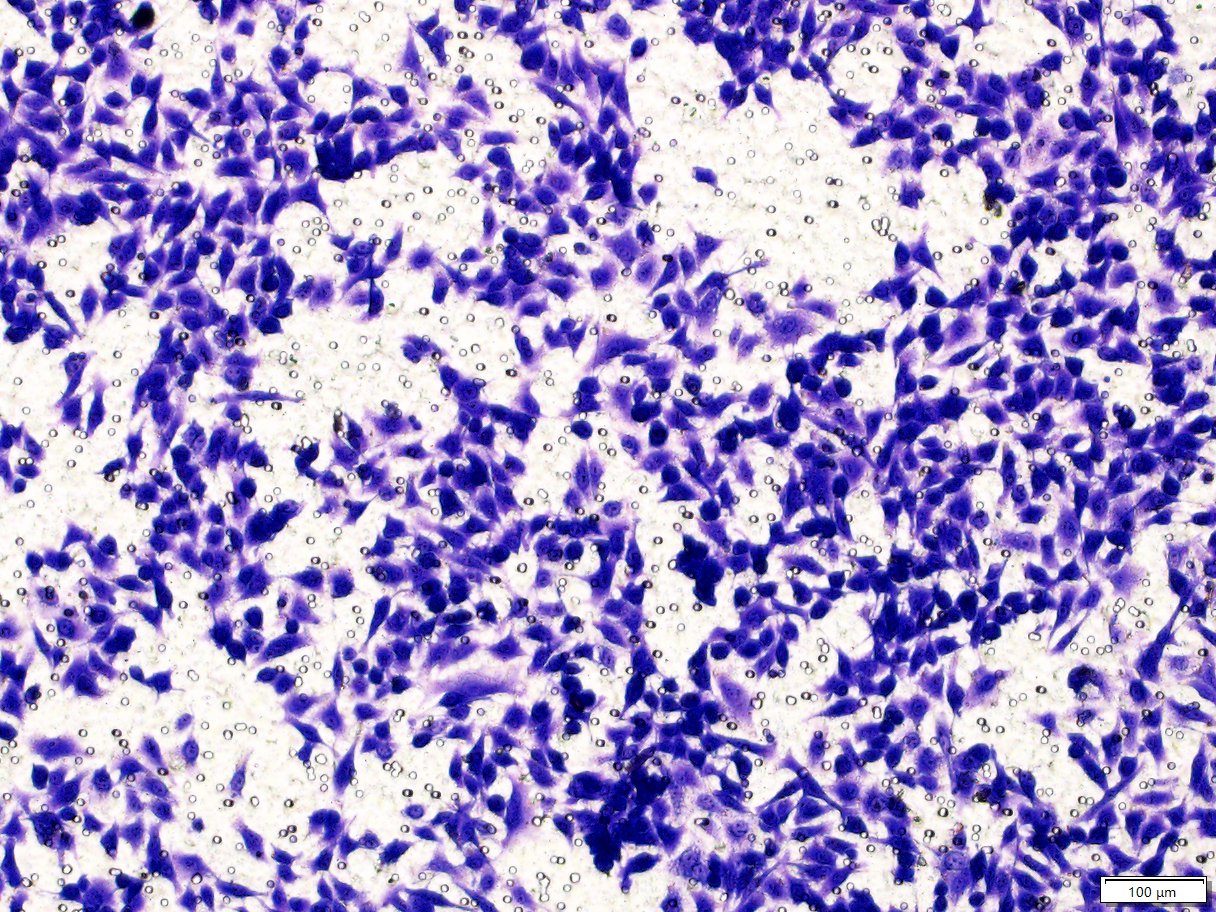

Supplement: Supplemental Information 12 [file peerj-cs-09-1651-s012.zip › Dataset 11/4-12.jpg]

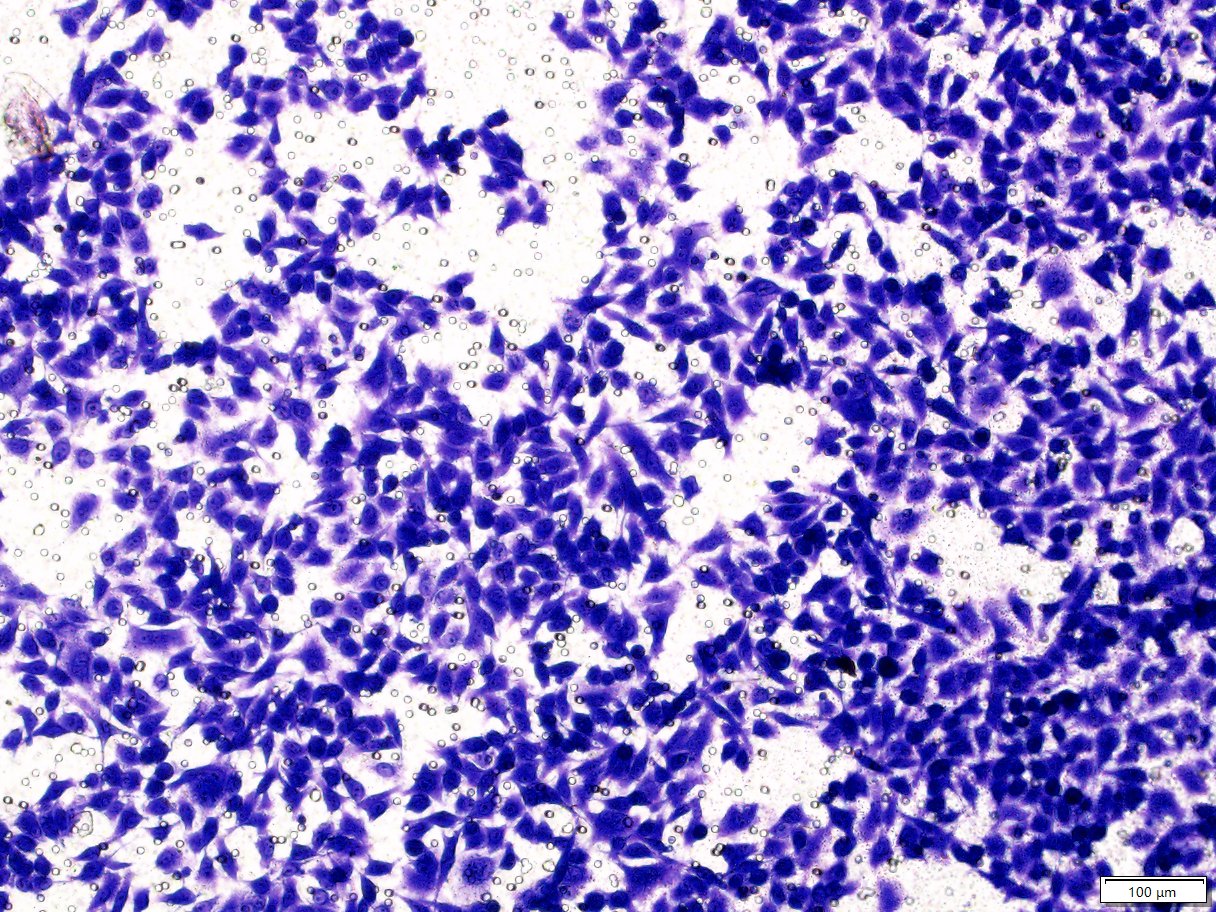

Supplement: Supplemental Information 12 [file peerj-cs-09-1651-s012.zip › Dataset 11/4-13.jpg]

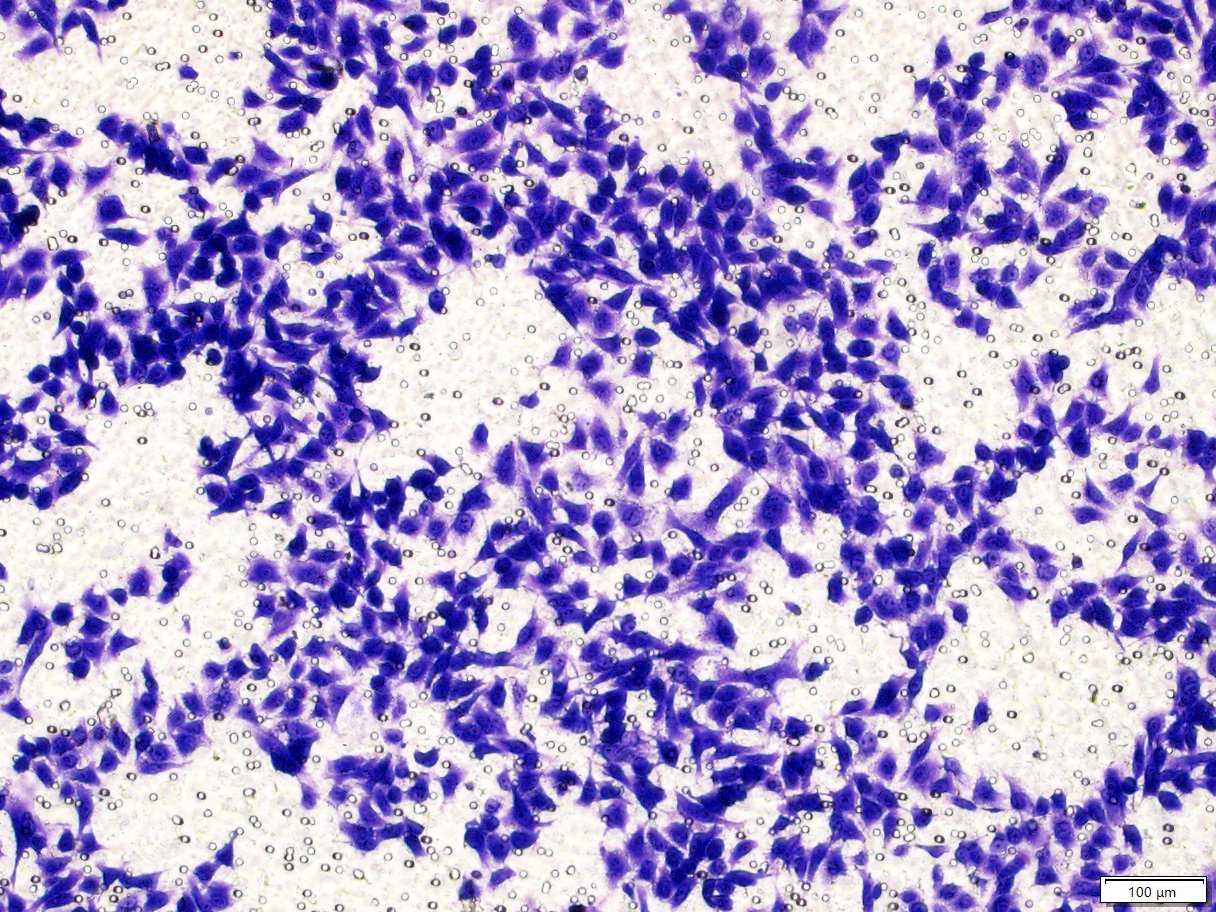

Supplement: Supplemental Information 12 [file peerj-cs-09-1651-s012.zip › Dataset 11/4-14.jpg]

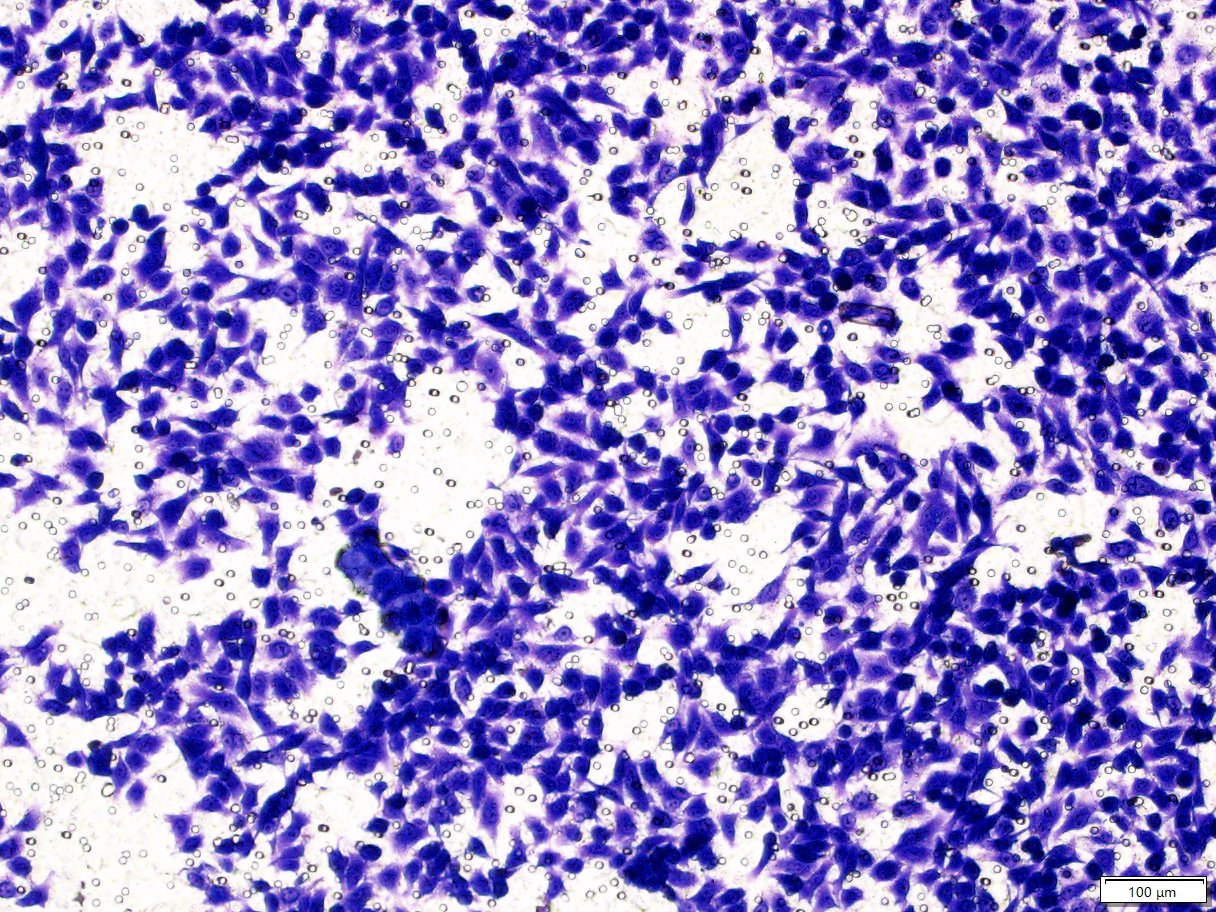

Supplement: Supplemental Information 12 [file peerj-cs-09-1651-s012.zip › Dataset 11/4-15.jpg]

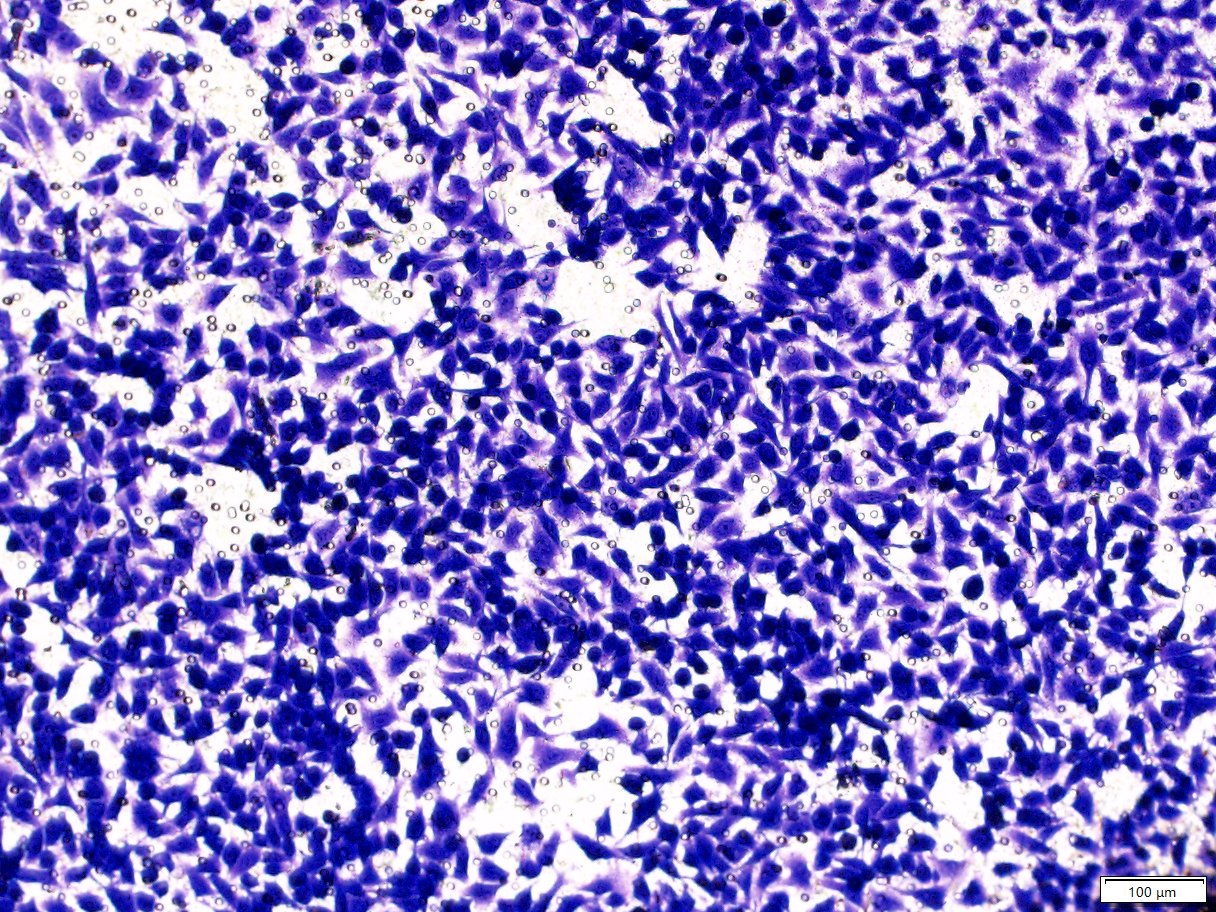

Supplement: Supplemental Information 12 [file peerj-cs-09-1651-s012.zip › Dataset 11/4-2.jpg]

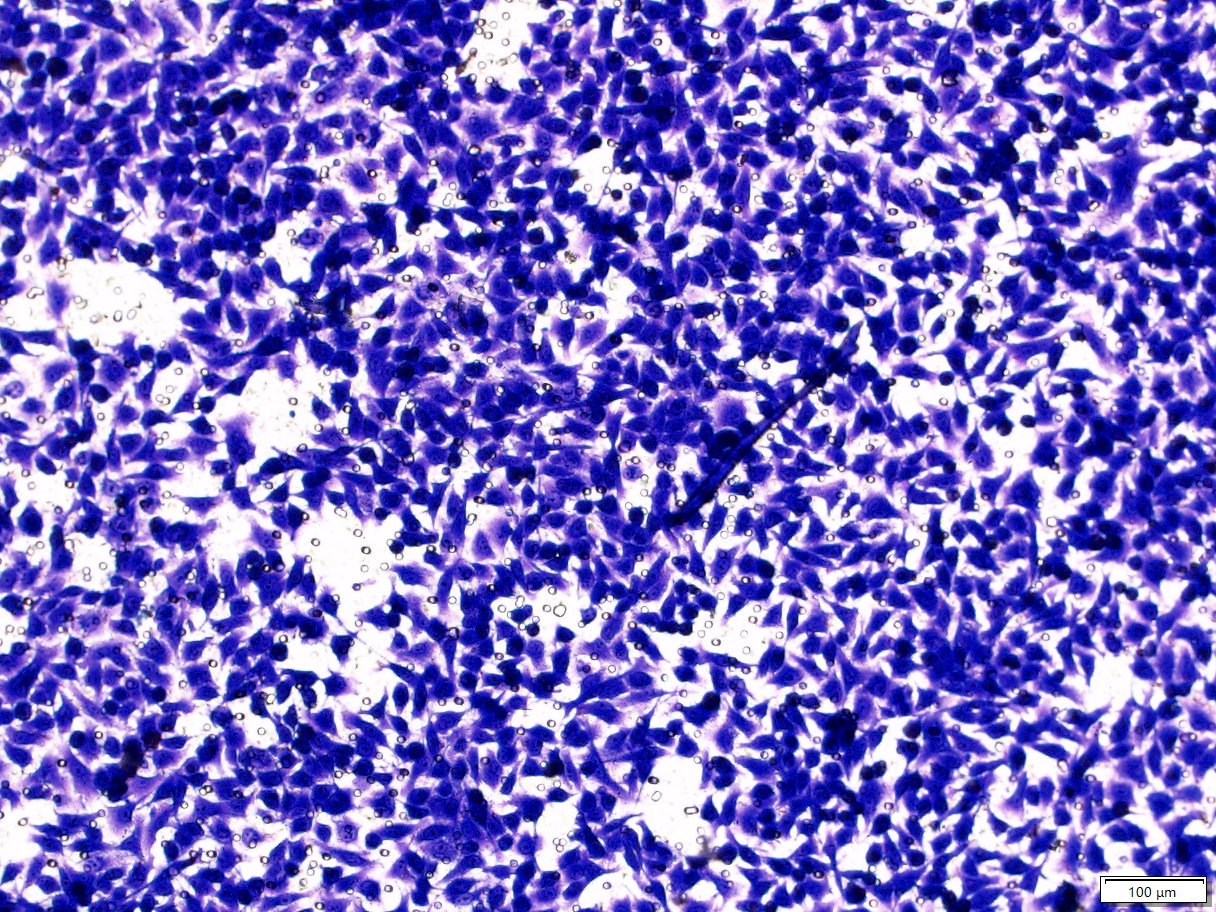

Supplement: Supplemental Information 12 [file peerj-cs-09-1651-s012.zip › Dataset 11/4-3.jpg]

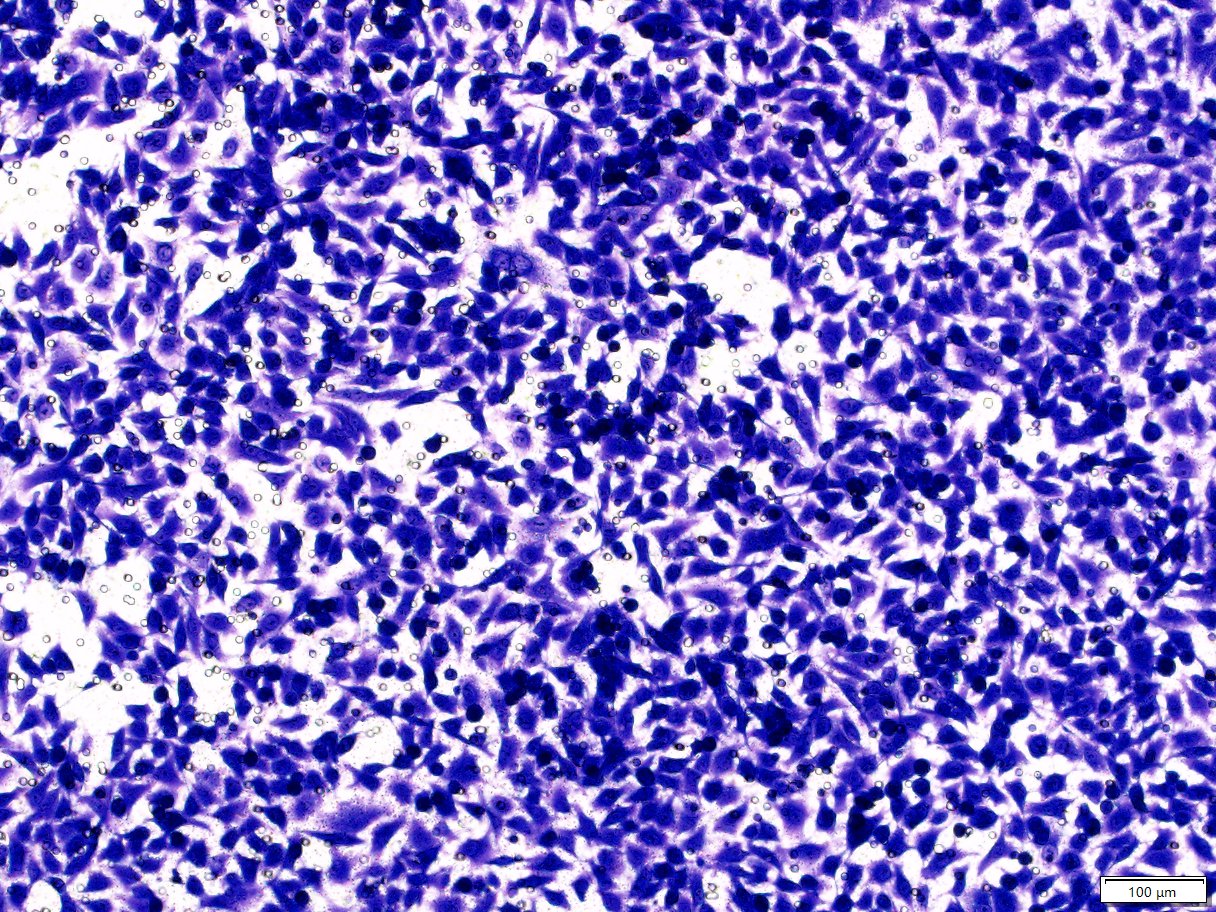

Supplement: Supplemental Information 12 [file peerj-cs-09-1651-s012.zip › Dataset 11/4-4.jpg]

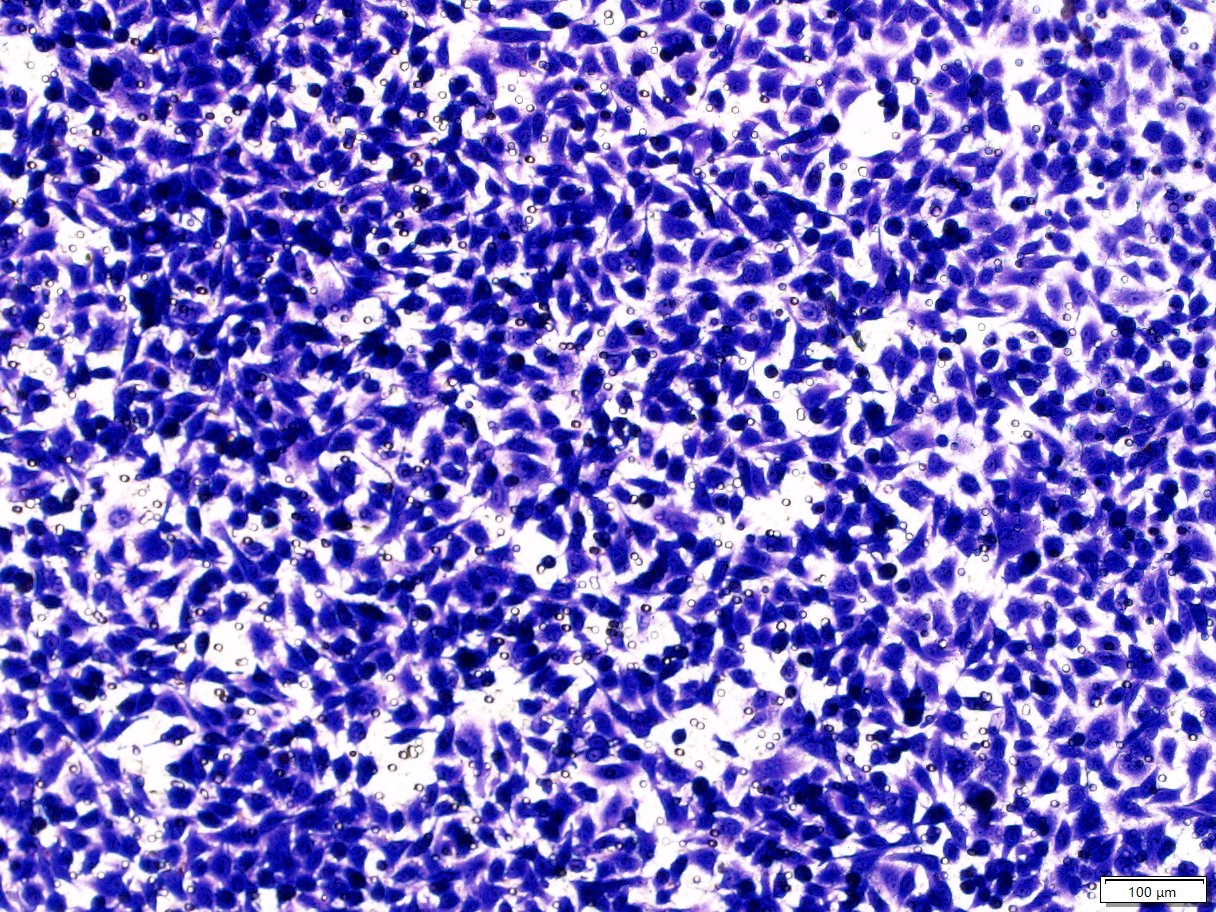

Supplement: Supplemental Information 12 [file peerj-cs-09-1651-s012.zip › Dataset 11/4-5.jpg]

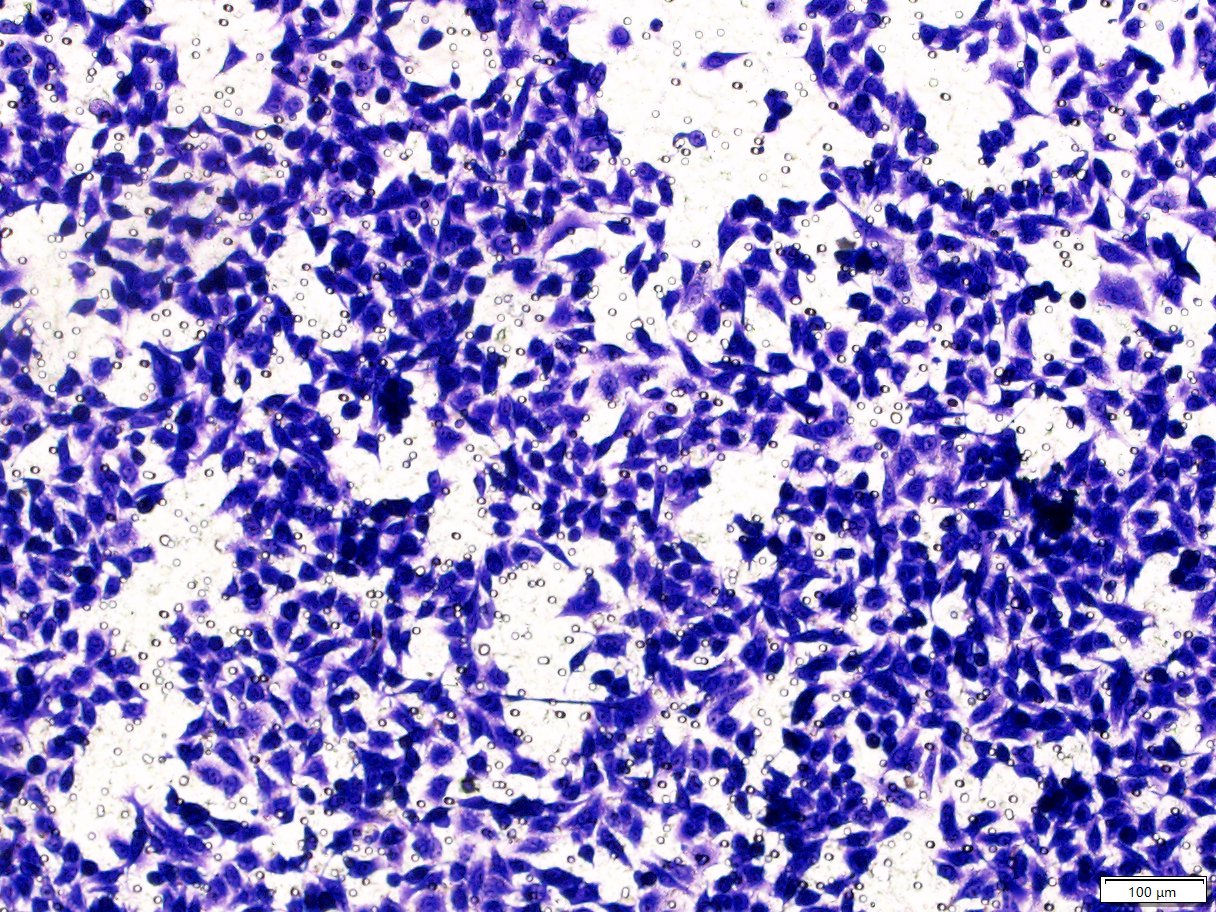

Supplement: Supplemental Information 12 [file peerj-cs-09-1651-s012.zip › Dataset 11/4-6.jpg]

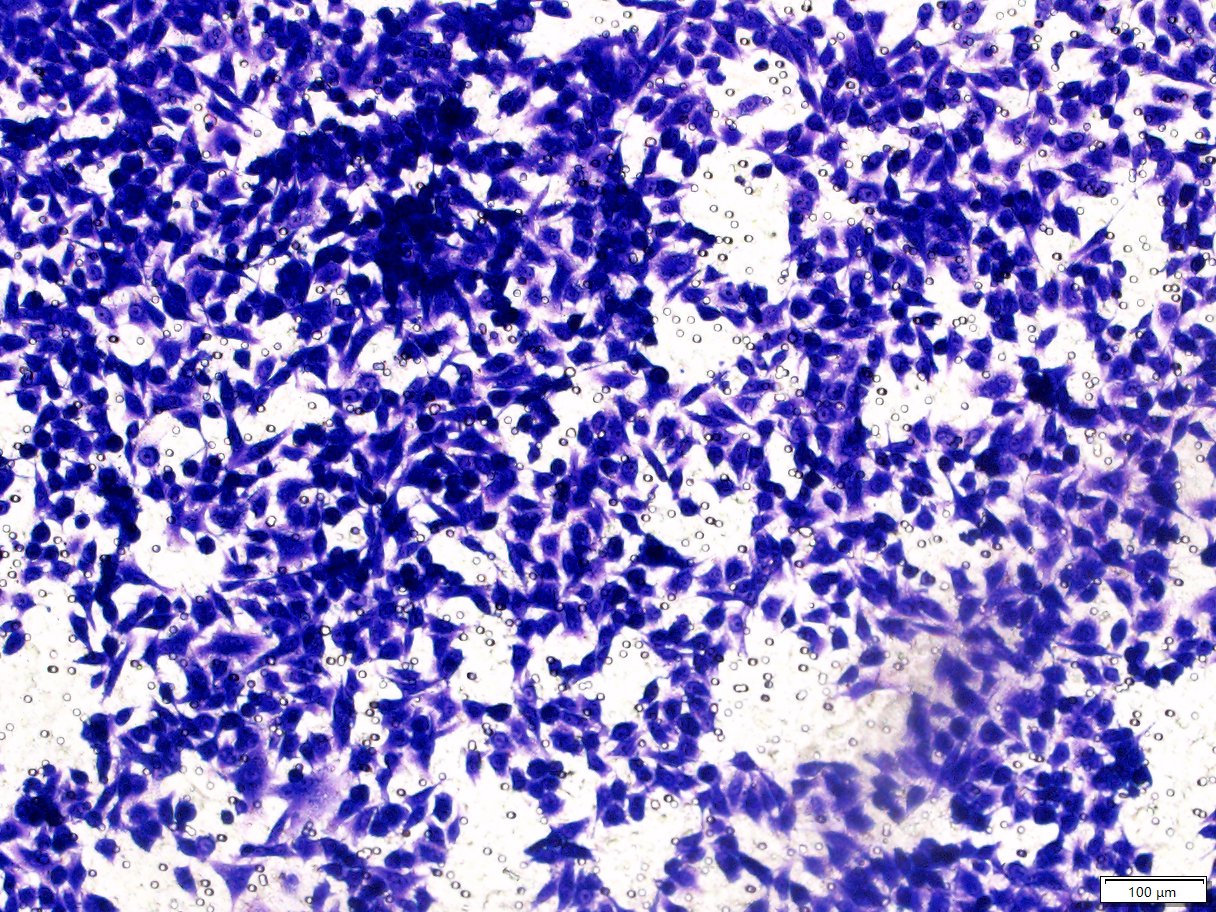

Supplement: Supplemental Information 12 [file peerj-cs-09-1651-s012.zip › Dataset 11/4-7.jpg]

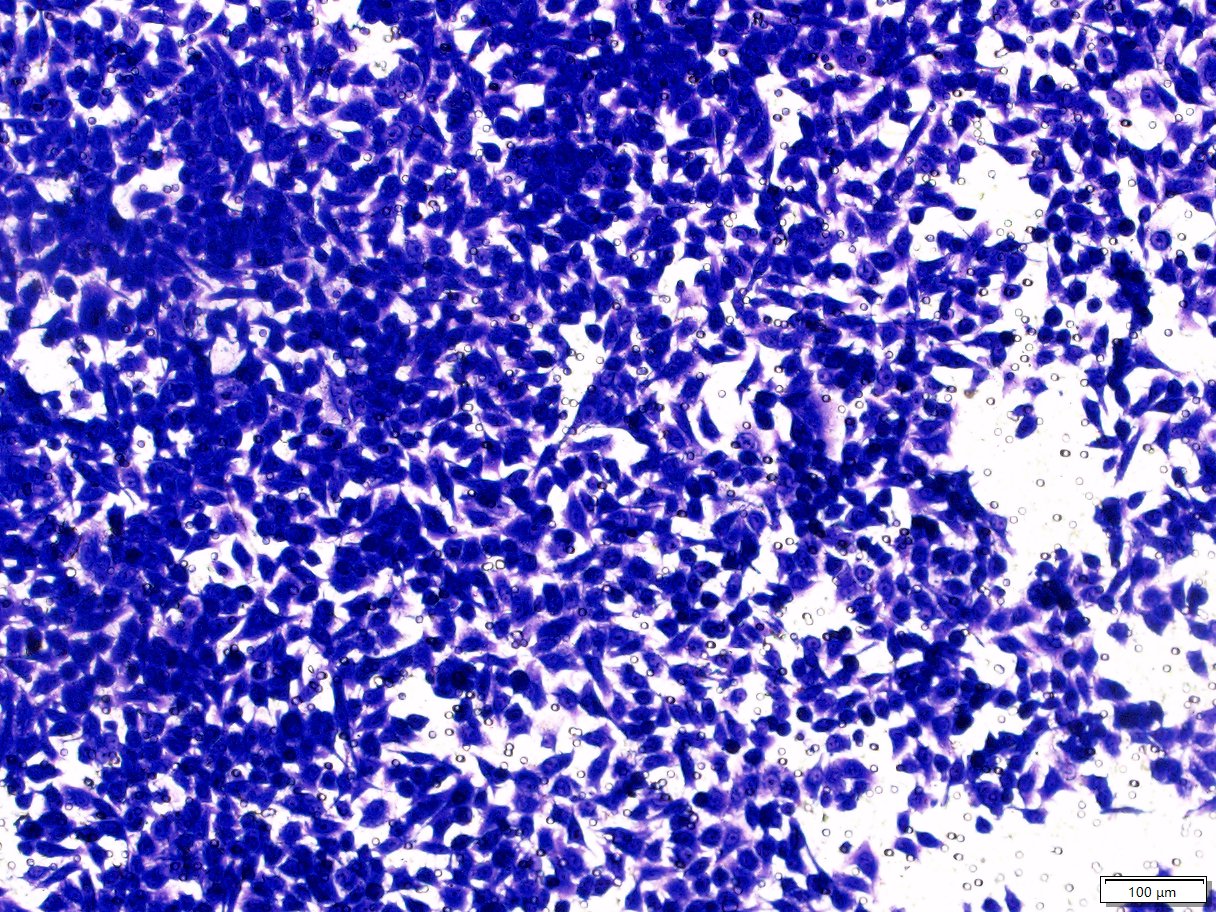

Supplement: Supplemental Information 12 [file peerj-cs-09-1651-s012.zip › Dataset 11/4-8.jpg]

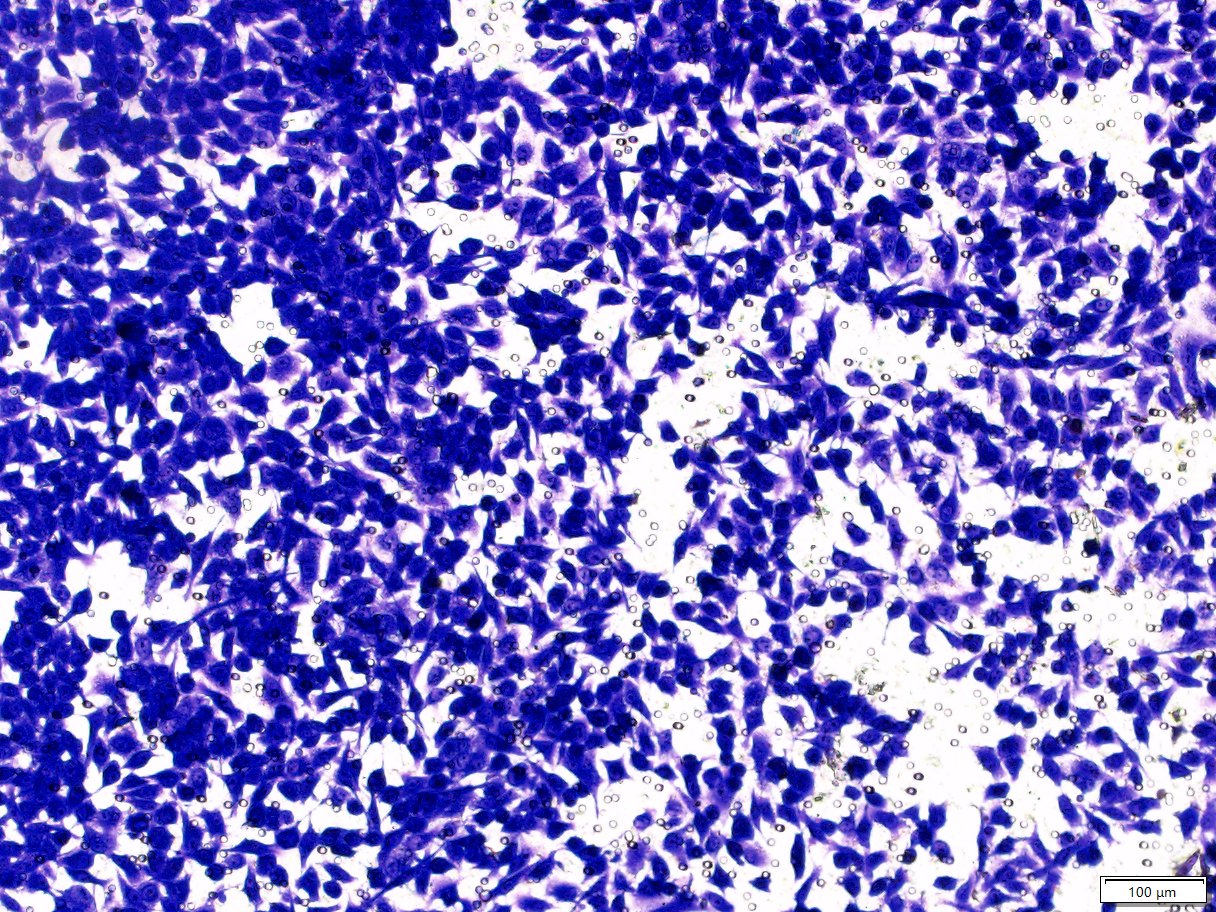

Supplement: Supplemental Information 12 [file peerj-cs-09-1651-s012.zip › Dataset 11/4-9.jpg]

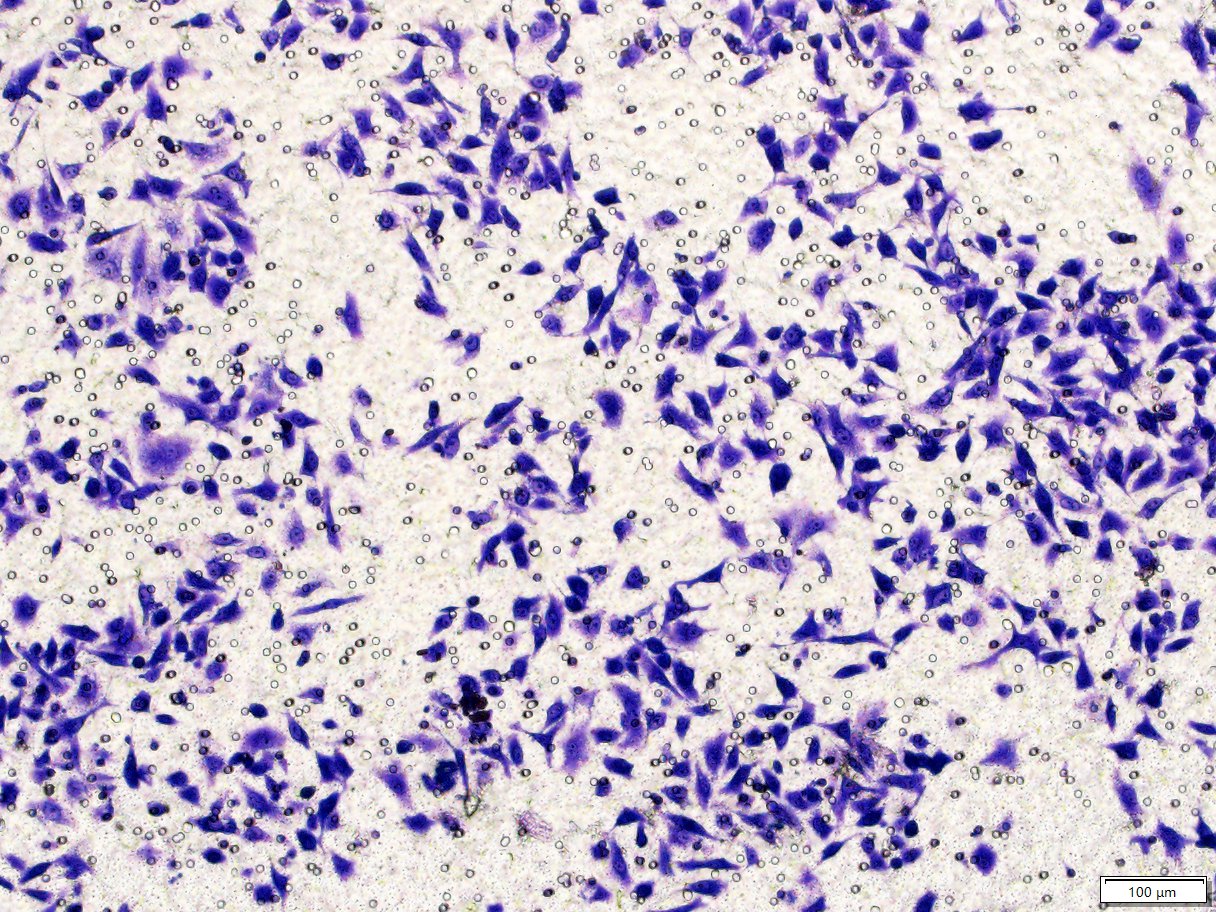

Supplement: Supplemental Information 12 [file peerj-cs-09-1651-s012.zip › Dataset 11/5+1.jpg]

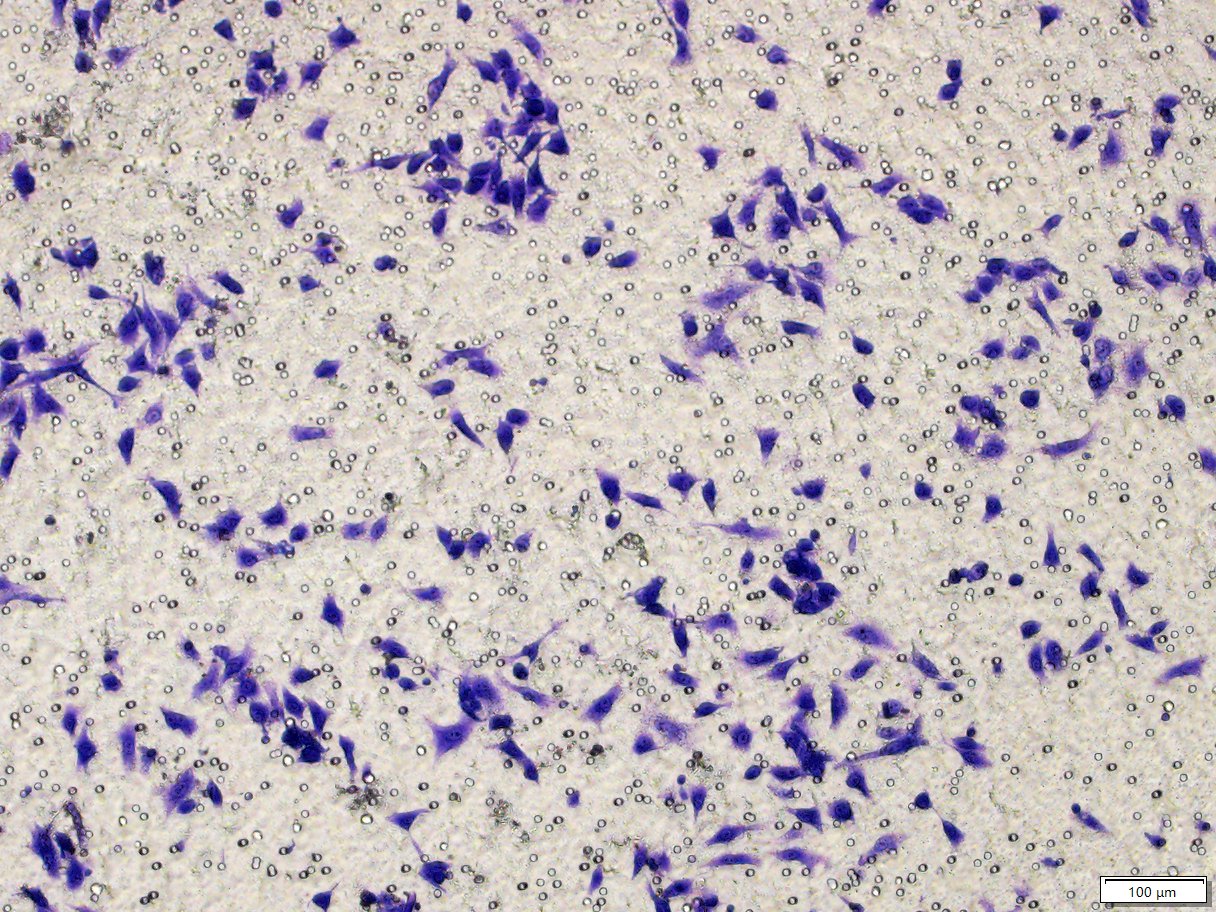

Supplement: Supplemental Information 12 [file peerj-cs-09-1651-s012.zip › Dataset 11/5+2.jpg]

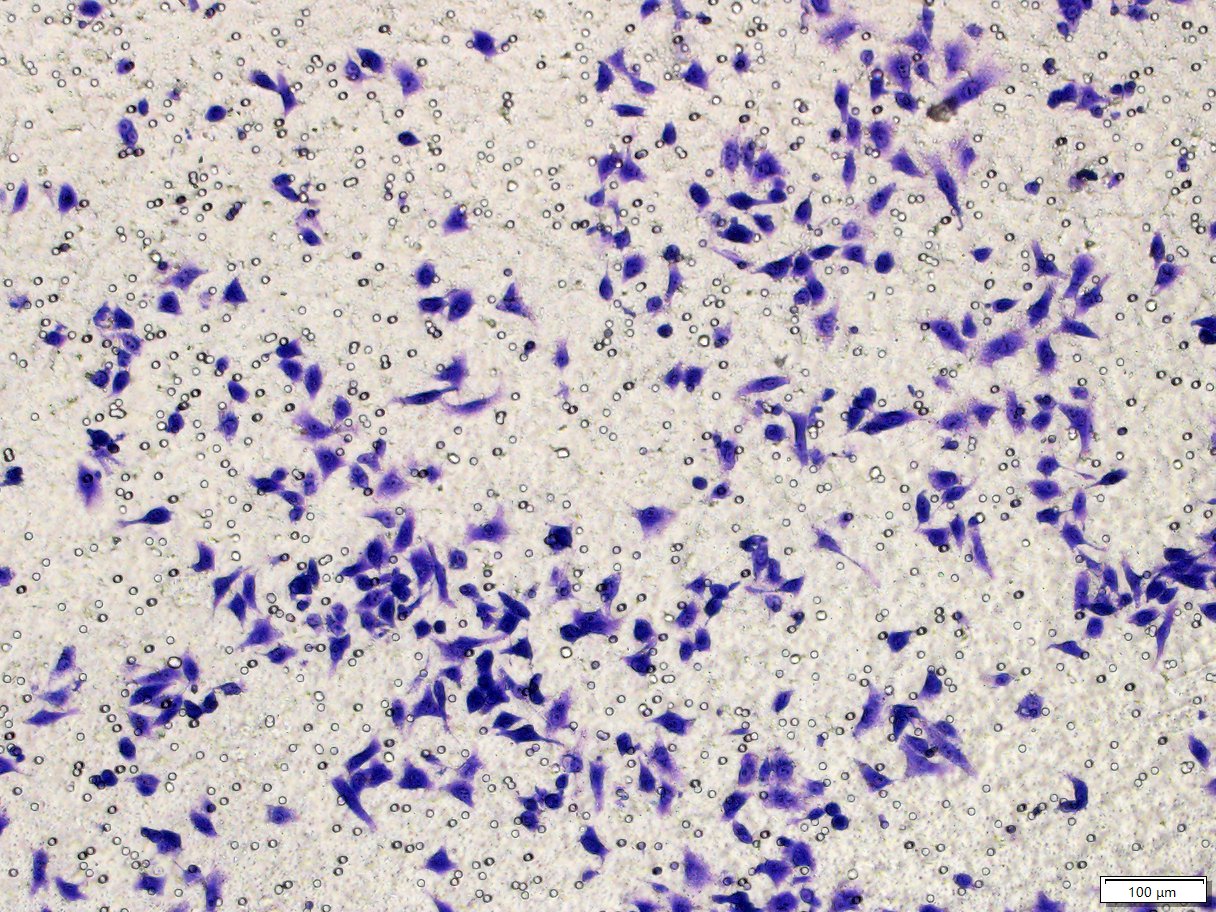

Supplement: Supplemental Information 12 [file peerj-cs-09-1651-s012.zip › Dataset 11/5+4.jpg]

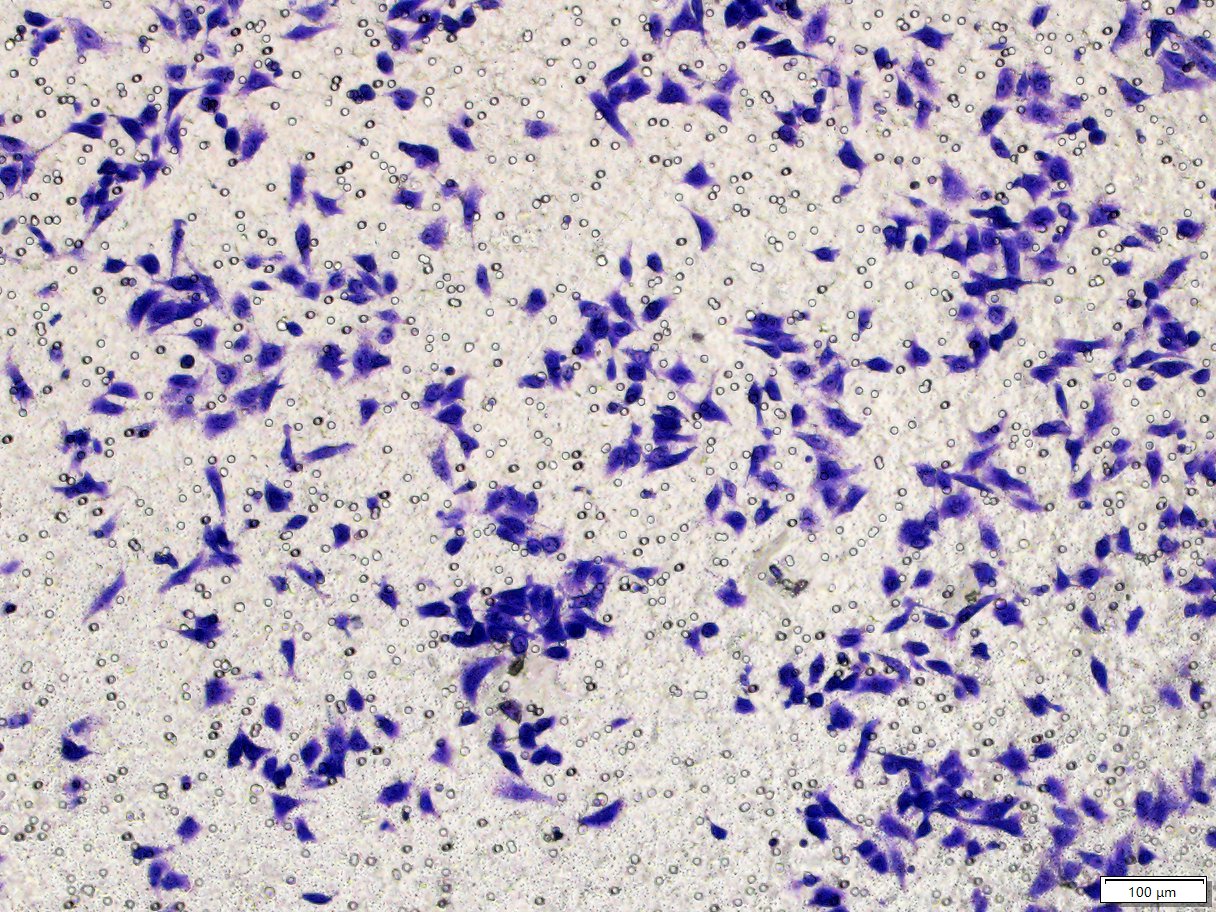

Supplement: Supplemental Information 12 [file peerj-cs-09-1651-s012.zip › Dataset 11/5+5.jpg]

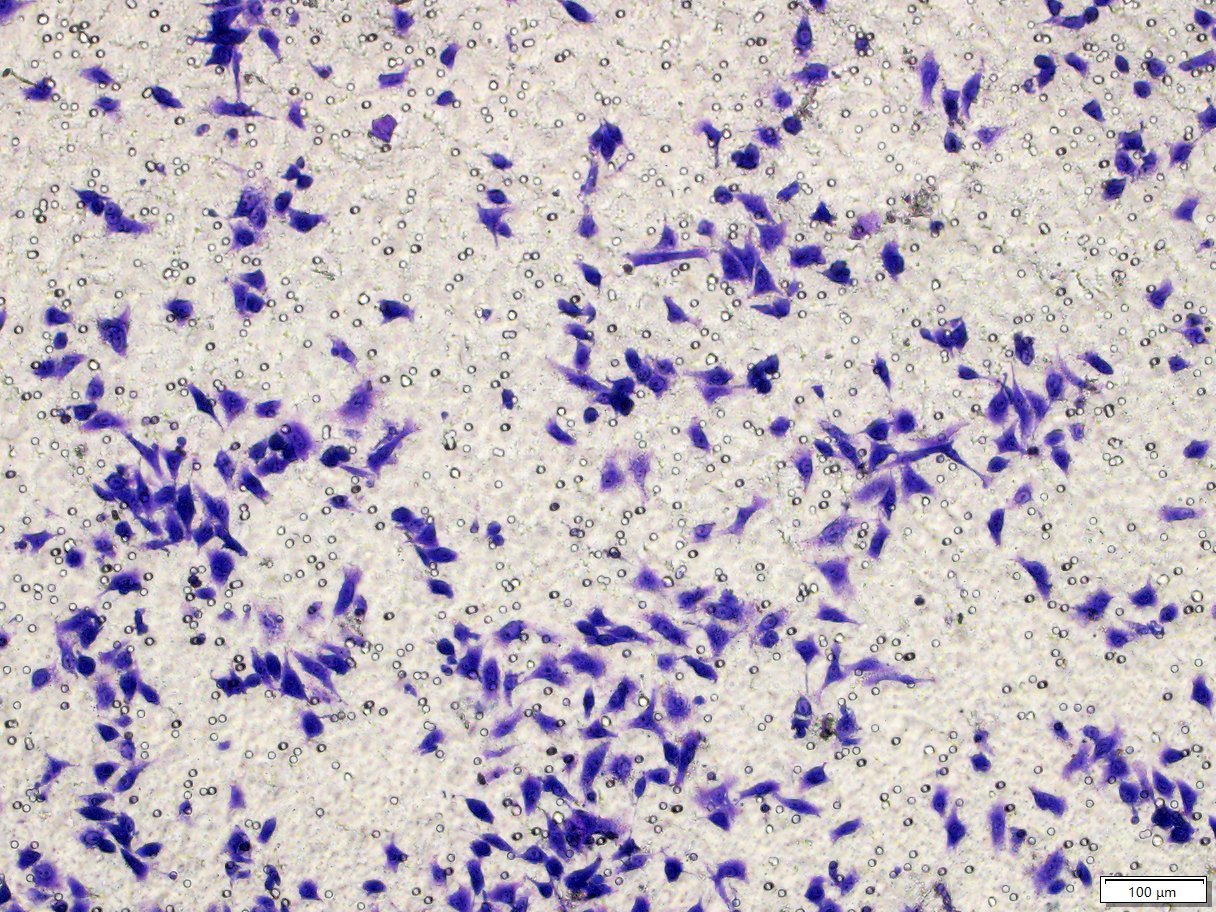

Supplement: Supplemental Information 12 [file peerj-cs-09-1651-s012.zip › Dataset 11/5+6.jpg]

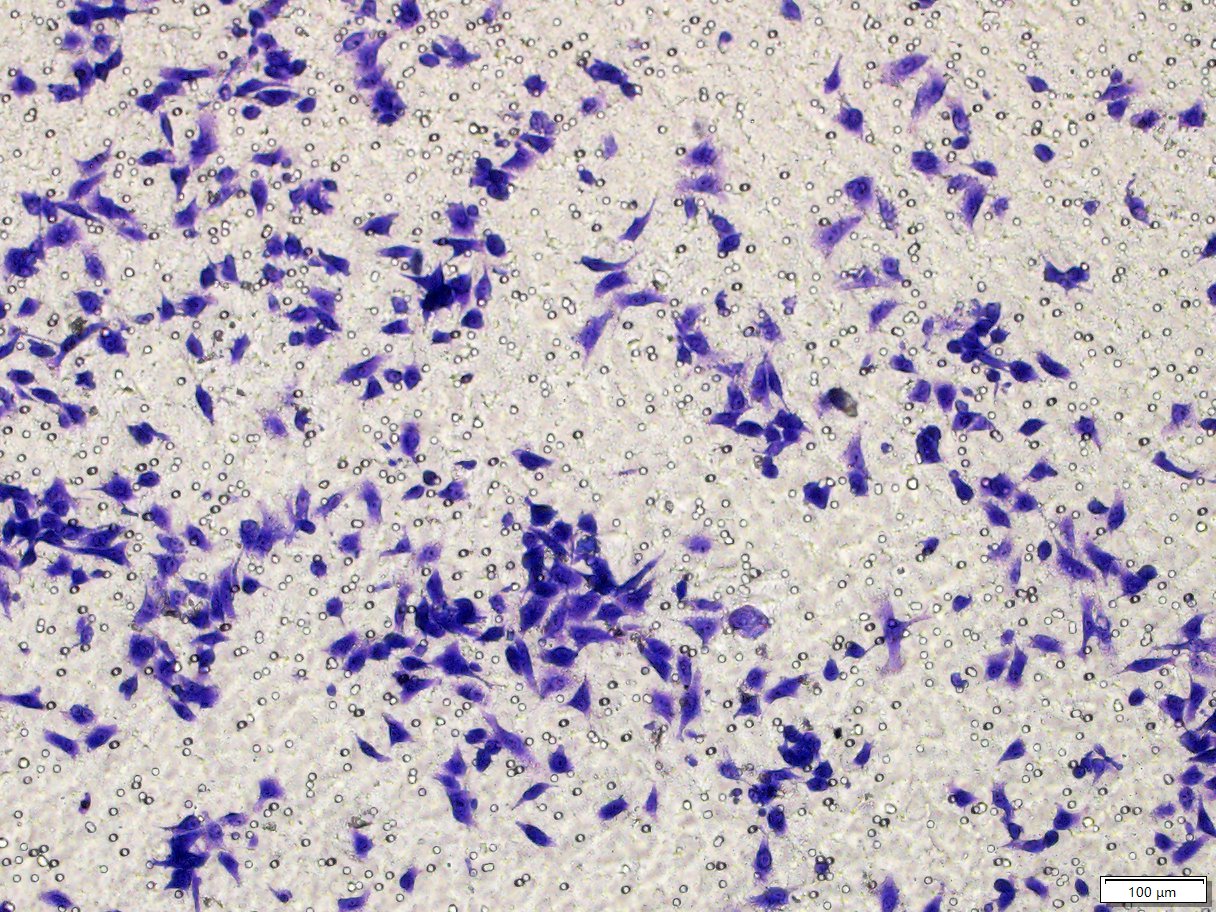

Supplement: Supplemental Information 12 [file peerj-cs-09-1651-s012.zip › Dataset 11/5+7.jpg]

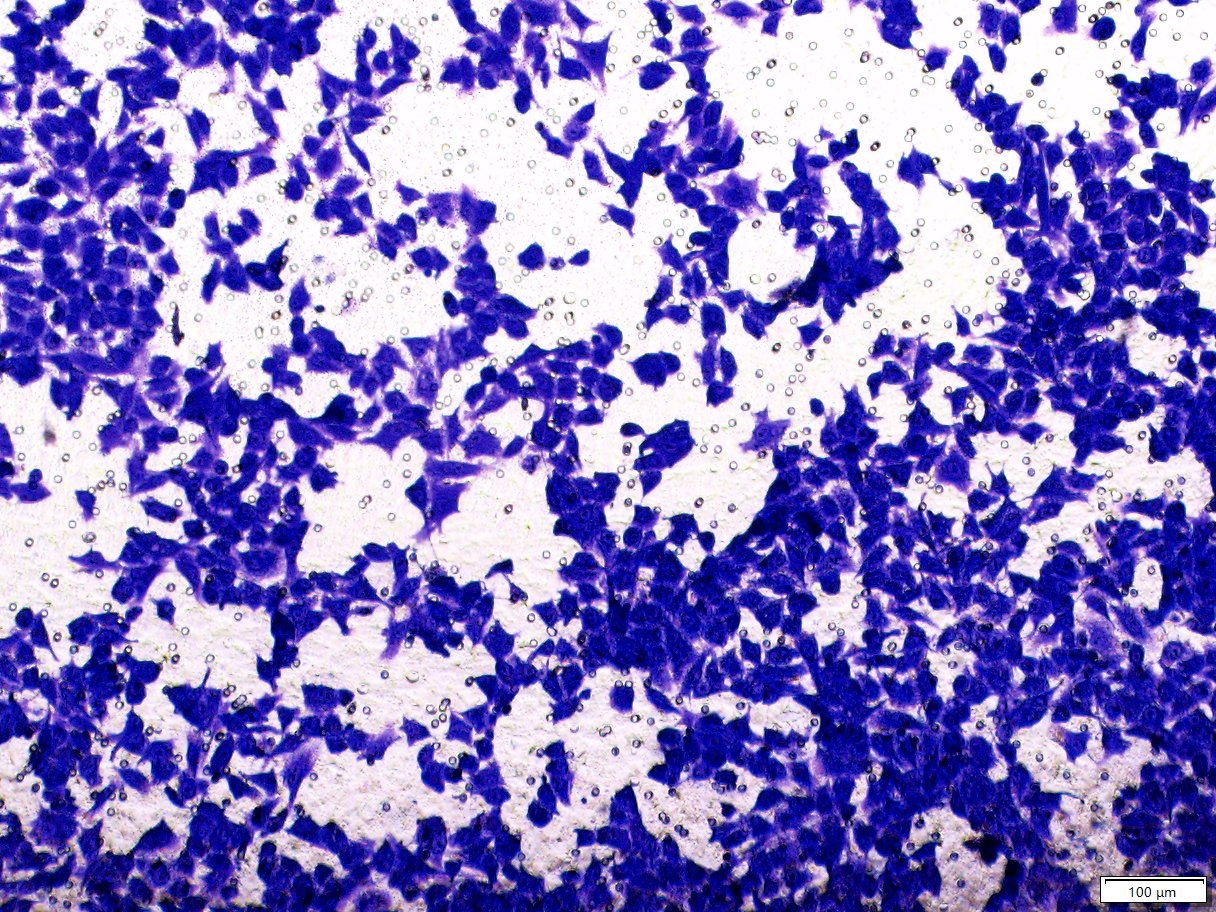

Supplement: Supplemental Information 12 [file peerj-cs-09-1651-s012.zip › Dataset 11/5-1.jpg]

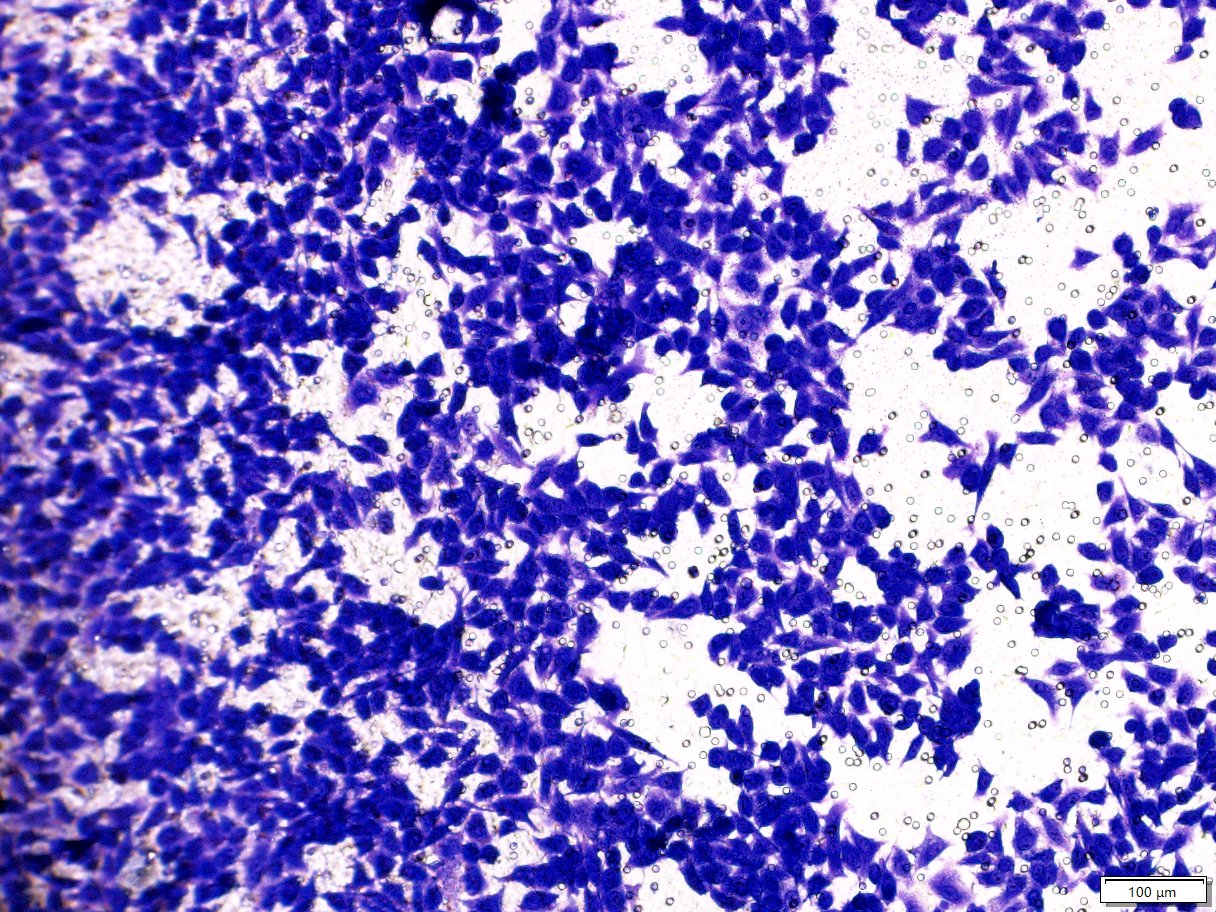

Supplement: Supplemental Information 12 [file peerj-cs-09-1651-s012.zip › Dataset 11/5-10.jpg]

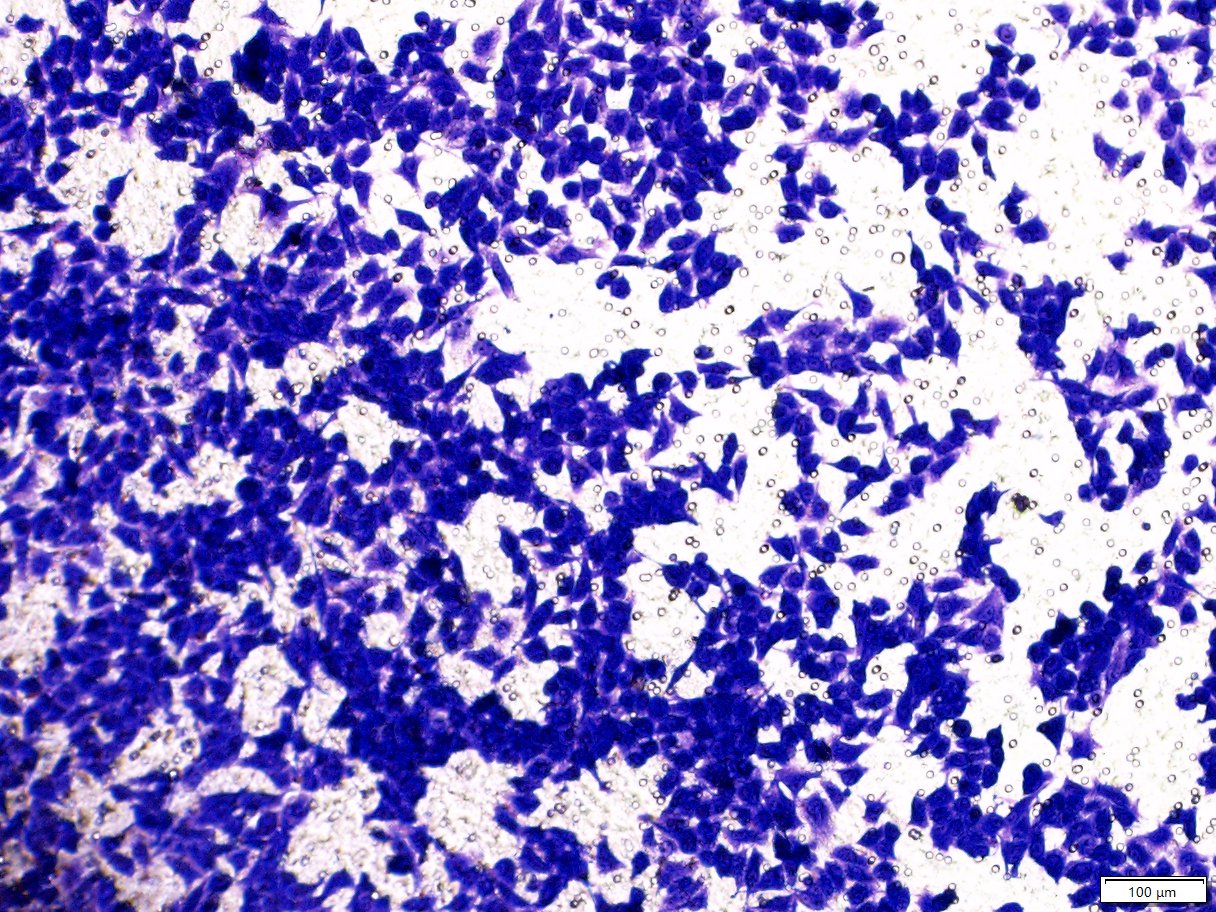

Supplement: Supplemental Information 12 [file peerj-cs-09-1651-s012.zip › Dataset 11/5-11.jpg]

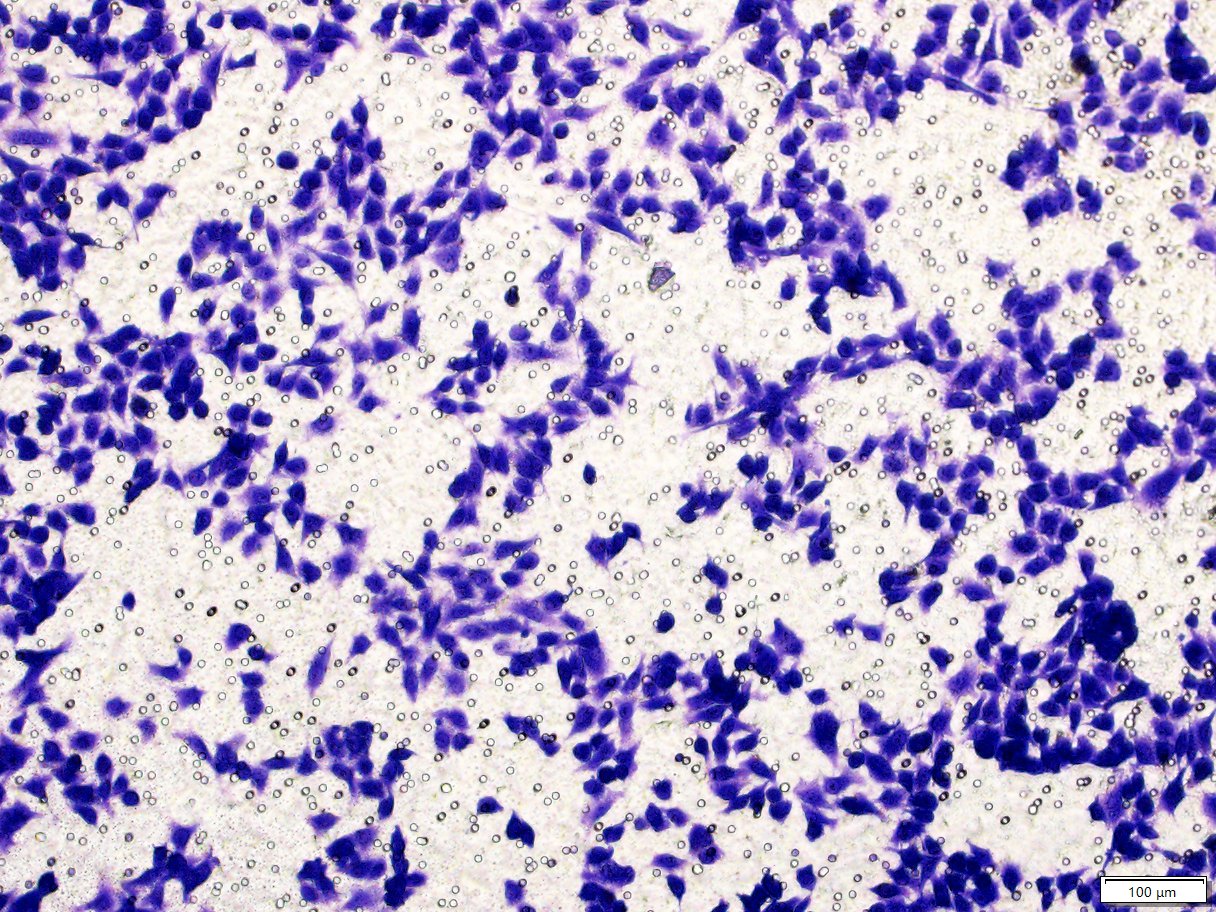

Supplement: Supplemental Information 12 [file peerj-cs-09-1651-s012.zip › Dataset 11/5-12.jpg]

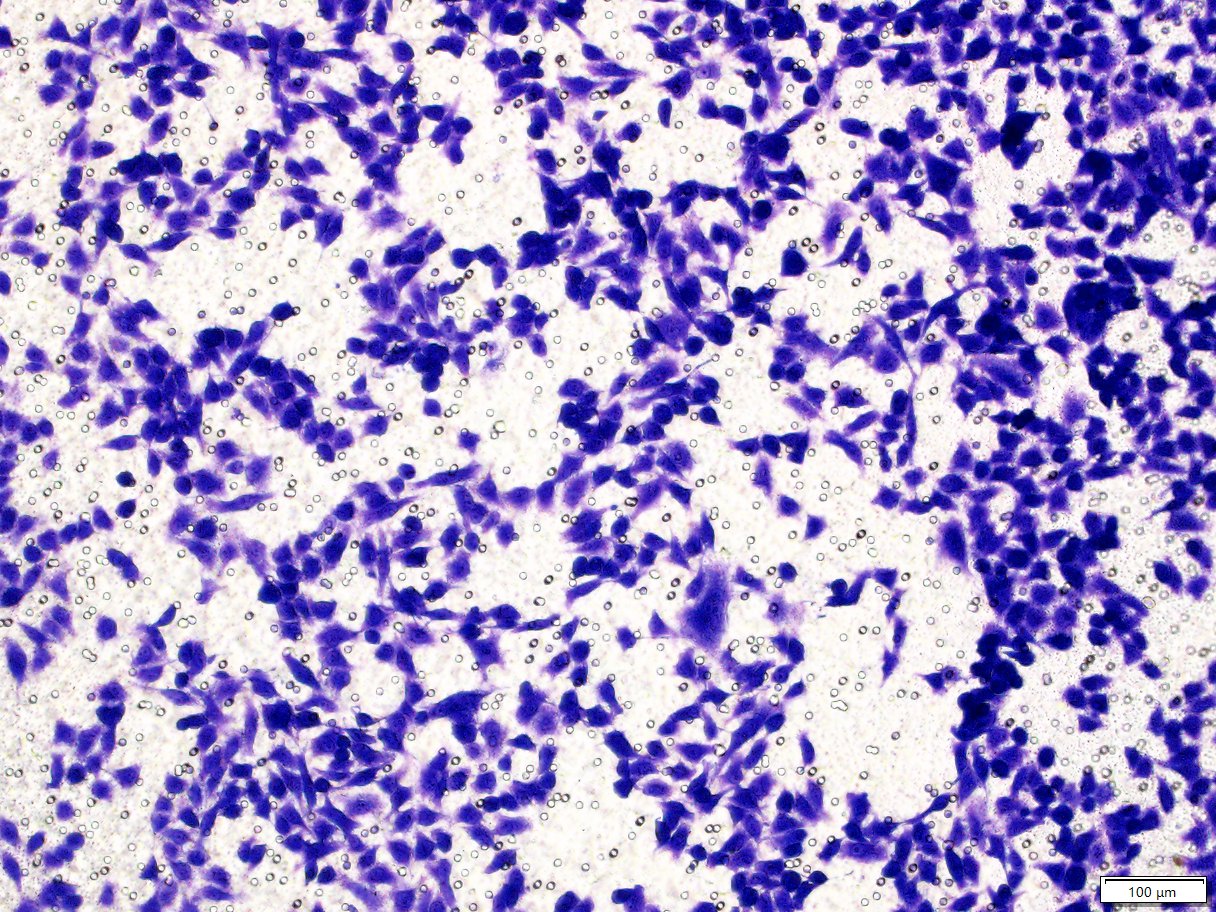

Supplement: Supplemental Information 12 [file peerj-cs-09-1651-s012.zip › Dataset 11/5-13.jpg]

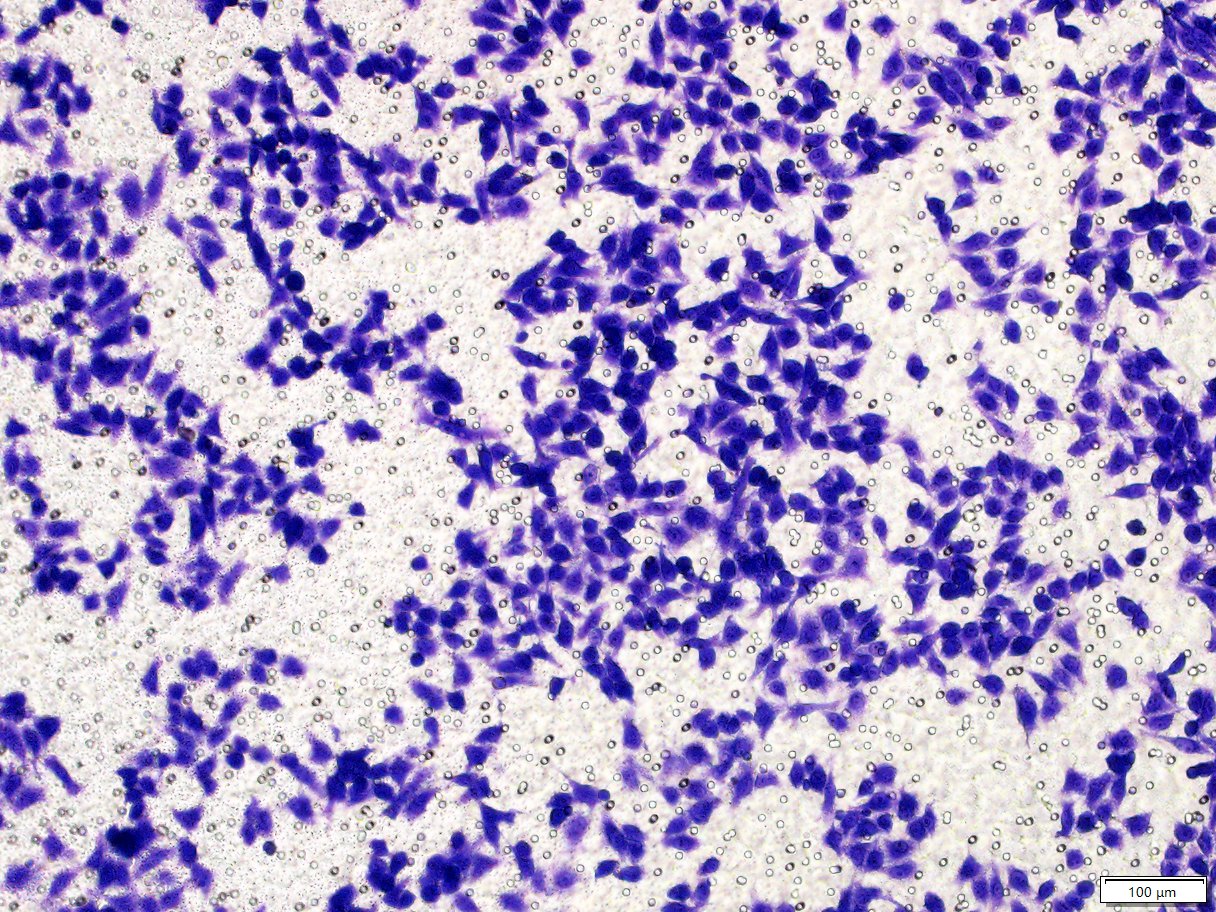

Supplement: Supplemental Information 12 [file peerj-cs-09-1651-s012.zip › Dataset 11/5-14.jpg]

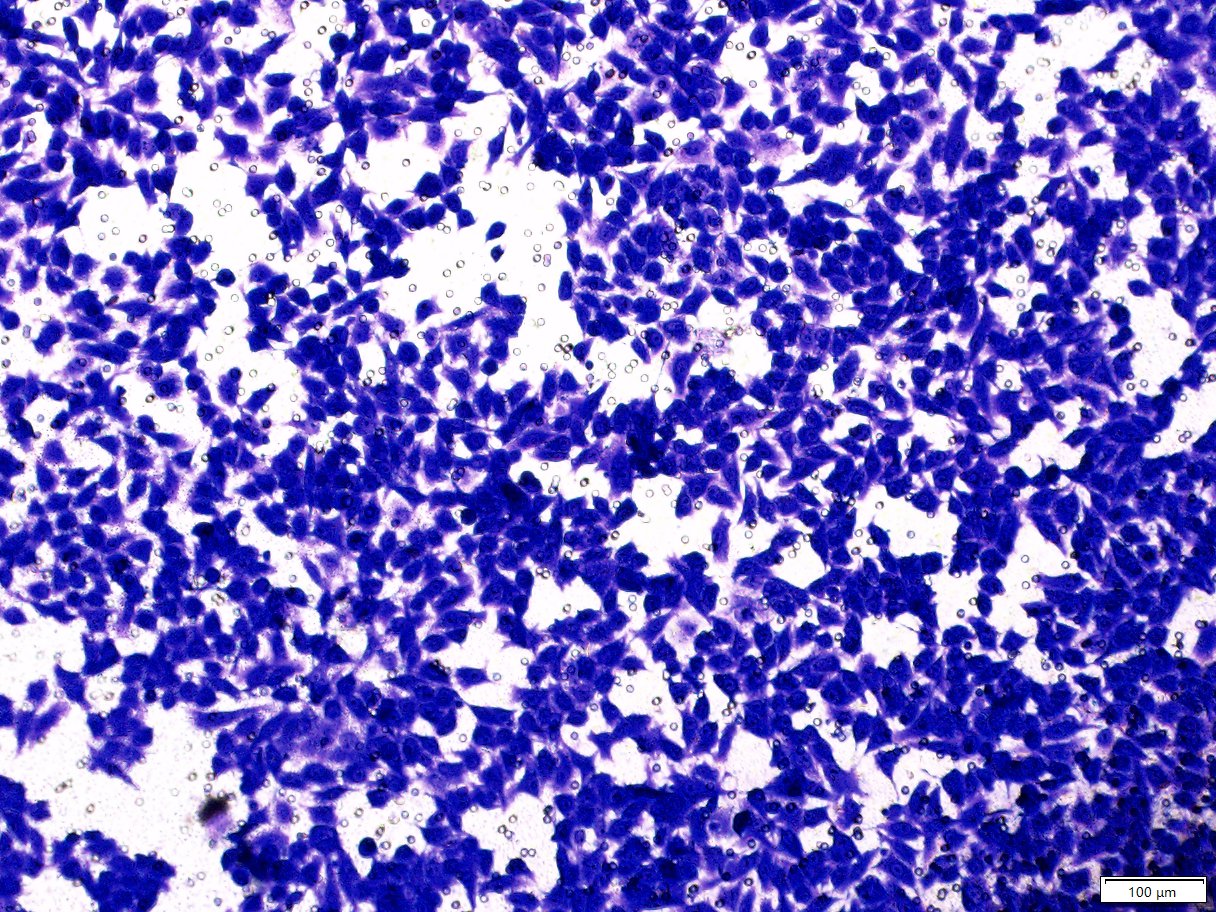

Supplement: Supplemental Information 12 [file peerj-cs-09-1651-s012.zip › Dataset 11/5-2.jpg]

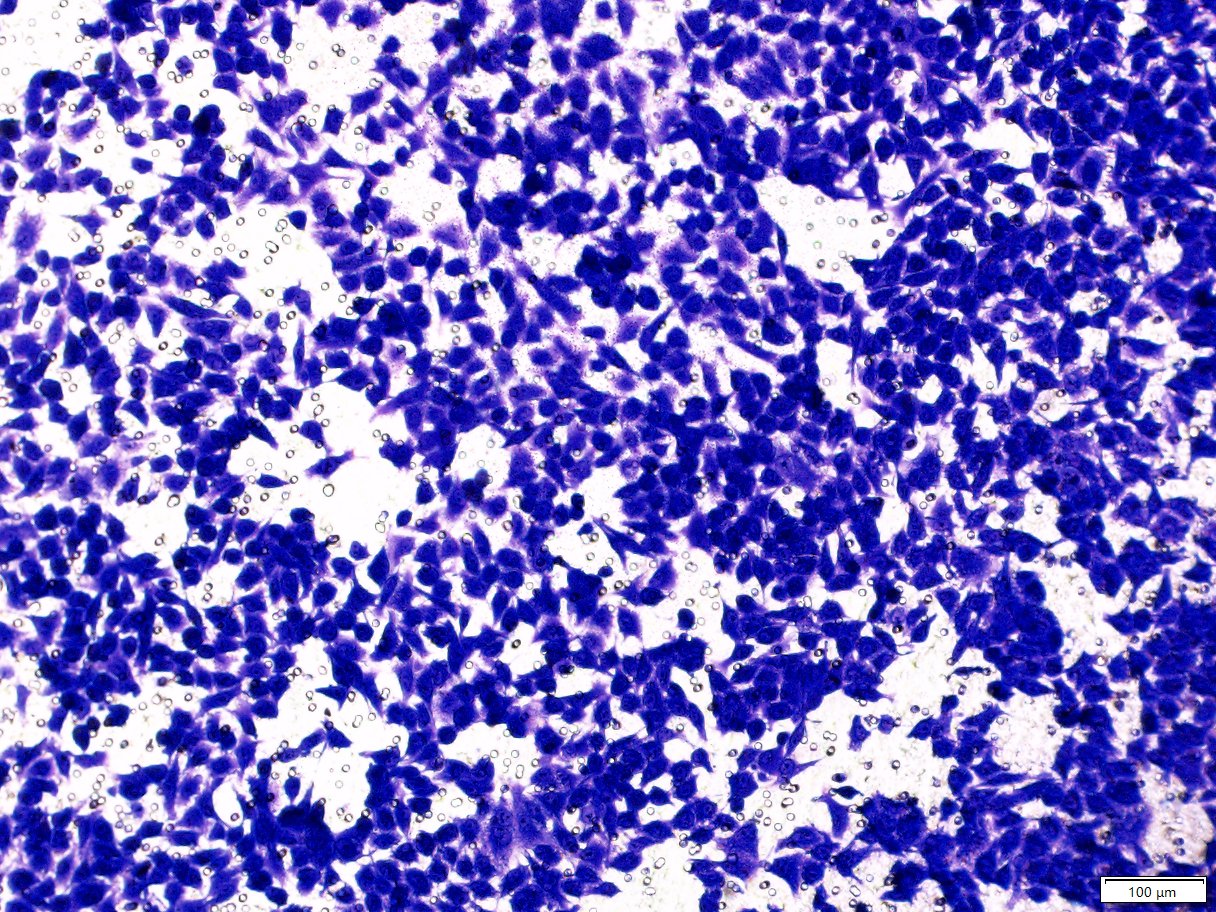

Supplement: Supplemental Information 12 [file peerj-cs-09-1651-s012.zip › Dataset 11/5-3.jpg]

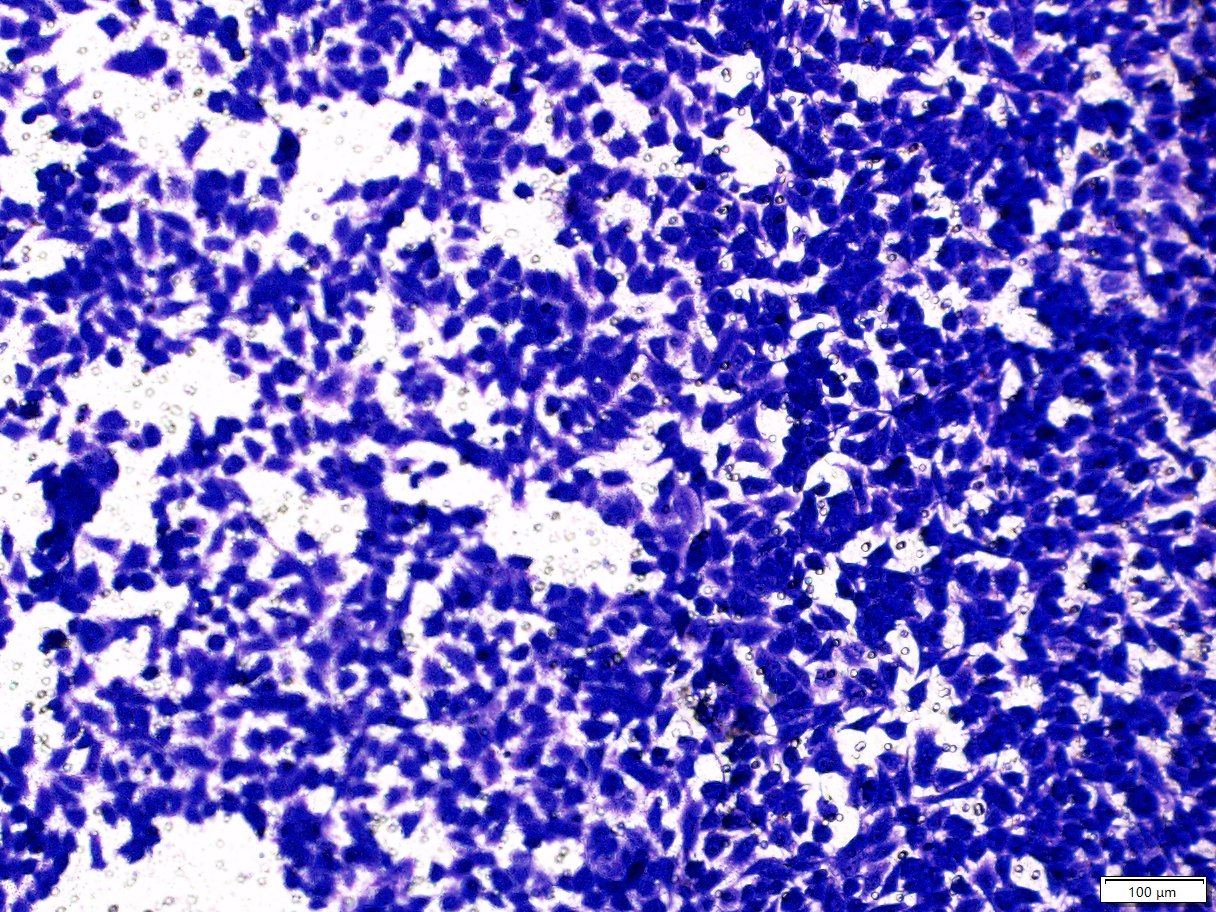

Supplement: Supplemental Information 12 [file peerj-cs-09-1651-s012.zip › Dataset 11/5-4.jpg]

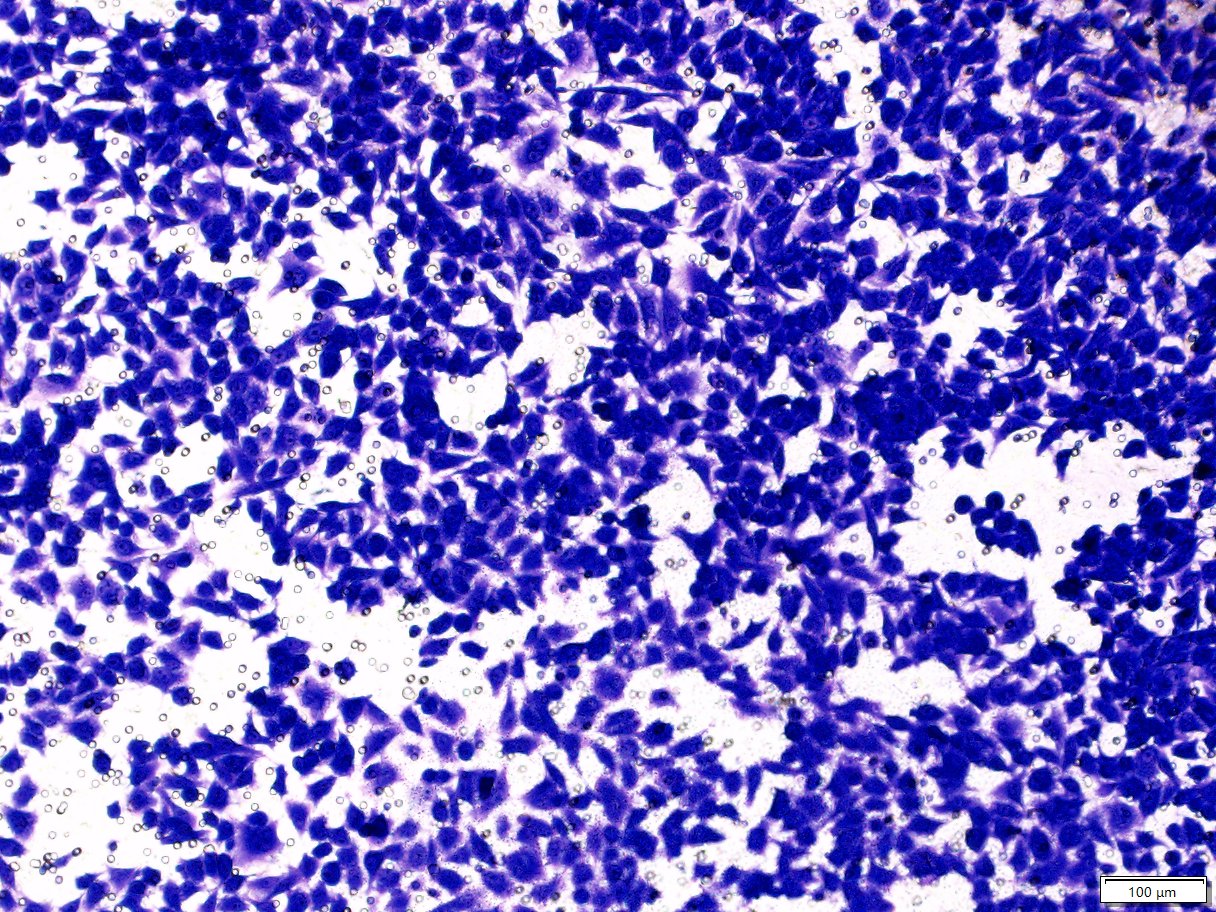

Supplement: Supplemental Information 12 [file peerj-cs-09-1651-s012.zip › Dataset 11/5-5.jpg]

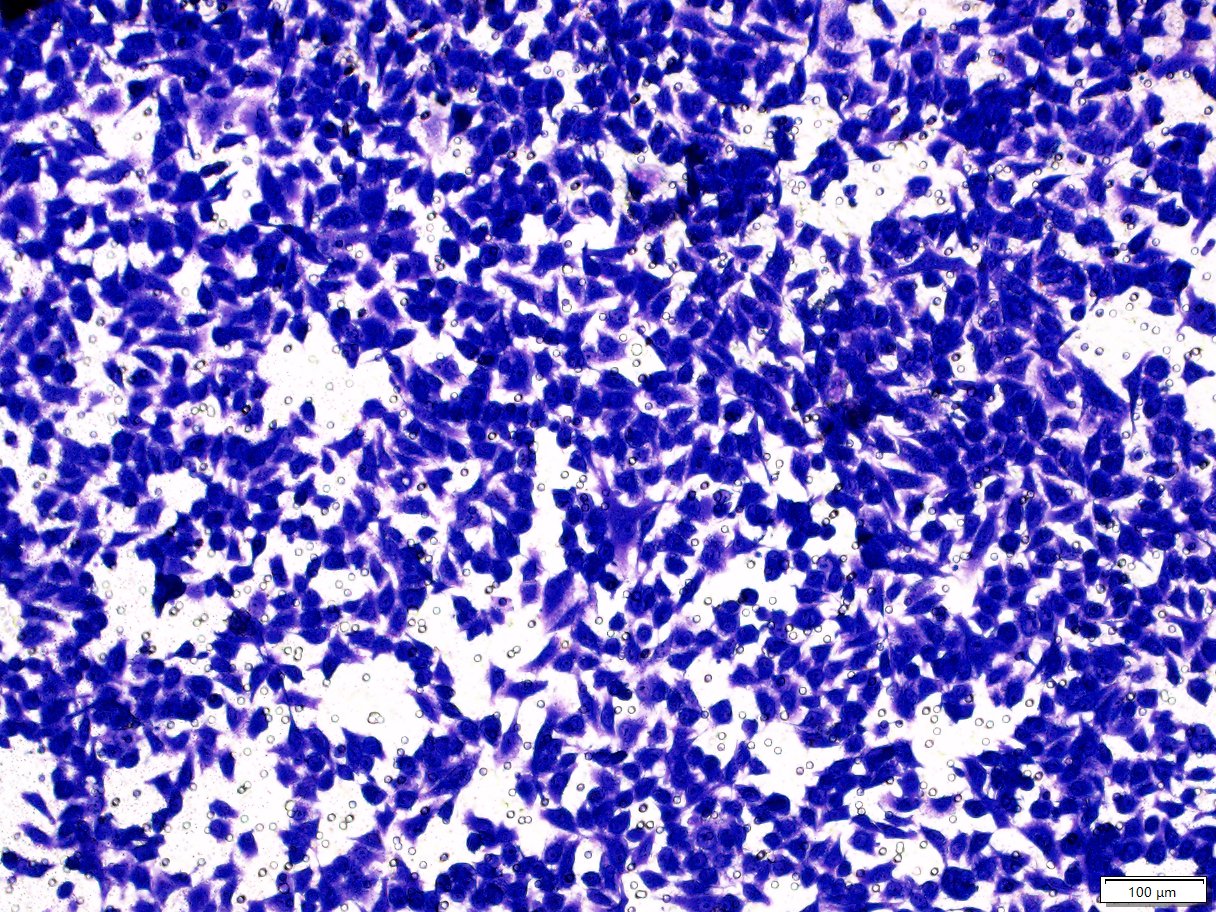

Supplement: Supplemental Information 12 [file peerj-cs-09-1651-s012.zip › Dataset 11/5-6.jpg]

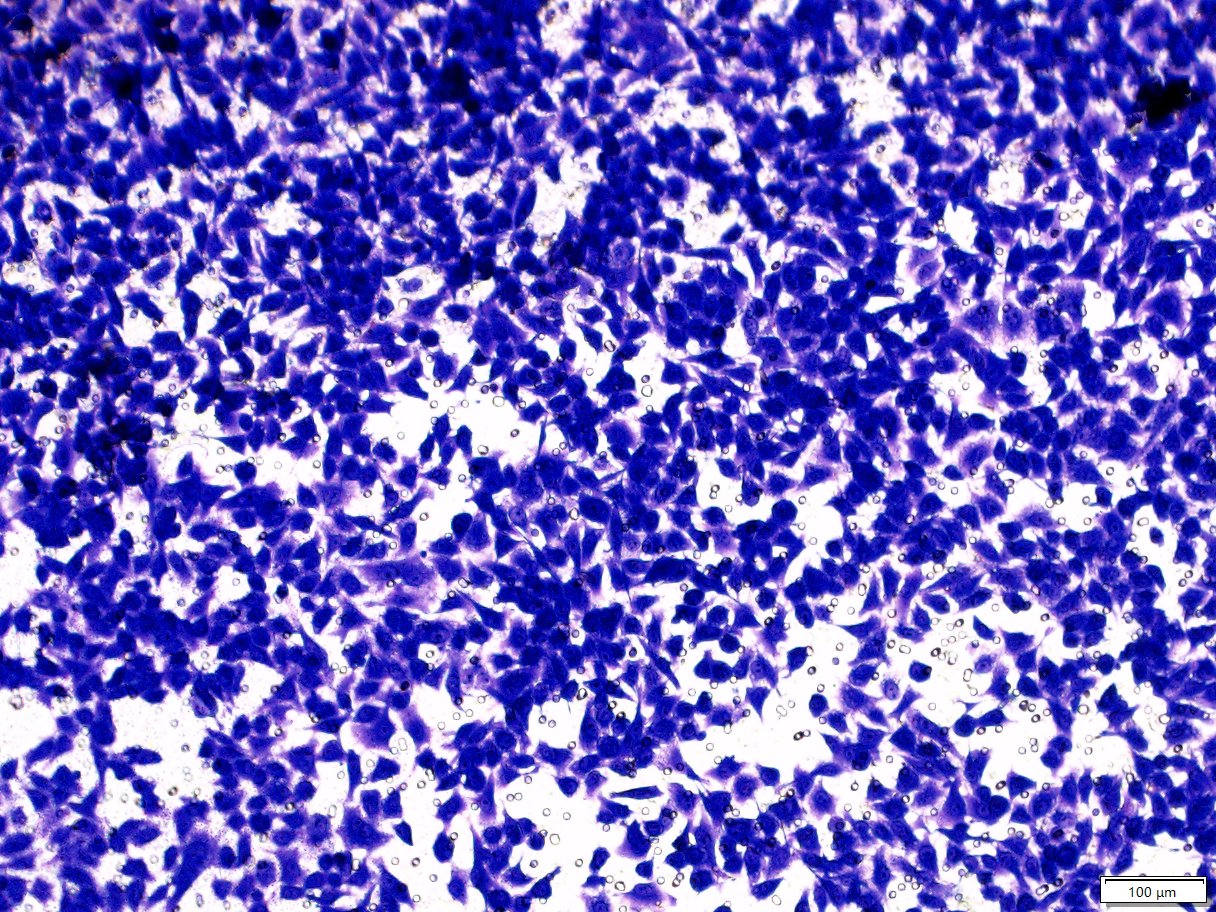

Supplement: Supplemental Information 12 [file peerj-cs-09-1651-s012.zip › Dataset 11/5-7.jpg]

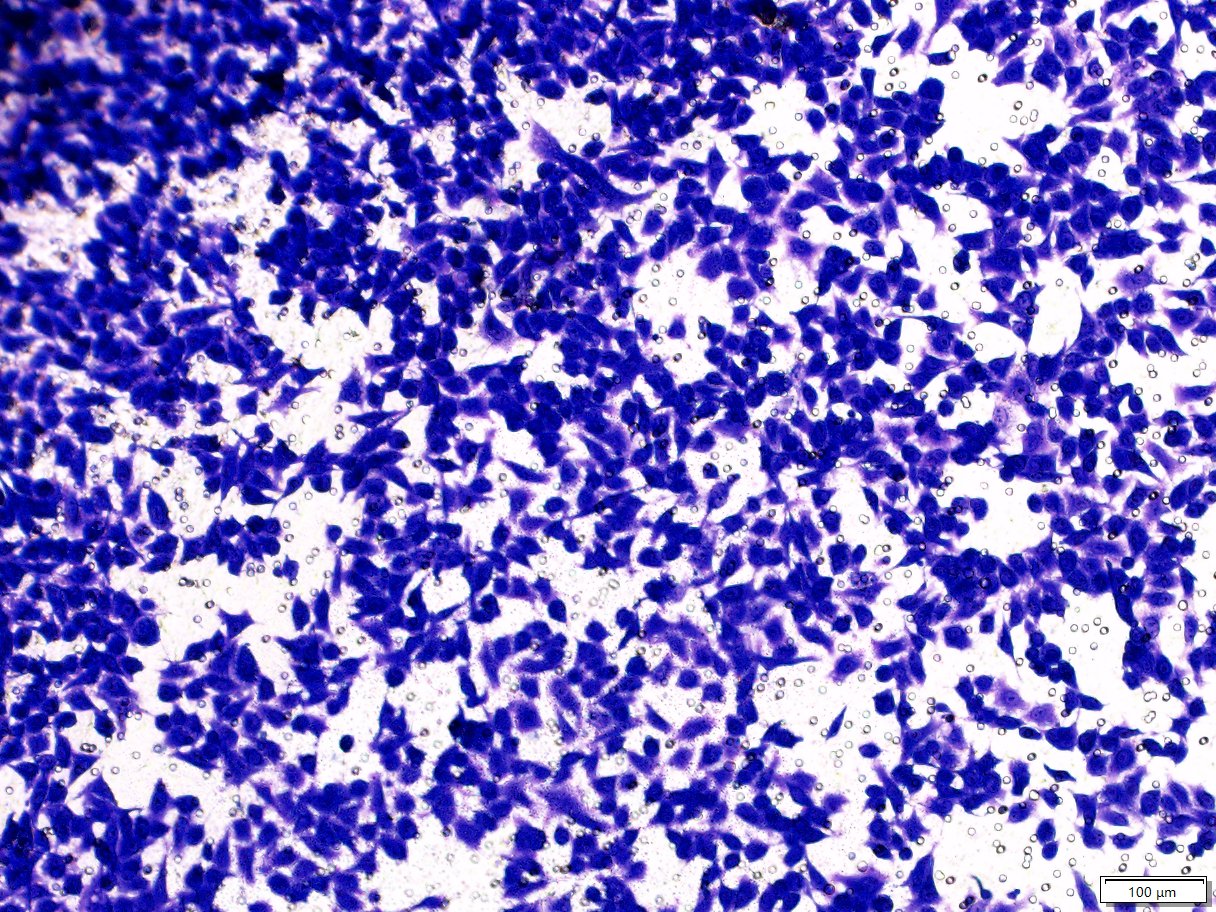

Supplement: Supplemental Information 12 [file peerj-cs-09-1651-s012.zip › Dataset 11/5-8.jpg]

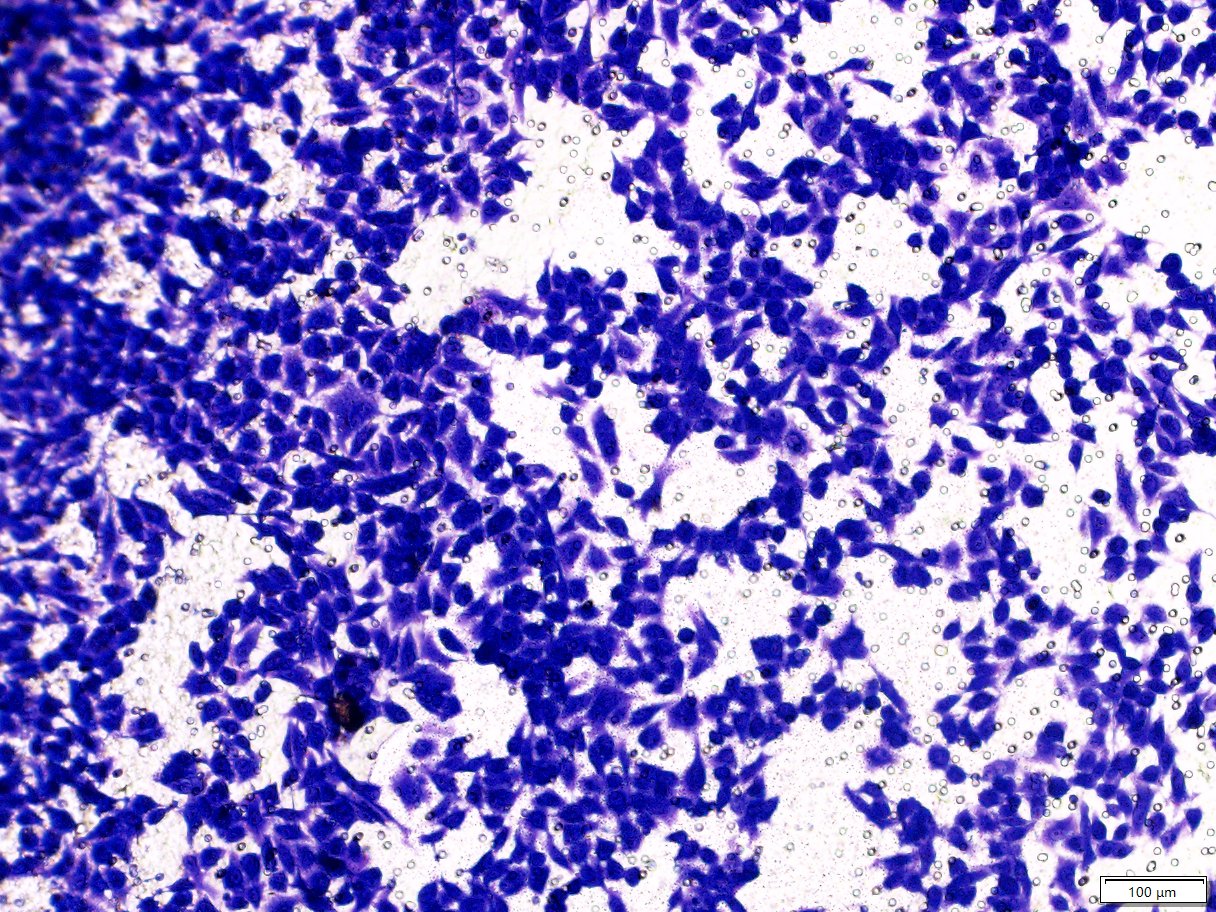

Supplement: Supplemental Information 12 [file peerj-cs-09-1651-s012.zip › Dataset 11/5-9.jpg]

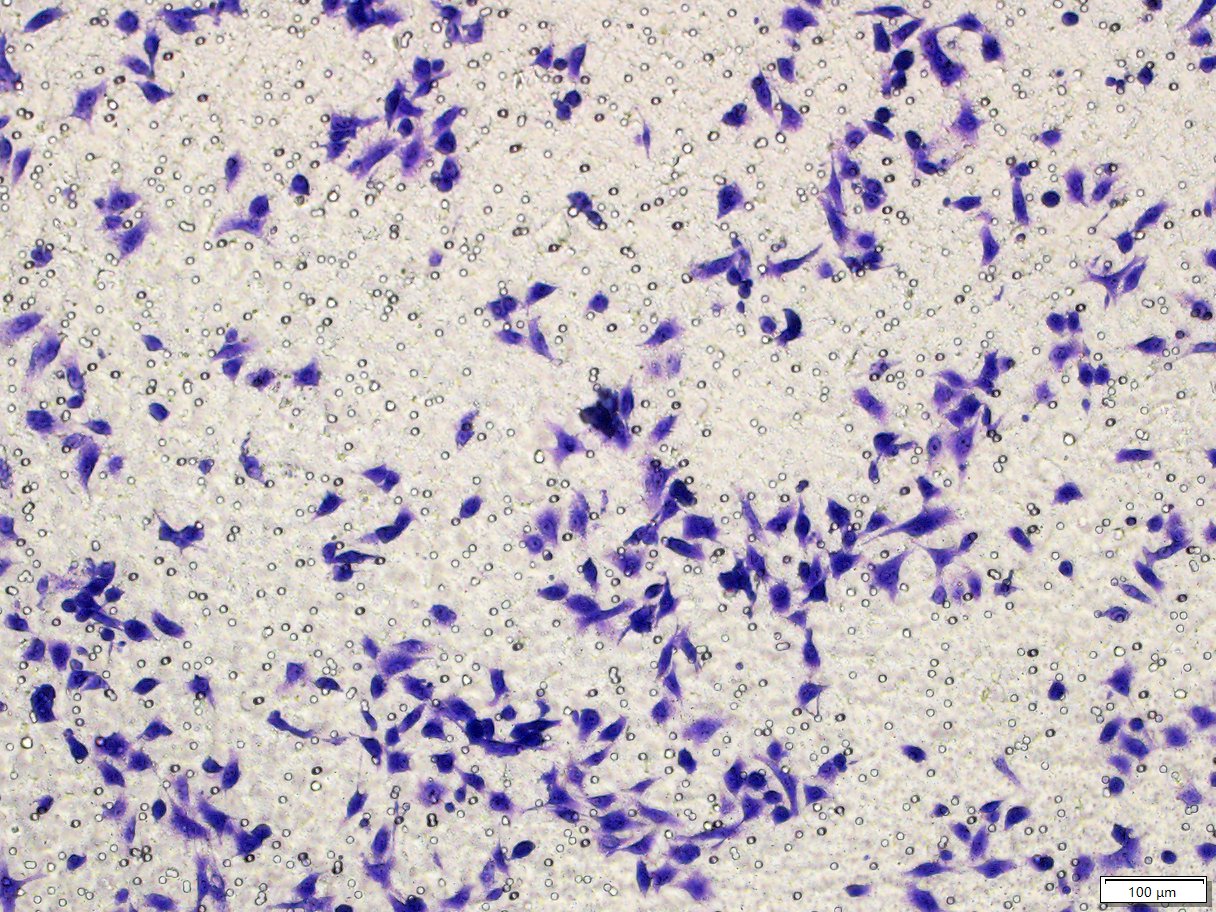

Supplement: Supplemental Information 12 [file peerj-cs-09-1651-s012.zip › Dataset 11/5.jpg]
